# Frameshift mutations at the C-terminus of HIST1H1E result in a specific DNA hypomethylation signature

**DOI:** 10.1186/s13148-019-0804-0

**Published:** 2020-01-07

**Authors:** Andrea Ciolfi, Erfan Aref-Eshghi, Simone Pizzi, Lucia Pedace, Evelina Miele, Jennifer Kerkhof, Elisabetta Flex, Simone Martinelli, Francesca Clementina Radio, Claudia A. L. Ruivenkamp, Gijs W. E. Santen, Emilia Bijlsma, Daniela Barge-Schaapveld, Katrin Ounap, Victoria Mok Siu, R. Frank Kooy, Bruno Dallapiccola, Bekim Sadikovic, Marco Tartaglia

**Affiliations:** 1 Genetics and Rare Diseases Research Division, Ospedale Pediatrico Bambino Gesù, IRCCS, 00146 Rome, Italy; 2 Department of Pathology and Laboratory Medicine, Western University, London, N6A 5C1 Canada; 3 Molecular Genetics Laboratory, Molecular Diagnostics Division, London Health Sciences Centre, London, N6A 5W9 Canada; 4 Department of Pediatric Onco-Hematology and Cell and Gene Therapy, Ospedale Pediatrico Bambino Gesù, IRCCS, 00146 Rome, Italy; 5 Department of Oncology and Molecular Medicine, Istituto Superiore di Sanità, 00161 Rome, Italy; 6 Department of Clinical Genetics, Leiden University Medical Center, Leiden, 2300 The Netherlands; 7 Department of Clinical Genetics, United Laboratories, Tartu University Hospital, 50406 Tartu, Estonia; 8 Institute of Clinical Medicine, University of Tartu, 50406 Tartu, Estonia; 9 Medical Genetics Program of Southwestern Ontario, London Health Sciences Centre, London, ON N6A 5W9 Canada; 10 Department of Medical Genetics, University of Antwerp, 2650 Antwerp, Belgium

**Keywords:** DNA methylation, Episignature, HIST1H1E, Rahman syndrome, Chromatin remodeling, Replicative senescence, Intellectual disability, Accelerated aging

## Abstract

**Background:**

We previously associated *HIST1H1E* mutations causing Rahman syndrome with a specific genome-wide methylation pattern.

**Results:**

Methylome analysis from peripheral blood samples of six affected subjects led us to identify a specific hypomethylated profile. This “episignature” was enriched for genes involved in neuronal system development and function. A computational classifier yielded full sensitivity and specificity in detecting subjects with Rahman syndrome. Applying this model to a cohort of undiagnosed probands allowed us to reach diagnosis in one subject.

**Conclusions:**

We demonstrate an epigenetic signature in subjects with Rahman syndrome that can be used to reach molecular diagnosis.

## Background

Insights on the role of chromatin in a neurodevelopmental context are rapidly emerging from human disease studies, and currently more than 40 genes encoding for proteins with role in the epigenetic machinery apparatus have been identified to cause developmental disorders when mutated [1, 2]. In these conditions, neurological dysfunction and intellectual disability (ID) are common features; even though, a variable set of developmental processes affecting other organs and systems are also observed to co-occur [2]. Frameshift mutations affecting *HIST1H1E* (MIM *142220) have been causally linked to the so-called Rahman syndrome (RMNS, MIM #61753), a recently recognized developmental disorder characterized by mild to severe ID, a distinctive facial gestalt, variable somatic overgrowth which may manifest in early infancy but is not observed in adults, and an aging appearance [3, 4]. As in the case of many neurodevelopmental disorders, RMNS exemplifies the challenges of reaching diagnosis on the basis of clinical criteria. Although the facial gestalt of affected subjects can help in recognition of the disease, no pathognomonic features can be used for a definitive clinical diagnosis. In early childhood, a tentative differential diagnosis may include Pallister-Killian syndrome and mild phenotypes within the spectrum of Weaver syndrome, Werner syndrome, and other progeroid disorders.

RMNS is caused by a narrow spectrum of functionally equivalent mutations affecting the C-terminus of HIST1H1E [3, 4], which is a member of the H1 histone family functioning as a structural component of chromatin to control the extent of DNA compaction, regulation of gene expression and DNA replication, recombination, and repair [5–8]. Consistent with the pleiotropic impact of altered chromatin compaction, this class of *HIST1H1E* mutations was found to perturb multiple cellular processes resulting in cellular senescence and replicative impasse [4]. Notably, we and others previously observed that dysregulation and loss of HIST1H1E function affect genome methylation [4, 8].

Based on the evidence that defects in genes involved in the maintenance of chromatin organization have specific genome-wide epigenetic patterns [9–16] and our previous preliminary findings in this disorder, here we explored the genome-wide DNA methylation profiles associated with these mutations using a more comprehensive approach to characterize and validate the DNA methylation signature (“episignature”) of this disorder. We provide data defining an episignature characterizing RMNS, and demonstrate that this signature involves genes with role in neural system pathways. We show that these changes are specific to RMNS and do not occur in other neurodevelopmental conditions with peripheral blood episignatures that are caused by mutations affecting chromatin regulators. Moreover, by creating a specific computational model, we show that the identified episignature can successfully be used to reach diagnosis of clinically unsolved cases.

## Methods

### Patients and cohorts

This study was approved by the Ethical Committee of the Ospedale Pediatrico Bambino Gesù (1702 OPBG 2018), and by the Western University Research Ethics Board (REB 106302). DNA specimens from the subjects included in this study were collected following procedures in accordance with the ethical standards of the declaration of Helsinki protocols and approved by the Review Boards of all involved institutions, with signed informed consents from the participating subjects/families. Genome-wide DNA methylation data from six previously published individuals presenting with RMNS (see [4]; subjects 1, 4, 5, 12, 13, and 14) were used to map the DNA methylation episignature and build a classification algorithm. The study included all individuals with RMNS for whom genomic DNA extracted from peripheral blood was available. The clinical phenotype of the analyzed patients was characterized by variable ID/developmental delay (DD) and a distinctive facial gestalt (e.g., prominent forehead and high anterior hairline, hypertelorism, broad nasal tip, and dysmorphic ears). Additional features variably included behavioral problems, hypotrichosis, cutis laxa, and skeletal and ectodermal abnormalities. Additional minor signs were present in single individuals. These samples were compared with a reference cohort of controls from a pool of healthy individuals in the London Health Sciences EpiSign Knowledge Database [17]. A larger set of controls used to assess the specificity of the classification model was compiled from three large databases of general population samples with various age and ethnicity (GSE42861, GSE87571, and GSE99863) [18–20]. Healthy controls included age- and sex-matched individuals without any neurodevelopmental presentations, ID, DD, congenital anomalies, or a diagnosis of a genetic syndrome. Samples from patients with other developmental syndromes caused by mutations in genes encoding other regulators of the epigenetic machinery (EpiSign Knowledge Database) were used to measure the specificity of the RMNS DNA methylation signature. These data include those described in our previous studies [9–16], and included patients with imprinting defect disorders (see “Results” section). Any subject used herein to represent each disorders had a molecularly confirmed diagnosis. The mutation report from each patient was reviewed according to the American College of Medical Genetics and Genomics guidelines for interpretation of genomic sequence variants [21], and only individuals confirmed to carry a pathogenic or likely pathogenic mutation together with a matched clinical diagnosis were used to represent a syndrome. We applied this classifier to a cohort of unsolved clinical cases to assess the diagnostic potentials of the RMNS DNA methylation episignatures (described in [17]).

### Methylation experiment and quality controls

Peripheral whole blood DNA was extracted using standard techniques. Following bisulfite conversion, DNA methylation analysis of the samples was performed using the Illumina Infinium methylation EPIC (850K) or 450K bead chip arrays (San Diego, CA), according to the manufacturer’s protocol. The resulting methylated and unmethylated signal intensity data were imported into R 3.5.1 for analysis. Normalization was performed using the Illumina normalization method with background correction using the *minfi* package [22]. Probes with detection *p* value > 0.01, those located on chromosomes X and Y, those known to contain SNPs at the CpG interrogation or single-nucleotide extension, and probes known to cross-react with chromosomal locations other than their target regions were removed. Arrays with more than 5% failure probe rate were excluded from the analysis. Sex of the subjects was predicted using the median signal intensities of the probes on the X and Y chromosomes and samples discordant between the labeled and predicted sex were not used for analysis. All of the samples were examined for genome-wide DNA methylation density, and those deviating from a bimodal distribution were excluded. Factor analysis using a principal component analysis (PCA) of all of the probes was performed to examine the batch effect and identify the outliers.

### Selection of matched controls for methylation profiling

All of the RMNS samples were assayed using the EPIC 850K array. Therefore, only controls assayed using the same platform were used for the analysis. Matching was done by age and sex using the *MatchIt* package [23]. For each patient, ten controls were selected from our database. This figure represented the largest number of controls available in our data to be matched to the patient group without impairing the matching quality. After every matching trial, a PCA was performed to detect outliers and examine the data structures. Outlier samples and those with aberrant data structures were removed before a second matching trial was conducted. The iteration was repeated until no outlier sample was detected in the first two components of the PCA.

### DNA methylation profiling

The analysis was performed according to our previously published protocol [14, 17]. The methylation level for each probe was measured as a beta value, calculated from the ratio of the methylated signals vs. the total sum of unmethylated and methylated signals, ranging between zero (no methylation) and one (full methylation). A linear regression modeling using the *limma* package was used to identify the differentially methylated probes [24]. For linear regression modeling, beta values were logit transformed to *M* values using the following equation: log_2_ (beta/(1-beta)). The analysis was adjusted for blood cell type compositions, estimated using the algorithm developed by Houseman and coworkers [25]. The estimated blood cell proportions were added to the model matrix of the linear models as confounding variables. The generated *p* values were moderated using the *eBayes* function in the *limma* package and were corrected for multiple testing using the Benjamini and Hochberg method. Probes with a corrected *p* value < 0.01 and a methylation difference greater than 10% were considered significant. The effect size cutoff of 10% was chosen to avoid reporting of probes with low effect size or those influenced by technical or random variations as conducted in our previous studies [14, 17].

### Clustering and dimension reduction

Following the analysis, the selected probes were examined using hierarchical clustering and multiple dimensional scaling to assess the structure of the identified episignature. Hierarchical clustering was performed using Ward’s method on Euclidean distance by the *gplots* package. Multiple dimensional scaling (MDS) was performed by scaling of the pair-wise Euclidean distances between the samples.

### Identification of the differentially methylated regions

To identify genomic regions harboring methylation changes (differentially methylated regions—DMRs), the DMRcate algorithm was used [26]. First, the *p* values were calculated for every probe using multivariable *limma* regression modeling. Next, these values were kernel smoothed to identify regions with a minimum of three probes no more than 1 kb apart and an average regional methylation difference > 10%. We selected regions with a Stouffer transformed false discovery rate (FDR) < 0.01 across the identified DMRs. The analysis was performed on the same sets of cases and controls used for methylation profiling and adjusted for blood cell type compositions.

### Functional analysis of differentially methylated regions

We analyzed the expression profiles of the DMRs-associated genes in 416 tissues/organs by means of large curated dataset of 65761 Affymetrix Human Genome U133 Plus 2.0 Array in Genevestigator V.7.3.1 tool (Nebion, Switzerland), and classified them by hierarchical clustering technique using Pearson correlation as similarity measure and optimal-leaf ordering. Gene-Set enrichment analysis was performed using latest Reactome annotations [27].

### Construction of a classification model for Rahman syndrome

To examine the level of overlap and sensitivity of the RMNS episignature to confounding factors such as age, sex, blood cell type compositions, and other developmental disorders, as well as to screen among unresolved patients, a supervised algorithm was developed. Given the majority of the samples to be tested were assayed using 450k array, we limited the analysis to probes shared by both array types. A “random forest” classifier was trained on the same set of patients and controls used previously using the *caret* package. A ten-fold cross validation was performed during the training to choose the best hyperparameter (mtry). Default values were used for other parameters. Based on the number of trees in this classifier voting for each of the two classes (RMNS vs. controls), the model allows for assigning a confidence score for the classification. Therefore, for each methylation profile supplied to the model, a value ranging 0–1, representing the confidence in predicting whether the subject has a DNA methylation profile similar to RMNS, was generated. By default, 0.5 is considered the classification cutoff. The final model was first applied to the training datasets to ensure the success of the training. To confirm that the classifier is not sensitive to the blood cell type compositions, we applied this model to methylation data from isolated cell populations of healthy individuals from gene expression omnibus (GEO) (GSE35069) [28] and supplied them to the classification model for prediction and examined the degree to which the scores were varied across different blood cell types. To determine the specificity of the model, we applied it to a DNA methylation array data form a cohort of healthy subjects. To understand whether this model was sensitive to other disorders caused by mutations in genes encoding proteins with role in epigenetic control and chromatin remodeling, we assessed data from a cohort of subjects with a confirmed clinical and molecular diagnosis of such syndromes. The validated model was used to screen for RMNS among a large group of individuals with various forms of neurodevelopmental presentations but no established diagnosis despite routine clinical and molecular assessments.

## Results

### RMNS generates a hypomethylated DNA methylation episignature

**Fig. 1:**
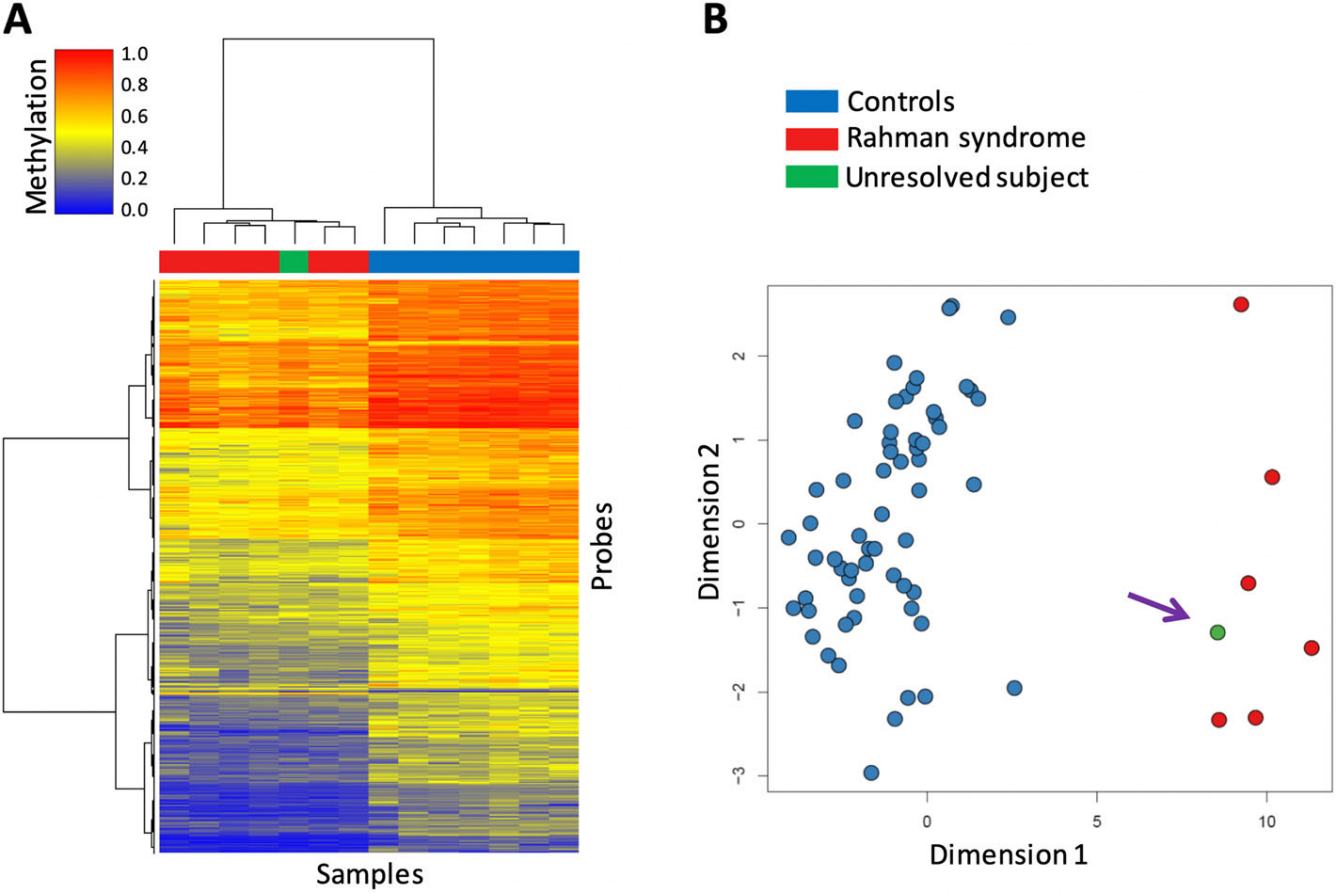
A specific episignature characterizes individuals affected by Rahman syndrome. **a** The DNA methylation profile of a set of seven healthy controls and seven affected individuals (including six patients with previously confirmed molecular diagnosis of Rahman syndrome and one previously undiagnosed subject) is visualized using hierarchical clustering analysis. Rows represent all of the differentially methylated CpG sites (~ 9000) and columns indicate the samples. The color scheme of the top panel is indicative of the class. Red, Rahman syndrome; Blue, controls; Green, undiagnosed individual. The heatmap color scale from blue to red represents the range of the methylation levels (beta values) between 0 and 1. Clustering is performed using Ward’s method on Euclidean distance. **b** The first two dimensions from multidimensional scaling (MDS) of the DNA methylation levels at CpG sites differentially methylated in Rahman syndrome (RMNS) completely separate all of the patients (red) and controls (blue) from each other. Addition of a subject later identified from a cohort of unresolved DD/ID patients (green—indicated with an arrow) to this analysis, clusters the proband with other RMNS. MDS was calculated by scaling of the pair-wise Euclidean distances between the samples

The study included six subjects with molecularly confirmed diagnosis of RMNS, sharing functionally equivalent frameshift mutations at the C-terminus of HIST1H1E (Table 1). For each patient, ten age- and sex-matched healthy controls (total *N* = 60) were selected for comparison. Following DNA methylation profiling of peripheral blood on Infinium EPIC arrays, a total of 840120 CpG sites (probes) passed the quality control criteria and were retained for analysis. The comparison identified 9553 differentially methylation CpGs between the patients and controls (*limma* regression modeling, > 10% methylation difference, and false discovery rate (FDR) < 0.01, adjusted for blood cell type compositions). Notably, from these probes, only 438 (< 5%) exhibited relative hypermethylation (Additional file 3: Table S1). Hierarchical clustering demonstrated a distinctive hypomethylation pattern among the patients relative to controls (Fig. 1a). To confirm that the observed pattern was not representative of an experimental batch effect, we assessed four healthy control samples, which had been processed on the same microarray batch as the patients, to the analysis, all of which were observed to cluster together and show a methylation pattern similar to controls for the differentially methylated probes (Fig. 1b). Mapping of DMRs harboring more than three consecutive CpGs (average regional methylation difference > 0.1, FDR < 0.01, adjusted for blood cell type compositions) identified DNA methylation changes at 616 genomic coordinates (hg19), all of which demonstrated relative hypomethylation in affected subjects, except for one slightly hypermethylated (Additional file 3: Table S2 and Additional file 1: Figure S1).

**Table 1:** Frameshift *HIST1H1E* mutations of the studied RMNS cohort

Nucleotide change	gnomAD	Amino acid change	Domain	CADD^a^	Subject
c.408dupG	–	p.Lys137GlufsTer59	C-terminal tail	34	S12
c.414dupC	–	p.Lys139GlnfsTer57	C-terminal tail	35	S4
c.430dupG	–	p.Ala144GlyfsTer52	C-terminal tail	26.8	S13
c.435dupC	–	p.Thr146HisfsTer50	C-terminal tail	25.3	S14
c.441dupC	–	p.Lys148GlnfsTer48	C-terminal tail	34	S1, S5

Nucleotide numbering reflects cDNA numbering with 1 corresponding to the A of the ATG translation initiation codon in the *HIST1H1E* reference sequence (RefSeq: NM_005321.2, NP_005312.1)

^a^CADD v1.4. All patients belong to the cohort reported by Flex et al. (4)

### Episignature of RMNS is specific and independent of confounding factors

**Fig. 2:**
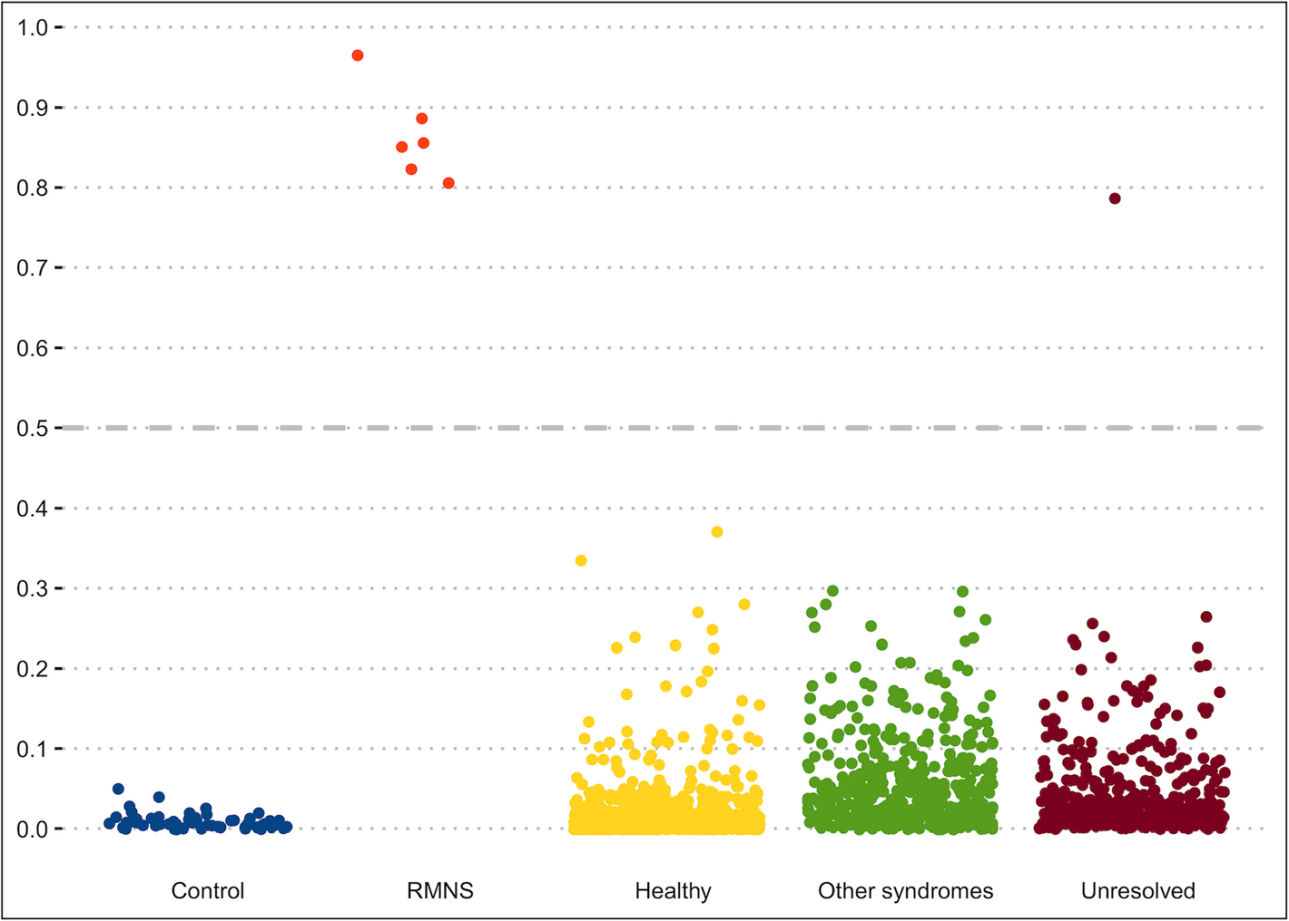
A classification model using DNA methylation data yields full sensitivity and specificity in classifying patients with Rahman syndrome. Each panel on the *x*-axis illustrates testing for a group of subjects with a distinct phenotype, as indicated on bottom of the panel. *Y*-axis represents scores generated by the classifier for different subjects as indicated by points on the plot. The scores range 0–1, with higher scores indicating a higher chance of having a methylation profile similar to Rahman syndrome (RMNS) (*y*-axis). By default, the classifier utilizes a cutoff of 0.5 for assigning the class; however, the vast majority of the tested individuals received a score close to 0 or 1. Therefore, for the purpose of better visualization, the points are jittered. Control (blue): 60 controls used to describe the signature and train the model; RMNS (red): six patients with RMNS used for identification of the episignature and training of the classifier; Healthy (yellow): 1678 controls used to measure the specificity of the model; Other syndromes (green): 502 patients with confirmed clinical and molecular diagnosis of various Mendelian disorders resulting from defects in epigenetic machinery; Unresolved (maroon): 453 patients with developmental abnormalities but without a diagnosis at the time of assessment

We assessed whether the epigenetic signature of RMNS is independent of factors that influence the DNA methylome such as age, sex, blood cell type composition, and whether the identified signature is specific and distinguishable from the DNA methylation patterns characterizing other developmental syndromes. Using all probes identified to be differentially methylated in RMNS, we trained a “random forest” classifier on the initial set of patients and controls used for discovery. The classifier was set to generate a score 0–1 for each test subject to indicate the probability of a methylation profile similar to RMNS. We first applied this classifier to 1678 whole blood methylation data from healthy males and females of various ethnic backgrounds (aged 2–94) (GSE42861, GSE87571, and GSE99863), all of which received low scores for RMNS and were classified as controls (Fig. 2). Next, we applied the model to an offset of 60 methylation array data files from six healthy individuals, each being assayed separately for whole blood, peripheral blood mononuclear cells, and granulocytes, as well as for seven isolated cell populations (CD4^+^ T, CD8^+^ T, CD56^+^ NK, CD19^+^ B, CD14^+^ monocytes, neutrophils, and eosinophils). All of these samples were classified as controls with a negligible inter-cell-type variability in the scores (Additional file 3: Table S3). Finally, we evaluated the specificity of RMNS in relation to other neurodevelopmental syndromes by applying the RMNS classifier to a total of 502 samples with a confirmed diagnosis of various syndromes including imprinting defect disorders (Angelman syndrome, Prader-Willi syndrome, Silver-Russell syndrome, and Beckwith-Wiedemann syndrome), BAFopathies (Coffin-Siris and Nicolaides-Baraitser syndromes), autosomal dominant cerebellar ataxia, deafness and narcolepsy, Floating-Harbor syndrome, Cornelia de Lange syndrome, Claes-Jensen syndrome, Helsmoortel-Van der Aa syndrome, ATRX syndrome, Kabuki syndrome, CHARGE syndrome, Fragile X syndrome, trisomy 21, Williams syndrome, and Somerville-Van der Aa syndrome, most of which are known to have their own DNA methylation episignatures [14, 17]. All specimens received low scores, indicating that their methylation profile does not resemble that of RMNS (Fig. 2), further demonstrating the specificity of the identified episignature for RMNS.

### Screening of an unresolved patient cohort using the episignature of RMNS

To test the use of the newly identified episignature in a clinical setting, we applied the RMNS classifier to a previously described cohort of specimens [17] with various developmental disorders who have remained unresolved following the routine clinical assessments. We assessed 453 subjects the majority of who had undergone CNV microarray testing as part of the standard clinical workup along with additional genetic testing in some cases, including targeted gene/panel or exome sequencing. These individuals presented with various forms of neurodevelopmental/multiple congenital anomalies disorders, including facial dysmorphism, DD/ID, degenerative neural disease, autism, and congenital heart and other organ defects, though none were suspected to have RMNS. Applying our classifier to this cohort, we classified one patient as a potential case of RMNS (Fig. 2; score = 0.78, maroon color). Separate assessments using hierarchical clustering and multiple dimensional scaling revealed that this case had a DNA methylation profile consistent with other confirmed RMNS cases (Fig. 1). The subject was a 2-year-old male with hypotonia, DD, feeding difficulties, benign external hydrocephalus of infancy, left-sided undescended inguinal testicle and right-sided retractile testicle, bilateral tympanostomy, and ventriculomegaly. Sequence variant assessment of the coding regions in 4600 genes considered to be involved in Mendelian genetic disorders as of the year 2015 was reported negative (LHSC MedExome research analysis). Subsequently, patient was offered a trio full exome sequencing in which a truncating variant was found in the *HIST1H1E* gene (c.436_458del, p.Thr146AspfsTer42; RefSeq: NM_005321.2, NP_005312.1), confirming the diagnosis of RMNS and the sensitivity of the generated DNA methylation episignature.

### Episignature of RMNS is enriched with genes involved in neural signal transduction

**Fig. 3:**
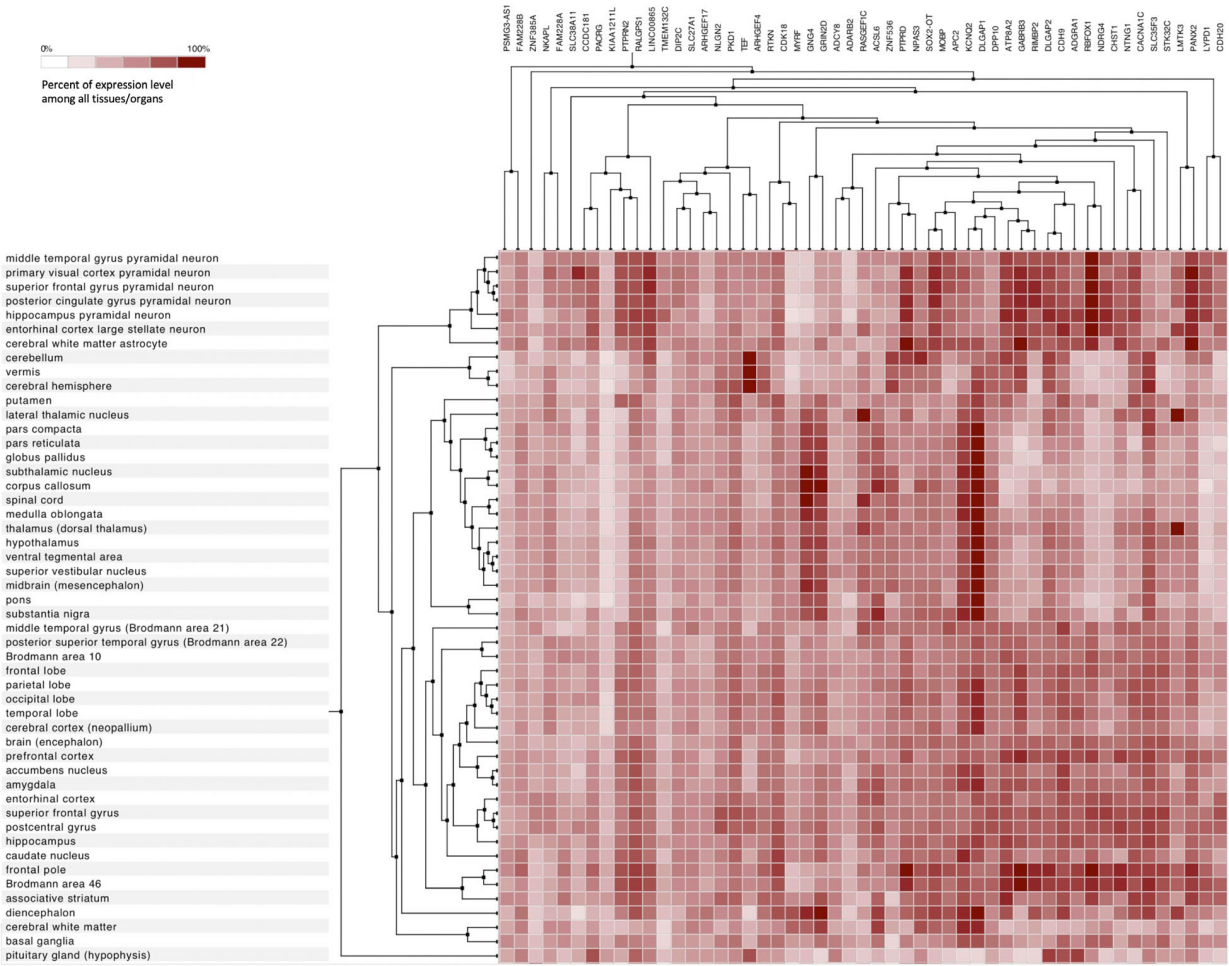
Brain-specific expression patterns for hypomethylated genes in Rahman syndrome. Gene expression profiles in brain tissues extracted from Additional file 2: Figure S2 (highlighted by the black square). Data are obtained from 65761 Affymetrix Human Genome U133 Plus 2.0 arrays in Genevestigator; hierarchical clustering is performed using Pearson correlation as similarity measure and optimal-leaf ordering

**Fig. 4:**
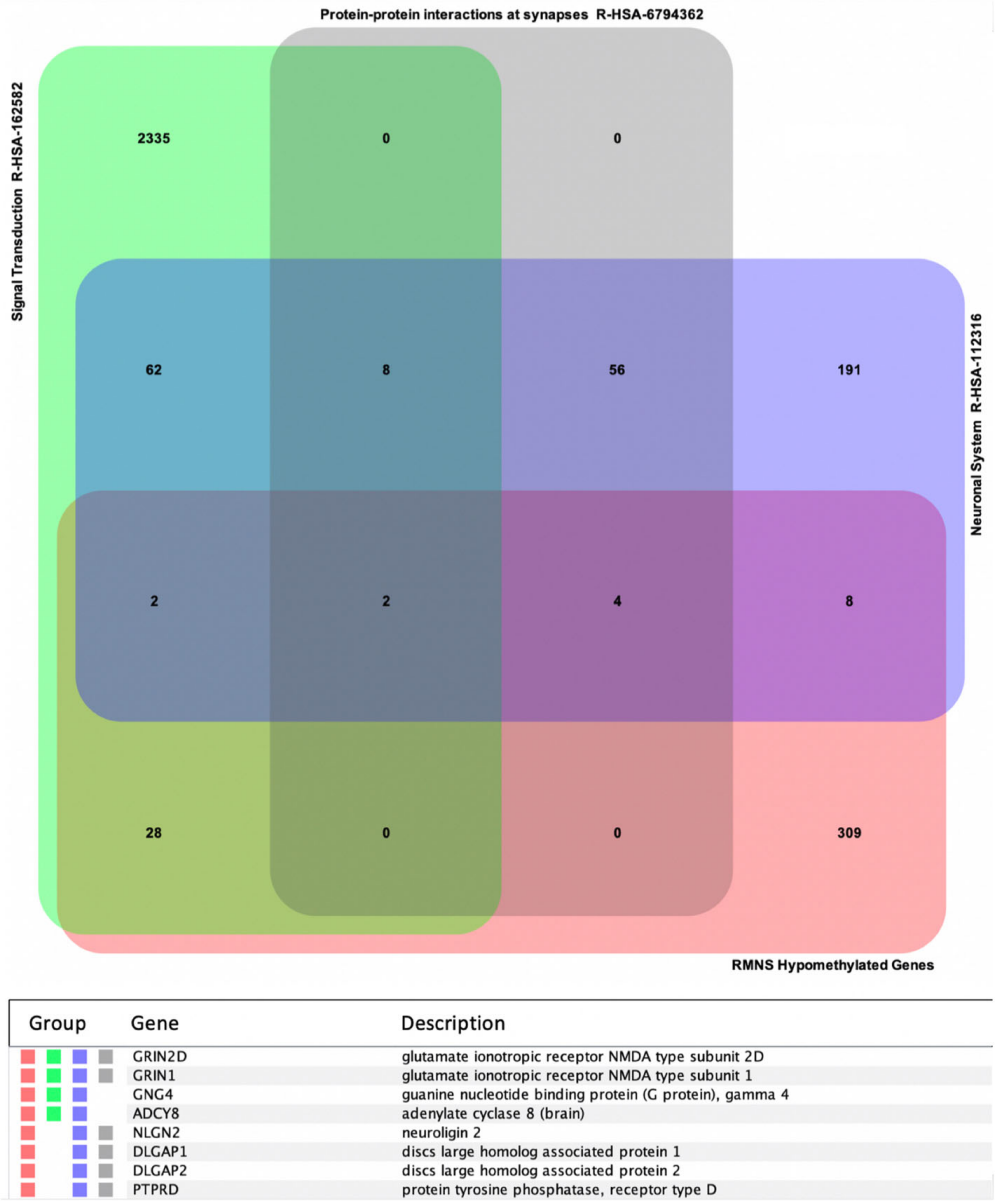
Functional characterization of hypomethylated genes in Rahman syndrome. Venn diagrams showing overlap among genes with hypomethylated regions in Rahman syndrome (RMNS) and Reactome pathways. In the diagram on top are depicted statistically significant-enriched gene-sets affecting neuronal pathways, extracted from Reactome, as described in Additional file 3: Table S4. The table on the bottom shows genes belonging to at least three groups

While replicative senescence is expected to have a major impact in most tissues of subjects with RMNS, we hypothesized that an altered control of gene expression associated with the aberrant methylation profile characterizing RMNS may significantly contribute to altered cellular function in postmitotic cells. Mapping DMRs on genomic coordinates allowed us to identify genes showing differential methylation levels in the affected subjects (Additional file 3: Table S2). To functionally characterize this gene-set, we took advantage of a large curated gene expression dataset (~ 65000 Affymetrix arrays on 416 anatomical parts) to identify co-expression profiles in different human organs/tissues. This analysis indicated that a major co-expression cluster involved genes that are highly expressed in brain tissues (Additional file 2: Figure S2; Fig. 3). Gene-set enrichment analysis based on Reactome dataset [27] also identified four significantly enriched groups (FDR < 0.01), including neuronal system, metabolism, signal transduction, and protein-protein interactions at synapses (Additional file 3: Table S4). According to this classification, eight genes with a significant hypomethylation profile were identified to be involved in neuronal signal transduction, mostly at synaptic level (i.e., *GRIN1*, *GRIN2D*, *GNG4*, *ADCY8*, *NLGN2*, *DLGAP1*, *DLGAP2*, and *PTPRD*) [29–36] (Fig. 4). Notwithstanding the occurrence of cell lineage specificity in the establishment of dynamic methylation pattering does require the generation of a more informative model system (e.g., iPSC-derived neuronal lines), these data suggest that altered neuronal function in RMNS may depend, at least in part, on dysregulated gene expression of key genes in neuronal cells.

## Discussion

We recently characterized the phenotypic profile of RMNS and the functional consequences of the frameshift *HIST1H1E* mutations underlying this trait [4]. The clinical profile of RMNS includes DD and ID, a distinctive facies, and features of accelerated aging. While the facial gestalt may help in recognition of the disorder, we noted that no pathognomonic features can be used for a definitive diagnosis based on clinical criteria. The dominantly acting mutations were found to alter chromatin compaction, disrupt nuclear lamina organization, and cause accelerated senescence, an endophenotype mirroring the signs of accelerated aging in patients. A first analysis directed to assess any epigenetic impact of *HIST1H1E* mutations allowed to document a different DNA methylation pattern in affected subjects compared to controls. Here, we expand further our previous analysis providing evidence for the occurrence of a specific episignature in RMNS.

In the recent years, genome-wide methylation array analysis has allowed to identify and characterize episignatures for an increasing number of diseases [9–16]. This epigenetic profiling has successfully been used to screen large cohorts of individuals with clinically unrecognized and complex traits, and clarify the clinical relevance of variants of uncertain significance emerged from genomic sequencing. In addition to classifying individual samples and variants, epigenetic profiling has proven to be useful for disease categorization, as recently showed for Coffin-Siris and Nicolaides-Baraitser syndromes [12]. We demonstrate that RMNS is characterized by a highly sensitive and specific episignature, which is defined by a particular hypomethylation profile with respect to healthy subjects. Currently, only a small number of patients with RMNS have been described in literature. All patients have been showed to carry functionally equivalent frameshift *HIST1H1E* mutations affecting the C-terminus of the protein. Other nonsynonymous variants, however, may present challenges for assessment of clinical impact on the protein function. In such cases, this RMNS epigenetic classifier may provide critical information to enable classification of such variants and ultimately a precise diagnosis, or alternatively to rule out a possible diagnosis of this syndrome.

Consistent with the recently collected data [4], the methylome analysis did not highlight a substantial change in the global methylation pattern in RMNS, with only 9553 CpG sites (1.1% of total probes) showing a differential methylation status between the patients and controls. These findings are in line with previous studies performed in cells with loss of histone H1 function documenting a minor impact on global DNA methylation [8]. In these cells, changes rather involved specific CpGs in regulatory regions, indicating a punctual effect on a relatively small subset of genes and cellular processes. In agreement with the data collected by Fan and colleagues, less than 5% of the differentially methylated probes was represented by a hypermethylation change, indicating that the changes in the methylation status driven by *HIST1H1E* mutations concern a global tendency in a reduction of methylation.

With the aim of exploring the functional impact of the differentially methylated regions in individuals presenting with RMNS, we observed that a relevant proportion of the genes containing these hypomethylated regions are predominantly expressed in brain. Among them, gene-set enrichment analysis highlighted distinctive hypomethylation pattern affecting genes encoding *N*-methyl-d-aspartate receptors (GRIN1, GRIN2D), G proteins (GNG4), adenylyl cyclases (ADY8) neuroligins (NLGN2), discs large associated proteins (DLGAP1/2), and receptor-type protein tyrosine phosphatase D (PTPRD), suggesting that chromatin remodeling driven by aberrant HIST1H1E function may result in a dysregulated epigenetic control of genes encoding proteins with role in synaptic transmission and neuronal function. *GRIN1* and *GRIN2D* encode different subunits of the M-methyl-d-aspartate (NMDA) receptor, which is a heteromeric glutamate-gated calcium ion channel essential for synaptic function in the brain [29, 30]. Similarly, GNG4 has been linked functionally to synaptic plasticity and cognitive function [31, 32], whereas adenylyl cyclases have been described to modulate markers of synaptic activity [33]. In the same way, neuroligins function as trans-synaptic adhesion molecules with a known role in synaptogenesis [34] and DLGAP1-4 interacts with members of the PSD95 family, NMDA receptors, and Shaker-type potassium channels to contribute to homeostatic synaptic plasticity [35]. While studies using informative in vitro and in vivo models to consider the proper cellular context are needed to dissect in deeper detail the molecular pathways involved in RMNS, the present findings suggest that dysregulation of these genes (and/or other genes whose expression in neuronal cells is controlled by HIST1H1E-mediated regulation of chromatin organization) may contribute to neurogenesis defects and/or abnormalities of synaptic plasticity in patients with RMNS. Remarkably, the present findings are in line with the data collected from the recent effort directed to identify episignatures for a large number of syndromic disorders with DD/ID, indicating that the overlap of these syndrome-specific epigenetic signatures is limited to a few genes and genomic regions [14, 17]. This finding suggests that these episignature could represent informative tools to be used to implement new multi-class computational models to gain new insights into disorders affecting the epigenetic machinery, helping to reclassify all of them on a functional basis.

In the past decades, epigenomics approaches have been mostly limited to research applications; recently, new technologies and data-driven strategies have made it possible the implementation of routine genome-wide DNA methylation testing in the clinical management of Mendelian conditions [37, 38]. Currently, there are 35 syndromes with defined episignatures [14, 17], and in most cases genomic methylation analysis is able to identify patients with these disorders, who may not be molecularly confirmed through standard genetic assessment including exome sequencing [12]. Moreover, DNA methylation microarray technology currently assesses ~850K CpG sites across the genome and provides an adequate gene-level resolution with advantages in terms of data management, interpretation, and costs compared to more comprehensive approaches (e.g., bisulfite genome sequencing), without suffering from analytical sensitivity taking into account all types of genetic variation. Current analytical pipelines make methylome datasets robust and highly reproducible in sample-to-sample and batch-to-batch comparisons, and consistent across age groups [17]. Moreover, the technology is scalable, enabling the assessment of large sample batches by means of application of automated algorithms, which is a logistical requirement as part of a routine screening protocol. It should be considered that while the use of DNA obtained from peripheral blood samples makes this assay easily supported by current diagnostic infrastructures, a limitation of this tool may concern the low tissue-specific resolution for a subset of disorders (e.g., Beckwith-Wiedemann syndrome) [39]. Further investigations and development of reference datasets in other accessible tissue types, such as buccal epithelium or fibroblasts, is needed to extend the utility of this assay from peripheral blood to other tissues.

## Conclusions

Overall, we provide evidence that RMNS is characterized by a sensitive and specific epigenetic signature, which could be used both to dissect molecular mechanisms contributing to disease pathogenesis and applied to diagnostic workflows for individuals with uncertain conditions or affected by disorders with partial clinical overlap to RMNS.

## Supplementary information

**Additional file 1: Figure S1.** Differentially methylated regions in Rahman syndrome (RMNS). Plots display chromosome coordinates vs CpG site’s methylation level, for RMNS samples (red) and controls (blue).

**Additional file 2: Figure S2.** Tissue-specific expression profiles for hypomethylated genes in Rahman syndrome (RMNS). The expression profiles from 416 tissues/anatomical parts are calculated using 65761 Affymetrix Human Genome U133 Plus 2.0 arrays in Genevestigator; hierarchical clustering is performed using Pearson correlation as similarity measure and optimal-leaf ordering. Black square highlights gene cluster with maximum expression in brain tissues (displayed in Fig. 3).

**Additional file 3: Table S1.** CpG sites showing differential methylation levels between Rahman syndrome (RMNS) and controls. **Table S2.** Regions showing differential methylation levels between RMNS and controls. **Table S3.** Classification scores generated for various blood cells. **Table S4.** Gene-set enrichment analysis on Reactome pathways for genes with hypomethylated regions in RMNS.

## Data Availability

The RMMS patients’ publically available microarray data sources mentioned in the study can be obtained from Gene Expression Omnibus (GEO).

## Abbreviations

DD: Developmental delay
DMR: Differentially methylated region
FDR: False discovery rate
GEO: Gene expression omnibus
ID: Intellectual disability
MDS: Multidimensional scaling
PCA: Principal component analysis
VUS: Variant(s) of uncertain significance
