# Supplementary material for: Frameshift mutations at the C-terminus of HIST1H1E result in a specific DNA hypomethylation signature
Source: Clin Epigenetics. 2020 Jan 7;12:7. doi: 10.1186/s13148-019-0804-0 (PMC6947958; doi:10.1186/s13148-019-0804-0)
Supplement: Supplementary file 1 — Additional file 1: Figure S1. Differentially methylated regions in Rahman syndrome (RMNS). Plots display chromosome coordinates vs CpG site’s methylation level, for RMNS samples (red) and controls (blue). [file 13148_2019_804_MOESM1_ESM.pdf]

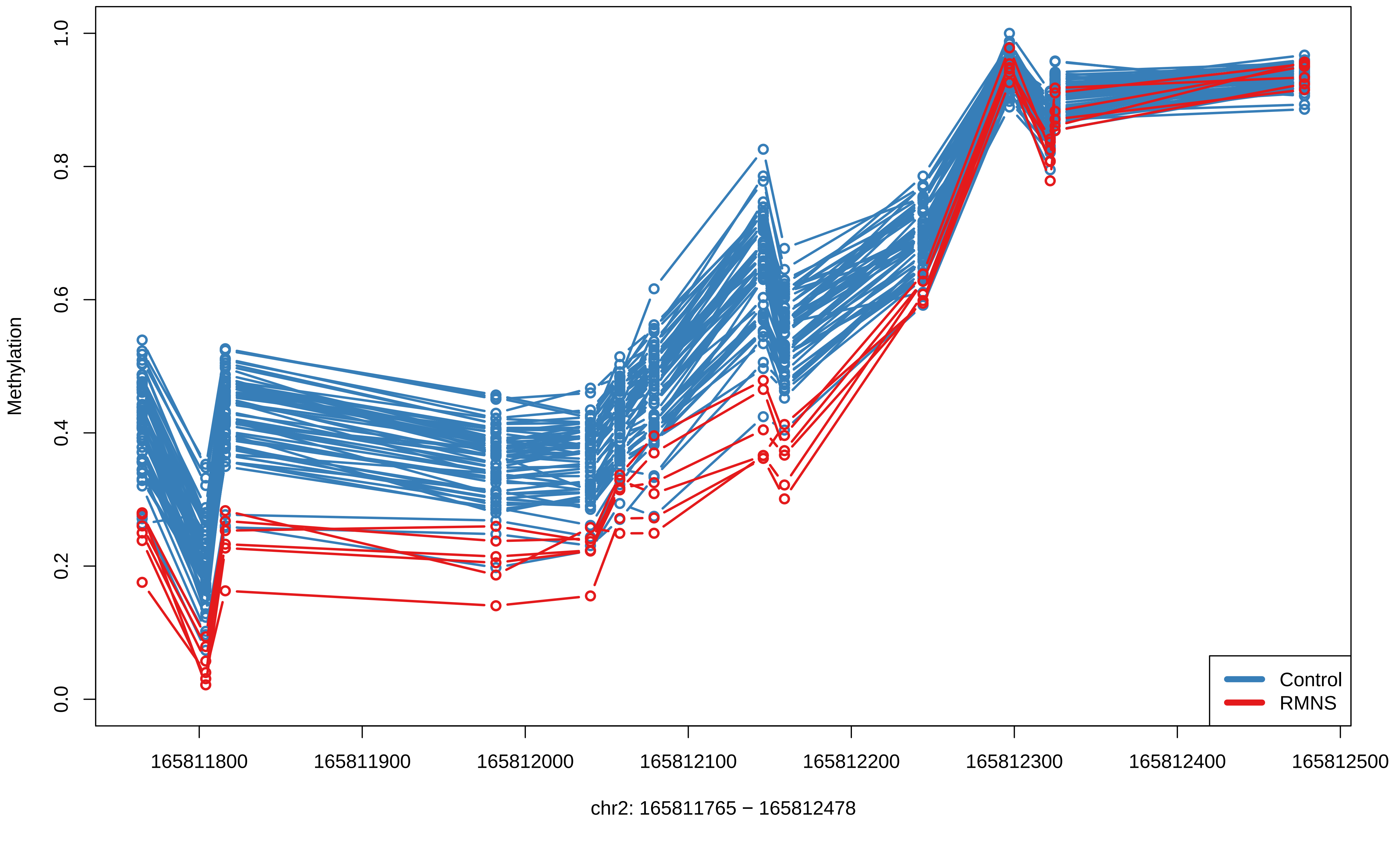

Methylation
0.0
0.2
0.4
0.6
0.8
1.0
165811800
165811900
165812000
165812100
165812200
165812300
165812400
165812500
chr2: 165811765 − 165812478
Control
RMNS

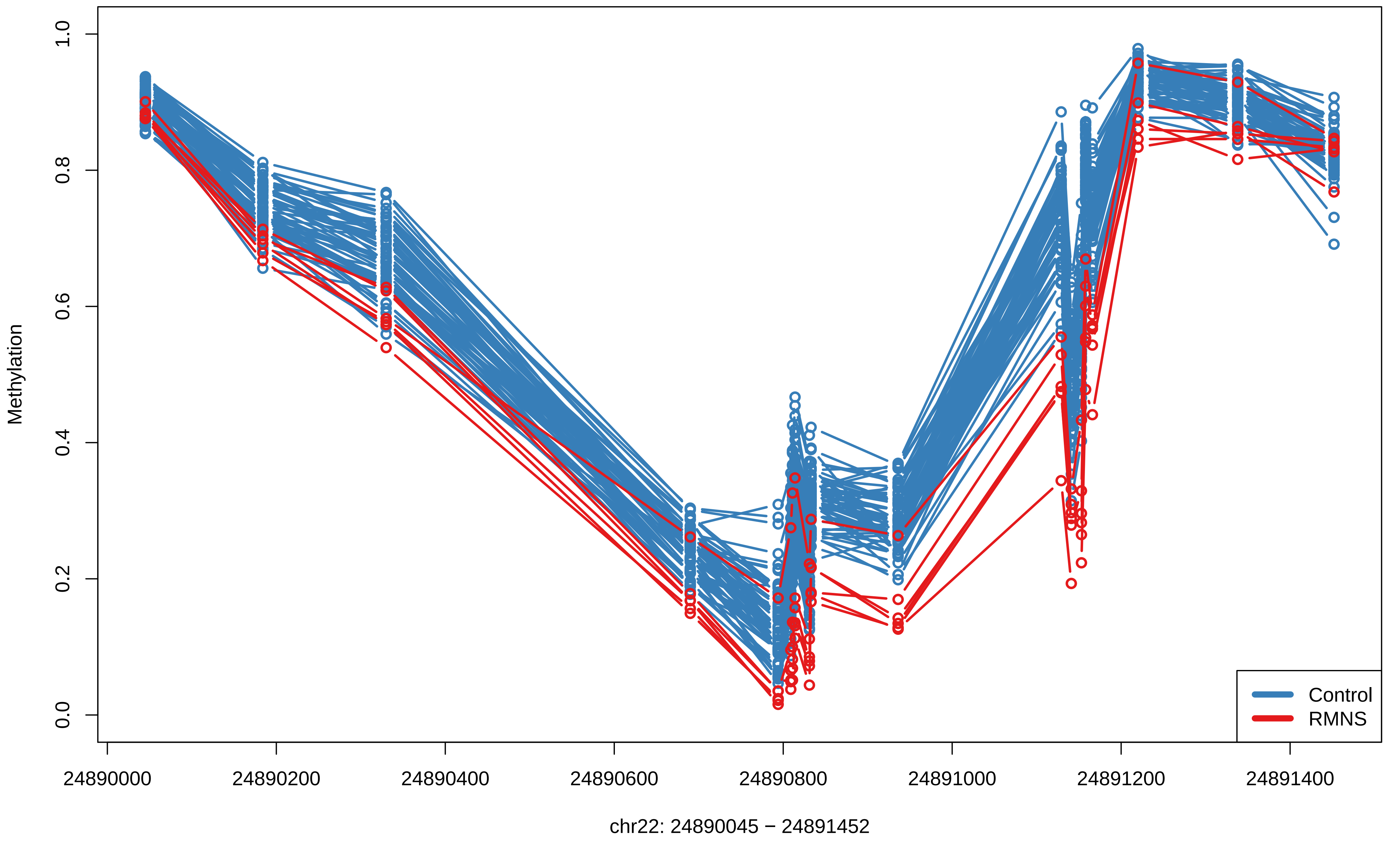

Methylation
0.0
0.2
0.4
0.6
0.8
1.0
24890000
24890200
24890400
24890600
24890800
24891000
24891200
24891400
chr22: 24890045 − 24891452
Control
RMNS

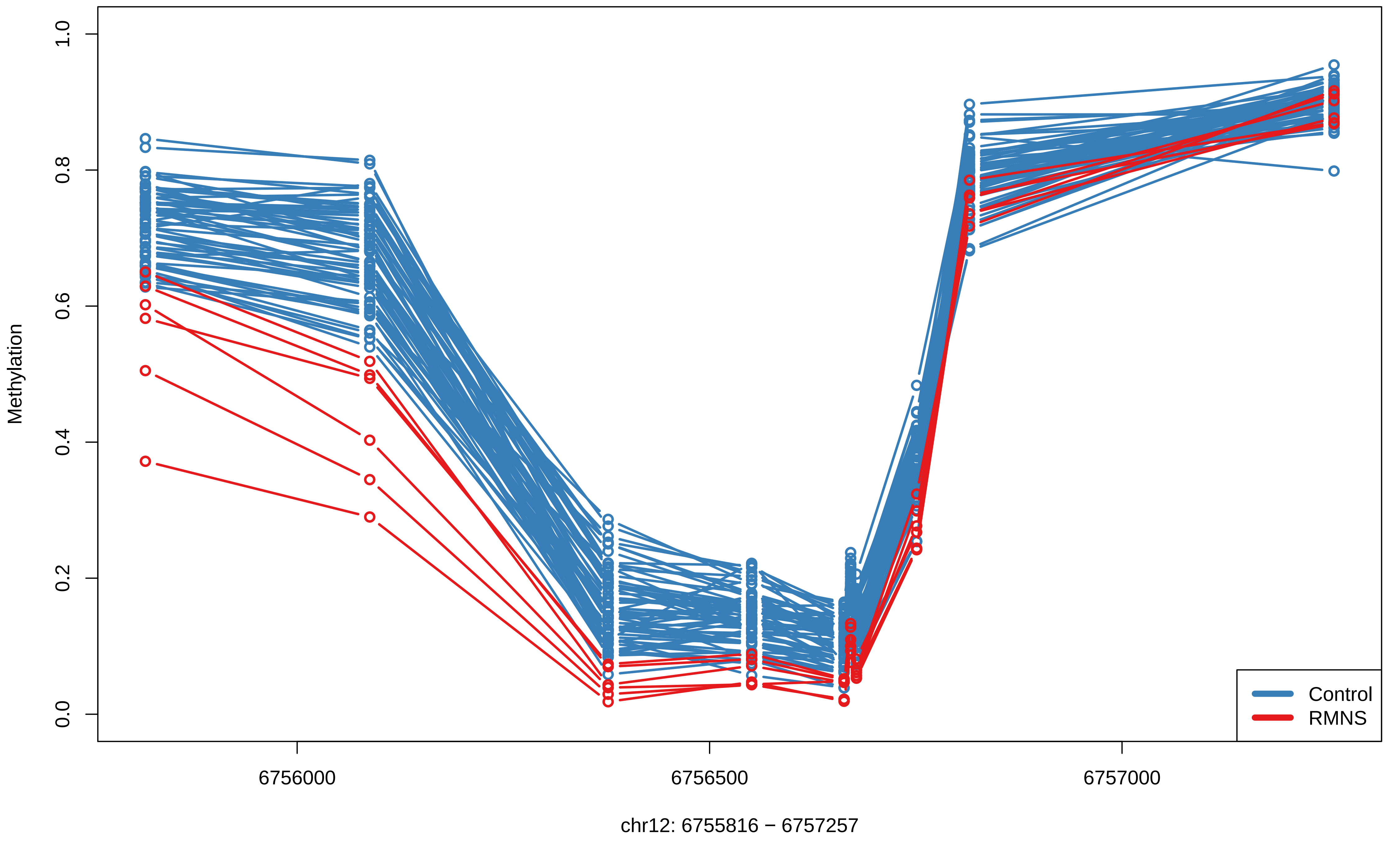

Methylation
0.0
0.2
0.4
0.6
0.8
1.0
6756000
6756500
6757000
chr12: 6755816 − 6757257
Control
RMNS

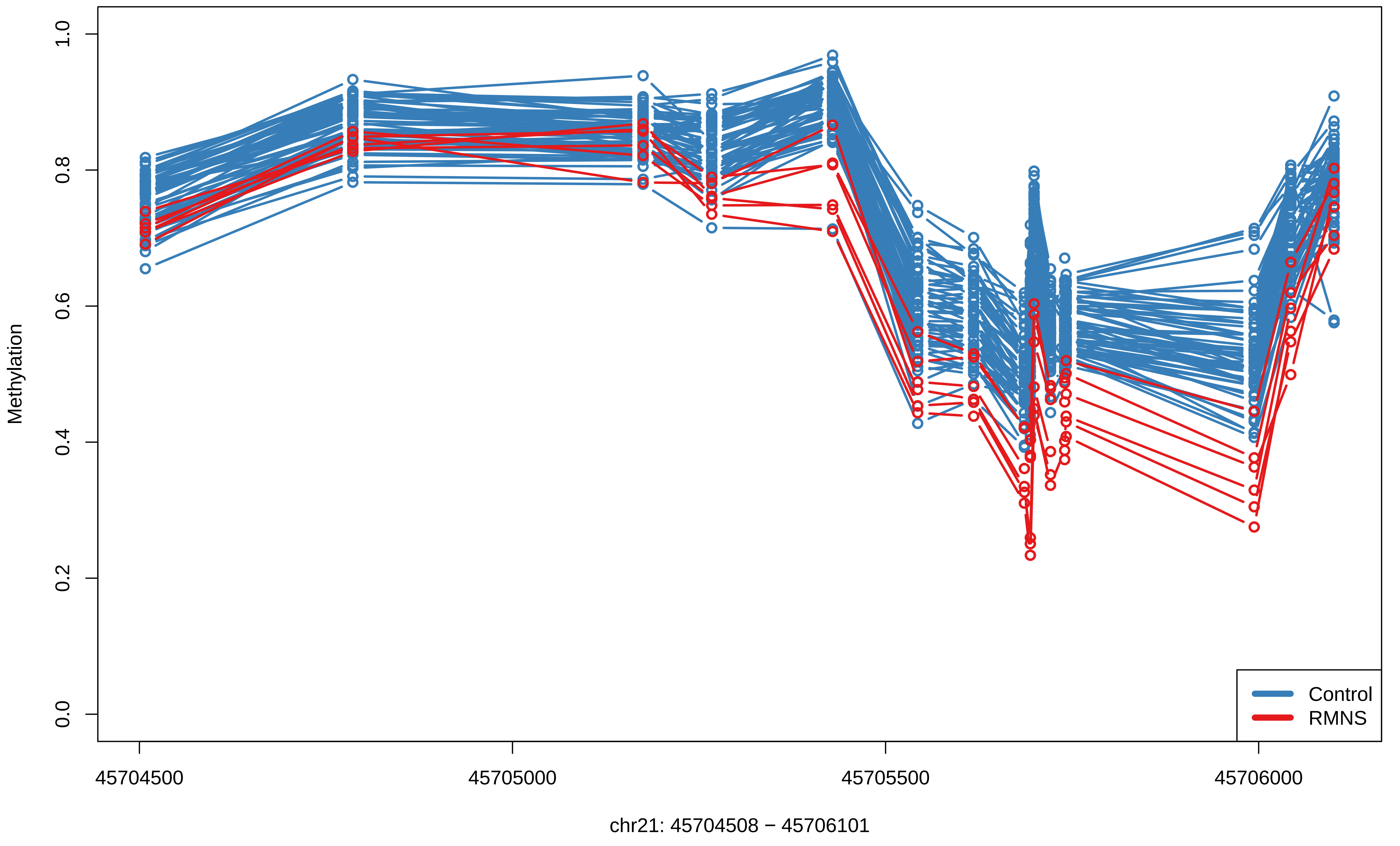

Methylation
1.0
0.8
0.6
0.4
0.2
0.0
45704500
45705000
45705500
45706000
chr21: 45704508 − 45706101
Control
RMNS

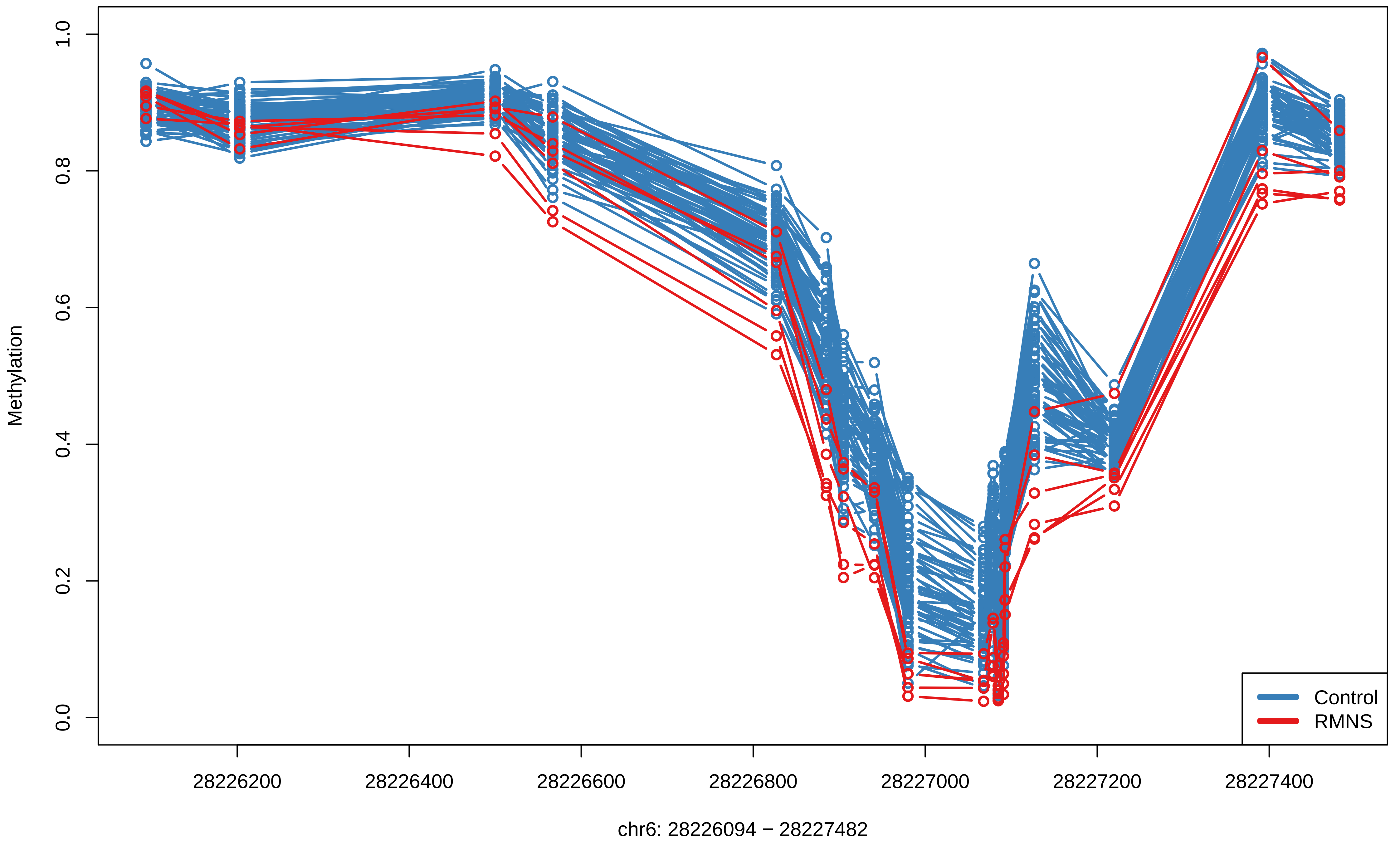

Methylation
1.0
0.8
0.6
0.4
0.2
0.0
28226200
28226400
28226600
28226800
28227000
28227200
28227400
chr6: 28226094 − 28227482
Control
RMNS

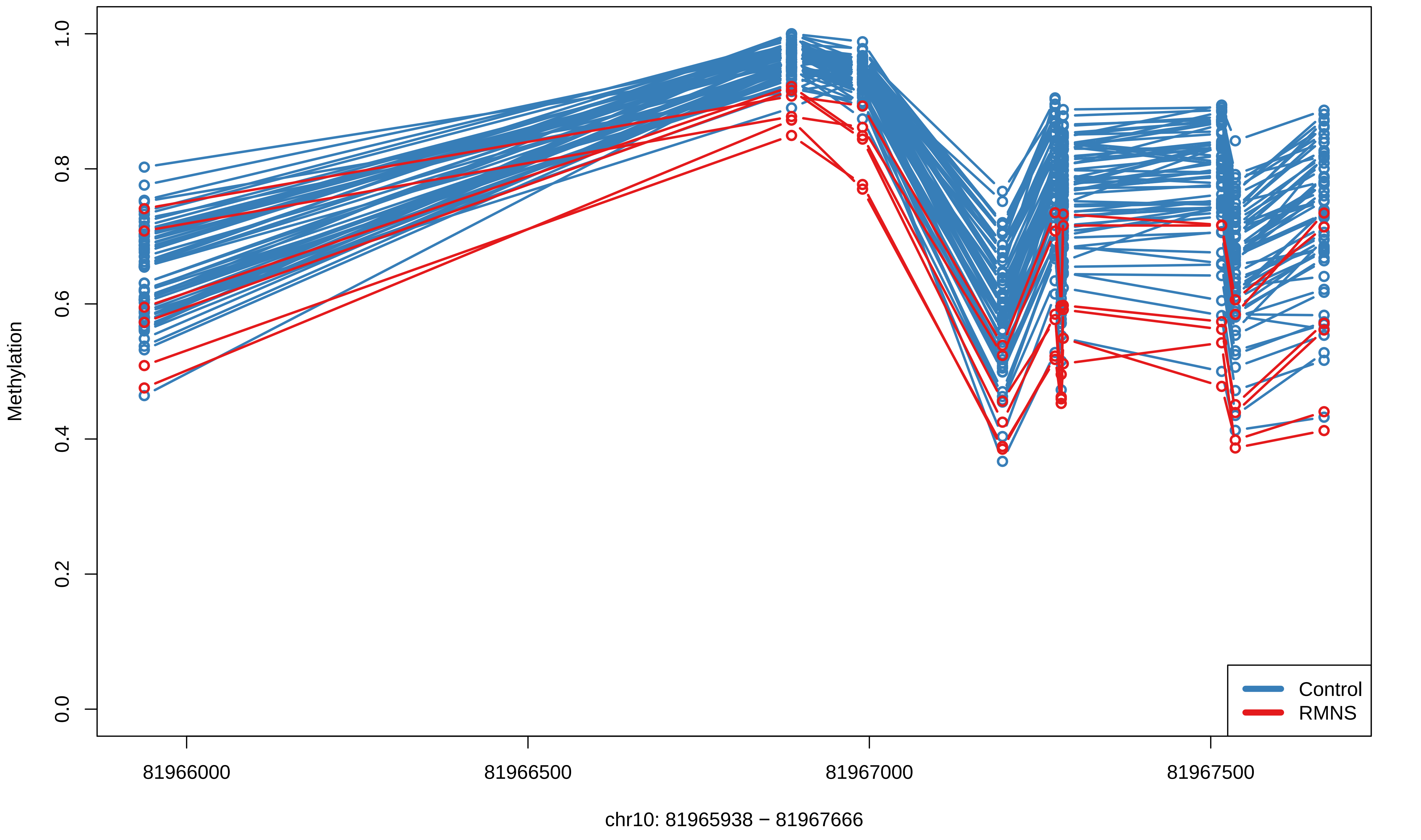
Methylation
1.0
0.8
0.6
0.4
0.2
0.0
81966000
81966500
81967000
81967500
chr10: 81965938 − 81967666
Control
RMNS

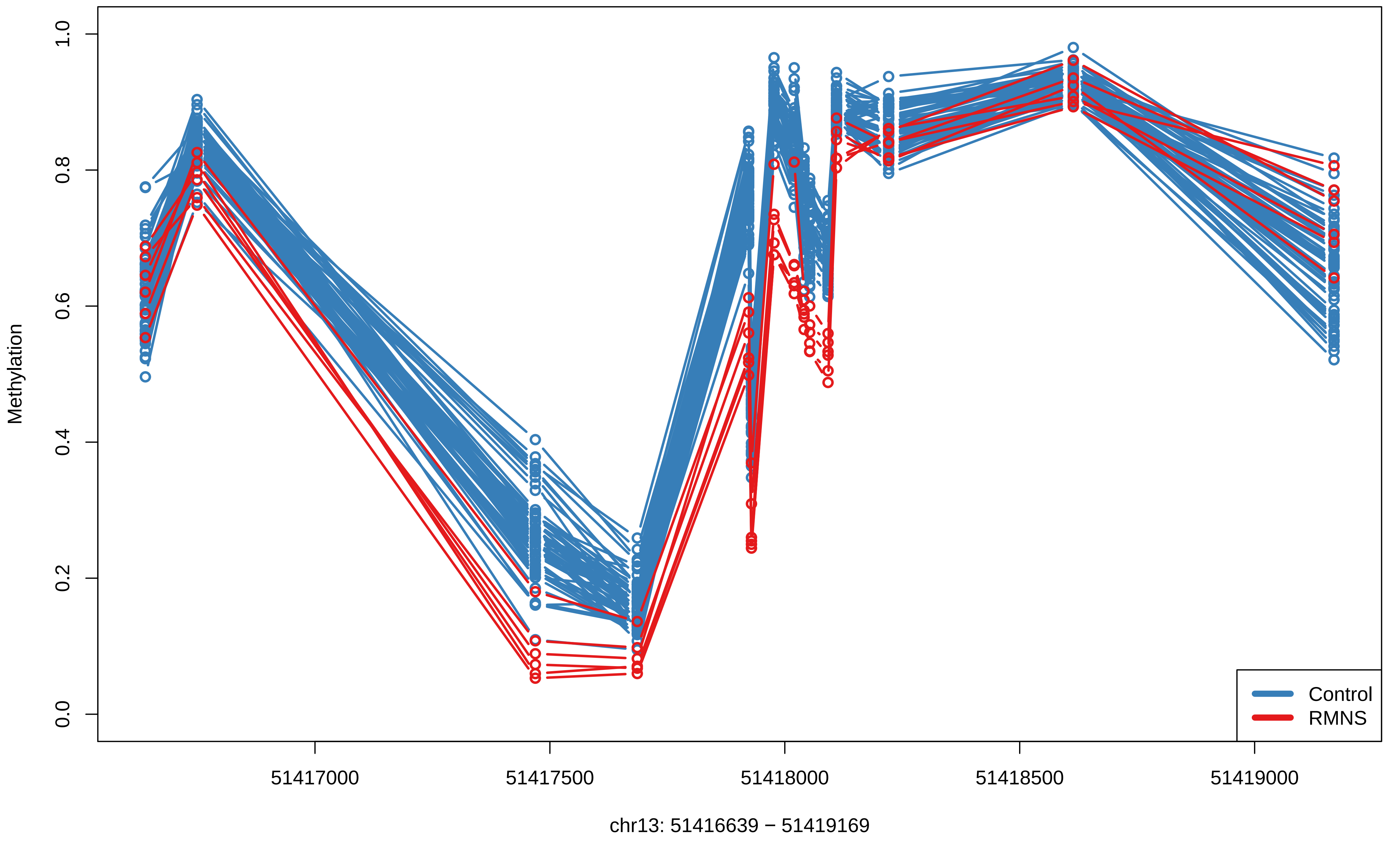

Methylation
0.0
0.2
0.4
0.6
0.8
1.0
51417000
51417500
51418000
51418500
51419000
chr13: 51416639 − 51419169
Control
RMNS

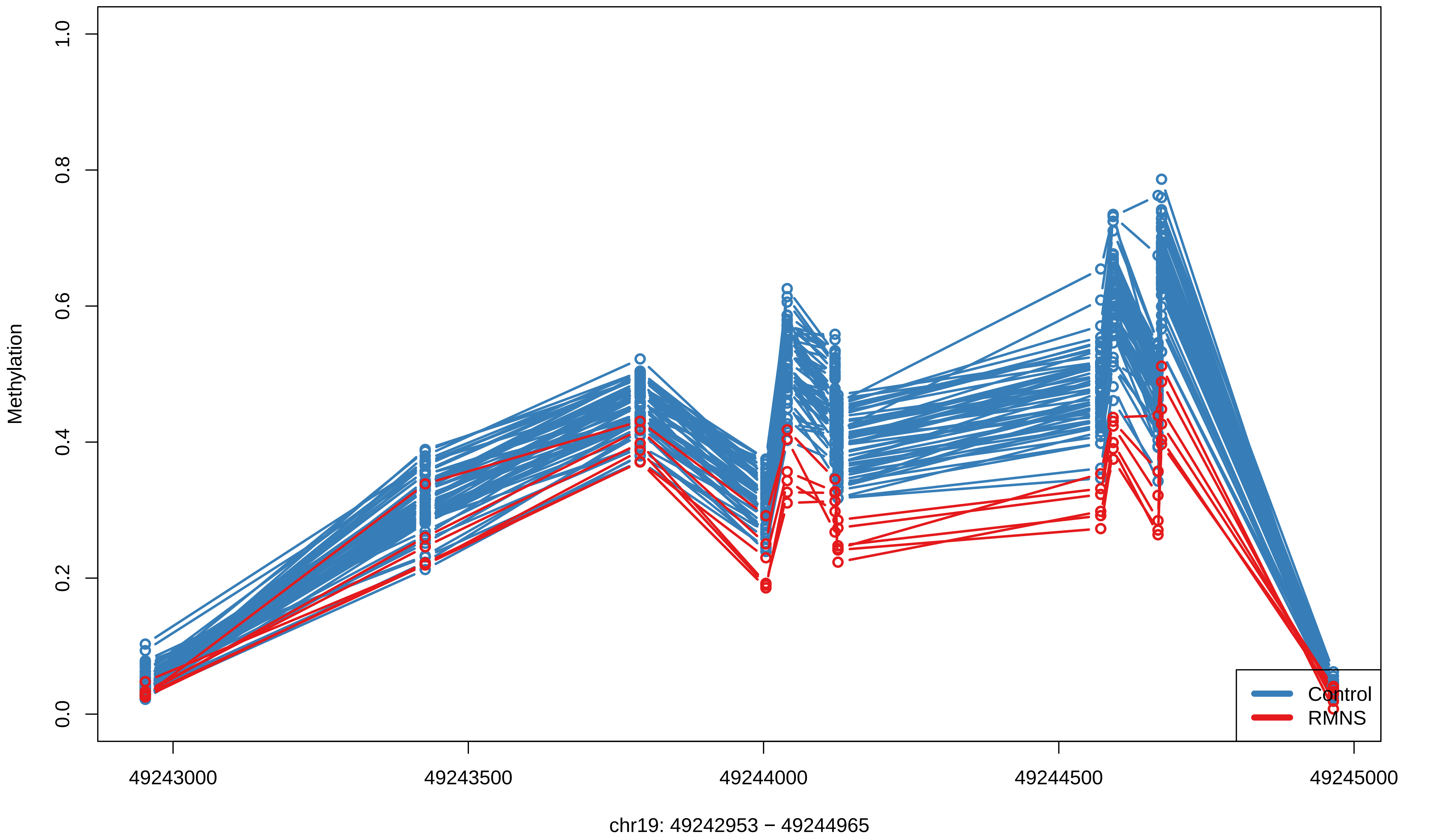

Methylation
1.0
0.8
0.6
0.4
0.2
0.0
49243000
49243500
49244000
49244500
49245000
chr19: 49242953 − 49244965
Control
RMNS

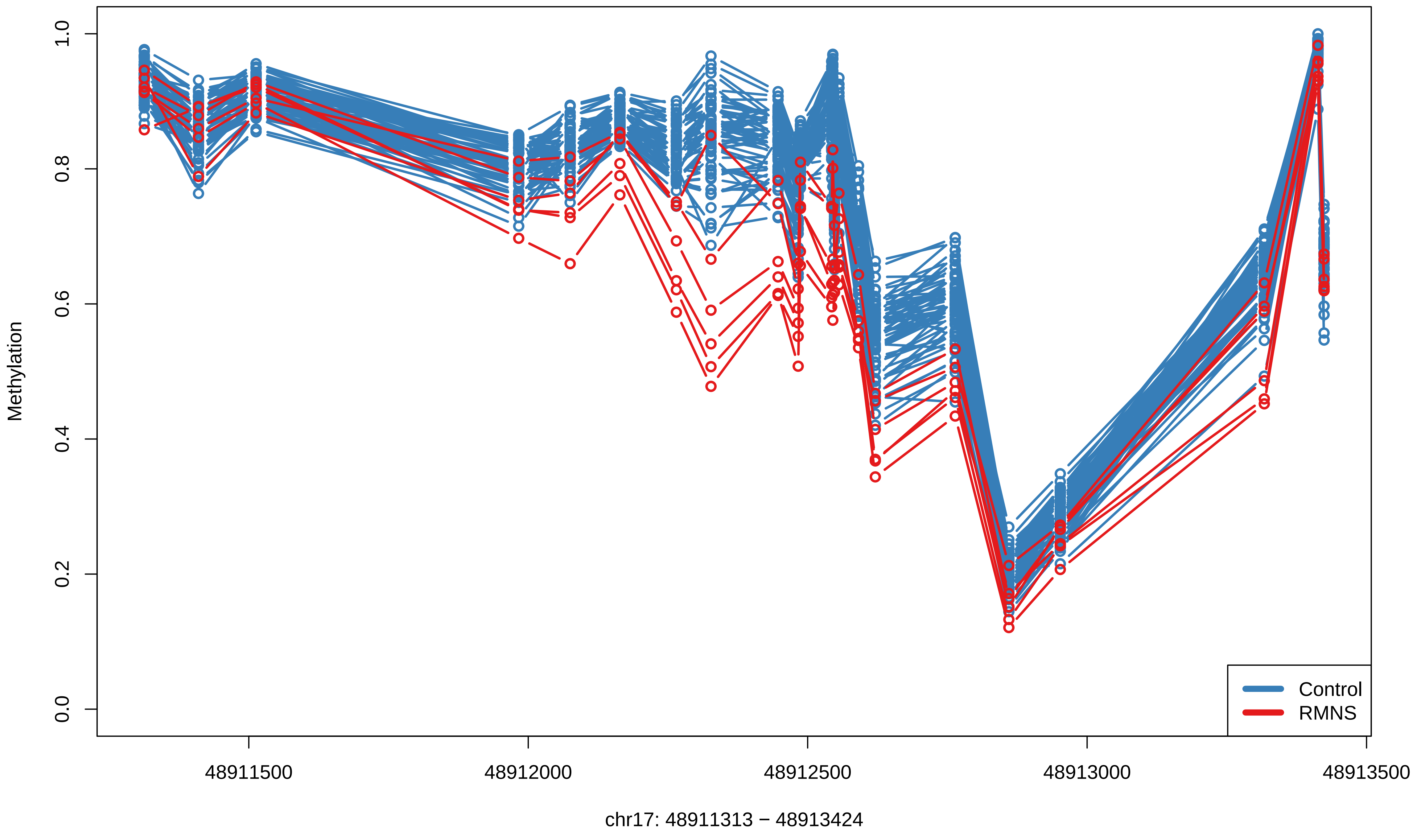

Methylation
1.0
0.8
0.6
0.4
0.2
0.0
48911500
48912000
48912500
48913000
48913500
chr17: 48911313 − 48913424
Control
RMNS

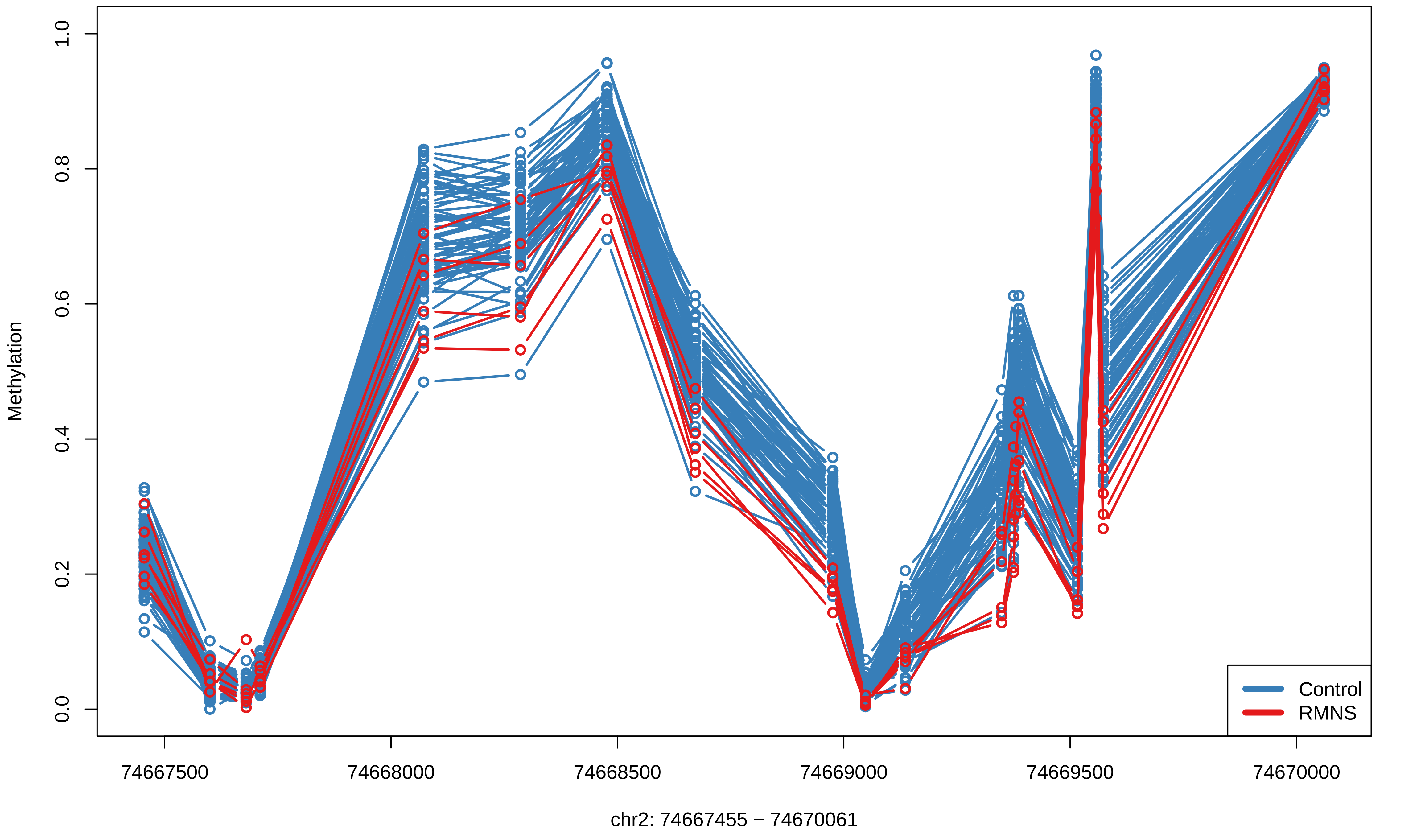

Methylation
1.0
0.8
0.6
0.4
0.2
0.0
74667500
74668000
74668500
74669000
74669500
74670000
chr2: 74667455 − 74670061
Control
RMNS

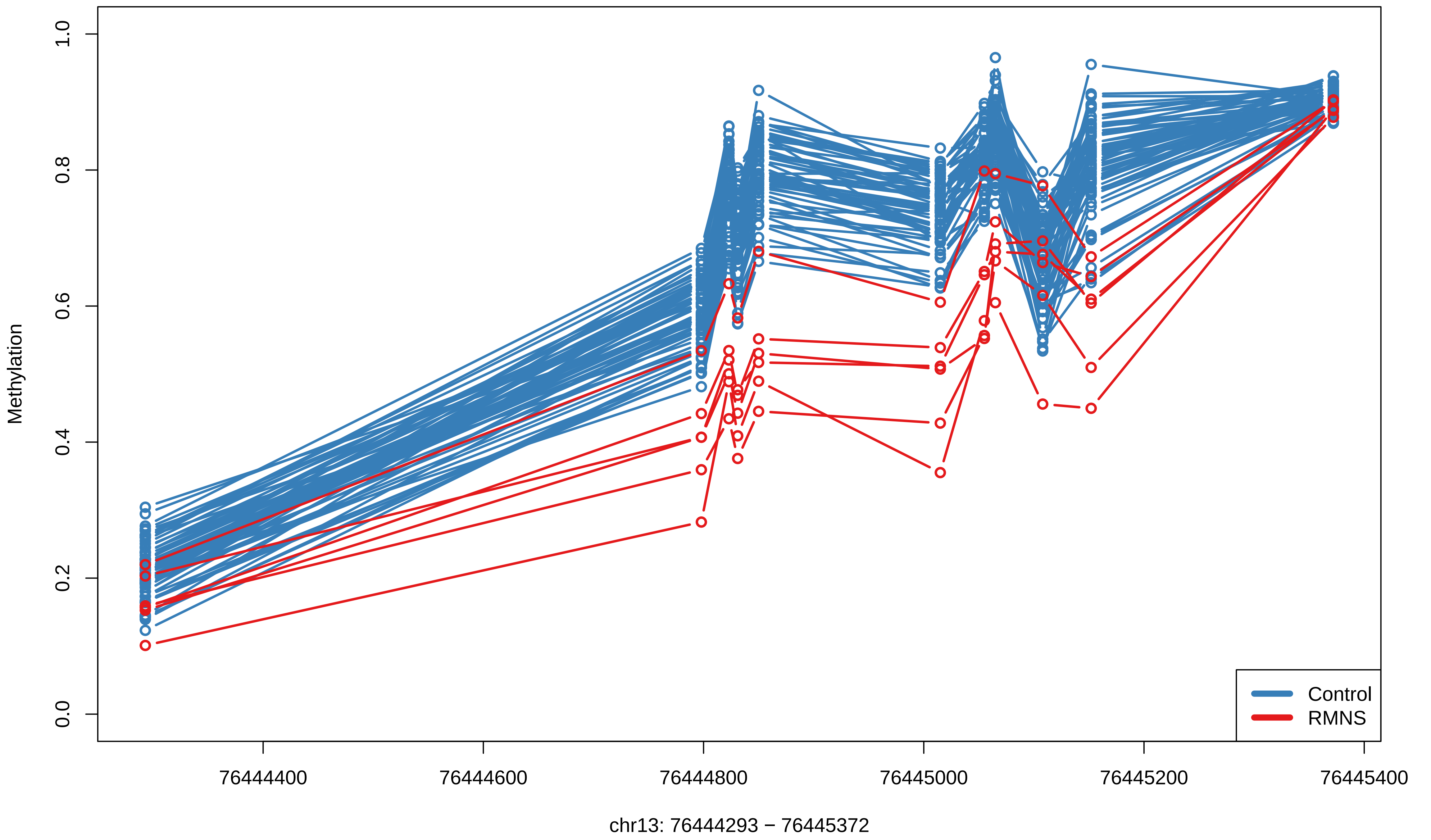

Methylation
1.0
0.8
0.6
0.4
0.2
0.0
76444400
76444600
76444800
76445000
76445200
76445400
chr13: 76444293 − 76445372
Control
RMNS

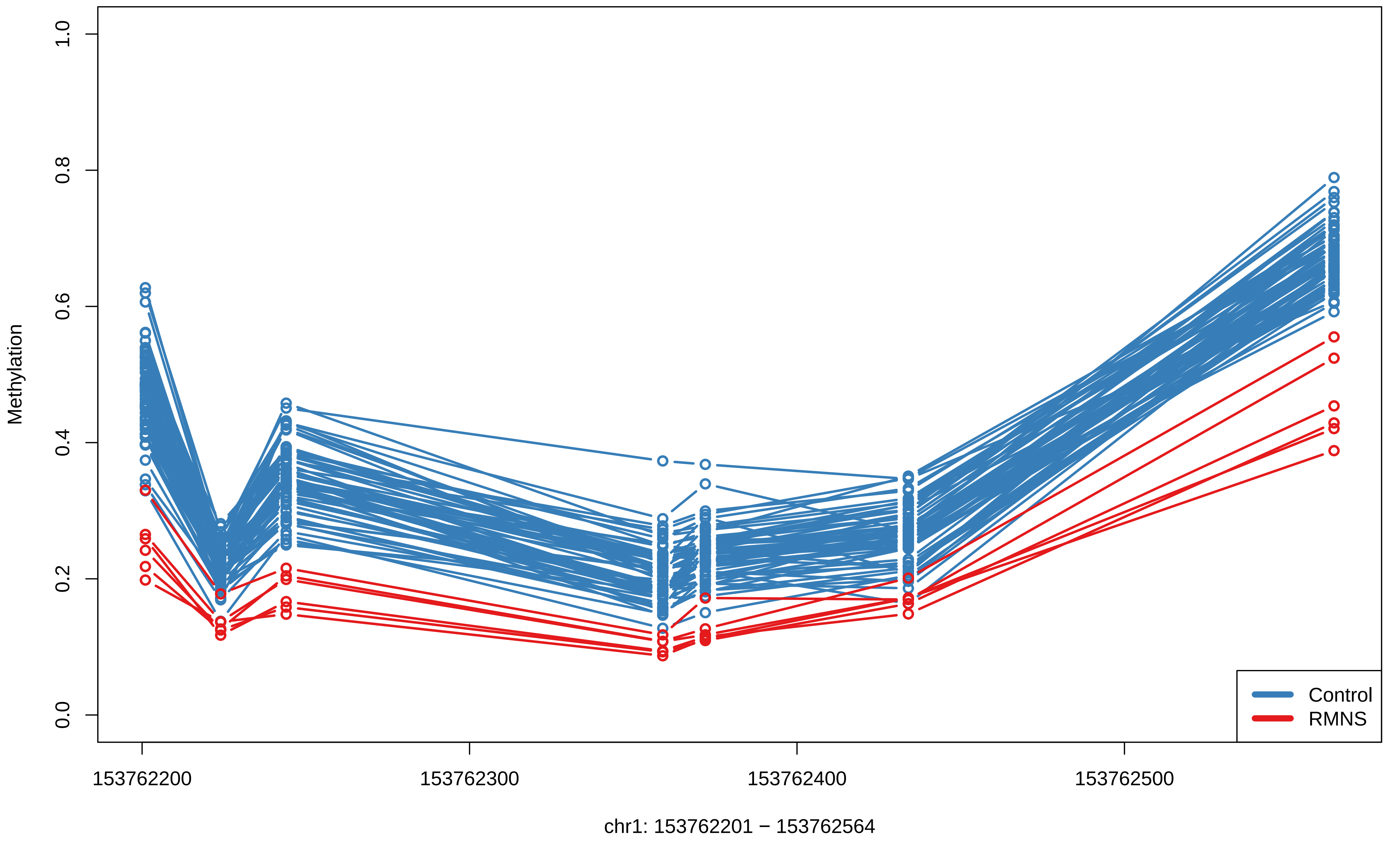

Methylation
0.0
0.2
0.4
0.6
0.8
1.0
153762200
153762300
153762400
153762500
chr1: 153762201 − 153762564
Control
RMNS

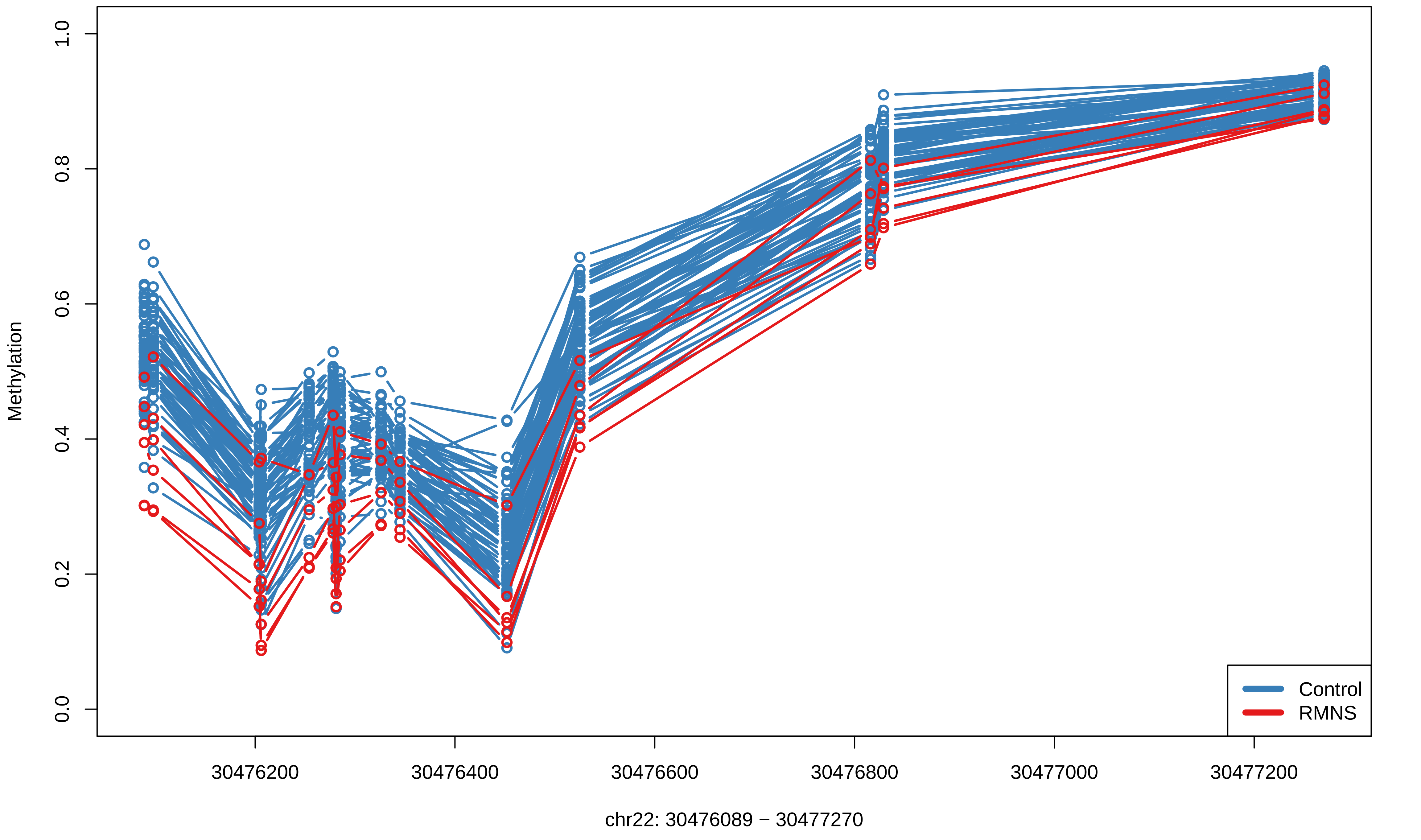
Methylation
0.0
0.2
0.4
0.6
0.8
1.0
30476200
30476400
30476600
30476800
30477000
30477200
chr22: 30476089 − 30477270
Control
RMNS

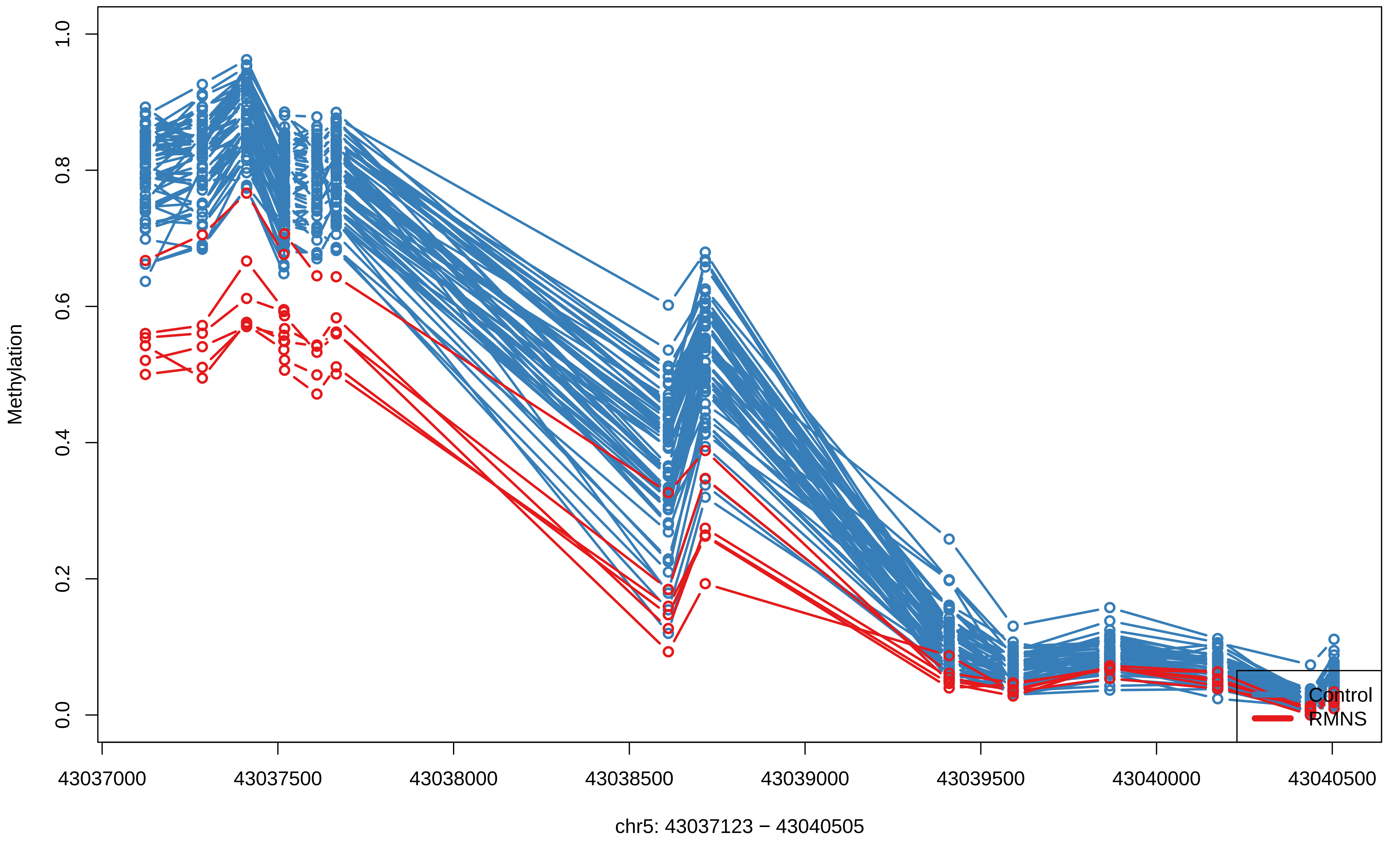

Methylation
0.0
0.2
0.4
0.6
0.8
1.0
43037000
43037500
43038000
43038500
43039000
43039500
43040000
43040500
chr5: 43037123 − 43040505
Control
RMNS

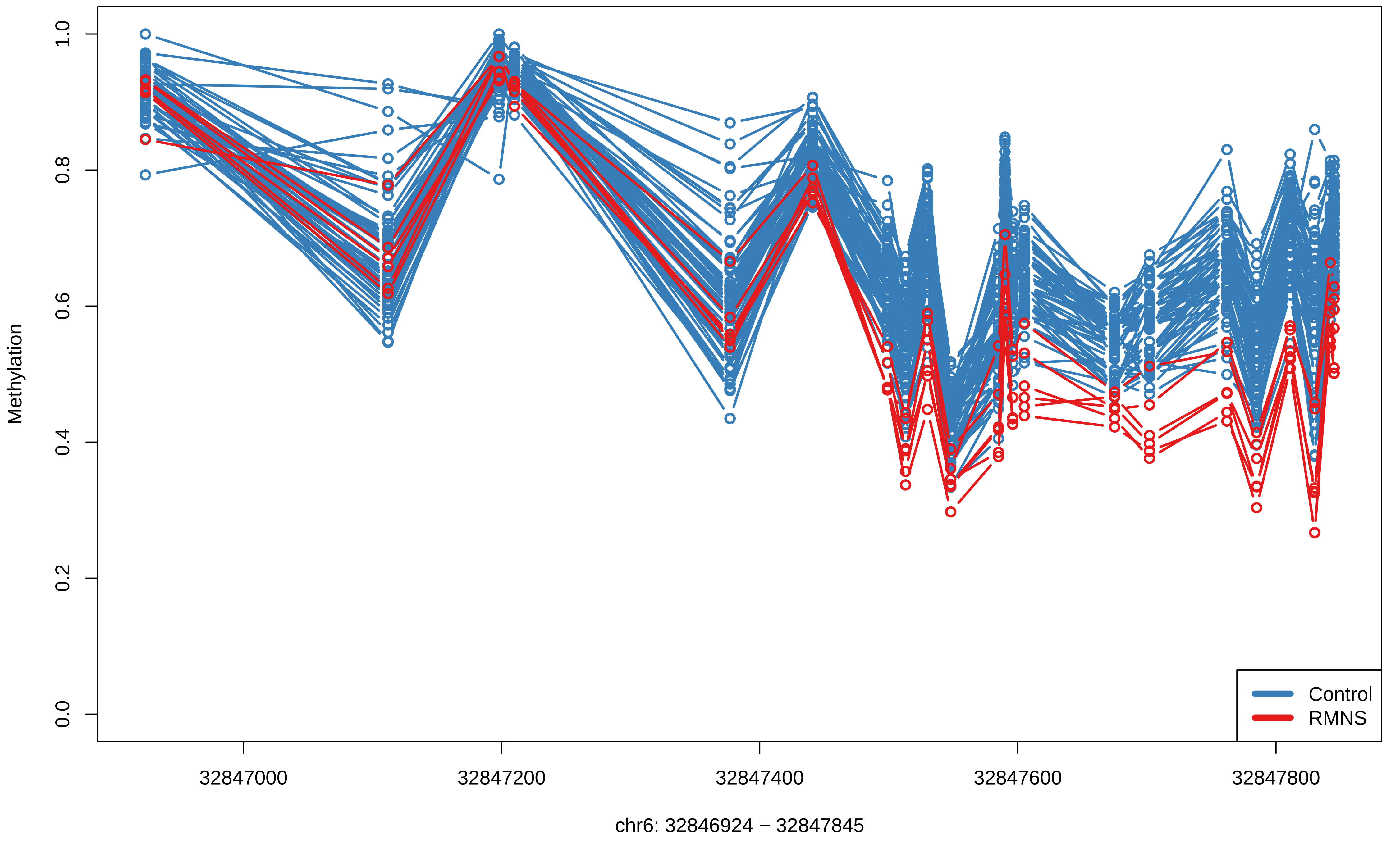

1.0
0.8
0.6
0.4
0.2
0.0
Methylation
32847000
32847200
32847400
32847600
32847800
chr6: 32846924 − 32847845
Control
RMNS

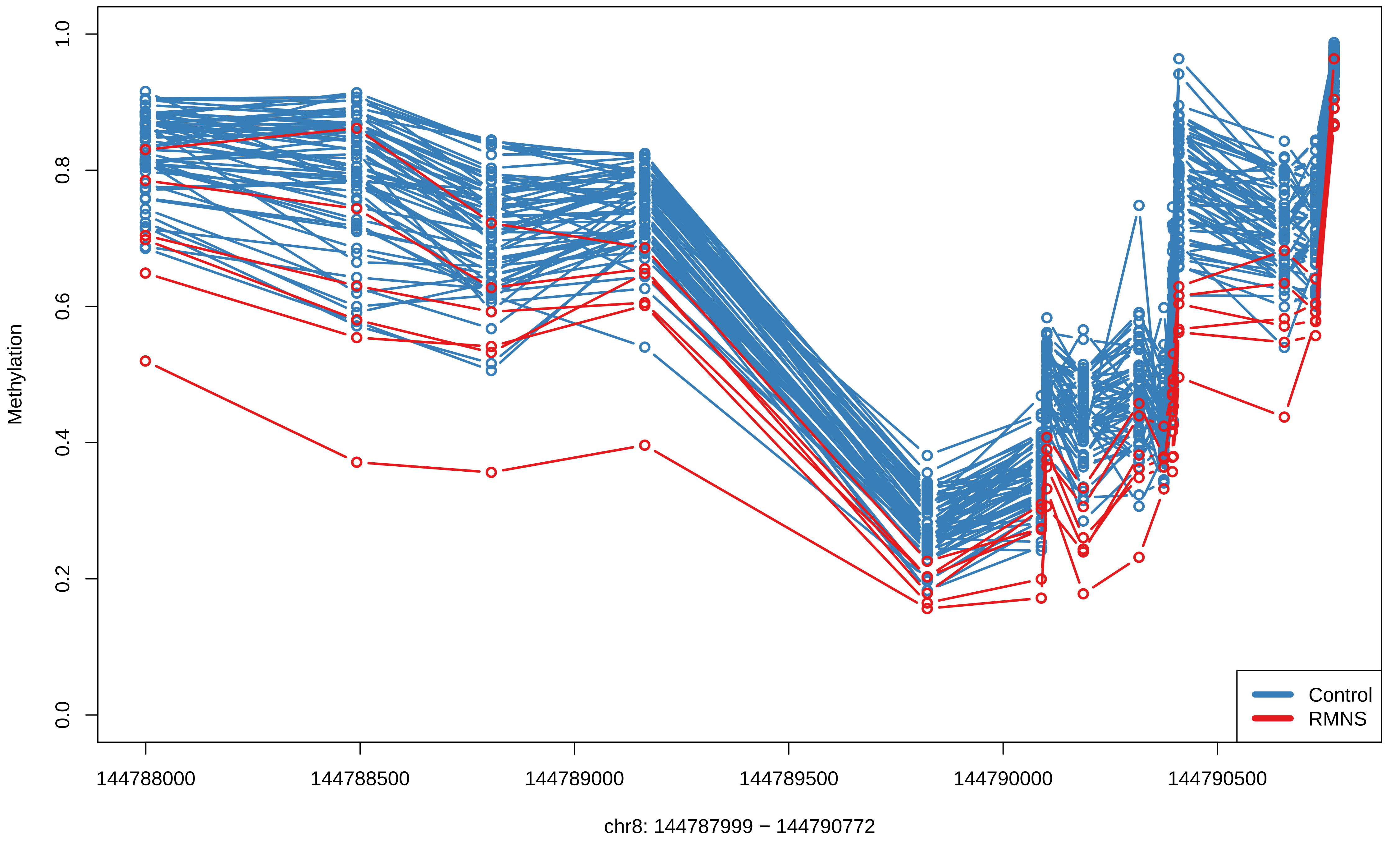
Methylation
0.0
0.2
0.4
0.6
0.8
1.0
144788000
144788500
144789000
144789500
144790000
144790500
chr8: 144787999 − 144790772
Control
RMNS

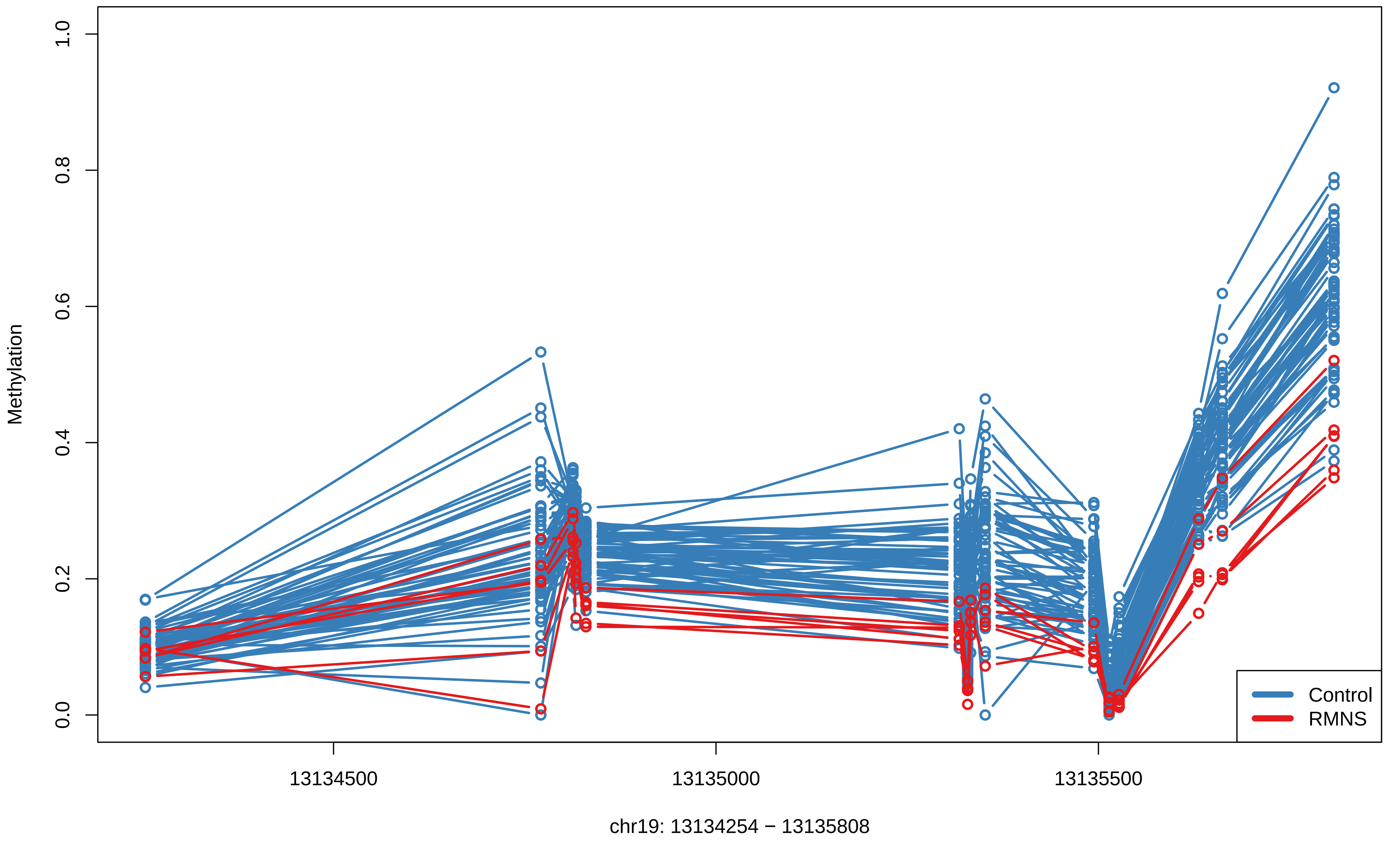

Methylation
0.0
0.2
0.4
0.6
0.8
1.0
13134500
13135000
13135500
chr19: 13134254 − 13135808
Control
RMNS

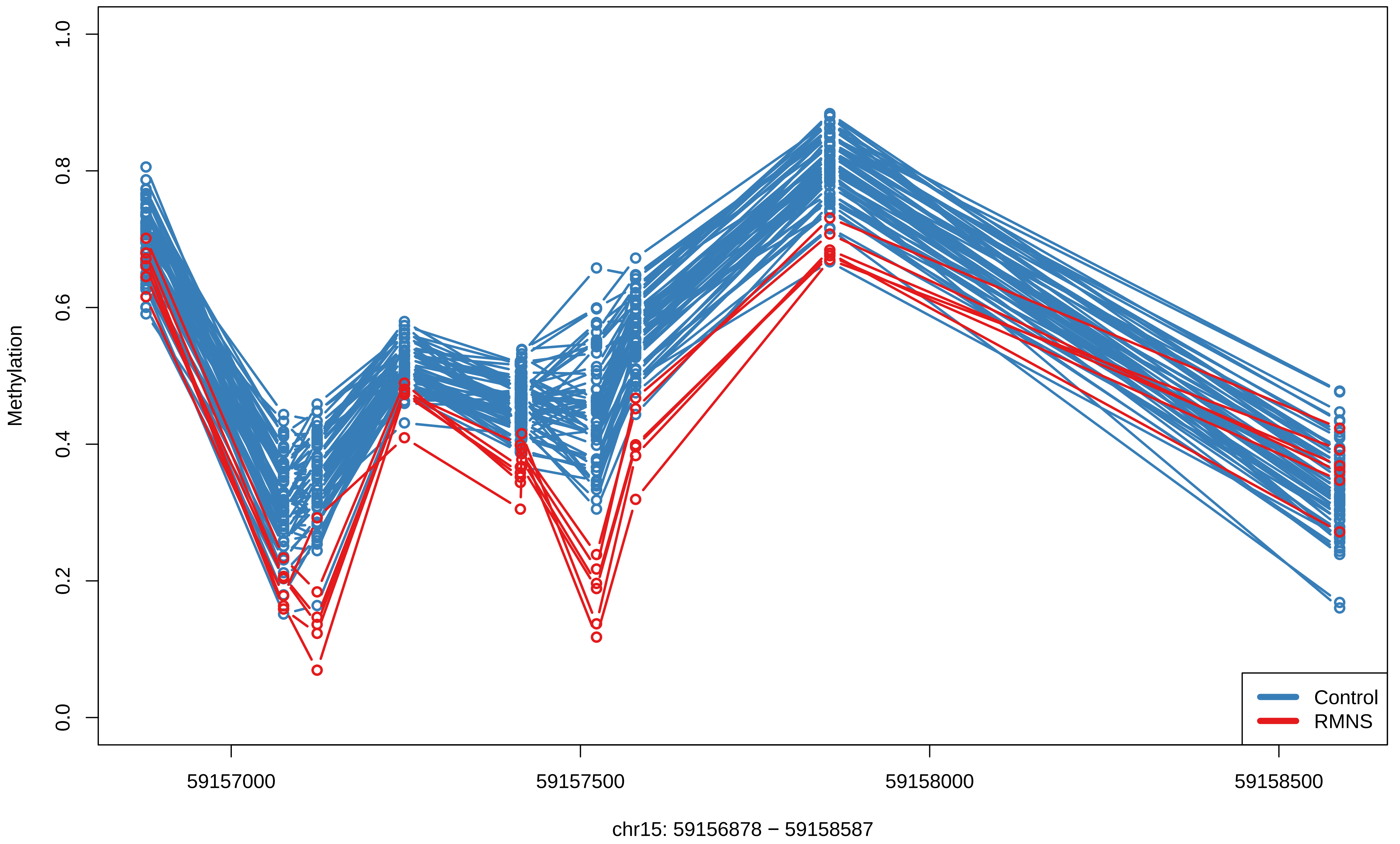

Methylation
0.0
0.2
0.4
0.6
0.8
1.0
59157000
59157500
59158000
59158500
chr15: 59156878 − 59158587
Control
RMNS

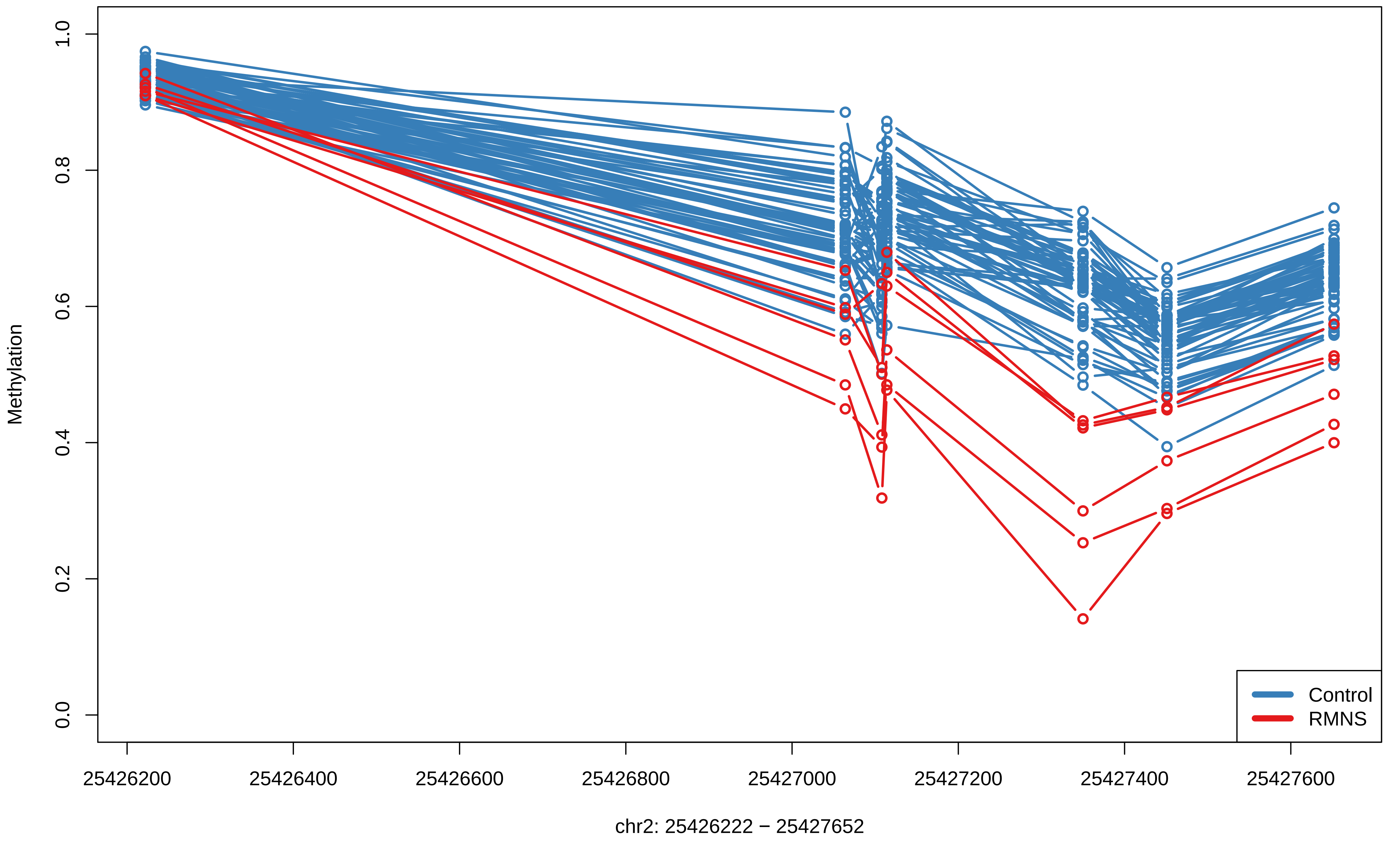

Methylation
1.0
0.8
0.6
0.4
0.2
0.0
25426200
25426400
25426600
25426800
25427000
25427200
25427400
25427600
chr2: 25426222 − 25427652
Control
RMNS

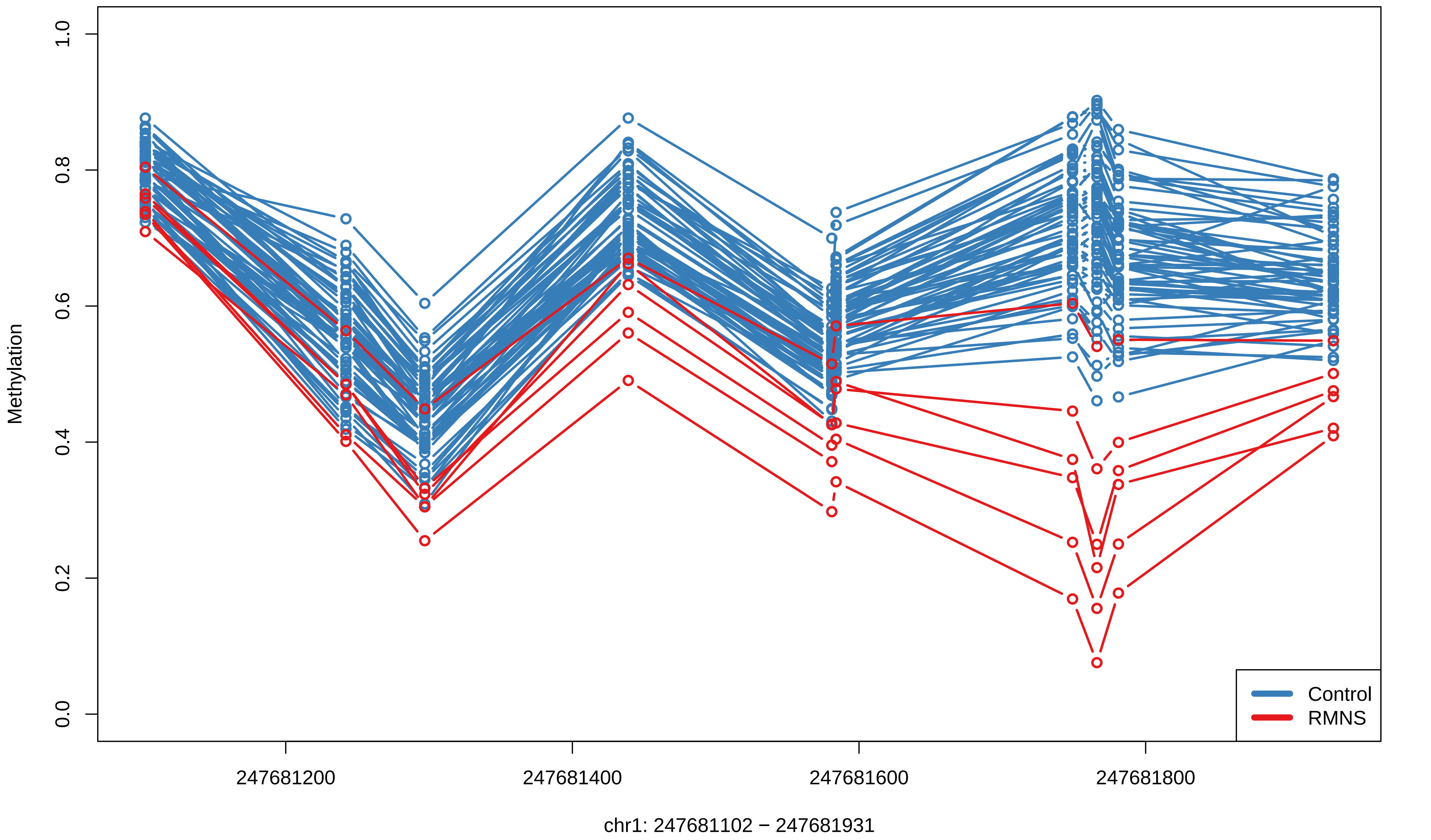

Methylation
0.0
0.2
0.4
0.6
0.8
1.0
247681200
247681400
247681600
247681800
chr1: 247681102 − 247681931
Control
RMNS

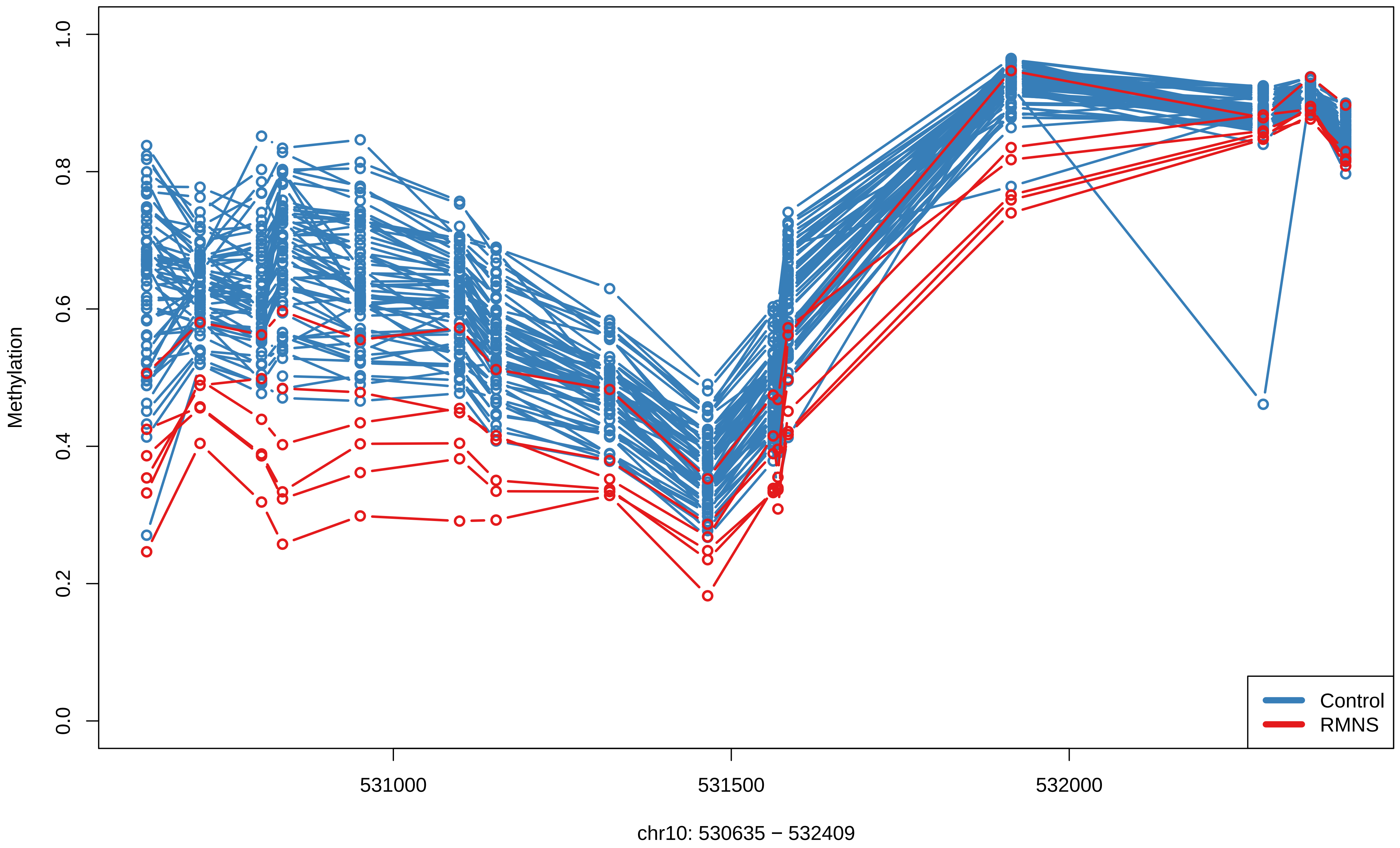

Methylation
1.0
0.8
0.6
0.4
0.2
0.0
531000
531500
532000
chr10: 530635 − 532409
Control
RMNS

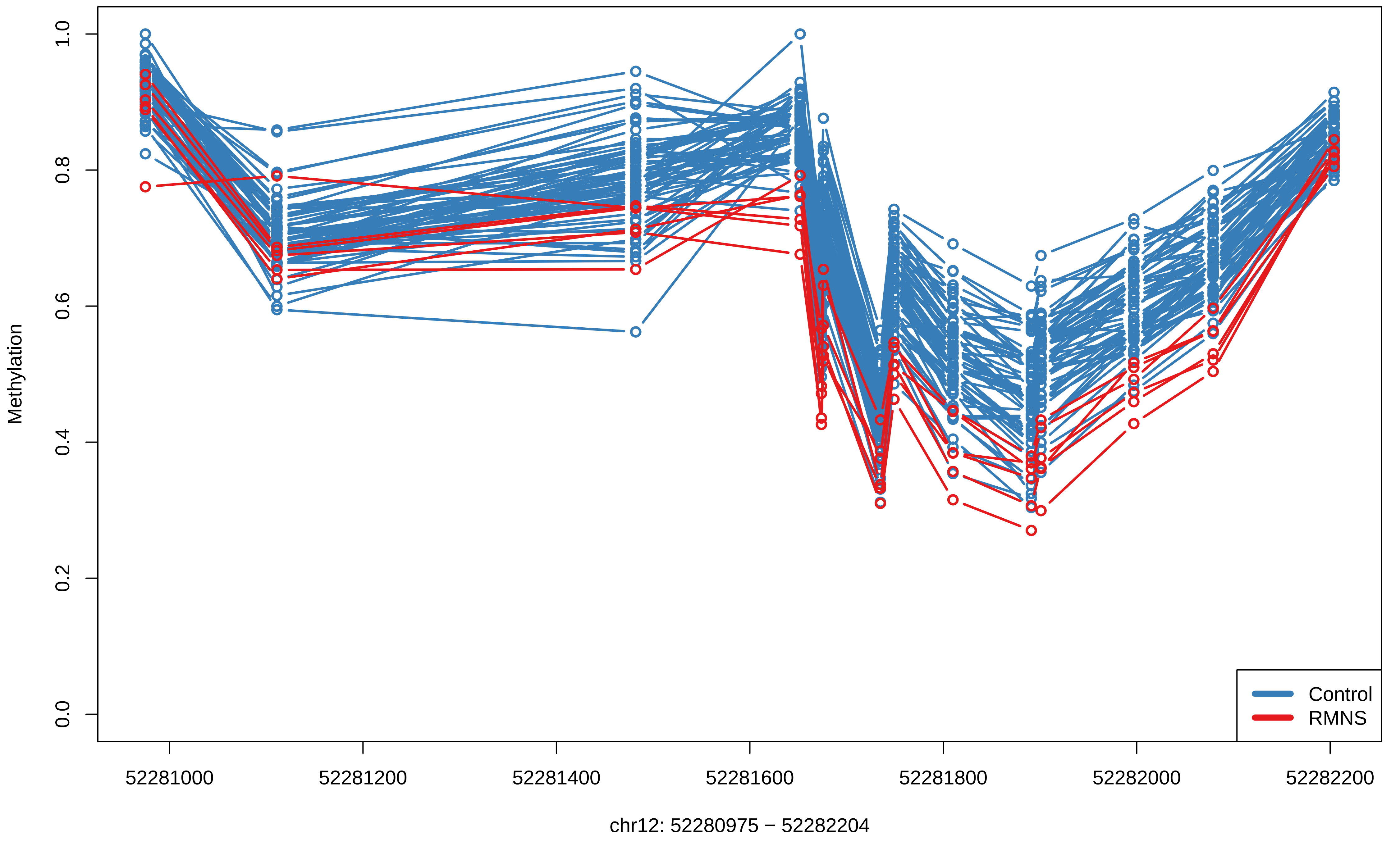

Methylation
1.0
0.8
0.6
0.4
0.2
0.0
52281000
52281200
52281400
52281600
52281800
52282000
52282200
chr12: 52280975 − 52282204
Control
RMNS

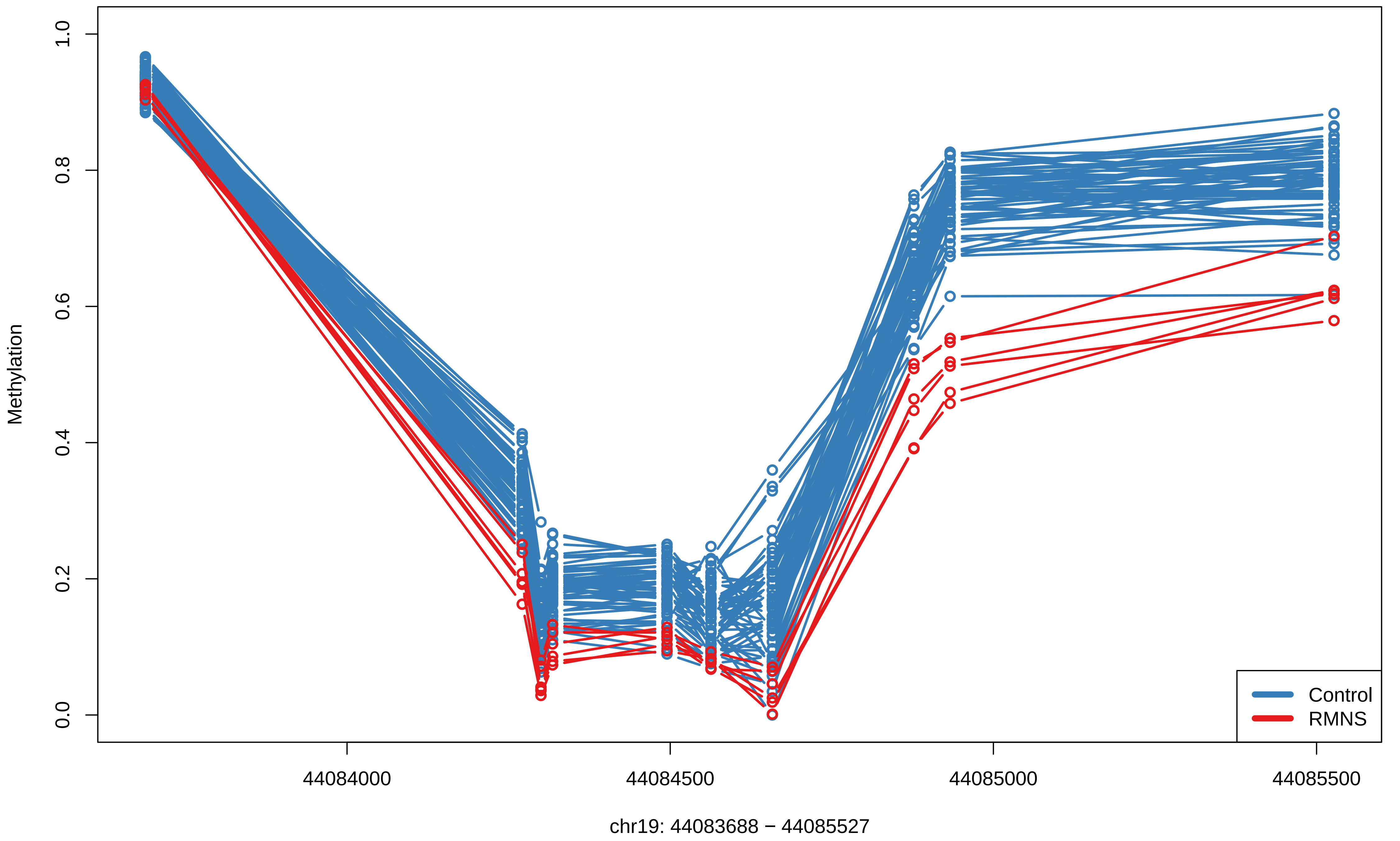

Methylation
1.0
0.8
0.6
0.4
0.2
0.0
44084000
44084500
44085000
44085500
chr19: 44083688 − 44085527
Control
RMNS

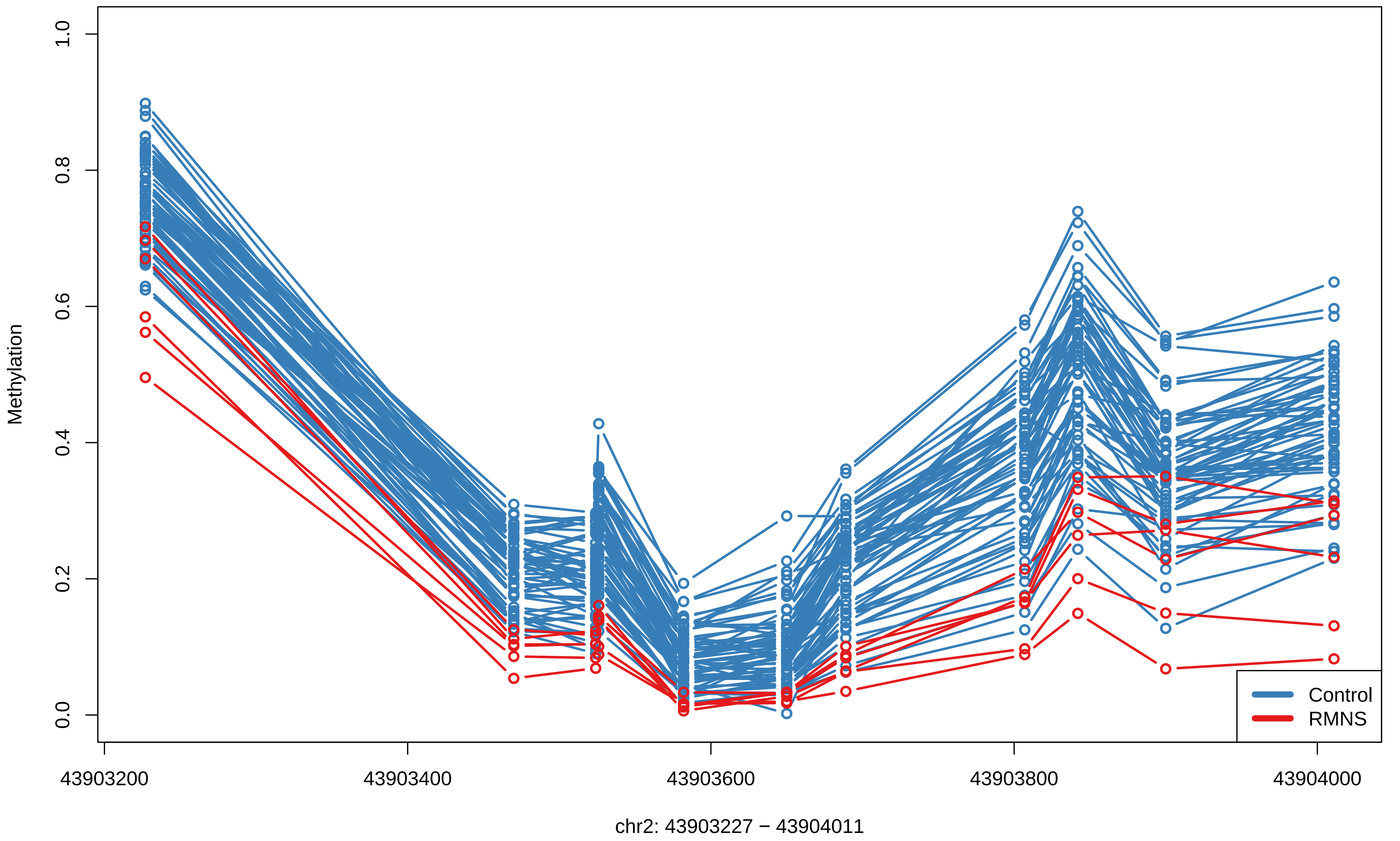

1.0
0.8
0.6
0.4
0.2
0.0
Methylation
43903200
43903400
43903600
43903800
43904000
chr2: 43903227 − 43904011
Control
RMNS

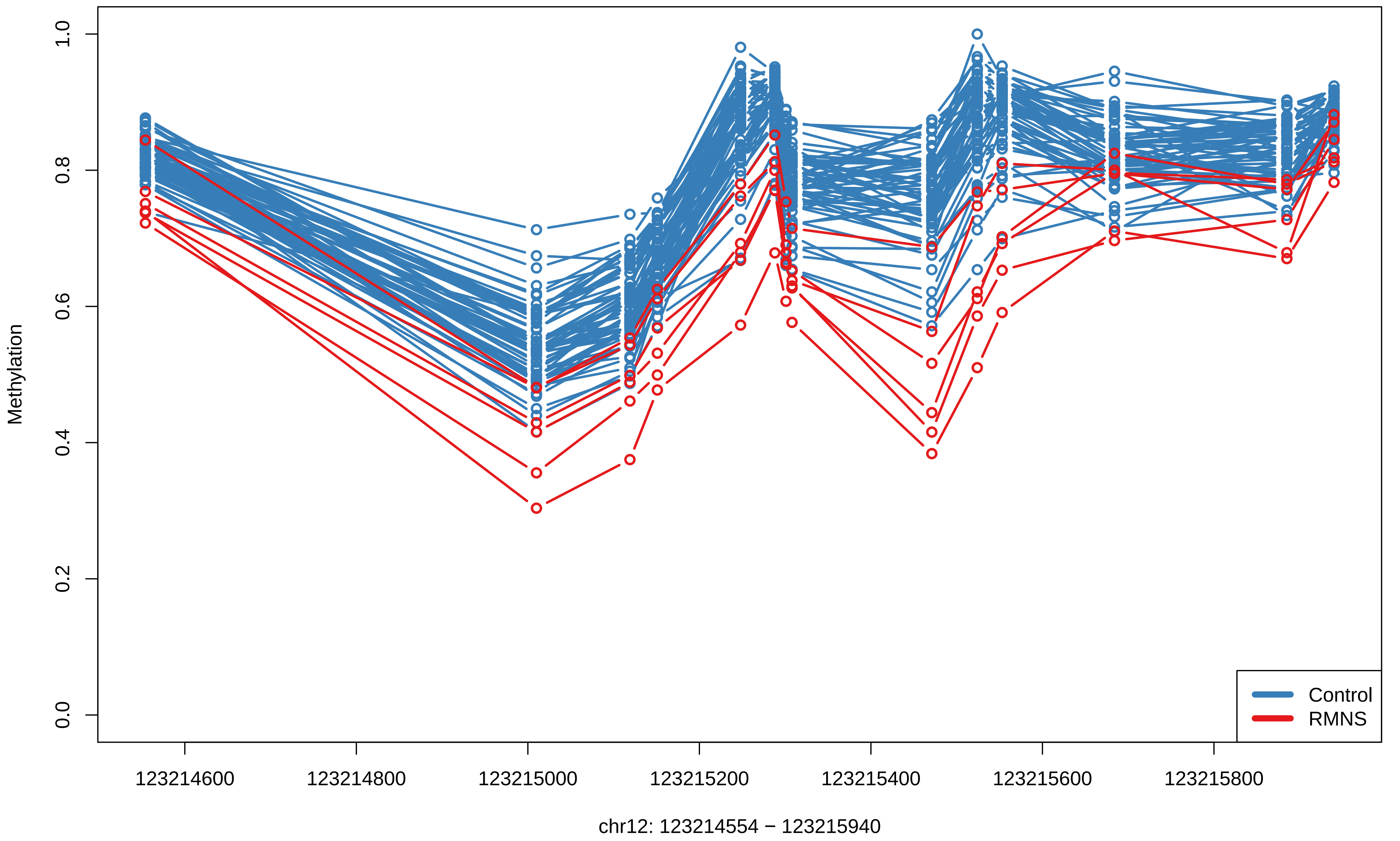

Methylation
0.0
0.2
0.4
0.6
0.8
1.0
123214600
123214800
123215000
123215200
123215400
123215600
123215800
chr12: 123214554 − 123215940
Control
RMNS

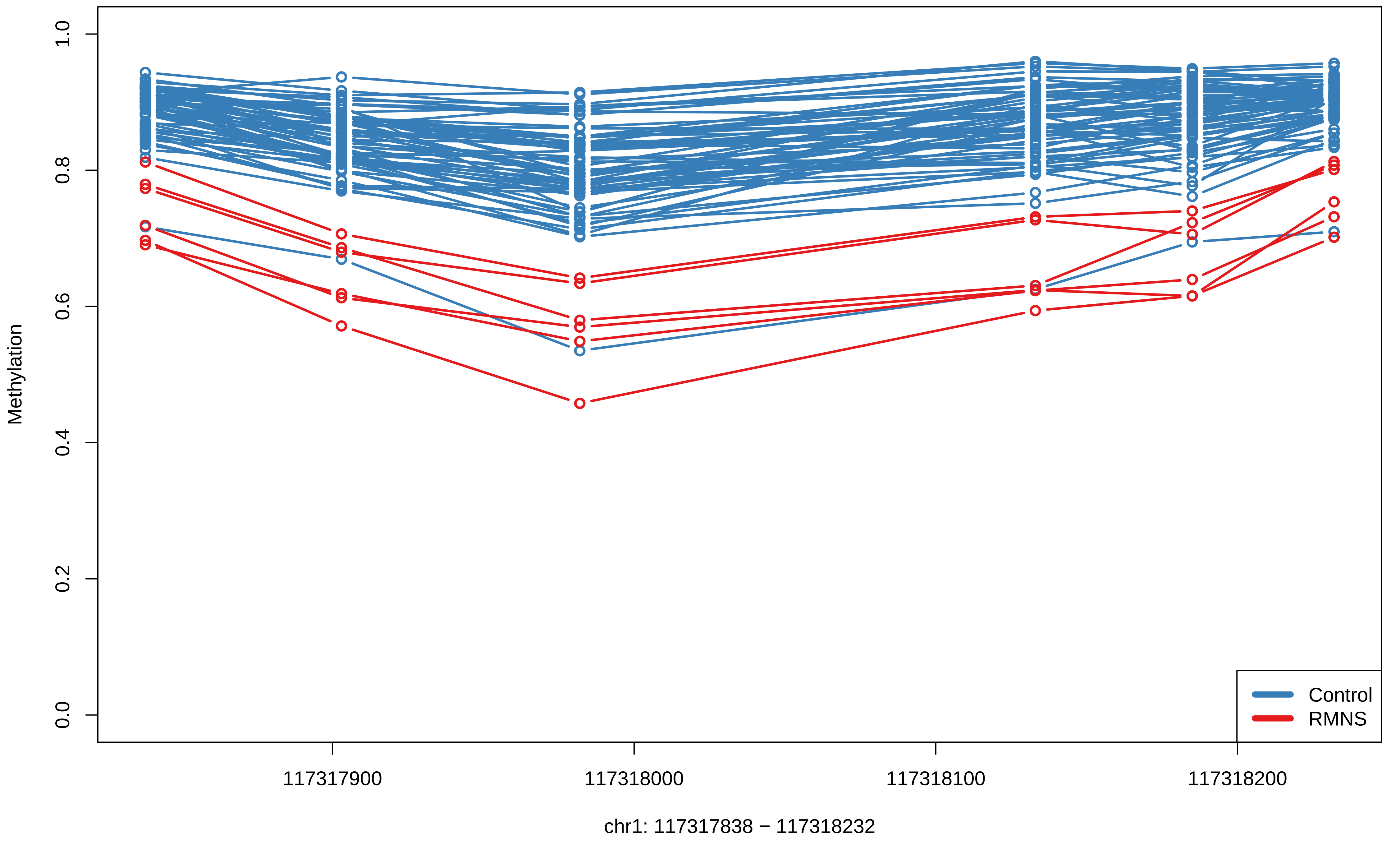

Methylation
0.0
0.2
0.4
0.6
0.8
1.0
117317900
117318000
117318100
117318200
chr1: 117317838 − 117318232
Control
RMNS

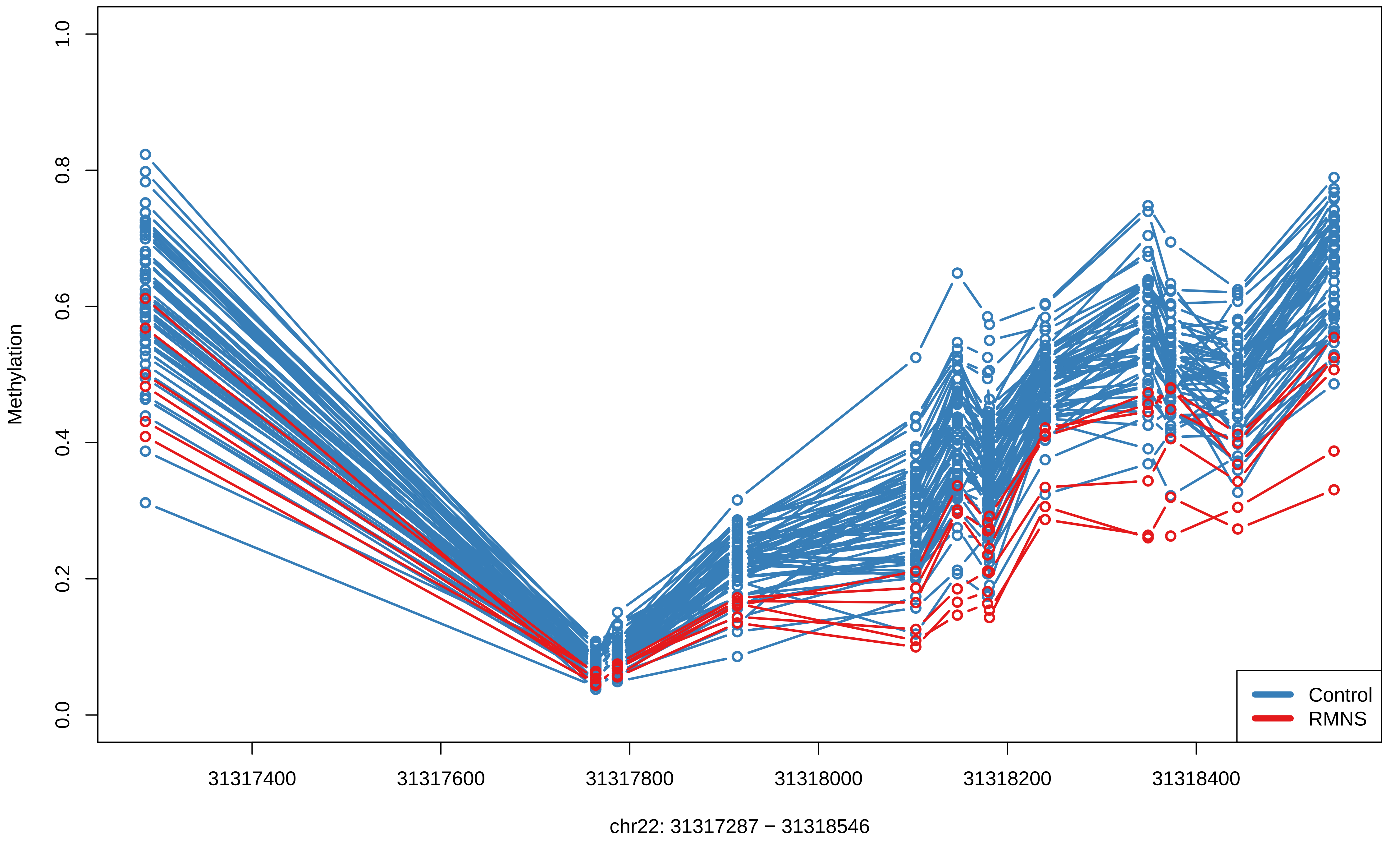

Methylation
1.0
0.8
0.6
0.4
0.2
0.0
31317400
31317600
31317800
31318000
31318200
31318400
chr22: 31317287 − 31318546
Control
RMNS

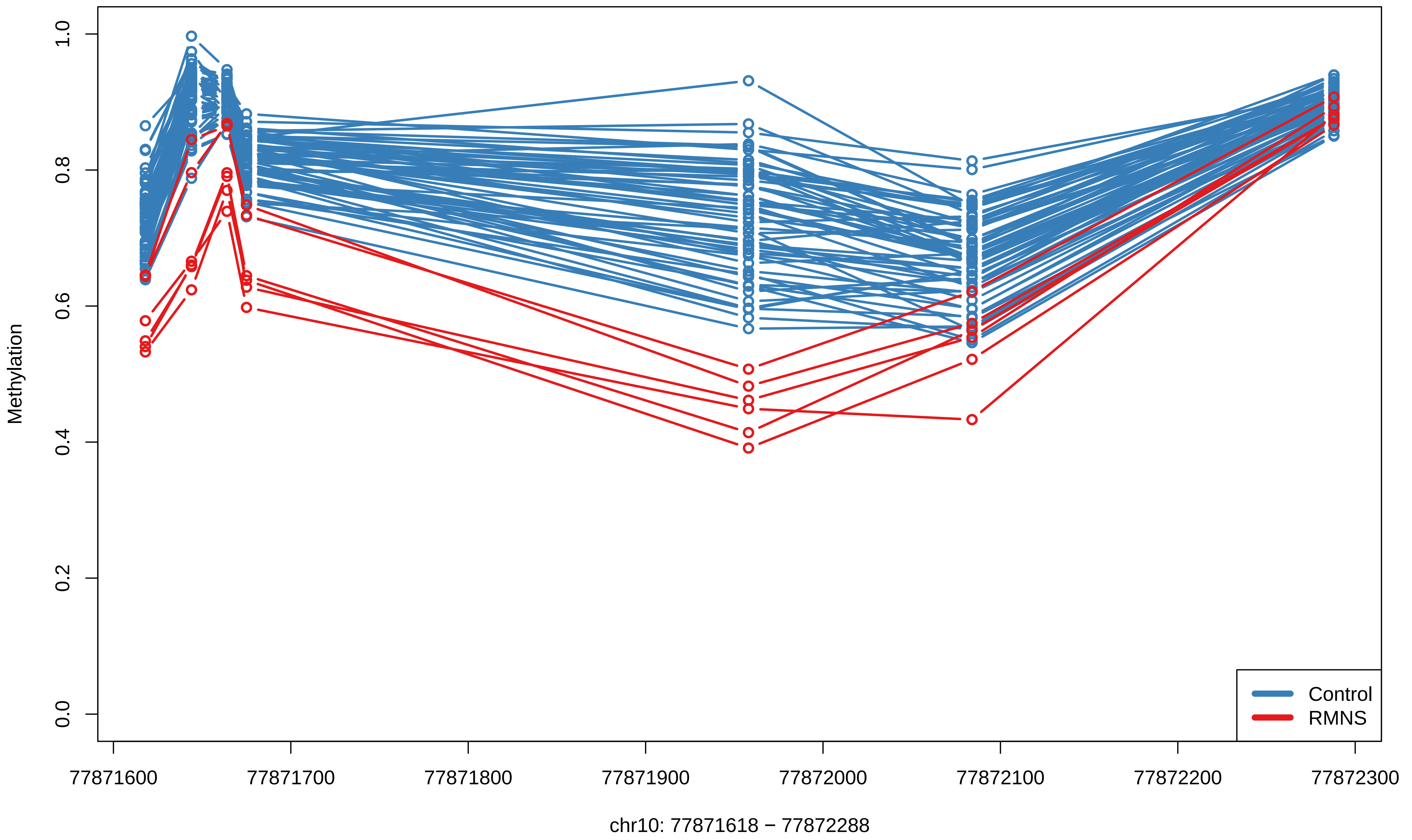

Methylation
1.0
0.8
0.6
0.4
0.2
0.0
77871600
77871700
77871800
77871900
77872000
77872100
77872200
77872300
chr10: 77871618 − 77872288
Control
RMNS

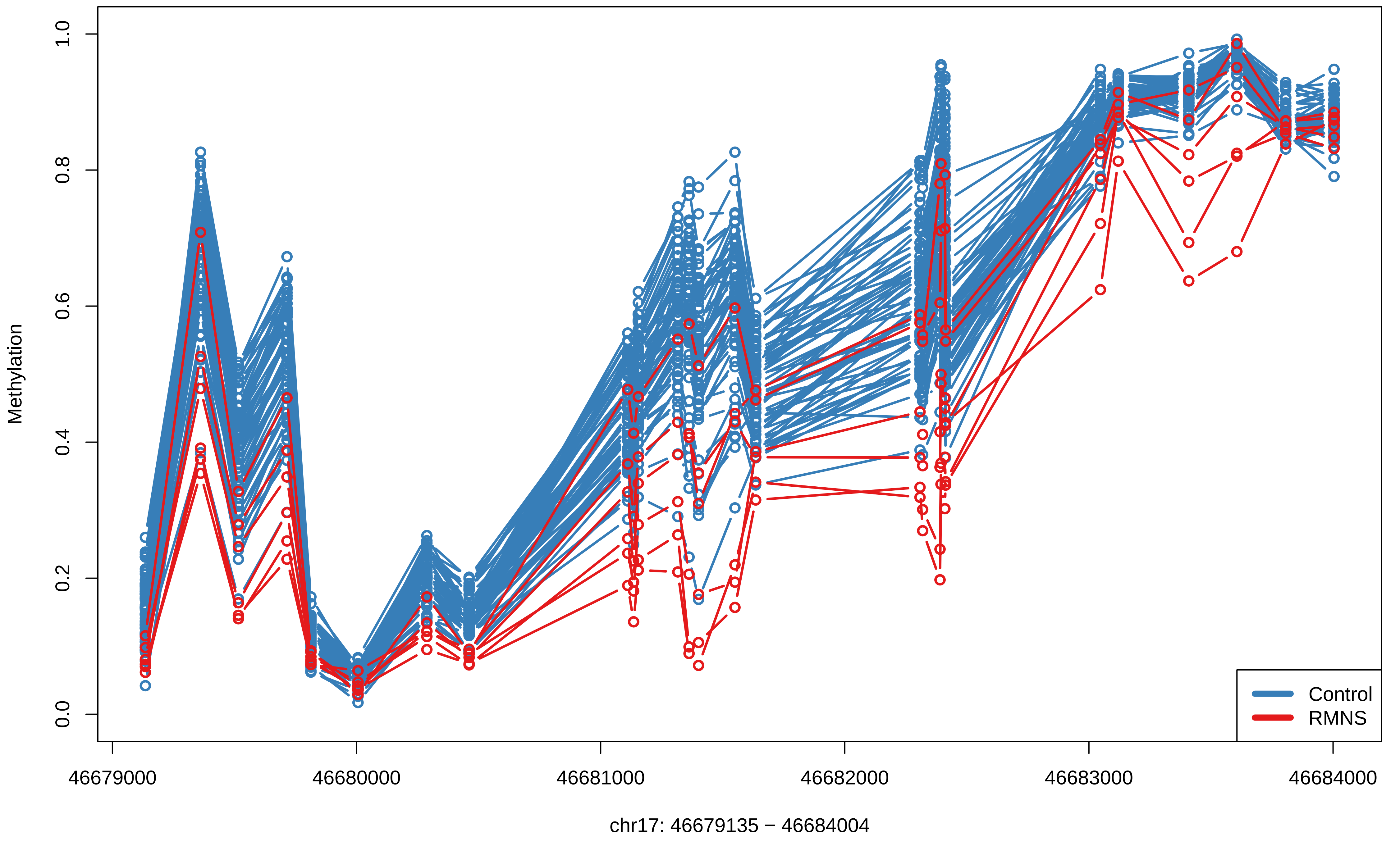

Methylation
0.0
0.2
0.4
0.6
0.8
1.0
46679000
46680000
46681000
46682000
46683000
46684000
chr17: 46679135 − 46684004
Control
RMNS

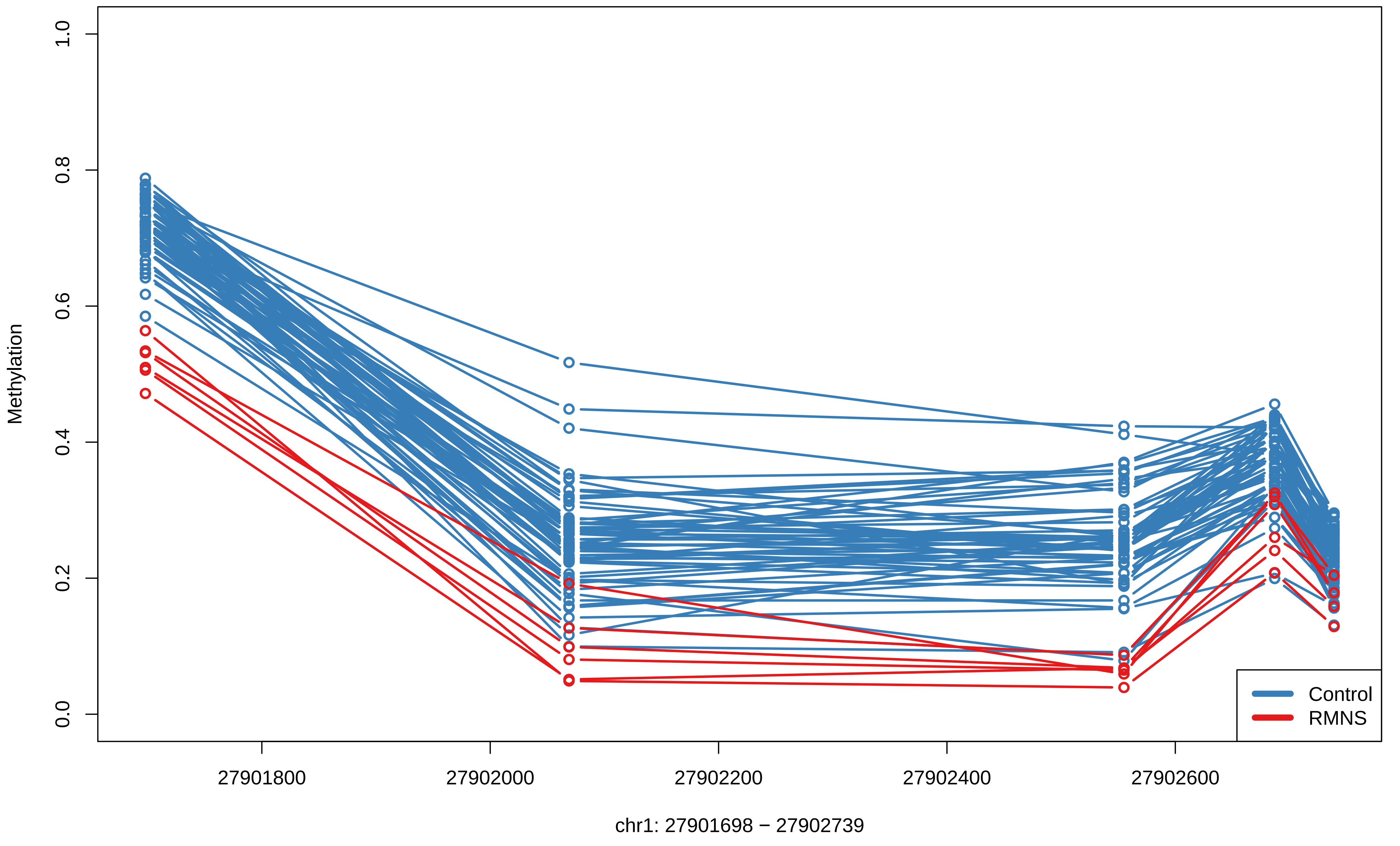

Methylation
0.0
0.2
0.4
0.6
0.8
1.0
27901800
27902000
27902200
27902400
27902600
chr1: 27901698 − 27902739
Control
RMNS

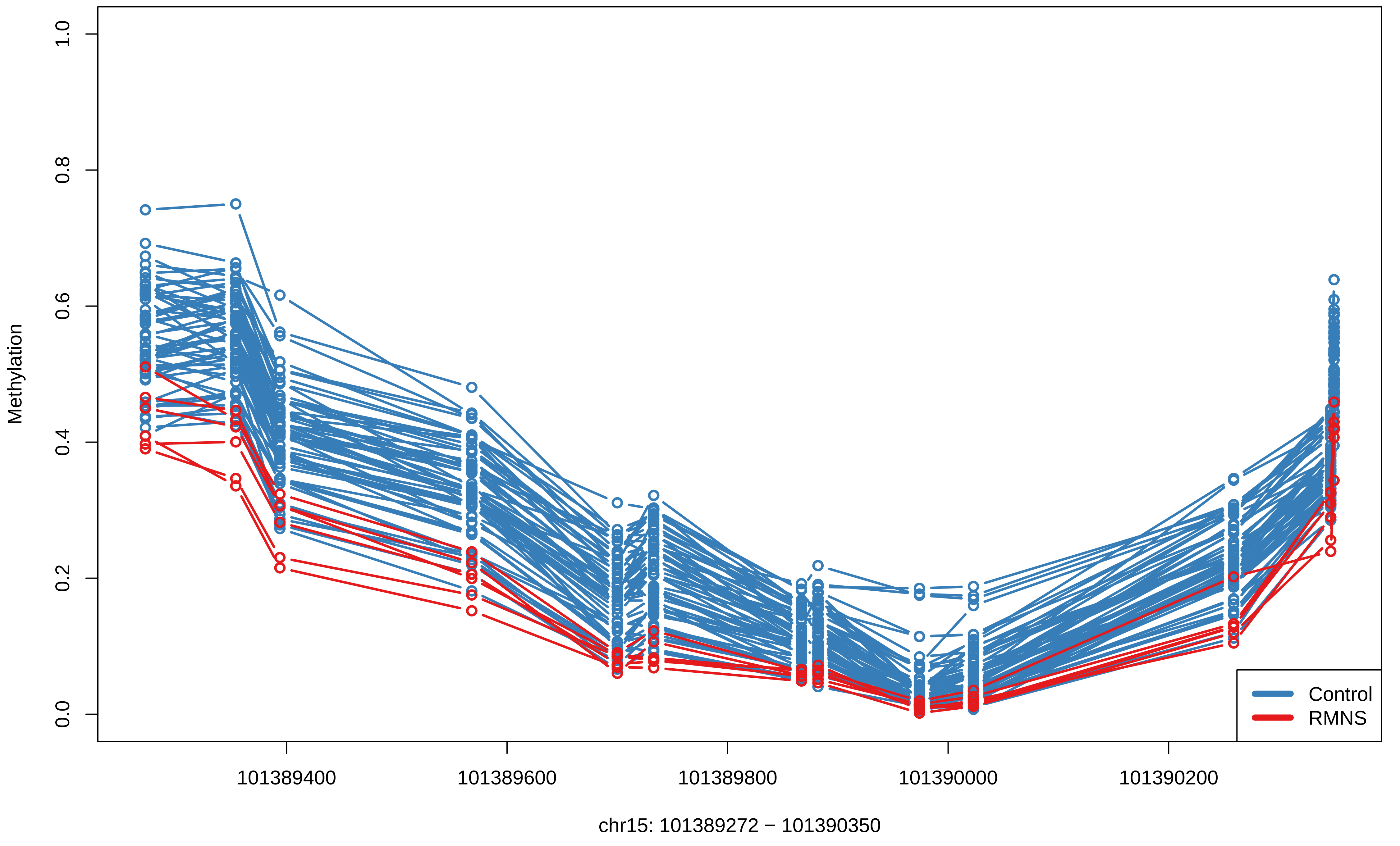

1.0
0.8
0.6
0.4
0.2
0.0
Methylation
101389400
101389600
101389800
101390000
101390200
chr15: 101389272 − 101390350
Control
RMNS

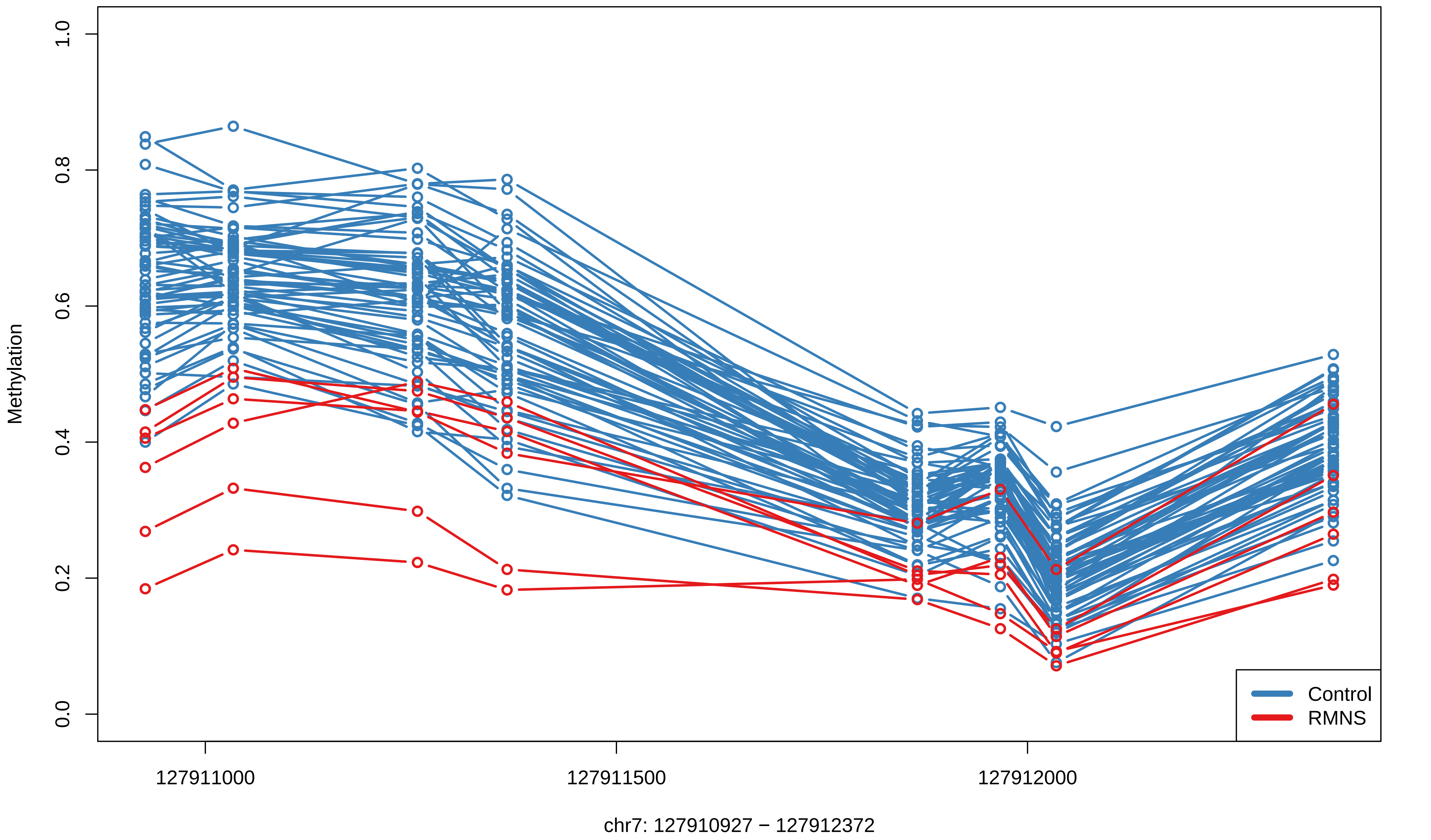

Methylation
0.0
0.2
0.4
0.6
0.8
1.0
127911000
127911500
127912000
chr7: 127910927 − 127912372
Control
RMNS

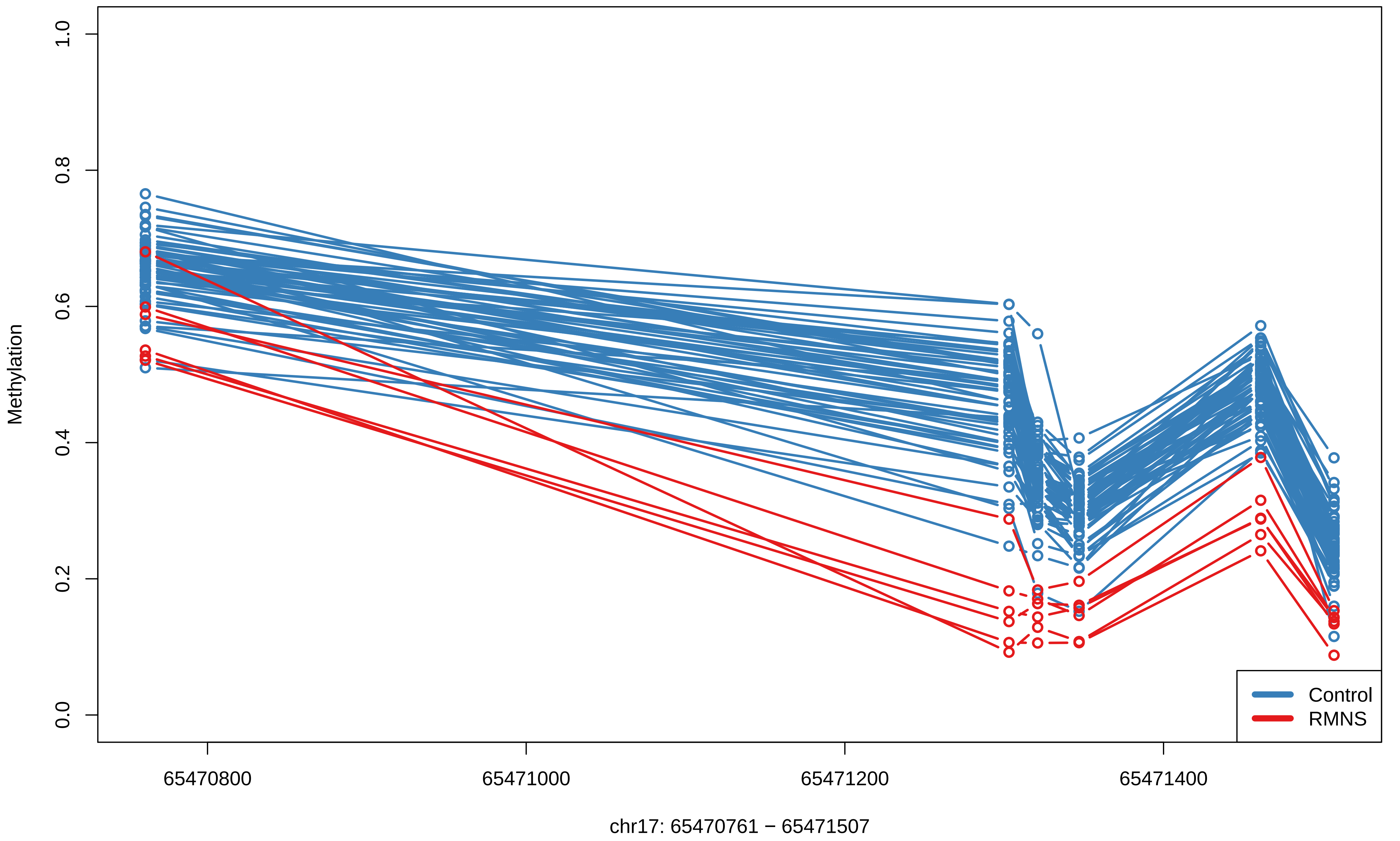
Methylation
0.0
0.2
0.4
0.6
0.8
1.0
65470800
65471000
65471200
65471400
chr17: 65470761 − 65471507
Control
RMNS

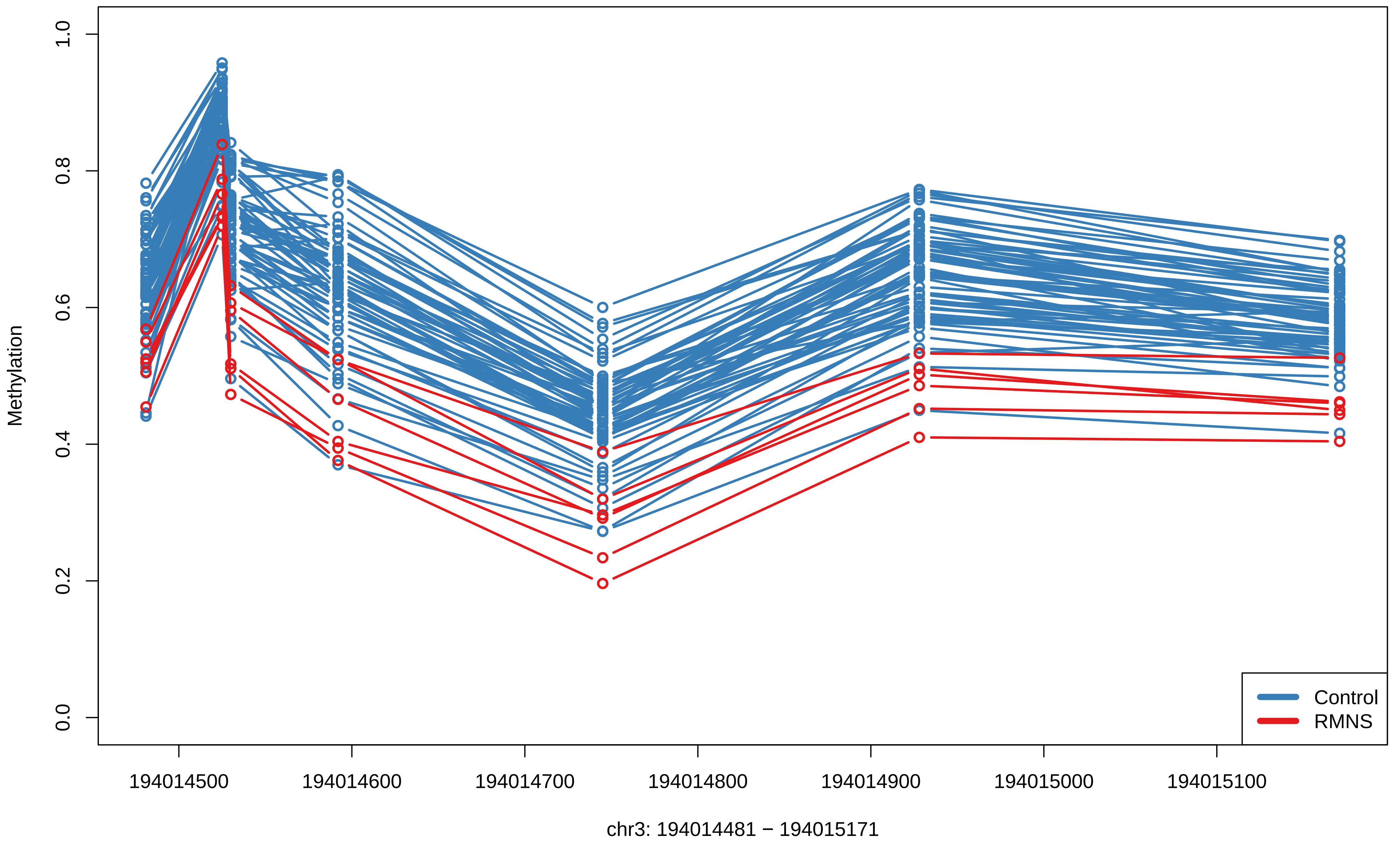

Methylation
1.0
0.8
0.6
0.4
0.2
0.0
194014500
194014600
194014700
194014800
194014900
194015000
194015100
chr3: 194014481 − 194015171
Control
RMNS

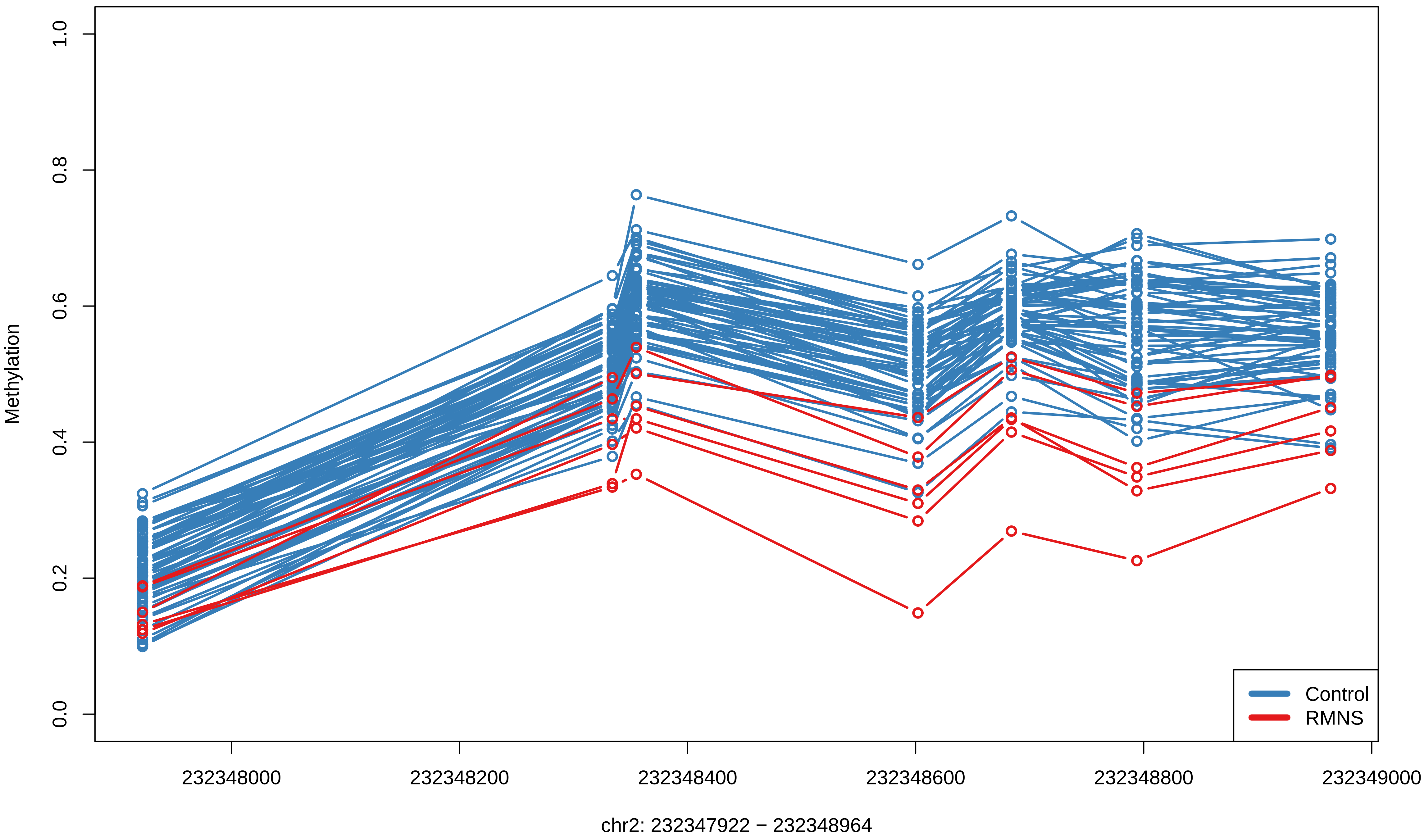
Methylation
0.0
0.2
0.4
0.6
0.8
1.0
232348000
232348200
232348400
232348600
232348800
232349000
chr2: 232347922 − 232348964
Control
RMNS

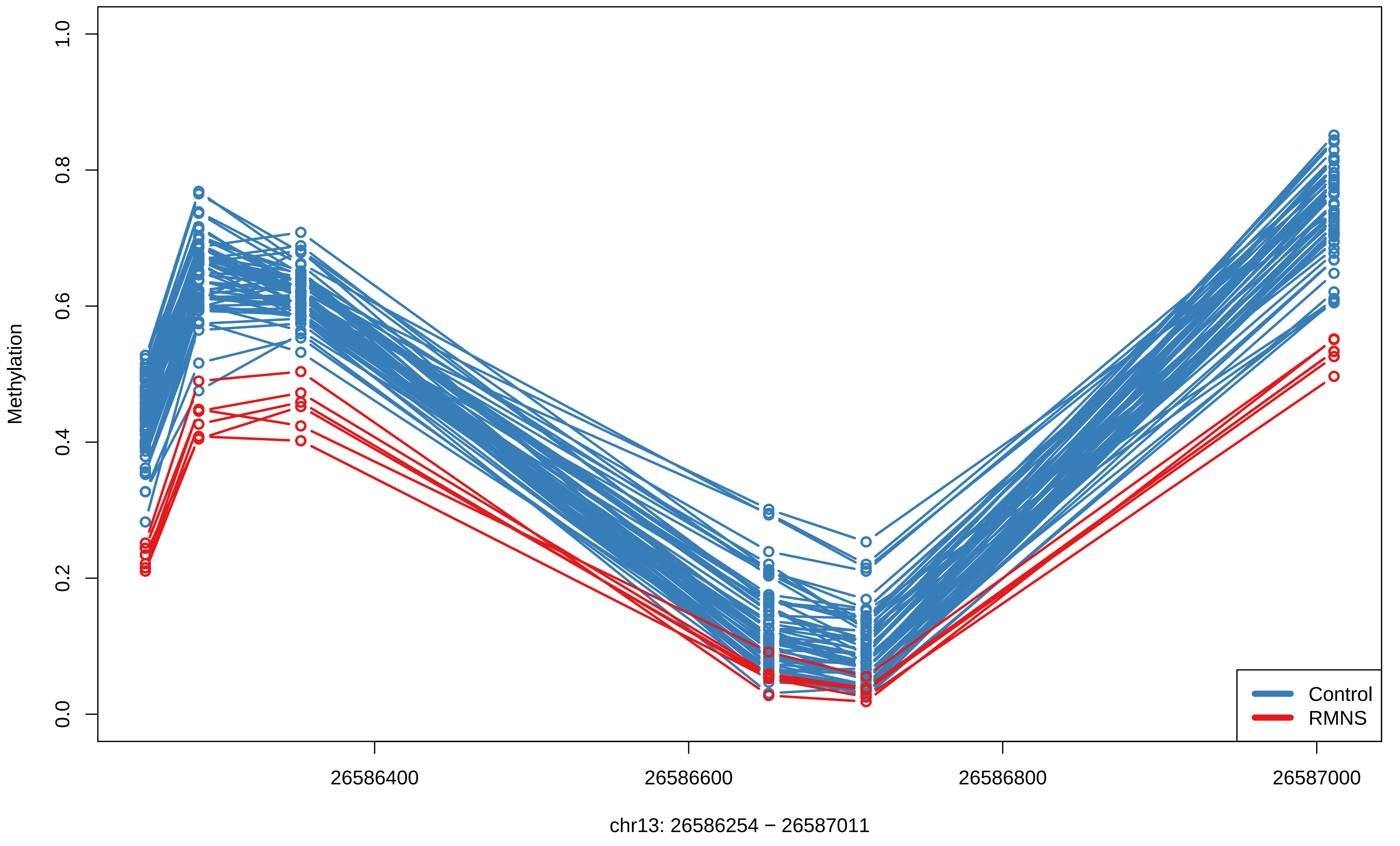

Methylation
0.0
0.2
0.4
0.6
0.8
1.0
26586400
26586600
26586800
26587000
chr13: 26586254 − 26587011
Control
RMNS

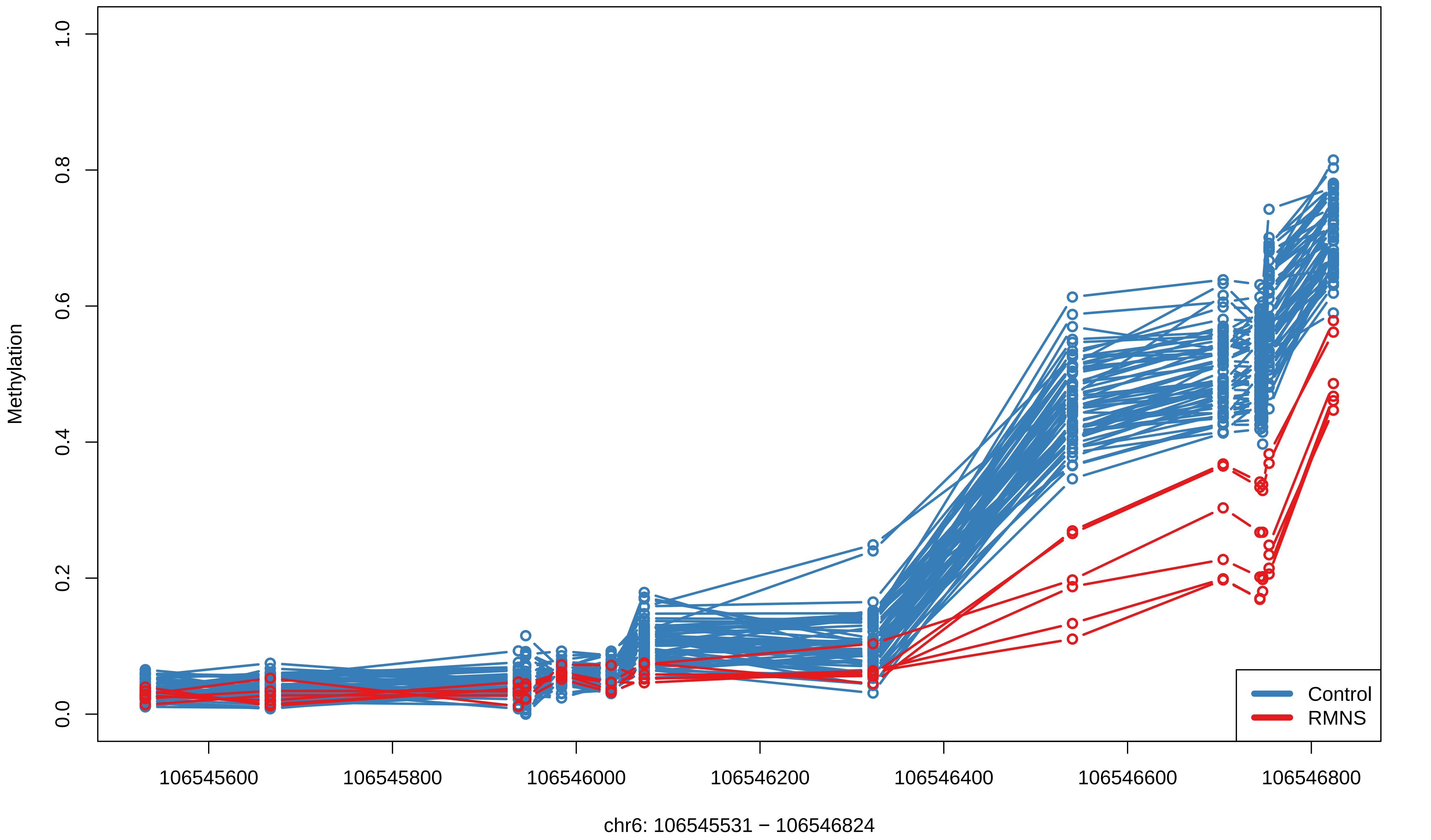

1.0
0.8
0.6
0.4
0.2
0.0
Methylation
106545600
106545800
106546000
106546200
106546400
106546600
106546800
chr6: 106545531 − 106546824
Control
RMNS

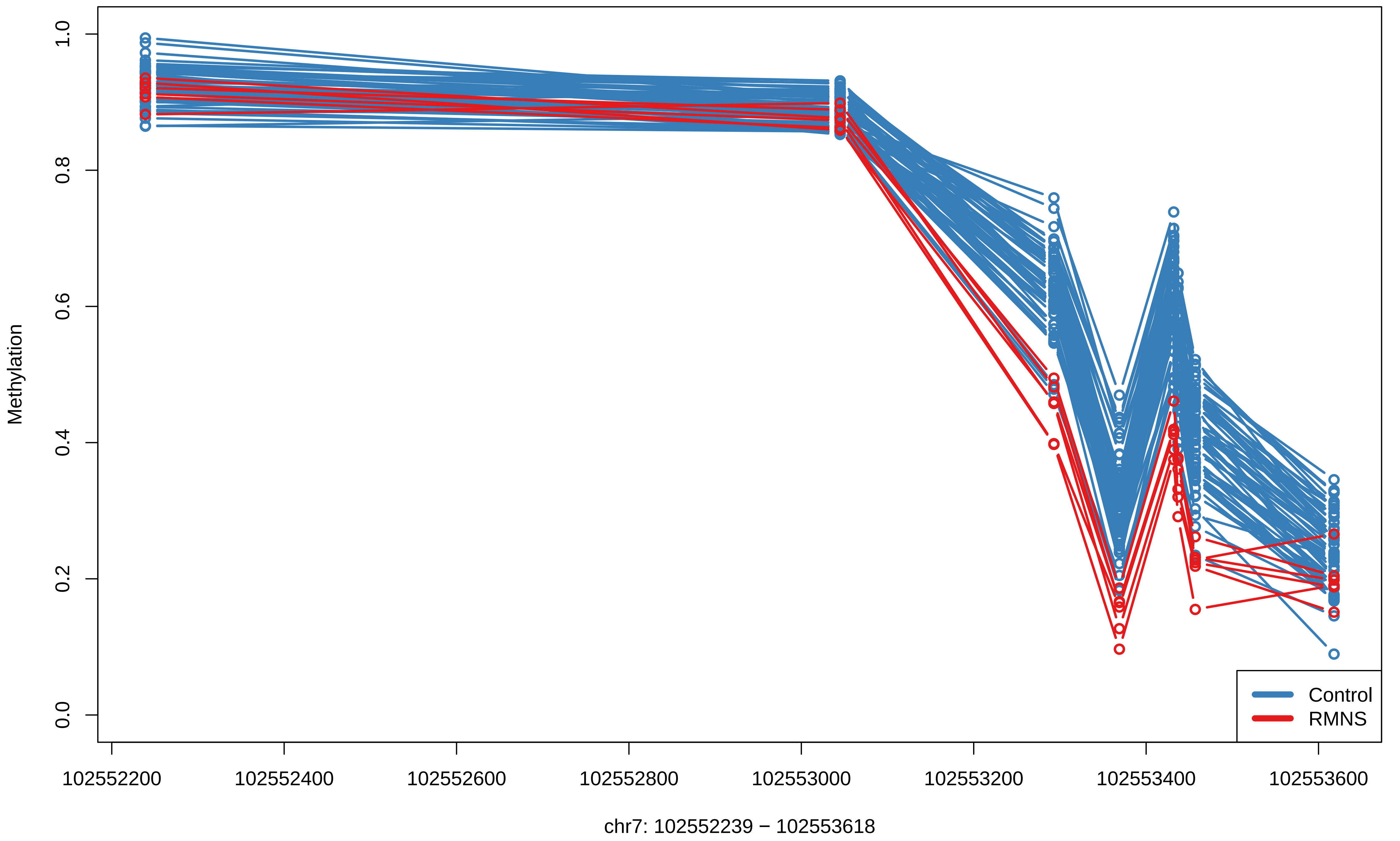

Methylation
1.0
0.8
0.6
0.4
0.2
0.0
102552200
102552400
102552600
102552800
102553000
102553200
102553400
102553600
chr7: 102552239 − 102553618
Control
RMNS

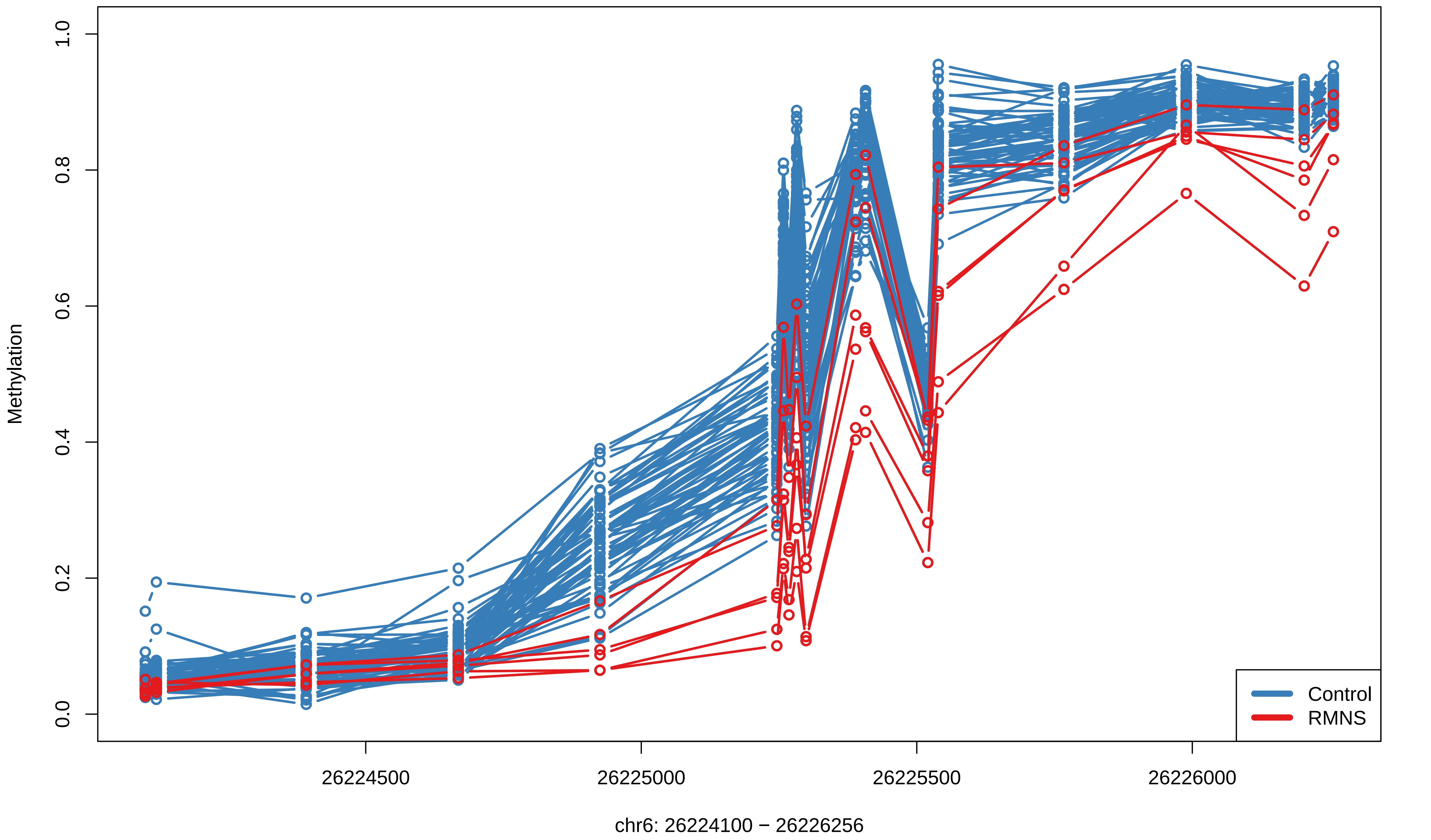

Methylation
0.0
0.2
0.4
0.6
0.8
1.0
26224500
26225000
26225500
26226000
chr6: 26224100 − 26226256
Control
RMNS

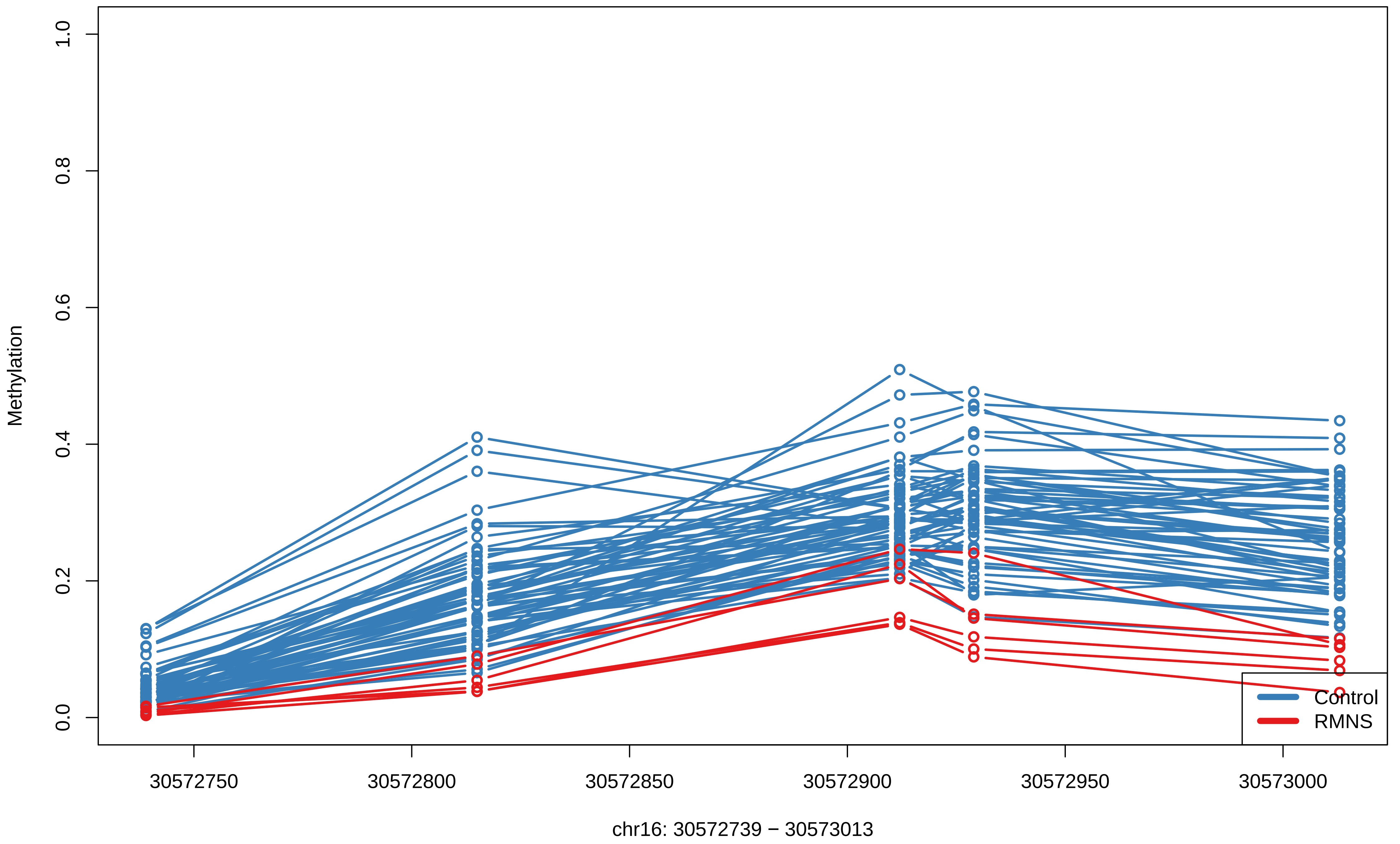

Methylation
1.0
0.8
0.6
0.4
0.2
0.0
30572750
30572800
30572850
30572900
30572950
30573000
chr16: 30572739 − 30573013
Control
RMNS

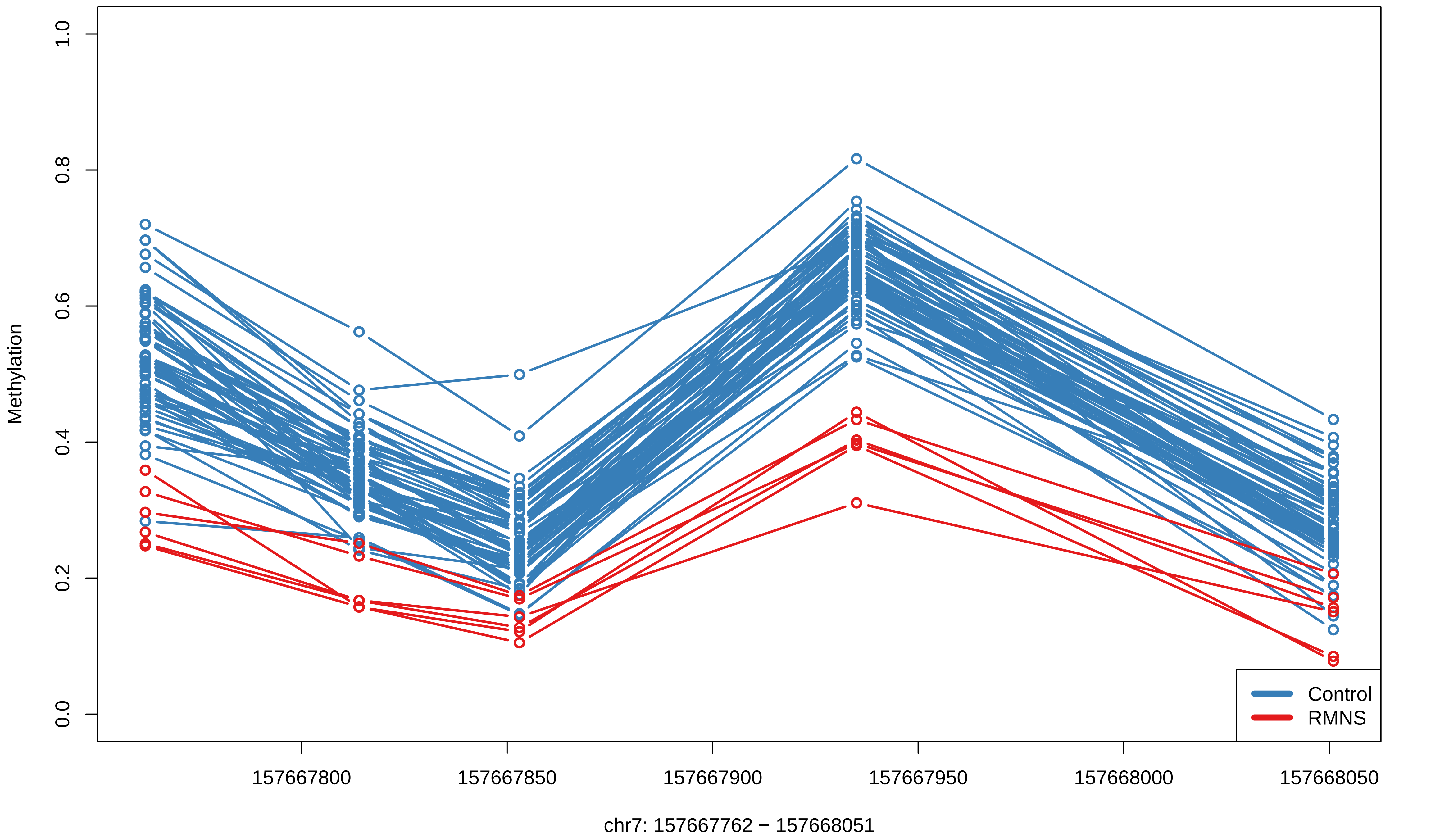

Methylation
1.0
0.8
0.6
0.4
0.2
0.0
157667800
157667850
157667900
157667950
157668000
157668050
chr7: 157667762 − 157668051
Control
RMNS

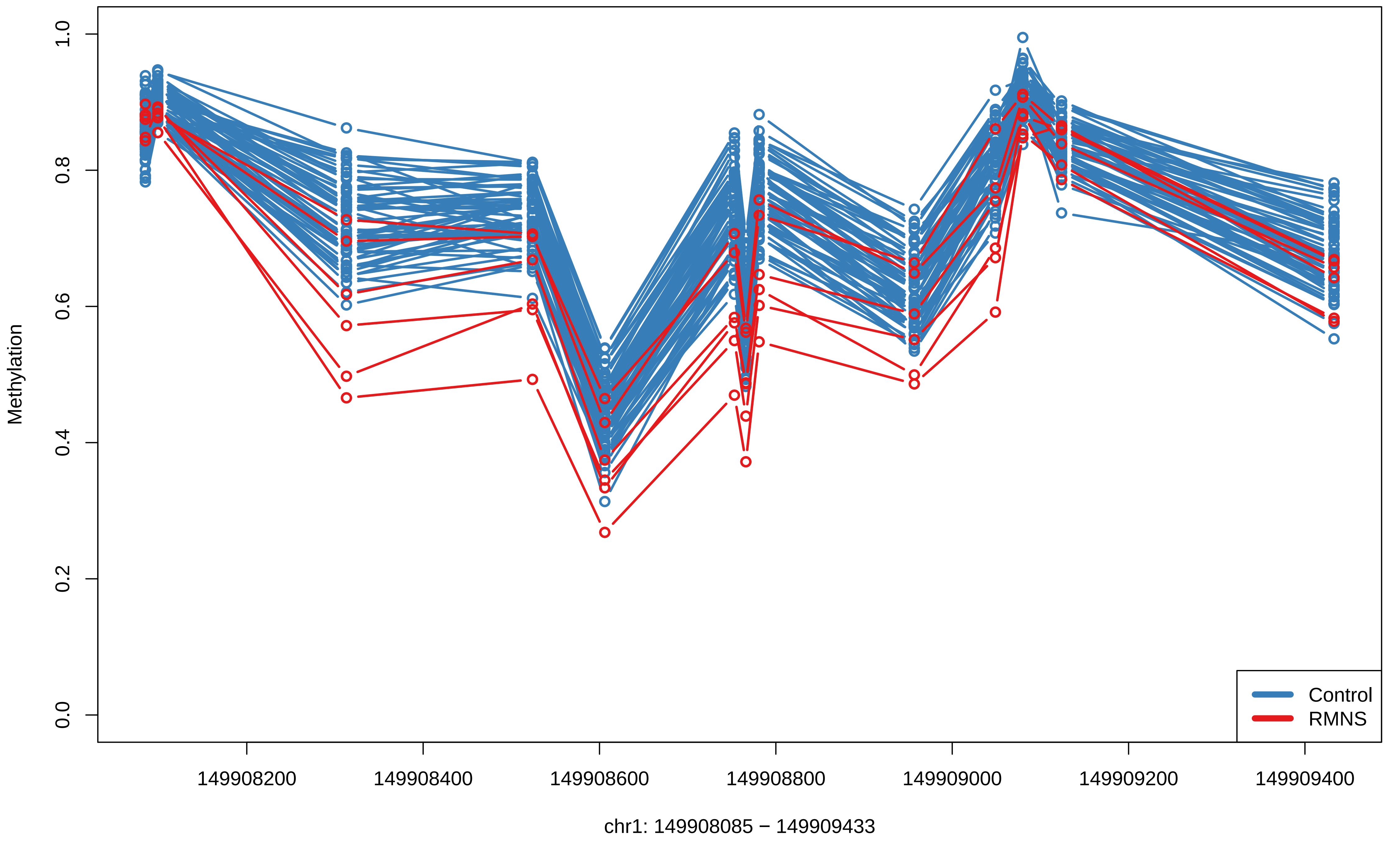

1.0
0.8
0.6
0.4
0.2
0.0
Methylation
149908200
149908400
149908600
149908800
149909000
149909200
149909400
chr1: 149908085 − 149909433
Control
RMNS

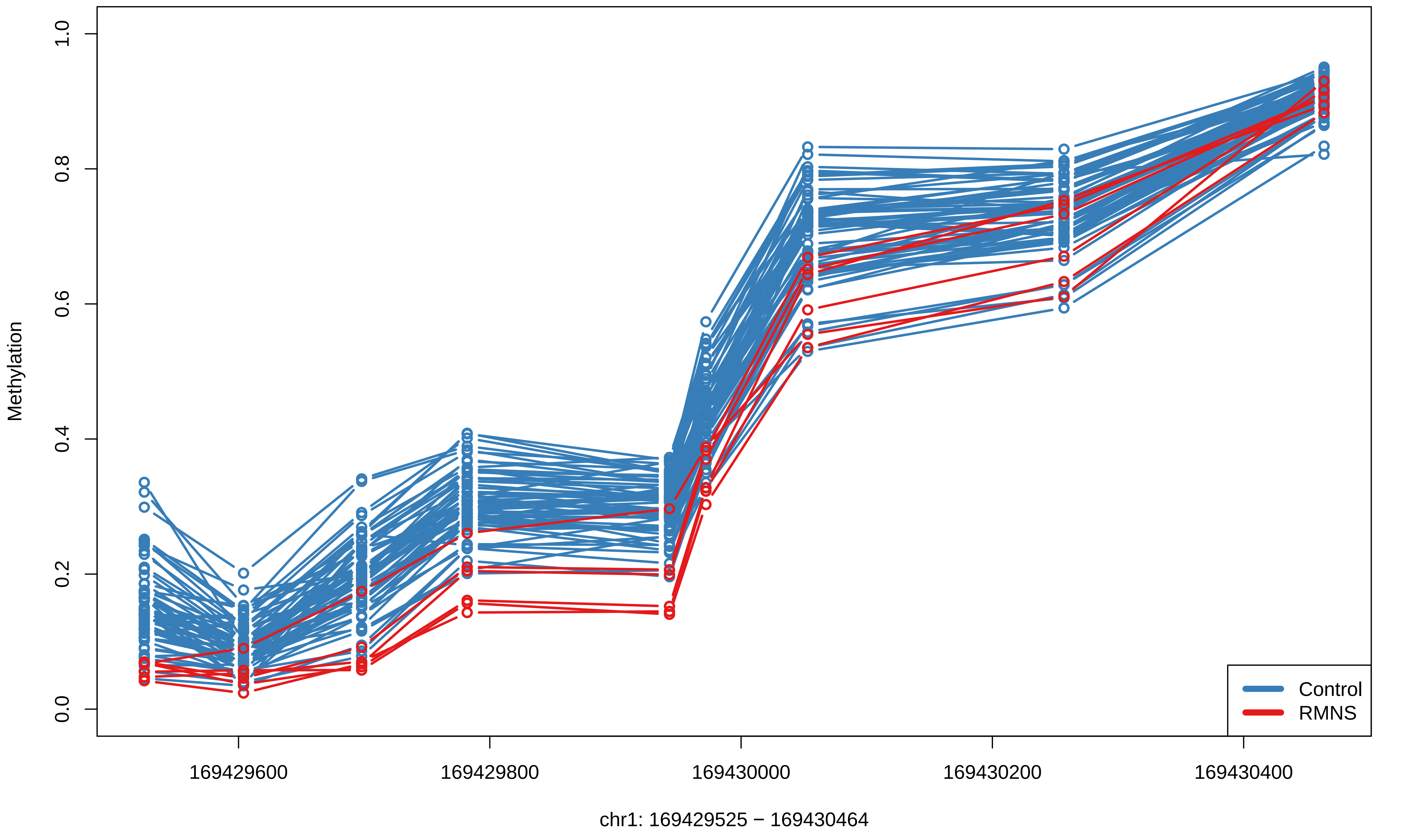
Methylation
0.0
0.2
0.4
0.6
0.8
1.0
169429600
169429800
169430000
169430200
169430400
chr1: 169429525 − 169430464
Control
RMNS

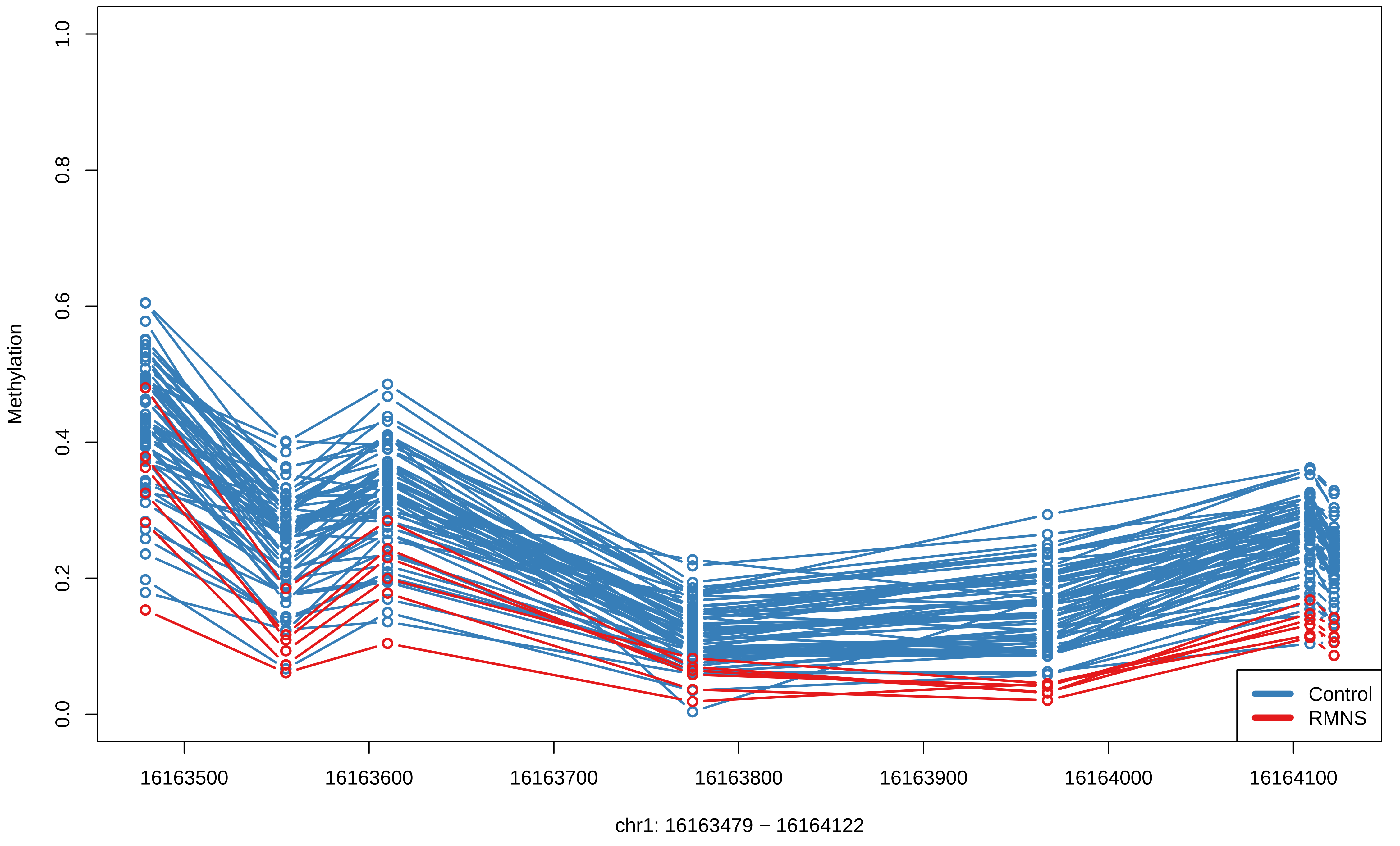

Methylation
0.0
0.2
0.4
0.6
0.8
1.0
16163500
16163600
16163700
16163800
16163900
16164000
16164100
chr1: 16163479 − 16164122
Control
RMNS

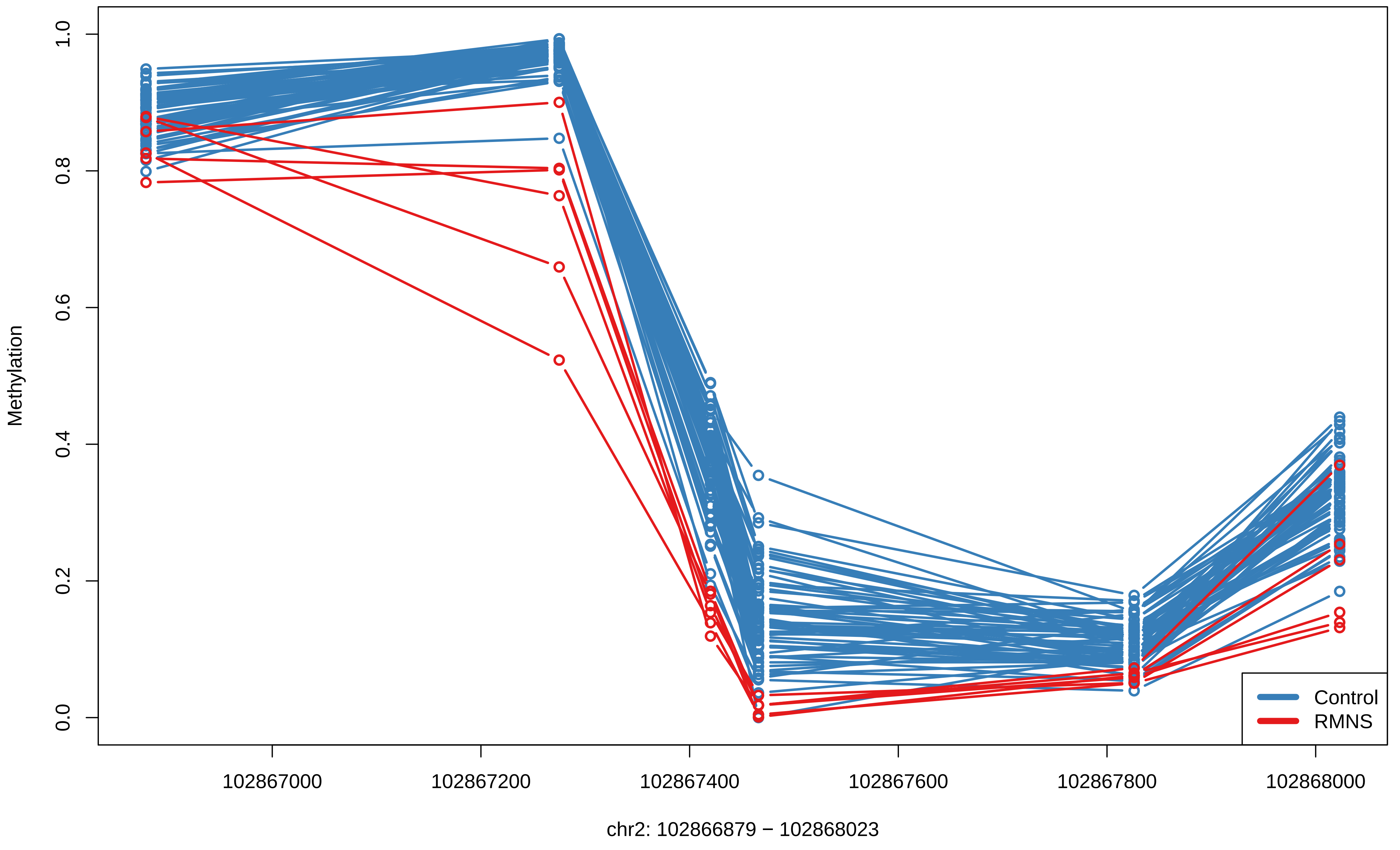

Methylation
1.0
0.8
0.6
0.4
0.2
0.0
102867000
102867200
102867400
102867600
102867800
102868000
chr2: 102866879 − 102868023
Control
RMNS

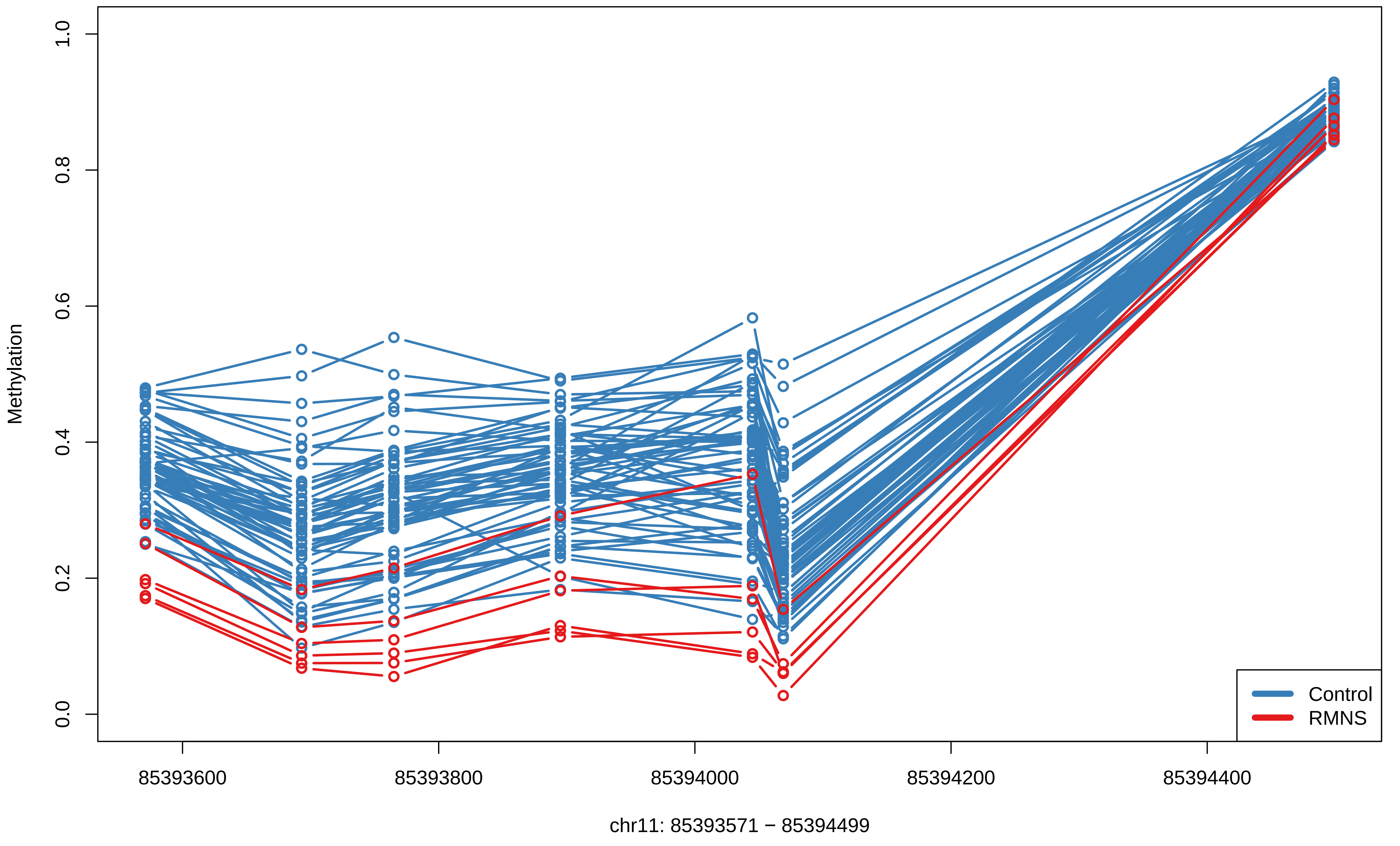

Methylation
0.0
0.2
0.4
0.6
0.8
1.0
85393600
85393800
85394000
85394200
85394400
chr11: 85393571 − 85394499
Control
RMNS

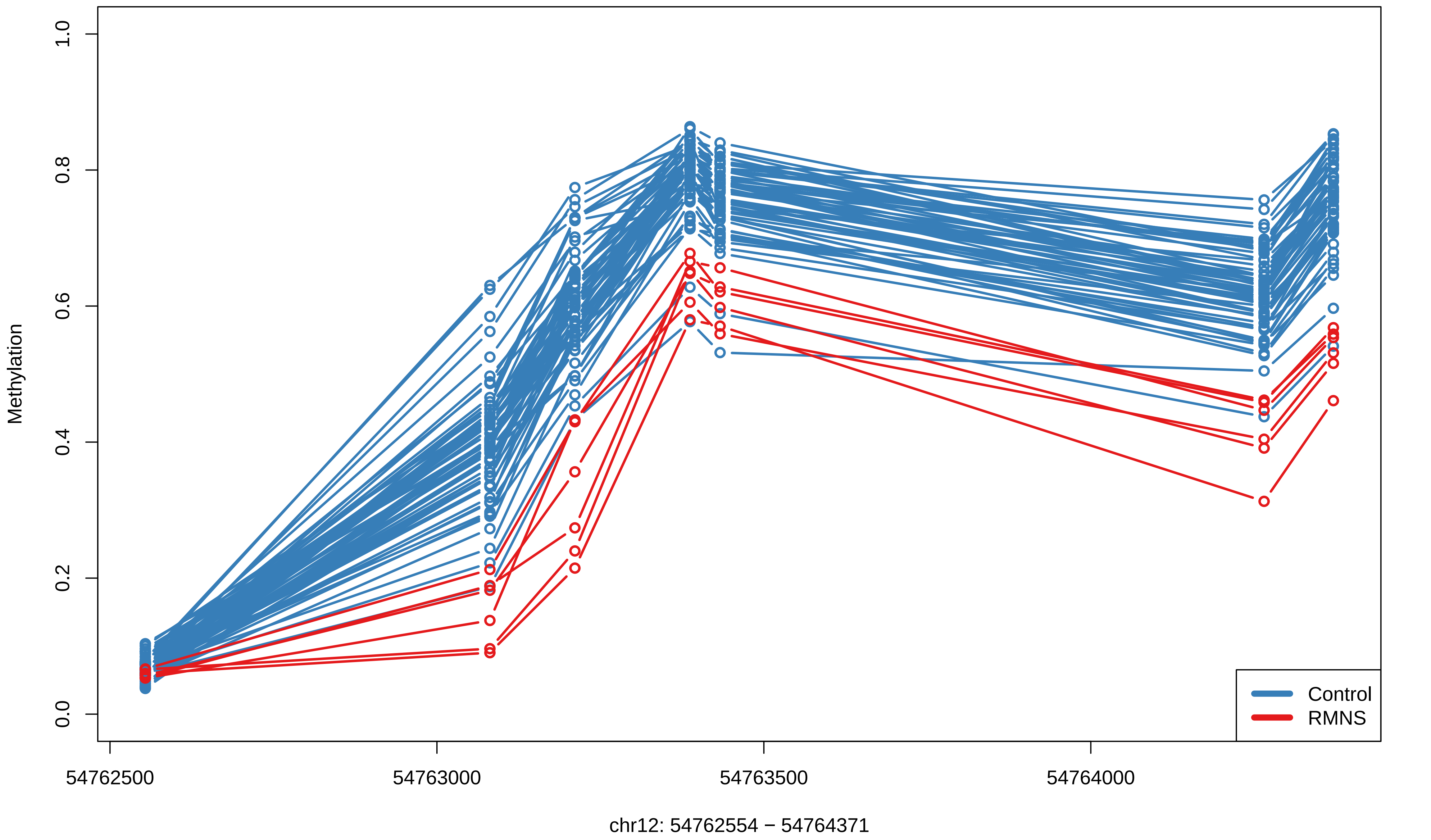

Methylation
1.0
0.8
0.6
0.4
0.2
0.0
54762500
54763000
54763500
54764000
chr12: 54762554 − 54764371
Control
RMNS

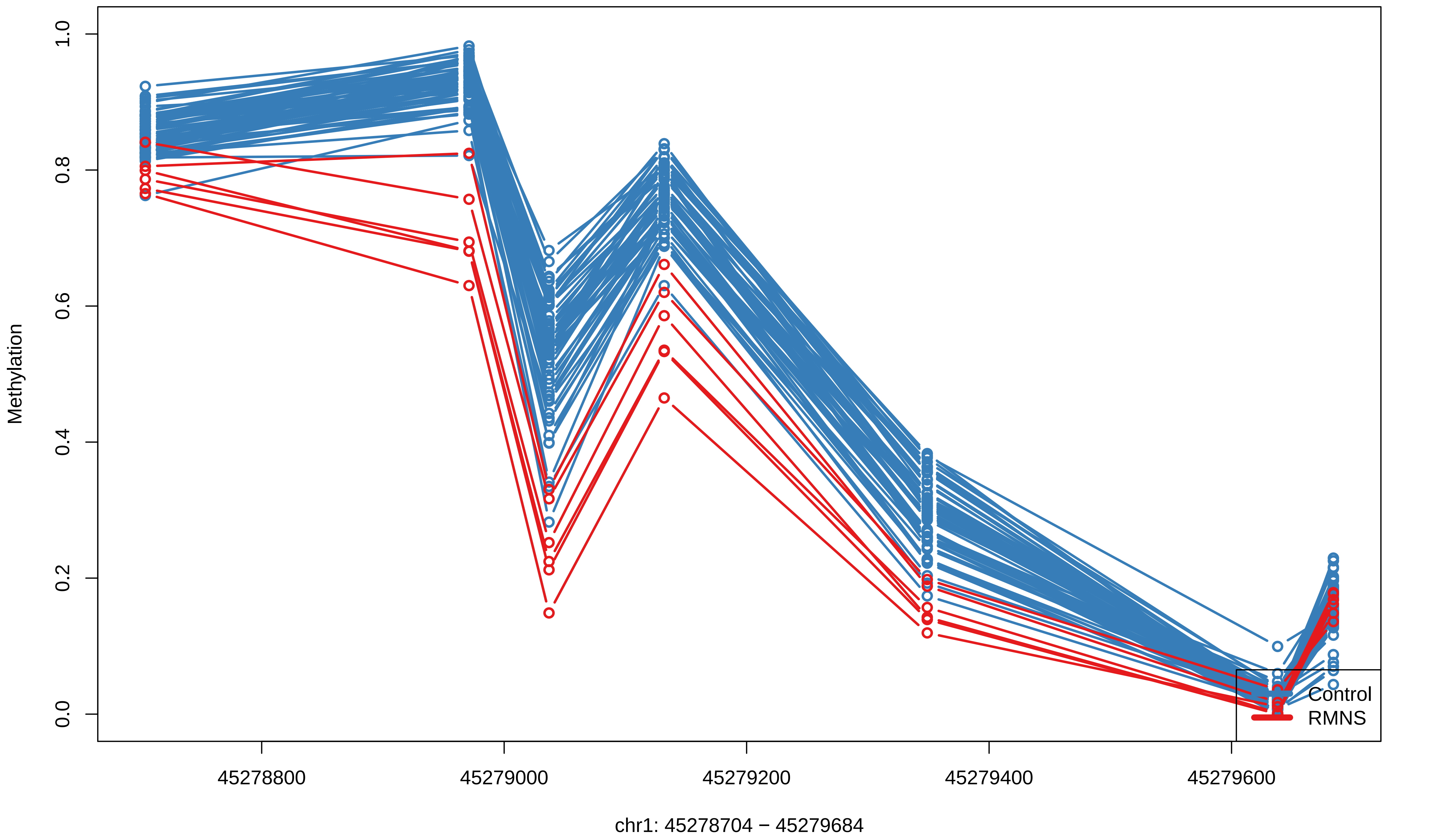

1.0
0.8
0.6
0.4
0.2
0.0
Methylation
45278800
45279000
45279200
45279400
45279600
chr1: 45278704 − 45279684
Control
RMNS

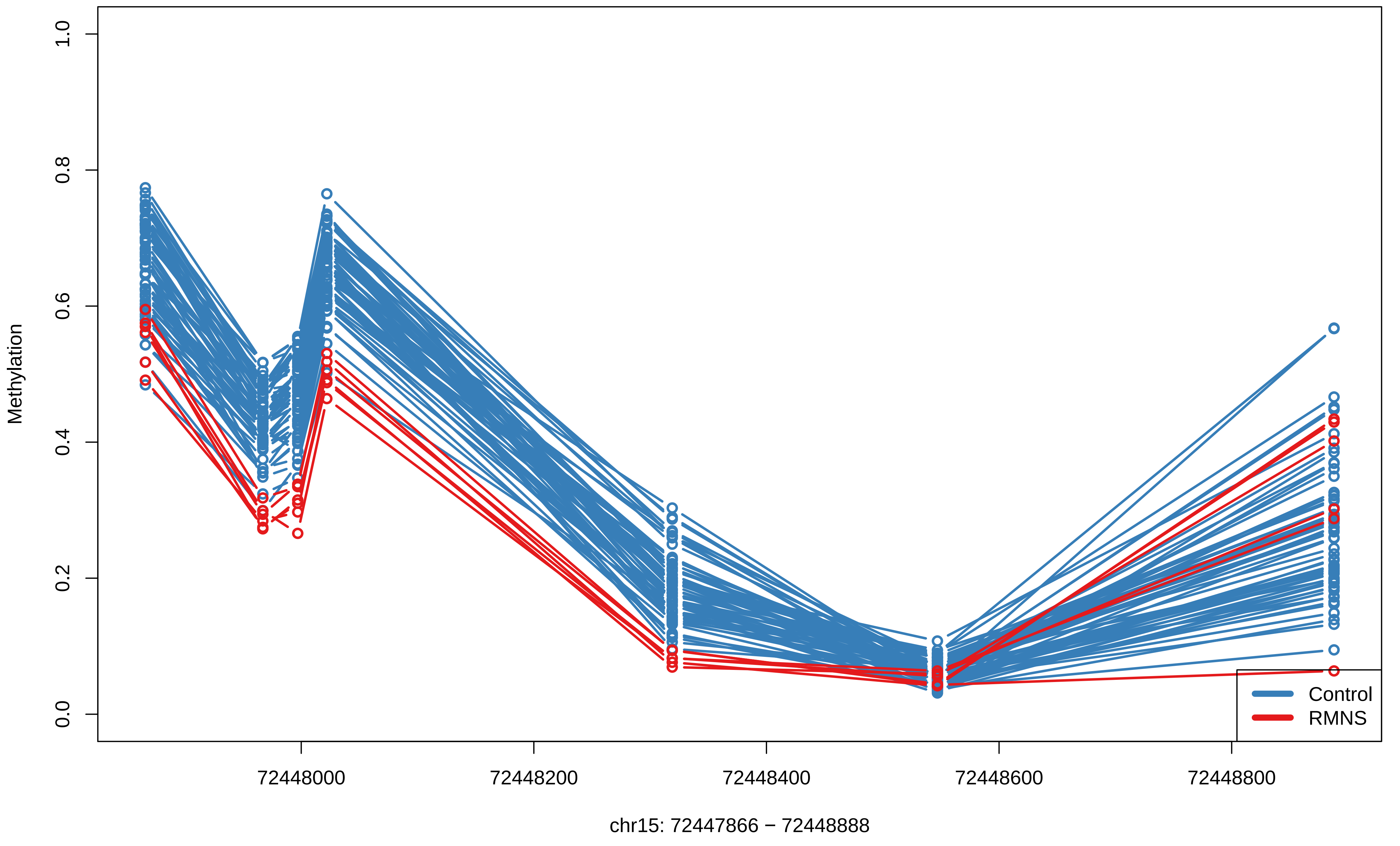

1.0
0.8
0.6
0.4
0.2
0.0
Methylation
72448000
72448200
72448400
72448600
72448800
chr15: 72447866 − 72448888
Control
RMNS

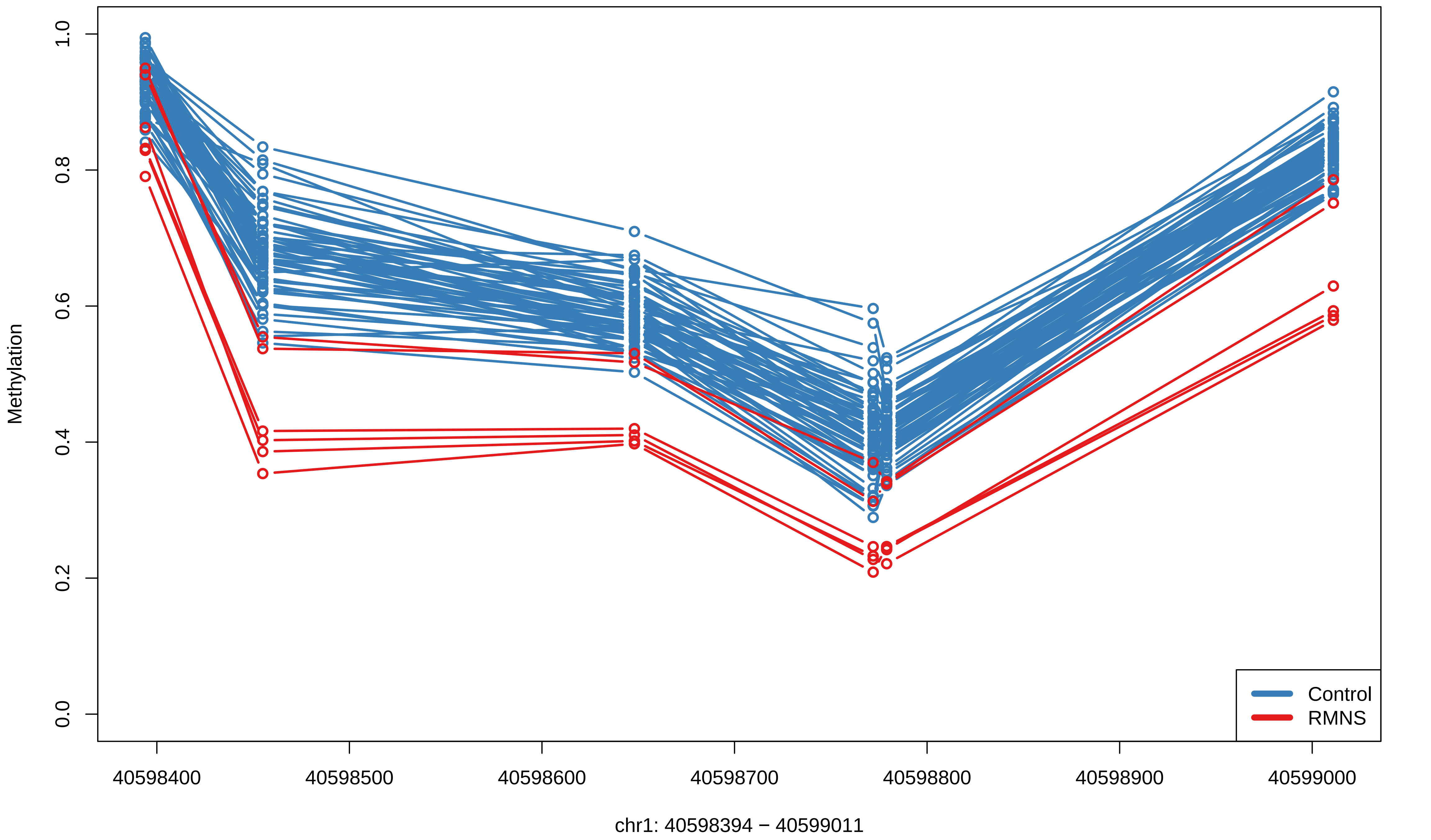

1.0
0.8
0.6
0.4
0.2
0.0
Methylation
40598400
40598500
40598600
40598700
40598800
40598900
40599000
chr1: 40598394 − 40599011
Control
RMNS

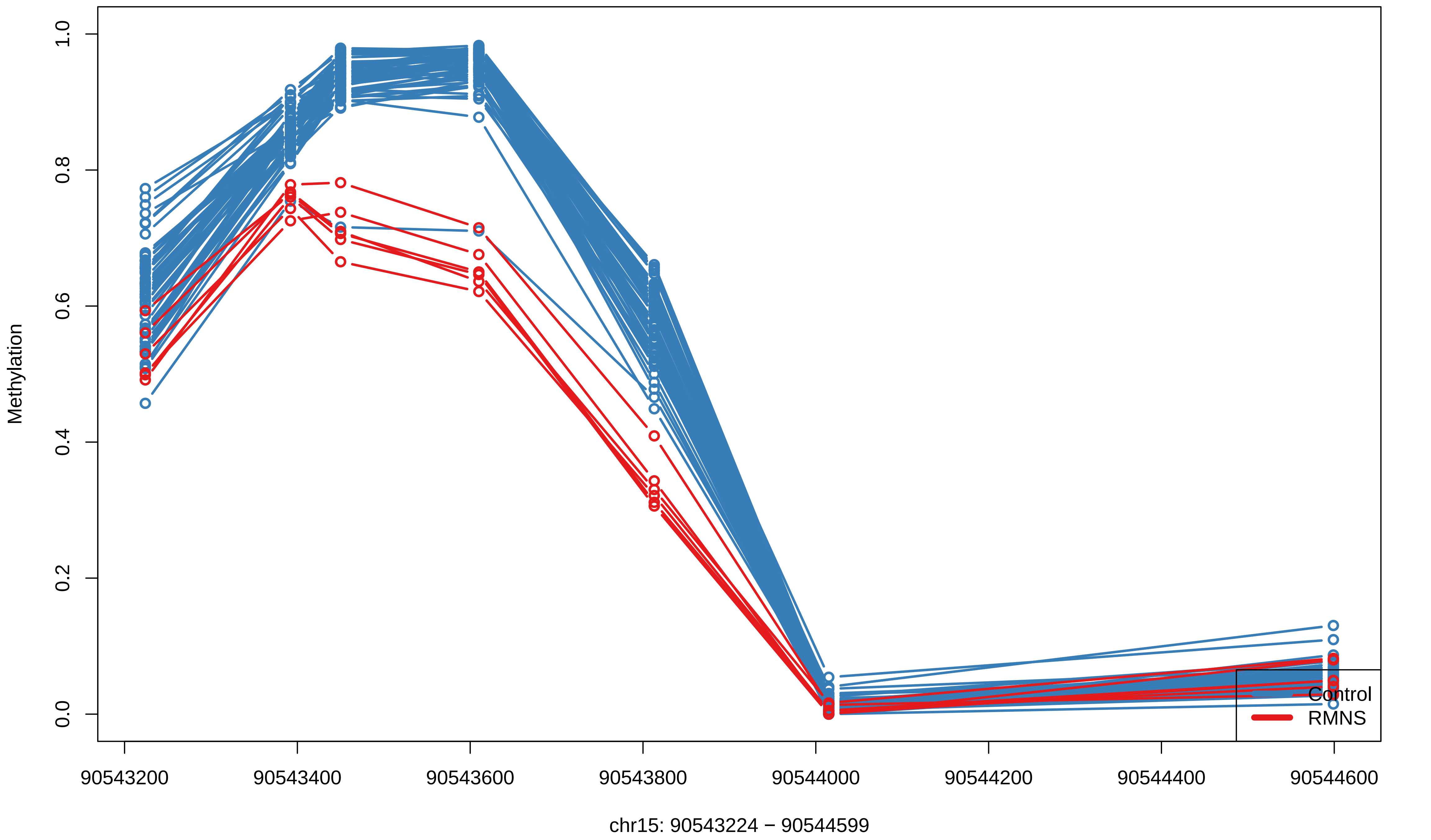

Methylation
0.0
0.2
0.4
0.6
0.8
1.0
90543200
90543400
90543600
90543800
90544000
90544200
90544400
90544600
chr15: 90543224 − 90544599
Control
RMNS

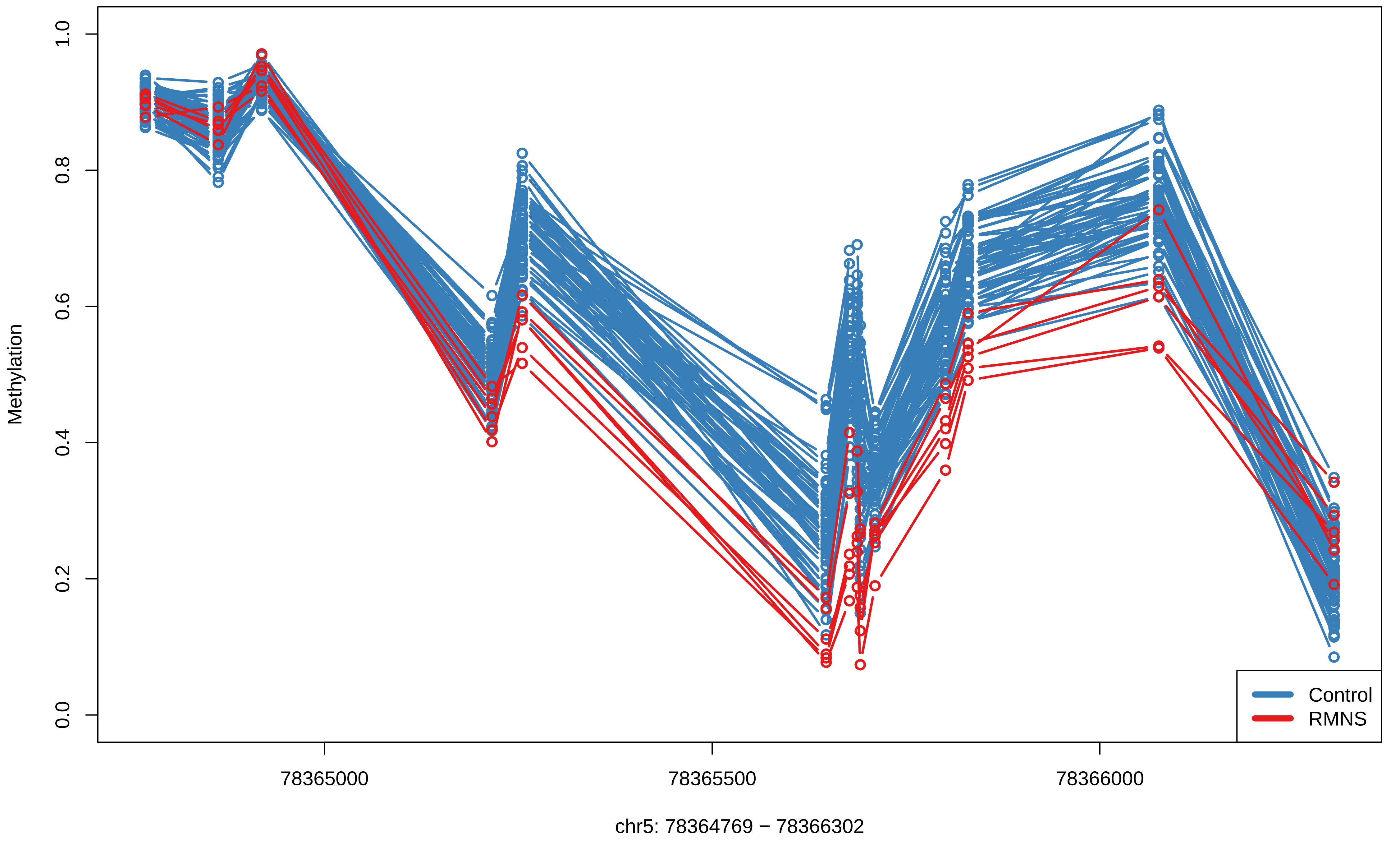

Methylation
0.0
0.2
0.4
0.6
0.8
1.0
78365000
78365500
78366000
chr5: 78364769 − 78366302
Control
RMNS

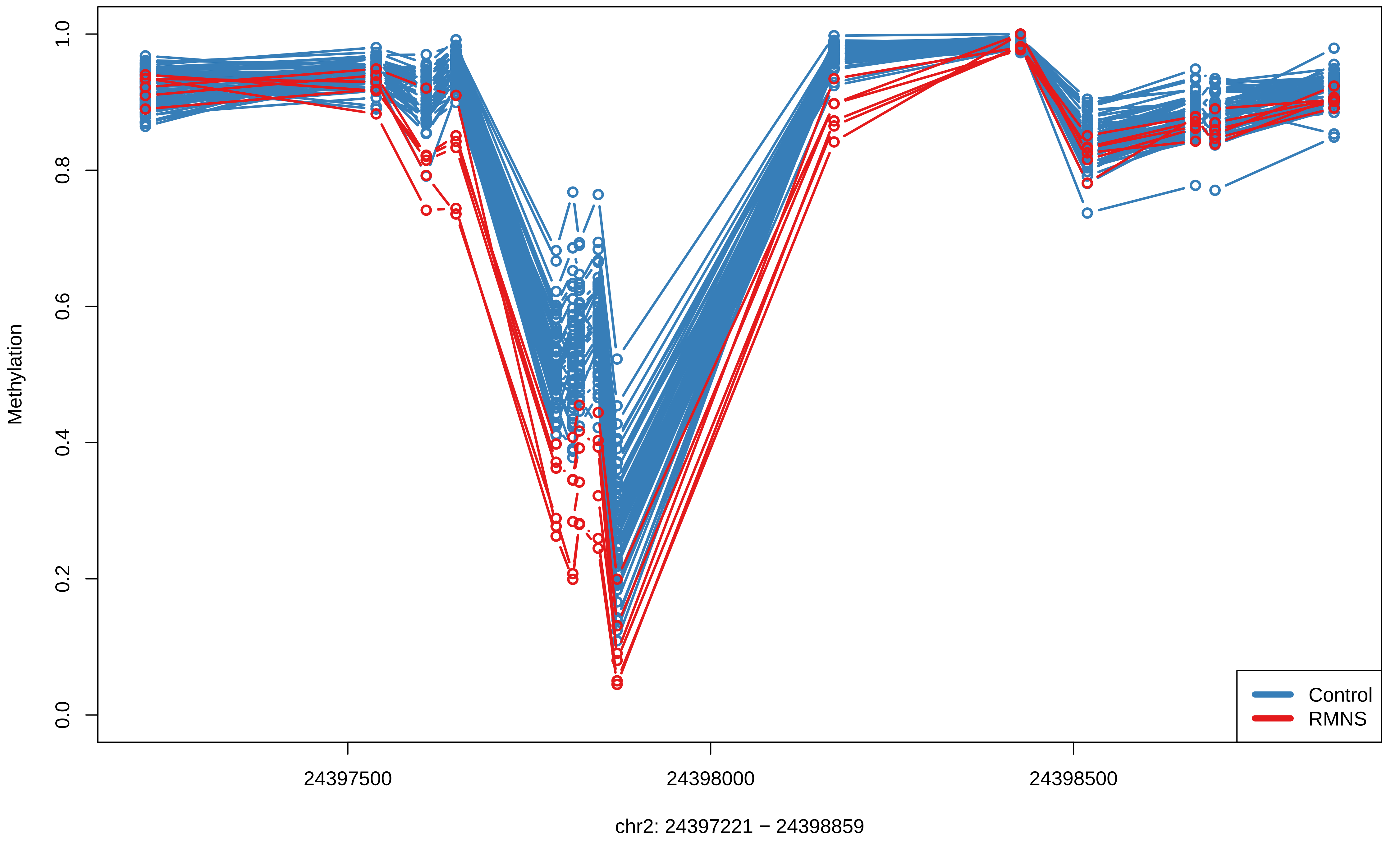

1.0
0.8
0.6
0.4
0.2
0.0
Methylation
24397500
24398000
24398500
chr2: 24397221 − 24398859
Control
RMNS

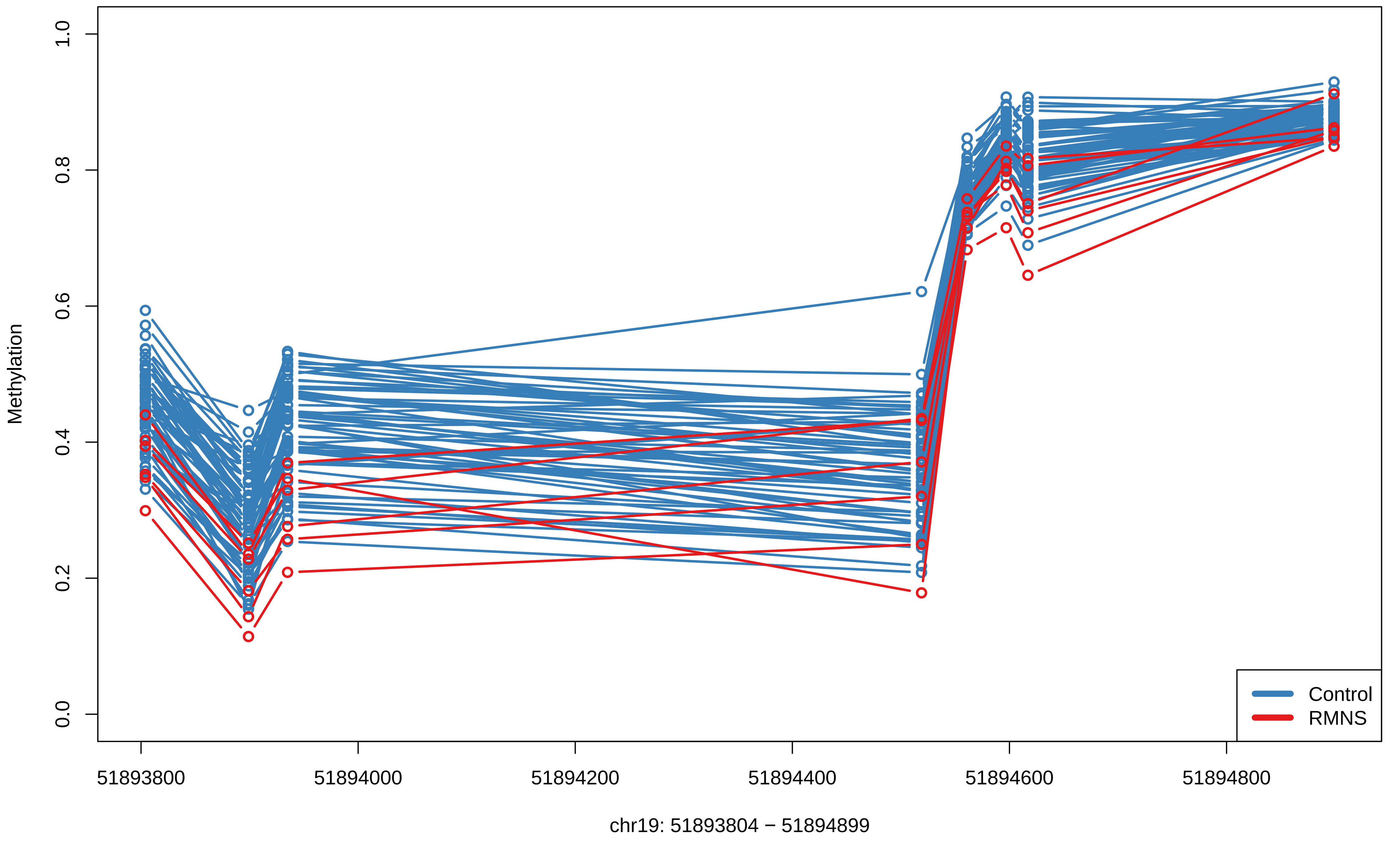

Methylation
0.0
0.2
0.4
0.6
0.8
1.0
51893800
51894000
51894200
51894400
51894600
51894800
chr19: 51893804 − 51894899
Control
RMNS

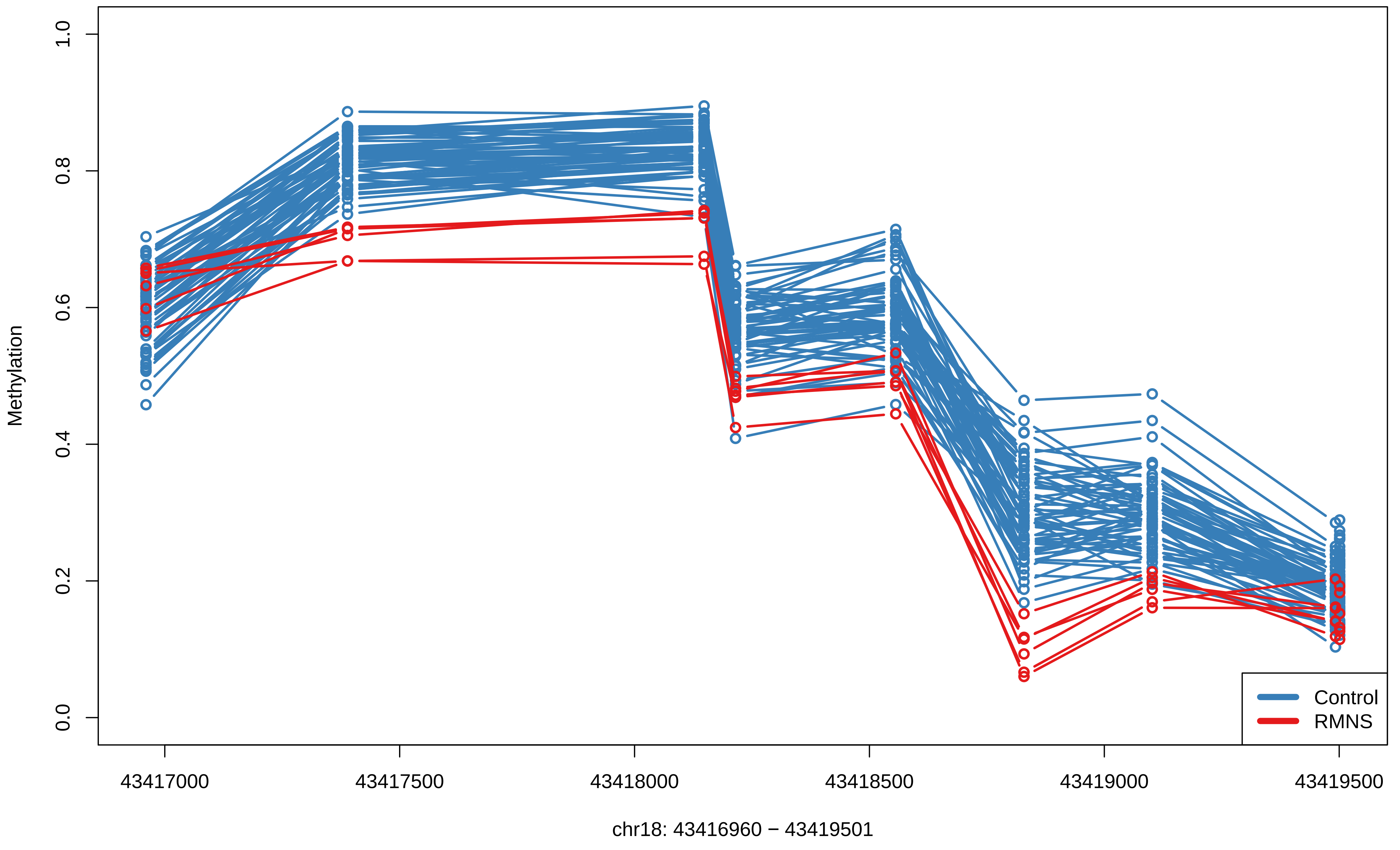

Methylation
1.0
0.8
0.6
0.4
0.2
0.0
43417000
43417500
43418000
43418500
43419000
43419500
chr18: 43416960 − 43419501
Control
RMNS

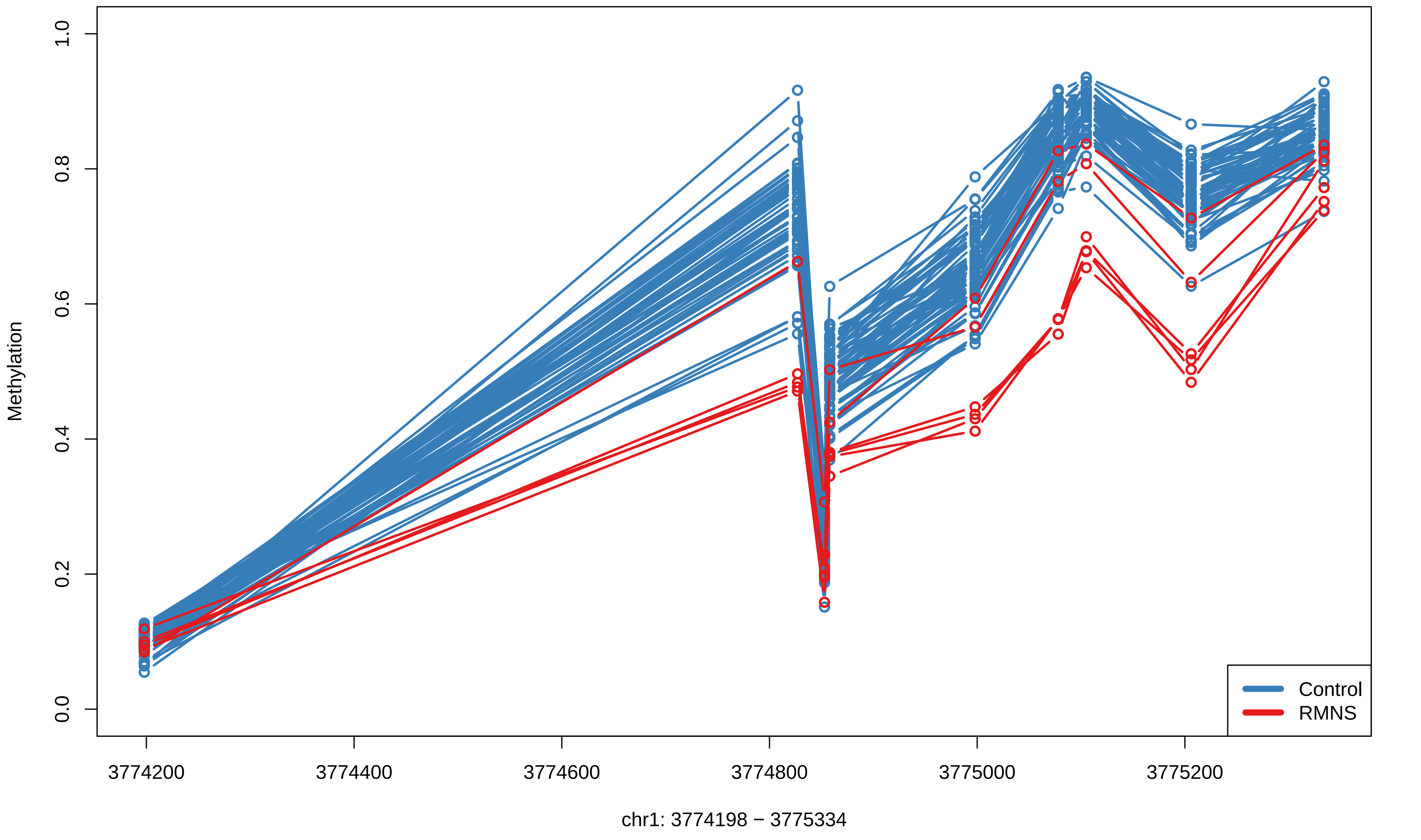

Methylation
1.0
0.8
0.6
0.4
0.2
0.0
3774200
3774400
3774600
3774800
3775000
3775200
chr1: 3774198 − 3775334
Control
RMNS

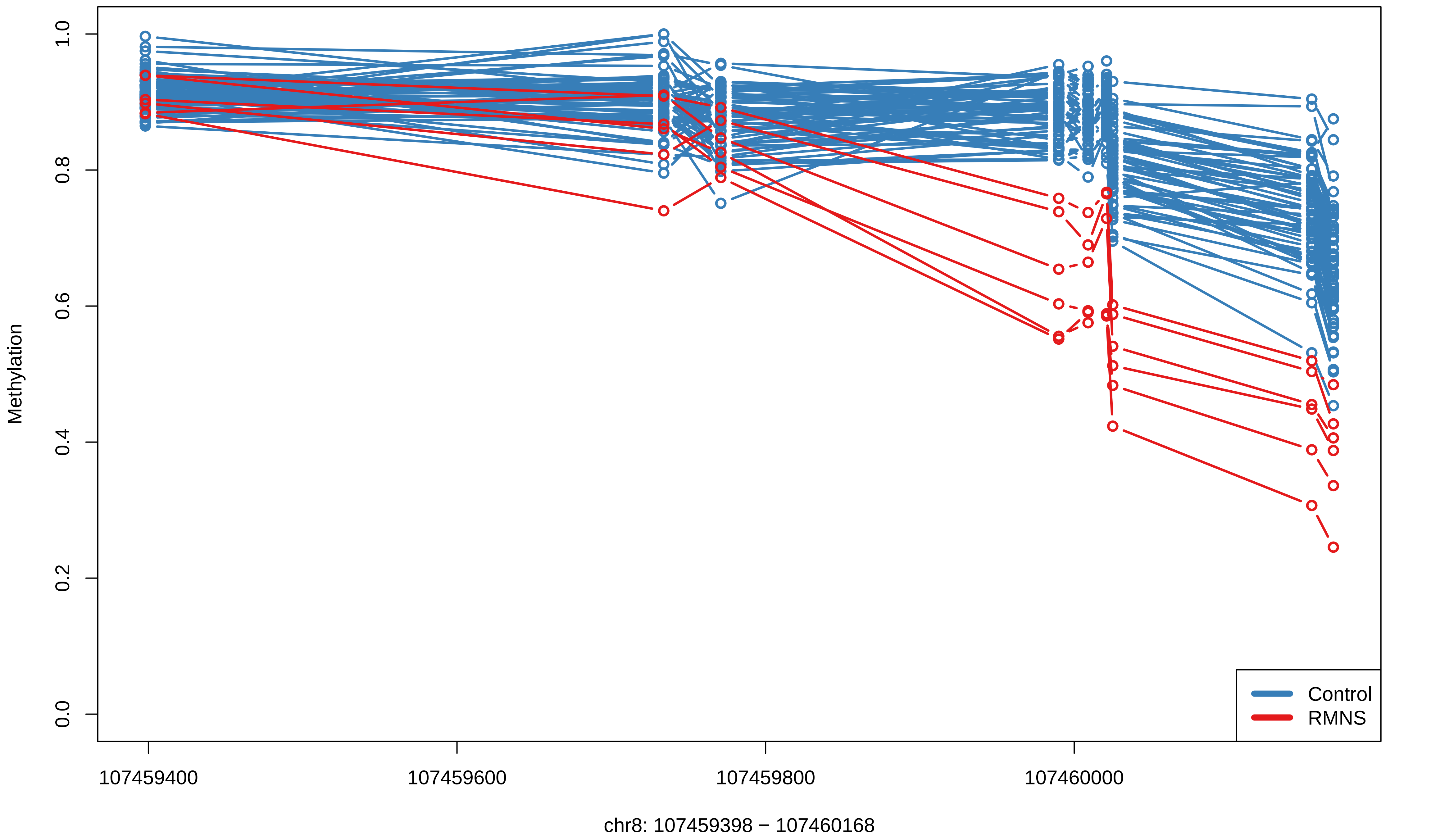

Methylation
1.0
0.8
0.6
0.4
0.2
0.0
107459400
107459600
107459800
107460000
chr8: 107459398 − 107460168
Control
RMNS

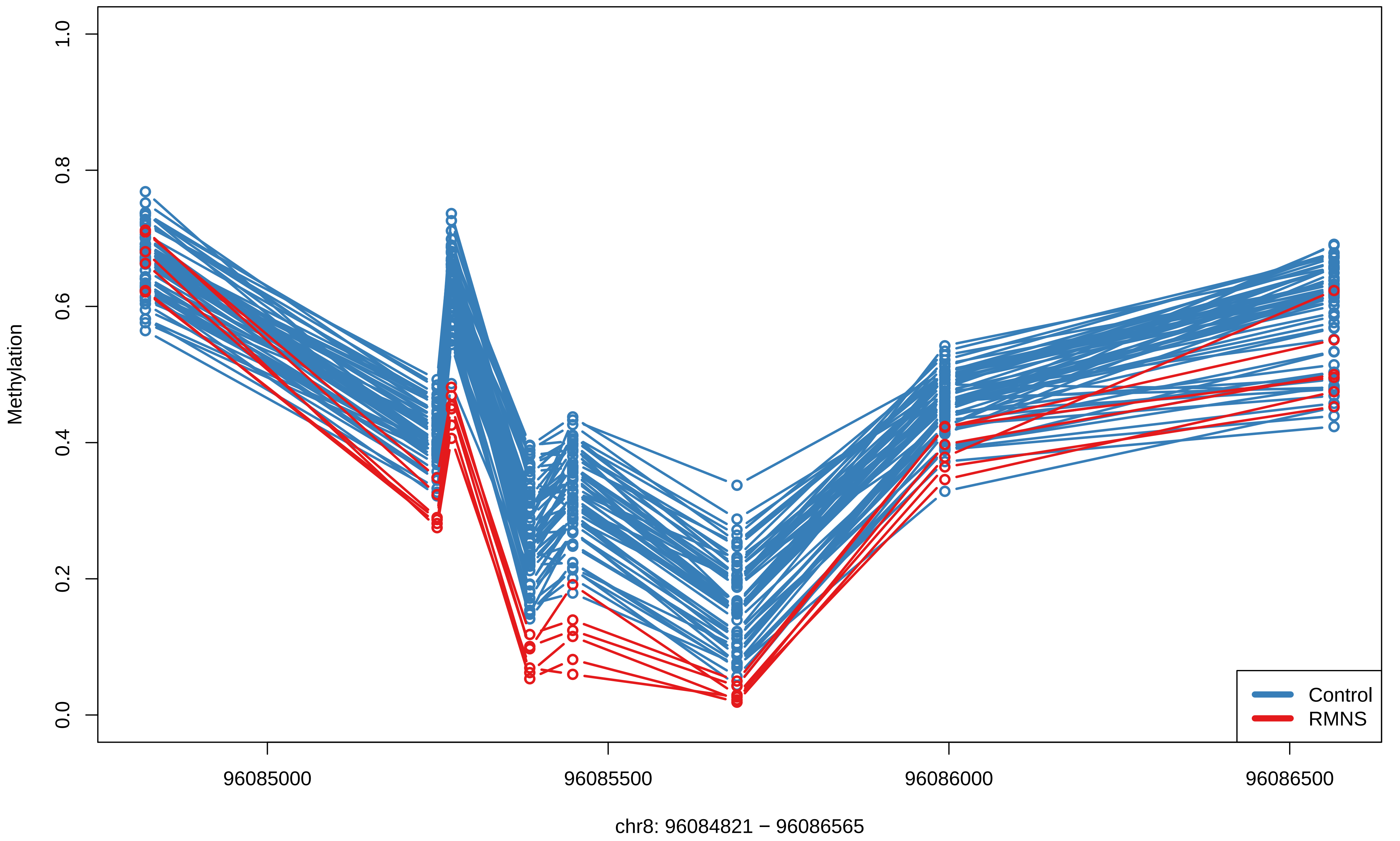
Methylation
0.0
0.2
0.4
0.6
0.8
1.0
96085000
96085500
96086000
96086500
chr8: 96084821 − 96086565
Control
RMNS

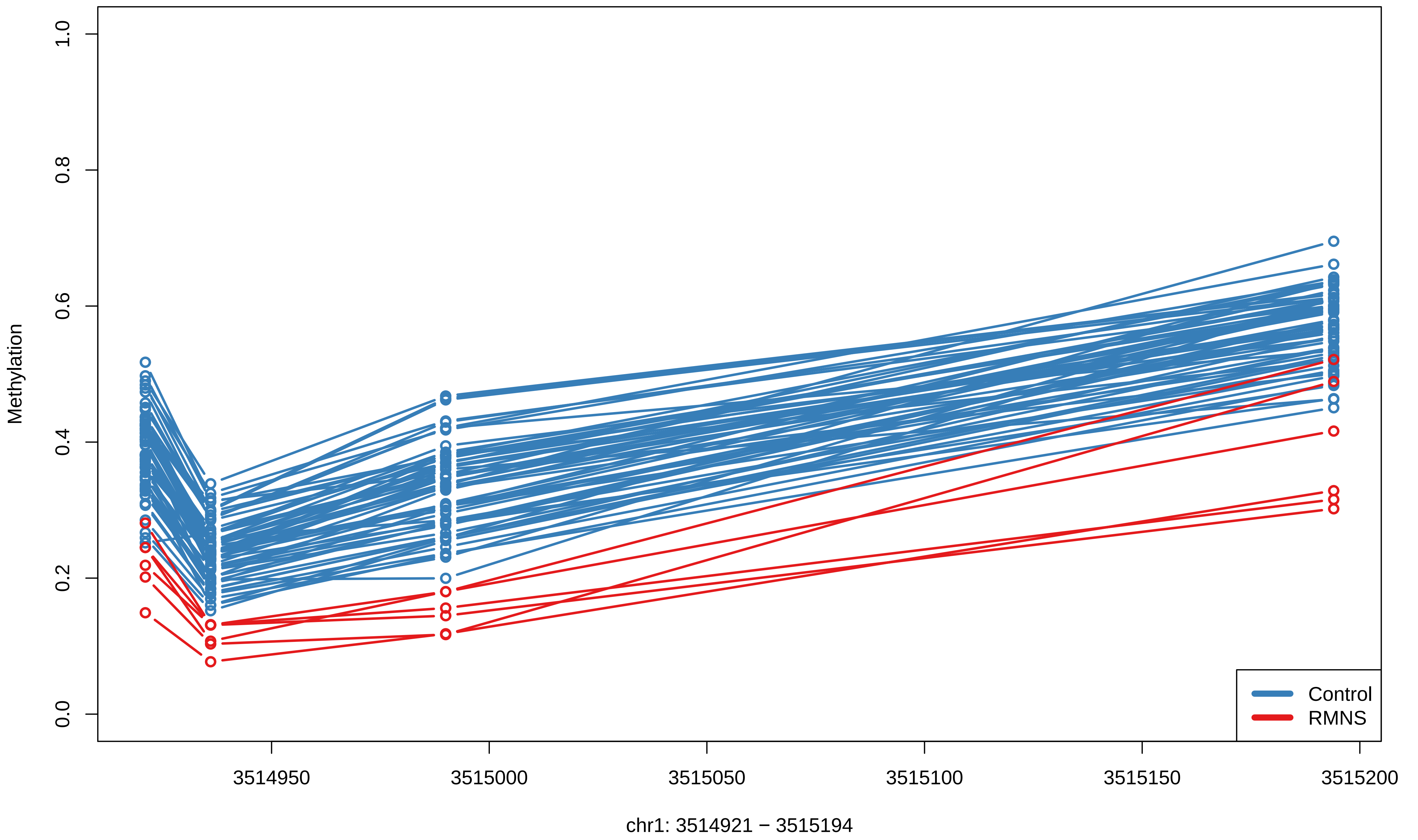

Methylation
0.0
0.2
0.4
0.6
0.8
1.0
3514950
3515000
3515050
3515100
3515150
3515200
chr1: 3514921 − 3515194
Control
RMNS

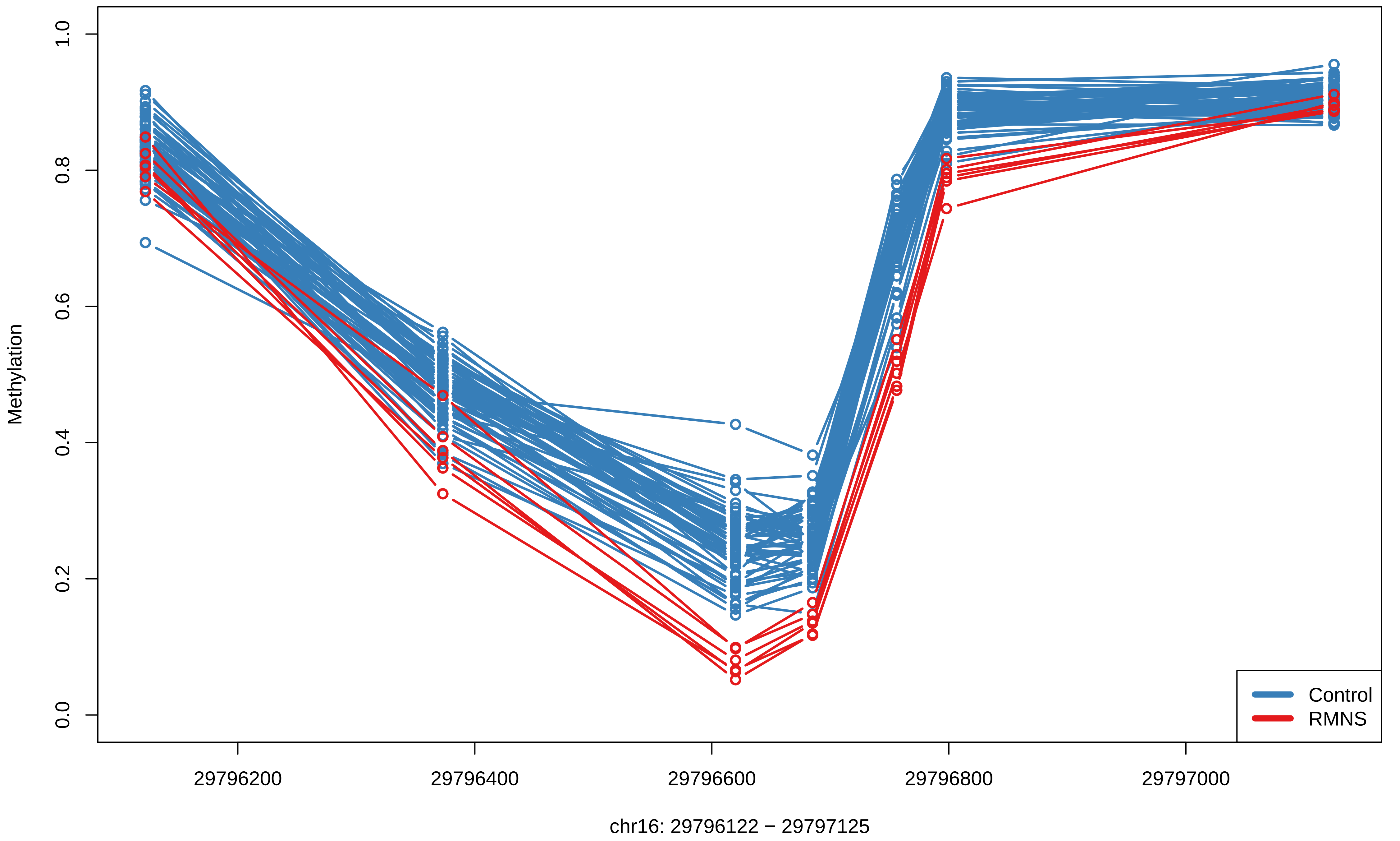

Methylation
0.0
0.2
0.4
0.6
0.8
1.0
29796200
29796400
29796600
29796800
29797000
chr16: 29796122 − 29797125
Control
RMNS

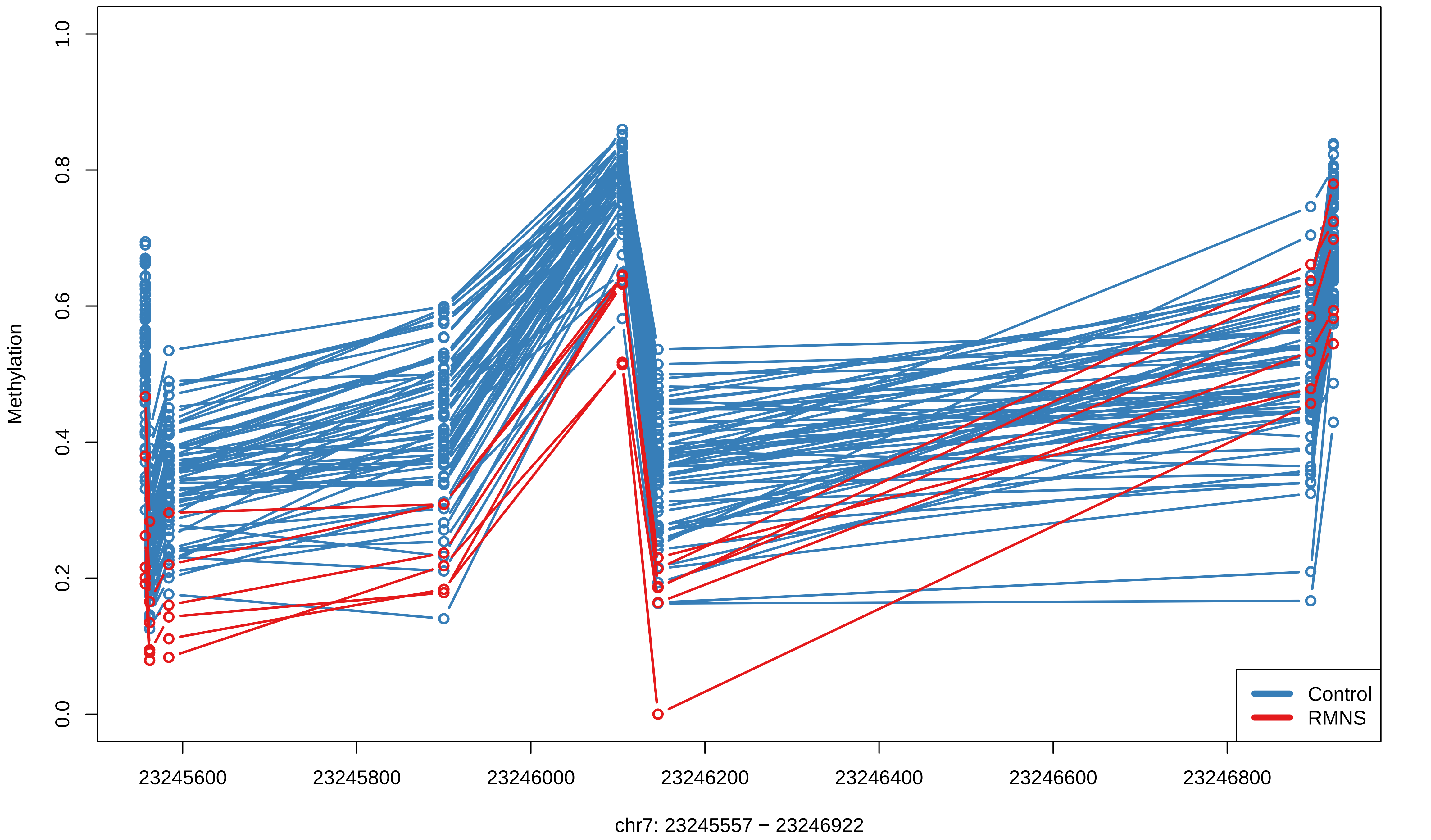

Methylation
0.0
0.2
0.4
0.6
0.8
1.0
23245600
23245800
23246000
23246200
23246400
23246600
23246800
chr7: 23245557 − 23246922
Control
RMNS

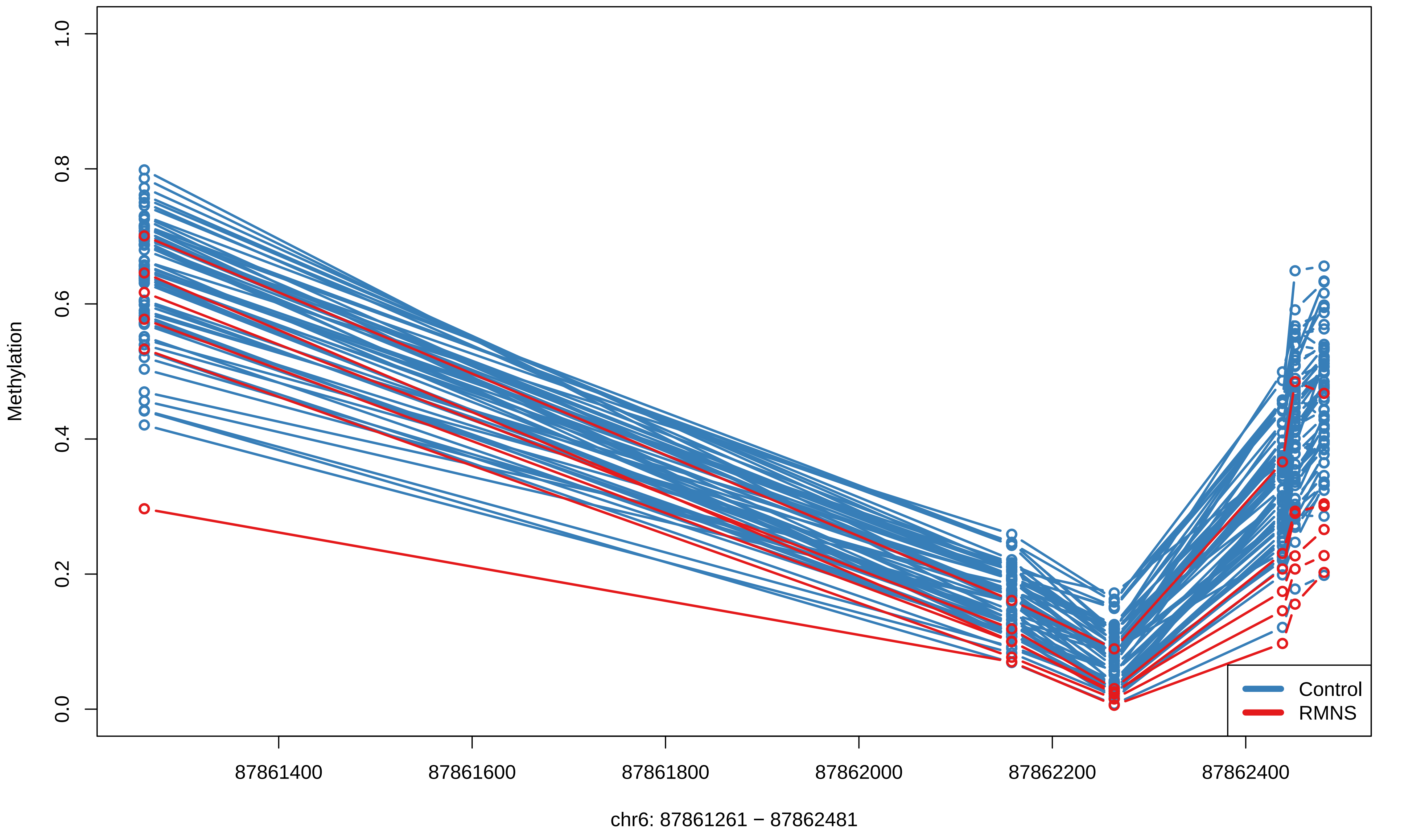

Methylation
0.0
0.2
0.4
0.6
0.8
1.0
87861400
87861600
87861800
87862000
87862200
87862400
chr6: 87861261 − 87862481
Control
RMNS

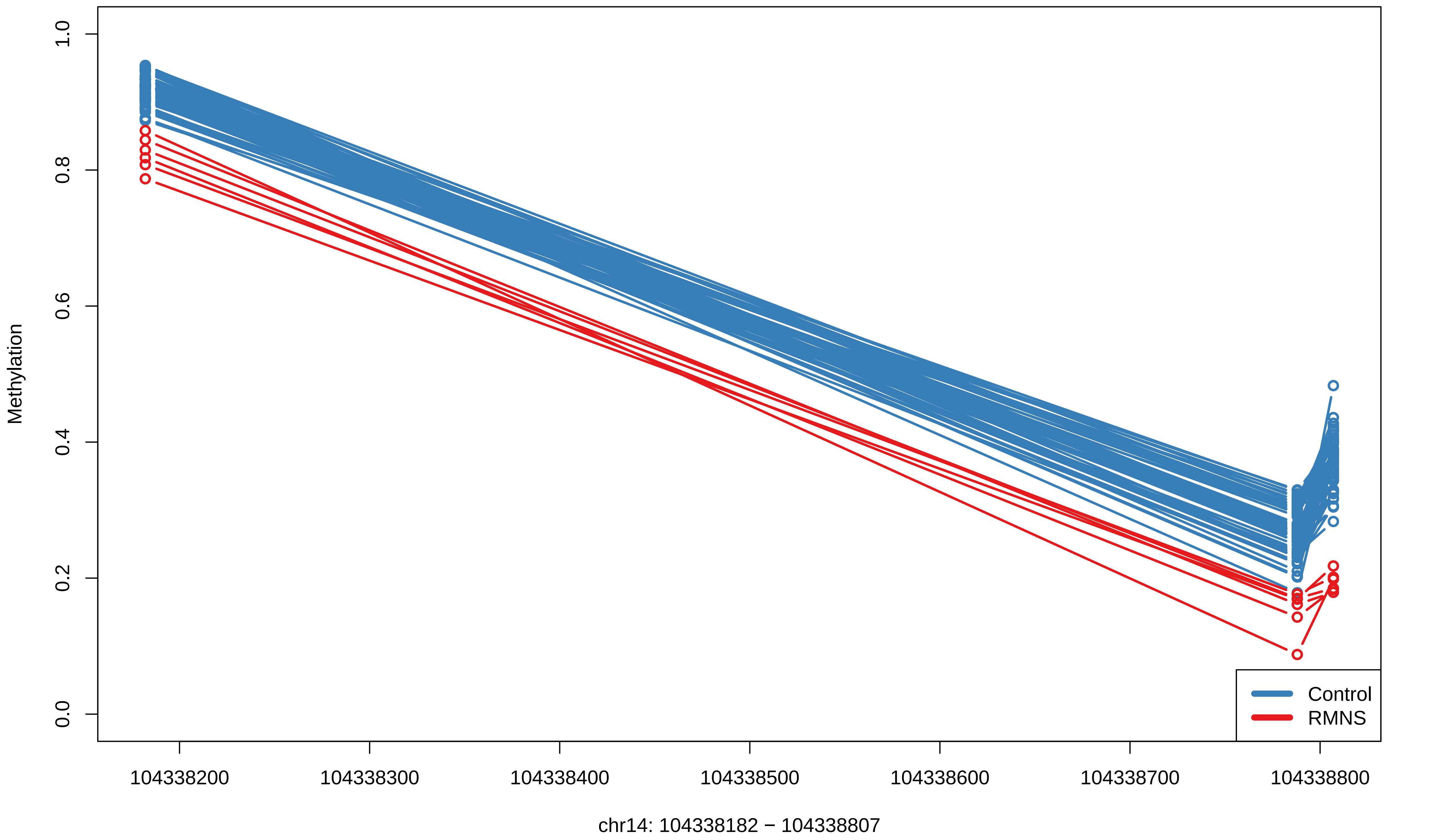

Methylation
0.0
0.2
0.4
0.6
0.8
1.0
104338200
104338300
104338400
104338500
104338600
104338700
104338800
chr14: 104338182 − 104338807
Control
RMNS

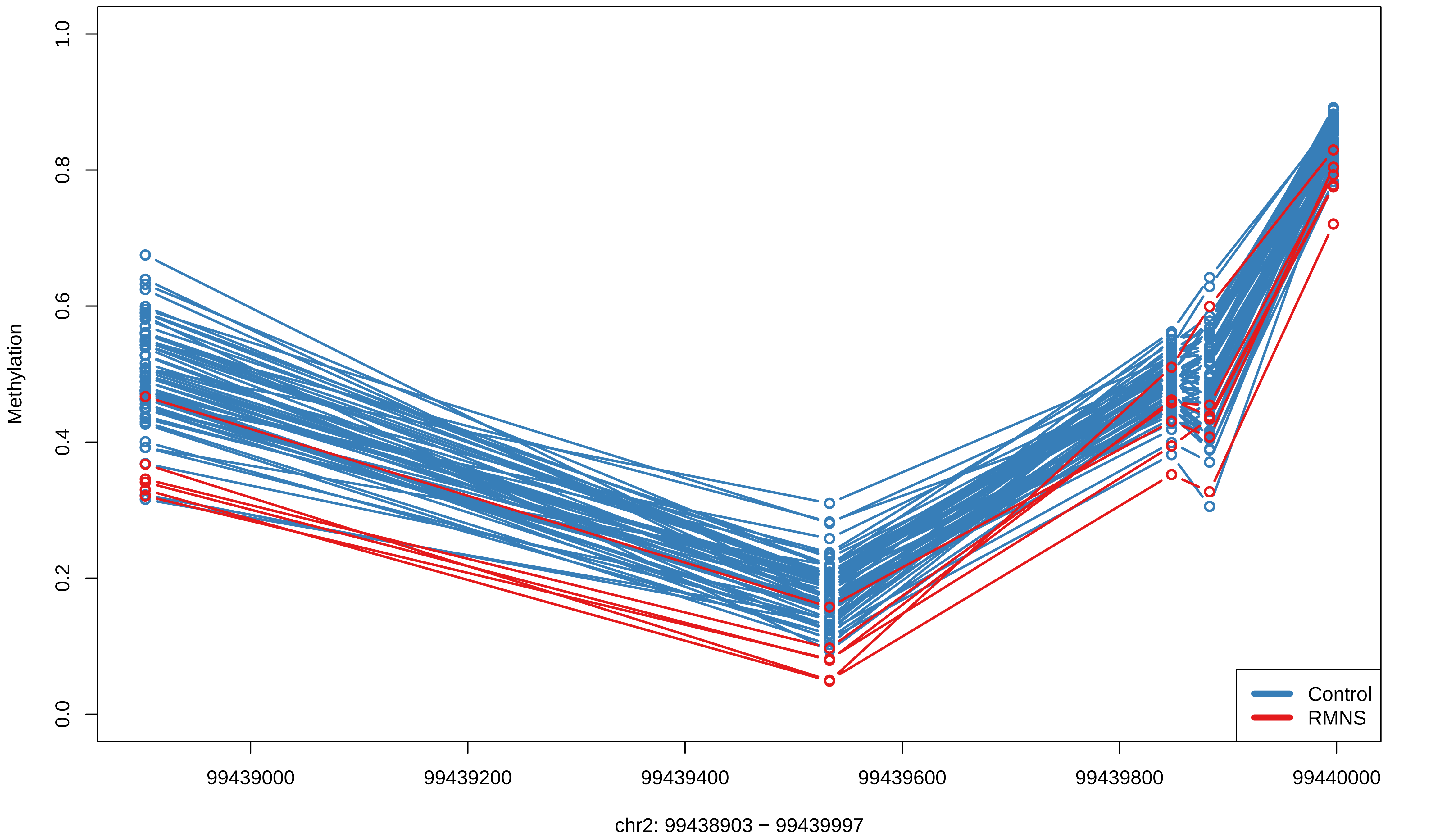

Methylation
0.0
0.2
0.4
0.6
0.8
1.0
99439000
99439200
99439400
99439600
99439800
99440000
chr2: 99438903 − 99439997
Control
RMNS

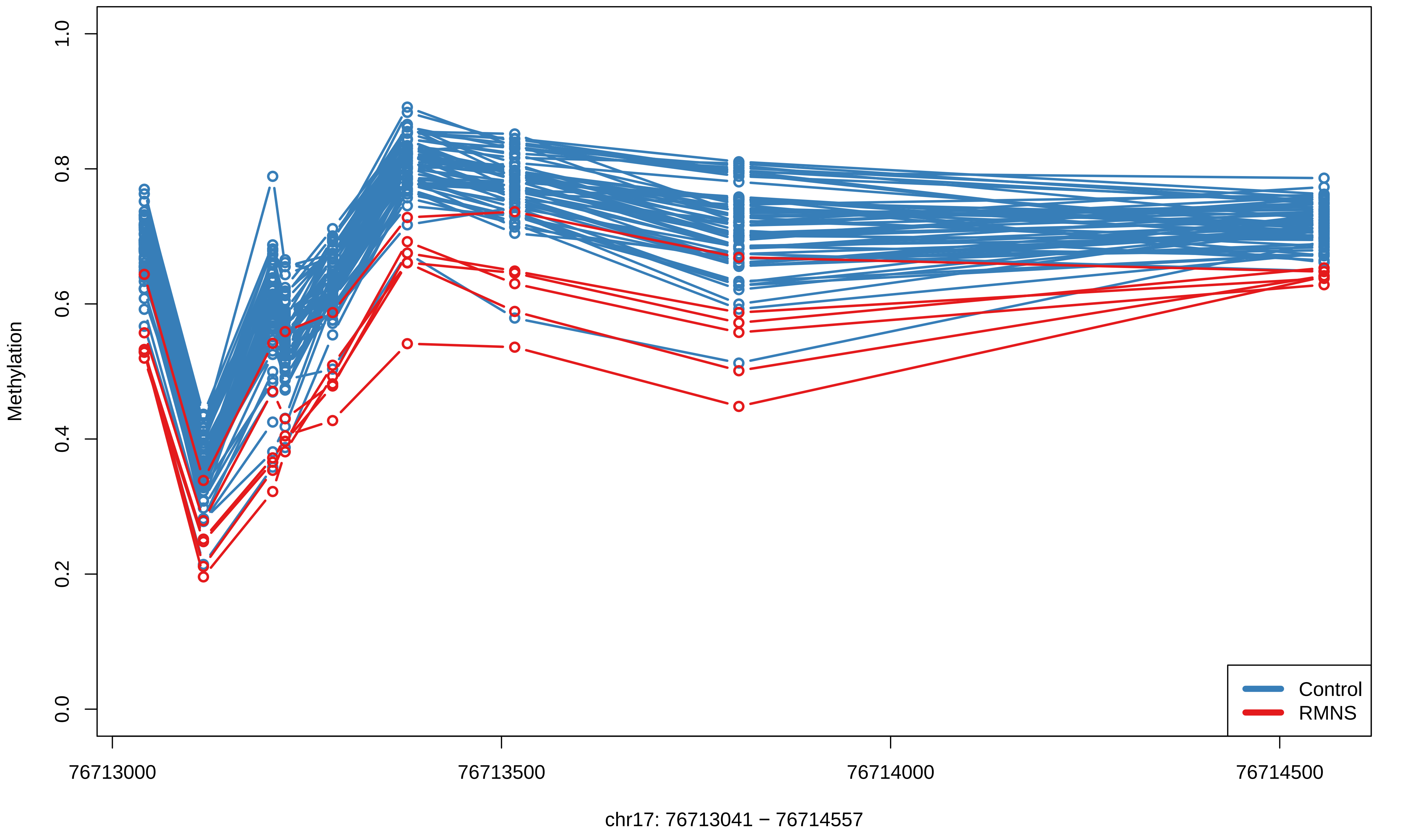

Methylation
1.0
0.8
0.6
0.4
0.2
0.0
76713000
76713500
76714000
76714500
chr17: 76713041 − 76714557
Control
RMNS

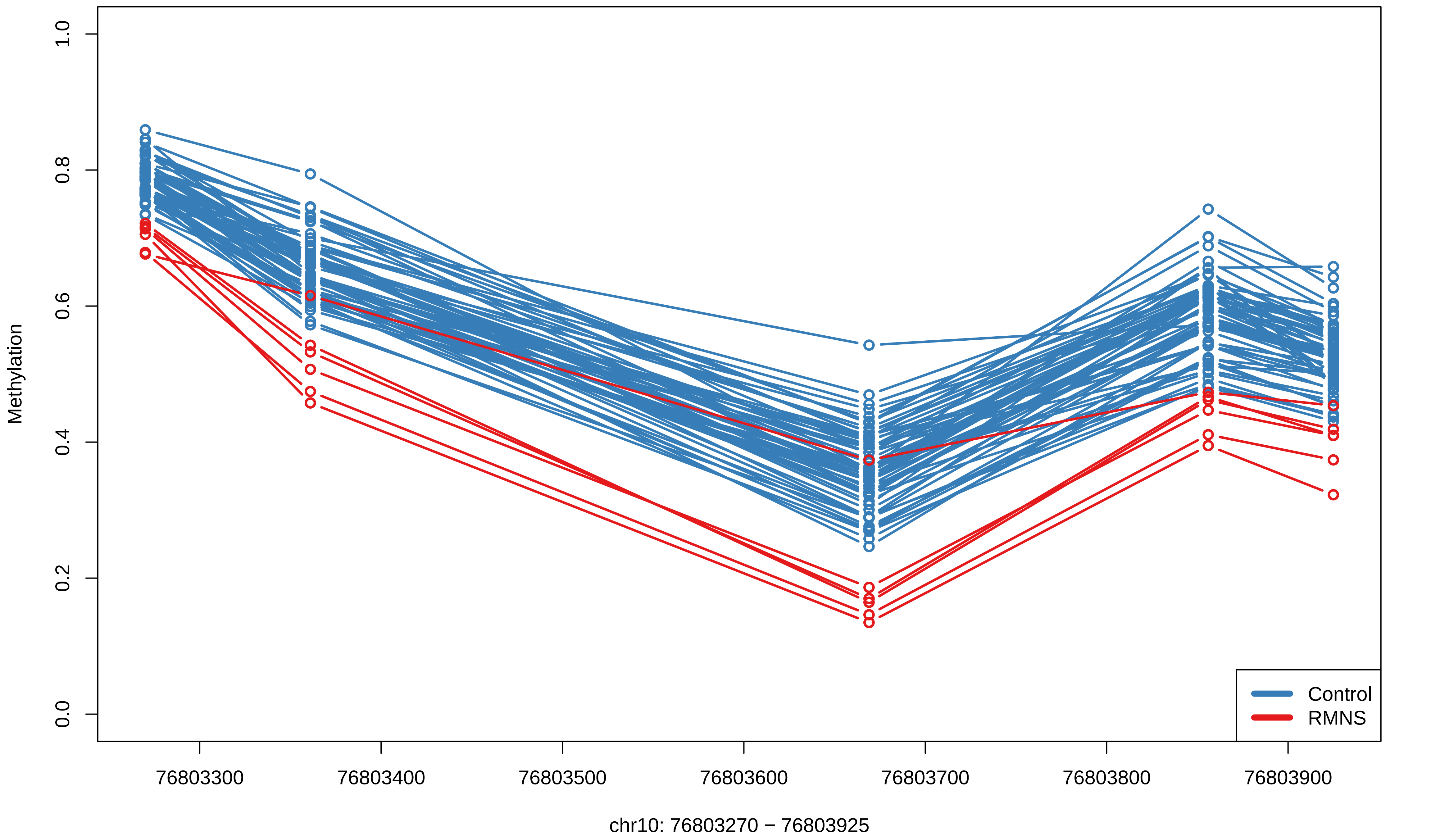

Methylation
1.0
0.8
0.6
0.4
0.2
0.0
76803300
76803400
76803500
76803600
76803700
76803800
76803900
chr10: 76803270 − 76803925
Control
RMNS

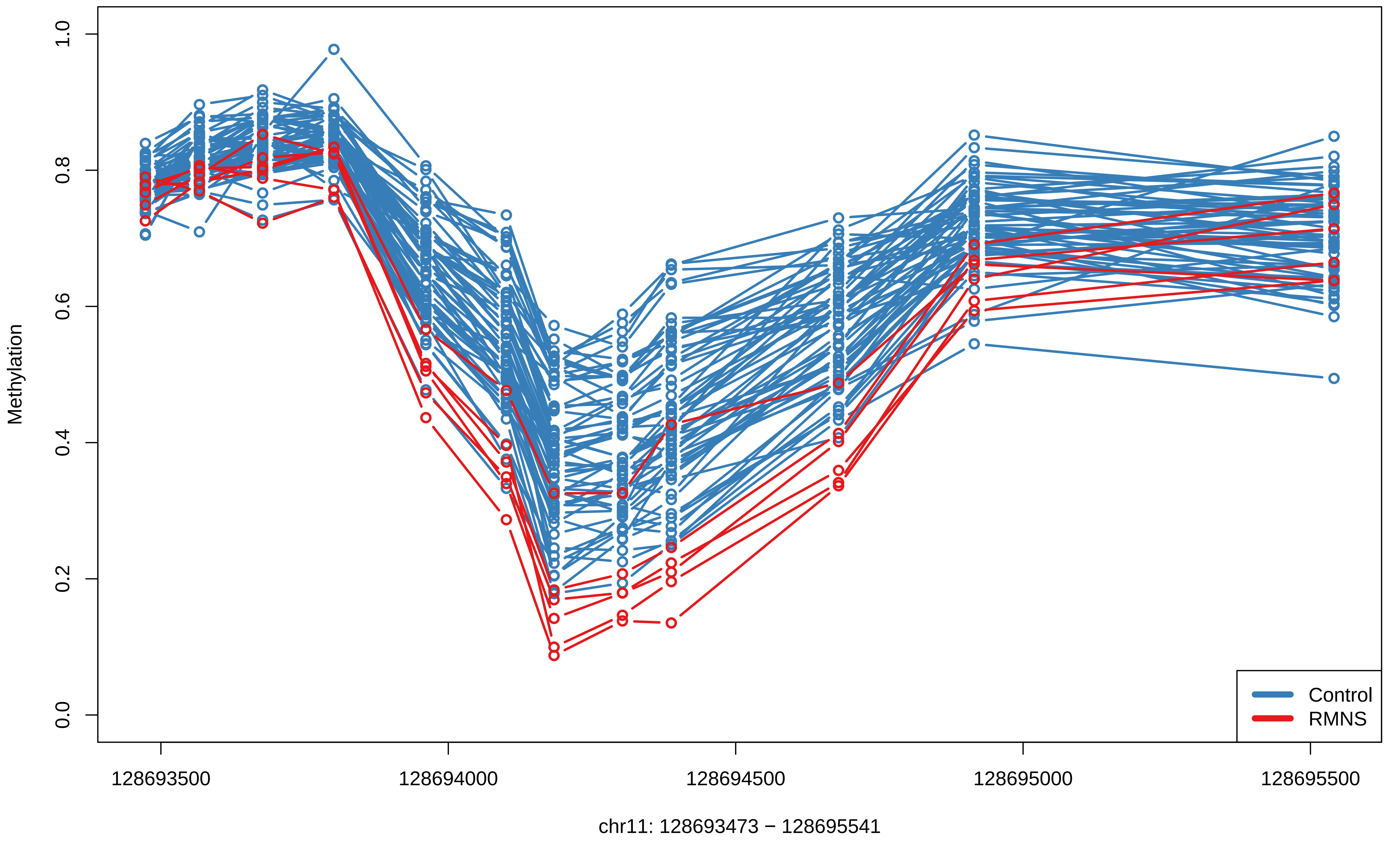

Methylation
1.0
0.8
0.6
0.4
0.2
0.0
128693500
128694000
128694500
128695000
128695500
chr11: 128693473 − 128695541
Control
RMNS

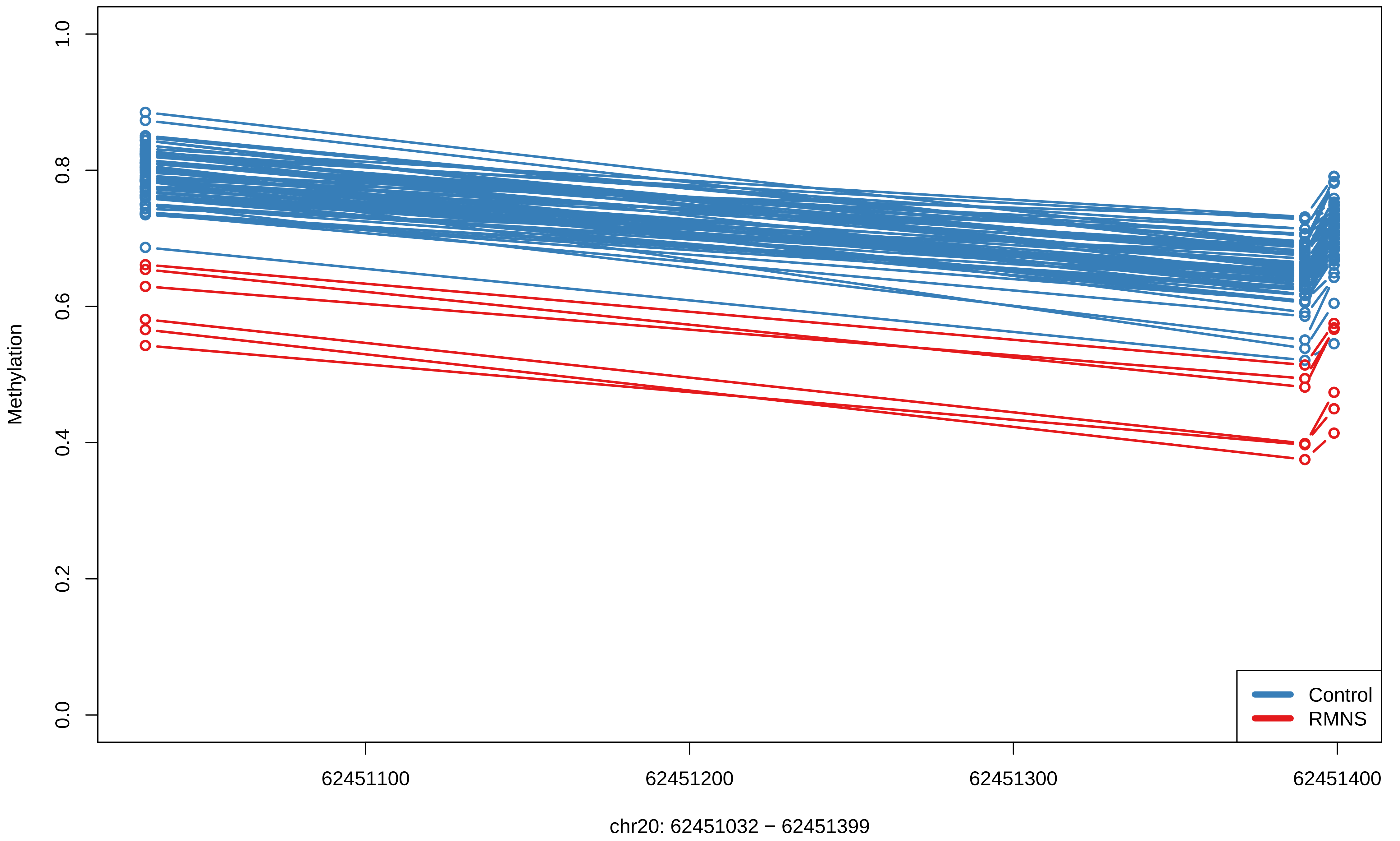

1.0
0.8
0.6
0.4
0.2
0.0
Methylation
62451100
62451200
62451300
62451400
chr20: 62451032 − 62451399
Control
RMNS

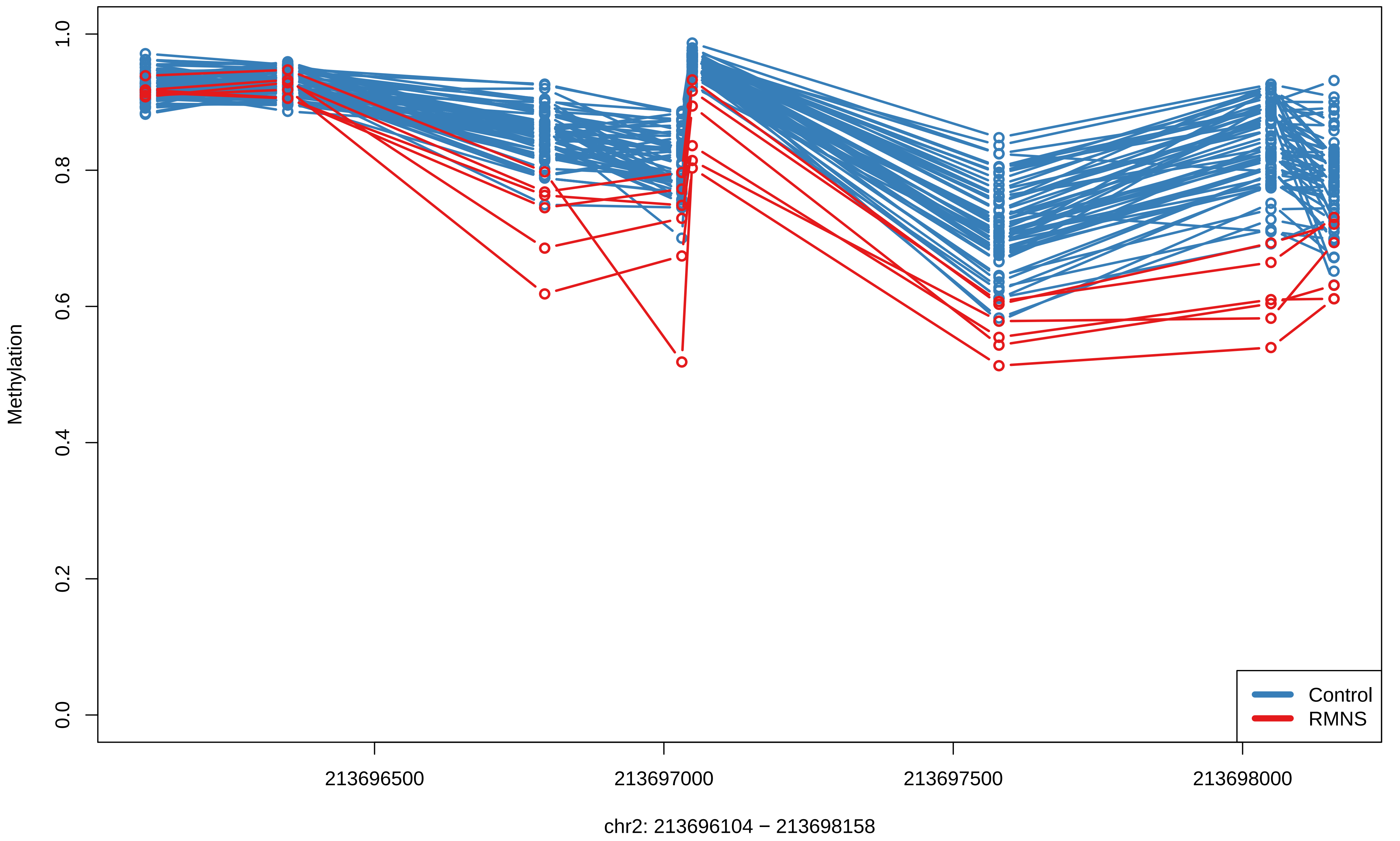

Methylation
1.0
0.8
0.6
0.4
0.2
0.0
213696500
213697000
213697500
213698000
chr2: 213696104 − 213698158
Control
RMNS

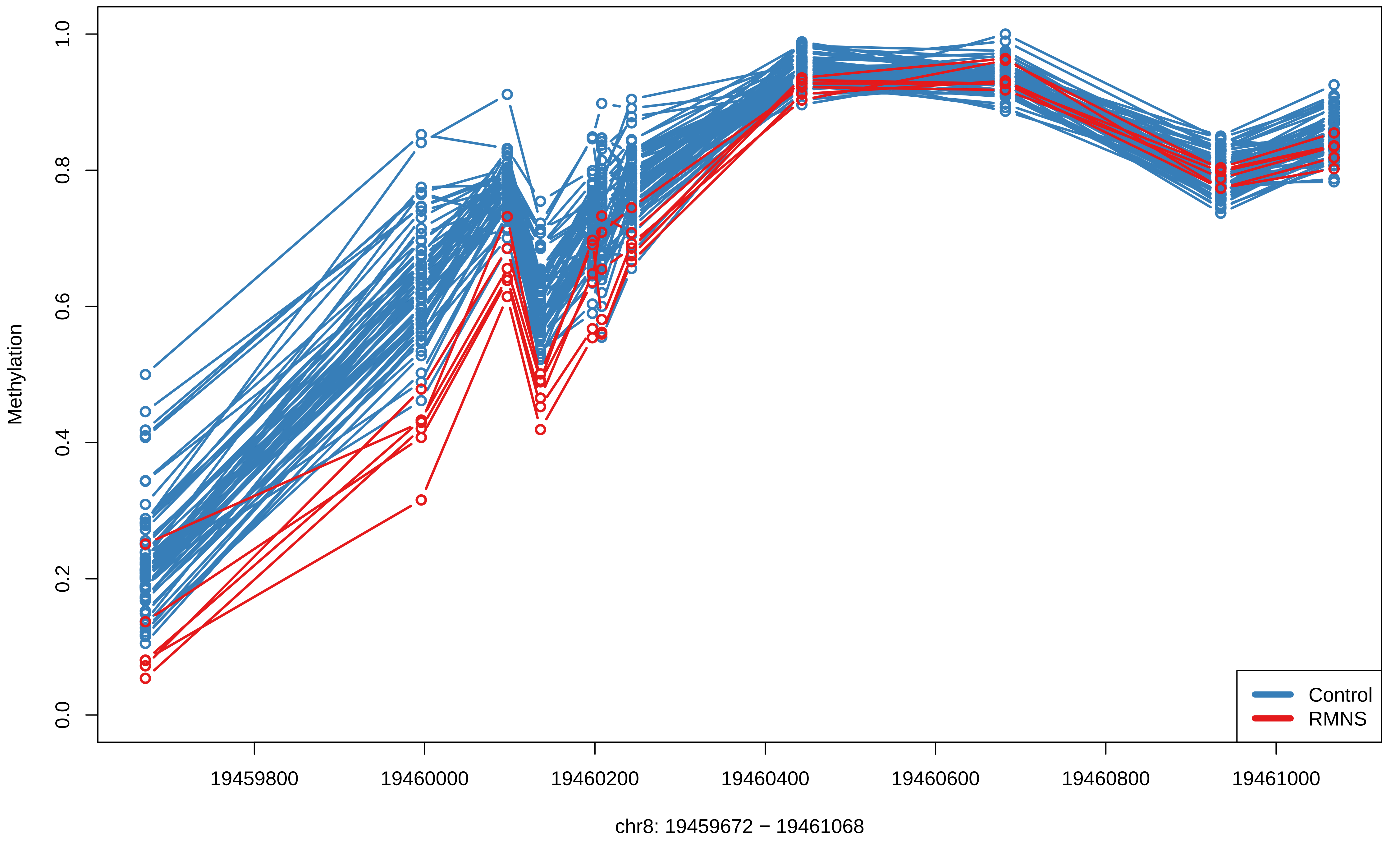
Methylation
0.0
0.2
0.4
0.6
0.8
1.0
19459800
19460000
19460200
19460400
19460600
19460800
19461000
chr8: 19459672 − 19461068
Control
RMNS

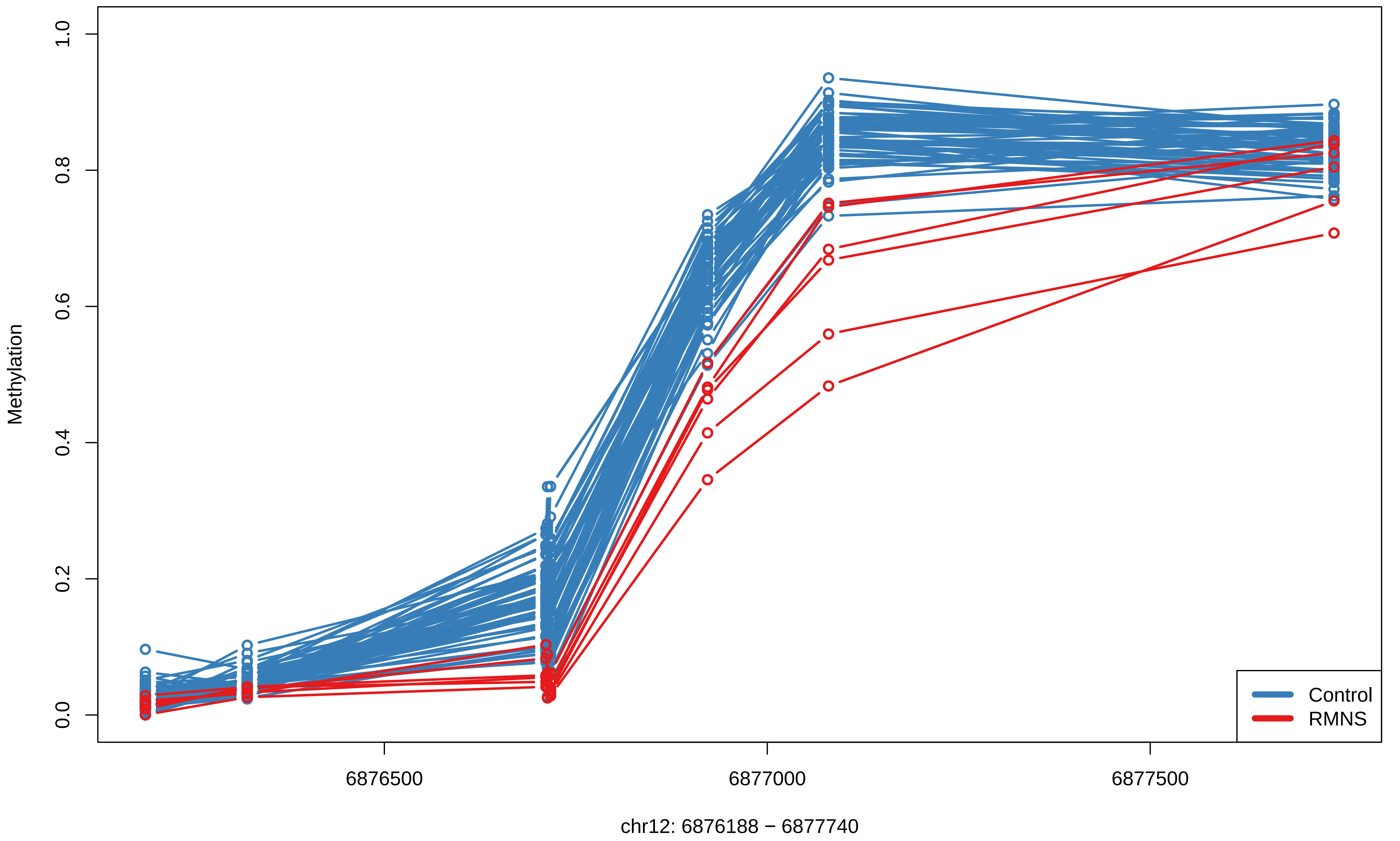

Methylation
1.0
0.8
0.6
0.4
0.2
0.0
6876500
6877000
6877500
chr12: 6876188 − 6877740
Control
RMNS

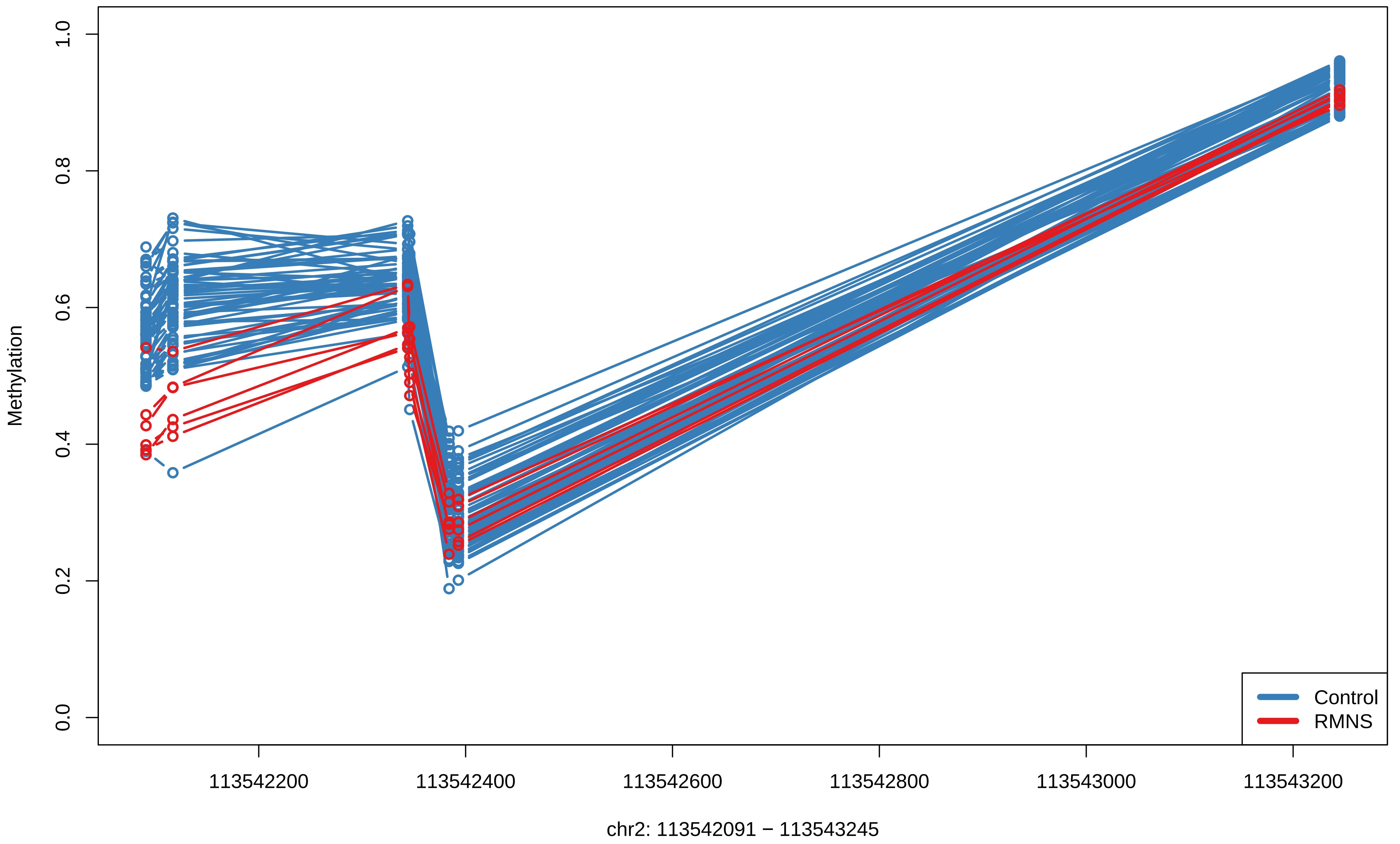

Methylation
1.0
0.8
0.6
0.4
0.2
0.0
113542200
113542400
113542600
113542800
113543000
113543200
chr2: 113542091 − 113543245
Control
RMNS

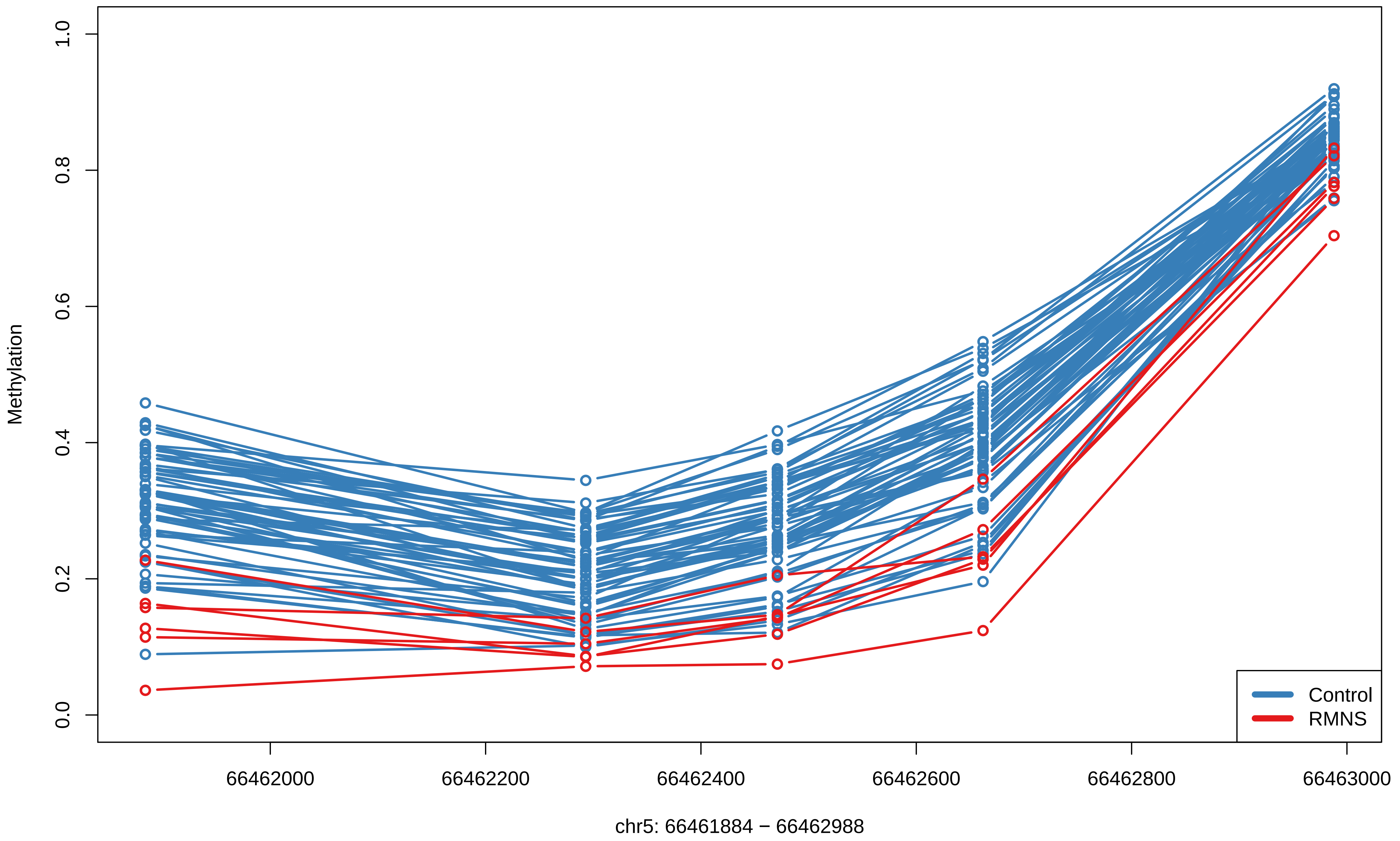

Methylation
0.0
0.2
0.4
0.6
0.8
1.0
66462000
66462200
66462400
66462600
66462800
66463000
chr5: 66461884 − 66462988
Control
RMNS

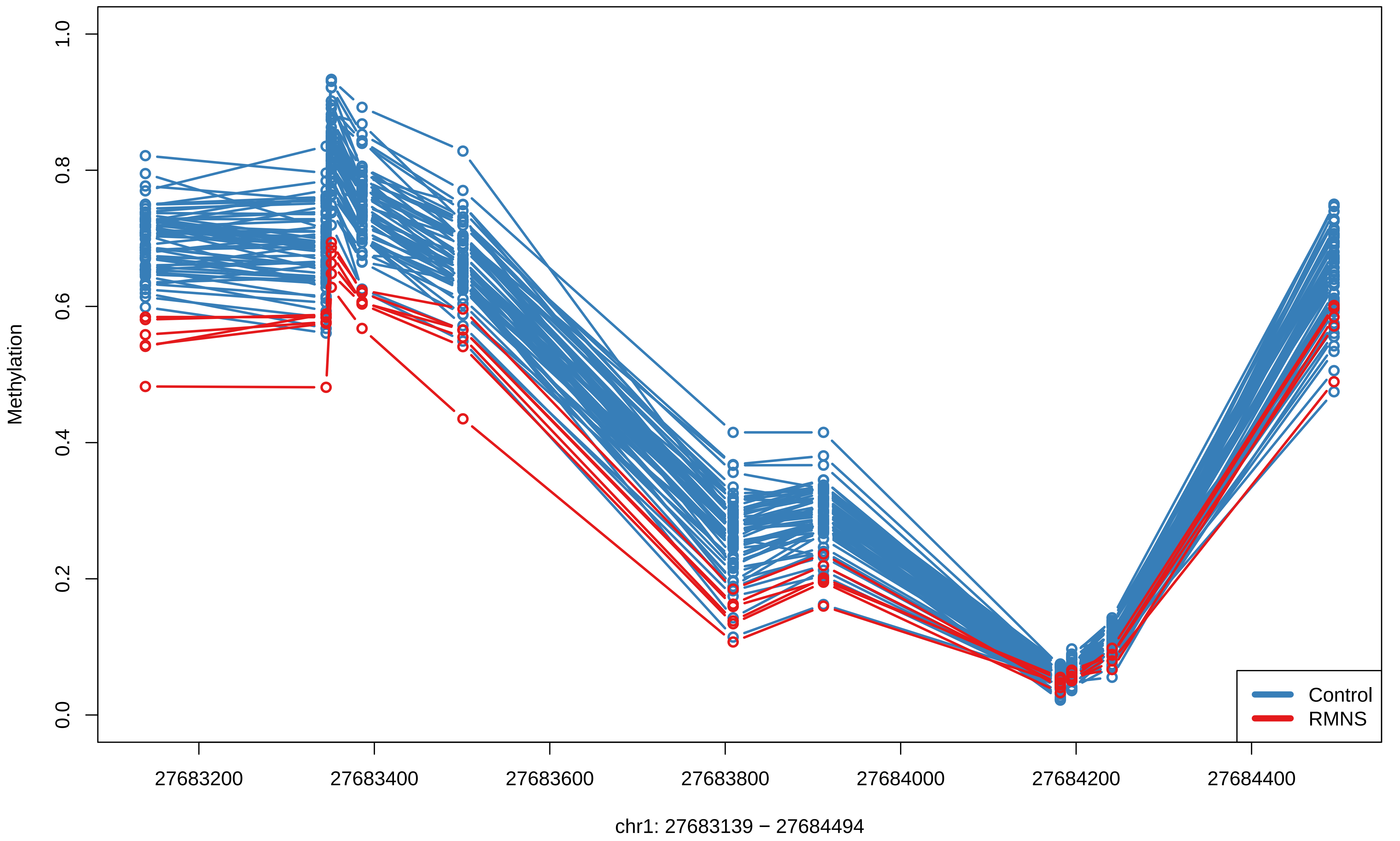

Methylation
1.0
0.8
0.6
0.4
0.2
0.0
27683200
27683400
27683600
27683800
27684000
27684200
27684400
chr1: 27683139 − 27684494
Control
RMNS

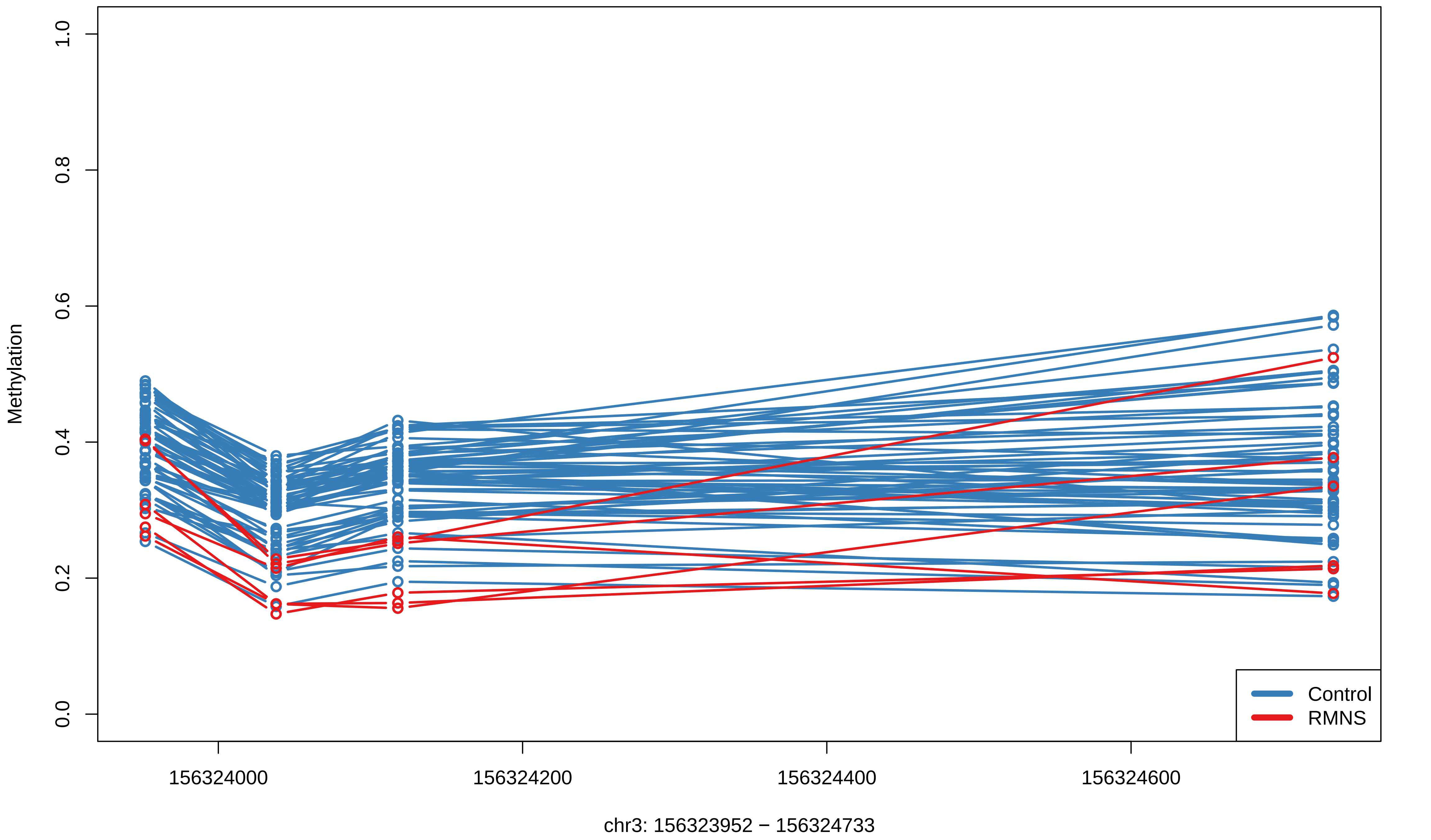

Methylation
0.0
0.2
0.4
0.6
0.8
1.0
156324000
156324200
156324400
156324600
chr3: 156323952 − 156324733
Control
RMNS

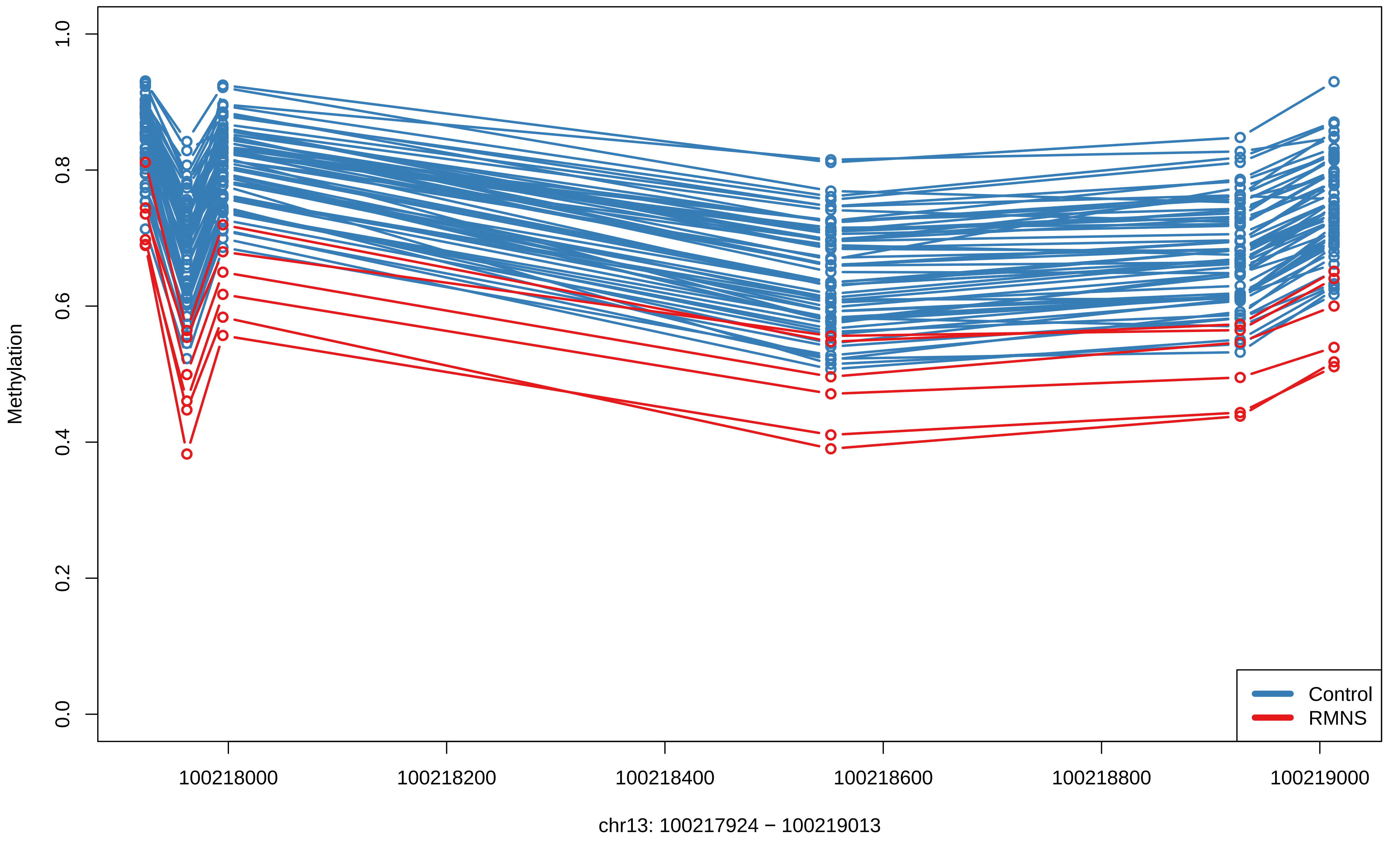

Methylation
1.0
0.8
0.6
0.4
0.2
0.0
100218000
100218200
100218400
100218600
100218800
100219000
chr13: 100217924 − 100219013
Control
RMNS

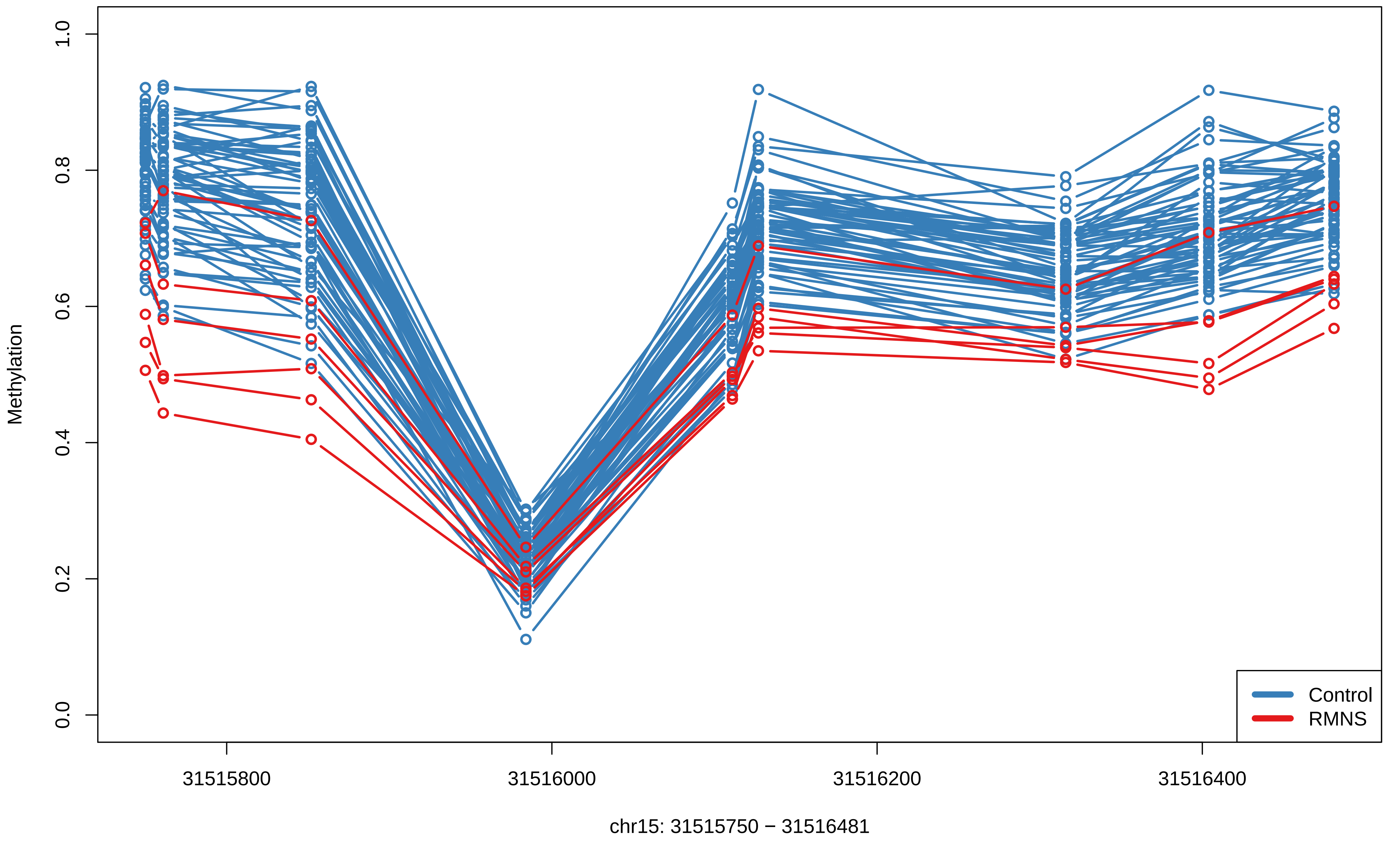

Methylation
0.0
0.2
0.4
0.6
0.8
1.0
31515800
31516000
31516200
31516400
chr15: 31515750 − 31516481
Control
RMNS

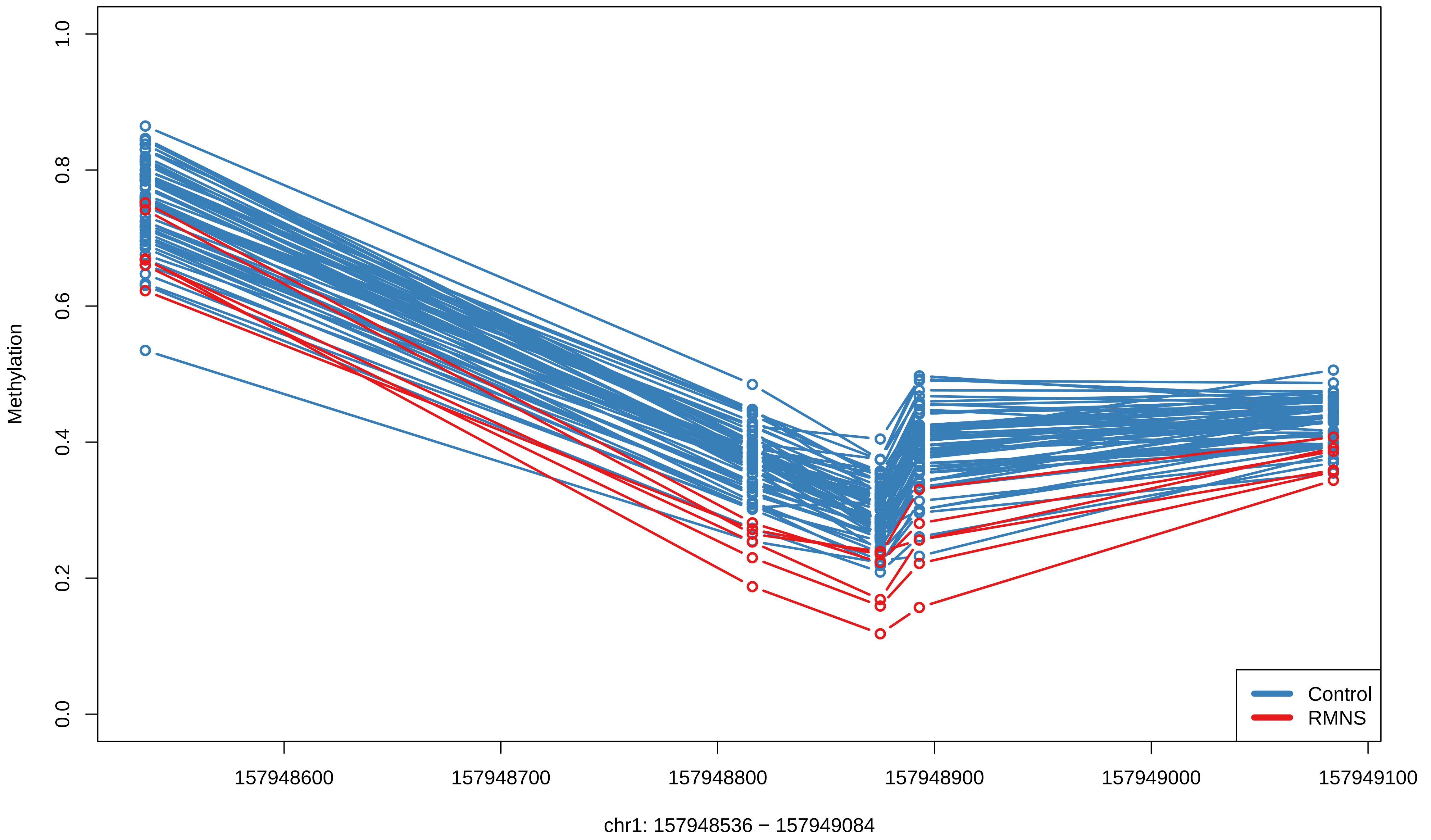

Methylation
0.0
0.2
0.4
0.6
0.8
1.0
157948600
157948700
157948800
157948900
157949000
157949100
chr1: 157948536 − 157949084
Control
RMNS

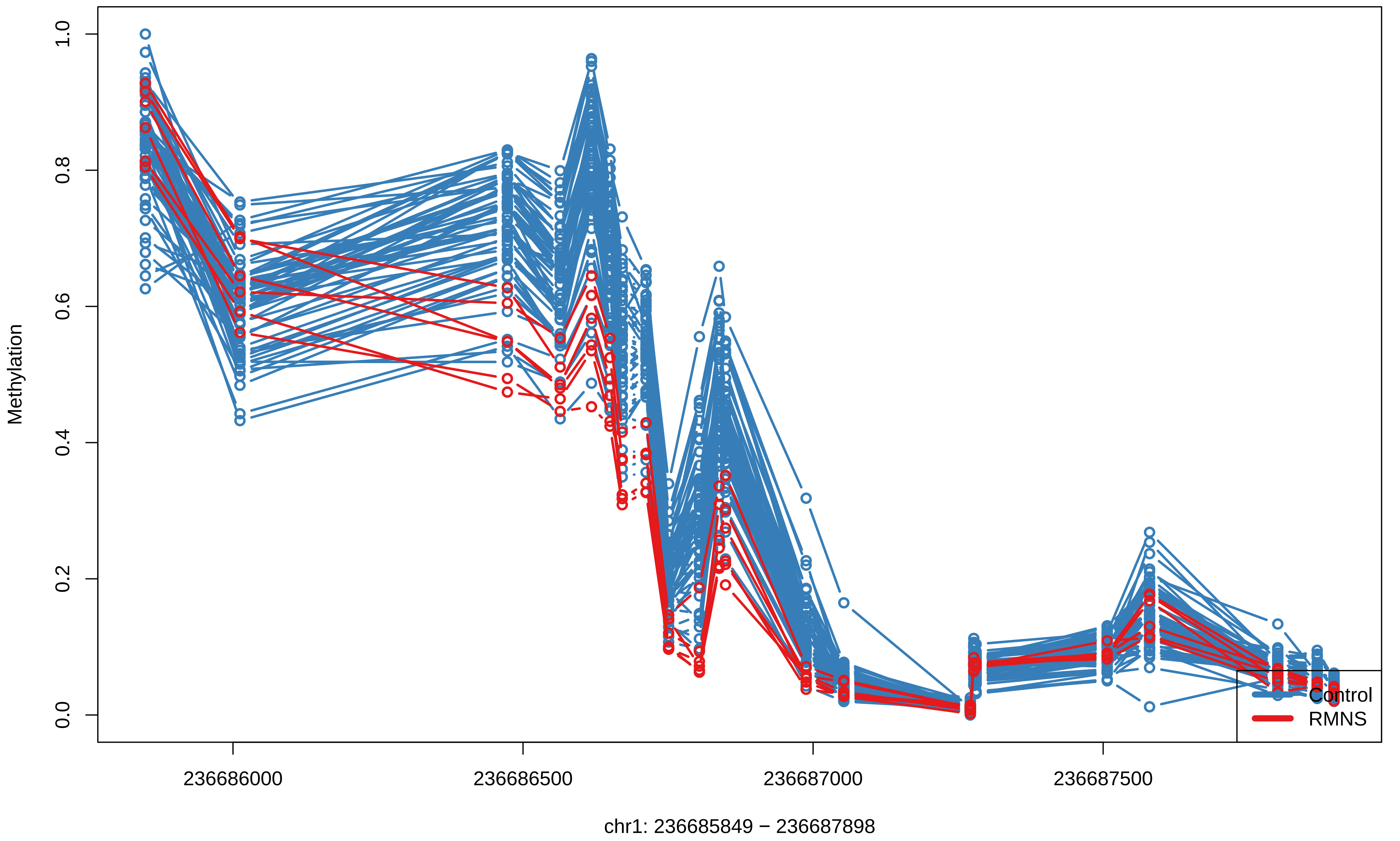

Methylation
1.0
0.8
0.6
0.4
0.2
0.0
236686000
236686500
236687000
236687500
chr1: 236685849 − 236687898
Control
RMNS

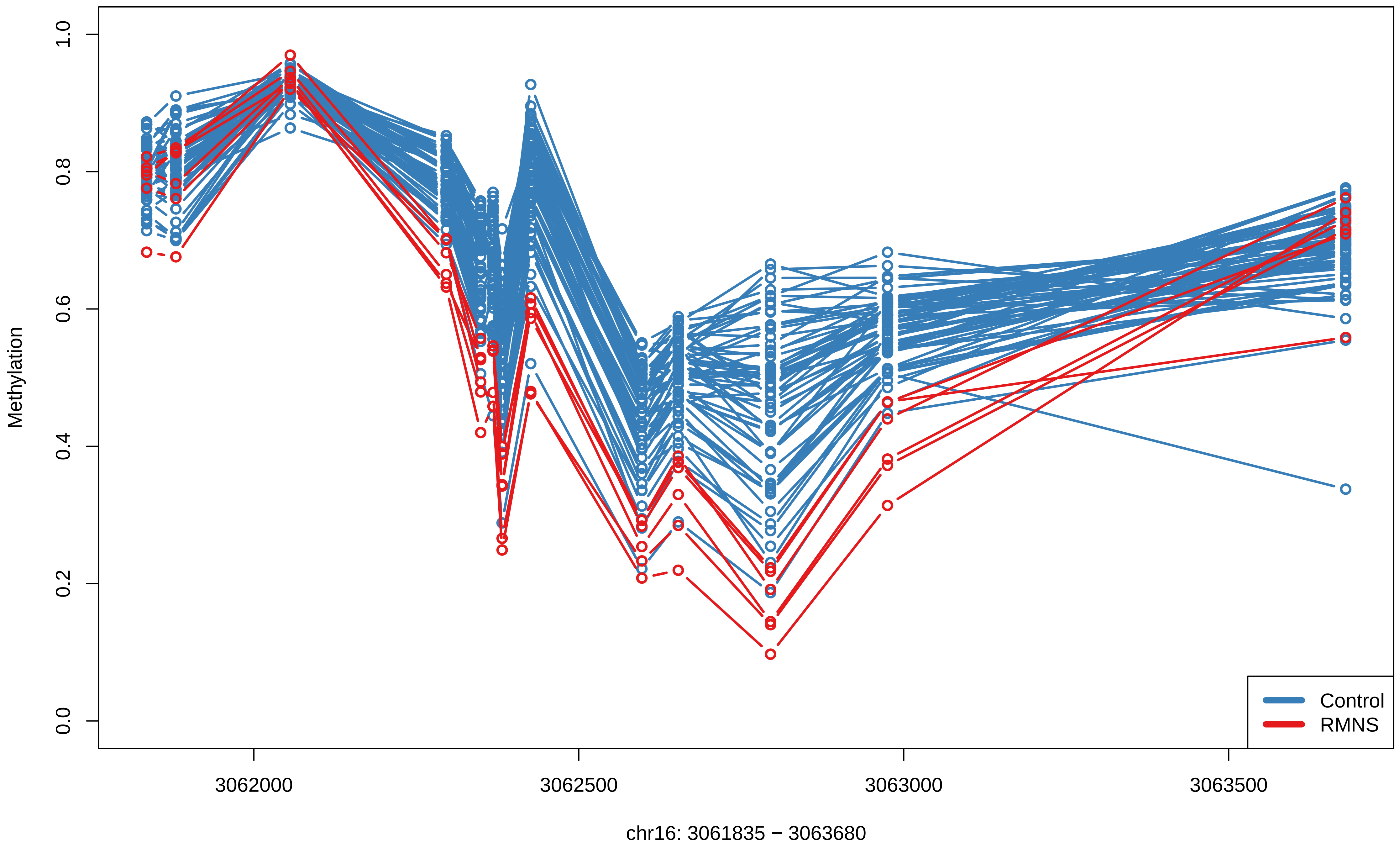

Methylation
0.0
0.2
0.4
0.6
0.8
1.0
3062000
3062500
3063000
3063500
chr16: 3061835 − 3063680
Control
RMNS

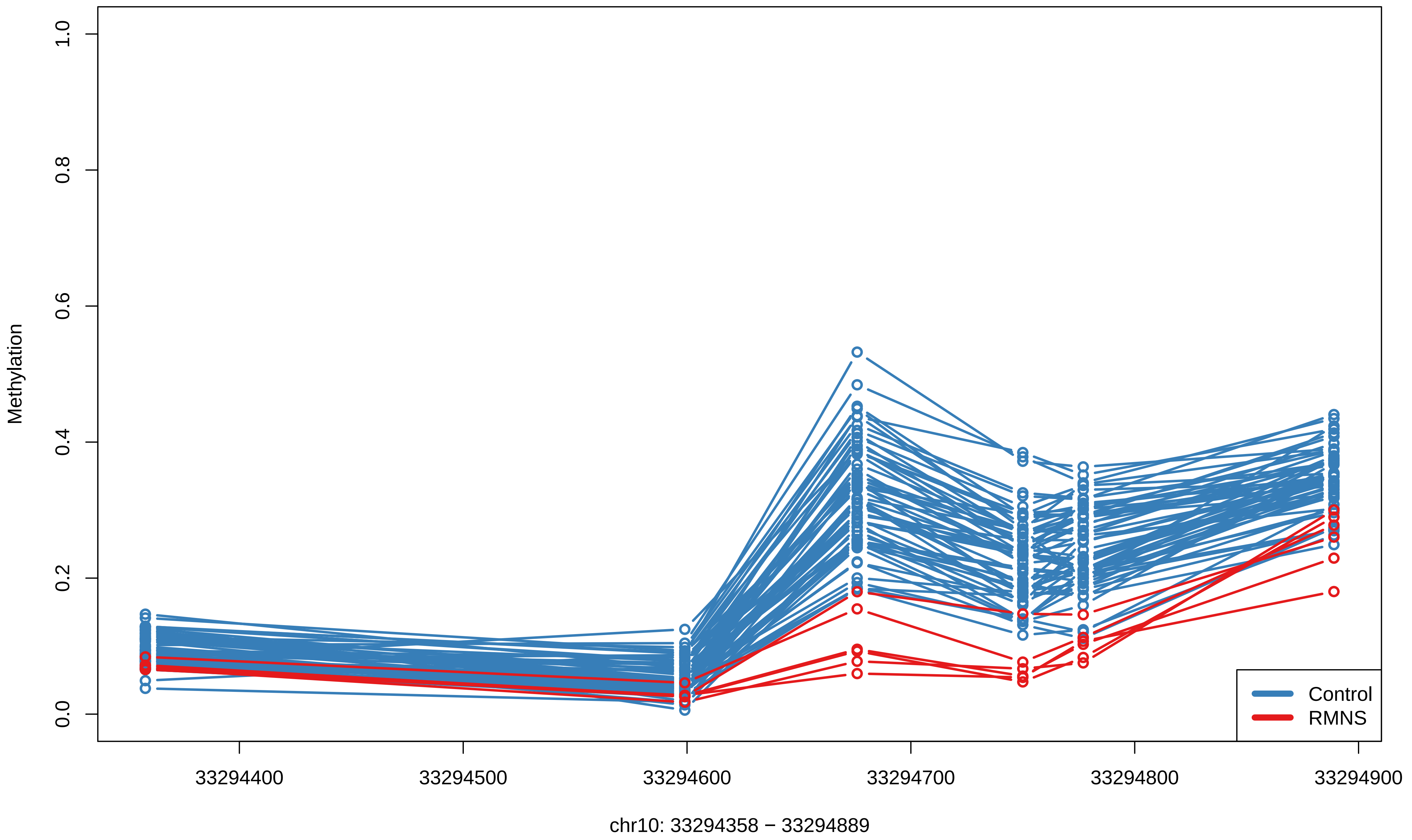
Methylation
0.0
0.2
0.4
0.6
0.8
1.0
33294400
33294500
33294600
33294700
33294800
33294900
chr10: 33294358 − 33294889
Control
RMNS

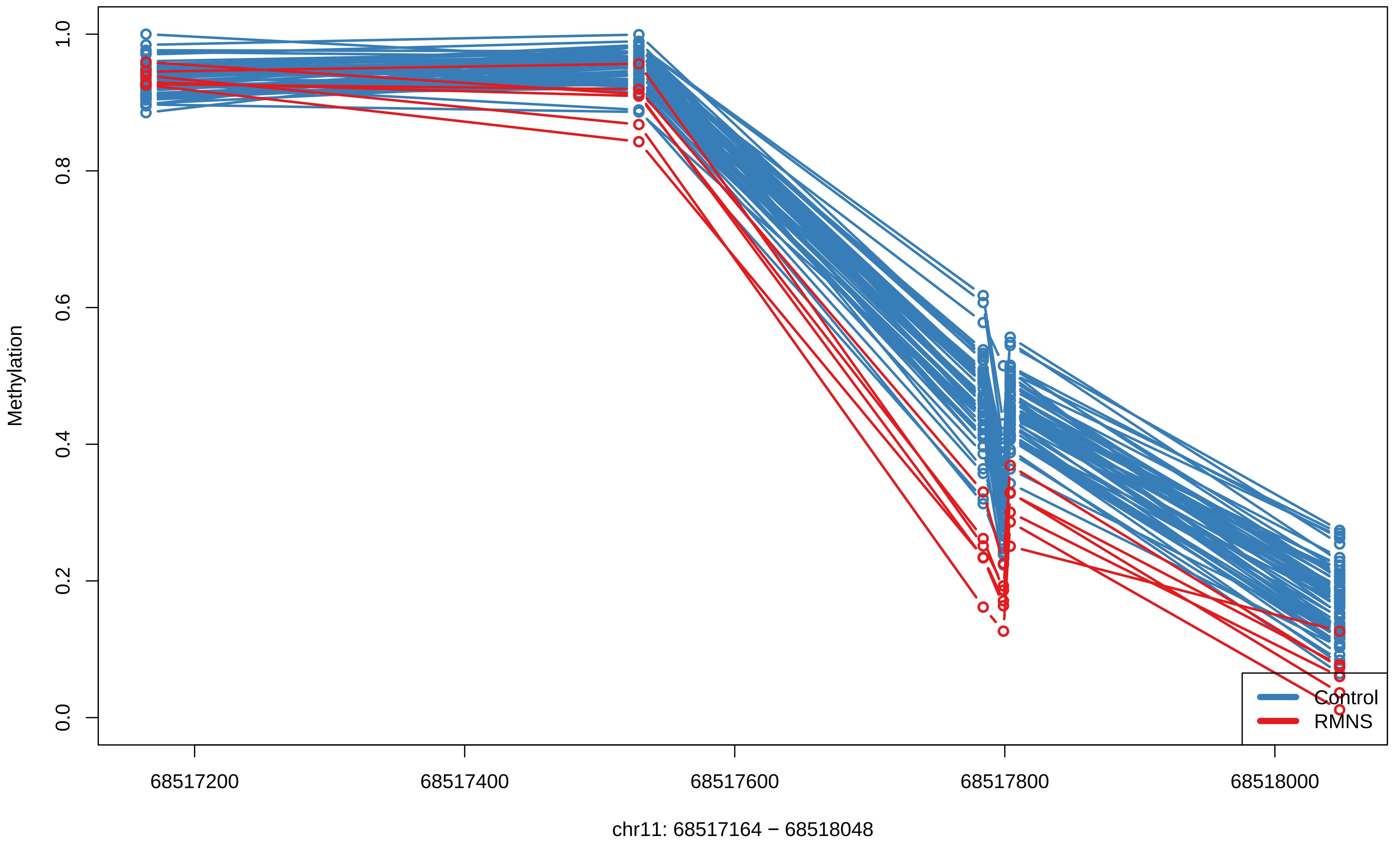

Methylation
1.0
0.8
0.6
0.4
0.2
0.0
68517200
68517400
68517600
68517800
68518000
chr11: 68517164 − 68518048
Control
RMNS

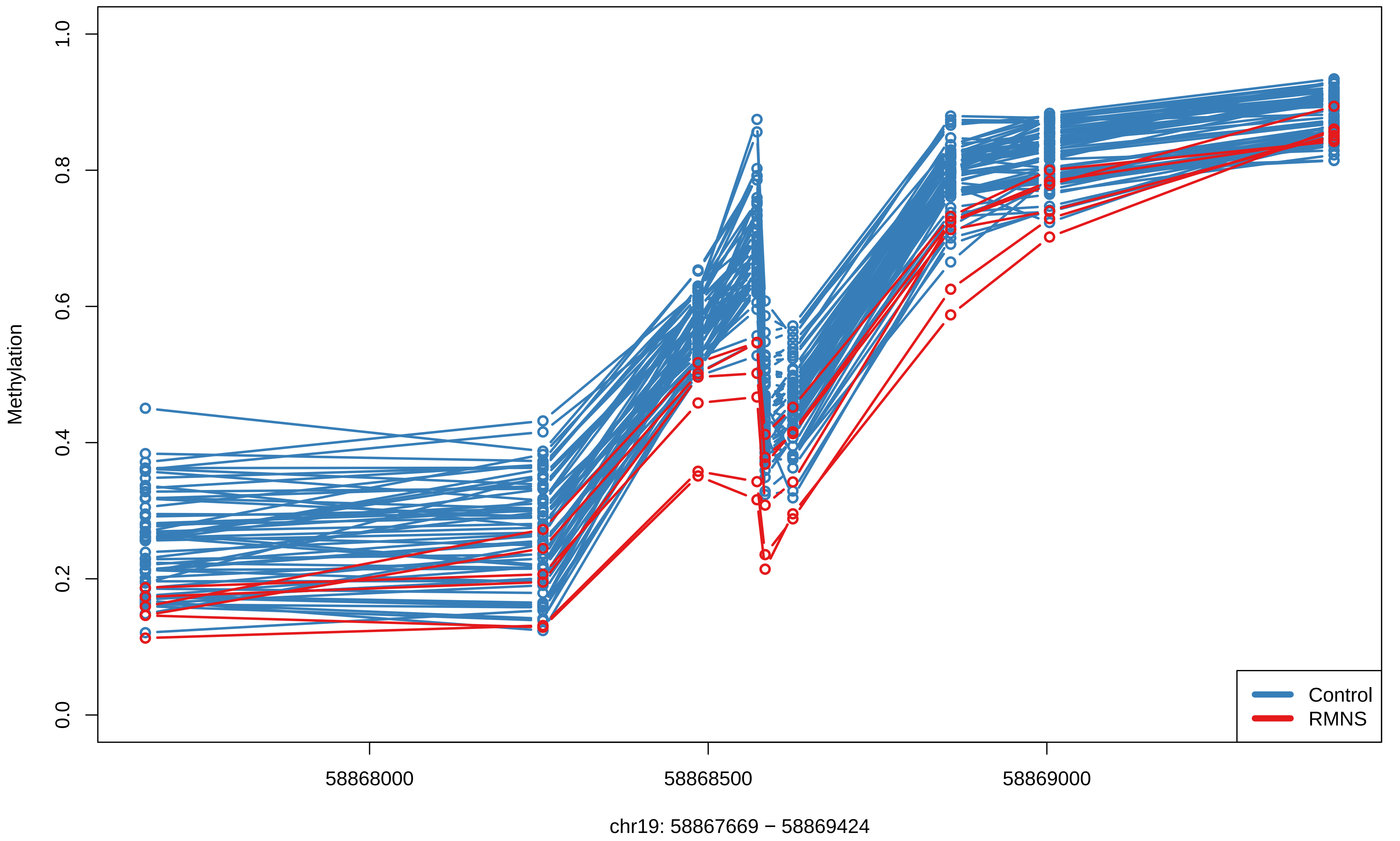

Methylation
0.0
0.2
0.4
0.6
0.8
1.0
58868000
58868500
58869000
chr19: 58867669 − 58869424
Control
RMNS

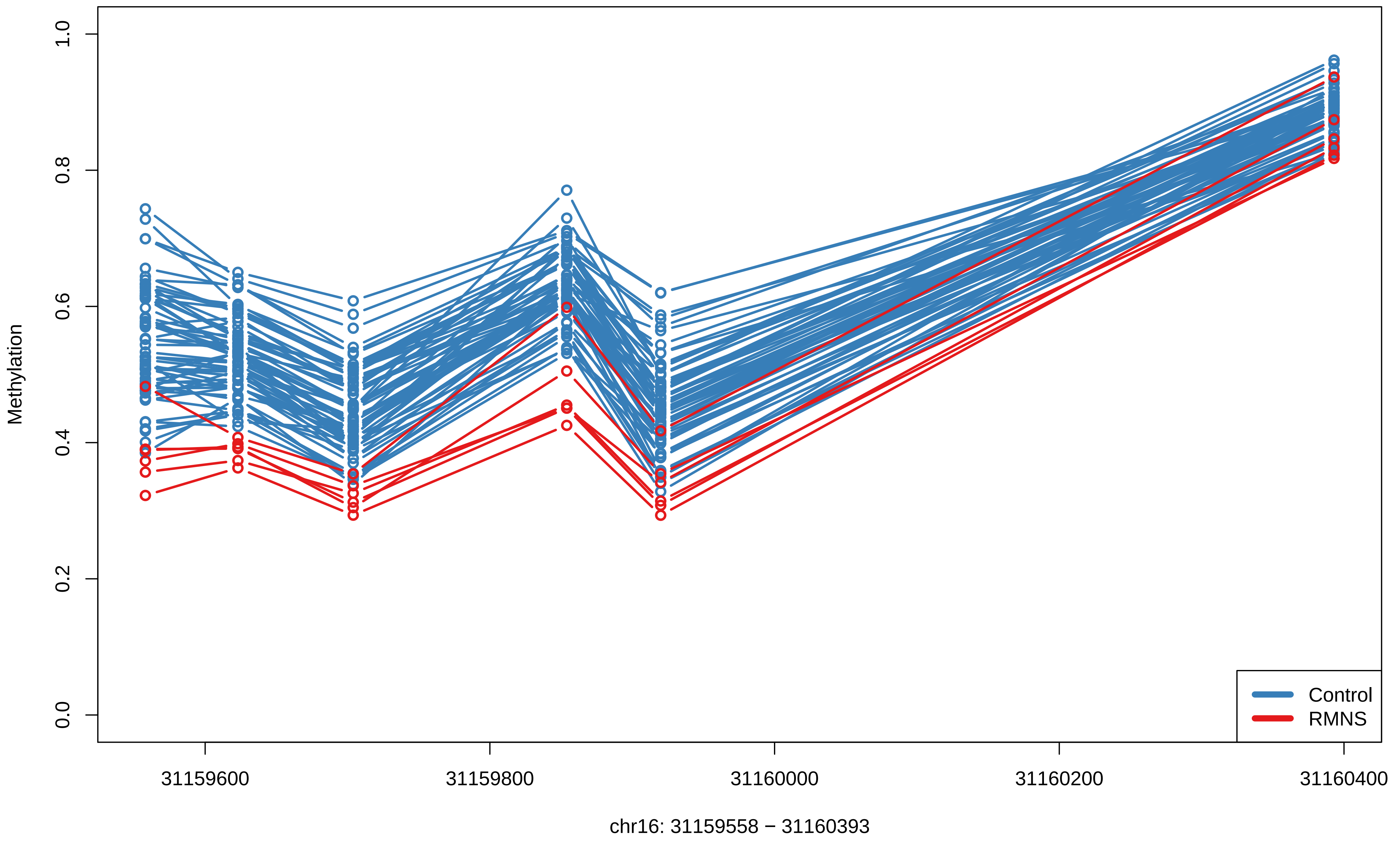

Methylation
0.0
0.2
0.4
0.6
0.8
1.0
31159600
31159800
31160000
31160200
31160400
chr16: 31159558 − 31160393
Control
RMNS

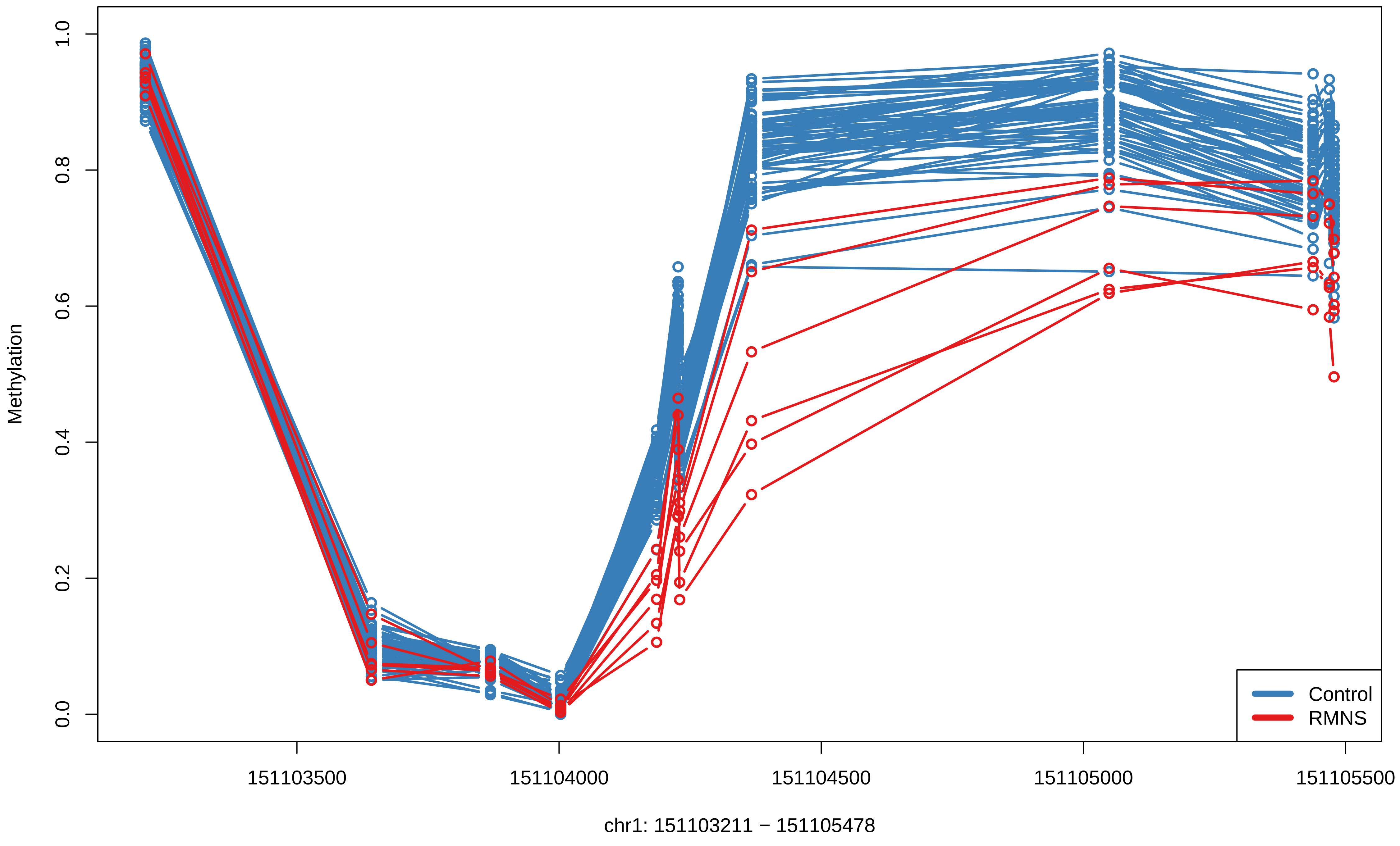

Methylation
0.0
0.2
0.4
0.6
0.8
1.0
151103500
151104000
151104500
151105000
151105500
chr1: 151103211 − 151105478
Control
RMNS

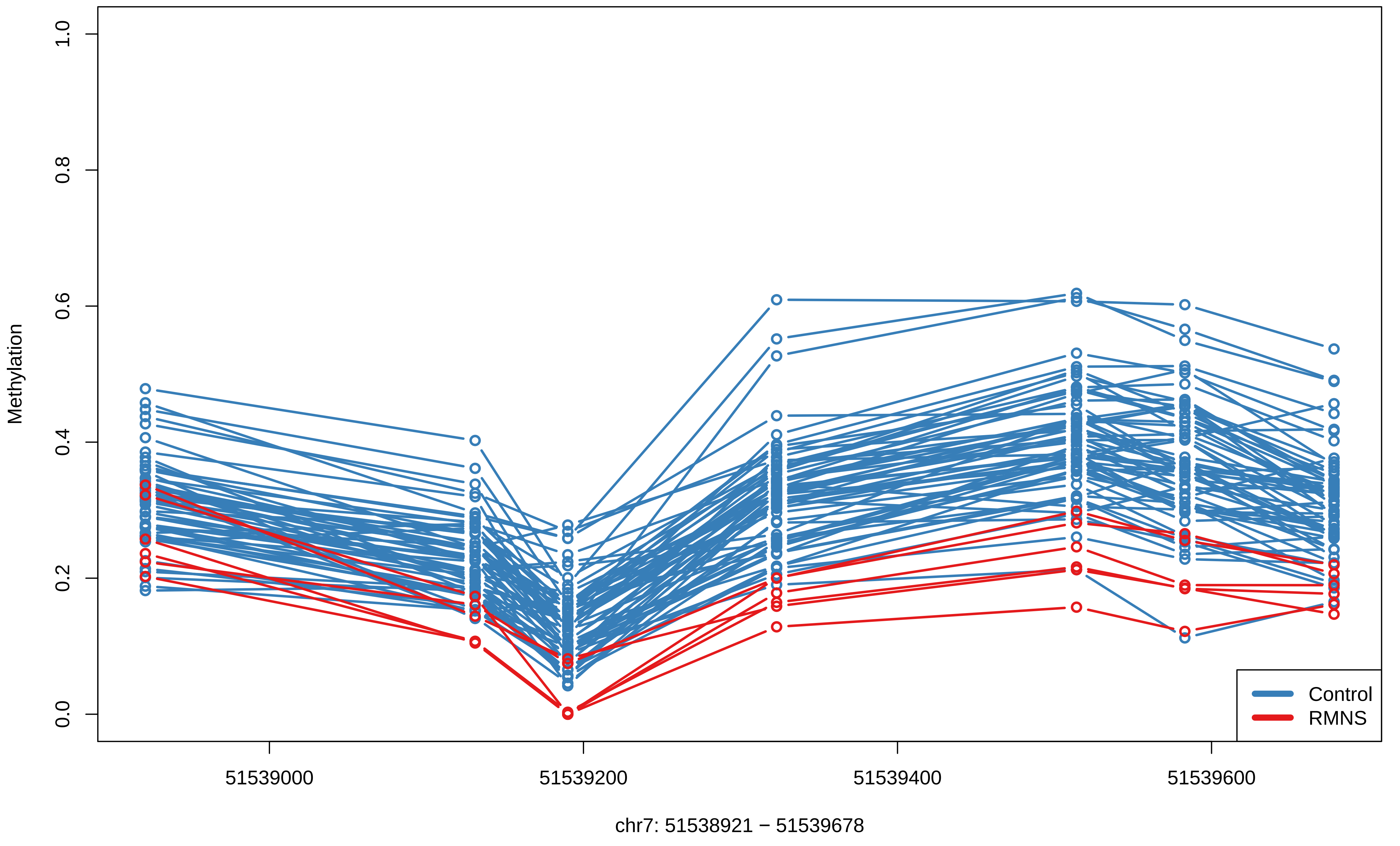

Methylation
0.0
0.2
0.4
0.6
0.8
1.0
51539000
51539200
51539400
51539600
chr7: 51538921 − 51539678
Control
RMNS

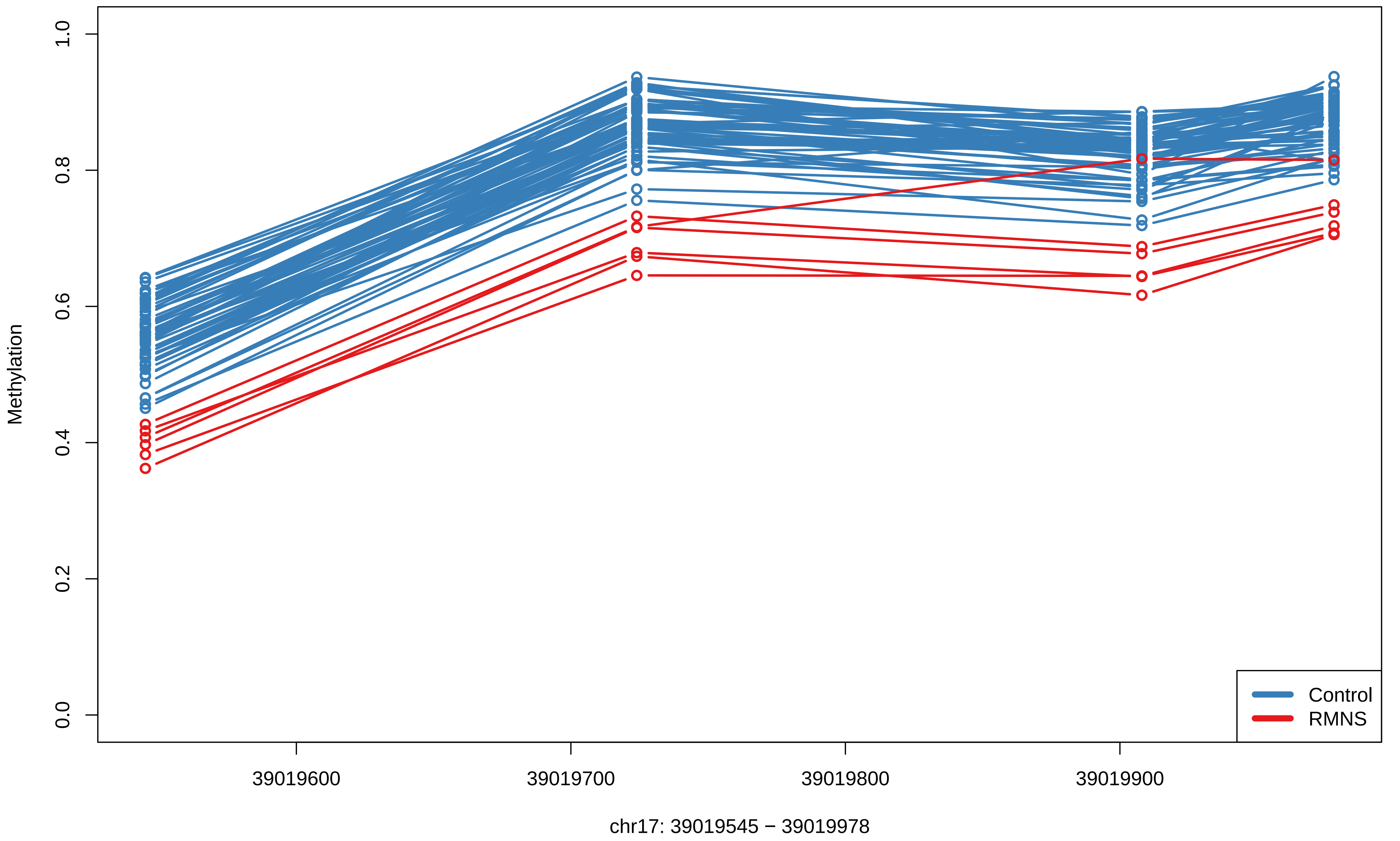
Methylation
1.0
0.8
0.6
0.4
0.2
0.0
39019600
39019700
39019800
39019900
chr17: 39019545 − 39019978
Control
RMNS

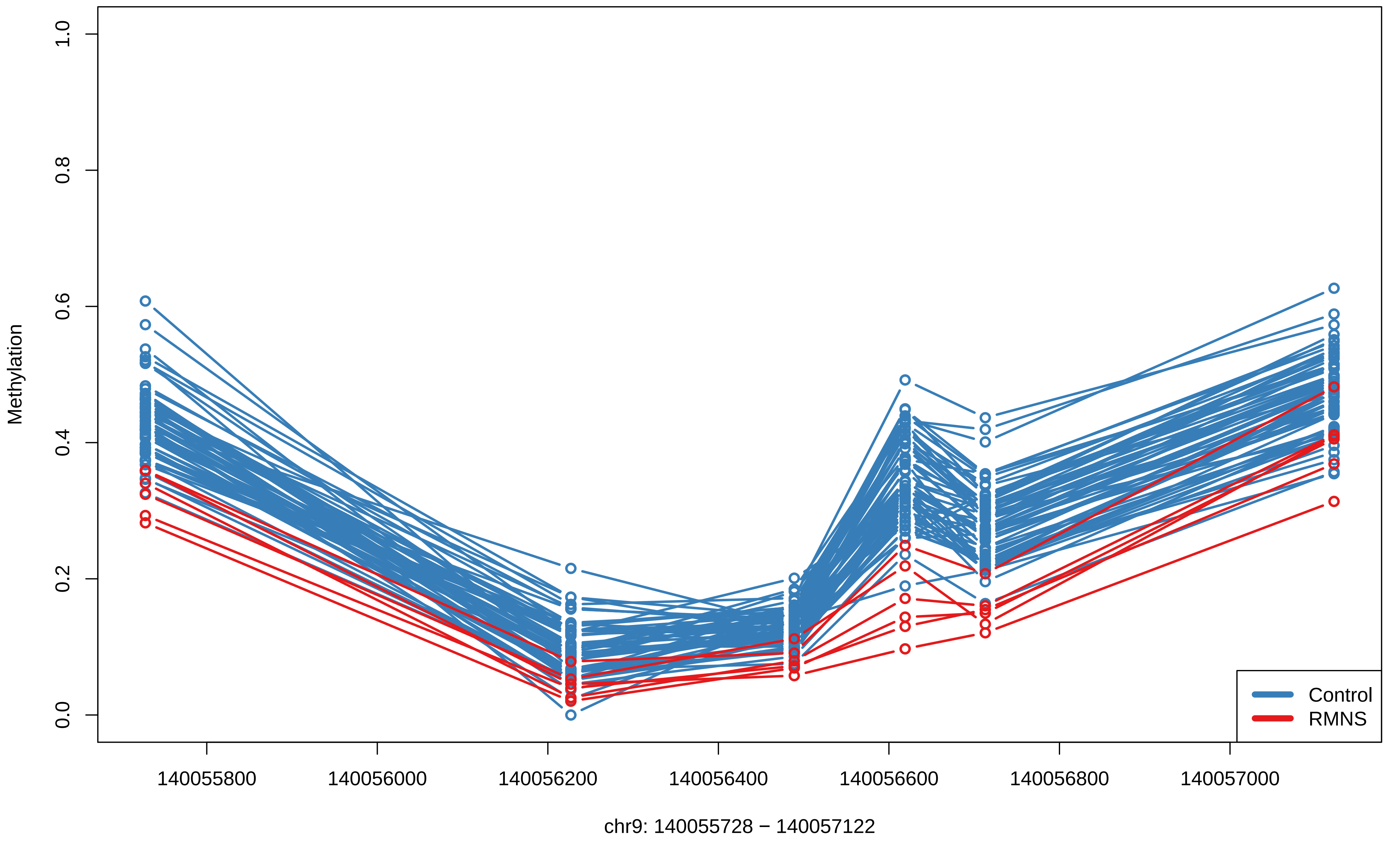
Methylation
1.0
0.8
0.6
0.4
0.2
0.0
140055800
140056000
140056200
140056400
140056600
140056800
140057000
chr9: 140055728 − 140057122
Control
RMNS

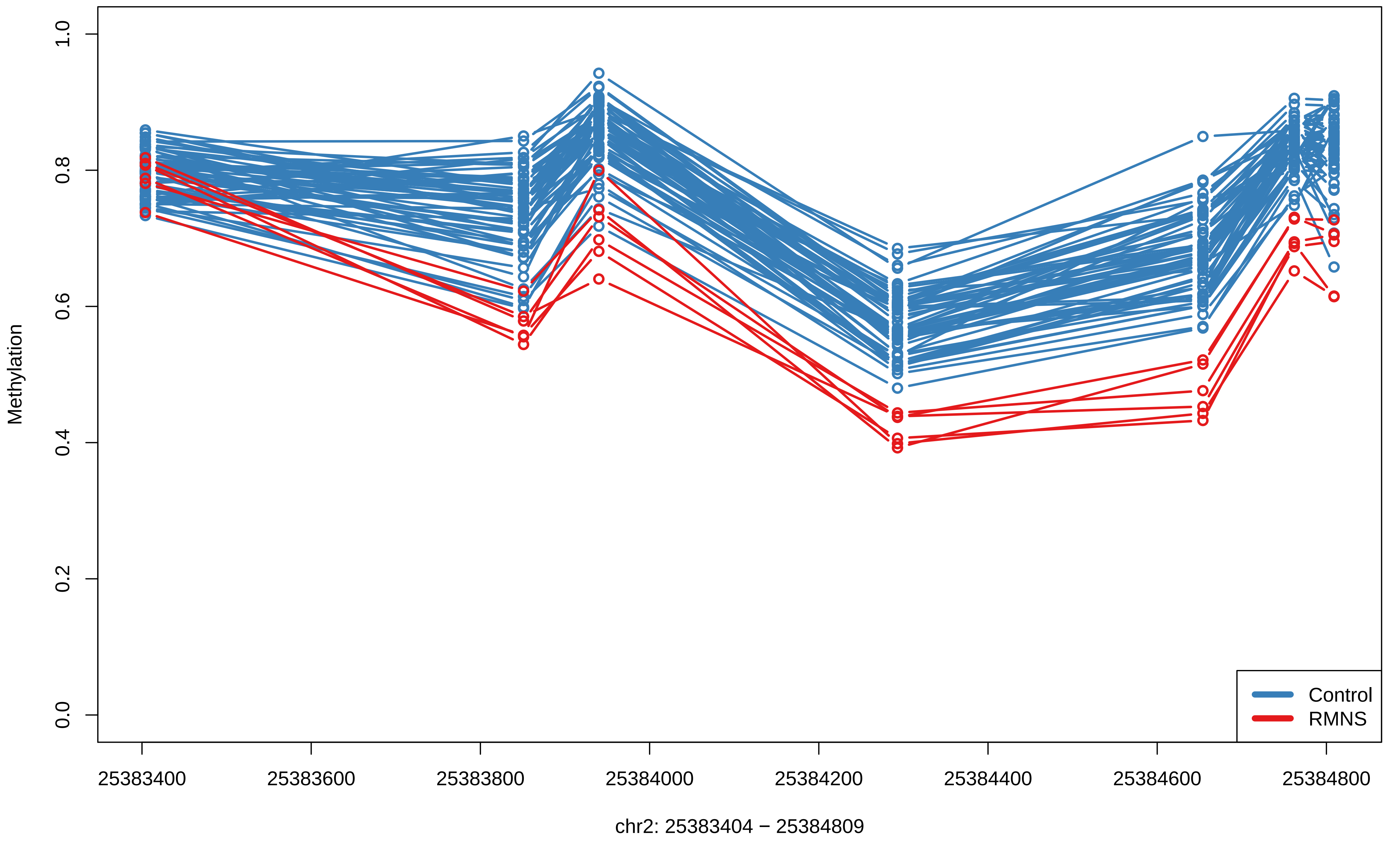

1.0
0.8
0.6
0.4
0.2
0.0
Methylation
25383400
25383600
25383800
25384000
25384200
25384400
25384600
25384800
chr2: 25383404 − 25384809
Control
RMNS

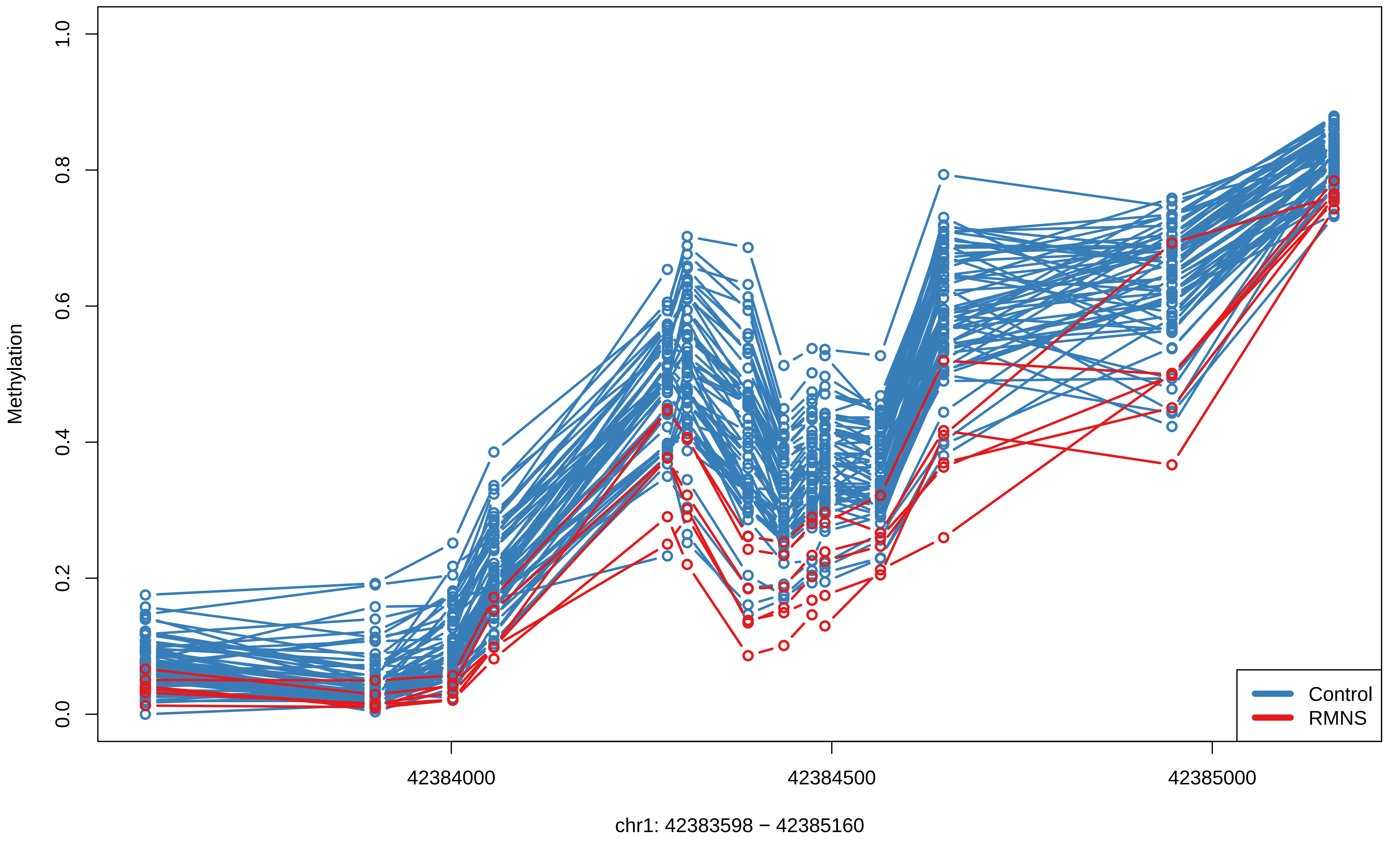

Methylation
0.0
0.2
0.4
0.6
0.8
1.0
42384000
42384500
42385000
chr1: 42383598 − 42385160
Control
RMNS

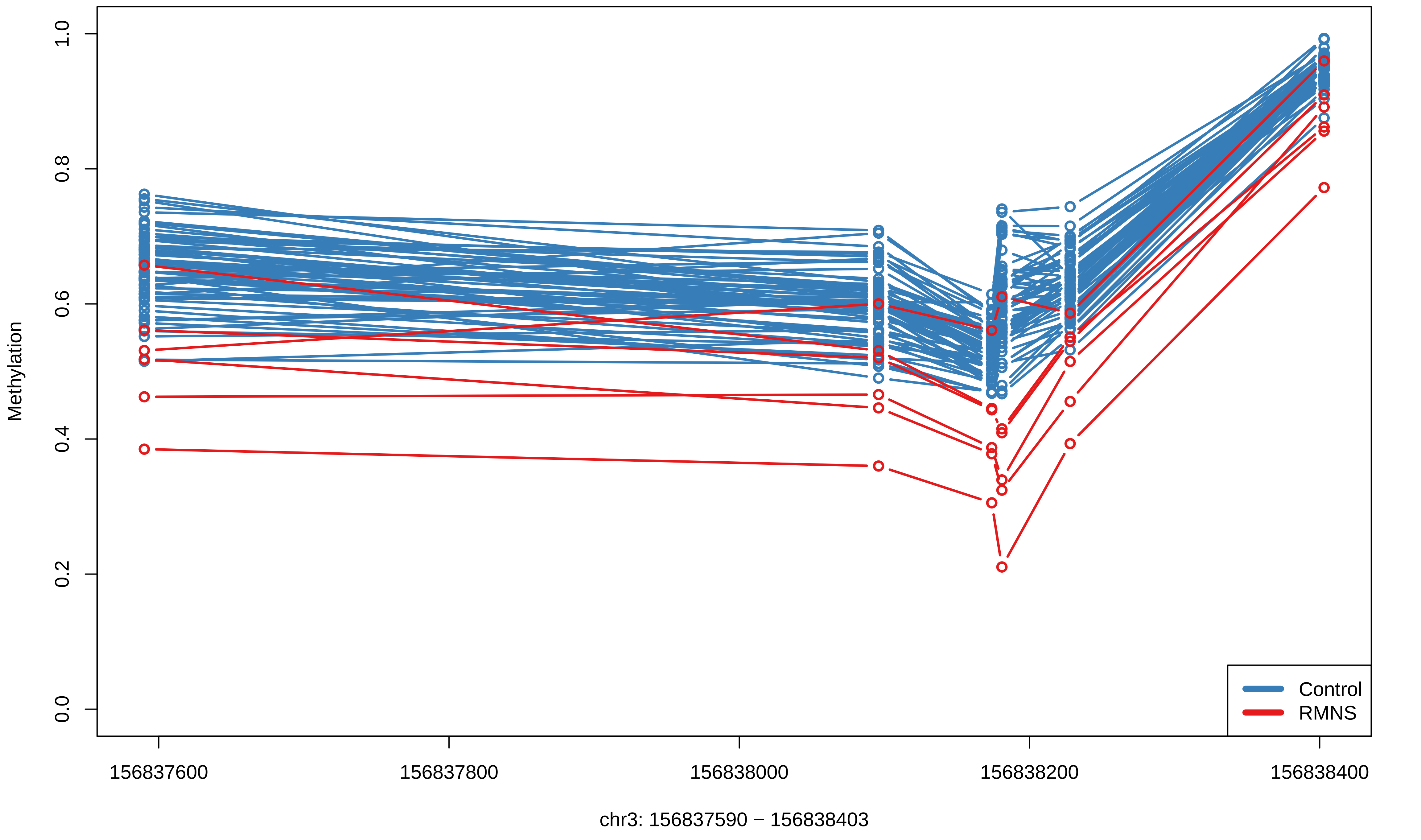

Methylation
0.0
0.2
0.4
0.6
0.8
1.0
156837600
156837800
156838000
156838200
156838400
chr3: 156837590 − 156838403
Control
RMNS

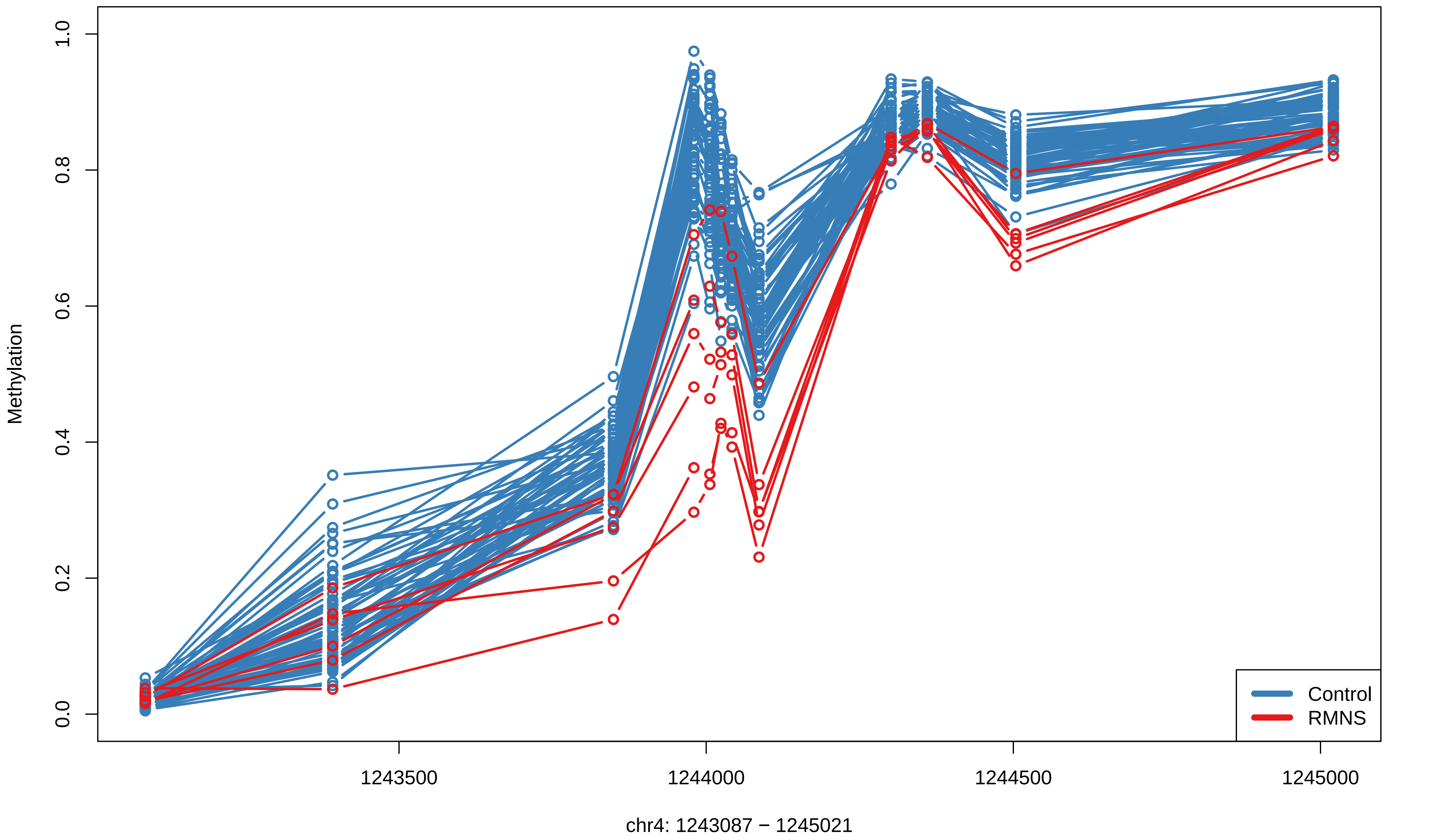

Methylation
0.0
0.2
0.4
0.6
0.8
1.0
1243500
1244000
1244500
1245000
chr4: 1243087 − 1245021
Control
RMNS

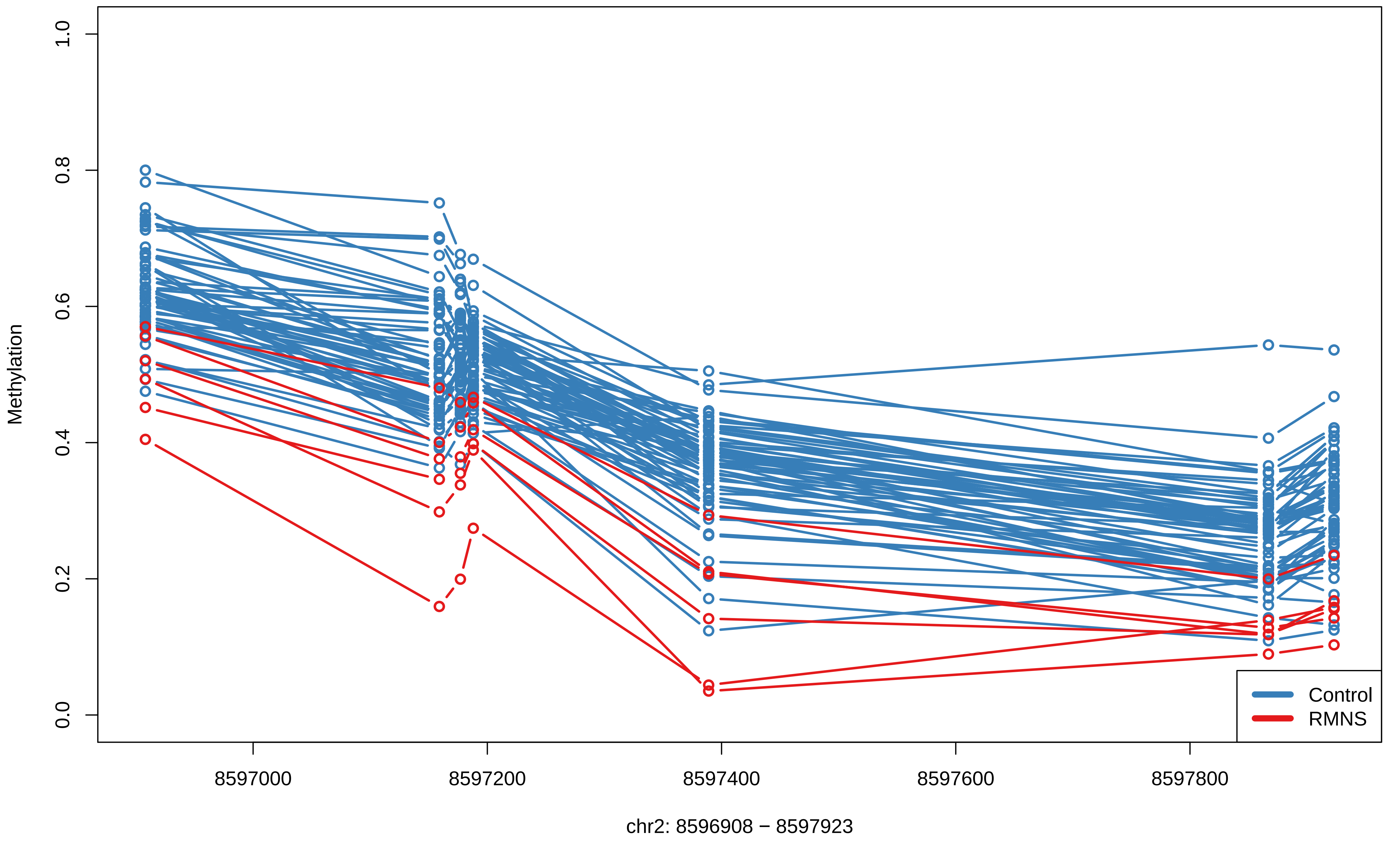

Methylation
0.0
0.2
0.4
0.6
0.8
1.0
8597000
8597200
8597400
8597600
8597800
chr2: 8596908 − 8597923
Control
RMNS

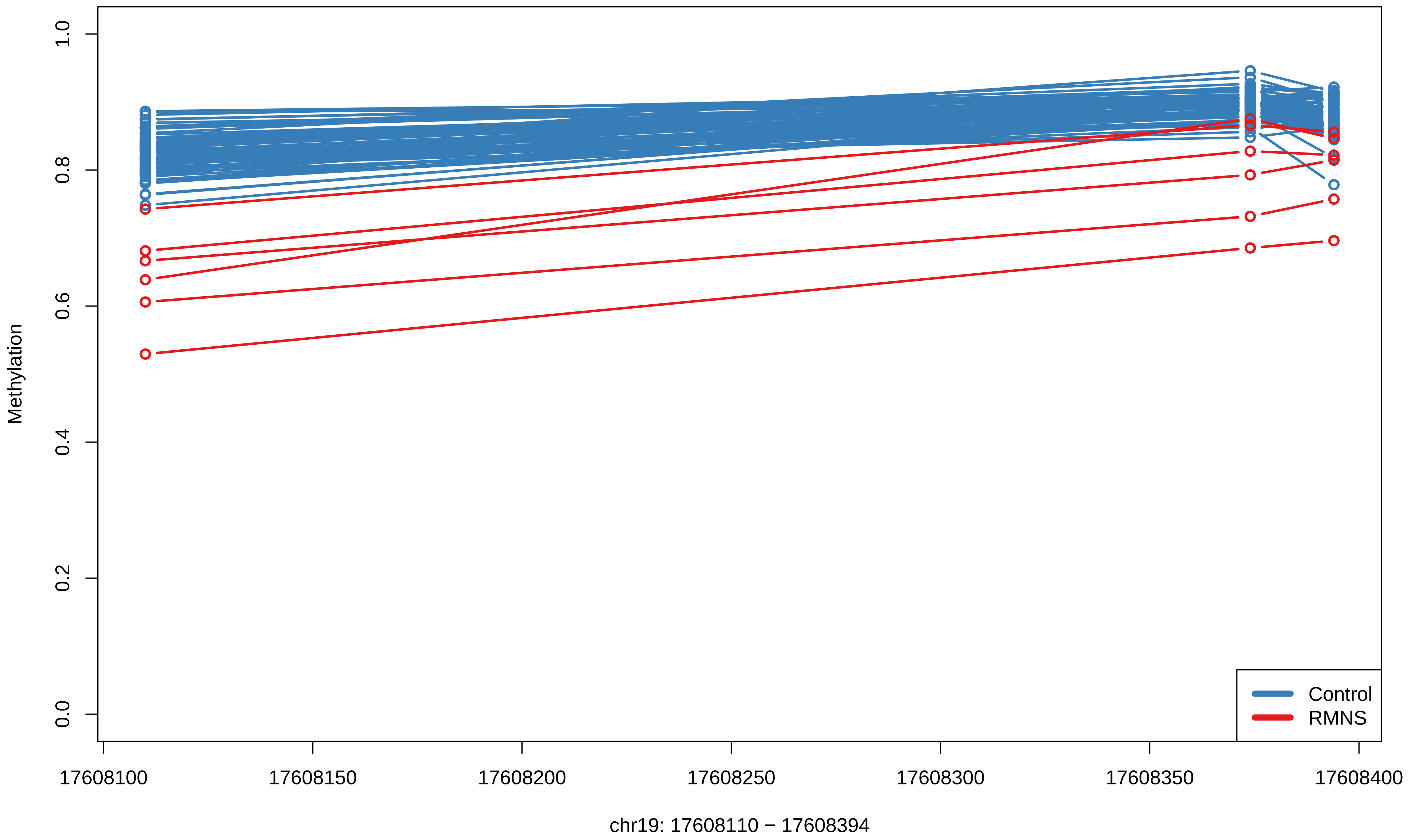

Methylation
1.0
0.8
0.6
0.4
0.2
0.0
Control
RMNS
17608100
17608150
17608200
17608250
17608300
17608350
17608400
chr19: 17608110 − 17608394

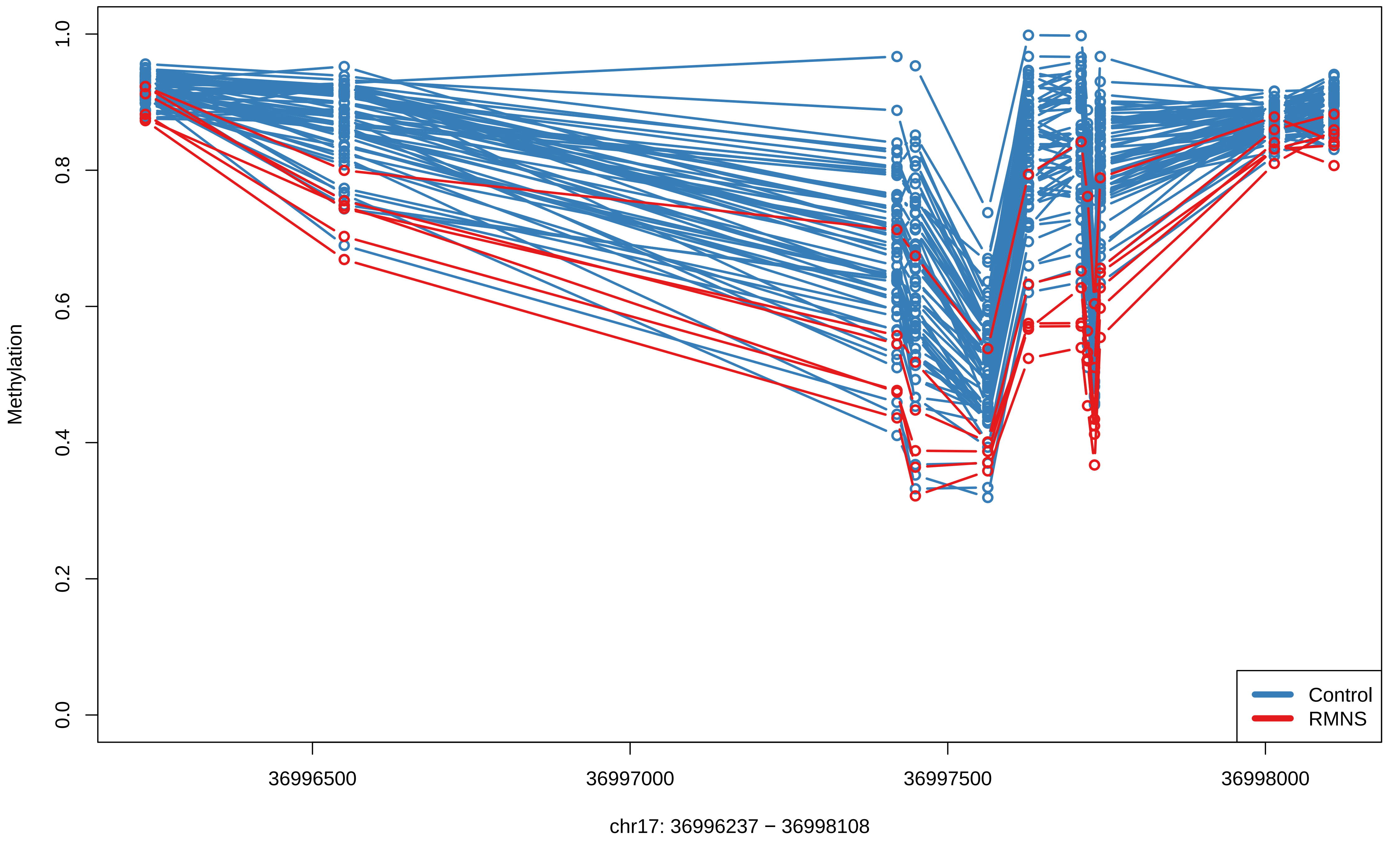

Methylation
1.0
0.8
0.6
0.4
0.2
0.0
36996500
36997000
36997500
36998000
chr17: 36996237 − 36998108
Control
RMNS

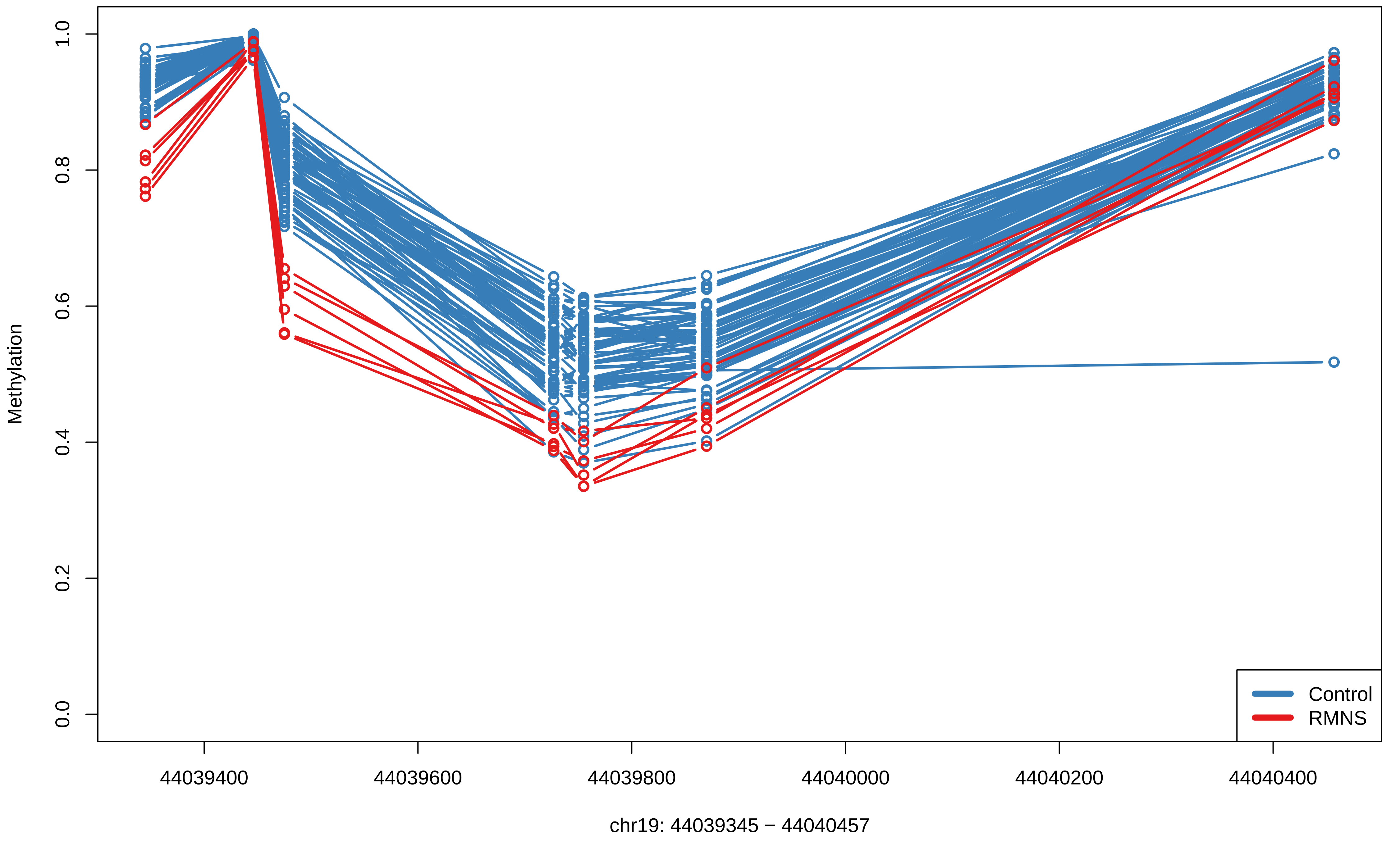

Methylation
0.0
0.2
0.4
0.6
0.8
1.0
44039400
44039600
44039800
44040000
44040200
44040400
chr19: 44039345 − 44040457
Control
RMNS

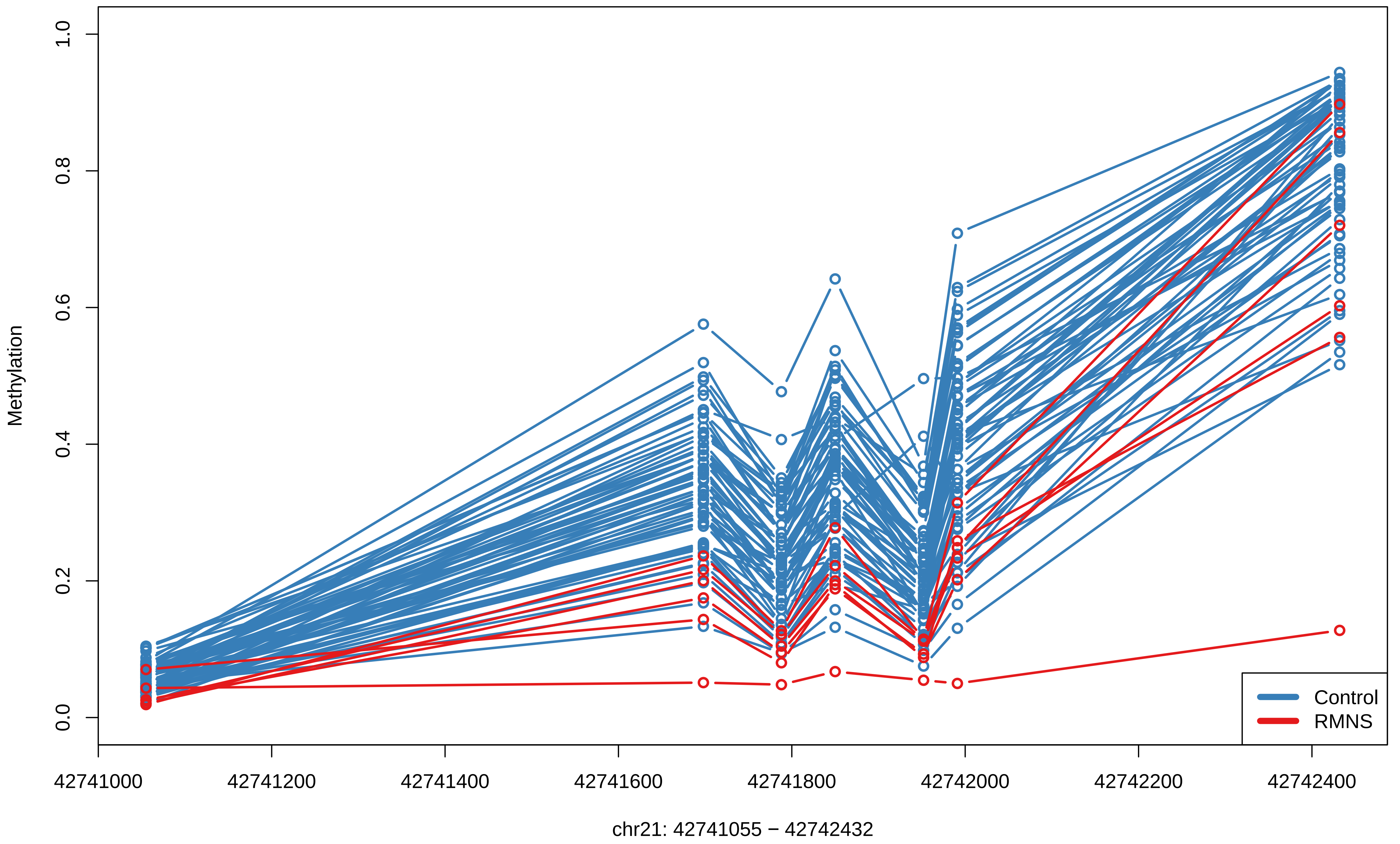

Methylation
0.0
0.2
0.4
0.6
0.8
1.0
42741000
42741200
42741400
42741600
42741800
42742000
42742200
42742400
chr21: 42741055 − 42742432
Control
RMNS

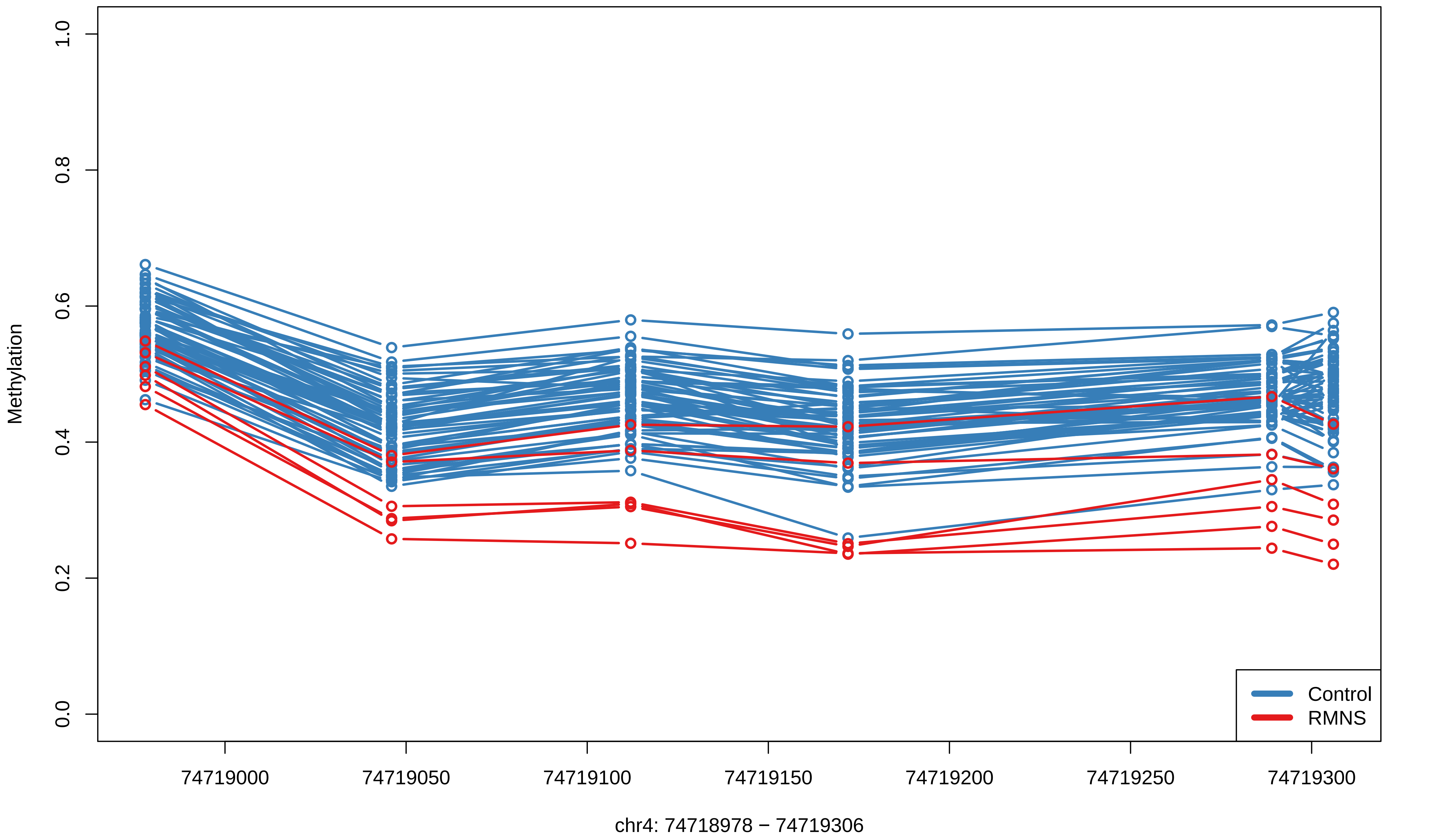

Methylation
0.0
0.2
0.4
0.6
0.8
1.0
74719000
74719050
74719100
74719150
74719200
74719250
74719300
chr4: 74718978 − 74719306
Control
RMNS

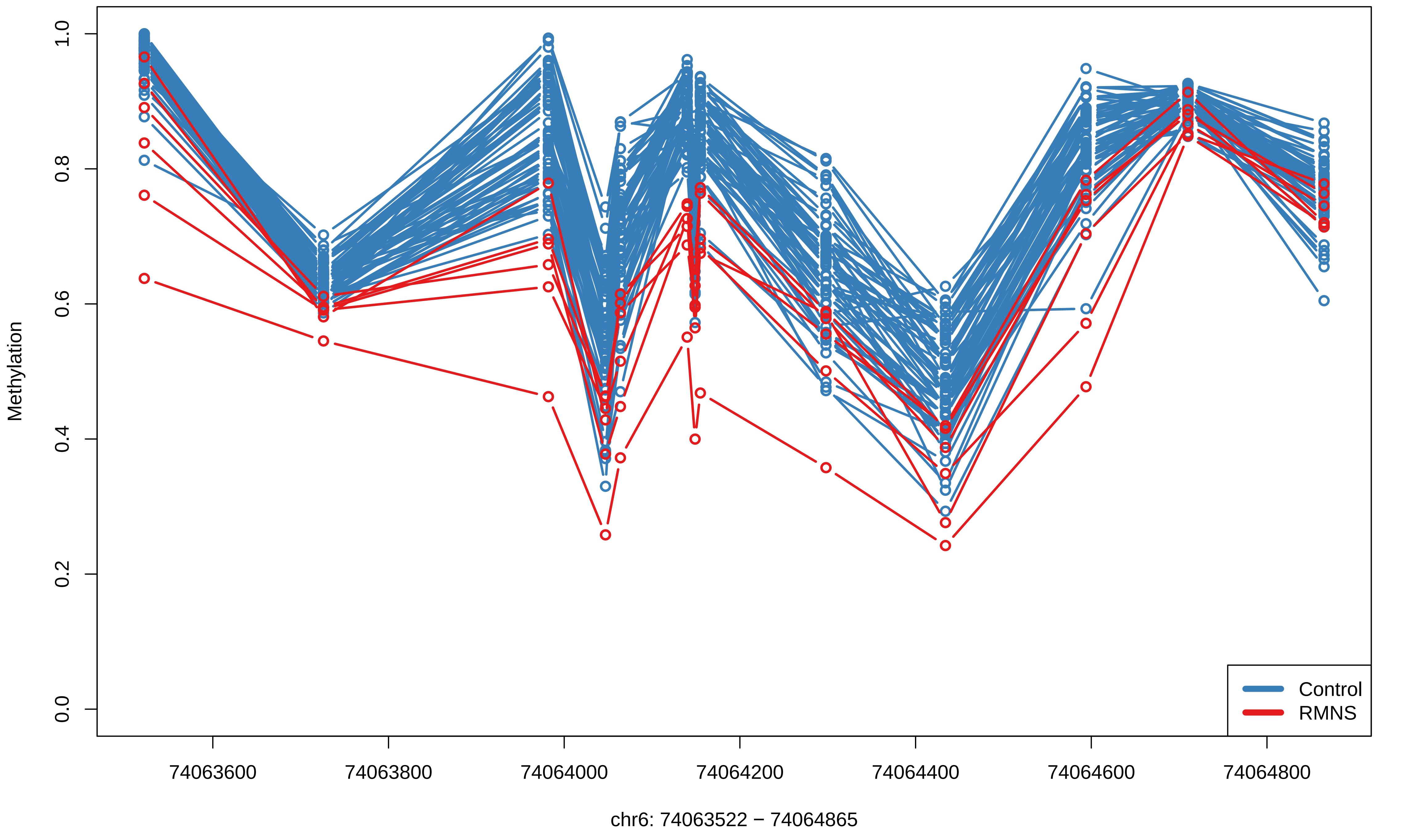

Methylation
1.0
0.8
0.6
0.4
0.2
0.0
74063600
74063800
74064000
74064200
74064400
74064600
74064800
chr6: 74063522 − 74064865
Control
RMNS

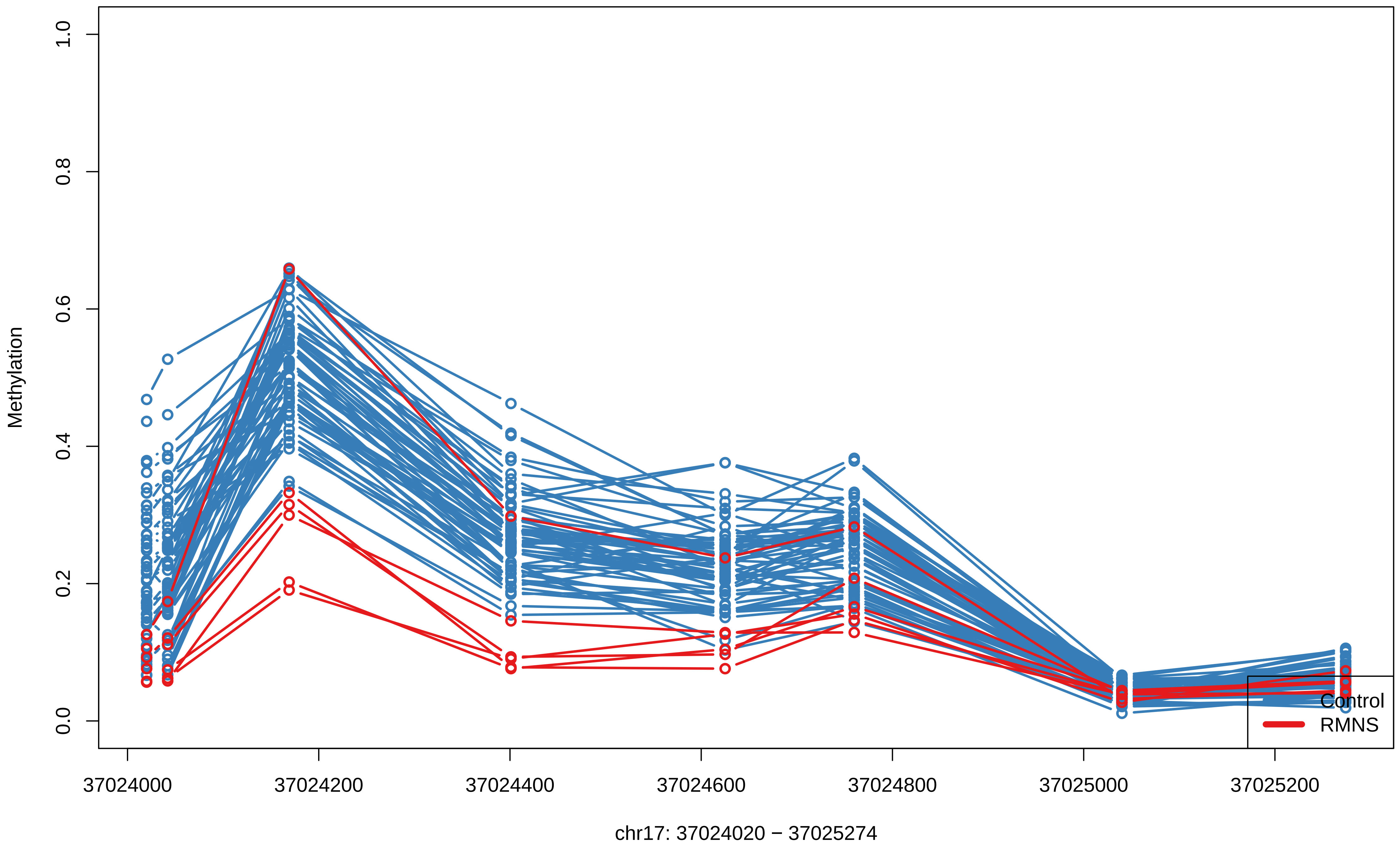
Methylation
0.0
0.2
0.4
0.6
0.8
1.0
37024000
37024200
37024400
37024600
37024800
37025000
37025200
chr17: 37024020 − 37025274
Control
RMNS

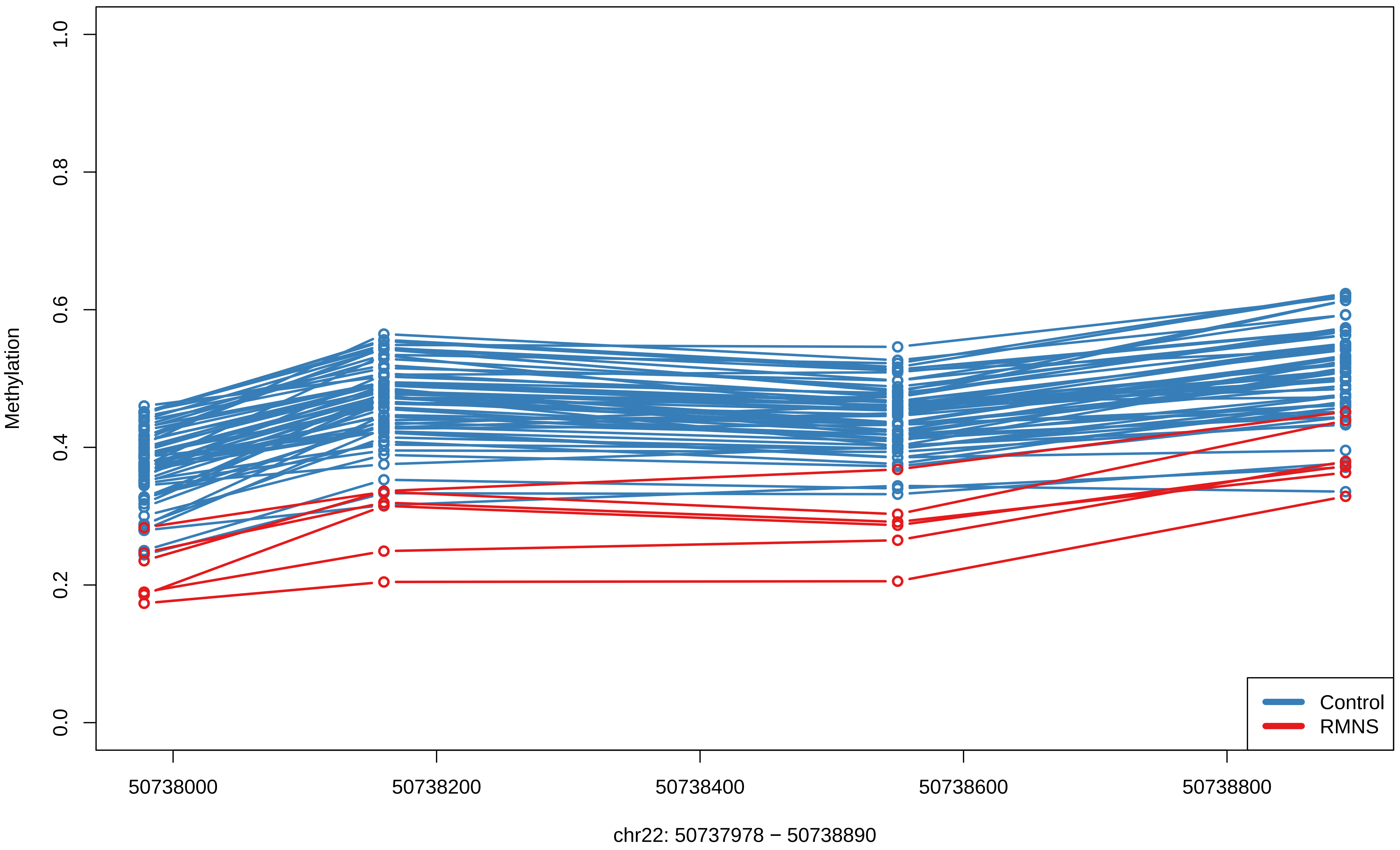

Methylation
0.0
0.2
0.4
0.6
0.8
1.0
50738000
50738200
50738400
50738600
50738800
chr22: 50737978 − 50738890
Control
RMNS

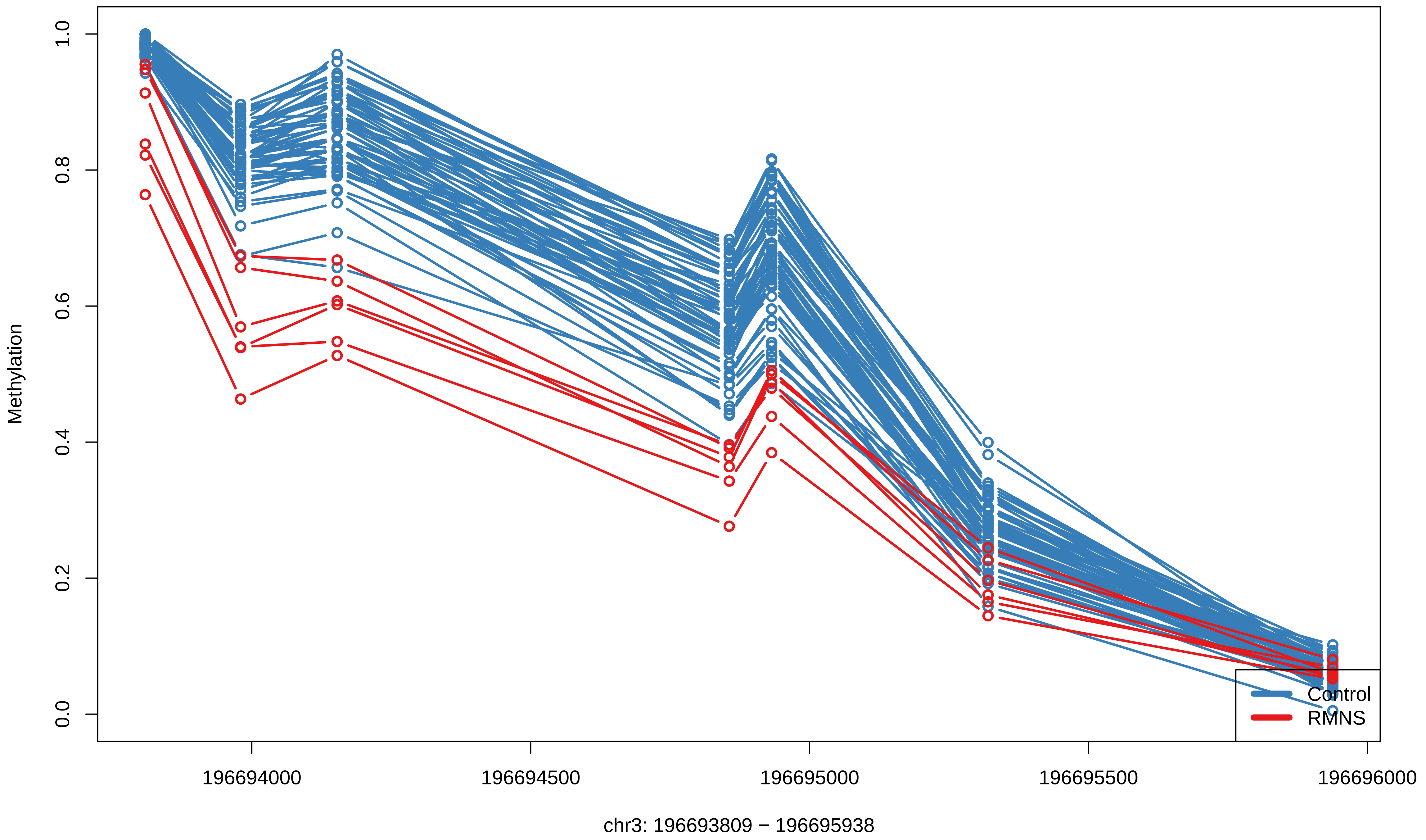

Methylation
0.0
0.2
0.4
0.6
0.8
1.0
196694000
196694500
196695000
196695500
196696000
chr3: 196693809 − 196695938
Control
RMNS

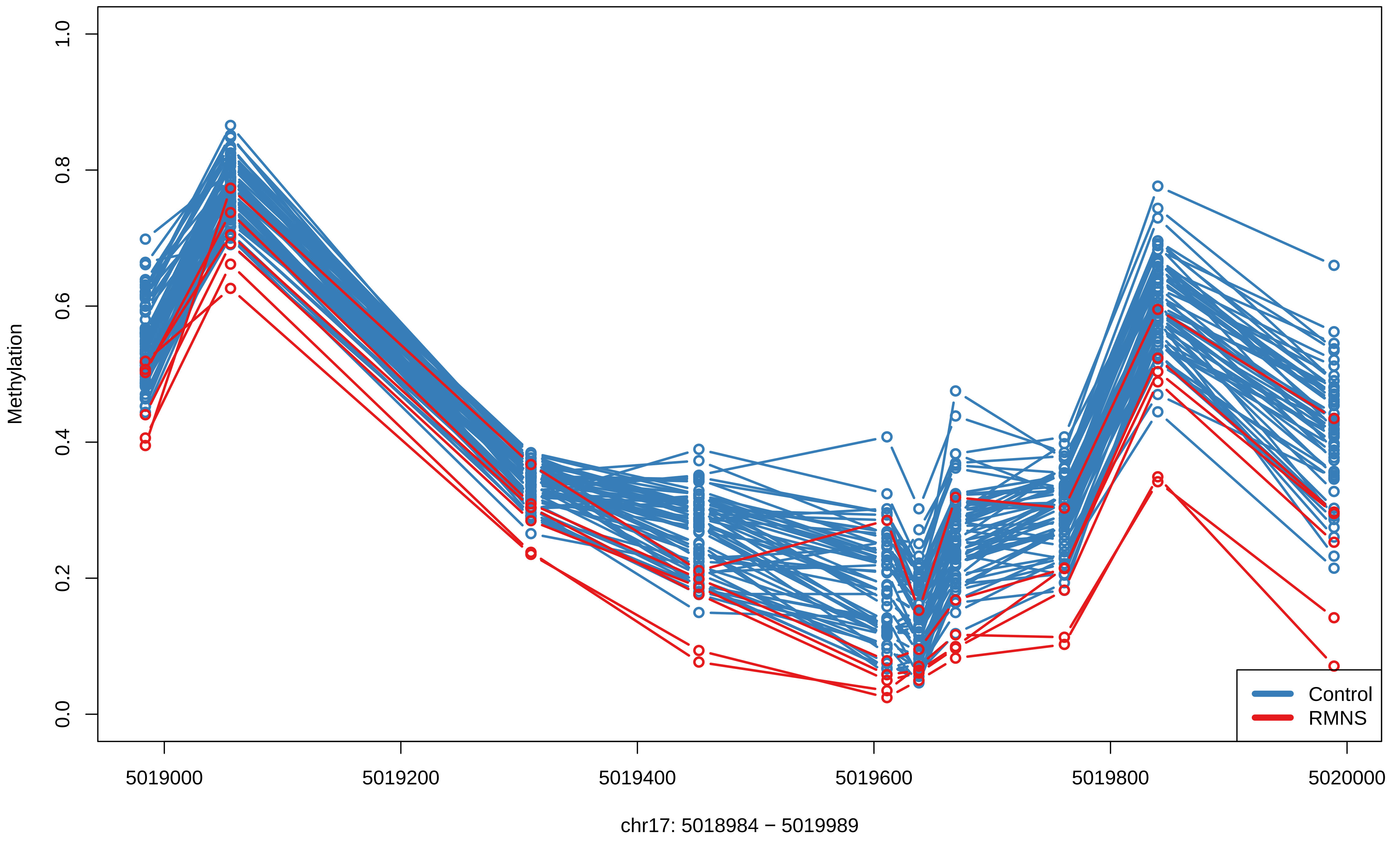

Methylation
1.0
0.8
0.6
0.4
0.2
0.0
5019000
5019200
5019400
5019600
5019800
5020000
chr17: 5018984 − 5019989
Control
RMNS

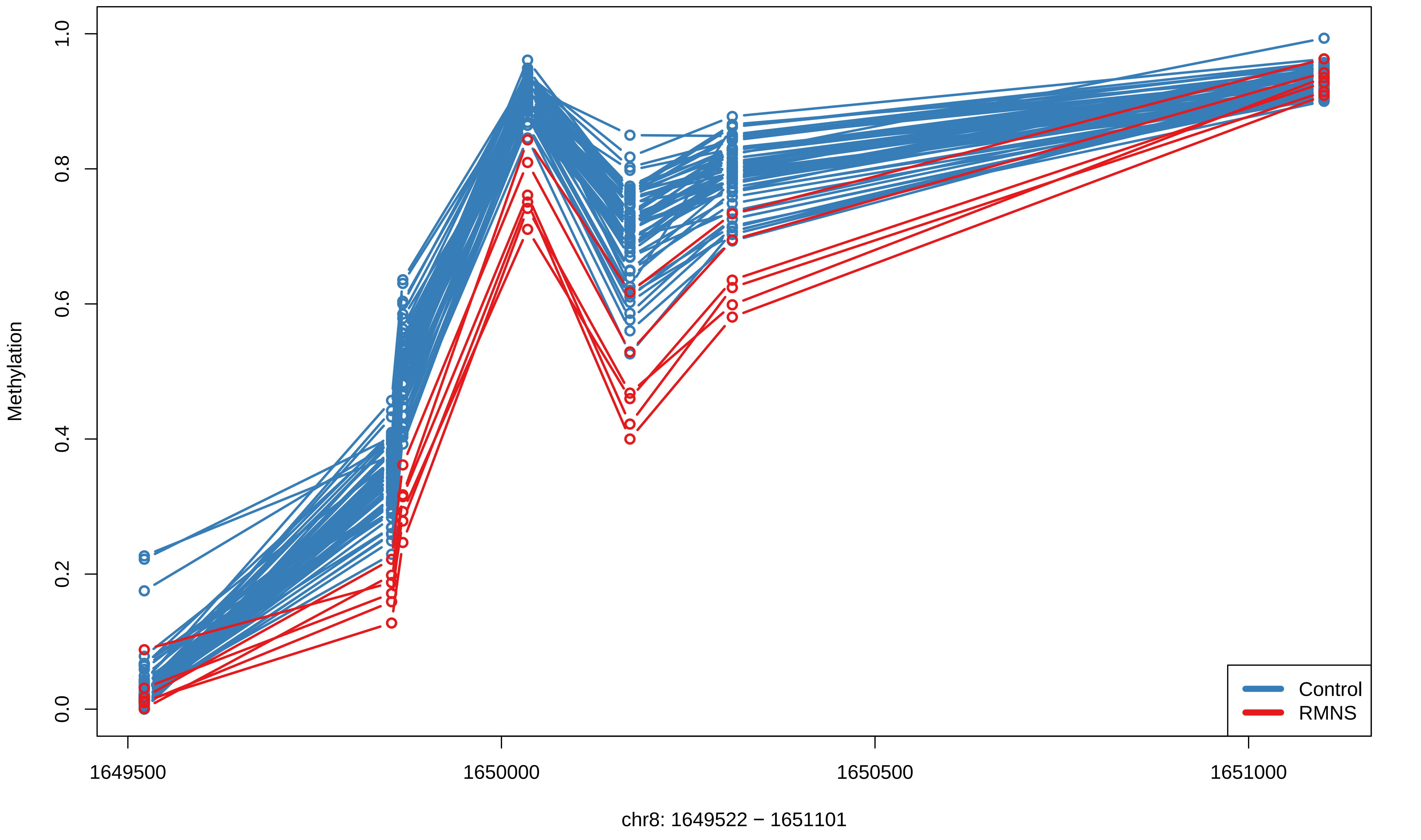

Methylation
1.0
0.8
0.6
0.4
0.2
0.0
1649500
1650000
1650500
1651000
chr8: 1649522 − 1651101
Control
RMNS

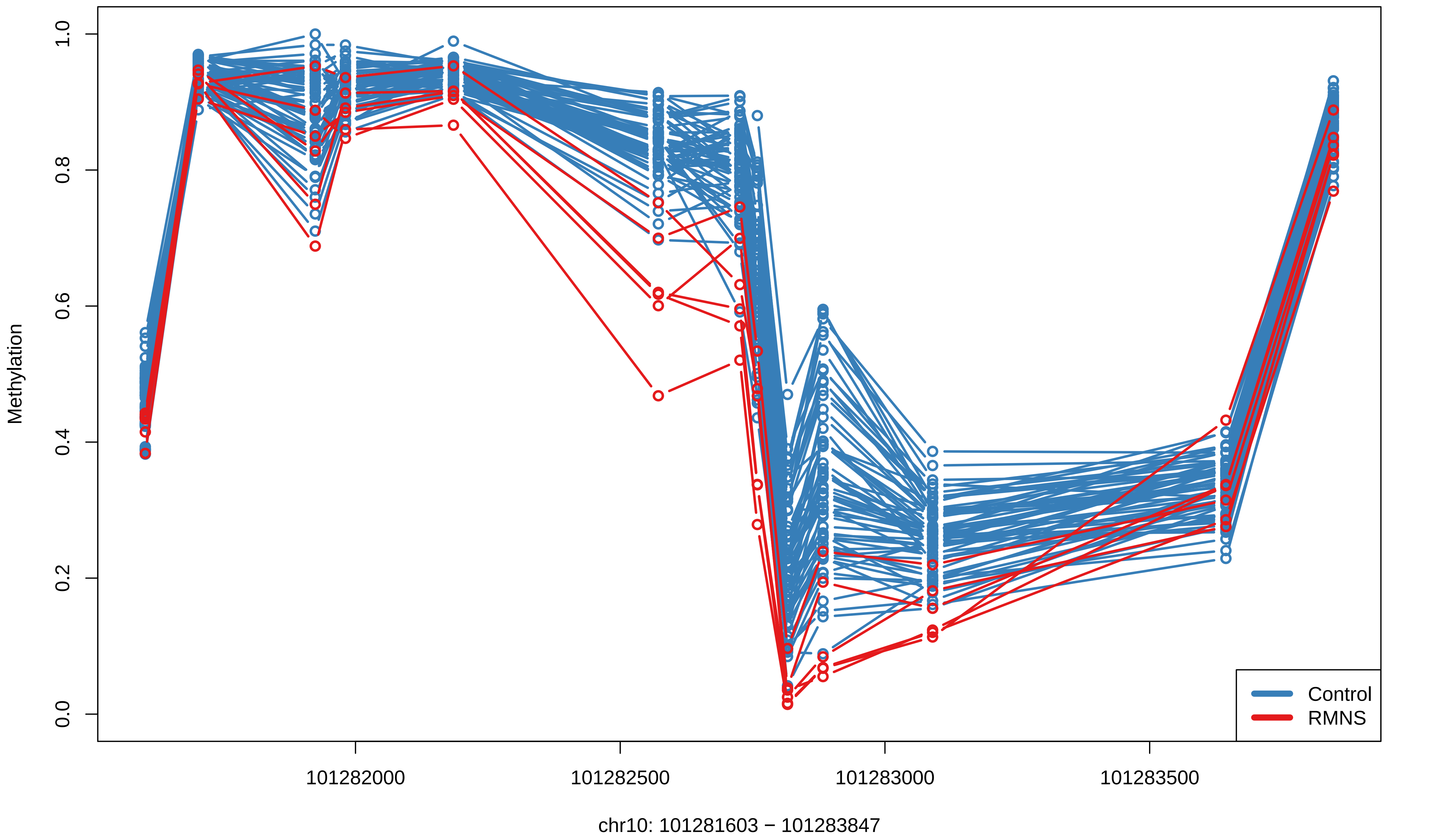

Methylation
0.0
0.2
0.4
0.6
0.8
1.0
101282000
101282500
101283000
101283500
chr10: 101281603 − 101283847
Control
RMNS

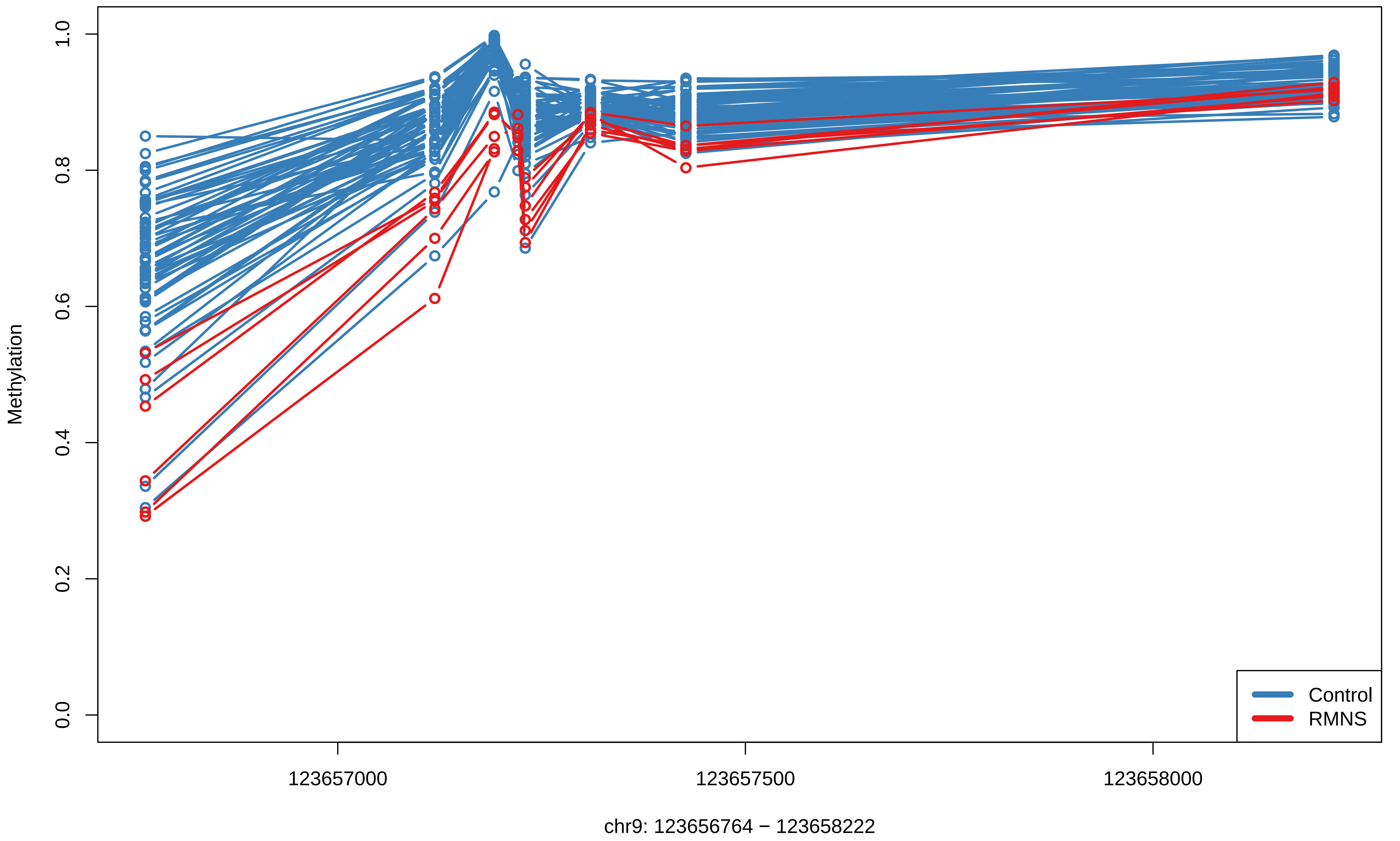

Methylation
1.0
0.8
0.6
0.4
0.2
0.0
123657000
123657500
123658000
chr9: 123656764 − 123658222
Control
RMNS

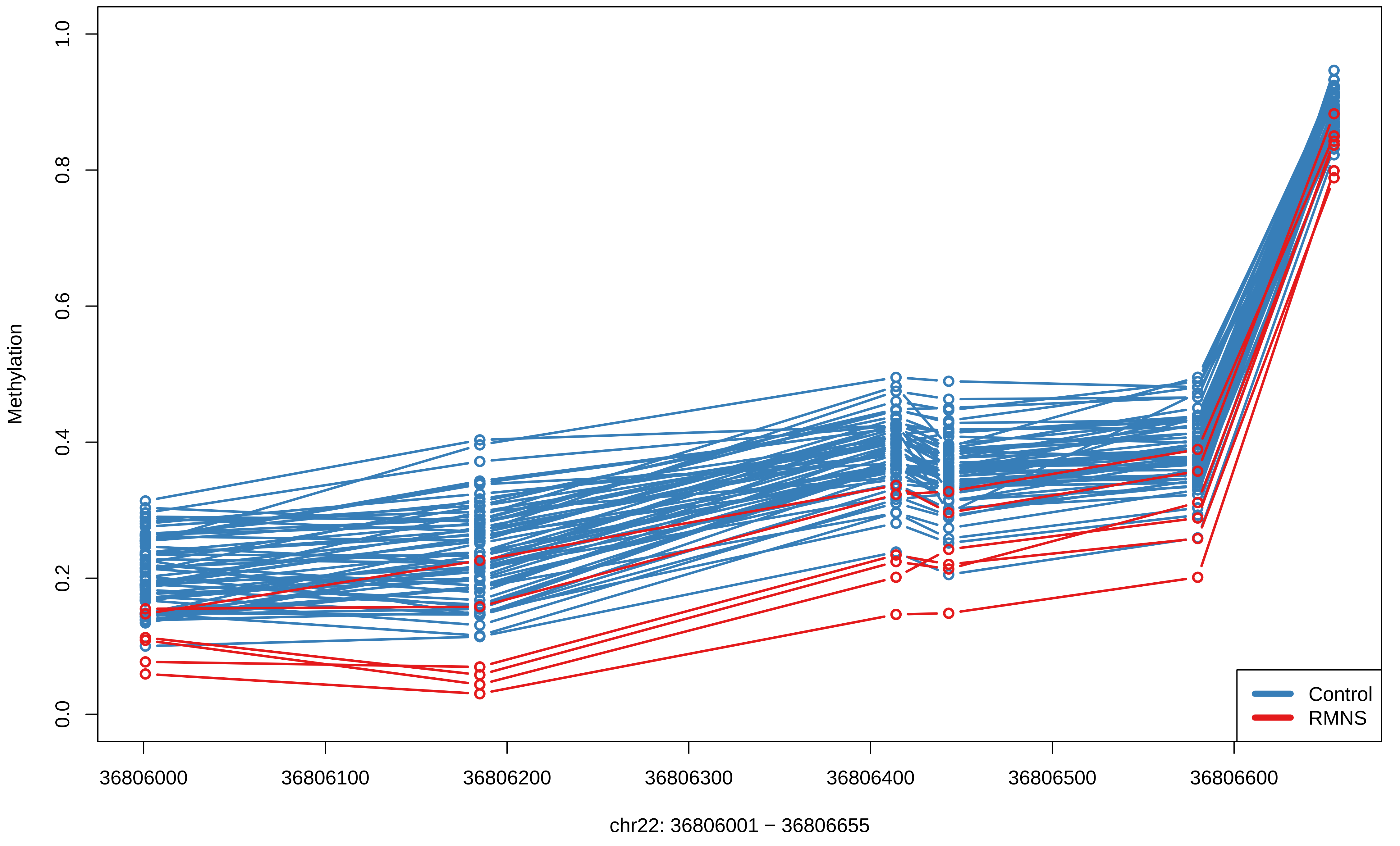

Methylation
0.0
0.2
0.4
0.6
0.8
1.0
36806000
36806100
36806200
36806300
36806400
36806500
36806600
chr22: 36806001 − 36806655
Control
RMNS

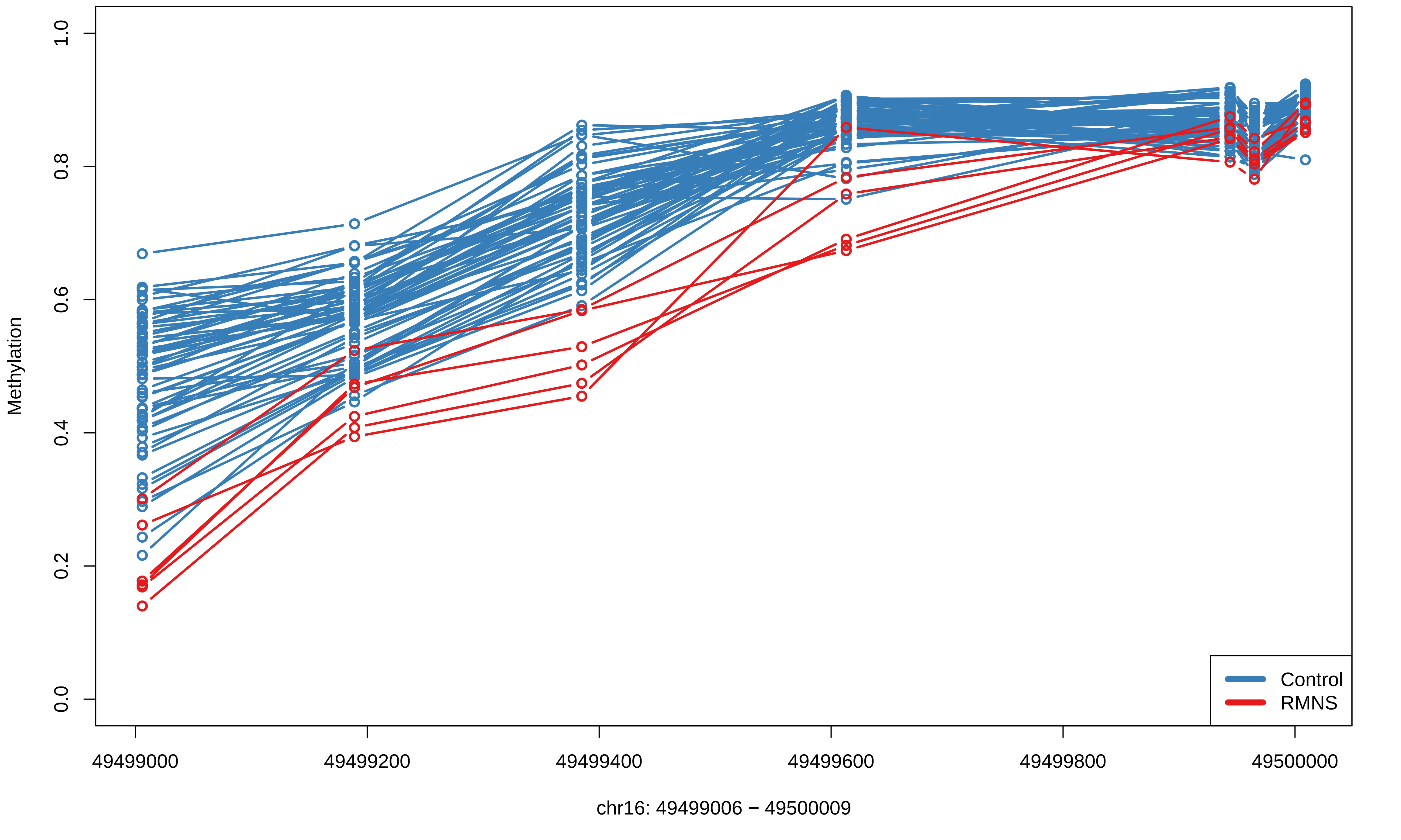
Methylation
1.0
0.8
0.6
0.4
0.2
0.0
49499000
49499200
49499400
49499600
49499800
49500000
chr16: 49499006 − 49500009
Control
RMNS

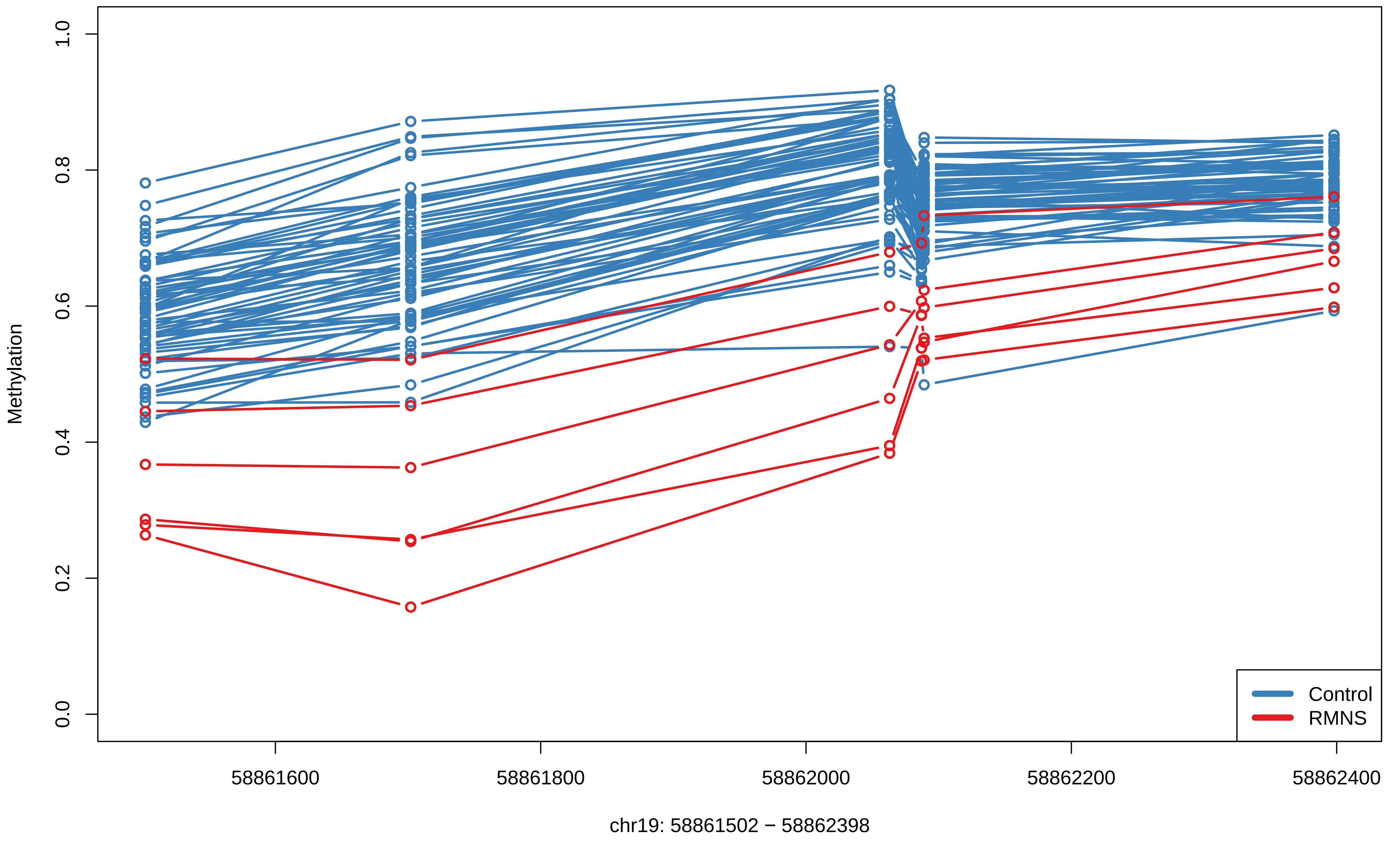

Methylation
0.0
0.2
0.4
0.6
0.8
1.0
58861600
58861800
58862000
58862200
58862400
chr19: 58861502 − 58862398
Control
RMNS

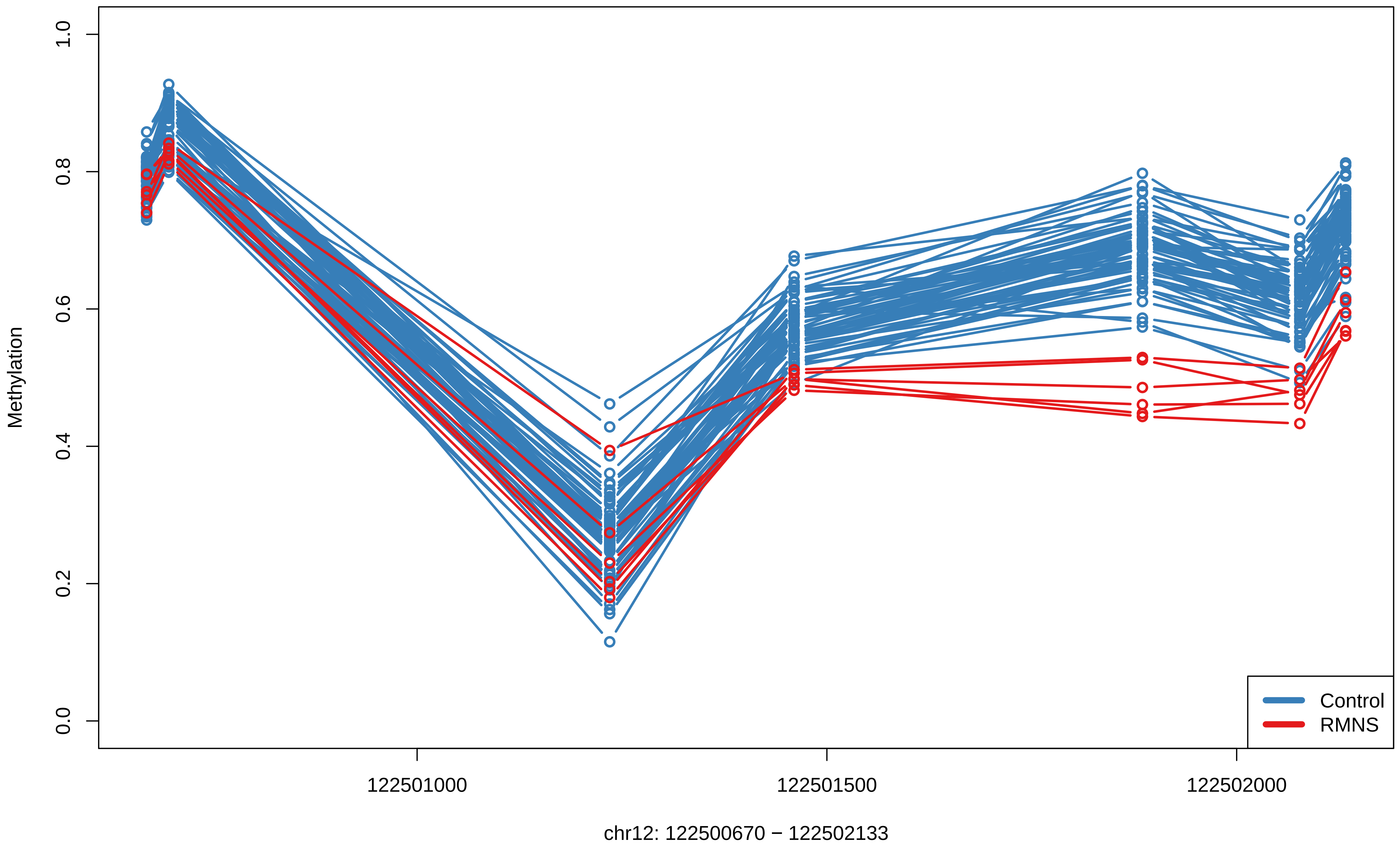

Methylation
0.0
0.2
0.4
0.6
0.8
1.0
122501000
122501500
122502000
chr12: 122500670 − 122502133
Control
RMNS

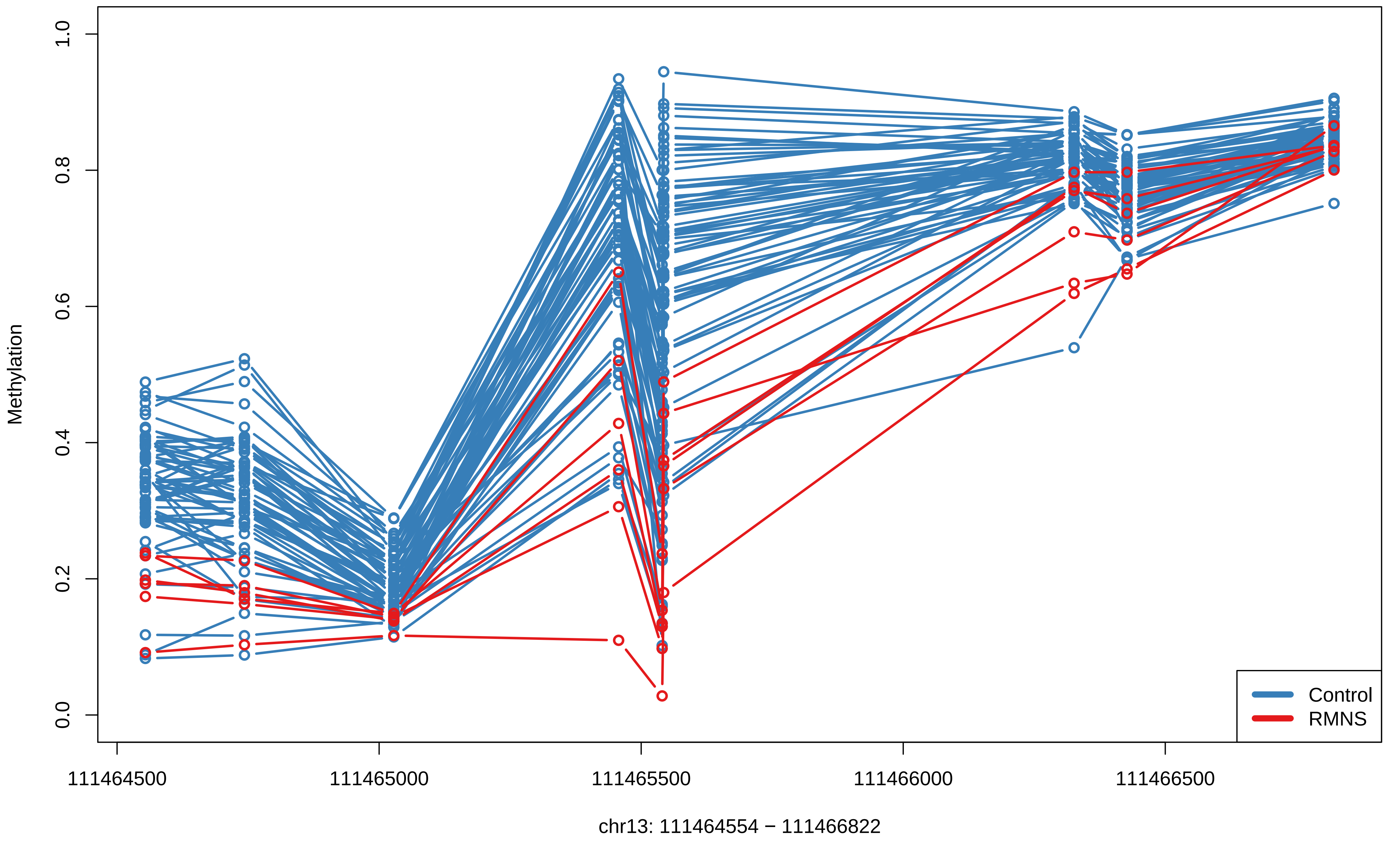

Methylation
0.0
0.2
0.4
0.6
0.8
1.0
111464500
111465000
111465500
111466000
111466500
chr13: 111464554 − 111466822
Control
RMNS

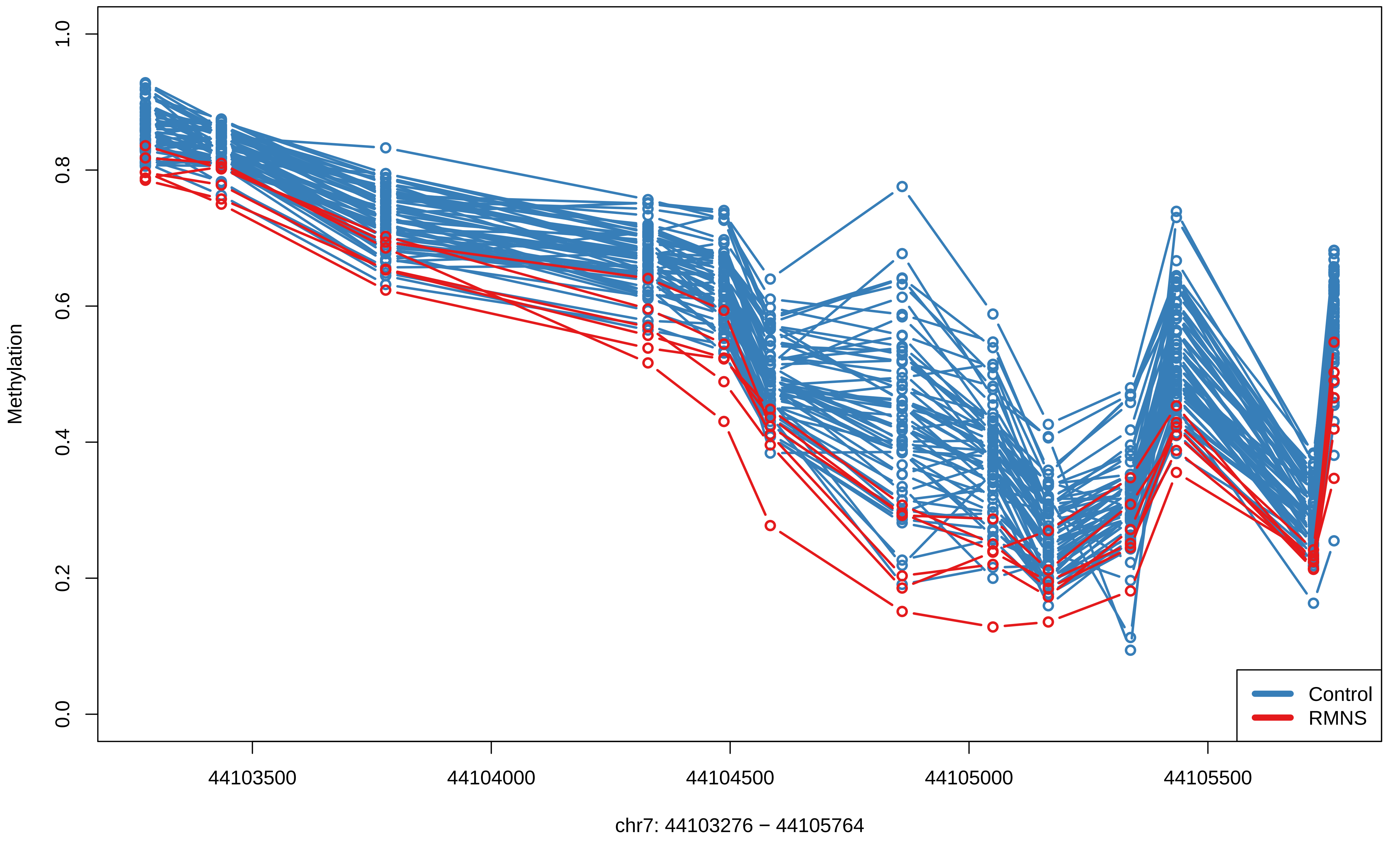

Methylation
0.0
0.2
0.4
0.6
0.8
1.0
44103500
44104000
44104500
44105000
44105500
chr7: 44103276 − 44105764
Control
RMNS

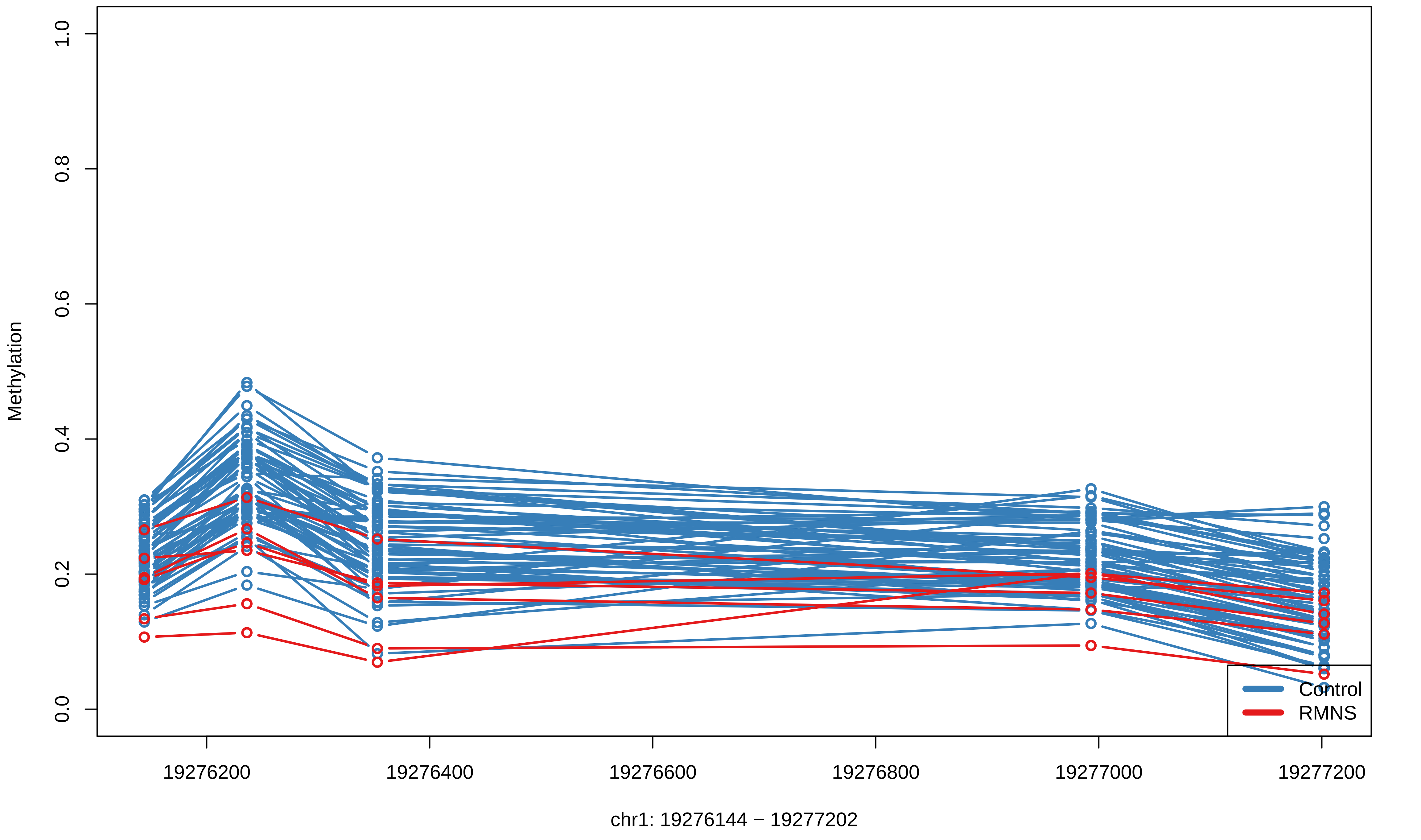

Methylation
0.0
0.2
0.4
0.6
0.8
1.0
19276200
19276400
19276600
19276800
19277000
19277200
chr1: 19276144 − 19277202
Control
RMNS

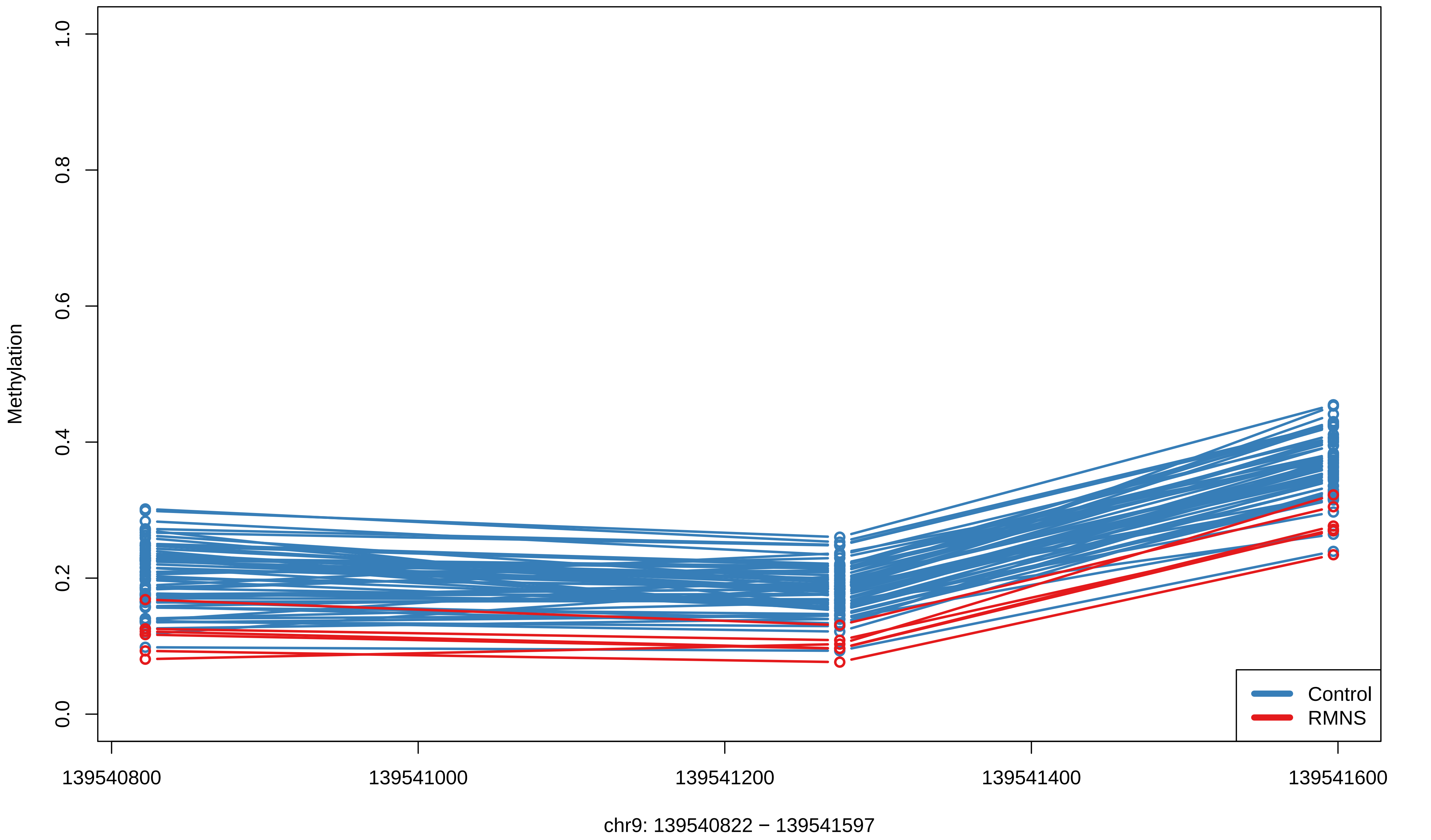

1.0
0.8
0.6
0.4
0.2
0.0
Methylation
139540800
139541000
139541200
139541400
139541600
chr9: 139540822 − 139541597
Control
RMNS

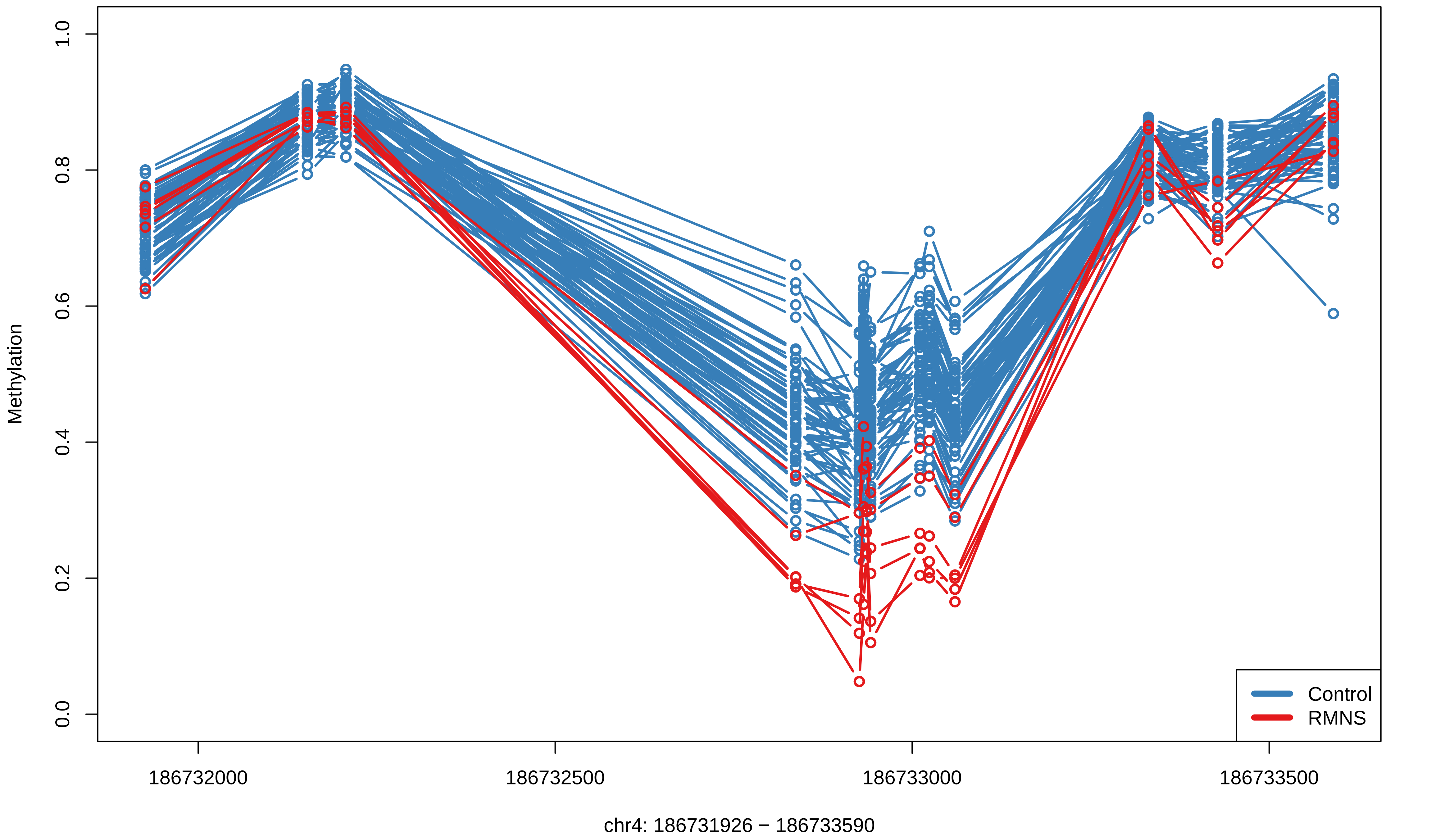

1.0
0.8
0.6
0.4
0.2
0.0
Methylation
186732000
186732500
186733000
186733500
chr4: 186731926 − 186733590
Control
RMNS

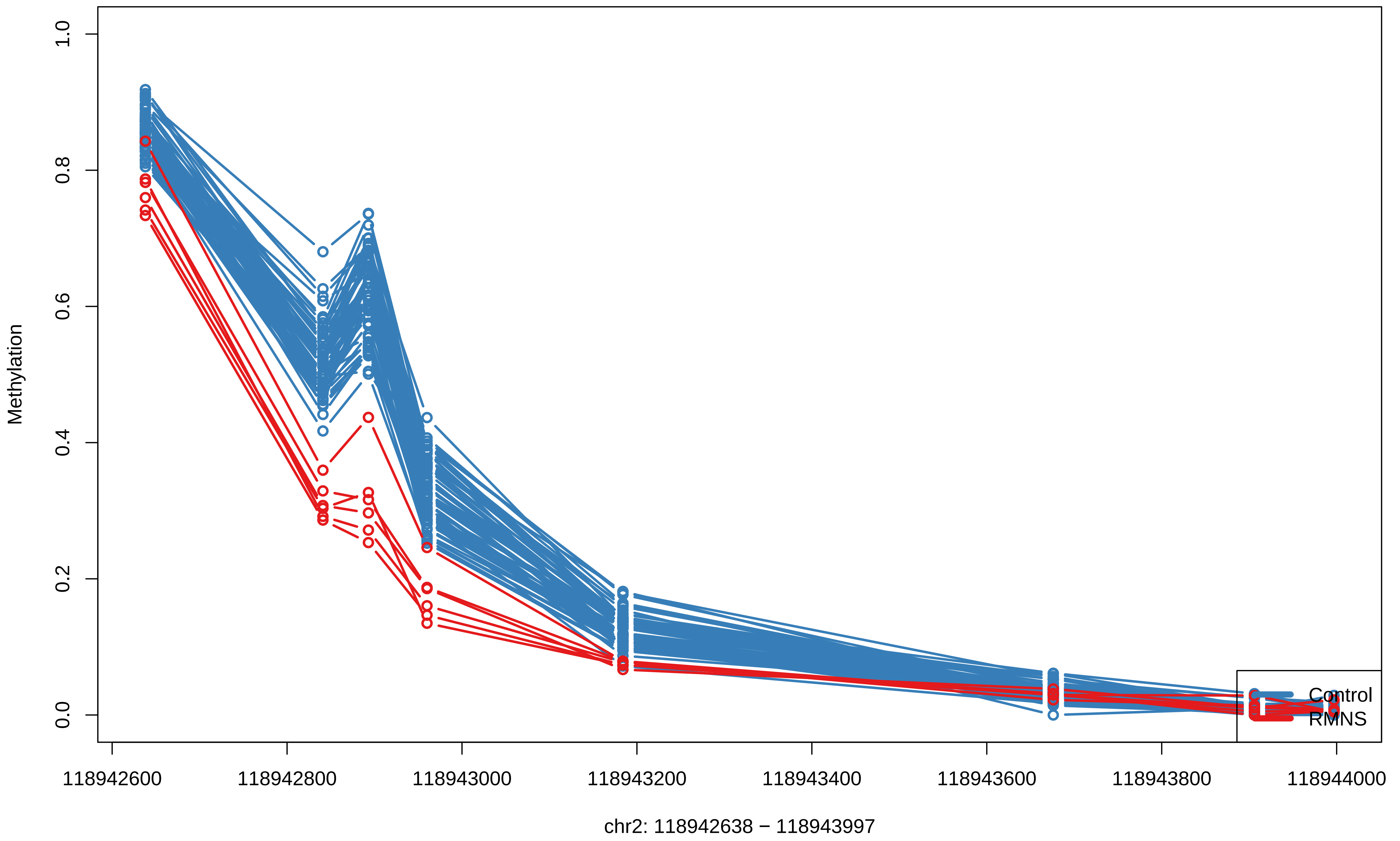

Methylation
1.0
0.8
0.6
0.4
0.2
0.0
118942600
118942800
118943000
118943200
118943400
118943600
118943800
118944000
chr2: 118942638 − 118943997
Control
RMNS

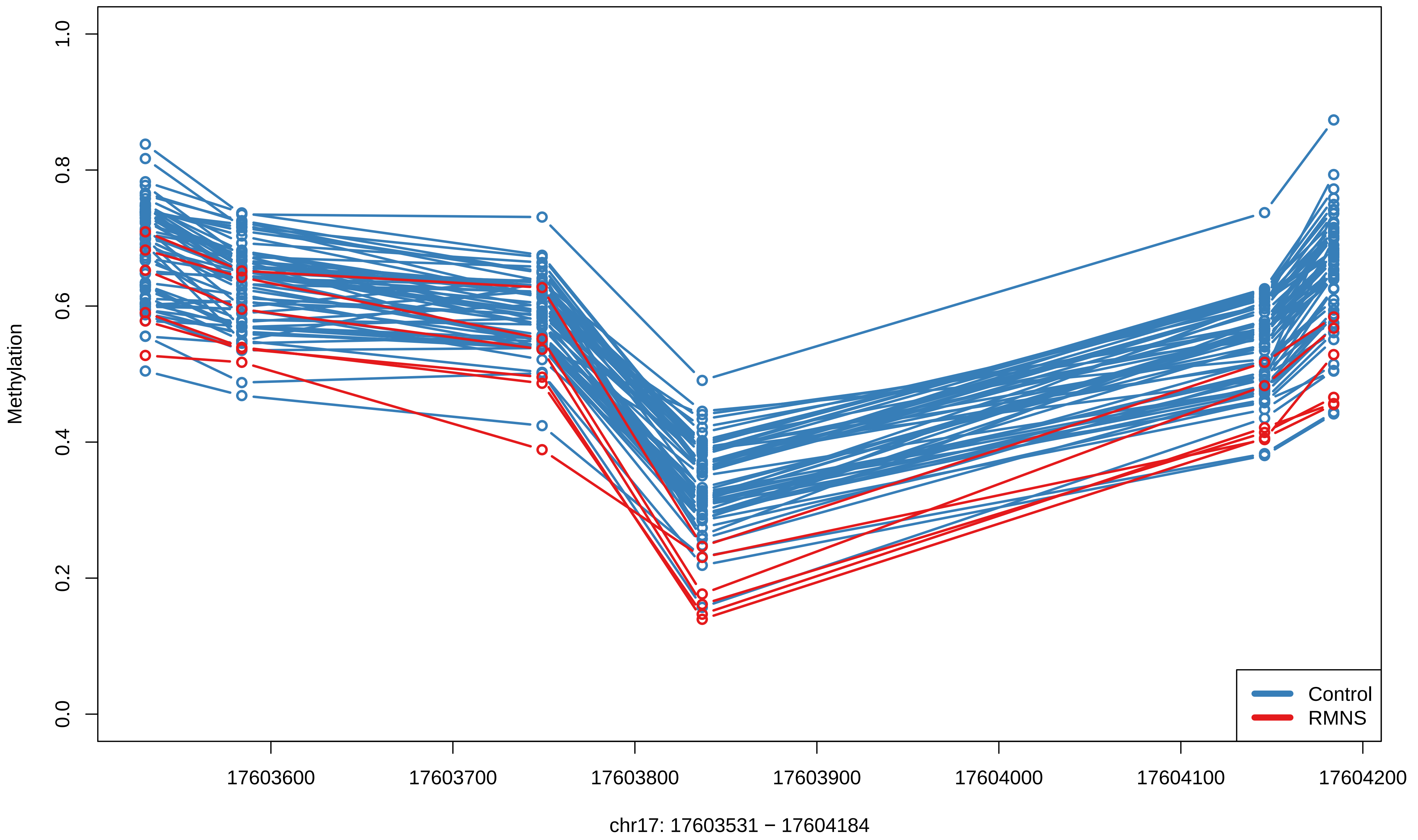
Methylation
0.0
0.2
0.4
0.6
0.8
1.0
17603600
17603700
17603800
17603900
17604000
17604100
17604200
chr17: 17603531 − 17604184
Control
RMNS

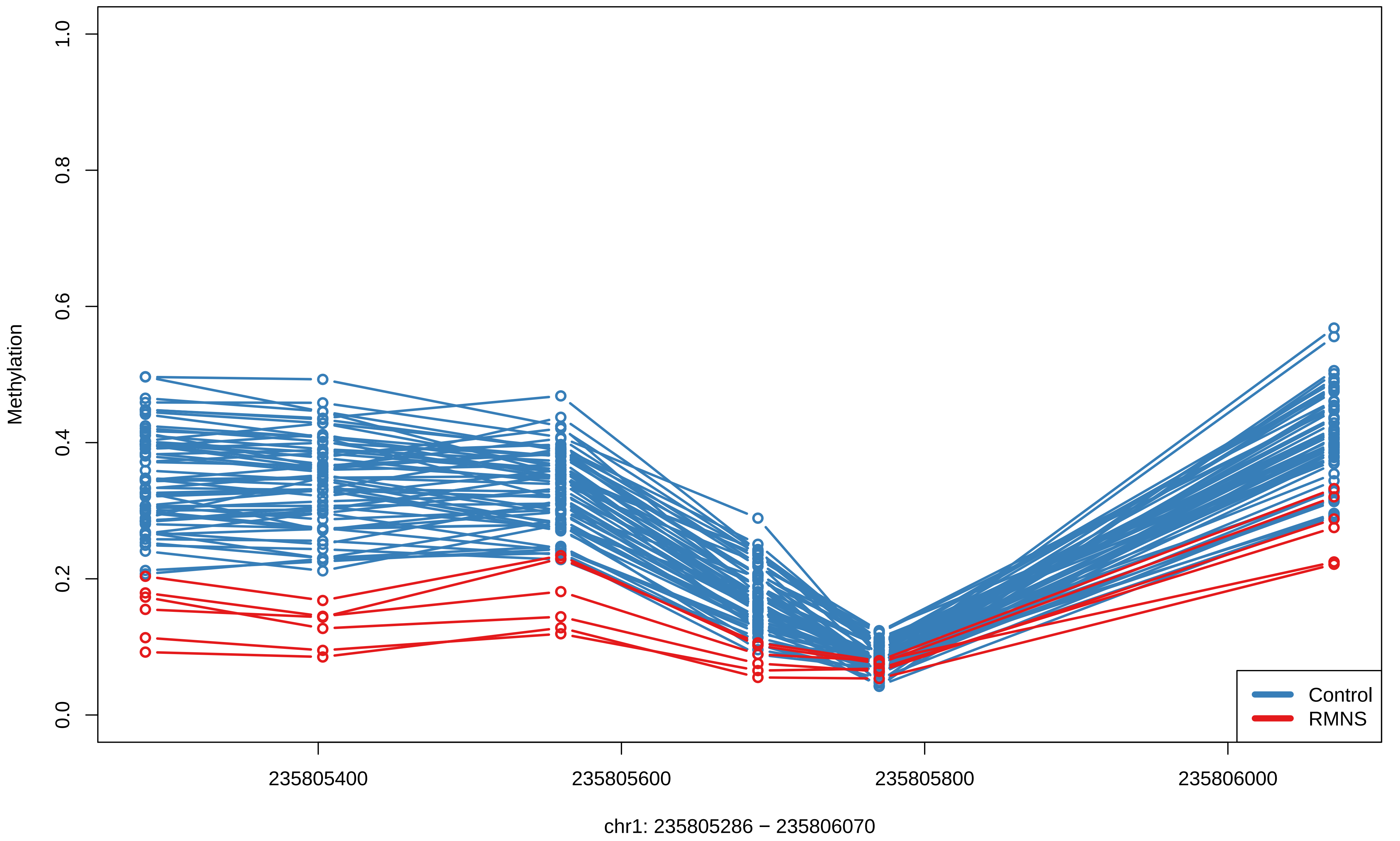

Methylation
1.0
0.8
0.6
0.4
0.2
0.0
235805400
235805600
235805800
235806000
chr1: 235805286 − 235806070
Control
RMNS

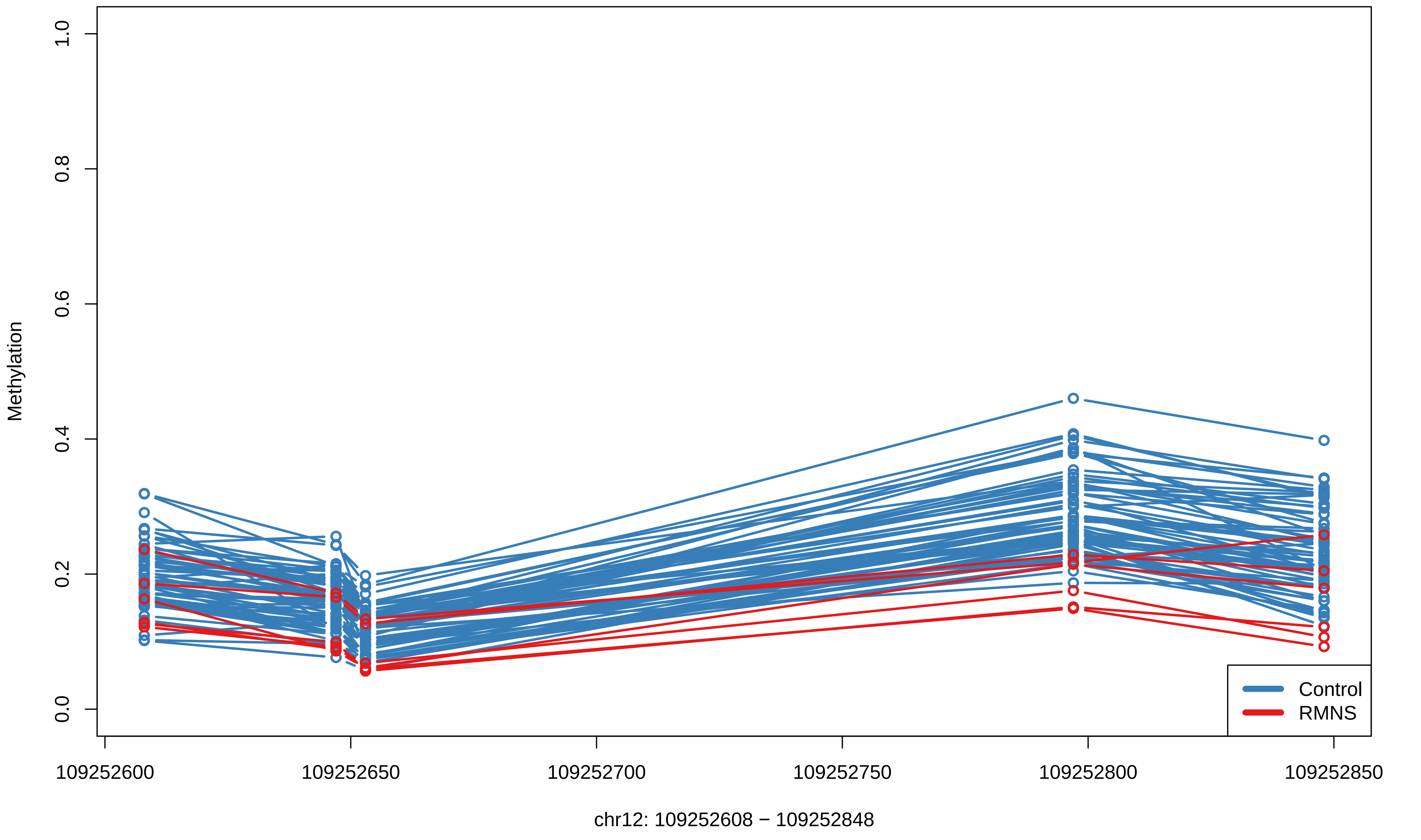

Methylation
0.0
0.2
0.4
0.6
0.8
1.0
109252600
109252650
109252700
109252750
109252800
109252850
chr12: 109252608 − 109252848
Control
RMNS

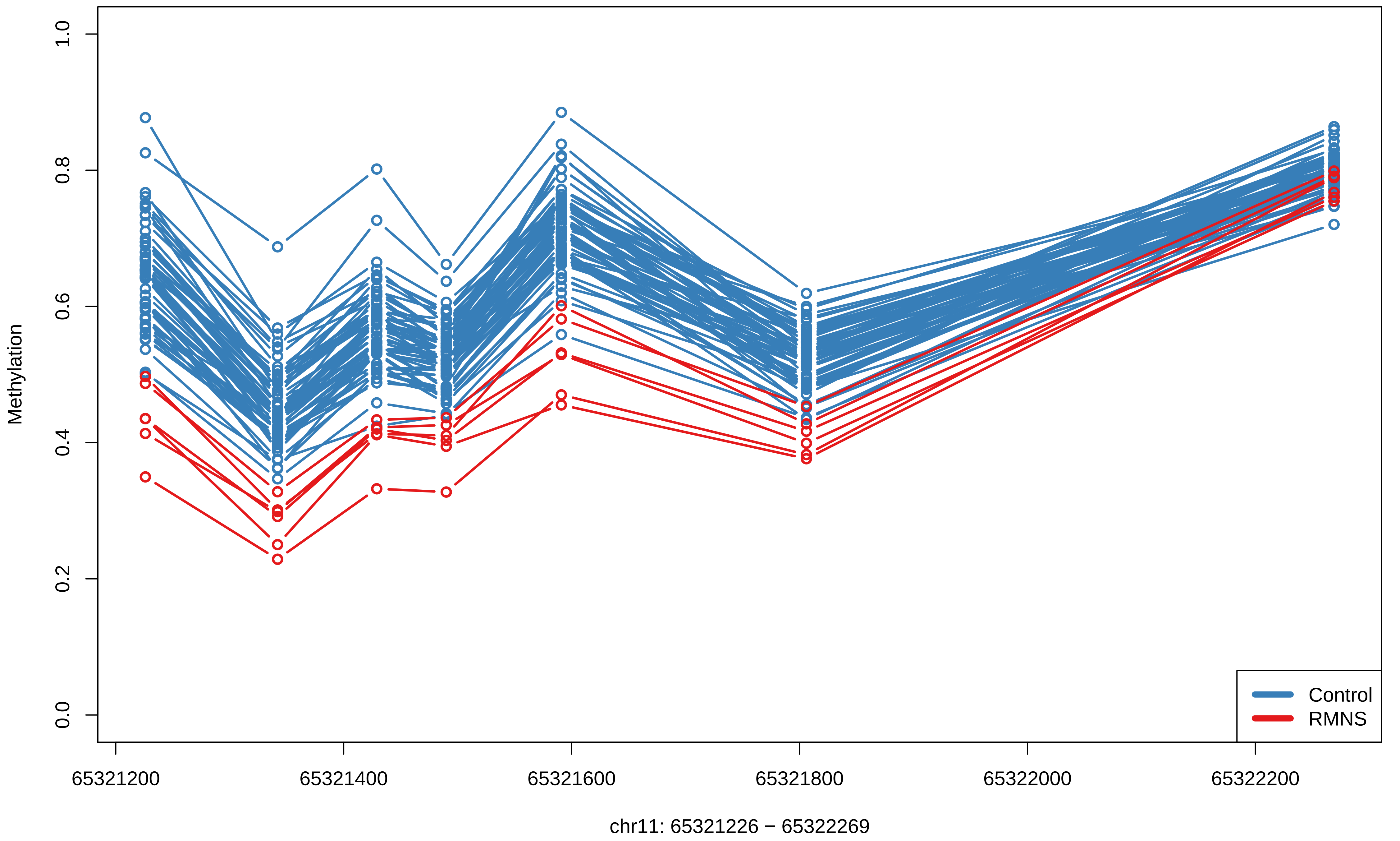

Methylation
1.0
0.8
0.6
0.4
0.2
0.0
65321200
65321400
65321600
65321800
65322000
65322200
chr11: 65321226 − 65322269
Control
RMNS

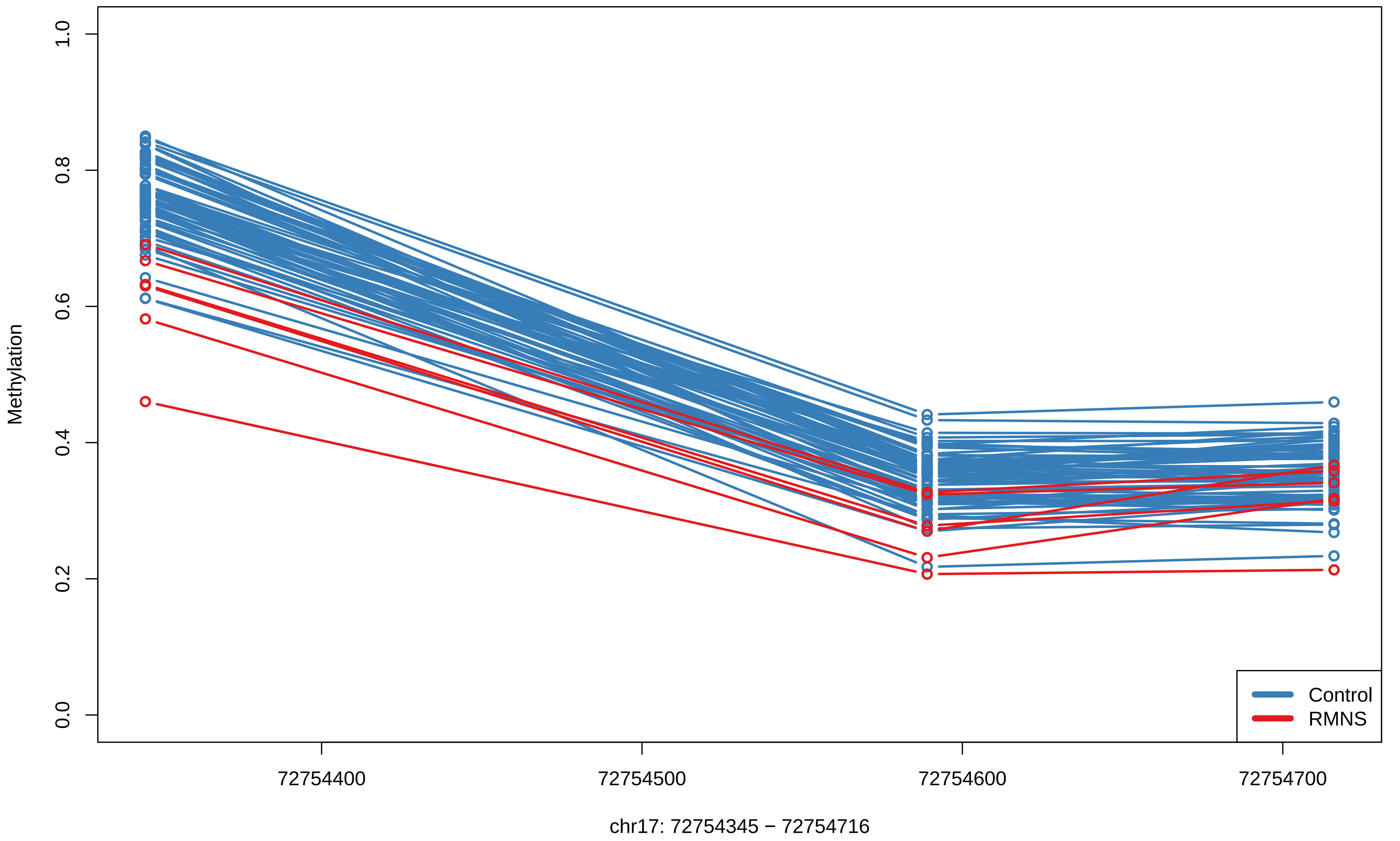

Methylation
0.0
0.2
0.4
0.6
0.8
1.0
72754400
72754500
72754600
72754700
chr17: 72754345 − 72754716
Control
RMNS

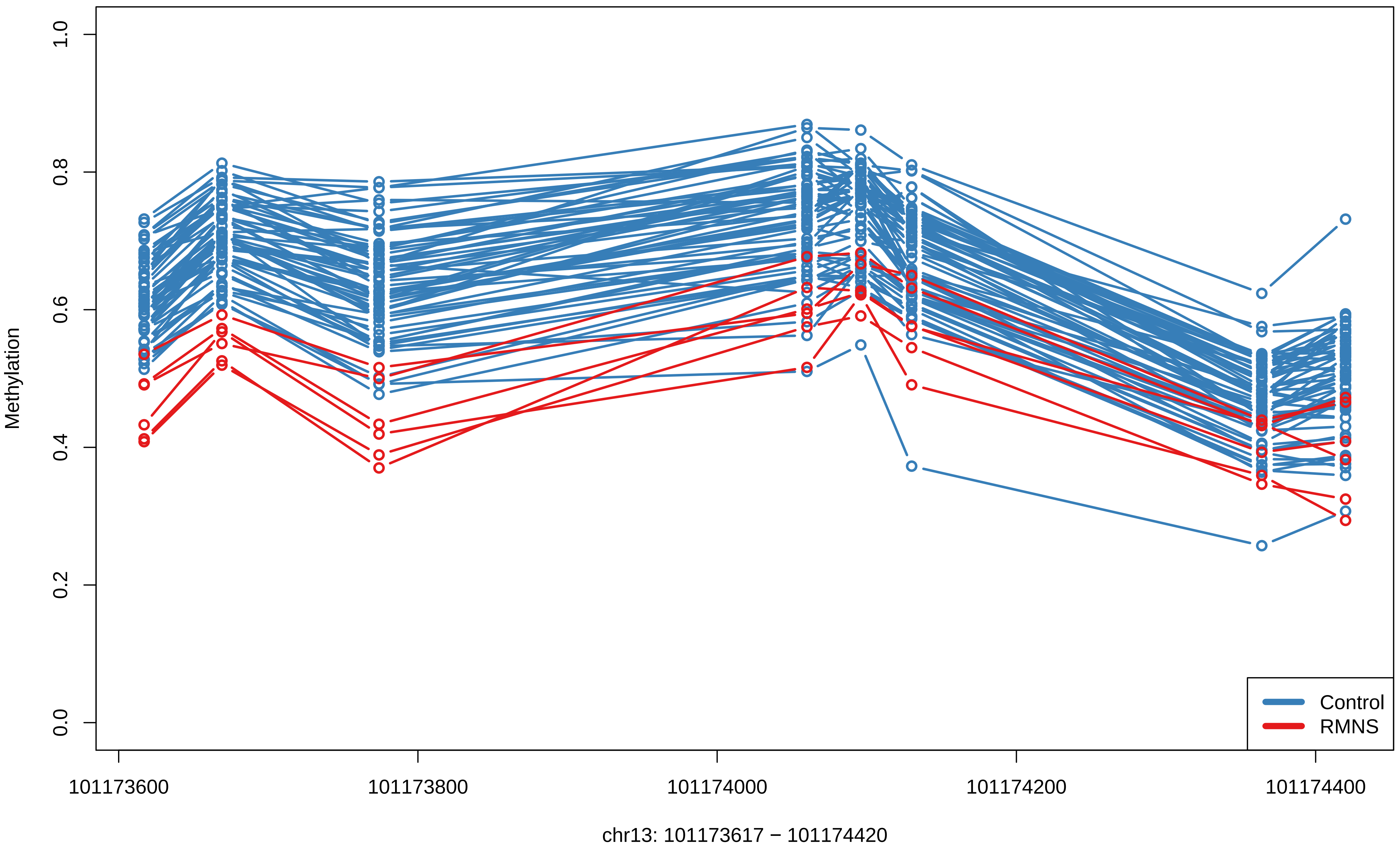

Methylation
0.0
0.2
0.4
0.6
0.8
1.0
101173600
101173800
101174000
101174200
101174400
chr13: 101173617 − 101174420
Control
RMNS

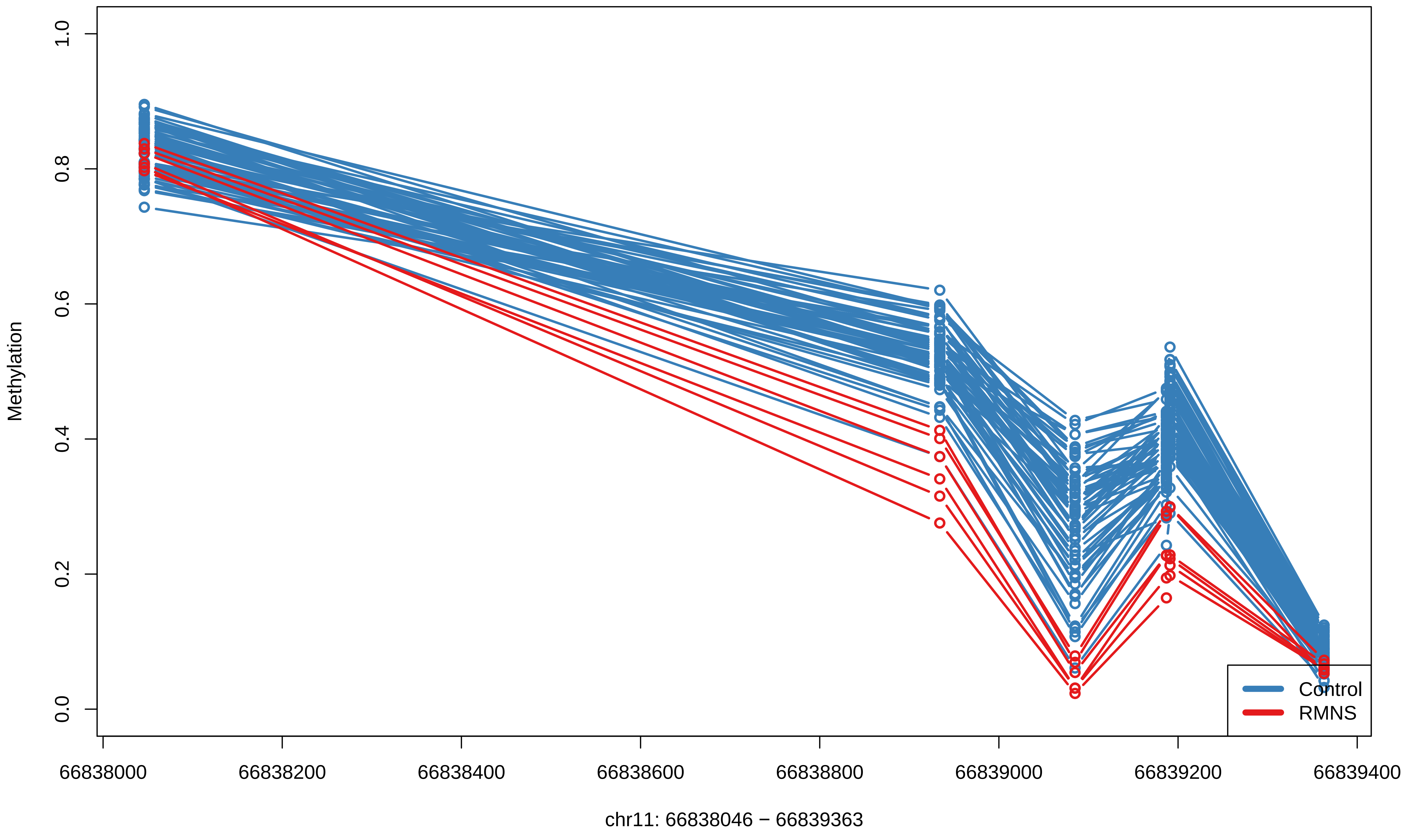

Methylation
1.0
0.8
0.6
0.4
0.2
0.0
66838000
66838200
66838400
66838600
66838800
66839000
66839200
66839400
chr11: 66838046 − 66839363
Control
RMNS

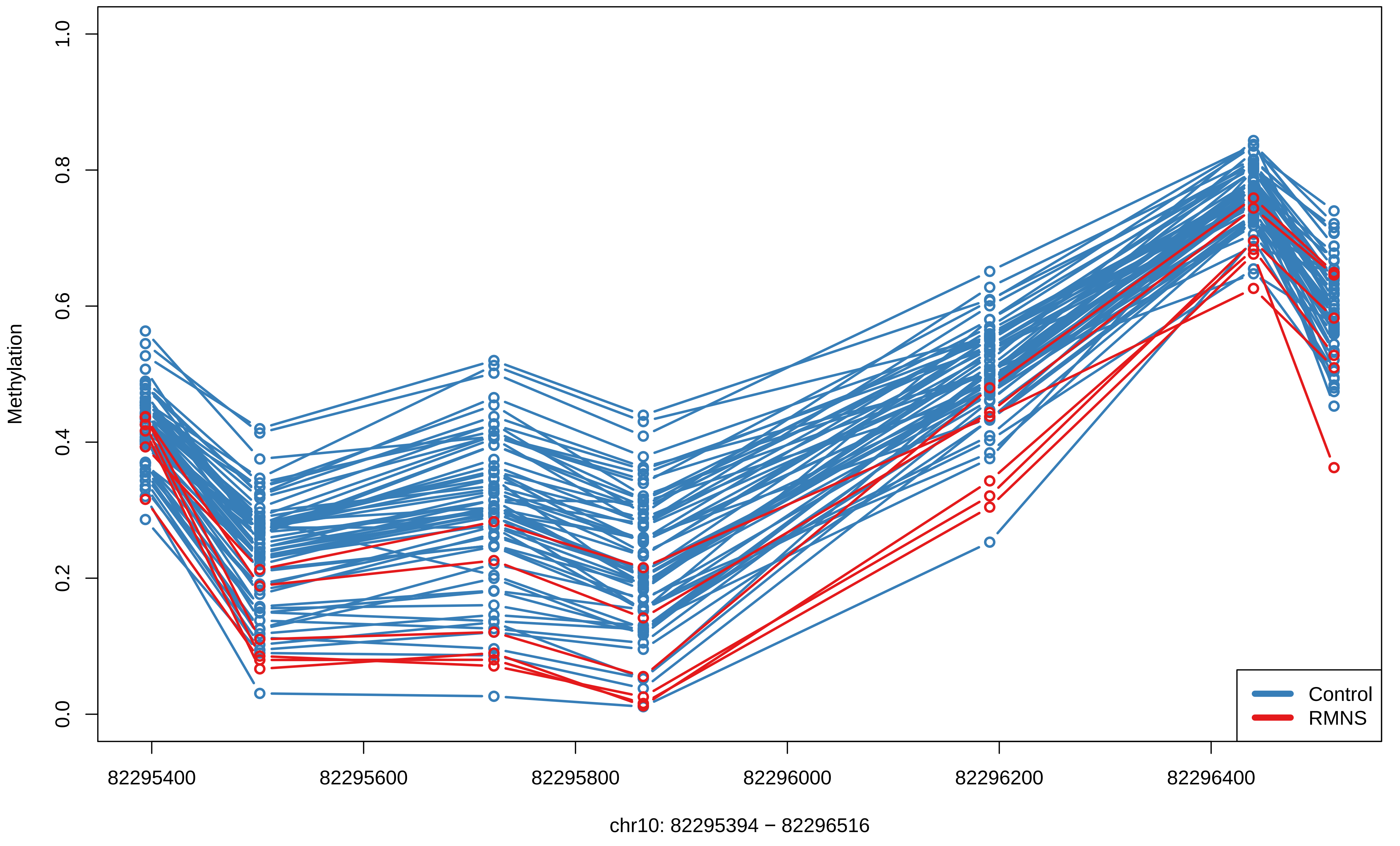

Methylation
0.0
0.2
0.4
0.6
0.8
1.0
82295400
82295600
82295800
82296000
82296200
82296400
chr10: 82295394 − 82296516
Control
RMNS

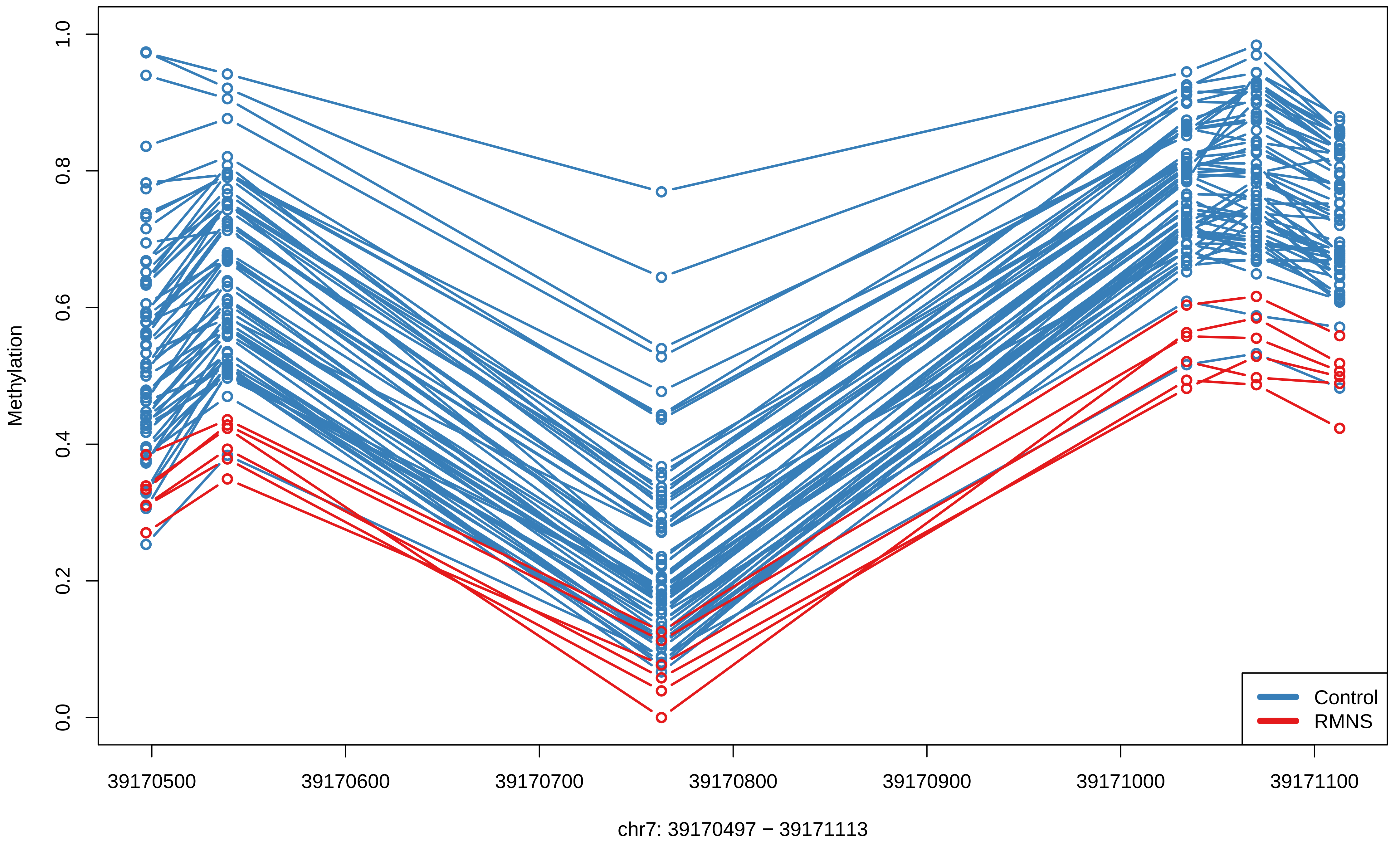

Methylation
0.0
0.2
0.4
0.6
0.8
1.0
39170500
39170600
39170700
39170800
39170900
39171000
39171100
chr7: 39170497 − 39171113
Control
RMNS

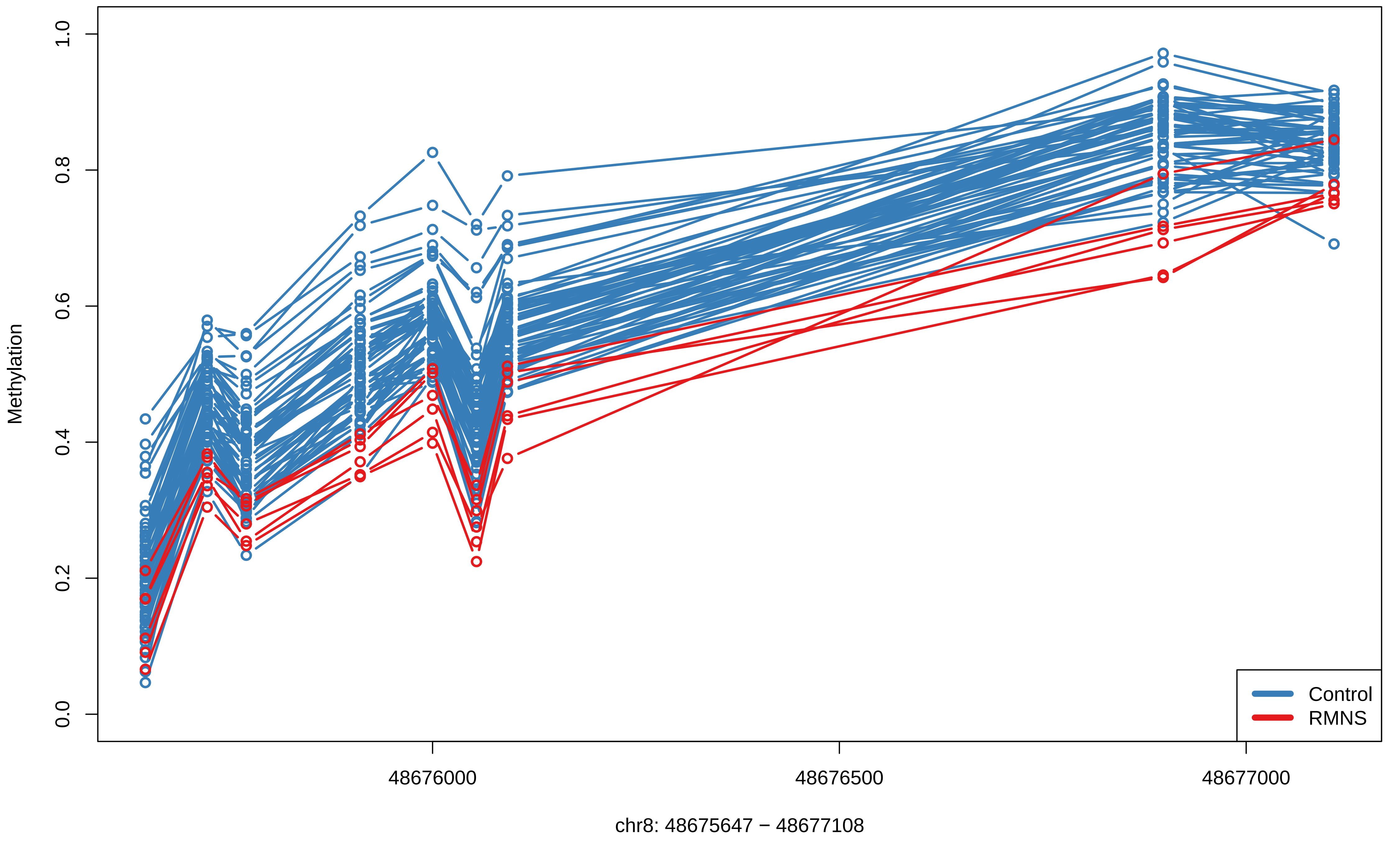

Methylation
1.0
0.8
0.6
0.4
0.2
0.0
48676000
48676500
48677000
chr8: 48675647 − 48677108
Control
RMNS

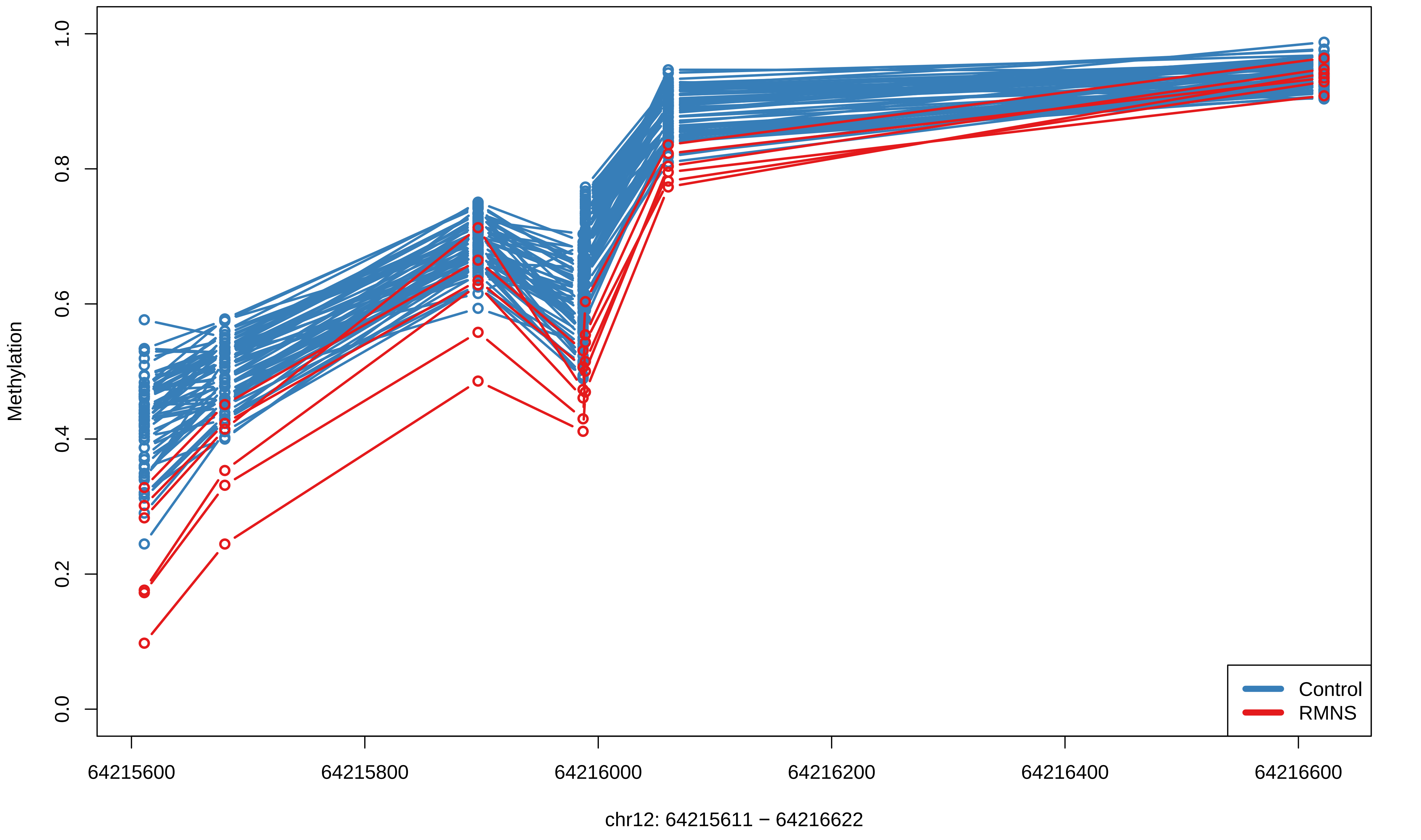

Methylation
0.0
0.2
0.4
0.6
0.8
1.0
64215600
64215800
64216000
64216200
64216400
64216600
chr12: 64215611 − 64216622
Control
RMNS

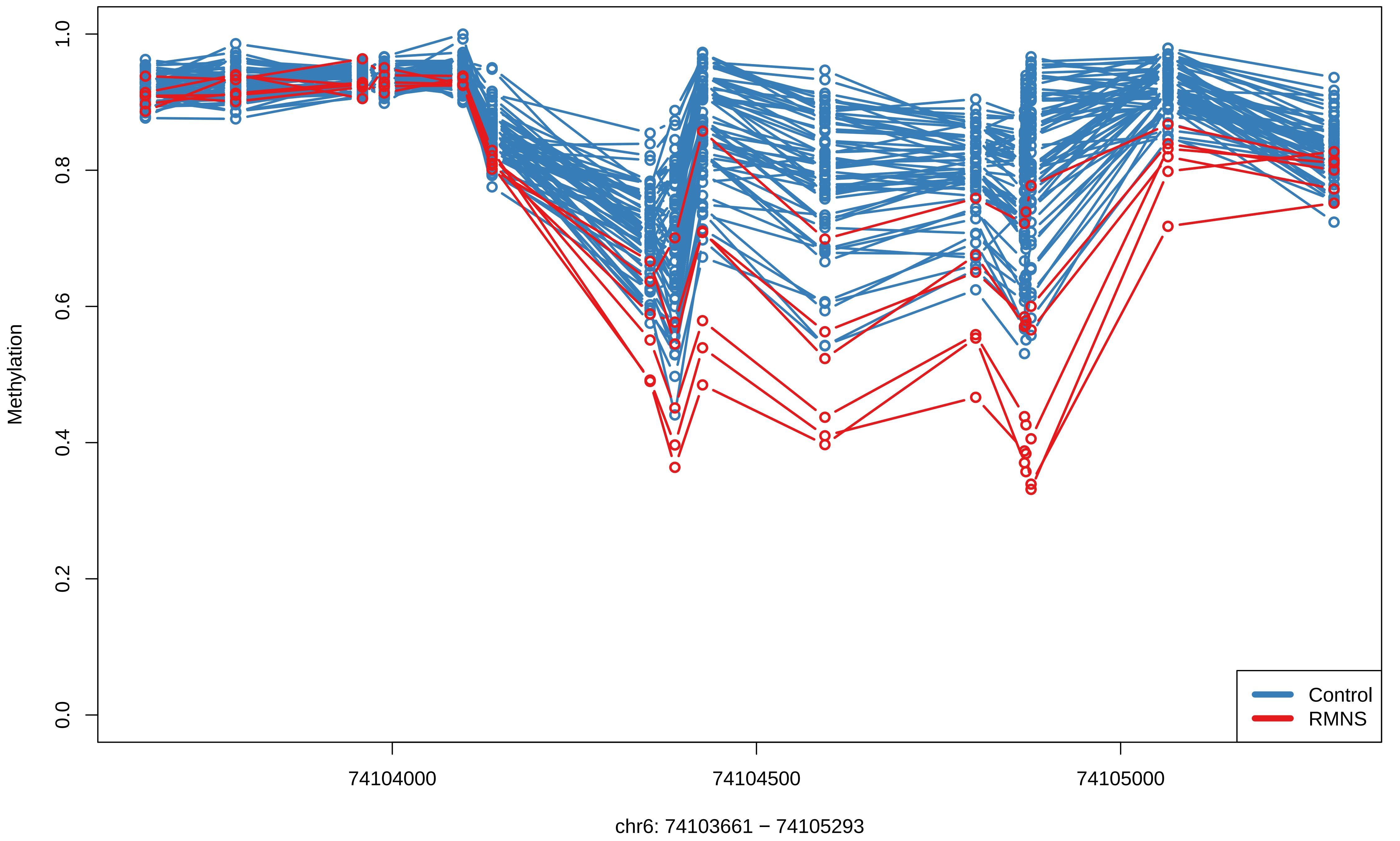
Methylation
0.0
0.2
0.4
0.6
0.8
1.0
74104000
74104500
74105000
chr6: 74103661 − 74105293
Control
RMNS

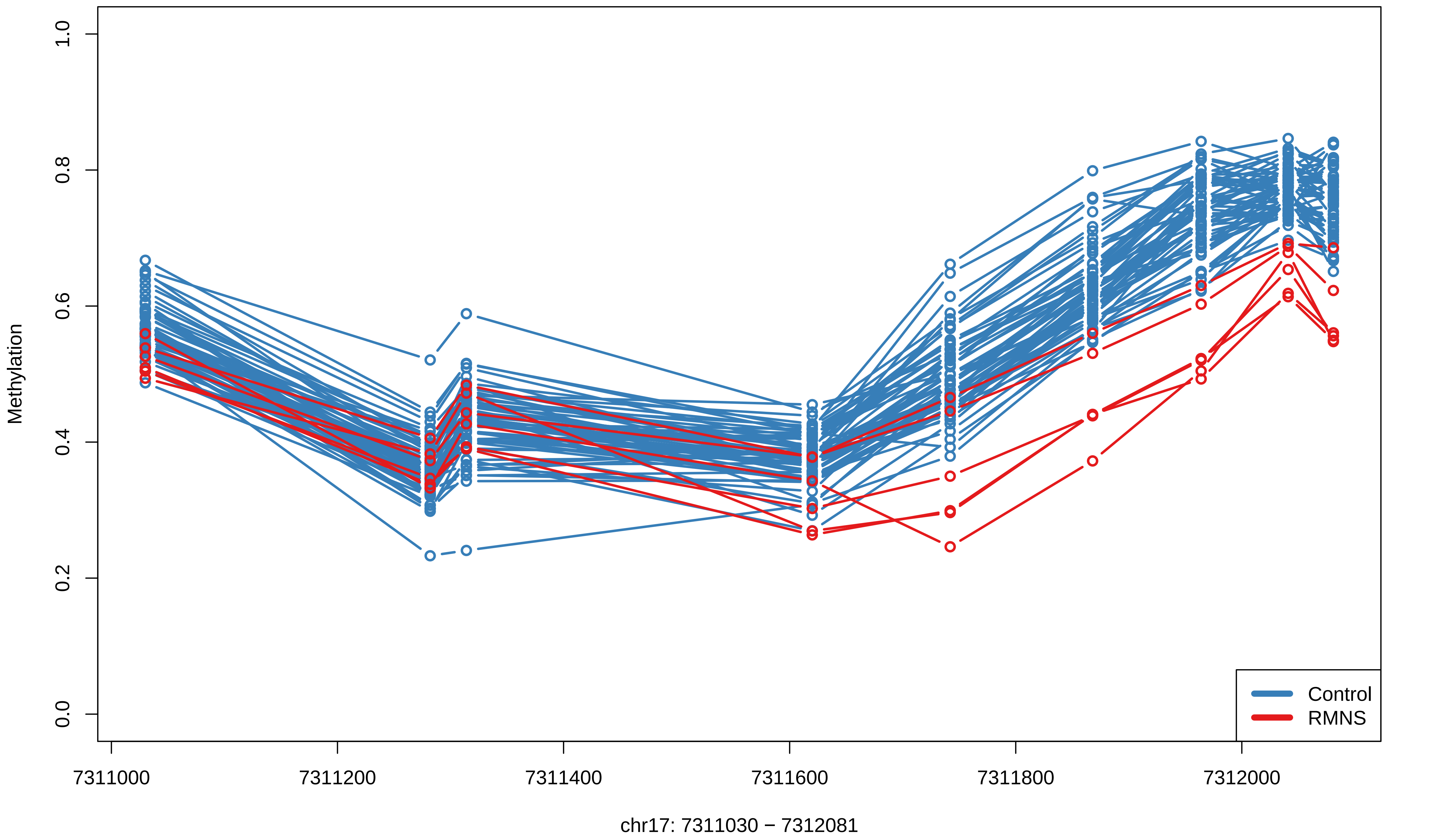

Methylation
0.0
0.2
0.4
0.6
0.8
1.0
7311000
7311200
7311400
7311600
7311800
7312000
chr17: 7311030 − 7312081
Control
RMNS

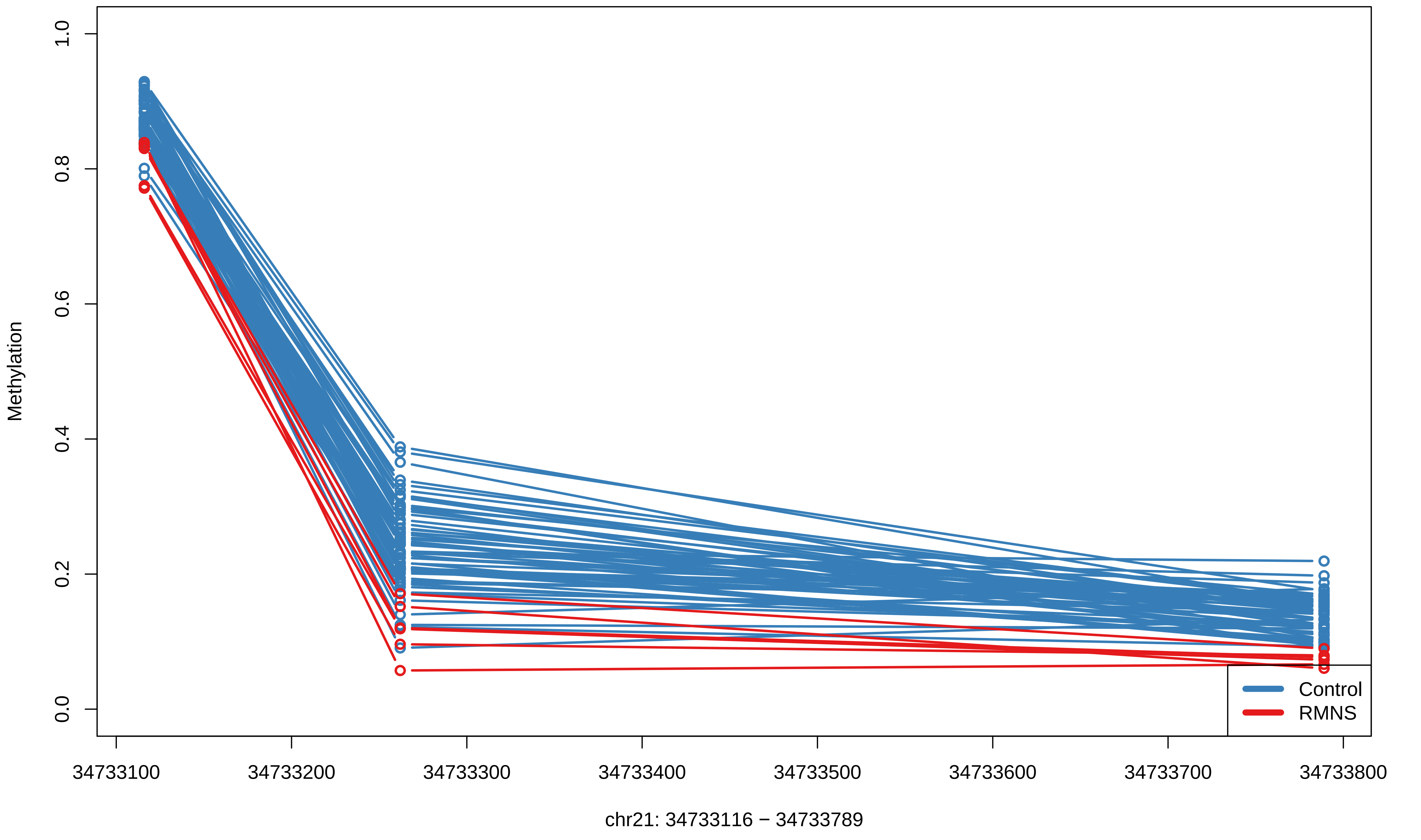

Methylation
0.0
0.2
0.4
0.6
0.8
1.0
34733100
34733200
34733300
34733400
34733500
34733600
34733700
34733800
chr21: 34733116 − 34733789
Control
RMNS

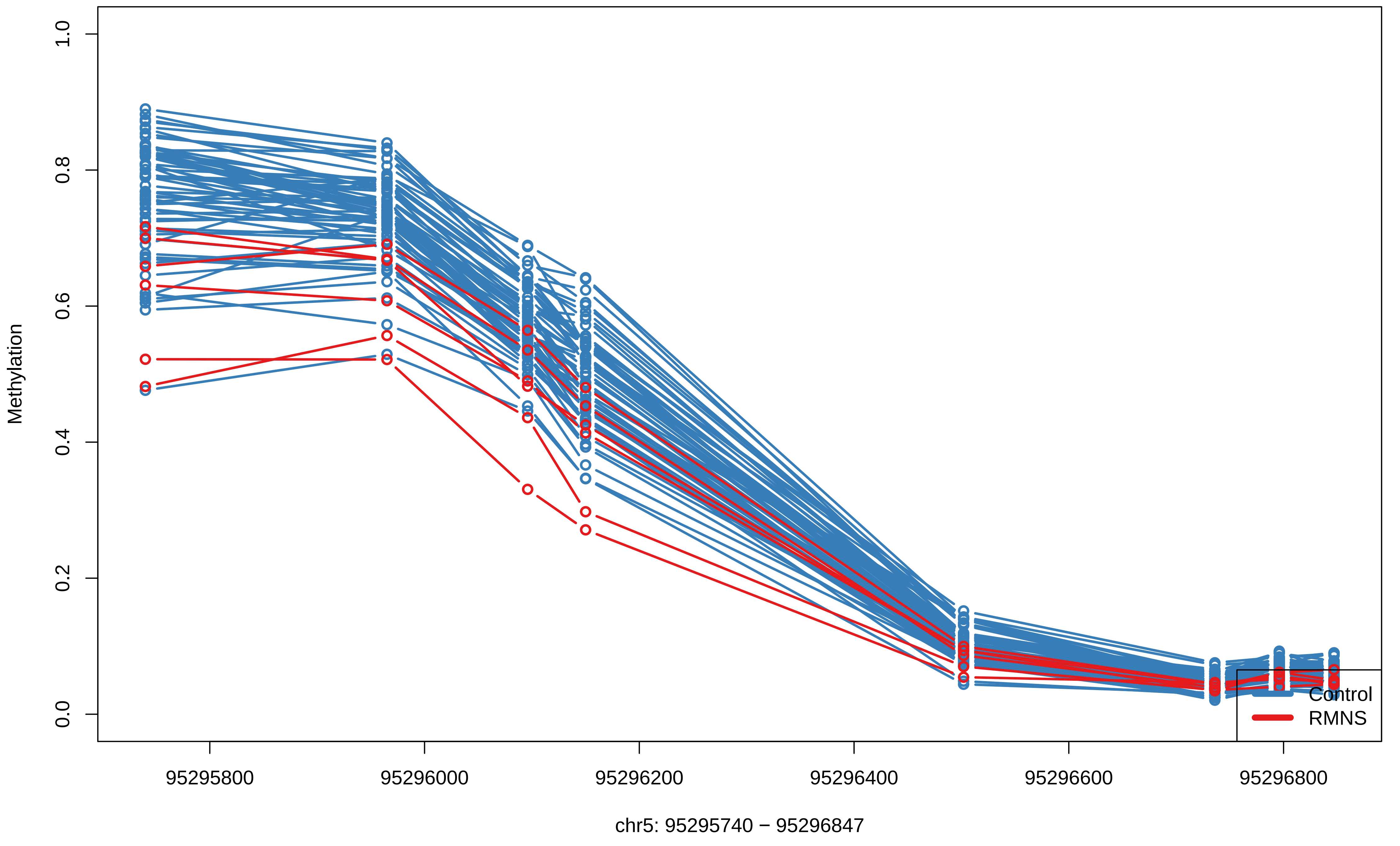

Methylation
0.0
0.2
0.4
0.6
0.8
1.0
95295800
95296000
95296200
95296400
95296600
95296800
chr5: 95295740 − 95296847
Control
RMNS

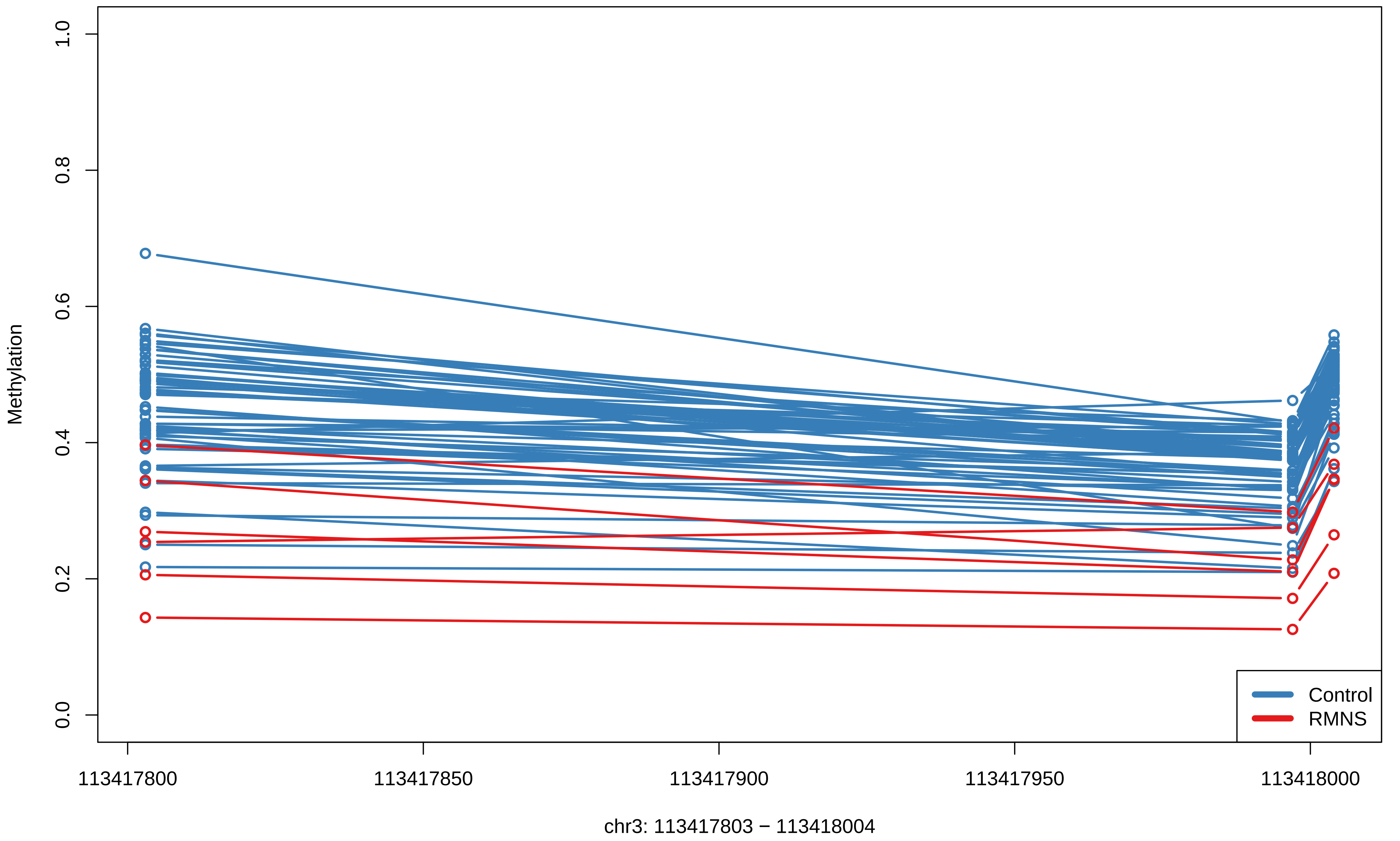

Methylation
0.0
0.2
0.4
0.6
0.8
1.0
113417800
113417850
113417900
113417950
113418000
chr3: 113417803 − 113418004
Control
RMNS

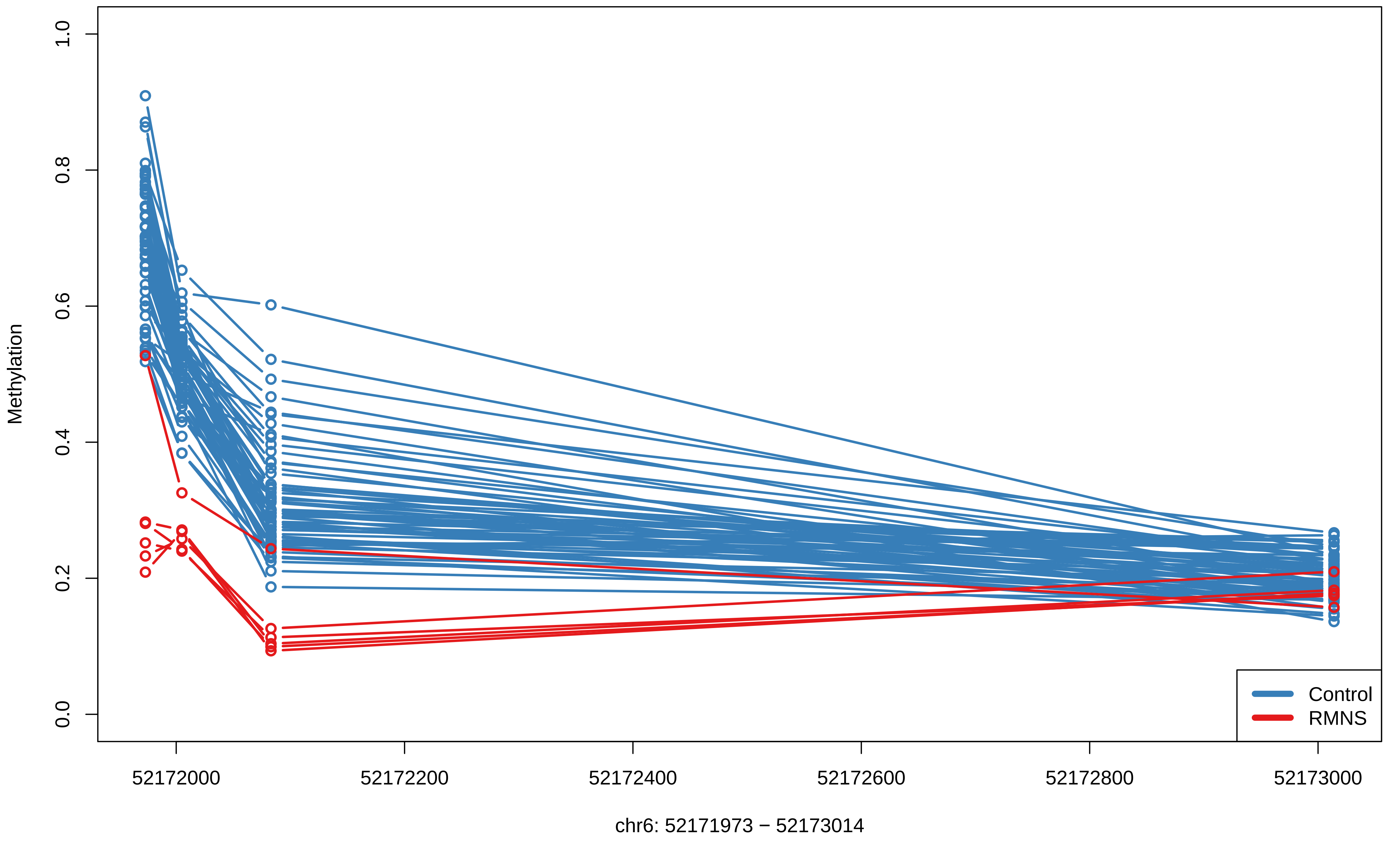

Methylation
1.0
0.8
0.6
0.4
0.2
0.0
52172000
52172200
52172400
52172600
52172800
52173000
chr6: 52171973 − 52173014
Control
RMNS

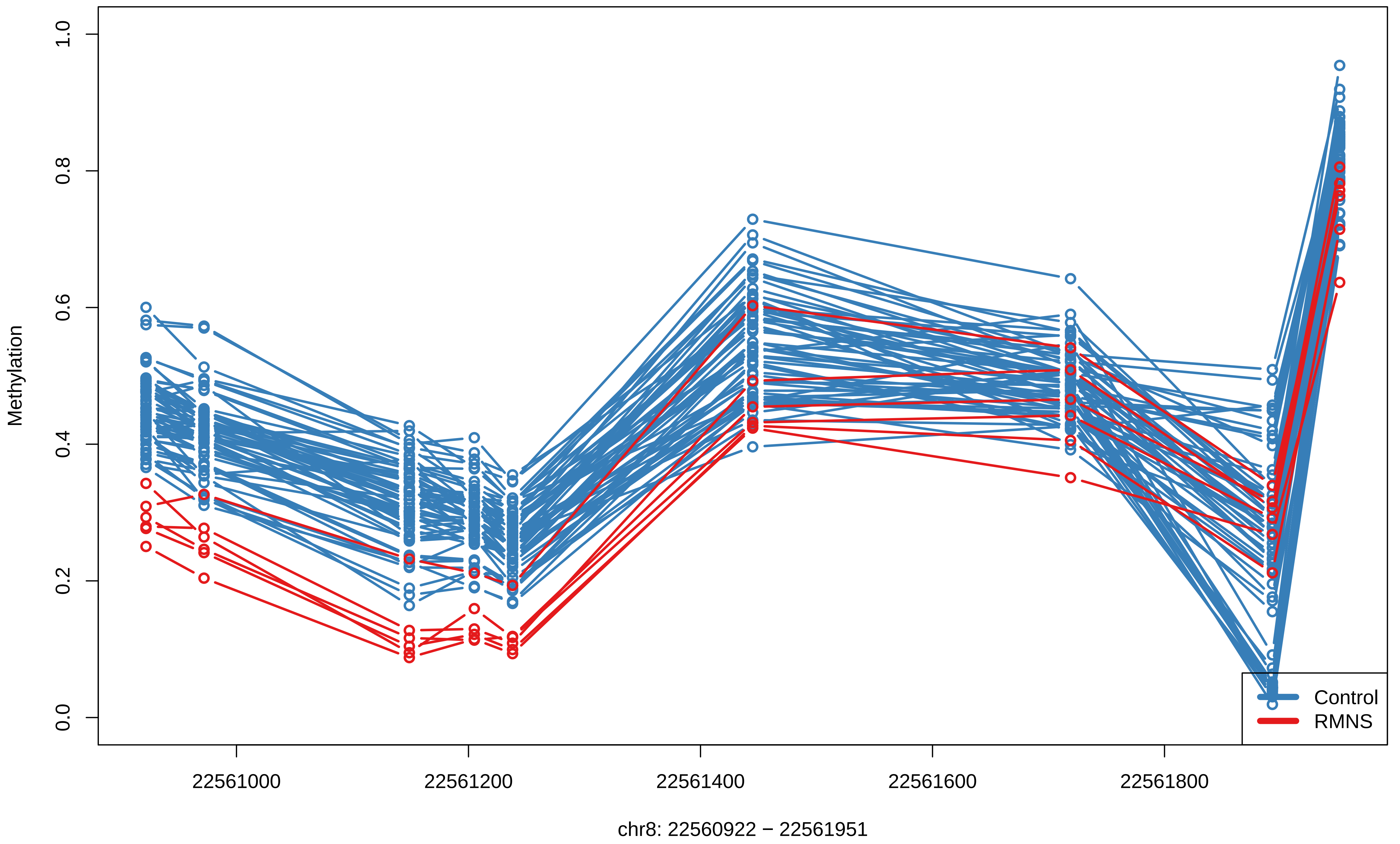

Methylation
1.0
0.8
0.6
0.4
0.2
0.0
22561000
22561200
22561400
22561600
22561800
chr8: 22560922 − 22561951
Control
RMNS

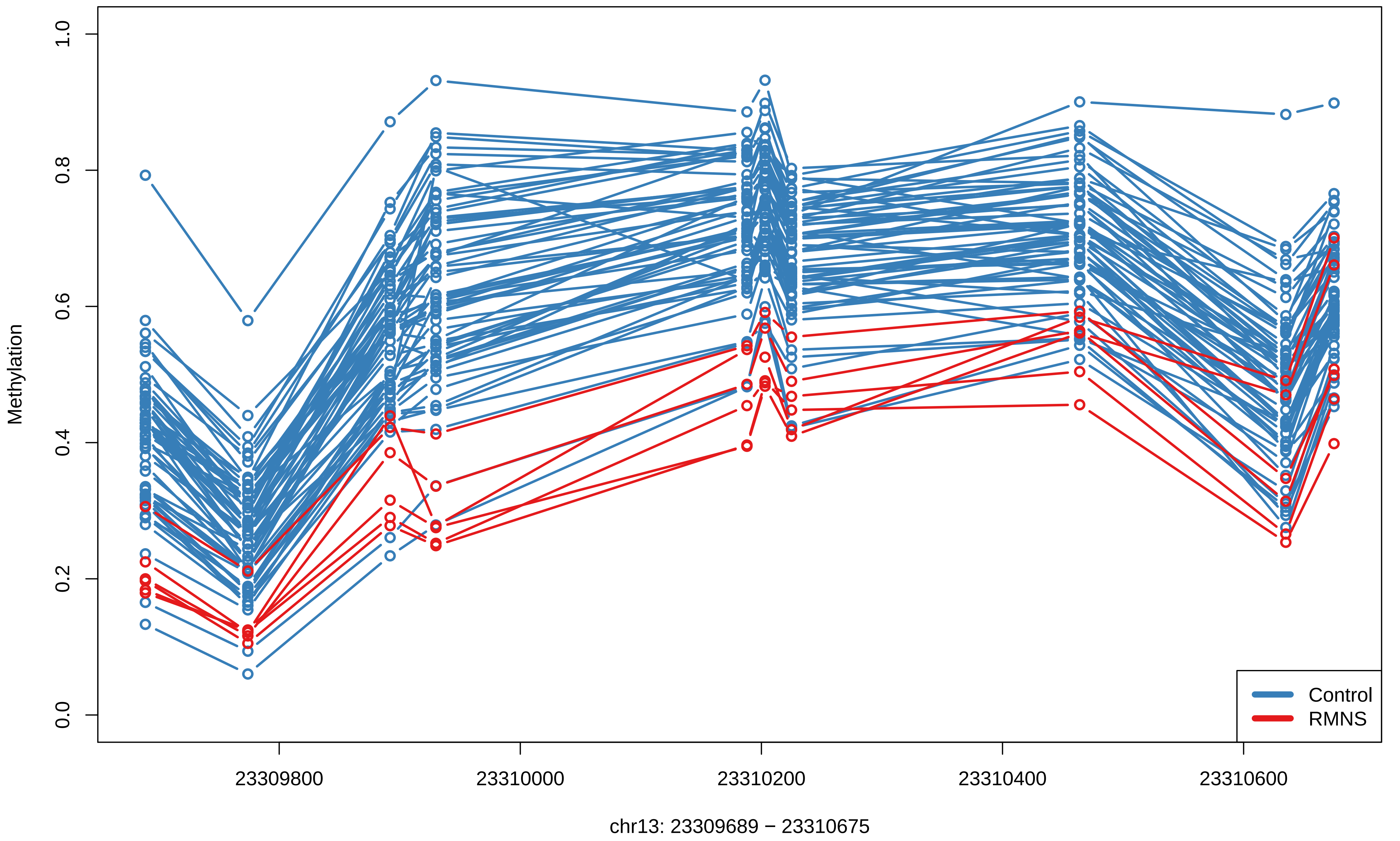

1.0
0.8
0.6
0.4
0.2
0.0
Methylation
23309800
23310000
23310200
23310400
23310600
chr13: 23309689 − 23310675
Control
RMNS

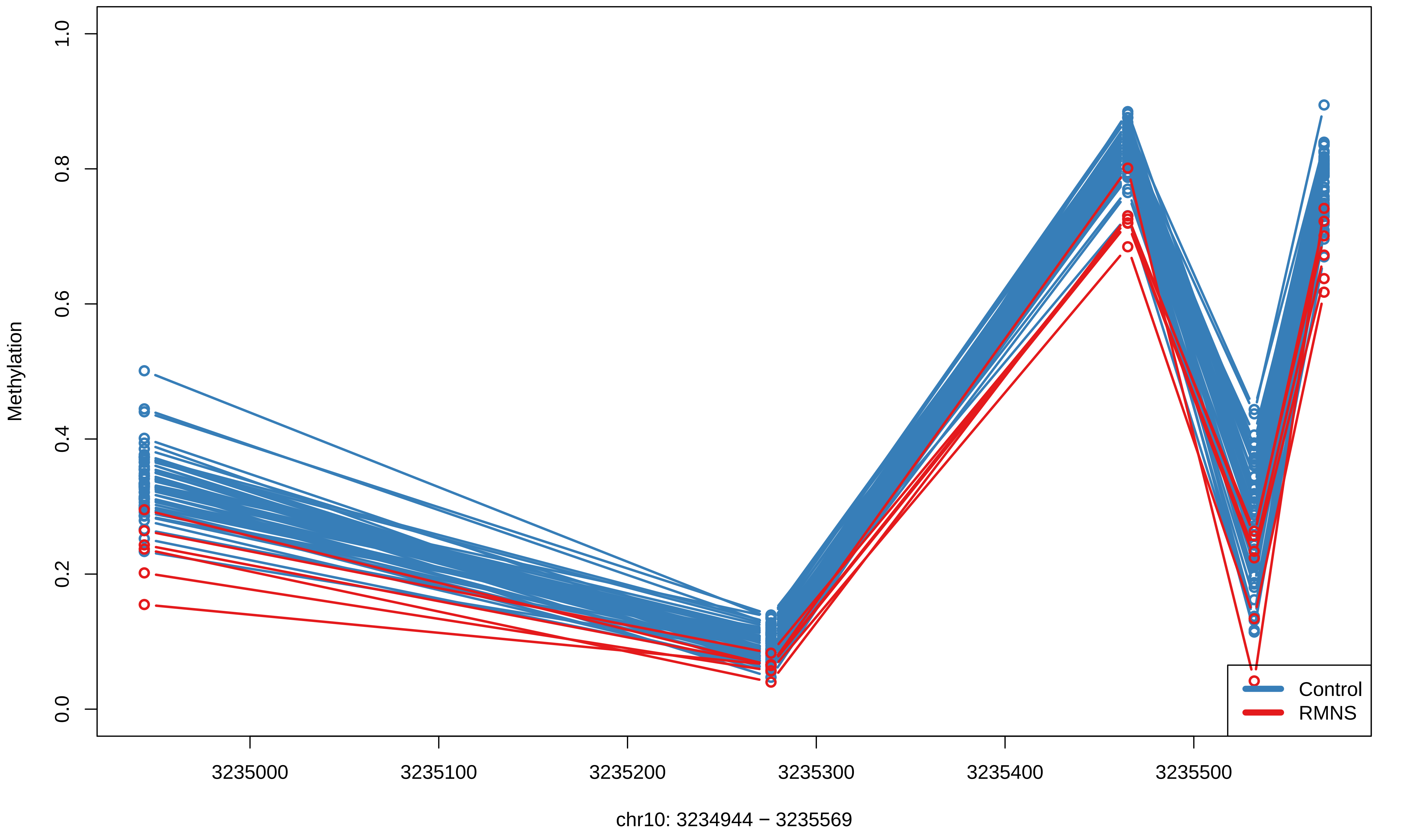

Methylation
1.0
0.8
0.6
0.4
0.2
0.0
3235000
3235100
3235200
3235300
3235400
3235500
chr10: 3234944 − 3235569
Control
RMNS

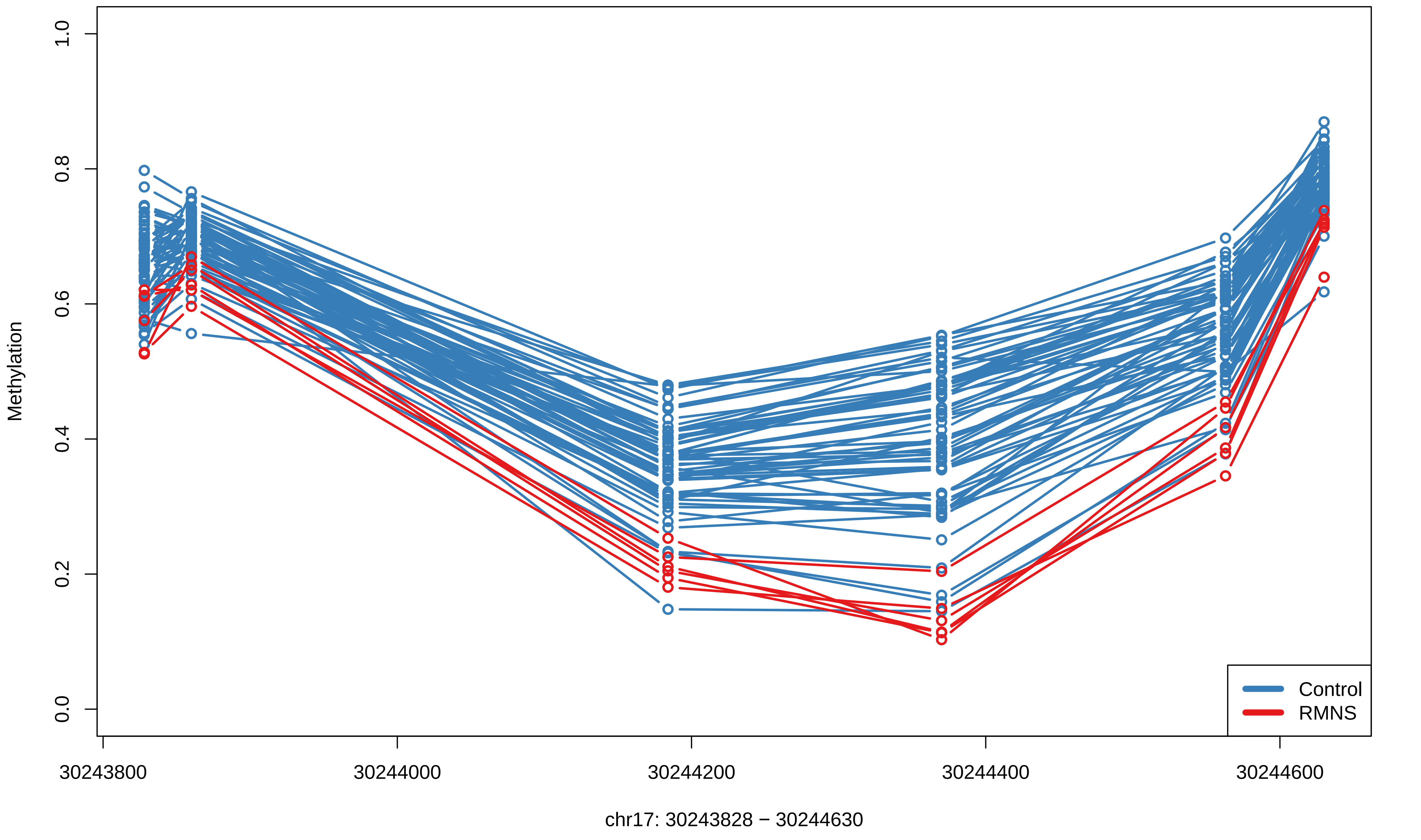

Methylation
0.0
0.2
0.4
0.6
0.8
1.0
30243800
30244000
30244200
30244400
30244600
chr17: 30243828 − 30244630
Control
RMNS

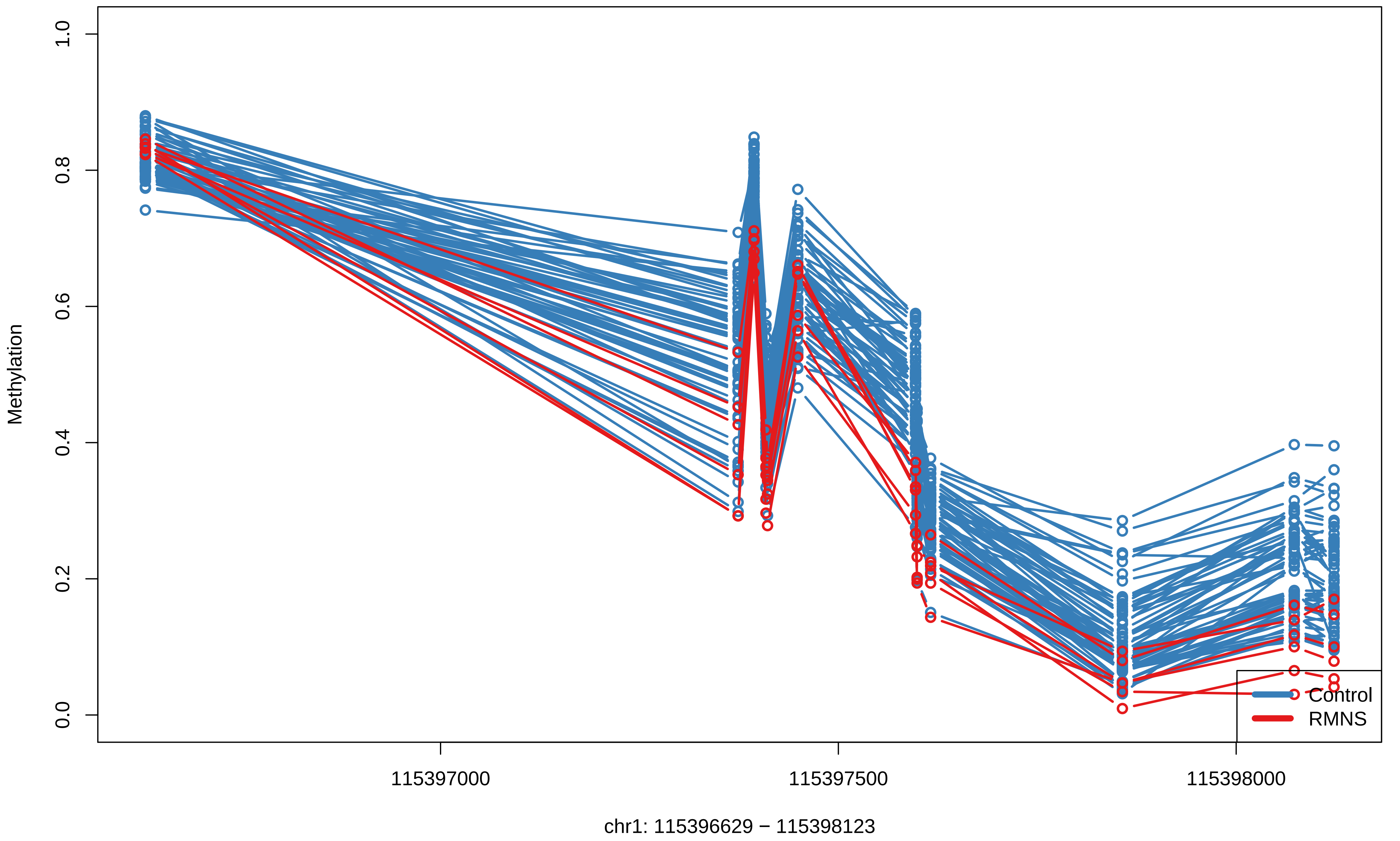

Methylation
1.0
0.8
0.6
0.4
0.2
0.0
115397000
115397500
115398000
chr1: 115396629 − 115398123
Control
RMNS

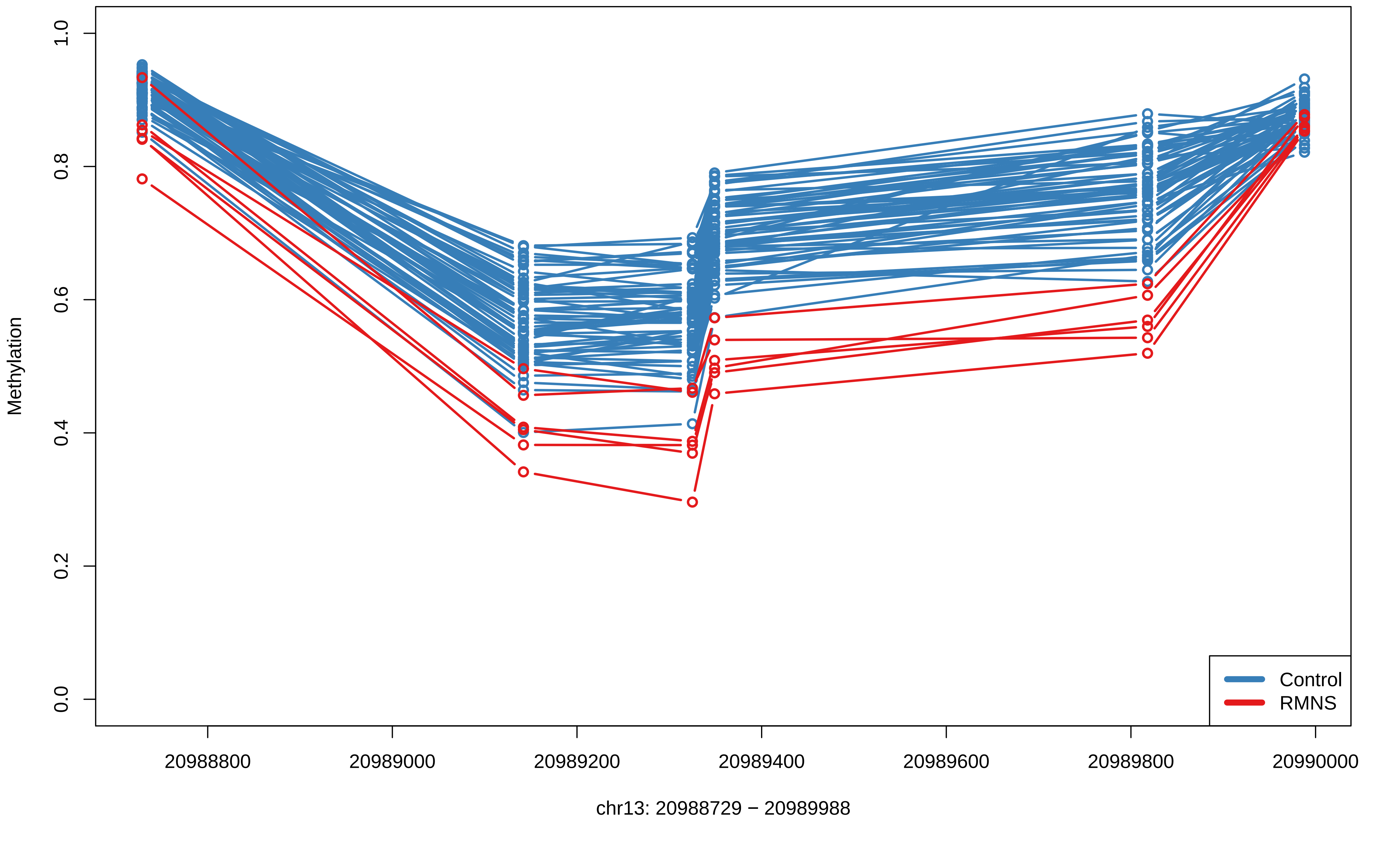

Methylation
0.0
0.2
0.4
0.6
0.8
1.0
20988800
20989000
20989200
20989400
20989600
20989800
20990000
chr13: 20988729 − 20989988
Control
RMNS

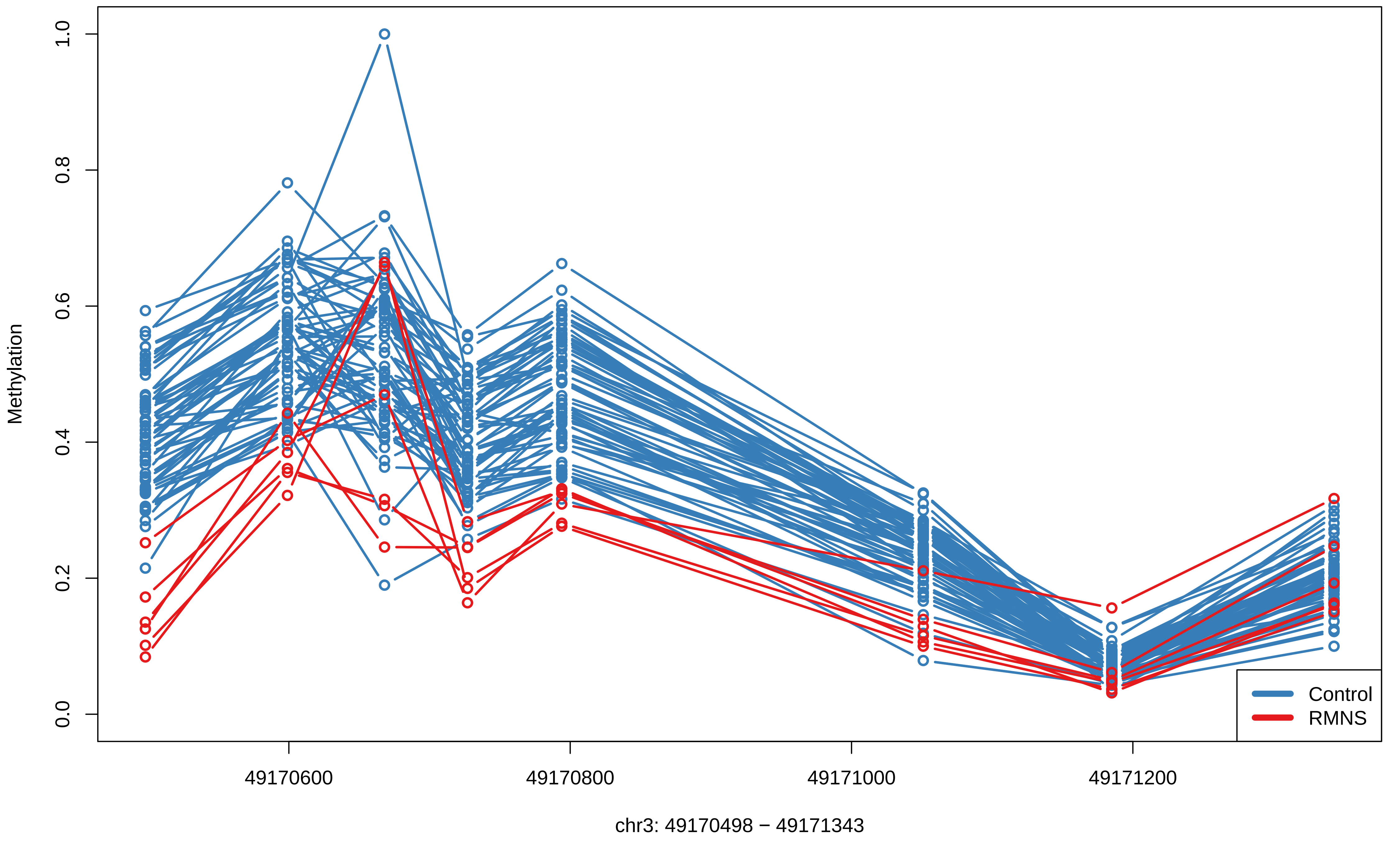

Methylation
0.0
0.2
0.4
0.6
0.8
1.0
49170600
49170800
49171000
49171200
chr3: 49170498 − 49171343
Control
RMNS

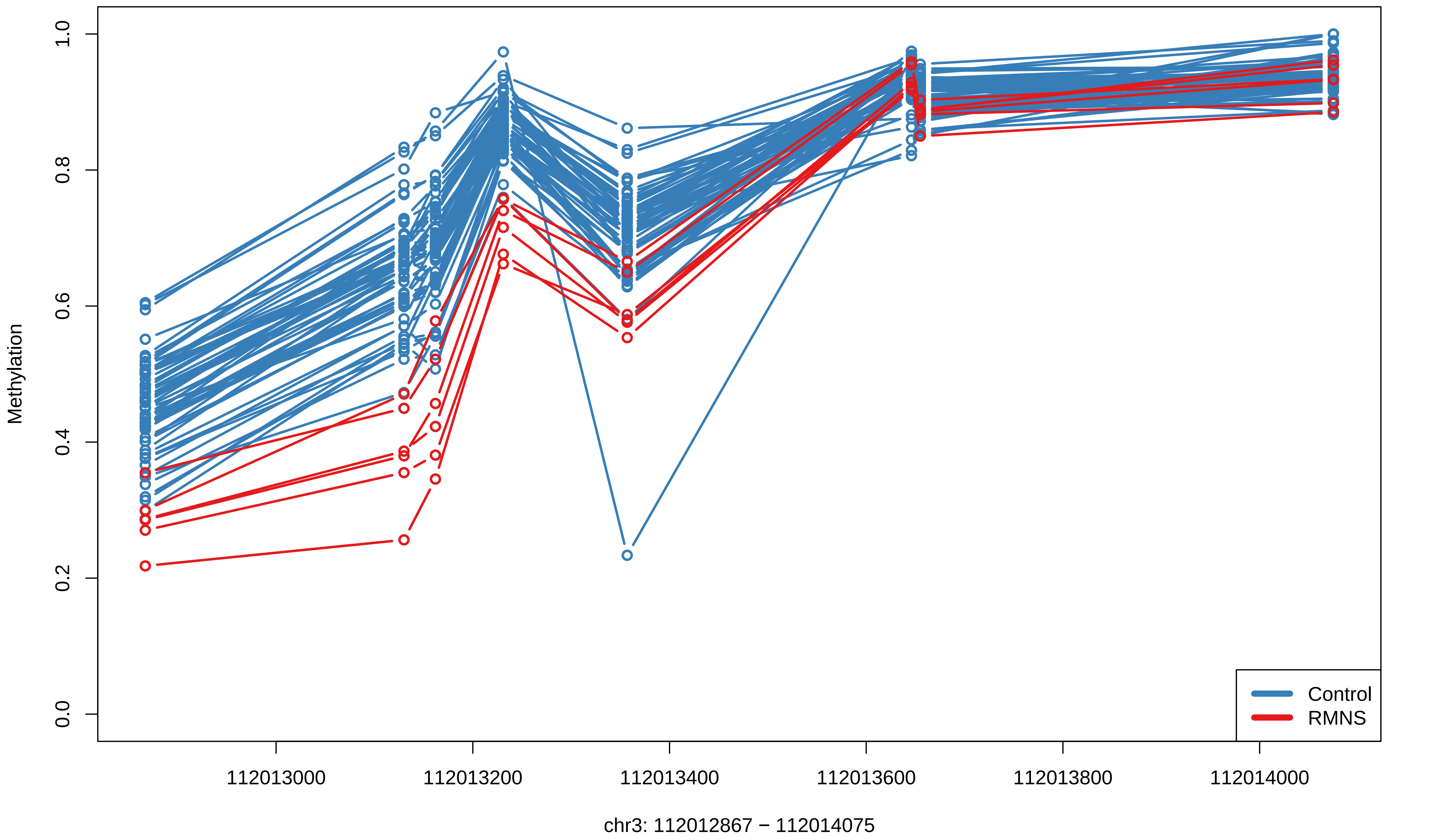

Methylation
0.0
0.2
0.4
0.6
0.8
1.0
112013000
112013200
112013400
112013600
112013800
112014000
chr3: 112012867 − 112014075
Control
RMNS

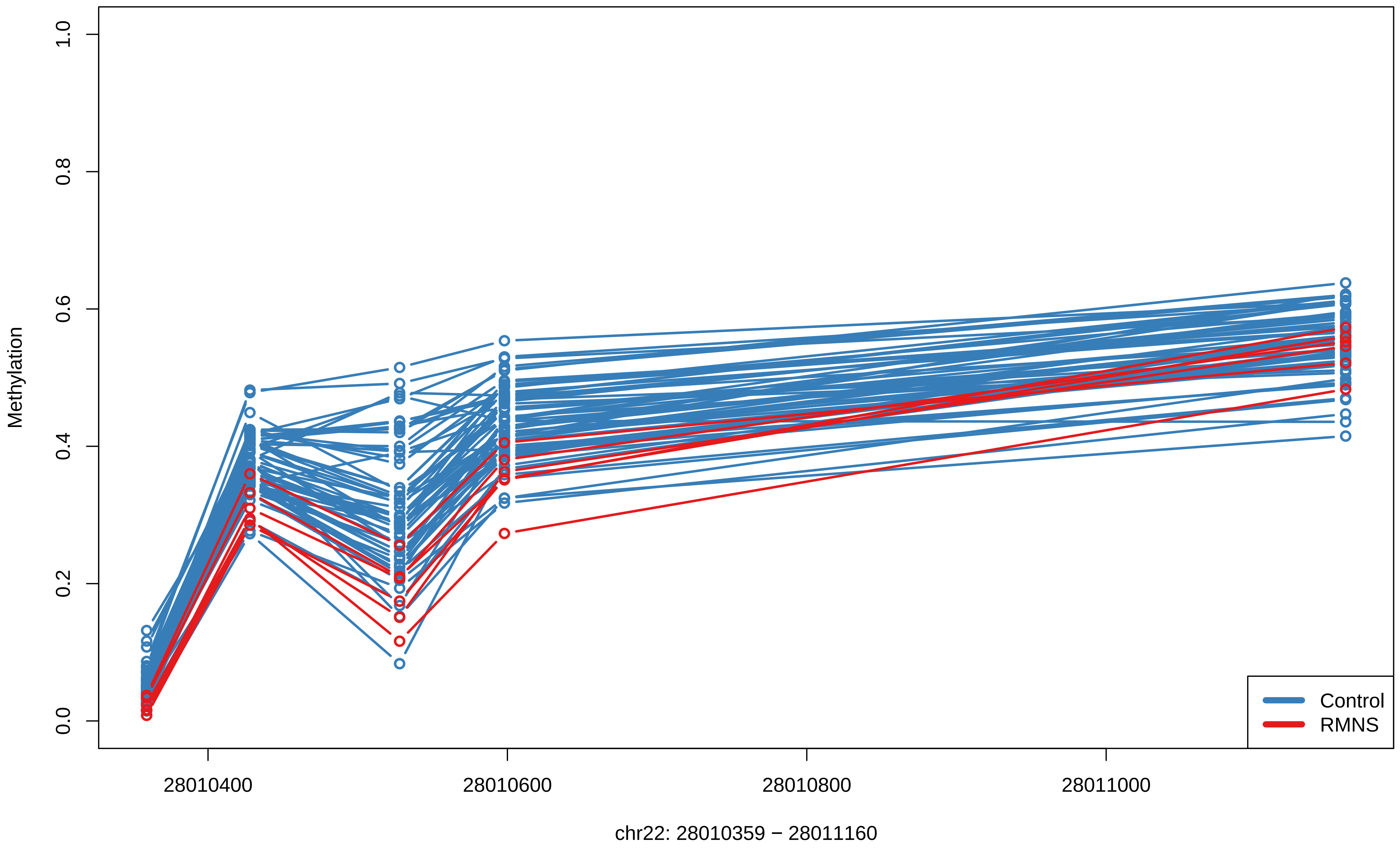

Methylation
0.0
0.2
0.4
0.6
0.8
1.0
28010400
28010600
28010800
28011000
chr22: 28010359 − 28011160
Control
RMNS

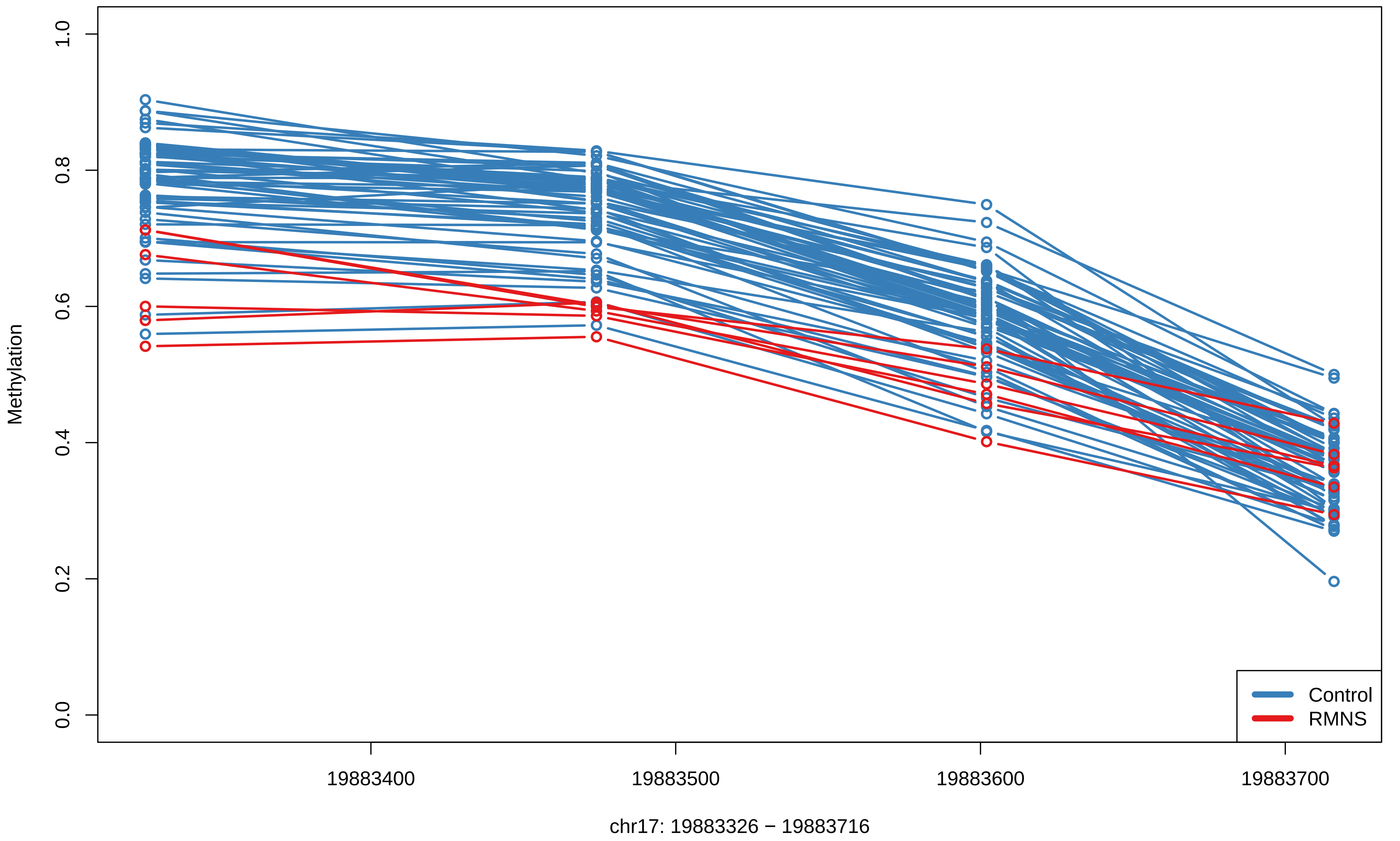

Methylation
1.0
0.8
0.6
0.4
0.2
0.0
19883400
19883500
19883600
19883700
chr17: 19883326 − 19883716
Control
RMNS

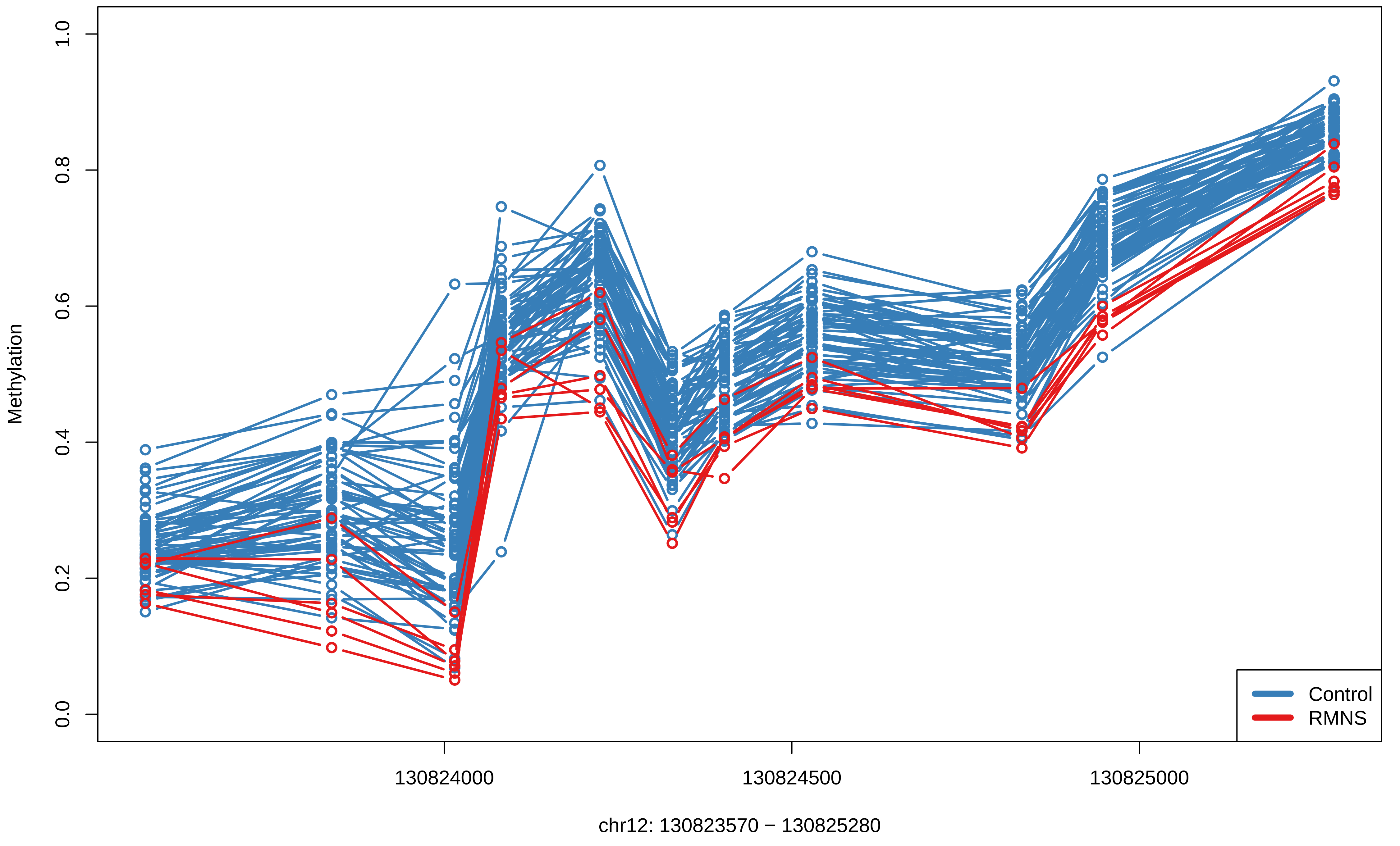

Methylation
1.0
0.8
0.6
0.4
0.2
0.0
130824000
130824500
130825000
chr12: 130823570 − 130825280
Control
RMNS

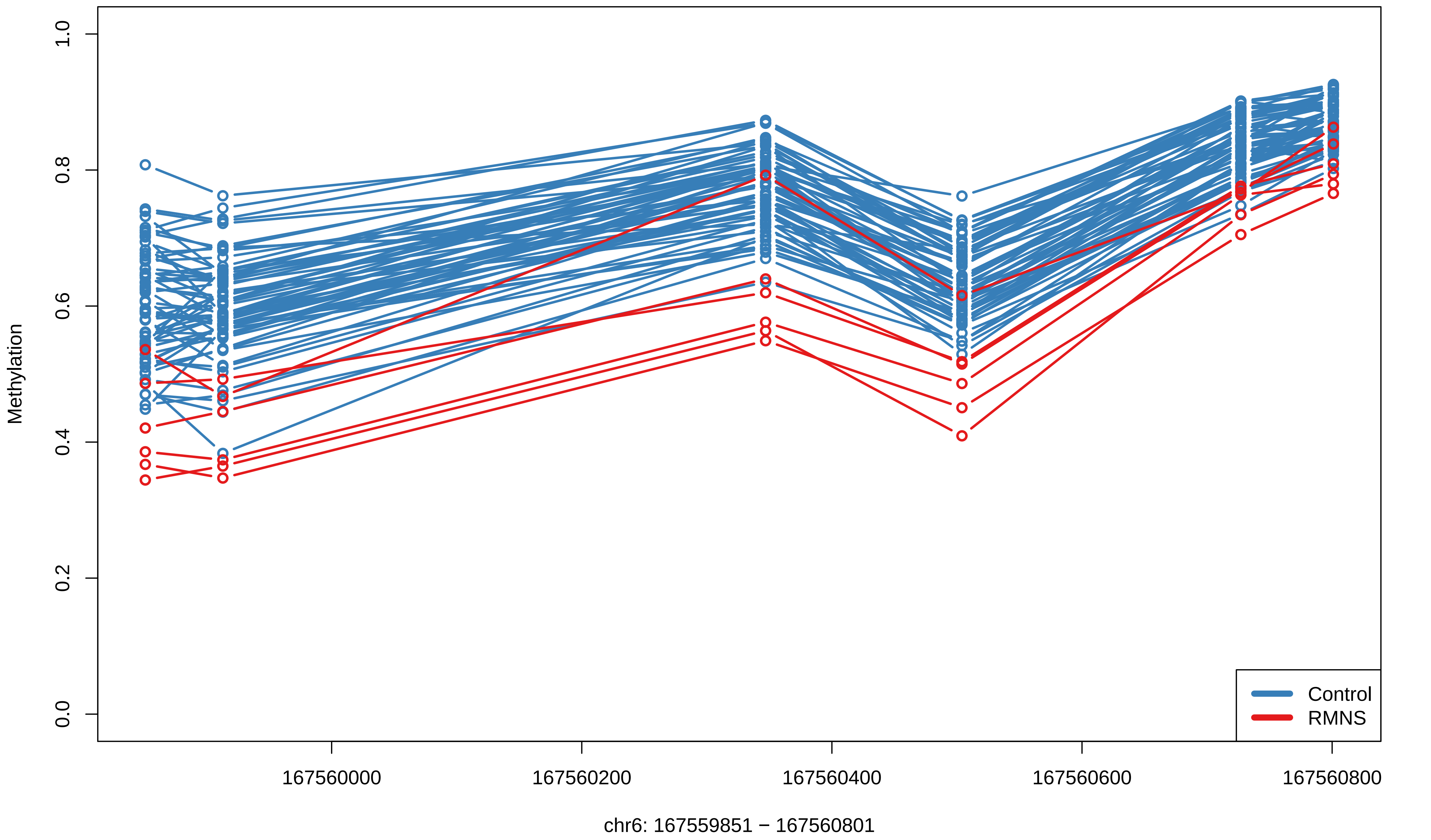

Methylation
0.0
0.2
0.4
0.6
0.8
1.0
167560000
167560200
167560400
167560600
167560800
chr6: 167559851 − 167560801
Control
RMNS

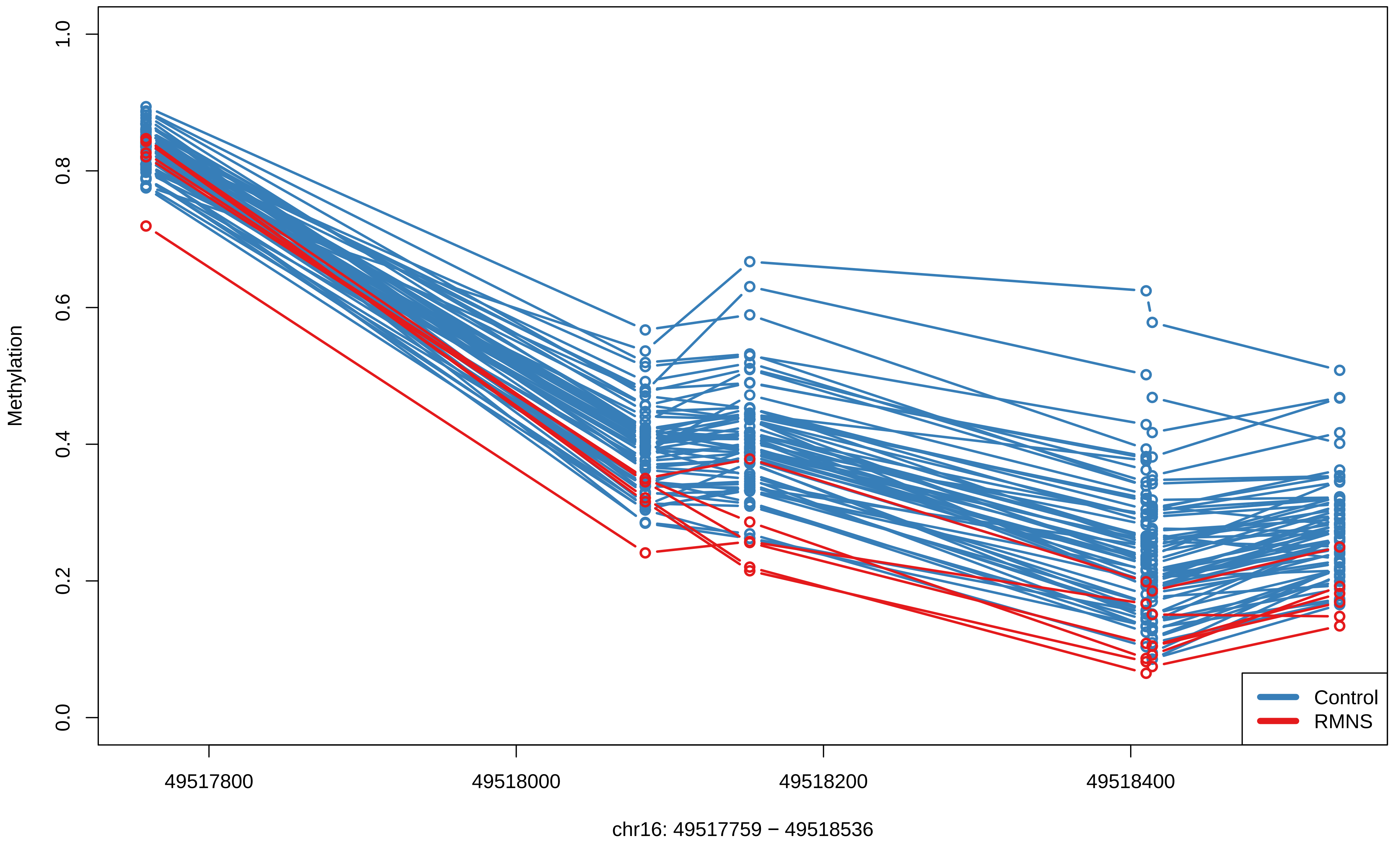

Methylation
1.0
0.8
0.6
0.4
0.2
0.0
49517800
49518000
49518200
49518400
chr16: 49517759 − 49518536
Control
RMNS

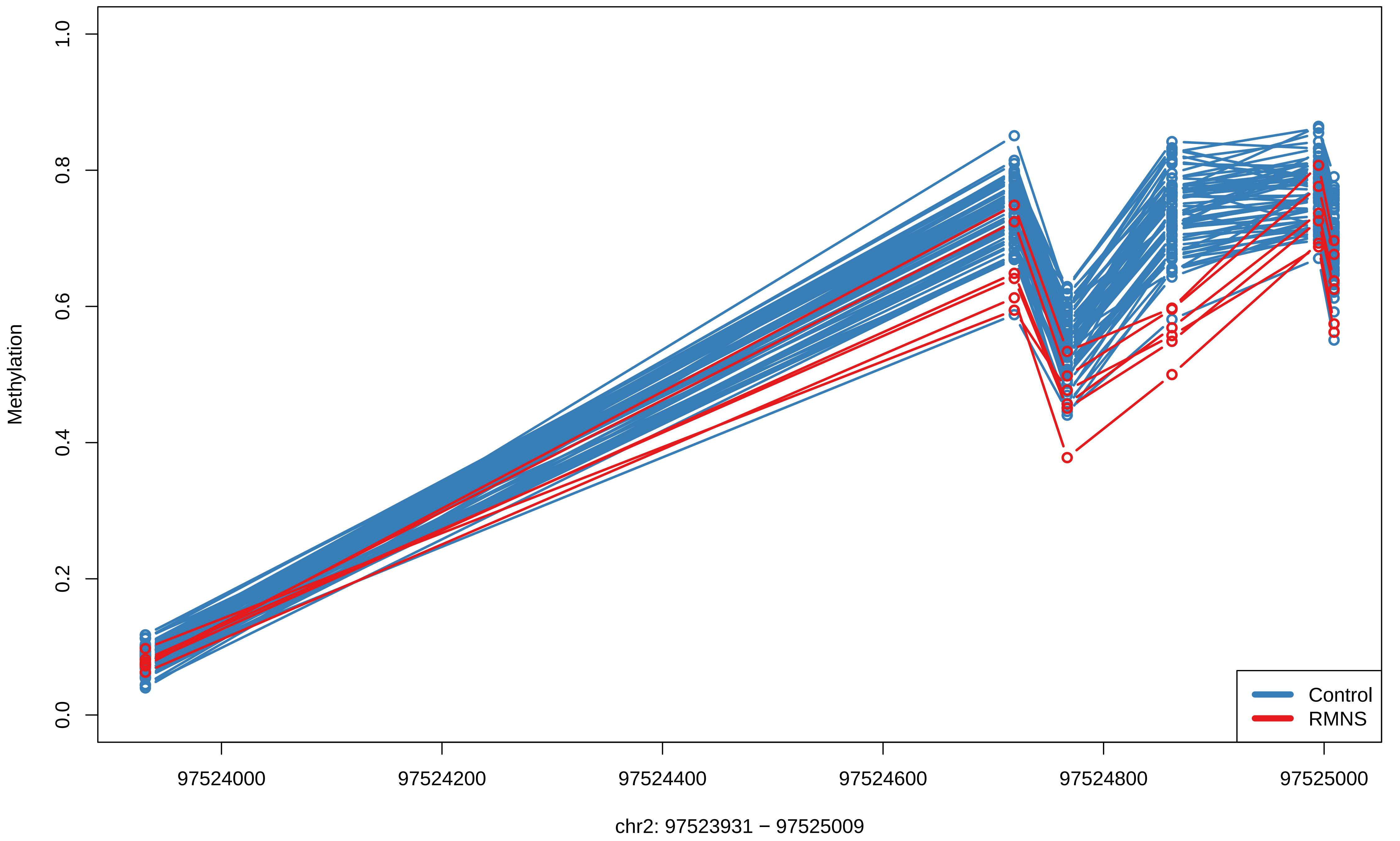

Methylation
0.0
0.2
0.4
0.6
0.8
1.0
97524000
97524200
97524400
97524600
97524800
97525000
chr2: 97523931 − 97525009
Control
RMNS

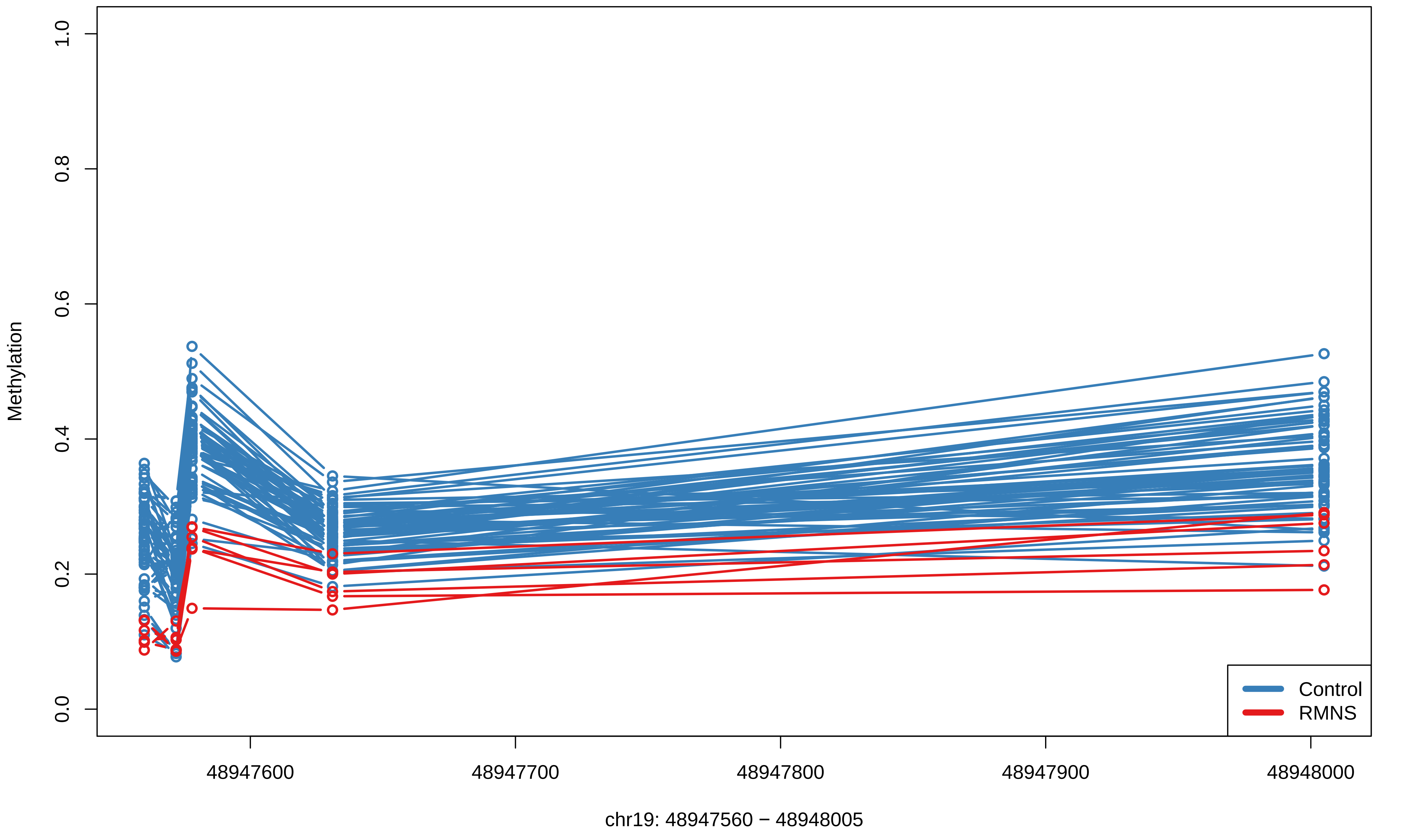

Methylation
0.0
0.2
0.4
0.6
0.8
1.0
48947600
48947700
48947800
48947900
48948000
chr19: 48947560 − 48948005
Control
RMNS

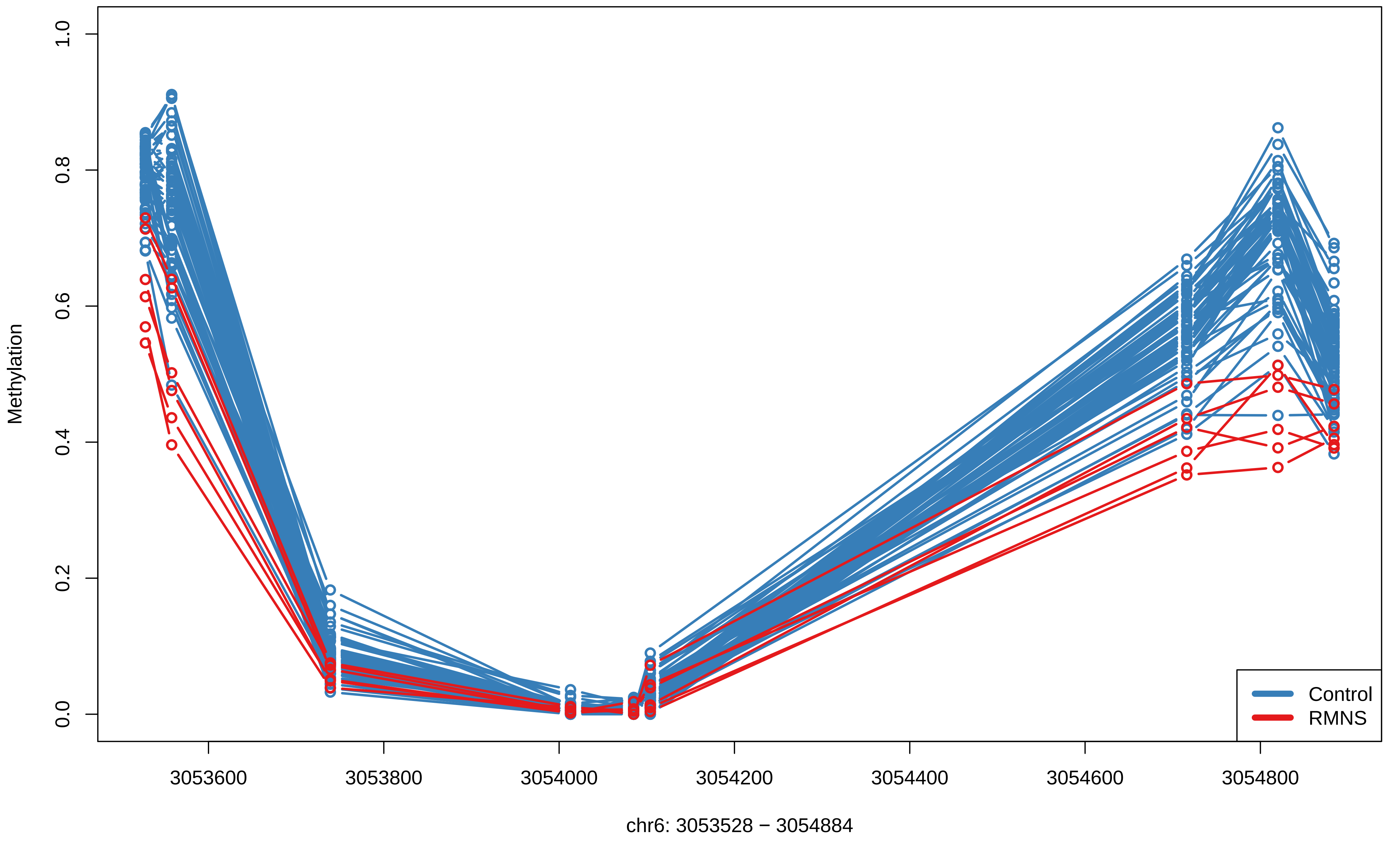

Methylation
0.0
0.2
0.4
0.6
0.8
1.0
3053600
3053800
3054000
3054200
3054400
3054600
3054800
chr6: 3053528 − 3054884
Control
RMNS

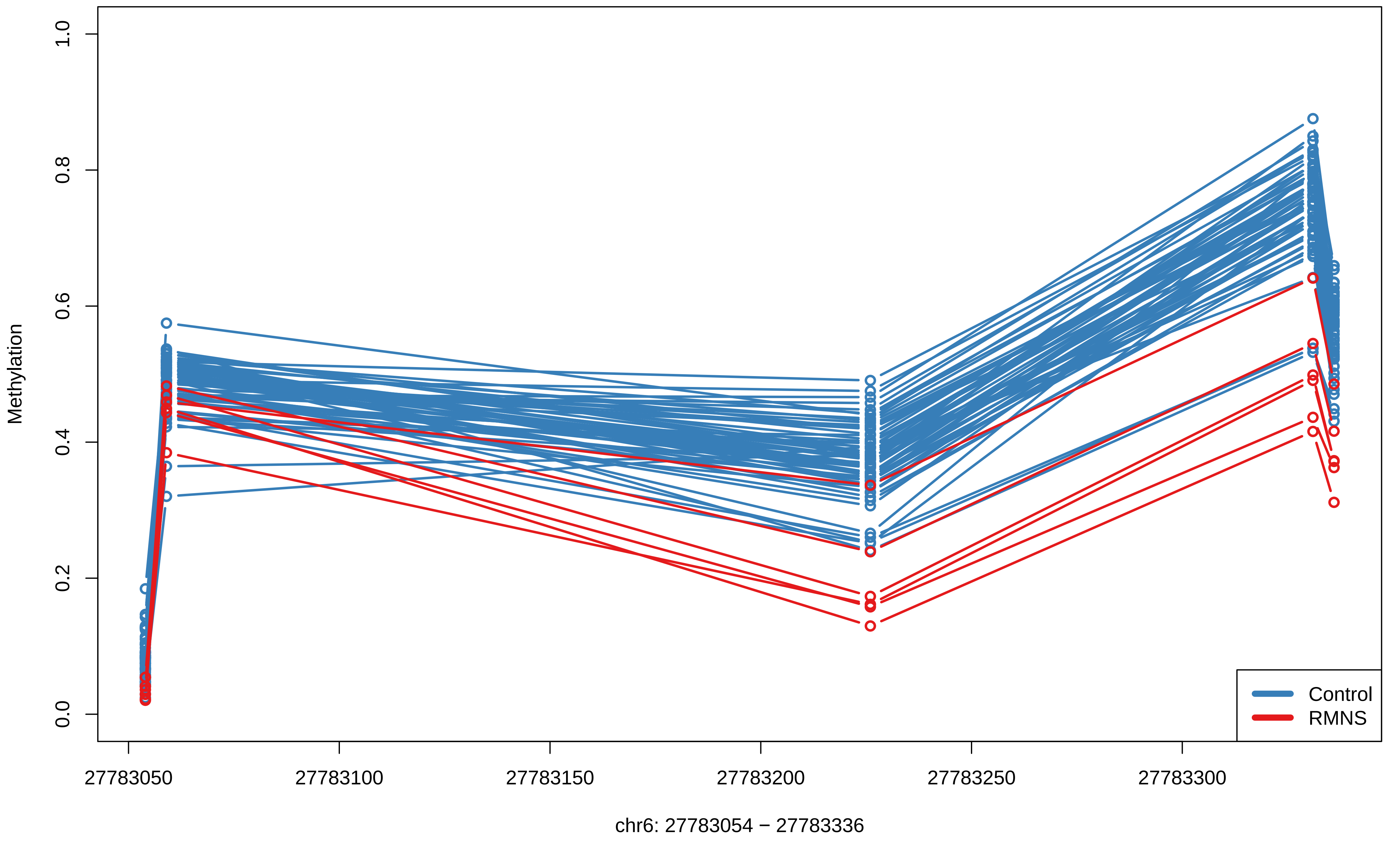

Methylation
0.0
0.2
0.4
0.6
0.8
1.0
27783050
27783100
27783150
27783200
27783250
27783300
chr6: 27783054 − 27783336
Control
RMNS

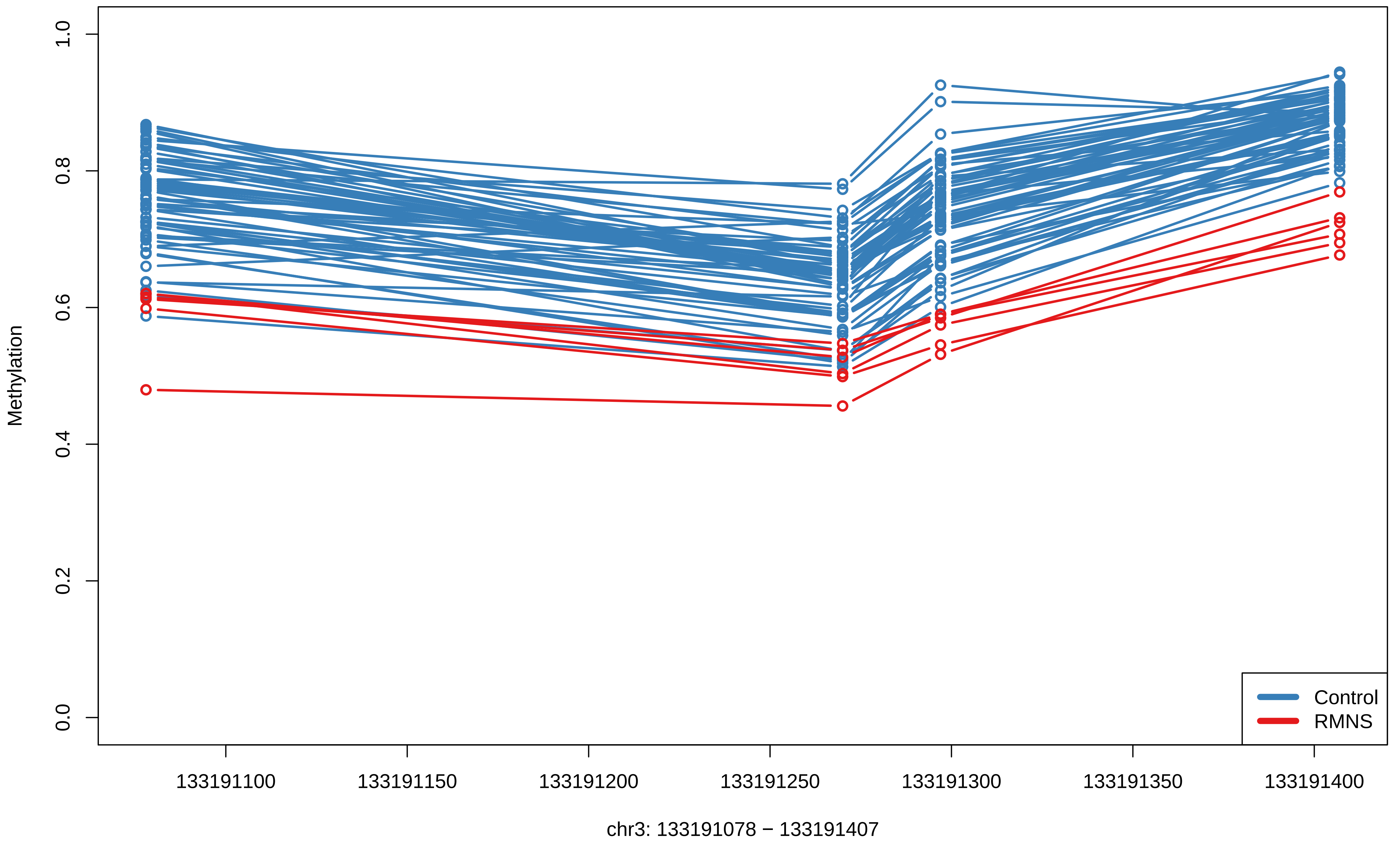

Methylation
1.0
0.8
0.6
0.4
0.2
0.0
133191100
133191150
133191200
133191250
133191300
133191350
133191400
chr3: 133191078 − 133191407
Control
RMNS

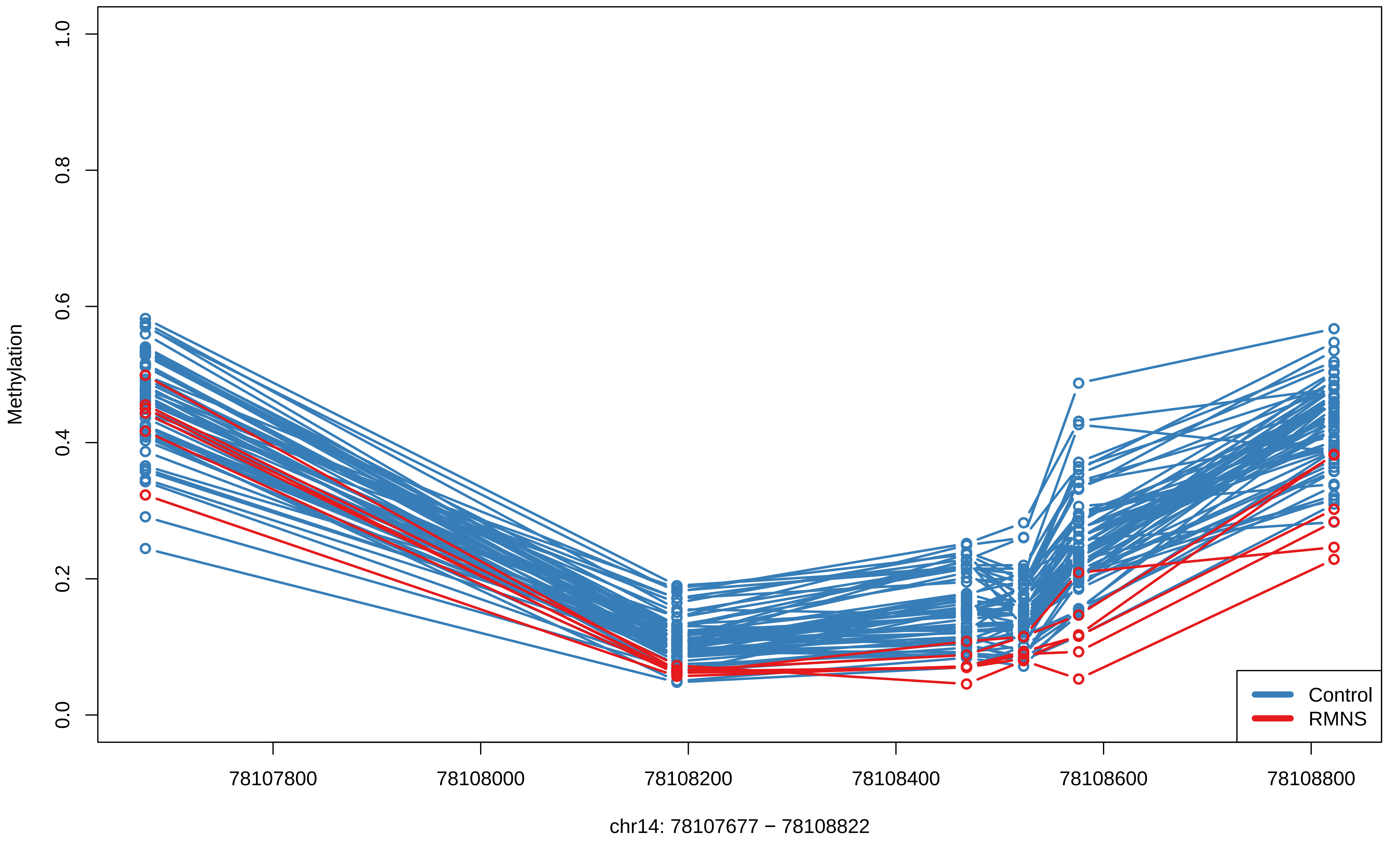

Methylation
0.0
0.2
0.4
0.6
0.8
1.0
78107800
78108000
78108200
78108400
78108600
78108800
chr14: 78107677 − 78108822
Control
RMNS

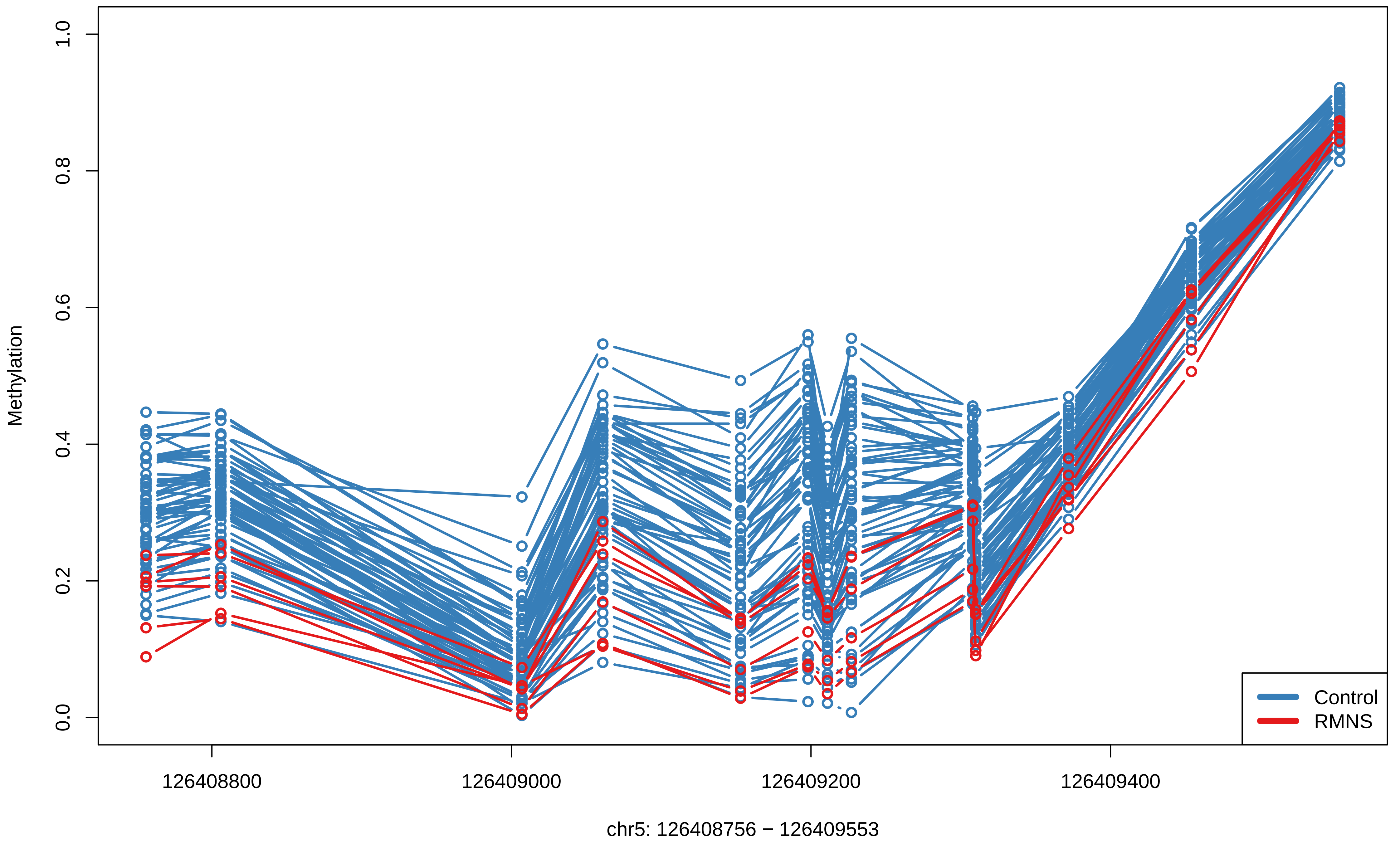

Methylation
1.0
0.8
0.6
0.4
0.2
0.0
126408800
126409000
126409200
126409400
chr5: 126408756 − 126409553
Control
RMNS

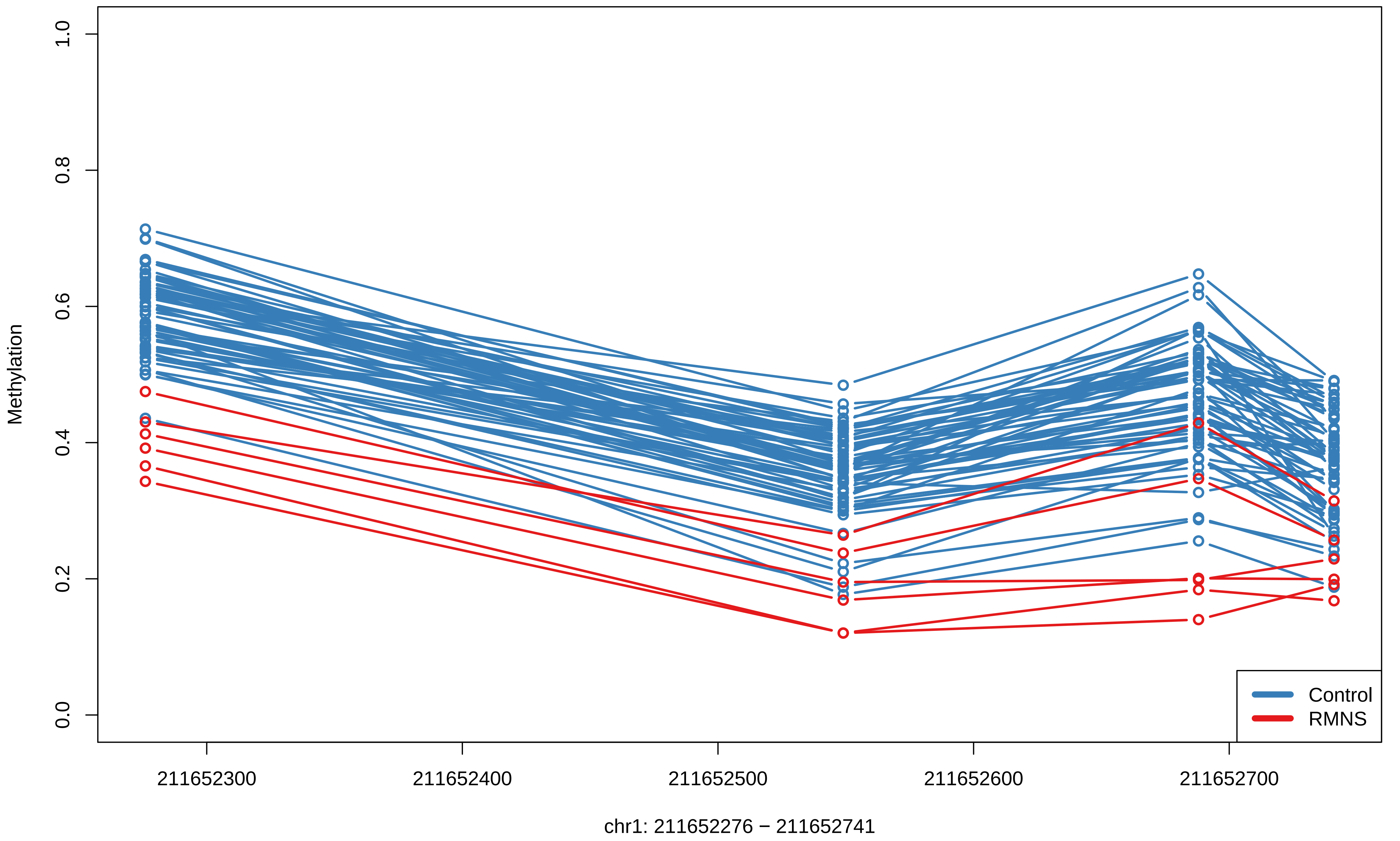

Methylation
0.0
0.2
0.4
0.6
0.8
1.0
211652300
211652400
211652500
211652600
211652700
chr1: 211652276 − 211652741
Control
RMNS

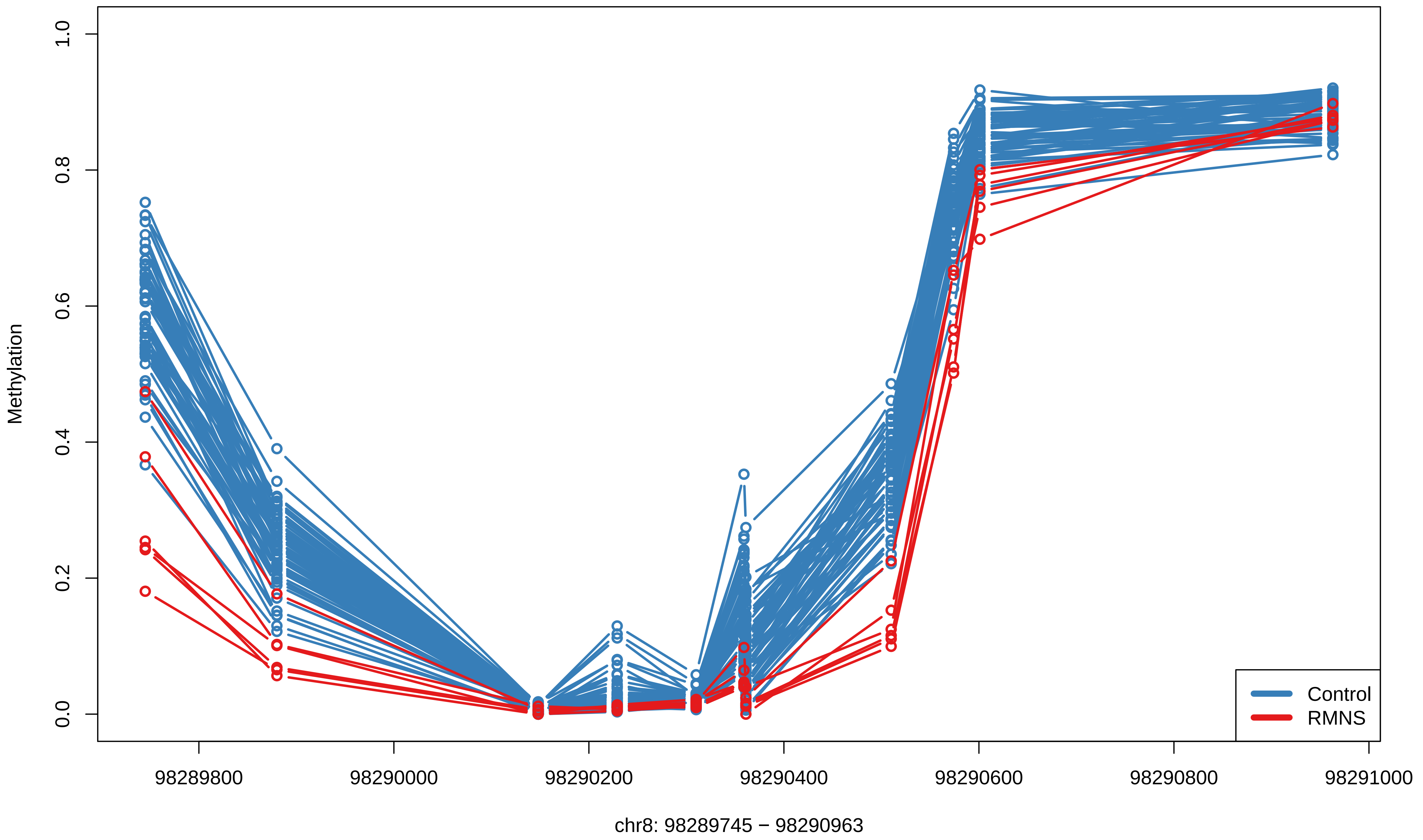

Methylation
0.0
0.2
0.4
0.6
0.8
1.0
98289800
98290000
98290200
98290400
98290600
98290800
98291000
chr8: 98289745 − 98290963
Control
RMNS

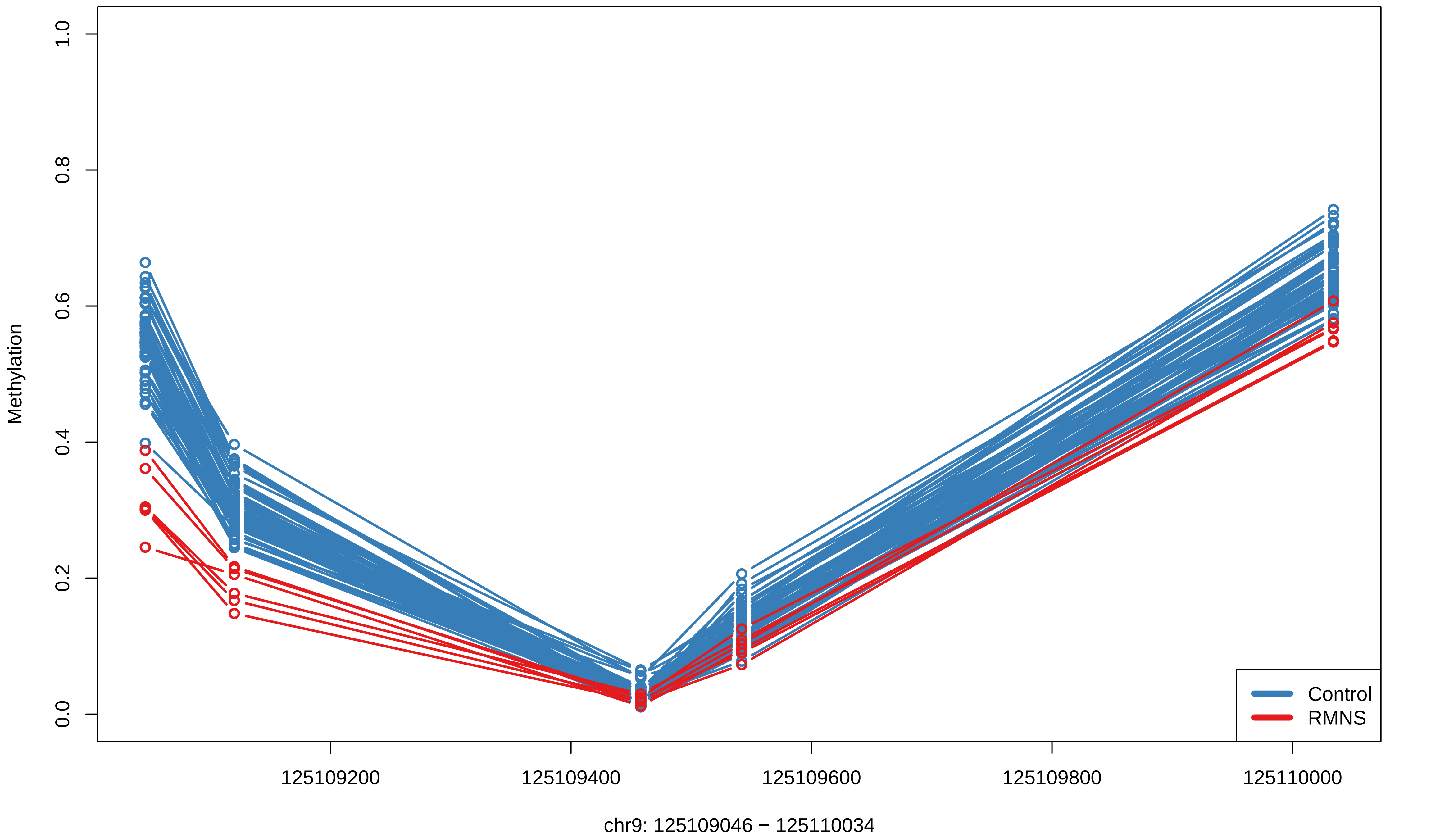

Methylation
0.0
0.2
0.4
0.6
0.8
1.0
125109200
125109400
125109600
125109800
125110000
chr9: 125109046 − 125110034
Control
RMNS

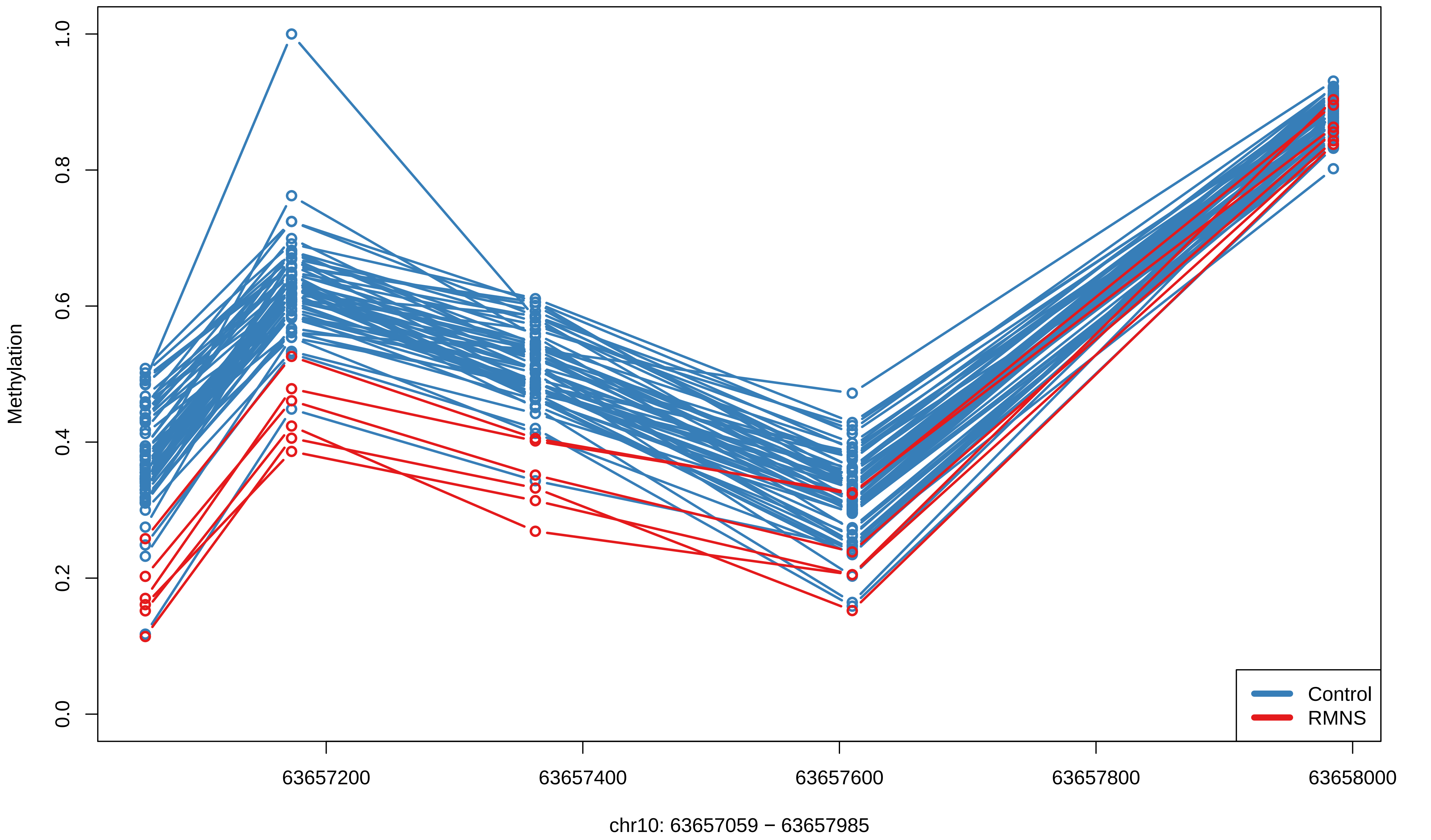

Methylation
0.0
0.2
0.4
0.6
0.8
1.0
63657200
63657400
63657600
63657800
63658000
chr10: 63657059 − 63657985
Control
RMNS

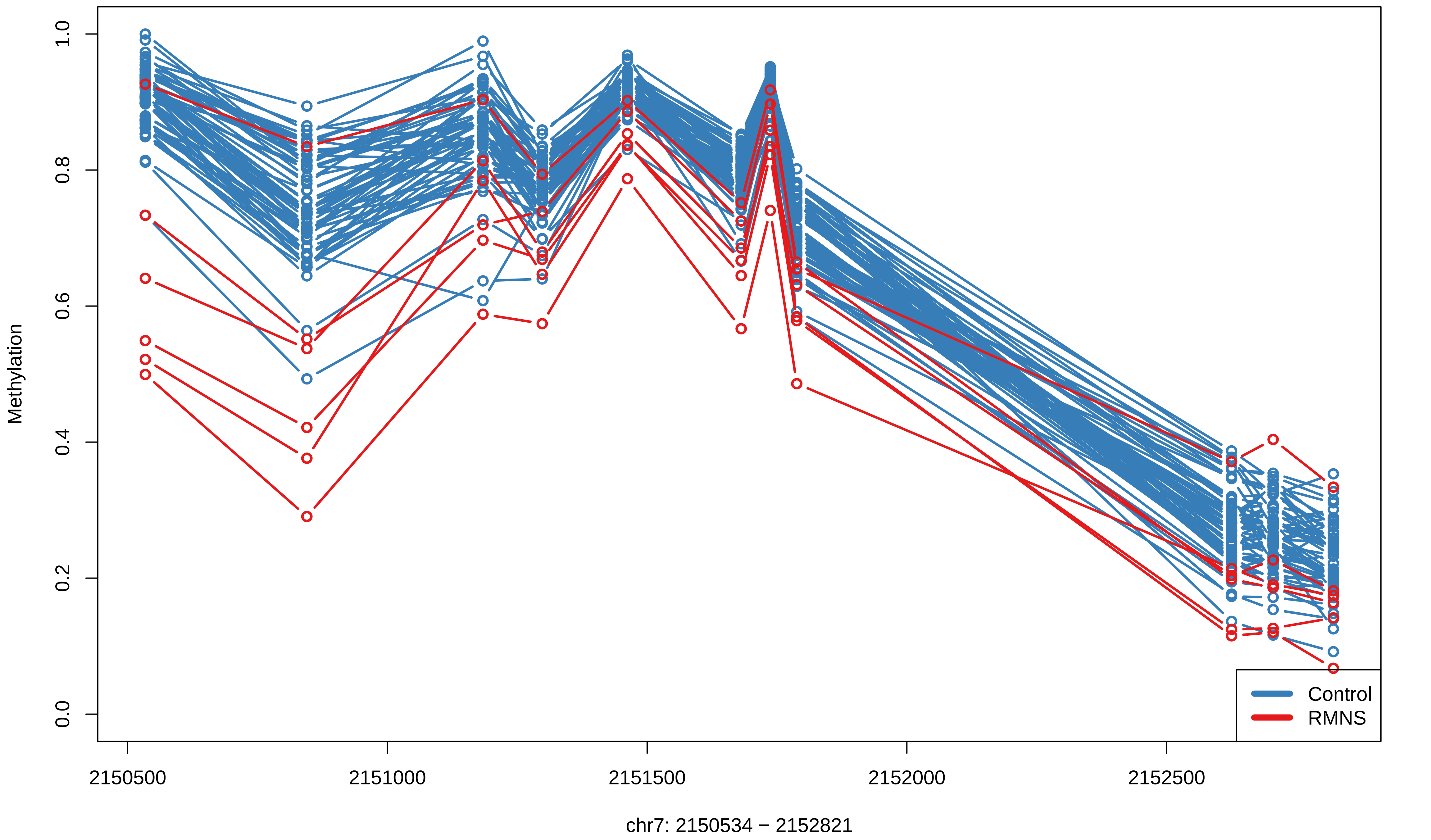
Methylation
1.0
0.8
0.6
0.4
0.2
0.0
2150500
2151000
2151500
2152000
2152500
chr7: 2150534 − 2152821
Control
RMNS

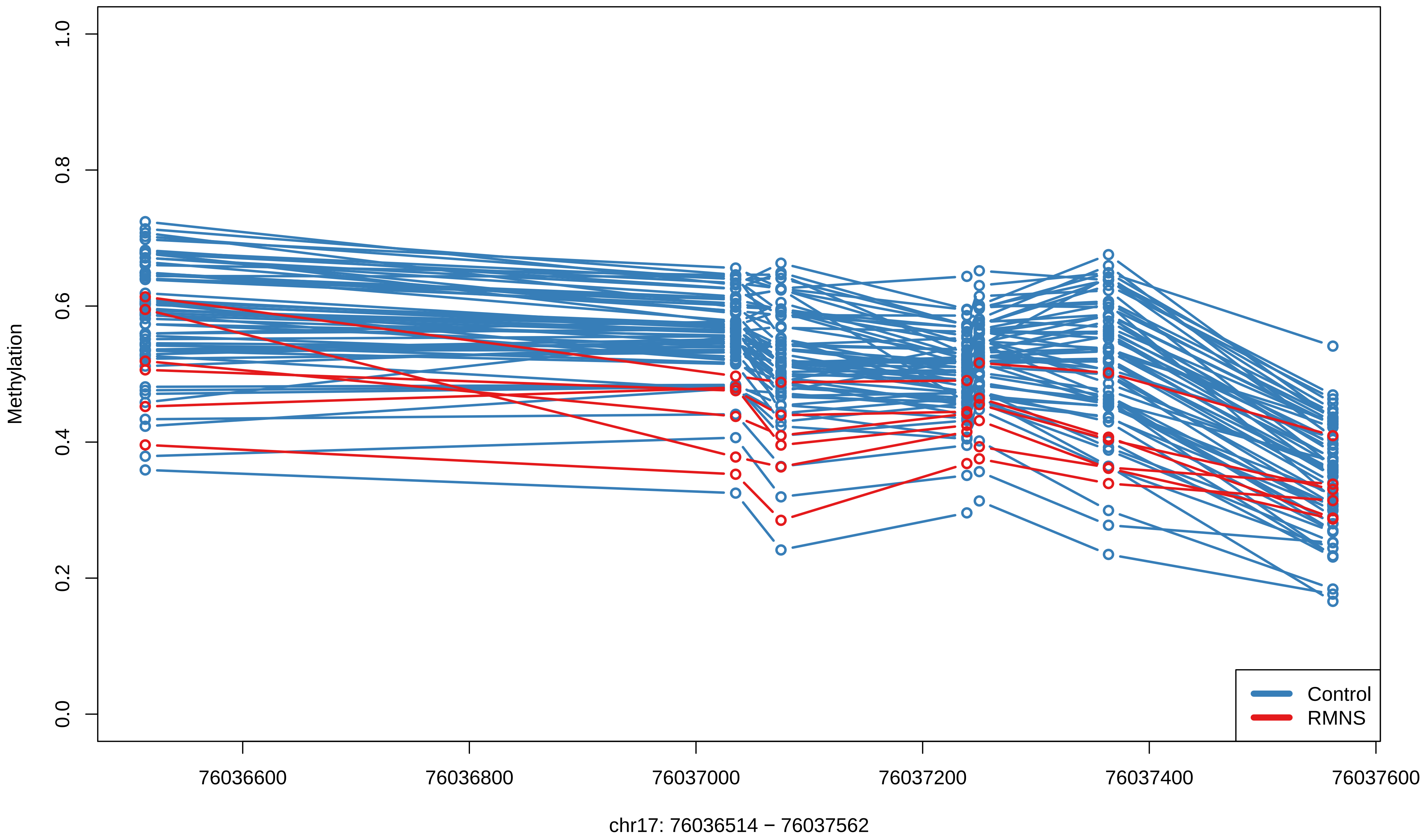

Methylation
0.0
0.2
0.4
0.6
0.8
1.0
76036600
76036800
76037000
76037200
76037400
76037600
chr17: 76036514 − 76037562
Control
RMNS

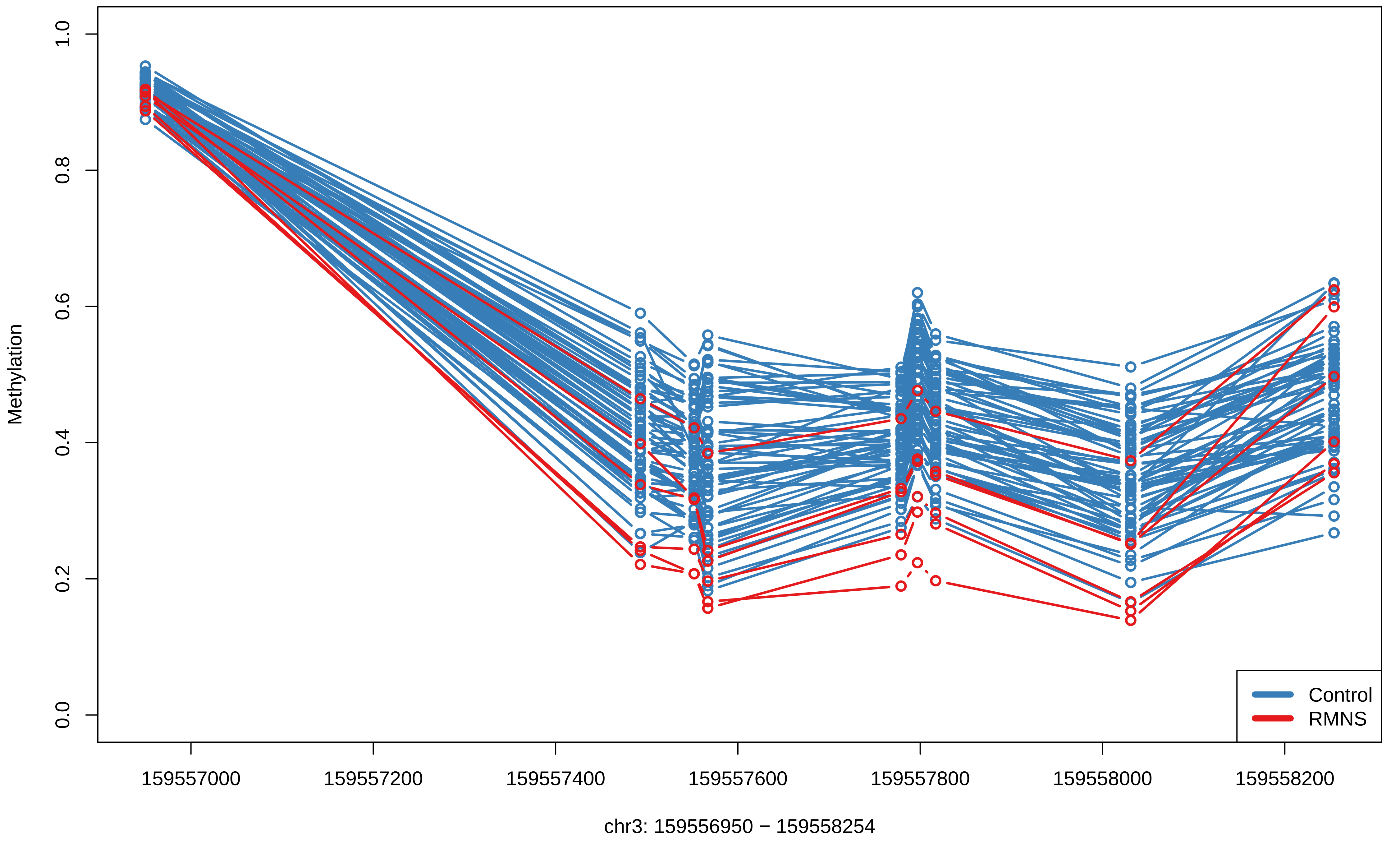

Methylation
1.0
0.8
0.6
0.4
0.2
0.0
159557000
159557200
159557400
159557600
159557800
159558000
159558200
chr3: 159556950 − 159558254
Control
RMNS

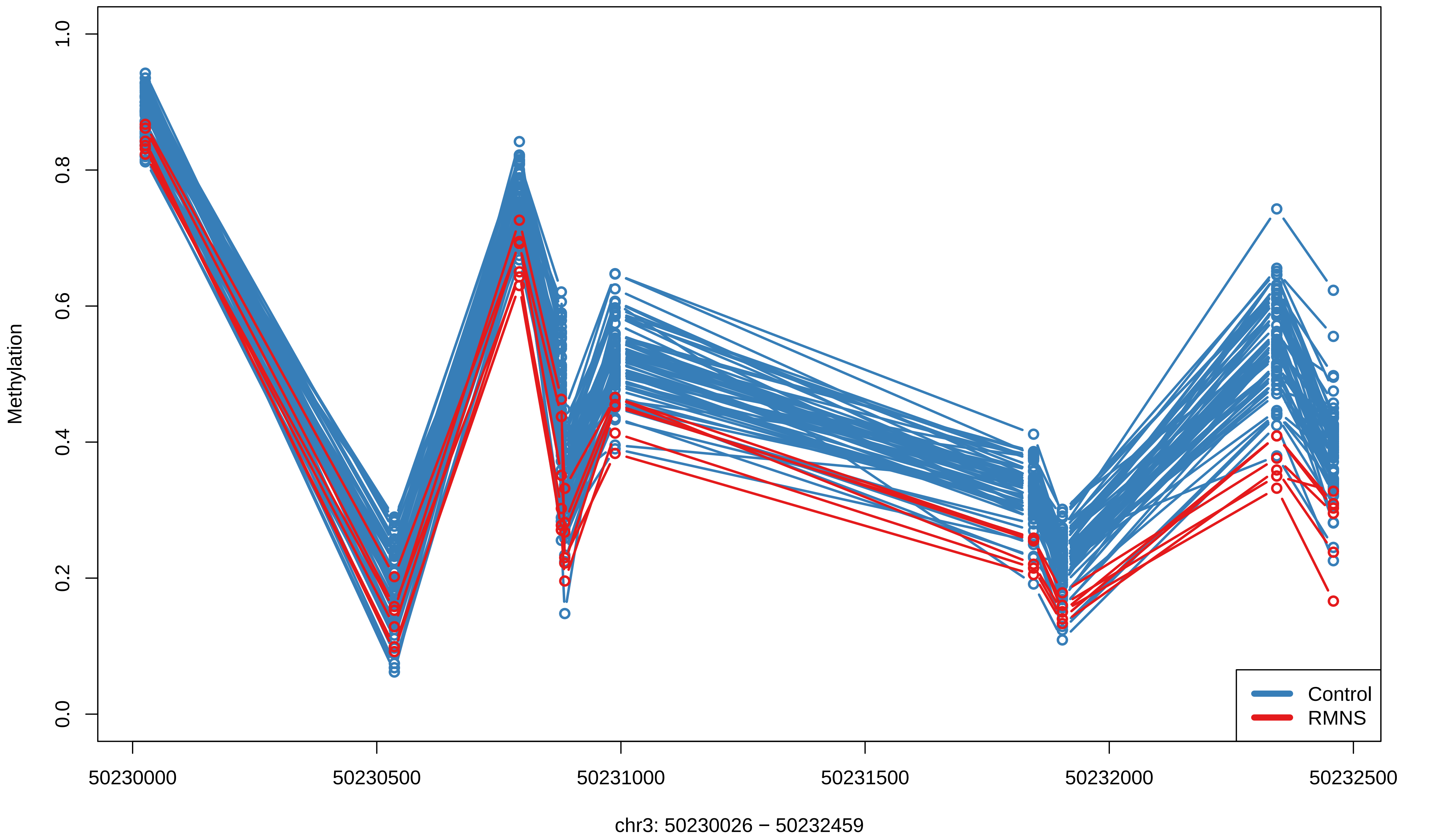

Methylation
0.0
0.2
0.4
0.6
0.8
1.0
50230000
50230500
50231000
50231500
50232000
50232500
chr3: 50230026 − 50232459
Control
RMNS

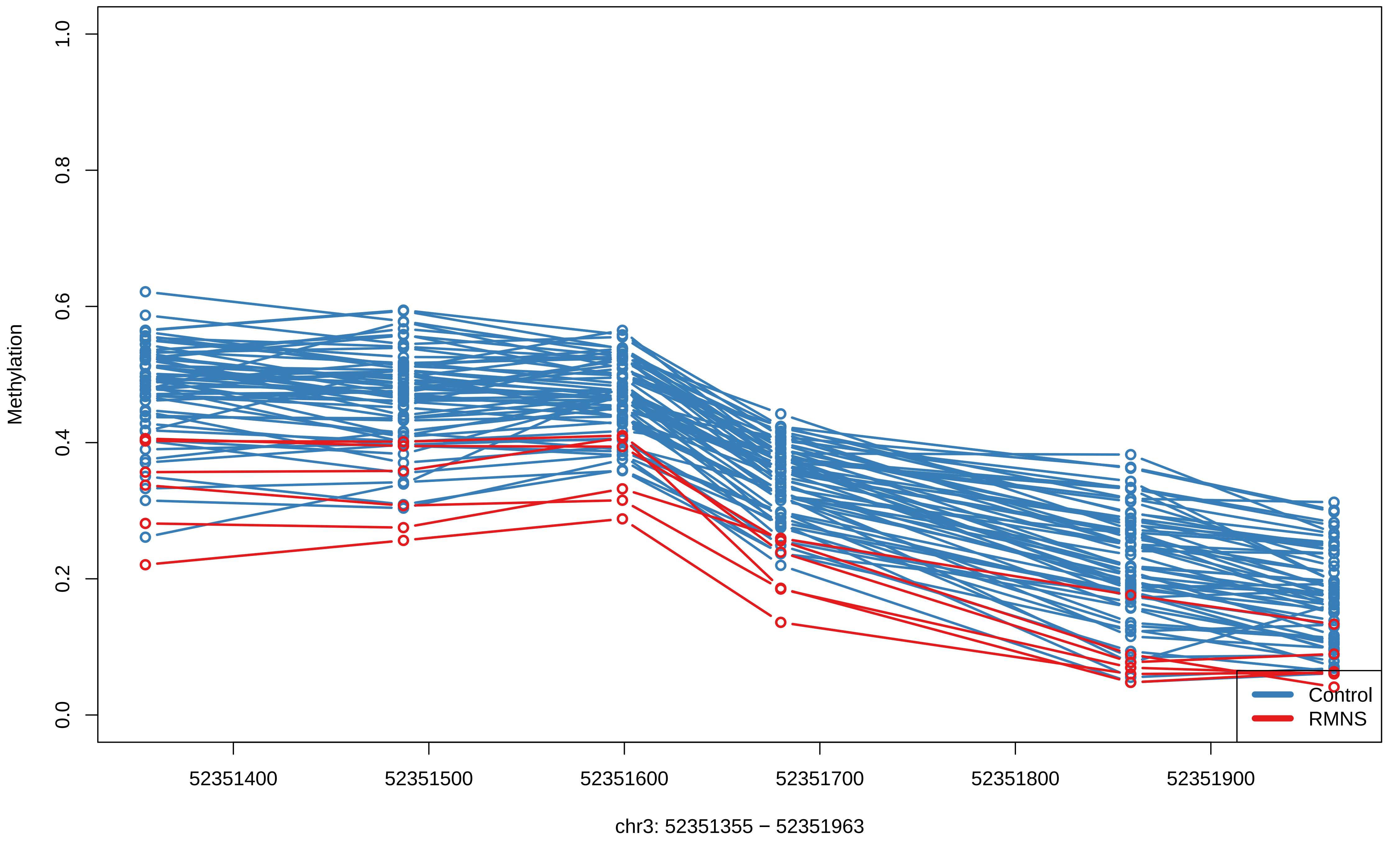

1.0
0.8
0.6
0.4
0.2
0.0
Methylation
52351400
52351500
52351600
52351700
52351800
52351900
chr3: 52351355 − 52351963
Control
RMNS

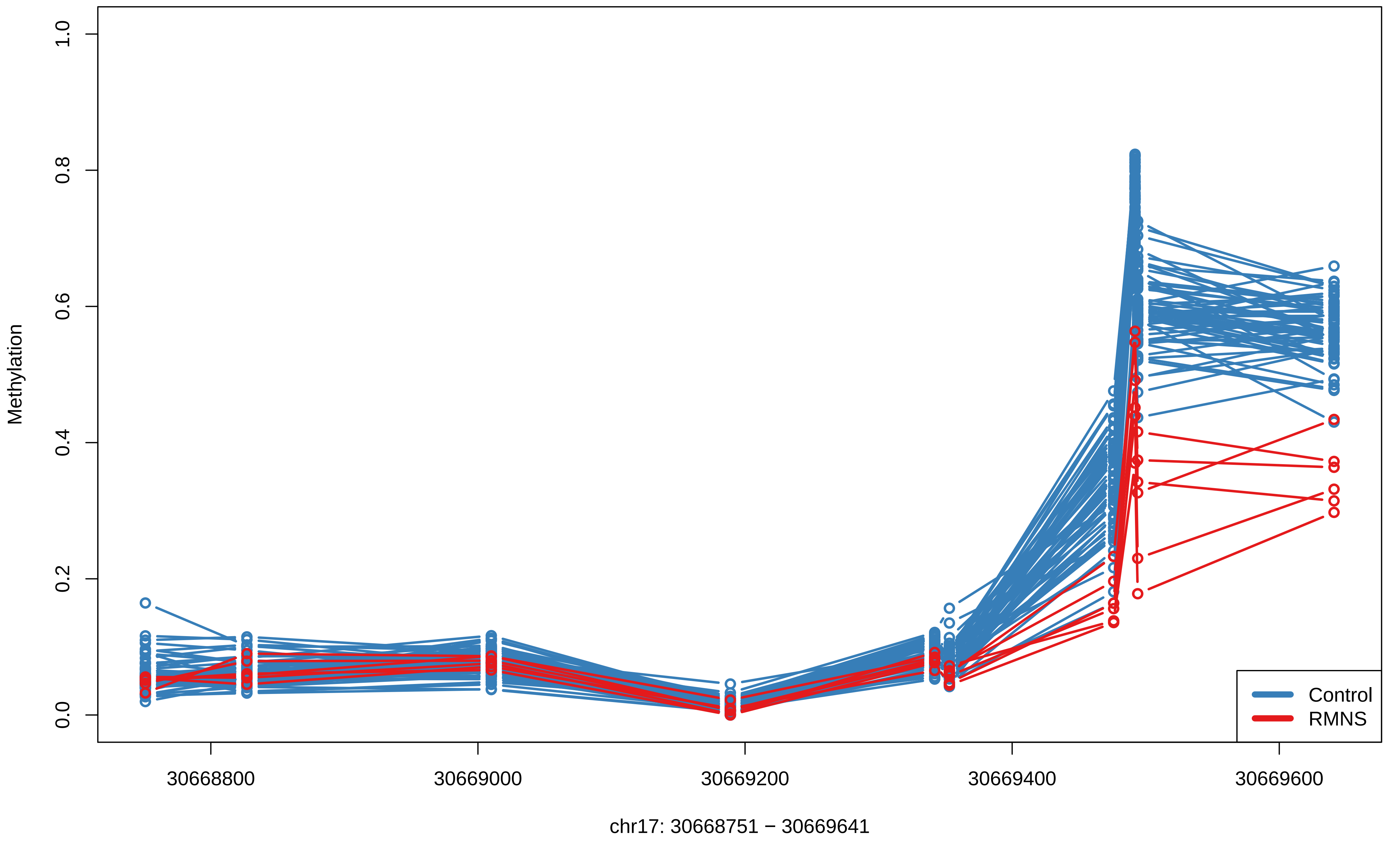

Methylation
0.0
0.2
0.4
0.6
0.8
1.0
30668800
30669000
30669200
30669400
30669600
chr17: 30668751 − 30669641
Control
RMNS

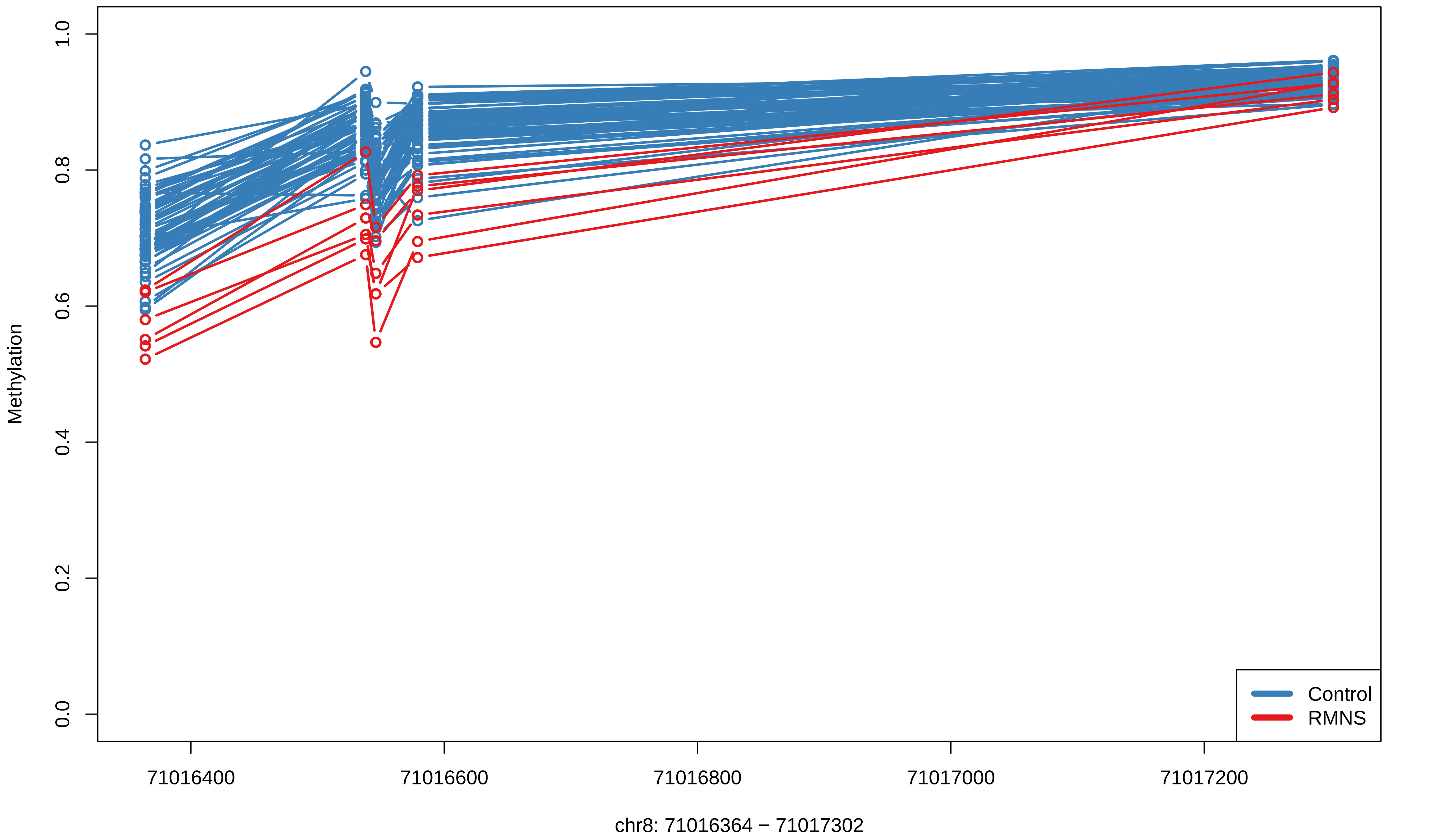

Methylation
1.0
0.8
0.6
0.4
0.2
0.0
71016400
71016600
71016800
71017000
71017200
chr8: 71016364 − 71017302
Control
RMNS

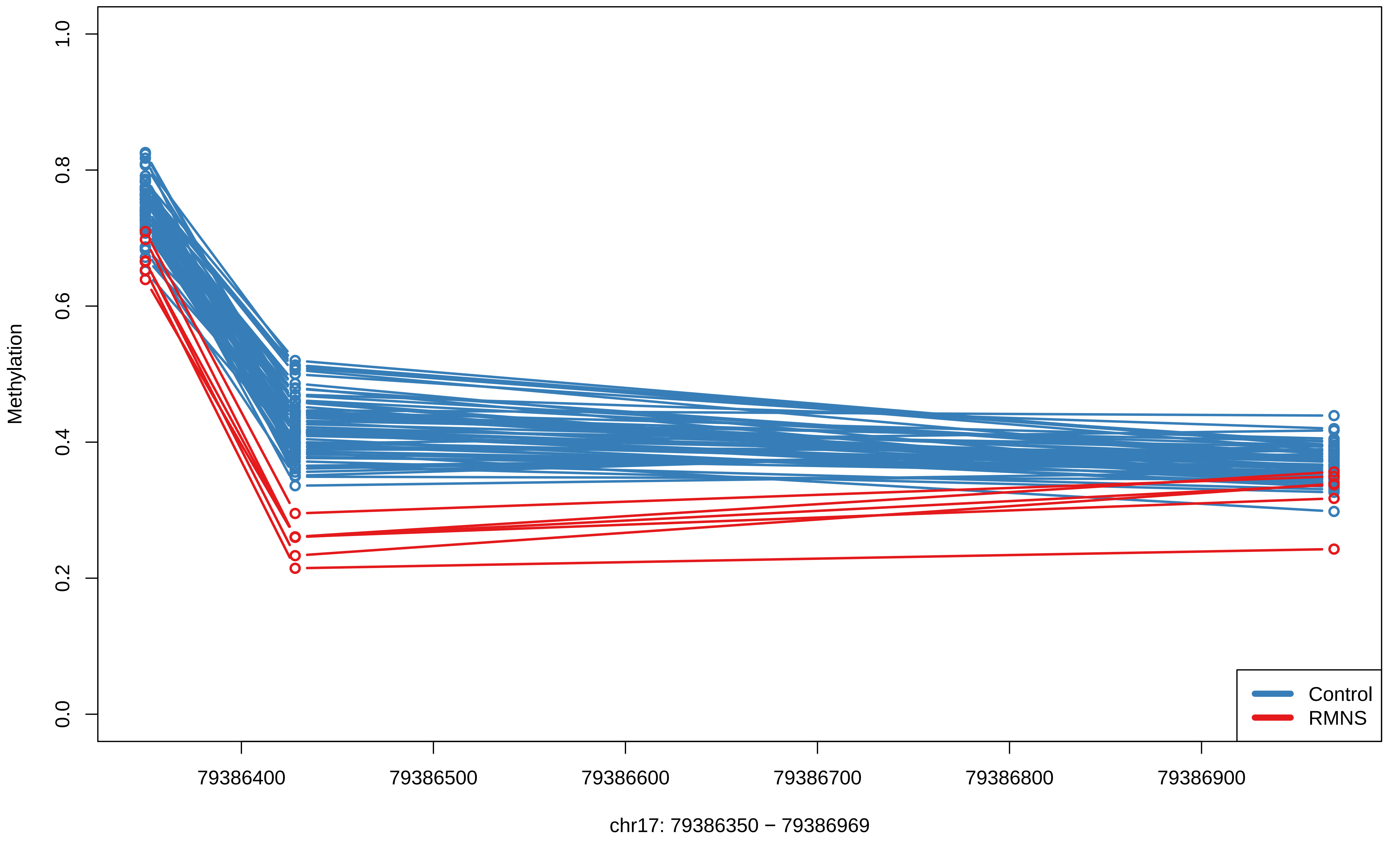

Methylation
0.0
0.2
0.4
0.6
0.8
1.0
79386400
79386500
79386600
79386700
79386800
79386900
chr17: 79386350 − 79386969
Control
RMNS

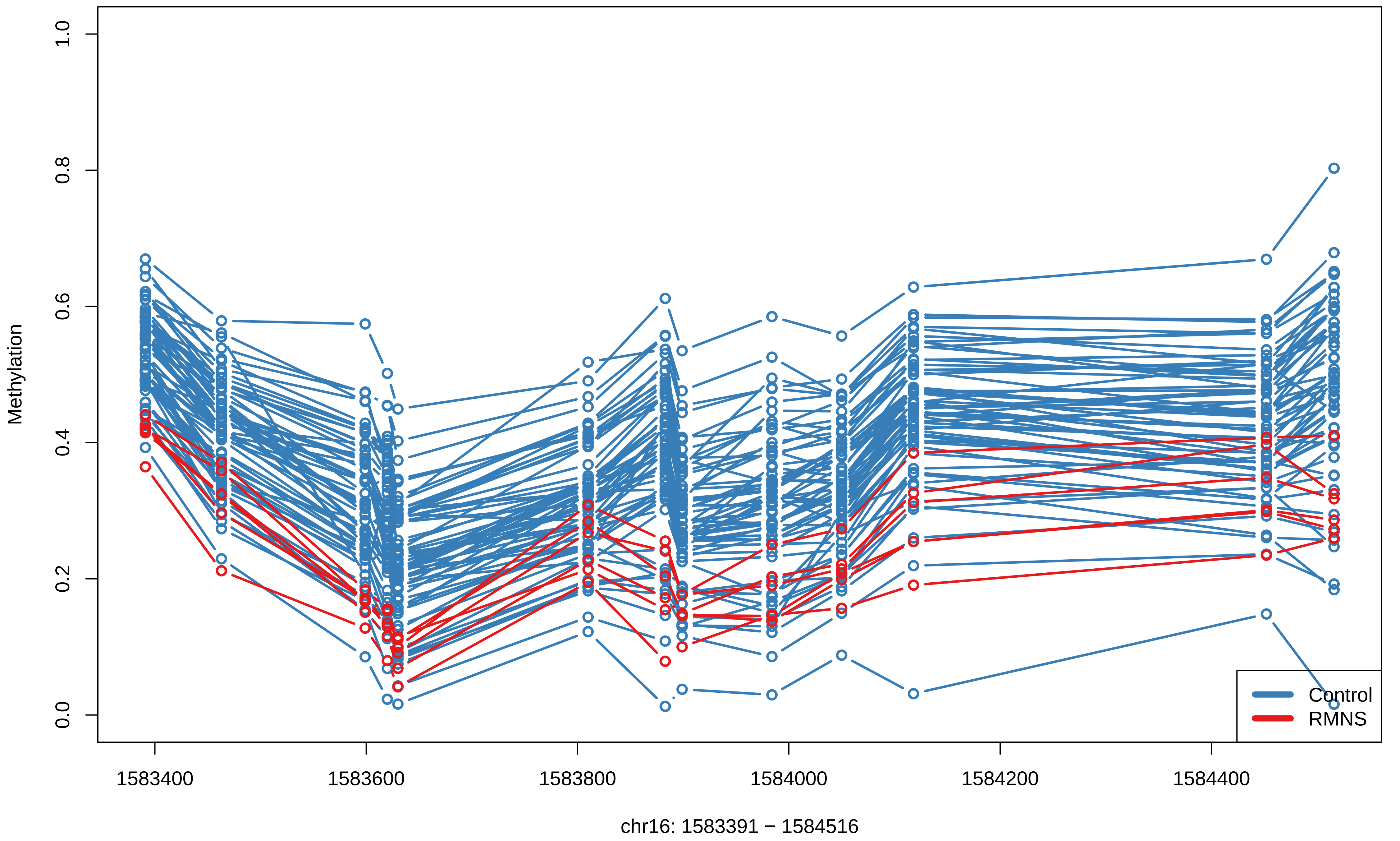

Methylation
0.0
0.2
0.4
0.6
0.8
1.0
1583400
1583600
1583800
1584000
1584200
1584400
chr16: 1583391 − 1584516
Control
RMNS

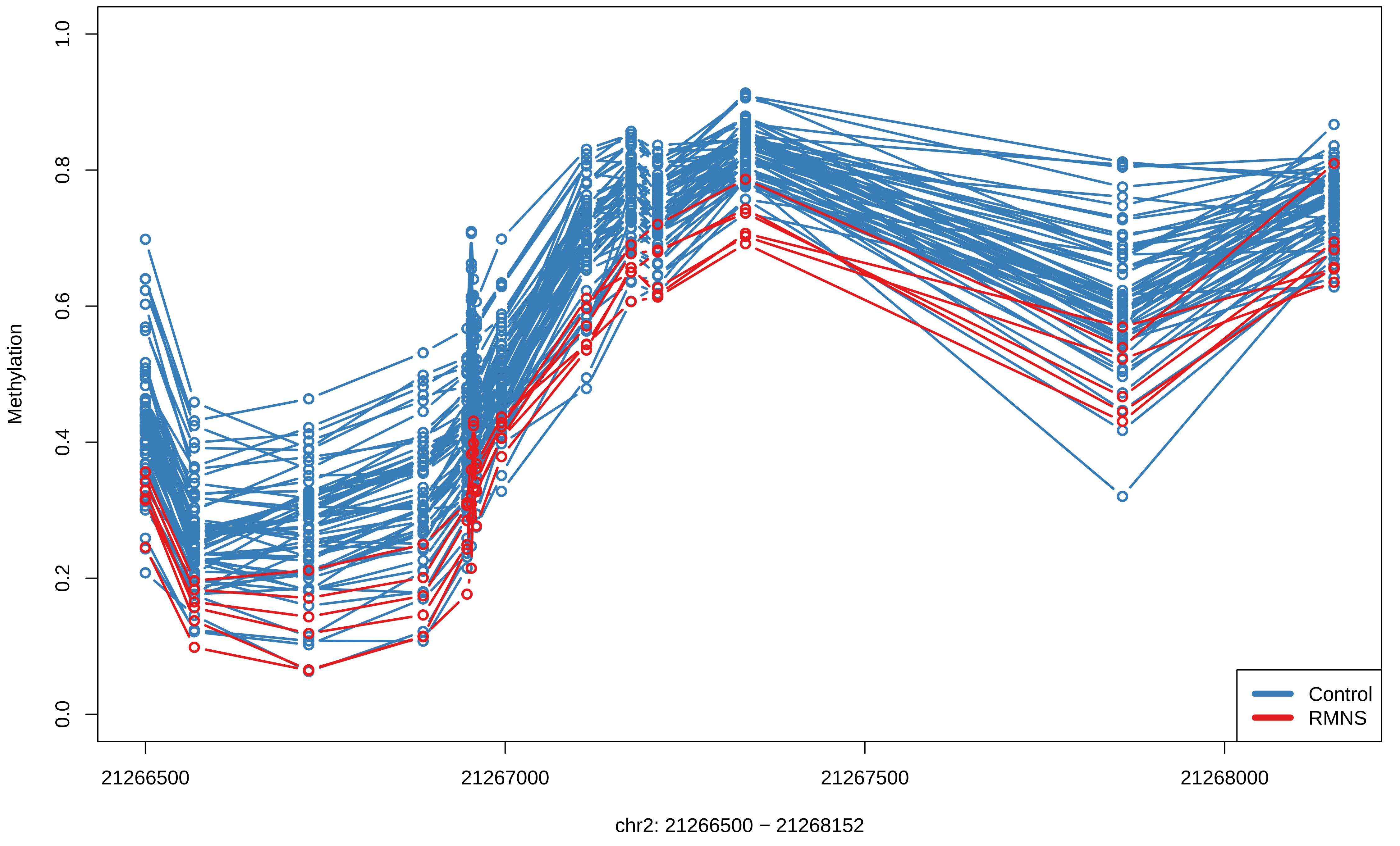
Methylation
0.0
0.2
0.4
0.6
0.8
1.0
21266500
21267000
21267500
21268000
chr2: 21266500 − 21268152
Control
RMNS

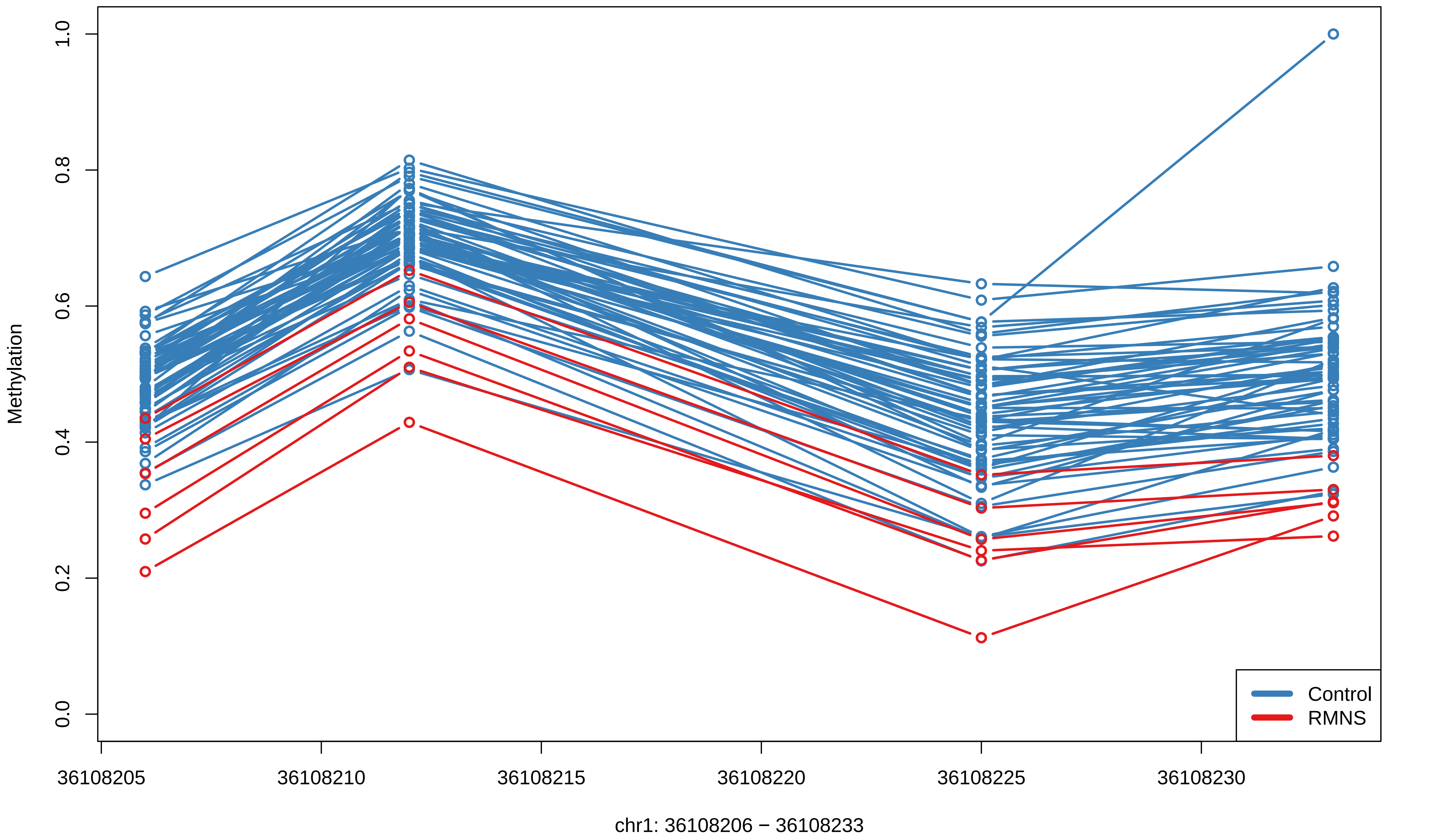

Methylation
0.0
0.2
0.4
0.6
0.8
1.0
36108205
36108210
36108215
36108220
36108225
36108230
chr1: 36108206 − 36108233
Control
RMNS

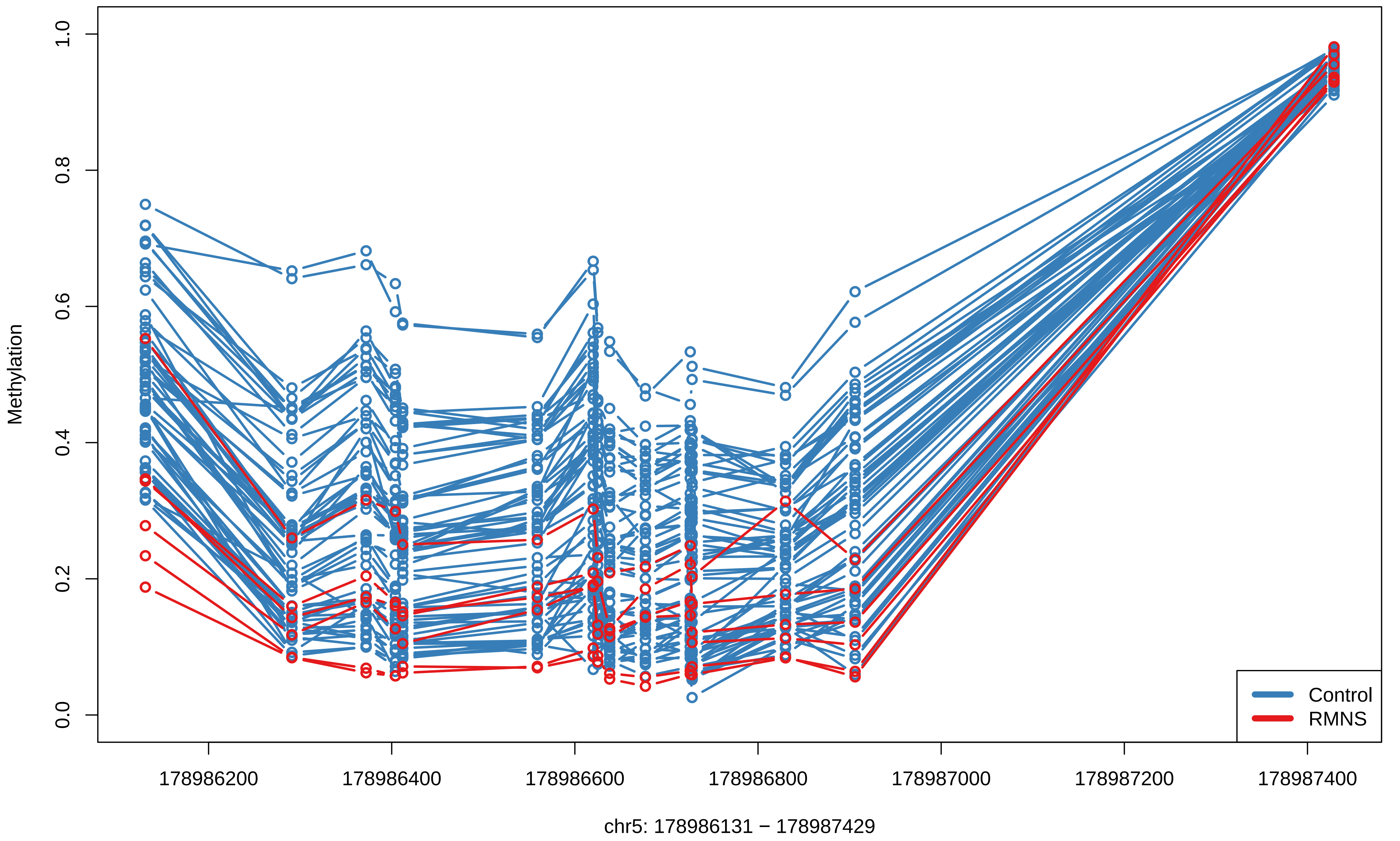

Methylation
0.0
0.2
0.4
0.6
0.8
1.0
178986200
178986400
178986600
178986800
178987000
178987200
178987400
chr5: 178986131 − 178987429
Control
RMNS

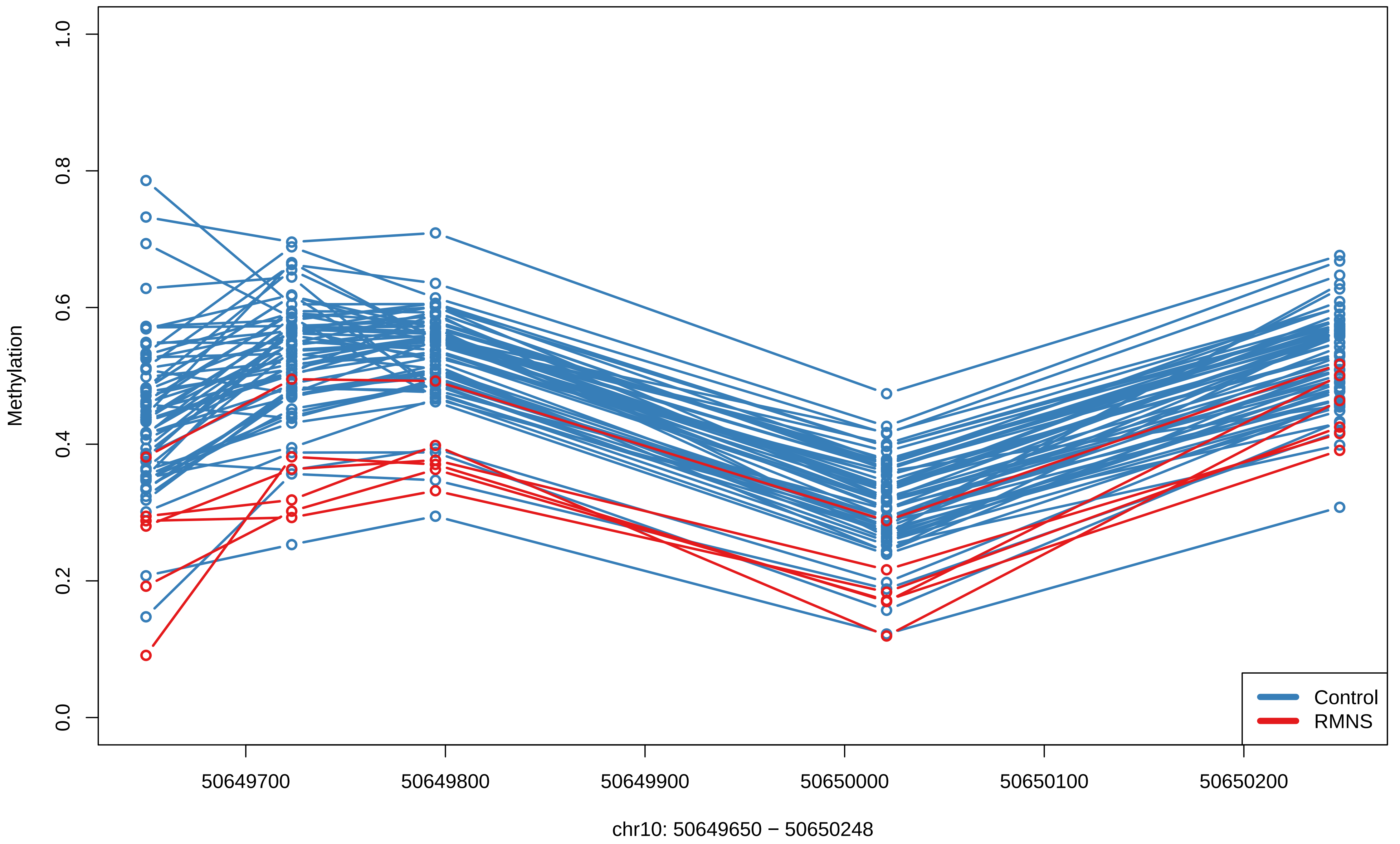
Methylation
0.0
0.2
0.4
0.6
0.8
1.0
50649700
50649800
50649900
50650000
50650100
50650200
chr10: 50649650 − 50650248
Control
RMNS

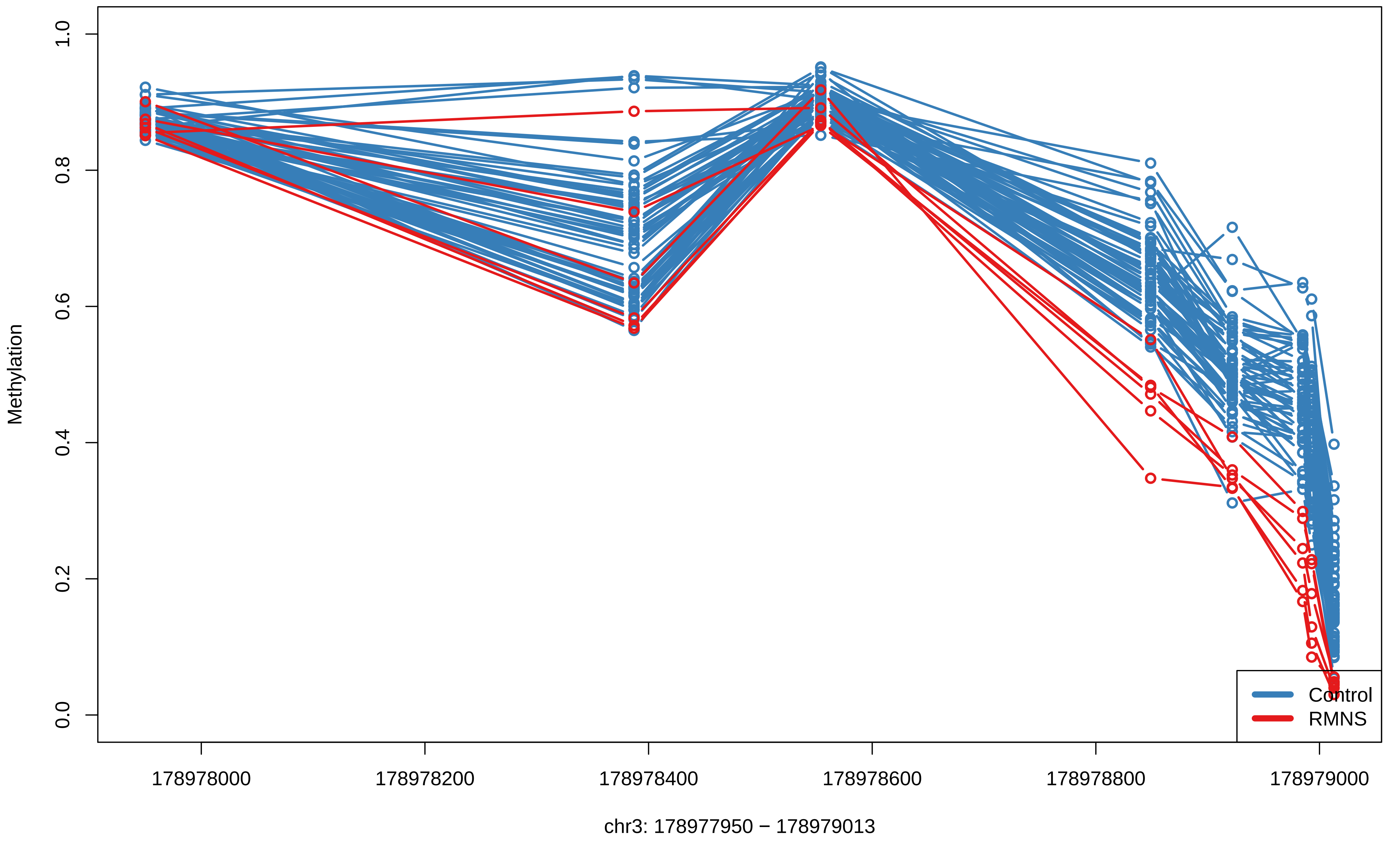

Methylation
0.0
0.2
0.4
0.6
0.8
1.0
178978000
178978200
178978400
178978600
178978800
178979000
chr3: 178977950 − 178979013
Control
RMNS

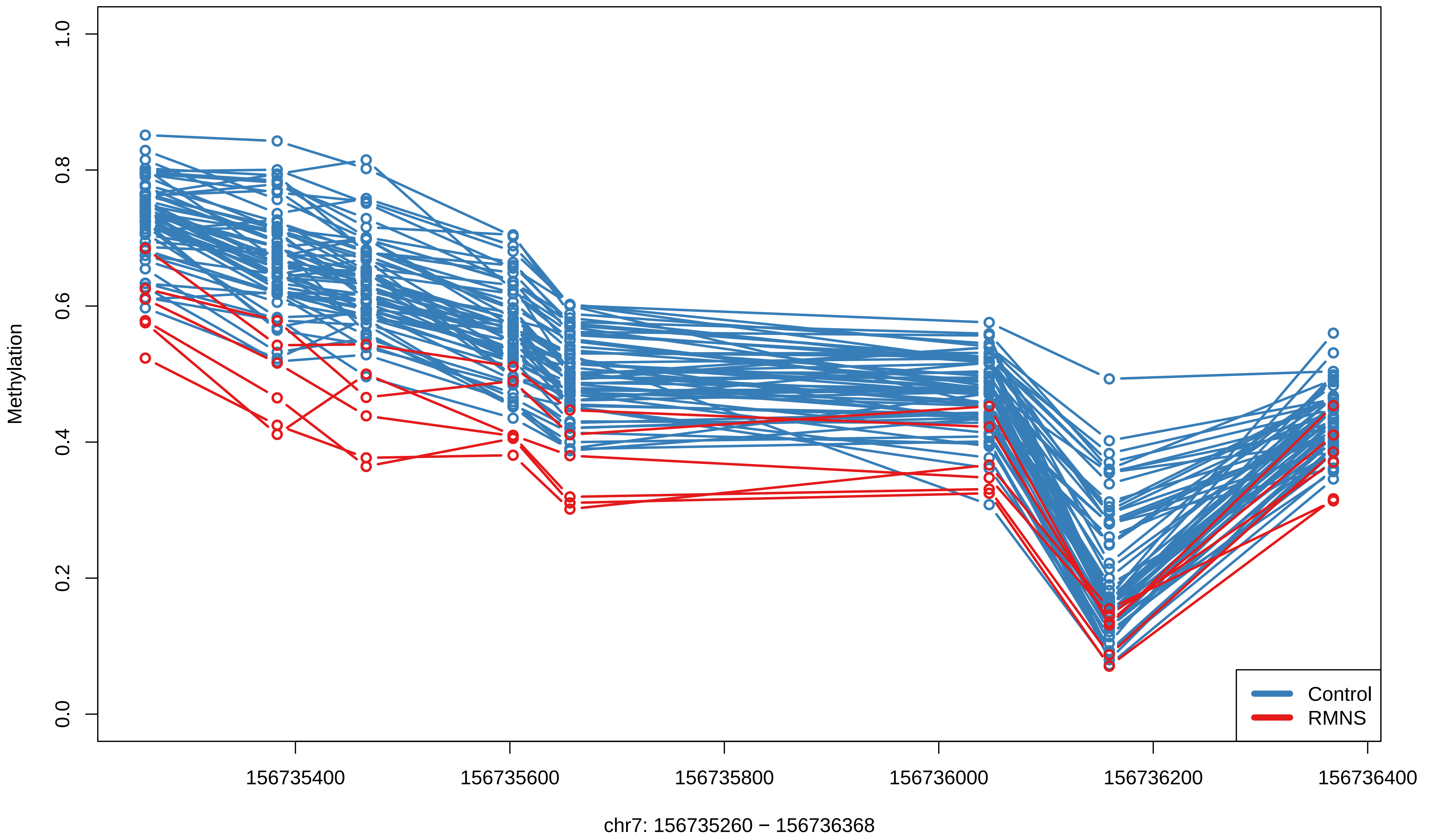

Methylation
1.0
0.8
0.6
0.4
0.2
0.0
156735400
156735600
156735800
156736000
156736200
156736400
chr7: 156735260 − 156736368
Control
RMNS

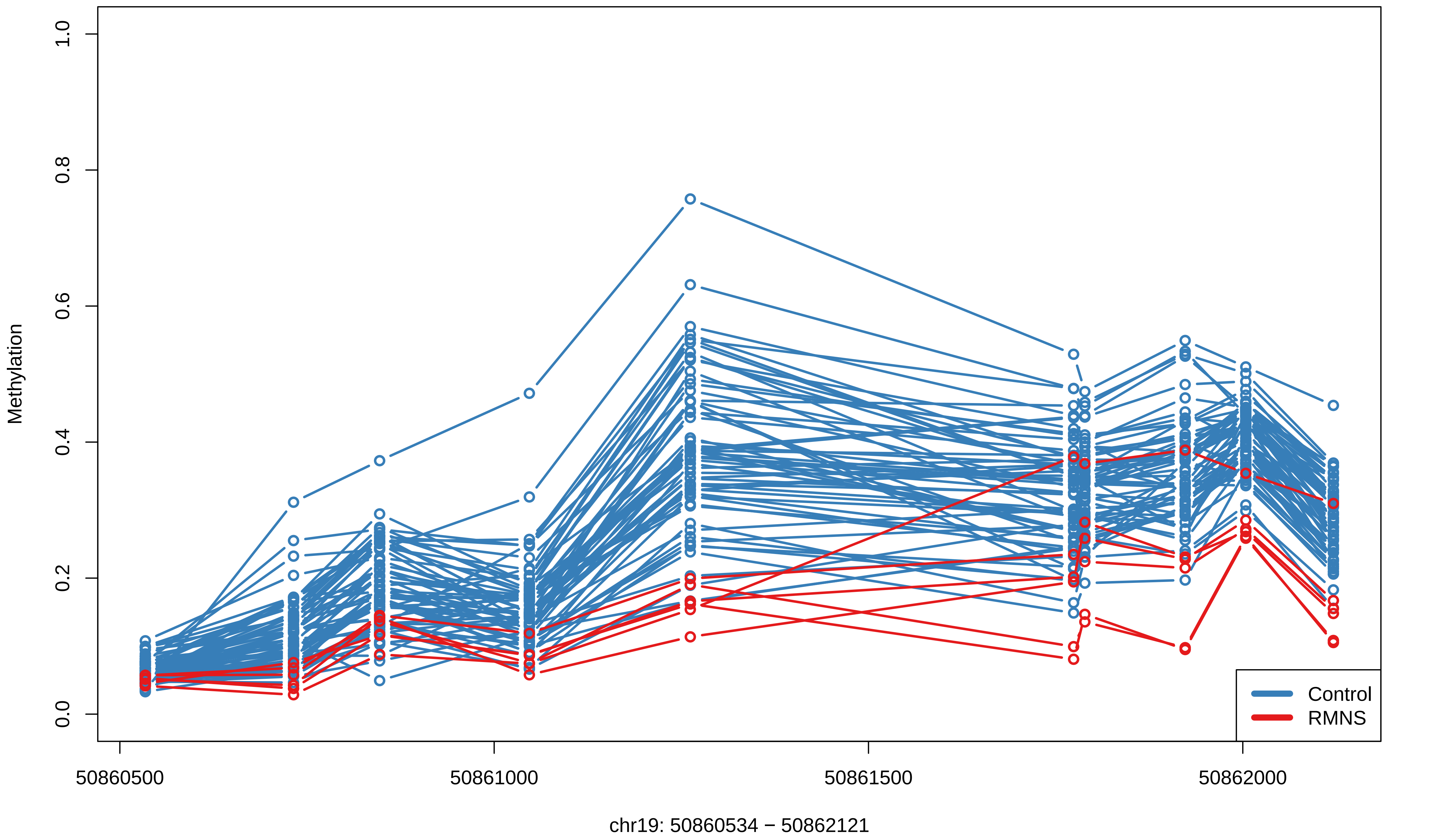

Methylation
0.0
0.2
0.4
0.6
0.8
1.0
50860500
50861000
50861500
50862000
chr19: 50860534 − 50862121
Control
RMNS

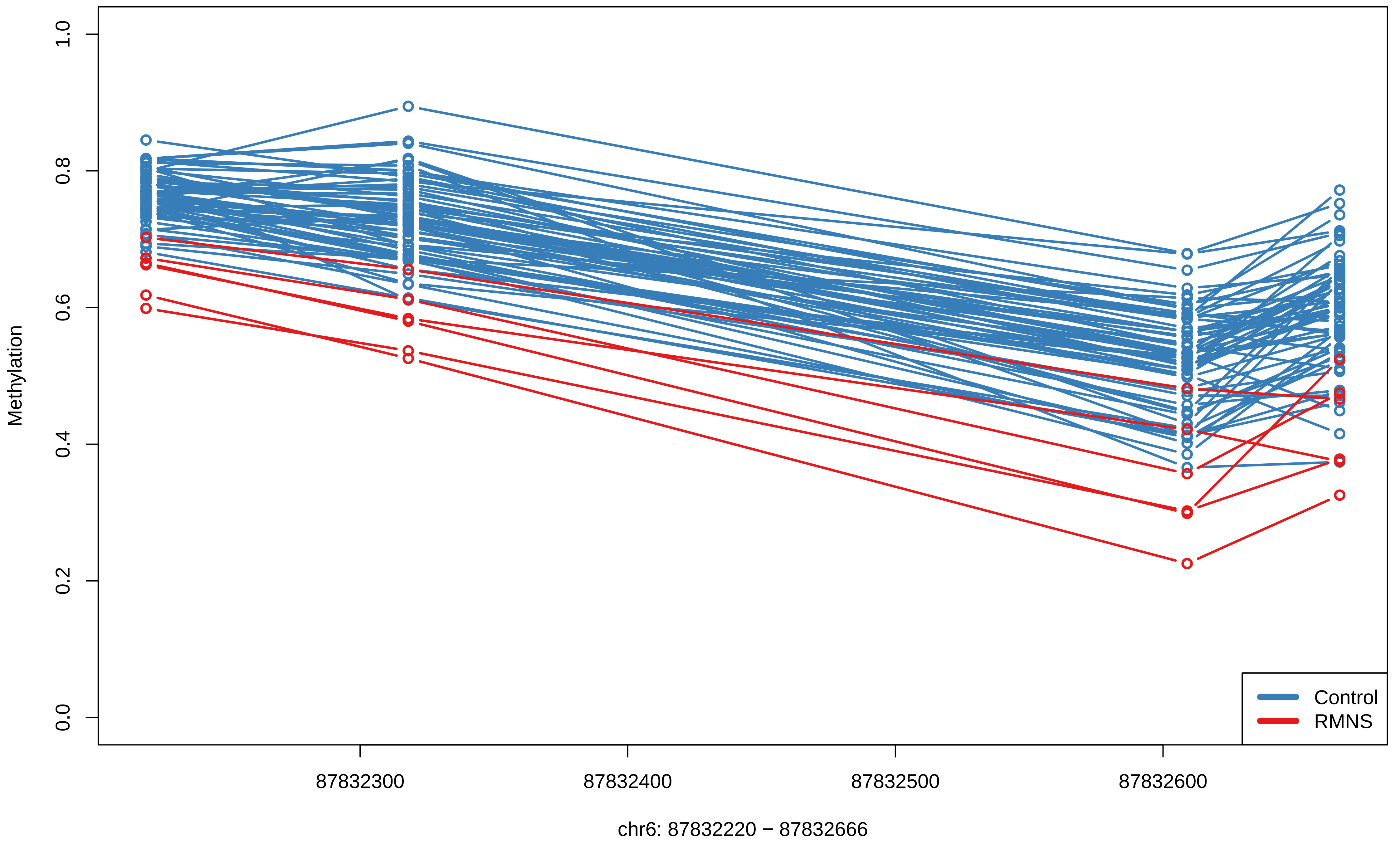

1.0
0.8
0.6
0.4
0.2
0.0
Methylation
87832300
87832400
87832500
87832600
chr6: 87832220 − 87832666
Control
RMNS

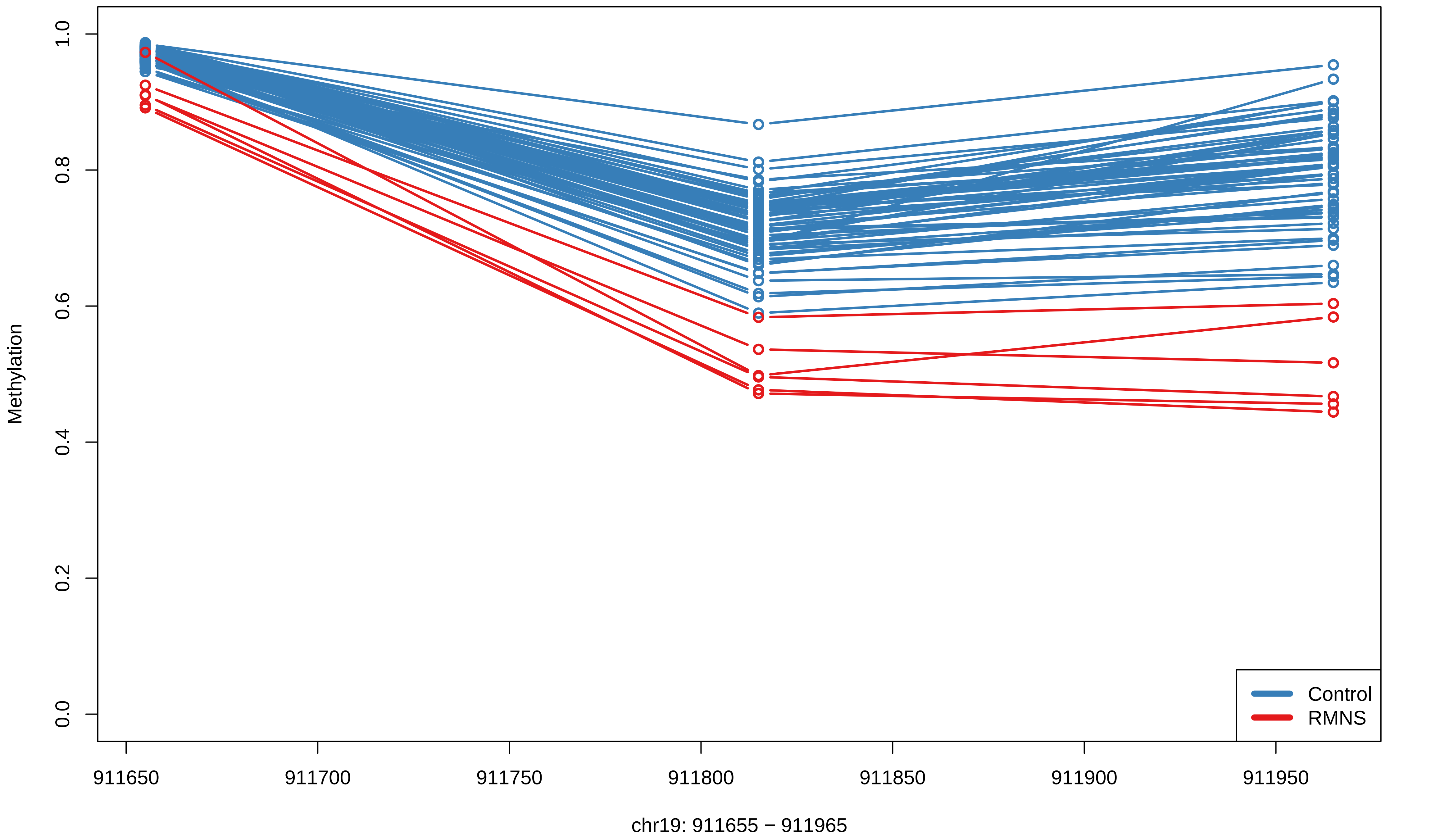

Methylation
1.0
0.8
0.6
0.4
0.2
0.0
911650
911700
911750
911800
911850
911900
911950
chr19: 911655 − 911965
Control
RMNS

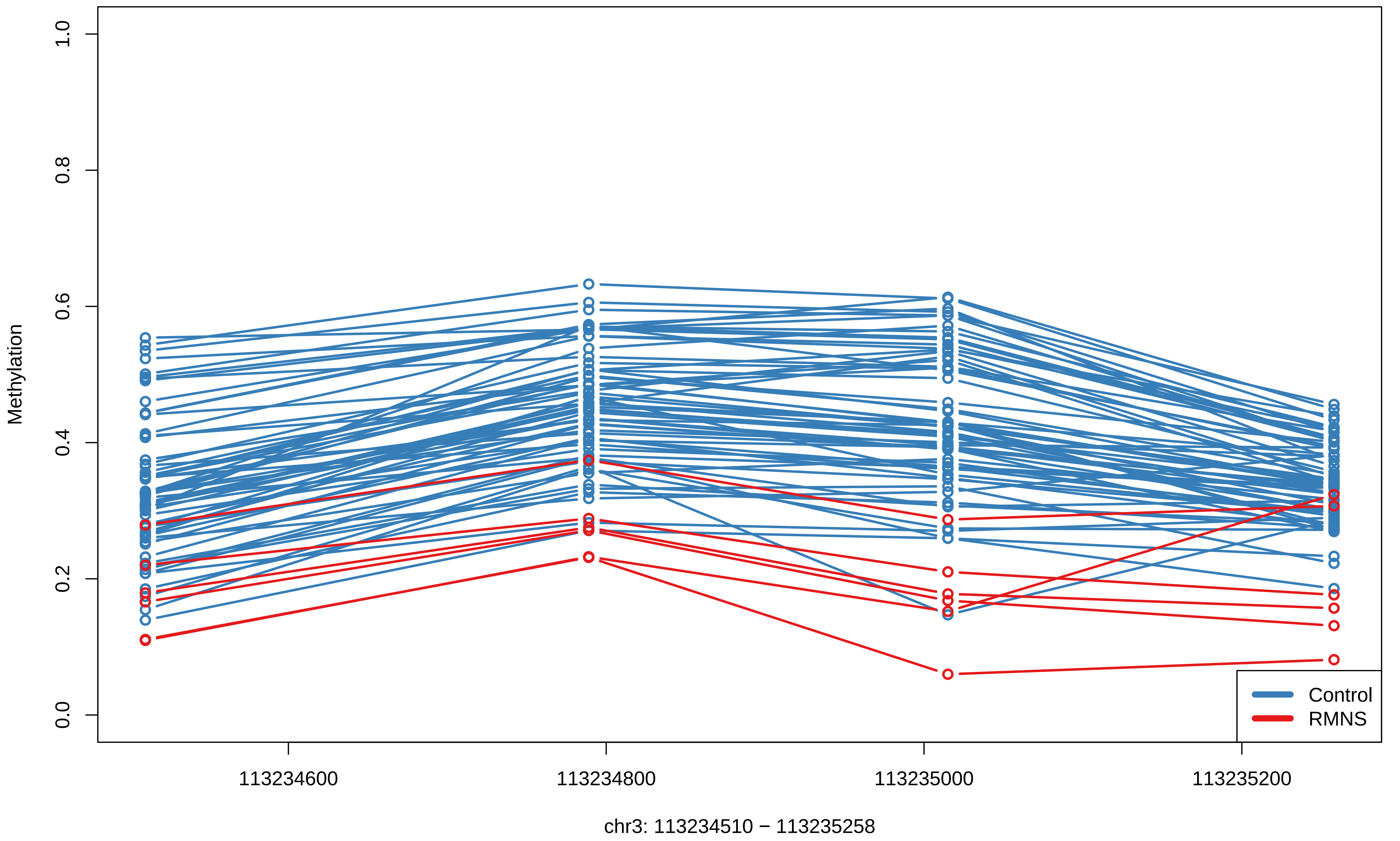

Methylation
0.0
0.2
0.4
0.6
0.8
1.0
113234600
113234800
113235000
113235200
chr3: 113234510 − 113235258
Control
RMNS

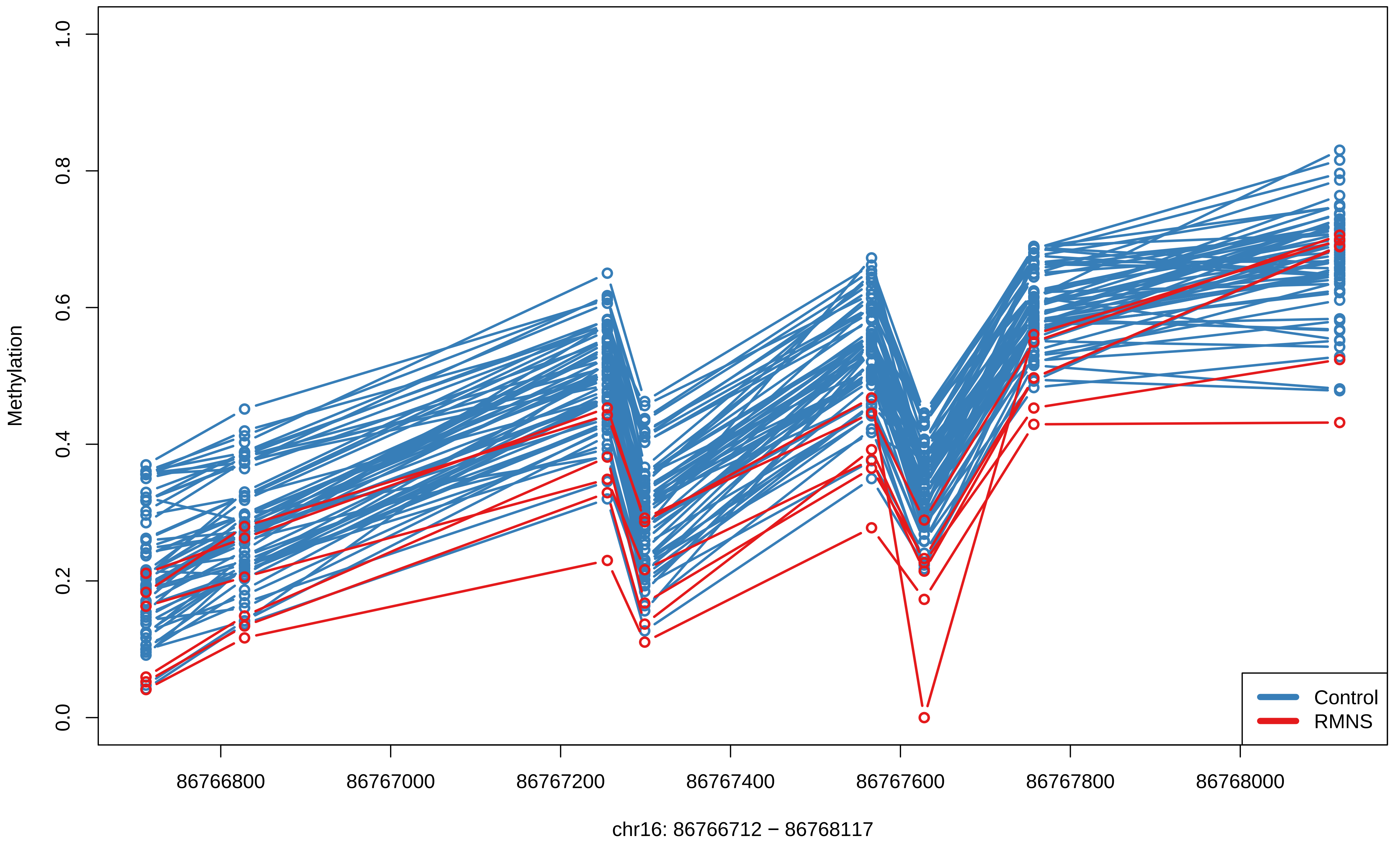

Methylation
1.0
0.8
0.6
0.4
0.2
0.0
86766800
86767000
86767200
86767400
86767600
86767800
86768000
chr16: 86766712 − 86768117
Control
RMNS

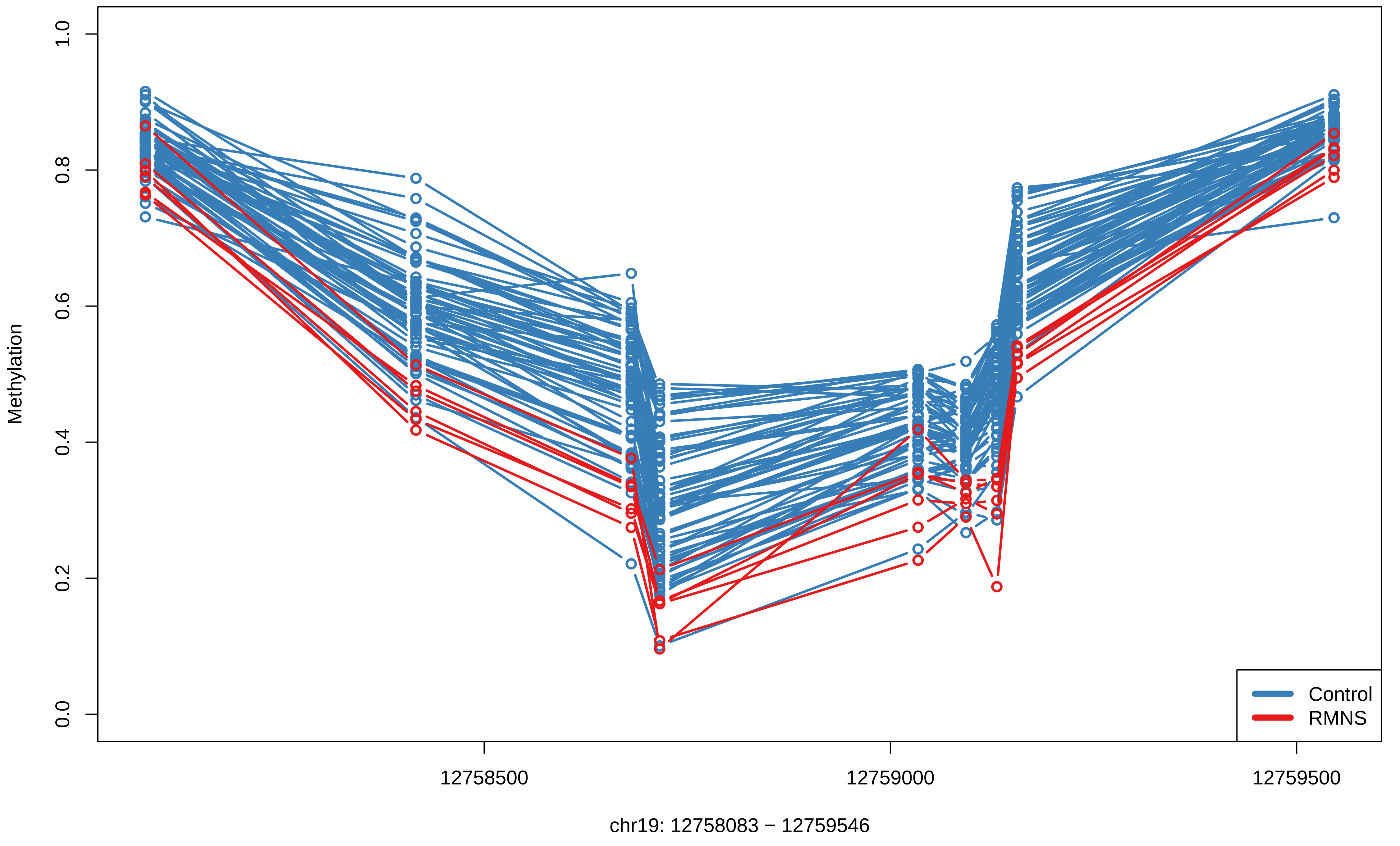

Methylation
0.0
0.2
0.4
0.6
0.8
1.0
12758500
12759000
12759500
chr19: 12758083 − 12759546
Control
RMNS

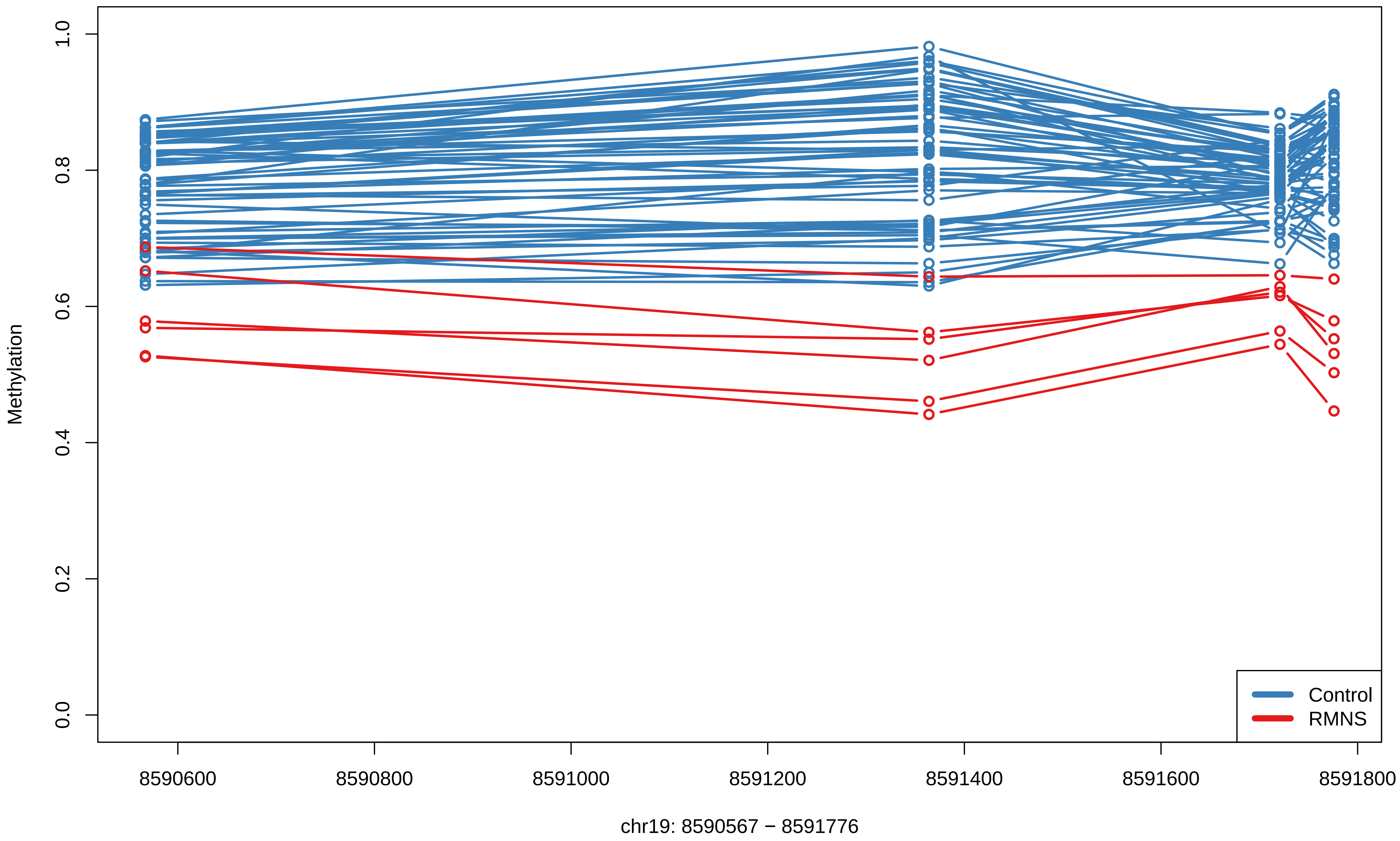

Methylation
1.0
0.8
0.6
0.4
0.2
0.0
8590600
8590800
8591000
8591200
8591400
8591600
8591800
chr19: 8590567 − 8591776
Control
RMNS

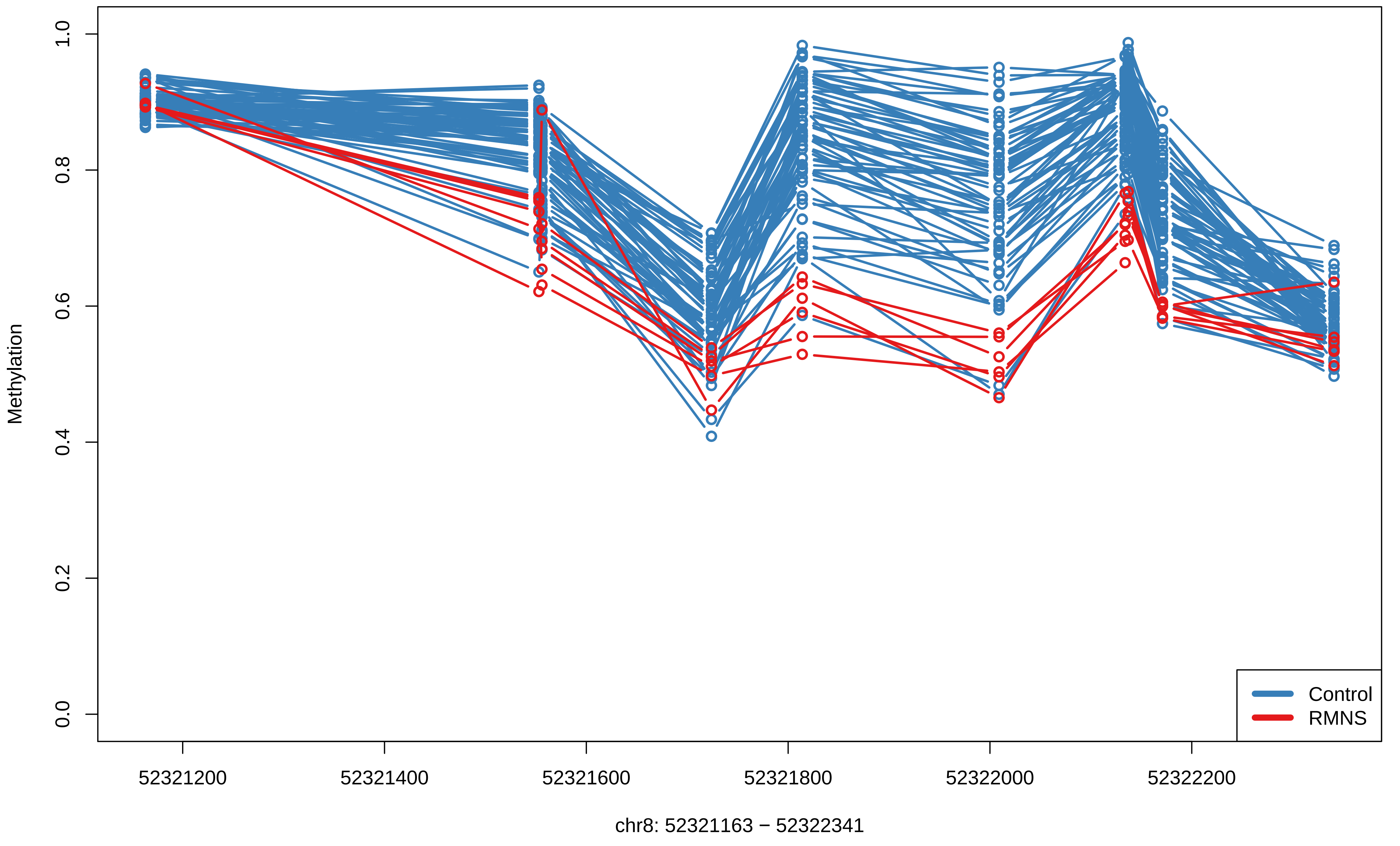

1.0
0.8
0.6
0.4
0.2
0.0
Methylation
52321200
52321400
52321600
52321800
52322000
52322200
chr8: 52321163 − 52322341
Control
RMNS

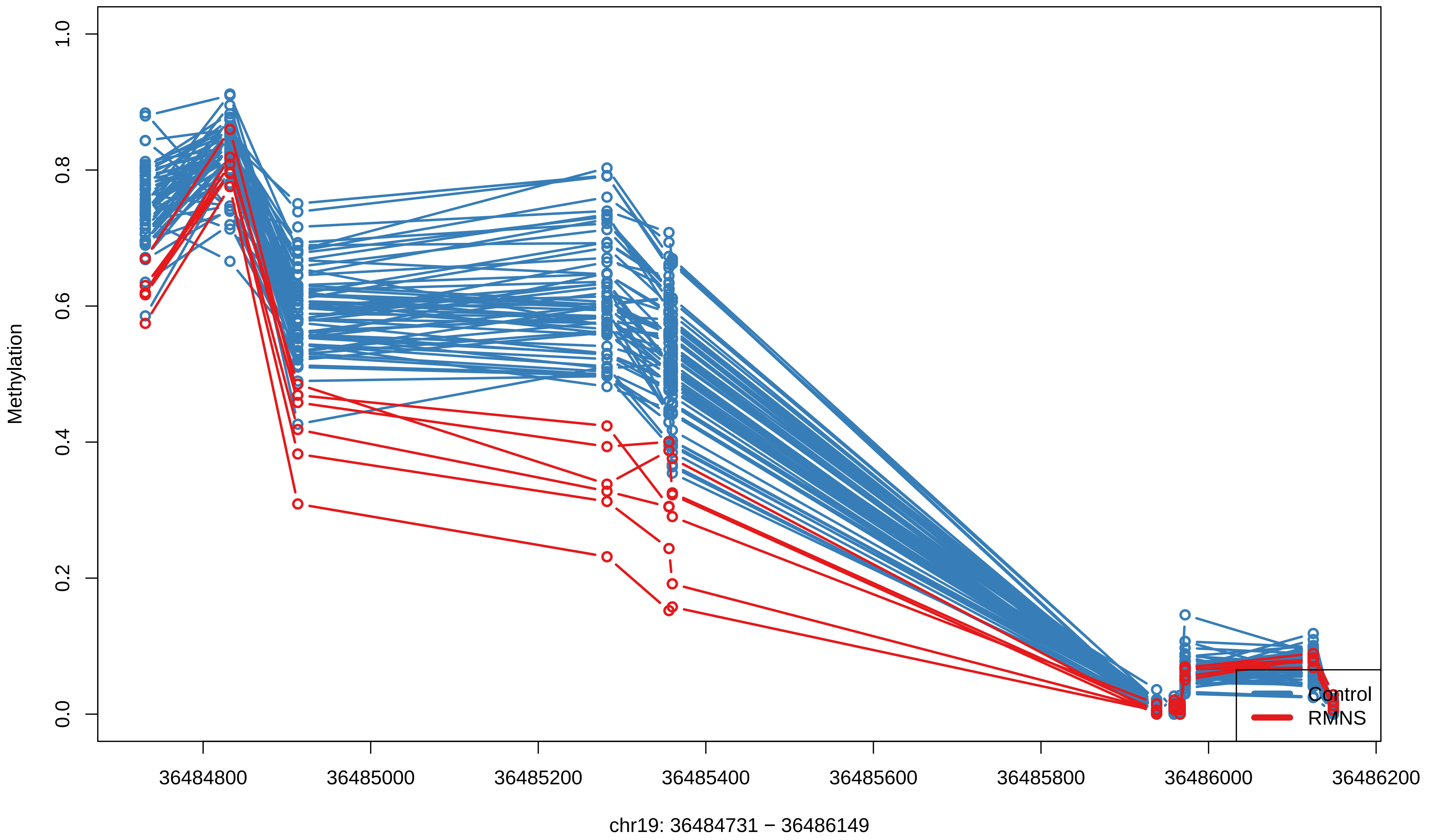

Methylation
1.0
0.8
0.6
0.4
0.2
0.0
36484800
36485000
36485200
36485400
36485600
36485800
36486000
36486200
chr19: 36484731 − 36486149
Control
RMNS

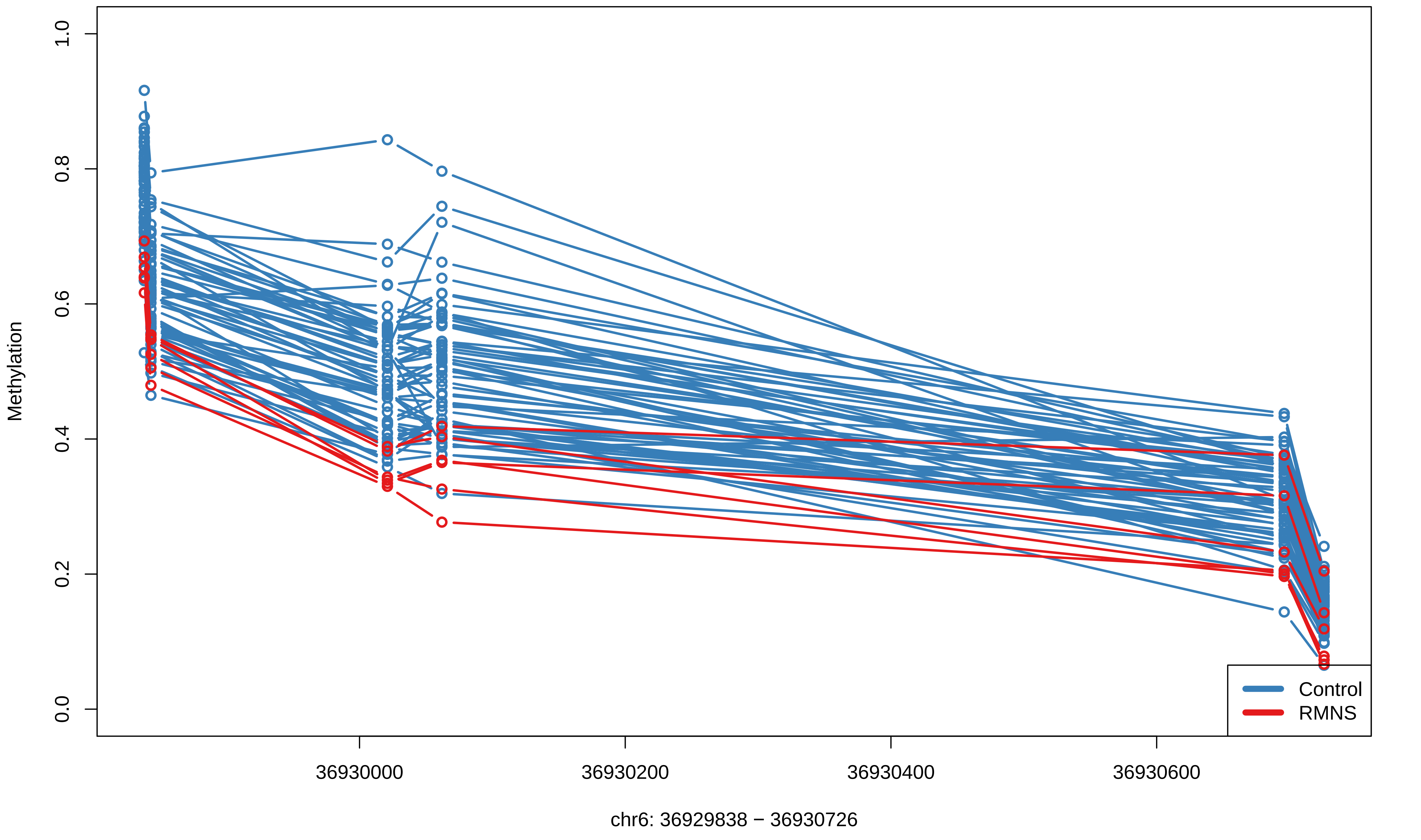

Methylation
0.0
0.2
0.4
0.6
0.8
1.0
36930000
36930200
36930400
36930600
chr6: 36929838 − 36930726
Control
RMNS

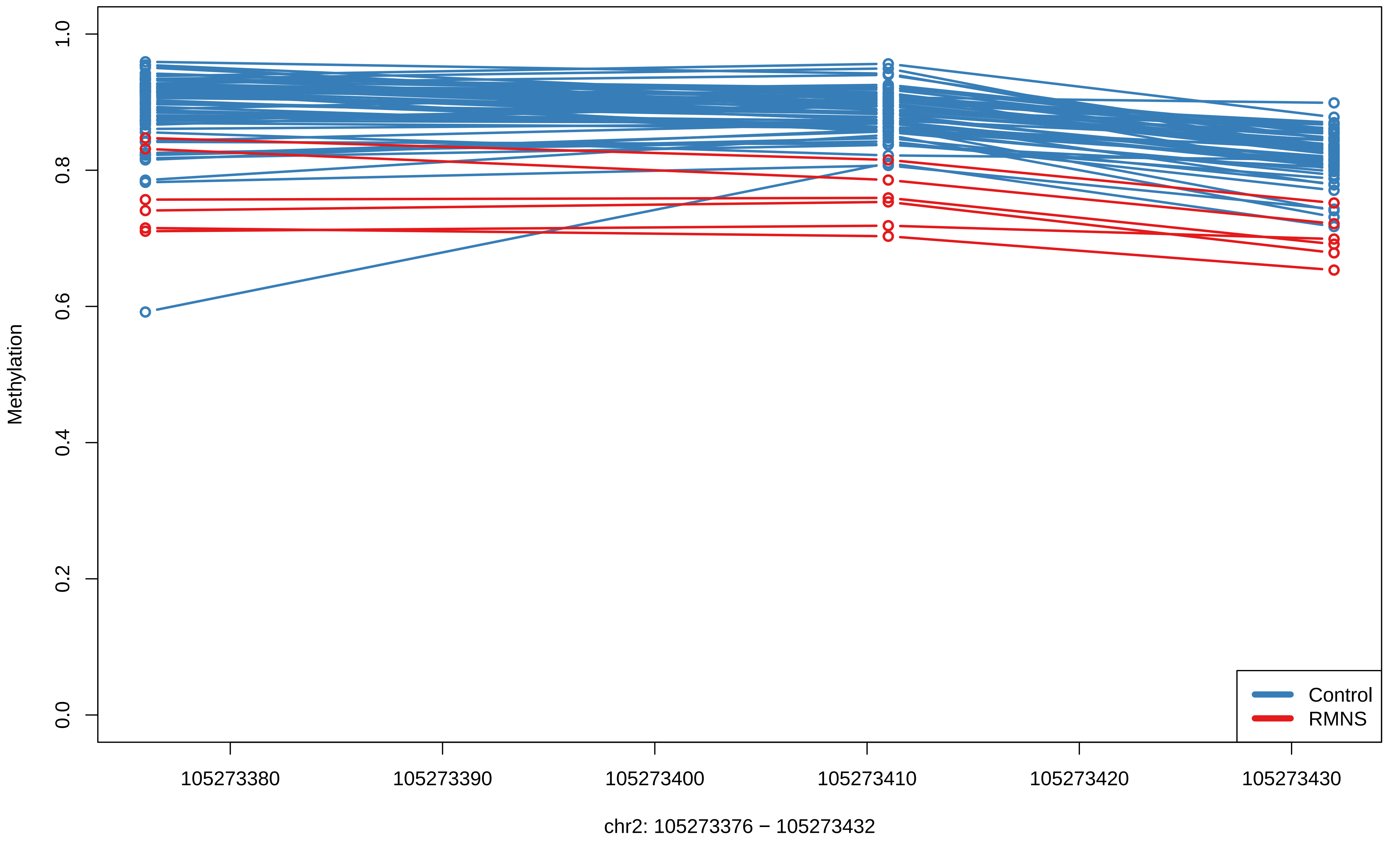

Methylation
0.0
0.2
0.4
0.6
0.8
1.0
105273380
105273390
105273400
105273410
105273420
105273430
chr2: 105273376 − 105273432
Control
RMNS

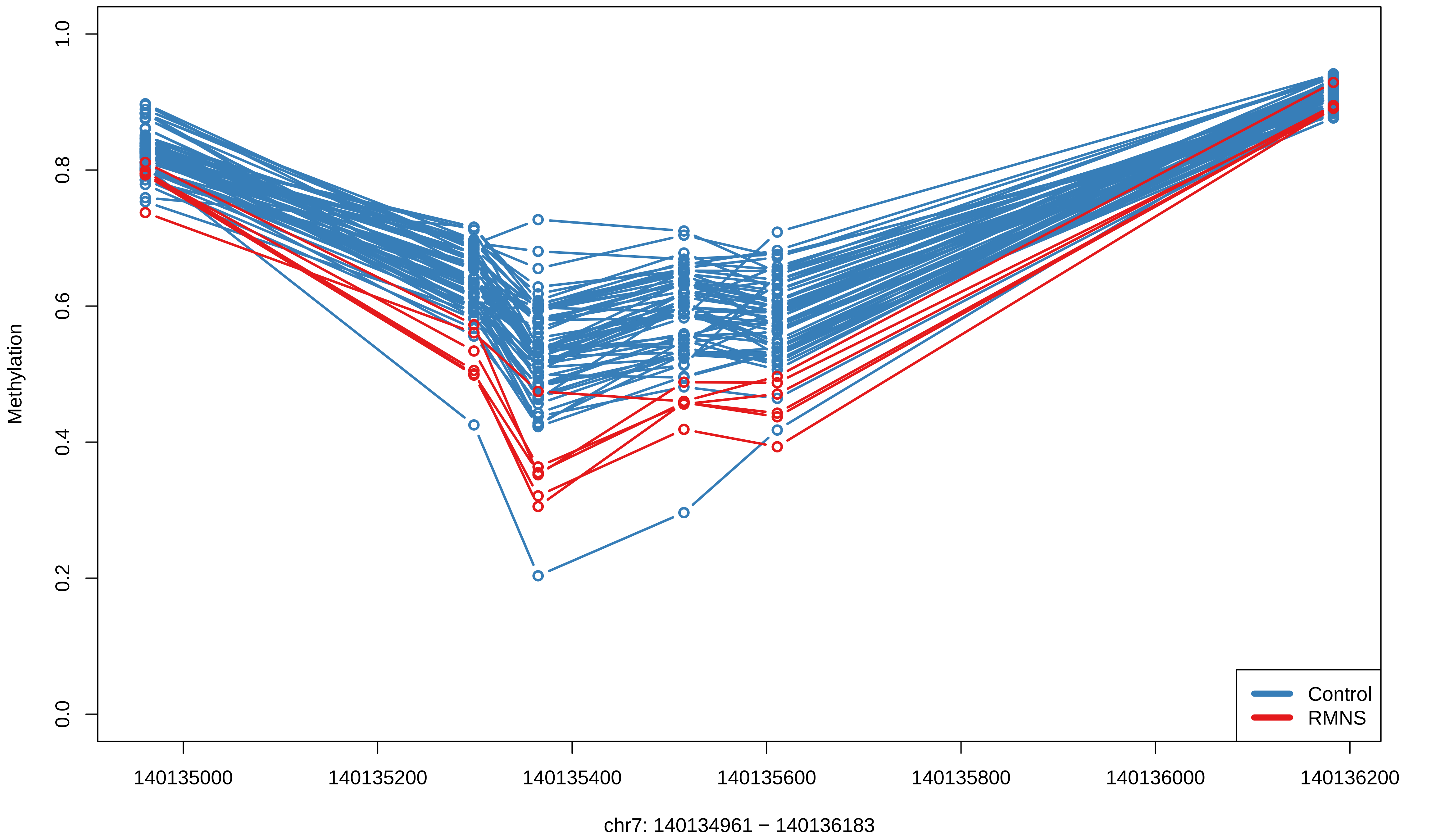

Methylation
1.0
0.8
0.6
0.4
0.2
0.0
140135000
140135200
140135400
140135600
140135800
140136000
140136200
chr7: 140134961 − 140136183
Control
RMNS

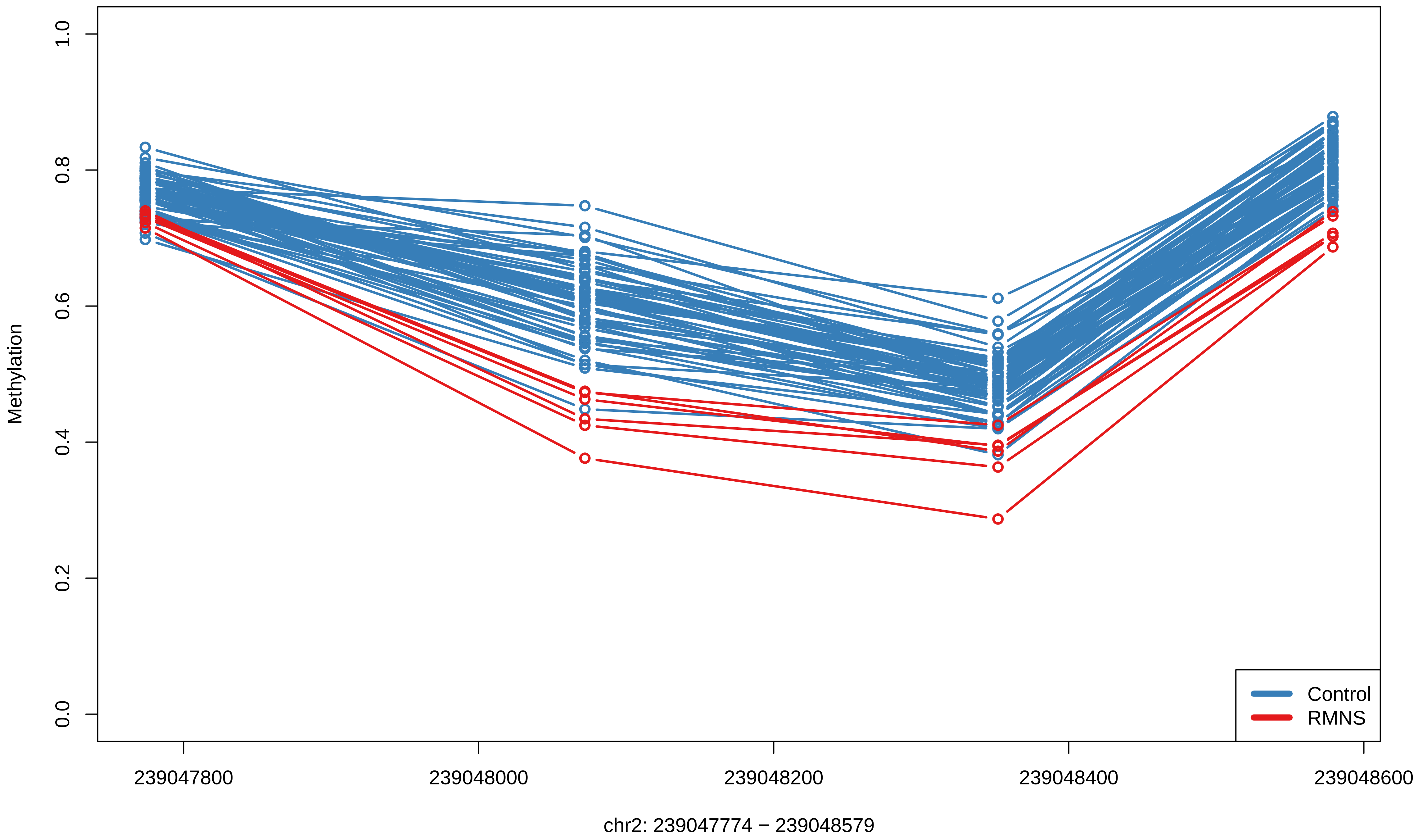

Methylation
0.0
0.2
0.4
0.6
0.8
1.0
239047800
239048000
239048200
239048400
239048600
chr2: 239047774 − 239048579
Control
RMNS

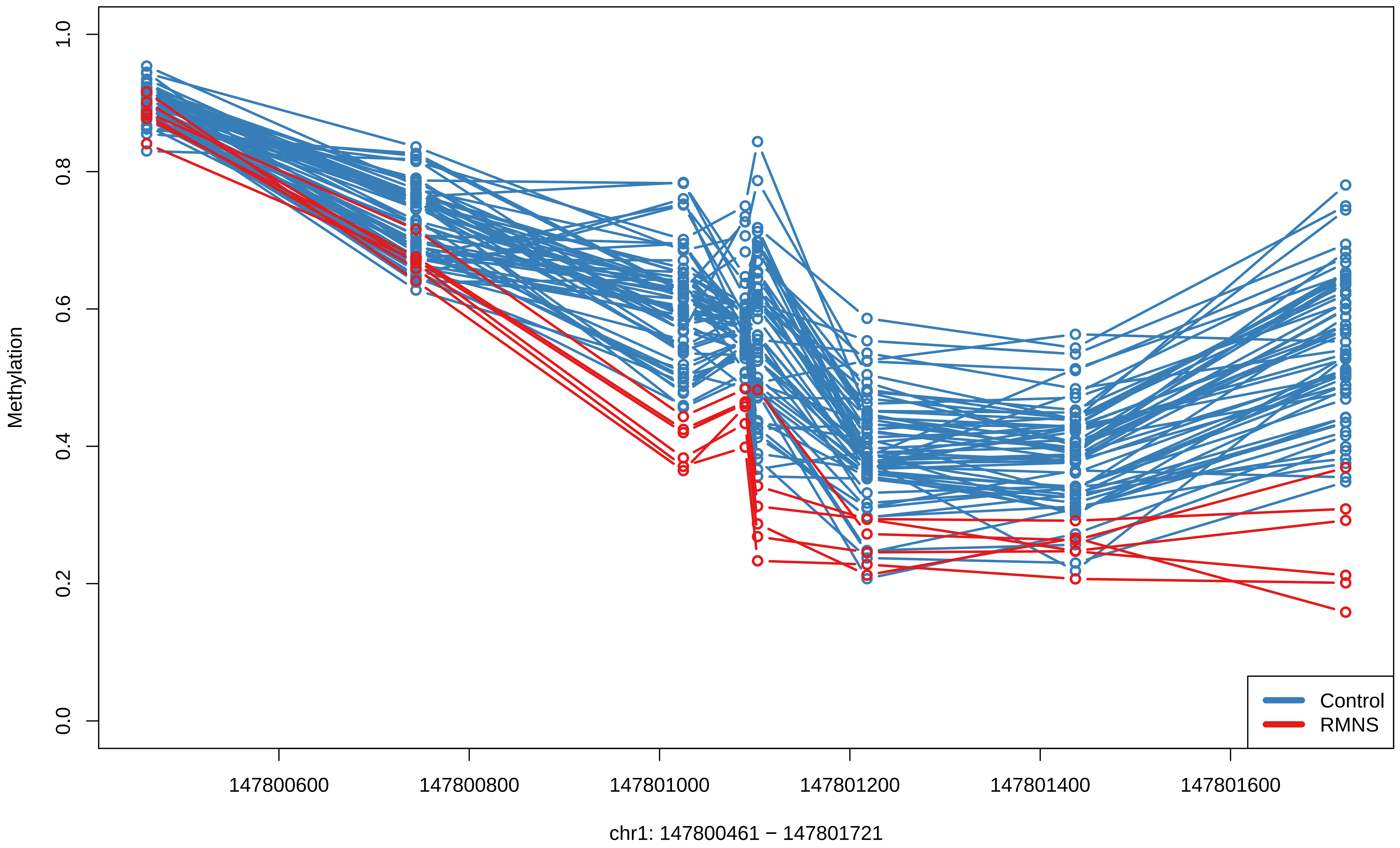

Methylation
1.0
0.8
0.6
0.4
0.2
0.0
147800600
147800800
147801000
147801200
147801400
147801600
chr1: 147800461 − 147801721
Control
RMNS

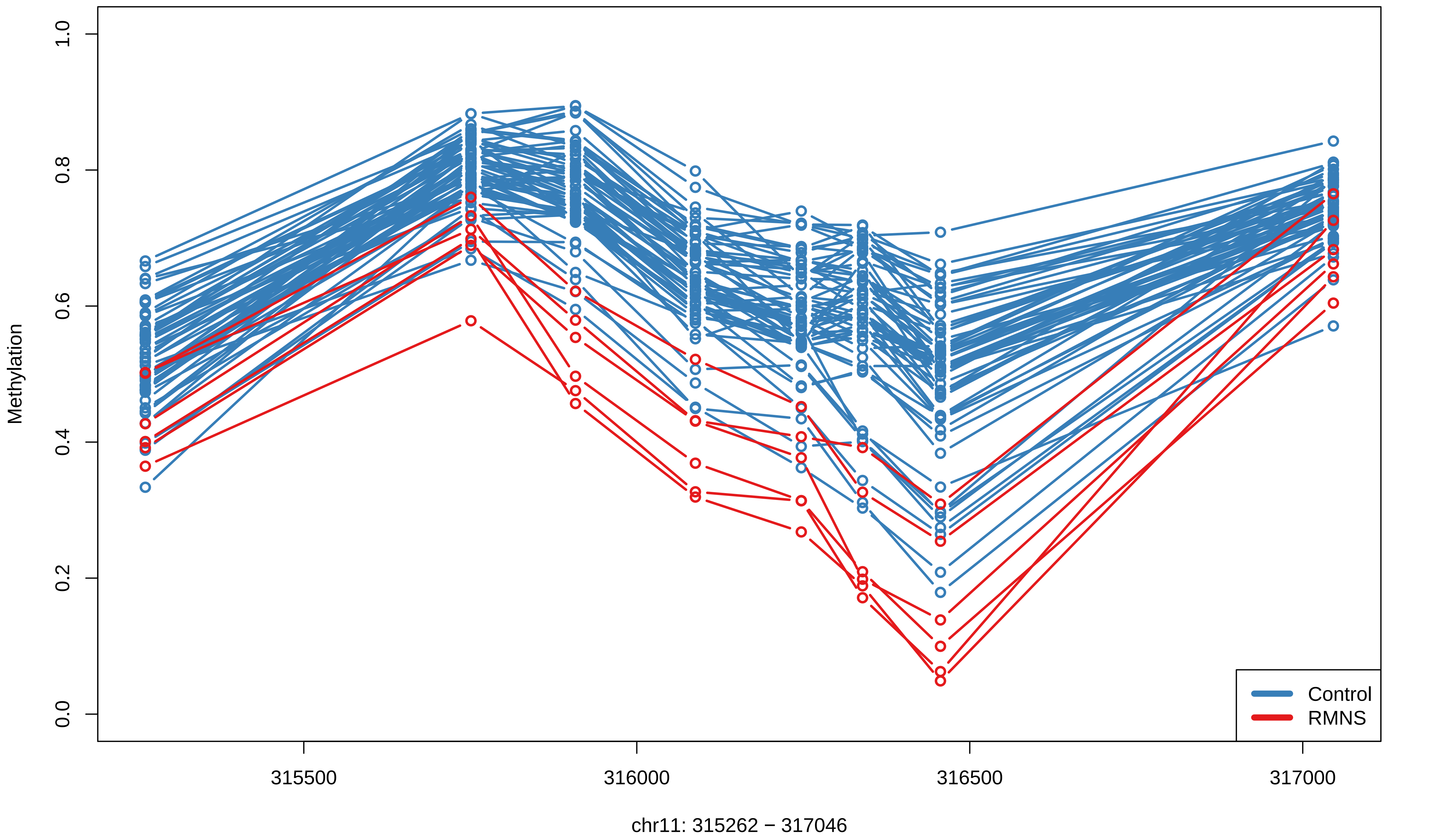
1.0
0.8
0.6
0.4
0.2
0.0
Methylation
315500
316000
316500
317000
chr11: 315262 − 317046
Control
RMNS

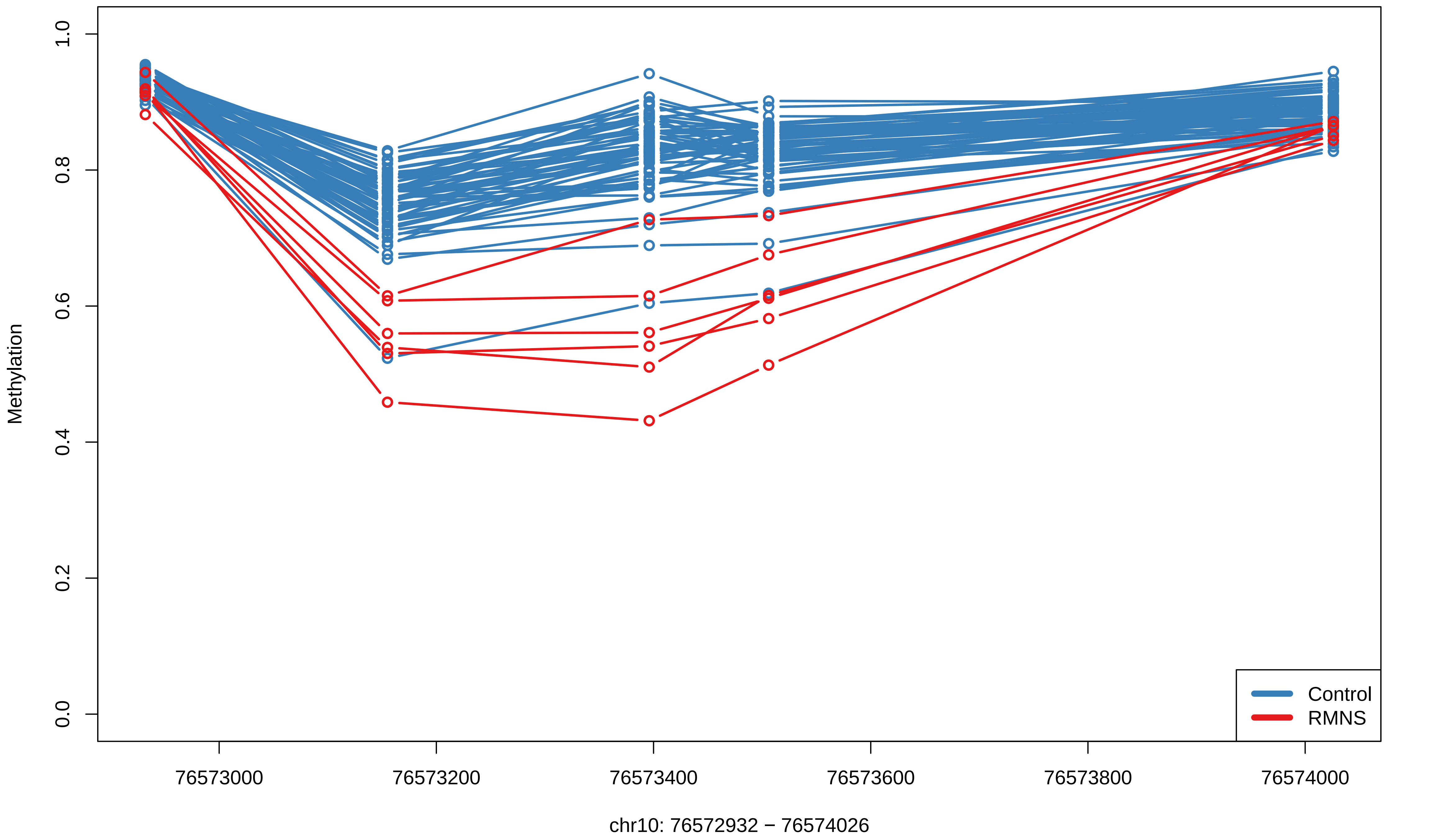

Methylation
0.0
0.2
0.4
0.6
0.8
1.0
76573000
76573200
76573400
76573600
76573800
76574000
chr10: 76572932 − 76574026
Control
RMNS

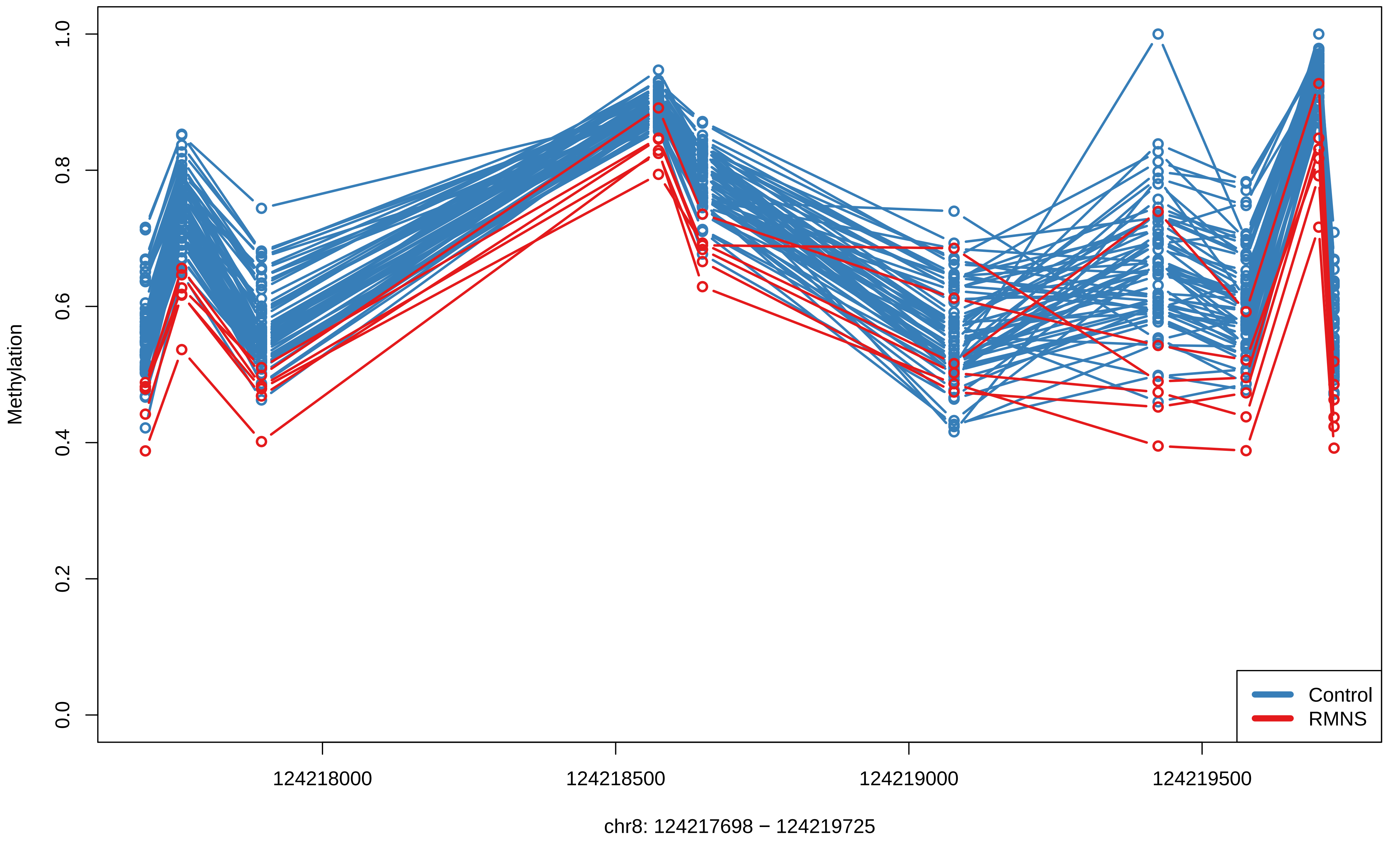
Methylation
1.0
0.8
0.6
0.4
0.2
0.0
124218000
124218500
124219000
124219500
chr8: 124217698 − 124219725
Control
RMNS

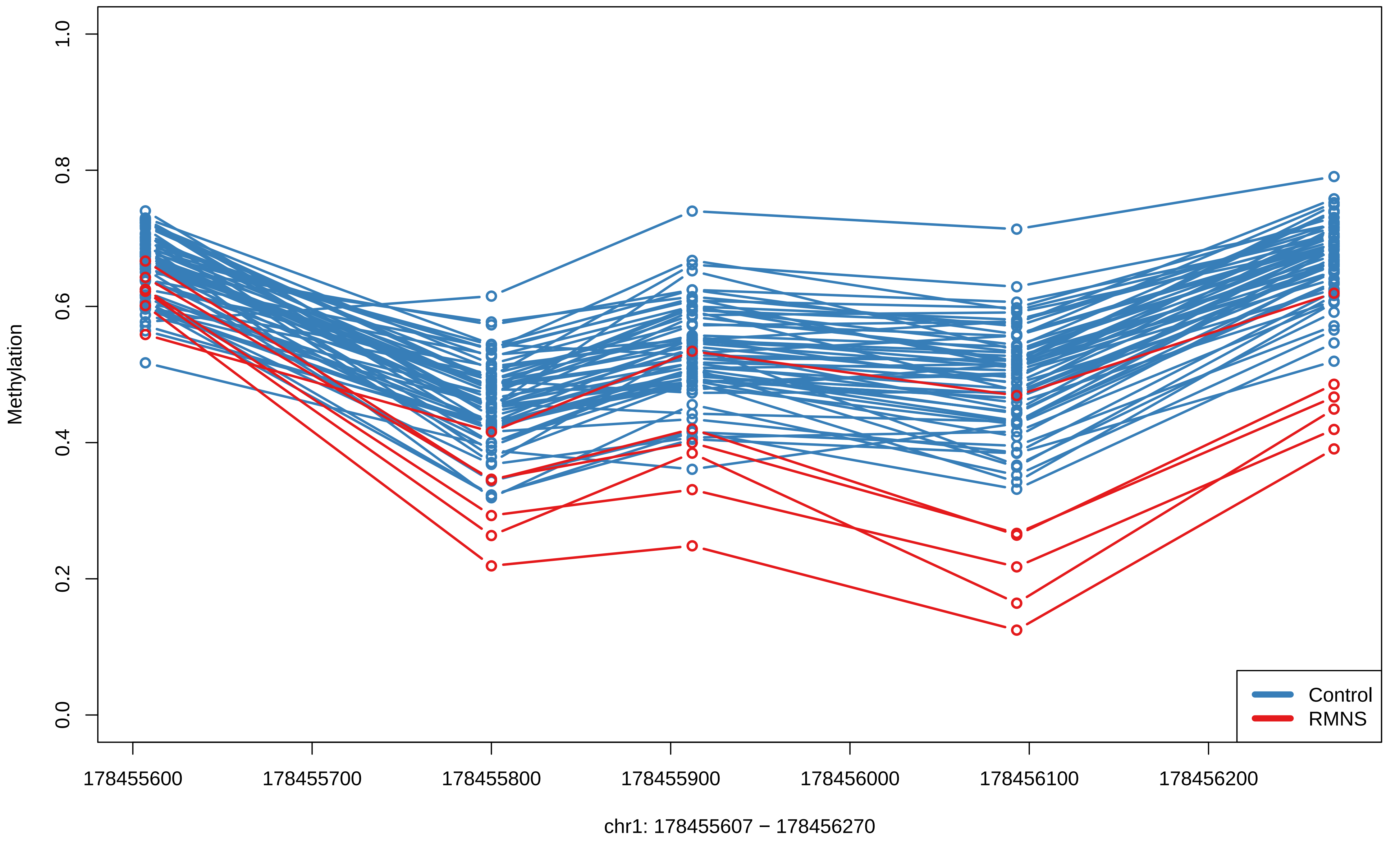

1.0
0.8
0.6
0.4
0.2
0.0
Methylation
178455600
178455700
178455800
178455900
178456000
178456100
178456200
chr1: 178455607 − 178456270
Control
RMNS

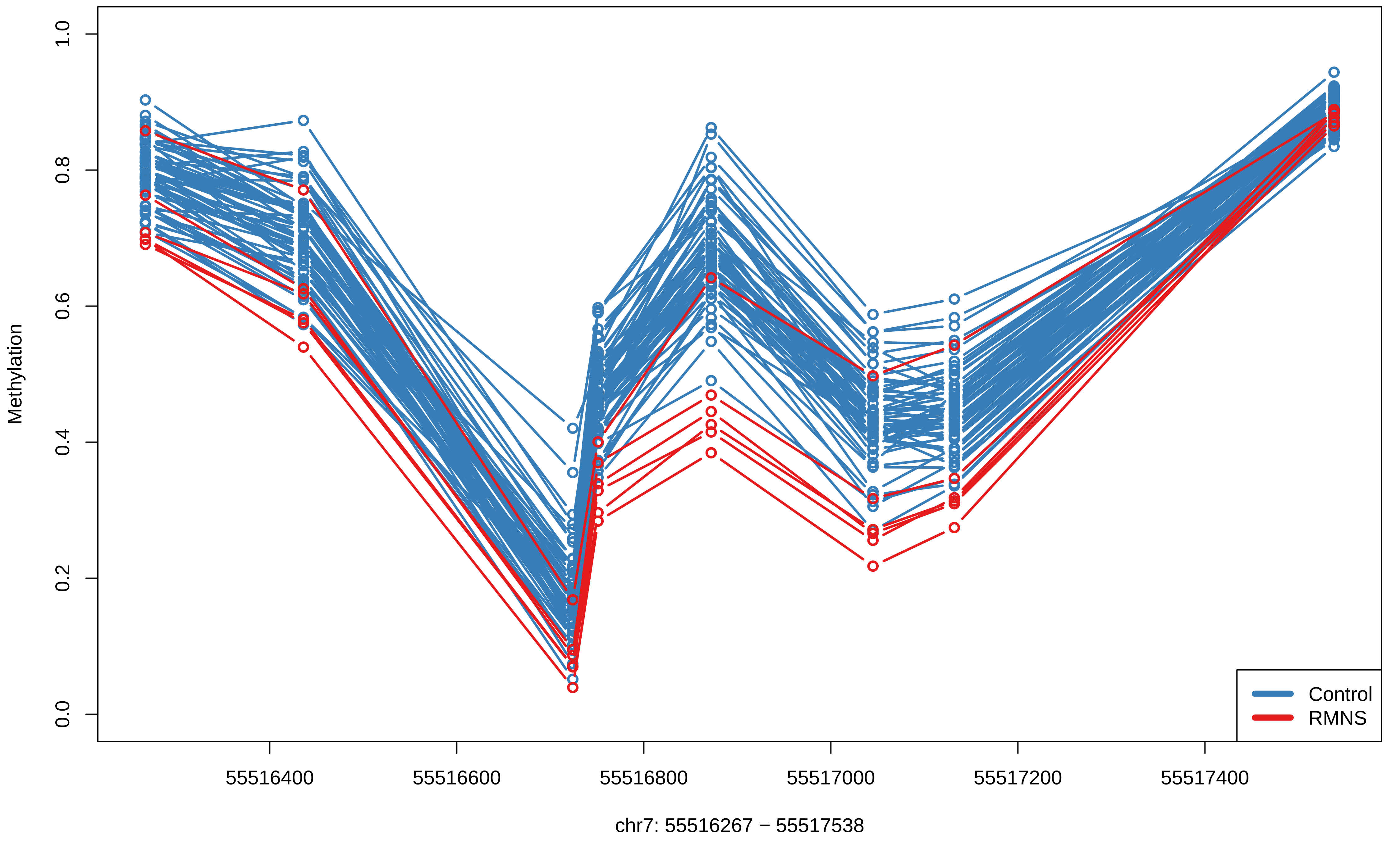

Methylation
0.0
0.2
0.4
0.6
0.8
1.0
55516400
55516600
55516800
55517000
55517200
55517400
chr7: 55516267 − 55517538
Control
RMNS

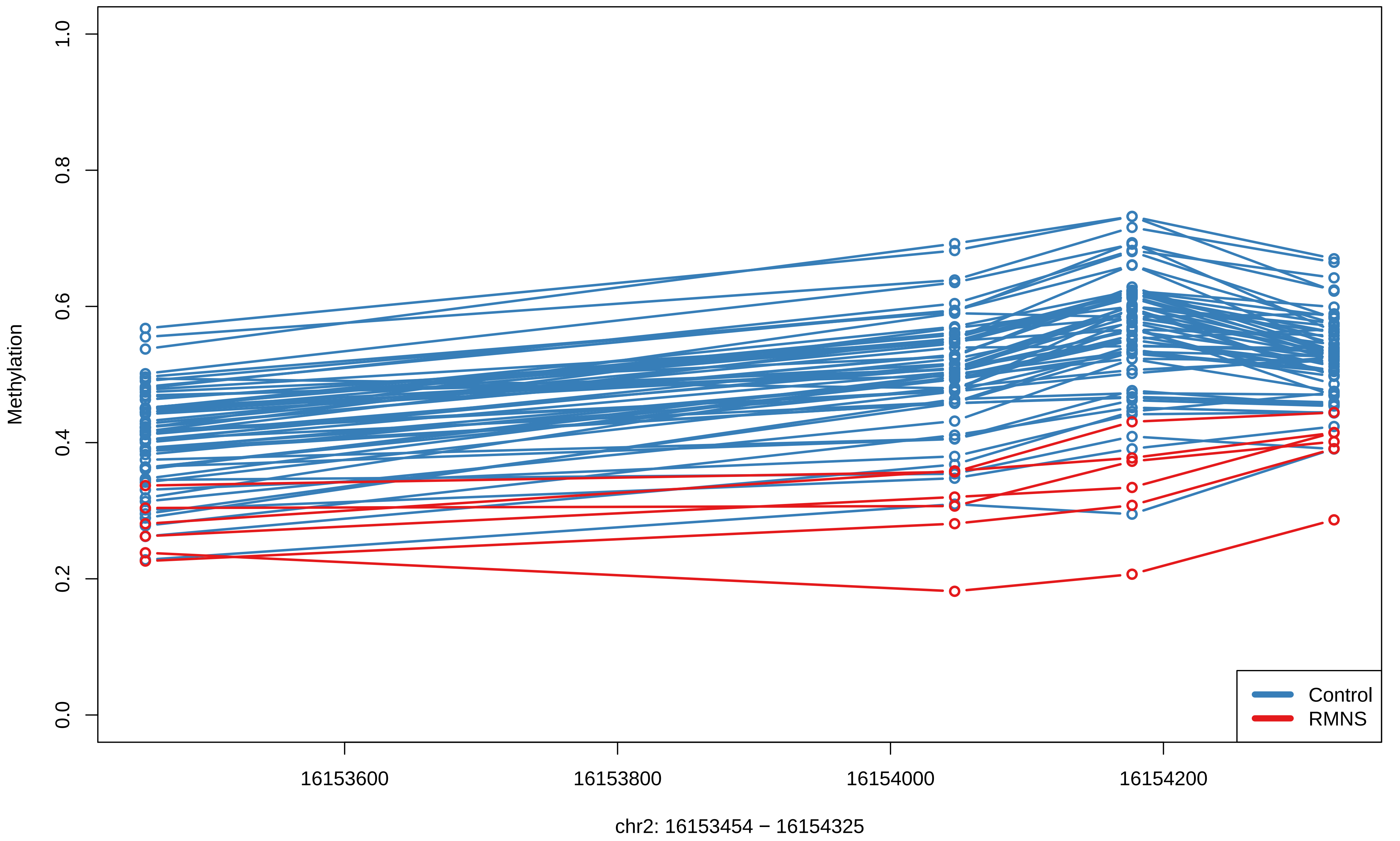

Methylation
0.0
0.2
0.4
0.6
0.8
1.0
16153600
16153800
16154000
16154200
chr2: 16153454 − 16154325
Control
RMNS

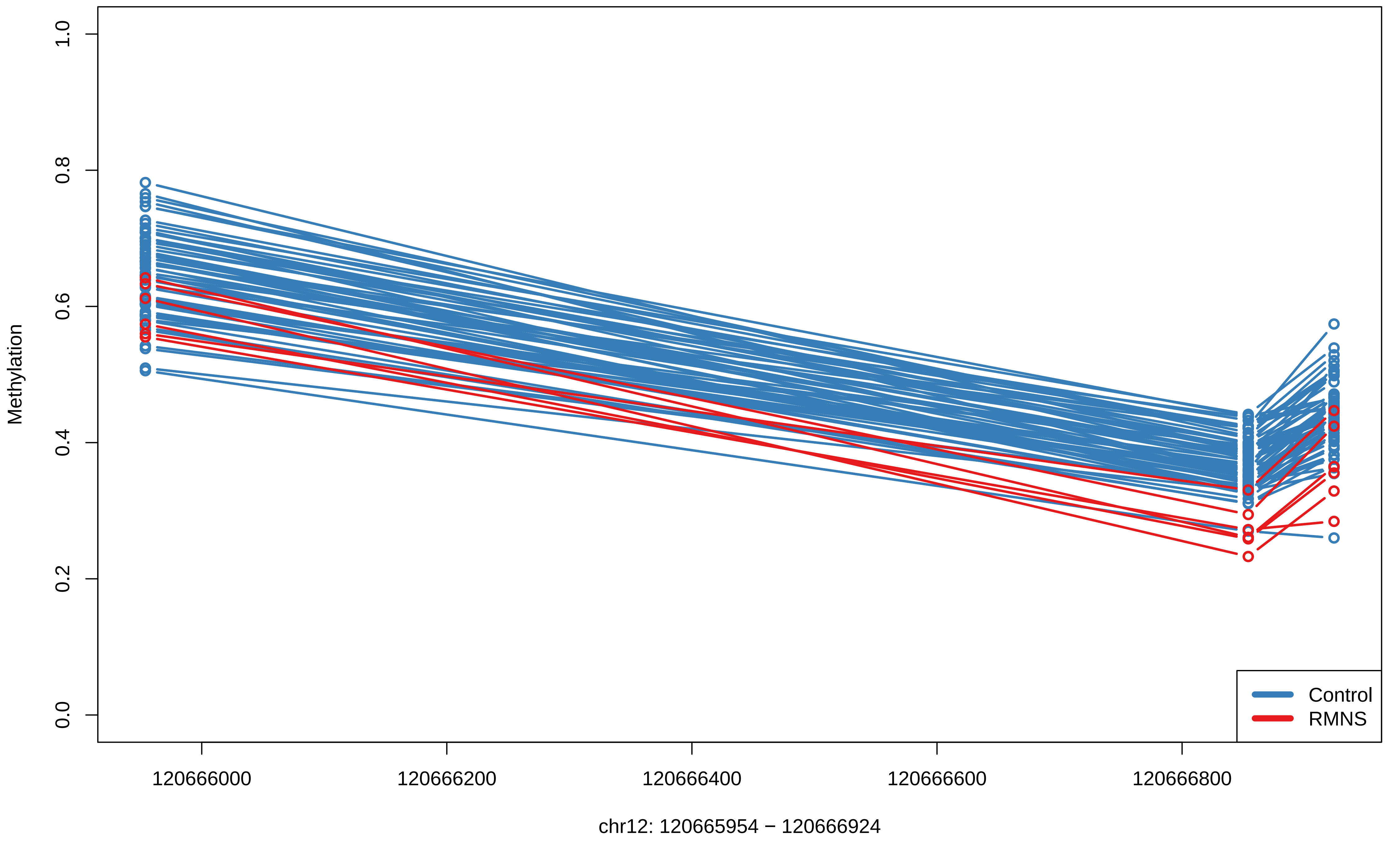

Methylation
1.0
0.8
0.6
0.4
0.2
0.0
120666000
120666200
120666400
120666600
120666800
chr12: 120665954 − 120666924
Control
RMNS

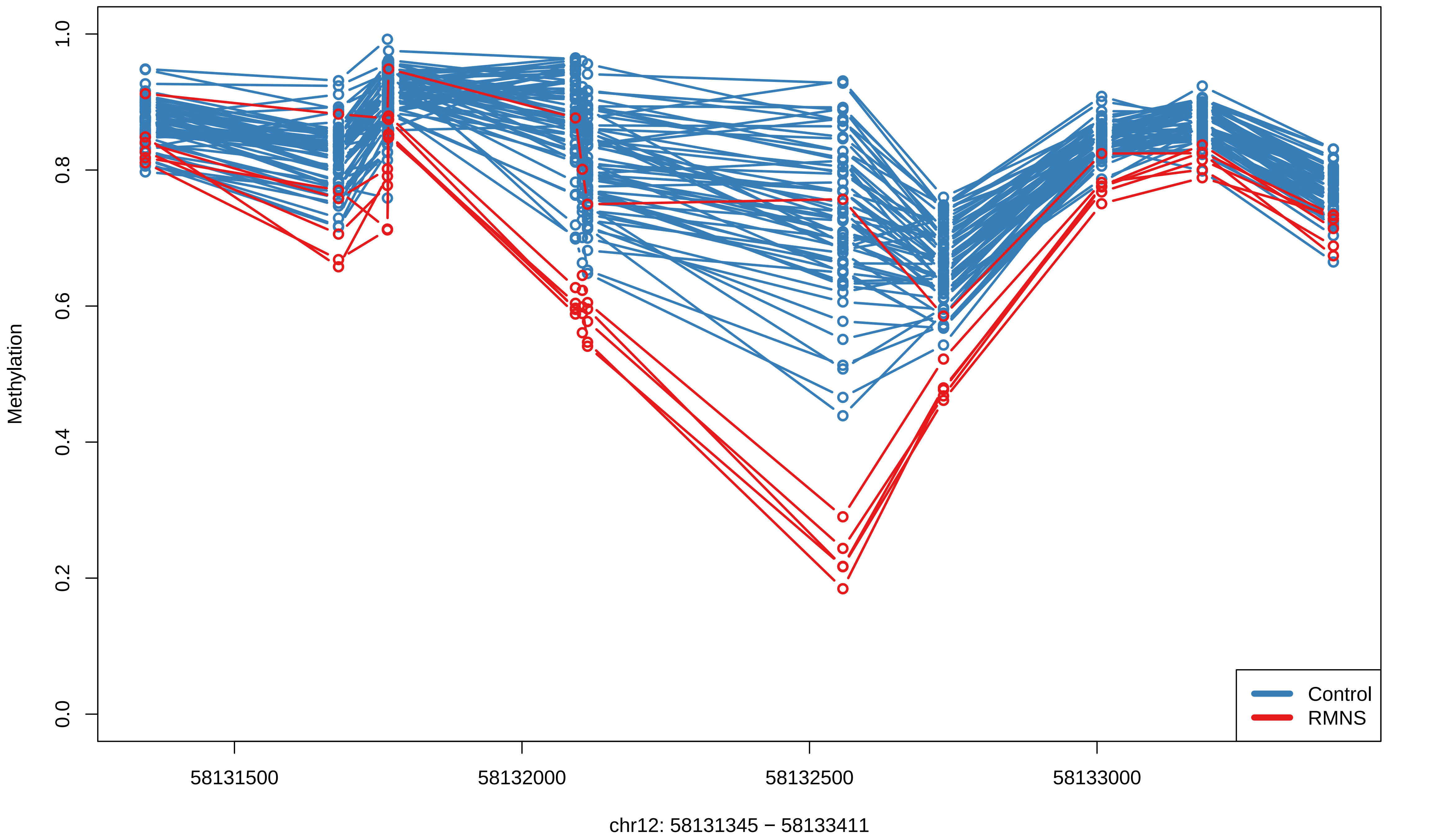

Methylation
1.0
0.8
0.6
0.4
0.2
0.0
58131500
58132000
58132500
58133000
chr12: 58131345 − 58133411
Control
RMNS

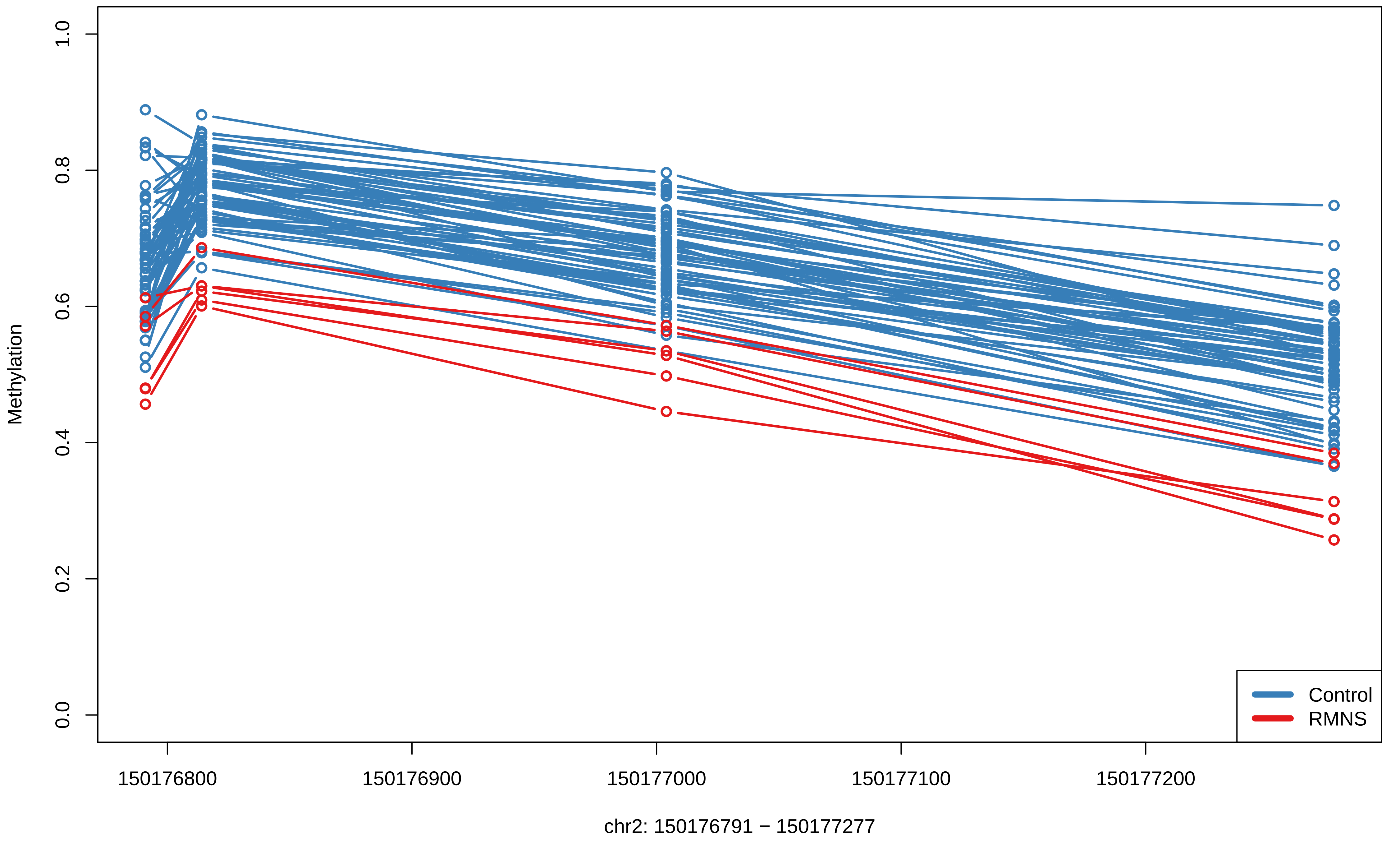

Methylation
0.0
0.2
0.4
0.6
0.8
1.0
150176800
150176900
150177000
150177100
150177200
chr2: 150176791 − 150177277
Control
RMNS

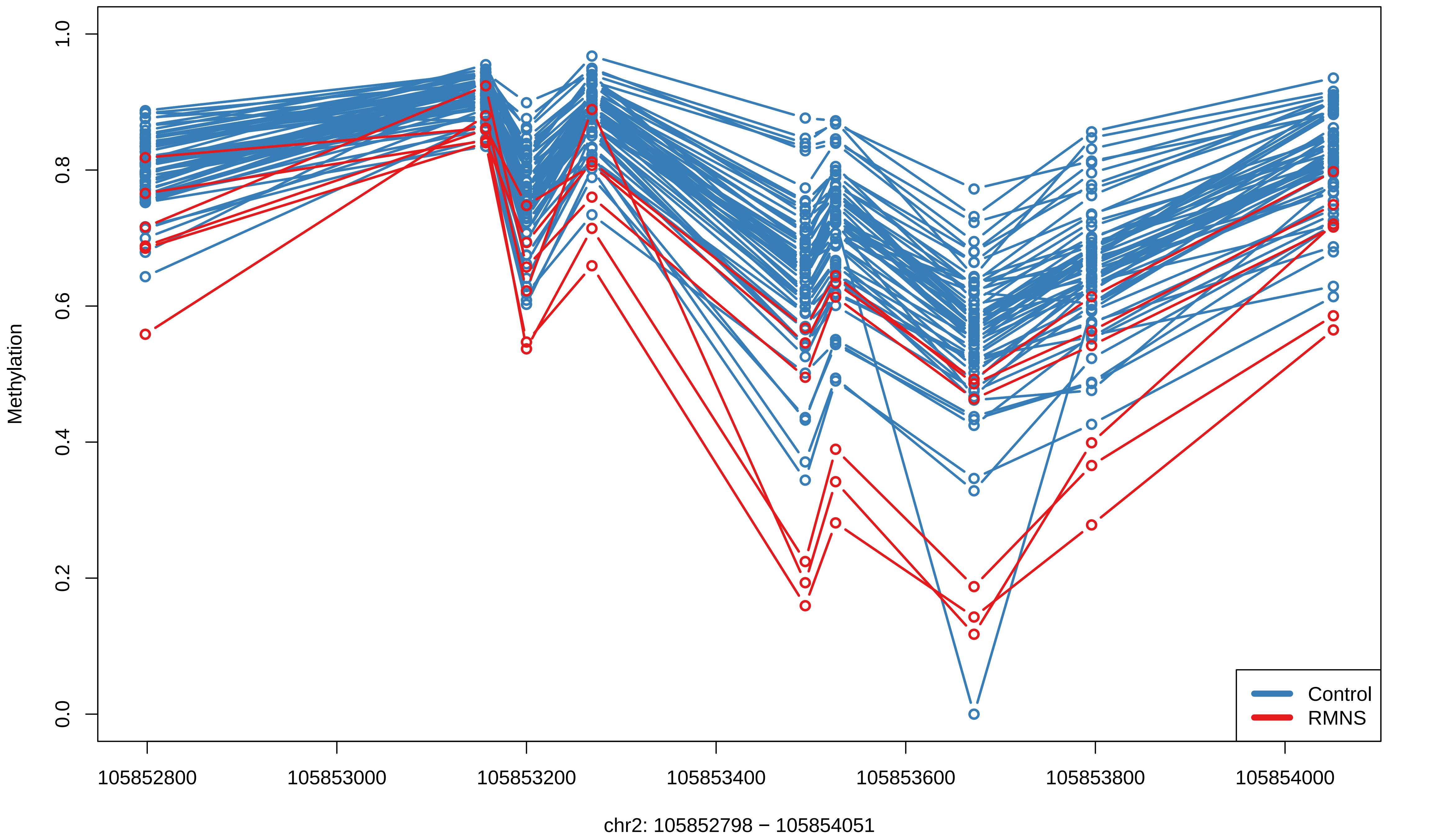

Methylation
1.0
0.8
0.6
0.4
0.2
0.0
105852800
105853000
105853200
105853400
105853600
105853800
105854000
chr2: 105852798 − 105854051
Control
RMNS

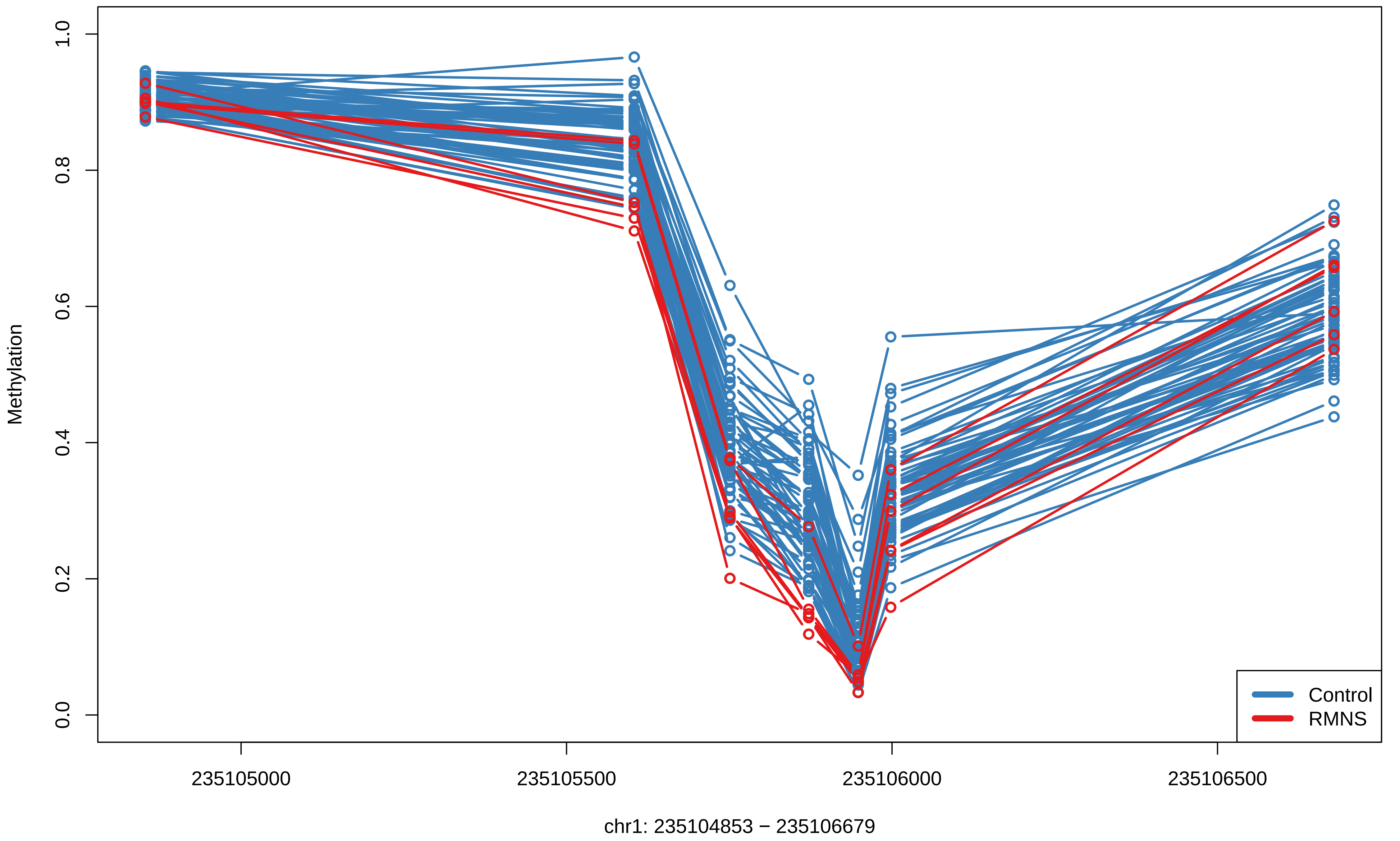

Methylation
1.0
0.8
0.6
0.4
0.2
0.0
235105000
235105500
235106000
235106500
chr1: 235104853 − 235106679
Control
RMNS

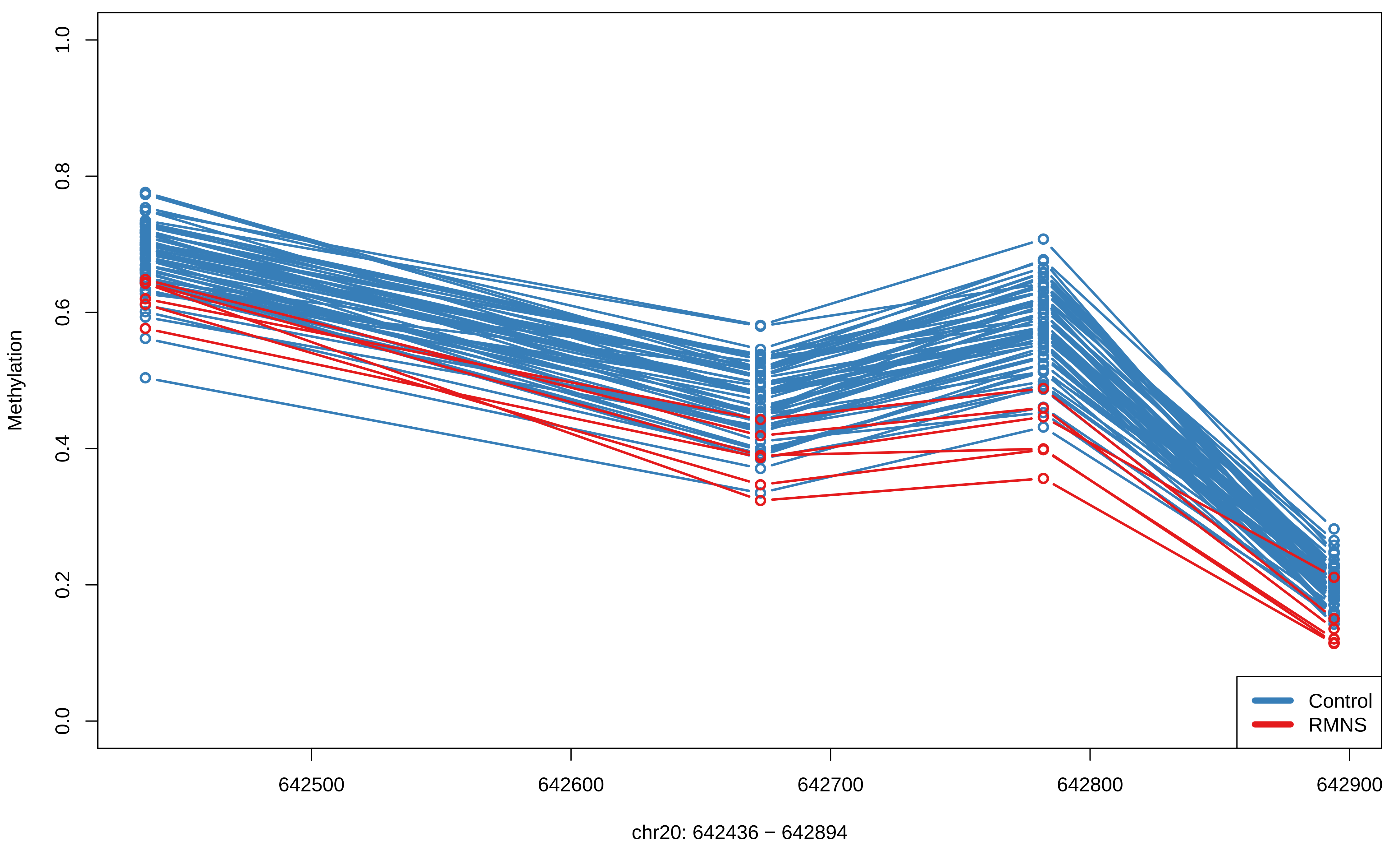

Methylation
0.0
0.2
0.4
0.6
0.8
1.0
642500
642600
642700
642800
642900
chr20: 642436 − 642894
Control
RMNS

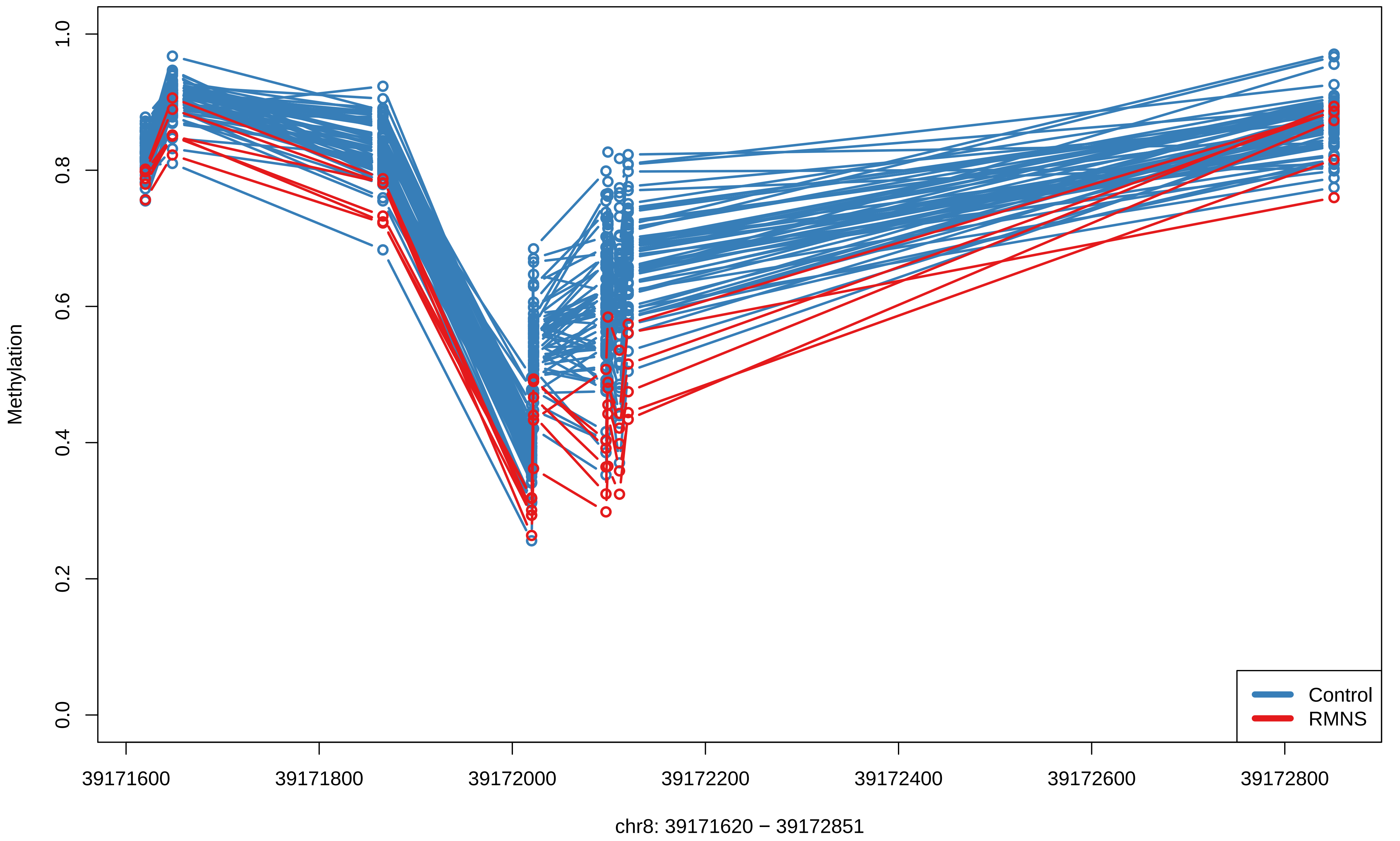
Methylation
1.0
0.8
0.6
0.4
0.2
0.0
39171600
39171800
39172000
39172200
39172400
39172600
39172800
chr8: 39171620 − 39172851
Control
RMNS

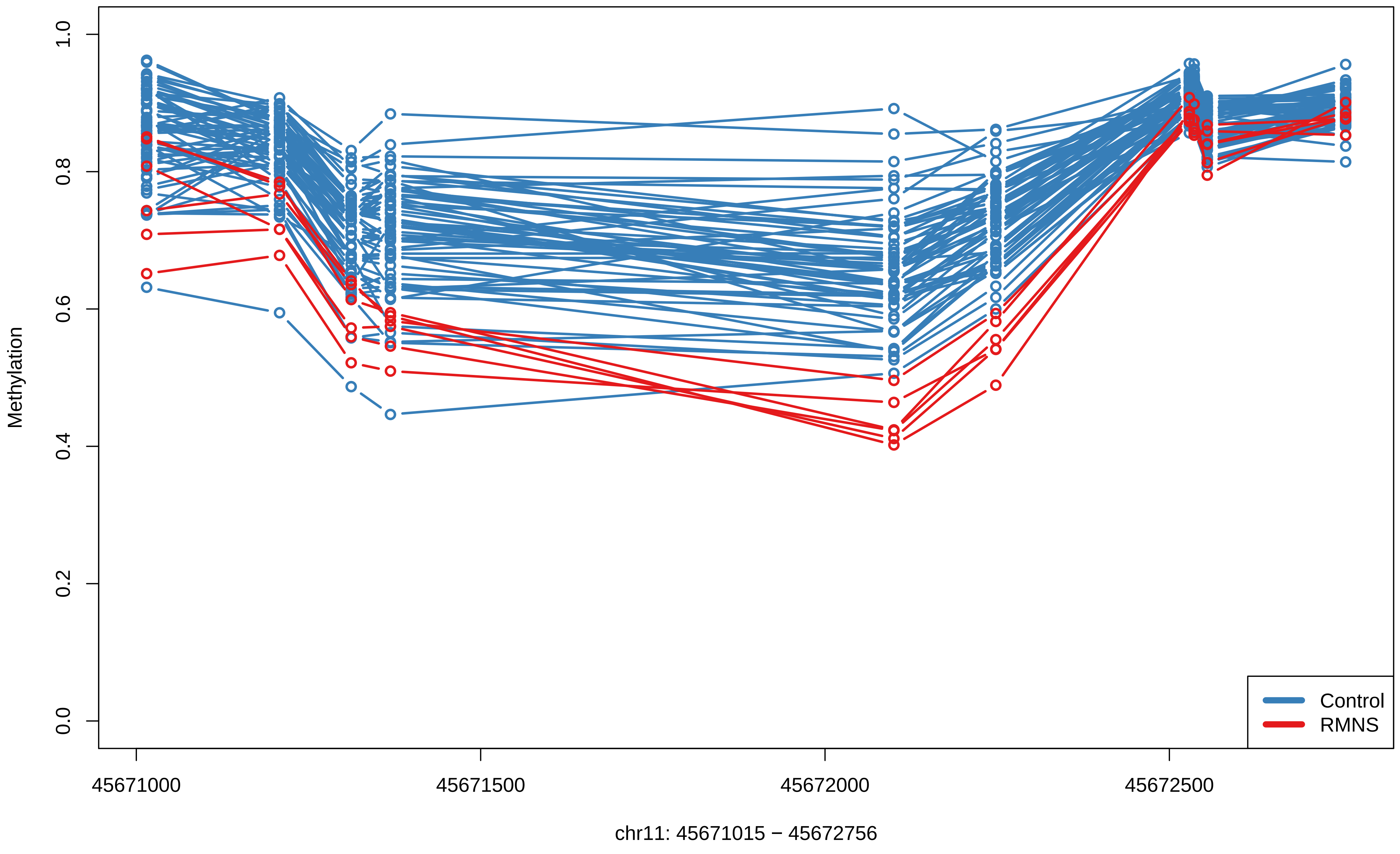

Methylation
1.0
0.8
0.6
0.4
0.2
0.0
45671000
45671500
45672000
45672500
chr11: 45671015 − 45672756
Control
RMNS

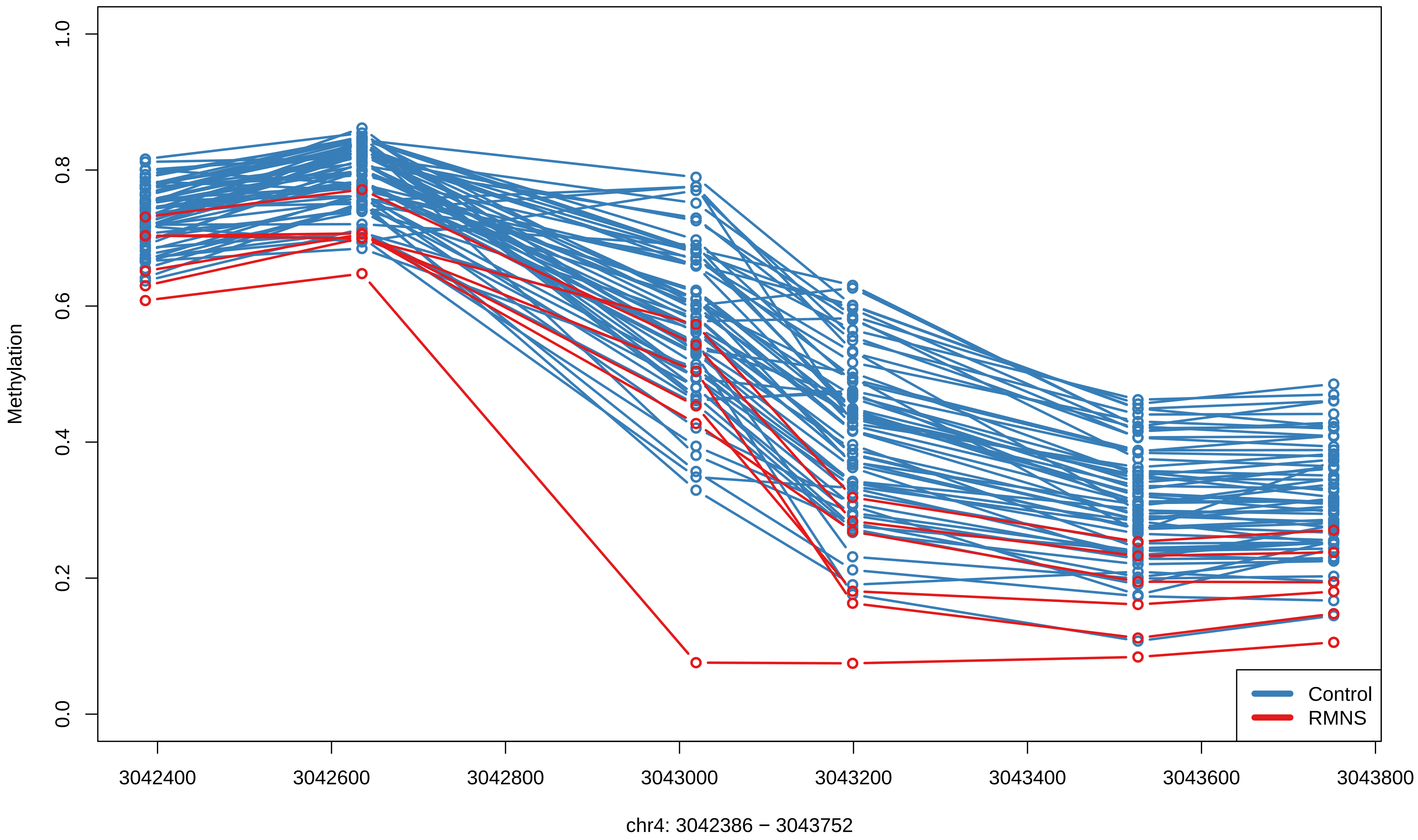

Methylation
0.0
0.2
0.4
0.6
0.8
1.0
3042400
3042600
3042800
3043000
3043200
3043400
3043600
3043800
chr4: 3042386 − 3043752
Control
RMNS

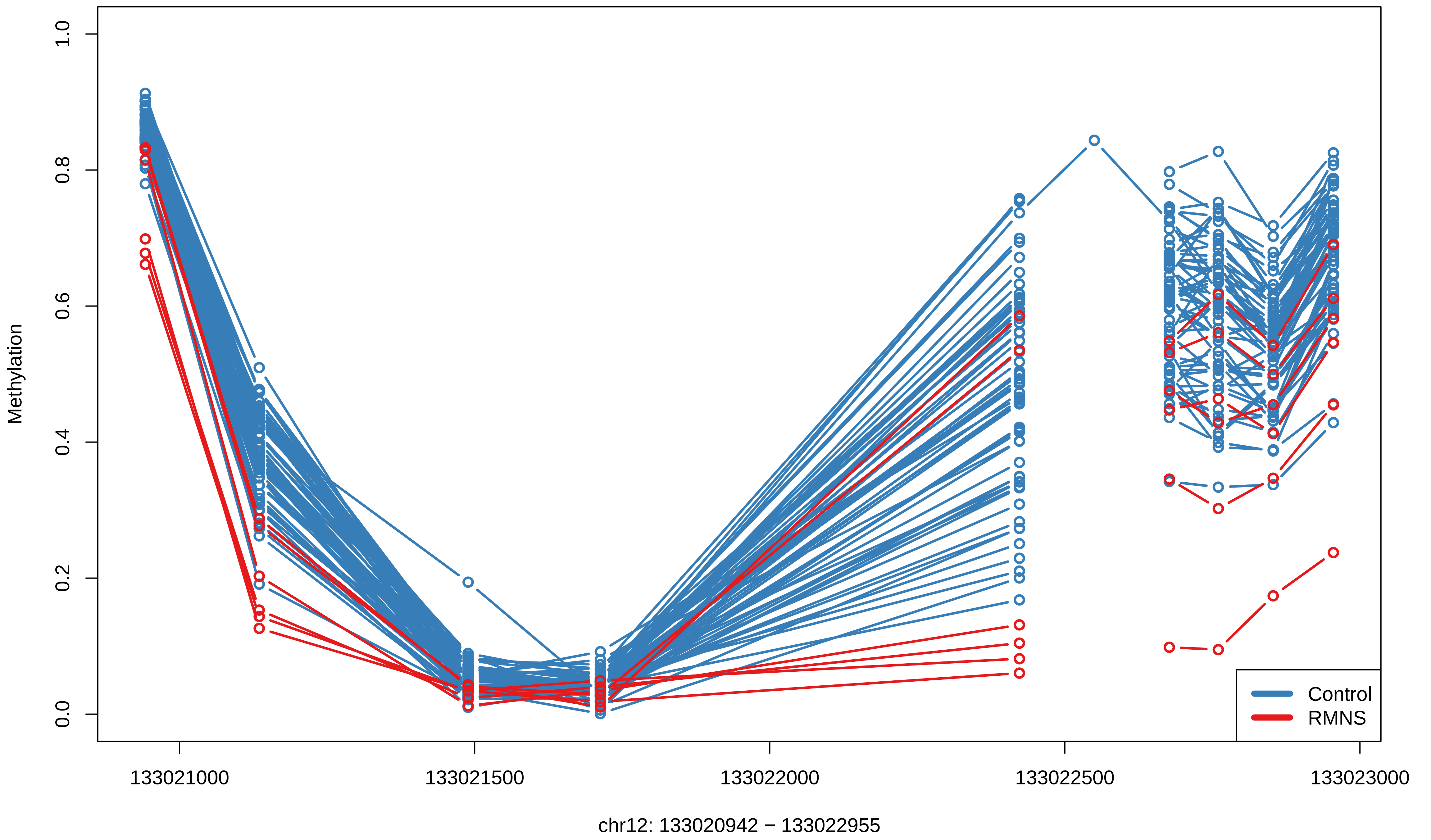

1.0
0.8
0.6
0.4
0.2
0.0
Methylation
133021000
133021500
133022000
133022500
133023000
chr12: 133020942 − 133022955
Control
RMNS

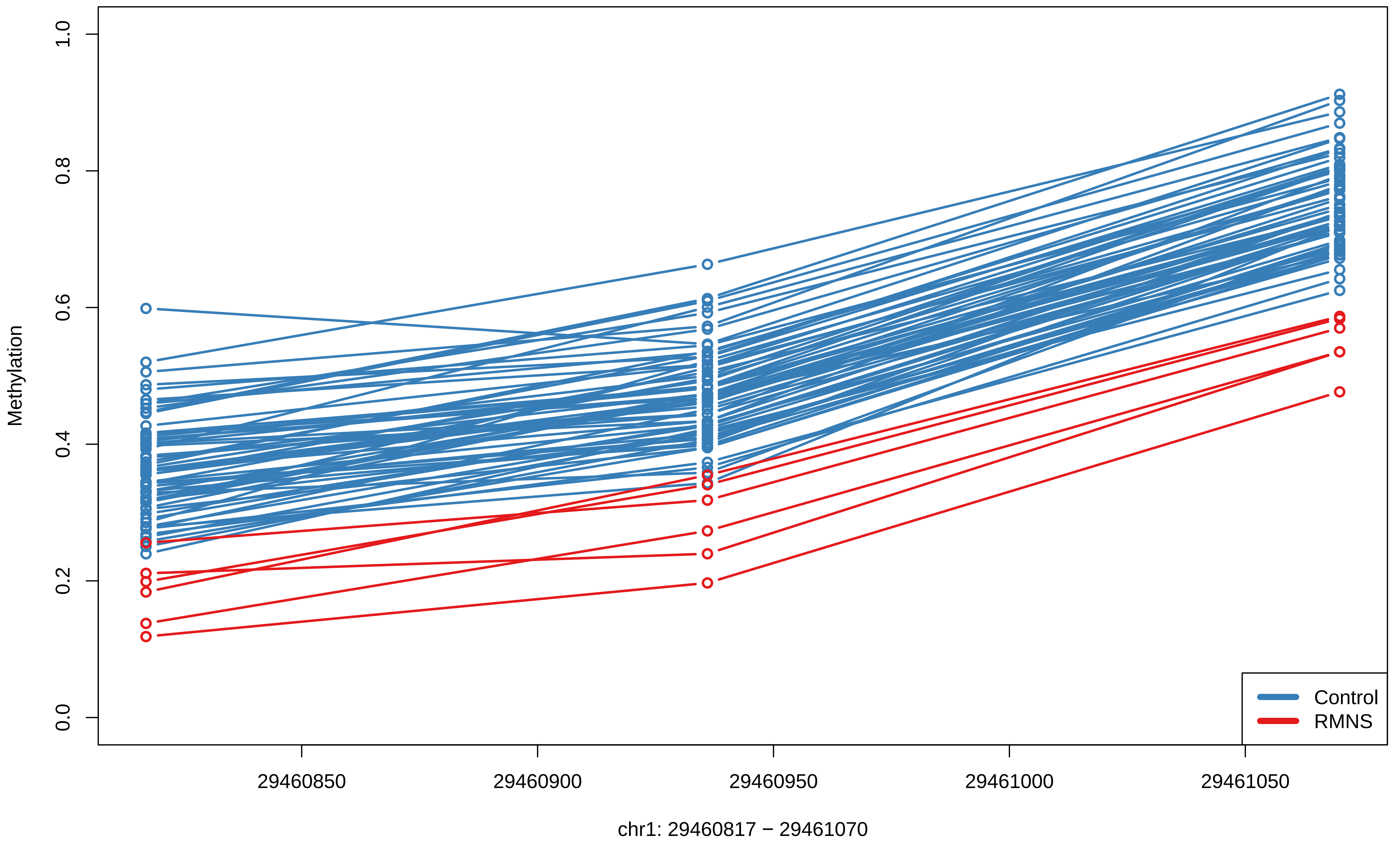
Methylation
0.0
0.2
0.4
0.6
0.8
1.0
29460850
29460900
29460950
29461000
29461050
chr1: 29460817 − 29461070
Control
RMNS

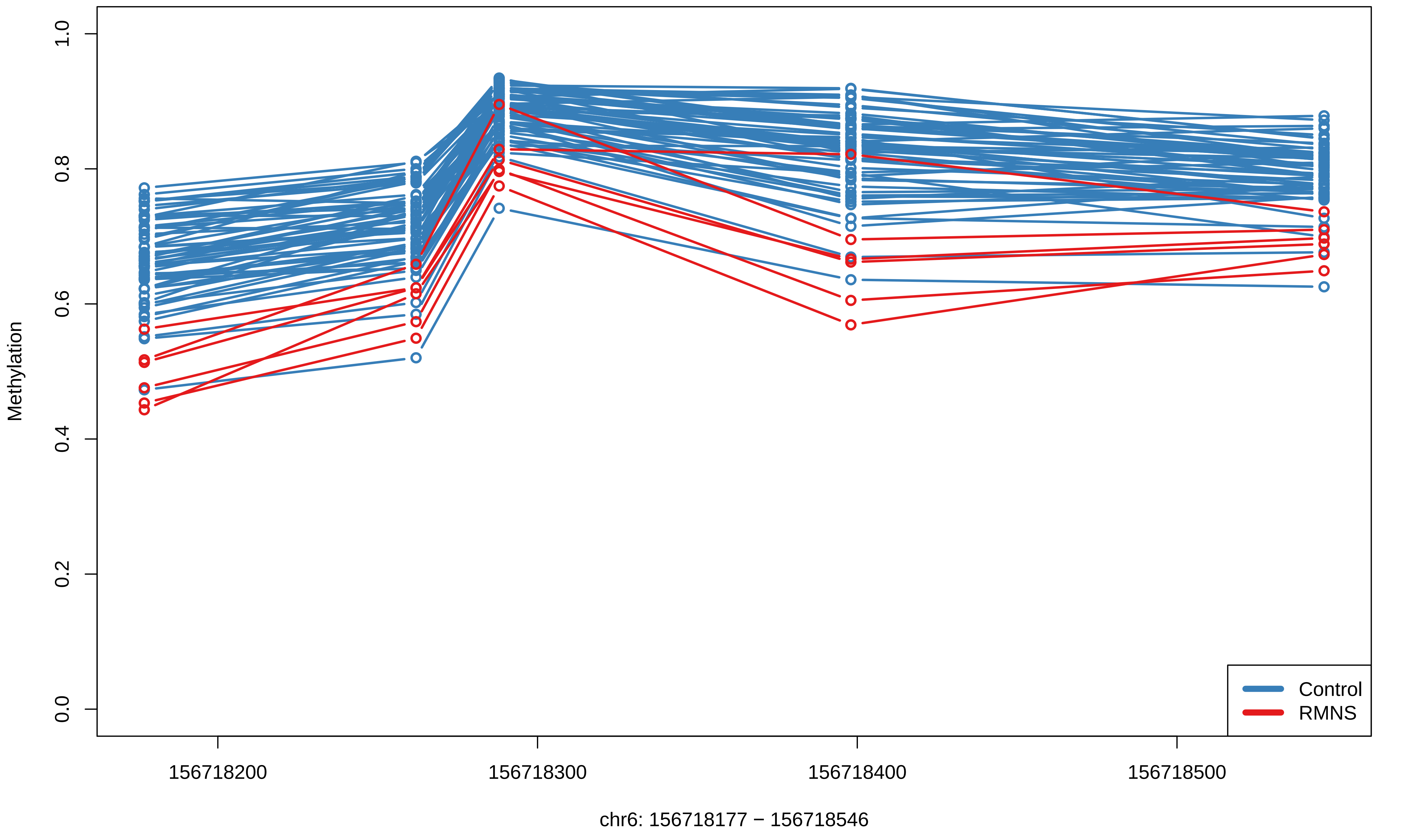

Methylation
0.0
0.2
0.4
0.6
0.8
1.0
156718200
156718300
156718400
156718500
chr6: 156718177 − 156718546
Control
RMNS

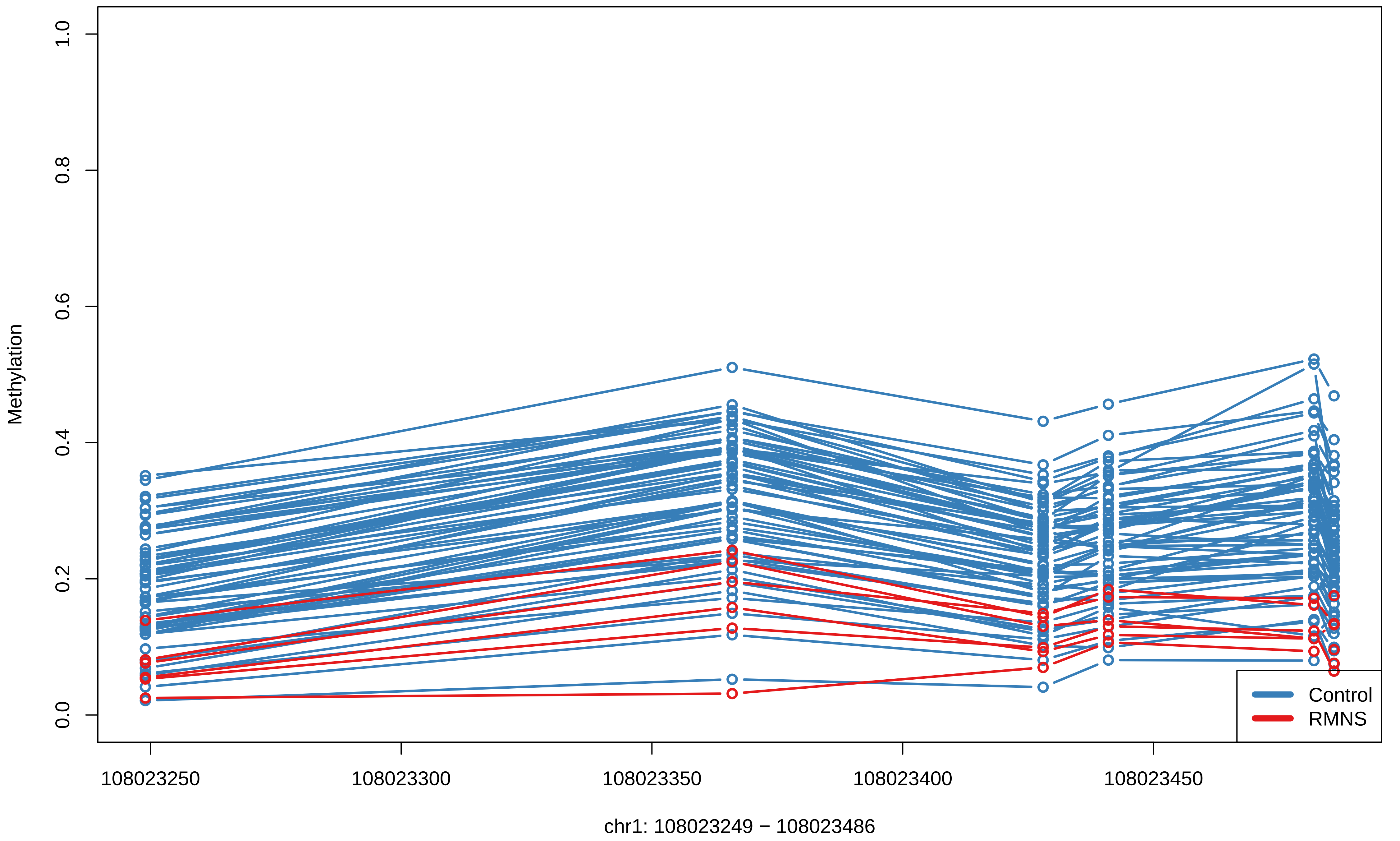

Methylation
1.0
0.8
0.6
0.4
0.2
0.0
108023250
108023300
108023350
108023400
108023450
chr1: 108023249 − 108023486
Control
RMNS

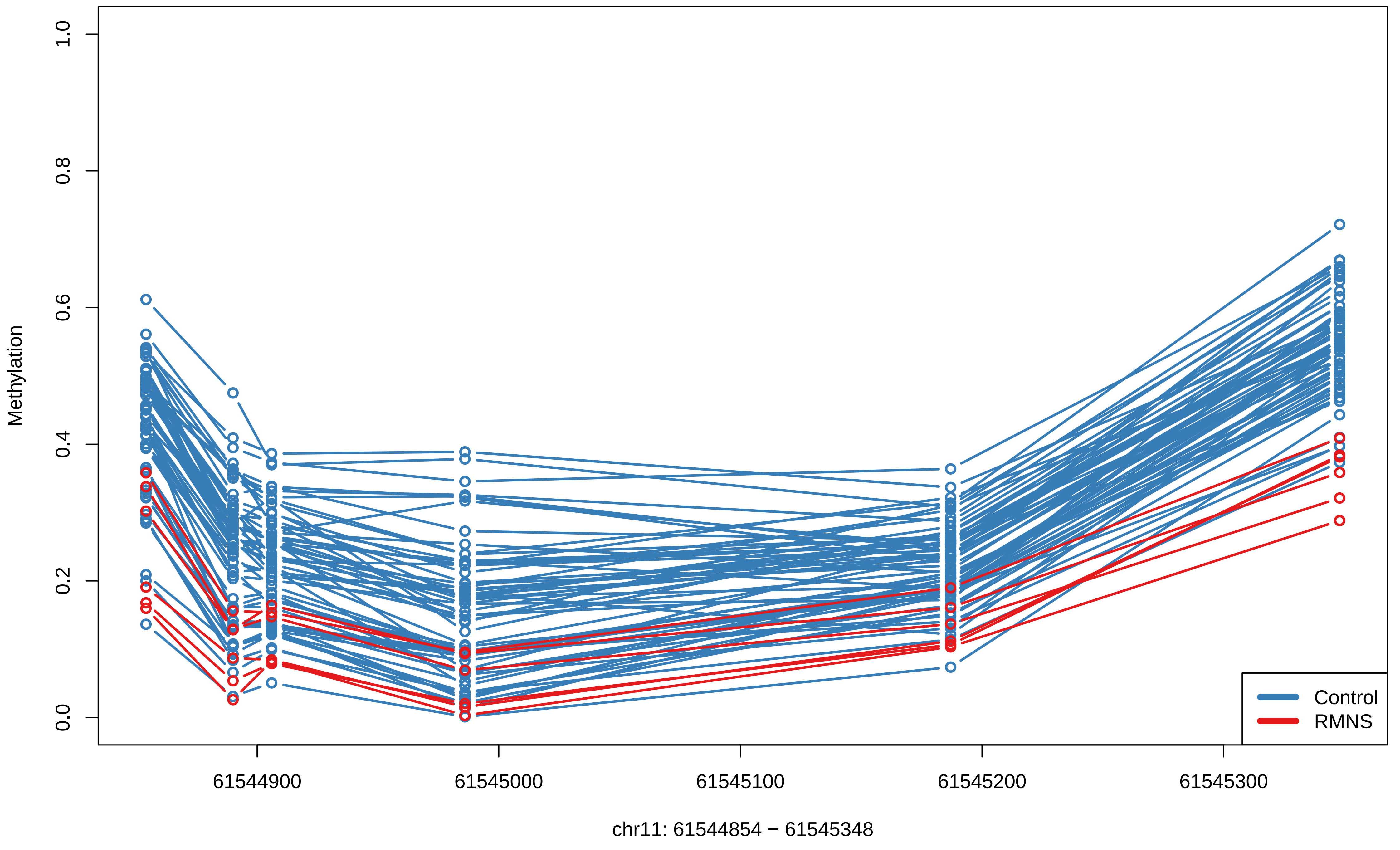

Methylation
0.0
0.2
0.4
0.6
0.8
1.0
61544900
61545000
61545100
61545200
61545300
chr11: 61544854 − 61545348
Control
RMNS

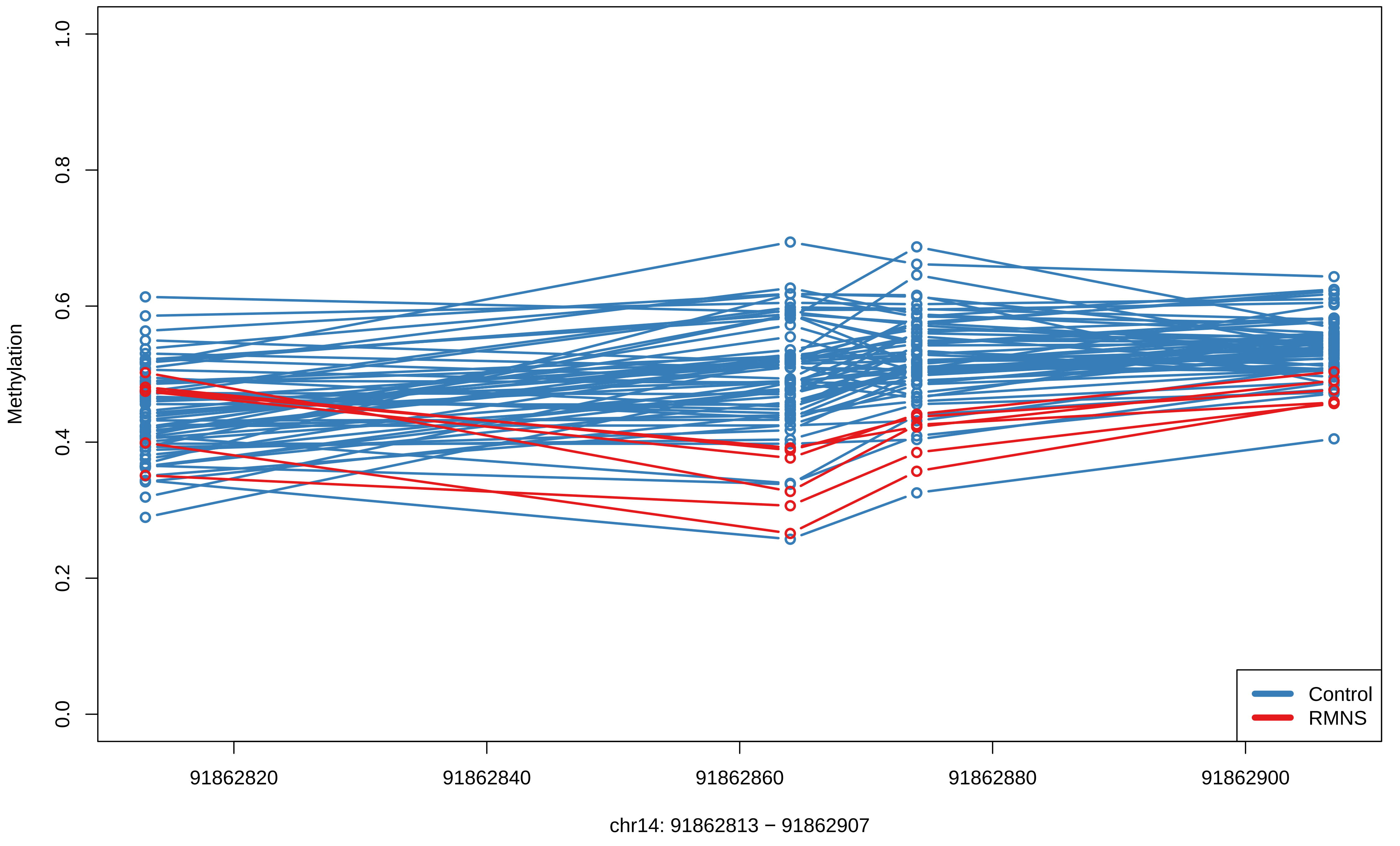
Methylation
0.0
0.2
0.4
0.6
0.8
1.0
91862820
91862840
91862860
91862880
91862900
chr14: 91862813 − 91862907
Control
RMNS

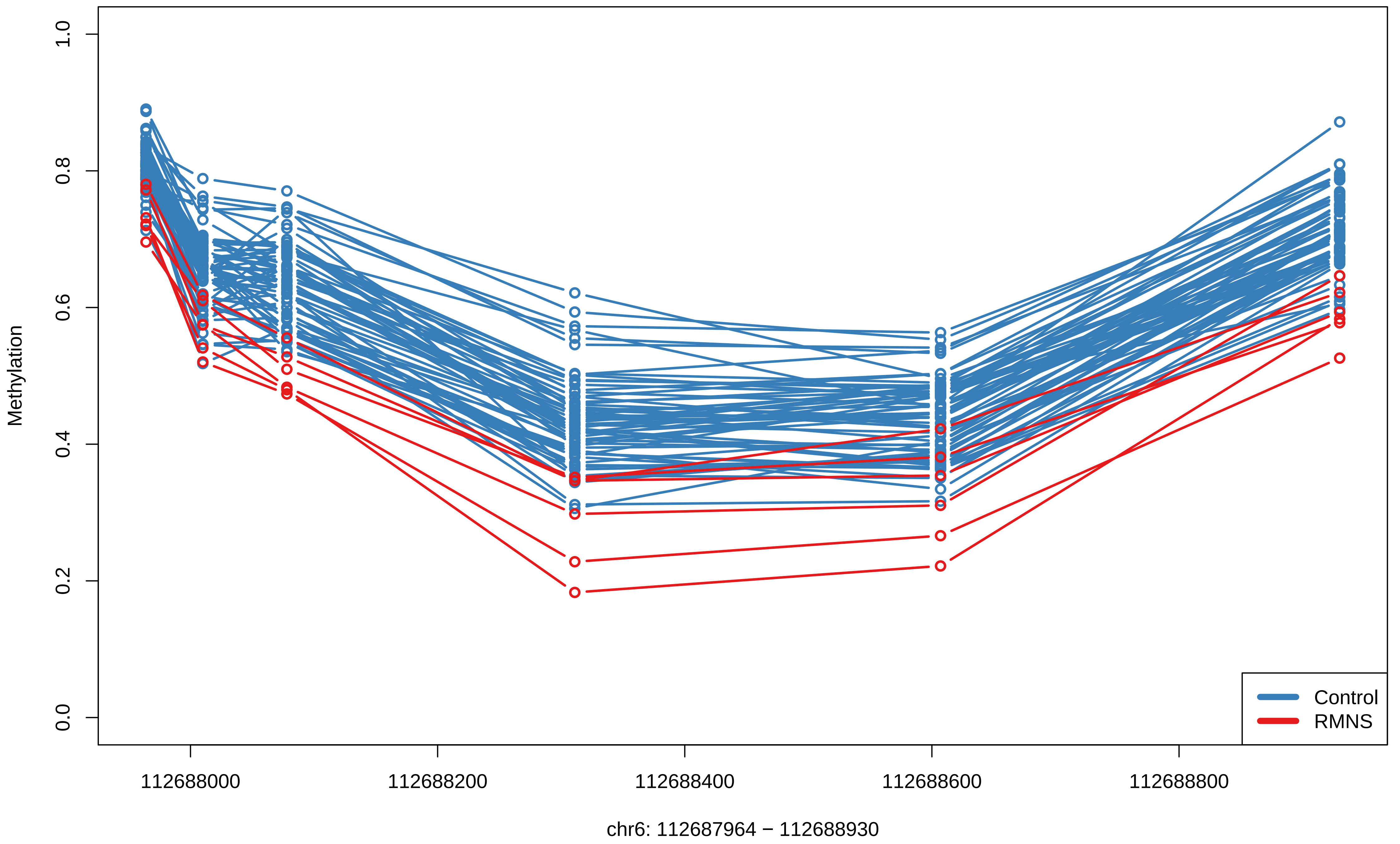

Methylation
0.0
0.2
0.4
0.6
0.8
1.0
112688000
112688200
112688400
112688600
112688800
chr6: 112687964 − 112688930
Control
RMNS

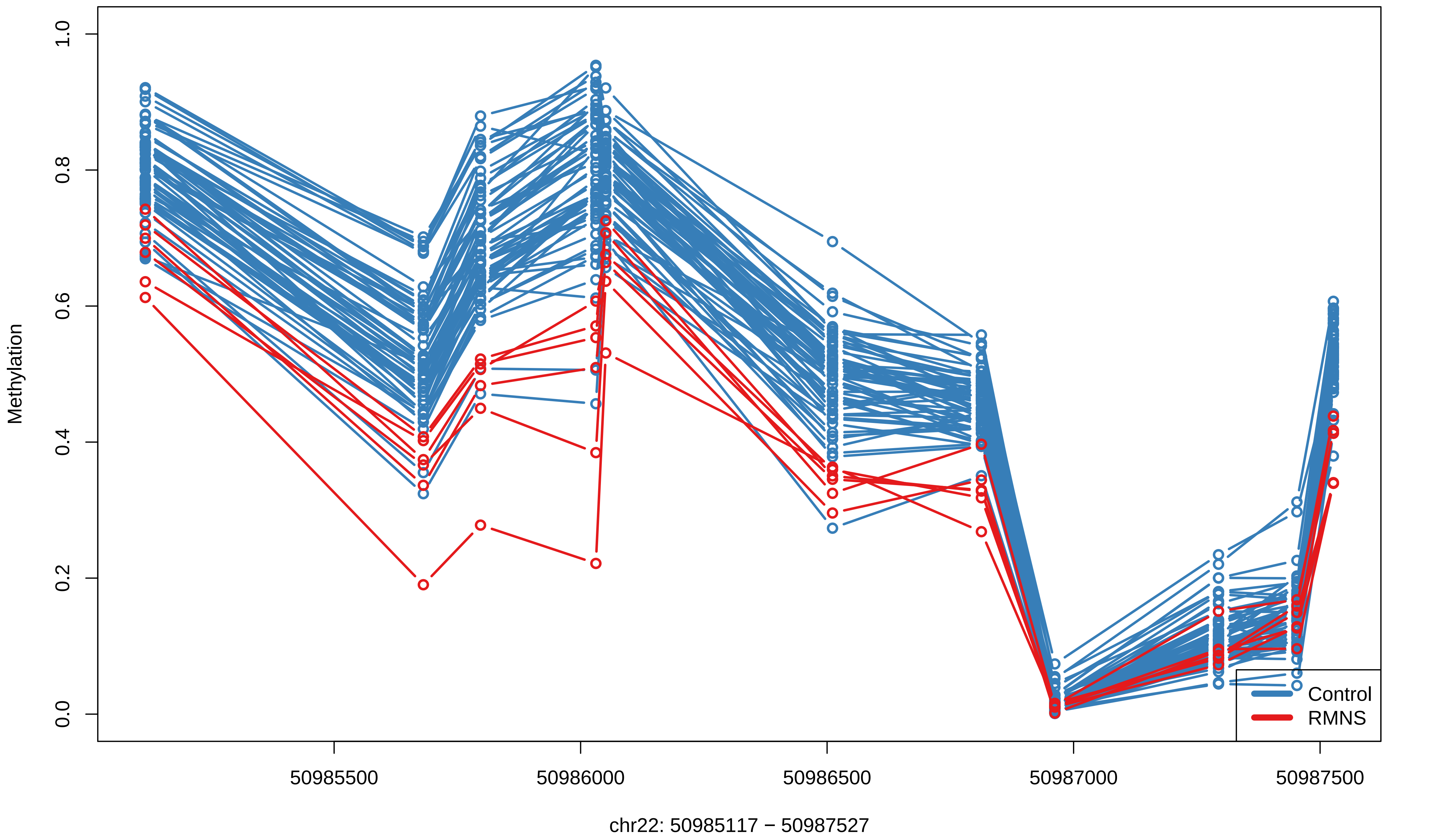

Methylation
0.0
0.2
0.4
0.6
0.8
1.0
50985500
50986000
50986500
50987000
50987500
chr22: 50985117 − 50987527
Control
RMNS

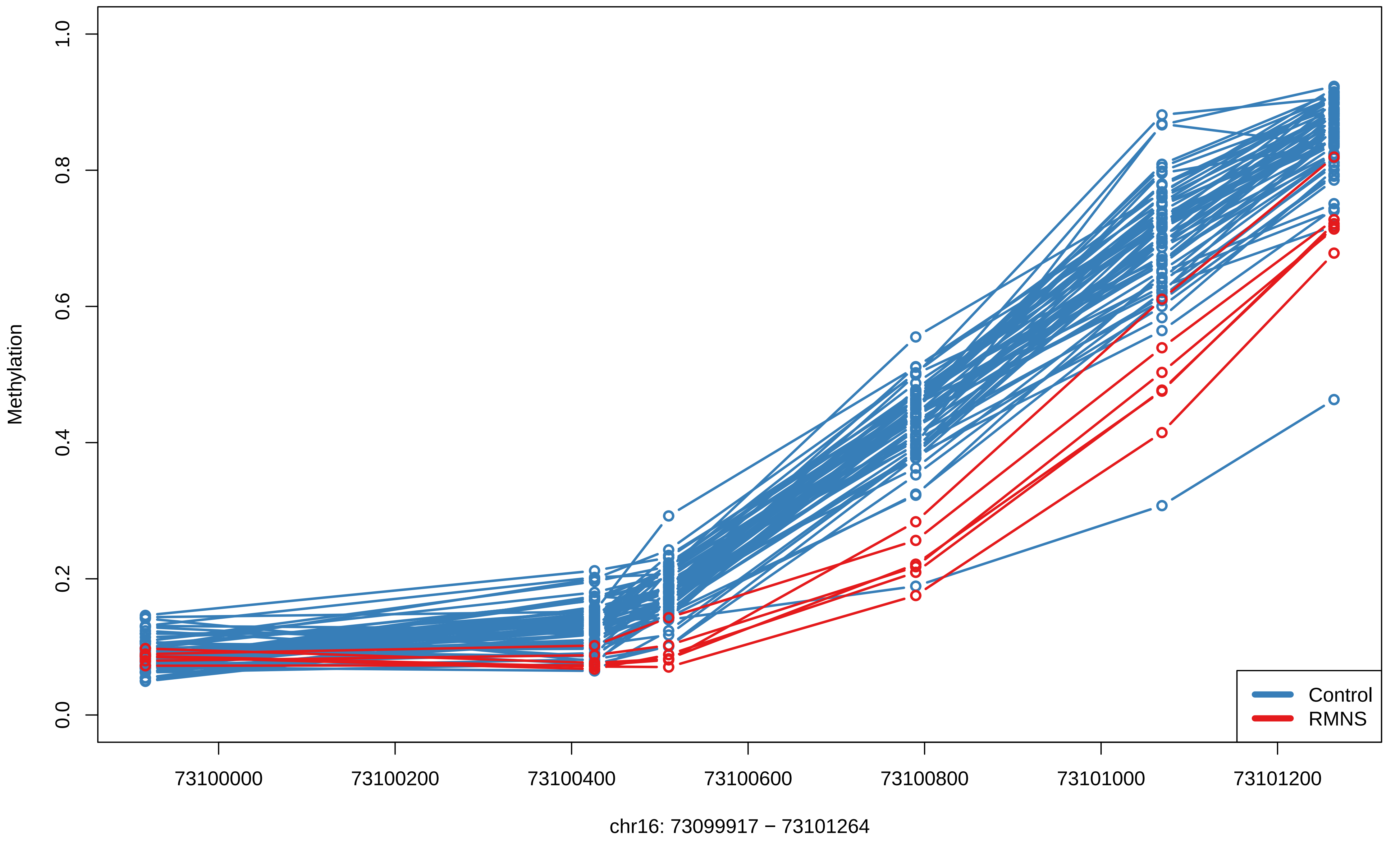

Methylation
0.0
0.2
0.4
0.6
0.8
1.0
73100000
73100200
73100400
73100600
73100800
73101000
73101200
chr16: 73099917 − 73101264
Control
RMNS

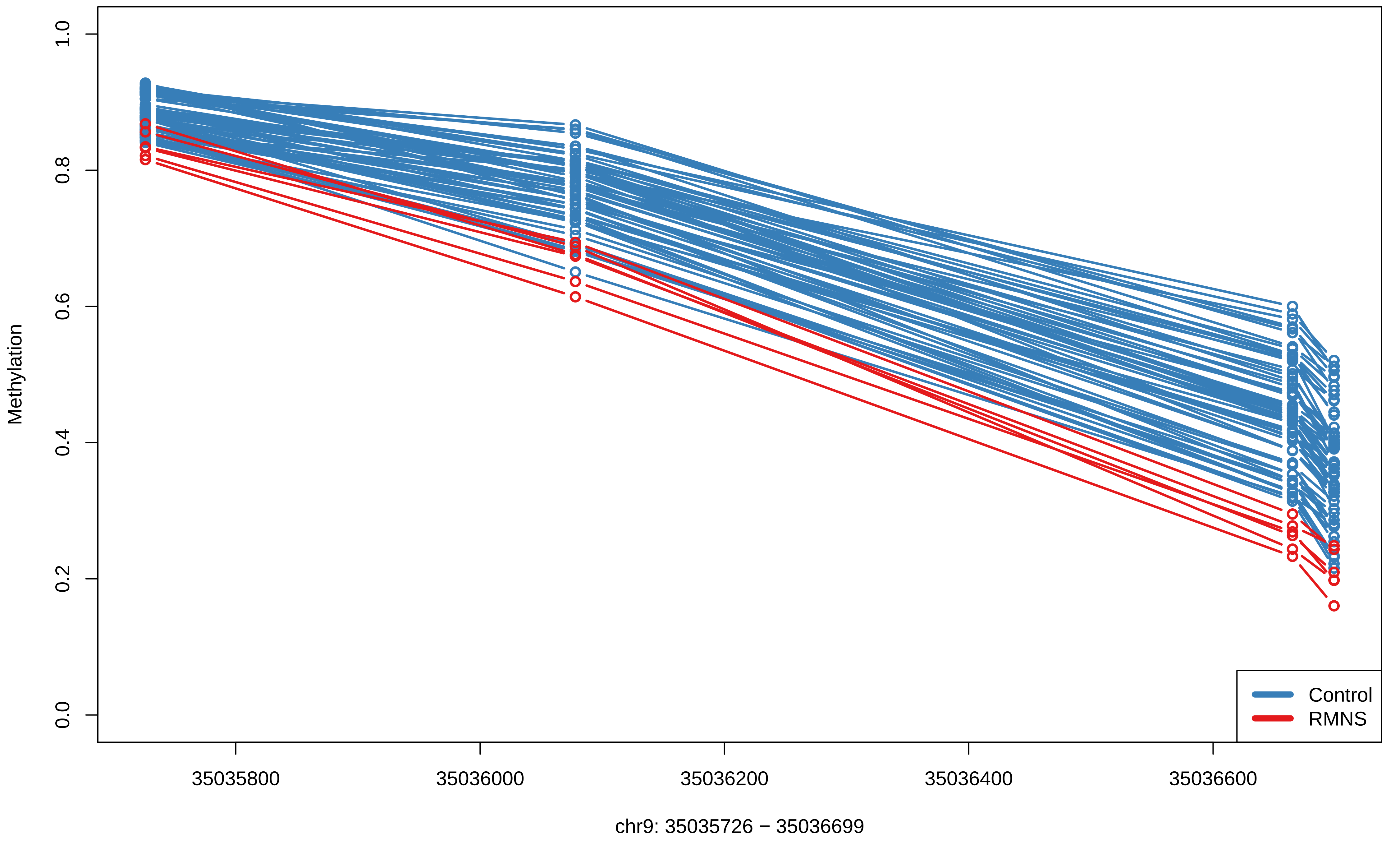

Methylation
0.0
0.2
0.4
0.6
0.8
1.0
35035800
35036000
35036200
35036400
35036600
chr9: 35035726 − 35036699
Control
RMNS

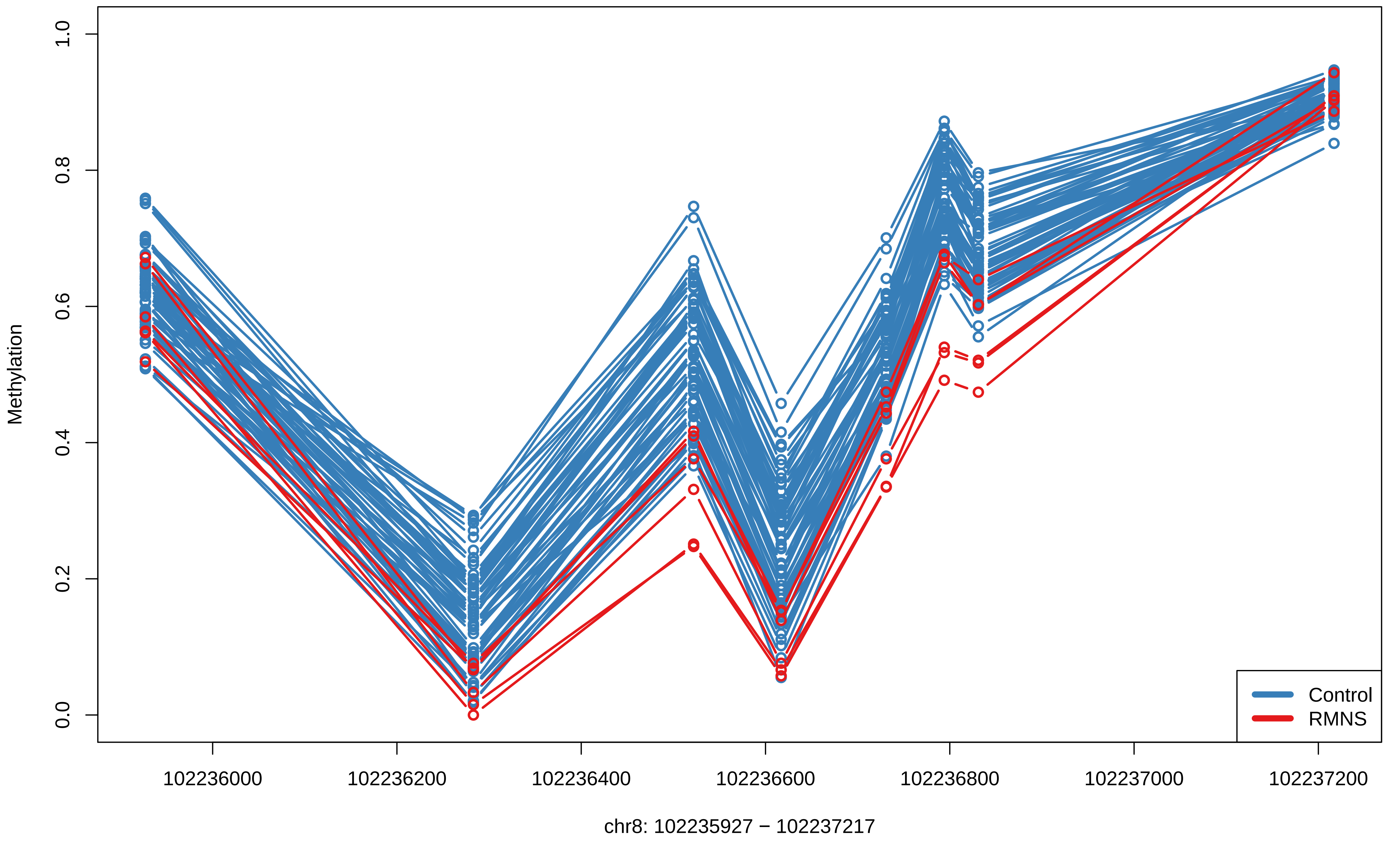

Methylation
0.0
0.2
0.4
0.6
0.8
1.0
102236000
102236200
102236400
102236600
102236800
102237000
102237200
chr8: 102235927 − 102237217
Control
RMNS

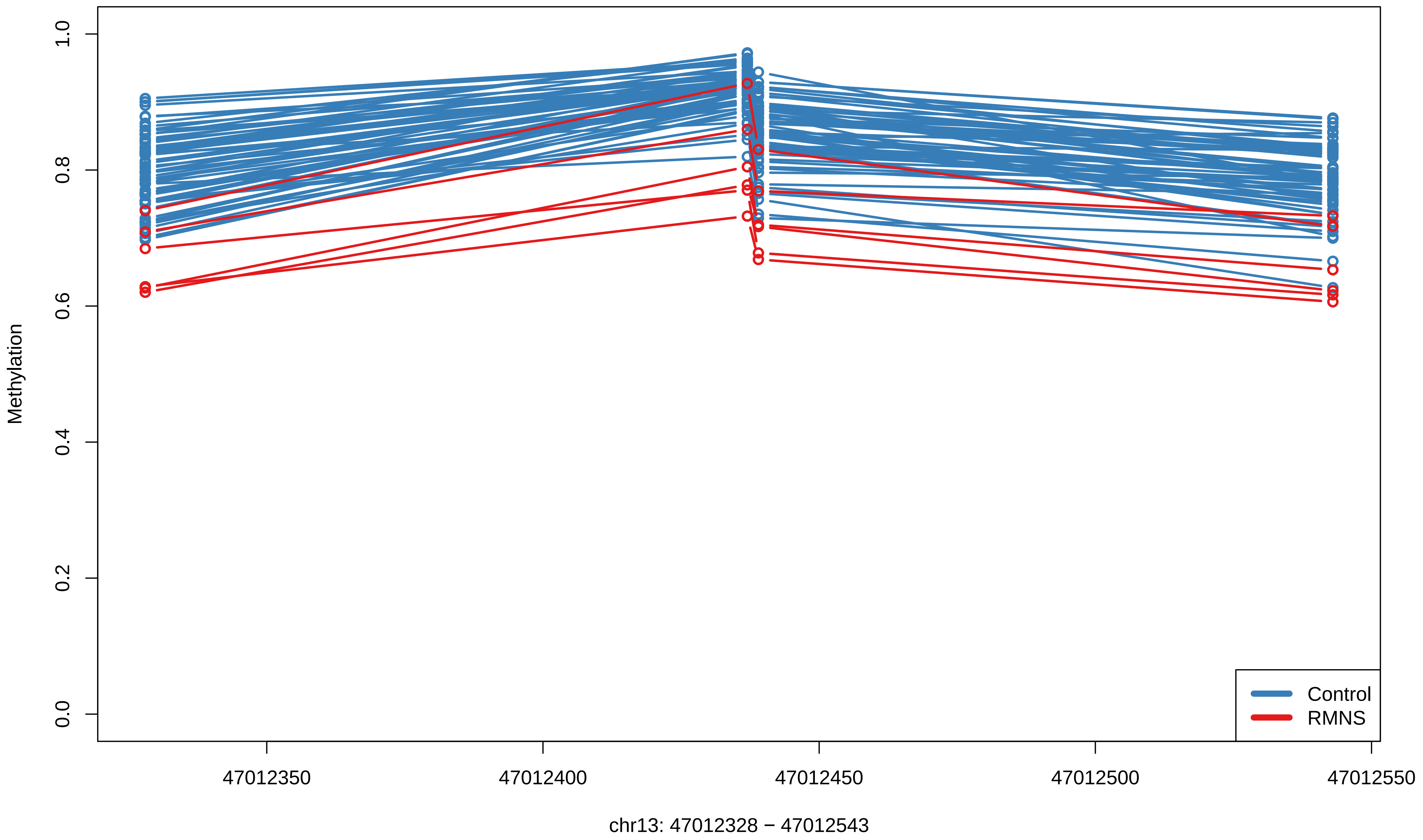

Methylation
1.0
0.8
0.6
0.4
0.2
0.0
47012350
47012400
47012450
47012500
47012550
chr13: 47012328 − 47012543
Control
RMNS

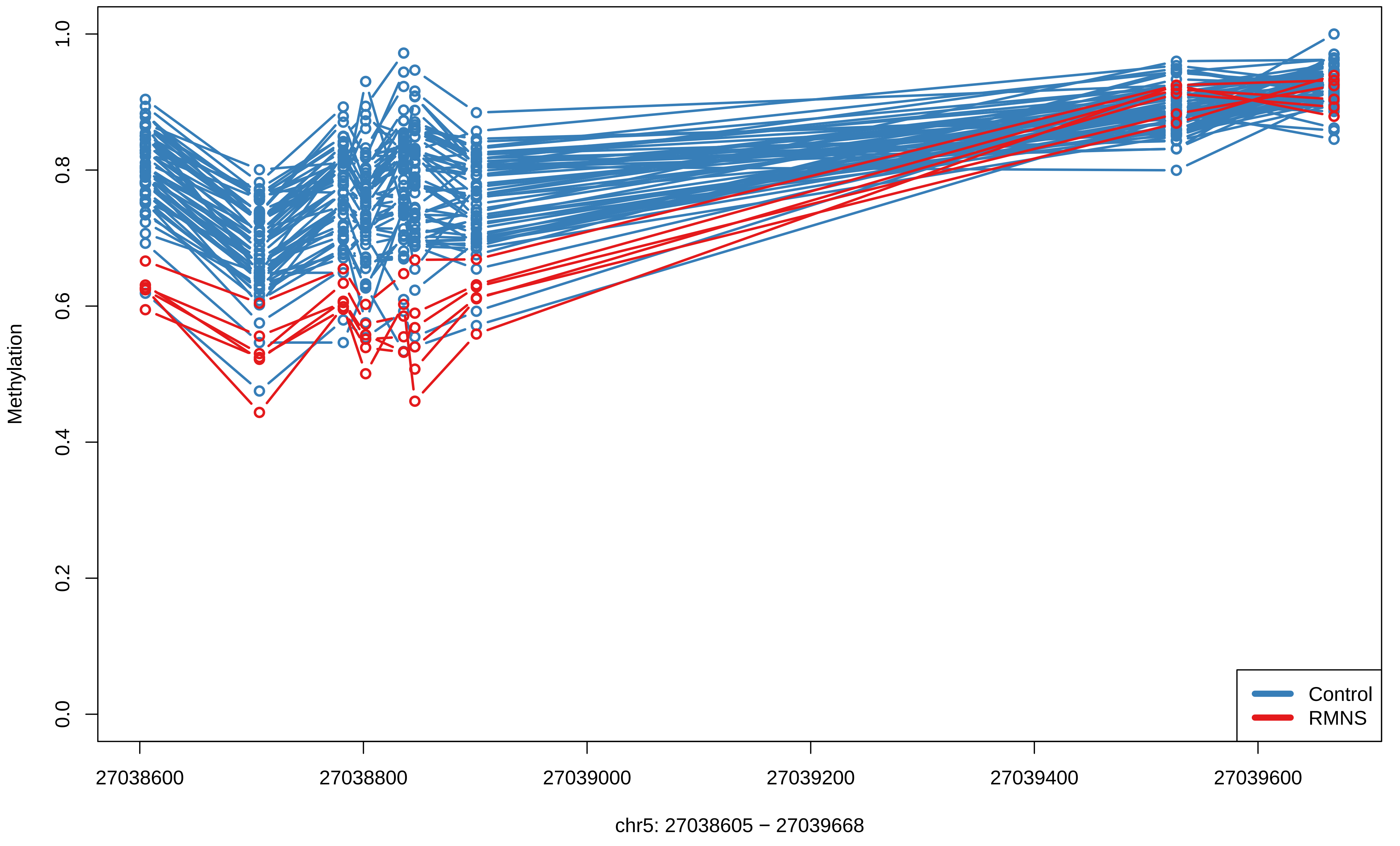

Methylation
0.0
0.2
0.4
0.6
0.8
1.0
27038600
27038800
27039000
27039200
27039400
27039600
chr5: 27038605 − 27039668
Control
RMNS

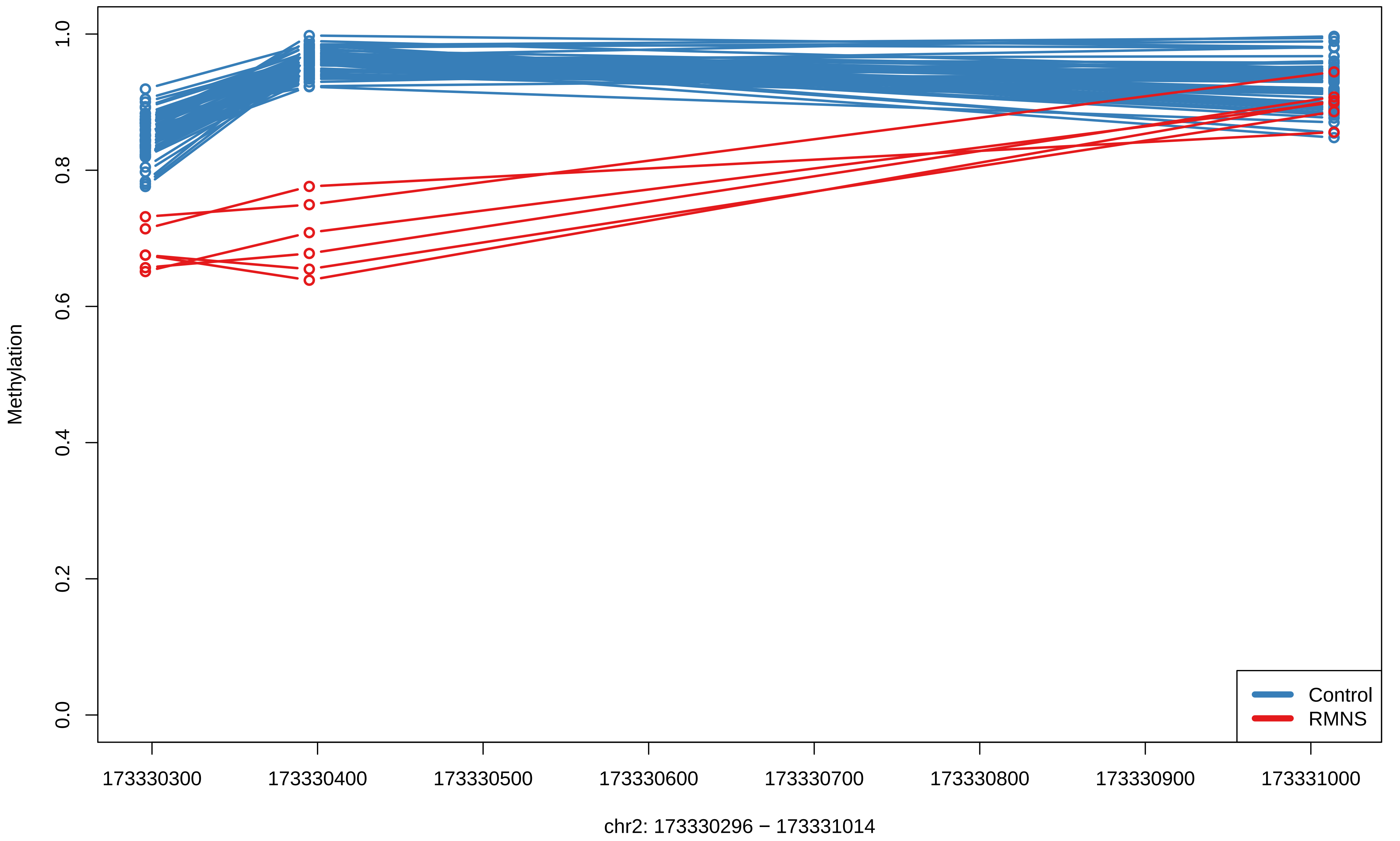

Methylation
1.0
0.8
0.6
0.4
0.2
0.0
Control
RMNS
173330300
173330400
173330500
173330600
173330700
173330800
173330900
173331000
chr2: 173330296 − 173331014

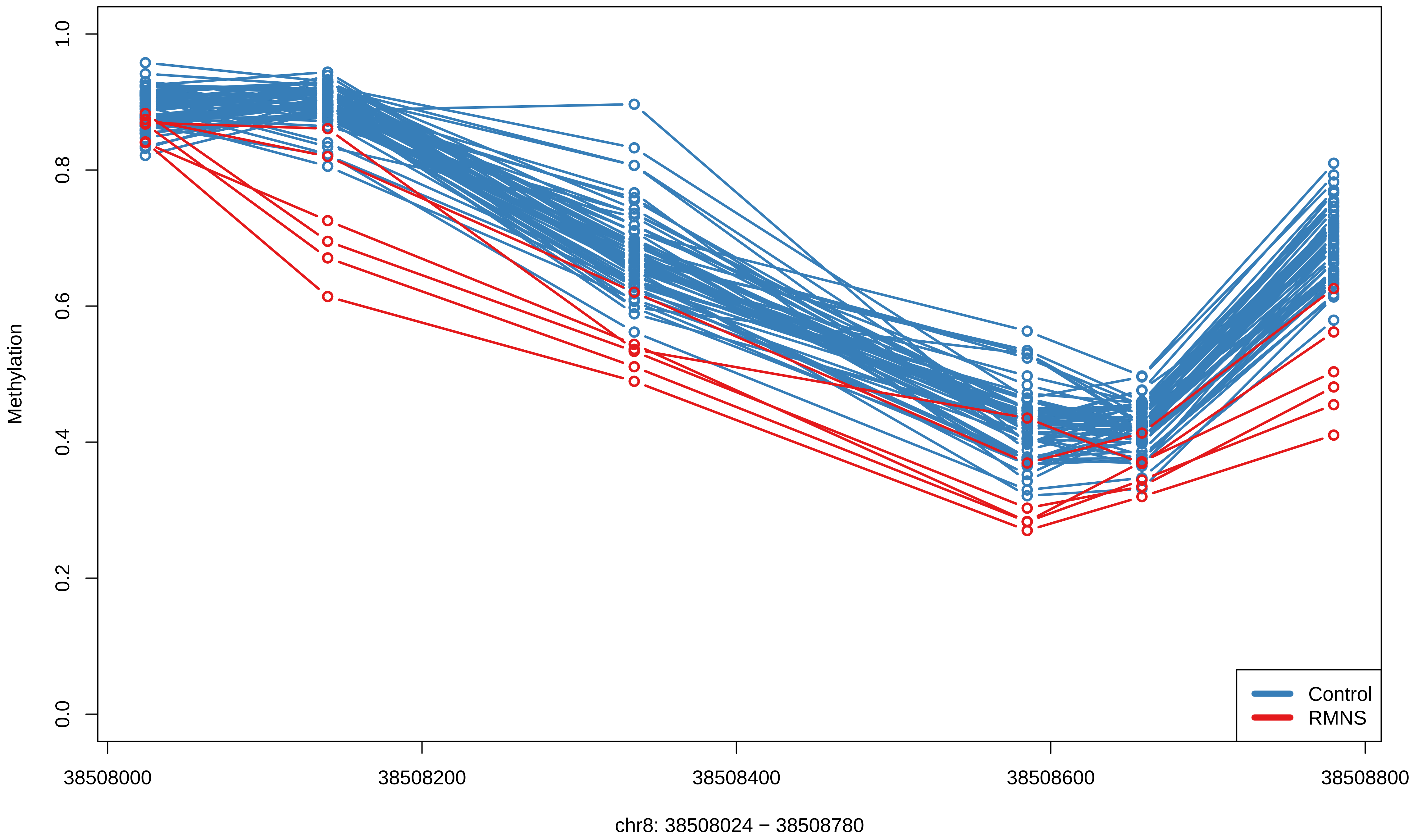

Methylation
1.0
0.8
0.6
0.4
0.2
0.0
38508000
38508200
38508400
38508600
38508800
chr8: 38508024 − 38508780
Control
RMNS

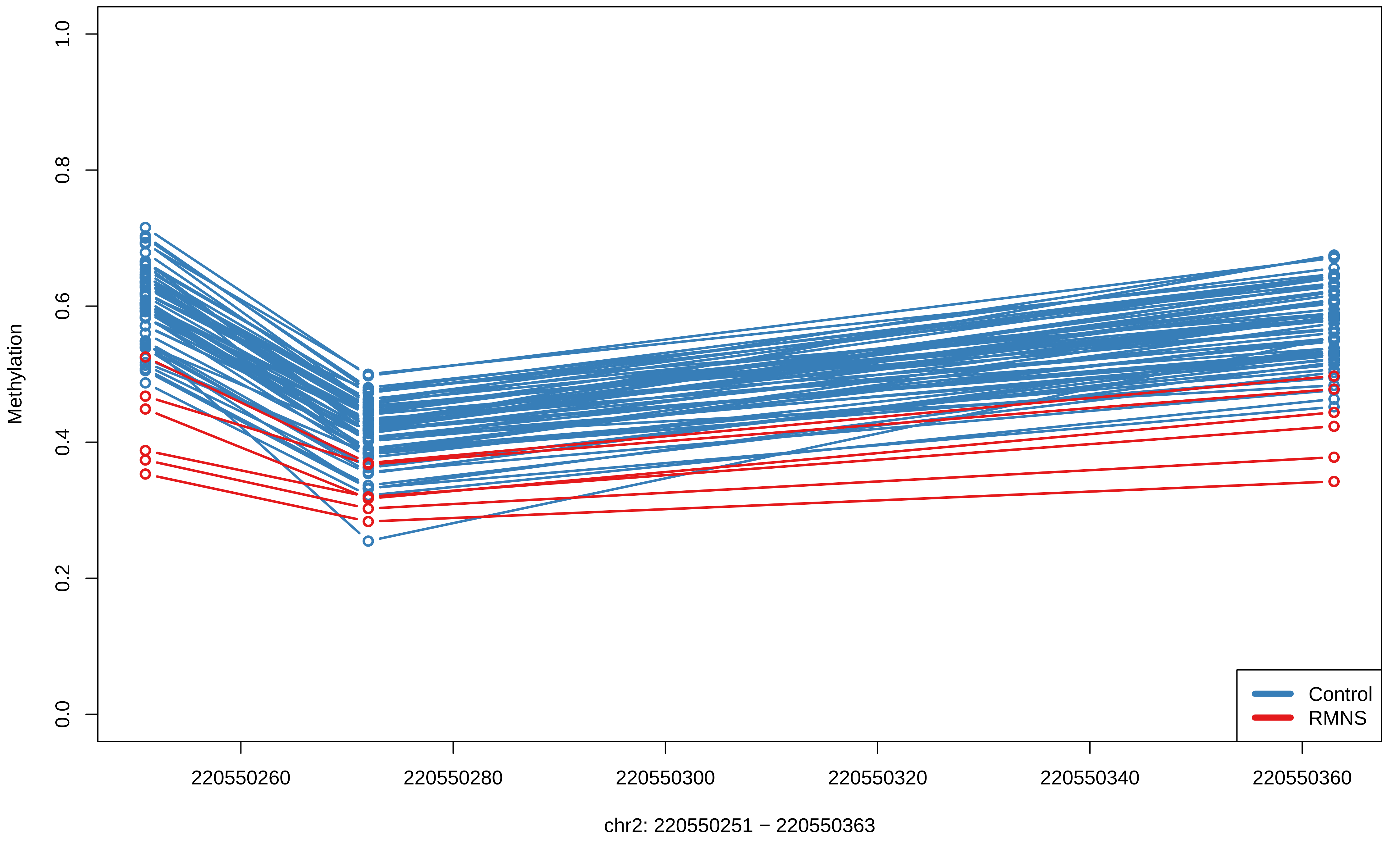

Methylation
0.0
0.2
0.4
0.6
0.8
1.0
220550260
220550280
220550300
220550320
220550340
220550360
chr2: 220550251 − 220550363
Control
RMNS

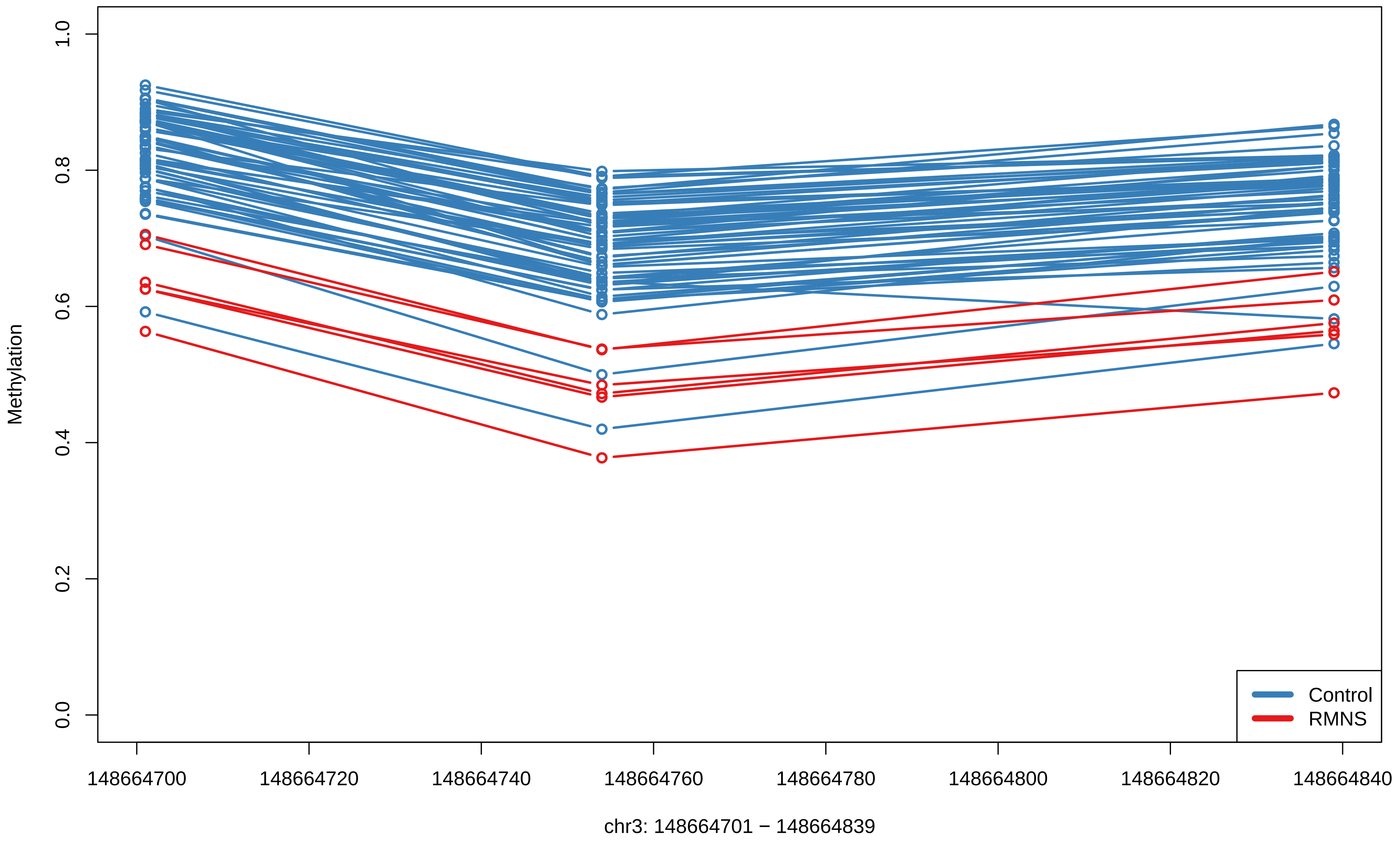

Methylation
1.0
0.8
0.6
0.4
0.2
0.0
148664700
148664720
148664740
148664760
148664780
148664800
148664820
148664840
chr3: 148664701 − 148664839
Control
RMNS

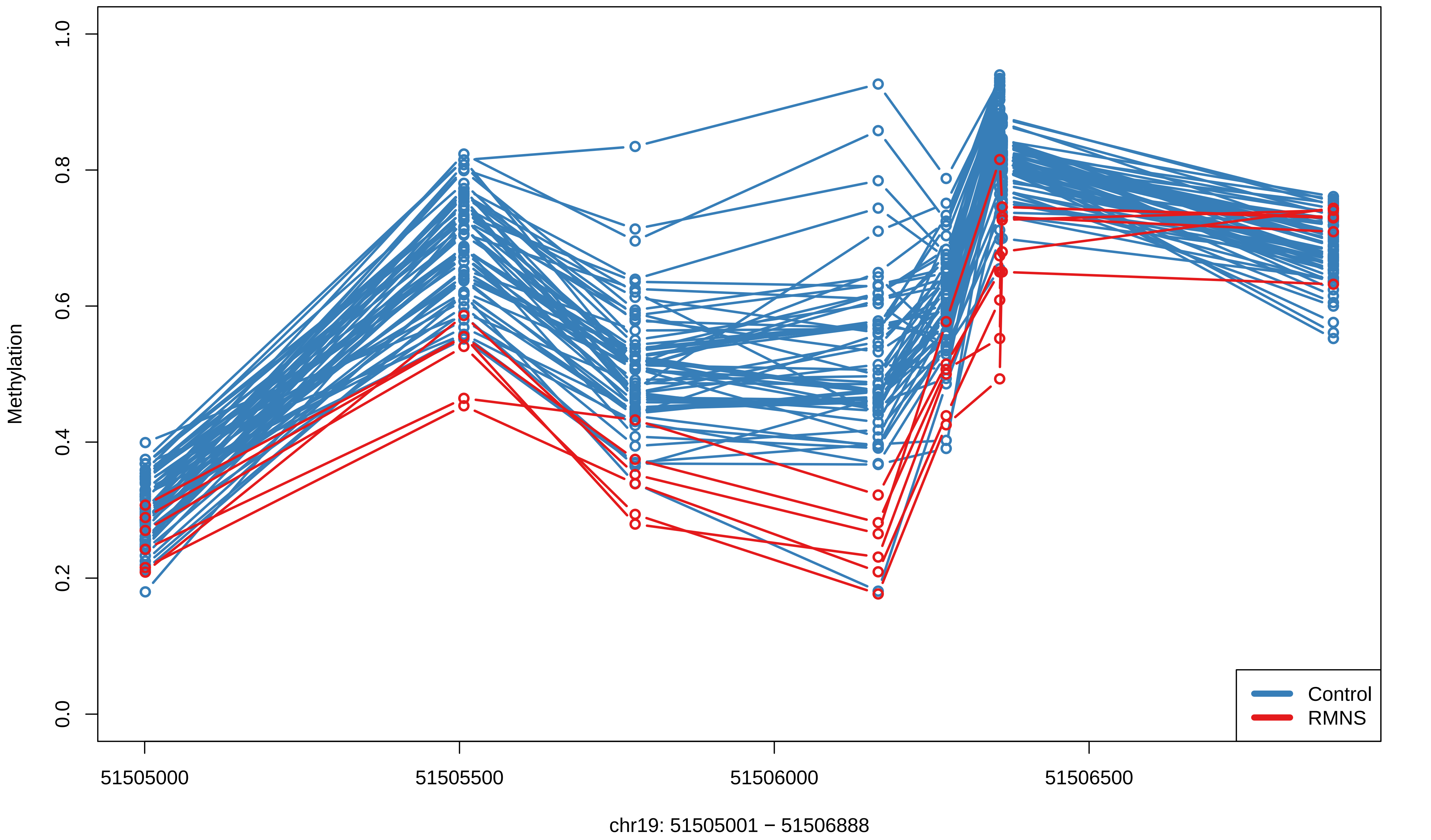

Methylation
1.0
0.8
0.6
0.4
0.2
0.0
51505000
51505500
51506000
51506500
chr19: 51505001 − 51506888
Control
RMNS

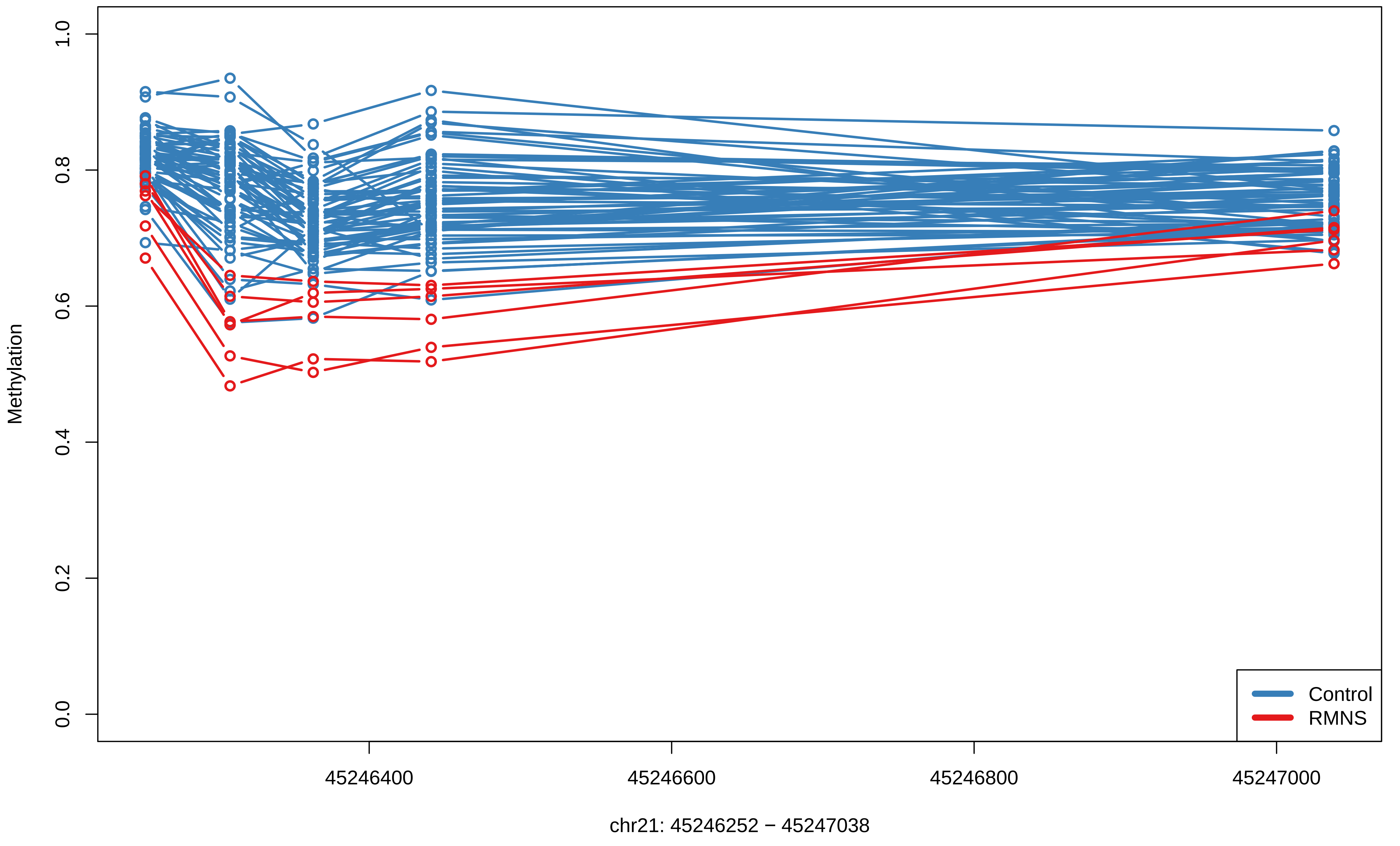

Methylation
1.0
0.8
0.6
0.4
0.2
0.0
45246400
45246600
45246800
45247000
chr21: 45246252 − 45247038
Control
RMNS

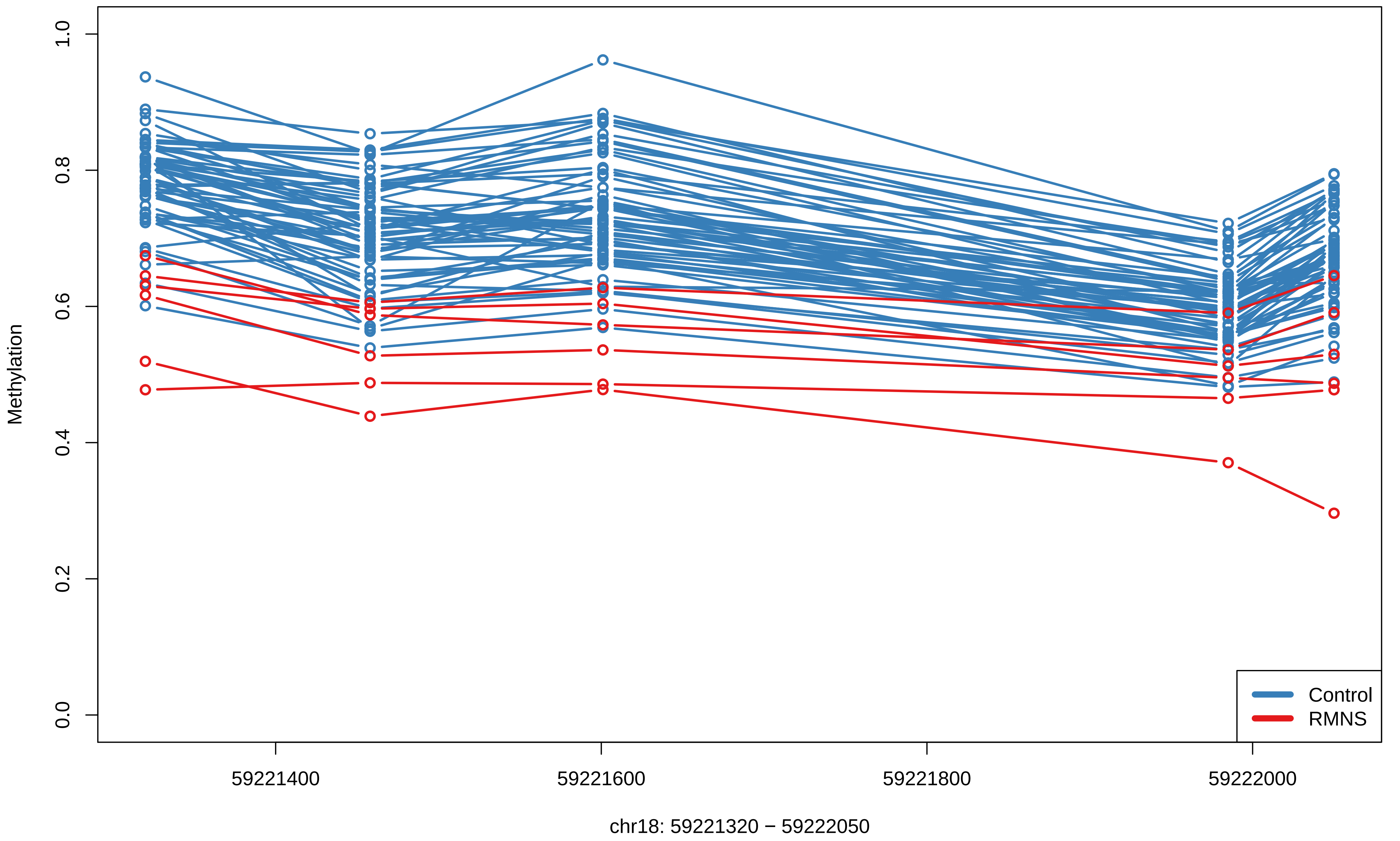

Methylation
0.0
0.2
0.4
0.6
0.8
1.0
59221400
59221600
59221800
59222000
chr18: 59221320 − 59222050
Control
RMNS

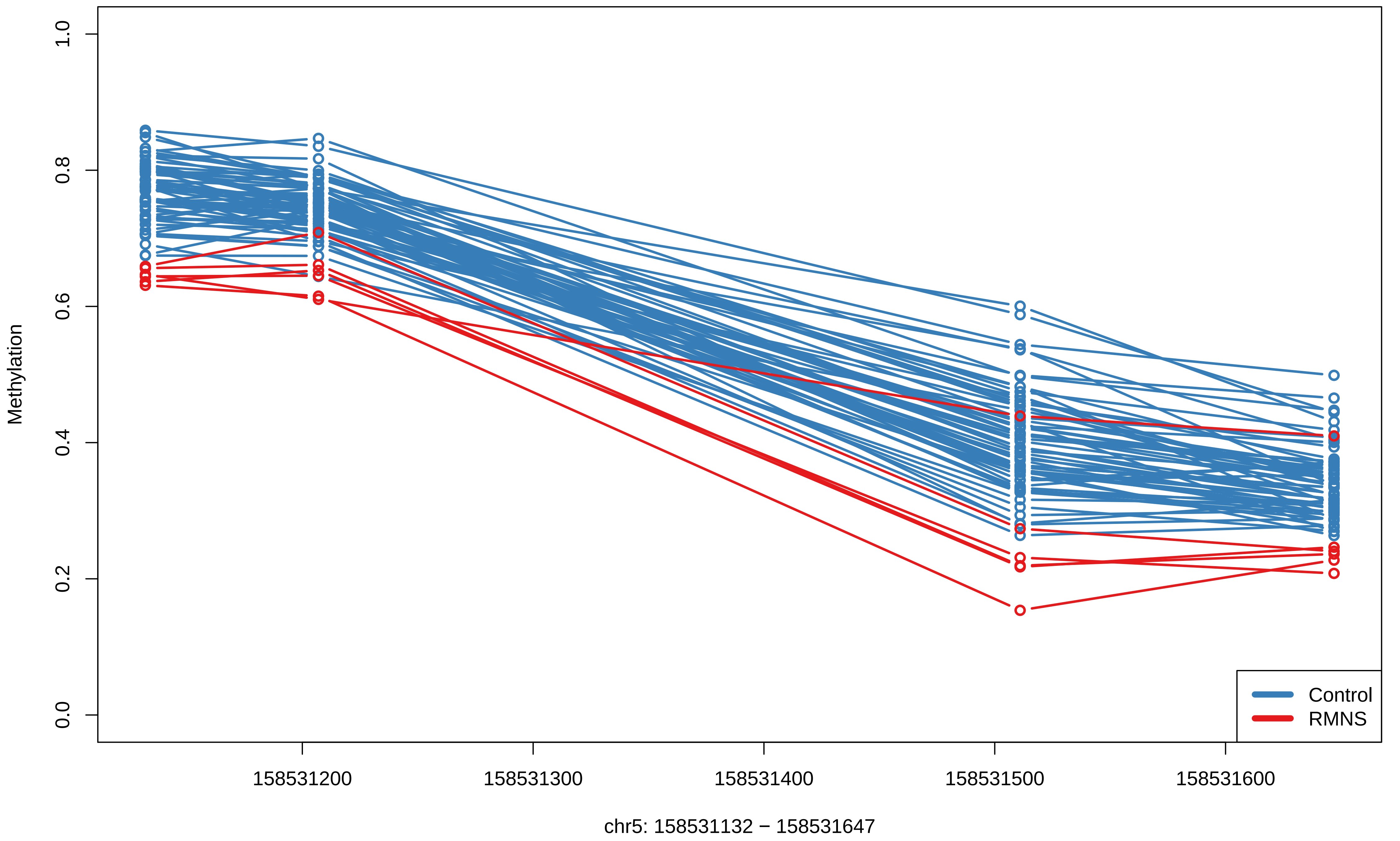

Methylation
1.0
0.8
0.6
0.4
0.2
0.0
158531200
158531300
158531400
158531500
158531600
chr5: 158531132 − 158531647
Control
RMNS

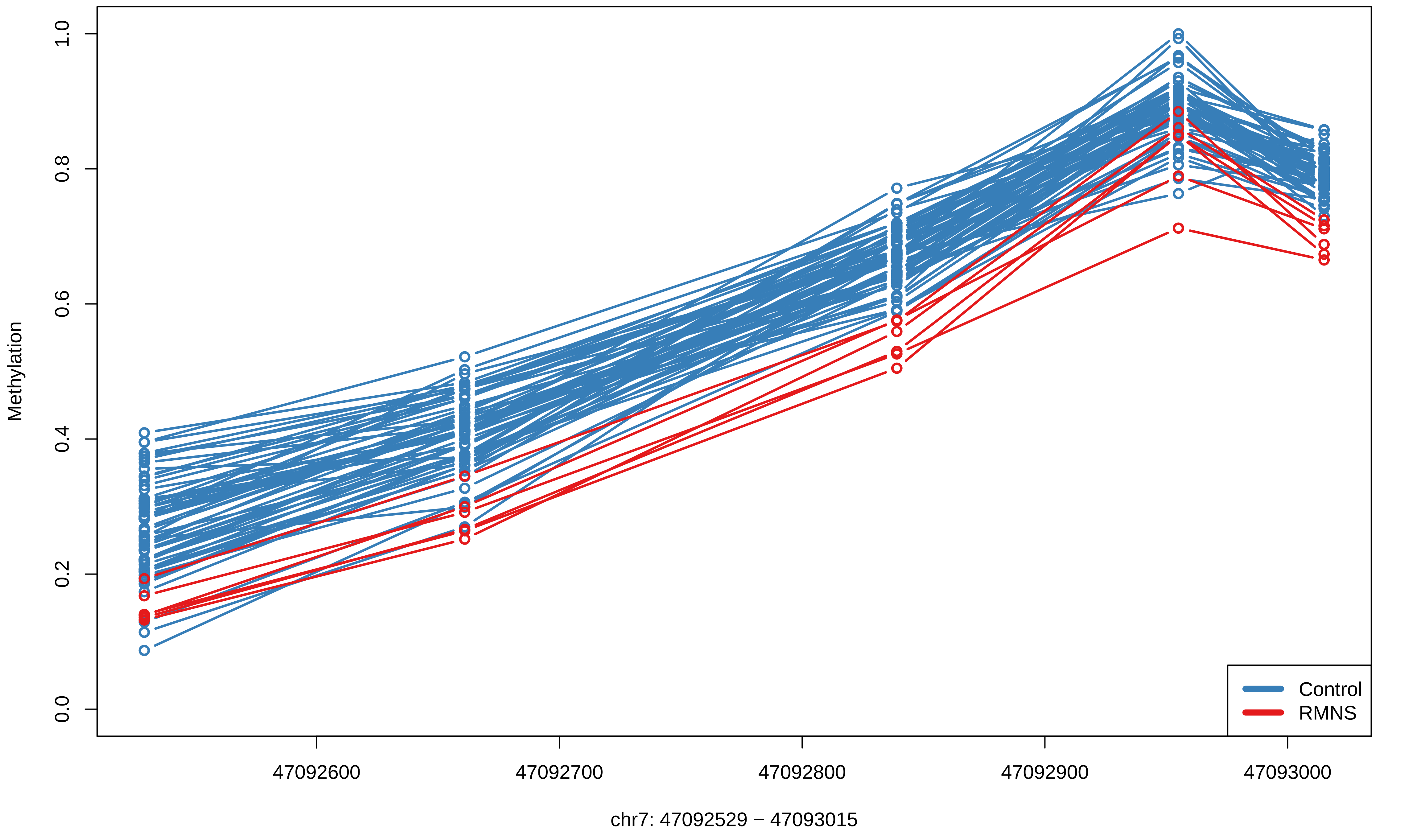

Methylation
0.0
0.2
0.4
0.6
0.8
1.0
47092600
47092700
47092800
47092900
47093000
chr7: 47092529 − 47093015
Control
RMNS

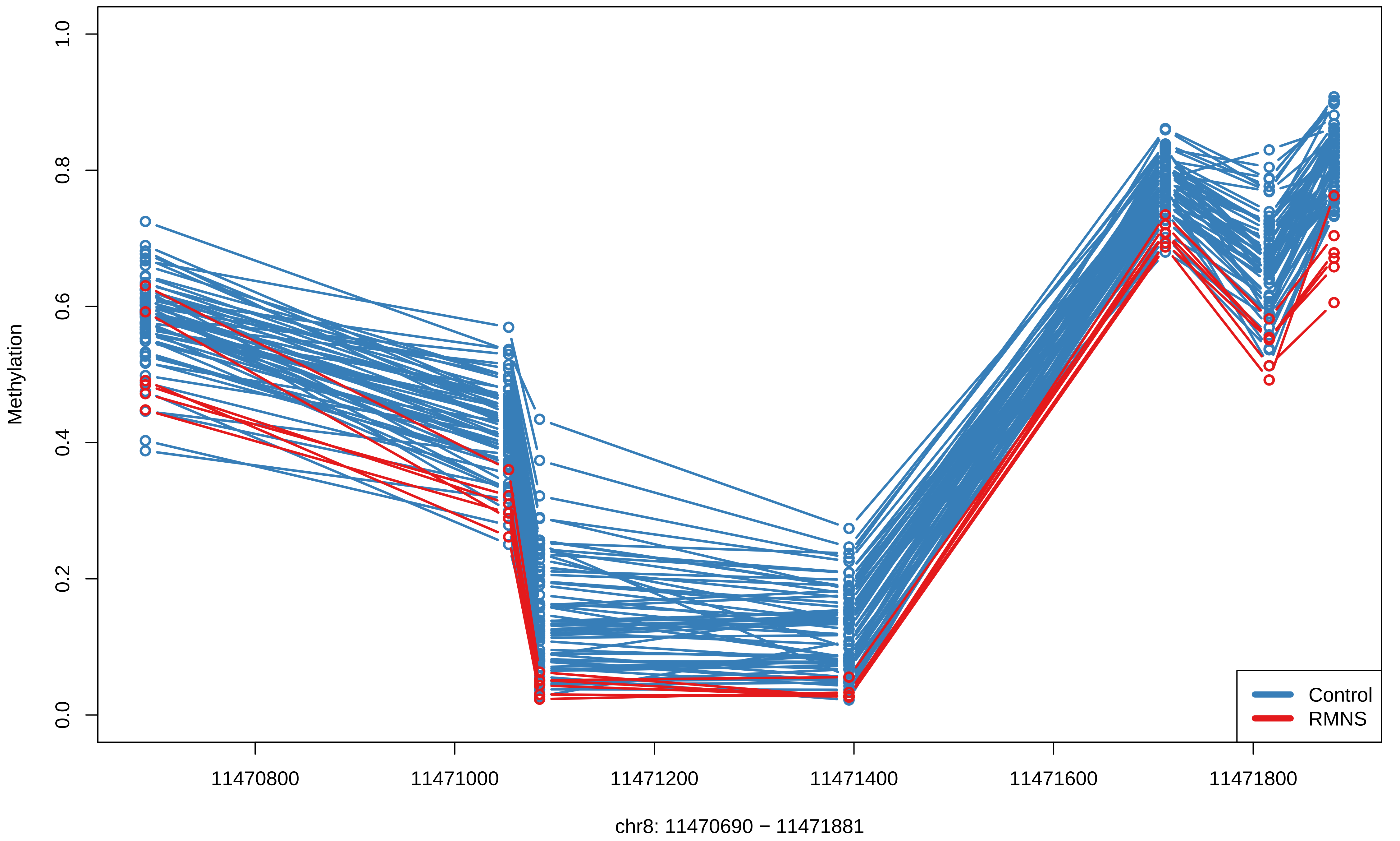

Methylation
0.0
0.2
0.4
0.6
0.8
1.0
11470800
11471000
11471200
11471400
11471600
11471800
chr8: 11470690 − 11471881
Control
RMNS

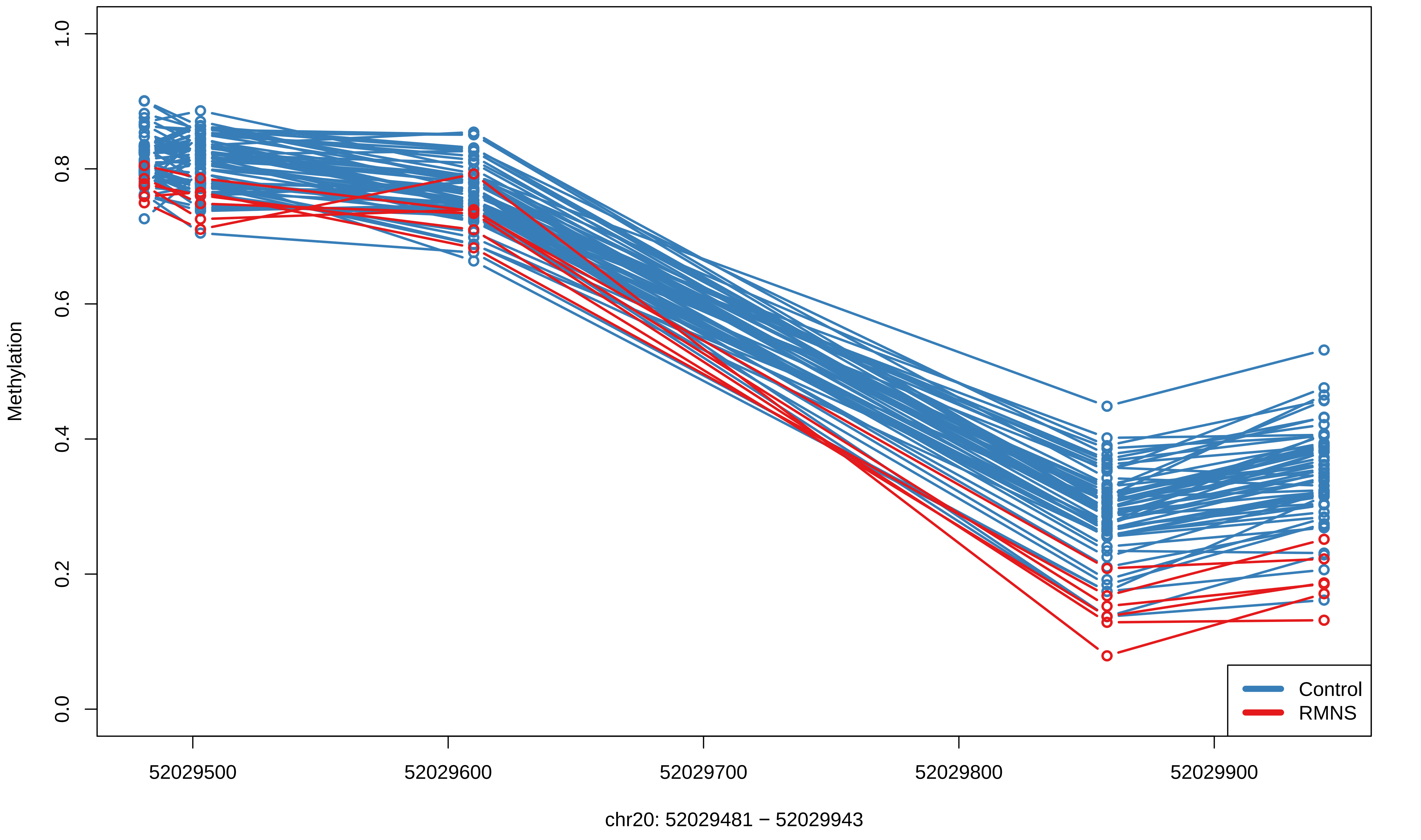

Methylation
1.0
0.8
0.6
0.4
0.2
0.0
52029500
52029600
52029700
52029800
52029900
chr20: 52029481 − 52029943
Control
RMNS

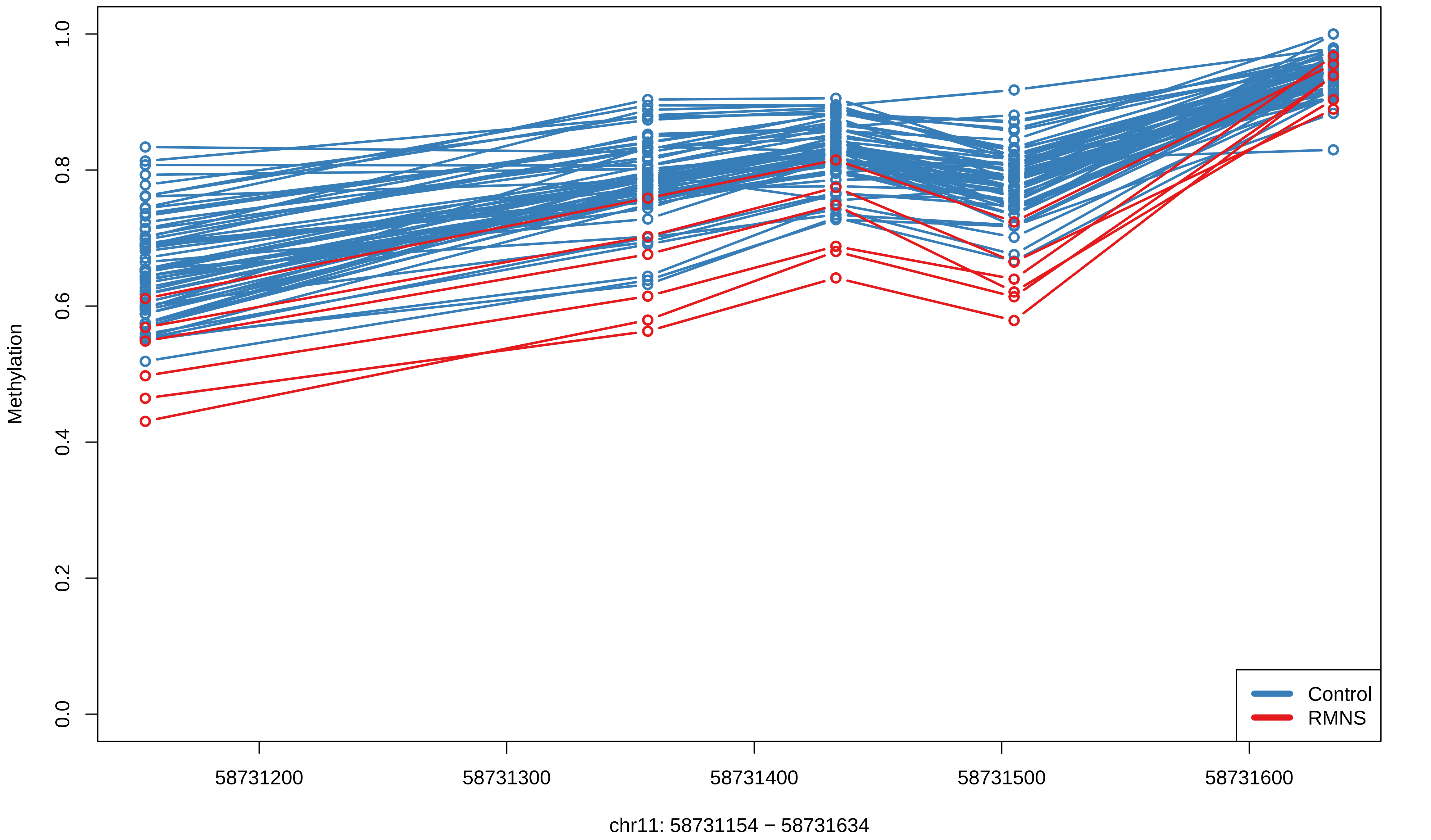

Methylation
1.0
0.8
0.6
0.4
0.2
0.0
58731200
58731300
58731400
58731500
58731600
chr11: 58731154 − 58731634
Control
RMNS

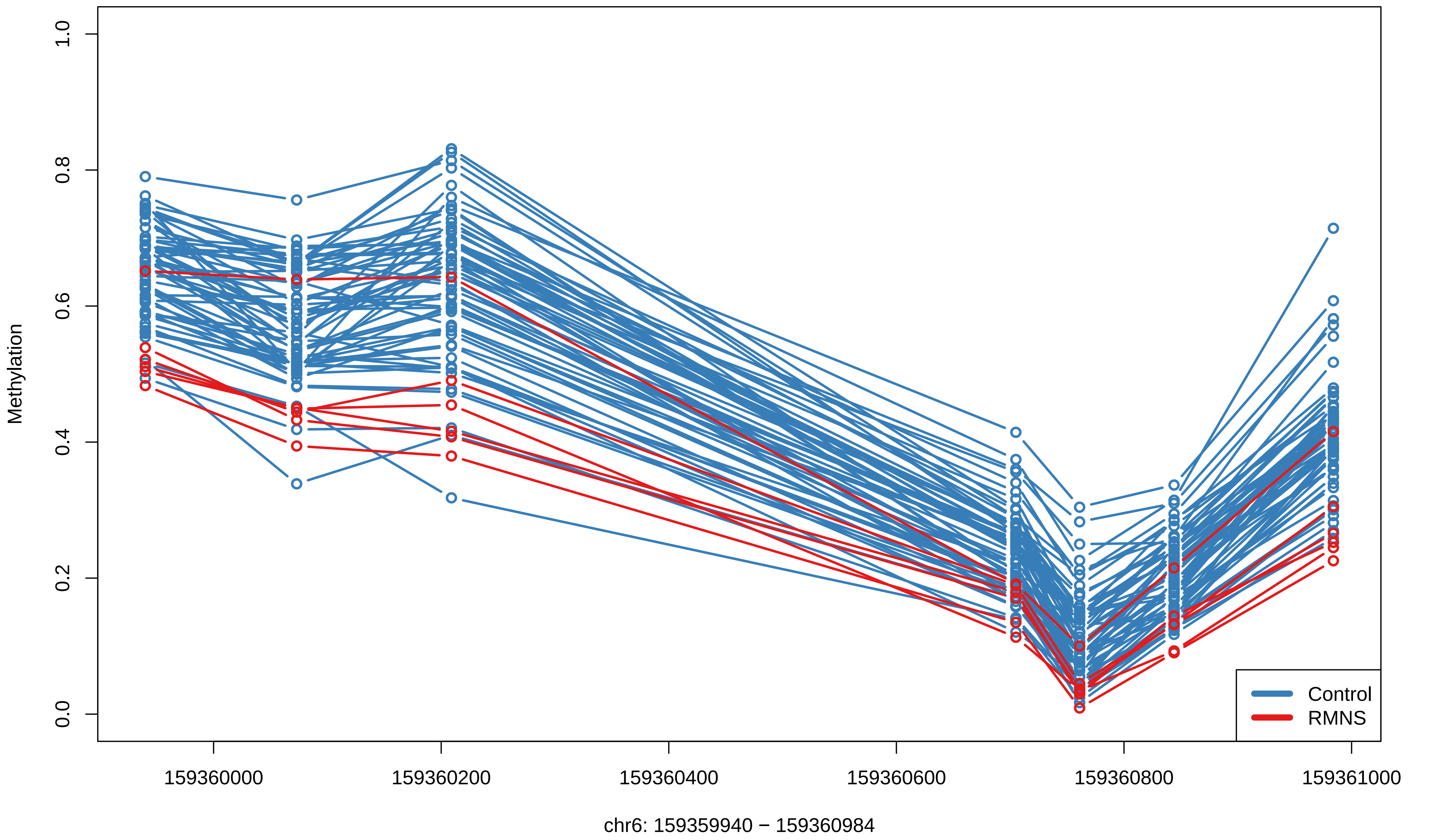

Methylation
0.0
0.2
0.4
0.6
0.8
1.0
159360000
159360200
159360400
159360600
159360800
159361000
chr6: 159359940 − 159360984
Control
RMNS

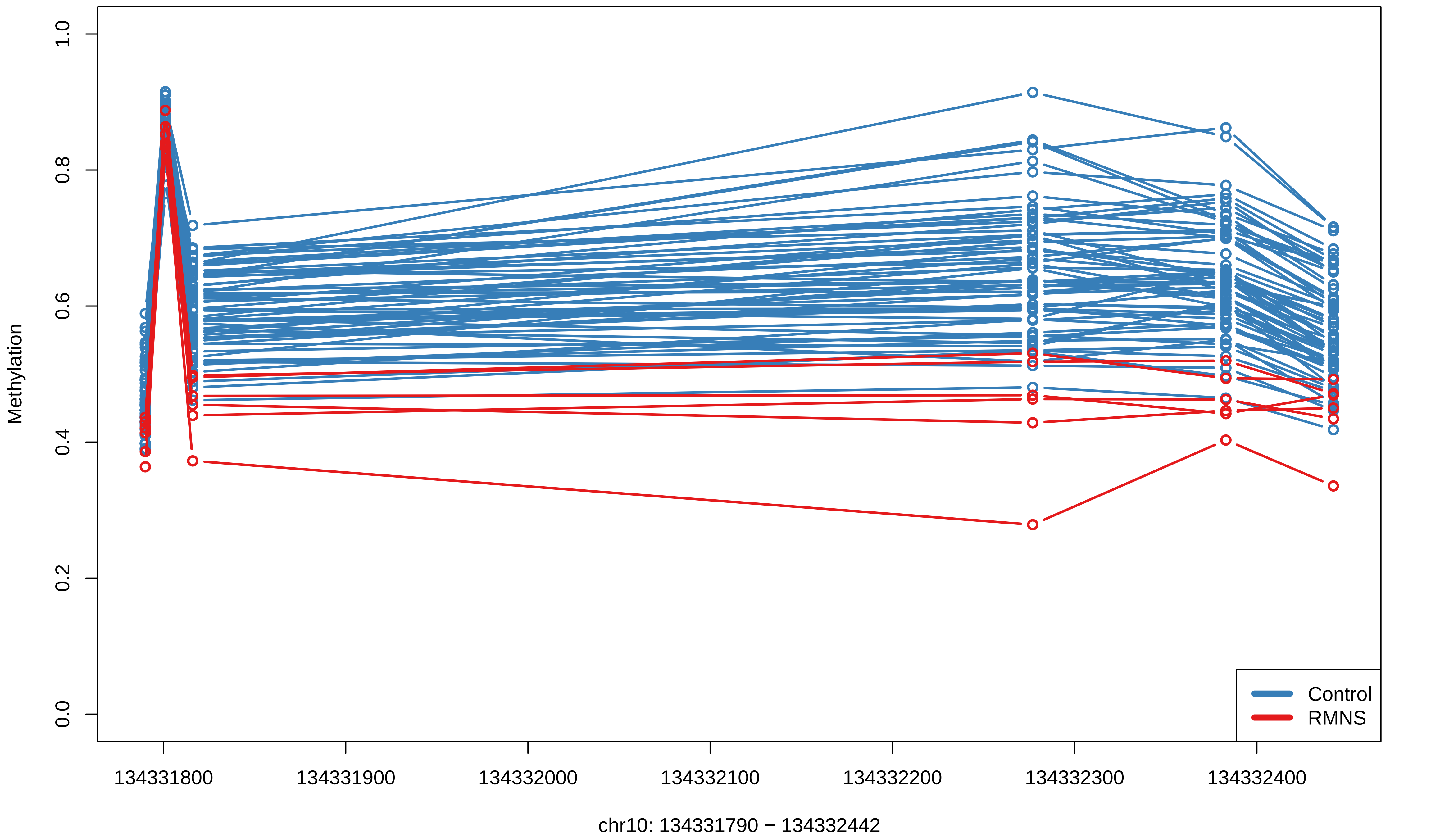

Methylation
1.0
0.8
0.6
0.4
0.2
0.0
134331800
134331900
134332000
134332100
134332200
134332300
134332400
chr10: 134331790 − 134332442
Control
RMNS

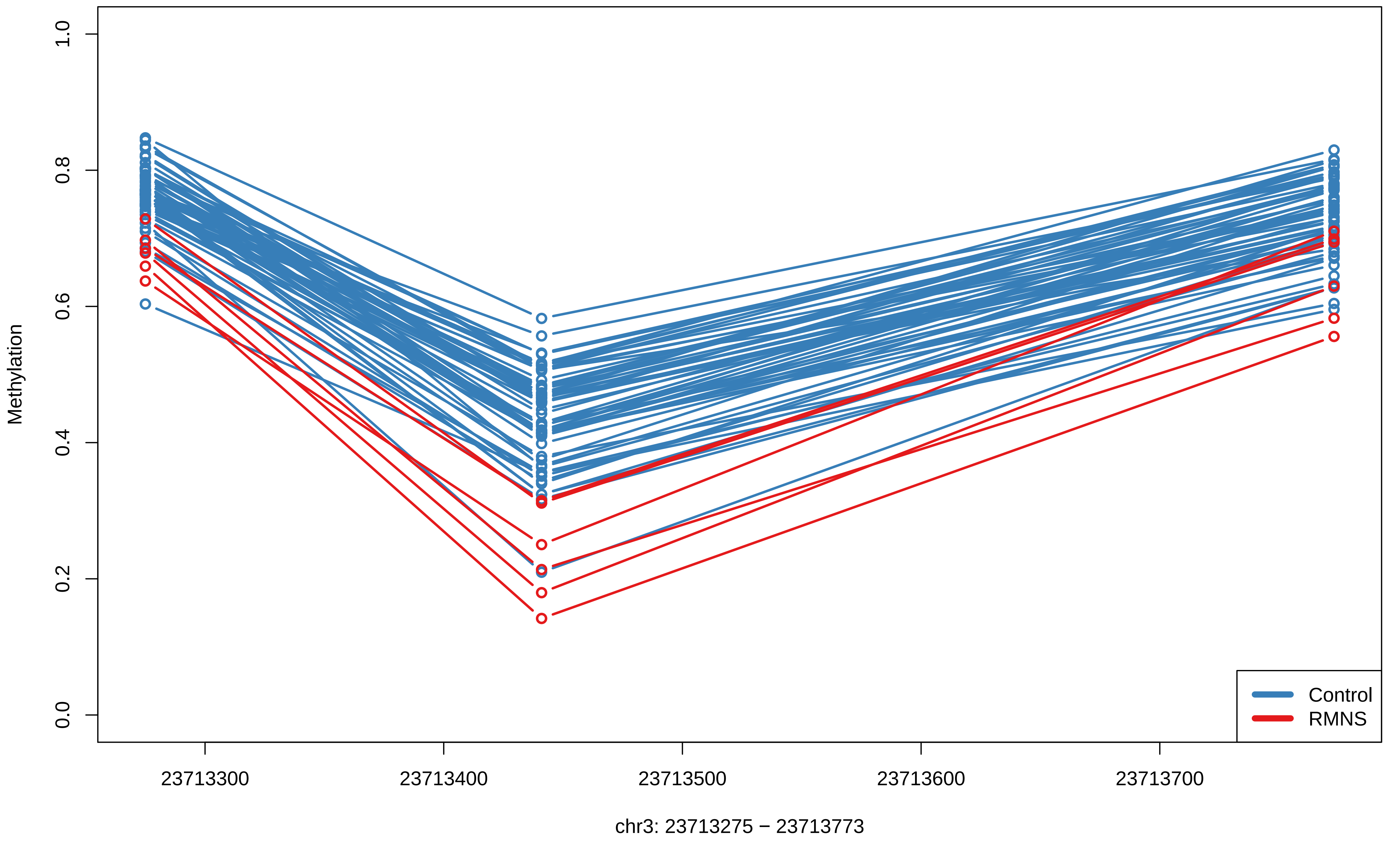

Methylation
1.0
0.8
0.6
0.4
0.2
0.0
23713300
23713400
23713500
23713600
23713700
chr3: 23713275 − 23713773
Control
RMNS

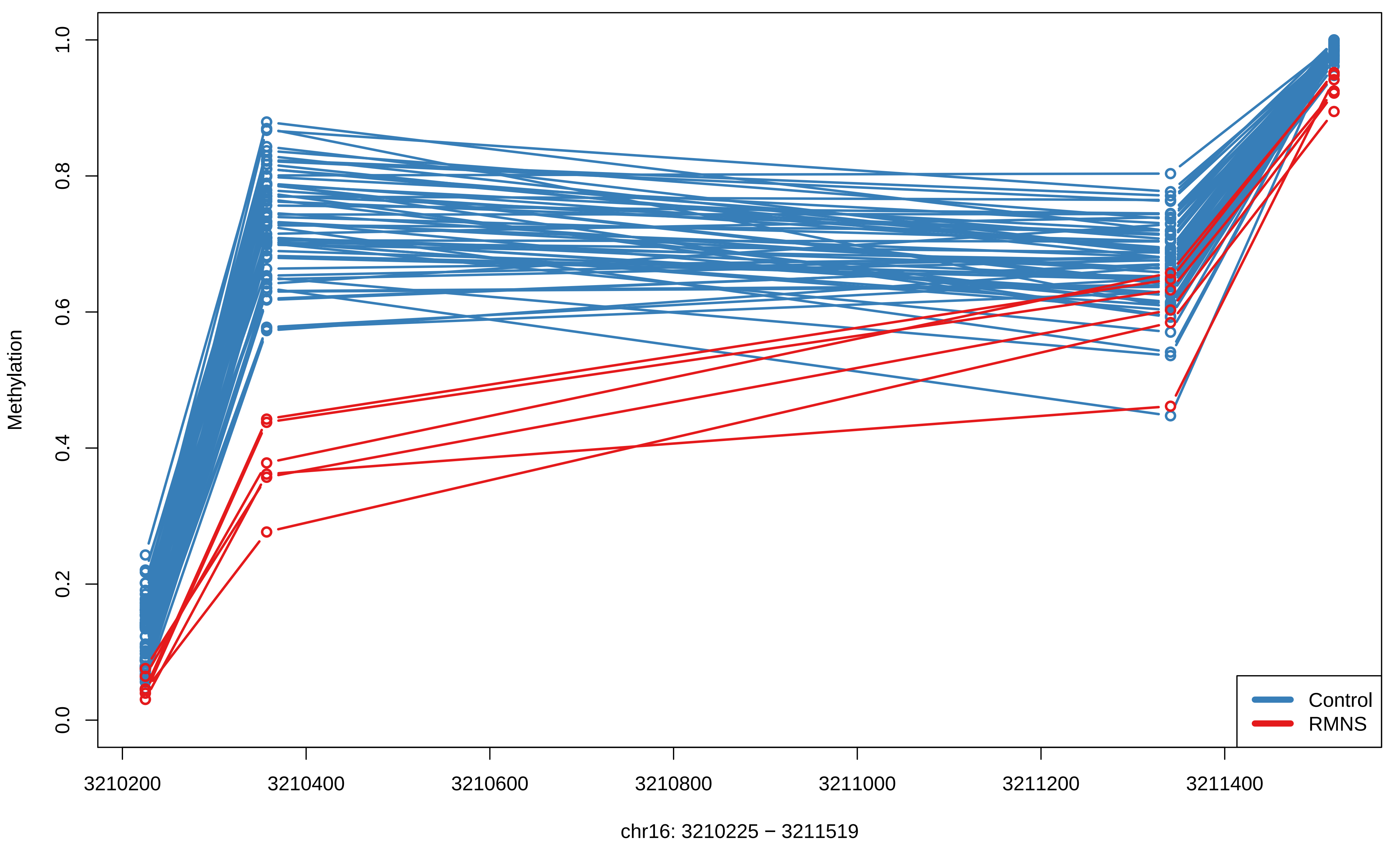

Methylation
0.0
0.2
0.4
0.6
0.8
1.0
3210200
3210400
3210600
3210800
3211000
3211200
3211400
chr16: 3210225 − 3211519
Control
RMNS

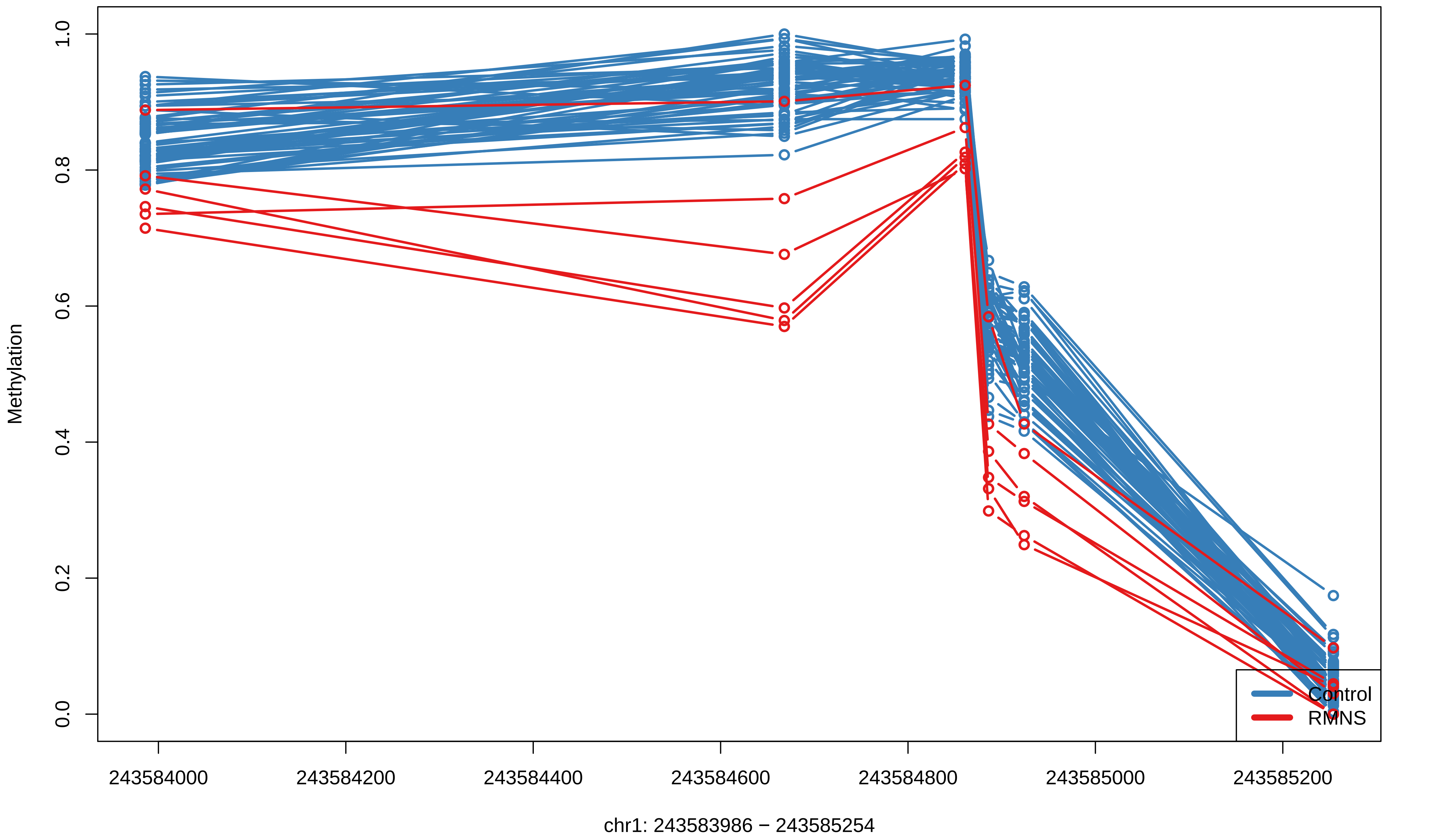

1.0
0.8
0.6
0.4
0.2
0.0
Methylation
243584000
243584200
243584400
243584600
243584800
243585000
243585200
chr1: 243583986 − 243585254
Control
RMNS

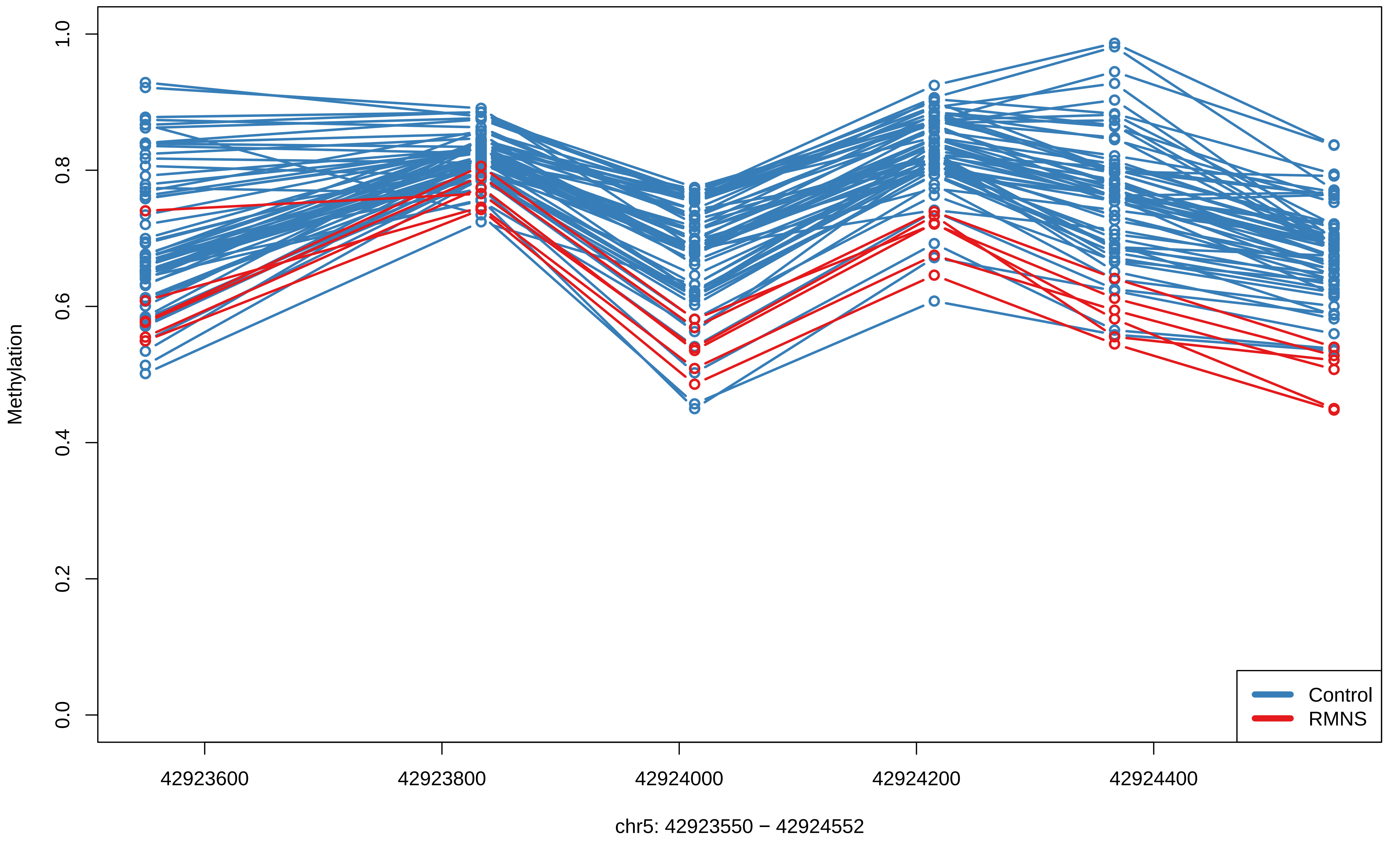

Methylation
0.0
0.2
0.4
0.6
0.8
1.0
42923600
42923800
42924000
42924200
42924400
chr5: 42923550 − 42924552
Control
RMNS

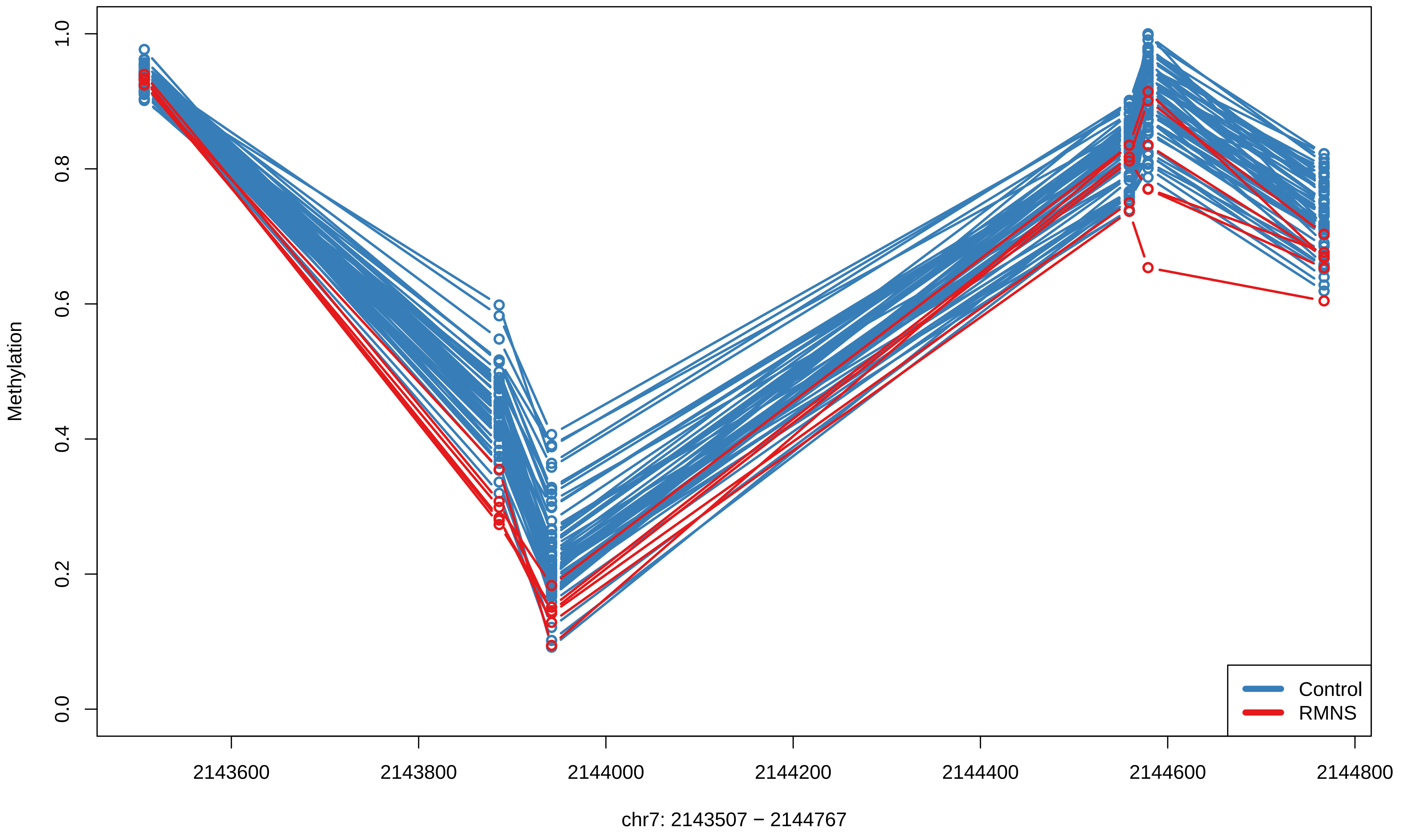

Methylation
0.0
0.2
0.4
0.6
0.8
1.0
2143600
2143800
2144000
2144200
2144400
2144600
2144800
chr7: 2143507 − 2144767
Control
RMNS

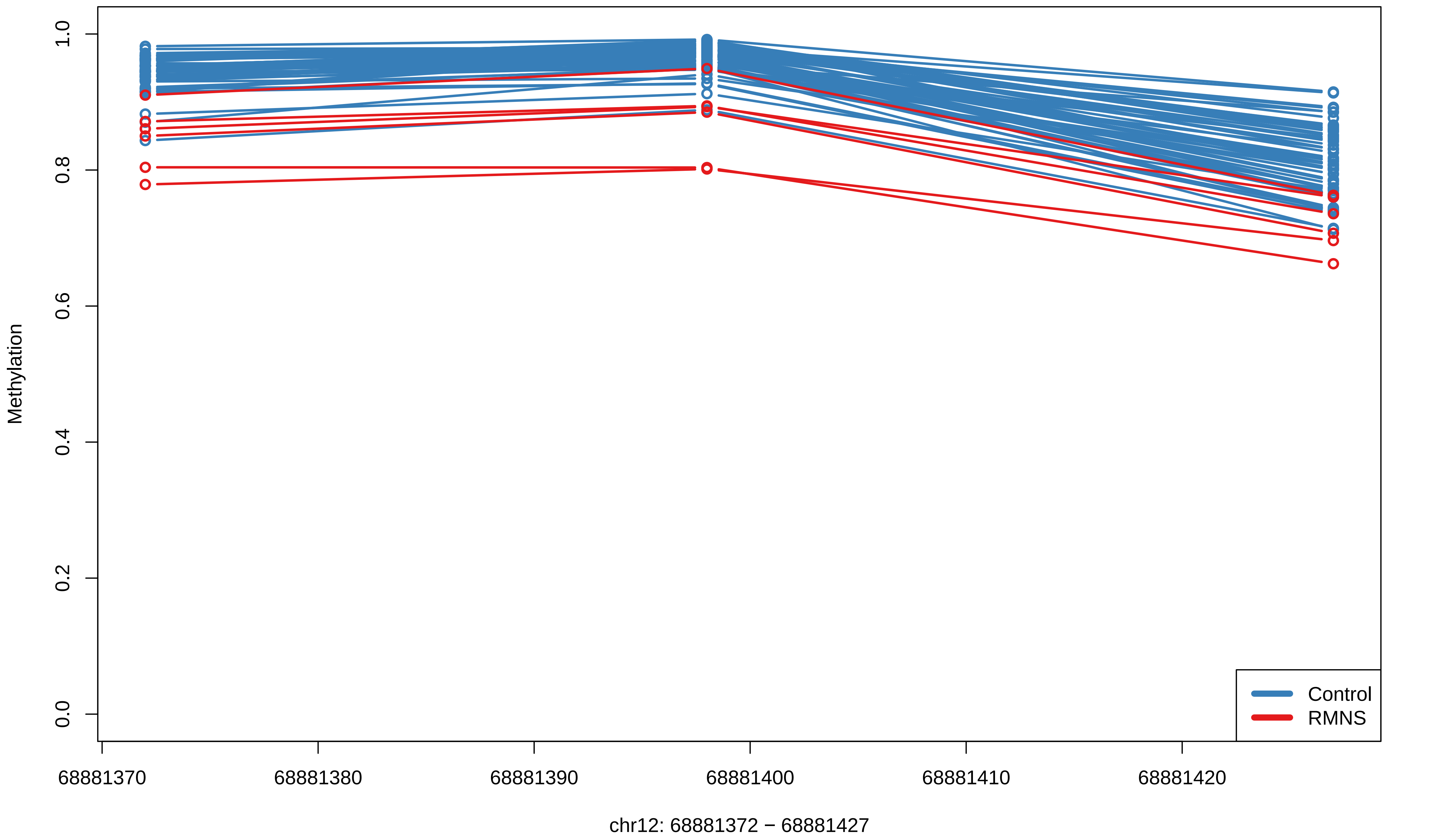

Methylation
1.0
0.8
0.6
0.4
0.2
0.0
68881370
68881380
68881390
68881400
68881410
68881420
chr12: 68881372 − 68881427
Control
RMNS

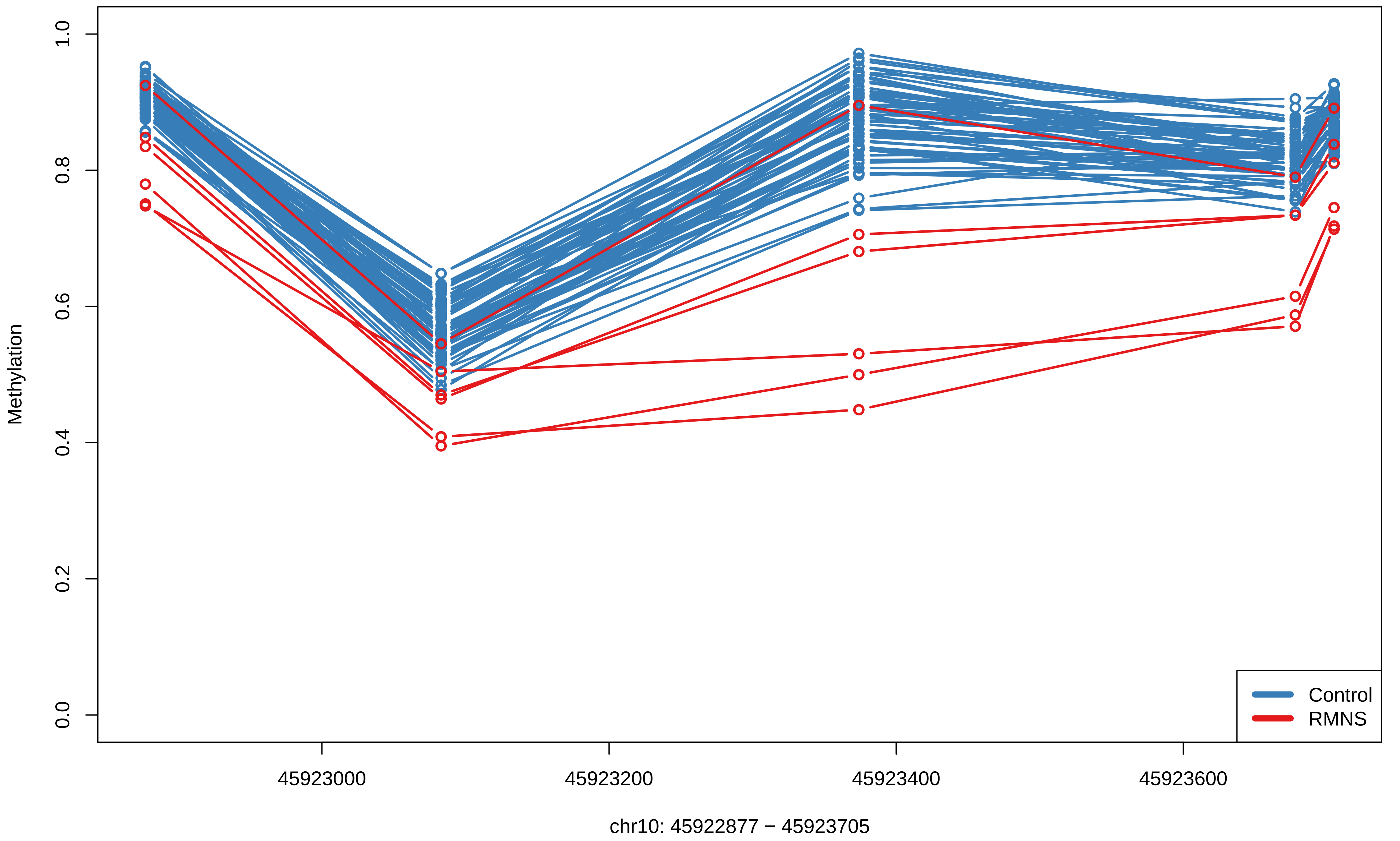

1.0
0.8
0.6
0.4
0.2
0.0
Methylation
45923000
45923200
45923400
45923600
chr10: 45922877 − 45923705
Control
RMNS

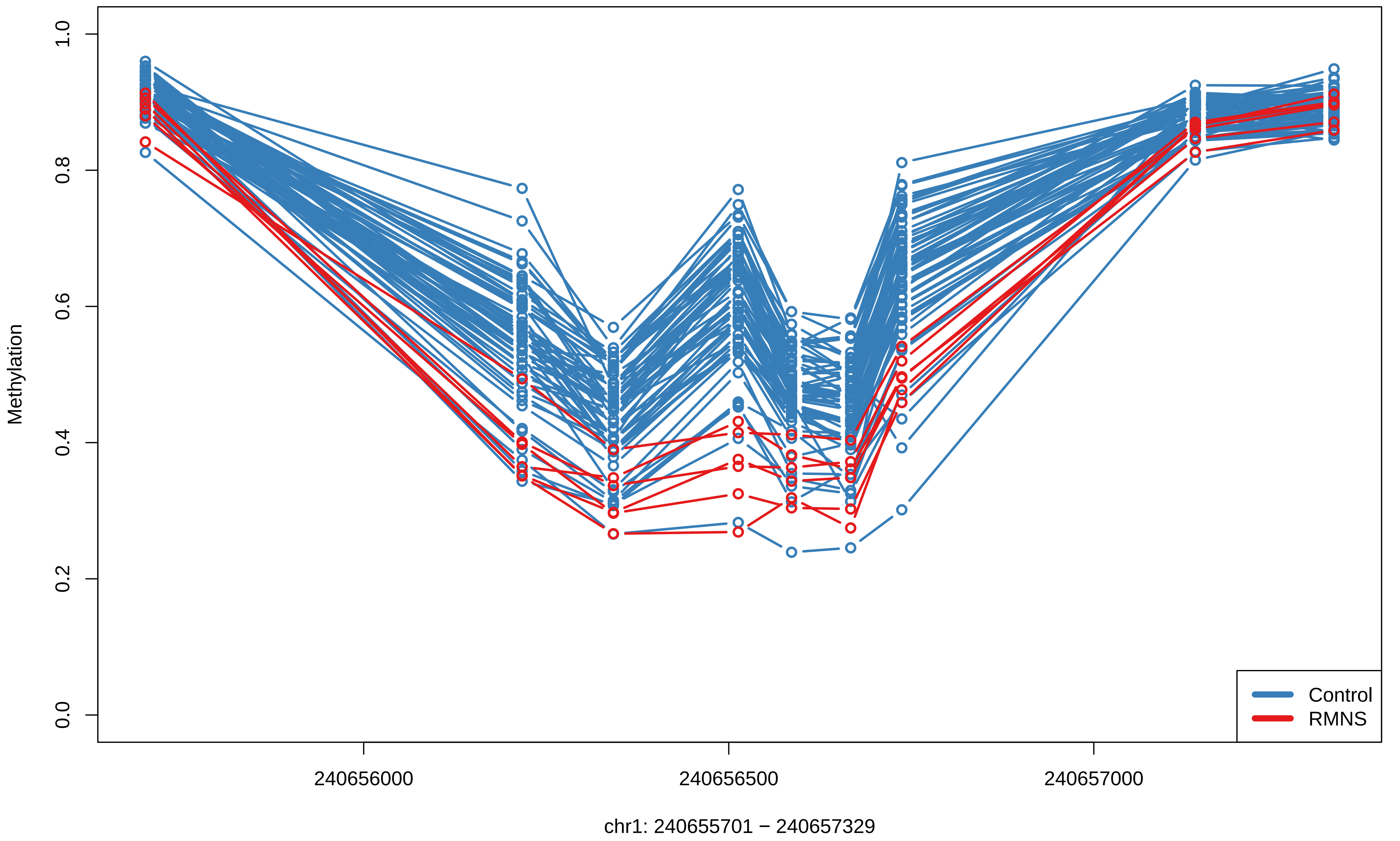
Methylation
1.0
0.8
0.6
0.4
0.2
0.0
240656000
240656500
240657000
chr1: 240655701 − 240657329
Control
RMNS

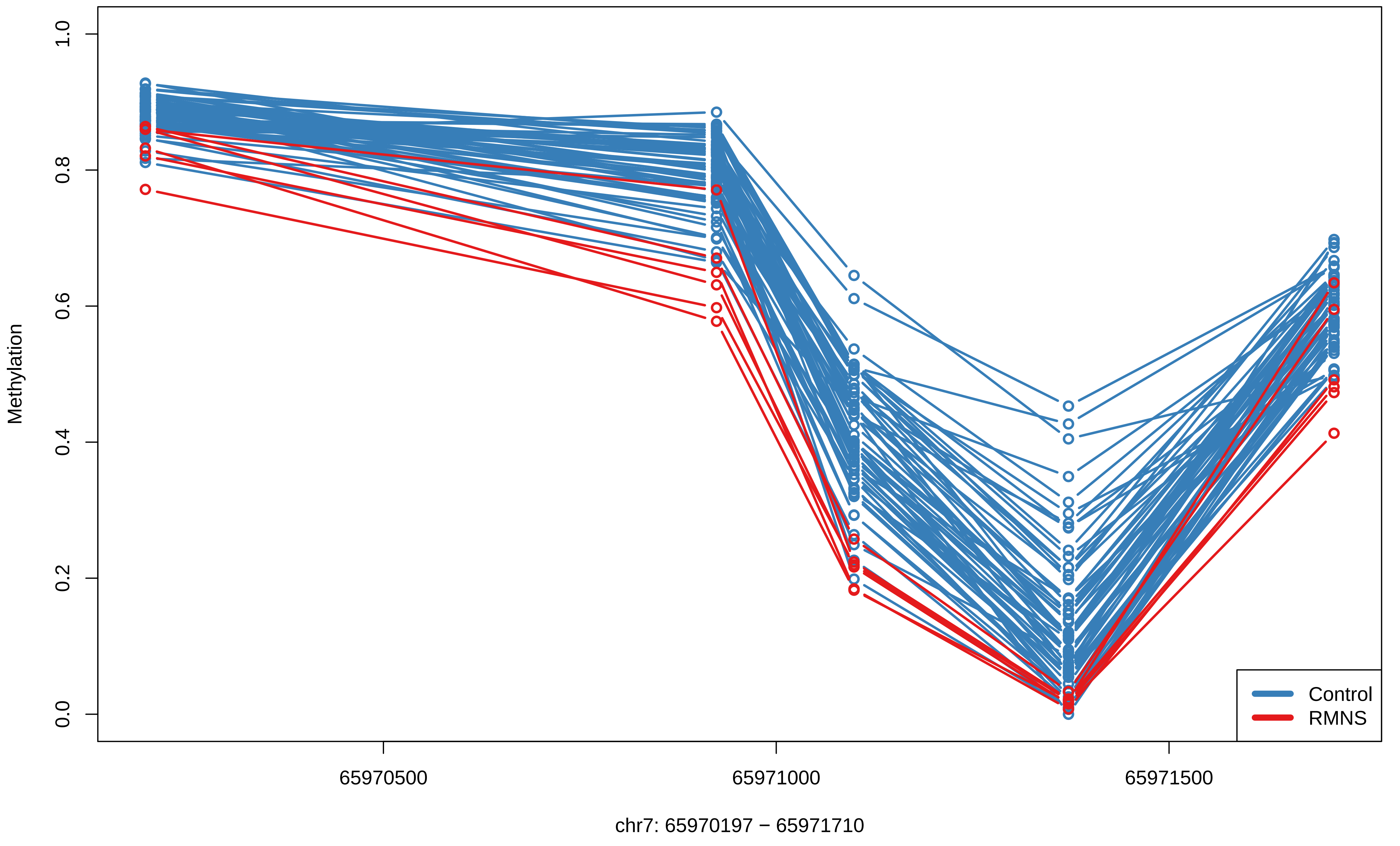
Methylation
0.0
0.2
0.4
0.6
0.8
1.0
65970500
65971000
65971500
chr7: 65970197 − 65971710
Control
RMNS

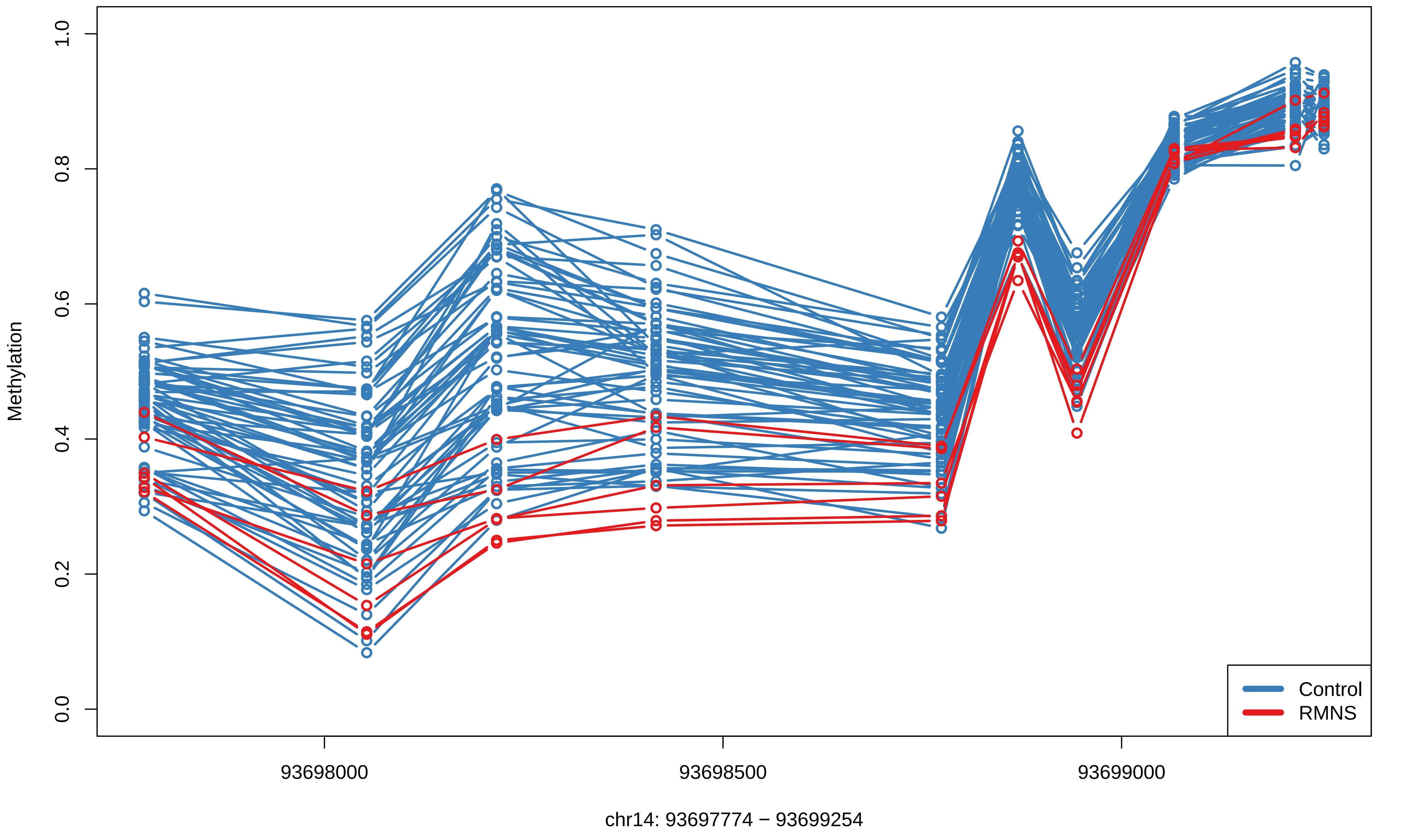
1.0
0.8
0.6
0.4
0.2
0.0
Methylation
93698000
93698500
93699000
chr14: 93697774 − 93699254
Control
RMNS

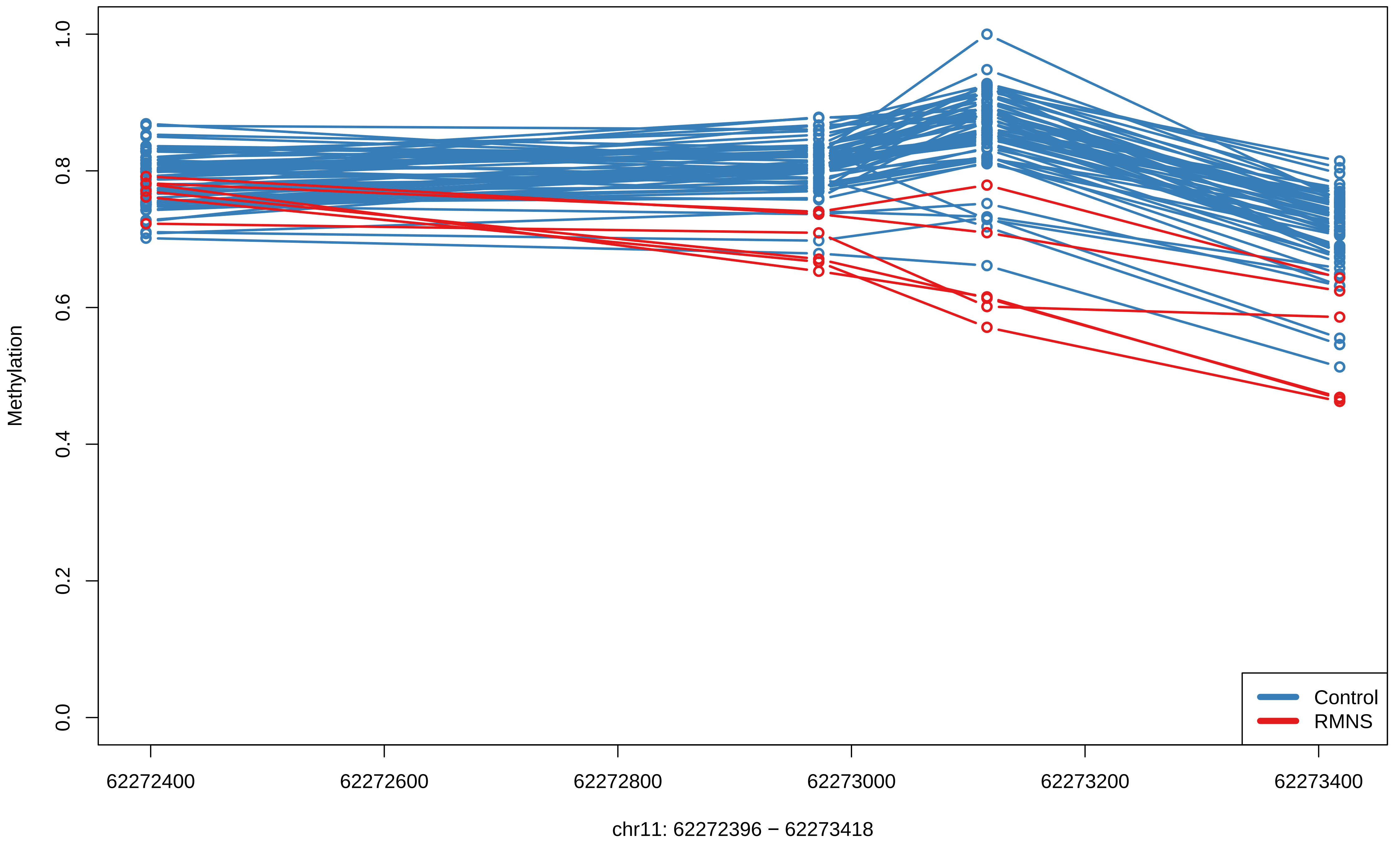
Methylation
0.0
0.2
0.4
0.6
0.8
1.0
62272400
62272600
62272800
62273000
62273200
62273400
chr11: 62272396 − 62273418
Control
RMNS

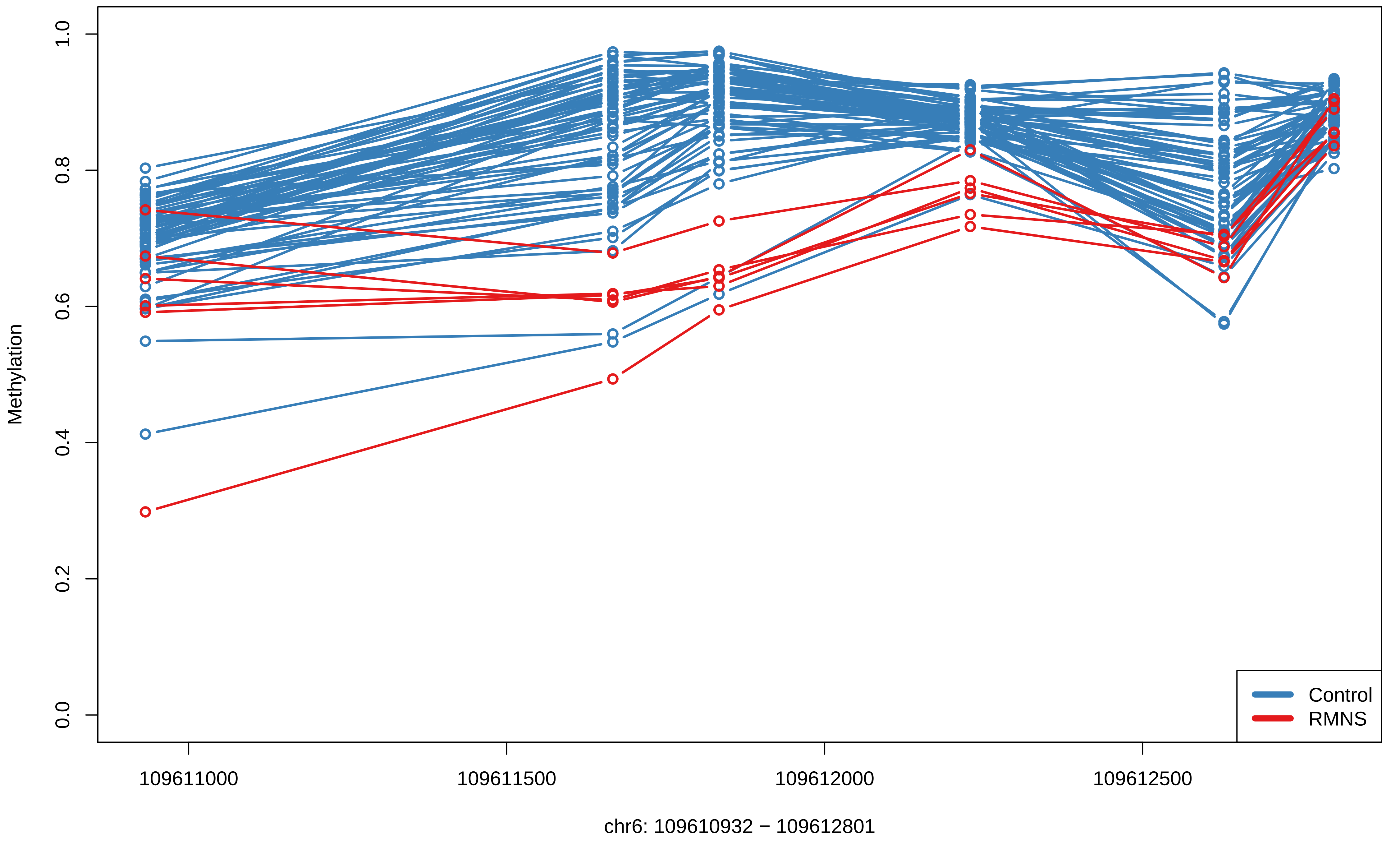

1.0
0.8
0.6
0.4
0.2
0.0
Methylation
109611000
109611500
109612000
109612500
chr6: 109610932 − 109612801
Control
RMNS

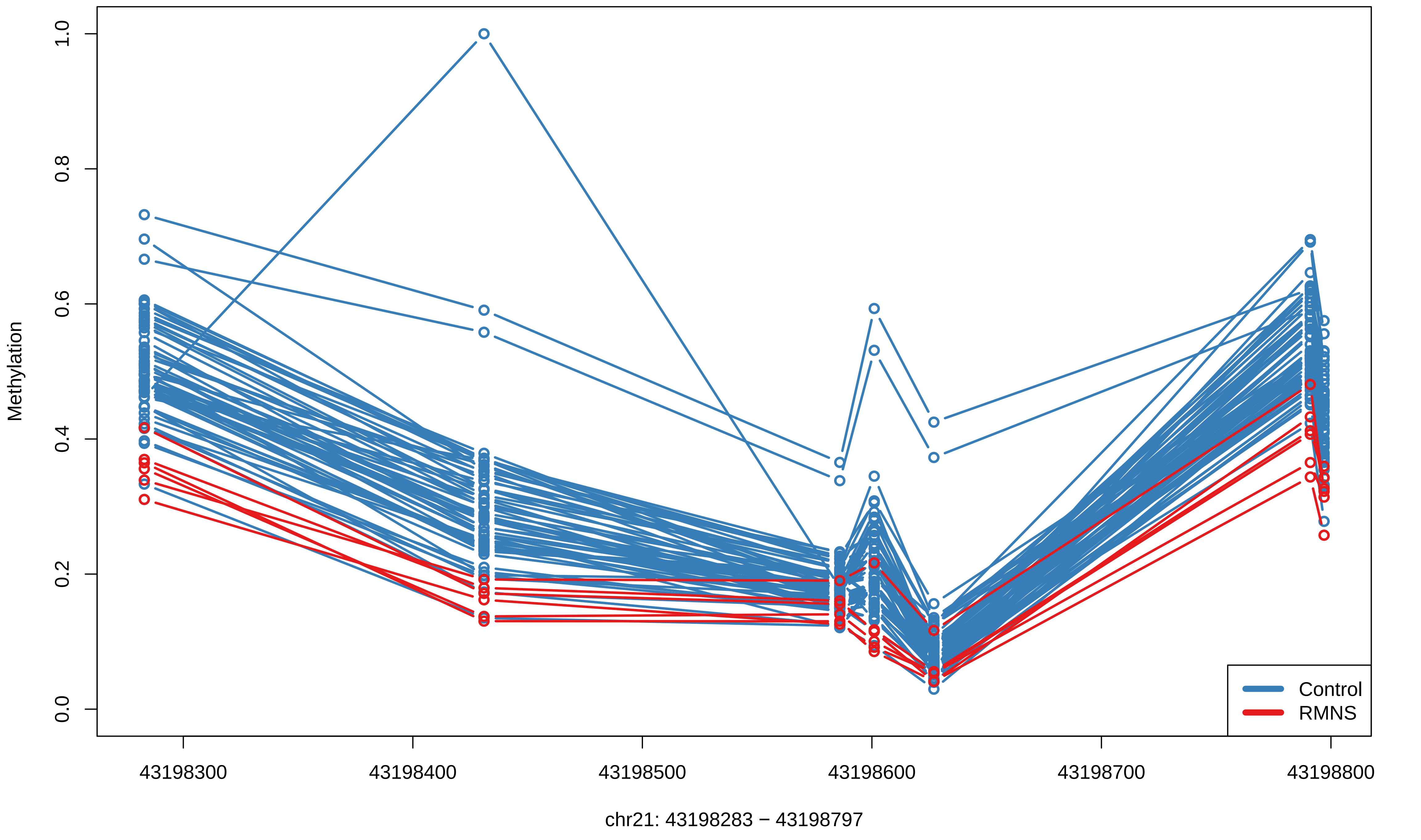

Methylation
1.0
0.8
0.6
0.4
0.2
0.0
43198300
43198400
43198500
43198600
43198700
43198800
chr21: 43198283 − 43198797
Control
RMNS

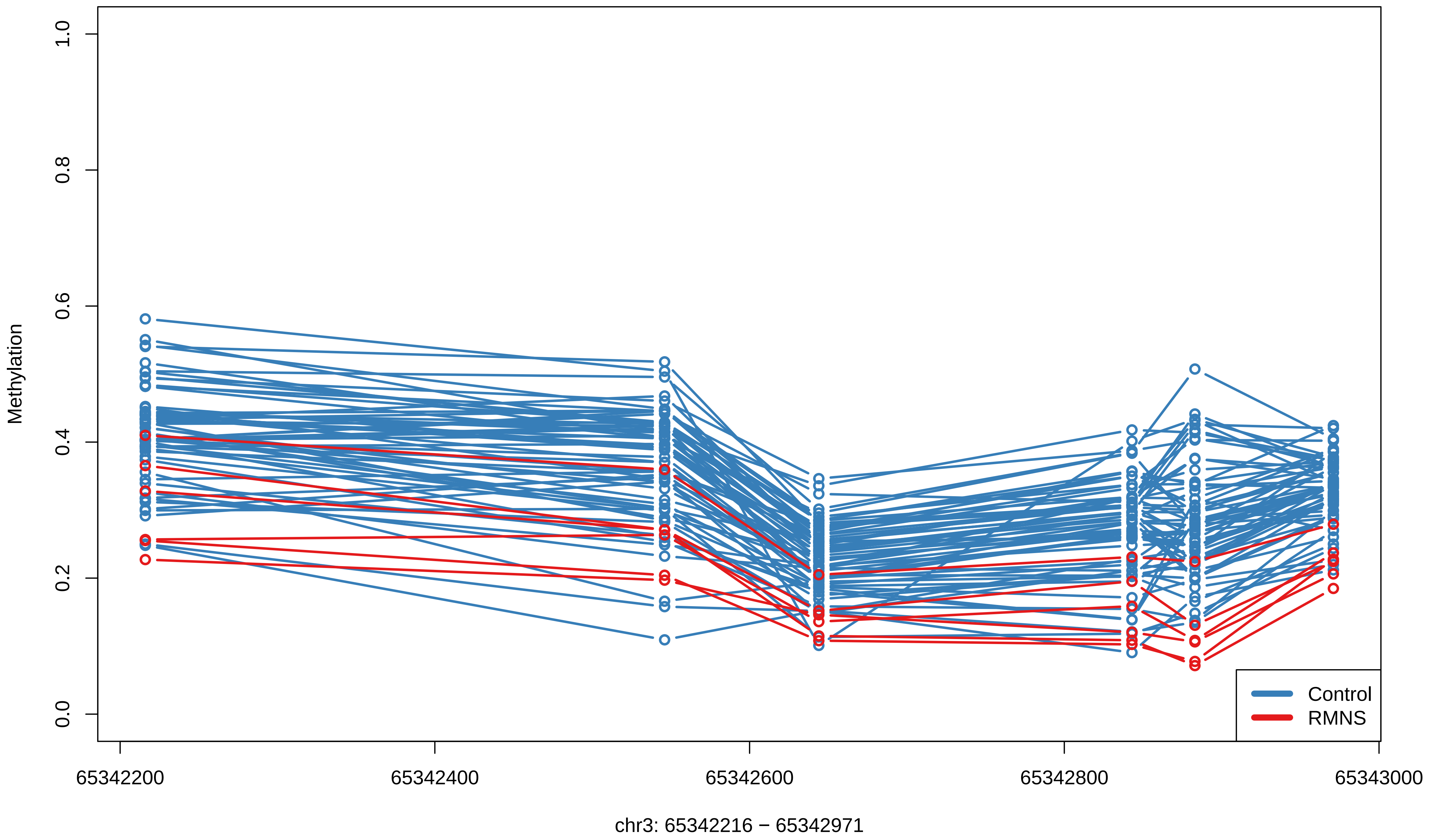
Methylation
1.0
0.8
0.6
0.4
0.2
0.0
65342200
65342400
65342600
65342800
65343000
chr3: 65342216 − 65342971
Control
RMNS

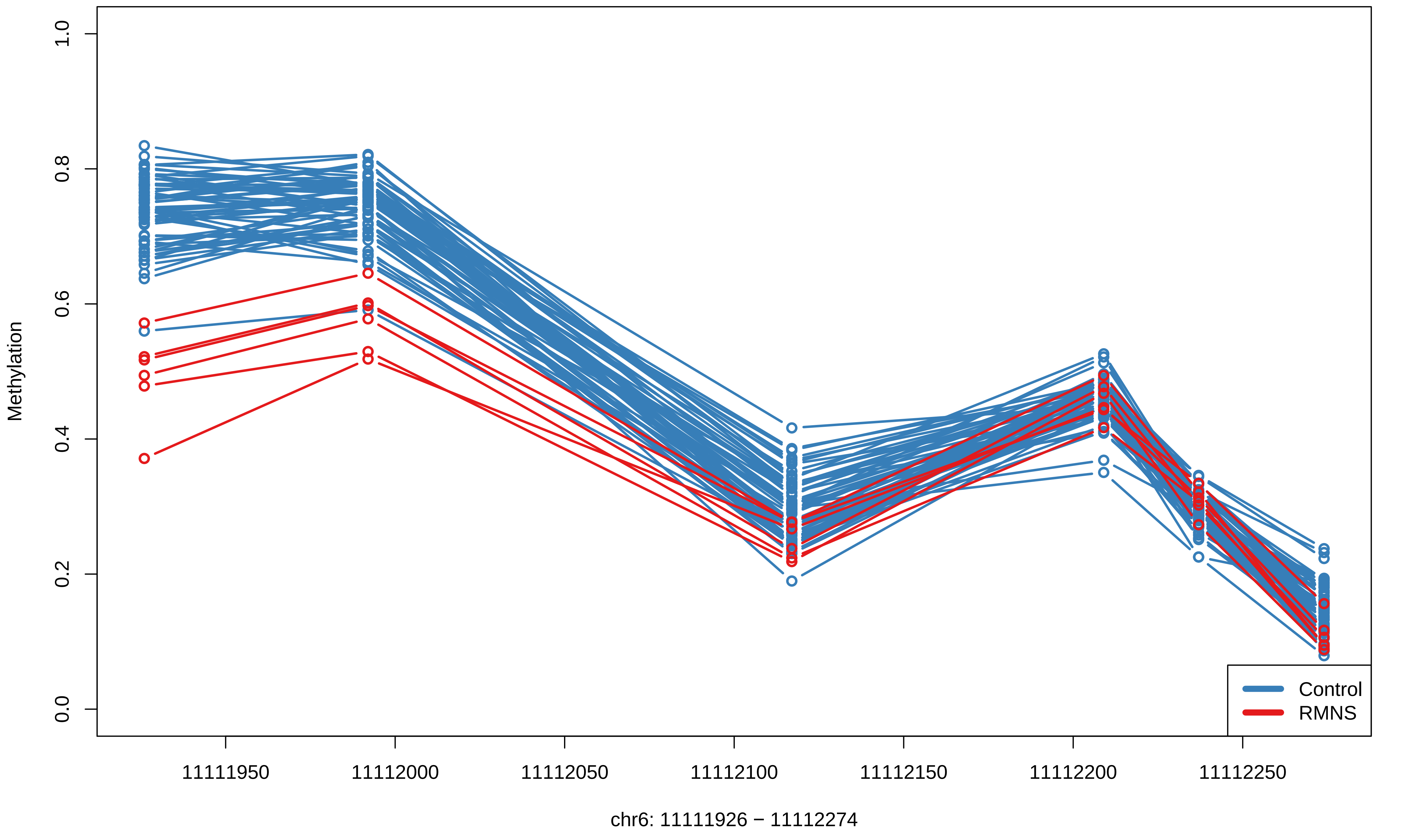

Methylation
0.0
0.2
0.4
0.6
0.8
1.0
11111950
11112000
11112050
11112100
11112150
11112200
11112250
chr6: 11111926 − 11112274
Control
RMNS

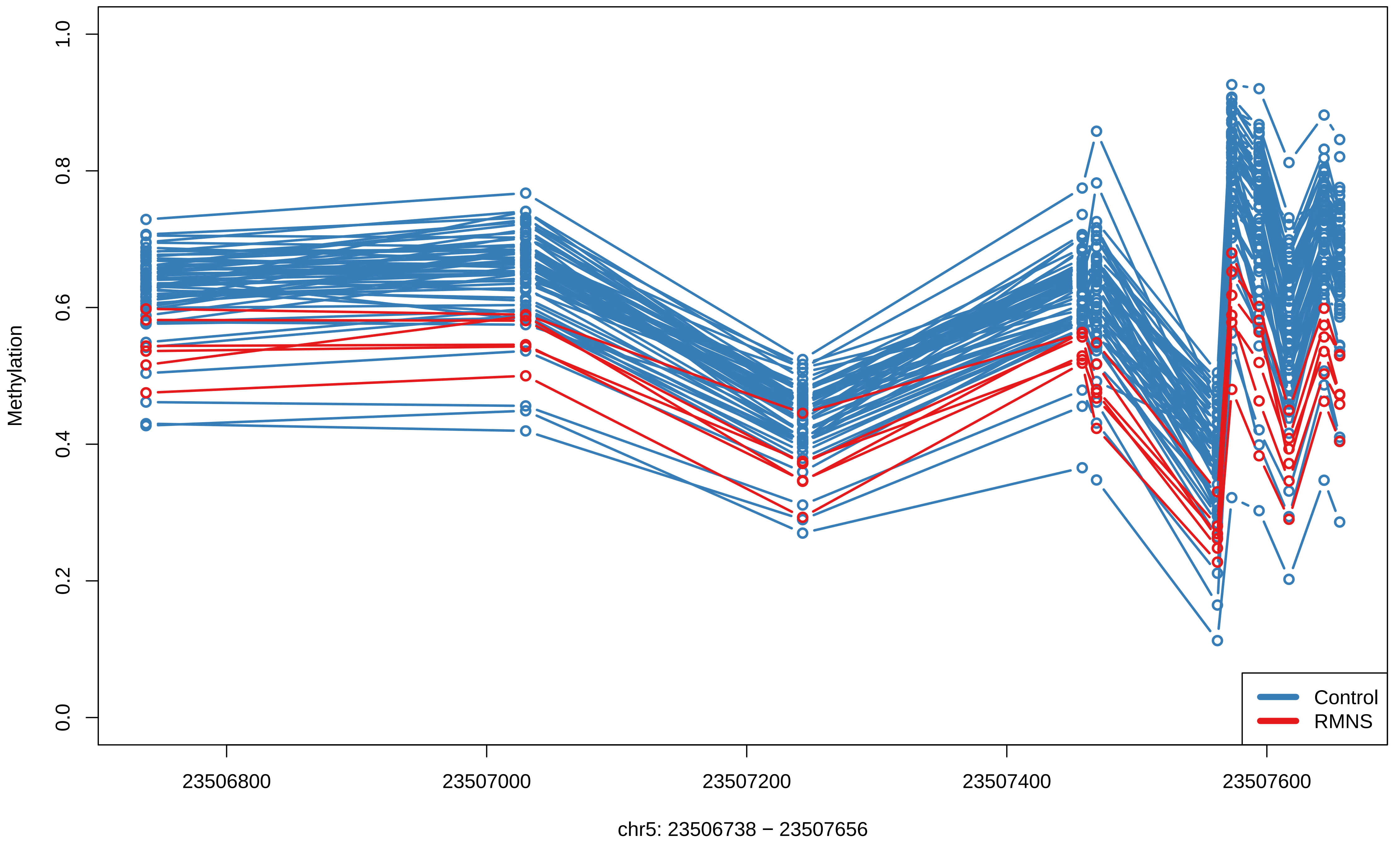

Methylation
0.0
0.2
0.4
0.6
0.8
1.0
23506800
23507000
23507200
23507400
23507600
chr5: 23506738 − 23507656
Control
RMNS

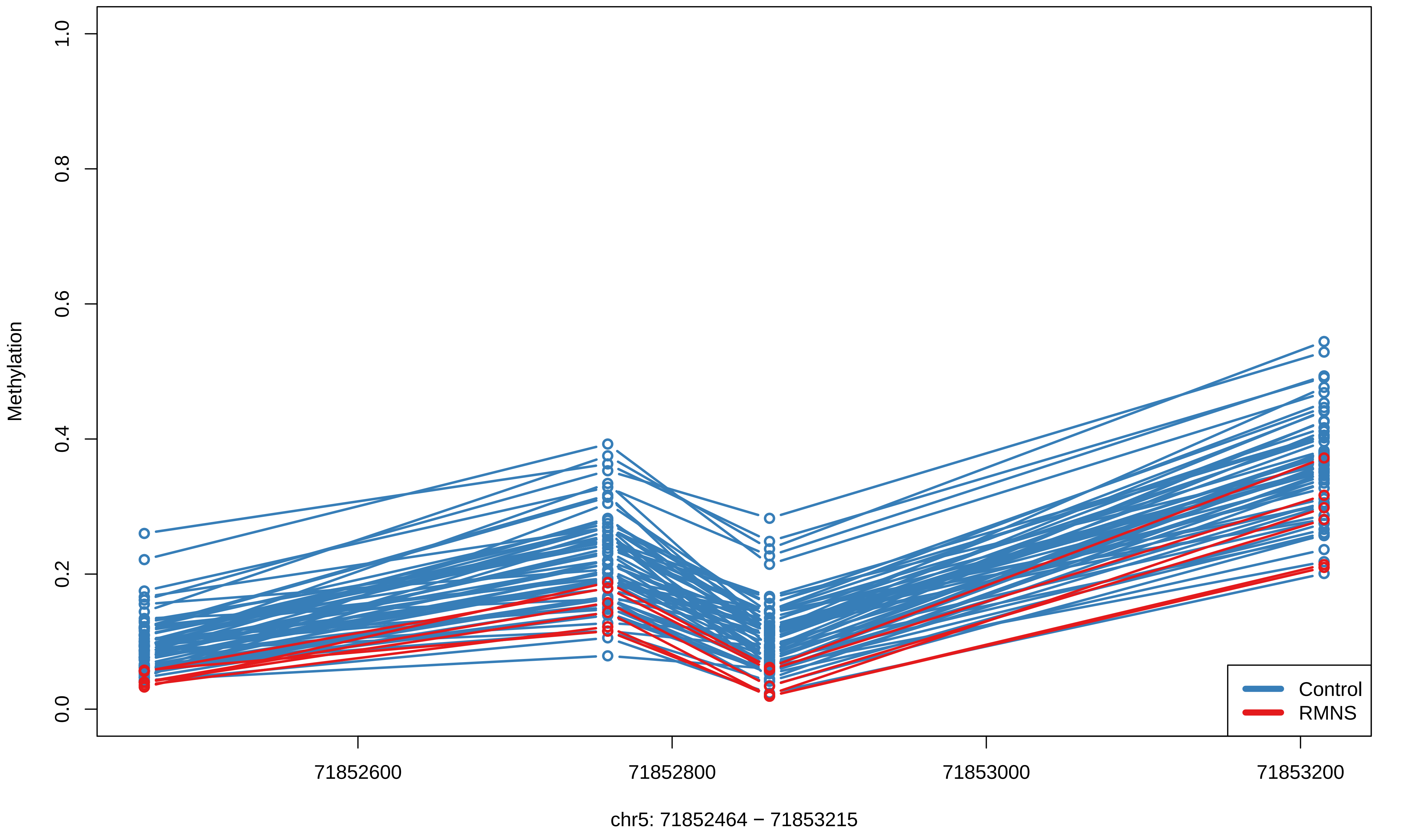

Methylation
0.0
0.2
0.4
0.6
0.8
1.0
71852600
71852800
71853000
71853200
chr5: 71852464 − 71853215
Control
RMNS

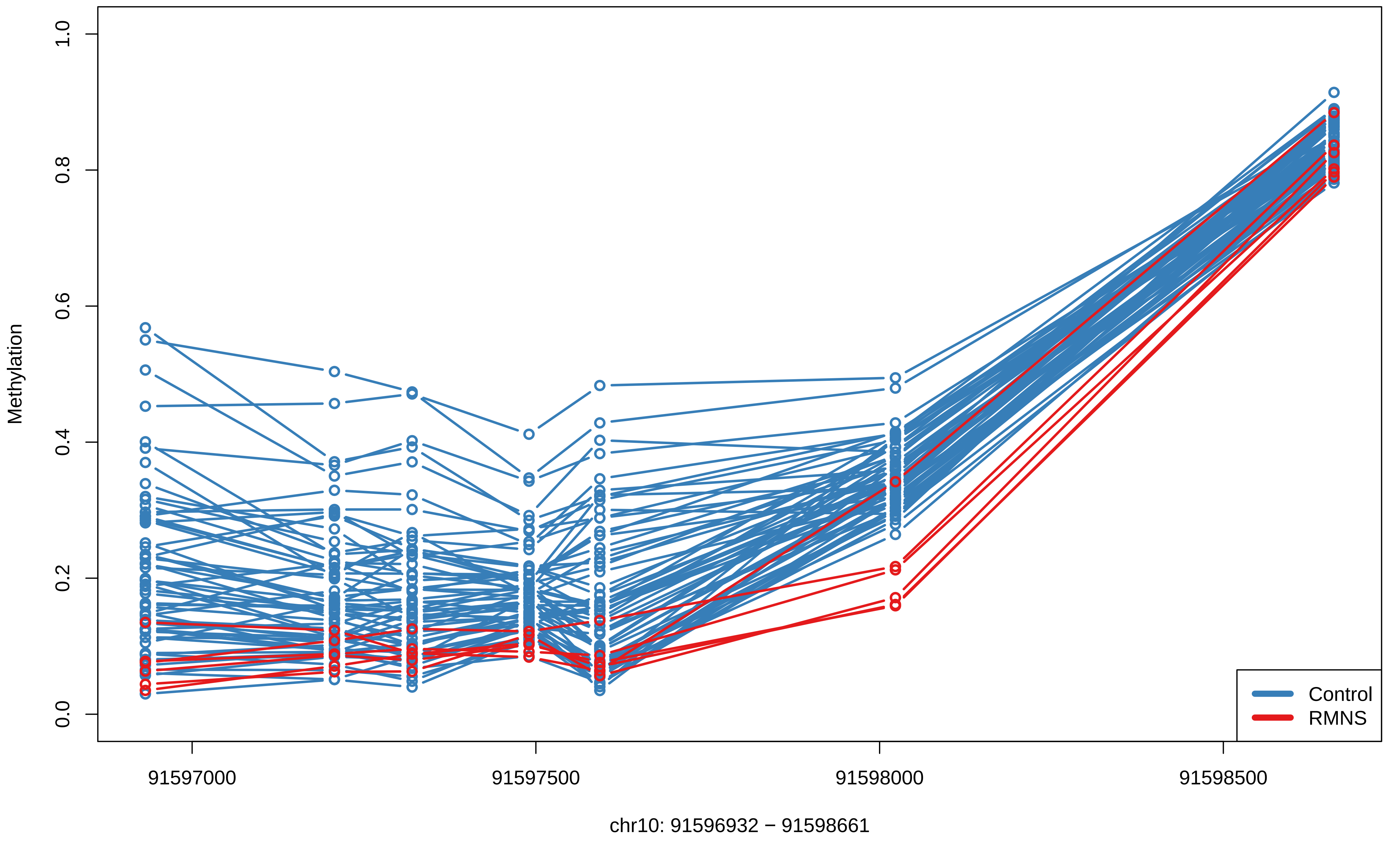

Methylation
0.0
0.2
0.4
0.6
0.8
1.0
91597000
91597500
91598000
91598500
chr10: 91596932 − 91598661
Control
RMNS

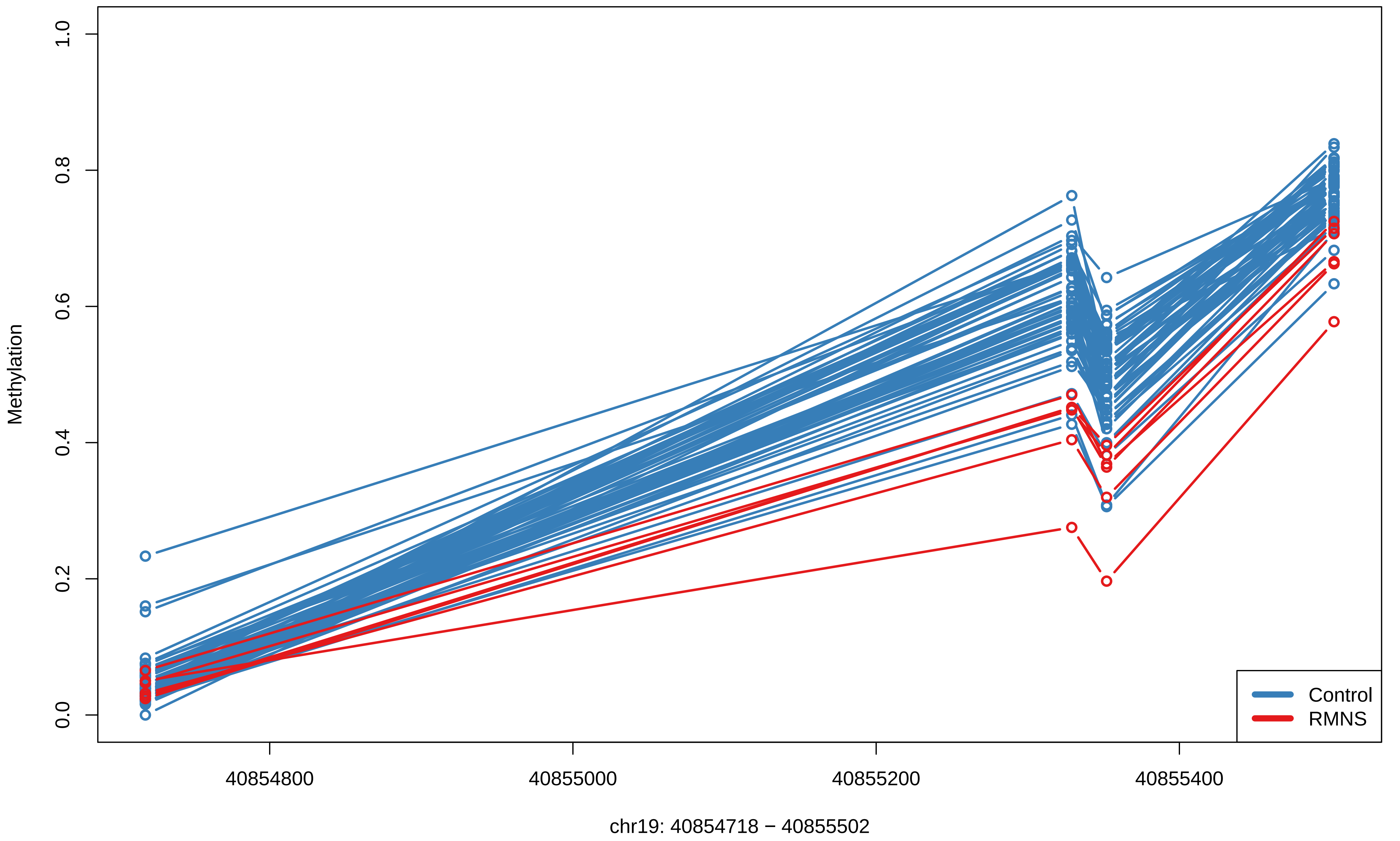

Methylation
0.0
0.2
0.4
0.6
0.8
1.0
40854800
40855000
40855200
40855400
chr19: 40854718 − 40855502
Control
RMNS

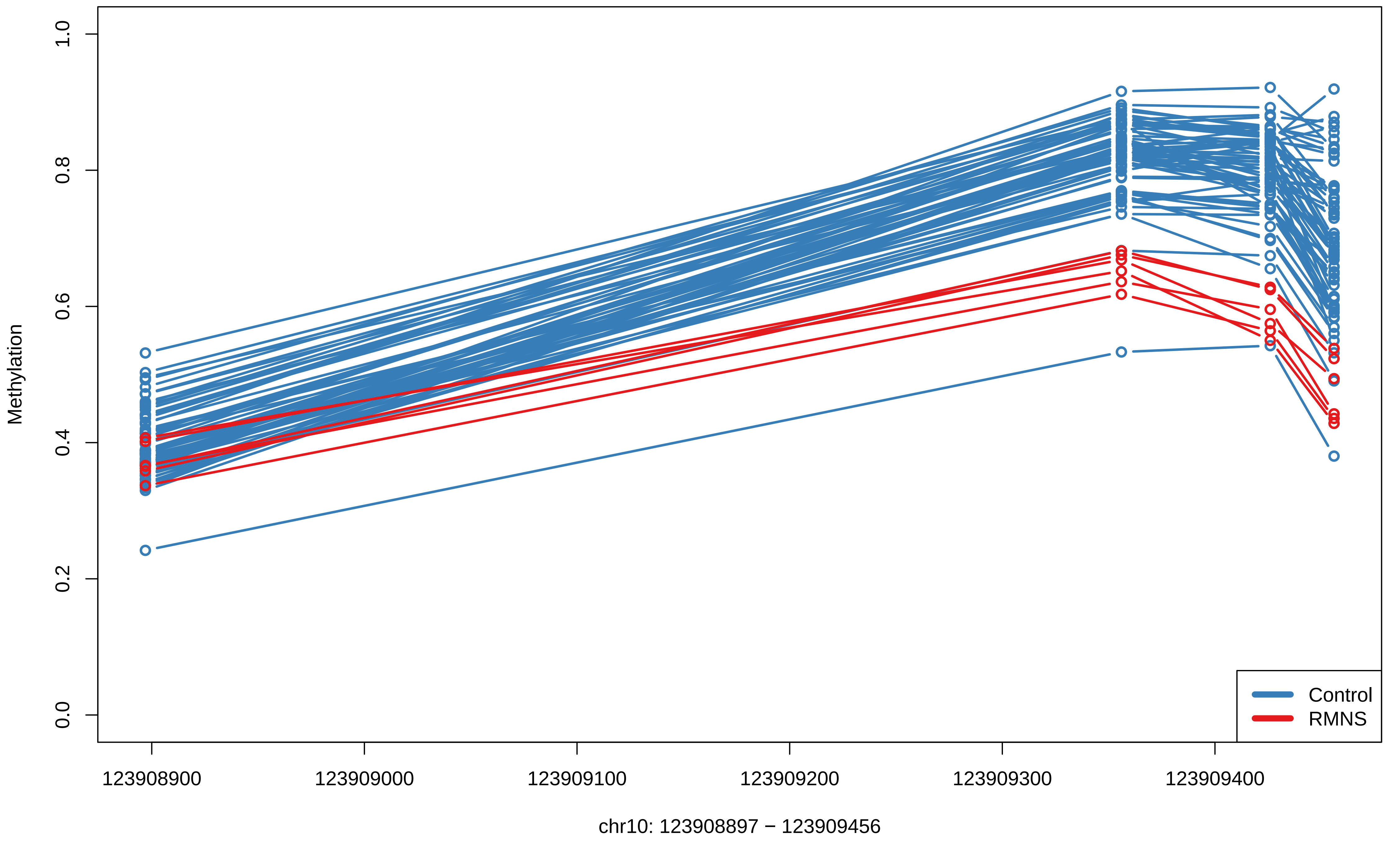

Methylation
0.0
0.2
0.4
0.6
0.8
1.0
123908900
123909000
123909100
123909200
123909300
123909400
chr10: 123908897 − 123909456
Control
RMNS

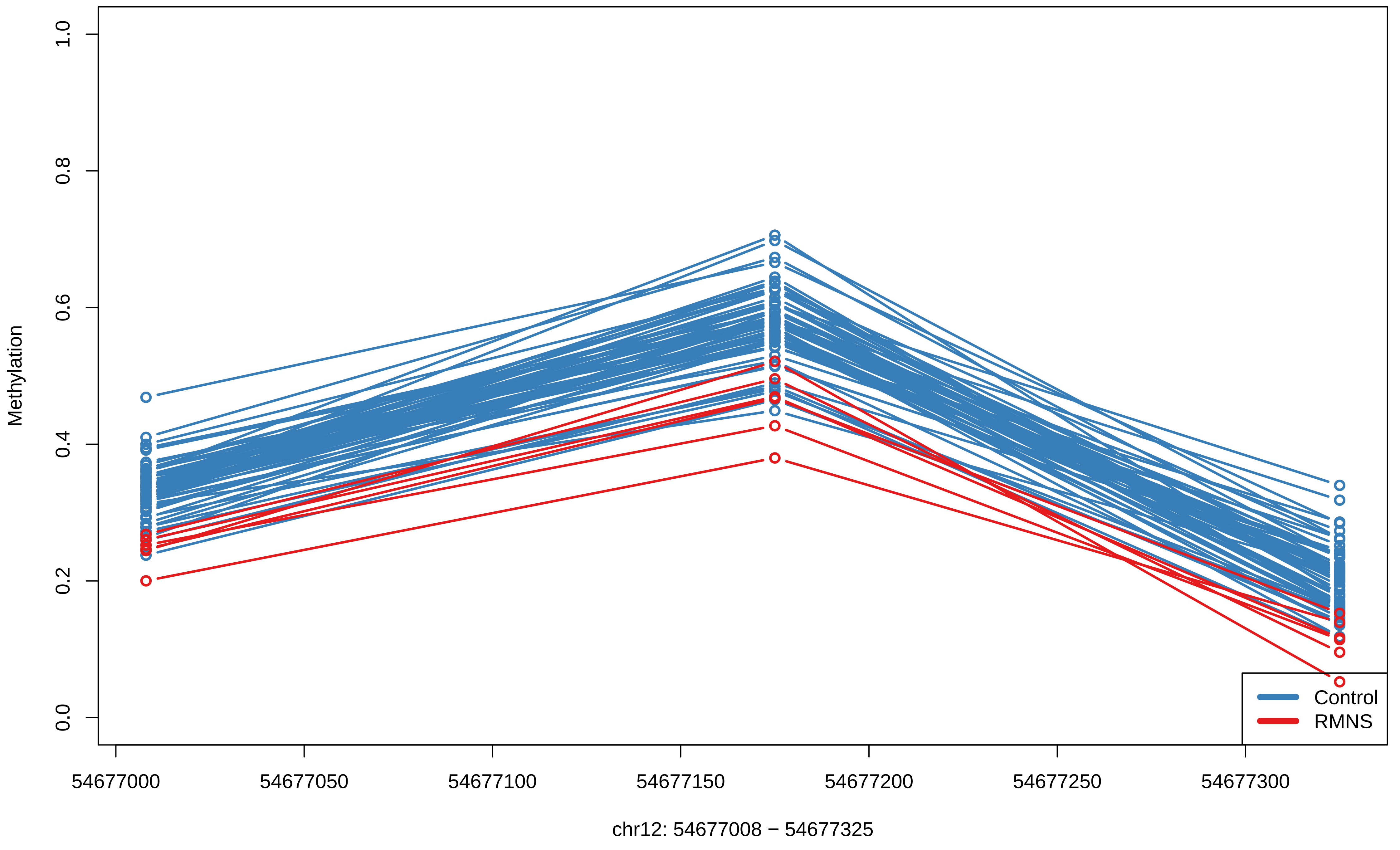

Methylation
0.0
0.2
0.4
0.6
0.8
1.0
54677000
54677050
54677100
54677150
54677200
54677250
54677300
chr12: 54677008 − 54677325
Control
RMNS

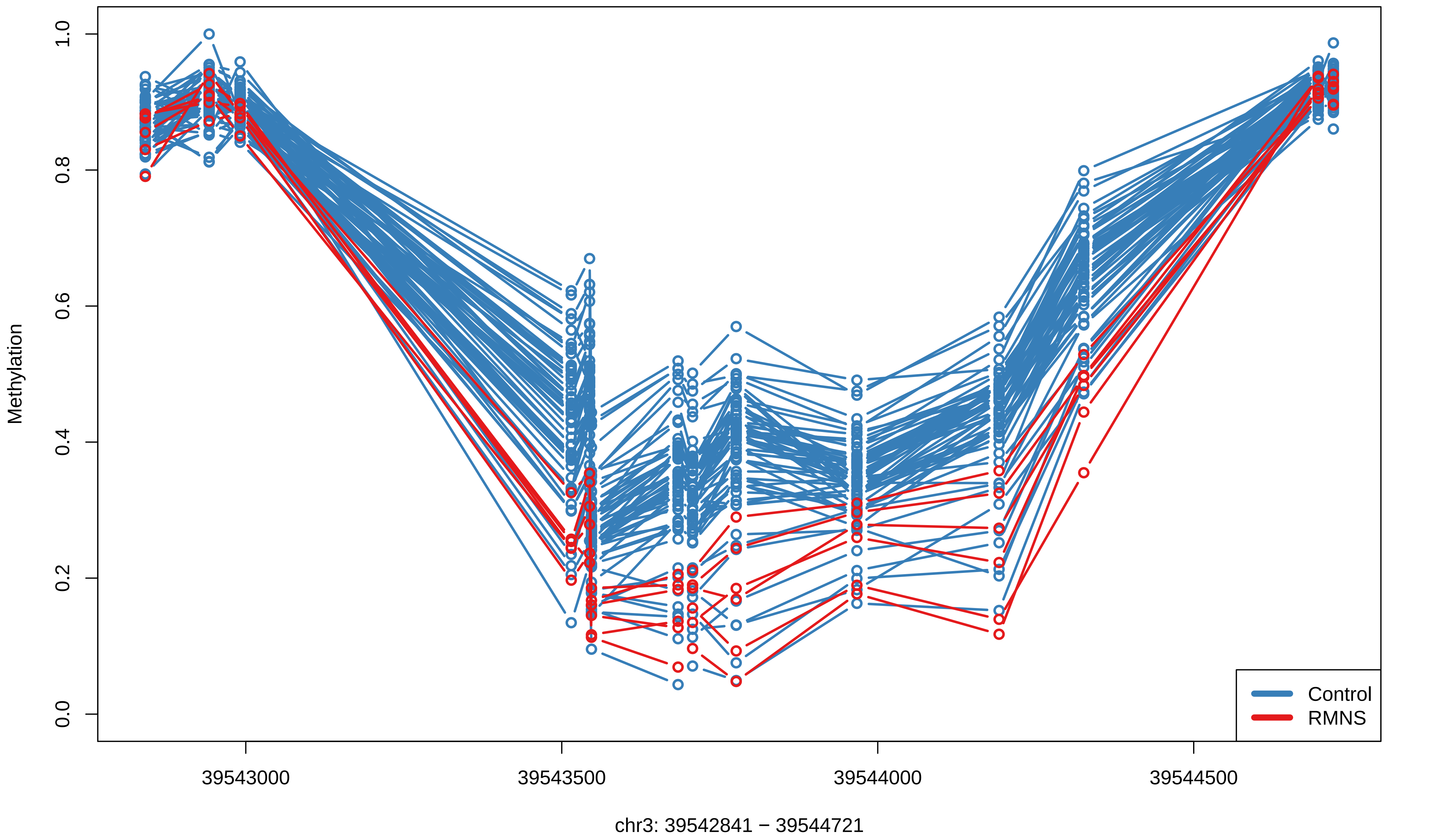

Methylation
1.0
0.8
0.6
0.4
0.2
0.0
39543000
39543500
39544000
39544500
chr3: 39542841 − 39544721
Control
RMNS

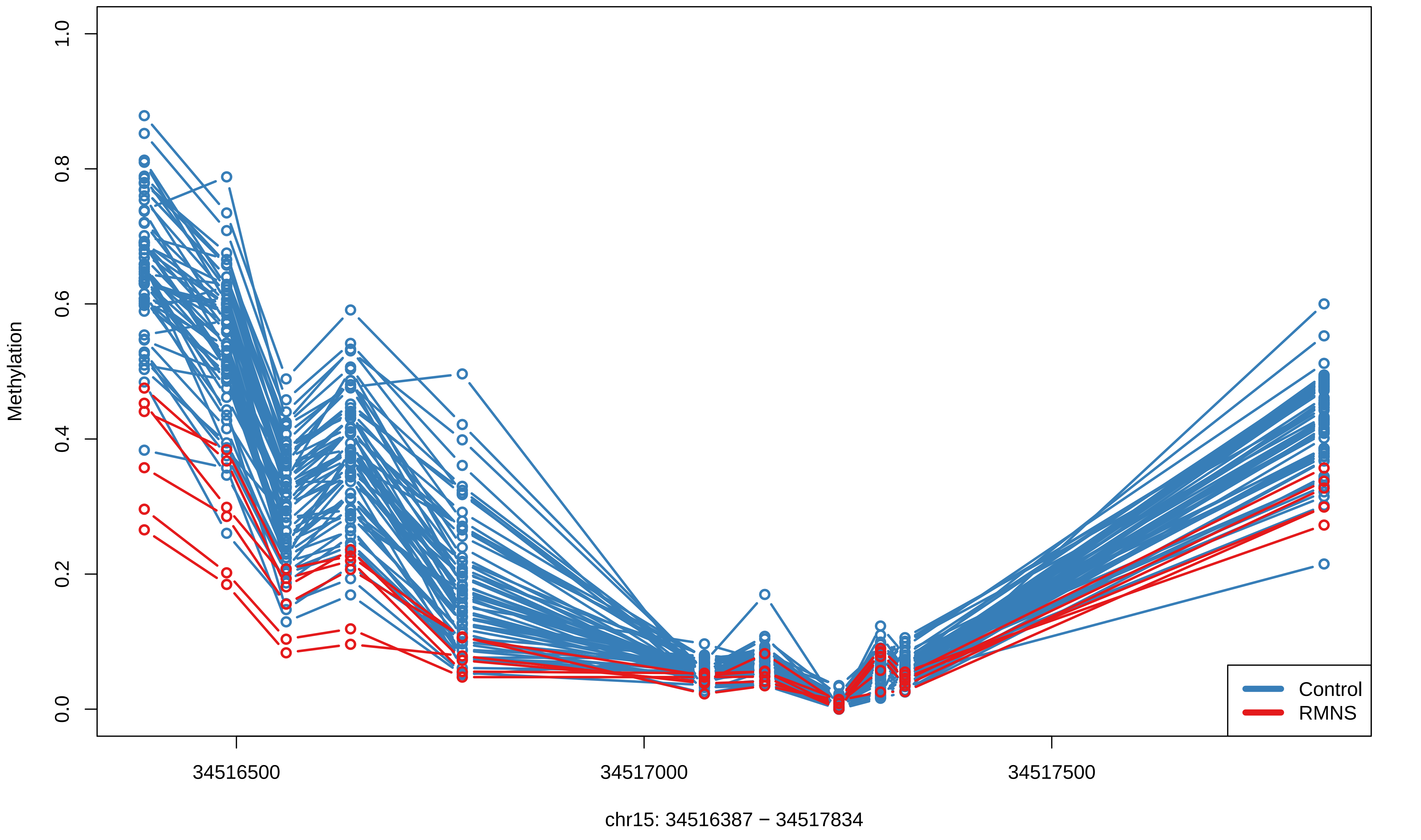

Methylation
0.0
0.2
0.4
0.6
0.8
1.0
34516500
34517000
34517500
chr15: 34516387 − 34517834
Control
RMNS

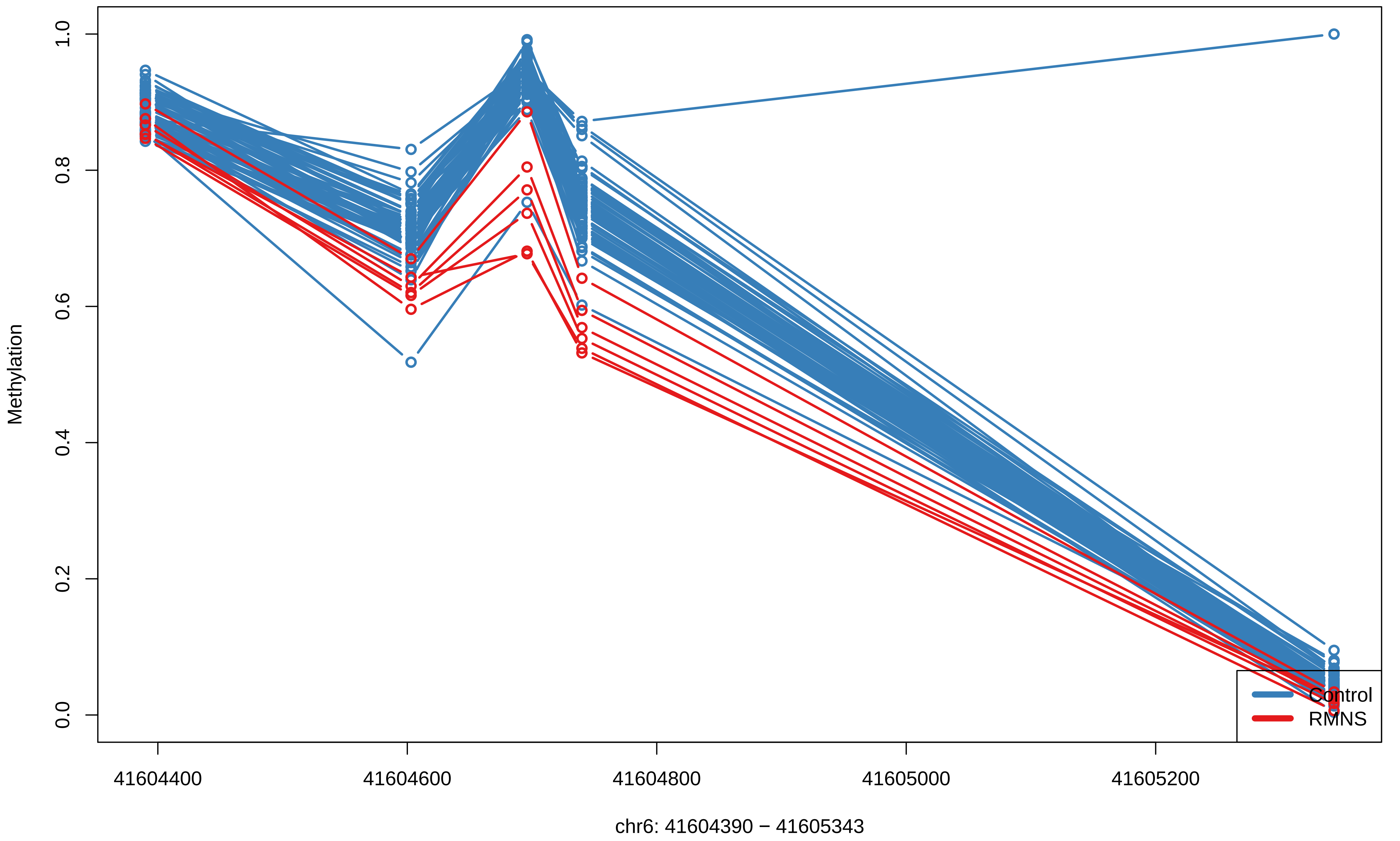

Methylation
0.0
0.2
0.4
0.6
0.8
1.0
41604400
41604600
41604800
41605000
41605200
chr6: 41604390 − 41605343
Control
RMNS

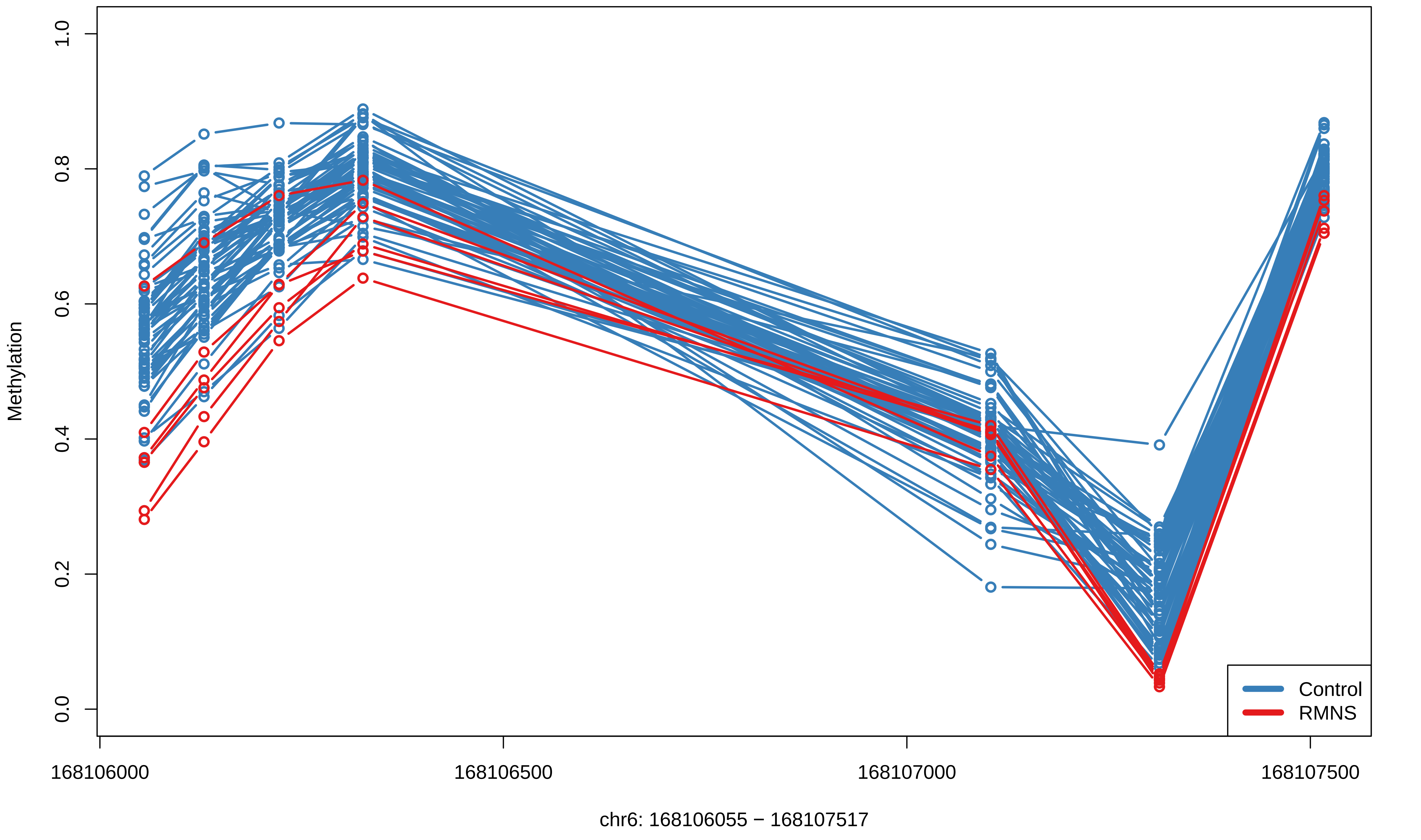

Methylation
0.0
0.2
0.4
0.6
0.8
1.0
168106000
168106500
168107000
168107500
chr6: 168106055 − 168107517
Control
RMNS

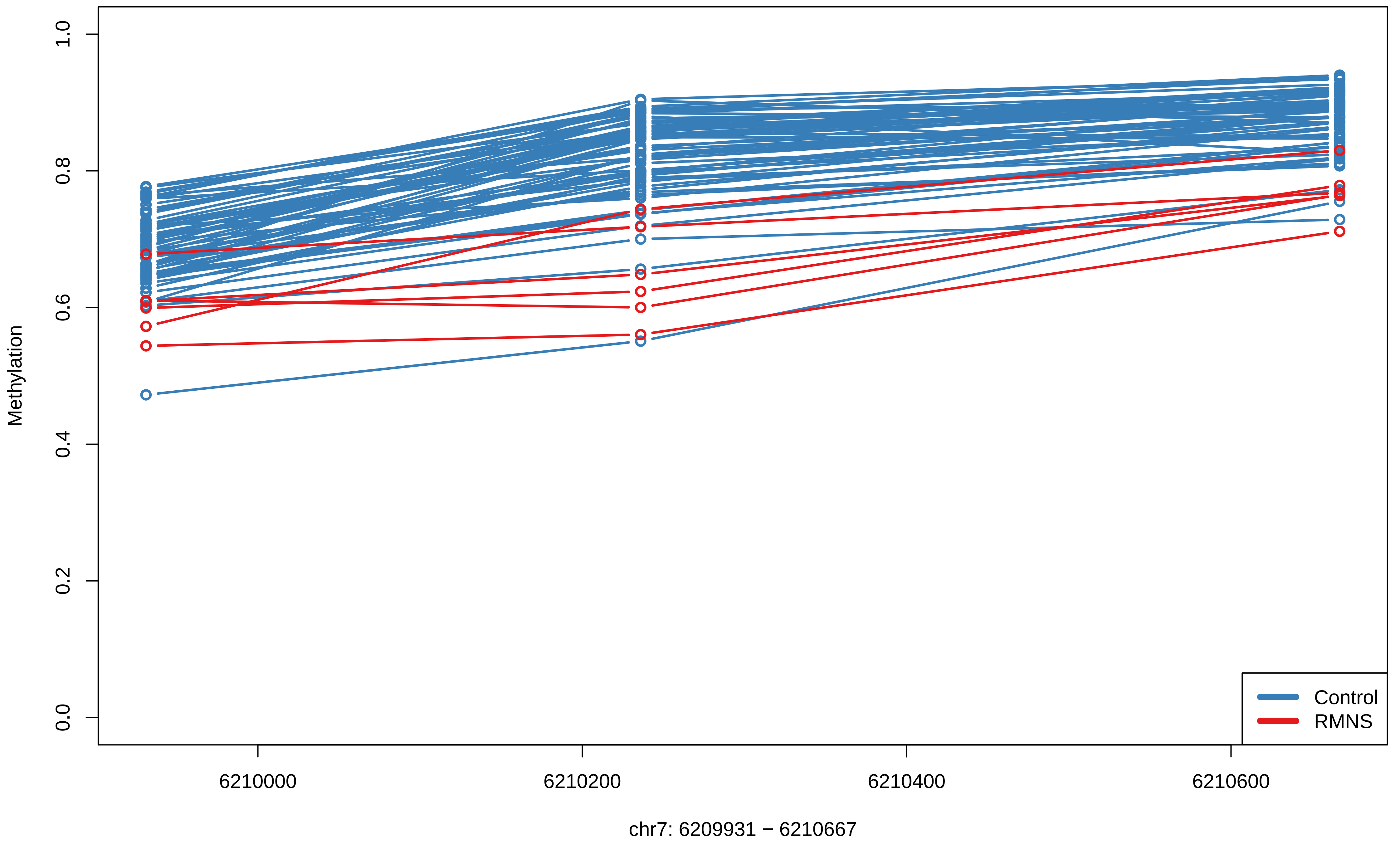

Methylation
1.0
0.8
0.6
0.4
0.2
0.0
6210000
6210200
6210400
6210600
chr7: 6209931 − 6210667
Control
RMNS

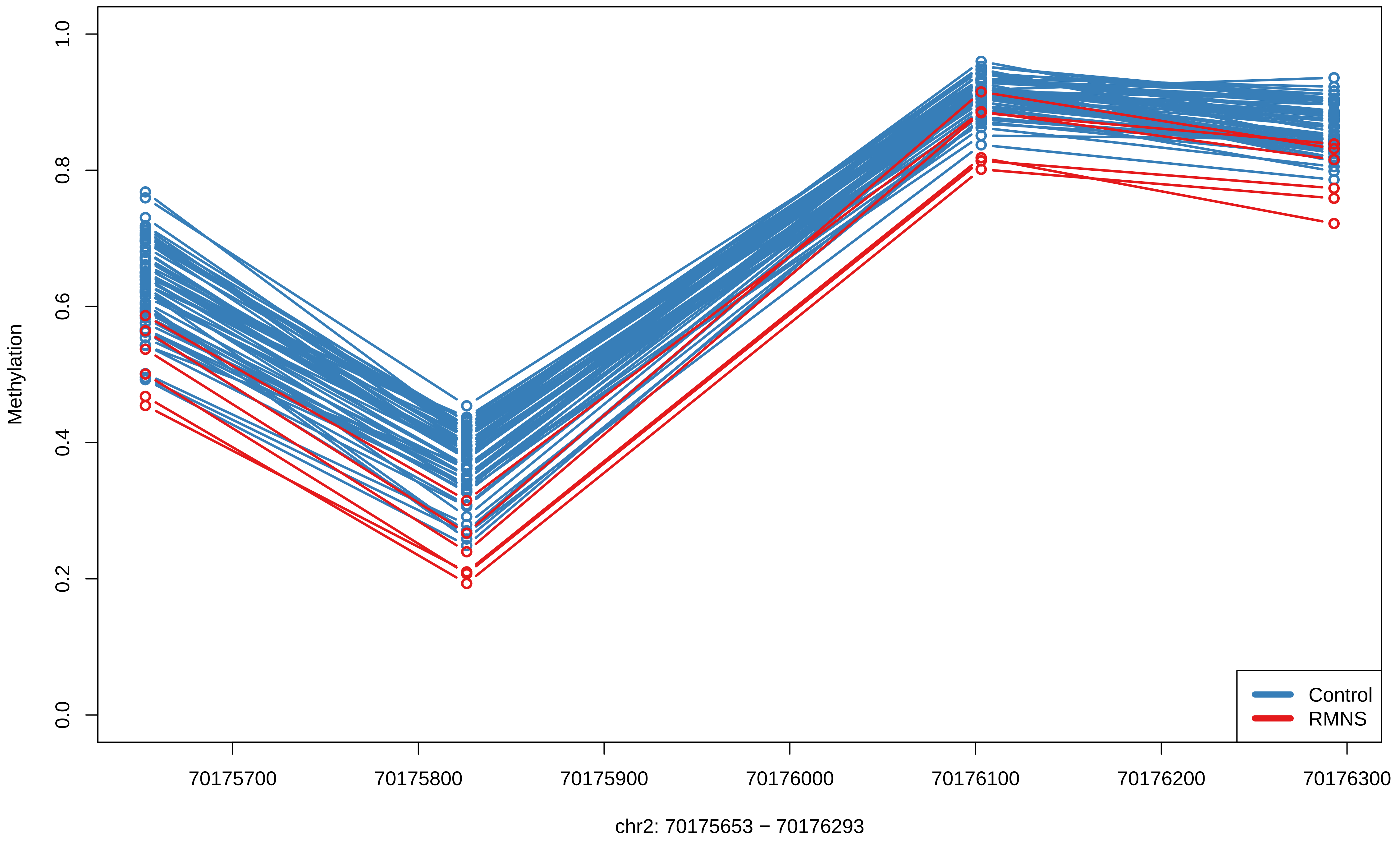

Methylation
0.0
0.2
0.4
0.6
0.8
1.0
70175700
70175800
70175900
70176000
70176100
70176200
70176300
chr2: 70175653 − 70176293
Control
RMNS

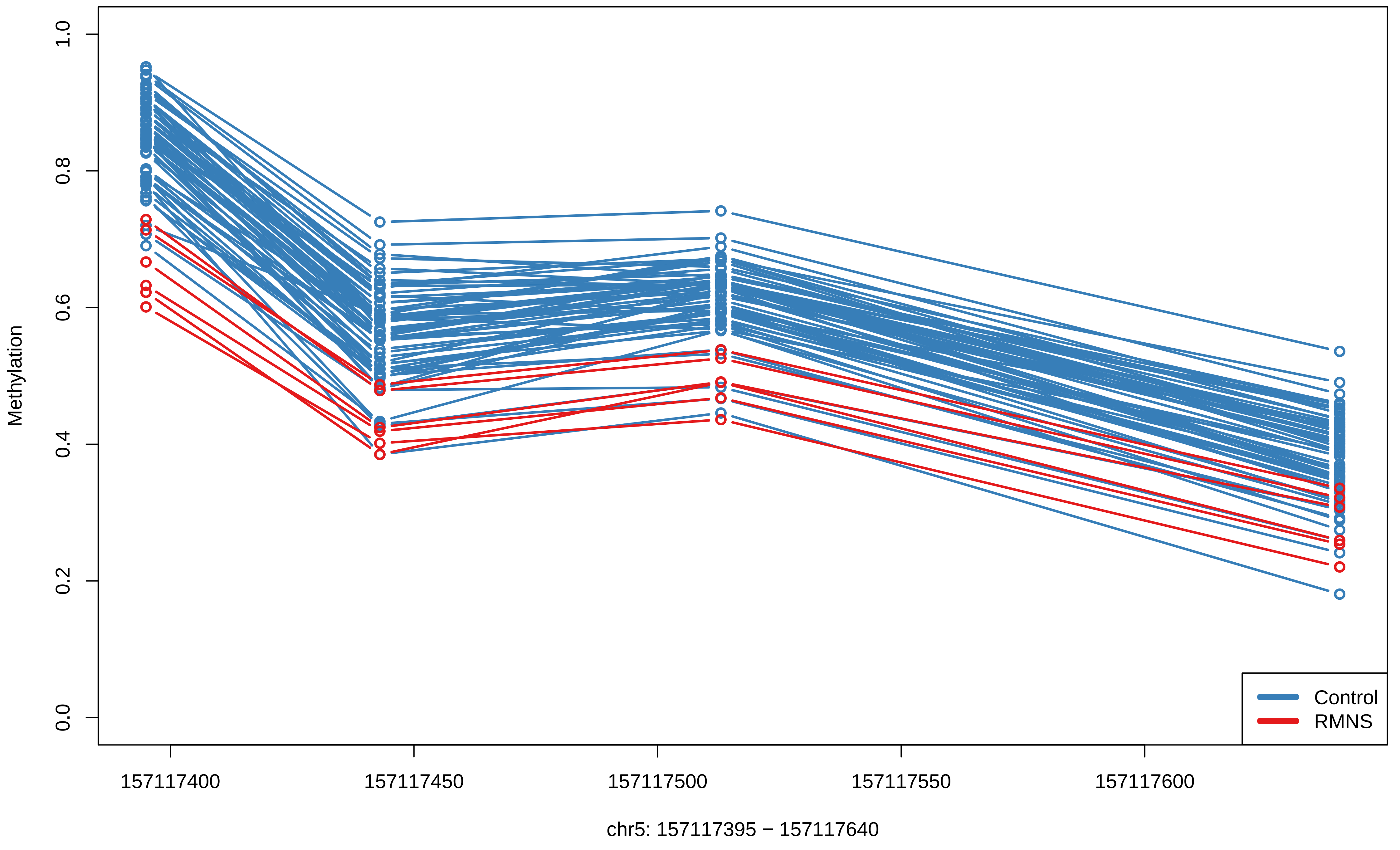

Methylation
0.0
0.2
0.4
0.6
0.8
1.0
157117400
157117450
157117500
157117550
157117600
chr5: 157117395 − 157117640
Control
RMNS

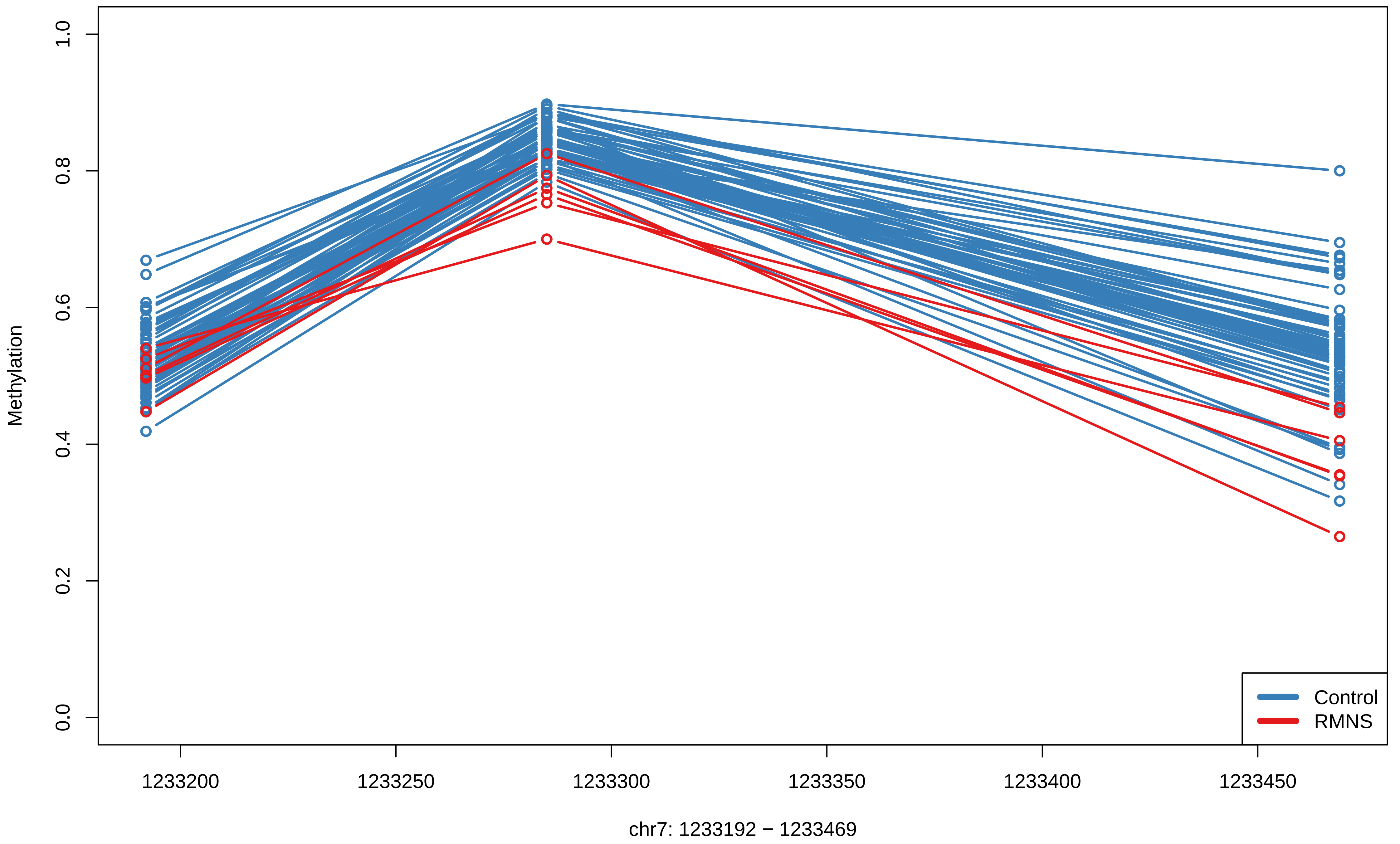

Methylation
1.0
0.8
0.6
0.4
0.2
0.0
1233200
1233250
1233300
1233350
1233400
1233450
chr7: 1233192 − 1233469
Control
RMNS

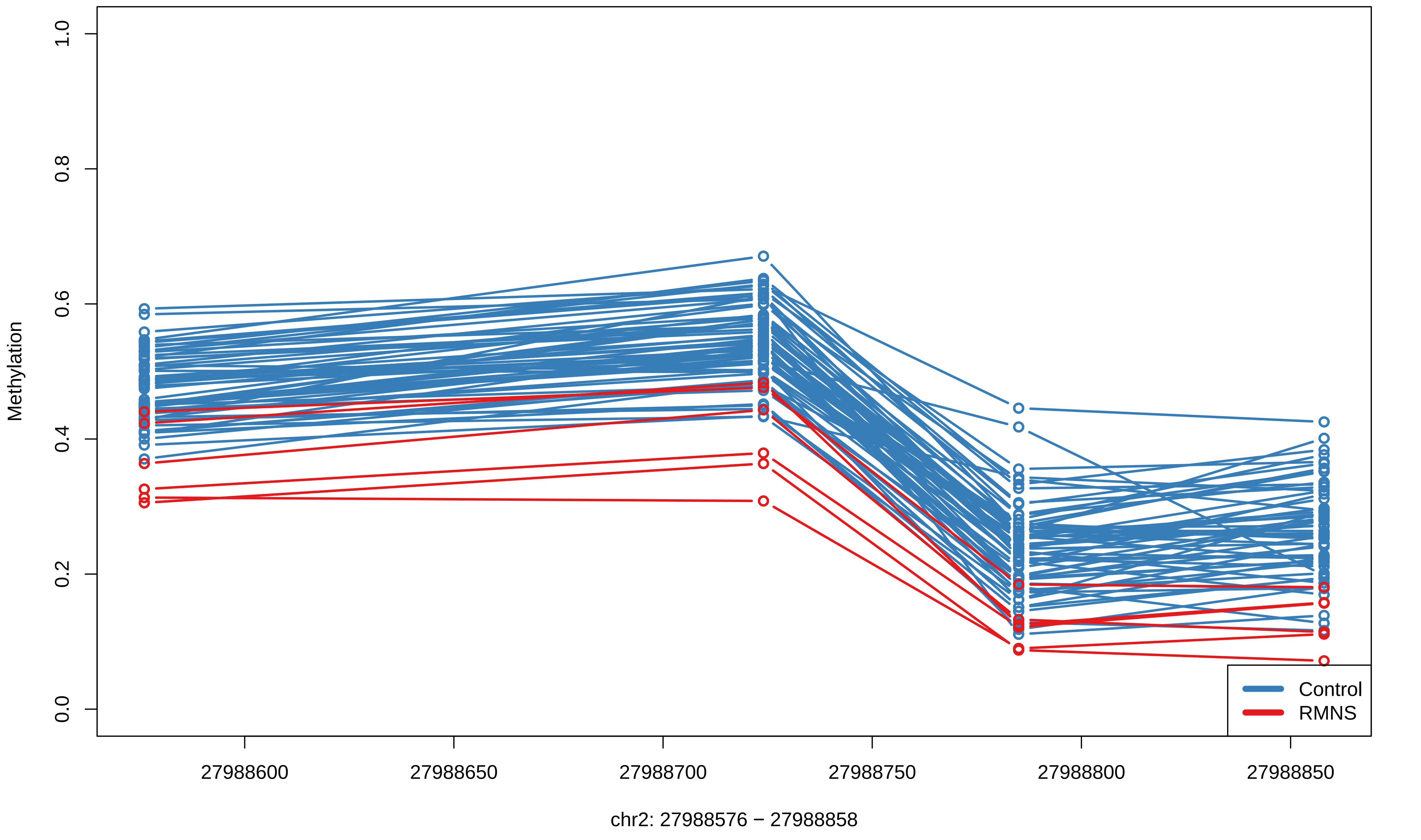

Methylation
0.0
0.2
0.4
0.6
0.8
1.0
27988600
27988650
27988700
27988750
27988800
27988850
chr2: 27988576 − 27988858
Control
RMNS

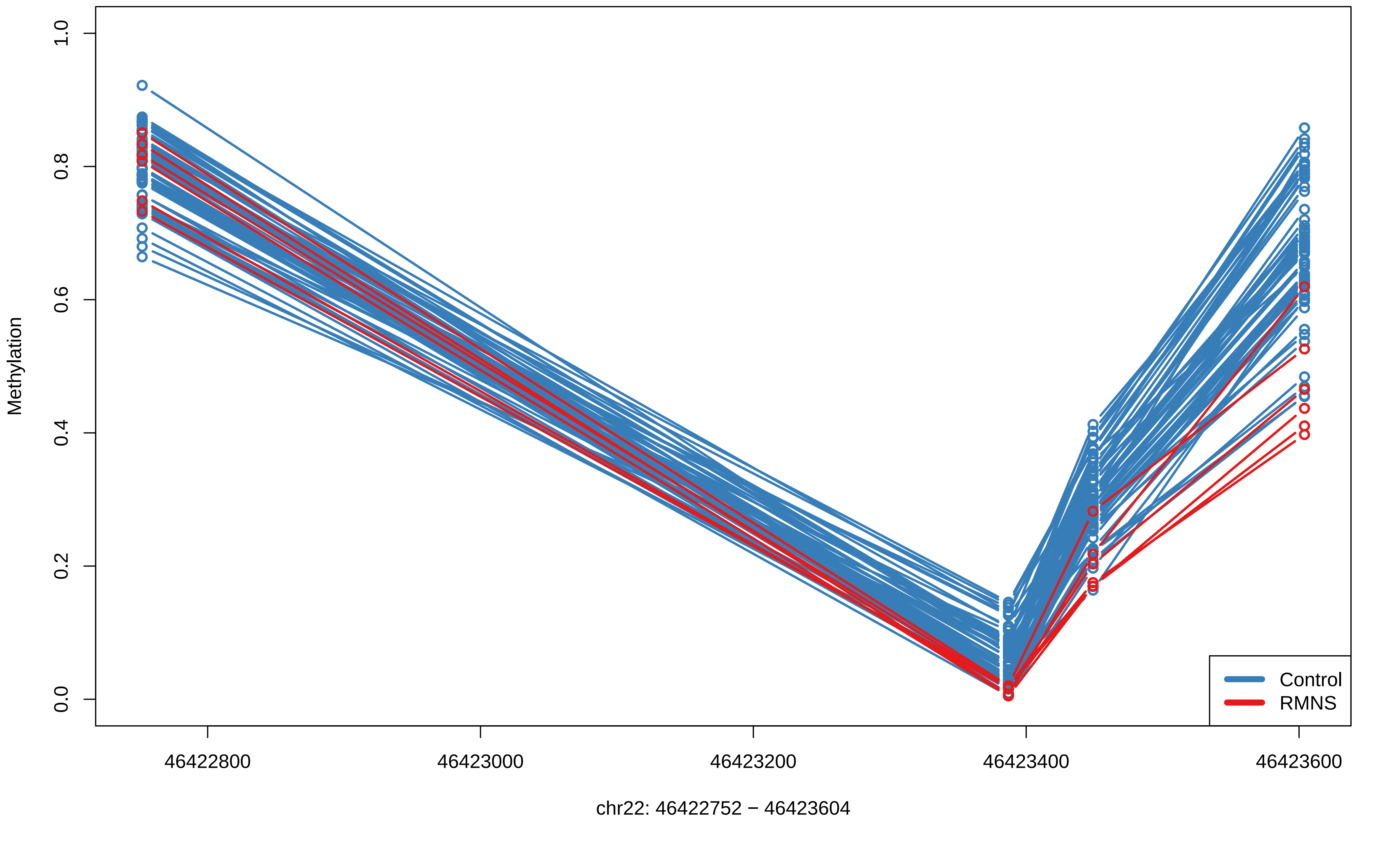

Methylation
0.0
0.2
0.4
0.6
0.8
1.0
46422800
46423000
46423200
46423400
46423600
chr22: 46422752 − 46423604
Control
RMNS

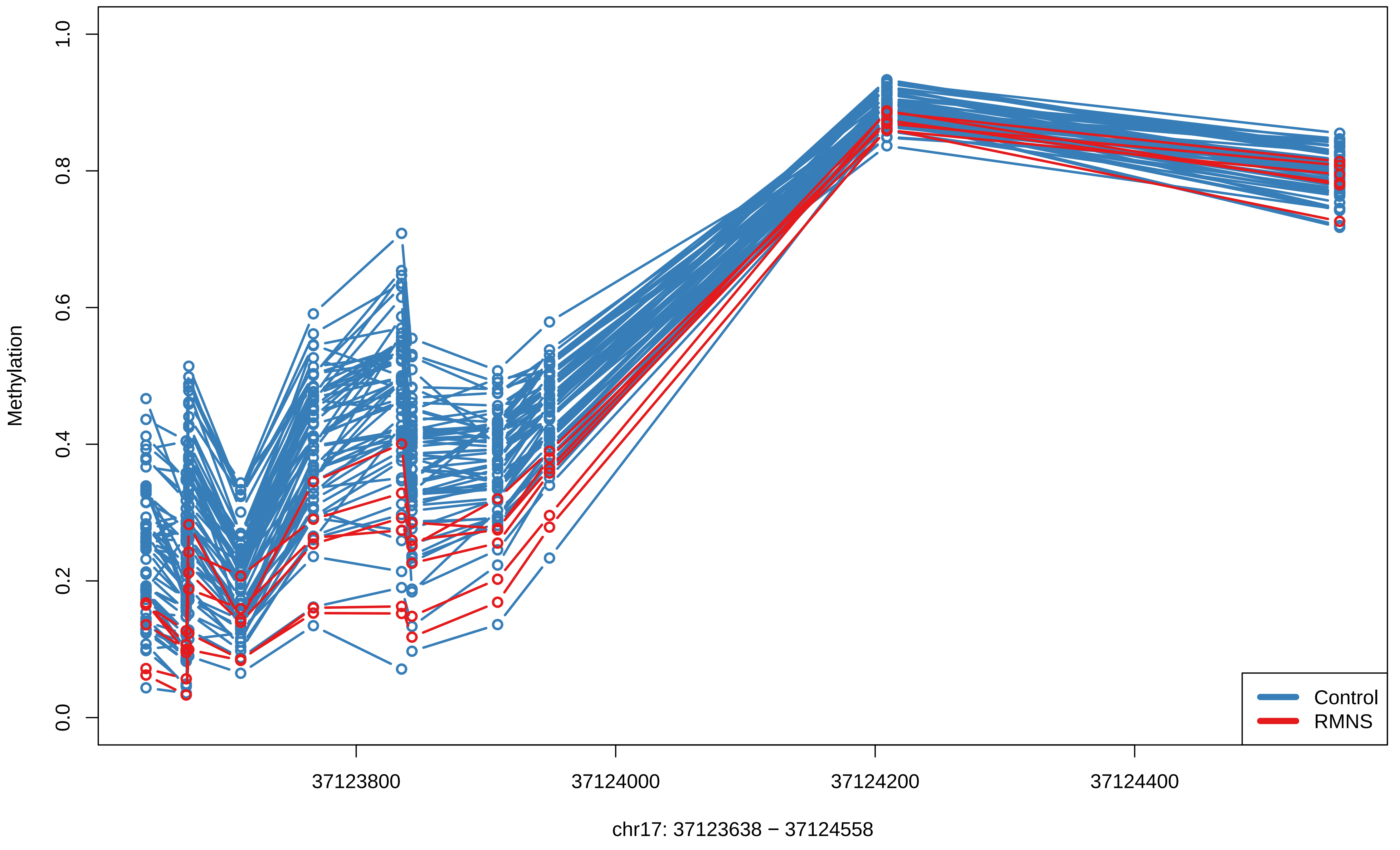
Methylation
0.0
0.2
0.4
0.6
0.8
1.0
37123800
37124000
37124200
37124400
chr17: 37123638 − 37124558
Control
RMNS

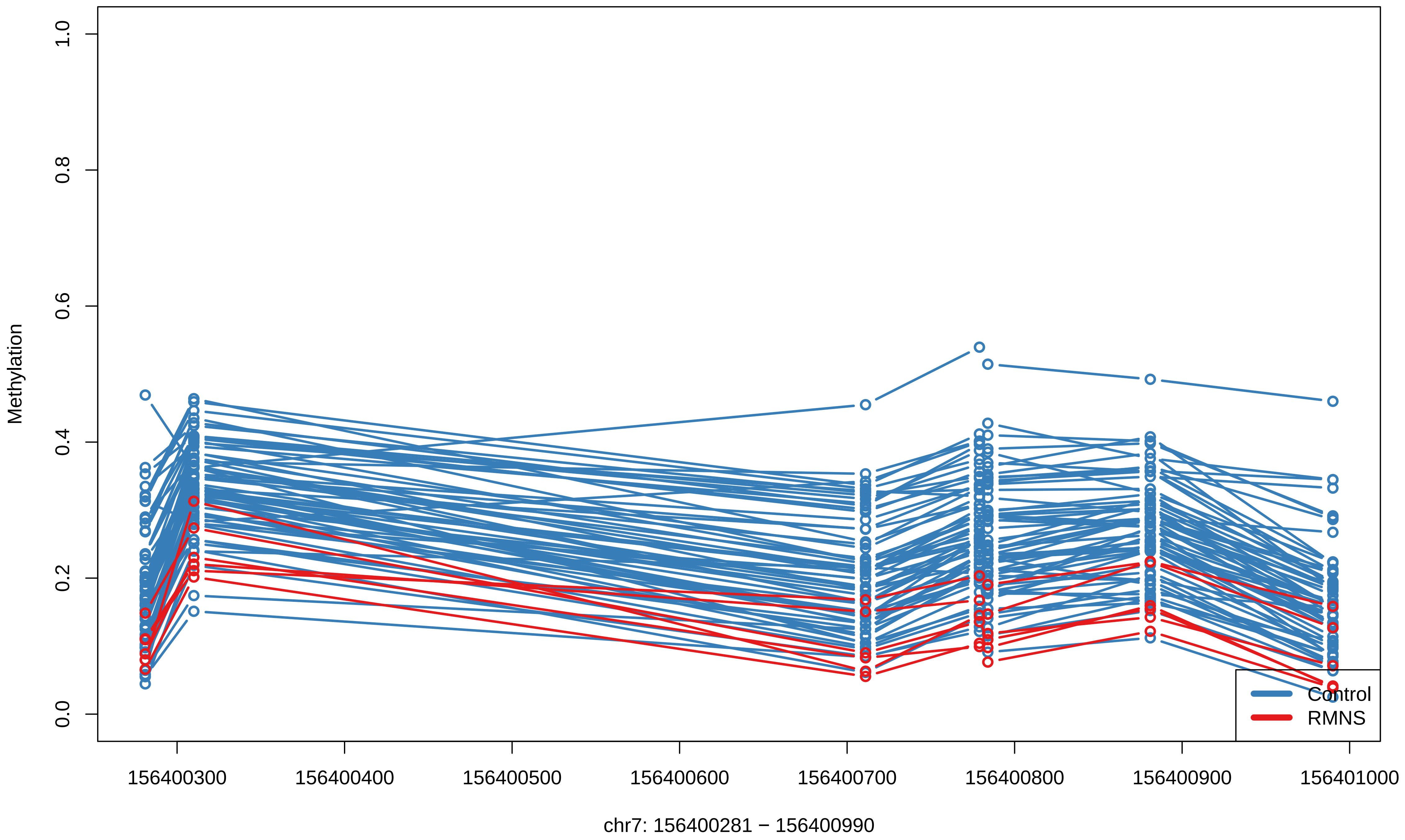

Methylation
1.0
0.8
0.6
0.4
0.2
0.0
156400300
156400400
156400500
156400600
156400700
156400800
156400900
156401000
chr7: 156400281 − 156400990
Control
RMNS

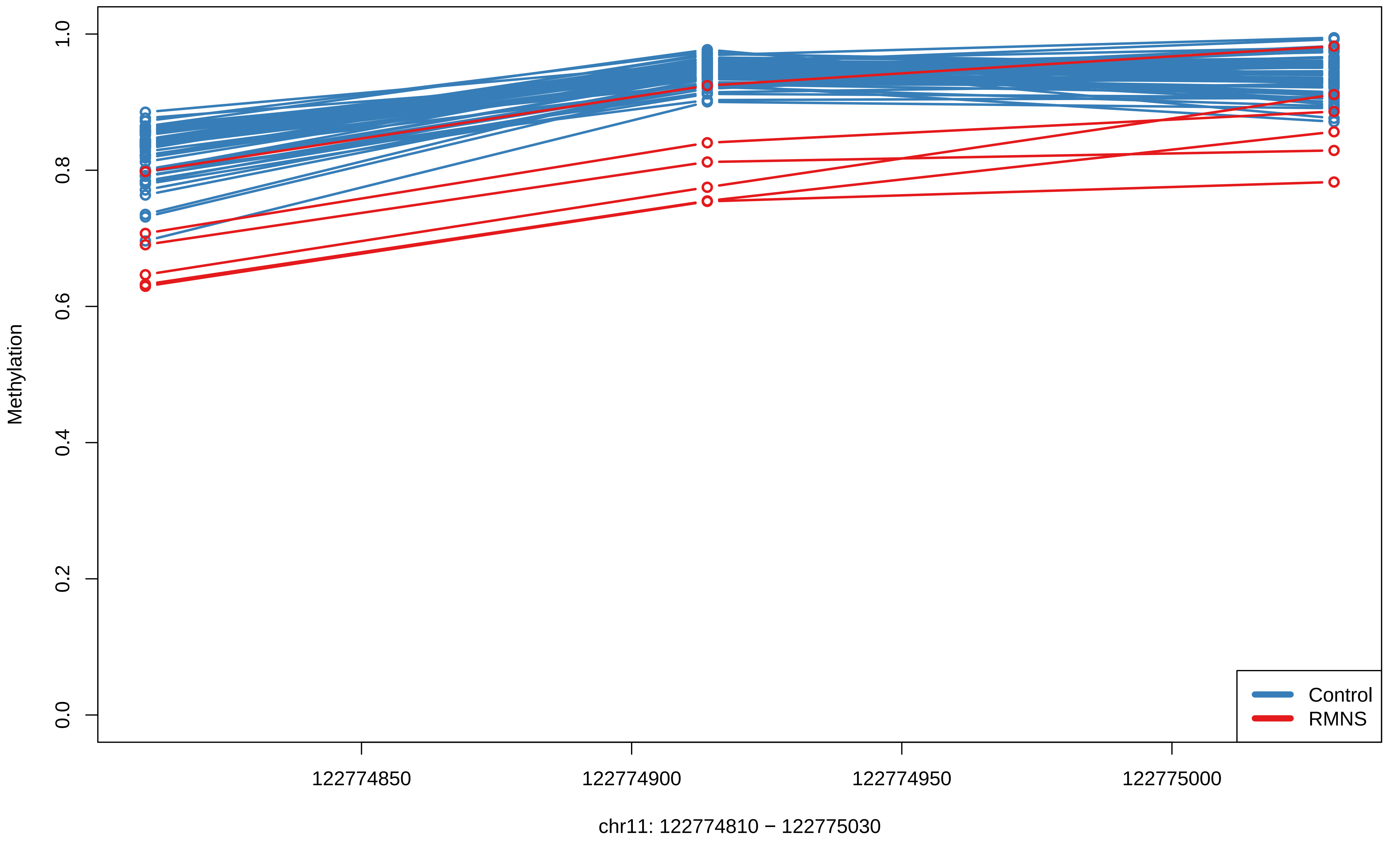

Methylation
0.0
0.2
0.4
0.6
0.8
1.0
122774850
122774900
122774950
122775000
chr11: 122774810 − 122775030
Control
RMNS

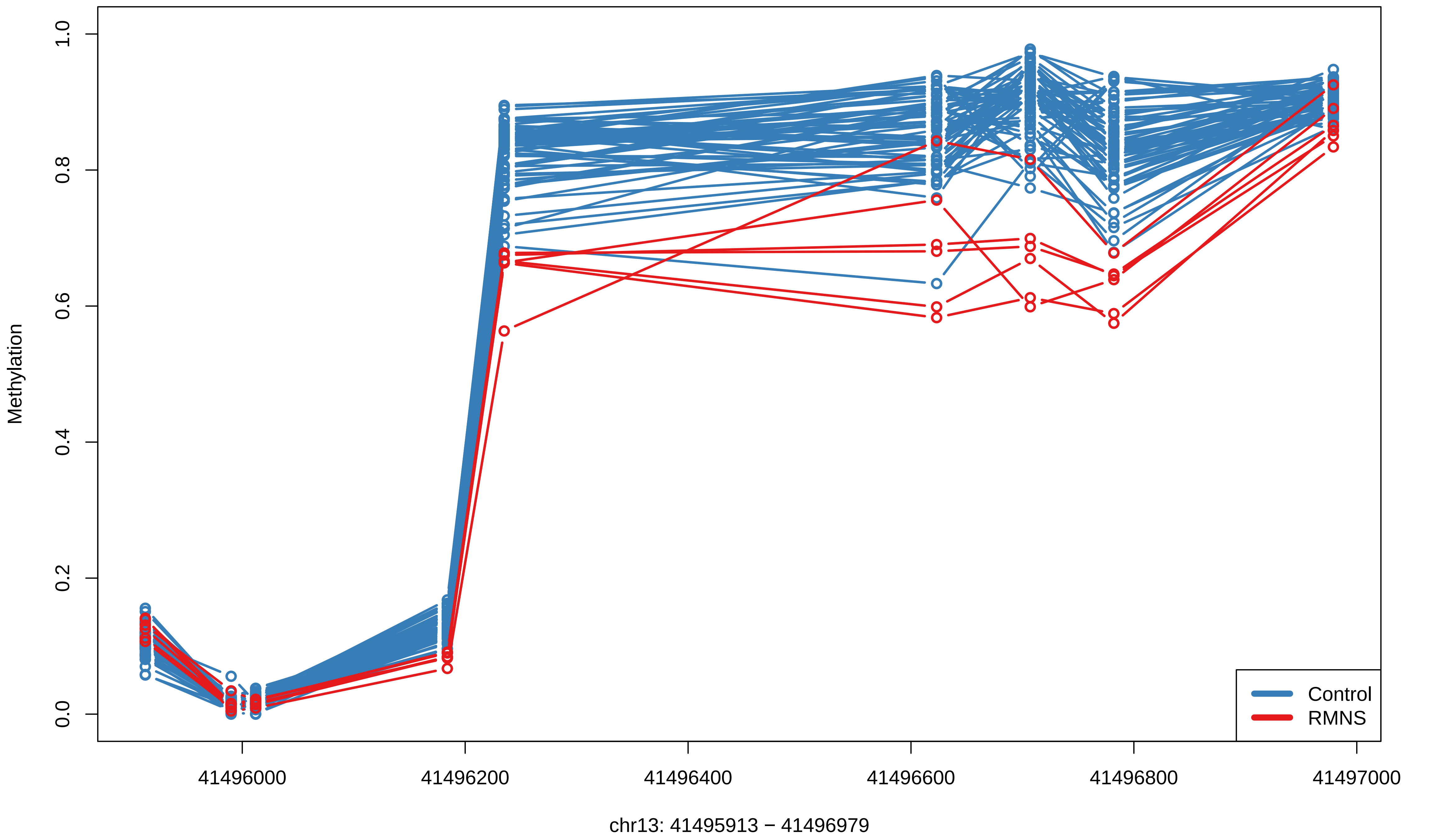

Methylation
1.0
0.8
0.6
0.4
0.2
0.0
41496000
41496200
41496400
41496600
41496800
41497000
chr13: 41495913 − 41496979
Control
RMNS

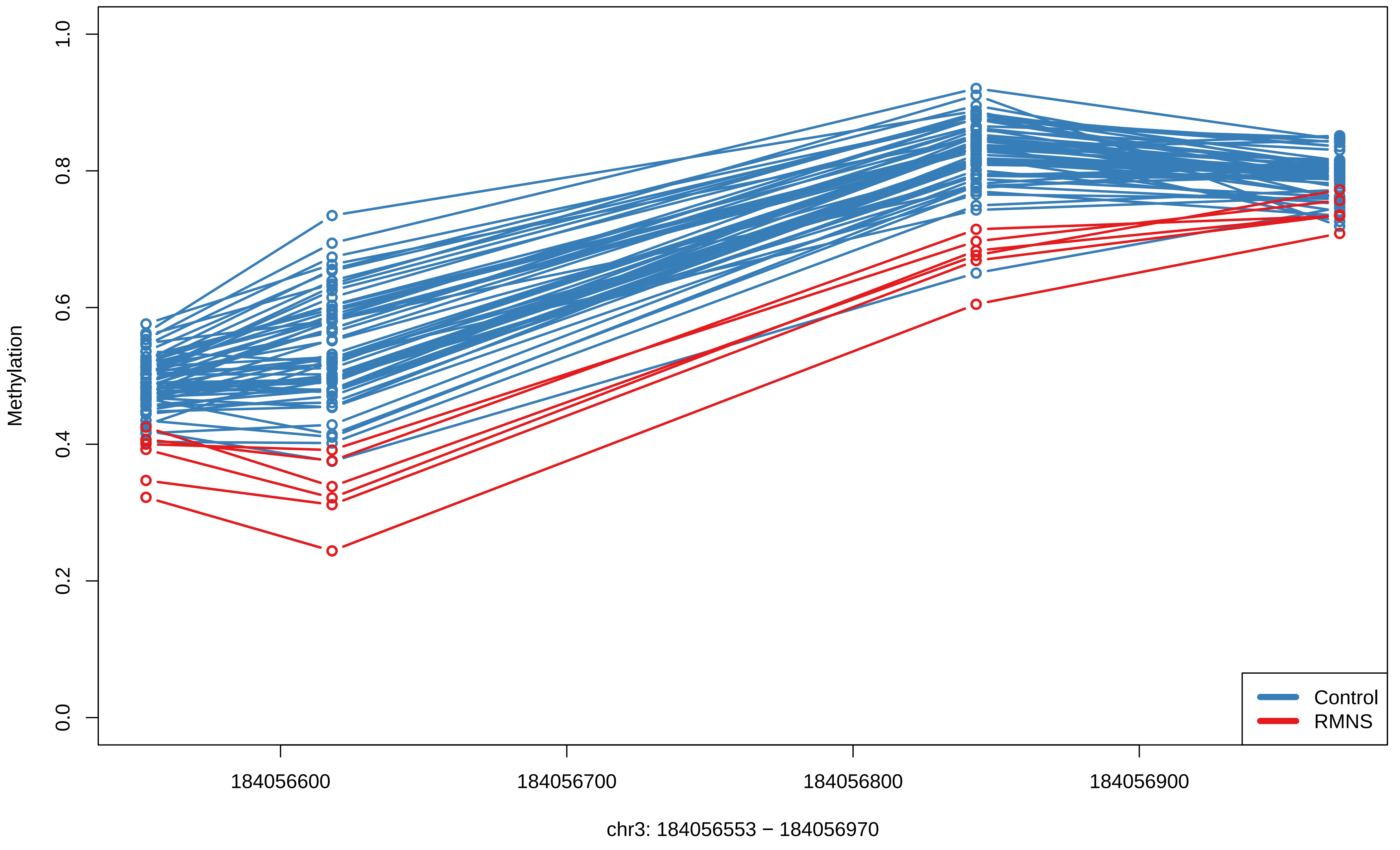

Methylation
1.0
0.8
0.6
0.4
0.2
0.0
184056600
184056700
184056800
184056900
chr3: 184056553 − 184056970
Control
RMNS

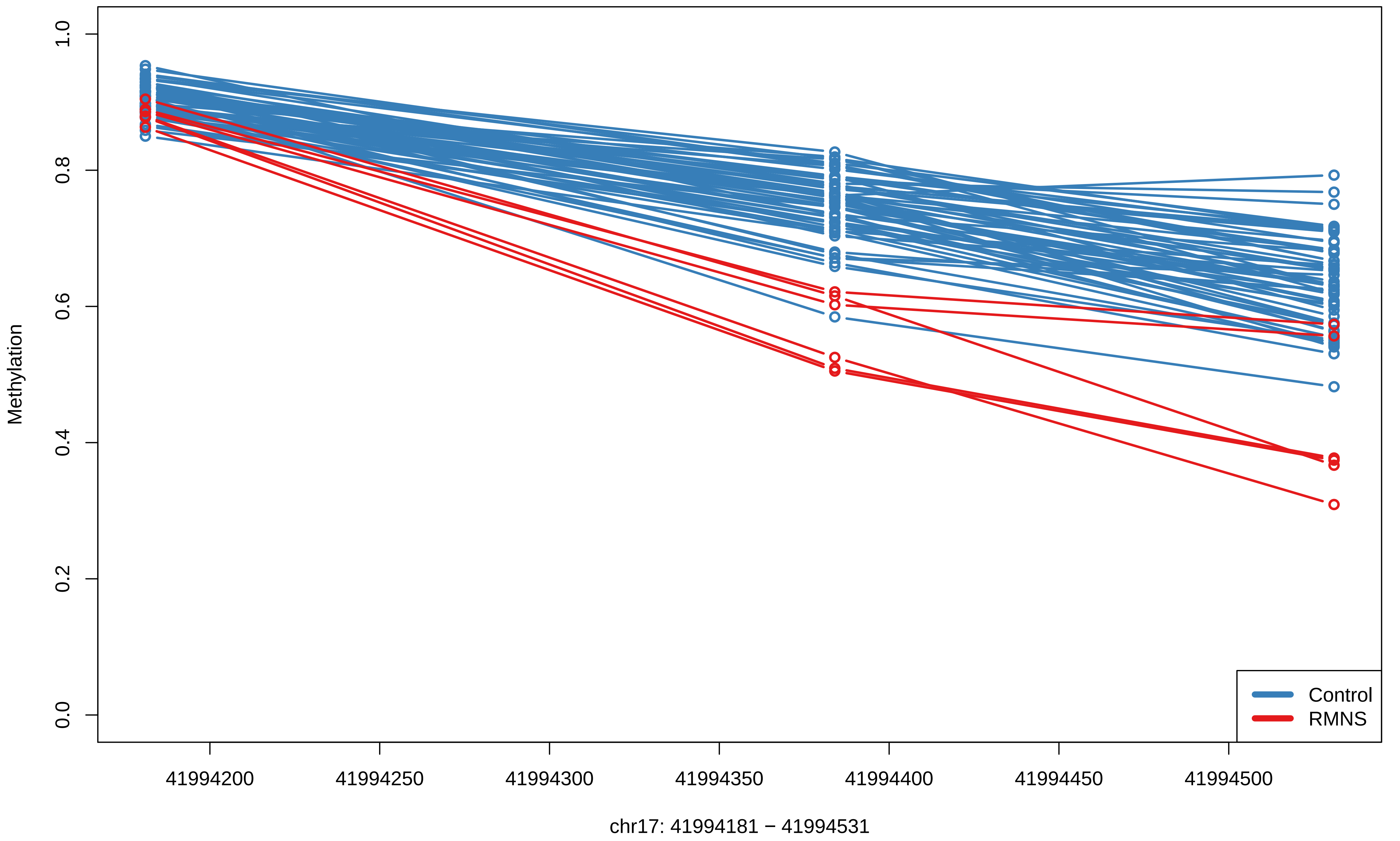
Methylation
0.0
0.2
0.4
0.6
0.8
1.0
41994200
41994250
41994300
41994350
41994400
41994450
41994500
chr17: 41994181 − 41994531
Control
RMNS

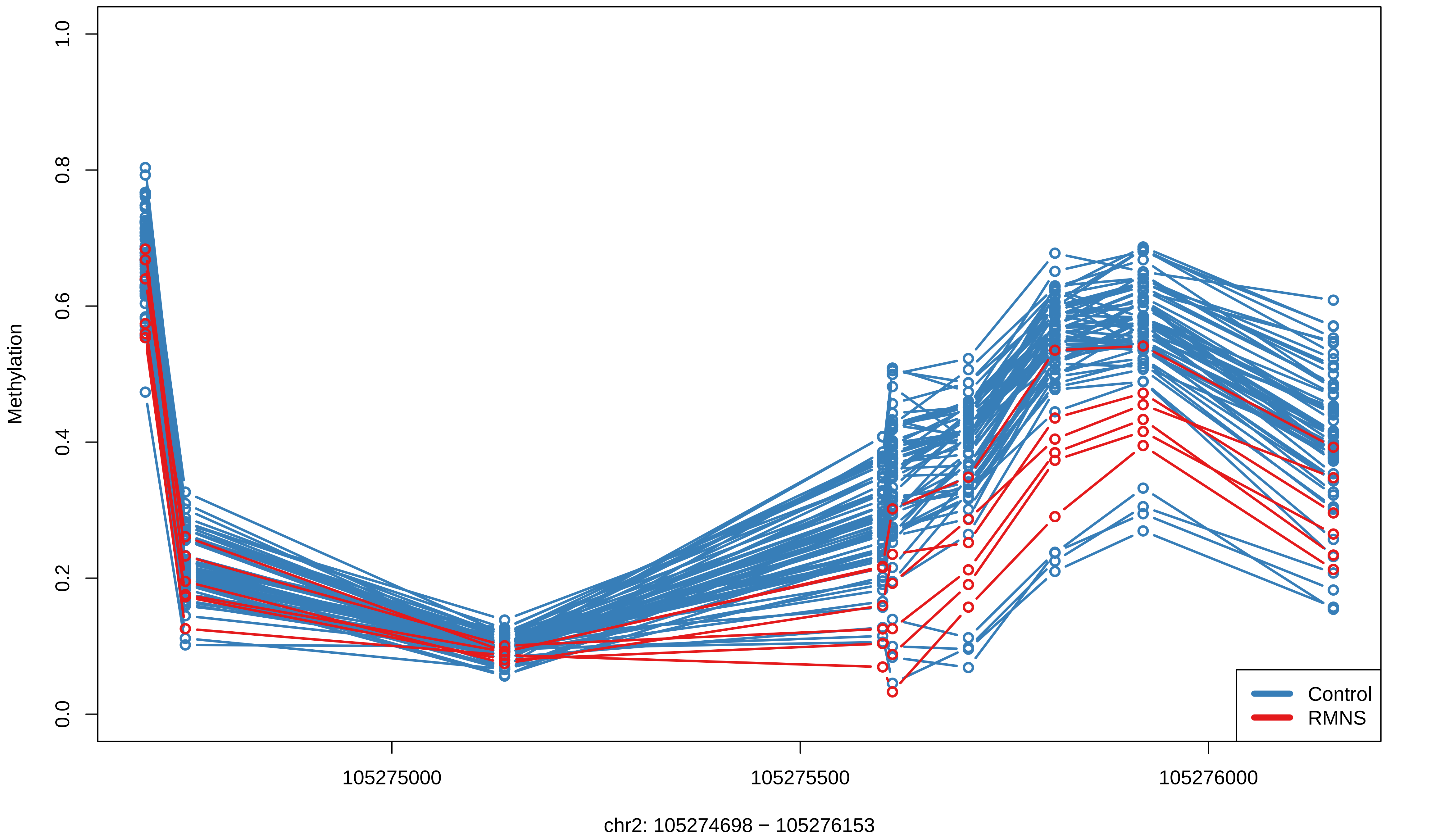

0.0
0.2
0.4
0.6
0.8
1.0
Methylation
105275000
105275500
105276000
chr2: 105274698 − 105276153
Control
RMNS

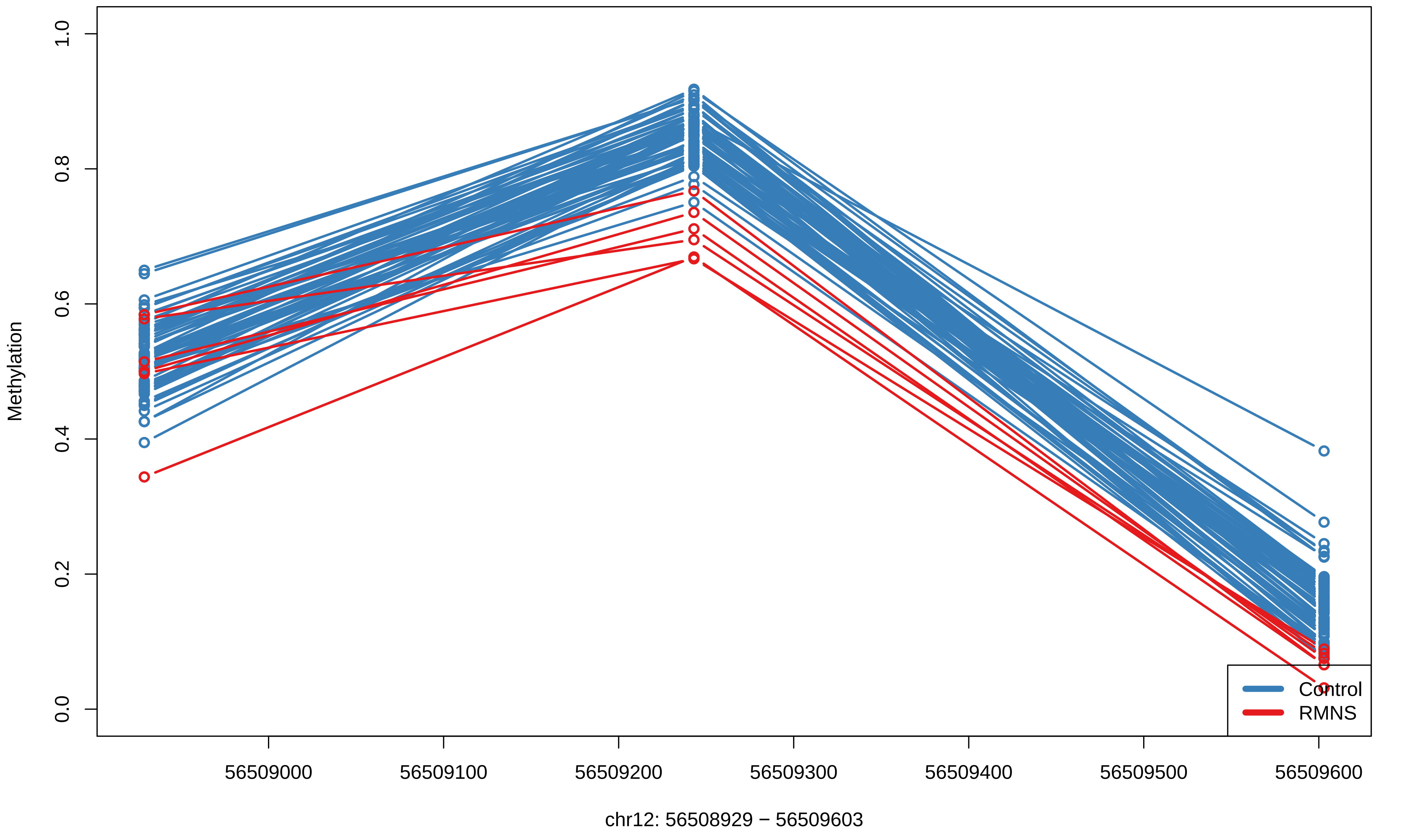

Methylation
0.0
0.2
0.4
0.6
0.8
1.0
56509000
56509100
56509200
56509300
56509400
56509500
56509600
chr12: 56508929 − 56509603
Control
RMNS

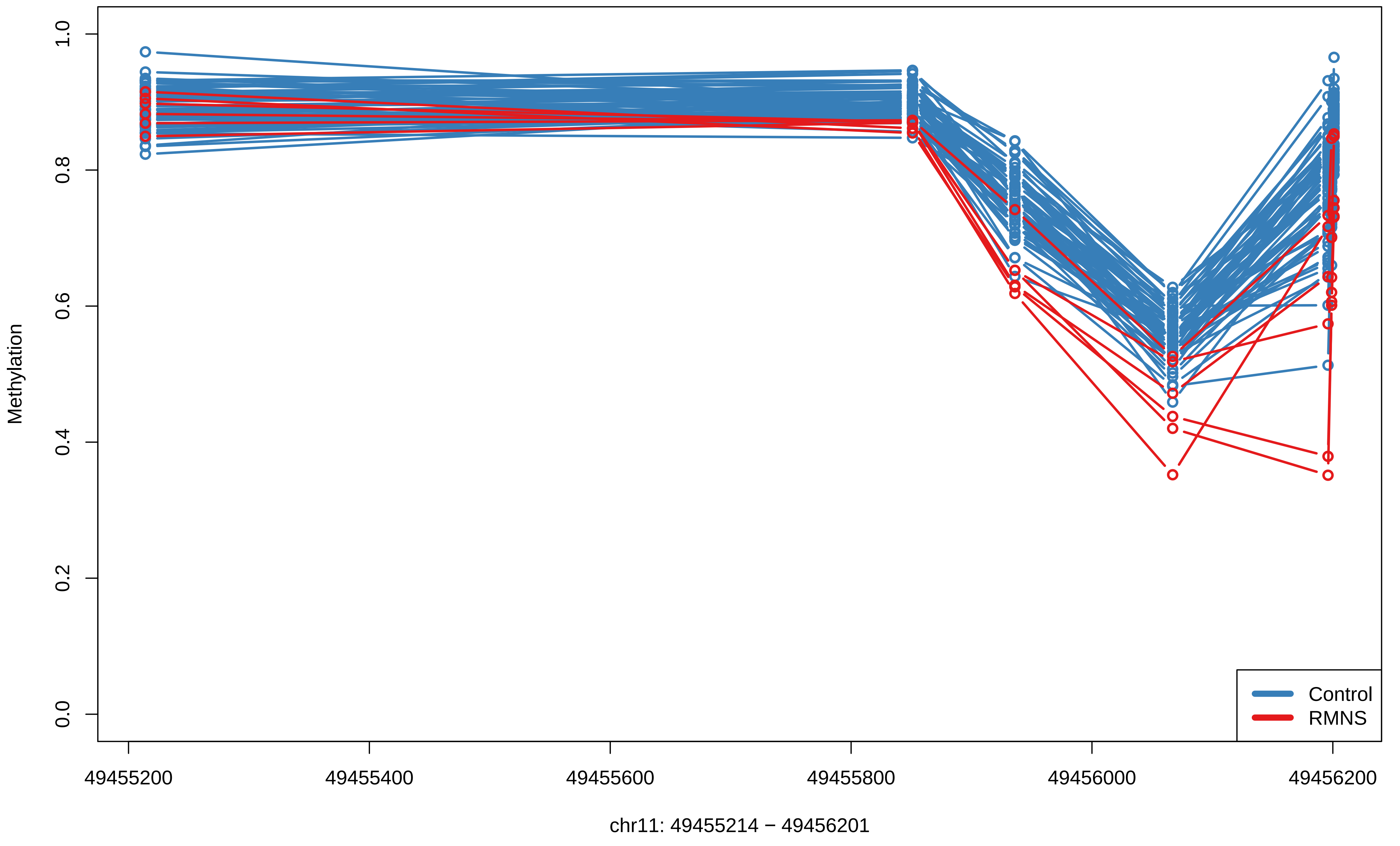

1.0
0.8
0.6
0.4
0.2
0.0
Methylation
Control
RMNS
49455200
49455400
49455600
49455800
49456000
49456200
chr11: 49455214 − 49456201

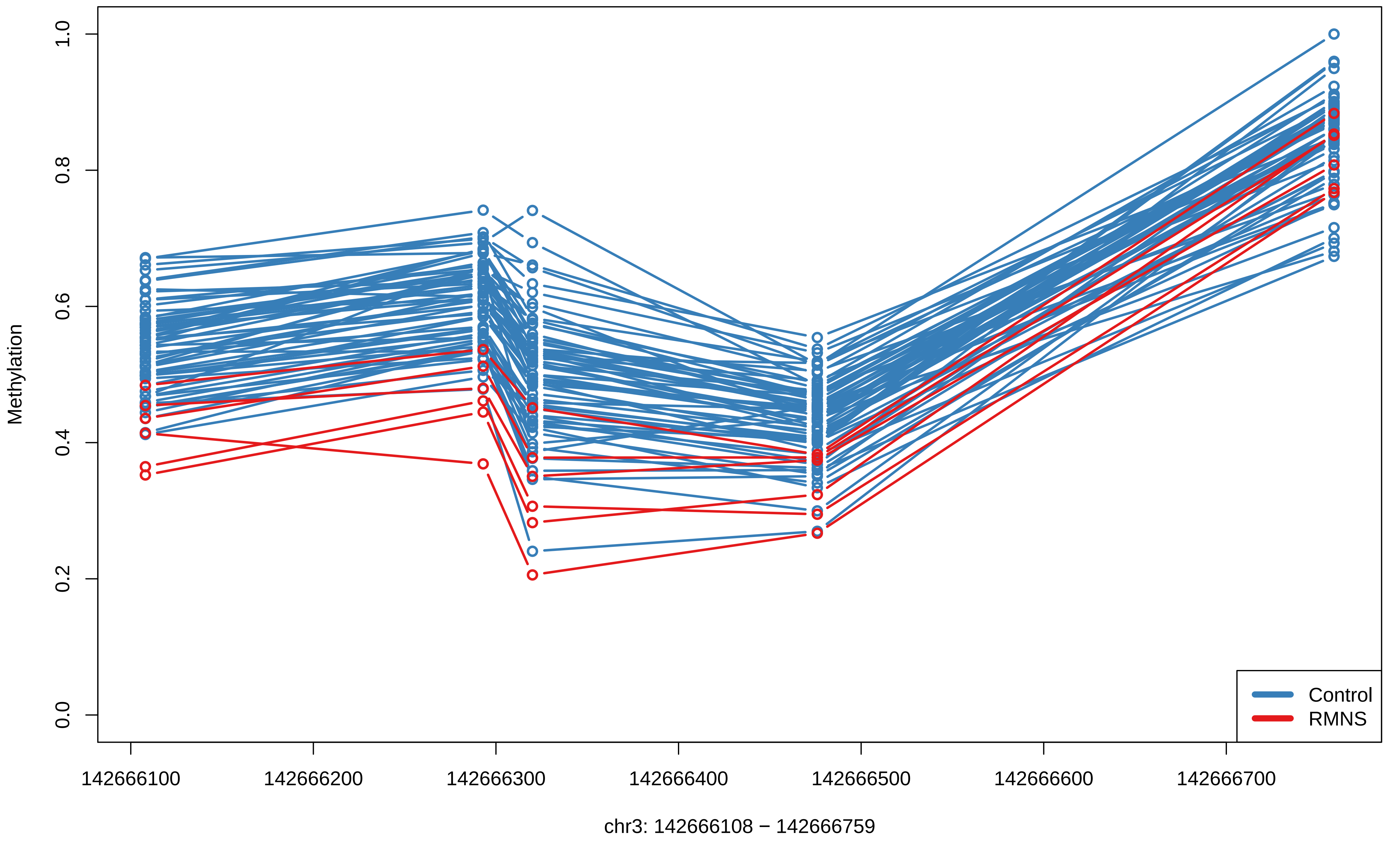

Methylation
1.0
0.8
0.6
0.4
0.2
0.0
142666100
142666200
142666300
142666400
142666500
142666600
142666700
chr3: 142666108 − 142666759
Control
RMNS

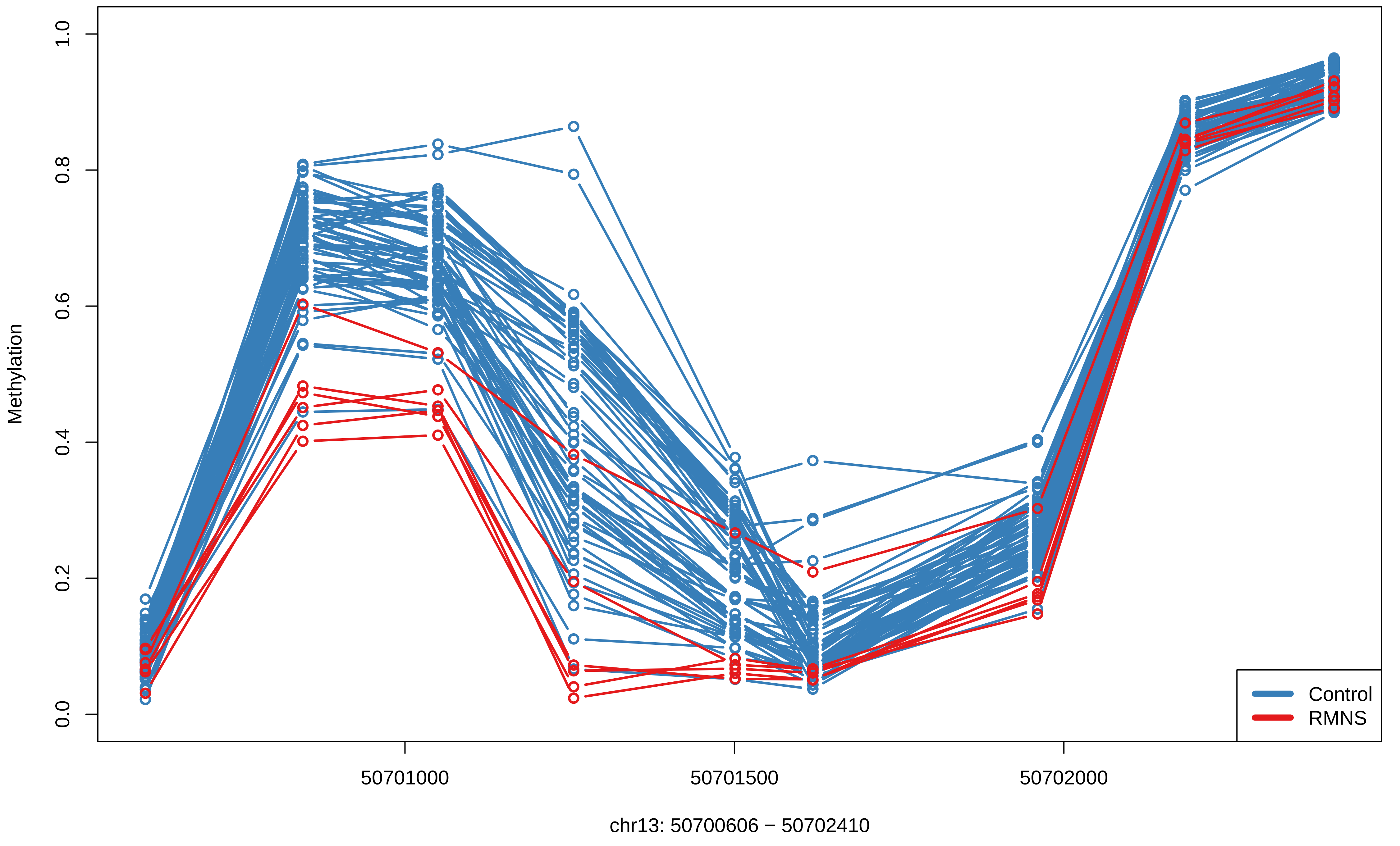

Methylation
0.0
0.2
0.4
0.6
0.8
1.0
50701000
50701500
50702000
chr13: 50700606 − 50702410
Control
RMNS

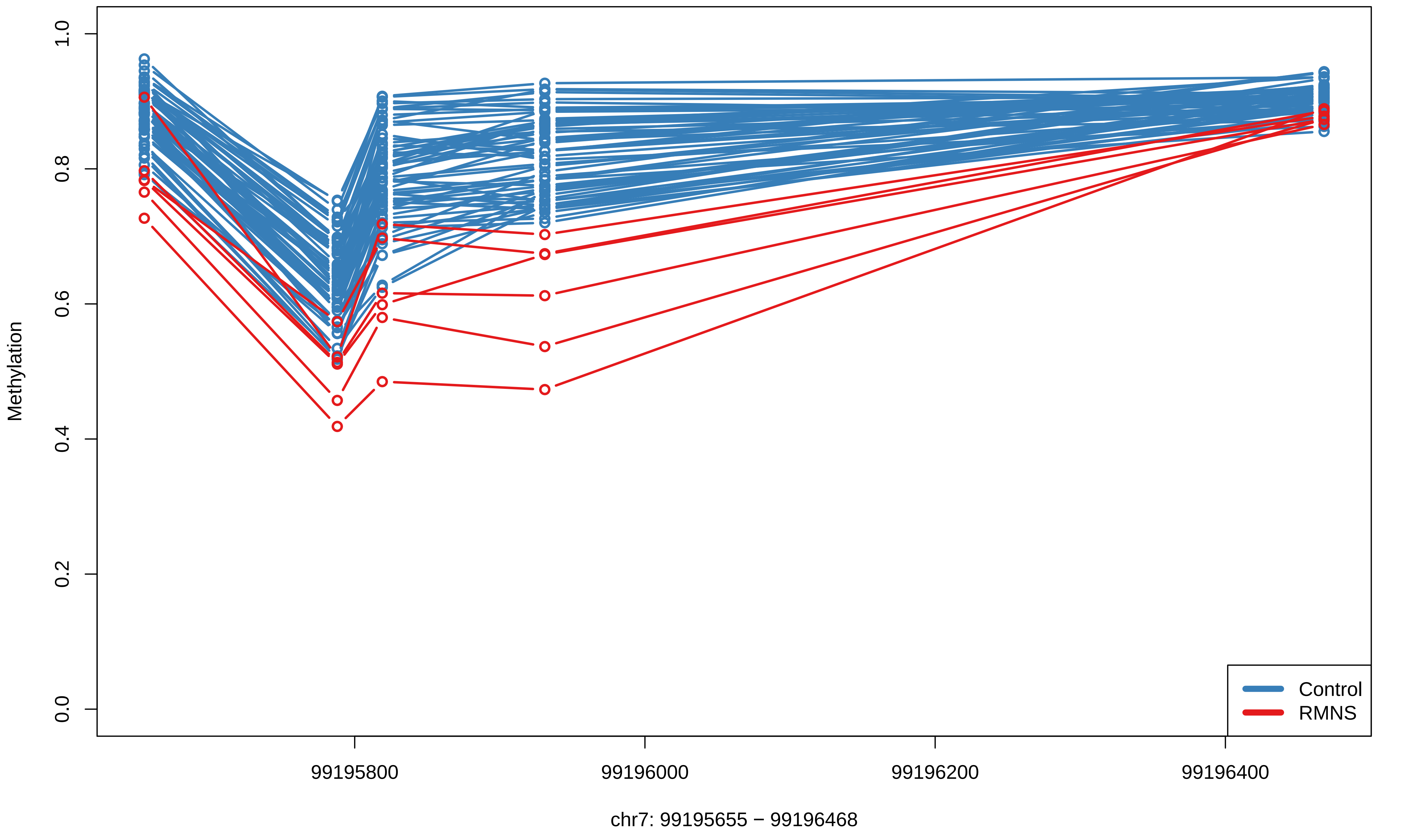

Methylation
1.0
0.8
0.6
0.4
0.2
0.0
99195800
99196000
99196200
99196400
chr7: 99195655 − 99196468
Control
RMNS

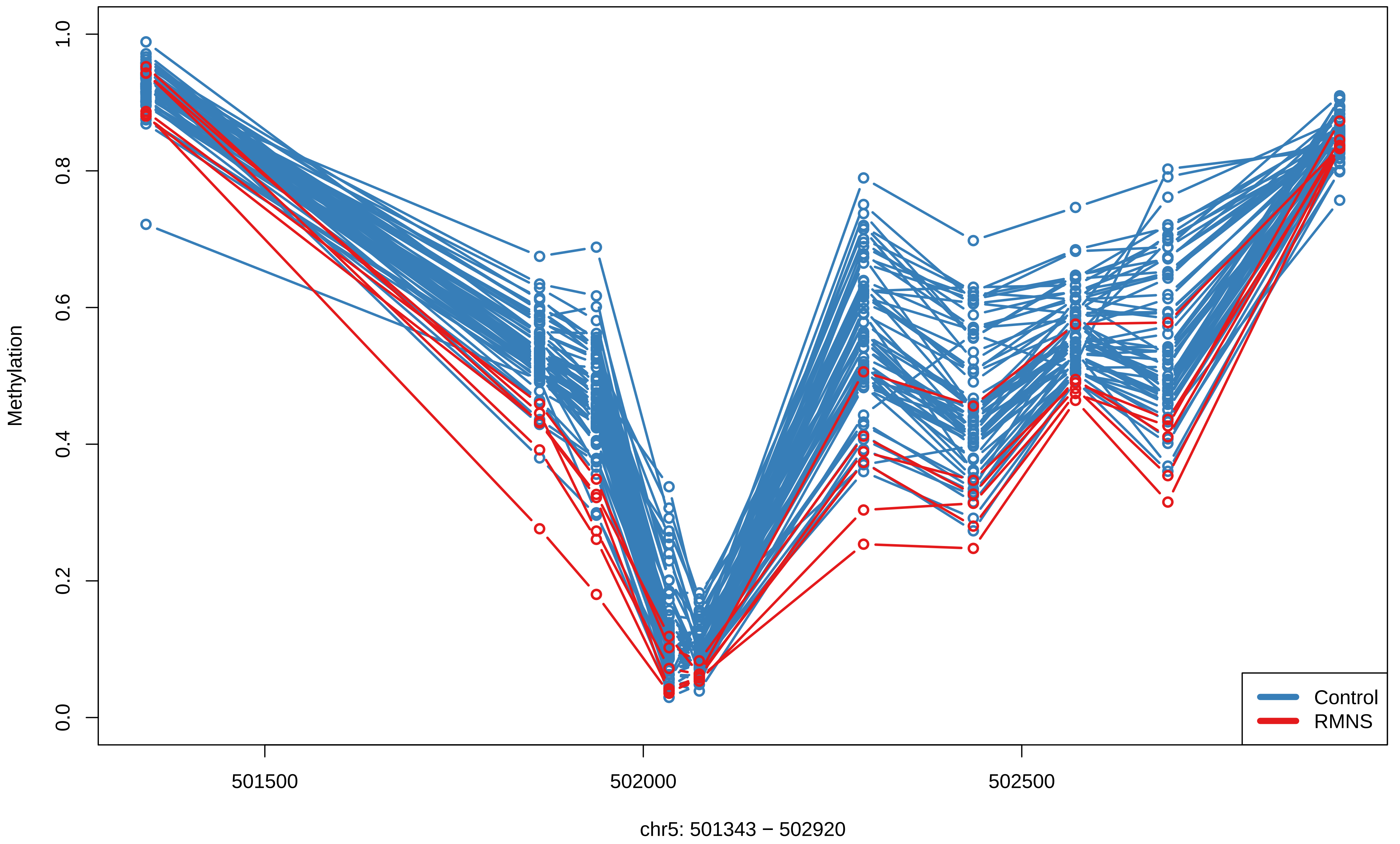

Methylation
1.0
0.8
0.6
0.4
0.2
0.0
501500
502000
502500
chr5: 501343 − 502920
Control
RMNS

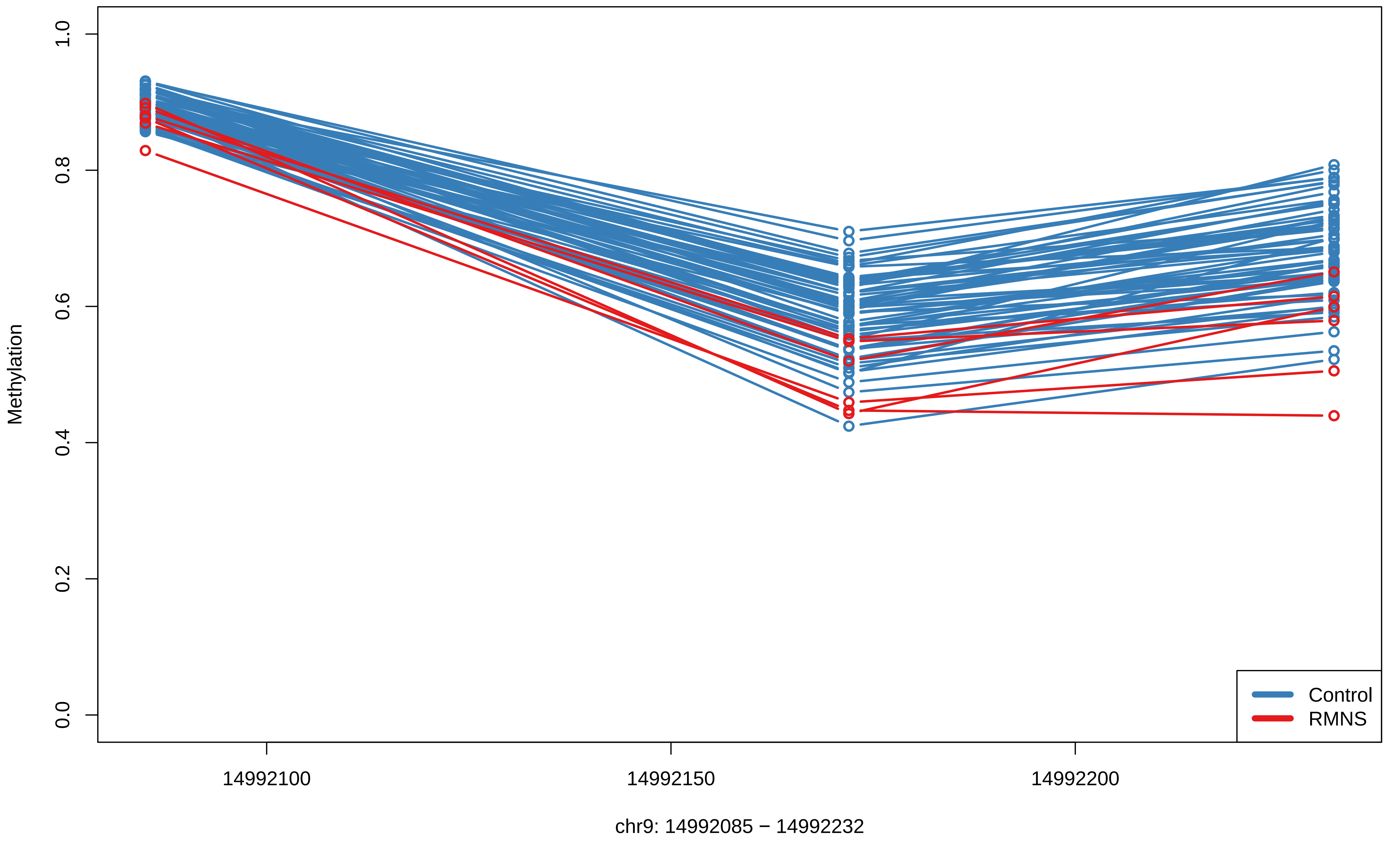

Methylation
0.0
0.2
0.4
0.6
0.8
1.0
14992100
14992150
14992200
chr9: 14992085 − 14992232
Control
RMNS

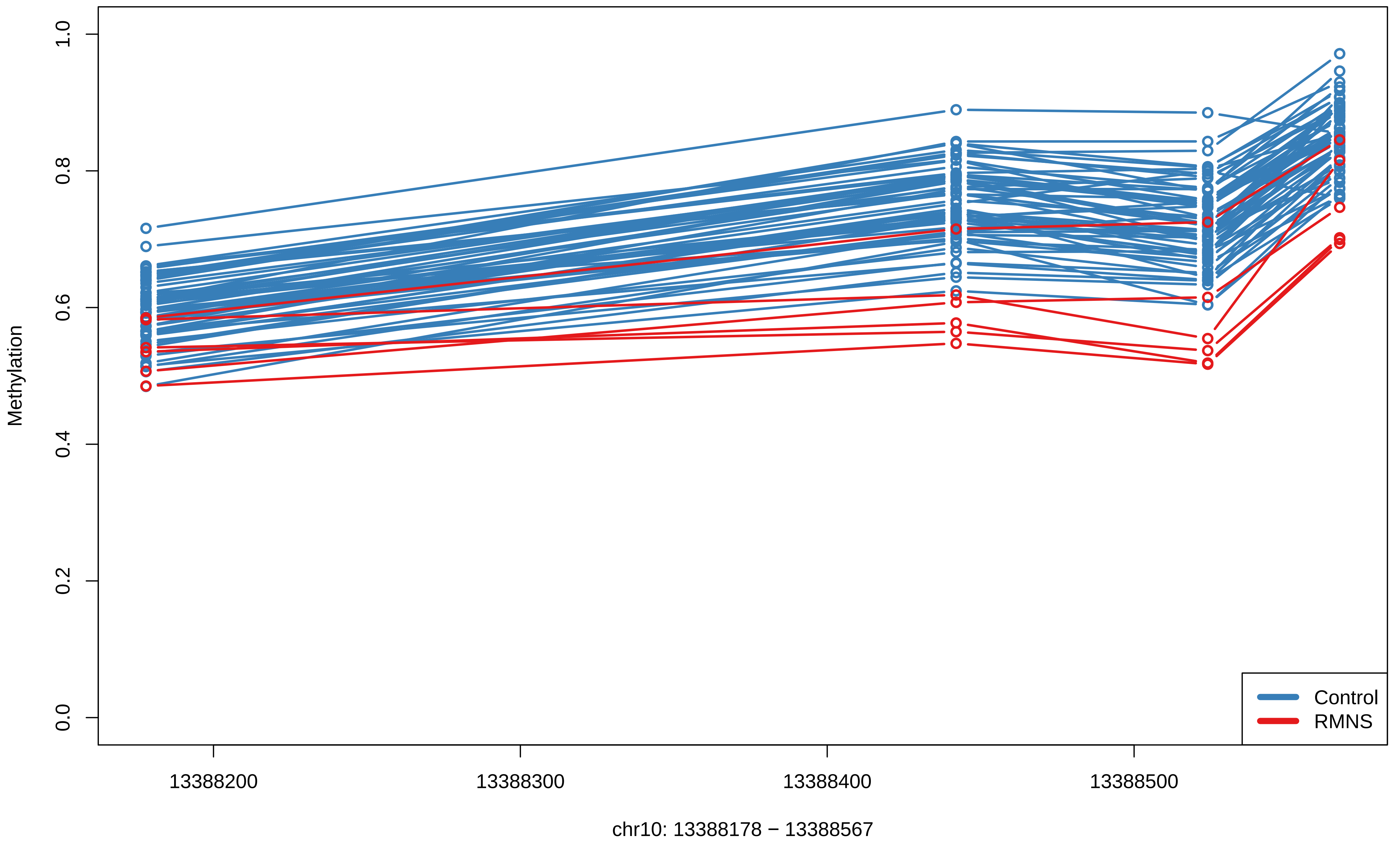

Methylation
1.0
0.8
0.6
0.4
0.2
0.0
13388200
13388300
13388400
13388500
chr10: 13388178 − 13388567
Control
RMNS

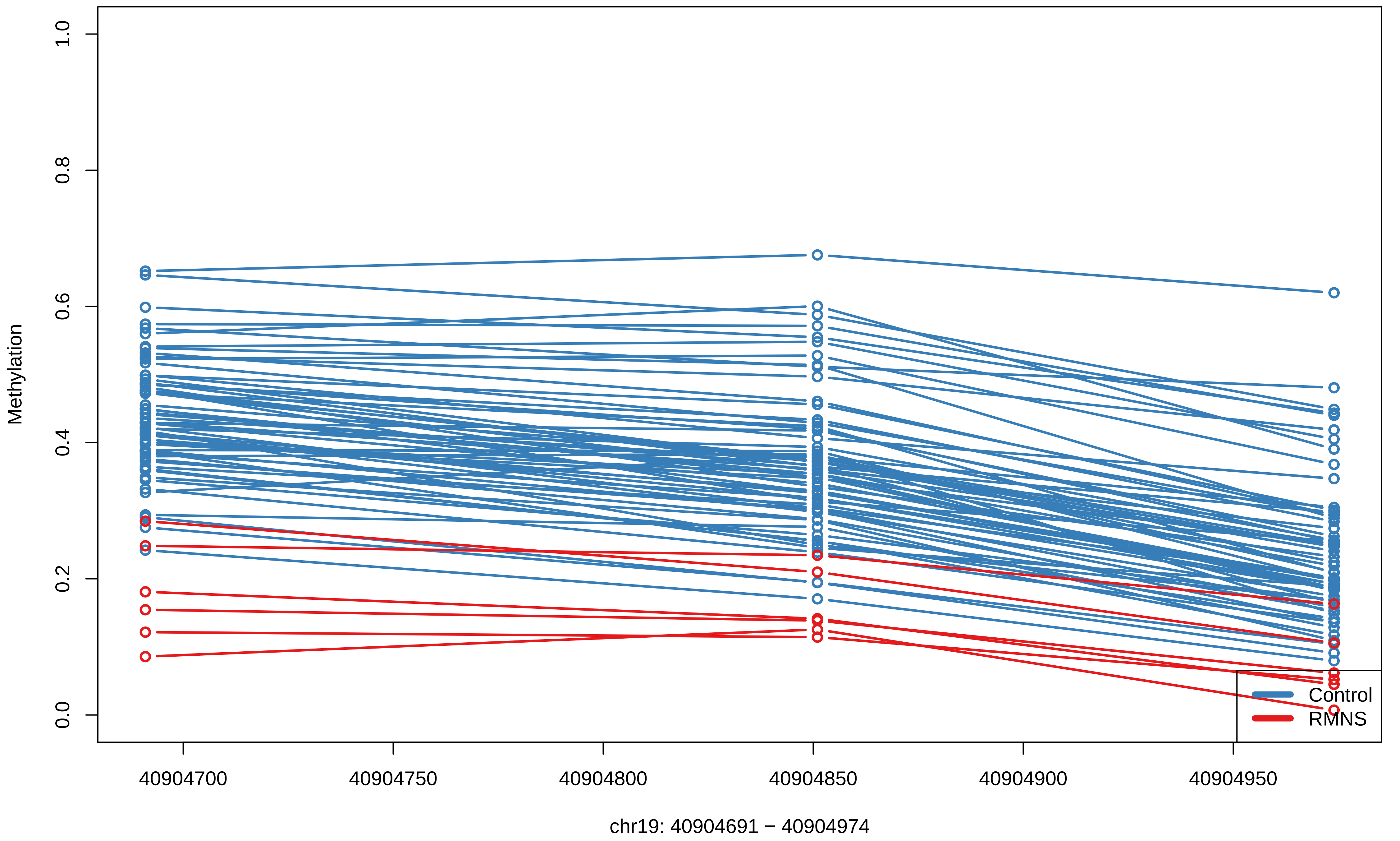

Methylation
0.0
0.2
0.4
0.6
0.8
1.0
40904700
40904750
40904800
40904850
40904900
40904950
chr19: 40904691 − 40904974
Control
RMNS

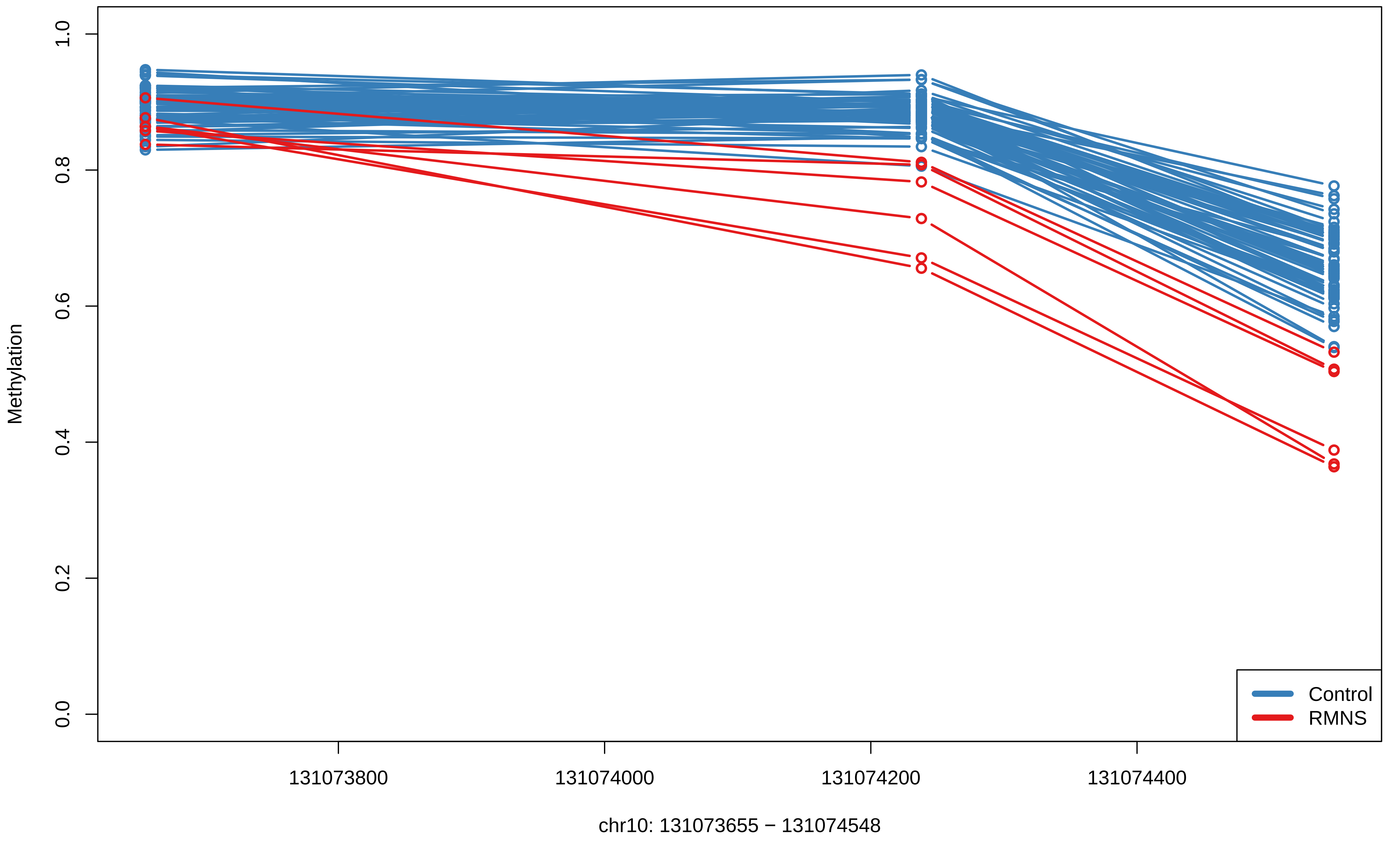

Methylation
1.0
0.8
0.6
0.4
0.2
0.0
131073800
131074000
131074200
131074400
chr10: 131073655 − 131074548
Control
RMNS

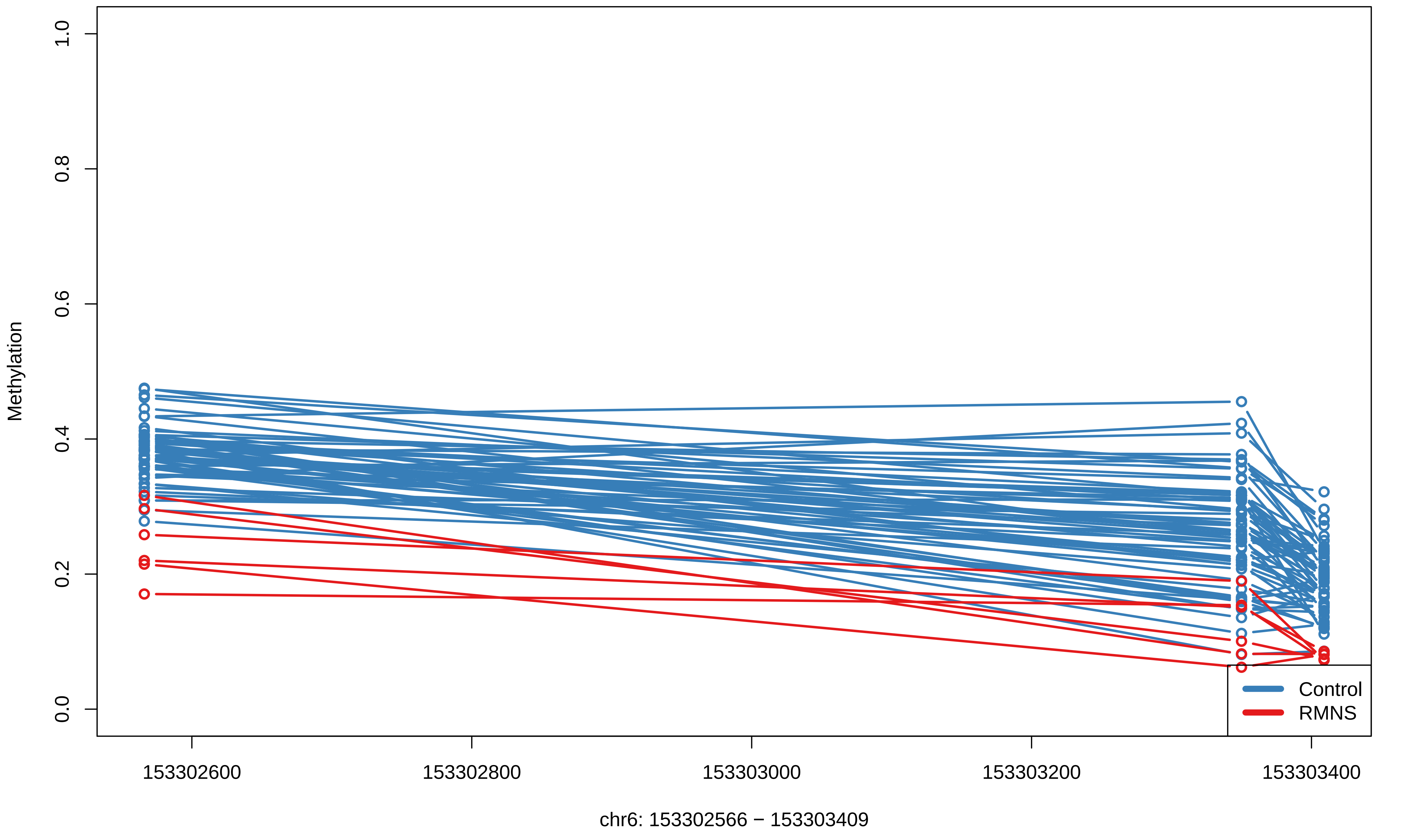

Methylation
0.0
0.2
0.4
0.6
0.8
1.0
153302600
153302800
153303000
153303200
153303400
chr6: 153302566 − 153303409
Control
RMNS

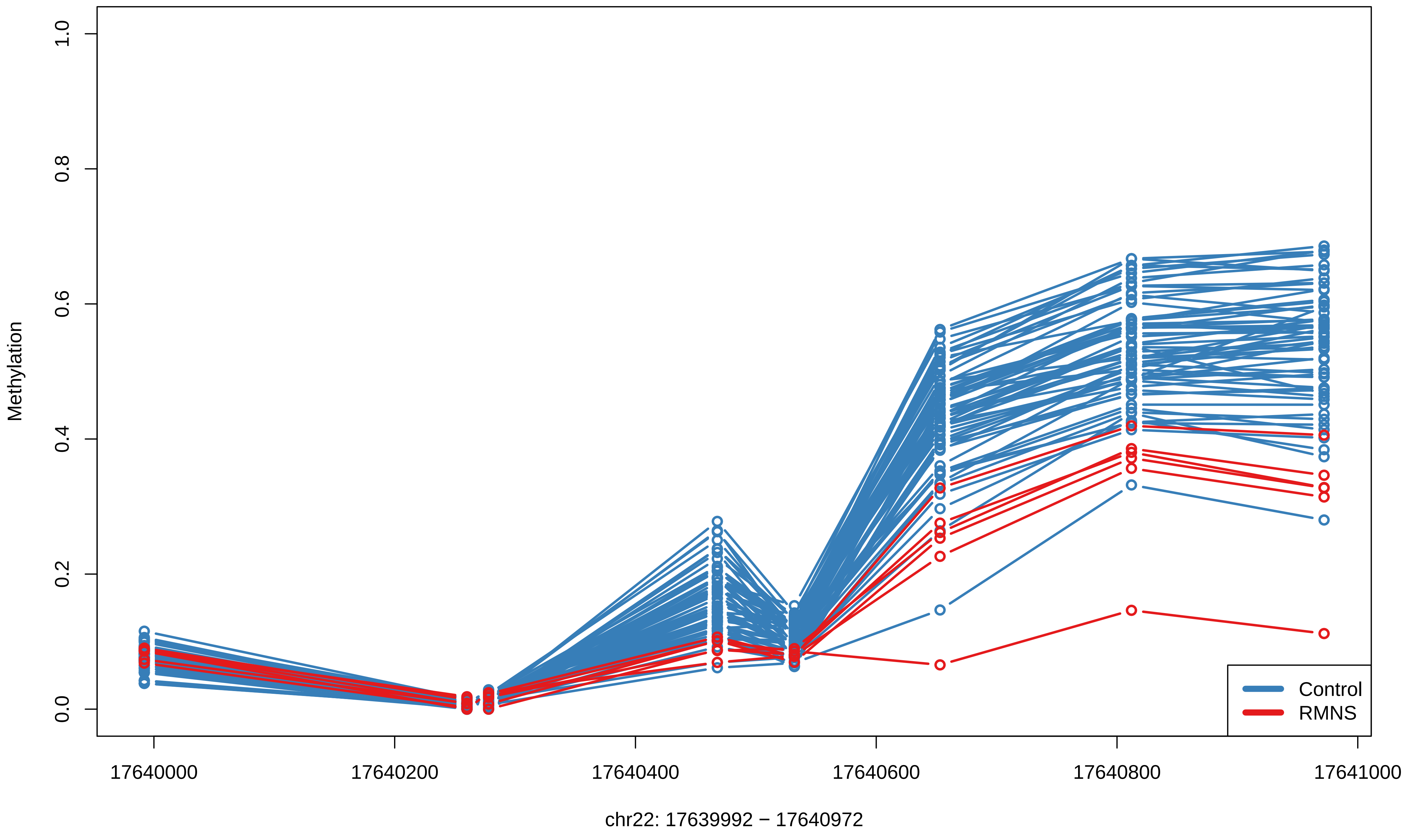

Methylation
0.0
0.2
0.4
0.6
0.8
1.0
17640000
17640200
17640400
17640600
17640800
17641000
chr22: 17639992 − 17640972
Control
RMNS

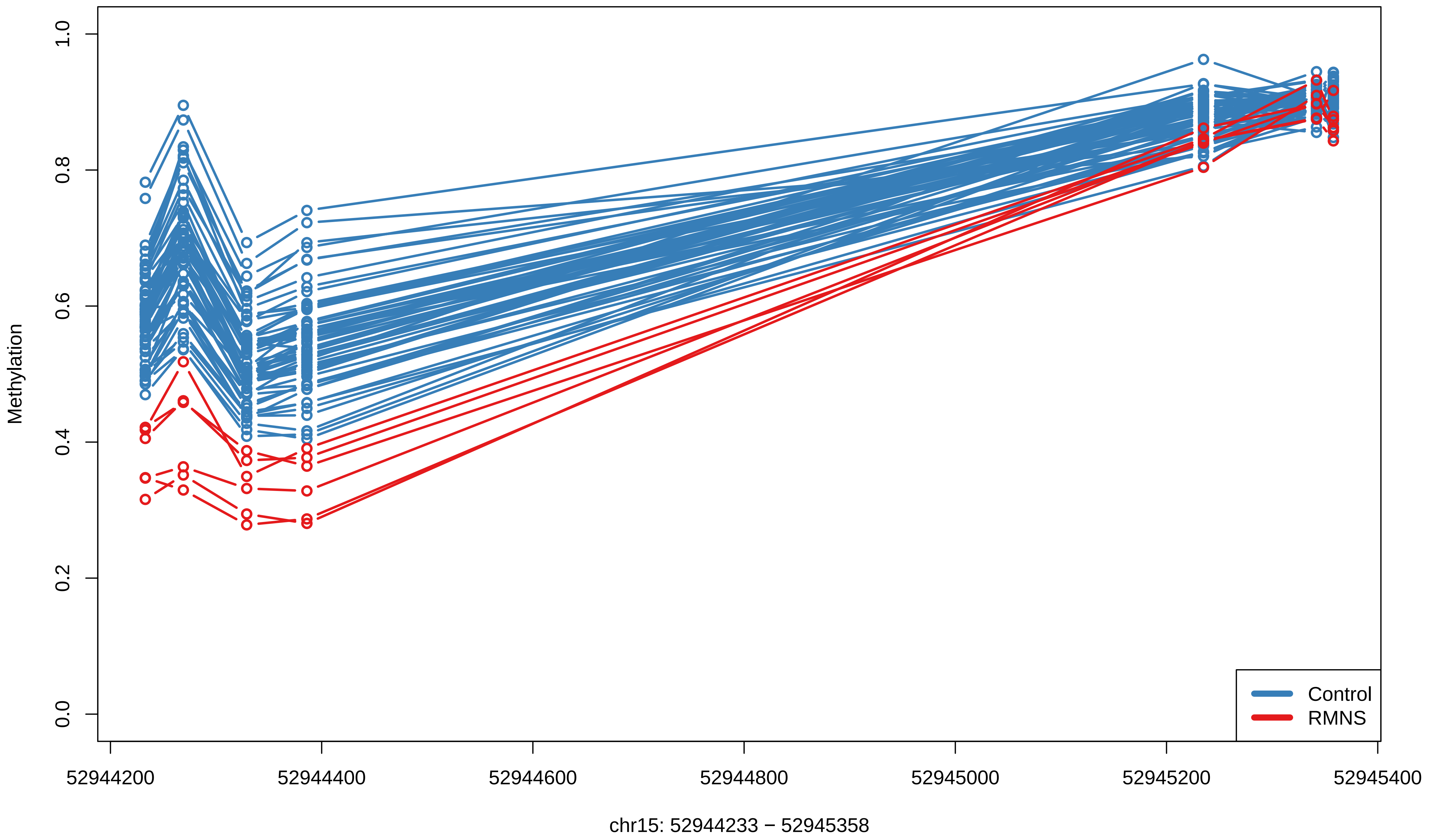

Methylation
0.0
0.2
0.4
0.6
0.8
1.0
52944200
52944400
52944600
52944800
52945000
52945200
52945400
chr15: 52944233 − 52945358
Control
RMNS

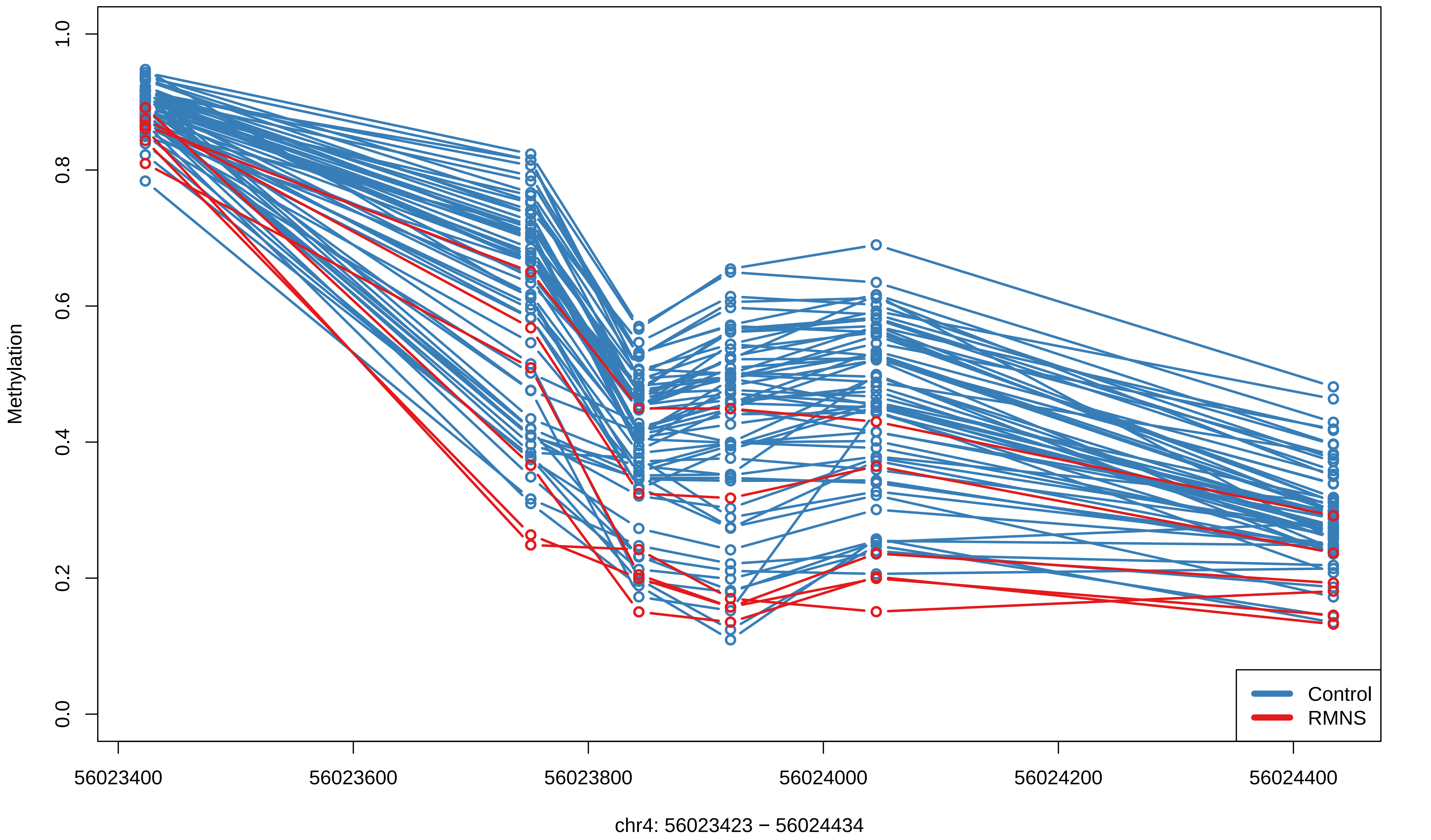
Methylation
1.0
0.8
0.6
0.4
0.2
0.0
56023400
56023600
56023800
56024000
56024200
56024400
chr4: 56023423 − 56024434
Control
RMNS

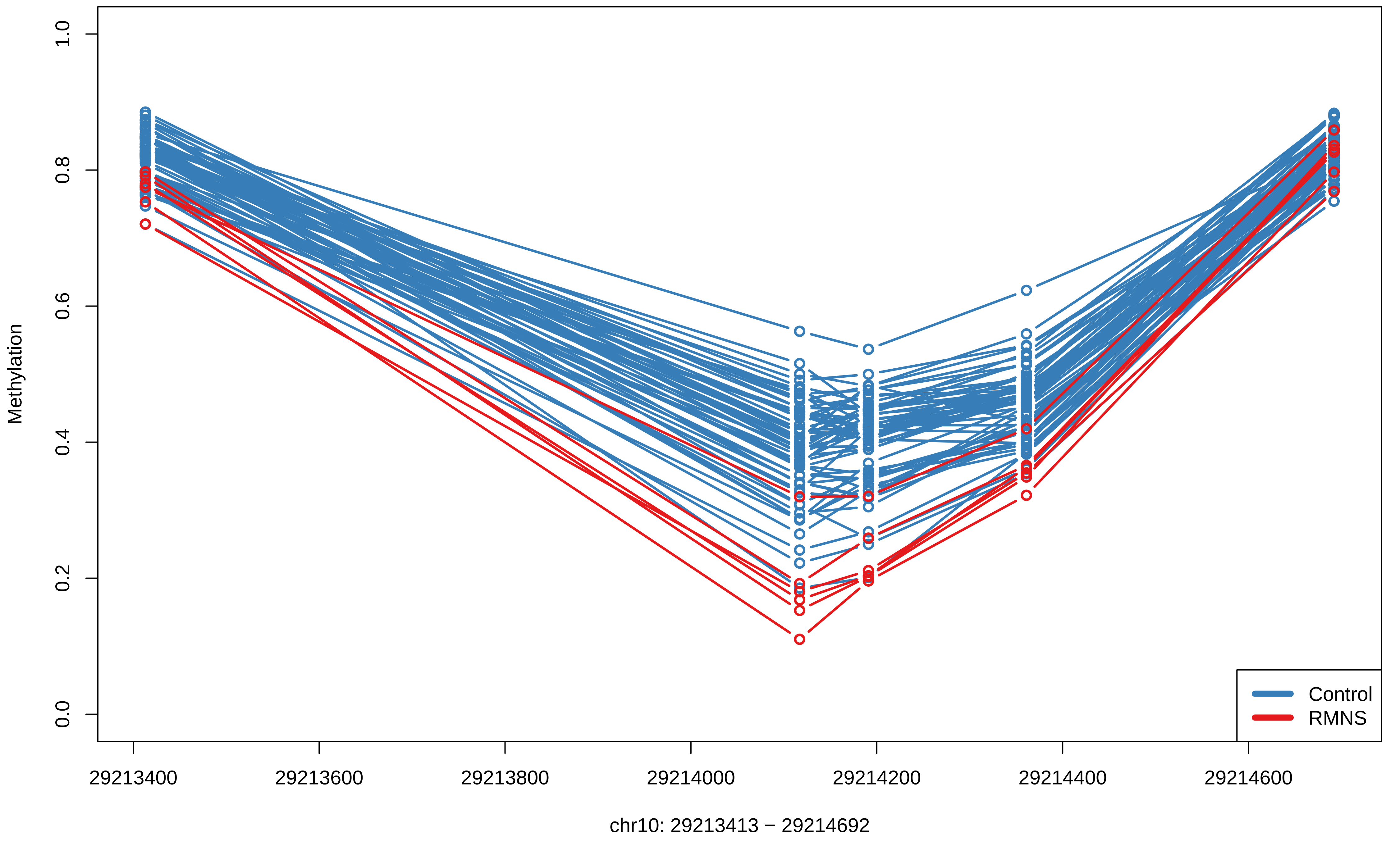

1.0
0.8
0.6
0.4
0.2
0.0
Methylation
29213400
29213600
29213800
29214000
29214200
29214400
29214600
chr10: 29213413 − 29214692
Control
RMNS

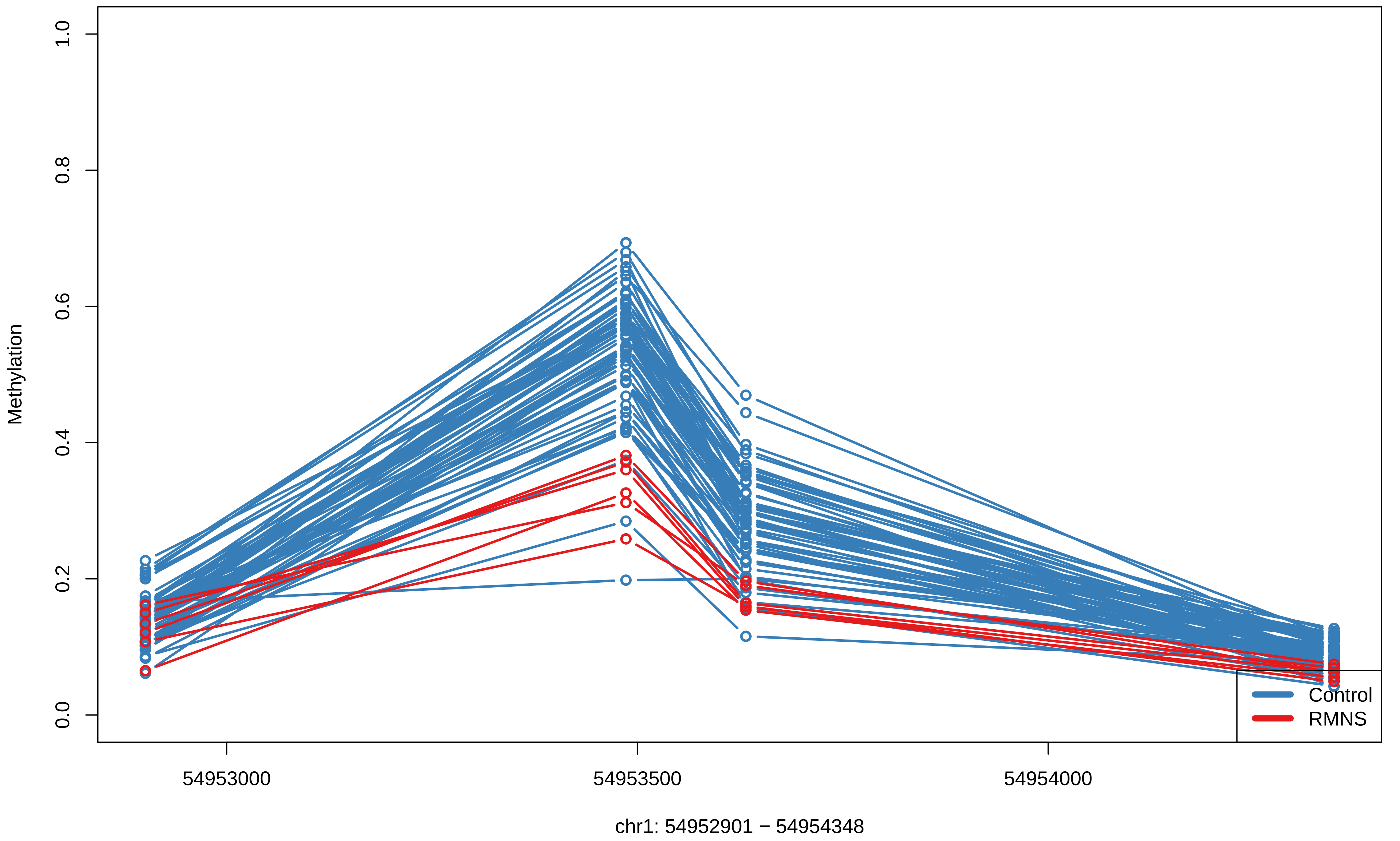

Methylation
0.0
0.2
0.4
0.6
0.8
1.0
54953000
54953500
54954000
chr1: 54952901 − 54954348
Control
RMNS

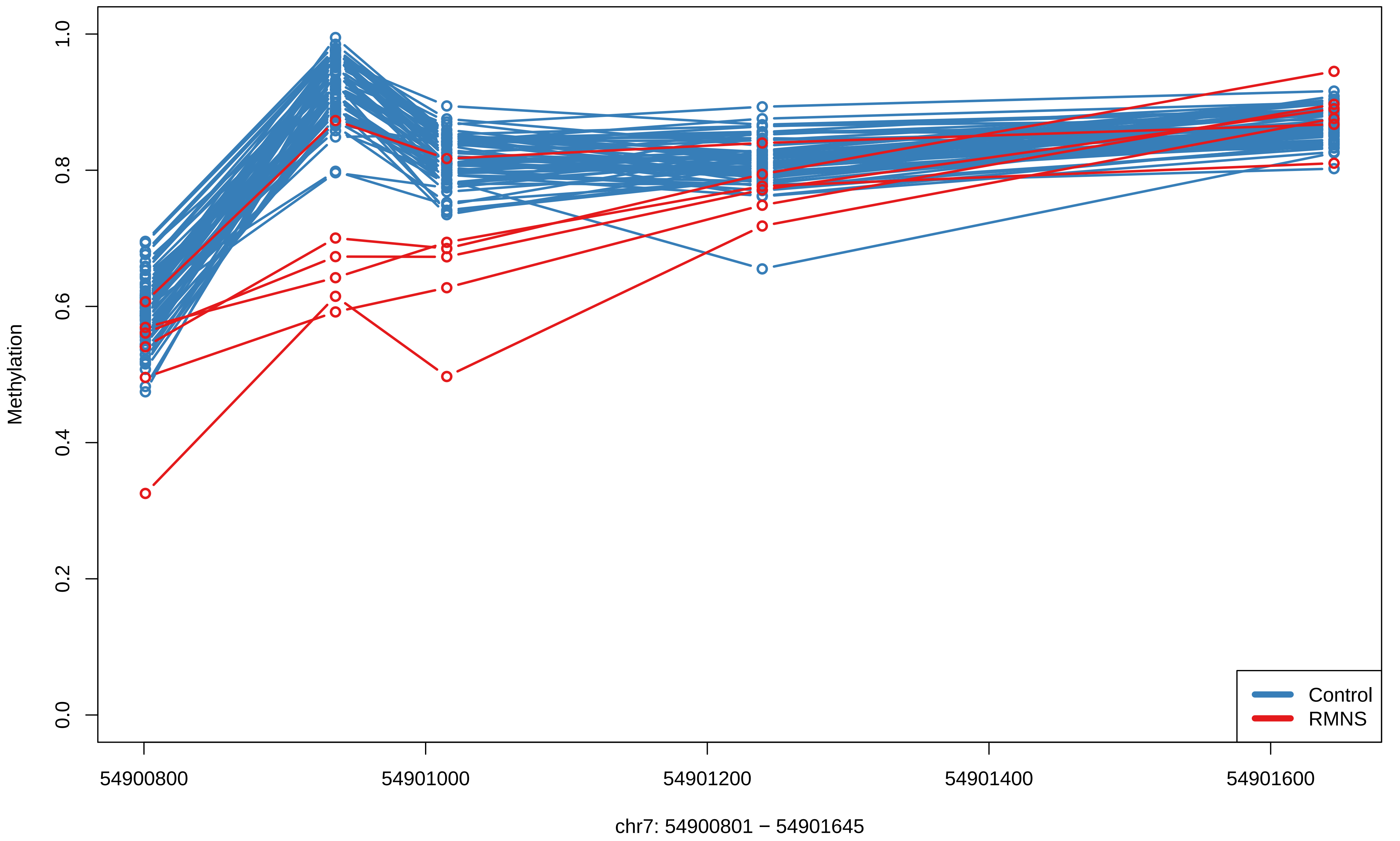

Methylation
1.0
0.8
0.6
0.4
0.2
0.0
54900800
54901000
54901200
54901400
54901600
chr7: 54900801 − 54901645
Control
RMNS

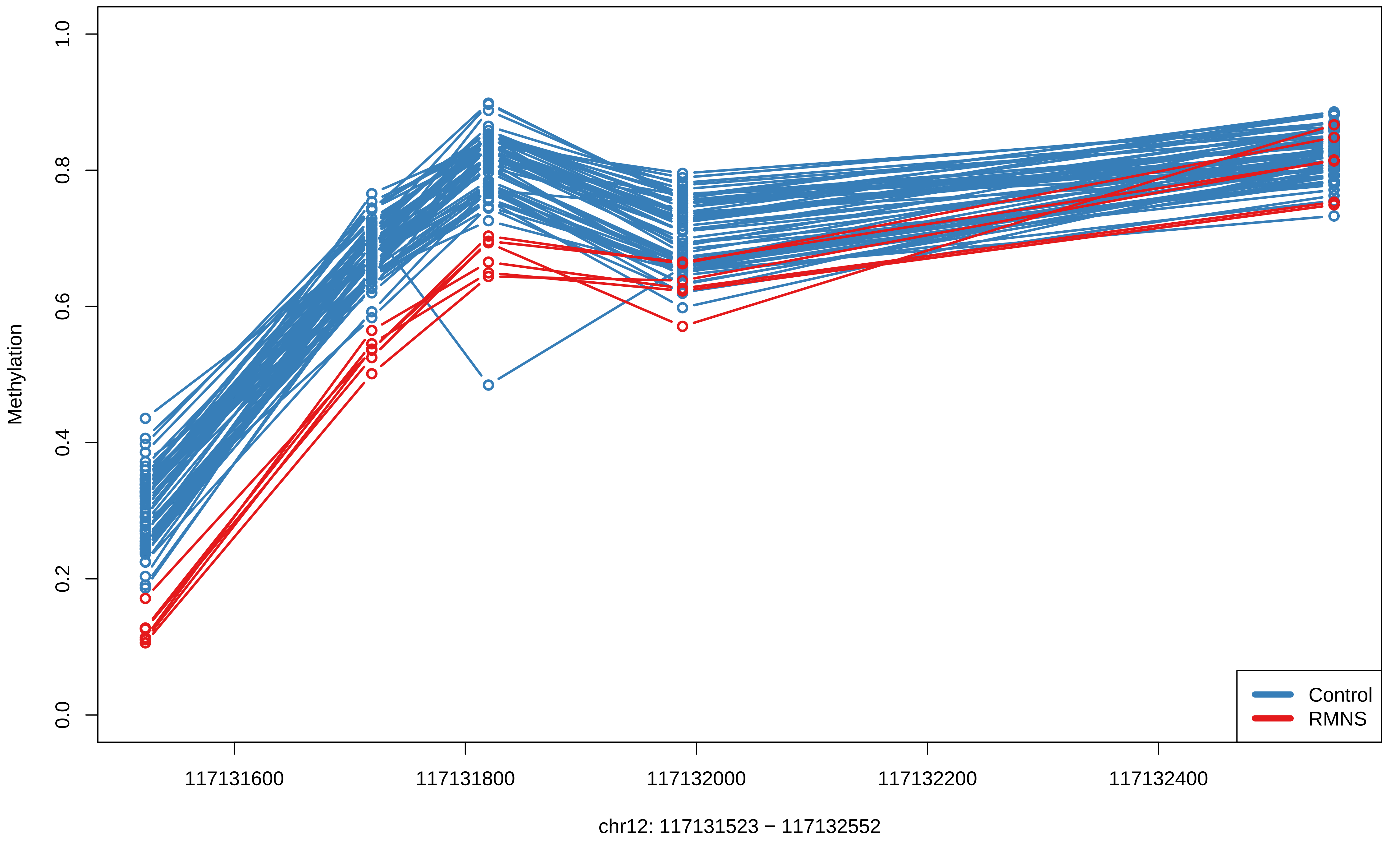

Methylation
0.0
0.2
0.4
0.6
0.8
1.0
117131600
117131800
117132000
117132200
117132400
chr12: 117131523 − 117132552
Control
RMNS

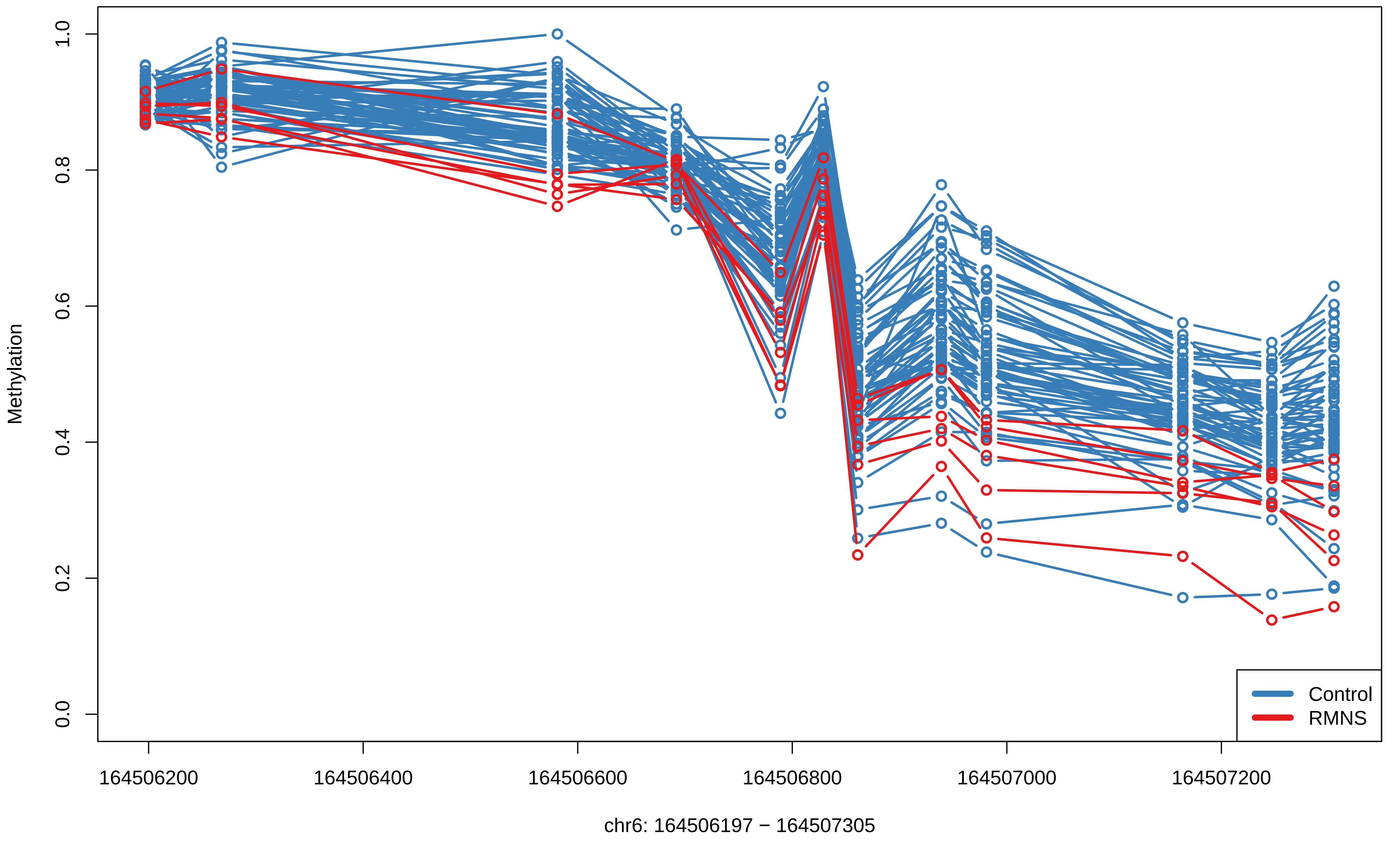

Methylation
0.0
0.2
0.4
0.6
0.8
1.0
164506200
164506400
164506600
164506800
164507000
164507200
chr6: 164506197 − 164507305
Control
RMNS

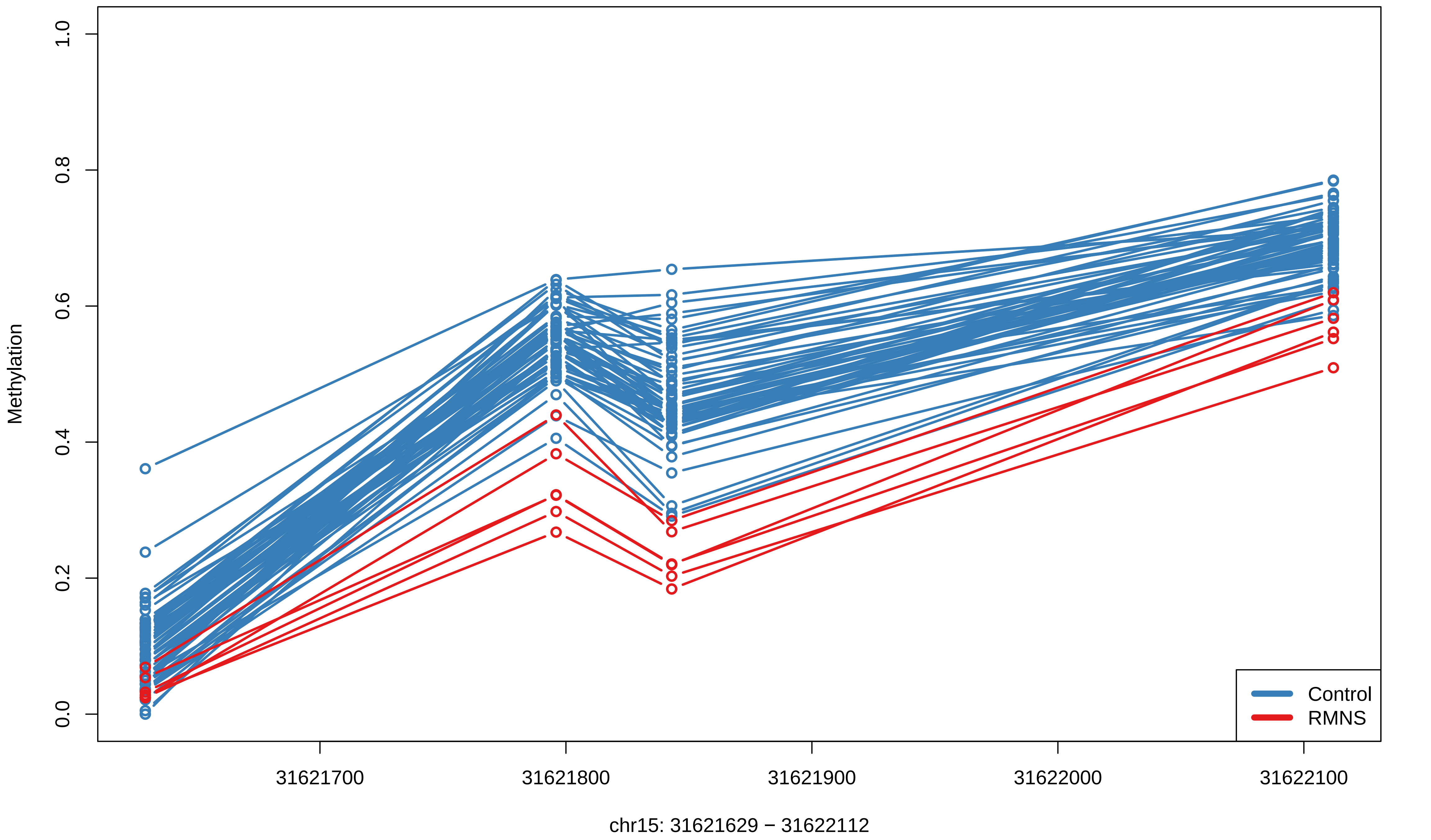

Methylation
0.0
0.2
0.4
0.6
0.8
1.0
31621700
31621800
31621900
31622000
31622100
chr15: 31621629 − 31622112
Control
RMNS

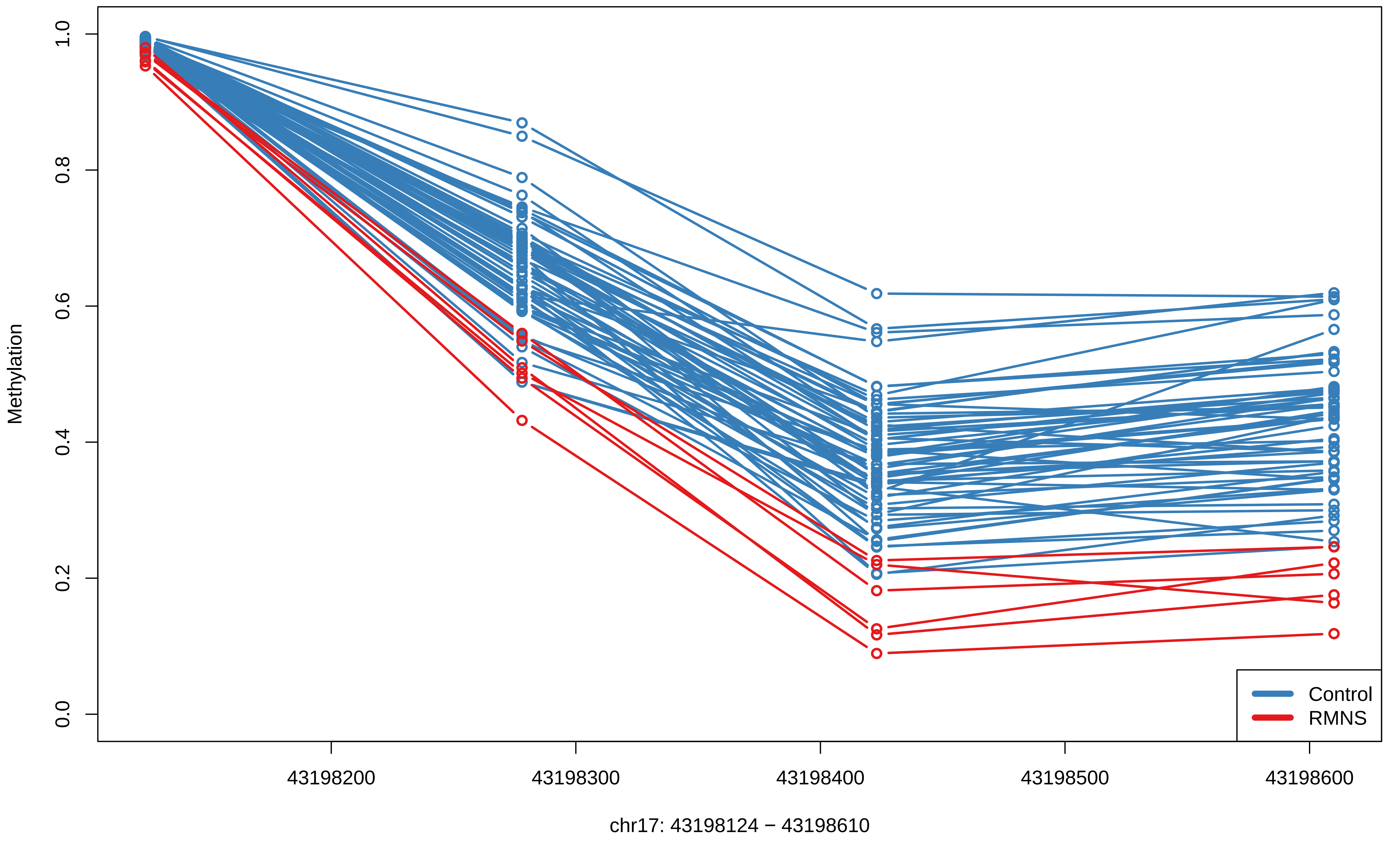

Methylation
1.0
0.8
0.6
0.4
0.2
0.0
43198200
43198300
43198400
43198500
43198600
chr17: 43198124 − 43198610
Control
RMNS

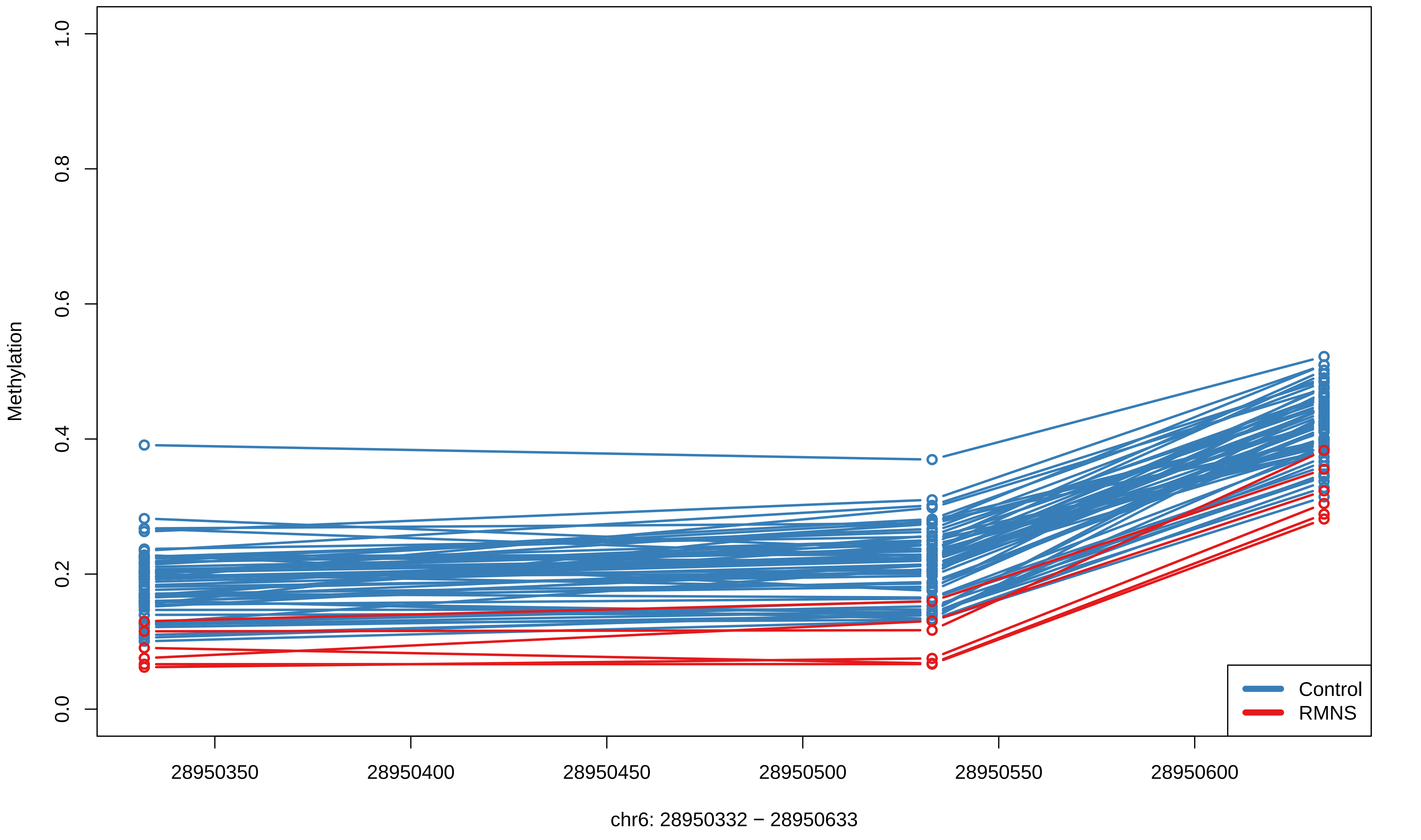

Methylation
0.0
0.2
0.4
0.6
0.8
1.0
28950350
28950400
28950450
28950500
28950550
28950600
chr6: 28950332 − 28950633
Control
RMNS

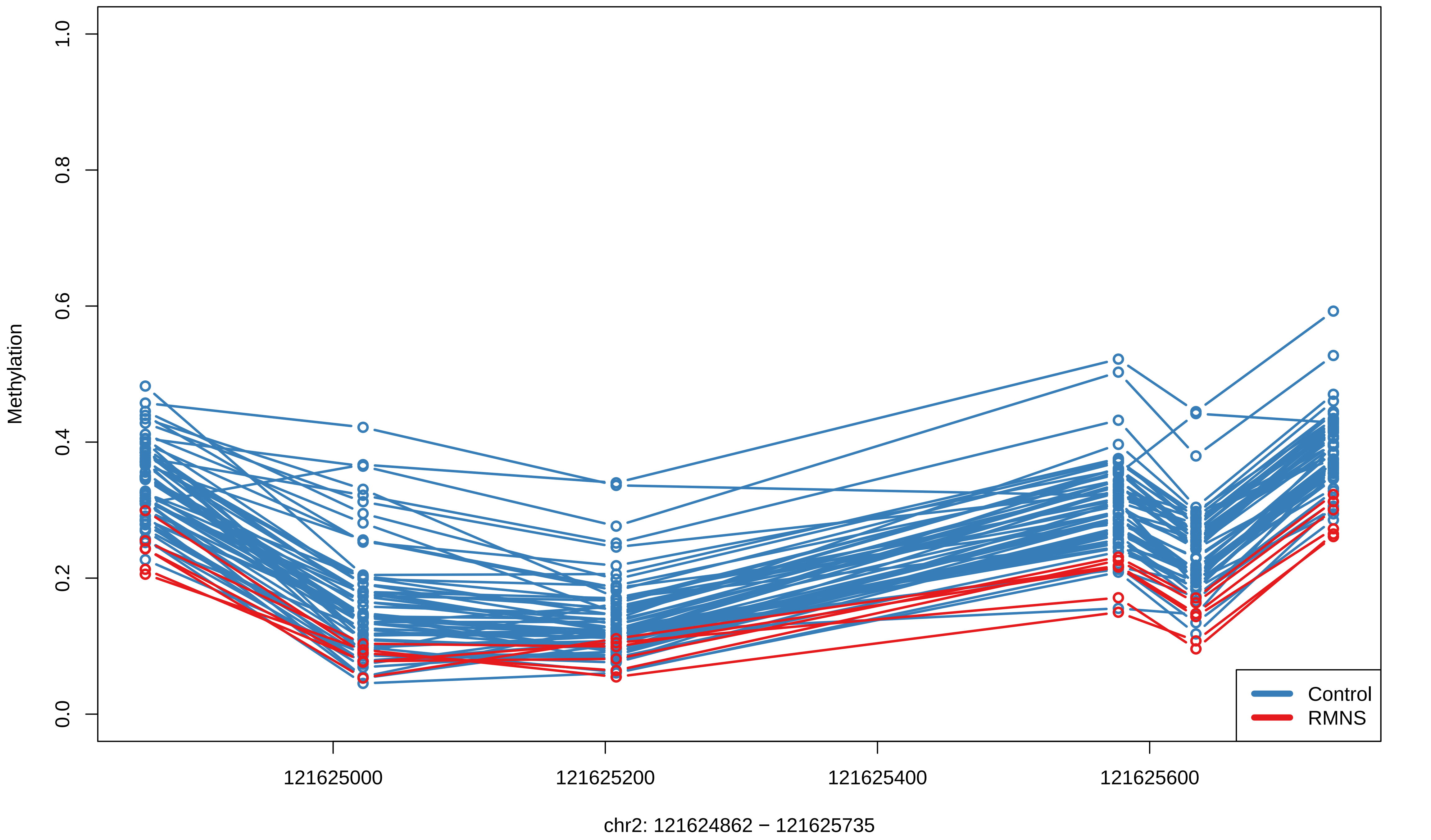

Methylation
0.0
0.2
0.4
0.6
0.8
1.0
121625000
121625200
121625400
121625600
chr2: 121624862 − 121625735
Control
RMNS

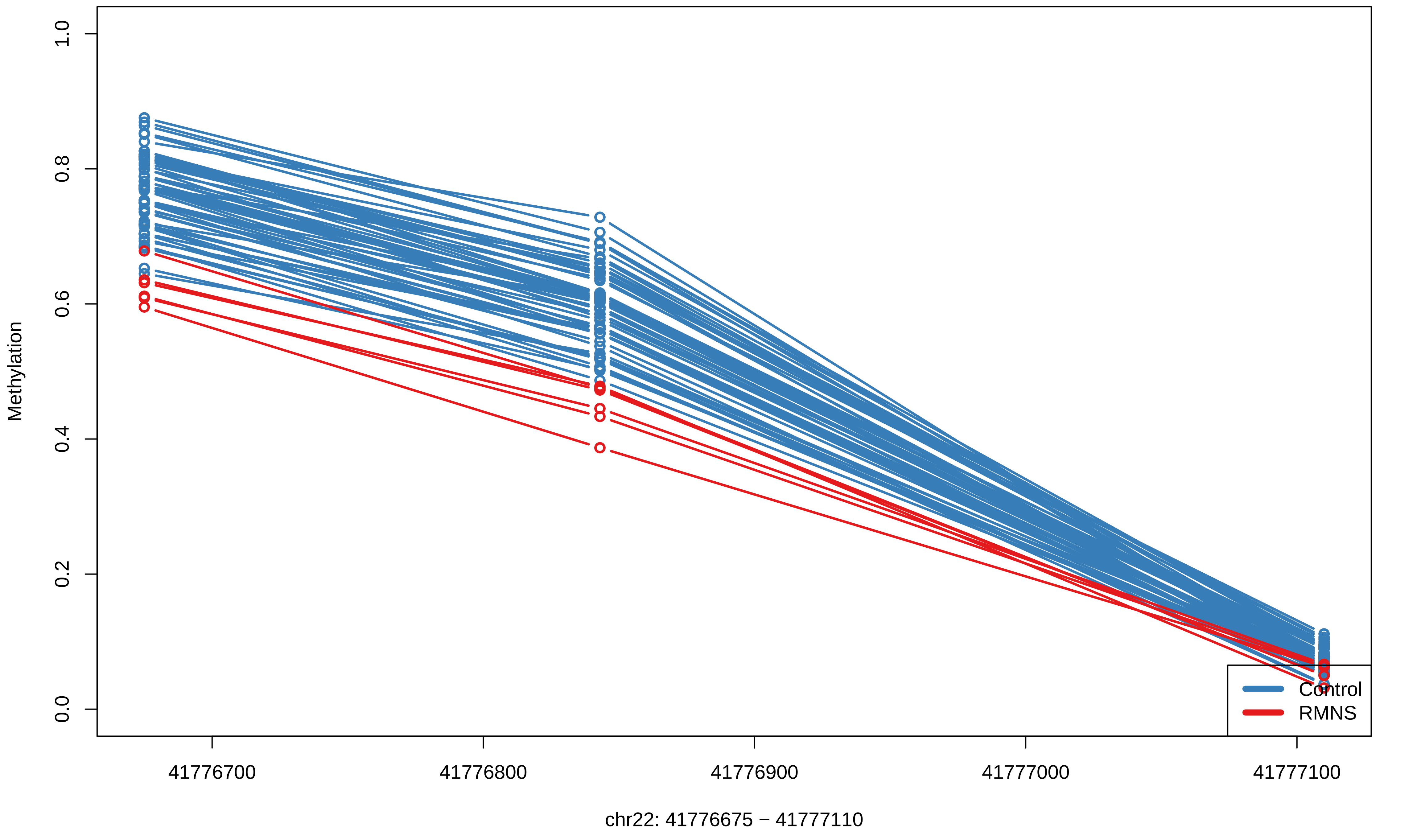

1.0
0.8
0.6
0.4
0.2
0.0
Methylation
41776700
41776800
41776900
41777000
41777100
chr22: 41776675 − 41777110
Control
RMNS

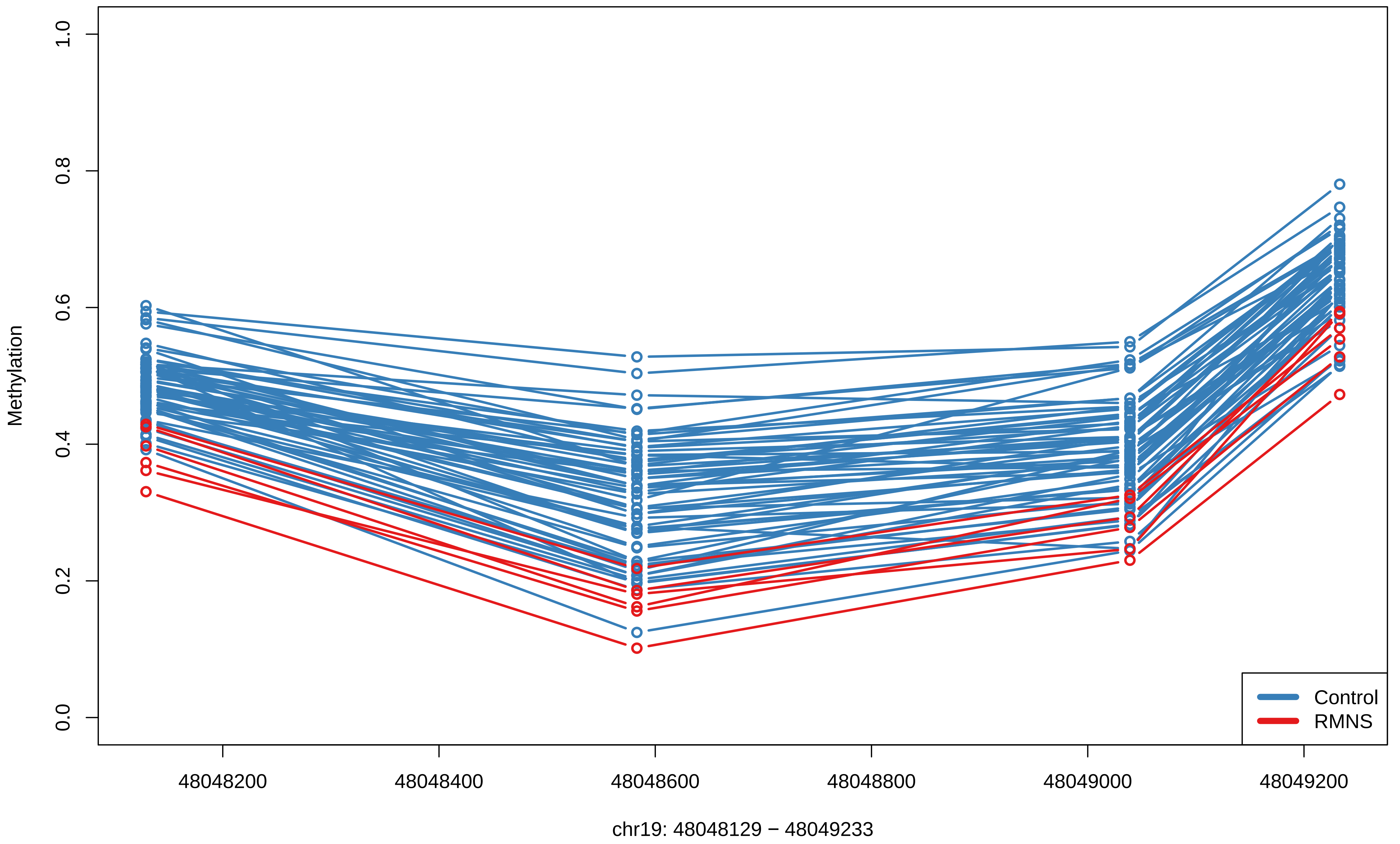

Methylation
0.0
0.2
0.4
0.6
0.8
1.0
48048200
48048400
48048600
48048800
48049000
48049200
chr19: 48048129 − 48049233
Control
RMNS

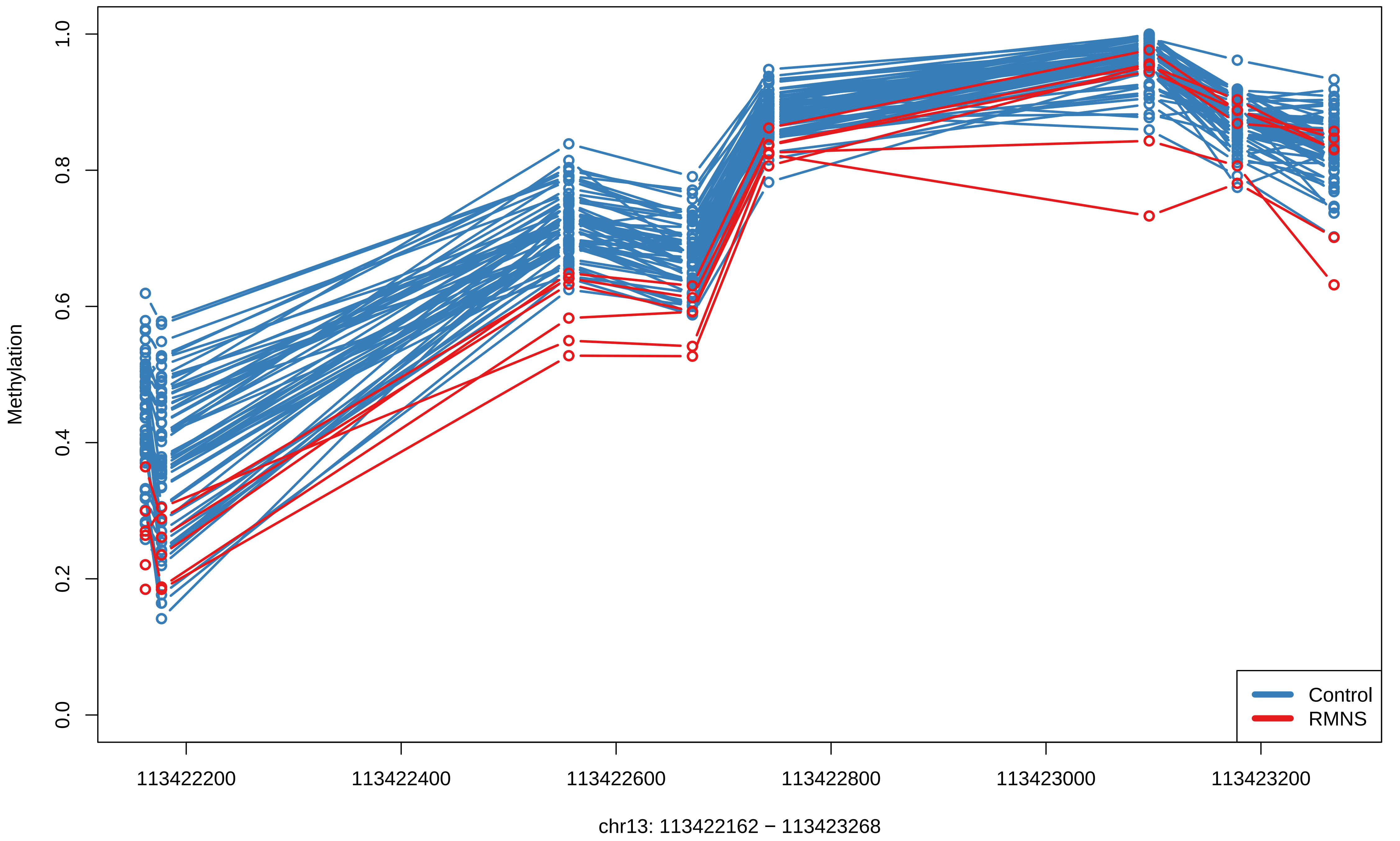

1.0
0.8
0.6
0.4
0.2
0.0
Methylation
113422200
113422400
113422600
113422800
113423000
113423200
chr13: 113422162 − 113423268
Control
RMNS

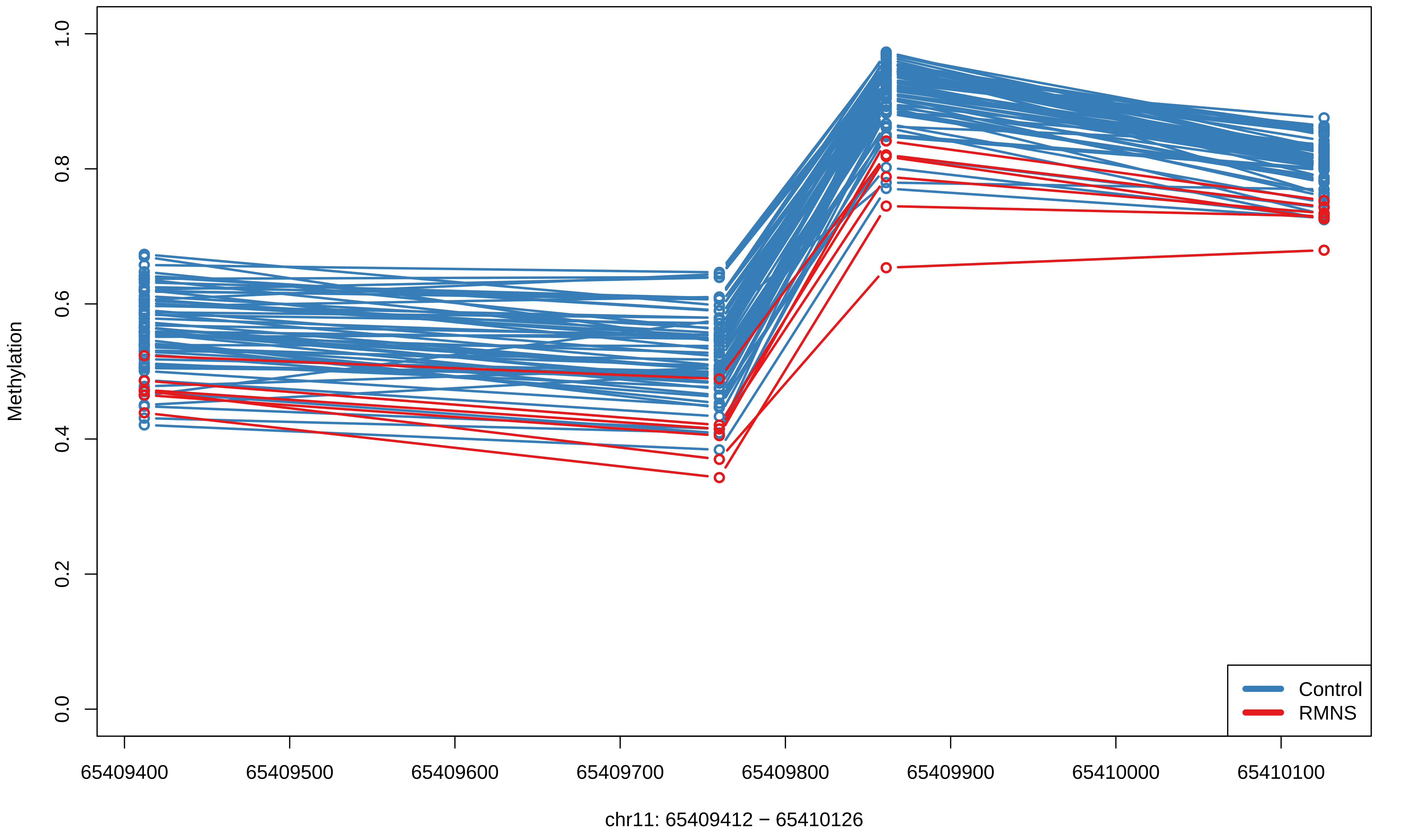

Methylation
1.0
0.8
0.6
0.4
0.2
0.0
65409400
65409500
65409600
65409700
65409800
65409900
65410000
65410100
chr11: 65409412 − 65410126
Control
RMNS

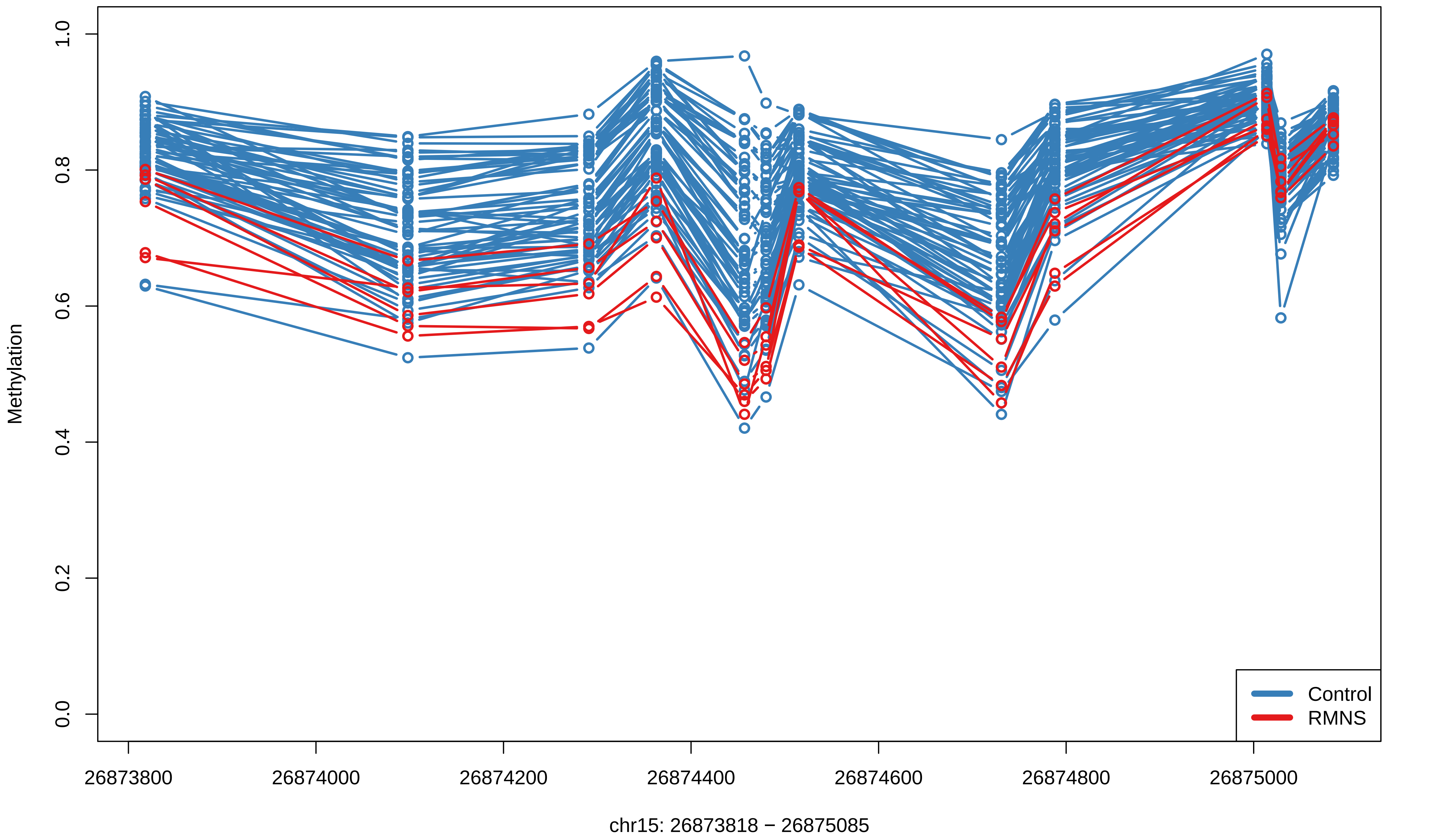

Methylation
1.0
0.8
0.6
0.4
0.2
0.0
26873800
26874000
26874200
26874400
26874600
26874800
26875000
chr15: 26873818 − 26875085
Control
RMNS

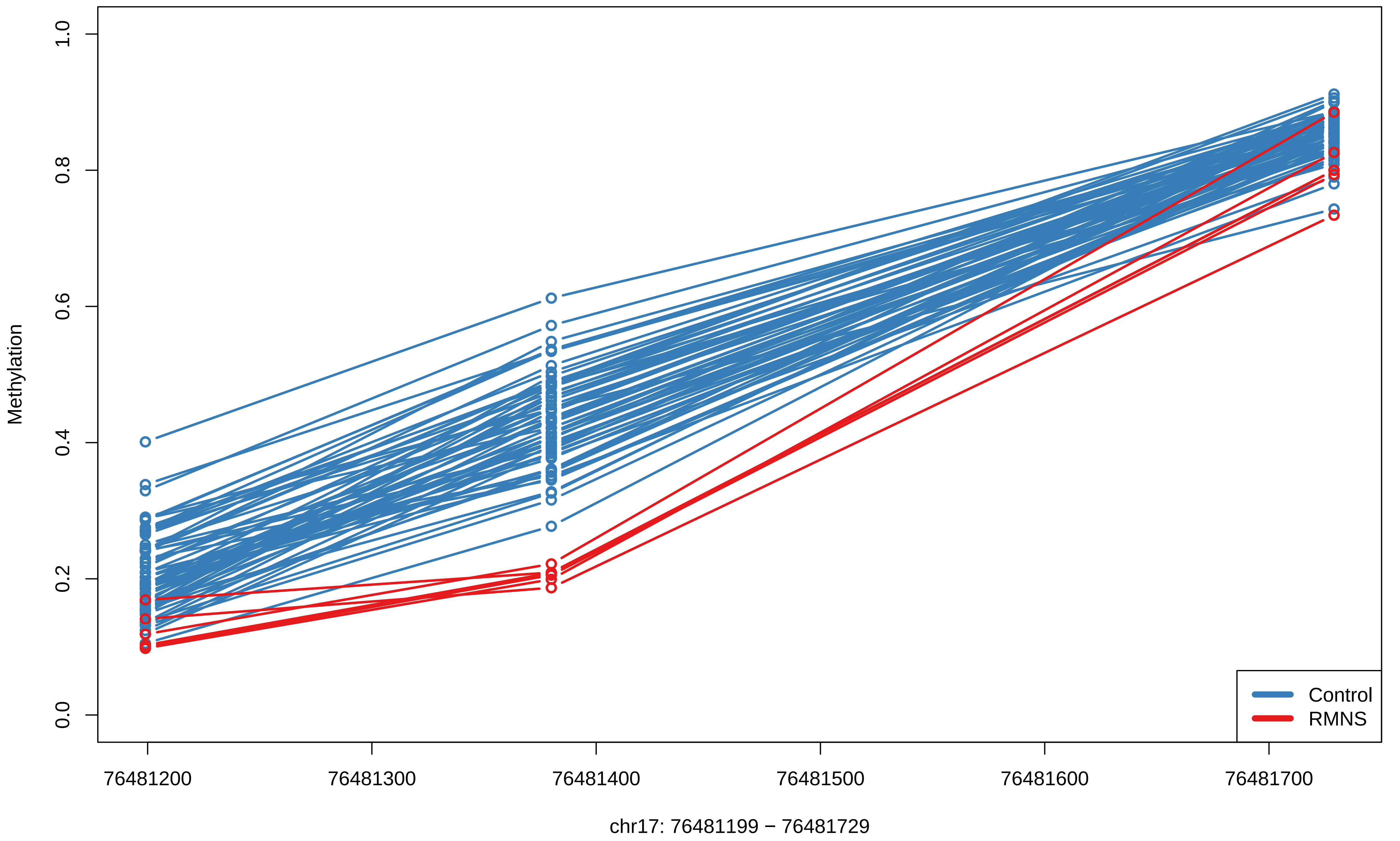

Methylation
0.0
0.2
0.4
0.6
0.8
1.0
76481200
76481300
76481400
76481500
76481600
76481700
chr17: 76481199 − 76481729
Control
RMNS

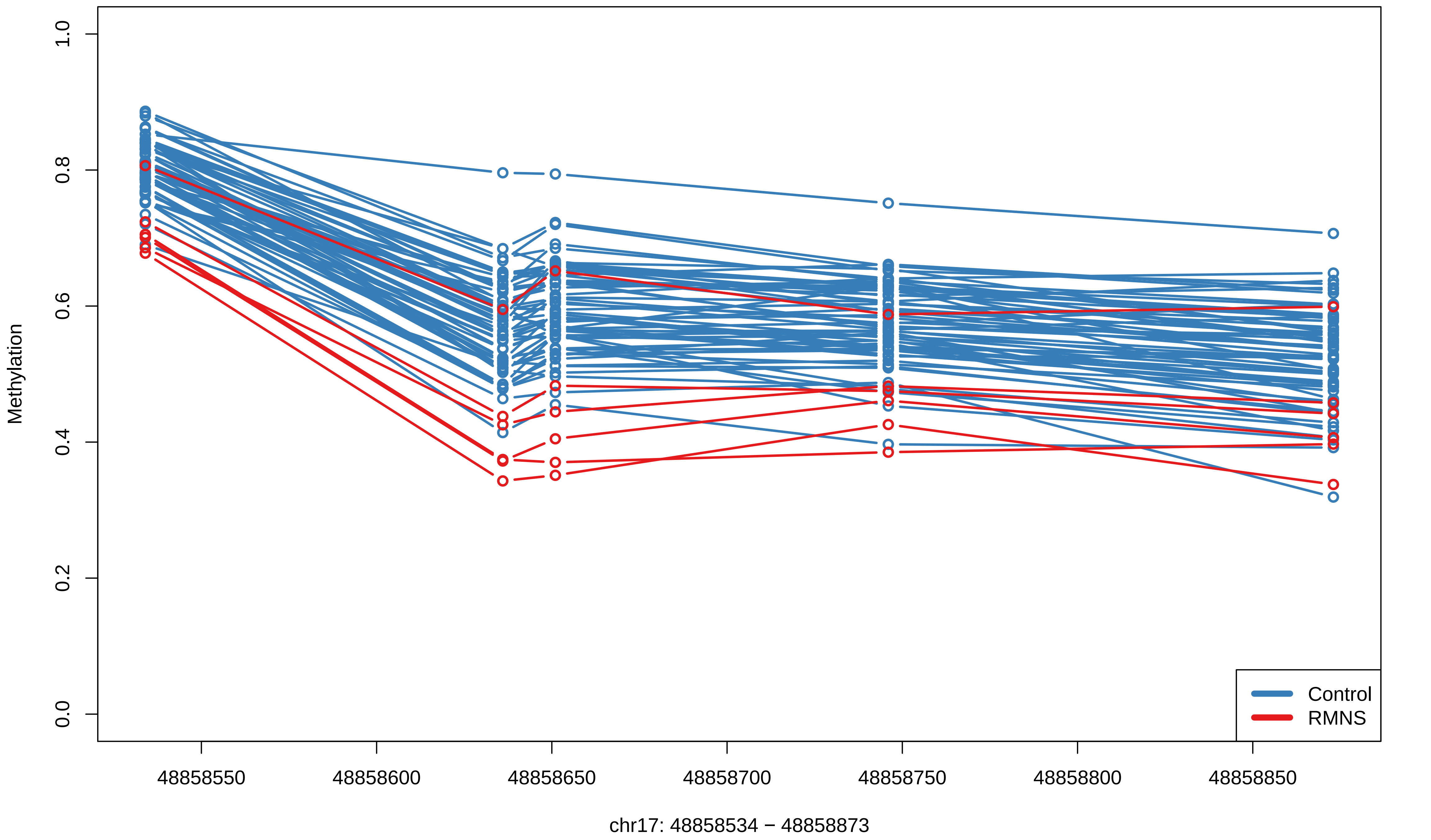

Methylation
0.0
0.2
0.4
0.6
0.8
1.0
48858550
48858600
48858650
48858700
48858750
48858800
48858850
chr17: 48858534 − 48858873
Control
RMNS

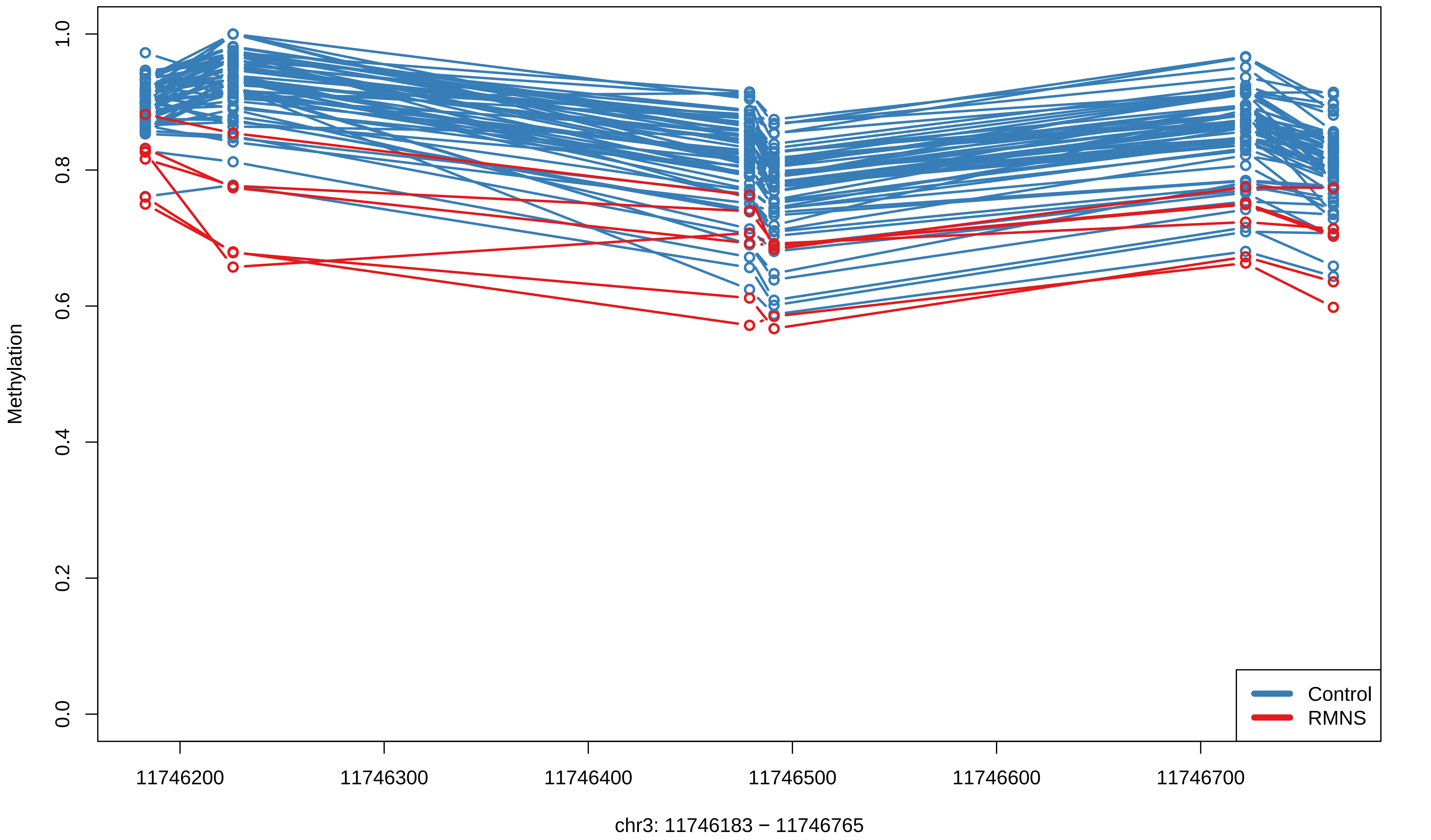

Methylation
0.0
0.2
0.4
0.6
0.8
1.0
11746200
11746300
11746400
11746500
11746600
11746700
chr3: 11746183 − 11746765
Control
RMNS

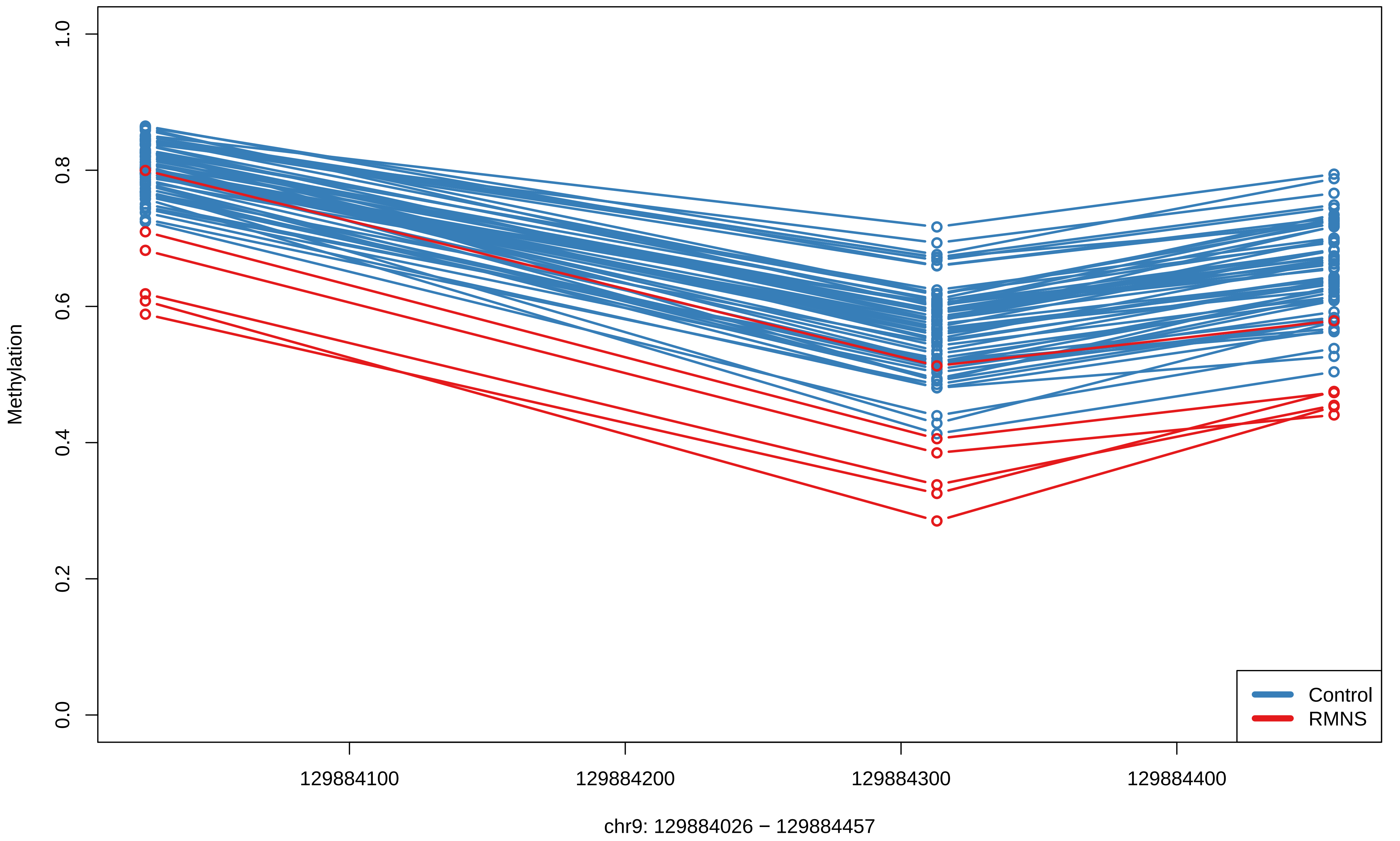

Methylation
0.0
0.2
0.4
0.6
0.8
1.0
129884100
129884200
129884300
129884400
chr9: 129884026 − 129884457
Control
RMNS

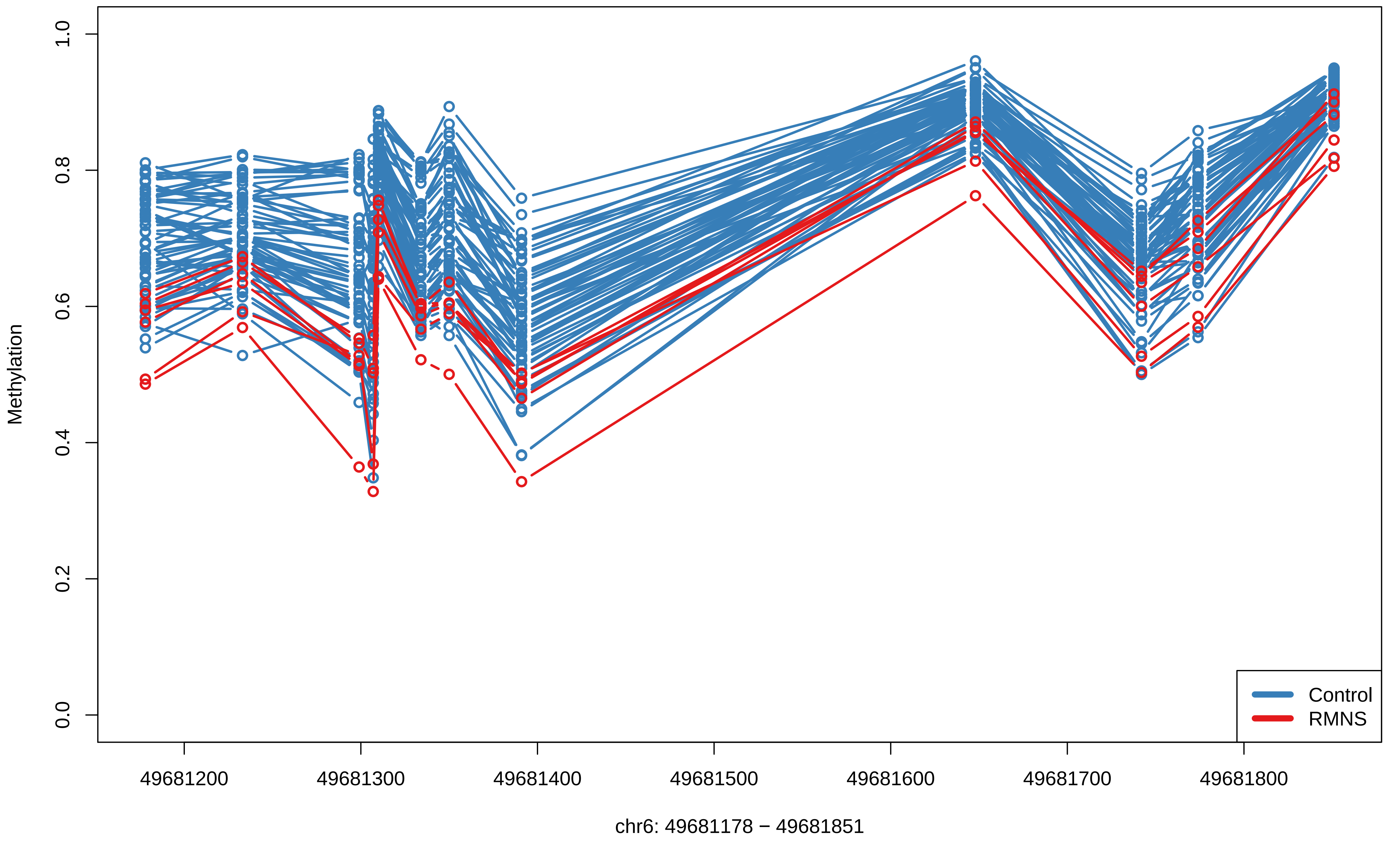

Methylation
1.0
0.8
0.6
0.4
0.2
0.0
49681200
49681300
49681400
49681500
49681600
49681700
49681800
chr6: 49681178 − 49681851
Control
RMNS

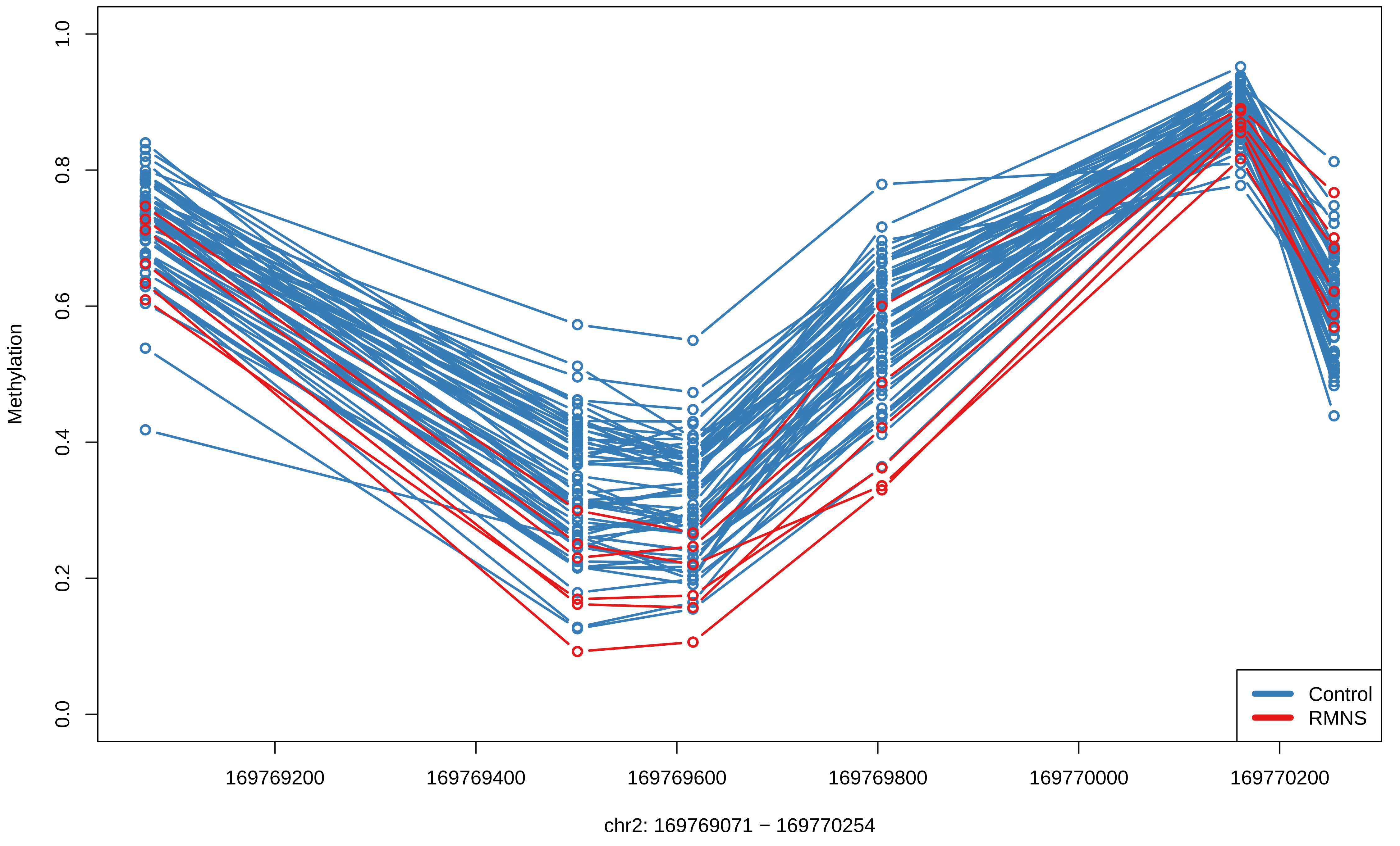

1.0
0.8
0.6
0.4
0.2
0.0
Methylation
169769200
169769400
169769600
169769800
169770000
169770200
chr2: 169769071 − 169770254
Control
RMNS

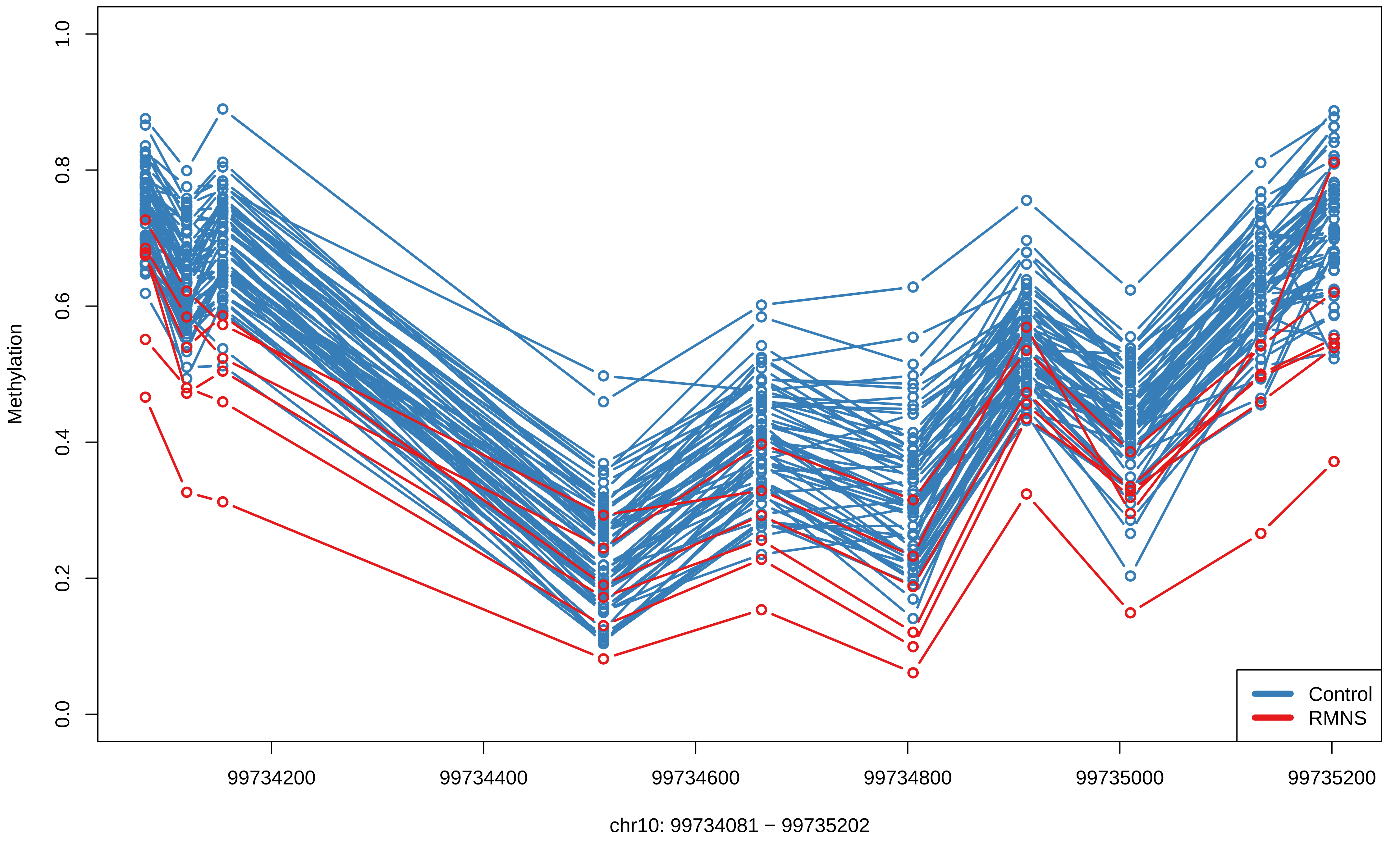

Methylation
0.0
0.2
0.4
0.6
0.8
1.0
99734200
99734400
99734600
99734800
99735000
99735200
chr10: 99734081 − 99735202
Control
RMNS

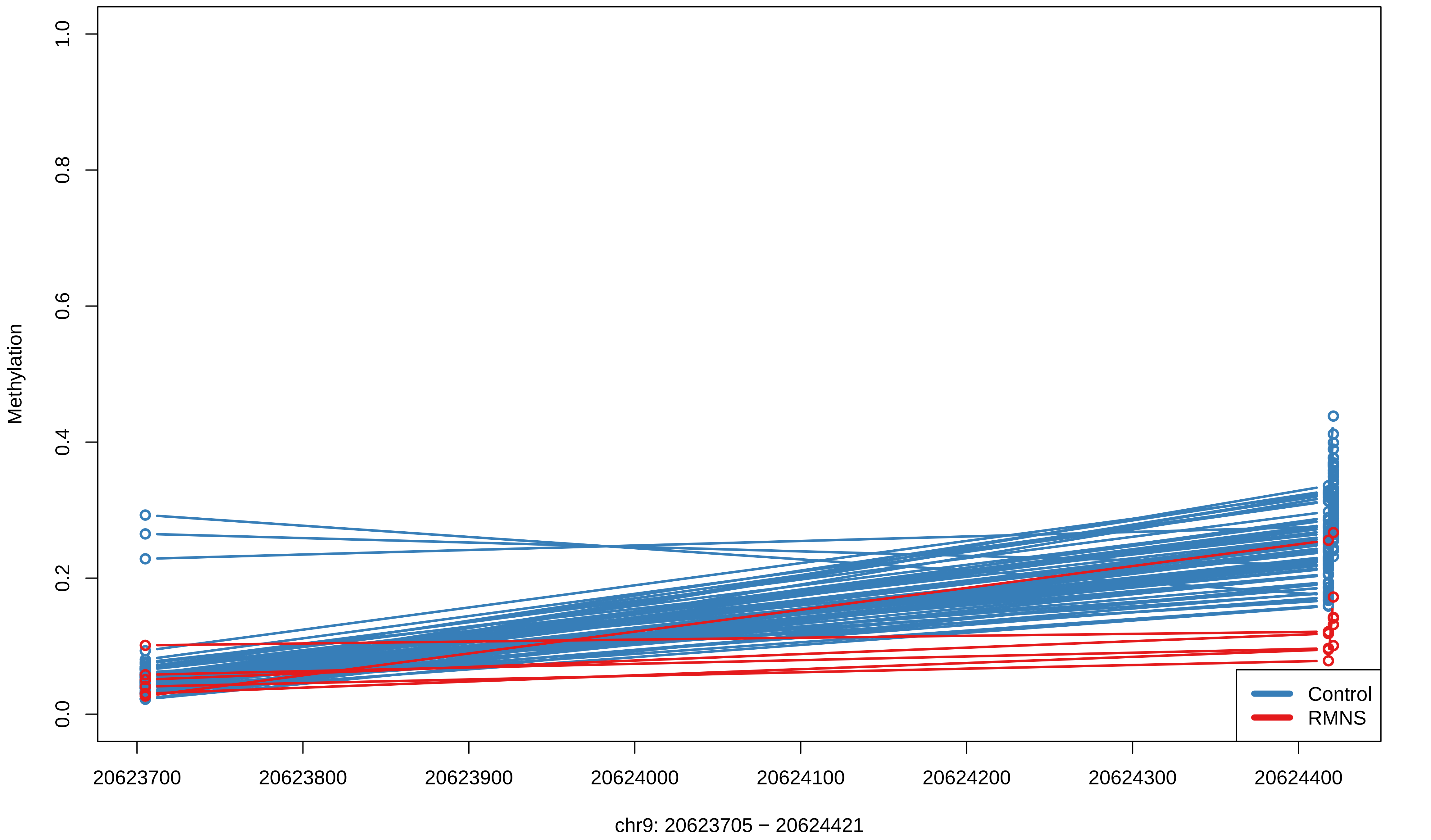

Methylation
0.0
0.2
0.4
0.6
0.8
1.0
20623700
20623800
20623900
20624000
20624100
20624200
20624300
20624400
chr9: 20623705 − 20624421
Control
RMNS

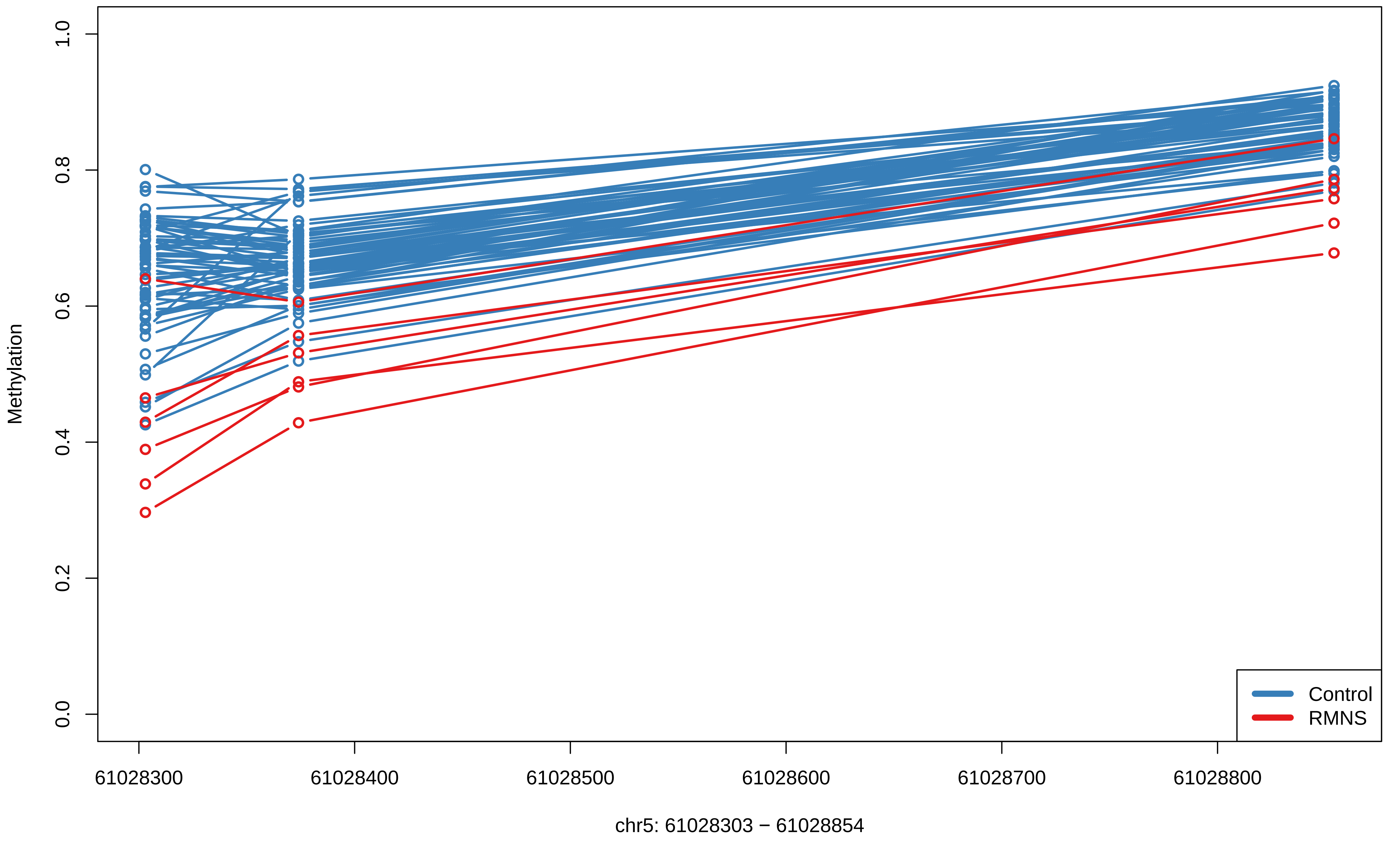

Methylation
1.0
0.8
0.6
0.4
0.2
0.0
61028300
61028400
61028500
61028600
61028700
61028800
chr5: 61028303 − 61028854
Control
RMNS

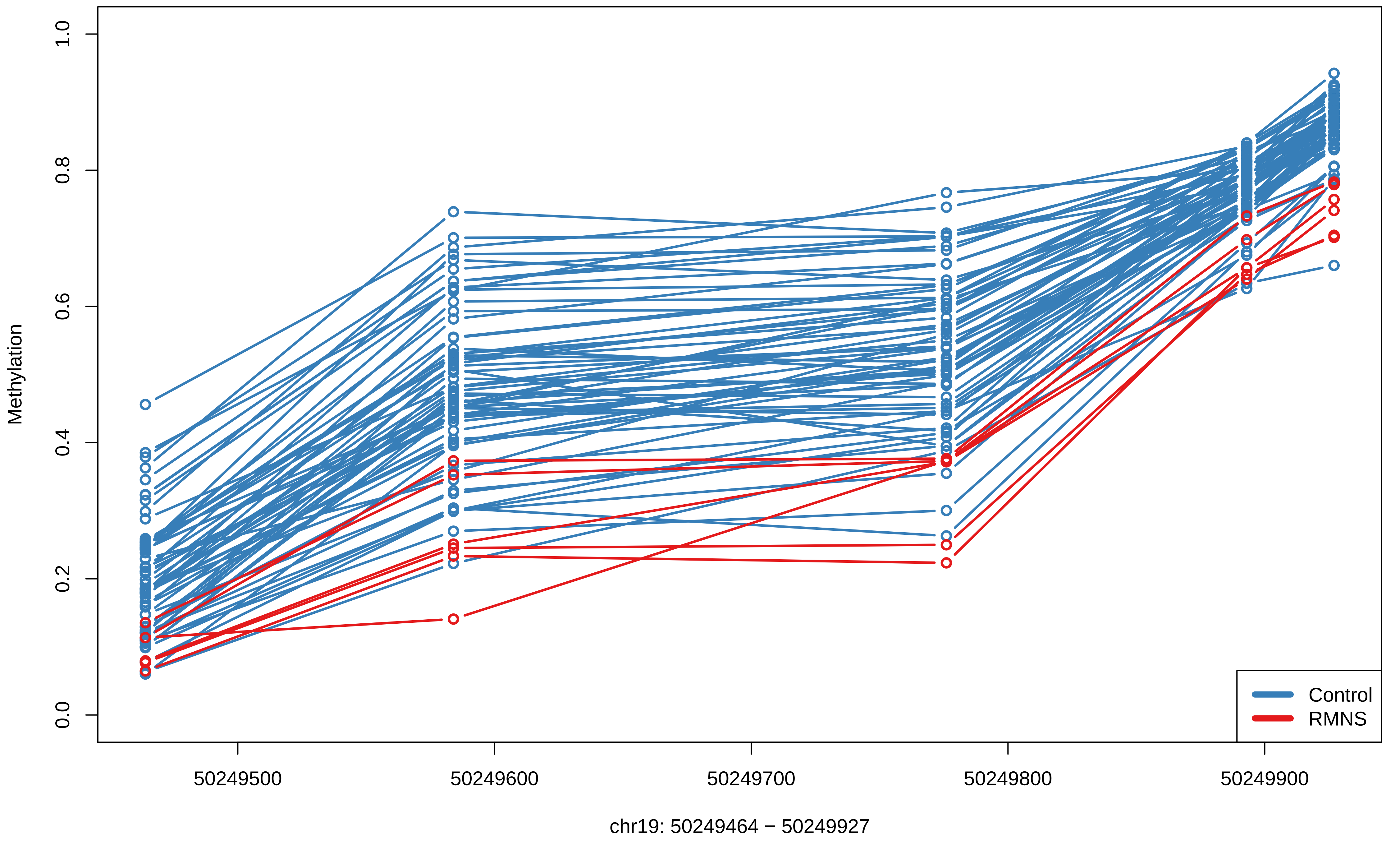

Methylation
0.0
0.2
0.4
0.6
0.8
1.0
50249500
50249600
50249700
50249800
50249900
chr19: 50249464 − 50249927
Control
RMNS

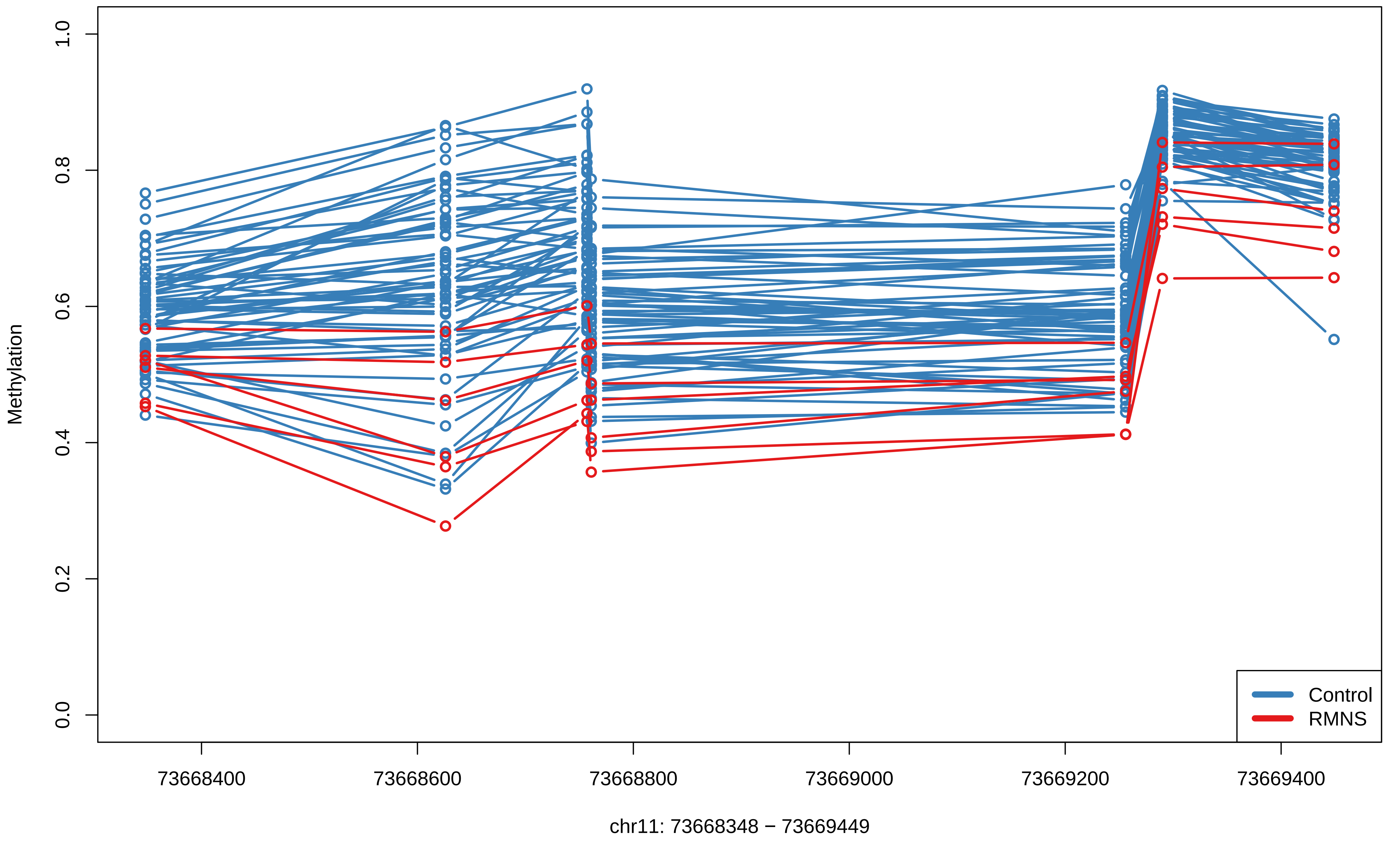
1.0
0.8
0.6
0.4
0.2
0.0
Methylation
73668400
73668600
73668800
73669000
73669200
73669400
chr11: 73668348 − 73669449
Control
RMNS

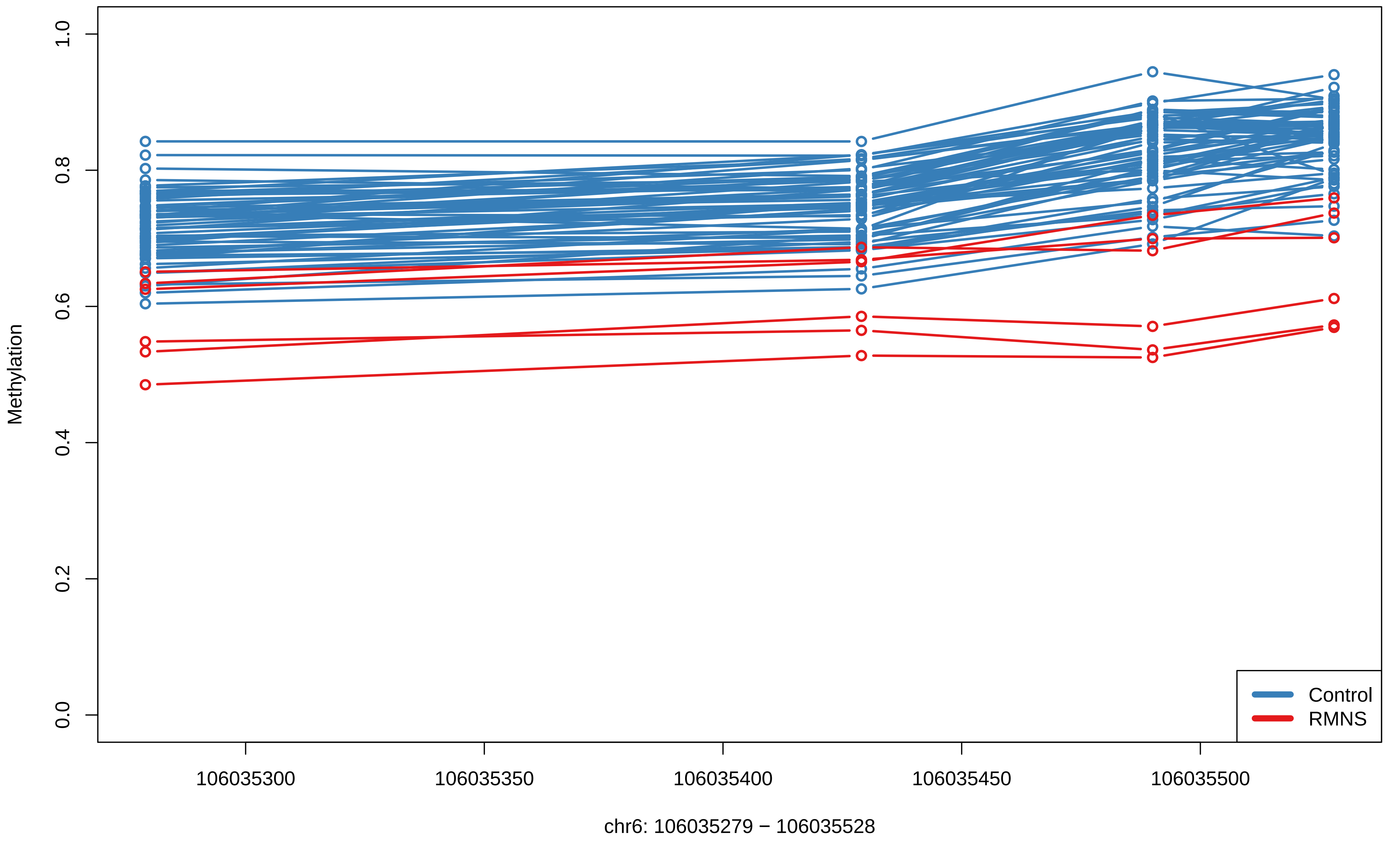

Methylation
0.0
0.2
0.4
0.6
0.8
1.0
106035300
106035350
106035400
106035450
106035500
chr6: 106035279 − 106035528
Control
RMNS

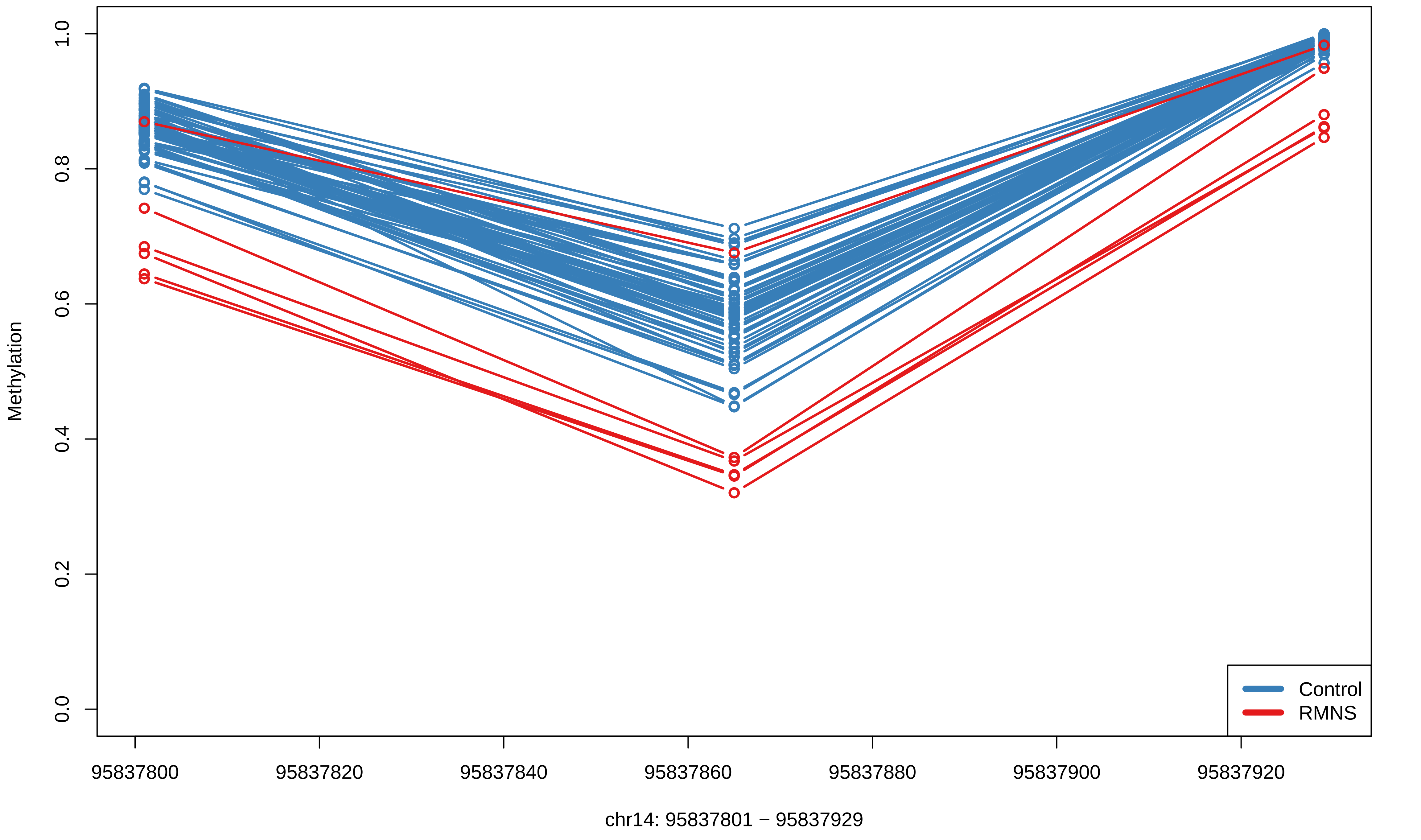

Methylation
1.0
0.8
0.6
0.4
0.2
0.0
95837800
95837820
95837840
95837860
95837880
95837900
95837920
chr14: 95837801 − 95837929
Control
RMNS

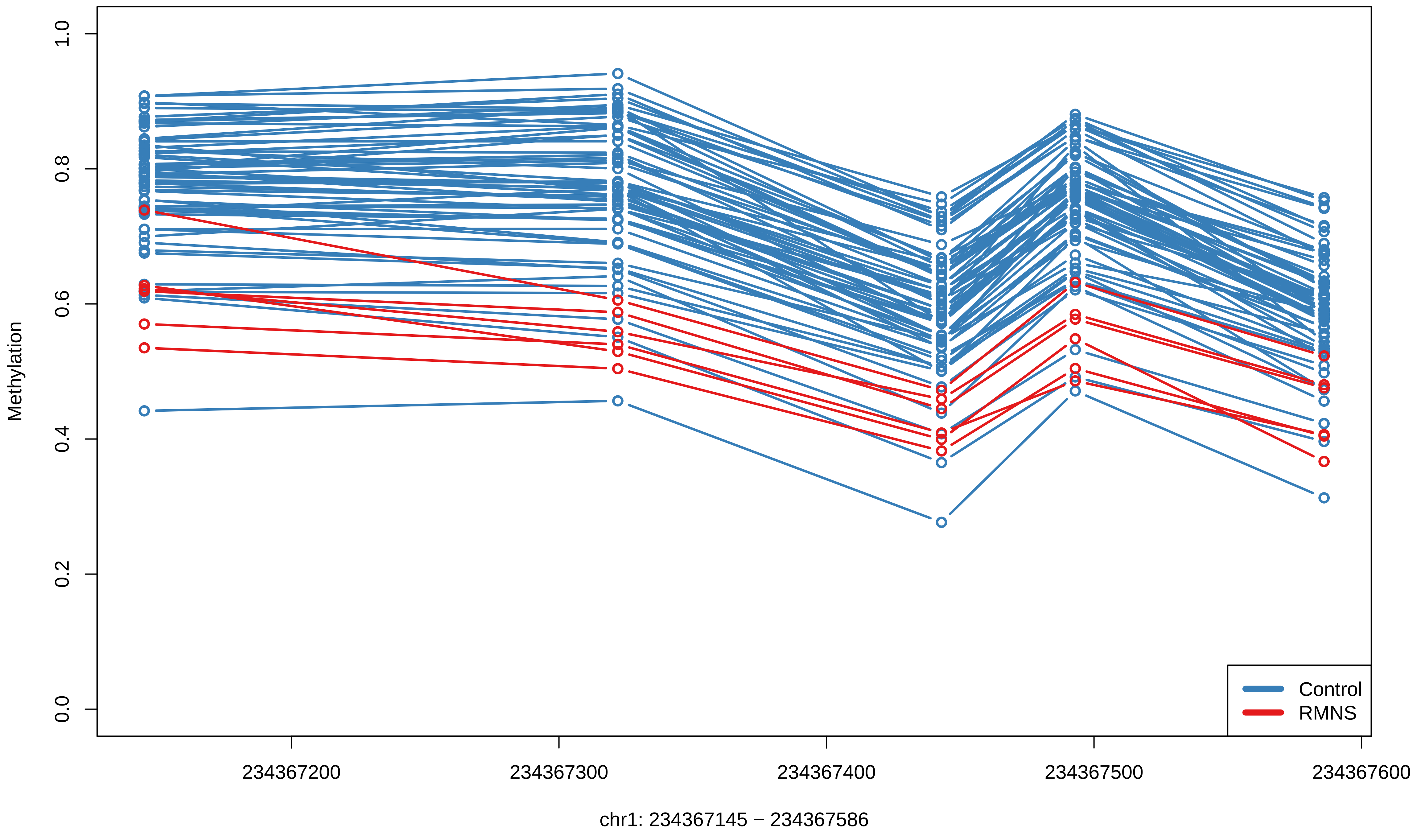

Methylation
0.0
0.2
0.4
0.6
0.8
1.0
234367200
234367300
234367400
234367500
234367600
chr1: 234367145 − 234367586
Control
RMNS

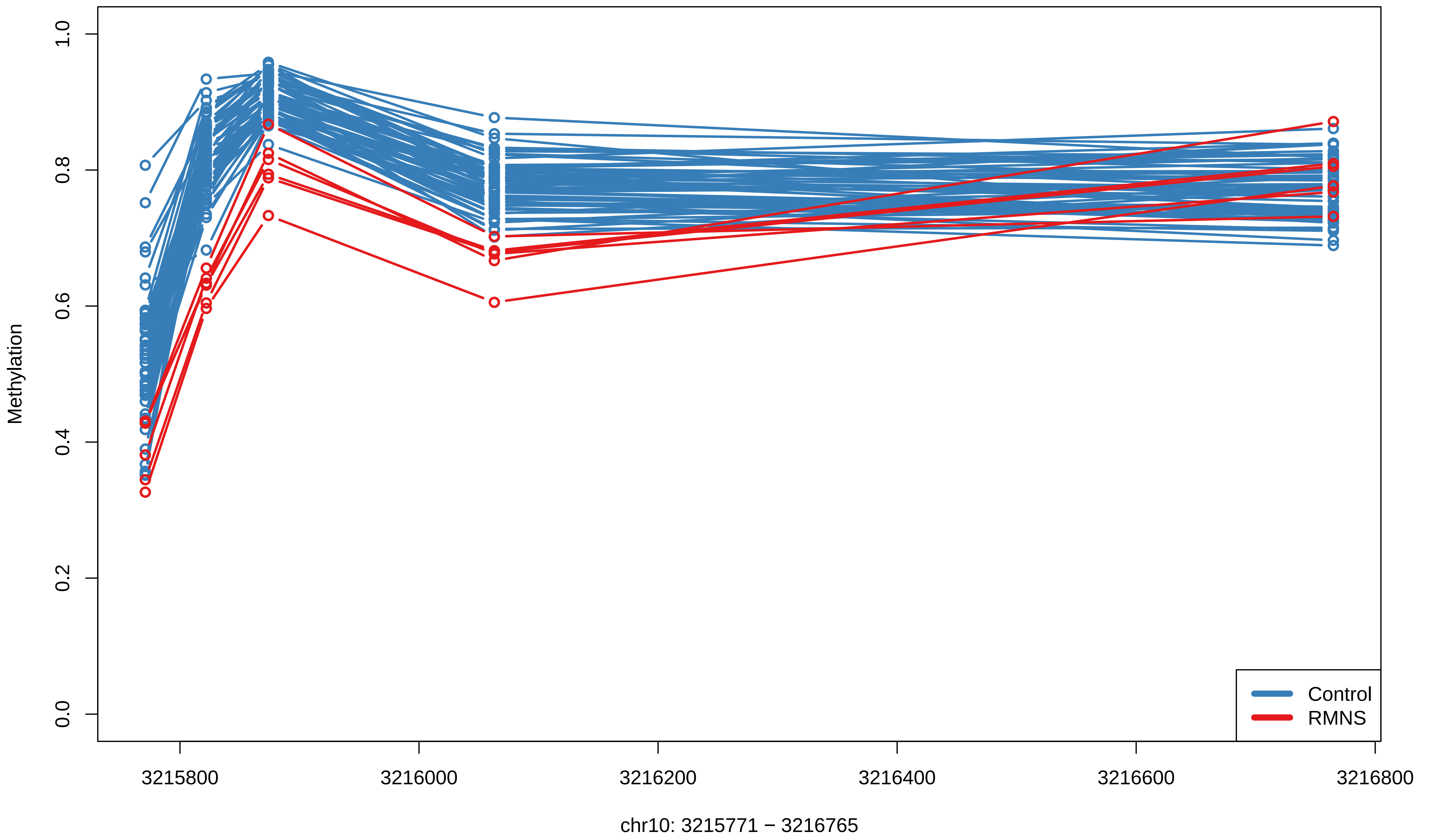

Methylation
0.0
0.2
0.4
0.6
0.8
1.0
3215800
3216000
3216200
3216400
3216600
3216800
chr10: 3215771 − 3216765
Control
RMNS

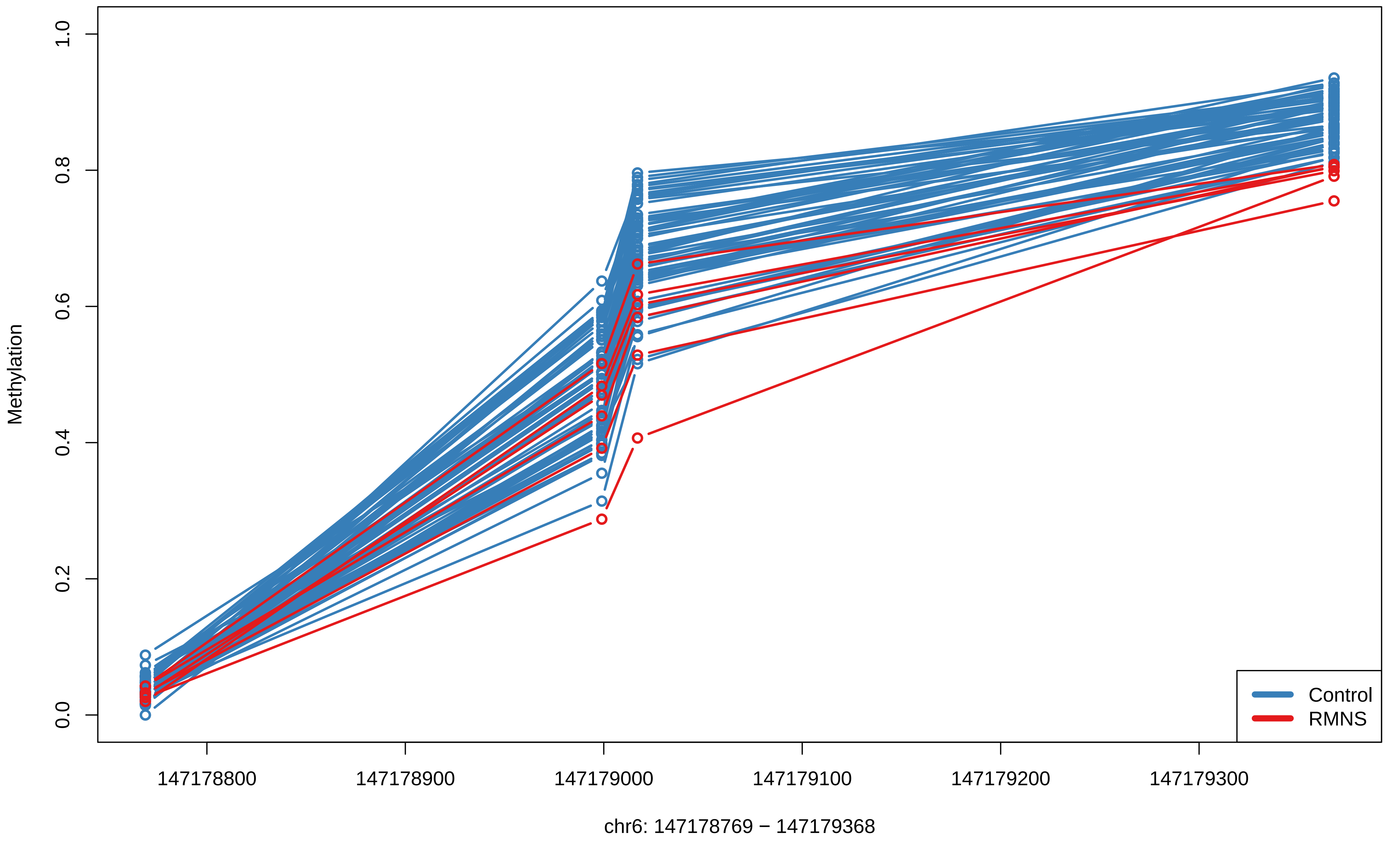

Methylation
0.0
0.2
0.4
0.6
0.8
1.0
147178800
147178900
147179000
147179100
147179200
147179300
chr6: 147178769 − 147179368
Control
RMNS

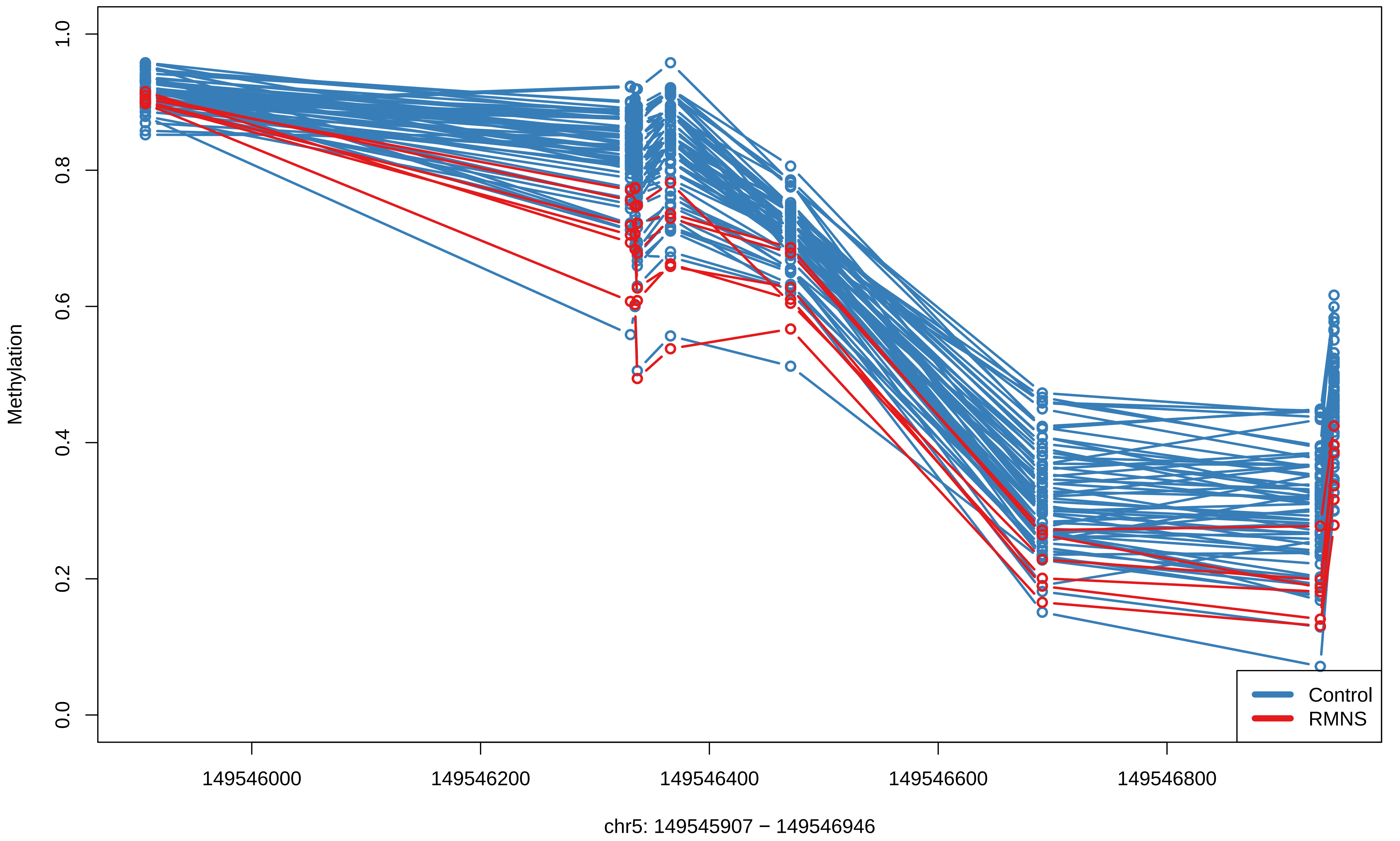

Methylation
1.0
0.8
0.6
0.4
0.2
0.0
149546000
149546200
149546400
149546600
149546800
chr5: 149545907 − 149546946
Control
RMNS

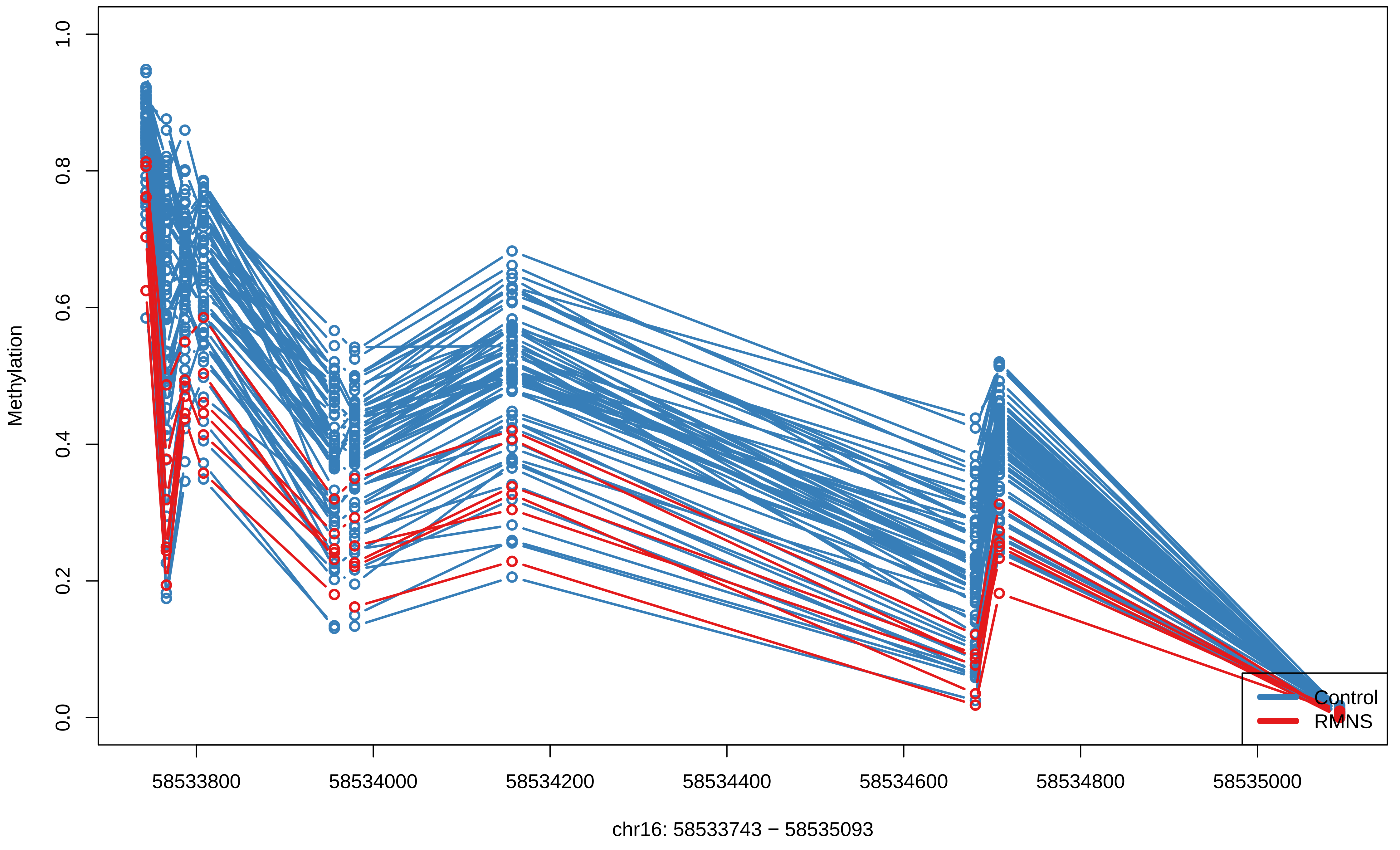

Methylation
1.0
0.8
0.6
0.4
0.2
0.0
58533800
58534000
58534200
58534400
58534600
58534800
58535000
chr16: 58533743 − 58535093
Control
RMNS

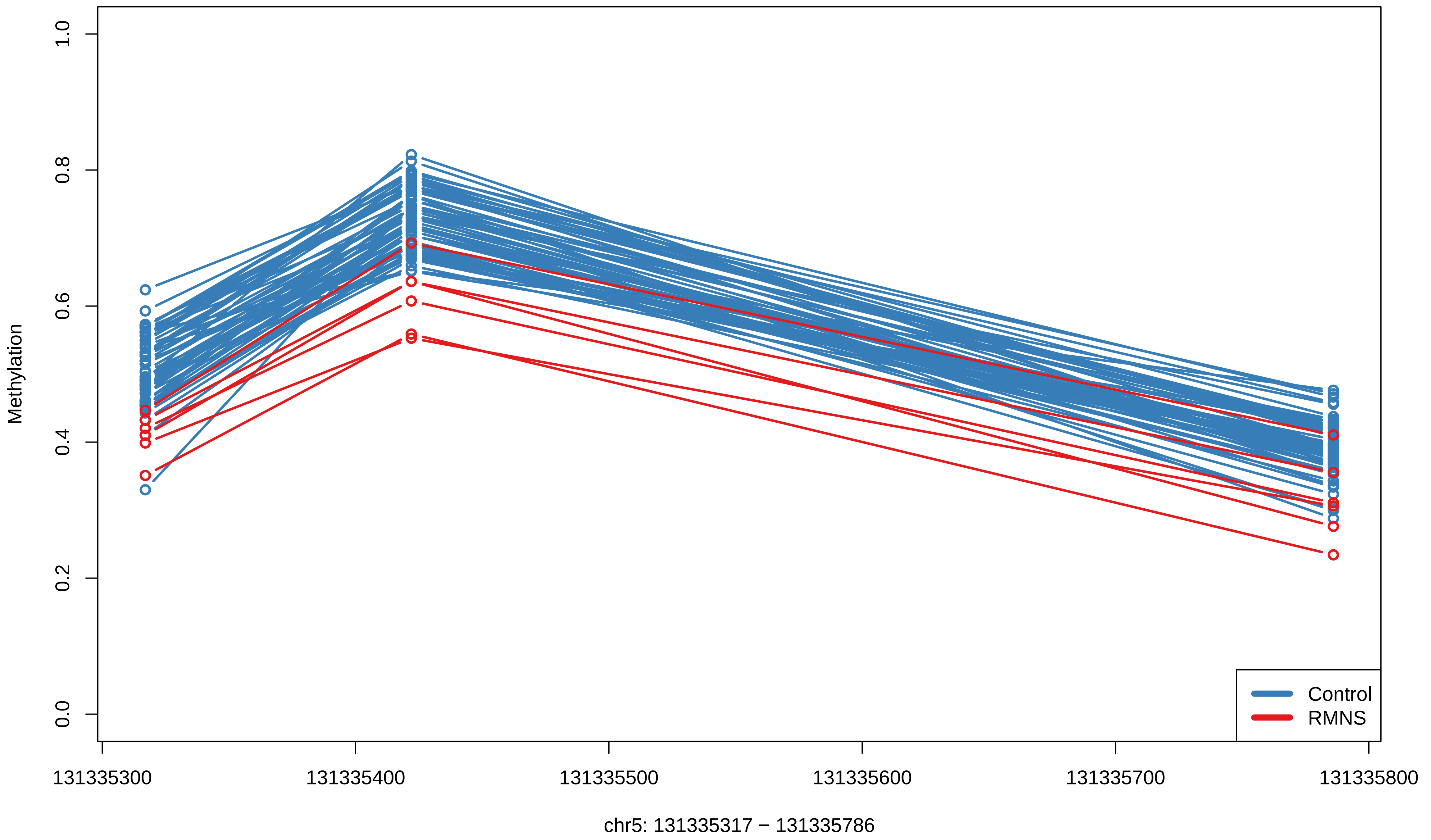

Methylation
0.0
0.2
0.4
0.6
0.8
1.0
131335300
131335400
131335500
131335600
131335700
131335800
chr5: 131335317 − 131335786
Control
RMNS

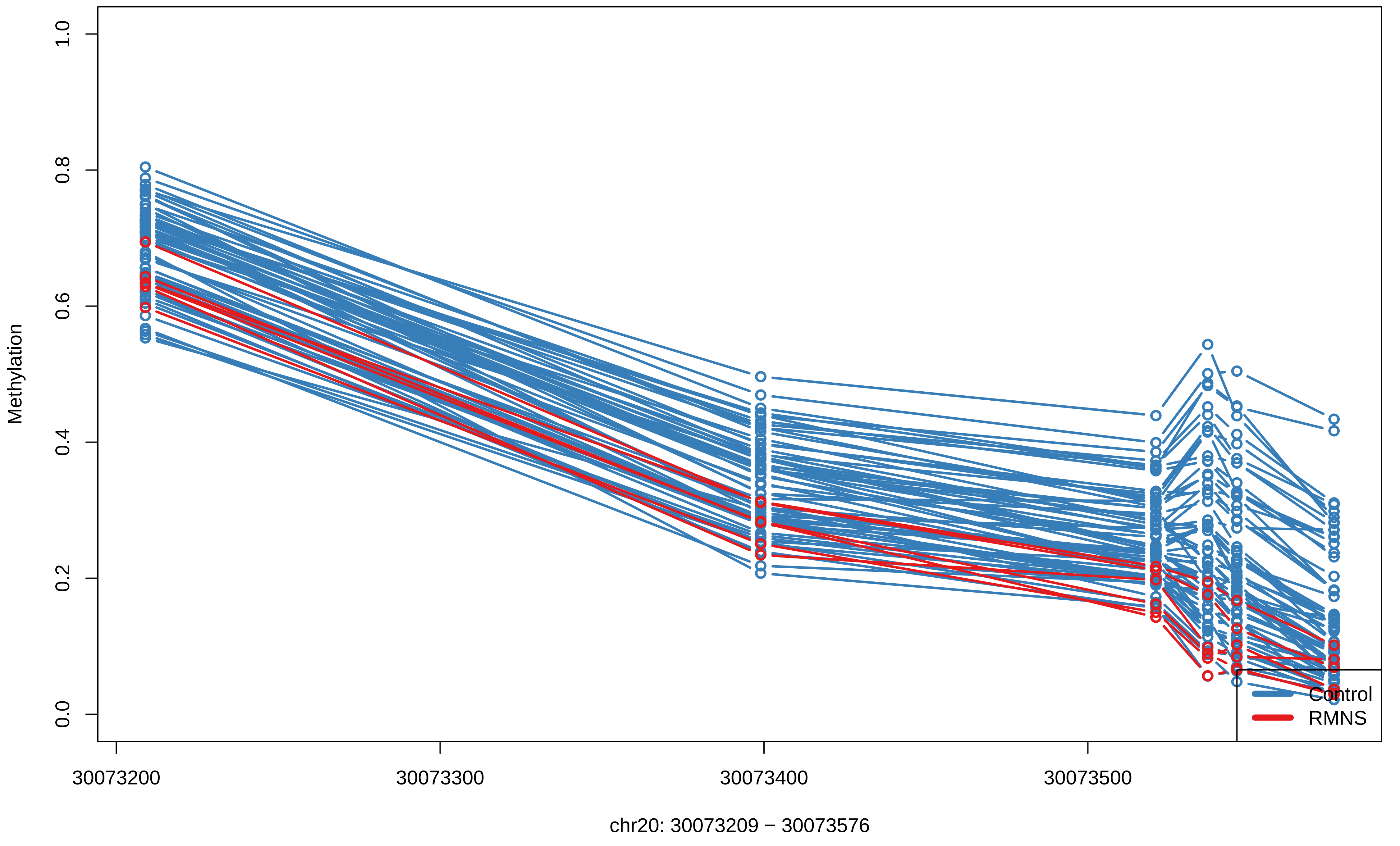

Methylation
1.0
0.8
0.6
0.4
0.2
0.0
30073200
30073300
30073400
30073500
chr20: 30073209 − 30073576
Control
RMNS

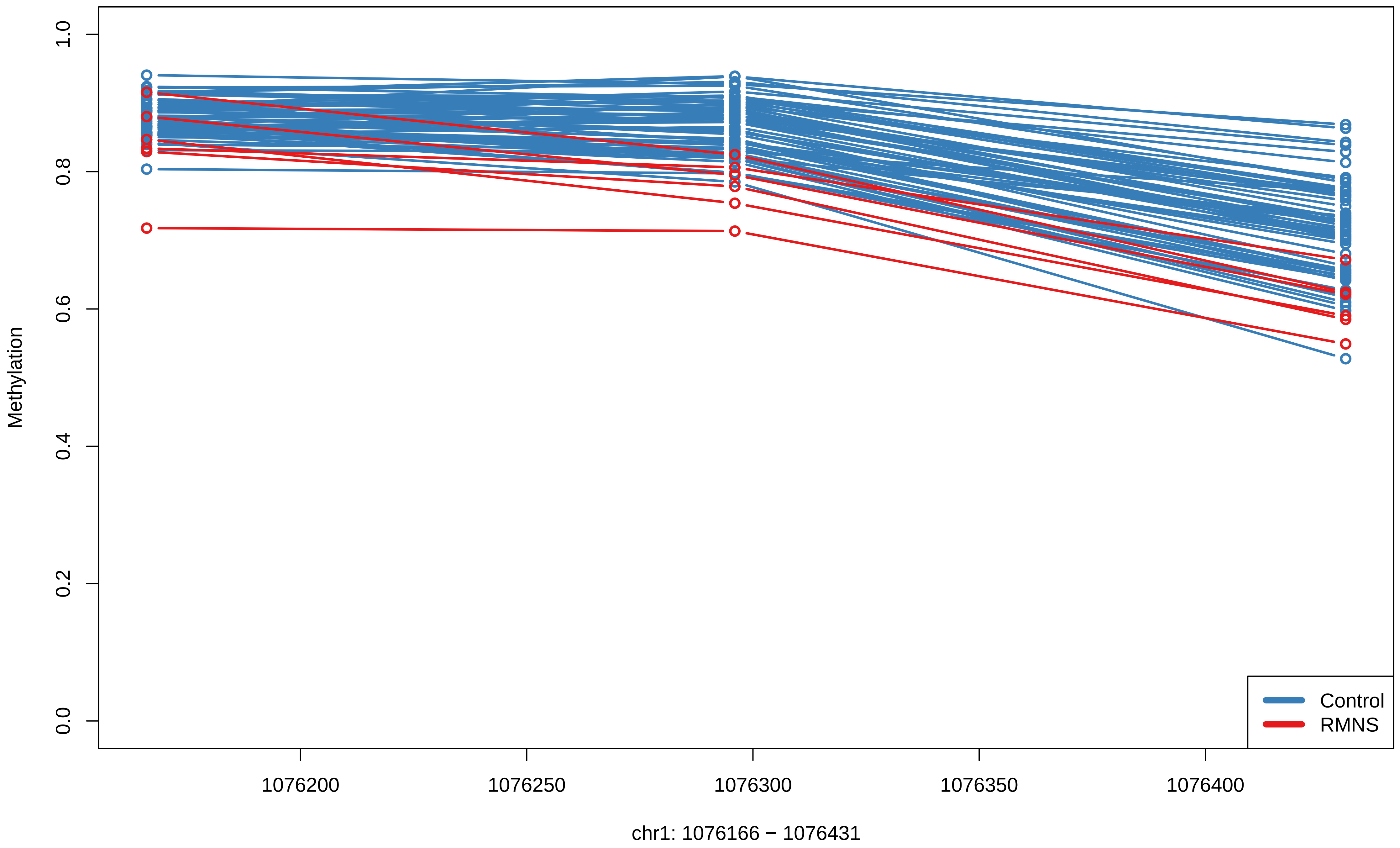

Methylation
0.0
0.2
0.4
0.6
0.8
1.0
1076200
1076250
1076300
1076350
1076400
chr1: 1076166 − 1076431
Control
RMNS

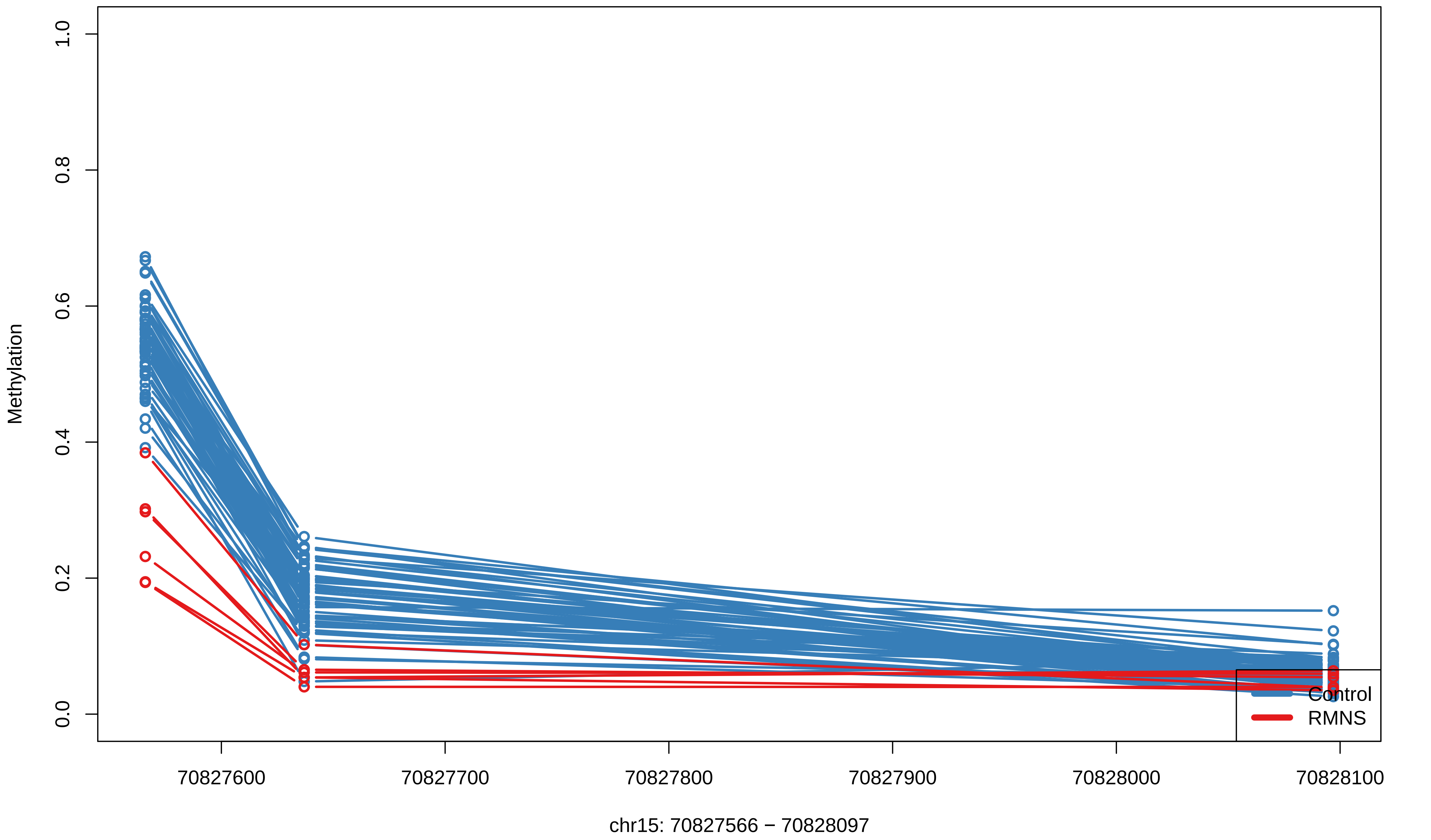

Methylation
0.0
0.2
0.4
0.6
0.8
1.0
70827600
70827700
70827800
70827900
70828000
70828100
Control
RMNS
chr15: 70827566 − 70828097

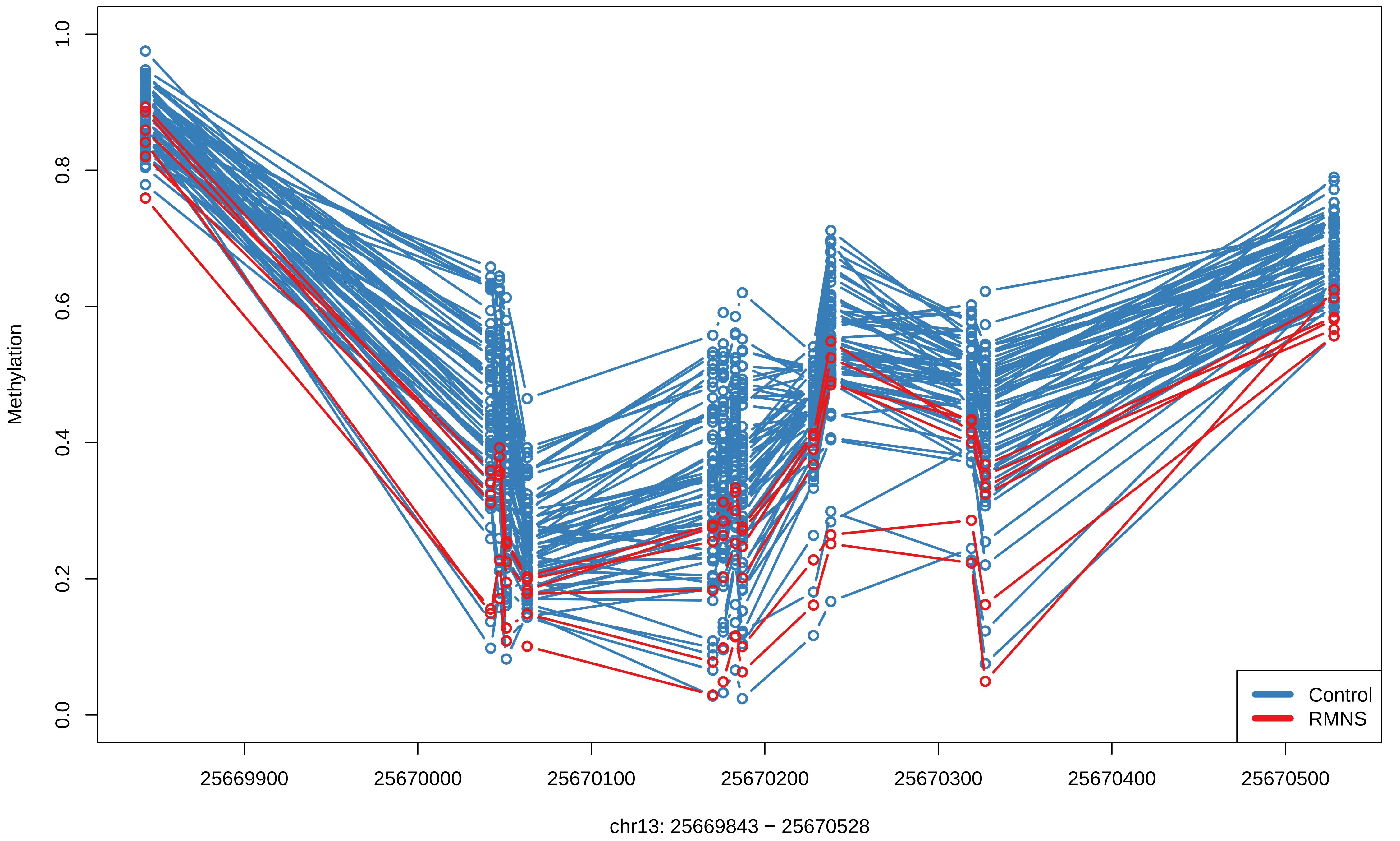

Methylation
0.0
0.2
0.4
0.6
0.8
1.0
25669900
25670000
25670100
25670200
25670300
25670400
25670500
chr13: 25669843 − 25670528
Control
RMNS

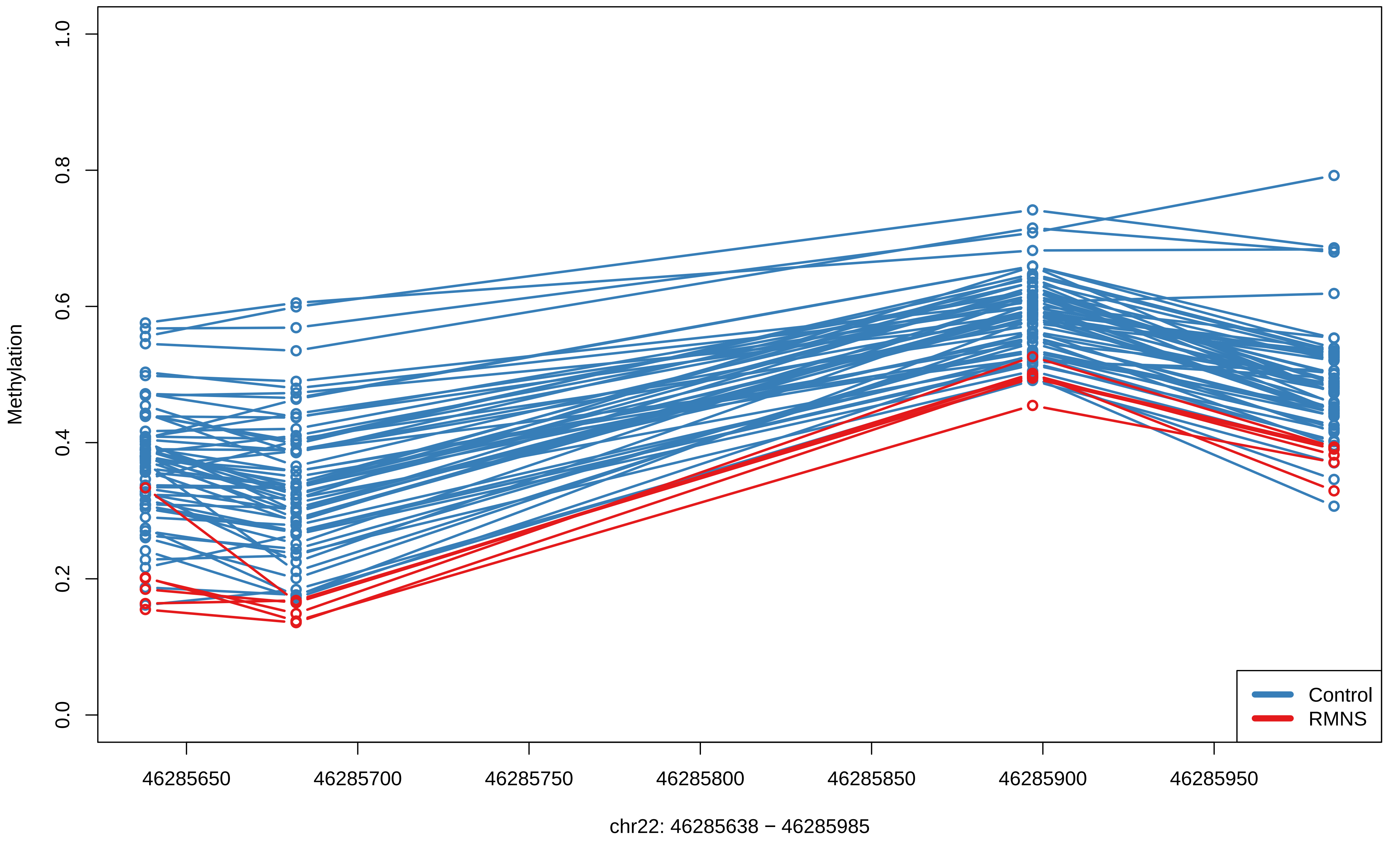

Methylation
0.0
0.2
0.4
0.6
0.8
1.0
46285650
46285700
46285750
46285800
46285850
46285900
46285950
chr22: 46285638 − 46285985
Control
RMNS

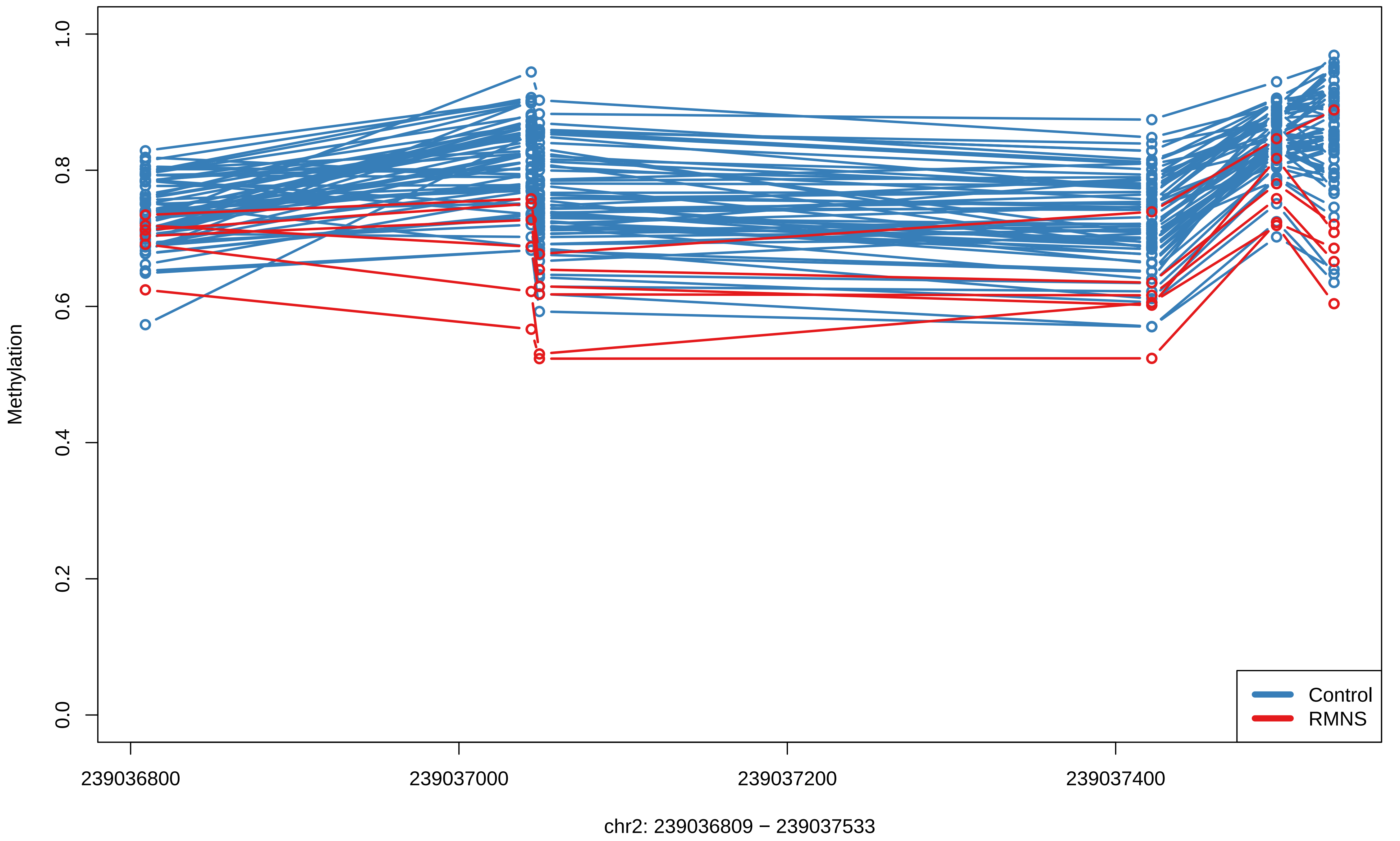

Methylation
0.0
0.2
0.4
0.6
0.8
1.0
239036800
239037000
239037200
239037400
chr2: 239036809 − 239037533
Control
RMNS

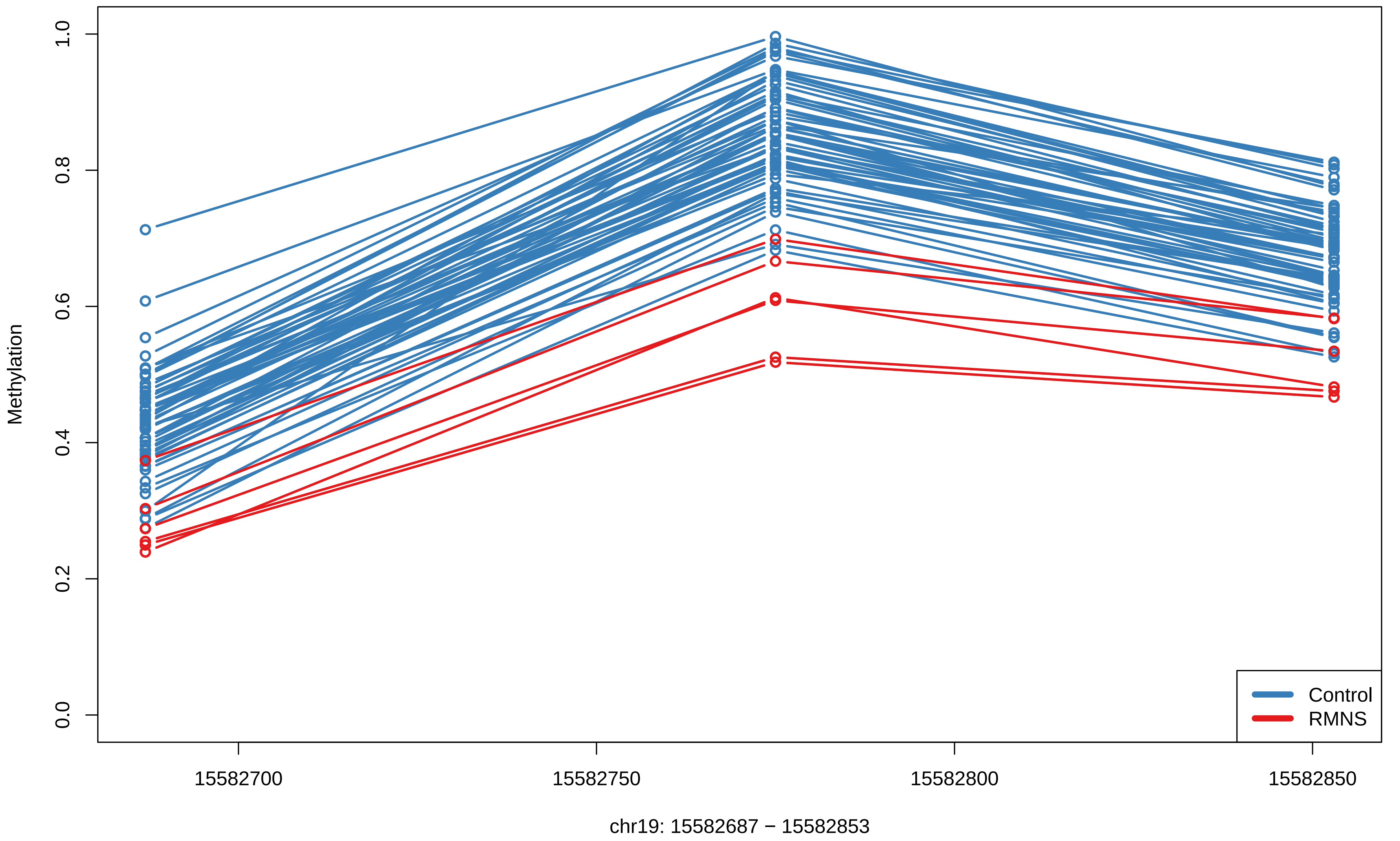

1.0
0.8
0.6
0.4
0.2
0.0
Methylation
15582700
15582750
15582800
15582850
chr19: 15582687 − 15582853
Control
RMNS

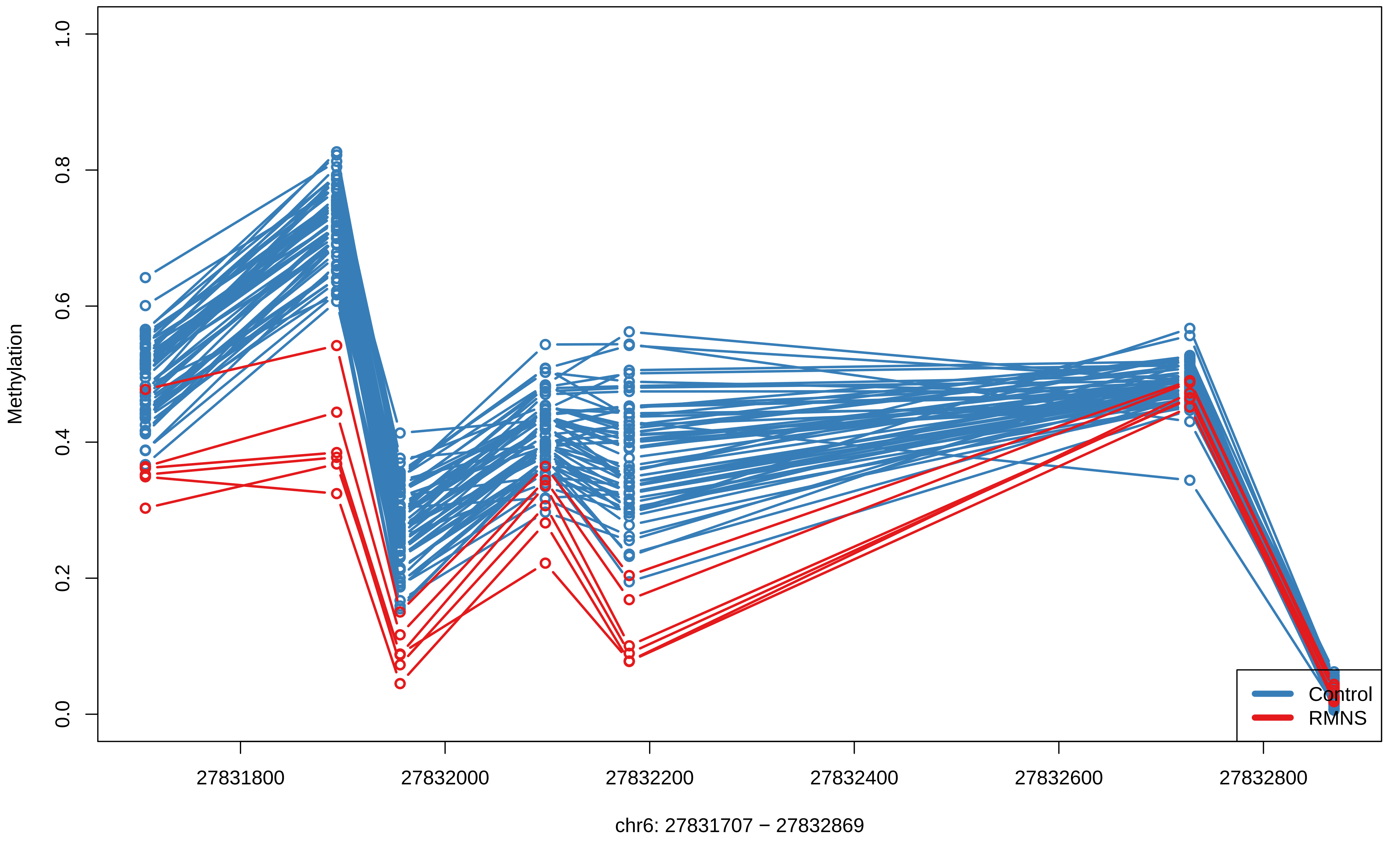

Methylation
0.0
0.2
0.4
0.6
0.8
1.0
27831800
27832000
27832200
27832400
27832600
27832800
chr6: 27831707 − 27832869
Control
RMNS

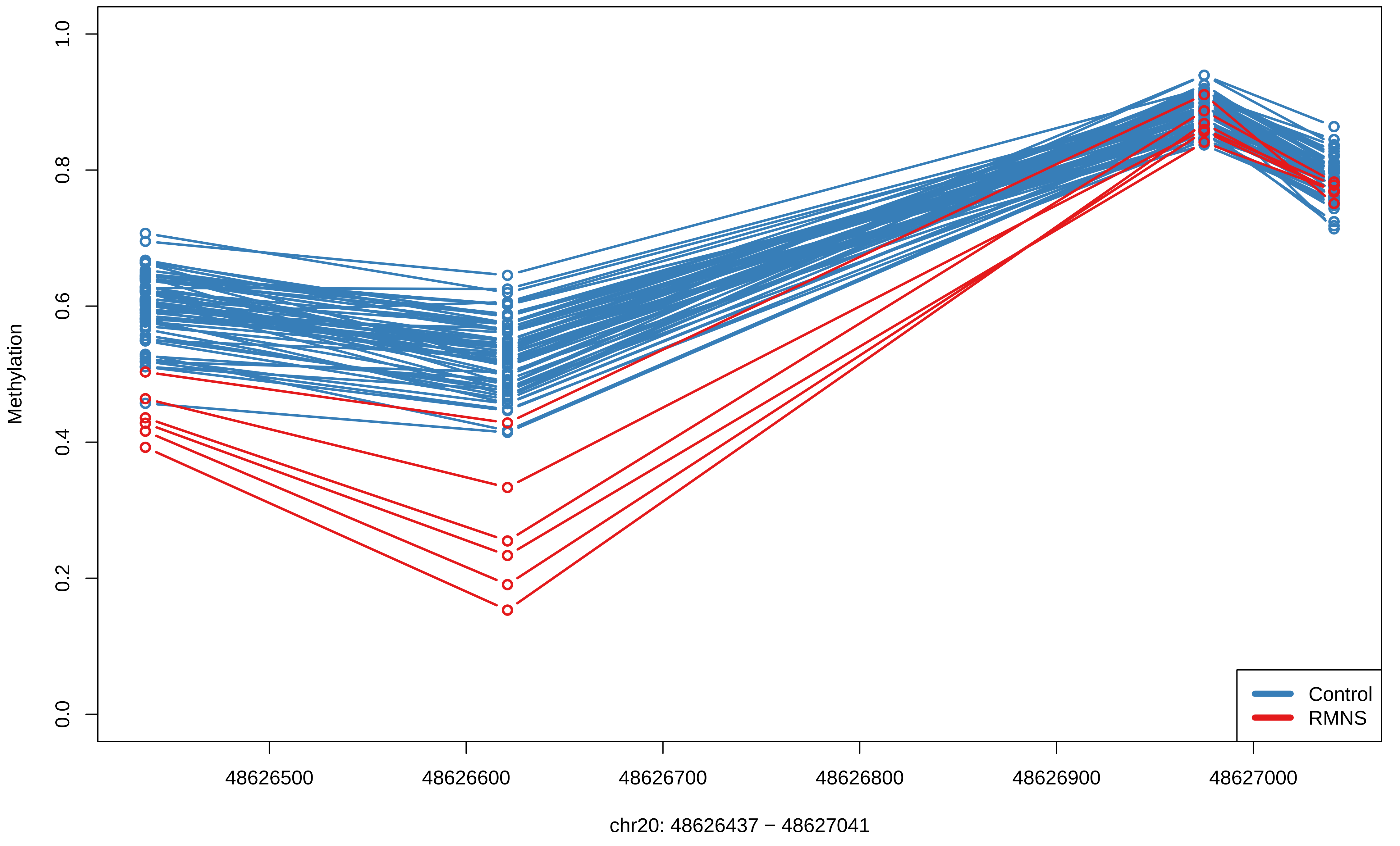

Methylation
0.0
0.2
0.4
0.6
0.8
1.0
48626500
48626600
48626700
48626800
48626900
48627000
chr20: 48626437 − 48627041
Control
RMNS

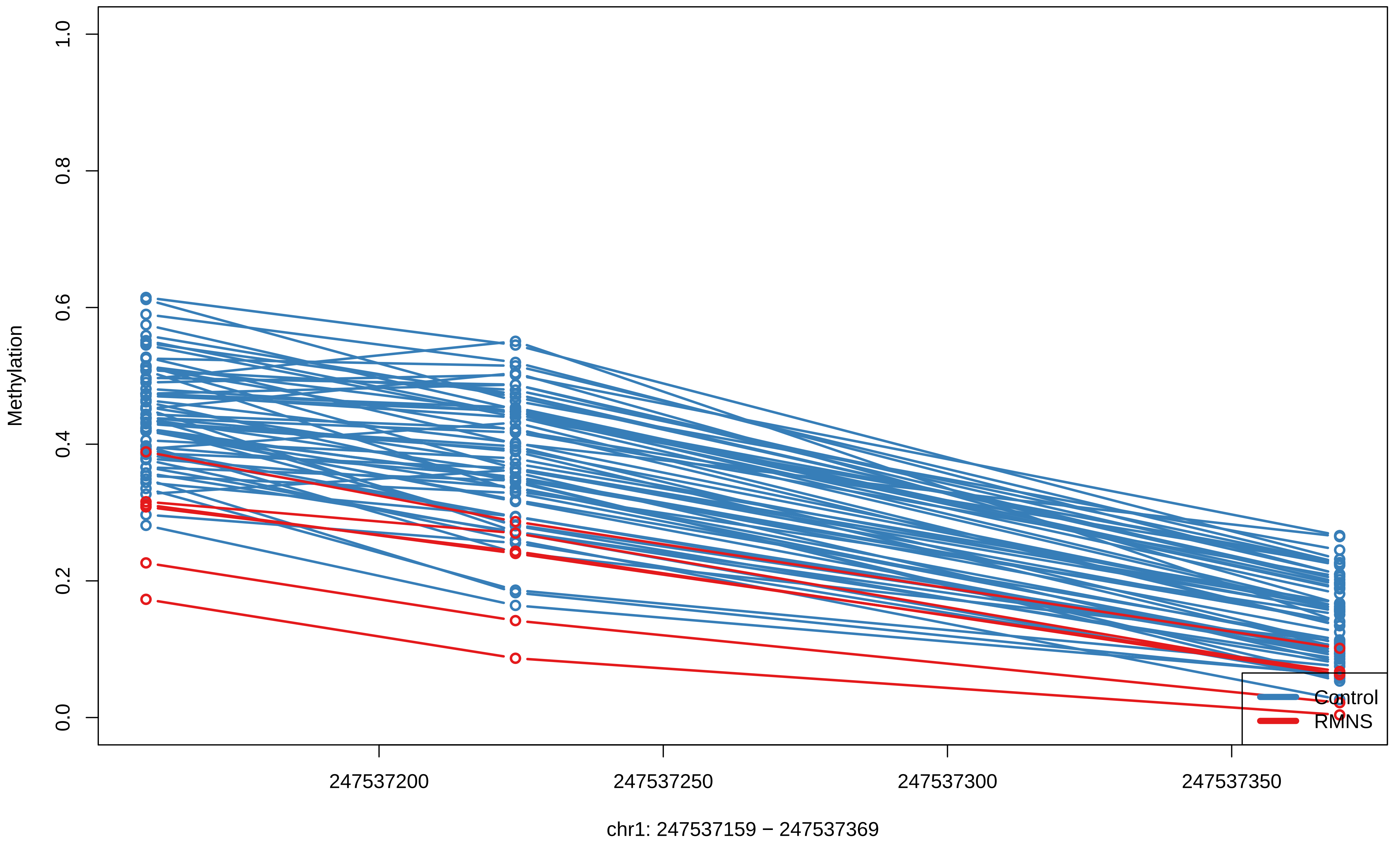

Methylation
0.0
0.2
0.4
0.6
0.8
1.0
247537200
247537250
247537300
247537350
chr1: 247537159 − 247537369
Control
RMNS

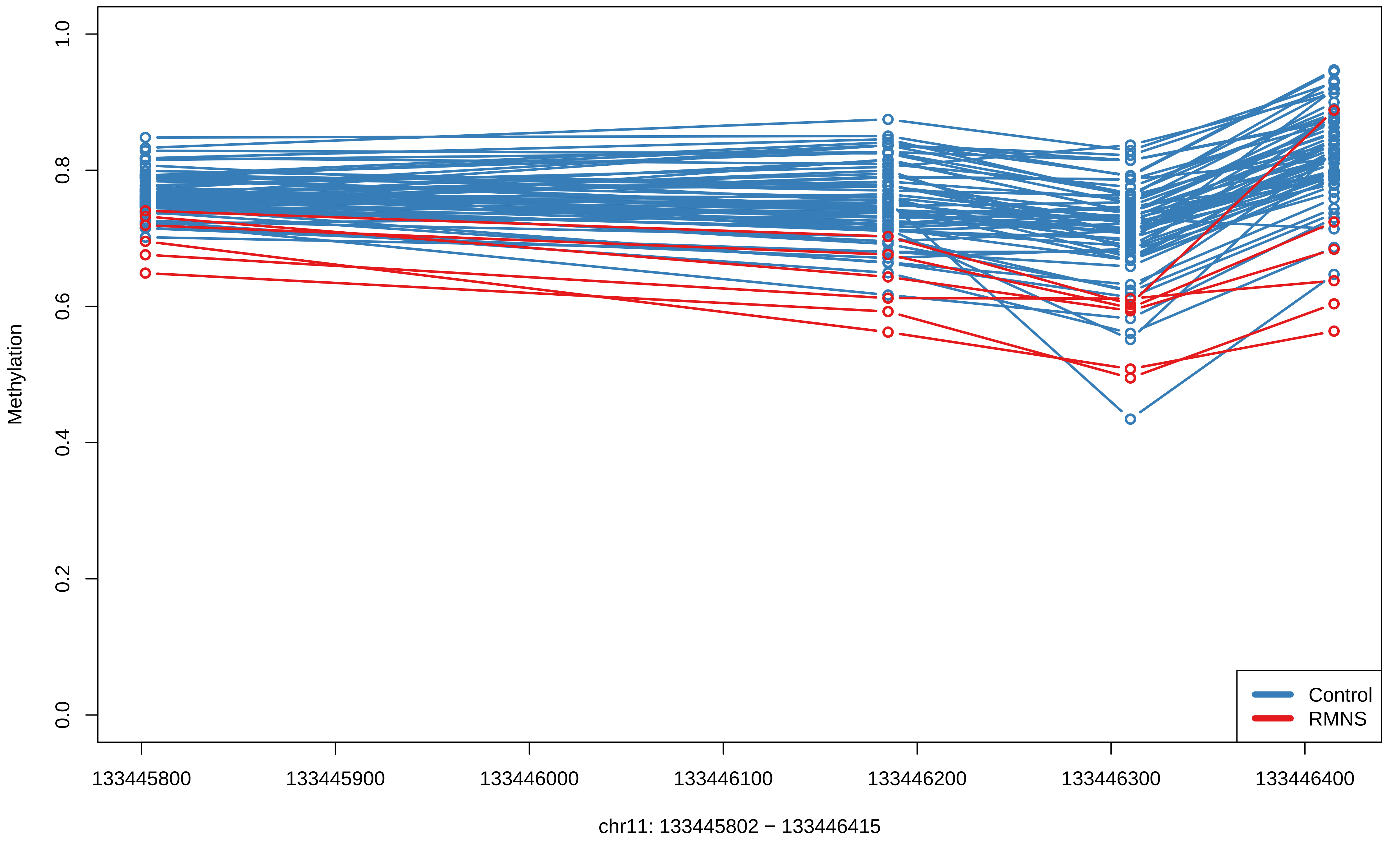

Methylation
1.0
0.8
0.6
0.4
0.2
0.0
Control
RMNS
133445800
133445900
133446000
133446100
133446200
133446300
133446400
chr11: 133445802 − 133446415

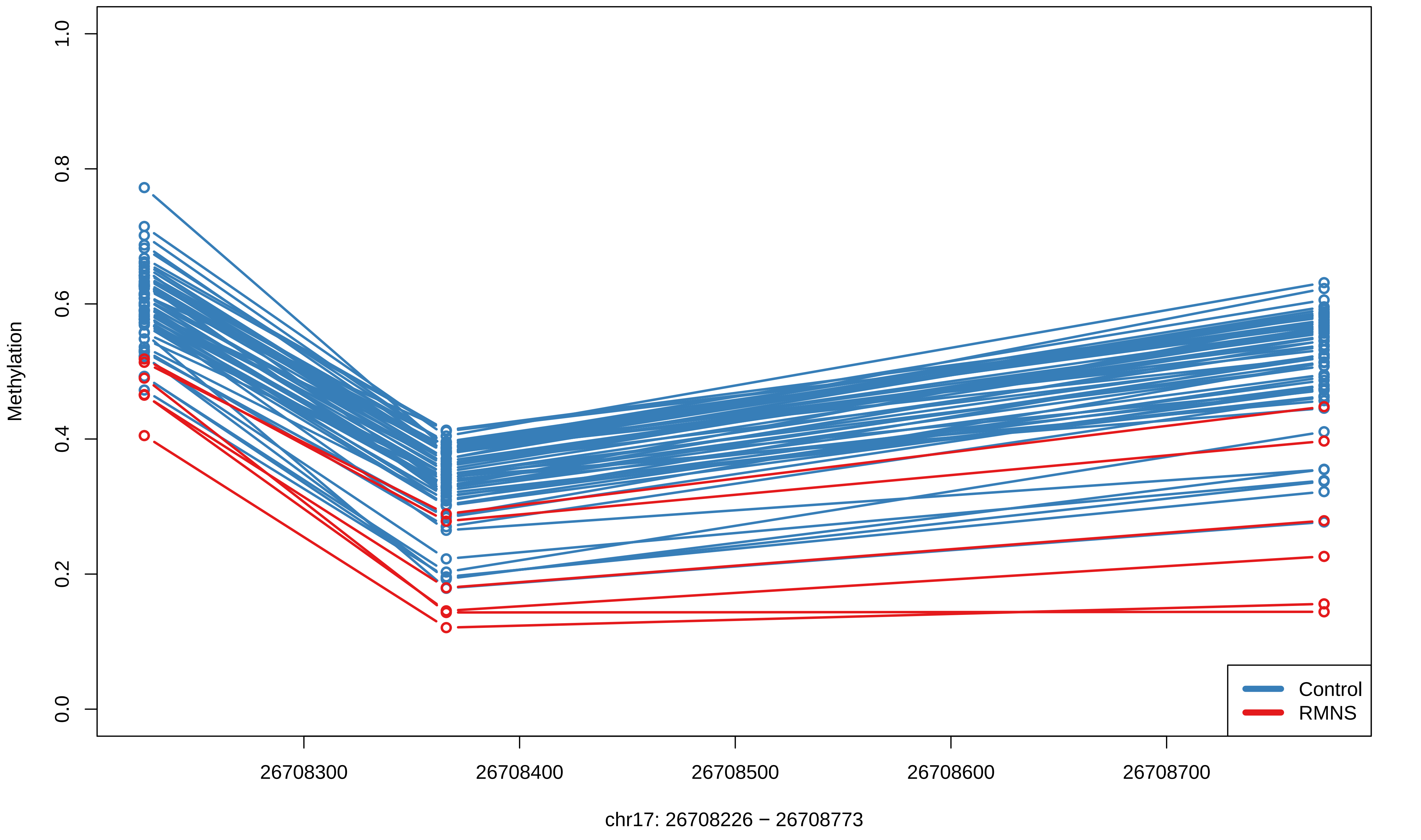

Methylation
1.0
0.8
0.6
0.4
0.2
0.0
26708300
26708400
26708500
26708600
26708700
chr17: 26708226 − 26708773
Control
RMNS

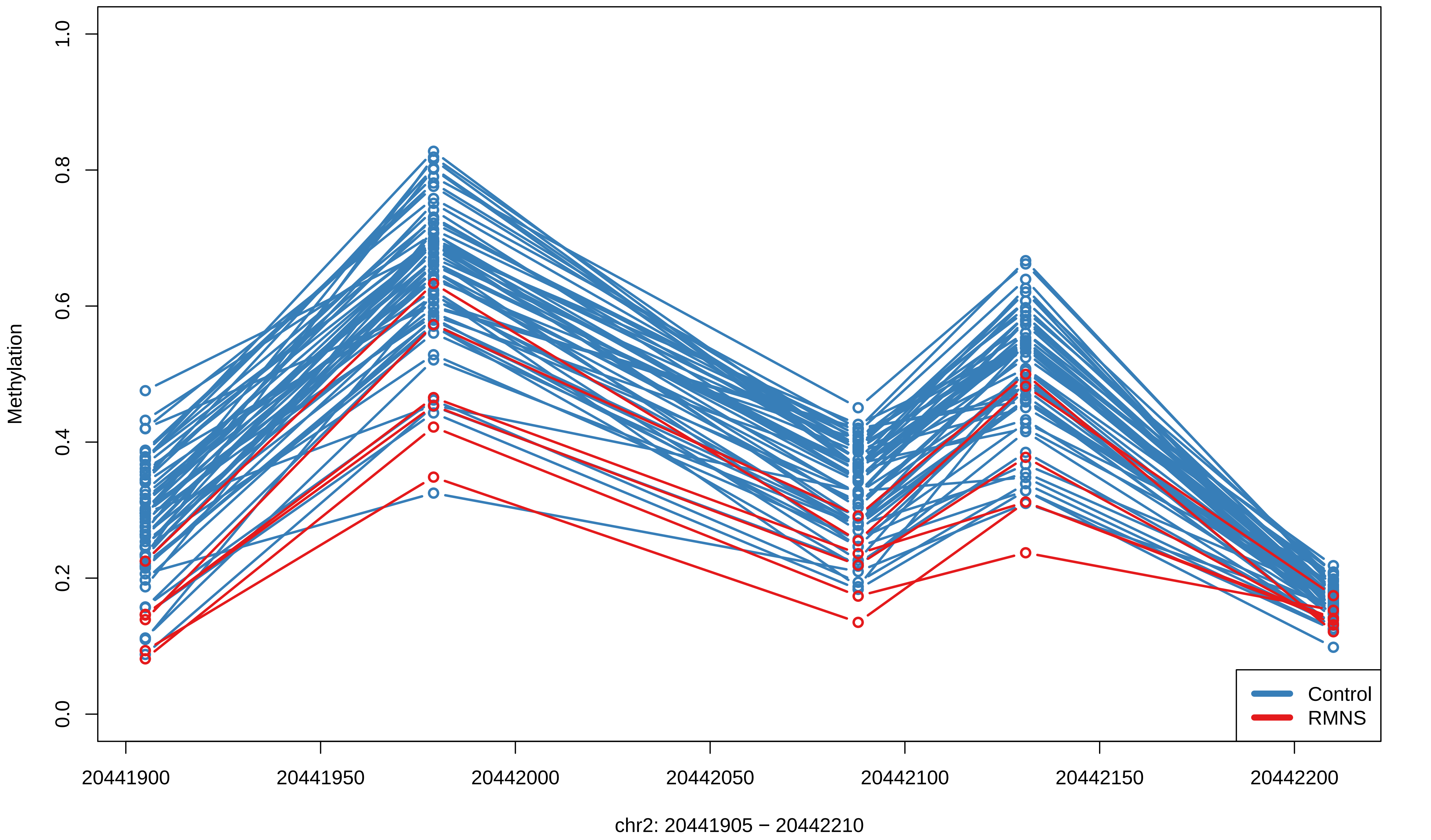

Methylation
0.0
0.2
0.4
0.6
0.8
1.0
20441900
20441950
20442000
20442050
20442100
20442150
20442200
chr2: 20441905 − 20442210
Control
RMNS

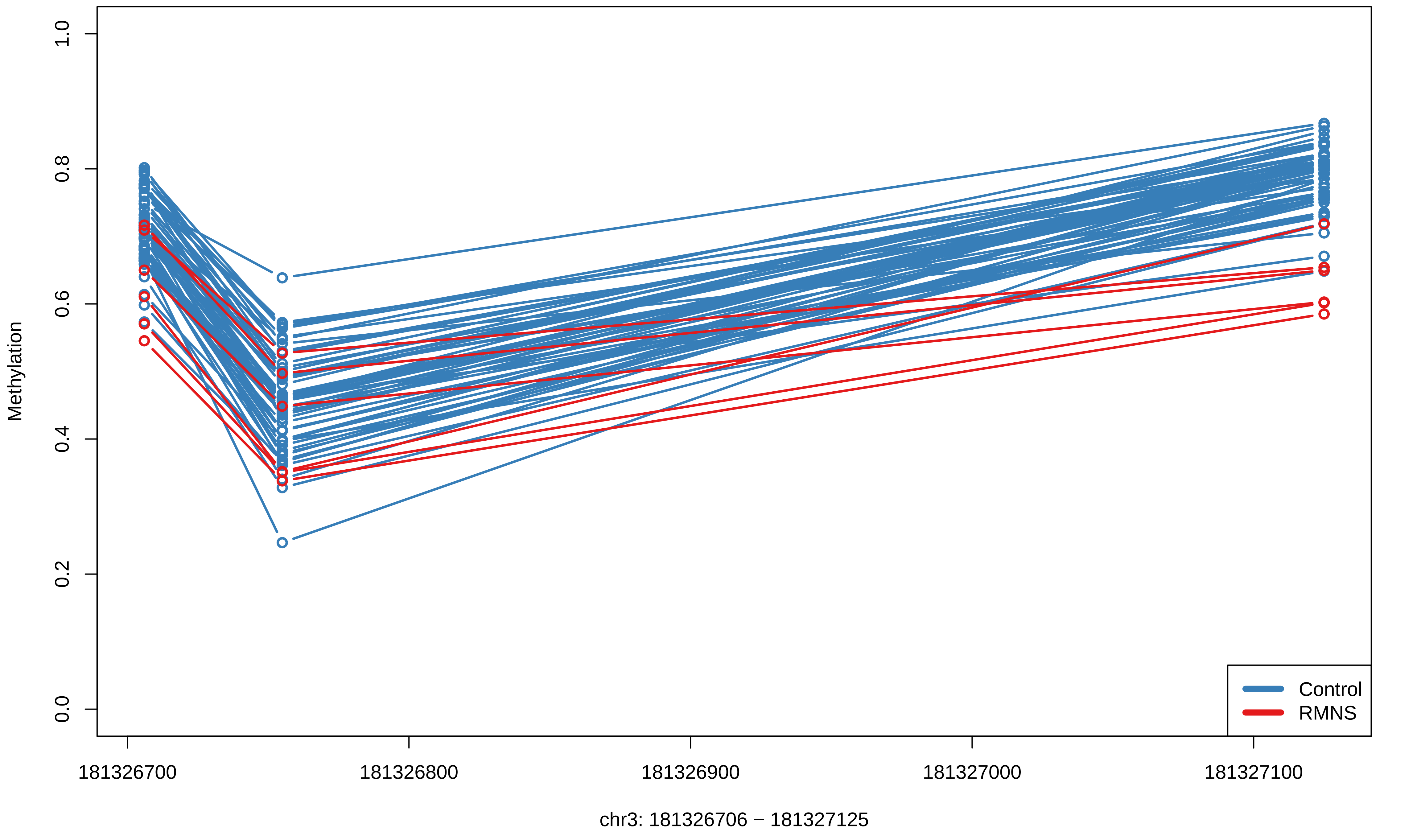

Methylation
0.0
0.2
0.4
0.6
0.8
1.0
181326700
181326800
181326900
181327000
181327100
chr3: 181326706 − 181327125
Control
RMNS

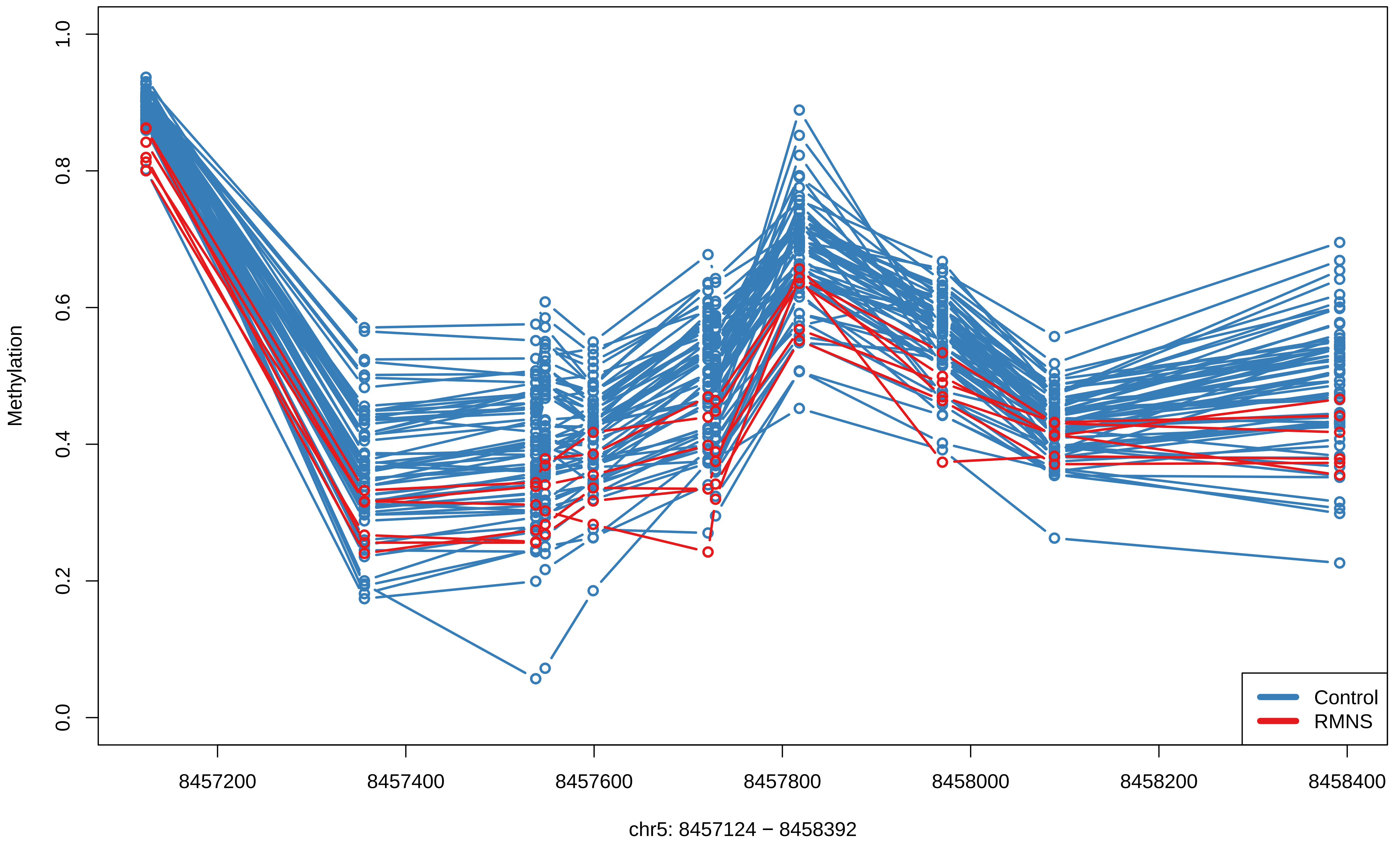

Methylation
0.0
0.2
0.4
0.6
0.8
1.0
8457200
8457400
8457600
8457800
8458000
8458200
8458400
chr5: 8457124 − 8458392
Control
RMNS

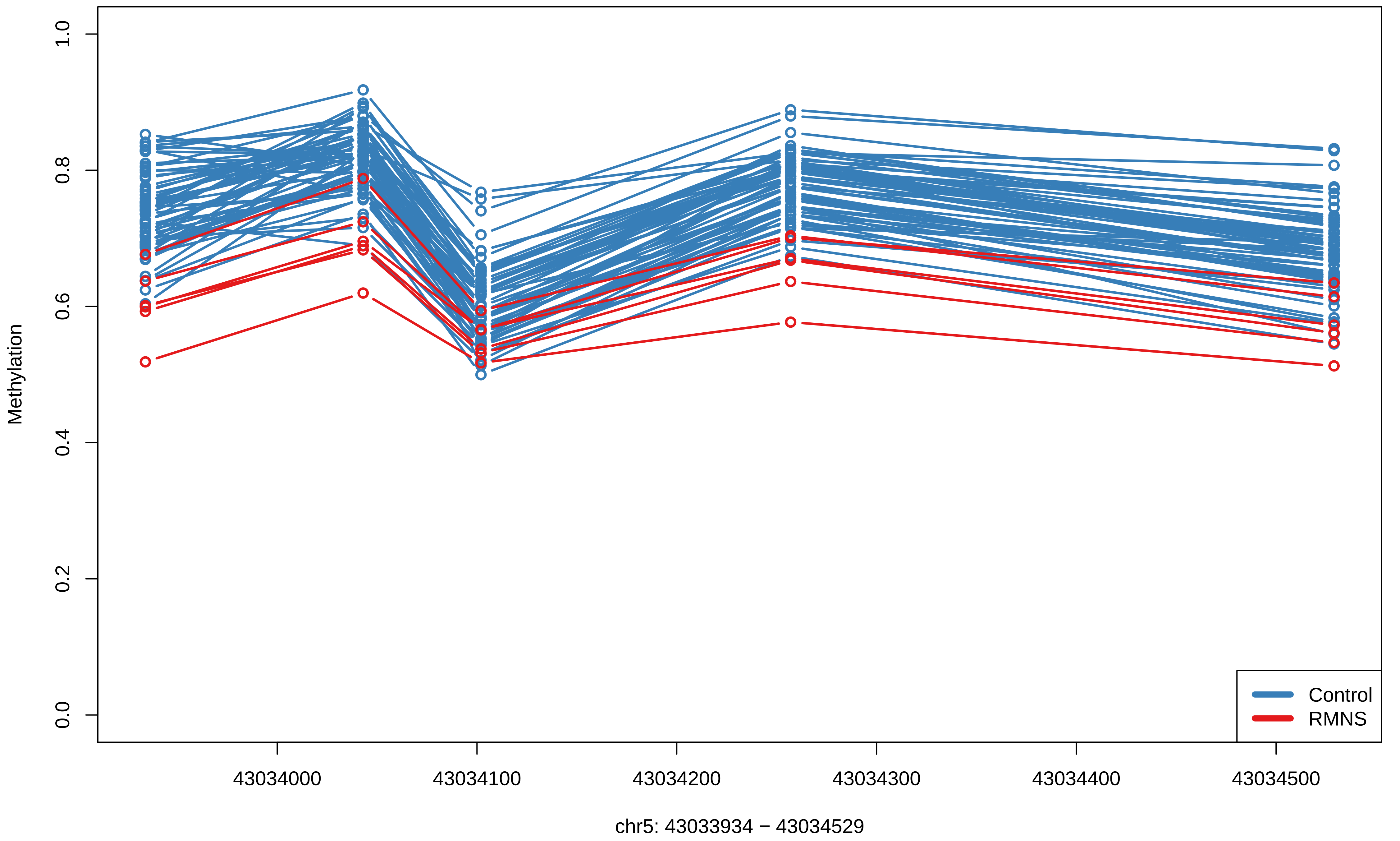

Methylation
1.0
0.8
0.6
0.4
0.2
0.0
43034000
43034100
43034200
43034300
43034400
43034500
chr5: 43033934 − 43034529
Control
RMNS

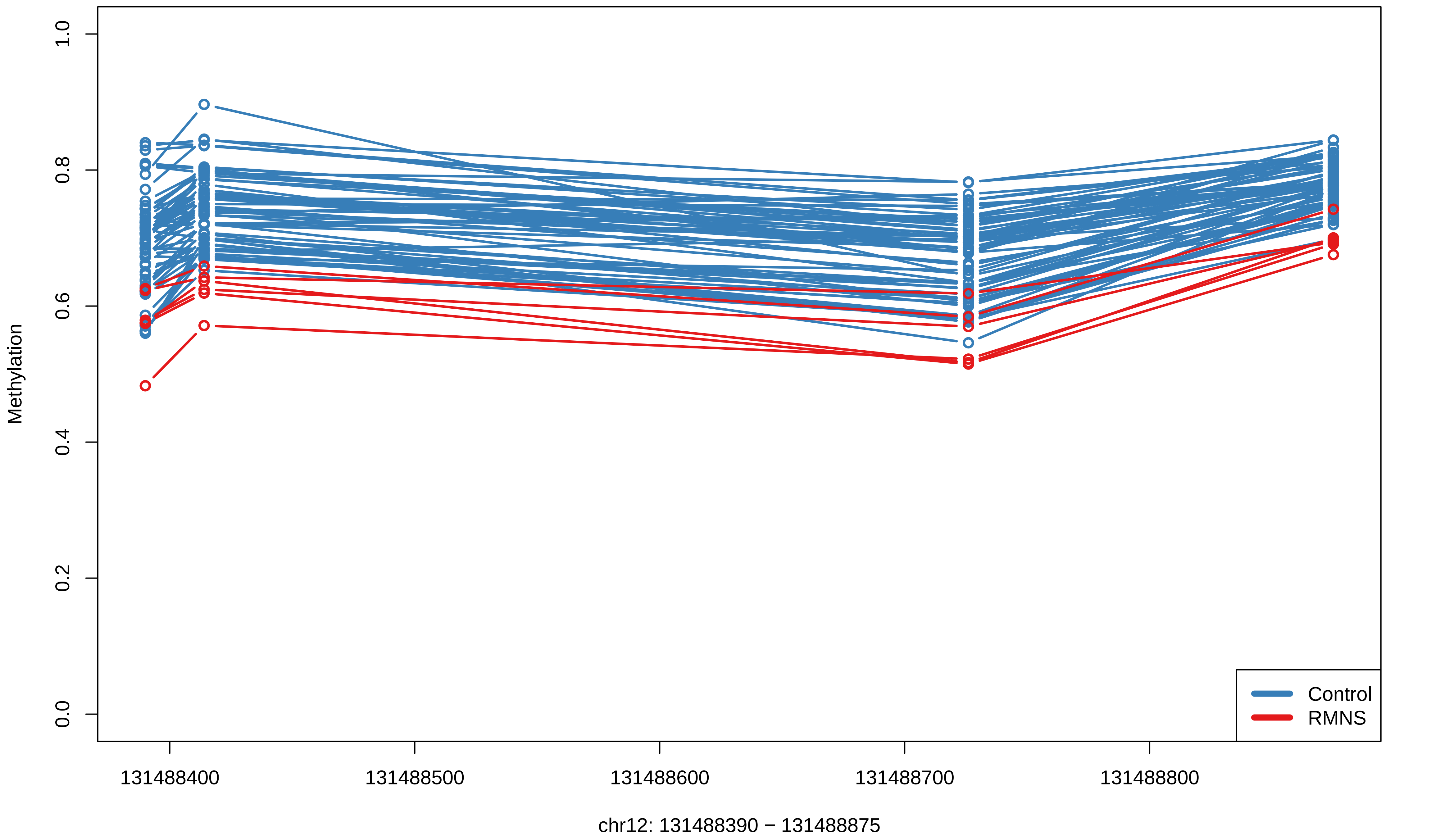

Methylation
0.0
0.2
0.4
0.6
0.8
1.0
131488400
131488500
131488600
131488700
131488800
chr12: 131488390 − 131488875
Control
RMNS

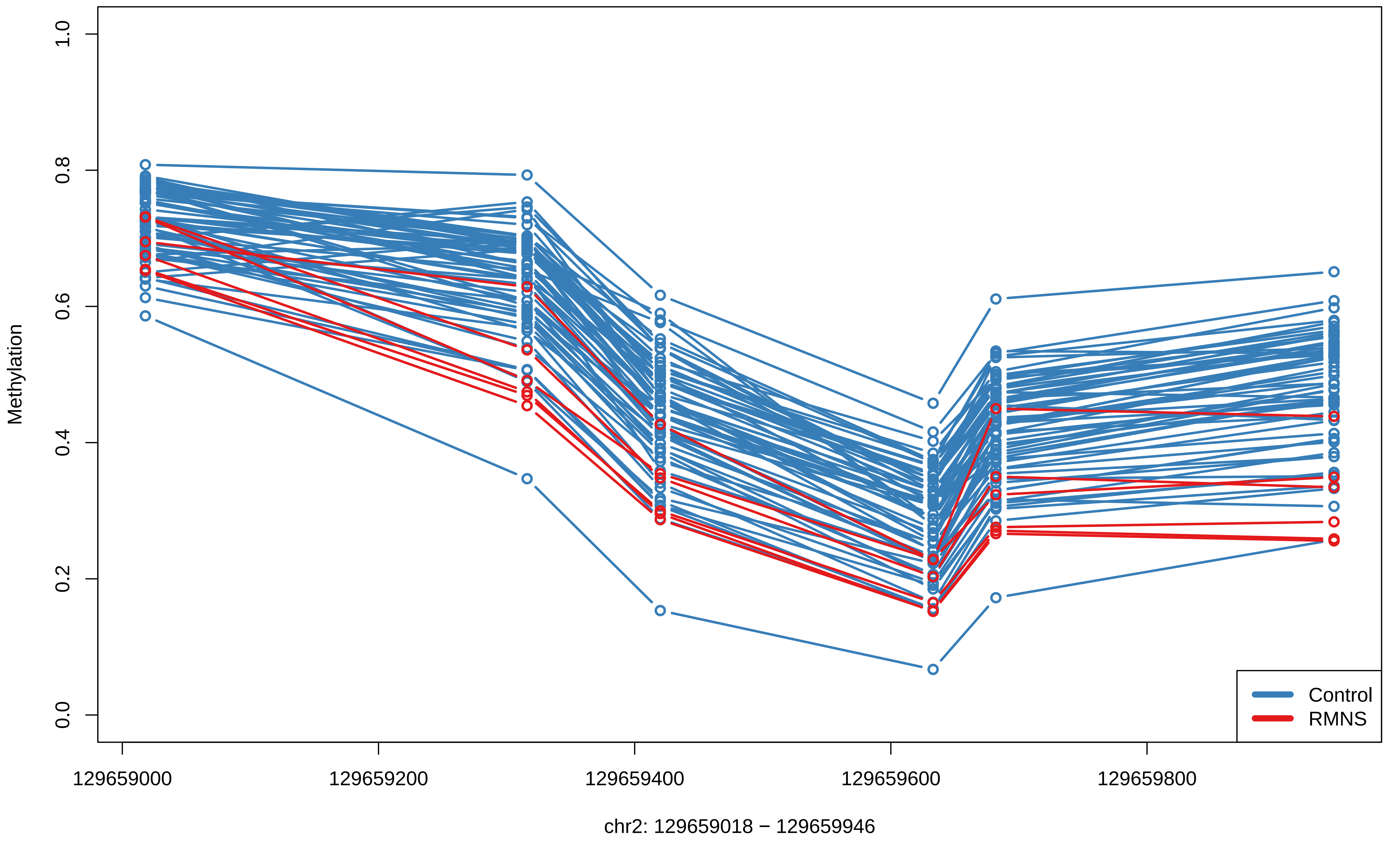

Methylation
1.0
0.8
0.6
0.4
0.2
0.0
129659000
129659200
129659400
129659600
129659800
chr2: 129659018 − 129659946
Control
RMNS

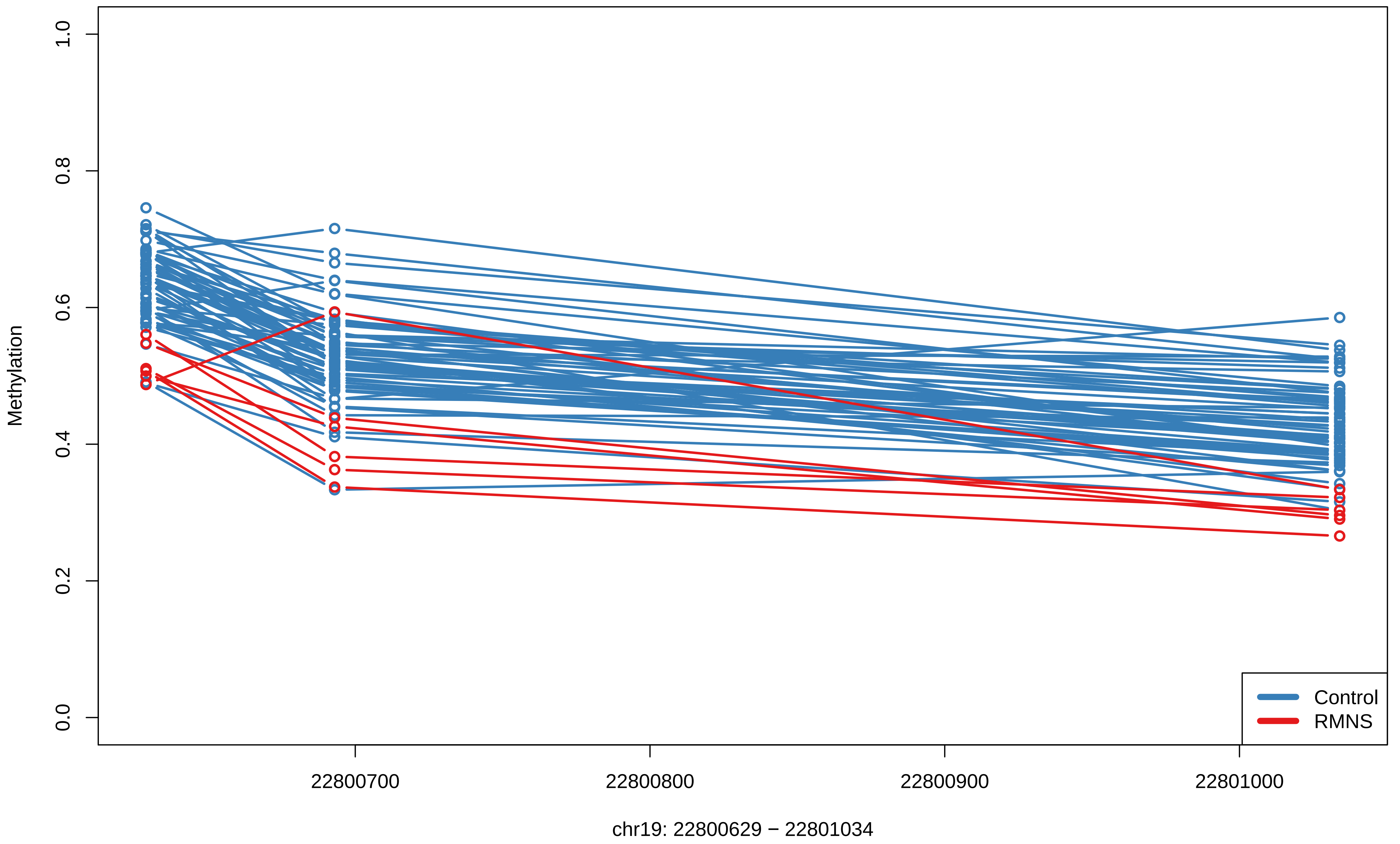

Methylation
0.0
0.2
0.4
0.6
0.8
1.0
22800700
22800800
22800900
22801000
chr19: 22800629 − 22801034
Control
RMNS

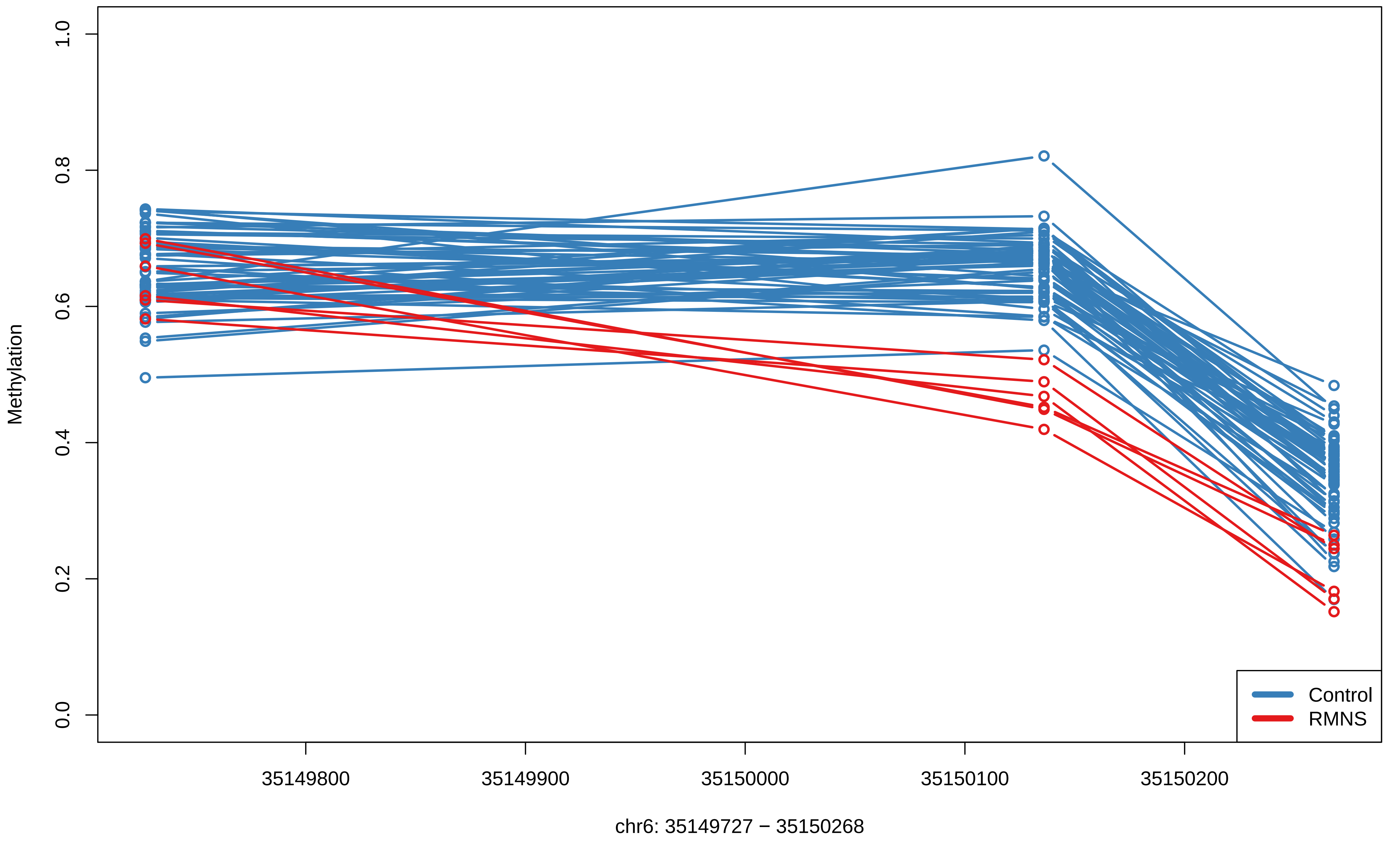

1.0
0.8
0.6
0.4
0.2
0.0
Methylation
35149800
35149900
35150000
35150100
35150200
chr6: 35149727 − 35150268
Control
RMNS

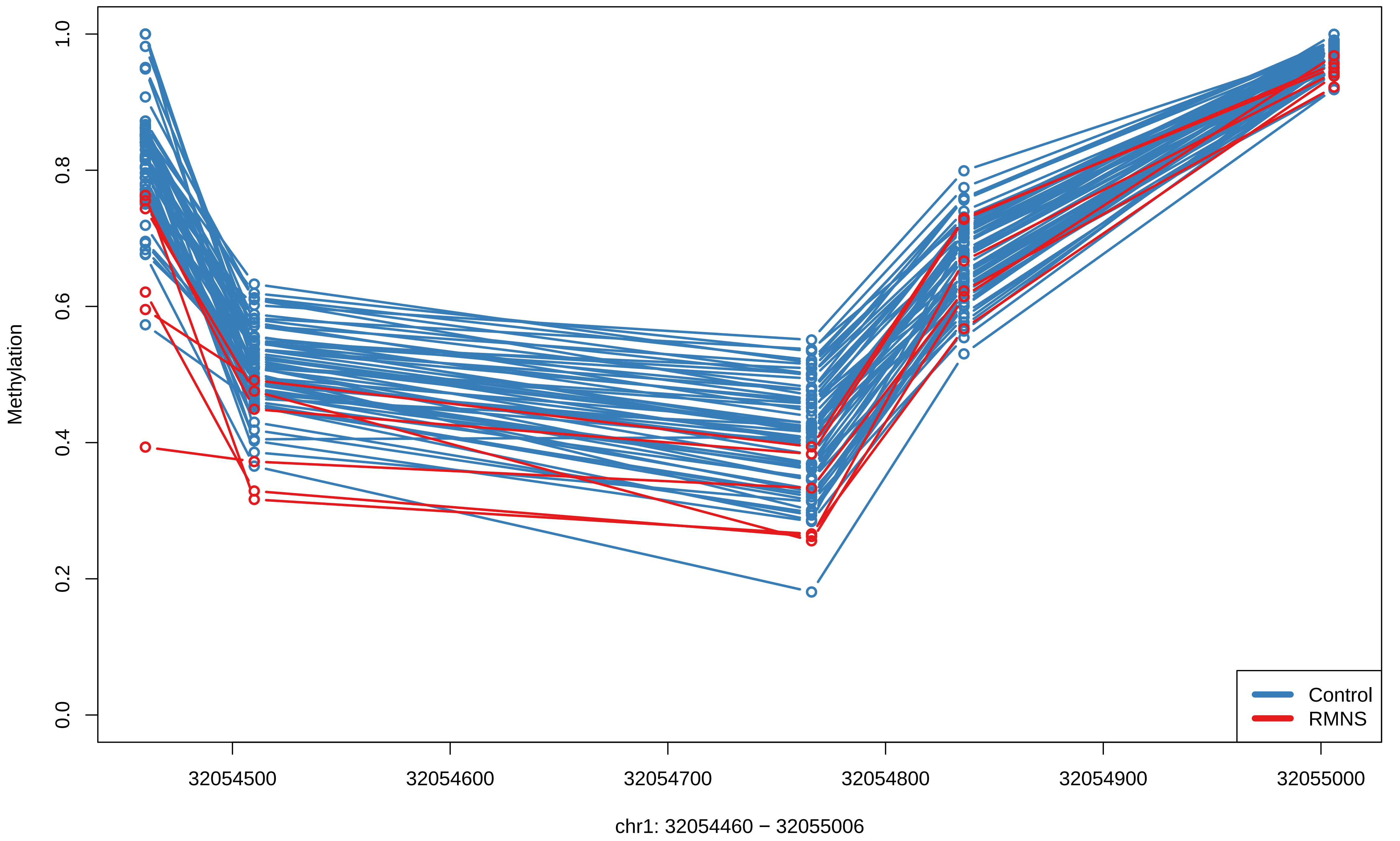

Methylation
0.0
0.2
0.4
0.6
0.8
1.0
32054500
32054600
32054700
32054800
32054900
32055000
chr1: 32054460 − 32055006
Control
RMNS

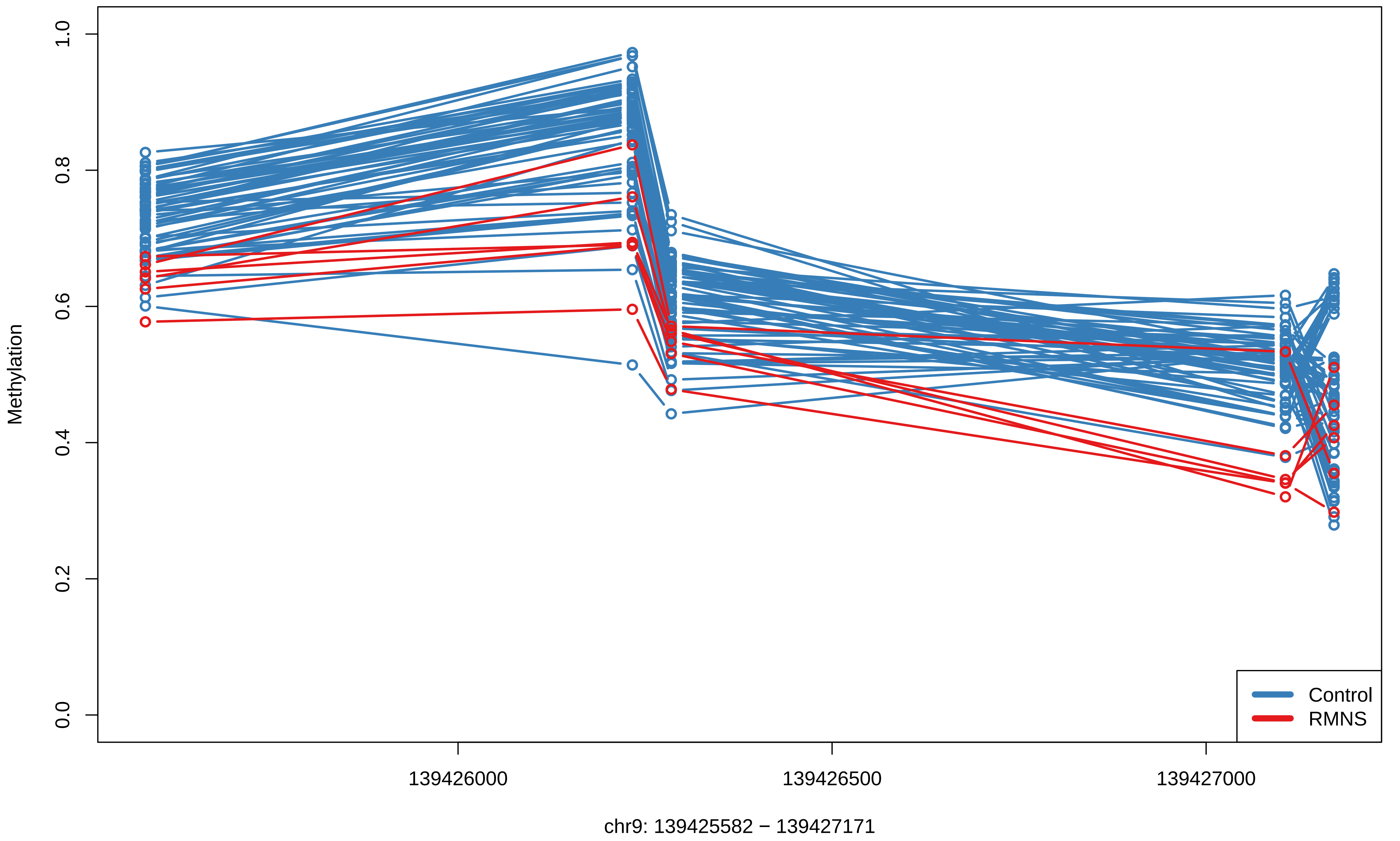

Methylation
0.0
0.2
0.4
0.6
0.8
1.0
139426000
139426500
139427000
chr9: 139425582 − 139427171
Control
RMNS

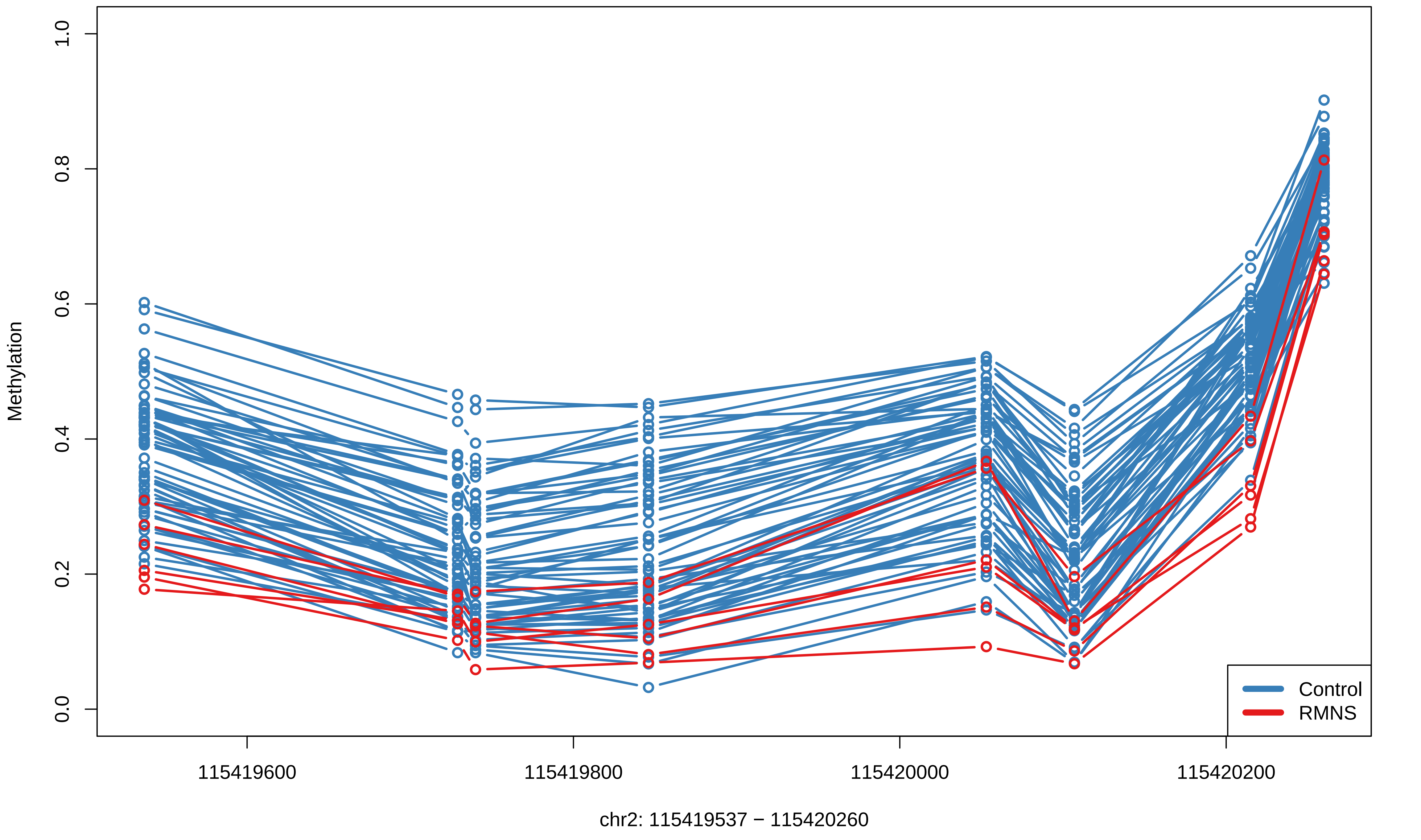

Methylation
0.0
0.2
0.4
0.6
0.8
1.0
115419600
115419800
115420000
115420200
chr2: 115419537 − 115420260
Control
RMNS

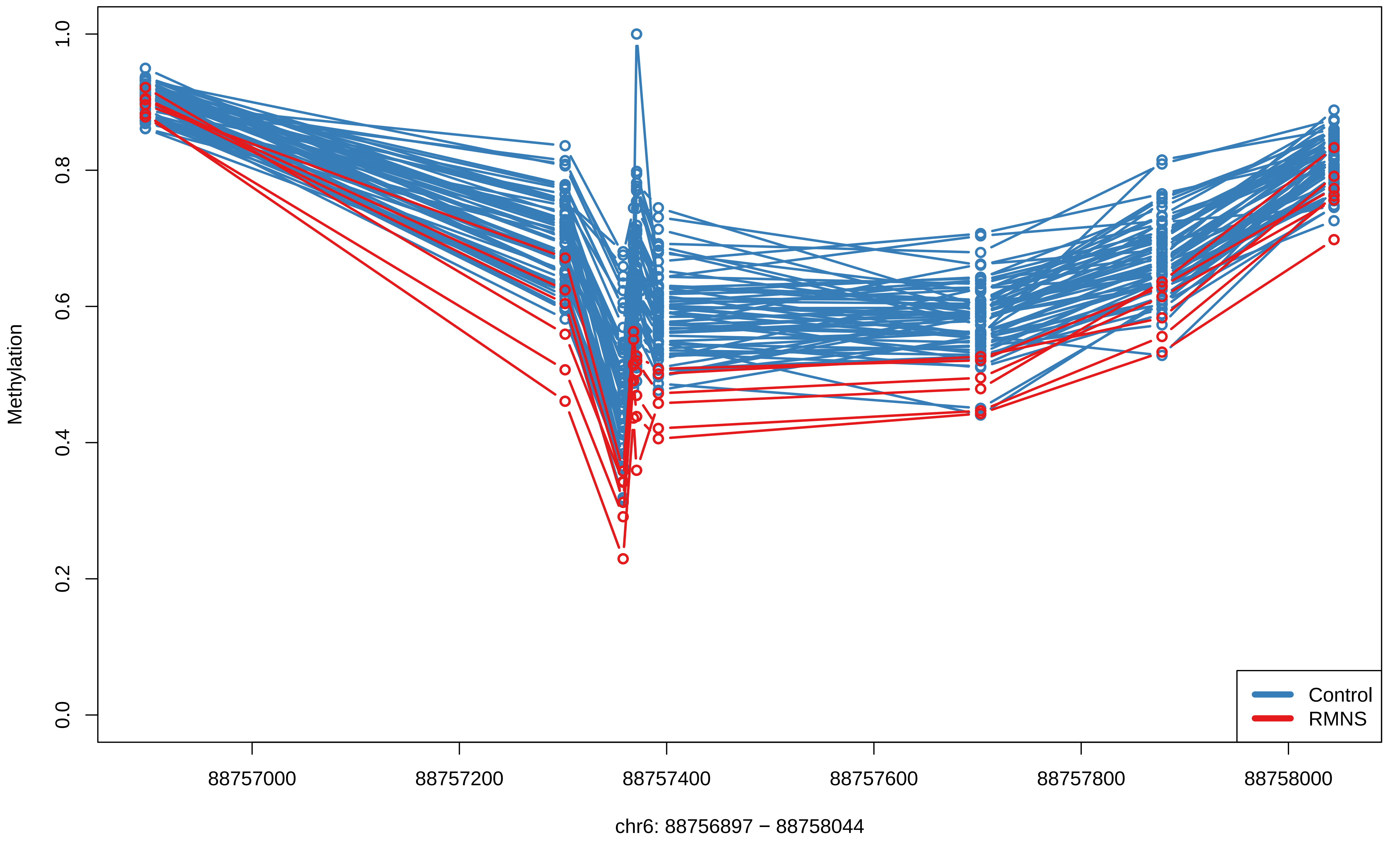

Methylation
1.0
0.8
0.6
0.4
0.2
0.0
88757000
88757200
88757400
88757600
88757800
88758000
chr6: 88756897 − 88758044
Control
RMNS

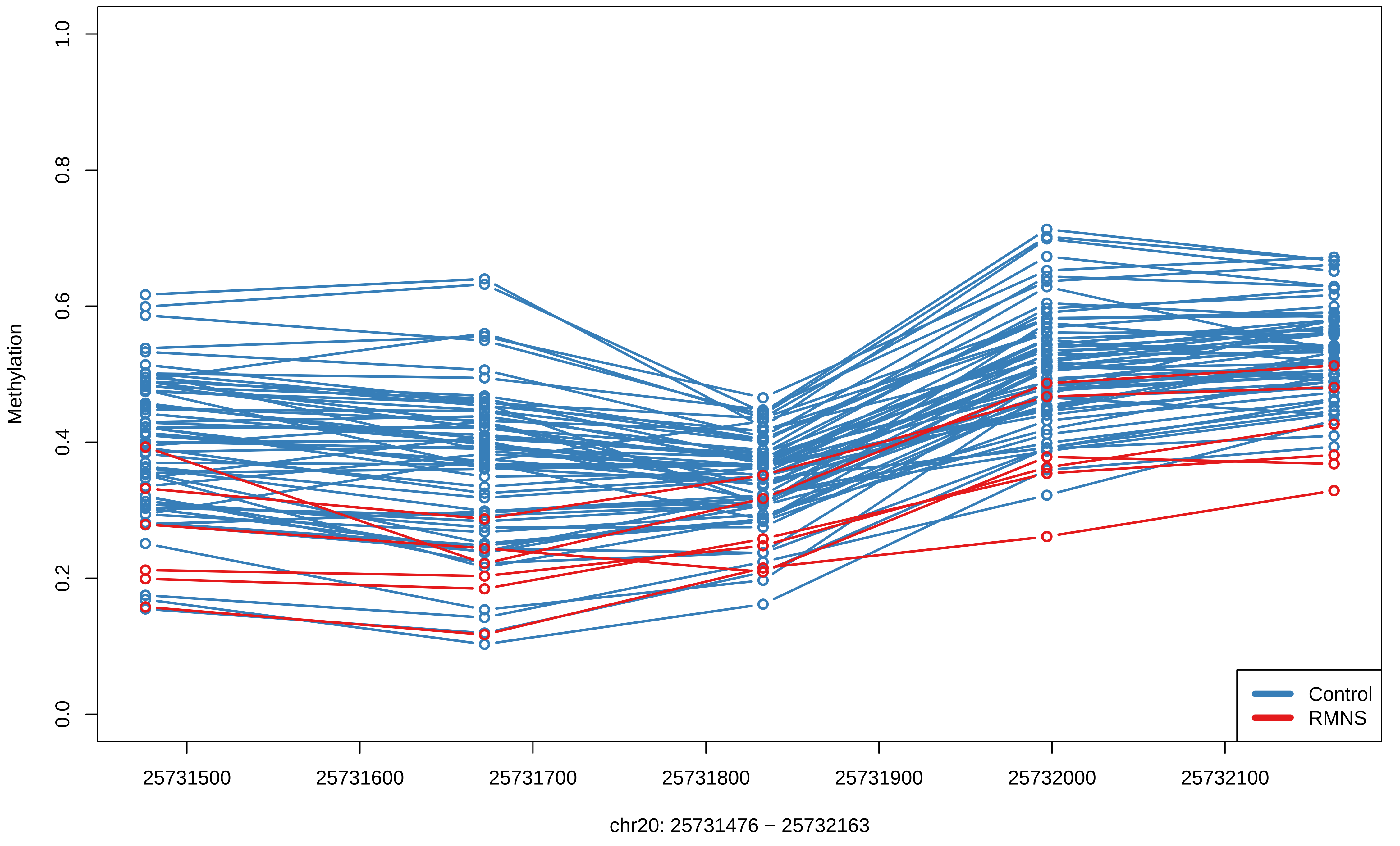

Methylation
0.0
0.2
0.4
0.6
0.8
1.0
25731500
25731600
25731700
25731800
25731900
25732000
25732100
chr20: 25731476 − 25732163
Control
RMNS

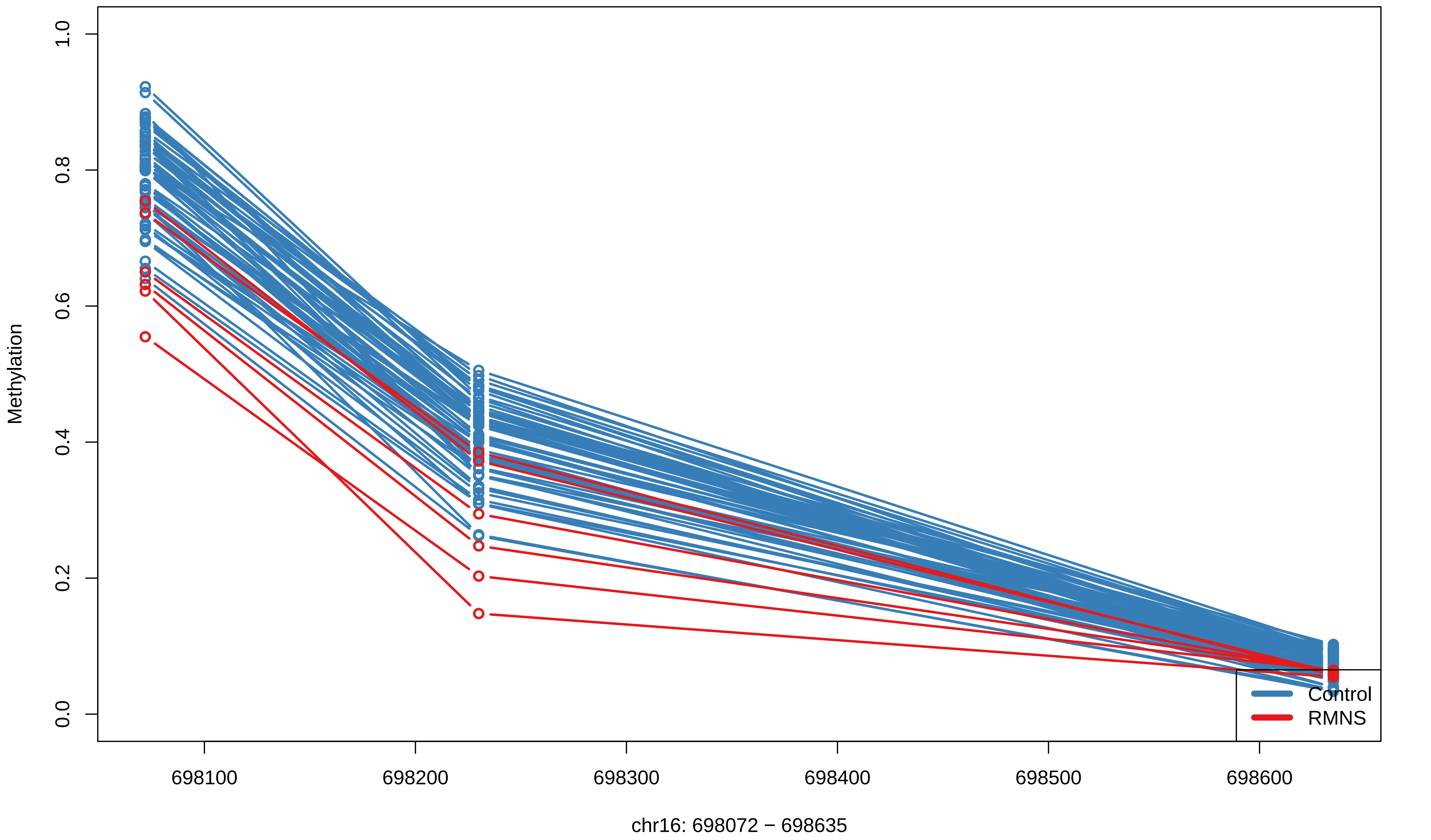

1.0
0.8
0.6
0.4
0.2
0.0
Methylation
698100
698200
698300
698400
698500
698600
chr16: 698072 − 698635
Control
RMNS

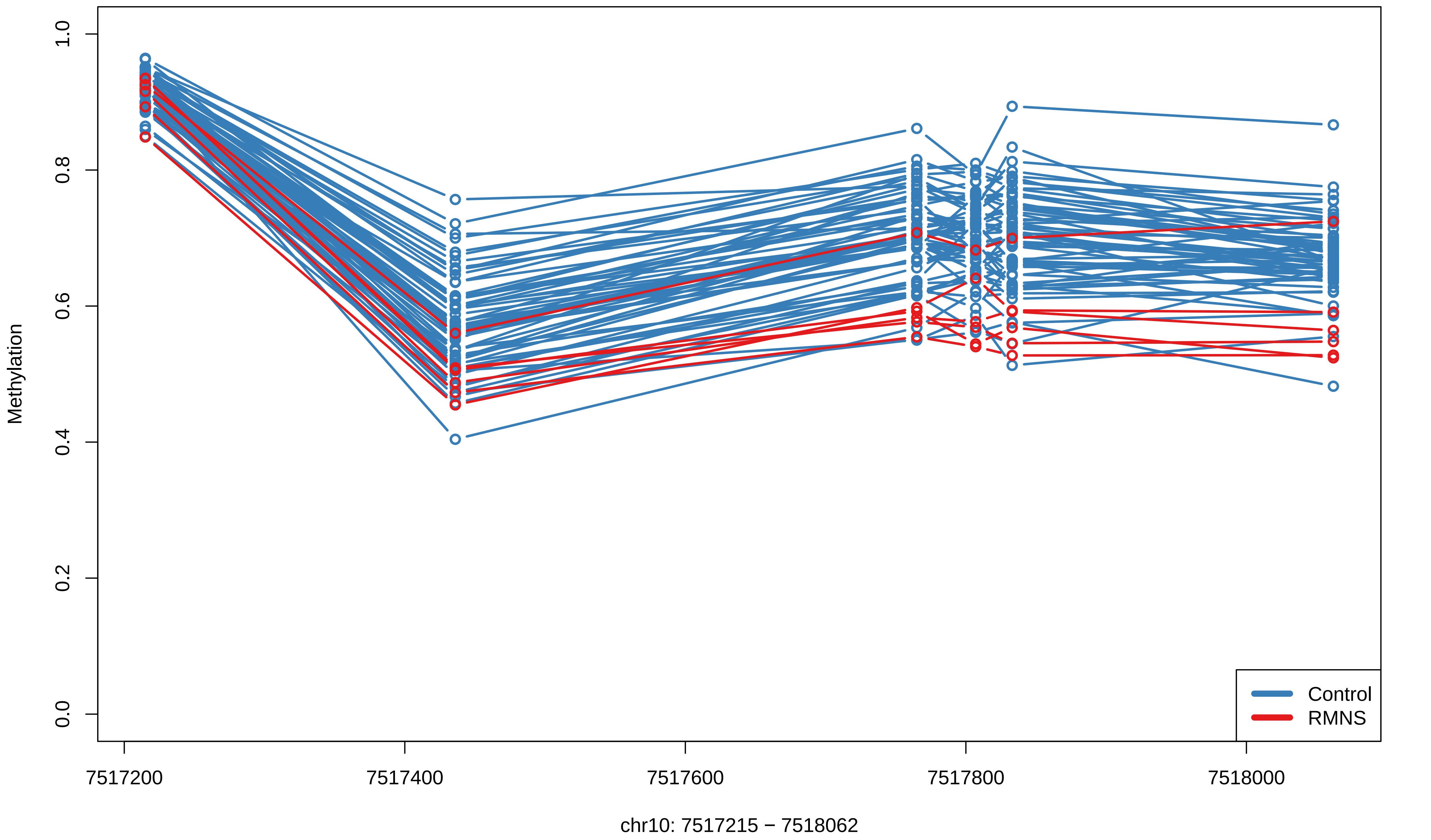

Methylation
1.0
0.8
0.6
0.4
0.2
0.0
7517200
7517400
7517600
7517800
7518000
chr10: 7517215 − 7518062
Control
RMNS

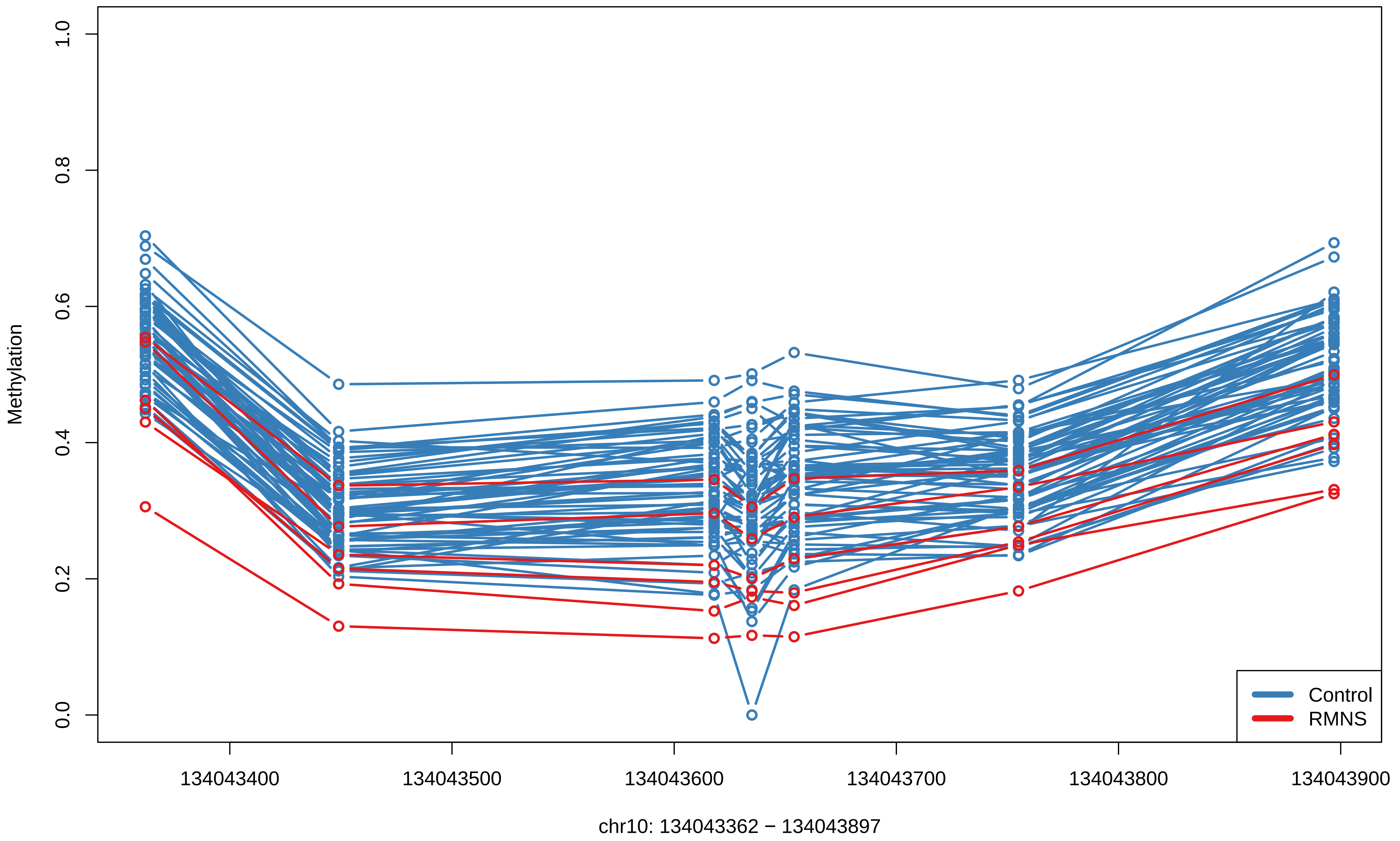

Methylation
0.0
0.2
0.4
0.6
0.8
1.0
134043400
134043500
134043600
134043700
134043800
134043900
chr10: 134043362 − 134043897
Control
RMNS

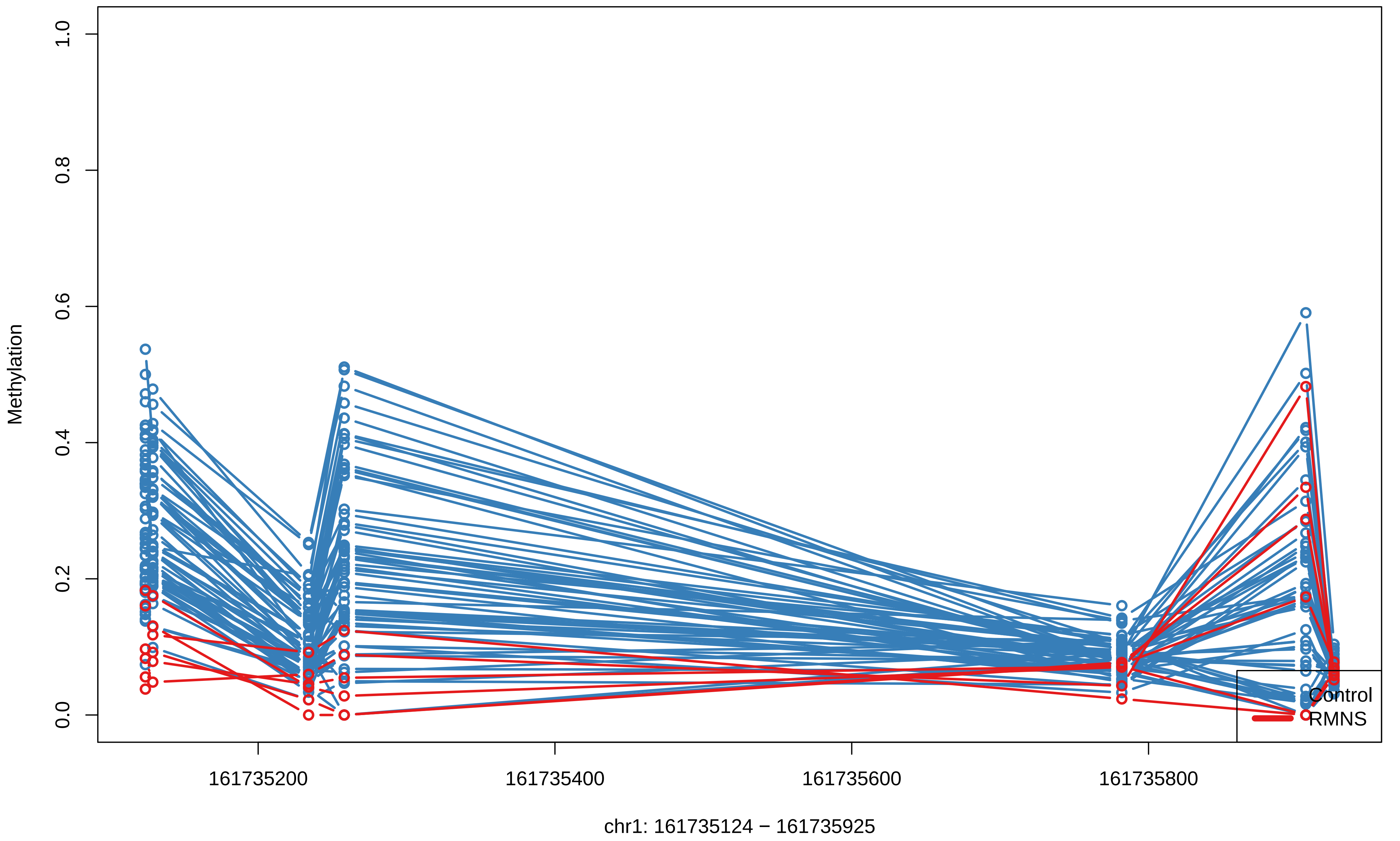

Methylation
1.0
0.8
0.6
0.4
0.2
0.0
161735200
161735400
161735600
161735800
chr1: 161735124 − 161735925
Control
RMNS

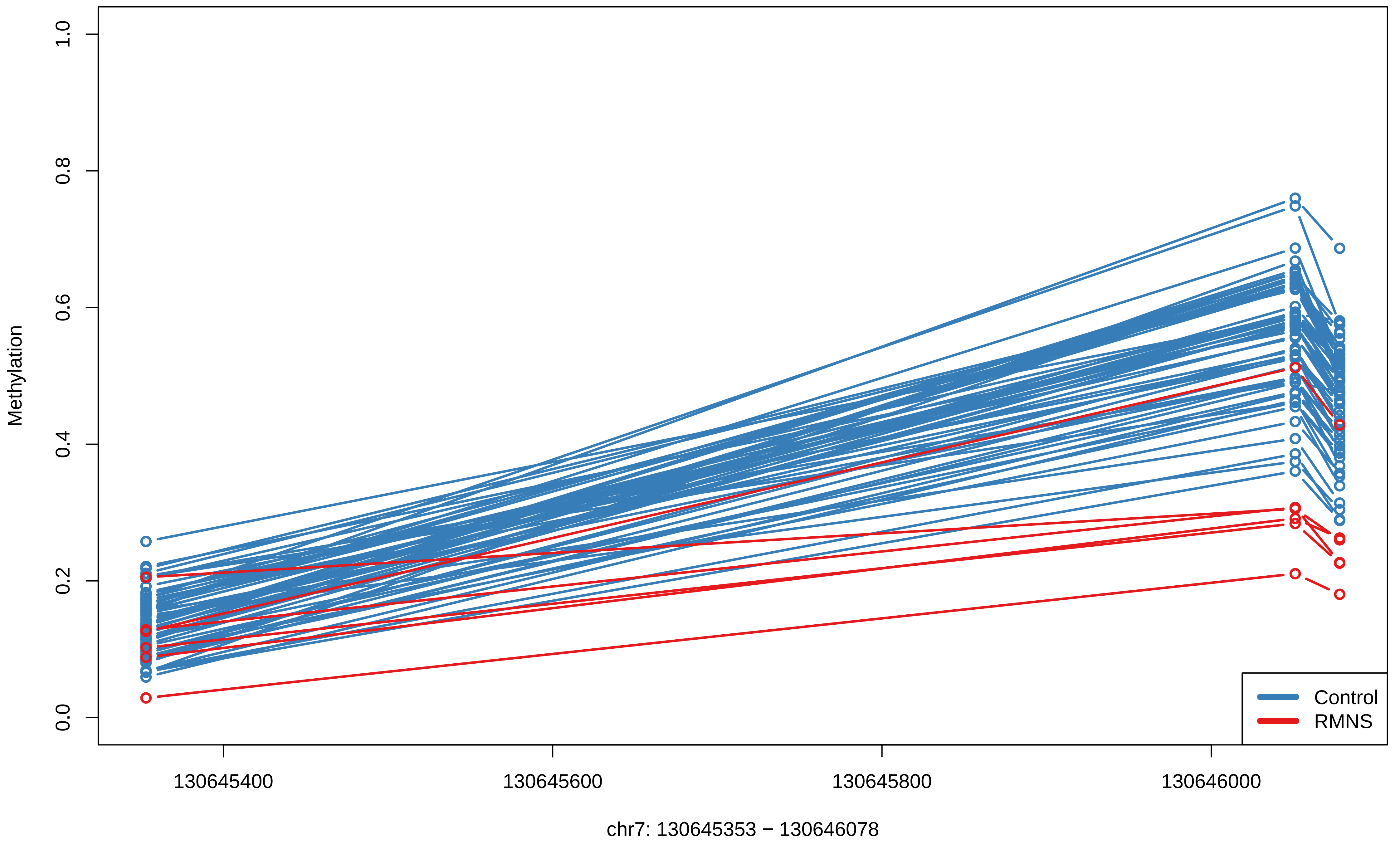

Methylation
0.0
0.2
0.4
0.6
0.8
1.0
130645400
130645600
130645800
130646000
chr7: 130645353 − 130646078
Control
RMNS

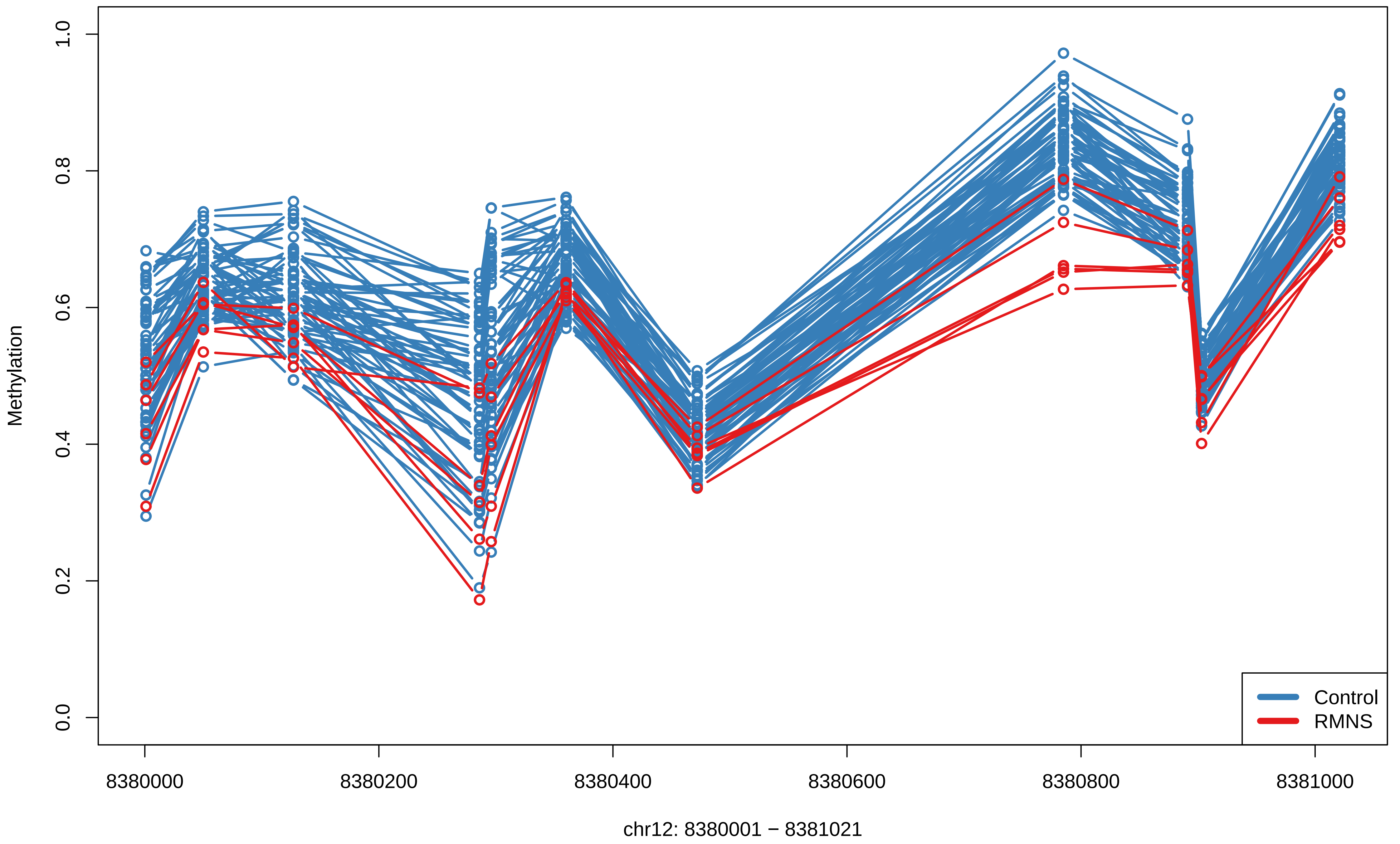

Methylation
0.0
0.2
0.4
0.6
0.8
1.0
8380000
8380200
8380400
8380600
8380800
8381000
chr12: 8380001 − 8381021
Control
RMNS

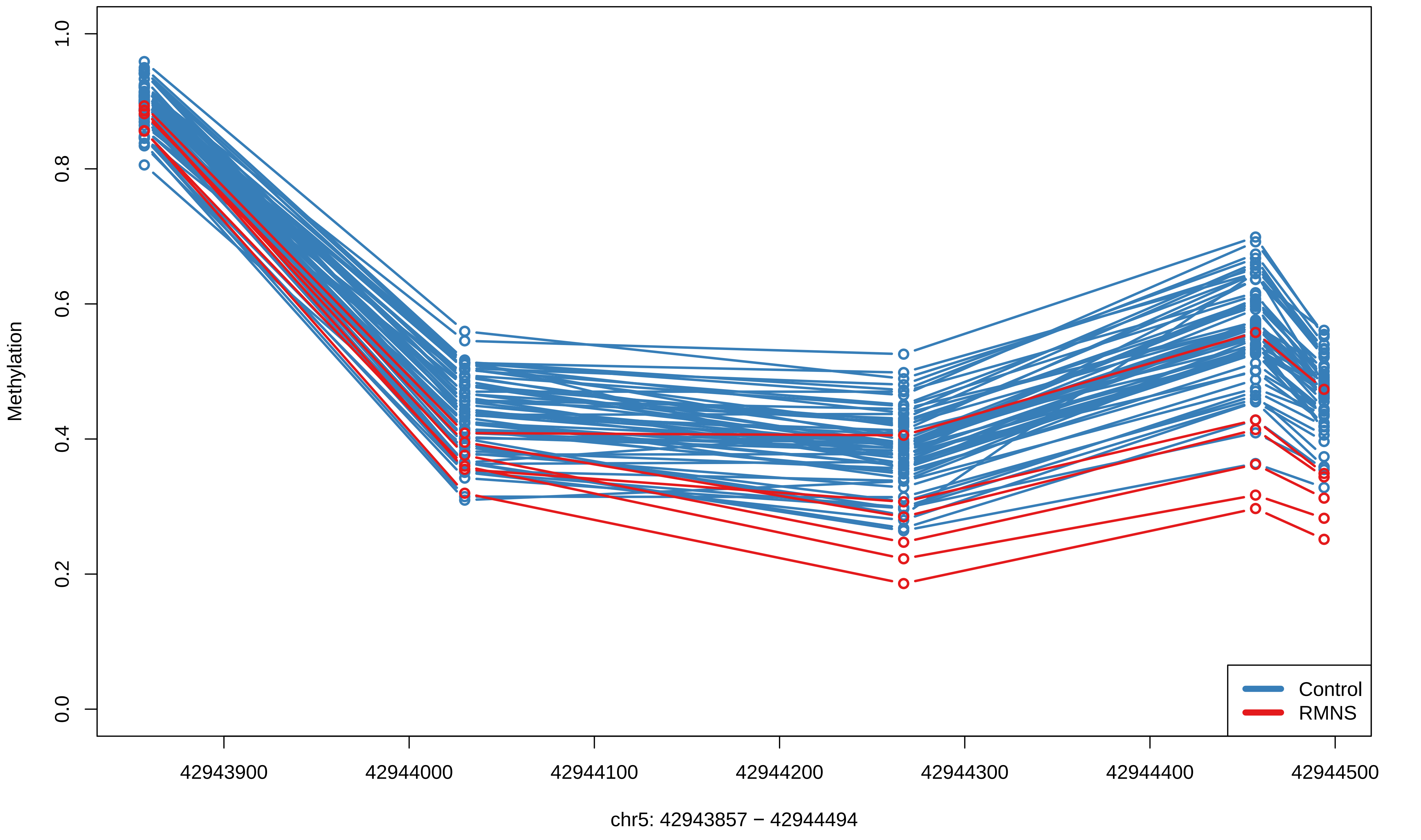

Methylation
1.0
0.8
0.6
0.4
0.2
0.0
42943900
42944000
42944100
42944200
42944300
42944400
42944500
chr5: 42943857 − 42944494
Control
RMNS

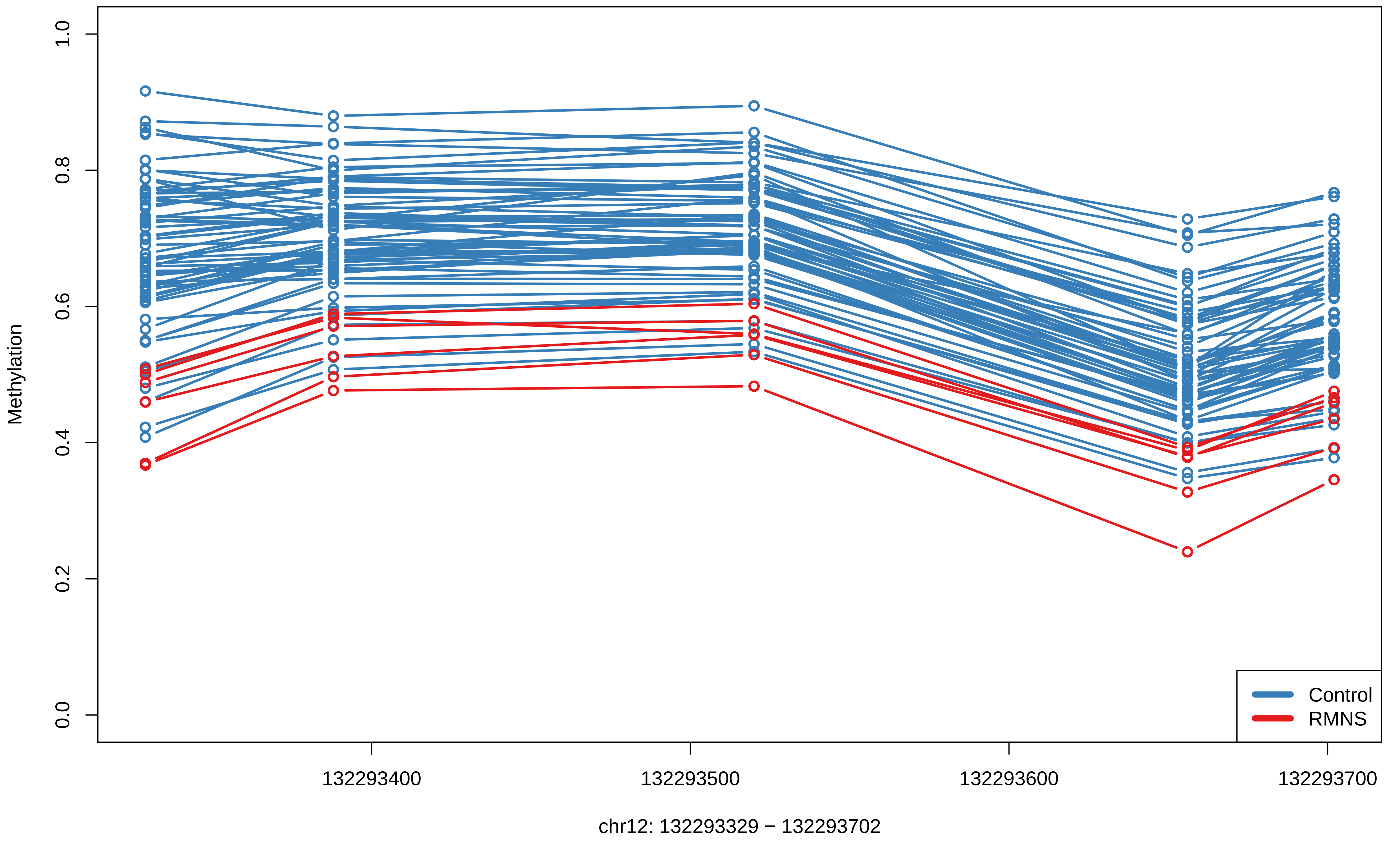

Methylation
0.0
0.2
0.4
0.6
0.8
1.0
132293400
132293500
132293600
132293700
chr12: 132293329 − 132293702
Control
RMNS

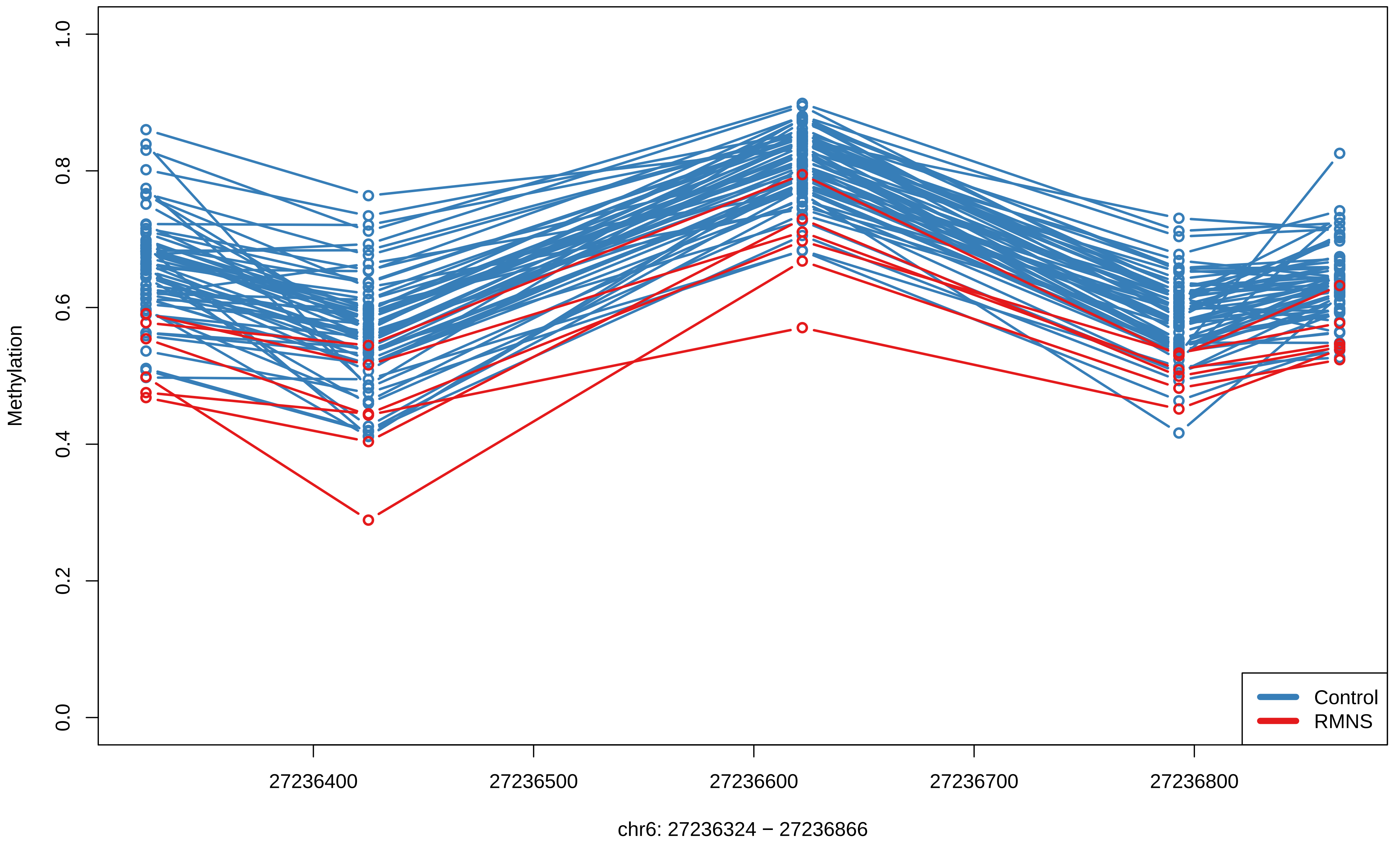

Methylation
0.0
0.2
0.4
0.6
0.8
1.0
27236400
27236500
27236600
27236700
27236800
chr6: 27236324 − 27236866
Control
RMNS

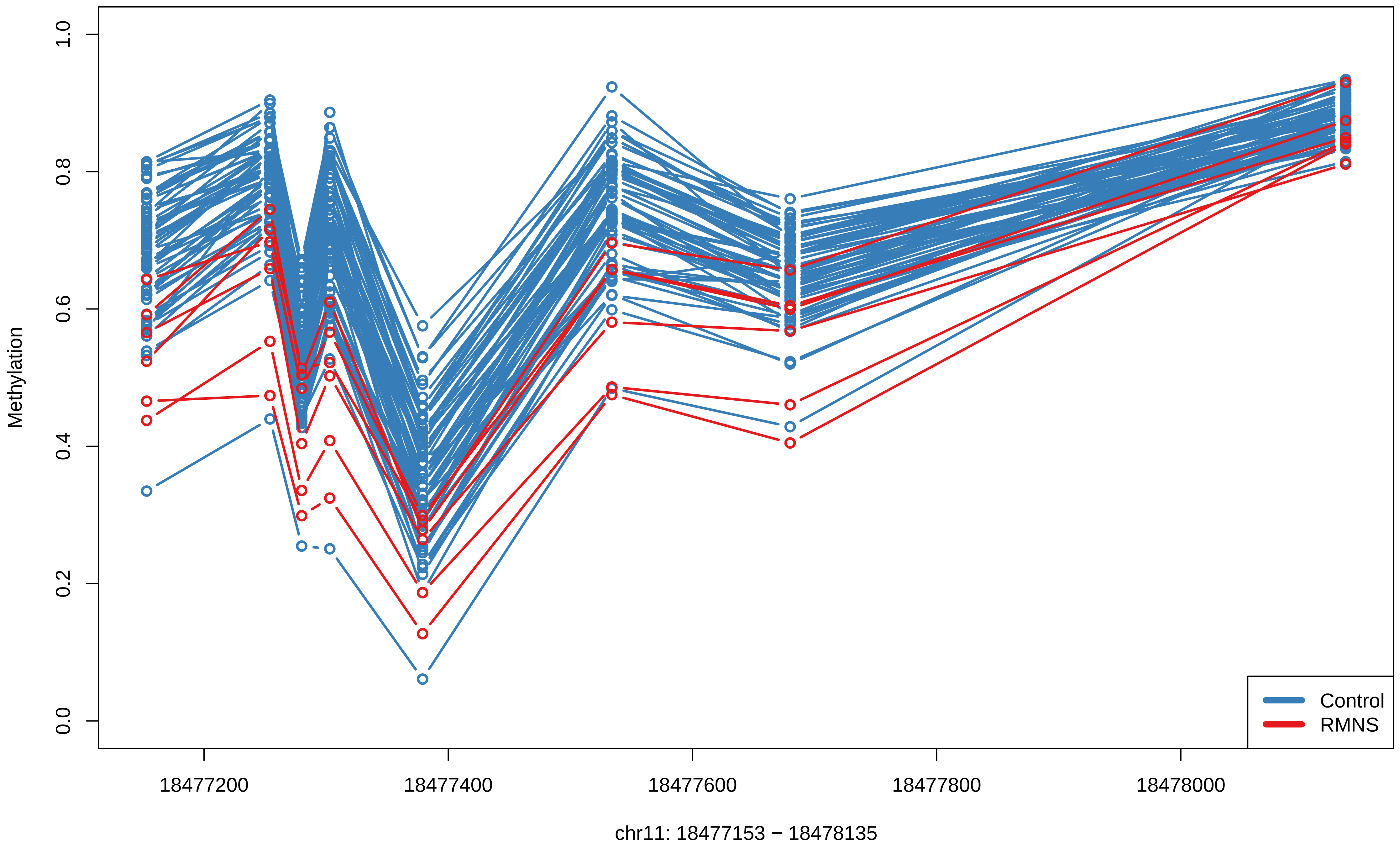

Methylation
0.0
0.2
0.4
0.6
0.8
1.0
18477200
18477400
18477600
18477800
18478000
chr11: 18477153 − 18478135
Control
RMNS

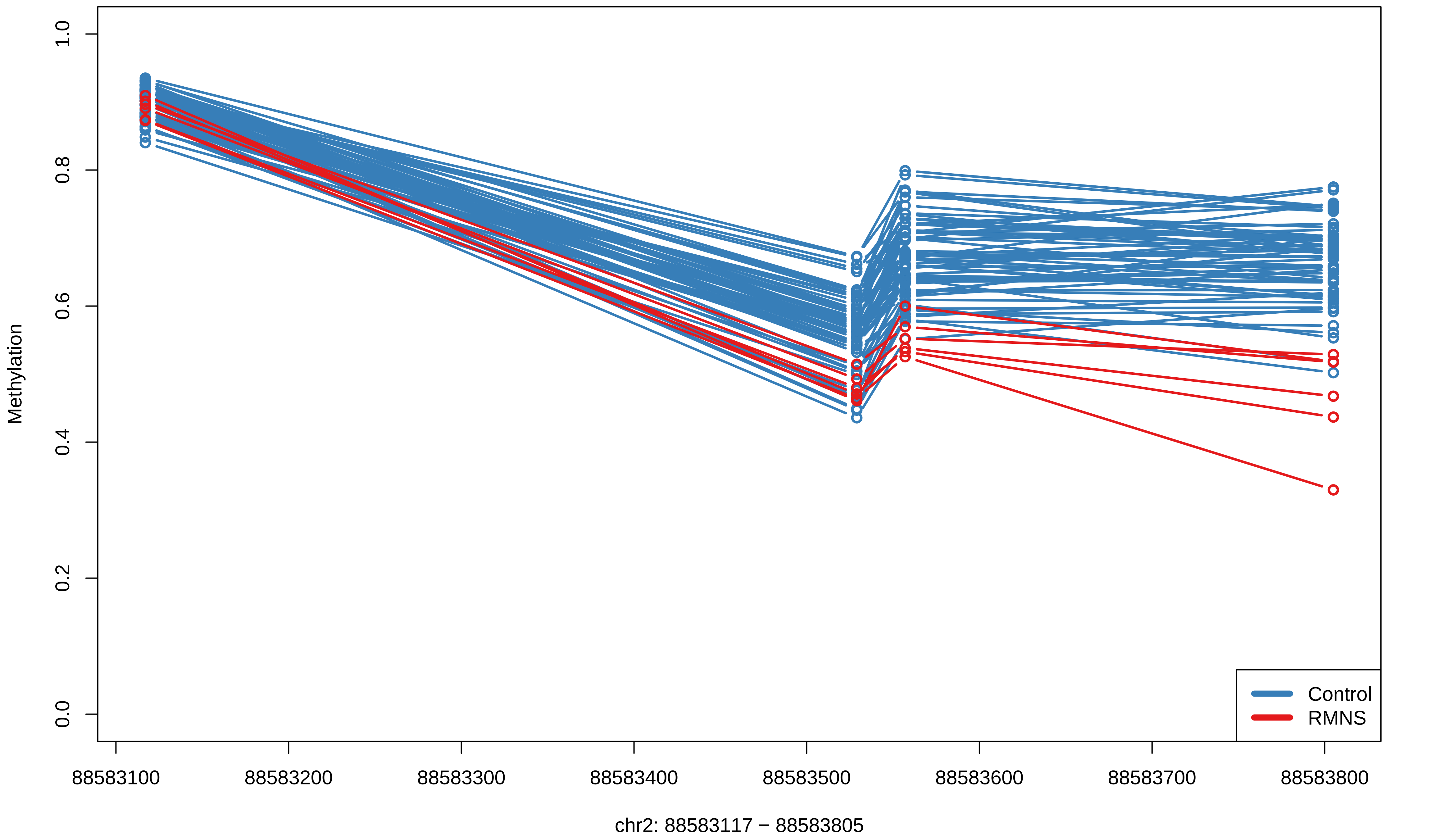

Methylation
0.0
0.2
0.4
0.6
0.8
1.0
Control
RMNS
88583100
88583200
88583300
88583400
88583500
88583600
88583700
88583800
chr2: 88583117 − 88583805

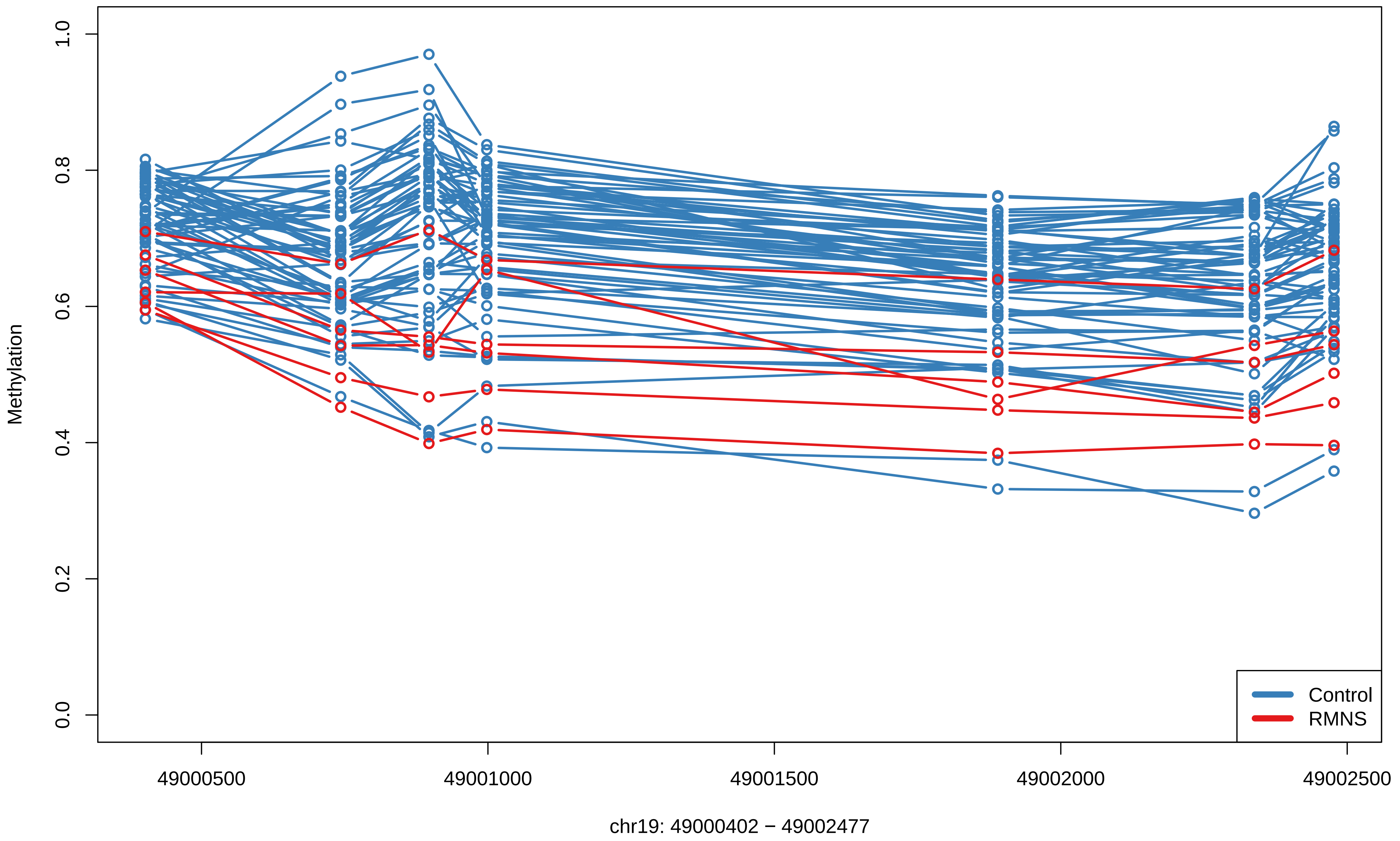

Methylation
0.0
0.2
0.4
0.6
0.8
1.0
49000500
49001000
49001500
49002000
49002500
chr19: 49000402 − 49002477
Control
RMNS

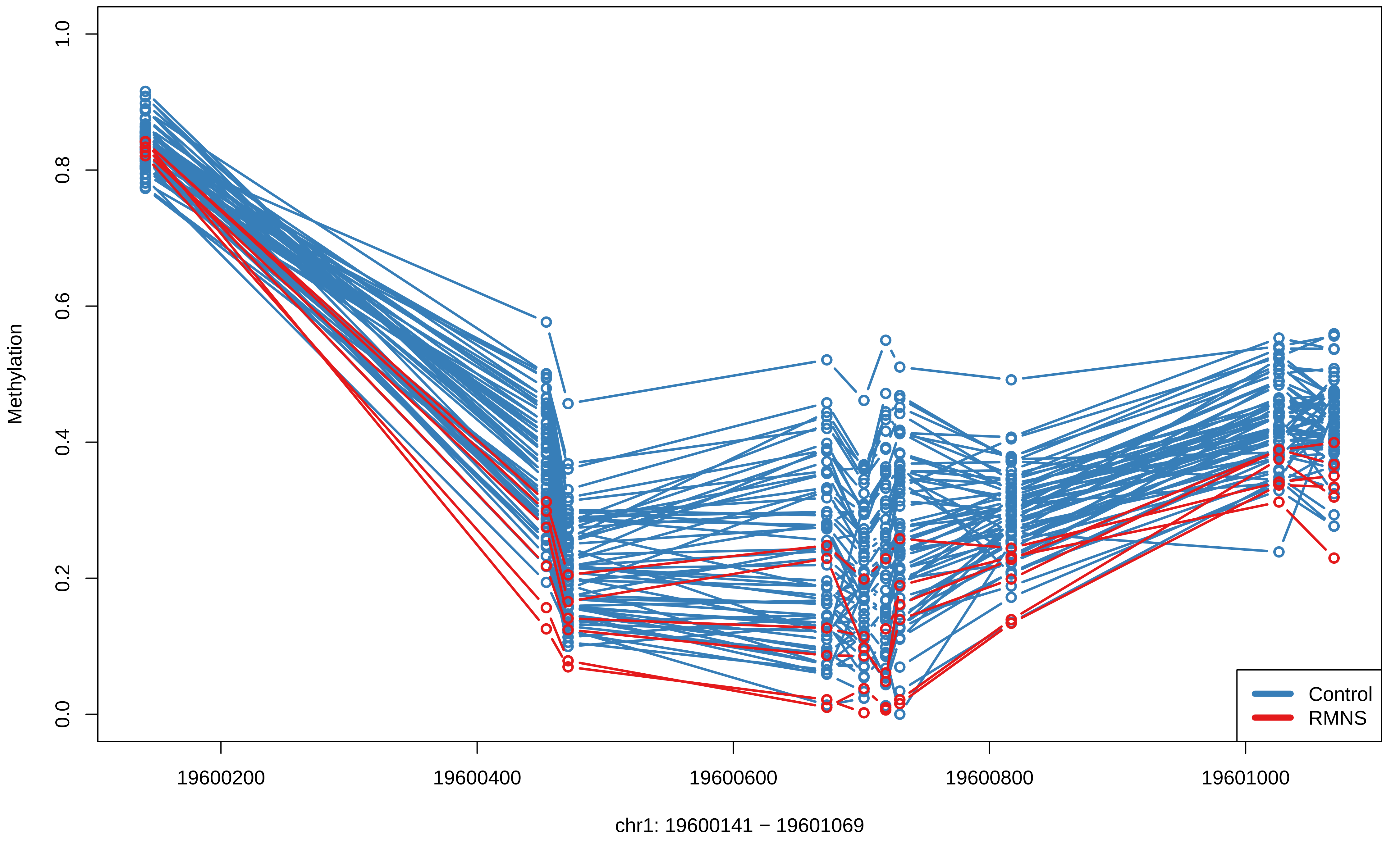

Methylation
0.0
0.2
0.4
0.6
0.8
1.0
19600200
19600400
19600600
19600800
19601000
chr1: 19600141 − 19601069
Control
RMNS

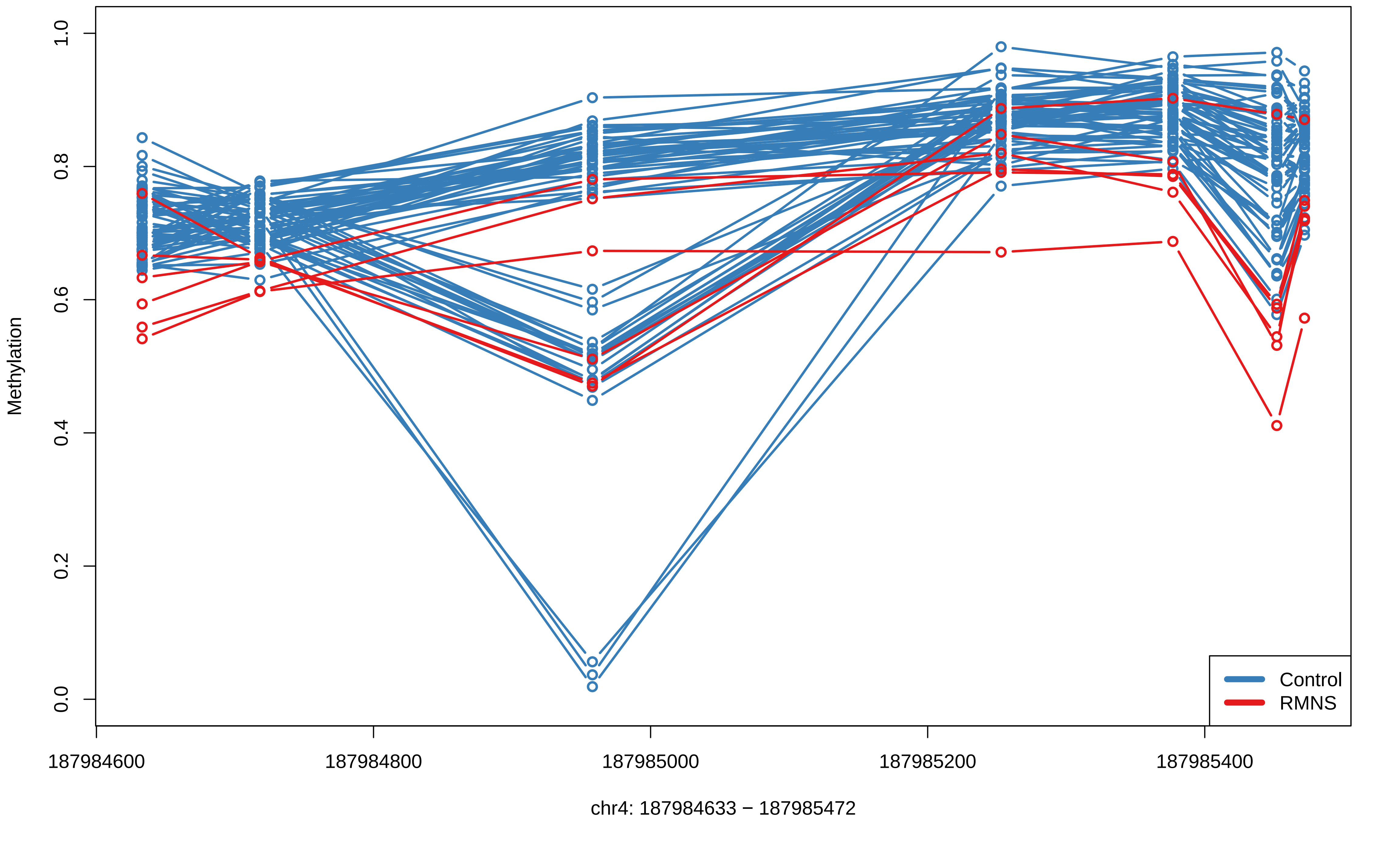

Methylation
0.0
0.2
0.4
0.6
0.8
1.0
187984600
187984800
187985000
187985200
187985400
chr4: 187984633 − 187985472
Control
RMNS

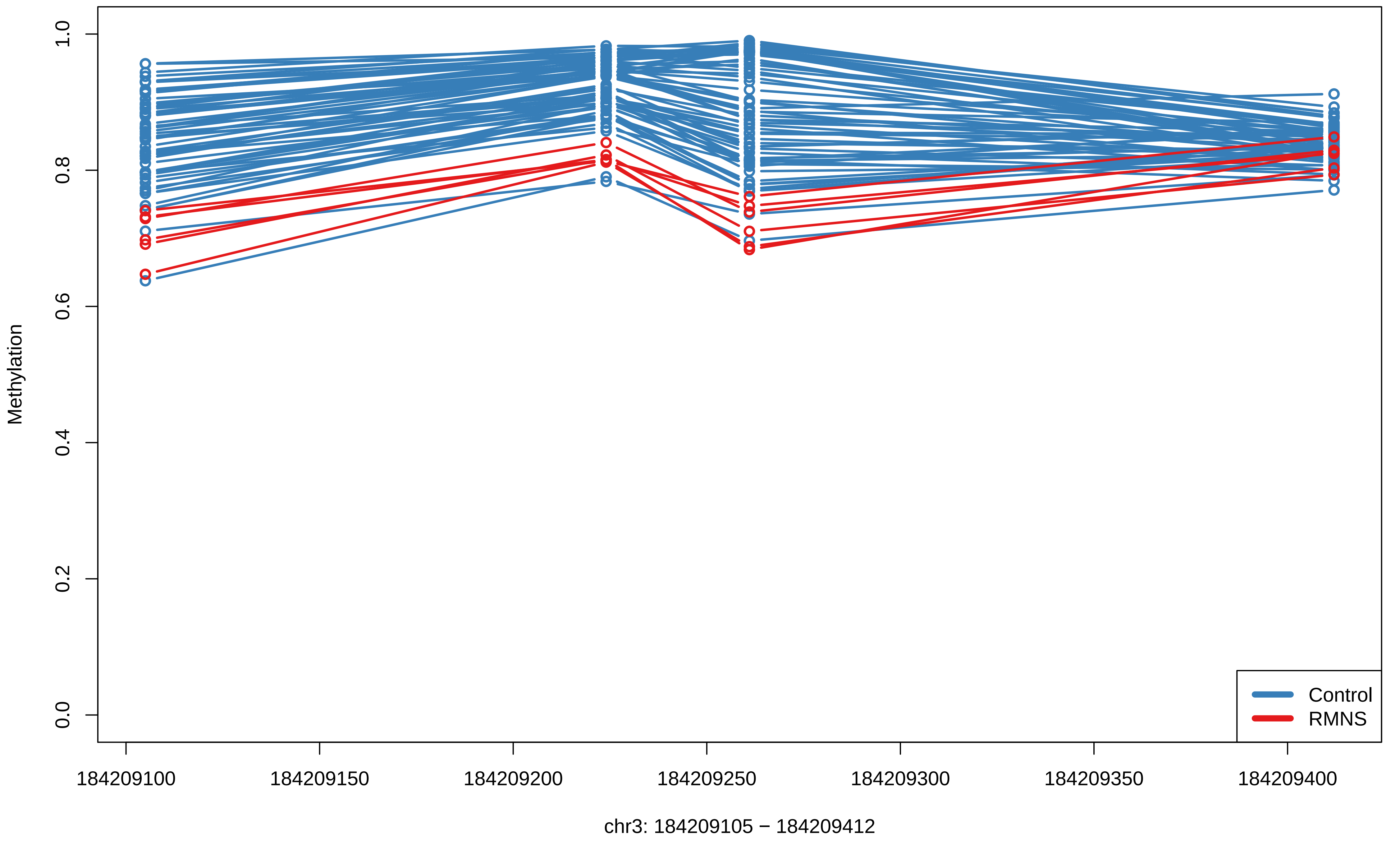

Methylation
0.0
0.2
0.4
0.6
0.8
1.0
184209100
184209150
184209200
184209250
184209300
184209350
184209400
chr3: 184209105 − 184209412
Control
RMNS

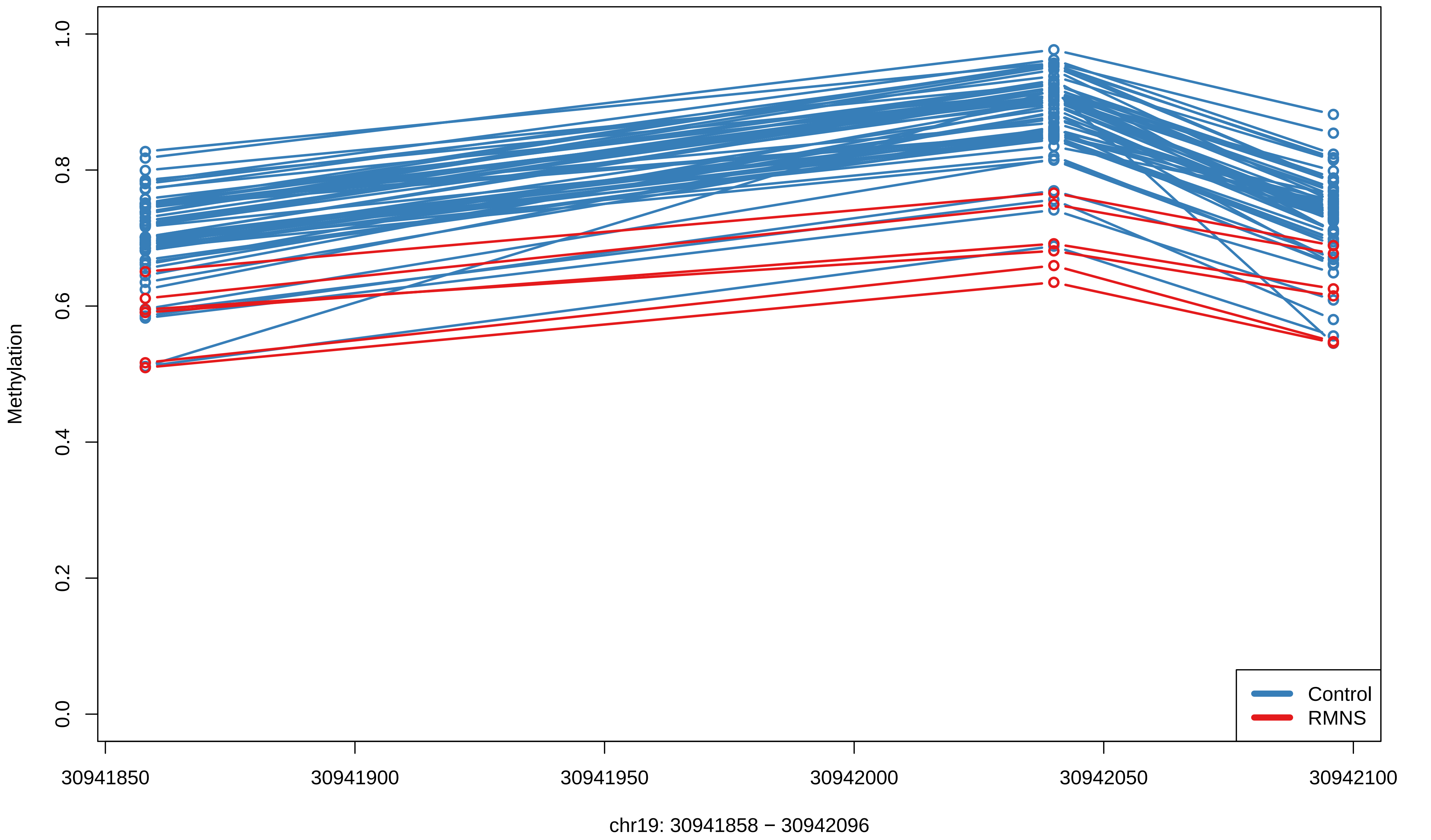

Methylation
1.0
0.8
0.6
0.4
0.2
0.0
30941850
30941900
30941950
30942000
30942050
30942100
chr19: 30941858 − 30942096
Control
RMNS

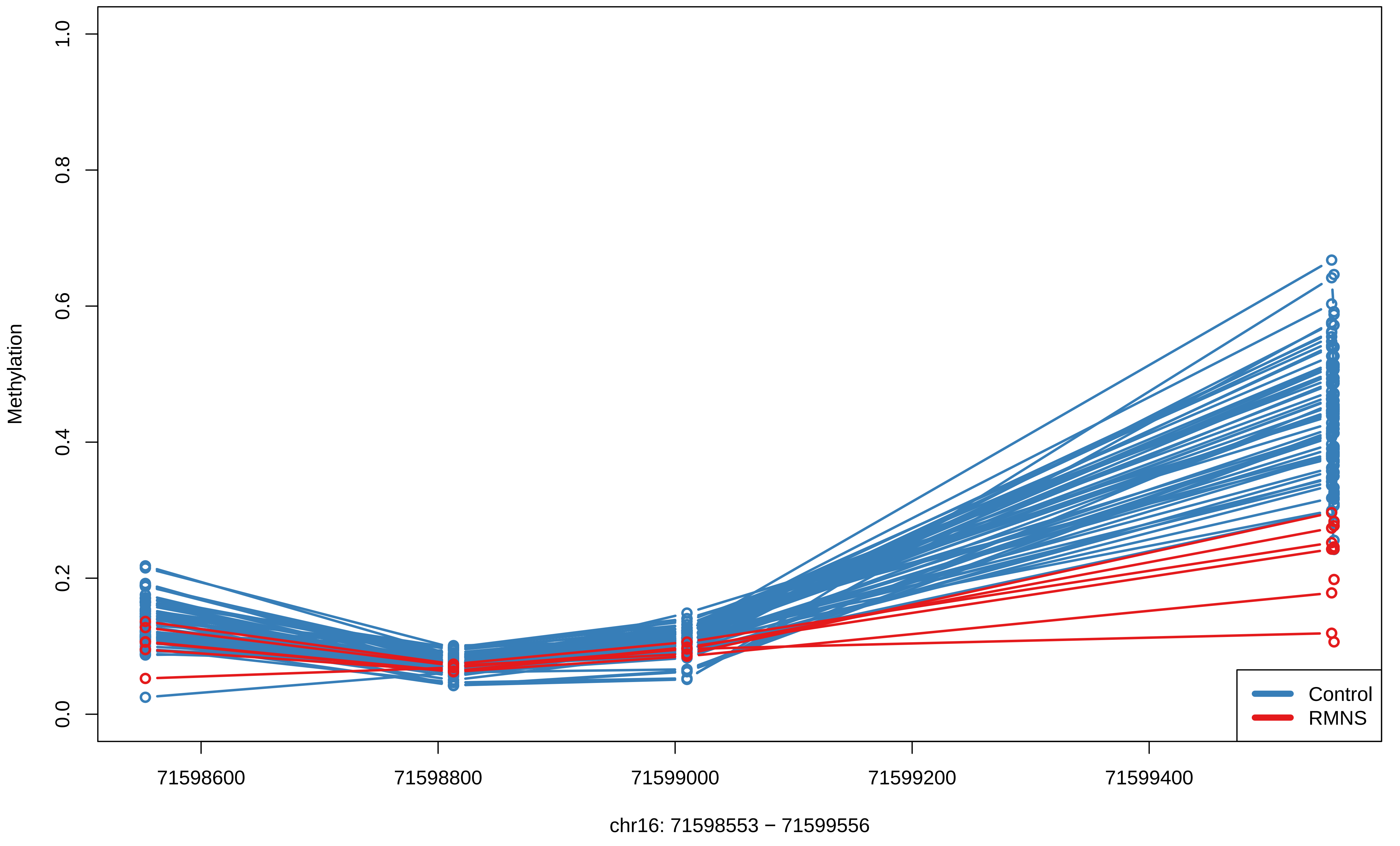
Methylation
0.0
0.2
0.4
0.6
0.8
1.0
71598600
71598800
71599000
71599200
71599400
chr16: 71598553 − 71599556
Control
RMNS

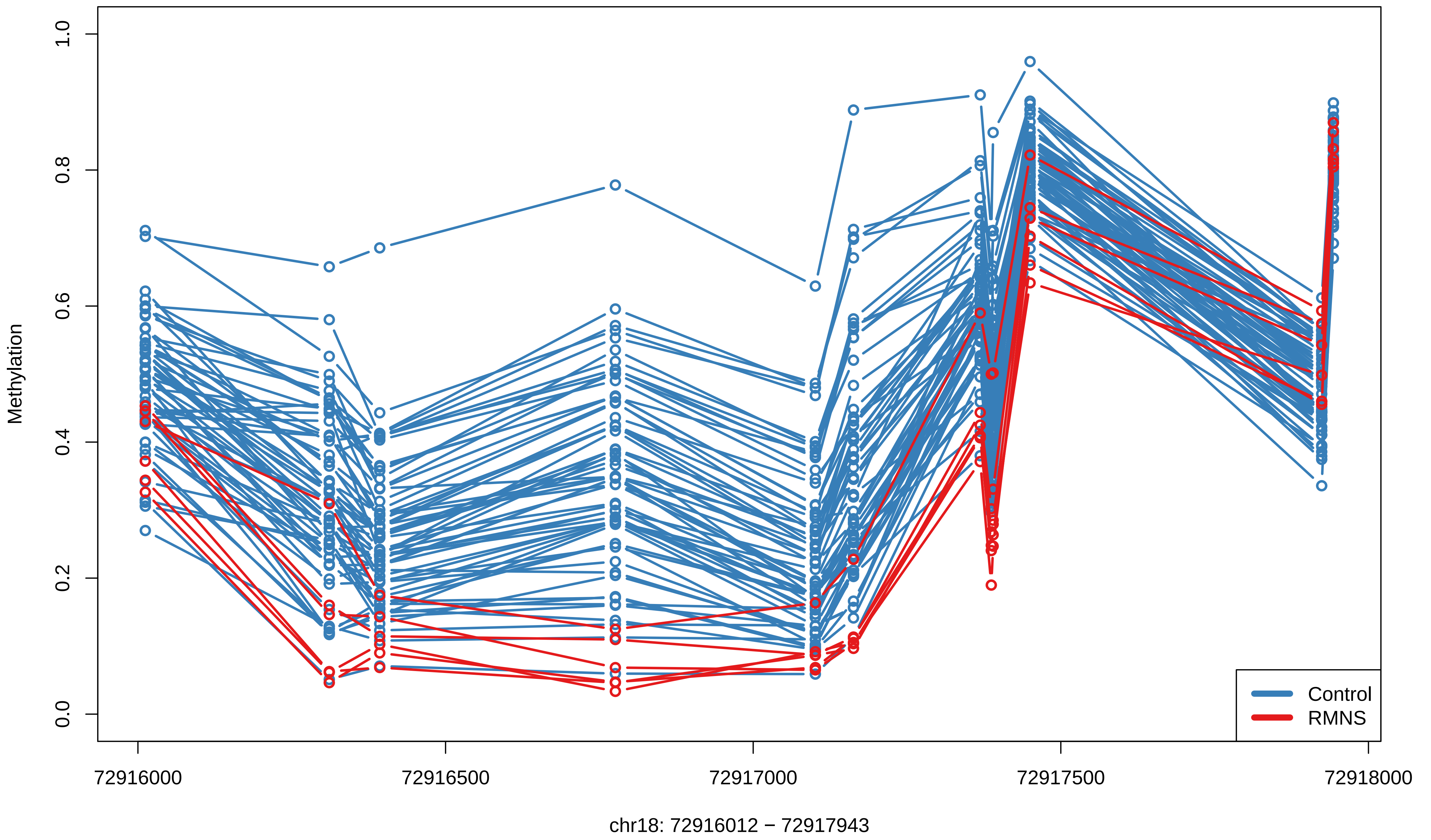

Methylation
0.0
0.2
0.4
0.6
0.8
1.0
72916000
72916500
72917000
72917500
72918000
chr18: 72916012 − 72917943
Control
RMNS

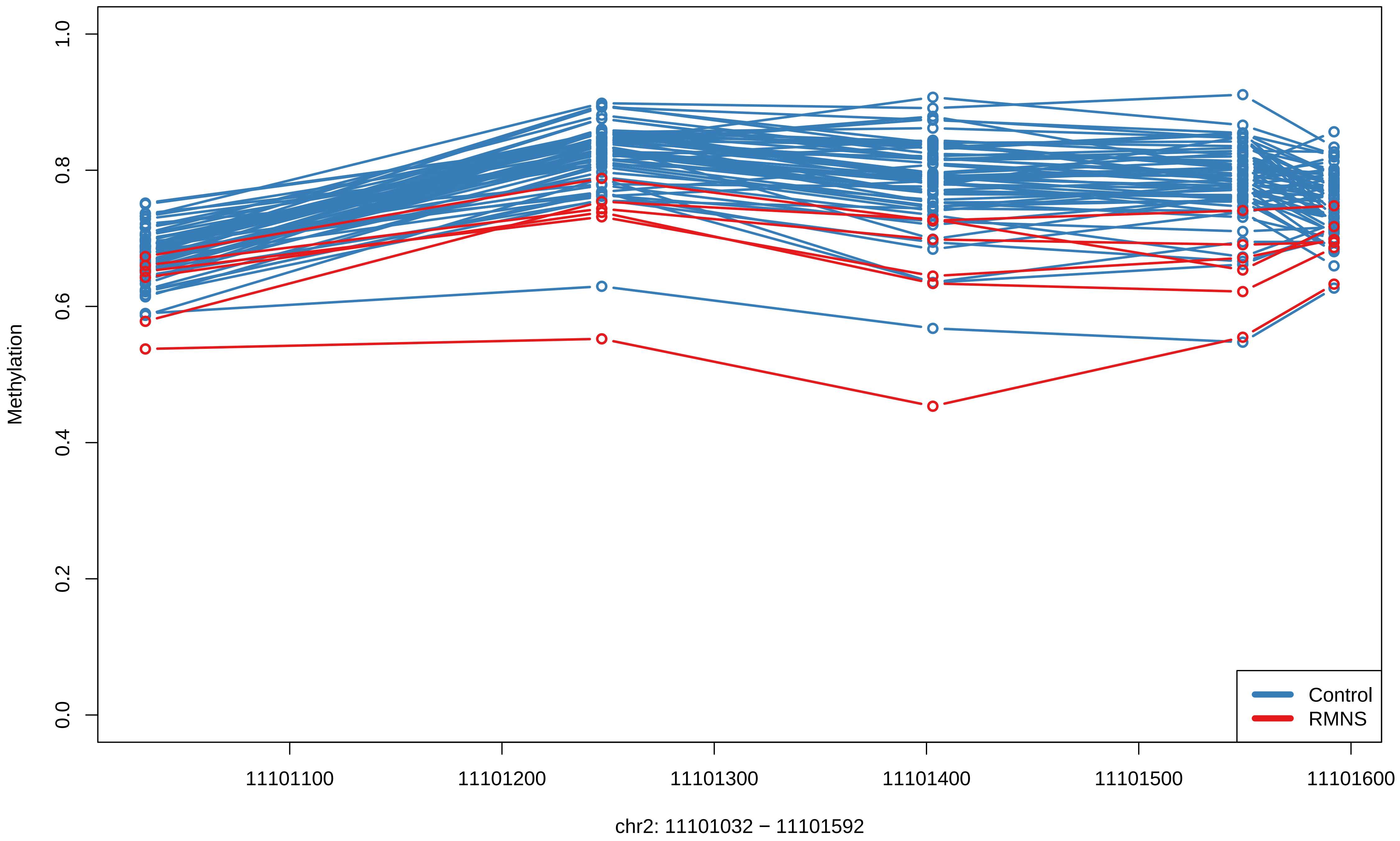

Methylation
0.0
0.2
0.4
0.6
0.8
1.0
11101100
11101200
11101300
11101400
11101500
11101600
chr2: 11101032 − 11101592
Control
RMNS

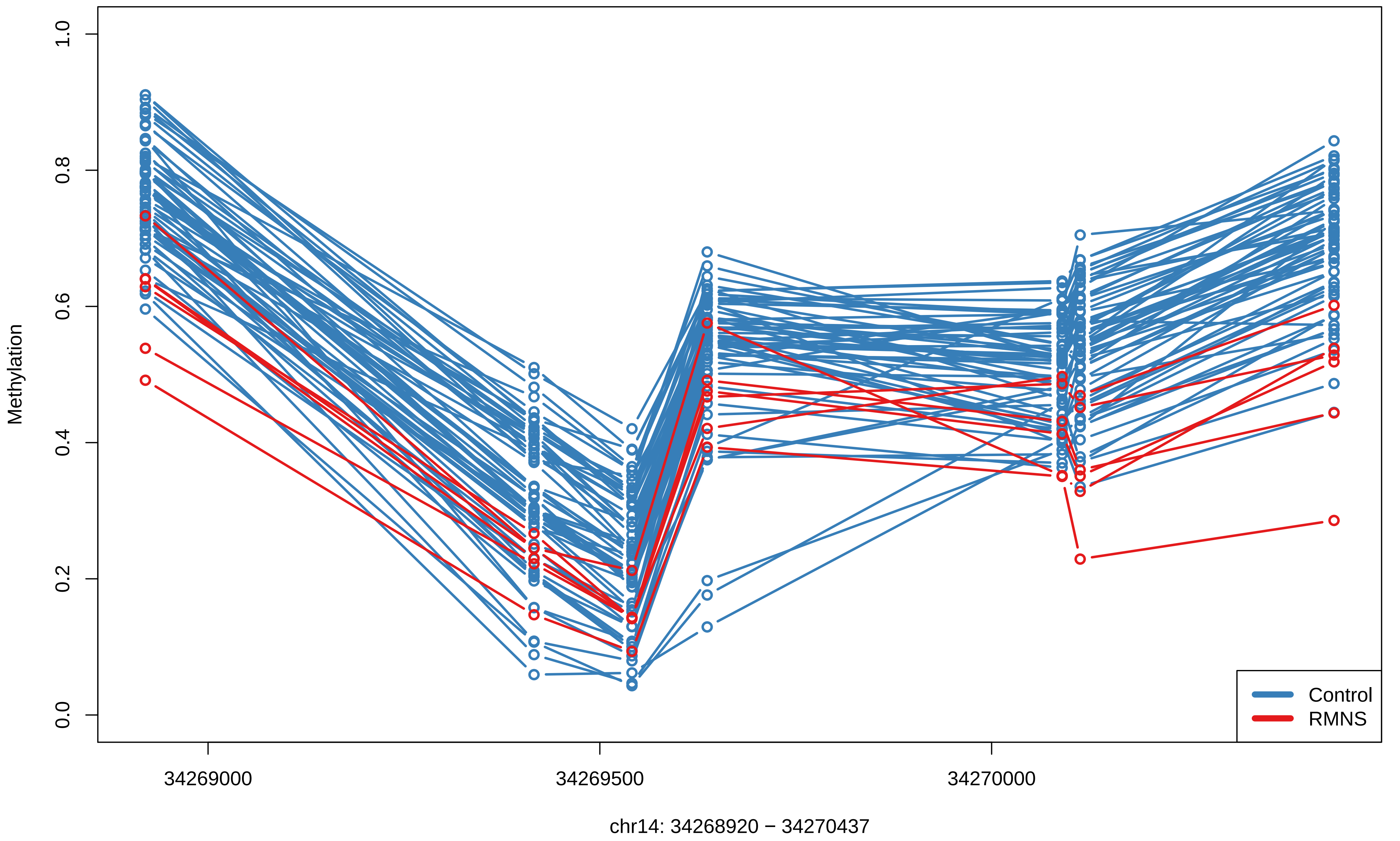

Methylation
0.0
0.2
0.4
0.6
0.8
1.0
34269000
34269500
34270000
chr14: 34268920 − 34270437
Control
RMNS

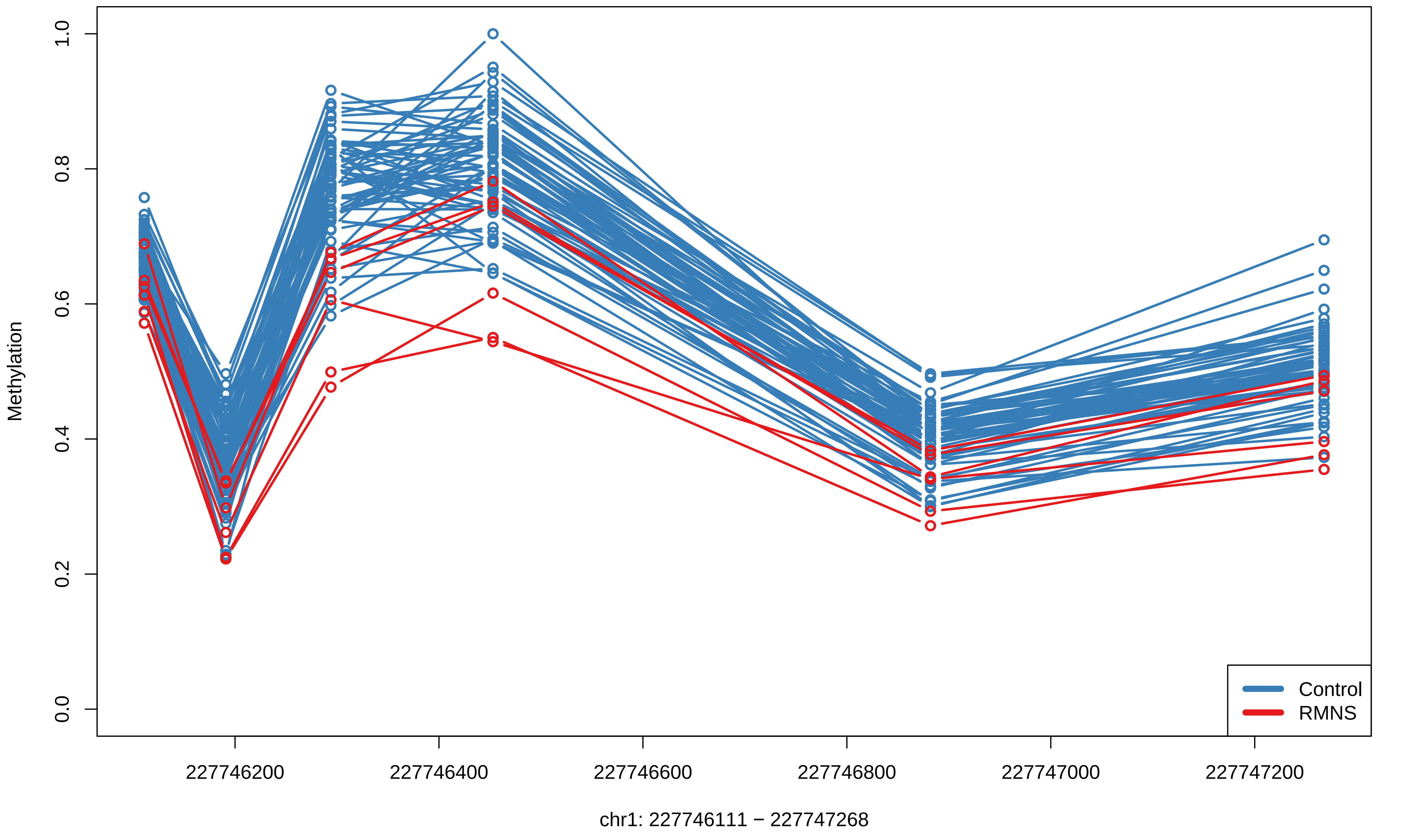

Methylation
0.0
0.2
0.4
0.6
0.8
1.0
227746200
227746400
227746600
227746800
227747000
227747200
chr1: 227746111 − 227747268
Control
RMNS

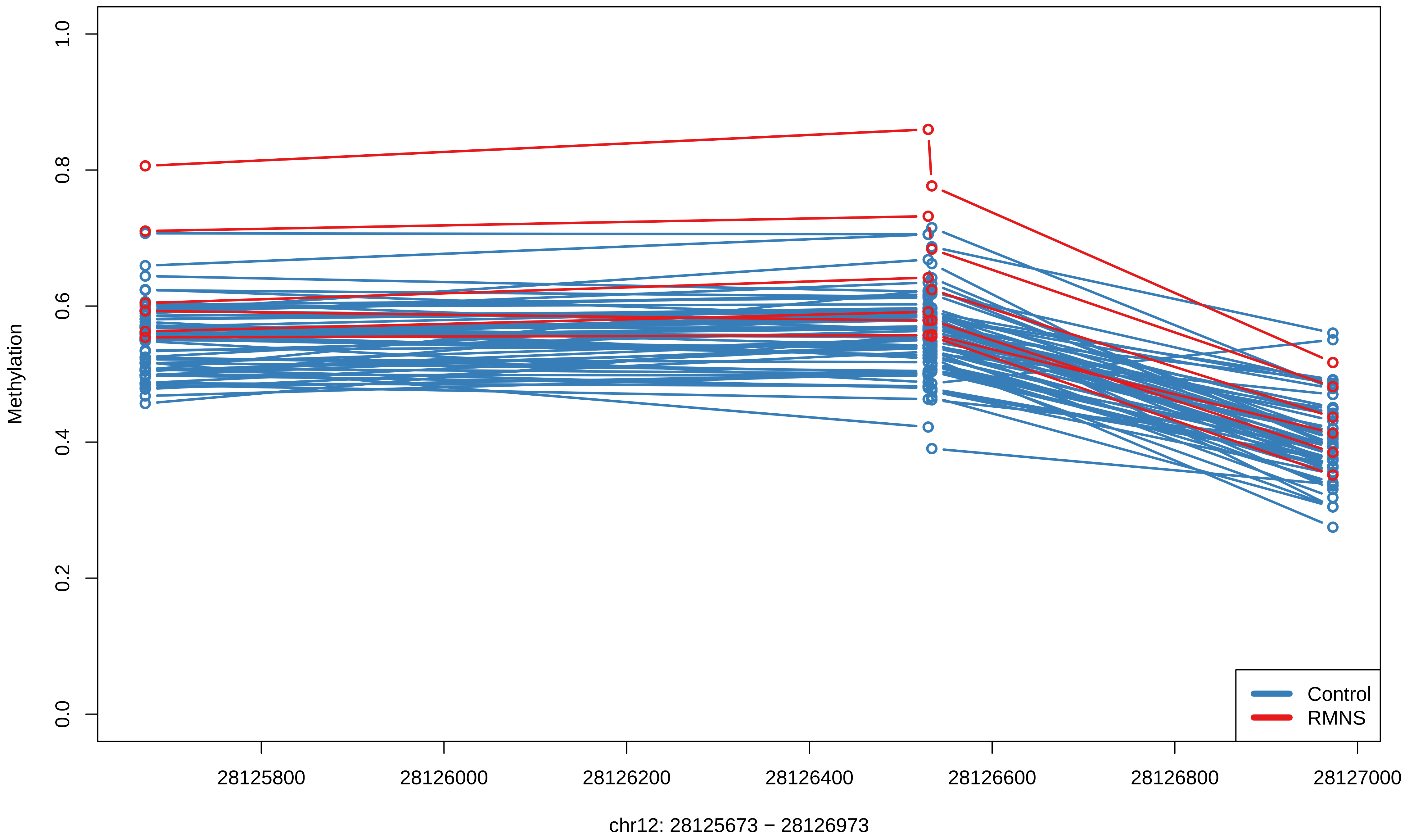

Methylation
0.0
0.2
0.4
0.6
0.8
1.0
28125800
28126000
28126200
28126400
28126600
28126800
28127000
chr12: 28125673 − 28126973
Control
RMNS

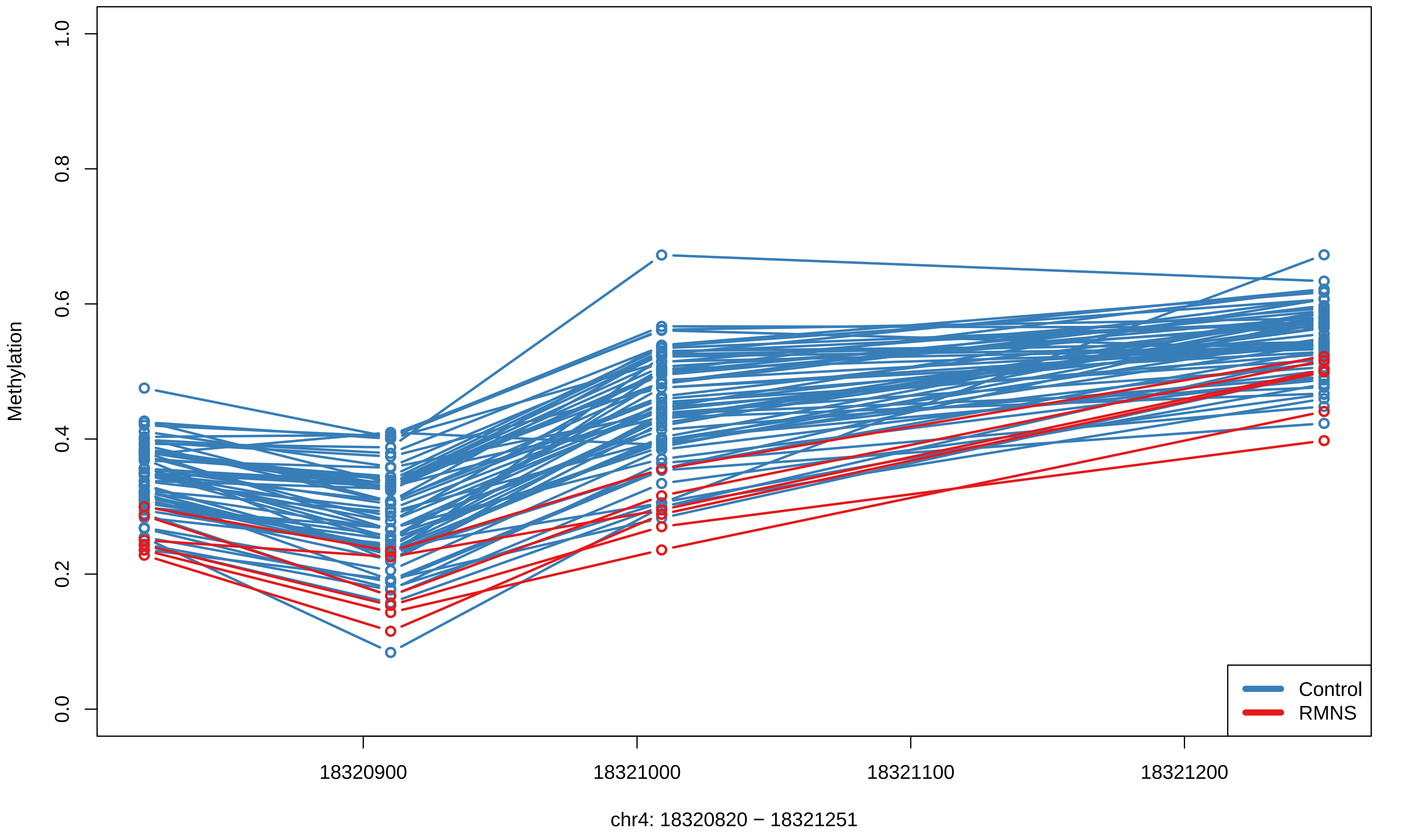

Methylation
1.0
0.8
0.6
0.4
0.2
0.0
18320900
18321000
18321100
18321200
chr4: 18320820 − 18321251
Control
RMNS

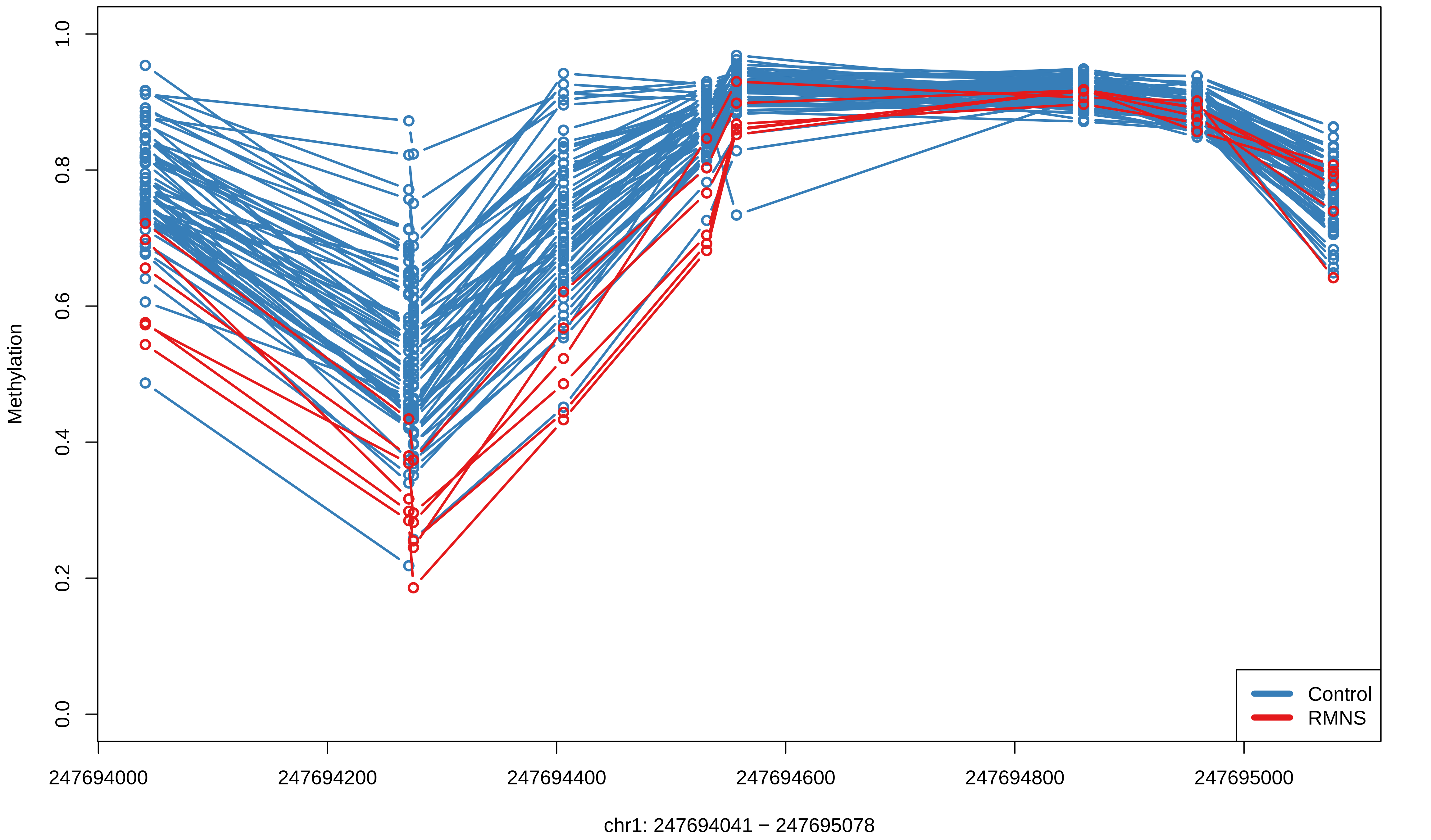
Methylation
1.0
0.8
0.6
0.4
0.2
0.0
247694000
247694200
247694400
247694600
247694800
247695000
chr1: 247694041 − 247695078
Control
RMNS

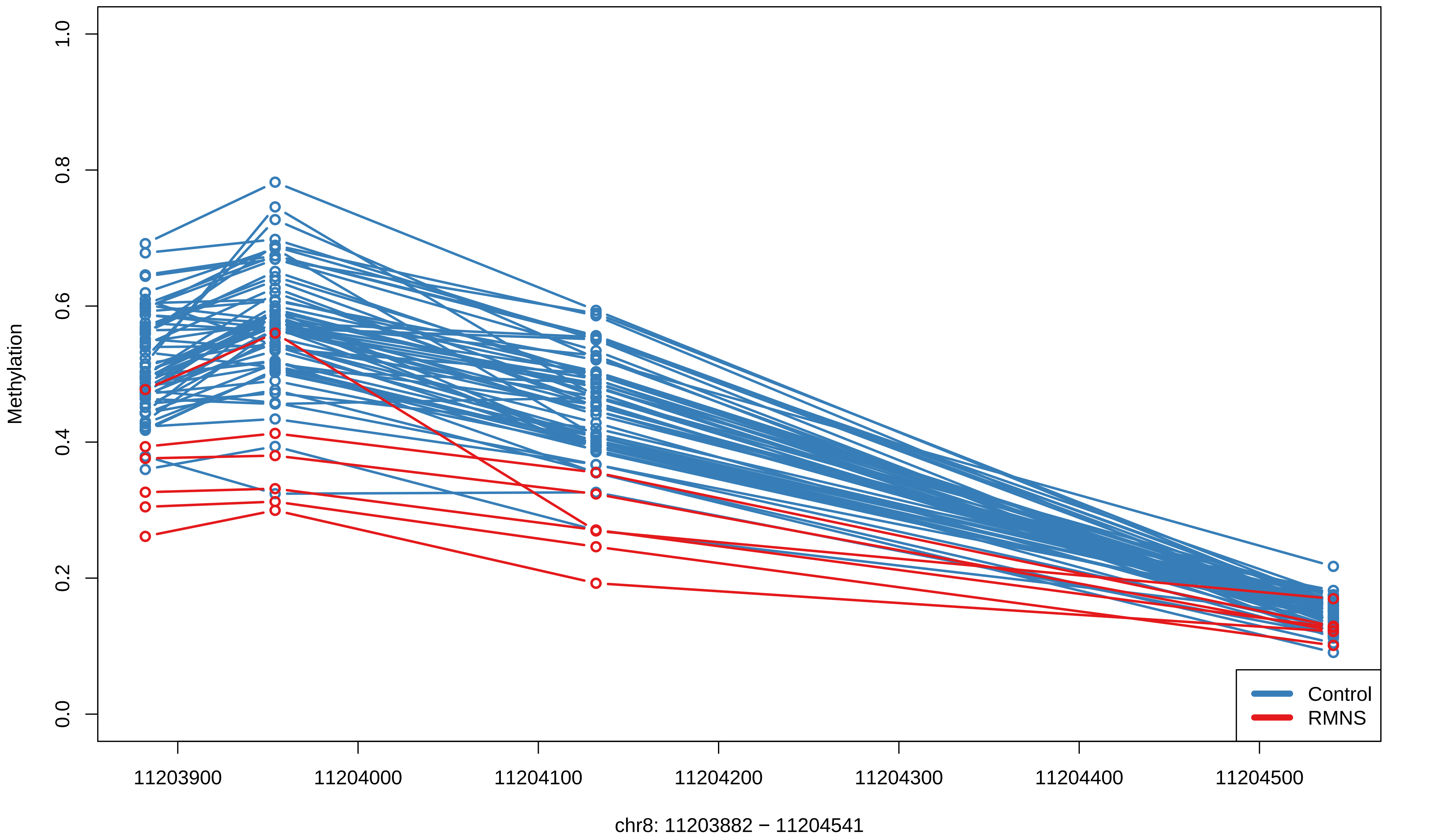

Methylation
0.0
0.2
0.4
0.6
0.8
1.0
11203900
11204000
11204100
11204200
11204300
11204400
11204500
chr8: 11203882 − 11204541
Control
RMNS

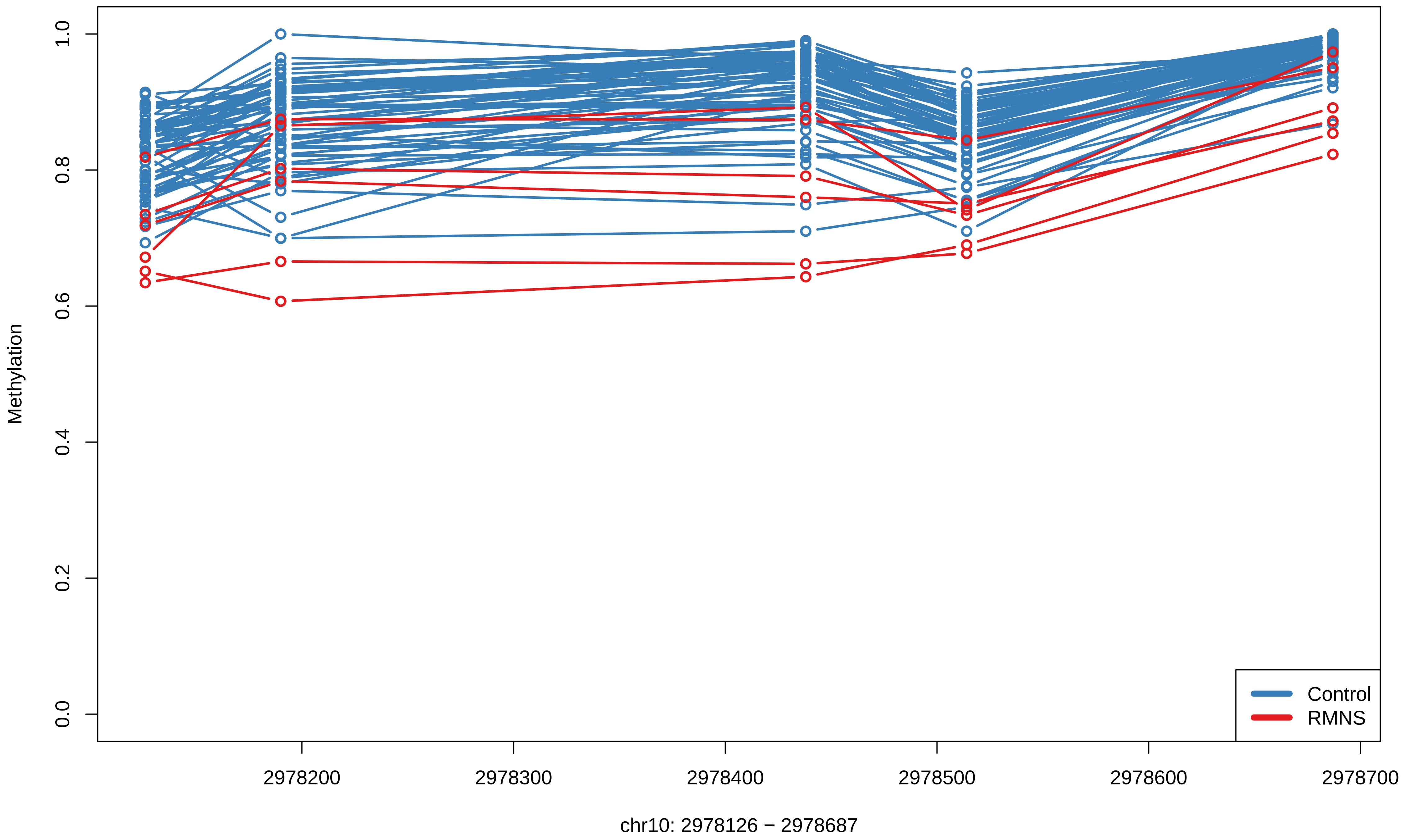

Methylation
1.0
0.8
0.6
0.4
0.2
0.0
2978200
2978300
2978400
2978500
2978600
2978700
chr10: 2978126 − 2978687
Control
RMNS

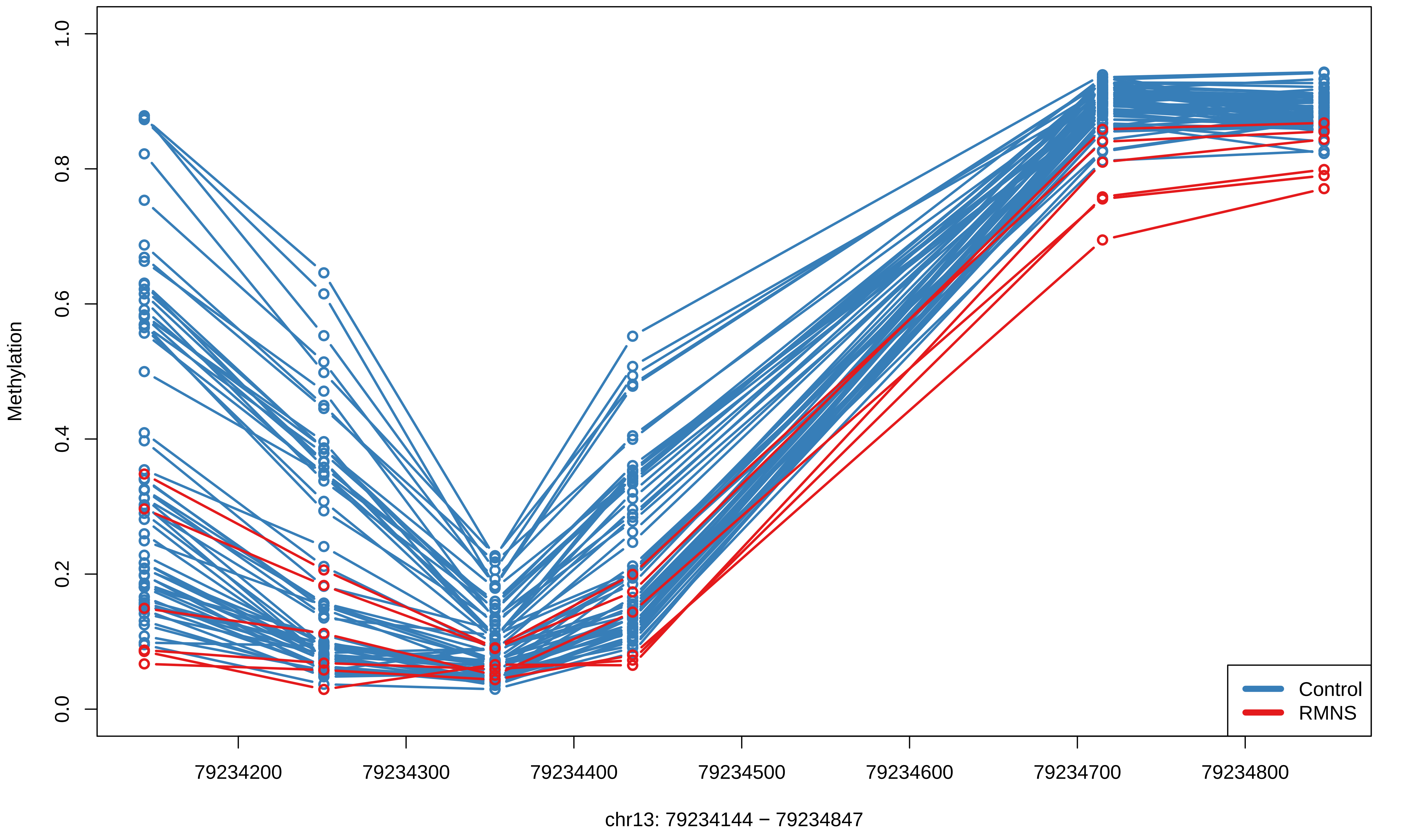

Methylation
1.0
0.8
0.6
0.4
0.2
0.0
79234200
79234300
79234400
79234500
79234600
79234700
79234800
chr13: 79234144 − 79234847
Control
RMNS

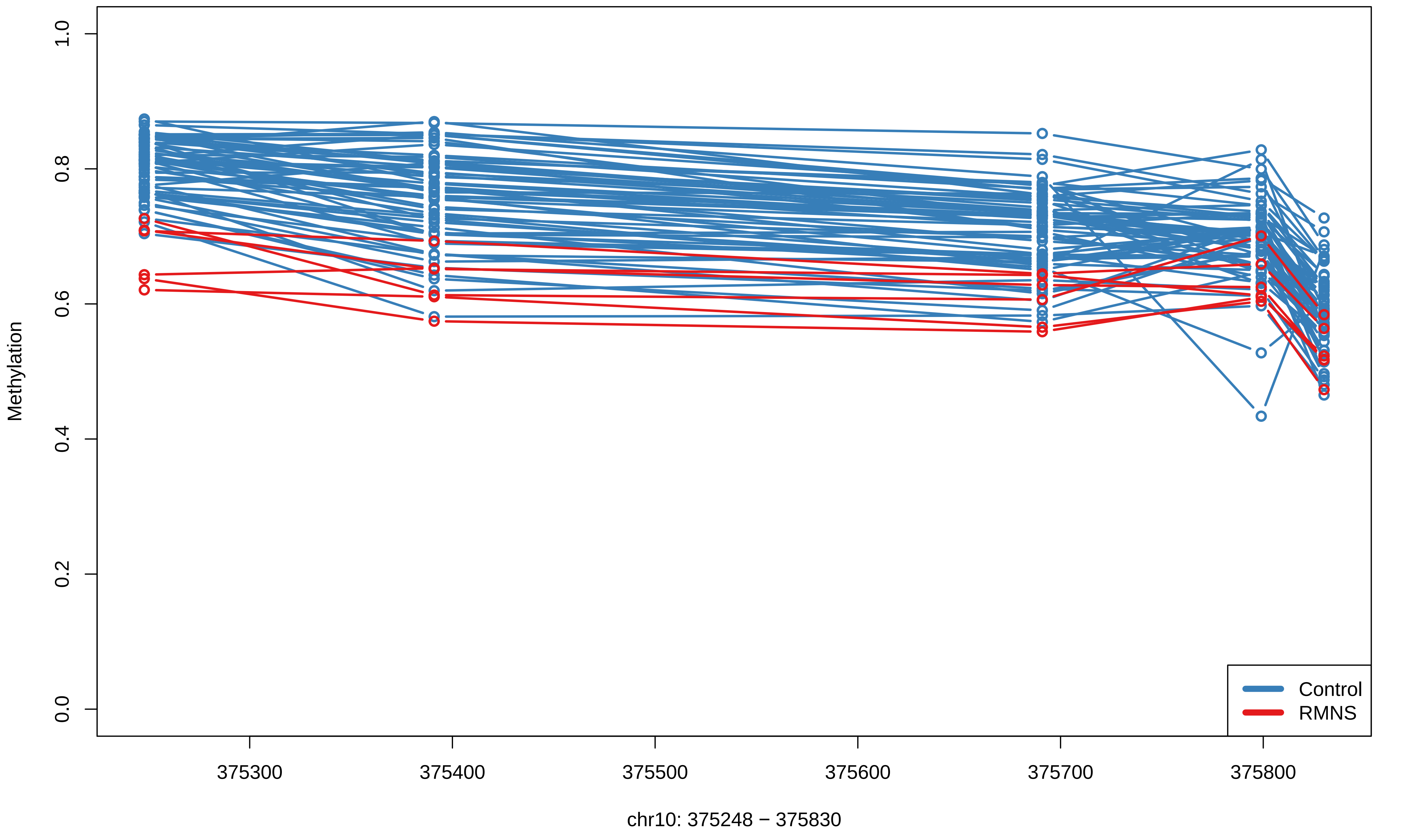

Methylation
1.0
0.8
0.6
0.4
0.2
0.0
375300
375400
375500
375600
375700
375800
chr10: 375248 − 375830
Control
RMNS

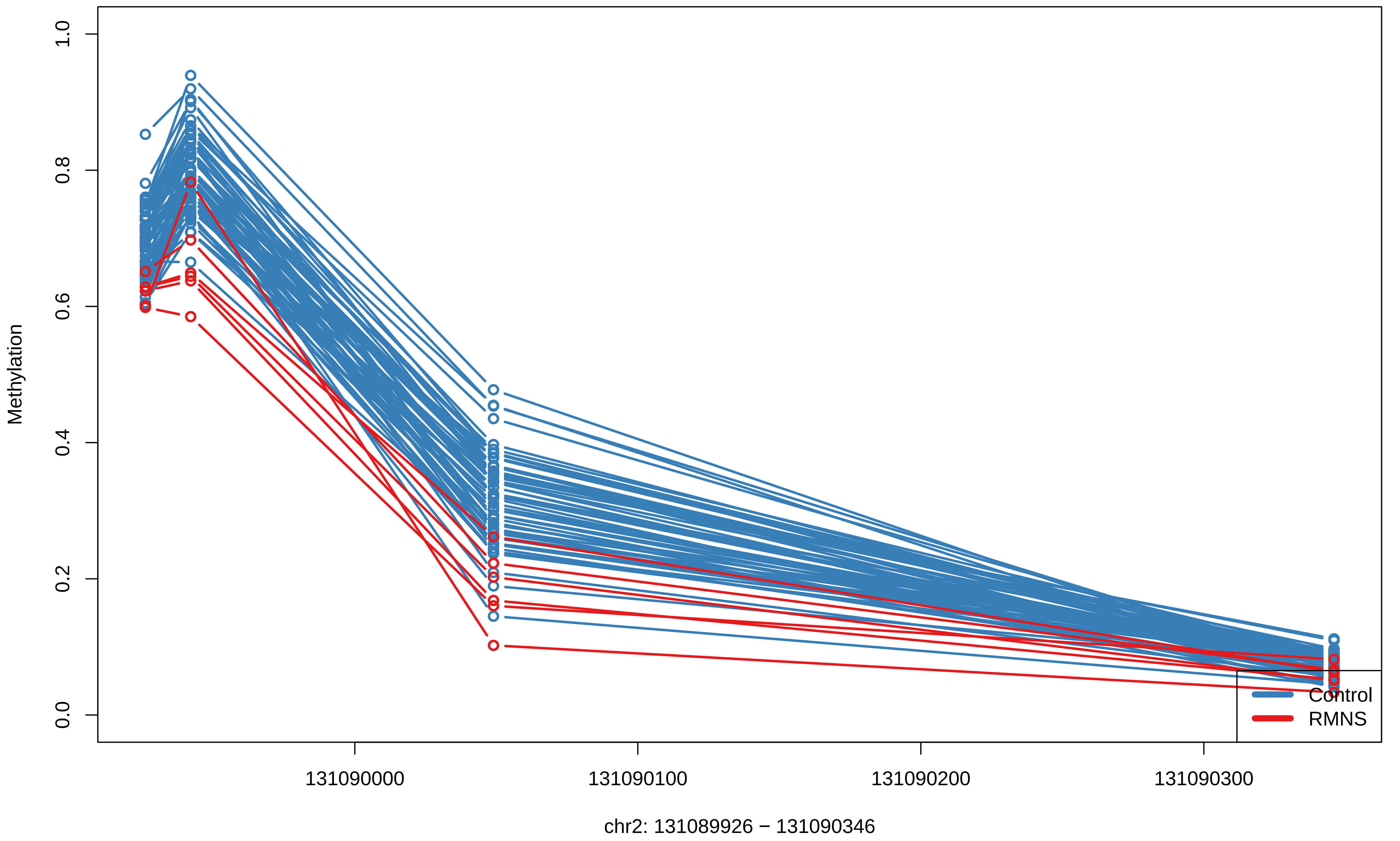
Methylation
0.0
0.2
0.4
0.6
0.8
1.0
131090000
131090100
131090200
131090300
chr2: 131089926 − 131090346
Control
RMNS

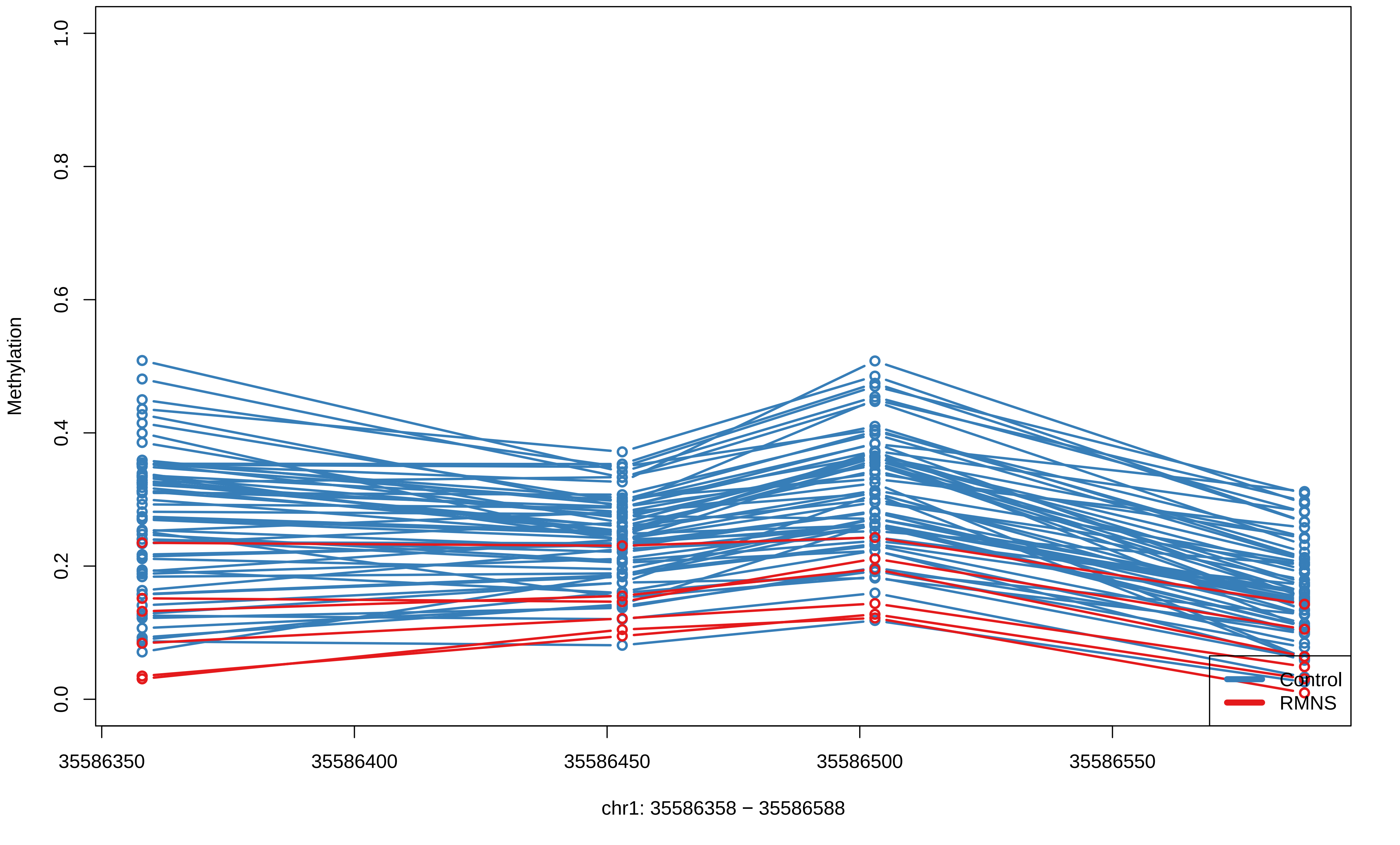

1.0
0.8
0.6
0.4
0.2
0.0
Methylation
Control
RMNS
35586350
35586400
35586450
35586500
35586550
chr1: 35586358 − 35586588

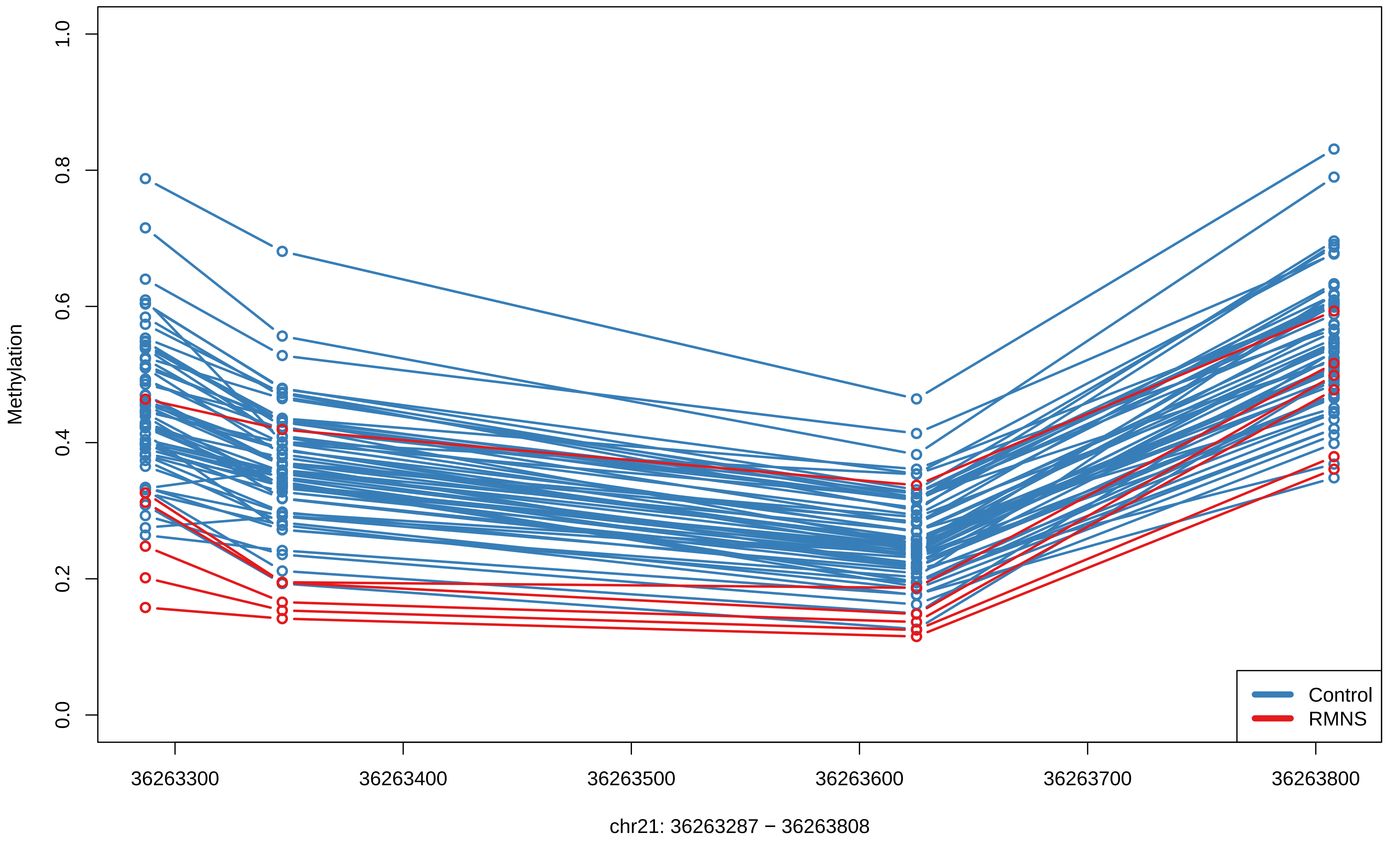

1.0
0.8
0.6
0.4
0.2
0.0
Methylation
36263300
36263400
36263500
36263600
36263700
36263800
chr21: 36263287 − 36263808
Control
RMNS

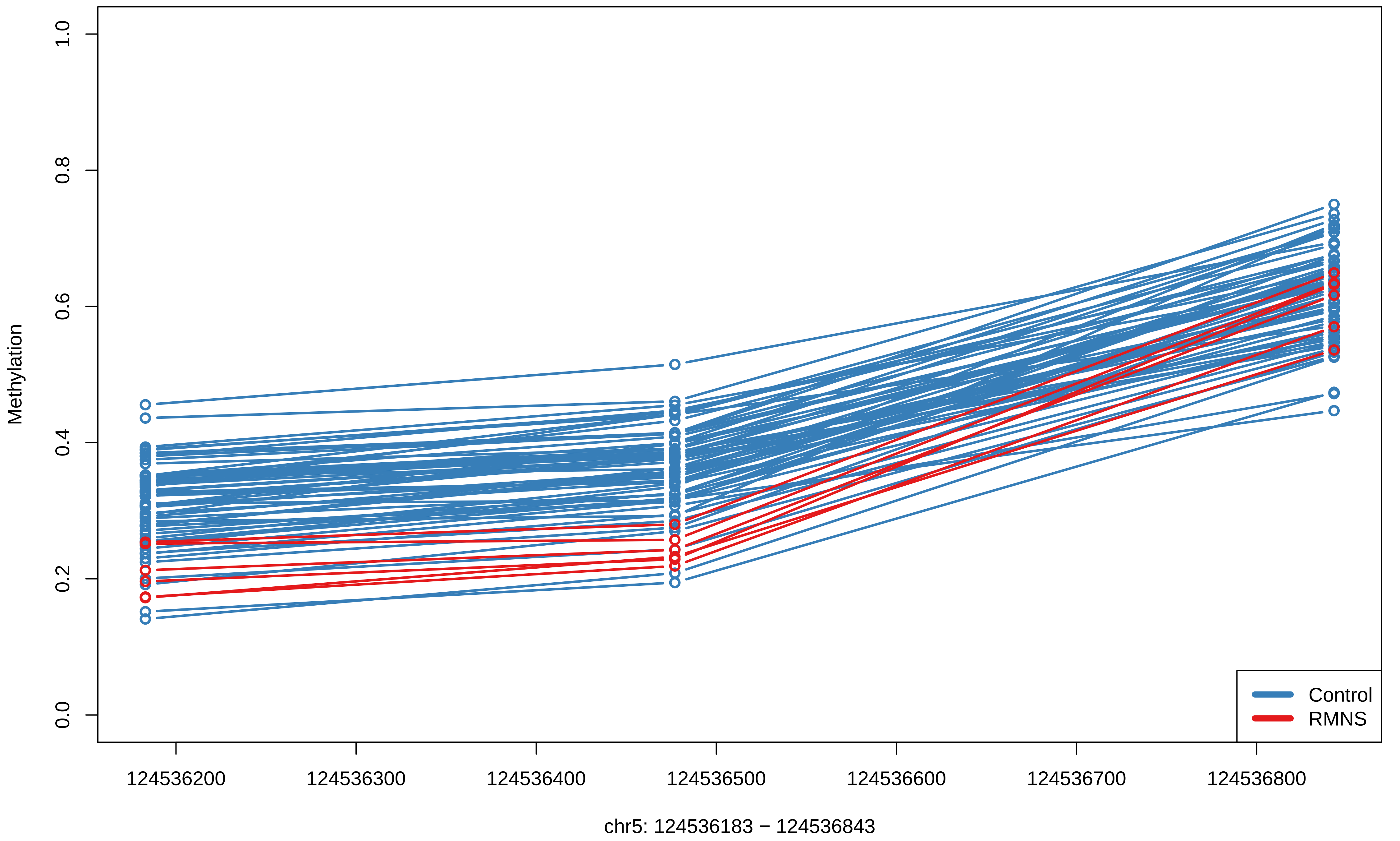

Methylation
0.0
0.2
0.4
0.6
0.8
1.0
124536200
124536300
124536400
124536500
124536600
124536700
124536800
chr5: 124536183 − 124536843
Control
RMNS

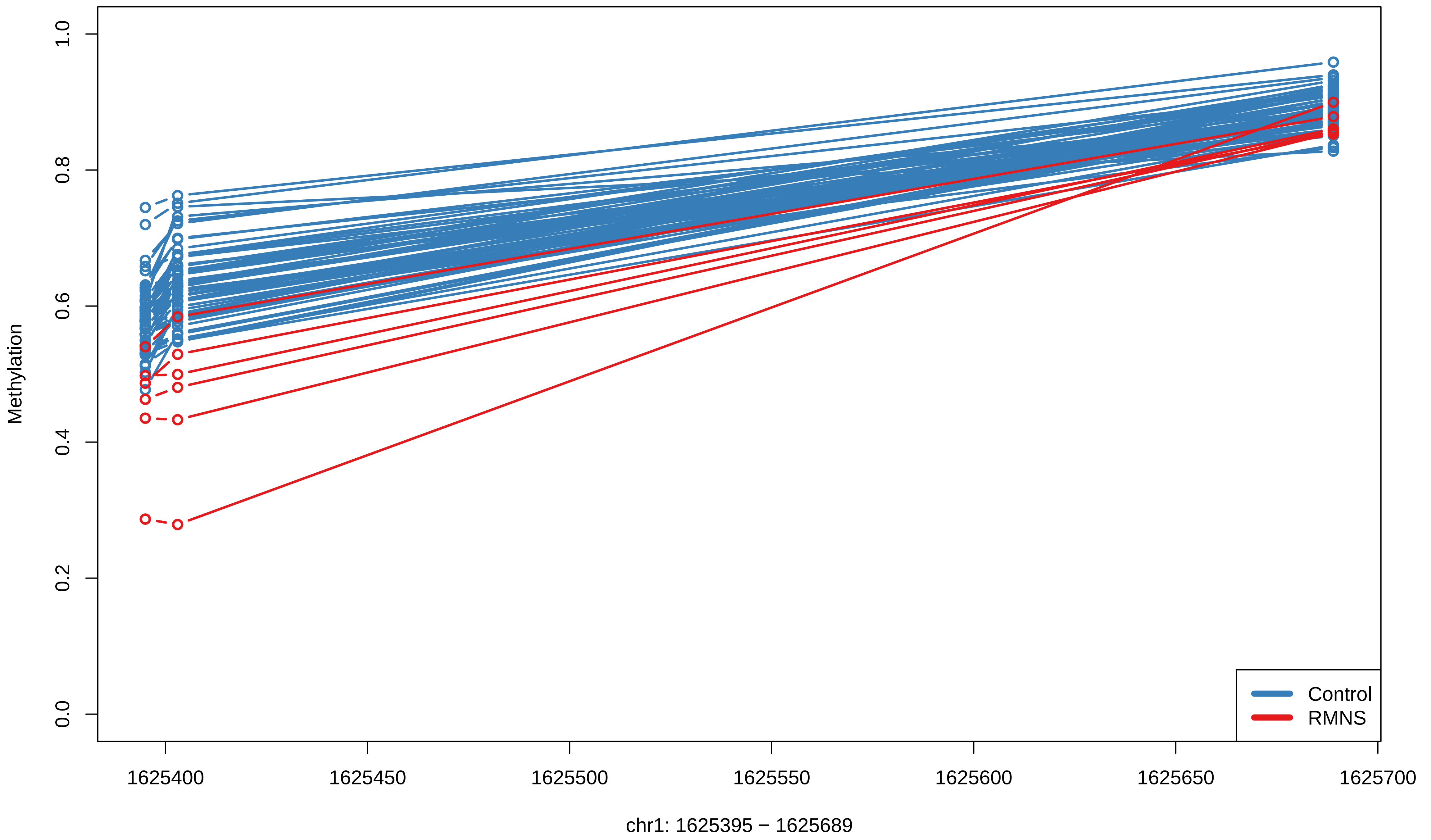
Methylation
0.0
0.2
0.4
0.6
0.8
1.0
1625400
1625450
1625500
1625550
1625600
1625650
1625700
chr1: 1625395 − 1625689
Control
RMNS

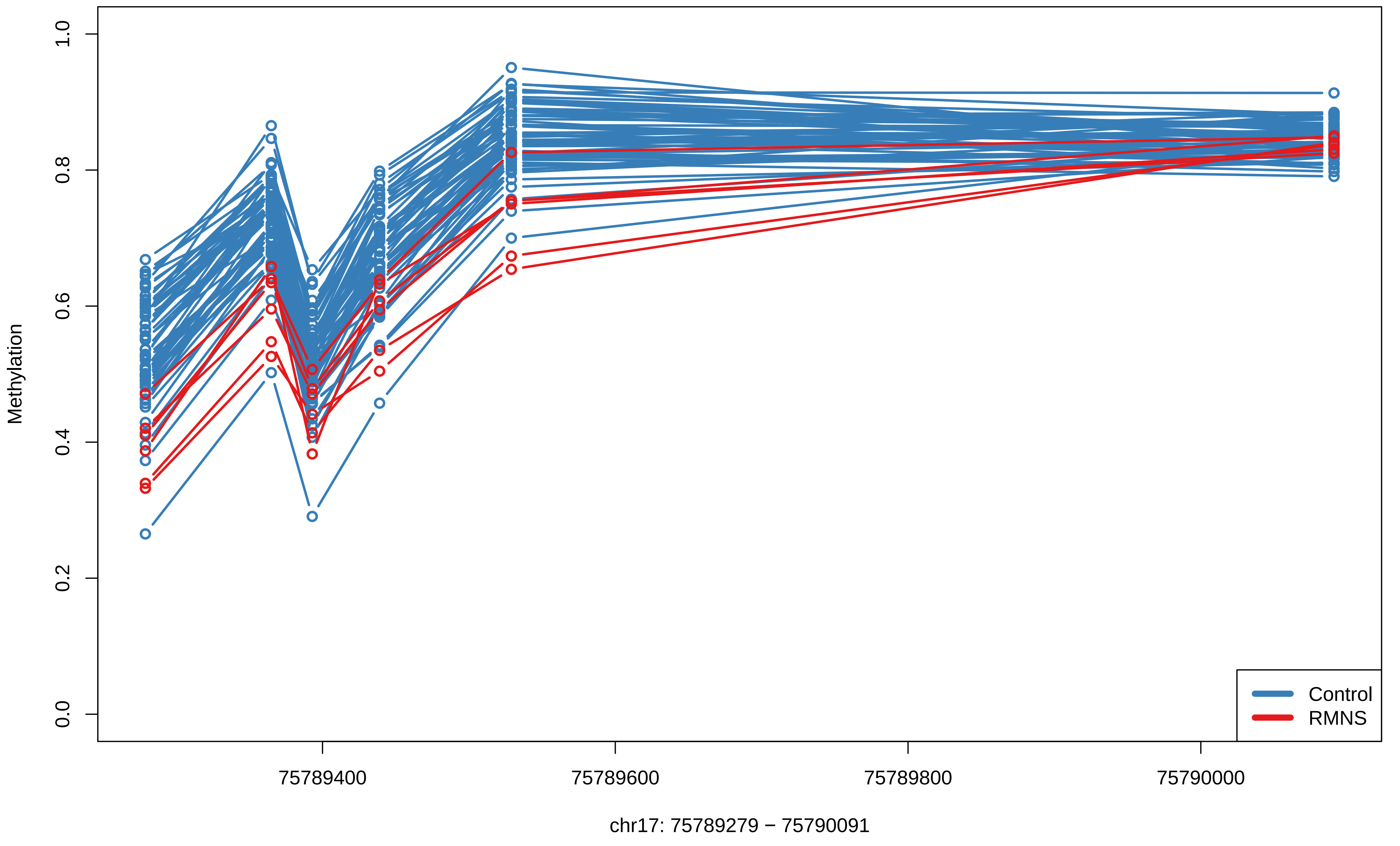

Methylation
1.0
0.8
0.6
0.4
0.2
0.0
75789400
75789600
75789800
75790000
chr17: 75789279 − 75790091
Control
RMNS

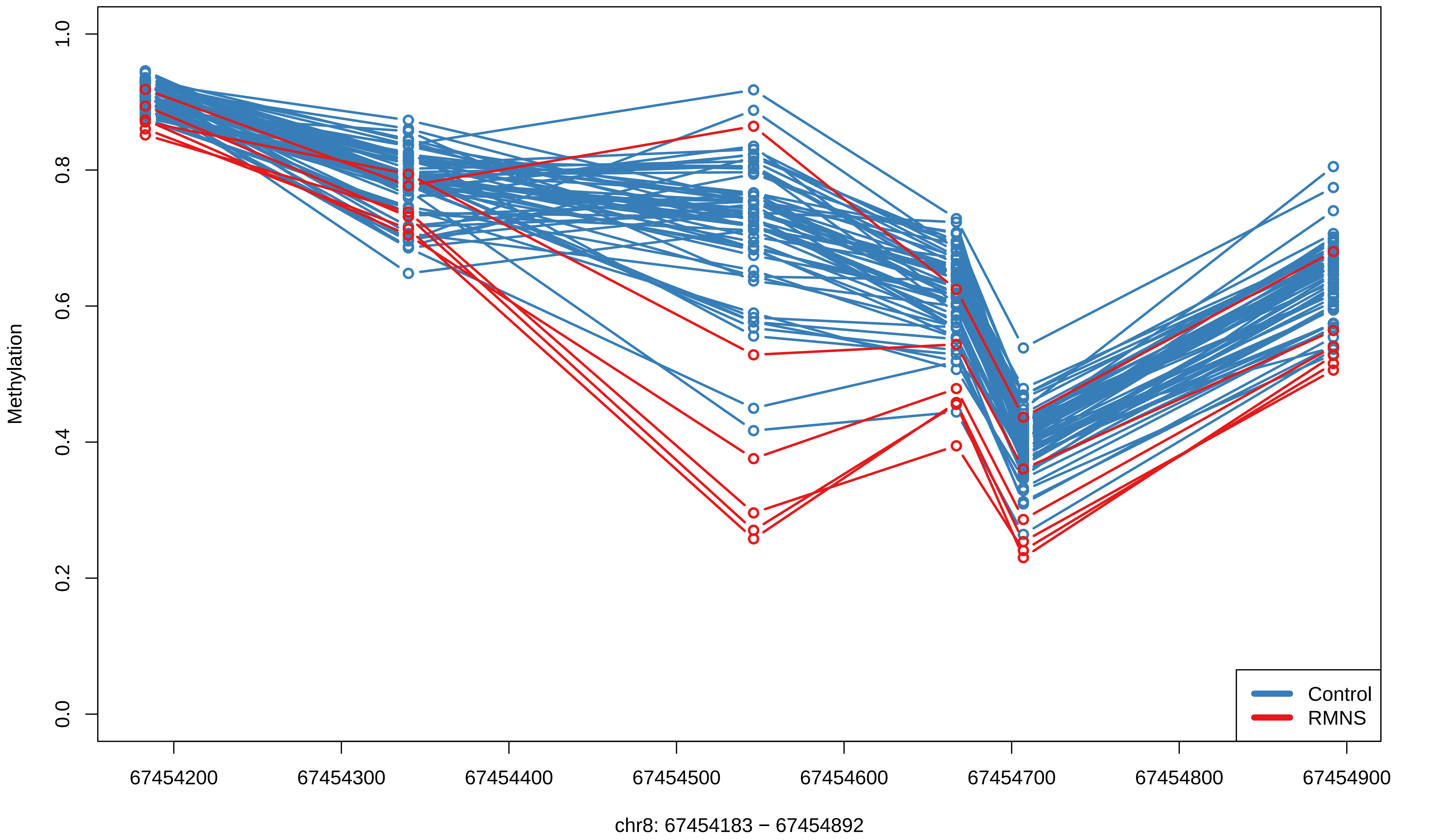

Methylation
1.0
0.8
0.6
0.4
0.2
0.0
67454200
67454300
67454400
67454500
67454600
67454700
67454800
67454900
chr8: 67454183 − 67454892
Control
RMNS

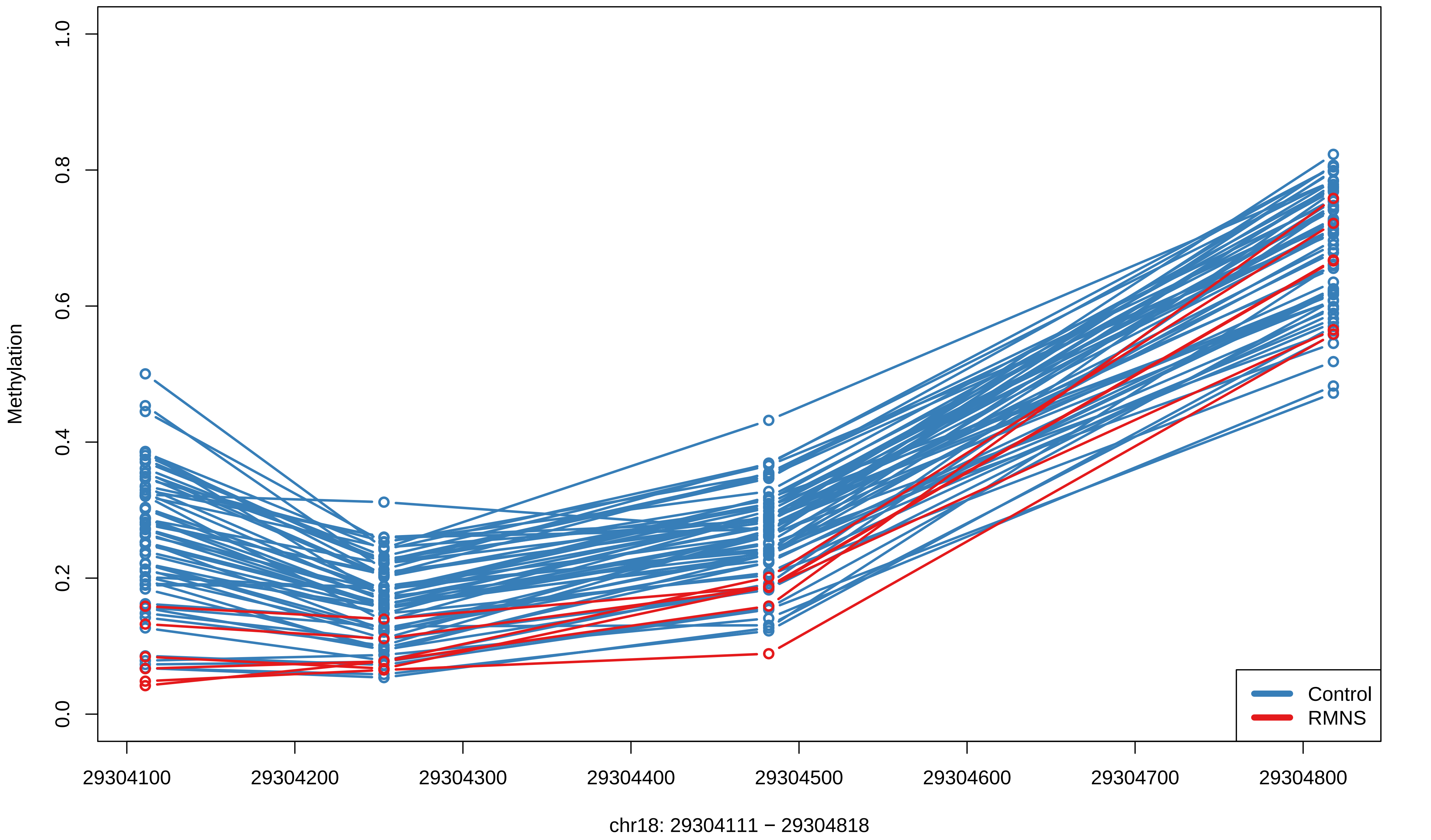

Methylation
0.0
0.2
0.4
0.6
0.8
1.0
29304100
29304200
29304300
29304400
29304500
29304600
29304700
29304800
chr18: 29304111 − 29304818
Control
RMNS

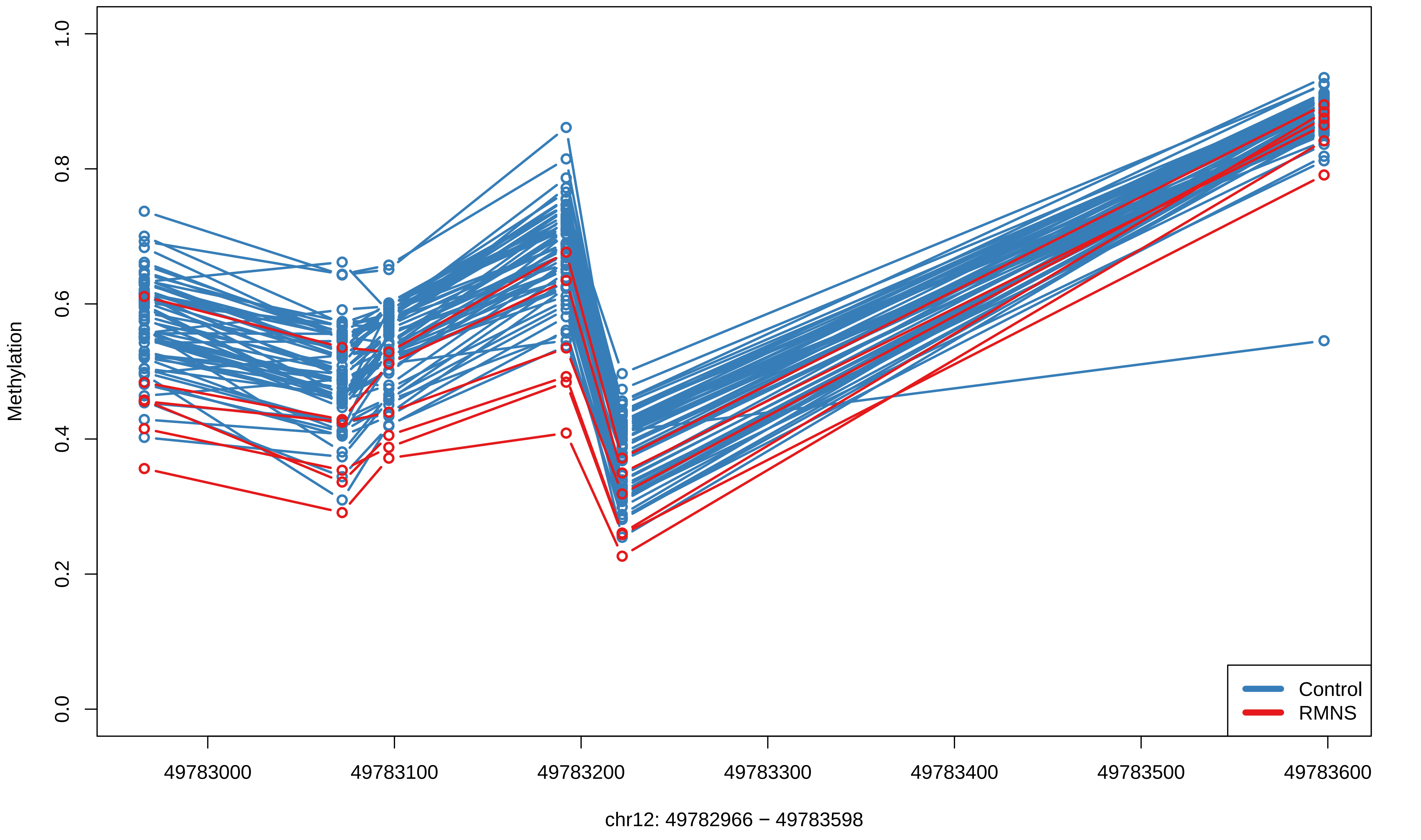

Methylation
0.0
0.2
0.4
0.6
0.8
1.0
49783000
49783100
49783200
49783300
49783400
49783500
49783600
chr12: 49782966 − 49783598
Control
RMNS

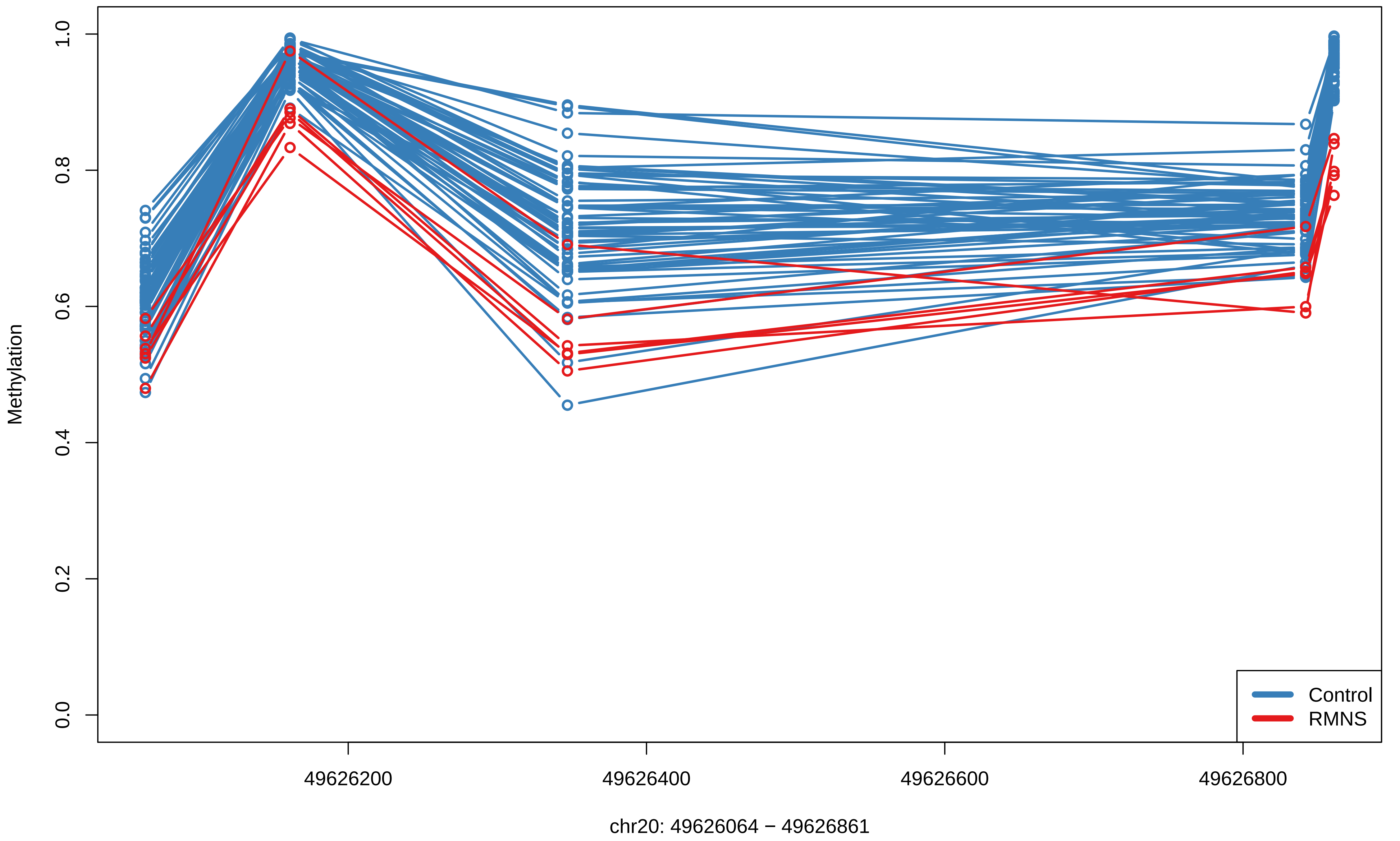

1.0
0.8
0.6
0.4
0.2
0.0
Methylation
49626200
49626400
49626600
49626800
chr20: 49626064 − 49626861
Control
RMNS

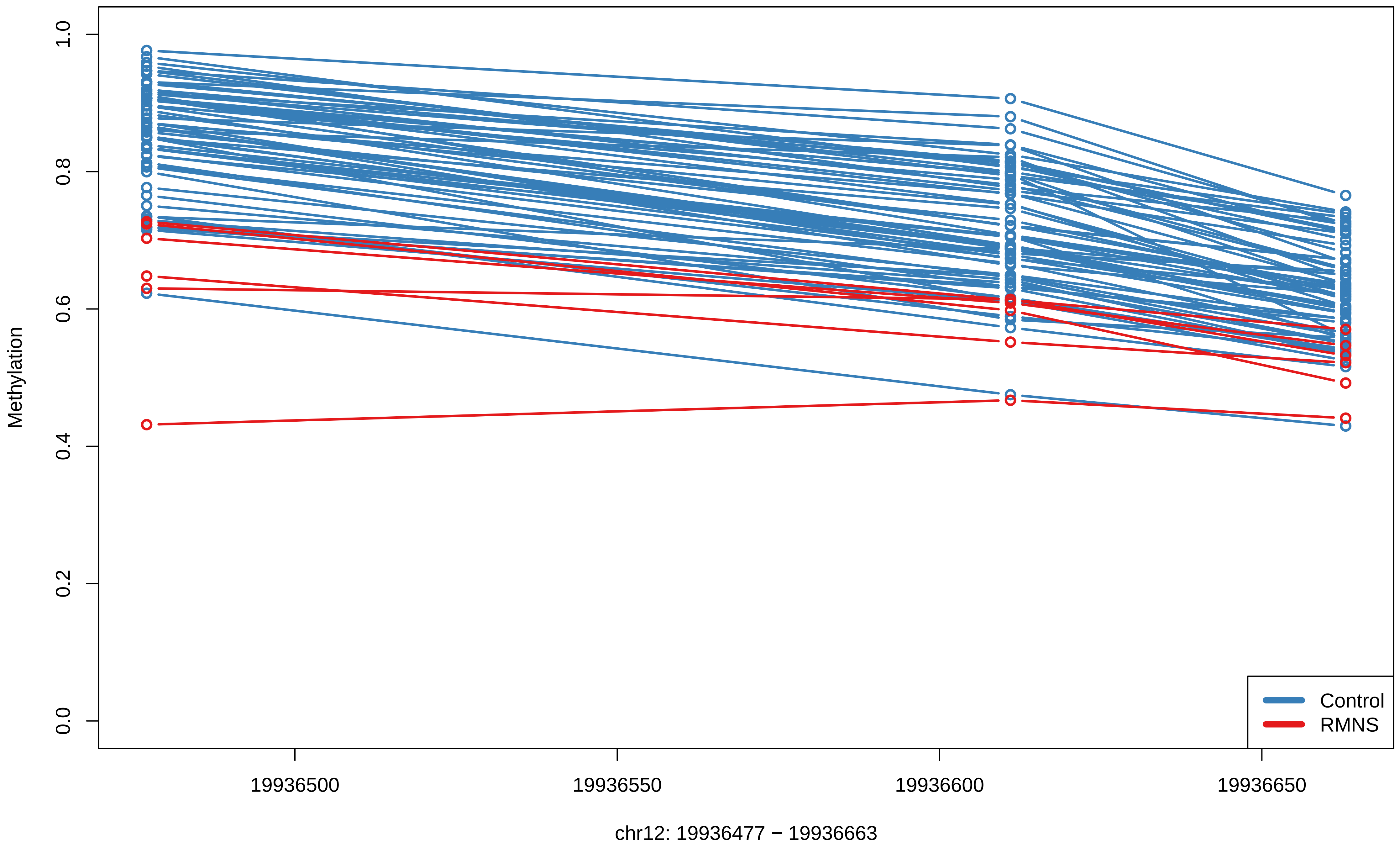

1.0
0.8
0.6
0.4
0.2
0.0
Methylation
19936500
19936550
19936600
19936650
chr12: 19936477 − 19936663
Control
RMNS

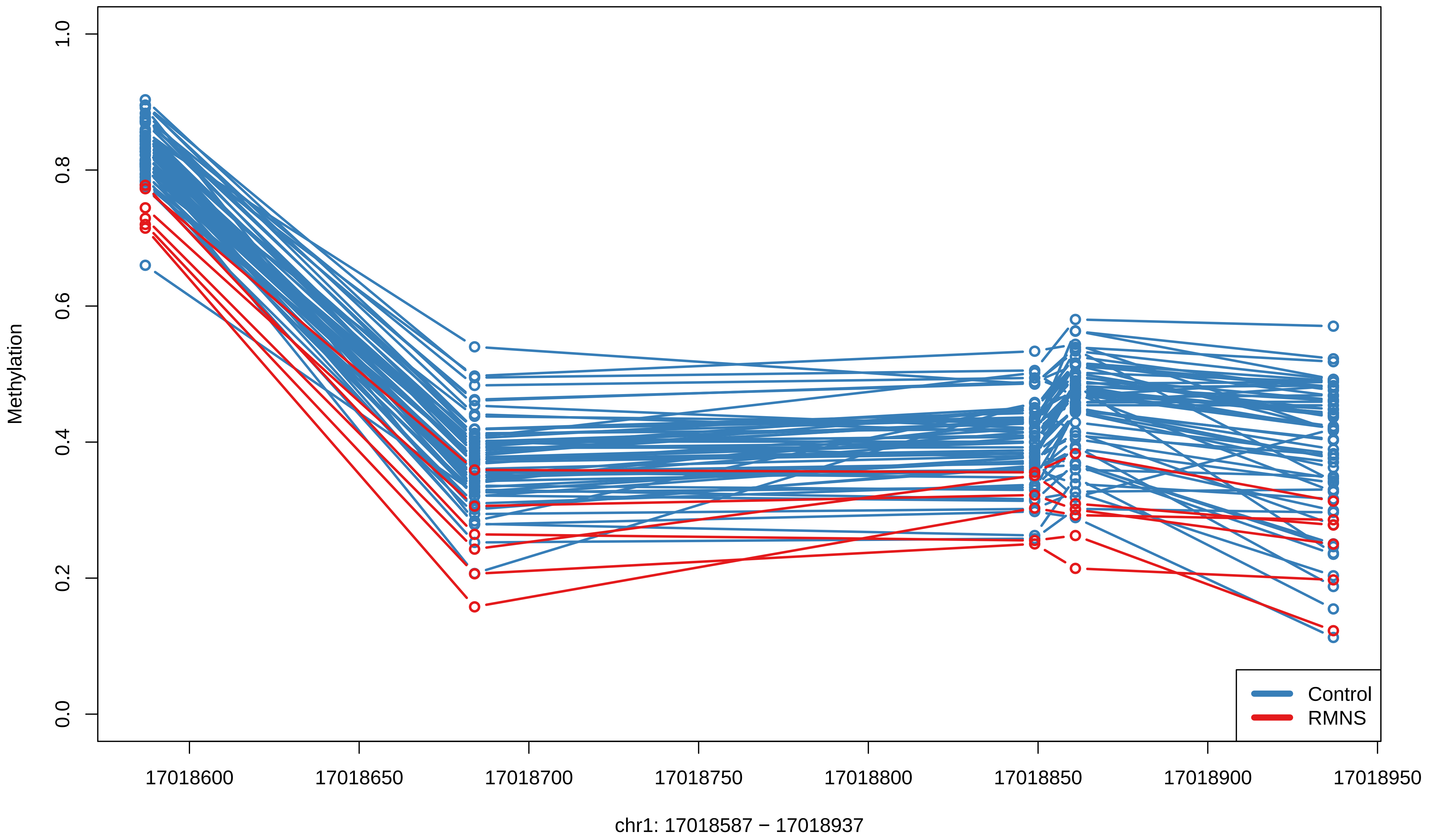
Methylation
0.0
0.2
0.4
0.6
0.8
1.0
17018600
17018650
17018700
17018750
17018800
17018850
17018900
17018950
chr1: 17018587 − 17018937
Control
RMNS

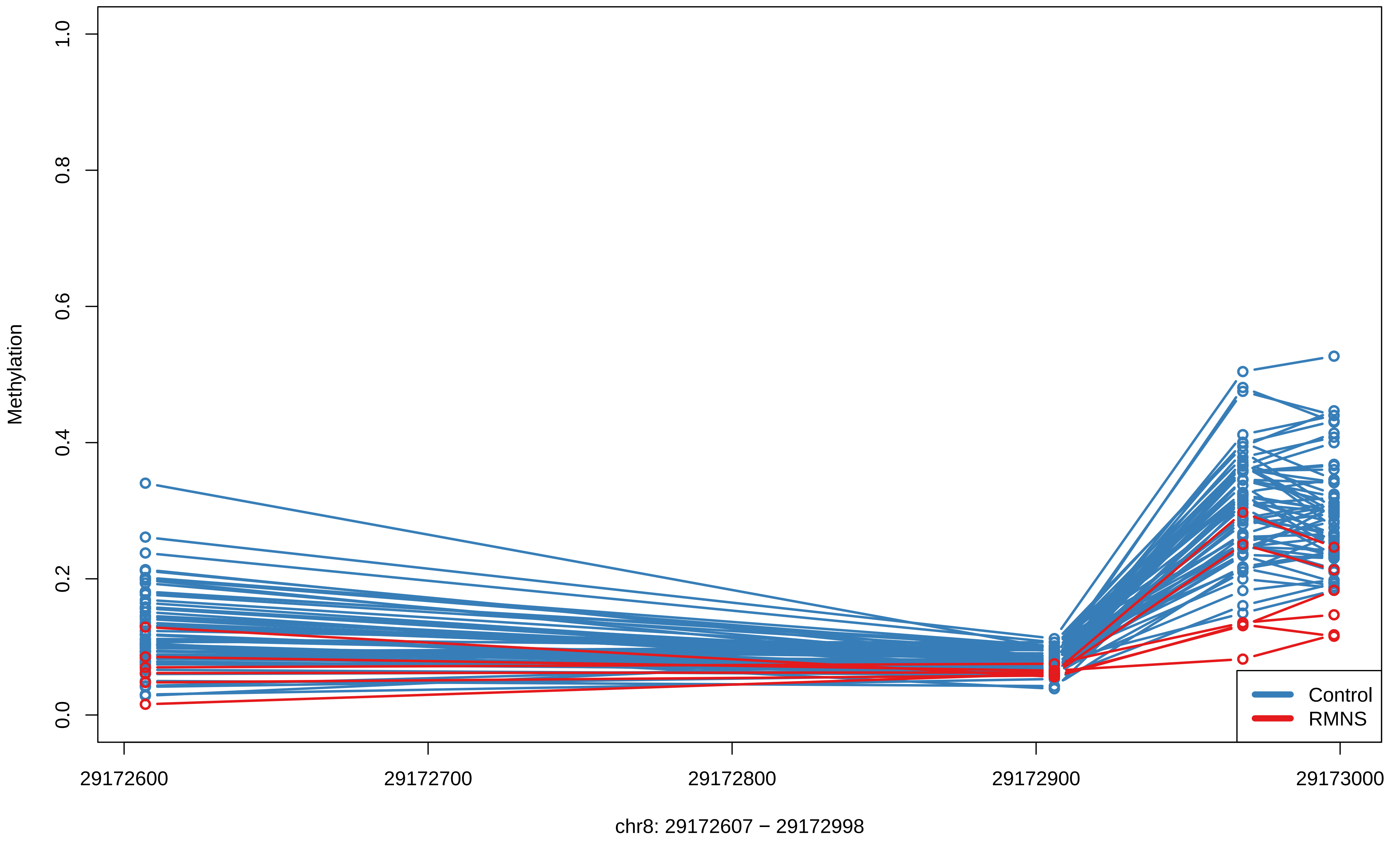

Methylation
0.0
0.2
0.4
0.6
0.8
1.0
29172600
29172700
29172800
29172900
29173000
chr8: 29172607 − 29172998
Control
RMNS

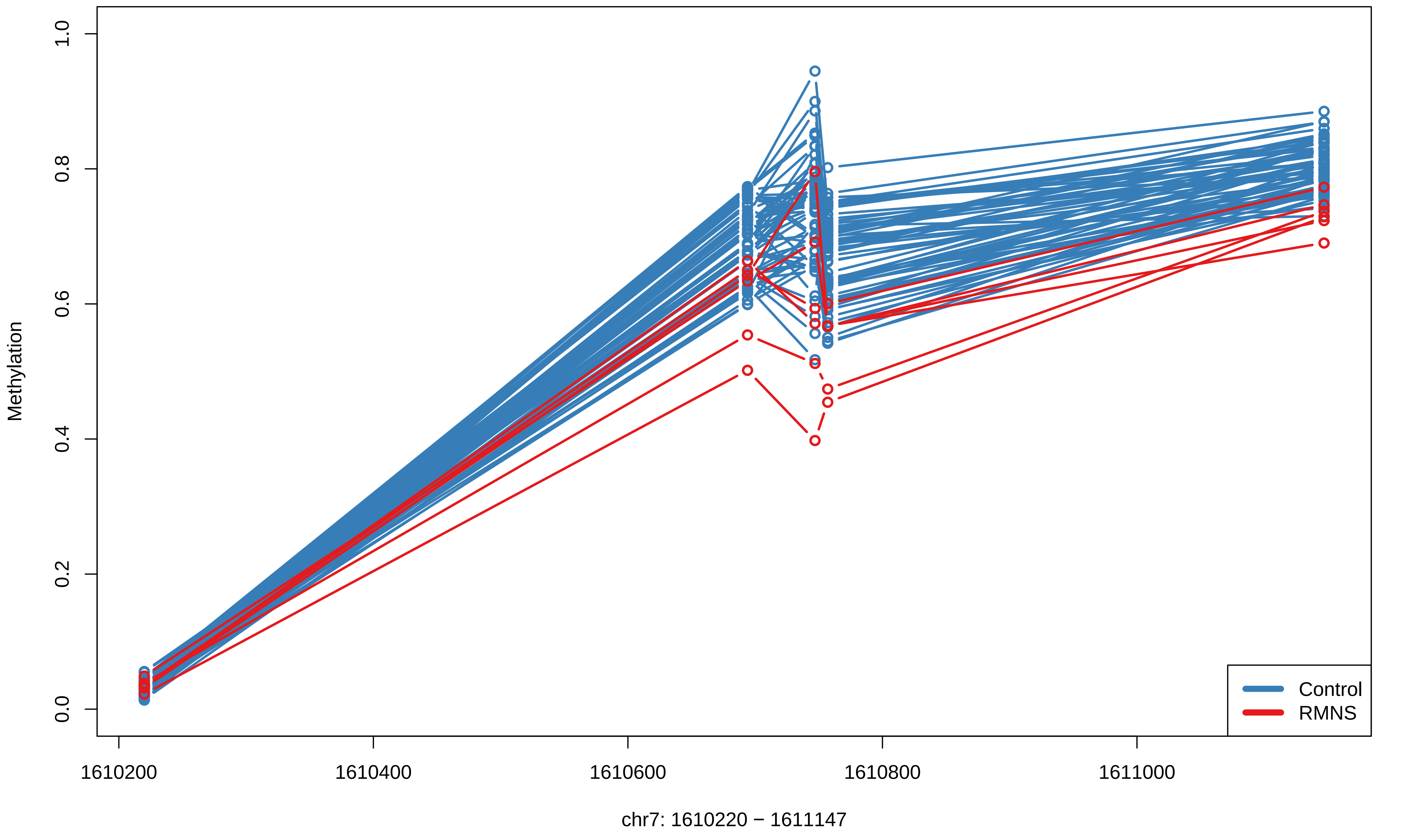

1.0
0.8
0.6
0.4
0.2
0.0
Methylation
1610200
1610400
1610600
1610800
1611000
chr7: 1610220 − 1611147
Control
RMNS

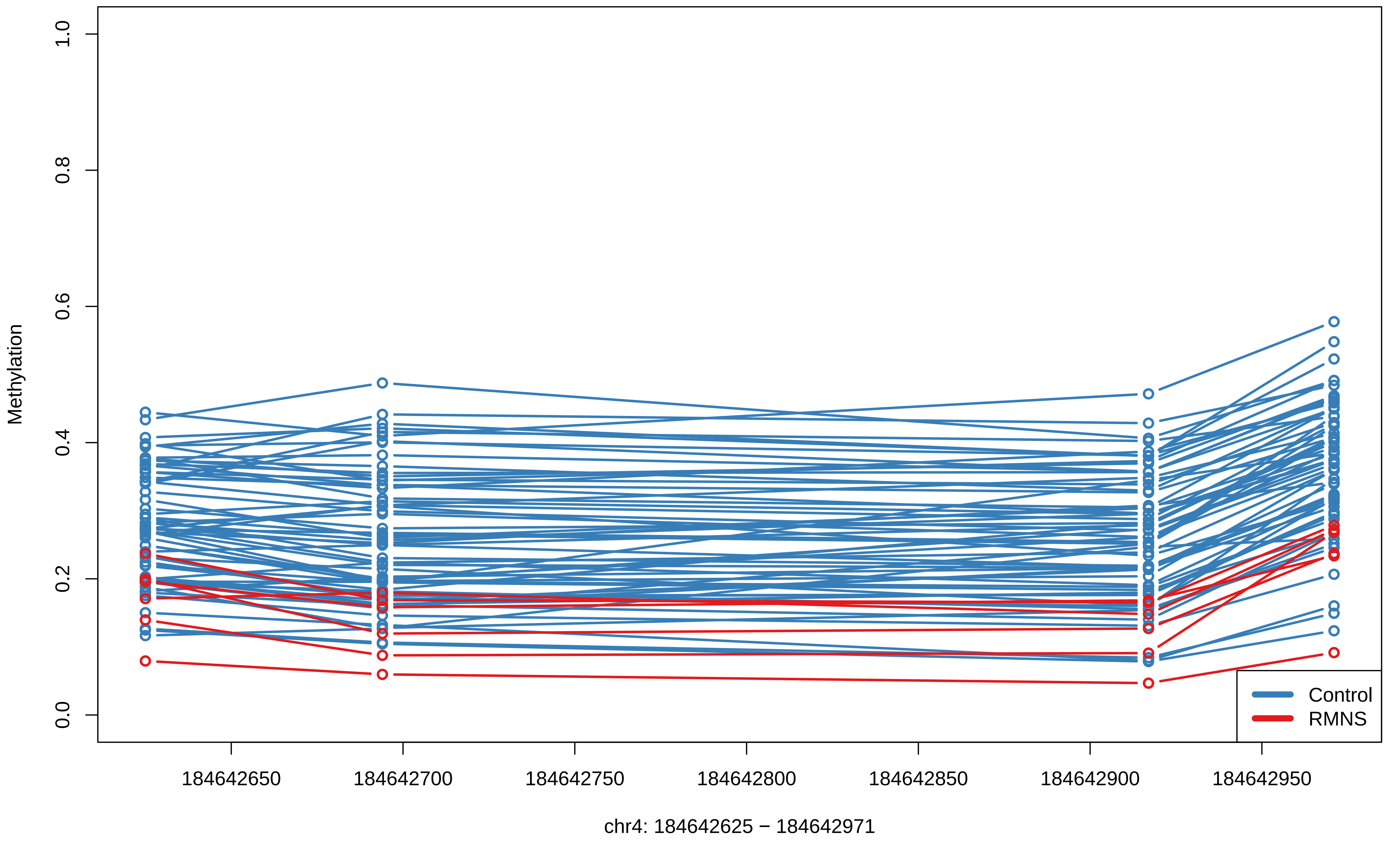

Methylation
0.0
0.2
0.4
0.6
0.8
1.0
184642650
184642700
184642750
184642800
184642850
184642900
184642950
chr4: 184642625 − 184642971
Control
RMNS

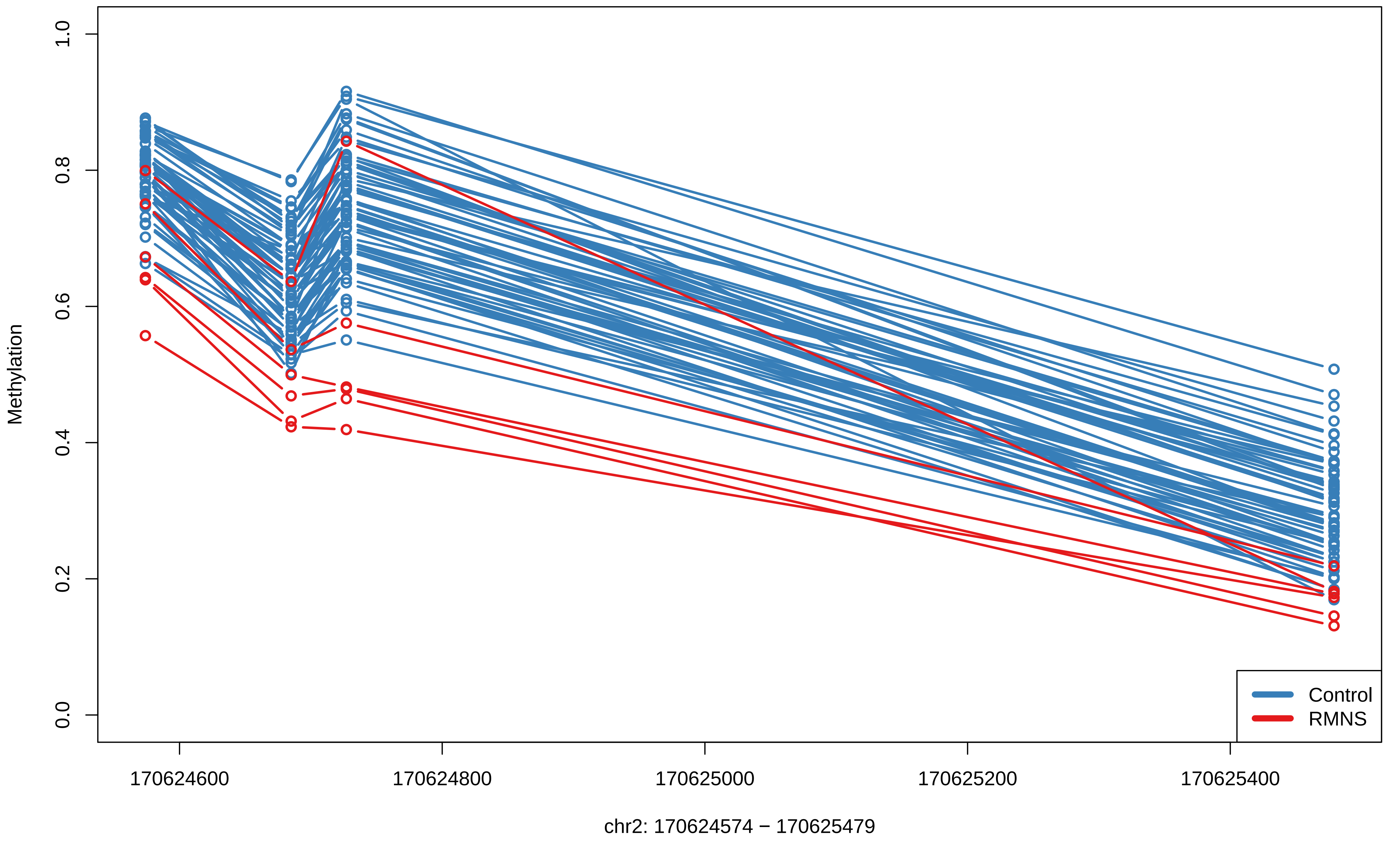

Methylation
0.0
0.2
0.4
0.6
0.8
1.0
170624600
170624800
170625000
170625200
170625400
chr2: 170624574 − 170625479
Control
RMNS

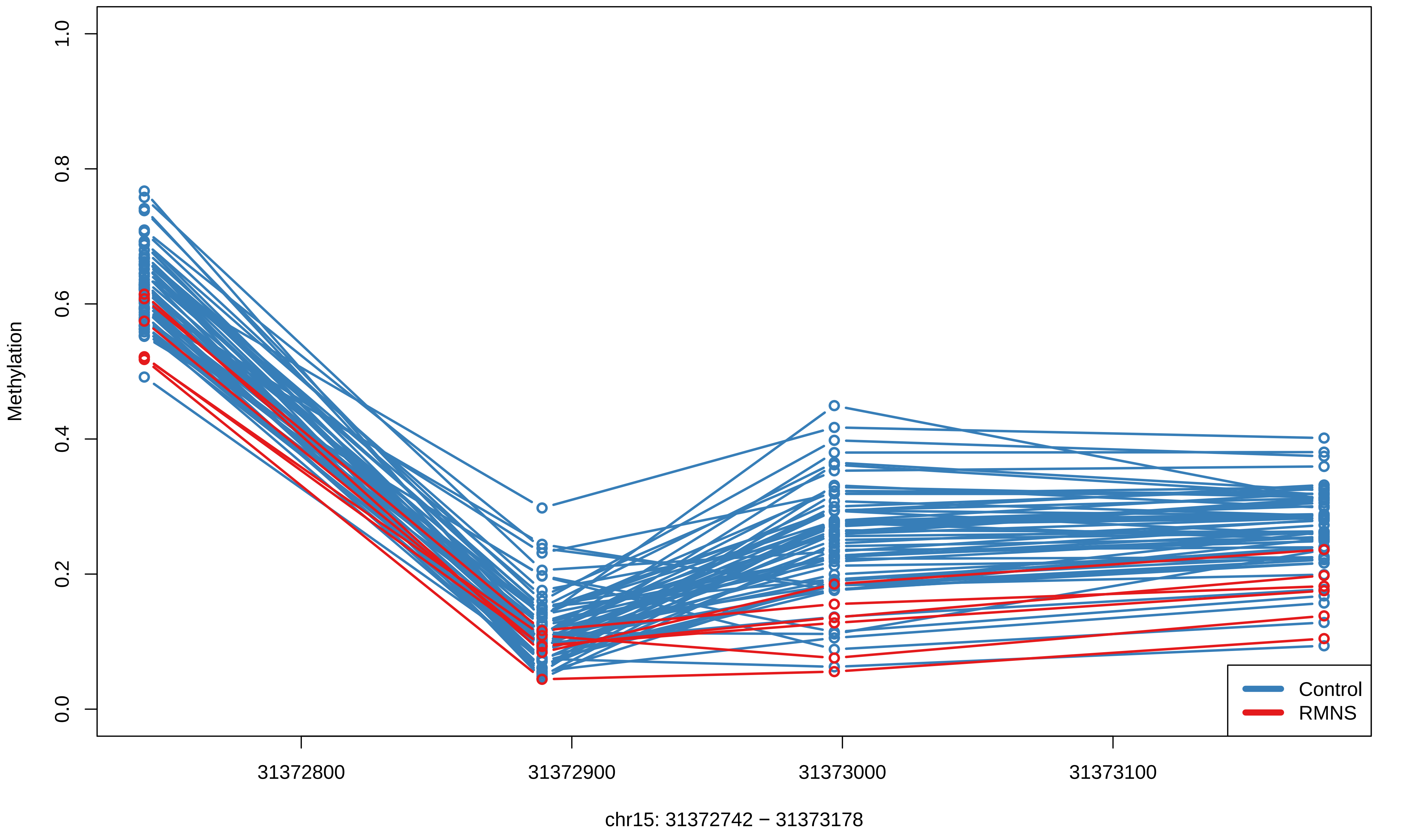

Methylation
0.0
0.2
0.4
0.6
0.8
1.0
31372800
31372900
31373000
31373100
chr15: 31372742 − 31373178
Control
RMNS

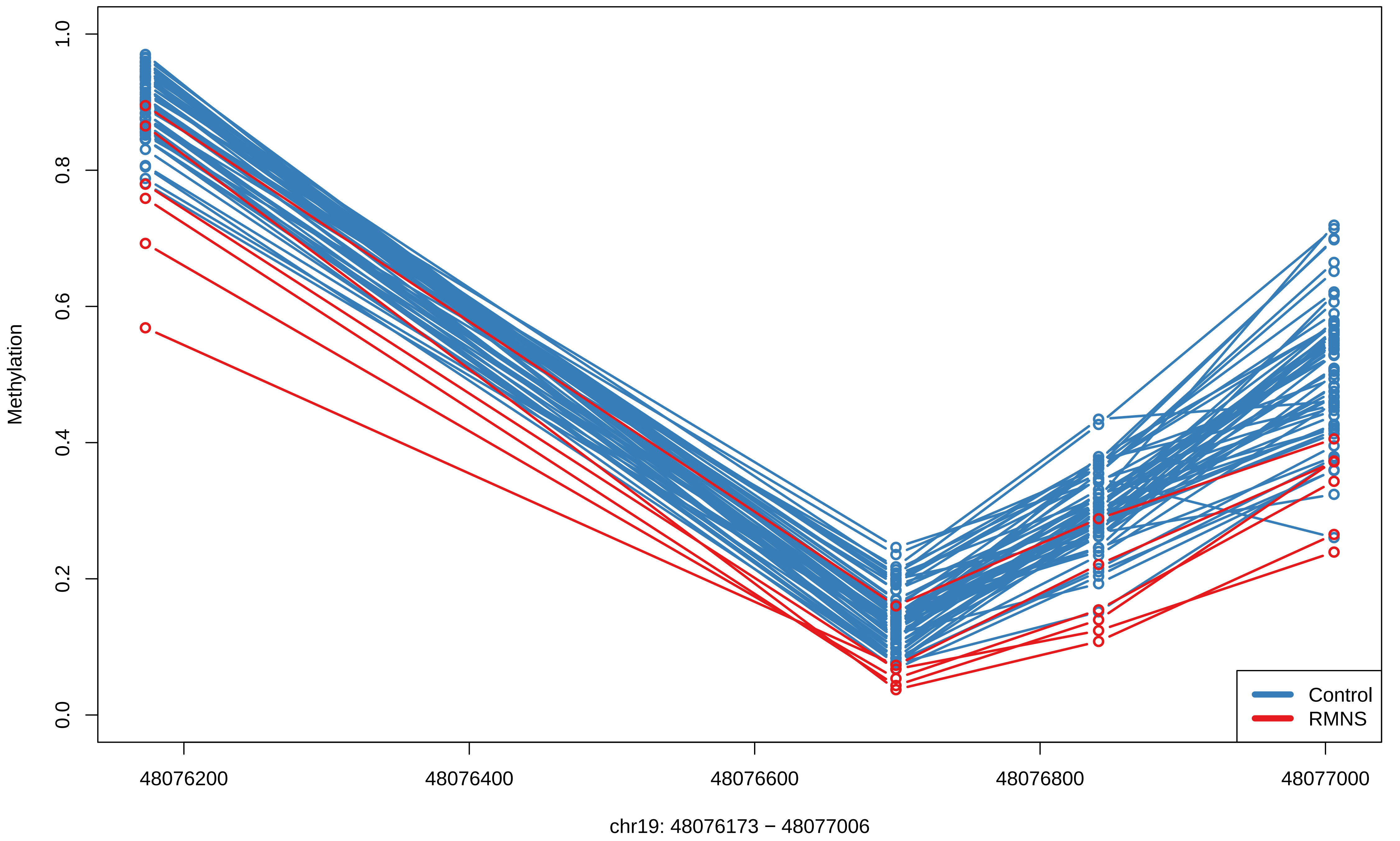

1.0
0.8
0.6
0.4
0.2
0.0
Methylation
48076200
48076400
48076600
48076800
48077000
chr19: 48076173 − 48077006
Control
RMNS

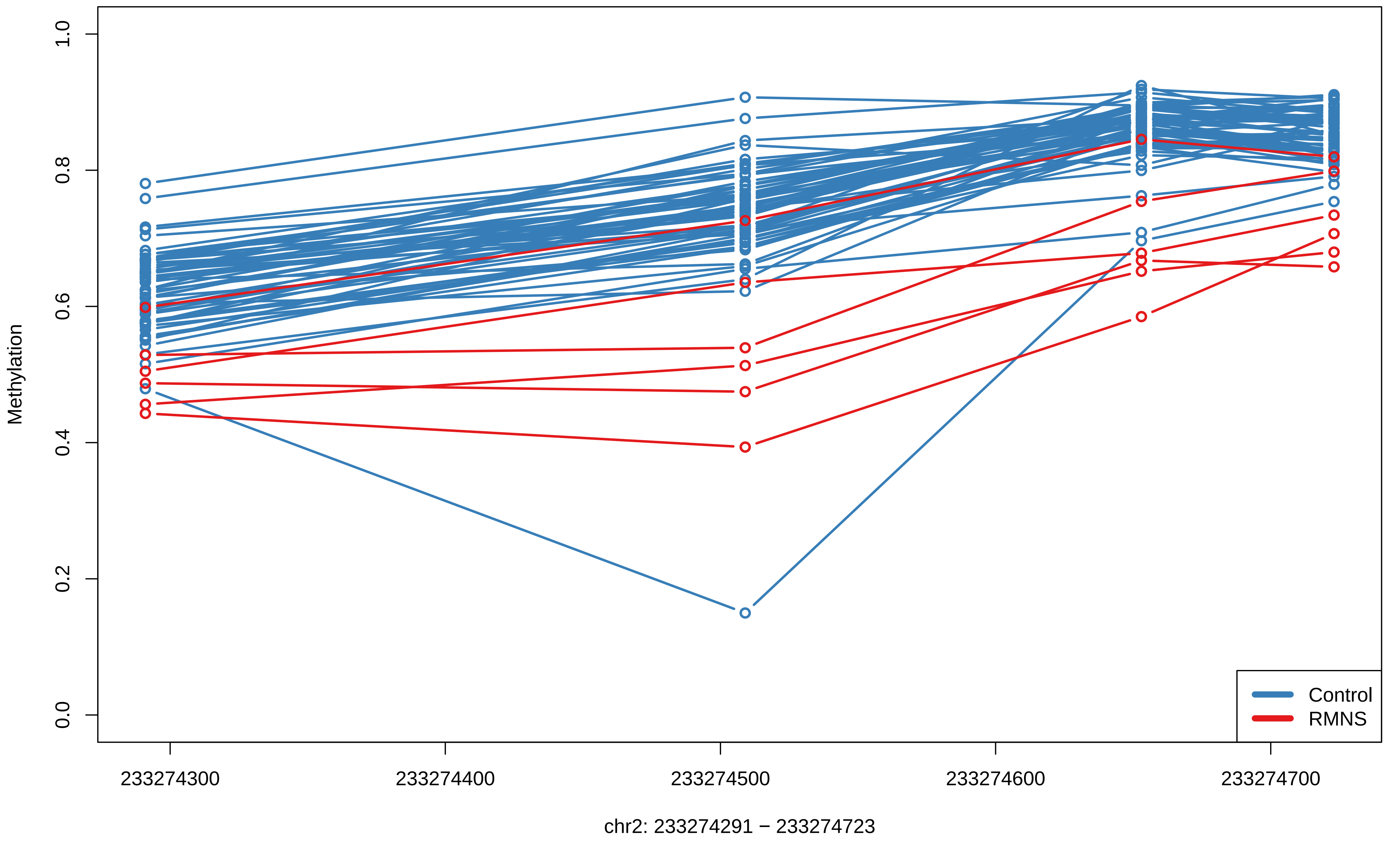

Methylation
1.0
0.8
0.6
0.4
0.2
0.0
233274300
233274400
233274500
233274600
233274700
chr2: 233274291 − 233274723
Control
RMNS

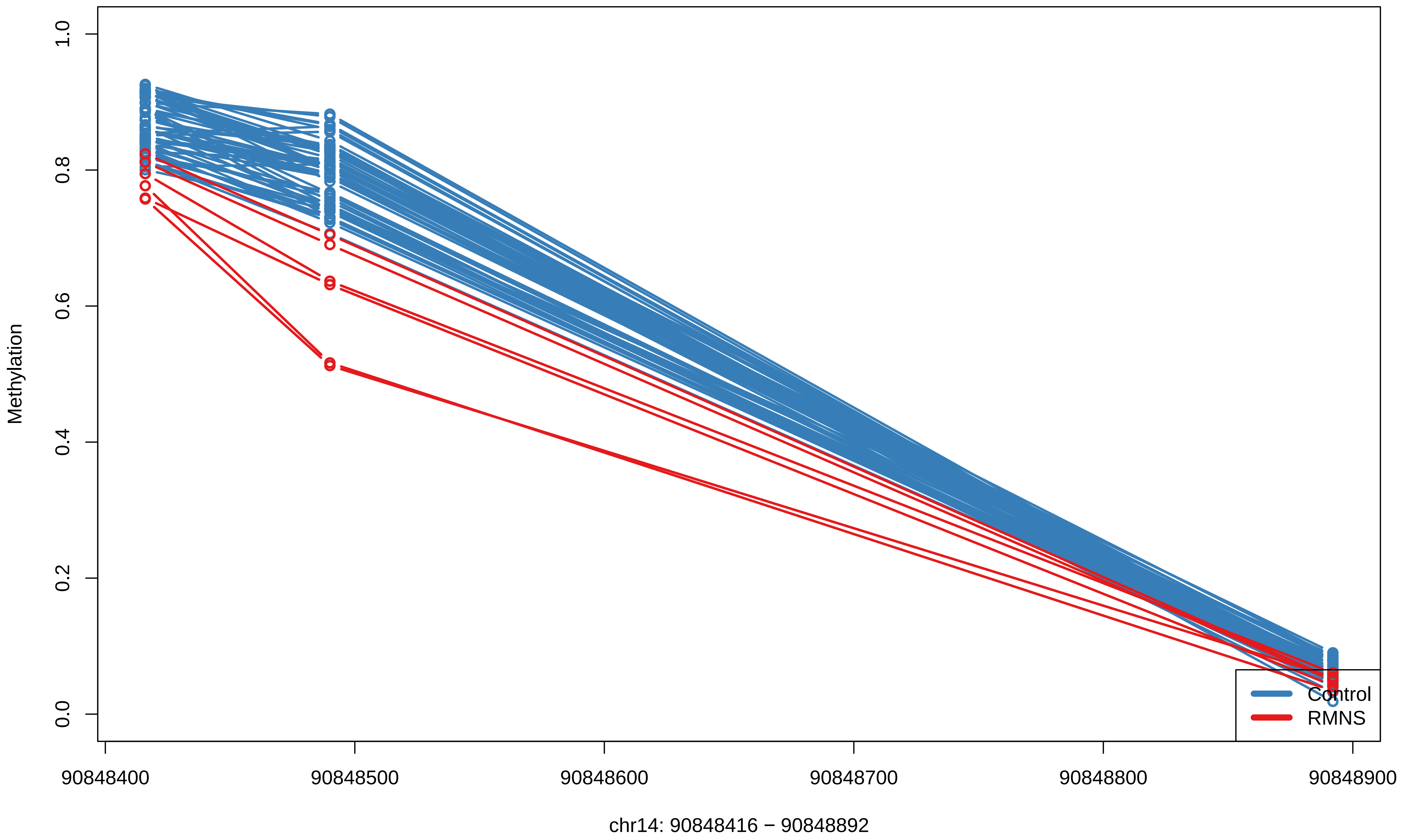

Methylation
1.0
0.8
0.6
0.4
0.2
0.0
90848400
90848500
90848600
90848700
90848800
90848900
chr14: 90848416 − 90848892
Control
RMNS

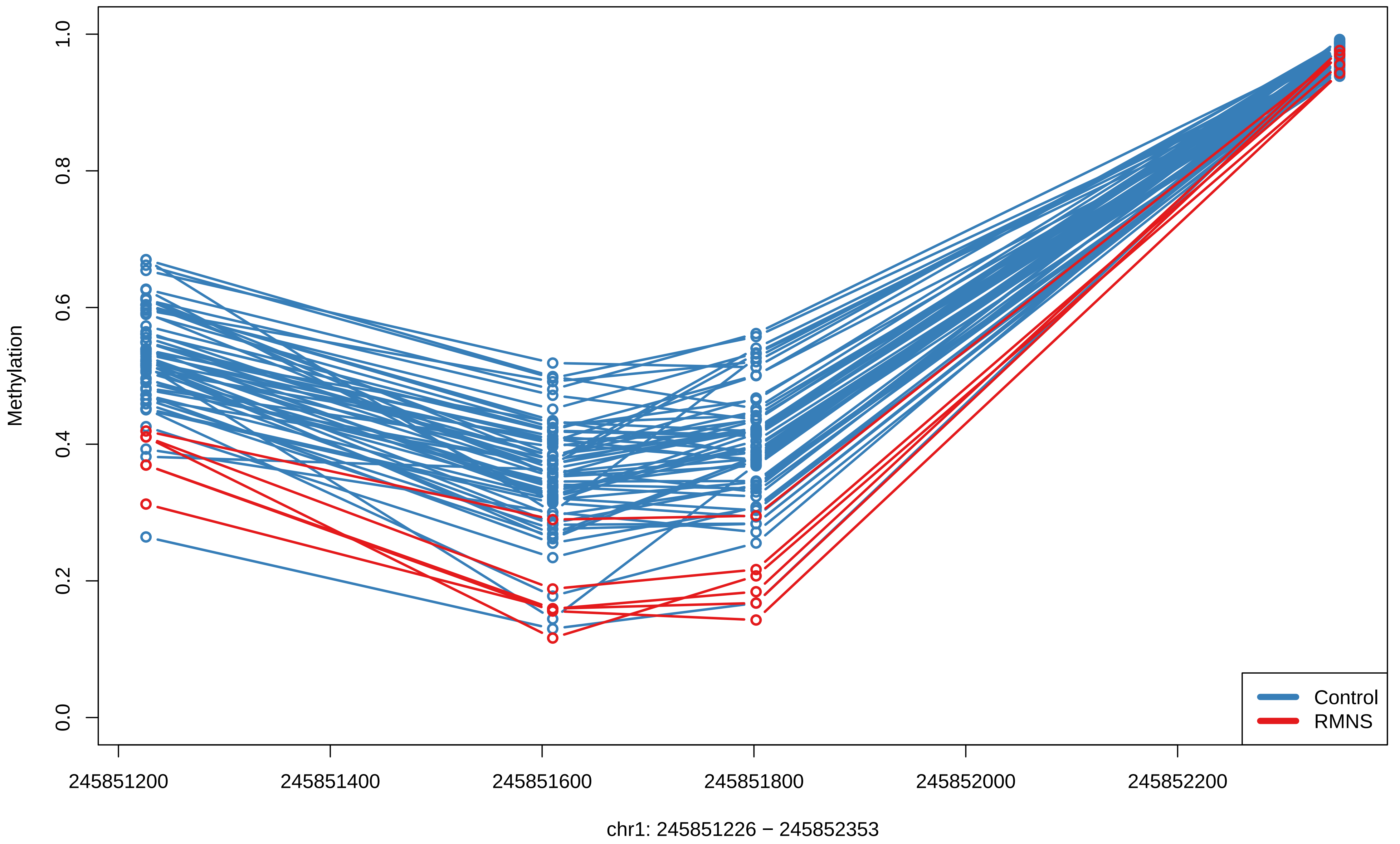

Methylation
1.0
0.8
0.6
0.4
0.2
0.0
245851200
245851400
245851600
245851800
245852000
245852200
chr1: 245851226 − 245852353
Control
RMNS

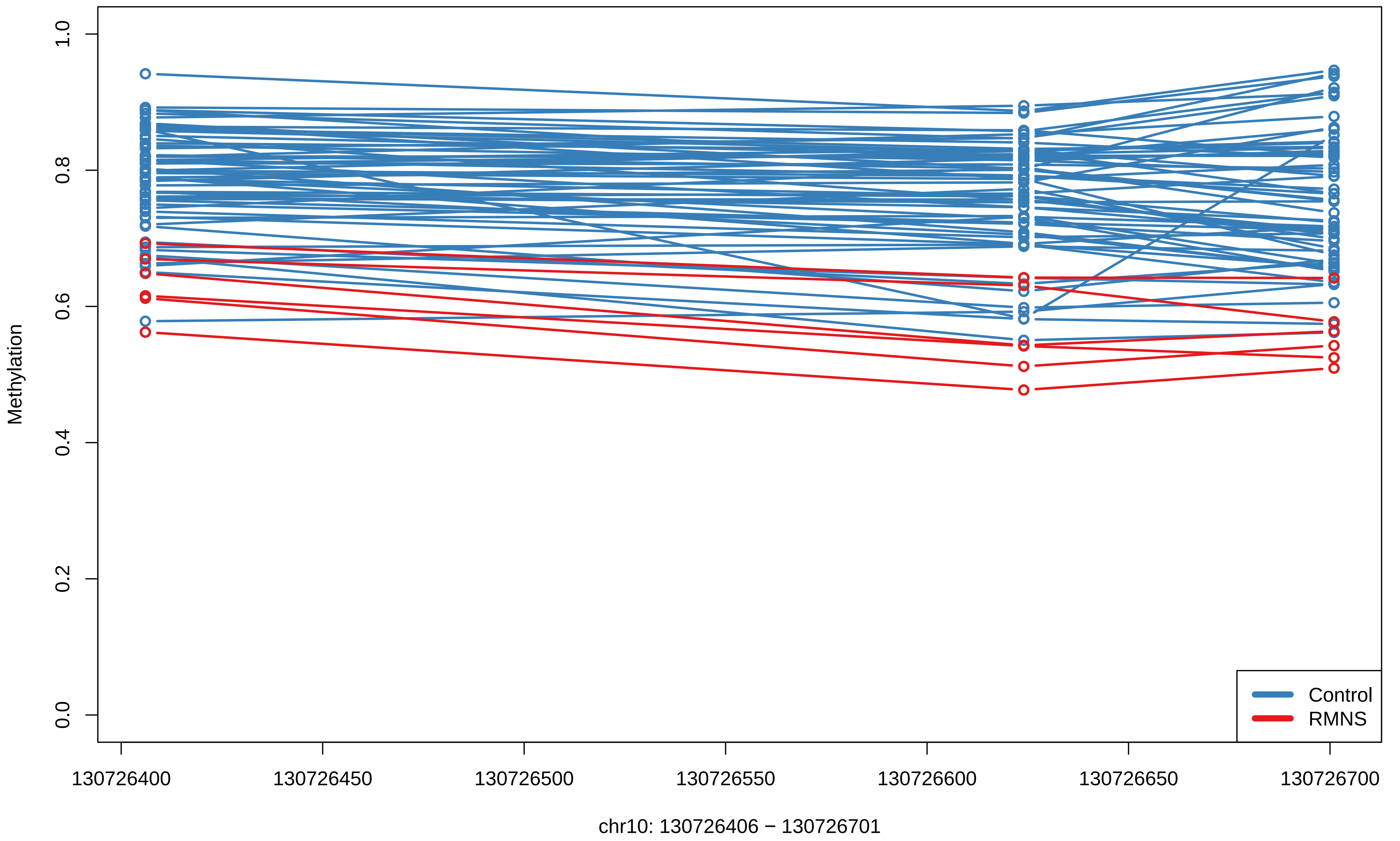

Methylation
1.0
0.8
0.6
0.4
0.2
0.0
130726400
130726450
130726500
130726550
130726600
130726650
130726700
chr10: 130726406 − 130726701
Control
RMNS

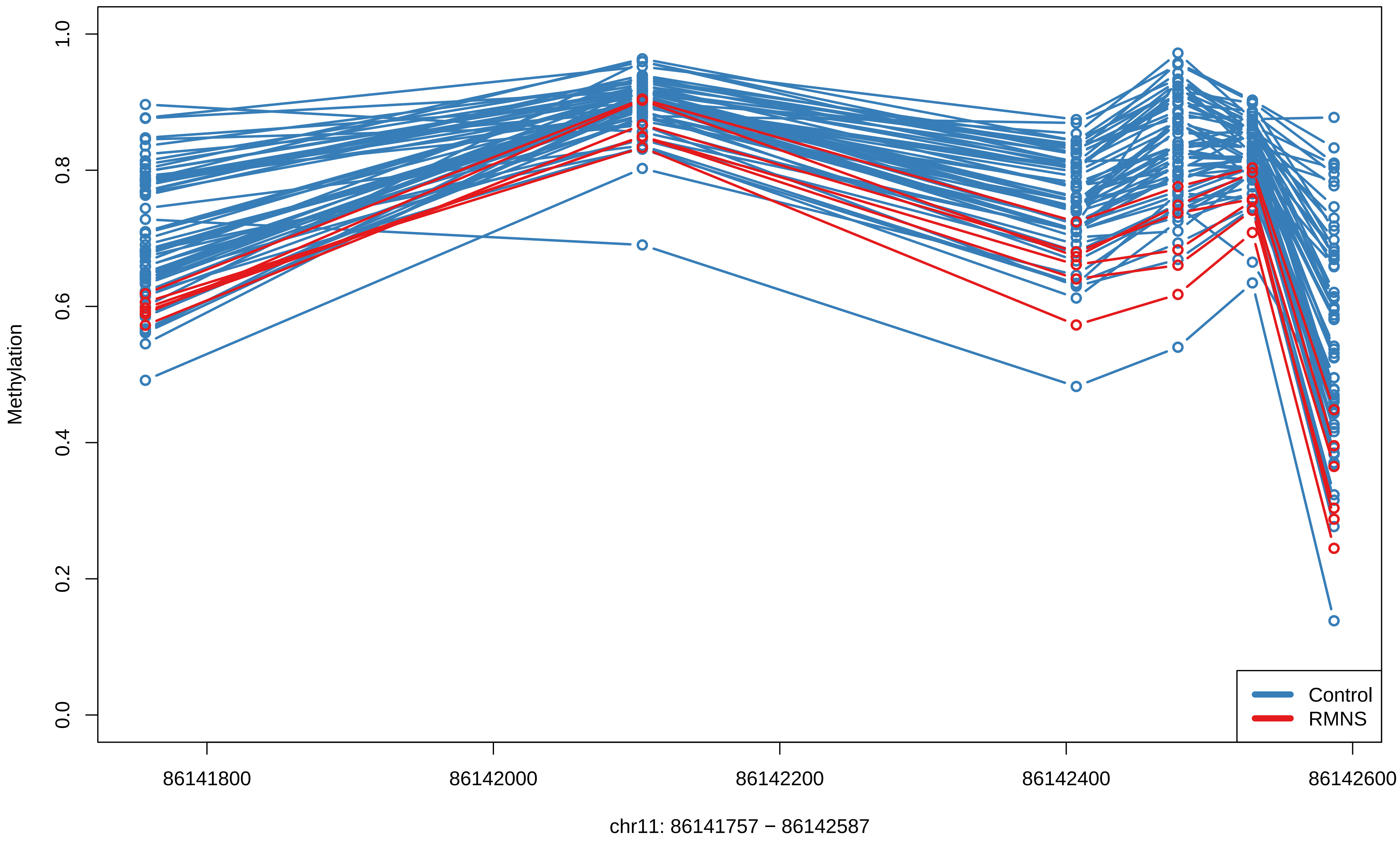

Methylation
0.0
0.2
0.4
0.6
0.8
1.0
86141800
86142000
86142200
86142400
86142600
chr11: 86141757 − 86142587
Control
RMNS

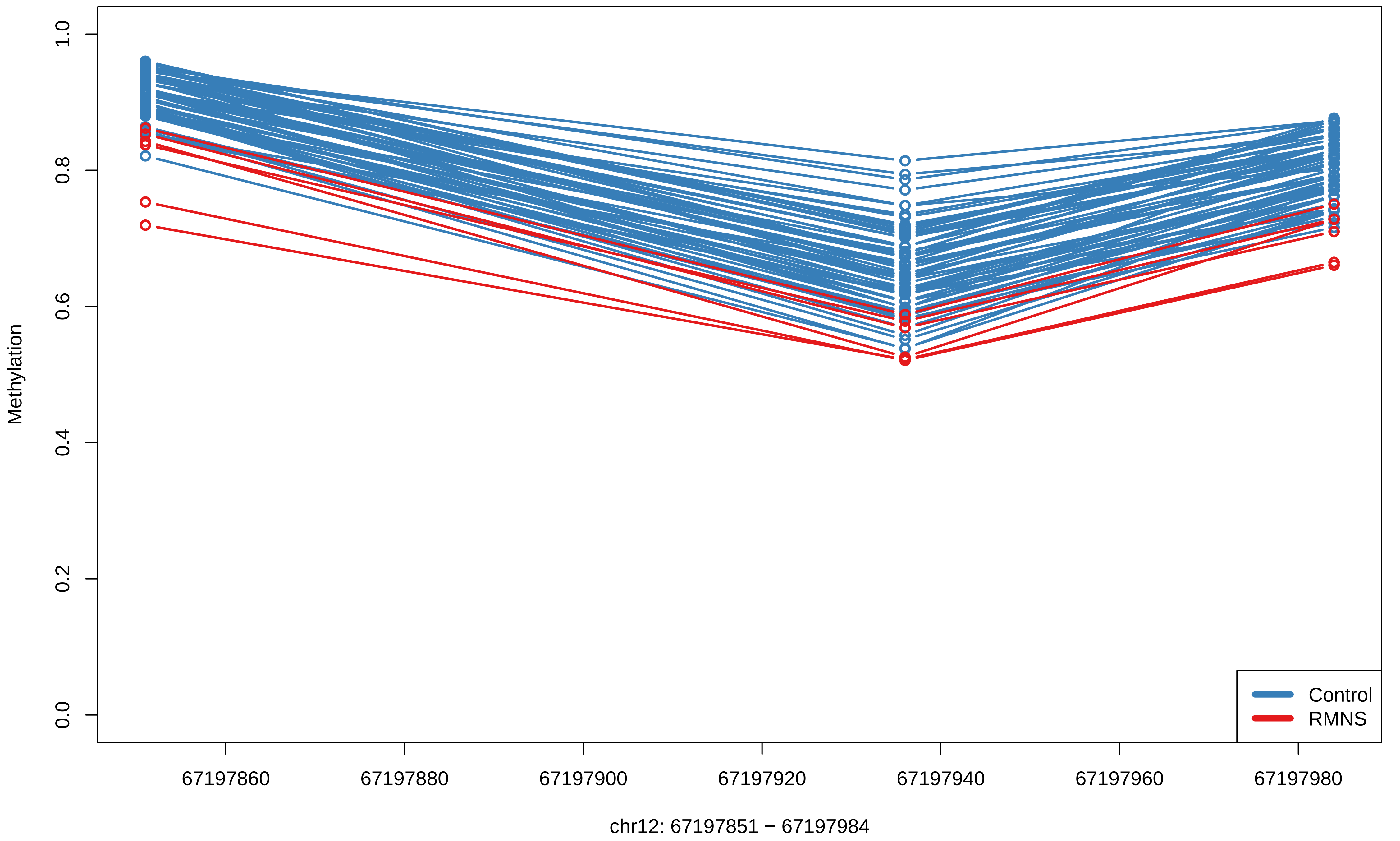

1.0
0.8
0.6
0.4
0.2
0.0
Methylation
67197860
67197880
67197900
67197920
67197940
67197960
67197980
chr12: 67197851 − 67197984
Control
RMNS

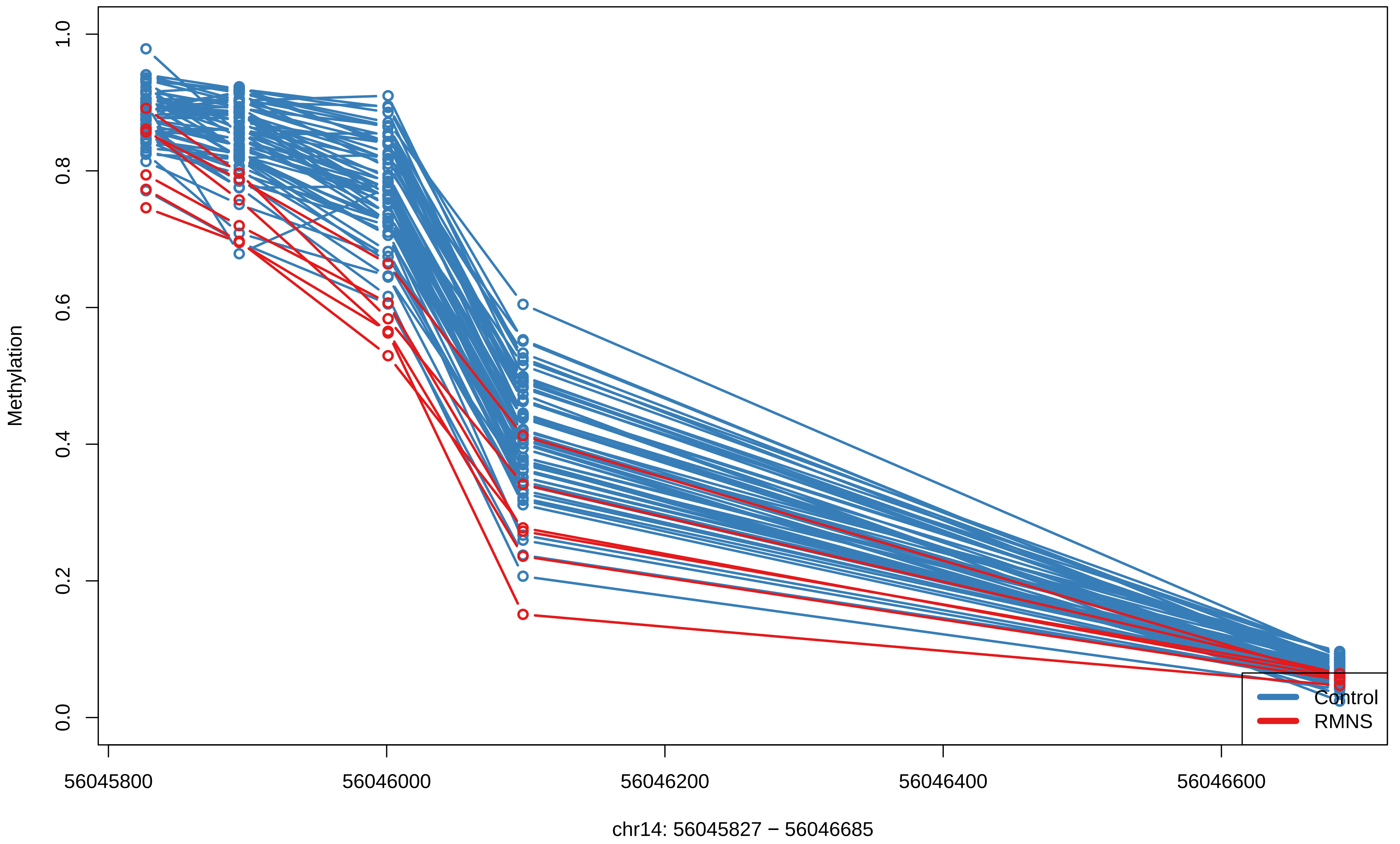

1.0
0.8
0.6
0.4
0.2
0.0
Methylation
56045800
56046000
56046200
56046400
56046600
chr14: 56045827 − 56046685
Control
RMNS

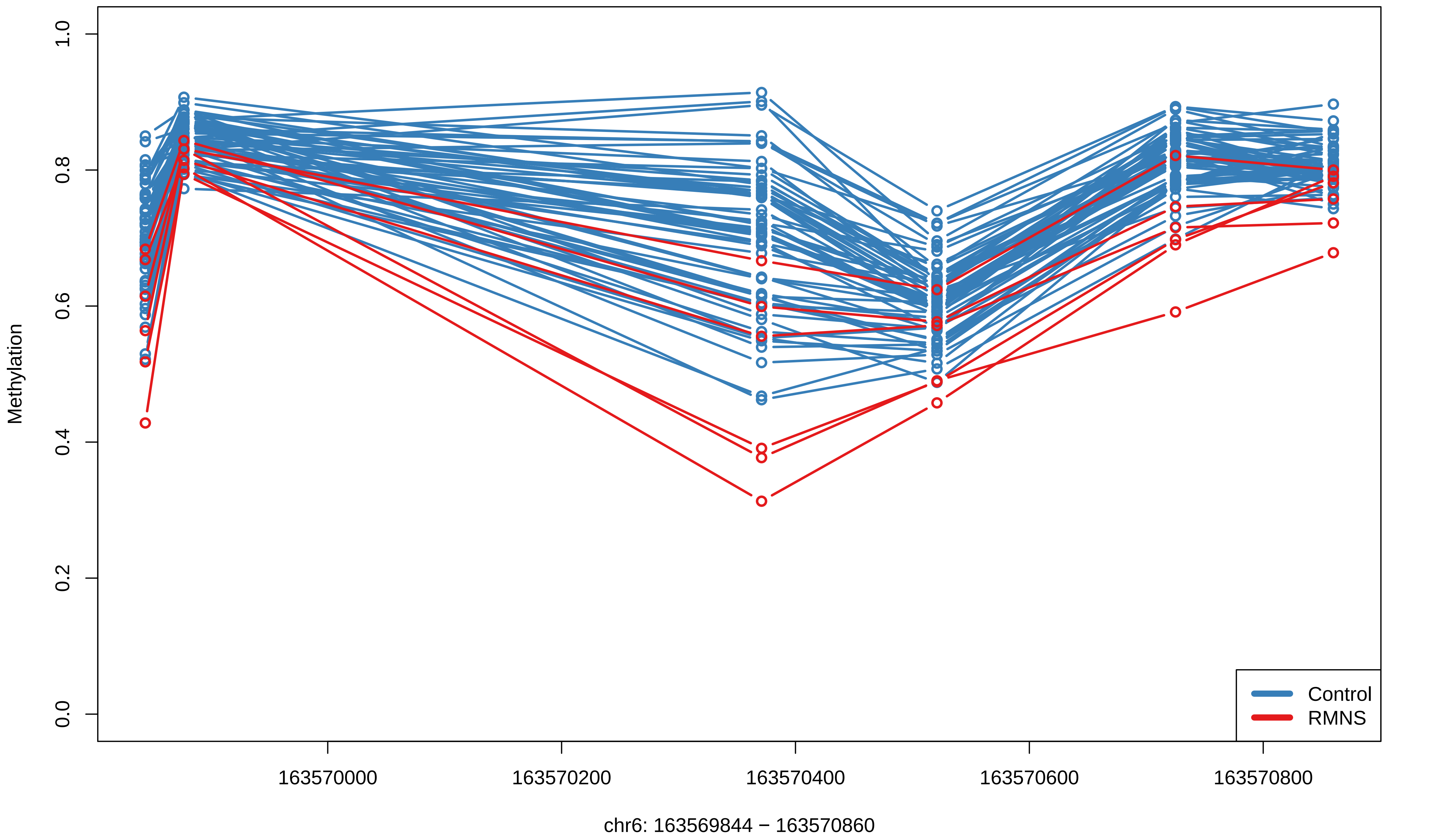

Methylation
1.0
0.8
0.6
0.4
0.2
0.0
163570000
163570200
163570400
163570600
163570800
chr6: 163569844 − 163570860
Control
RMNS

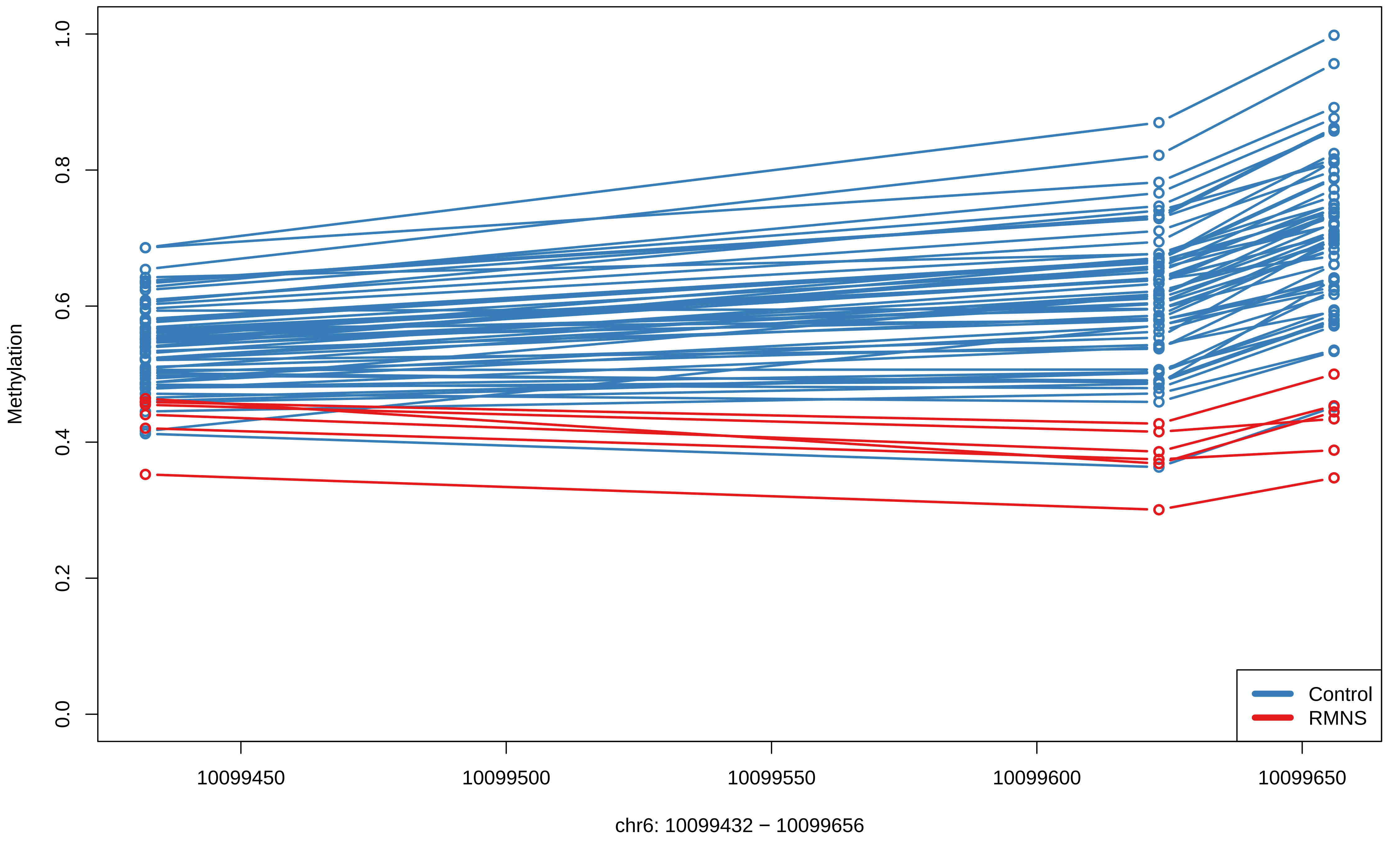

Methylation
1.0
0.8
0.6
0.4
0.2
0.0
10099450
10099500
10099550
10099600
10099650
chr6: 10099432 − 10099656
Control
RMNS

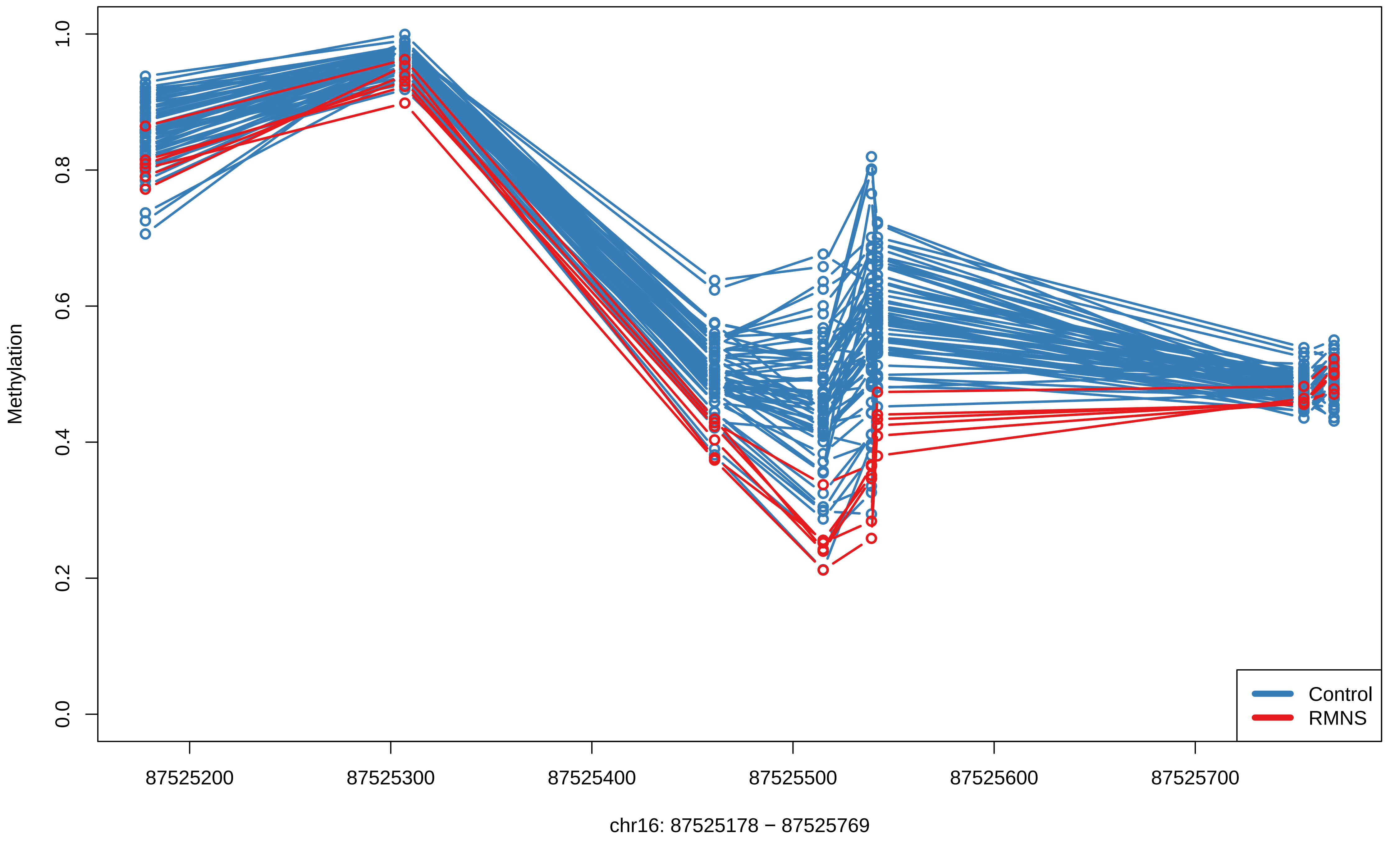

Methylation
0.0
0.2
0.4
0.6
0.8
1.0
87525200
87525300
87525400
87525500
87525600
87525700
chr16: 87525178 − 87525769
Control
RMNS

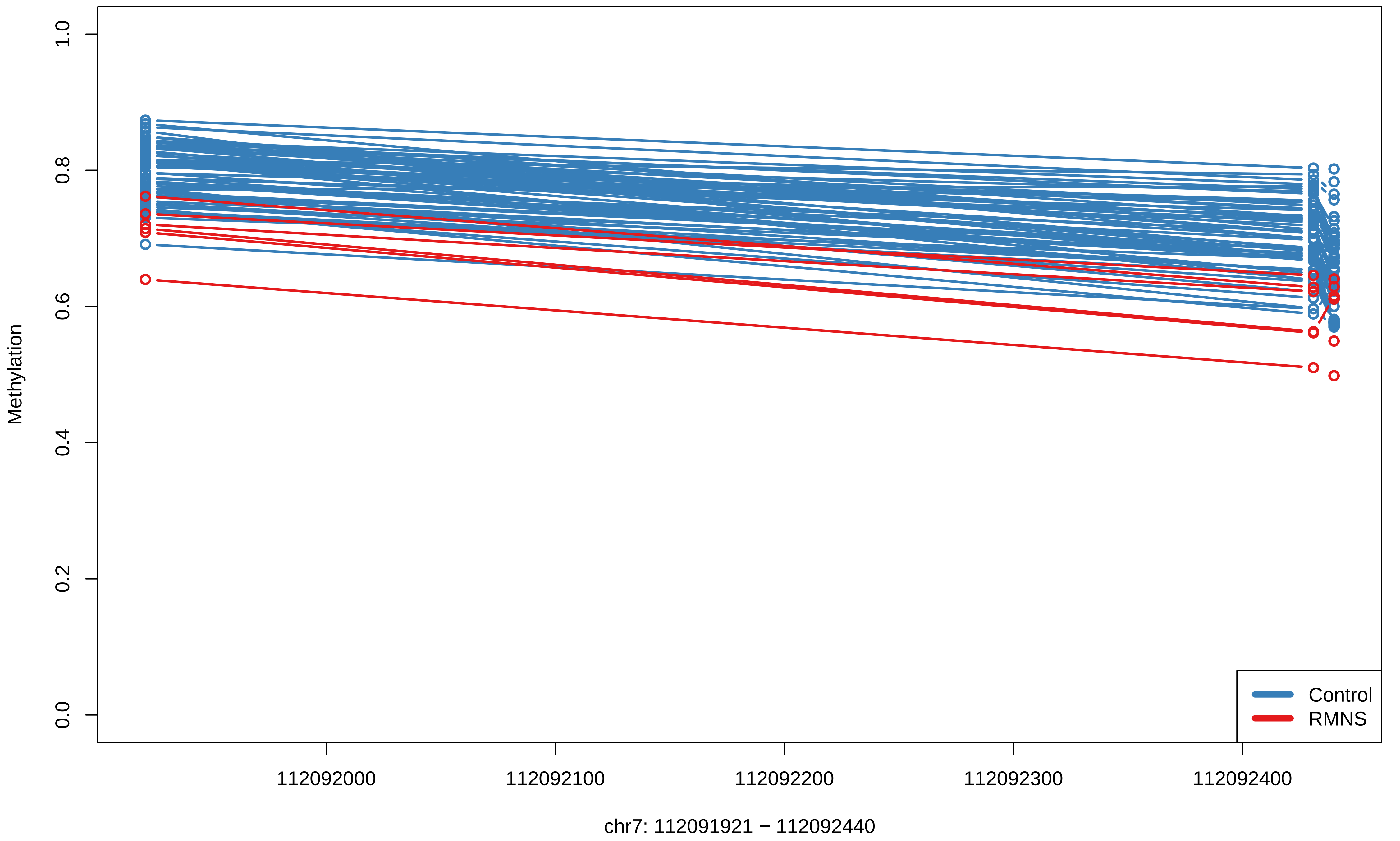

Methylation
1.0
0.8
0.6
0.4
0.2
0.0
112092000
112092100
112092200
112092300
112092400
chr7: 112091921 − 112092440
Control
RMNS

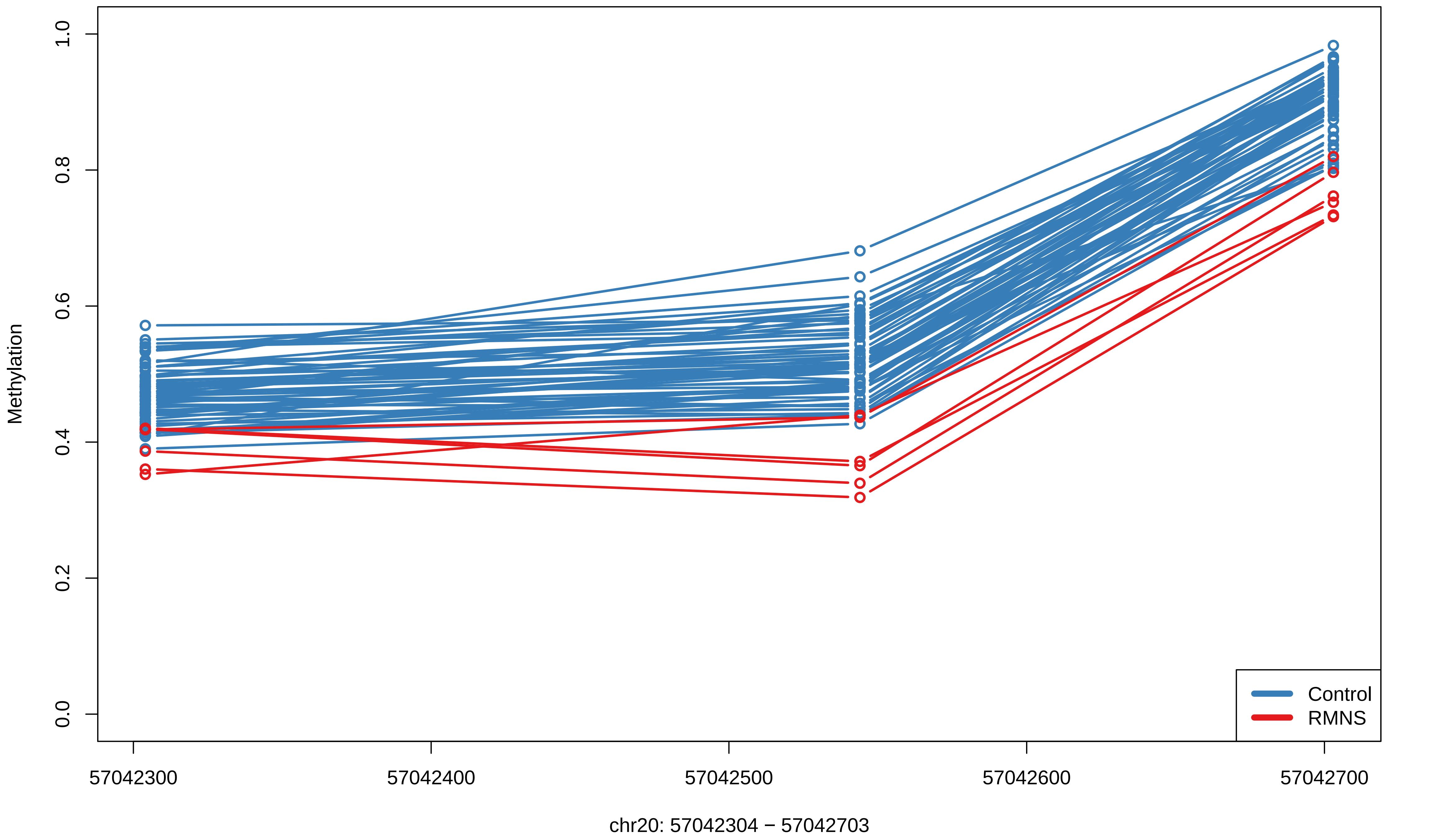

Methylation
0.0
0.2
0.4
0.6
0.8
1.0
57042300
57042400
57042500
57042600
57042700
chr20: 57042304 − 57042703
Control
RMNS

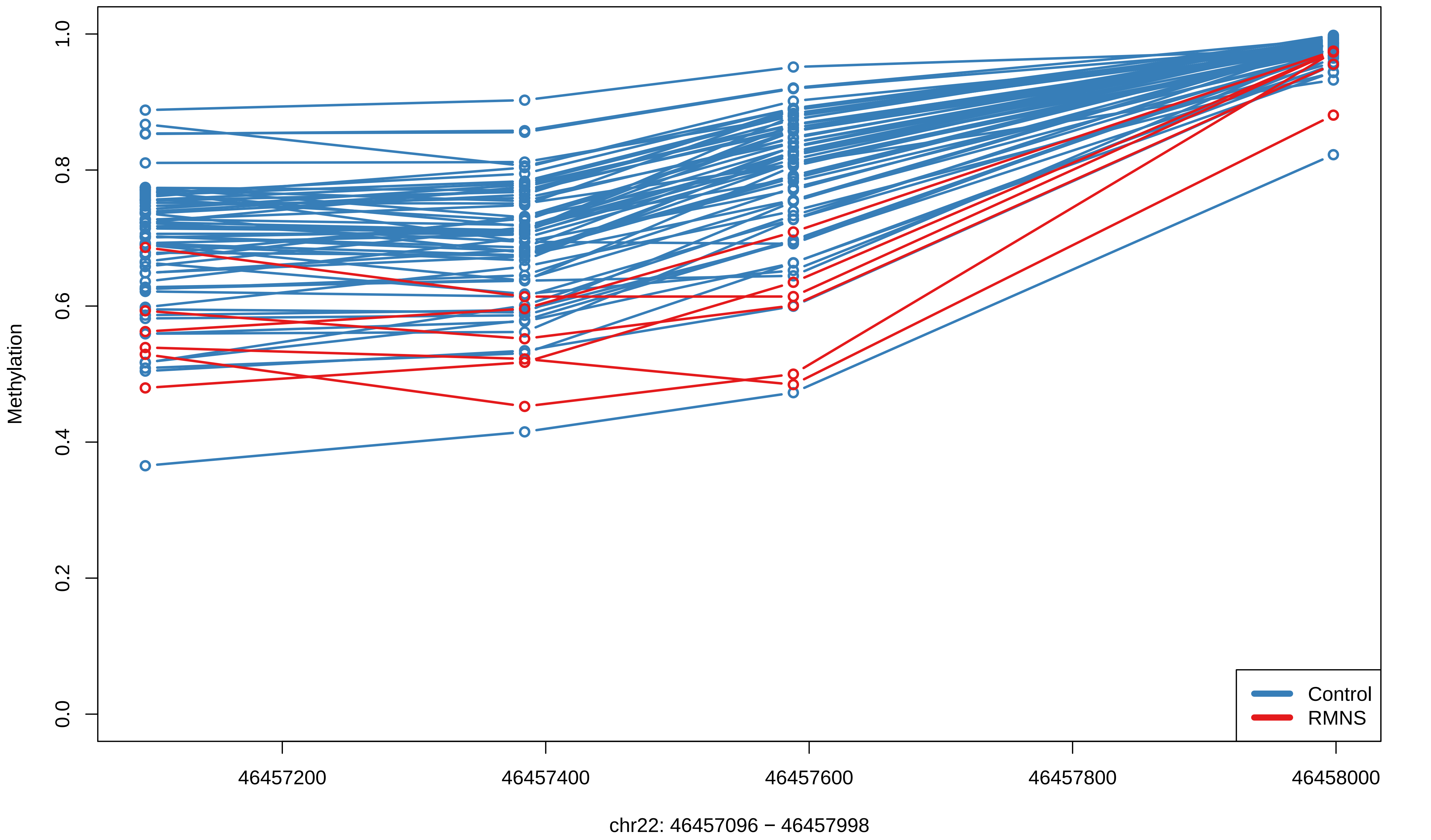

Methylation
1.0
0.8
0.6
0.4
0.2
0.0
46457200
46457400
46457600
46457800
46458000
chr22: 46457096 − 46457998
Control
RMNS

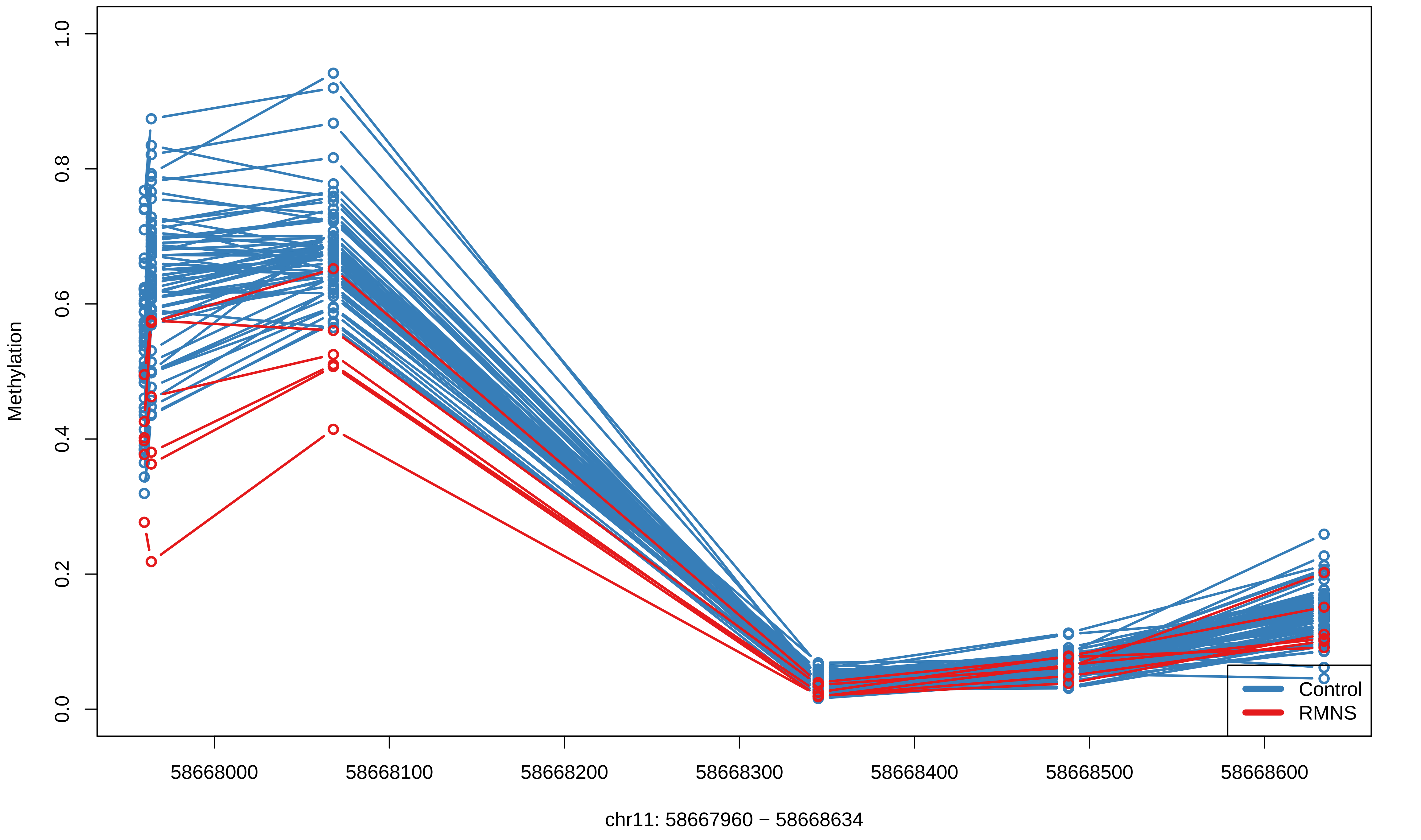

1.0
0.8
0.6
0.4
0.2
0.0
Methylation
58668000
58668100
58668200
58668300
58668400
58668500
58668600
chr11: 58667960 − 58668634
Control
RMNS

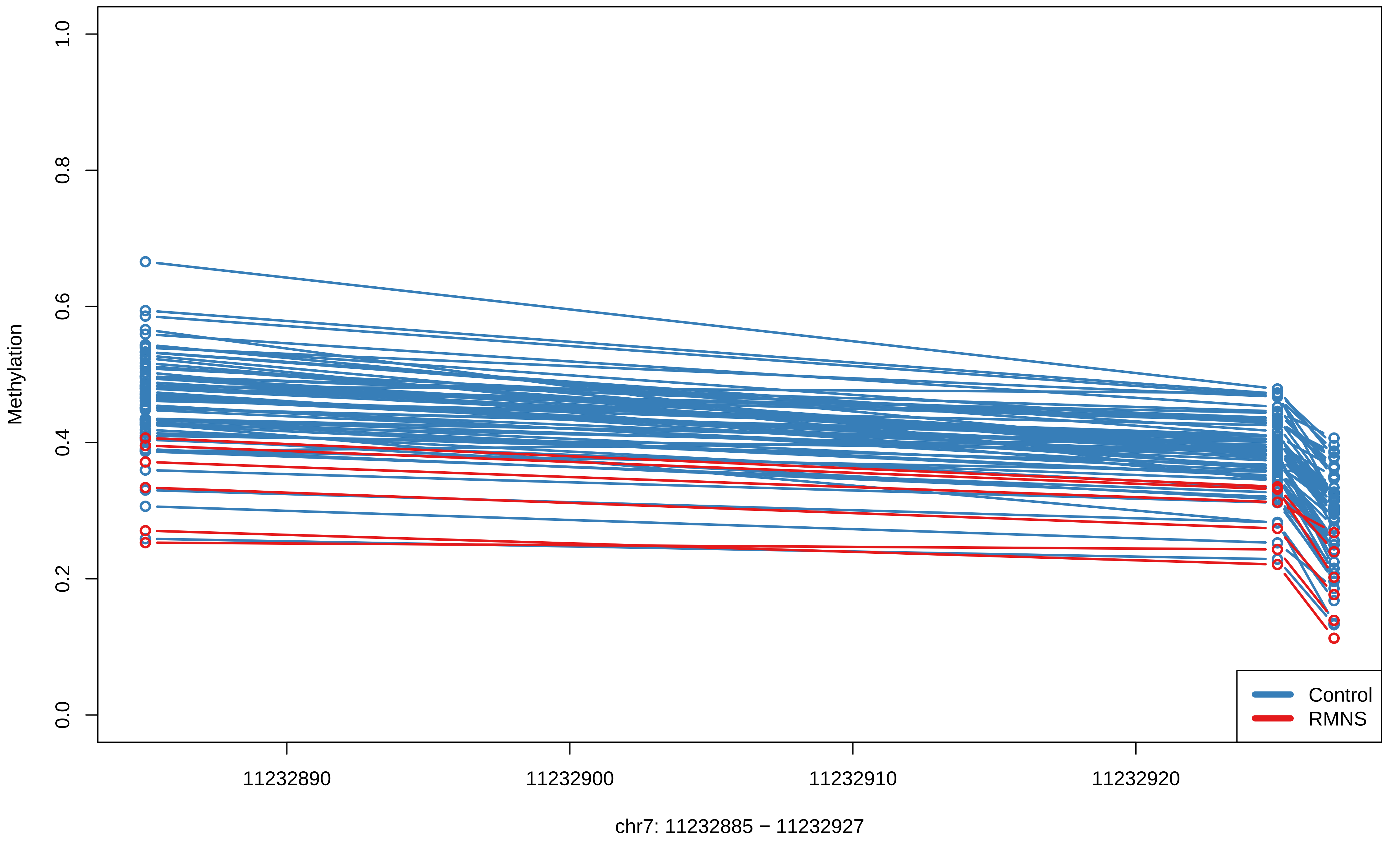

Methylation
0.0
0.2
0.4
0.6
0.8
1.0
11232890
11232900
11232910
11232920
chr7: 11232885 − 11232927
Control
RMNS

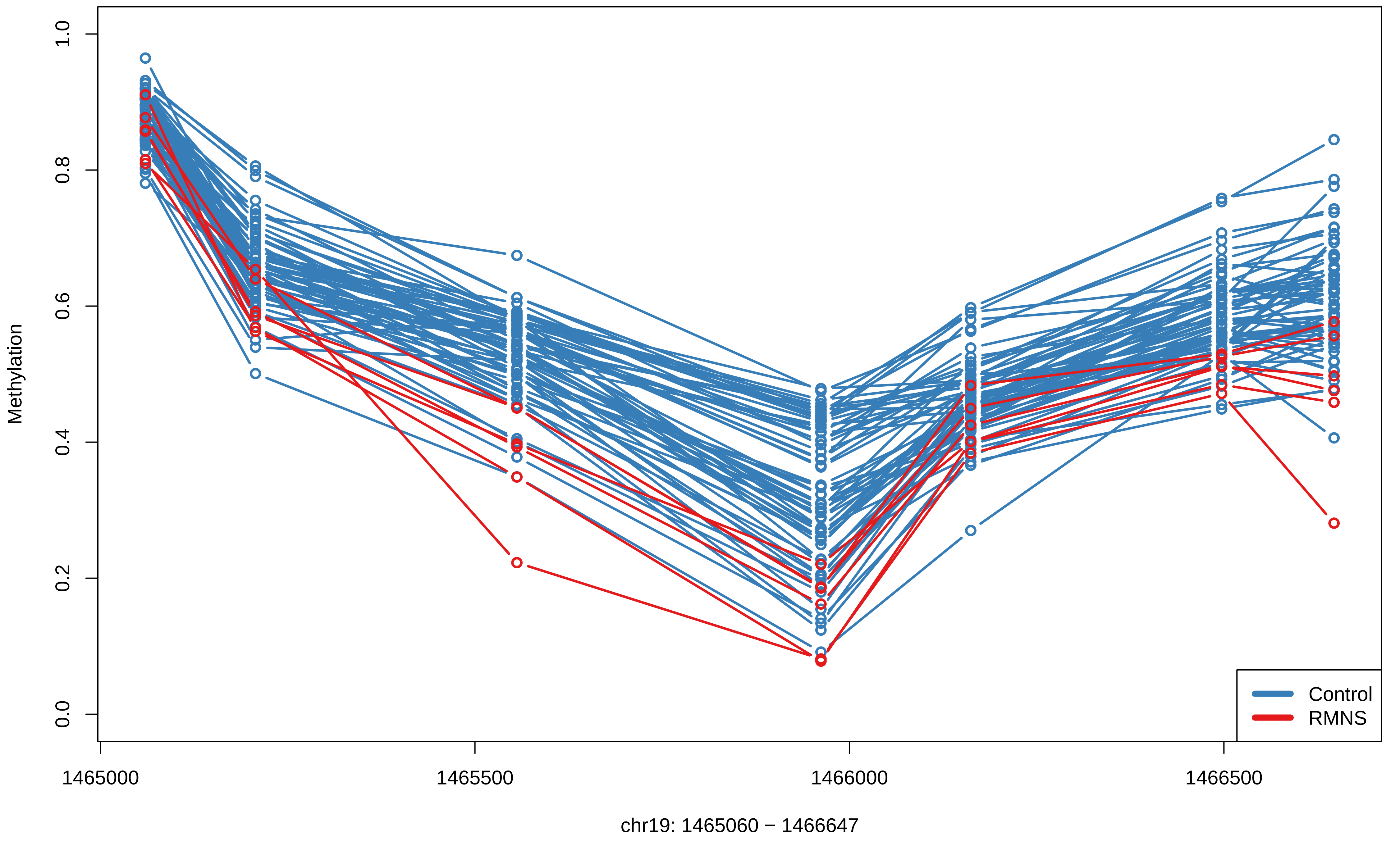

Methylation
1.0
0.8
0.6
0.4
0.2
0.0
1465000
1465500
1466000
1466500
chr19: 1465060 − 1466647
Control
RMNS

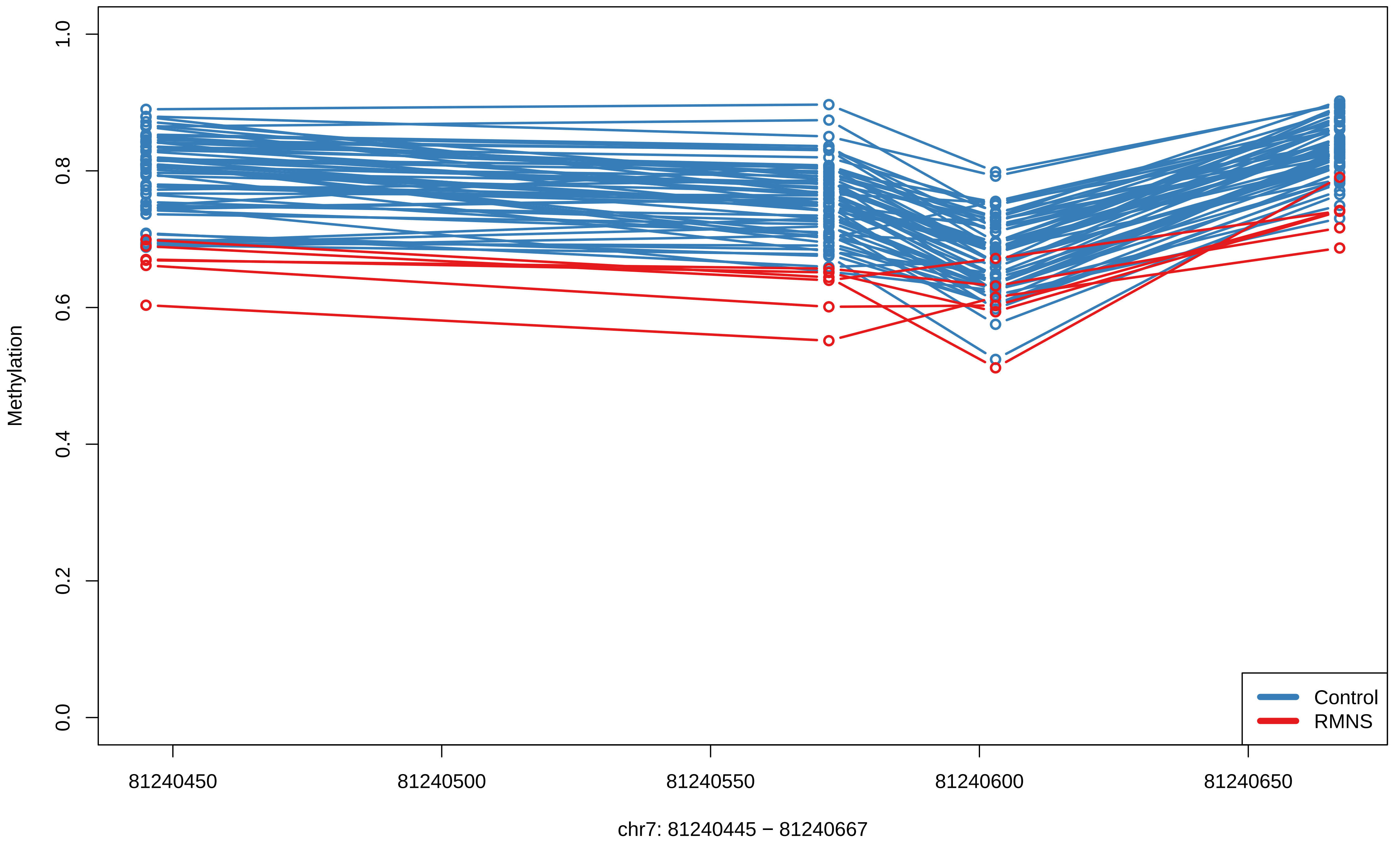

Methylation
1.0
0.8
0.6
0.4
0.2
0.0
81240450
81240500
81240550
81240600
81240650
chr7: 81240445 − 81240667
Control
RMNS

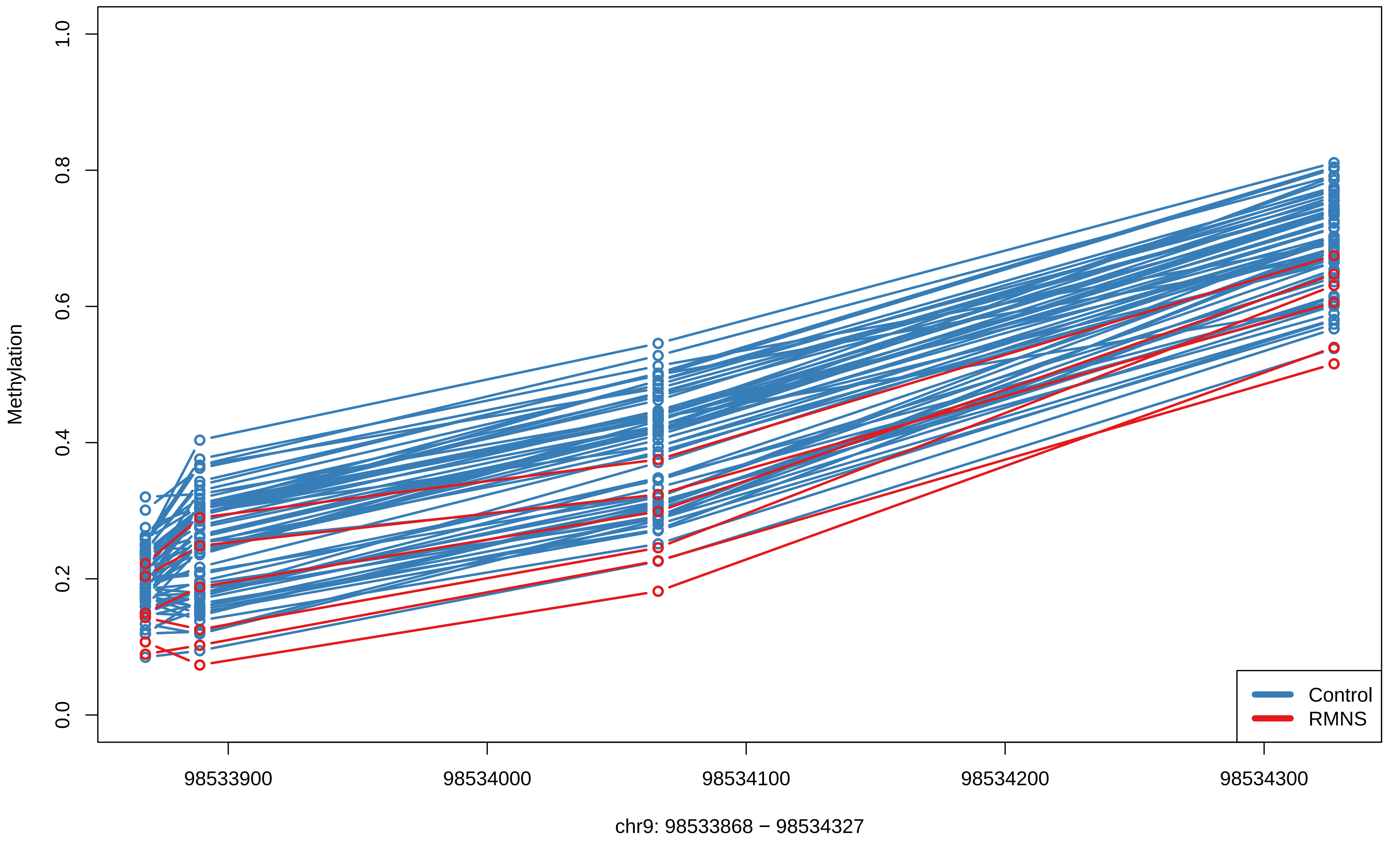

Methylation
1.0
0.8
0.6
0.4
0.2
0.0
98533900
98534000
98534100
98534200
98534300
chr9: 98533868 − 98534327
Control
RMNS

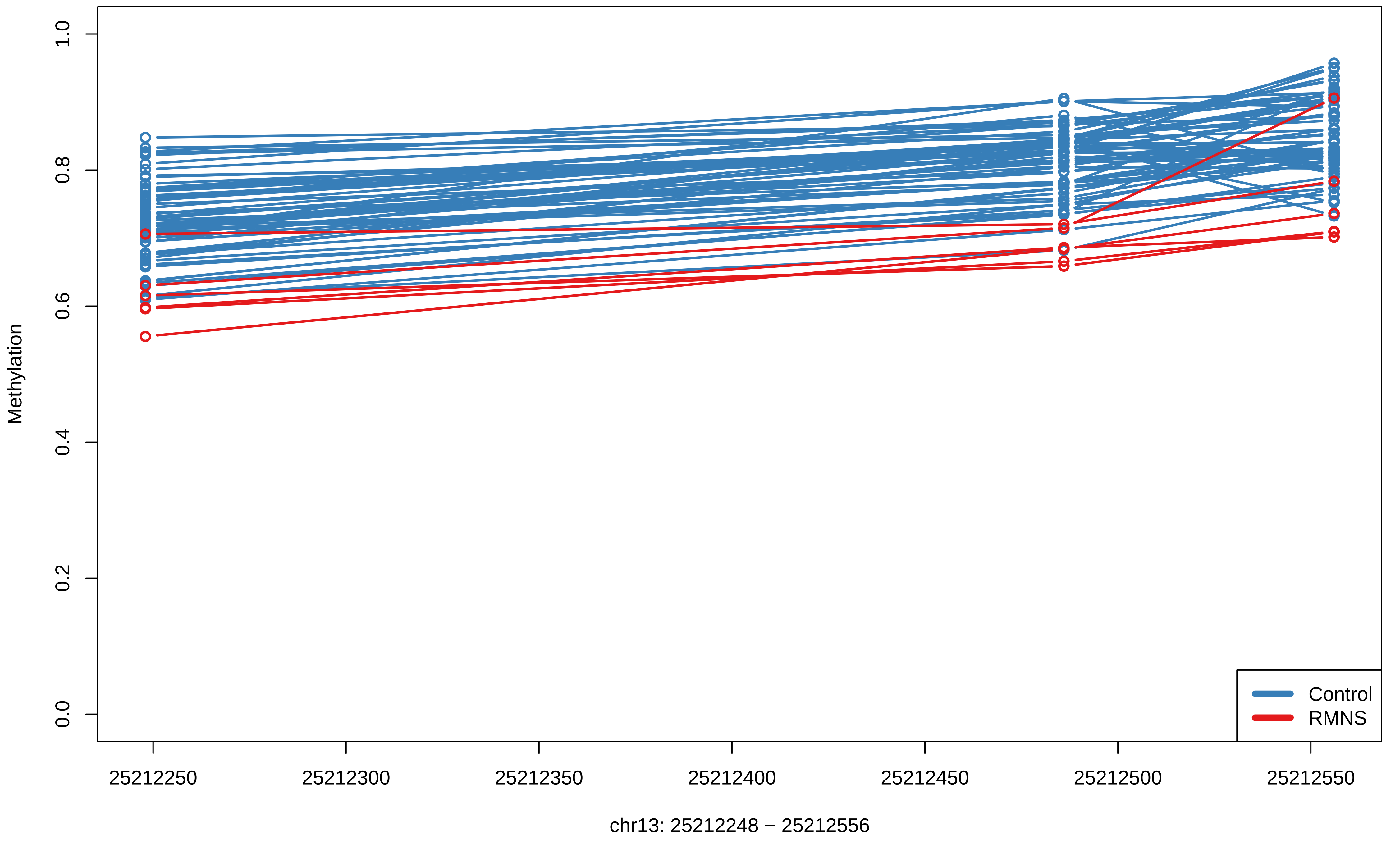

Methylation
0.0
0.2
0.4
0.6
0.8
1.0
25212250
25212300
25212350
25212400
25212450
25212500
25212550
chr13: 25212248 − 25212556
Control
RMNS

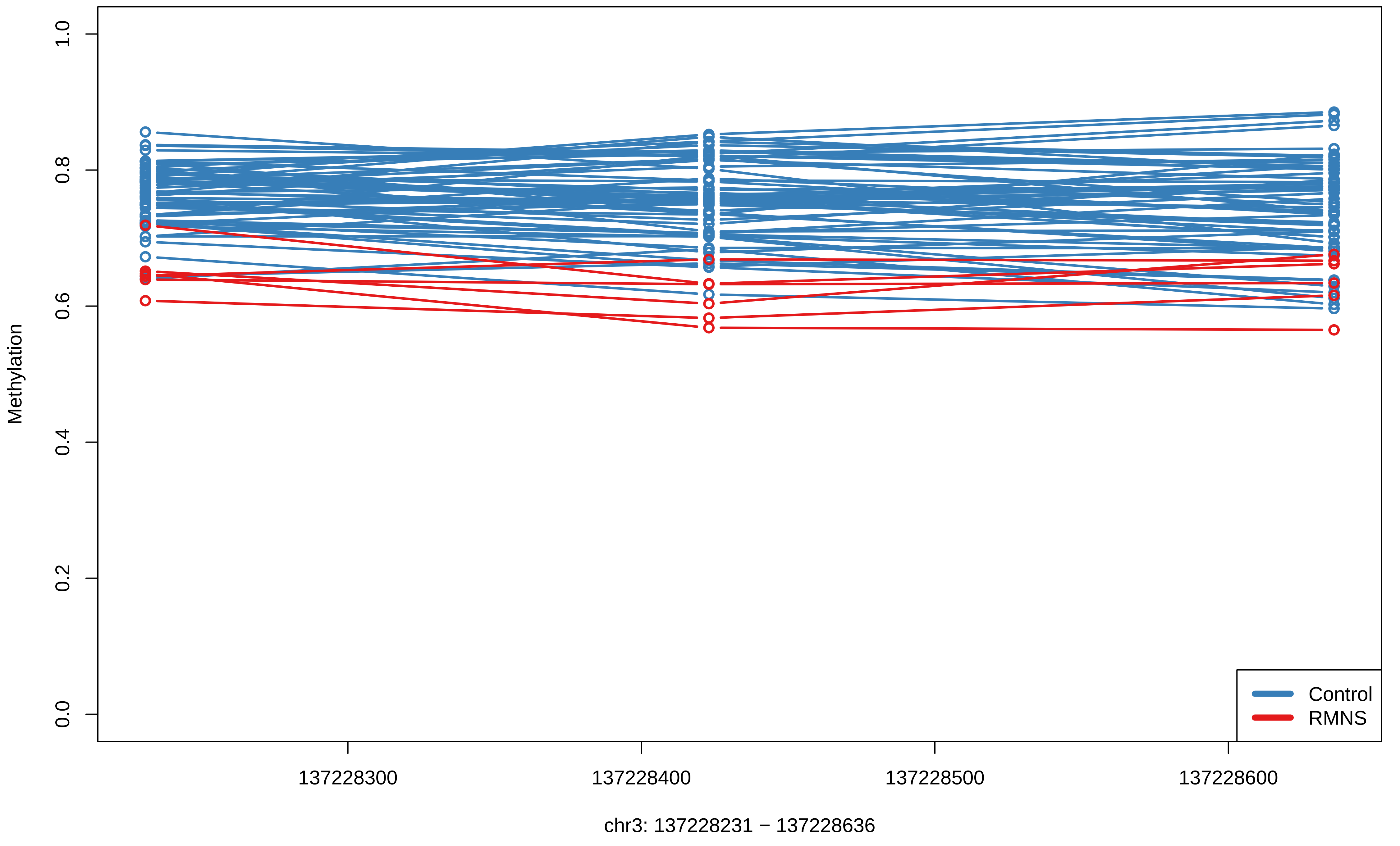

Methylation
1.0
0.8
0.6
0.4
0.2
0.0
137228300
137228400
137228500
137228600
chr3: 137228231 − 137228636
Control
RMNS

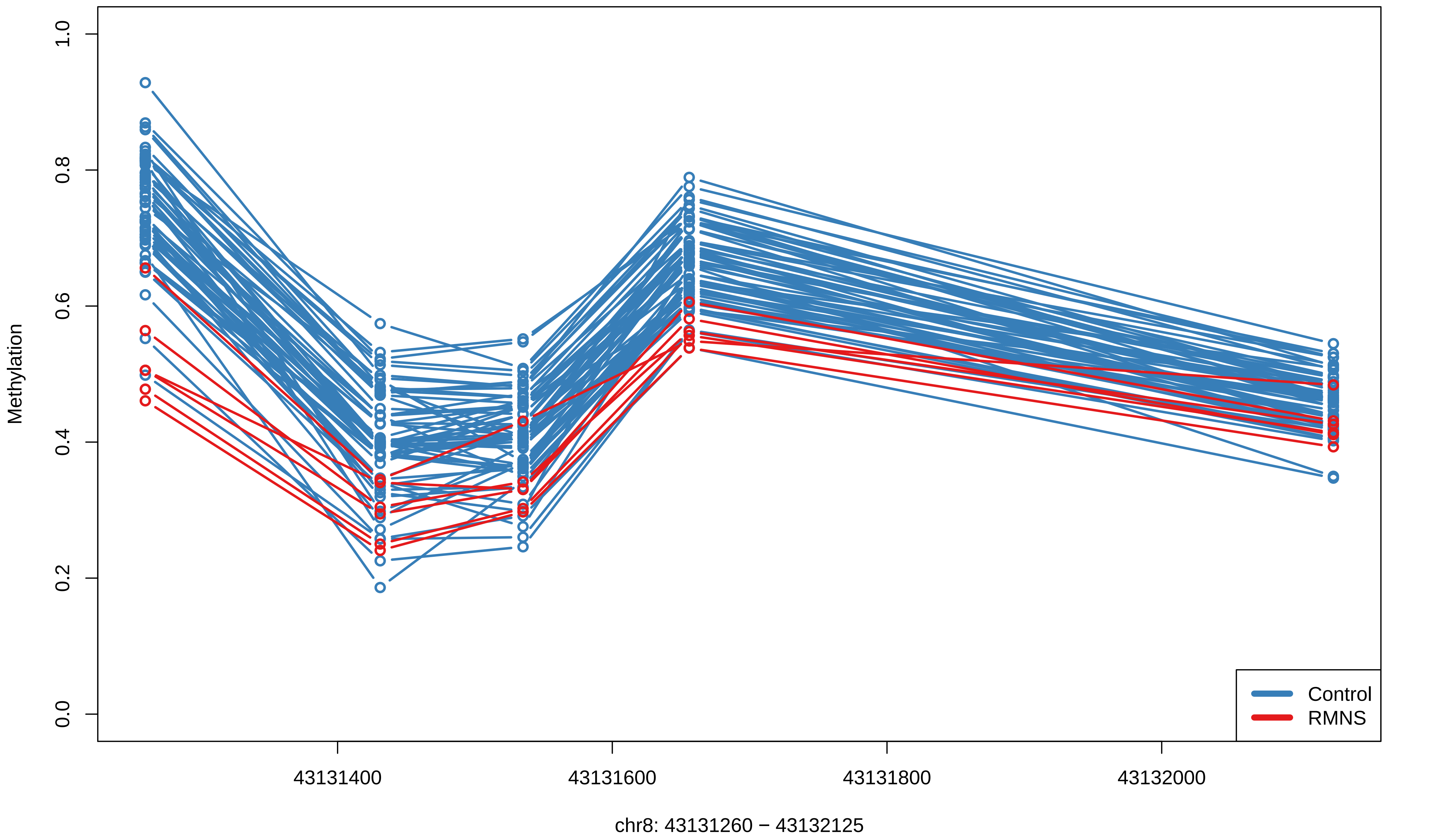

Methylation
0.0
0.2
0.4
0.6
0.8
1.0
43131400
43131600
43131800
43132000
chr8: 43131260 − 43132125
Control
RMNS

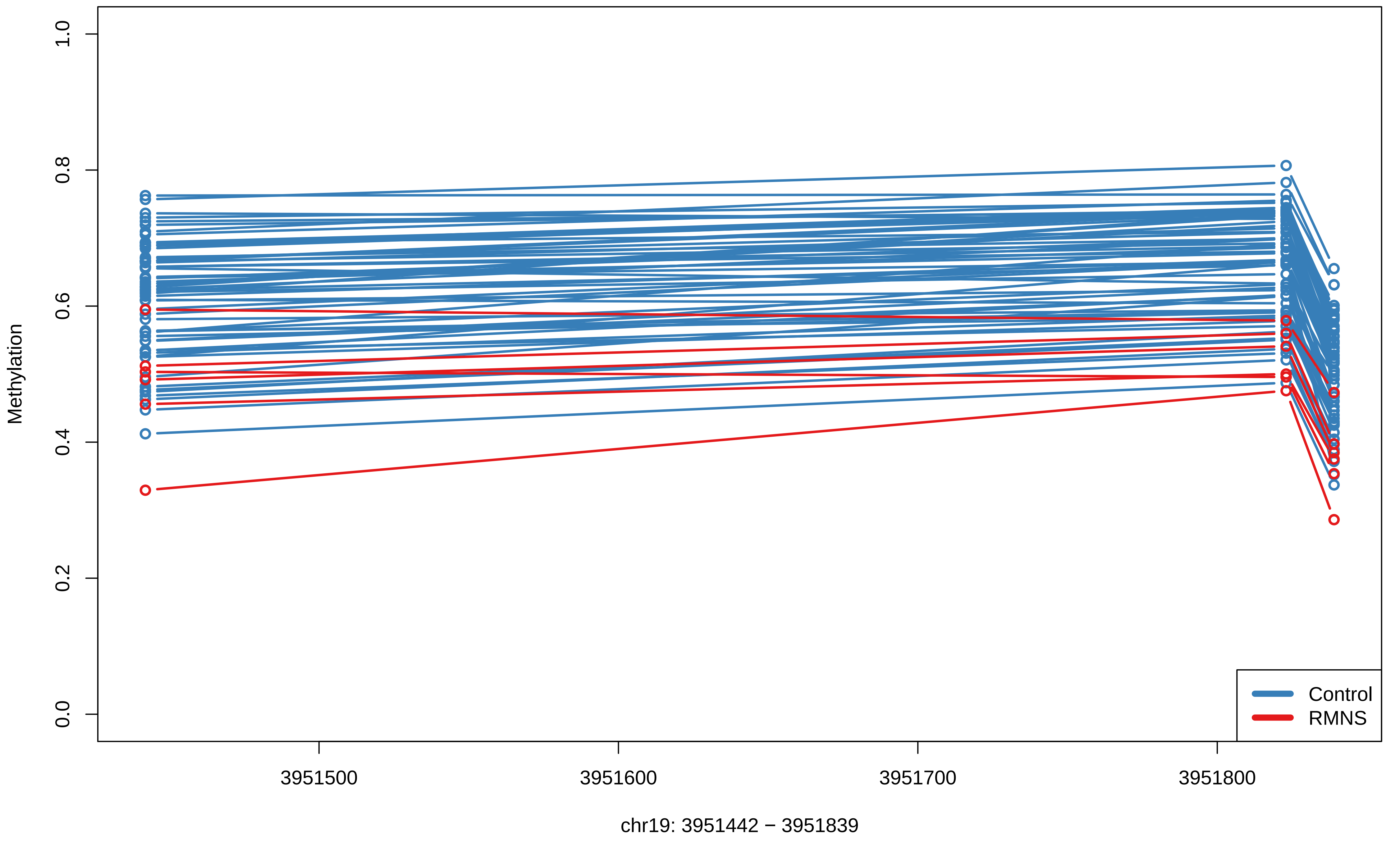

Methylation
1.0
0.8
0.6
0.4
0.2
0.0
3951500
3951600
3951700
3951800
chr19: 3951442 − 3951839
Control
RMNS

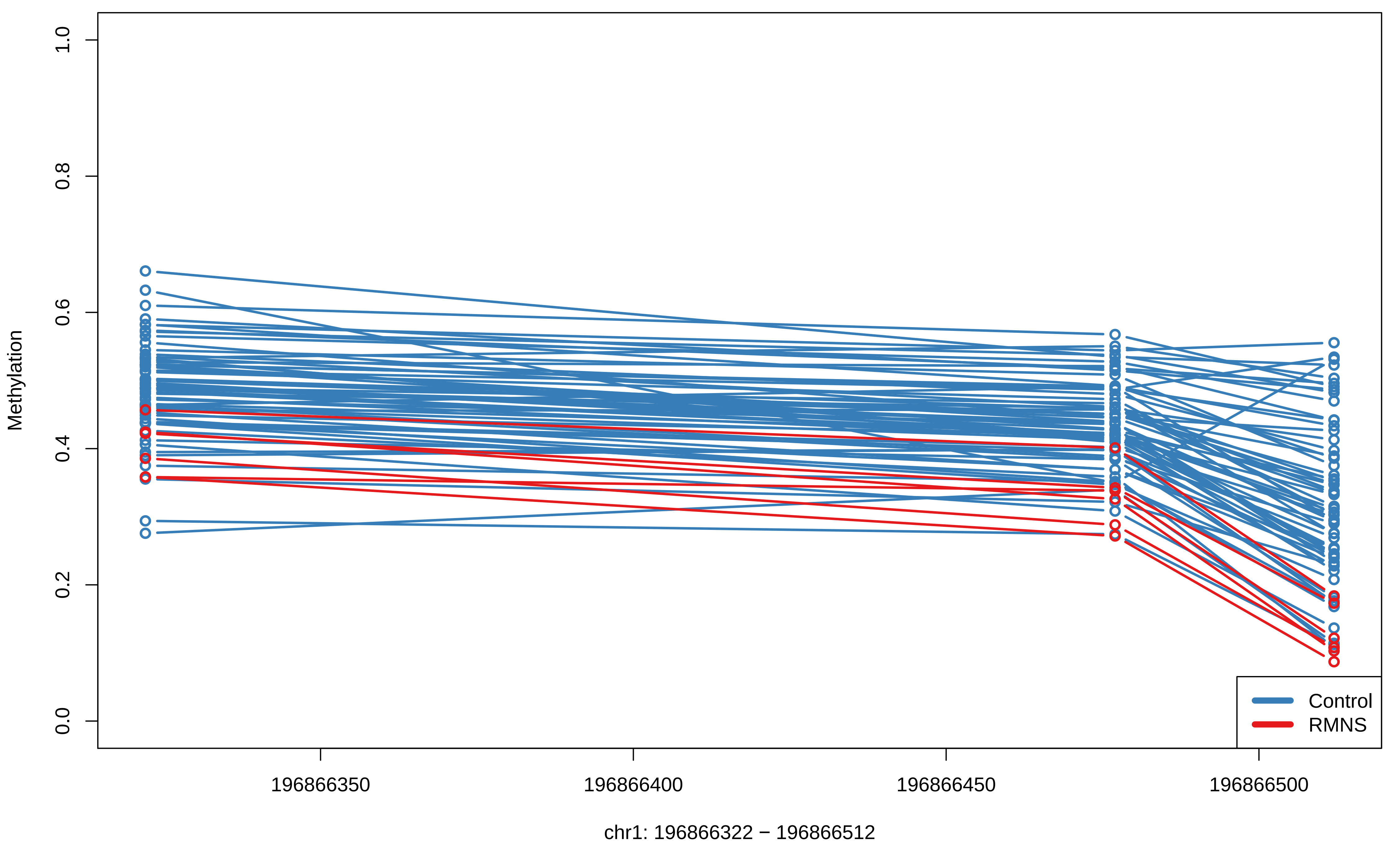

Methylation
0.0
0.2
0.4
0.6
0.8
1.0
196866350
196866400
196866450
196866500
chr1: 196866322 − 196866512
Control
RMNS

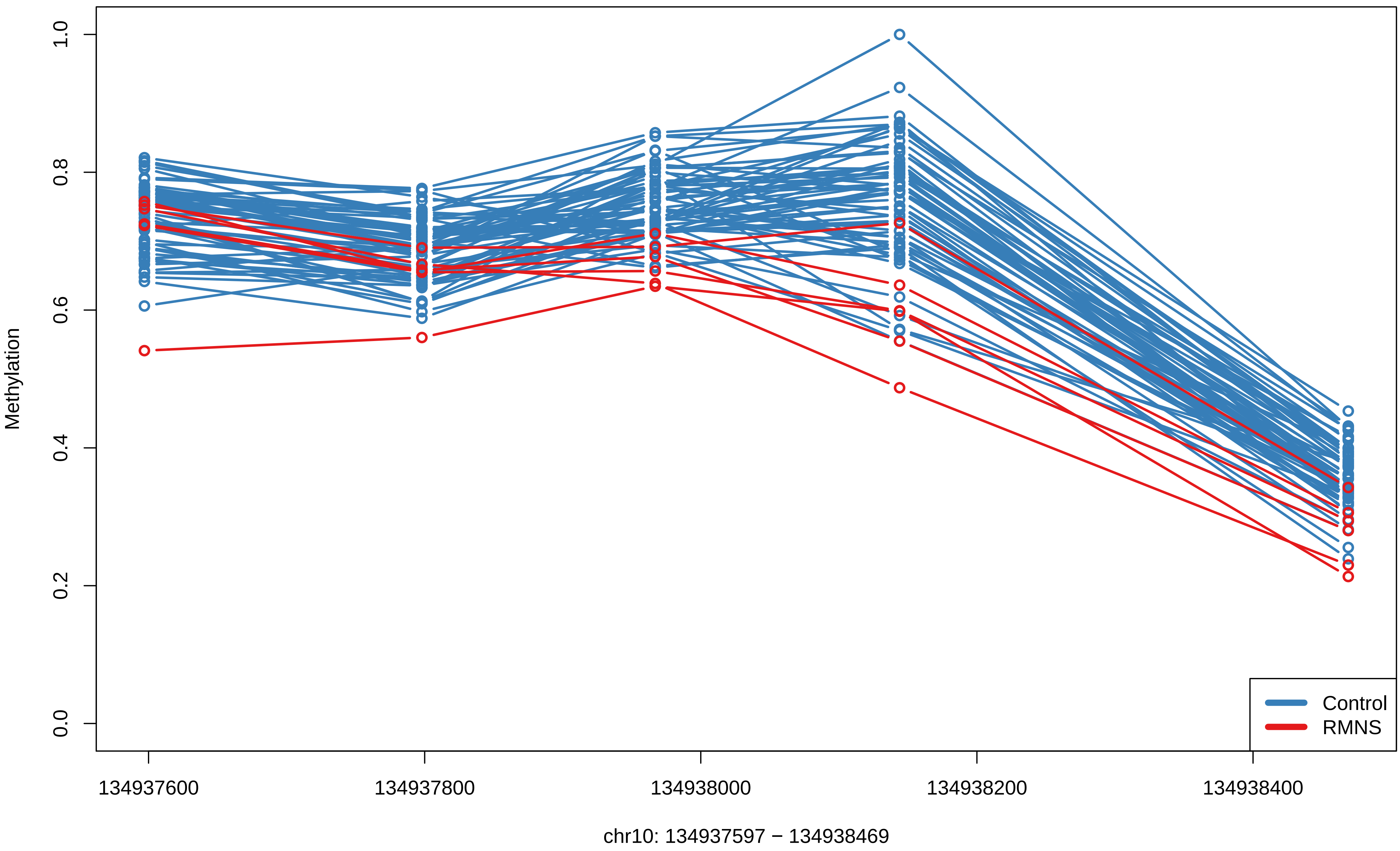

Methylation
0.0
0.2
0.4
0.6
0.8
1.0
134937600
134937800
134938000
134938200
134938400
chr10: 134937597 − 134938469
Control
RMNS

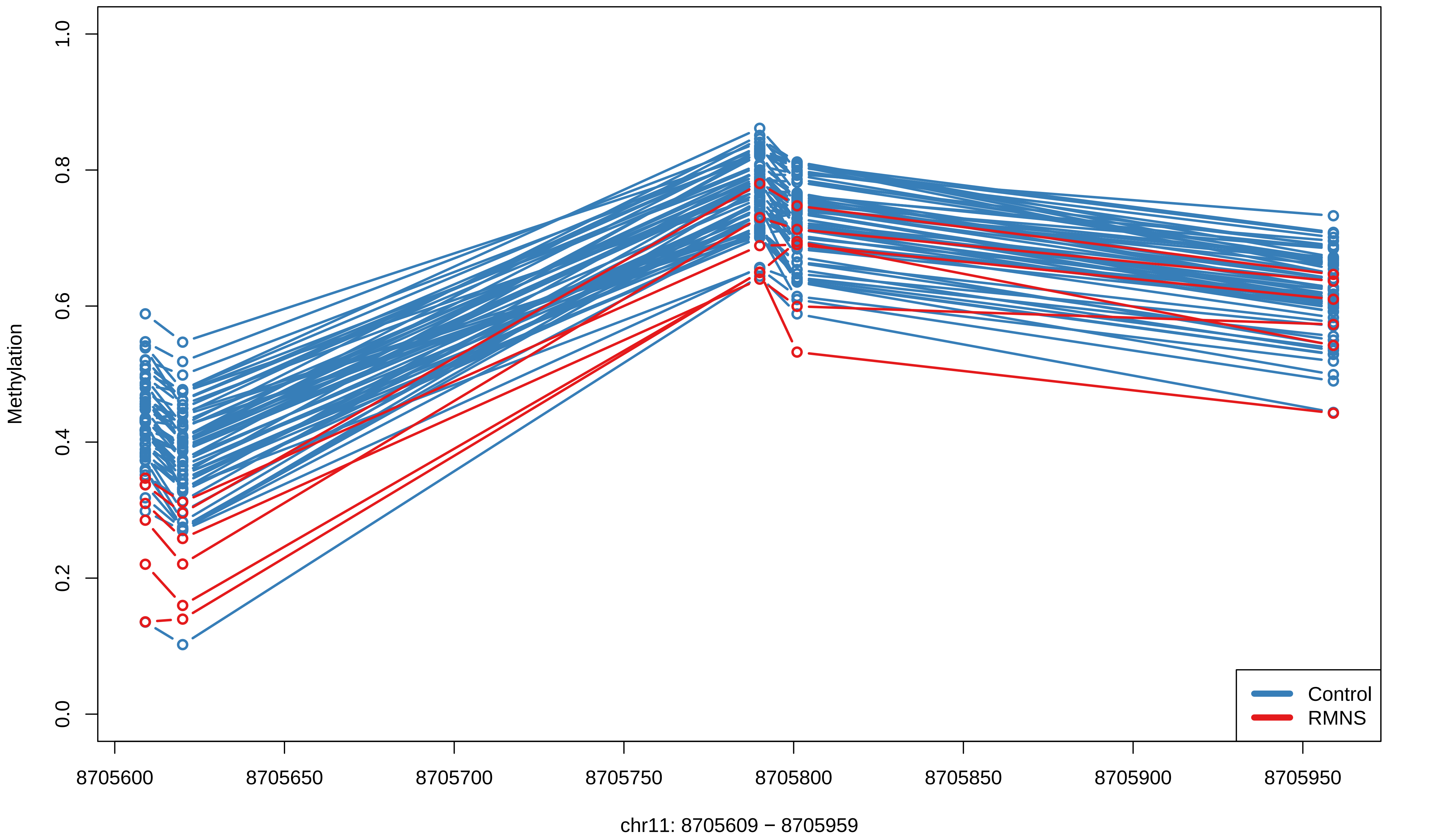

Methylation
1.0
0.8
0.6
0.4
0.2
0.0
8705600
8705650
8705700
8705750
8705800
8705850
8705900
8705950
chr11: 8705609 − 8705959
Control
RMNS

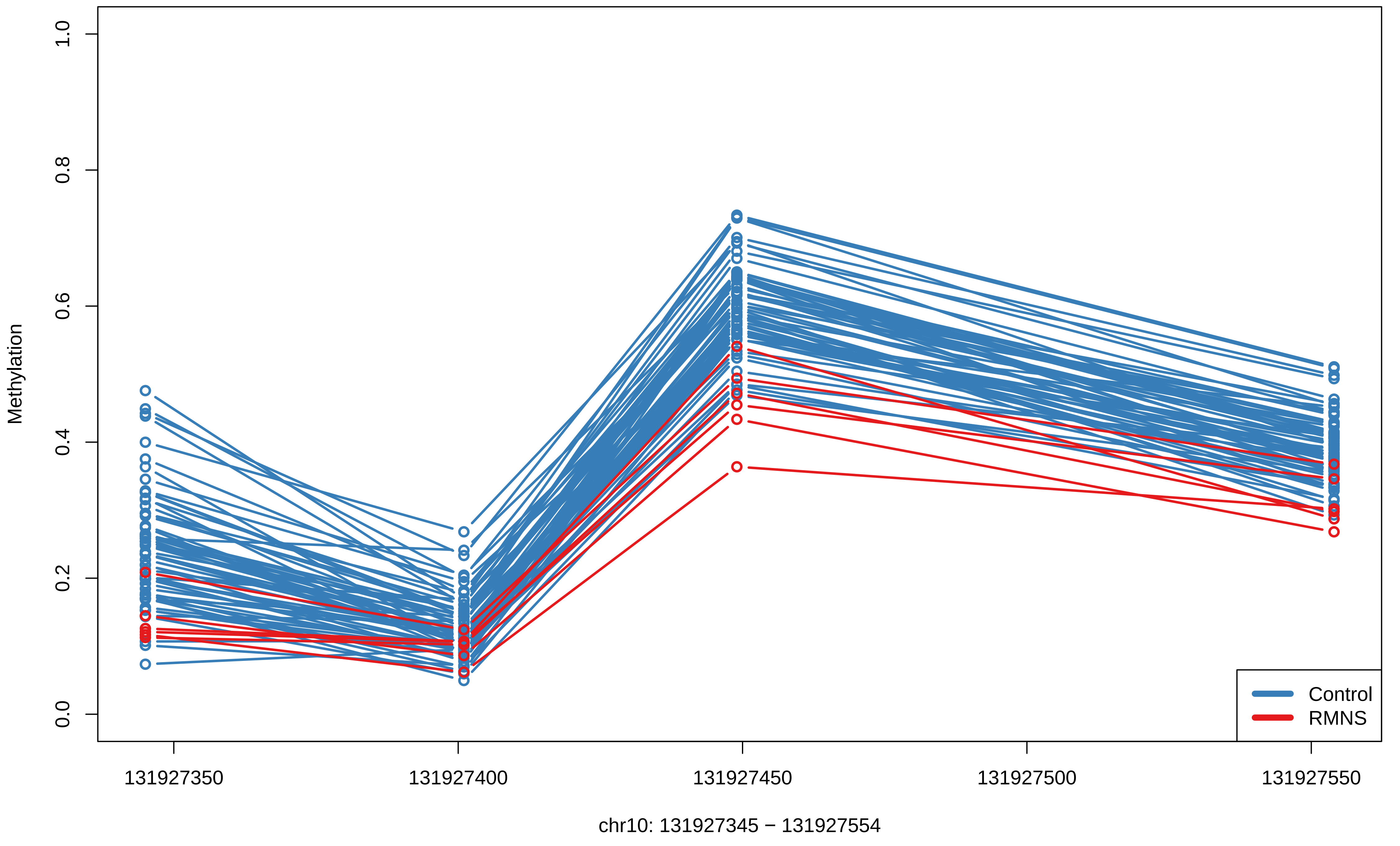

Methylation
0.0
0.2
0.4
0.6
0.8
1.0
131927350
131927400
131927450
131927500
131927550
chr10: 131927345 − 131927554
Control
RMNS

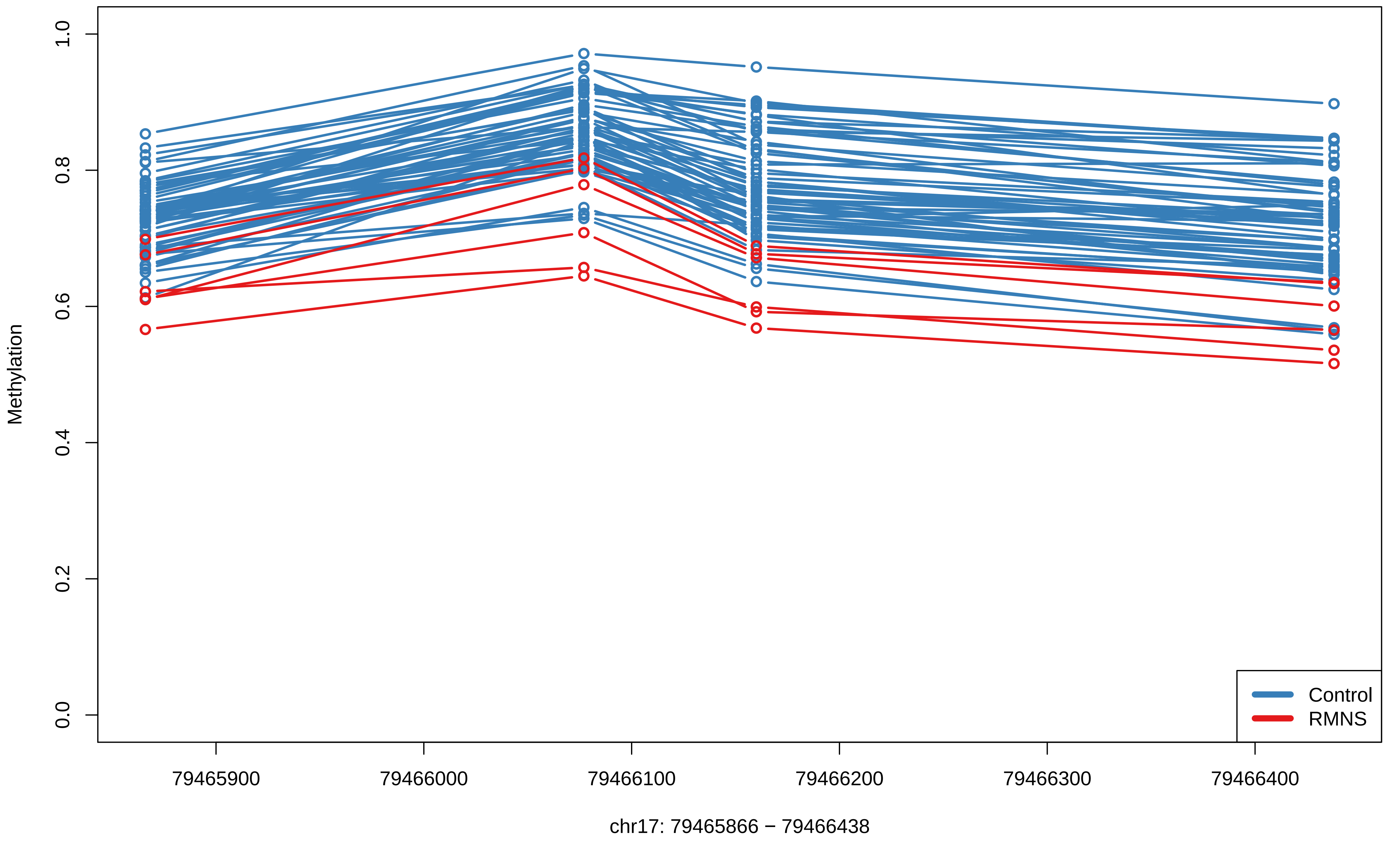

Methylation
0.0
0.2
0.4
0.6
0.8
1.0
79465900
79466000
79466100
79466200
79466300
79466400
chr17: 79465866 − 79466438
Control
RMNS

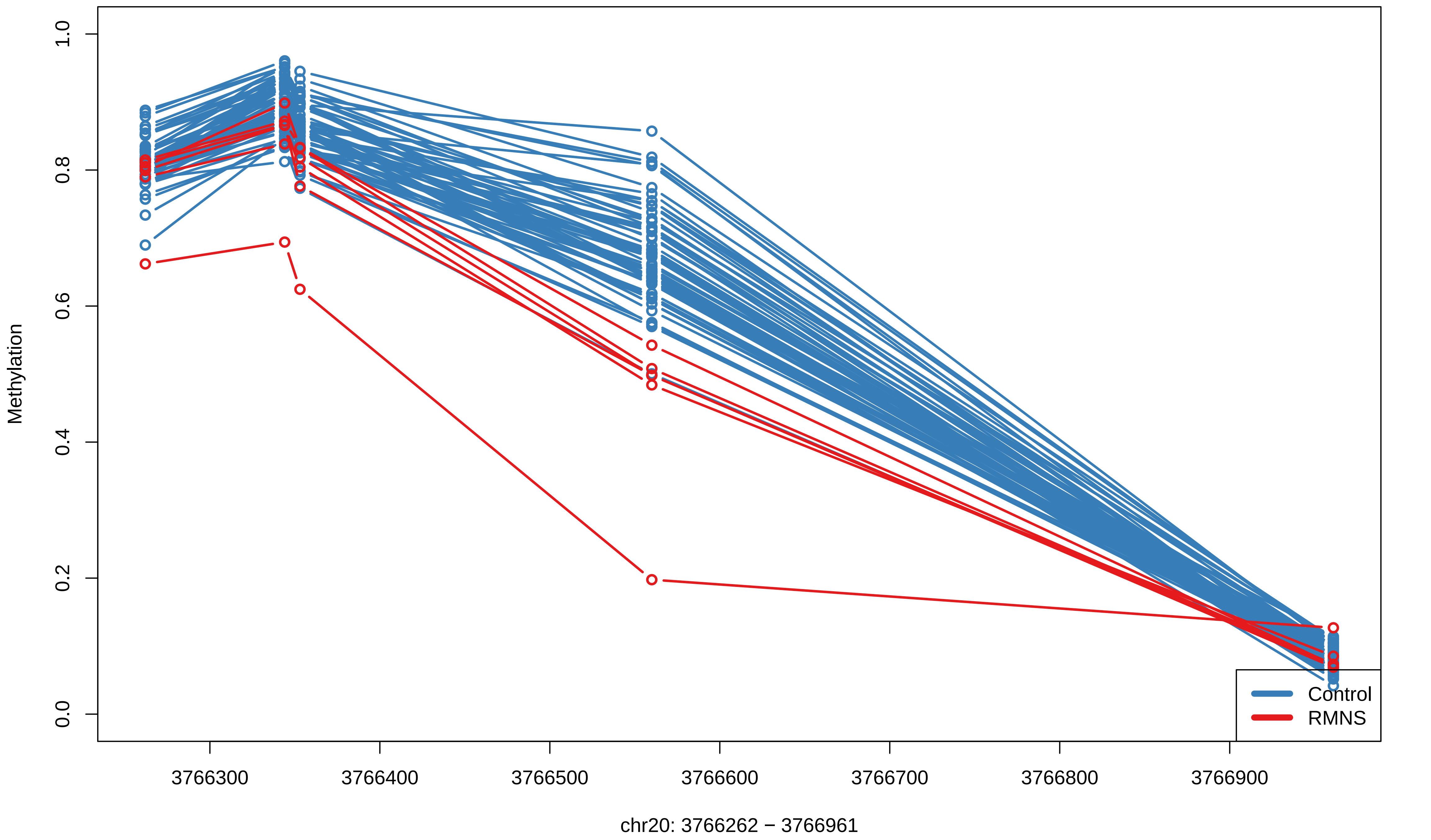

1.0
0.8
0.6
0.4
0.2
0.0
Methylation
3766300
3766400
3766500
3766600
3766700
3766800
3766900
chr20: 3766262 − 3766961
Control
RMNS

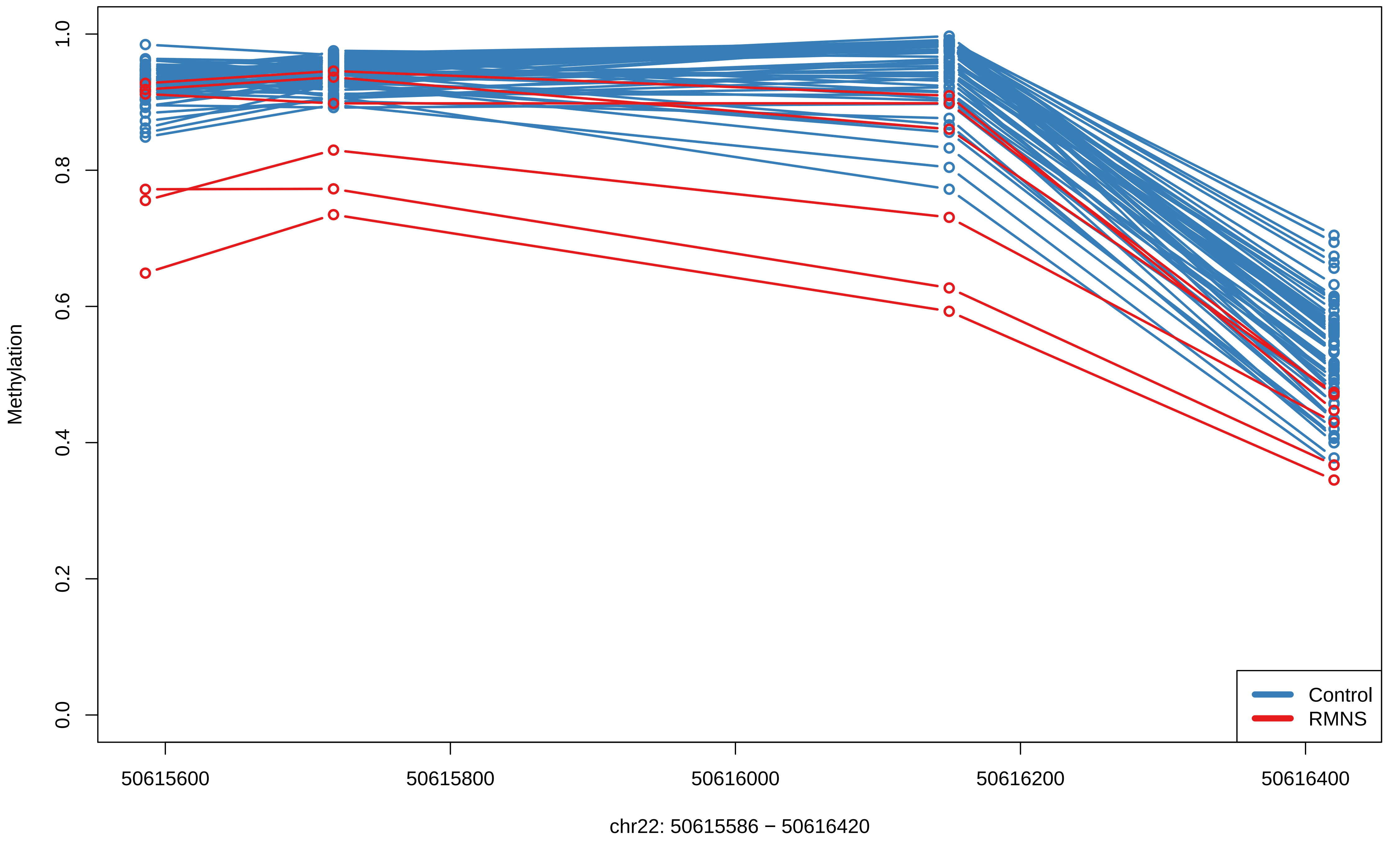

Methylation
1.0
0.8
0.6
0.4
0.2
0.0
50615600
50615800
50616000
50616200
50616400
chr22: 50615586 − 50616420
Control
RMNS

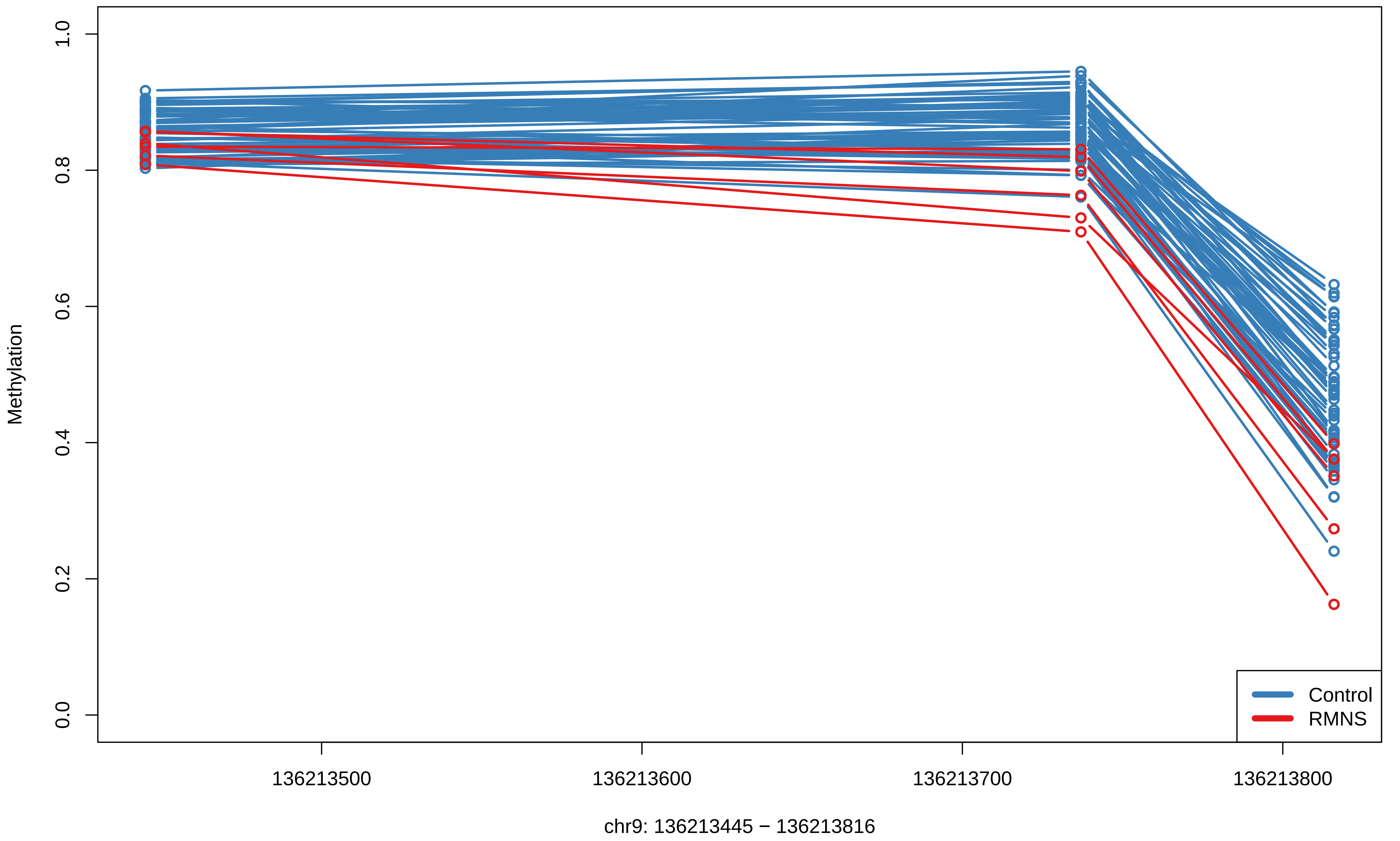

Methylation
0.0
0.2
0.4
0.6
0.8
1.0
136213500
136213600
136213700
136213800
chr9: 136213445 − 136213816
Control
RMNS

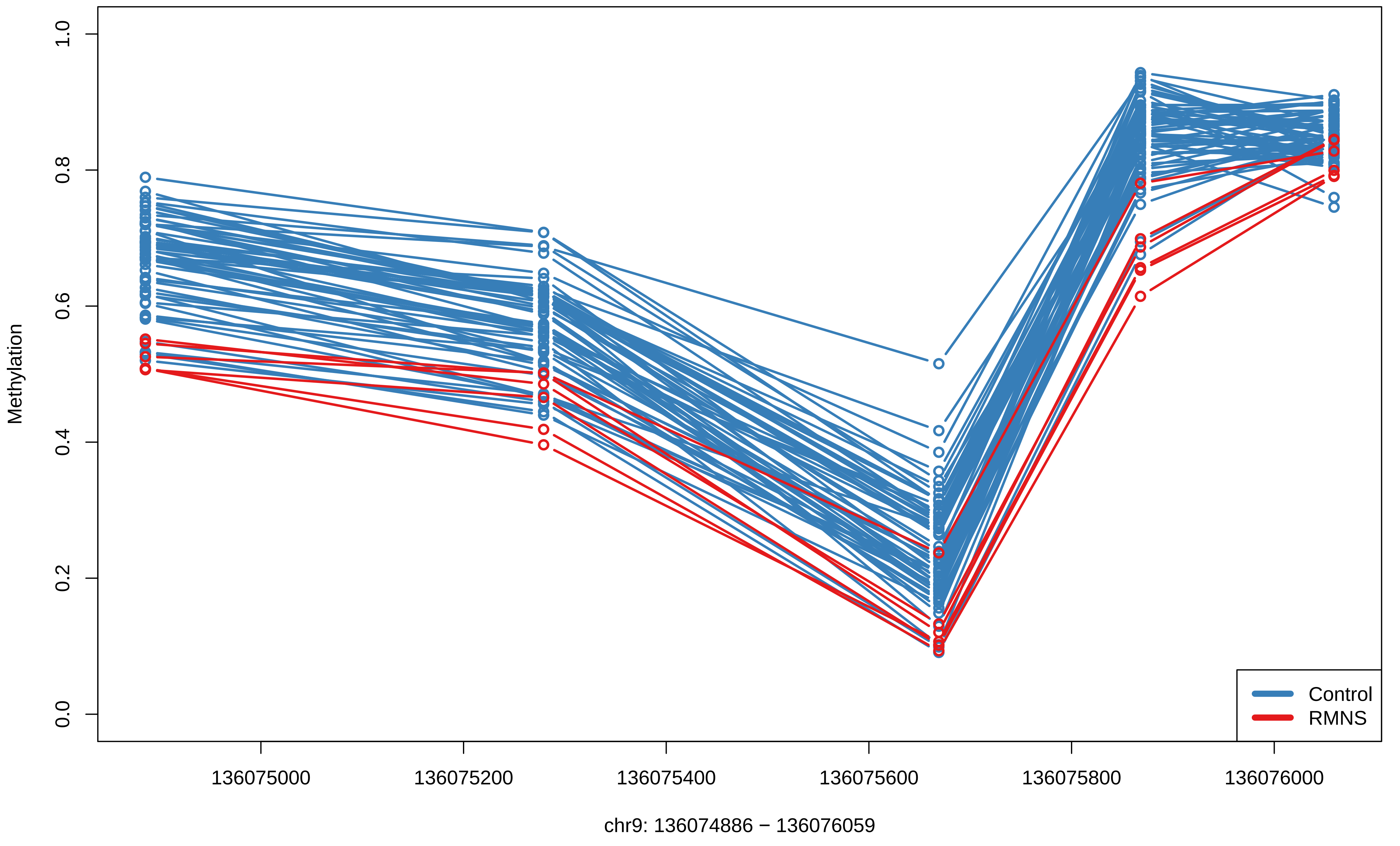

1.0
0.8
0.6
0.4
0.2
0.0
Methylation
136075000
136075200
136075400
136075600
136075800
136076000
chr9: 136074886 − 136076059
Control
RMNS

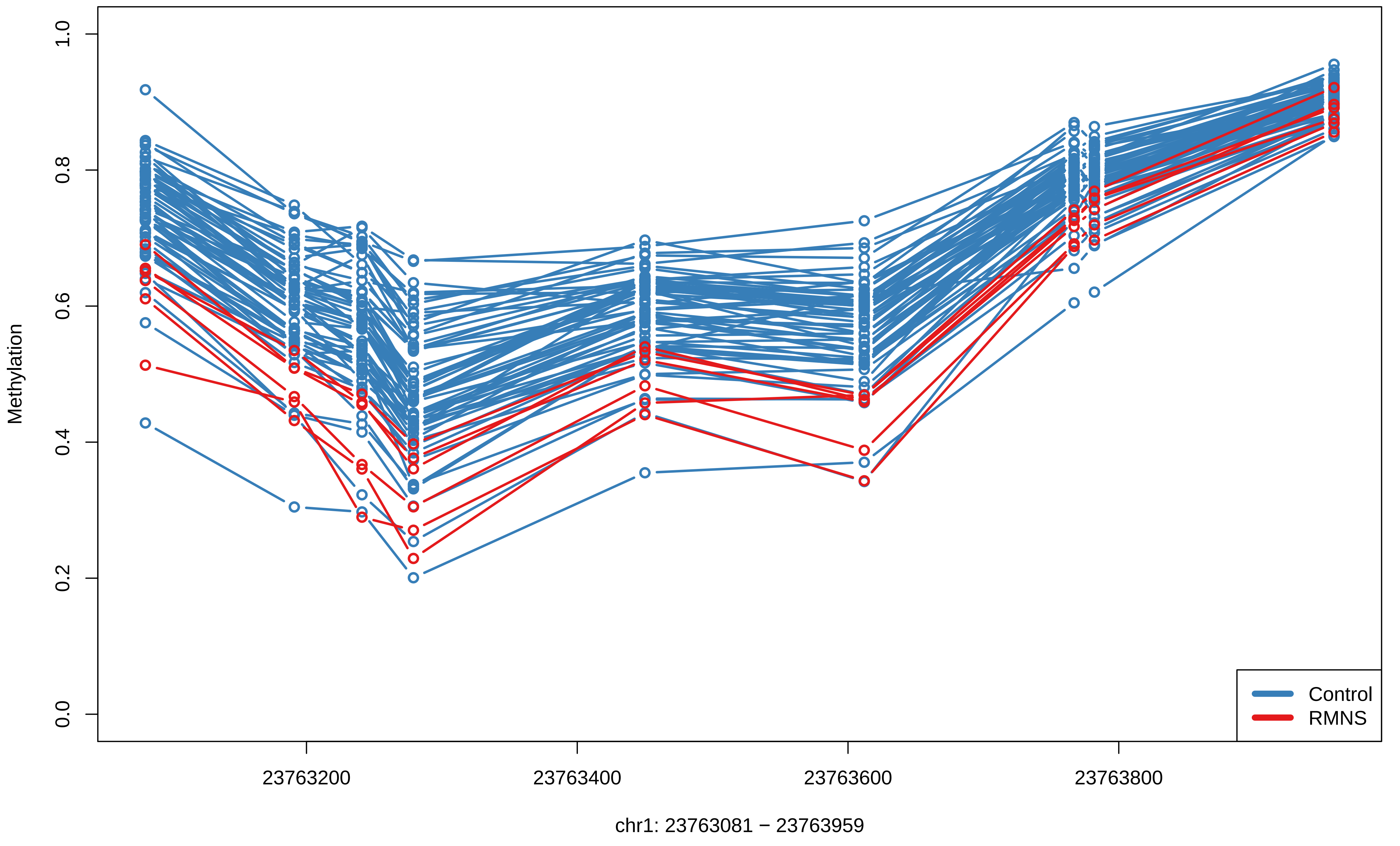

Methylation
0.0
0.2
0.4
0.6
0.8
1.0
23763200
23763400
23763600
23763800
chr1: 23763081 − 23763959
Control
RMNS

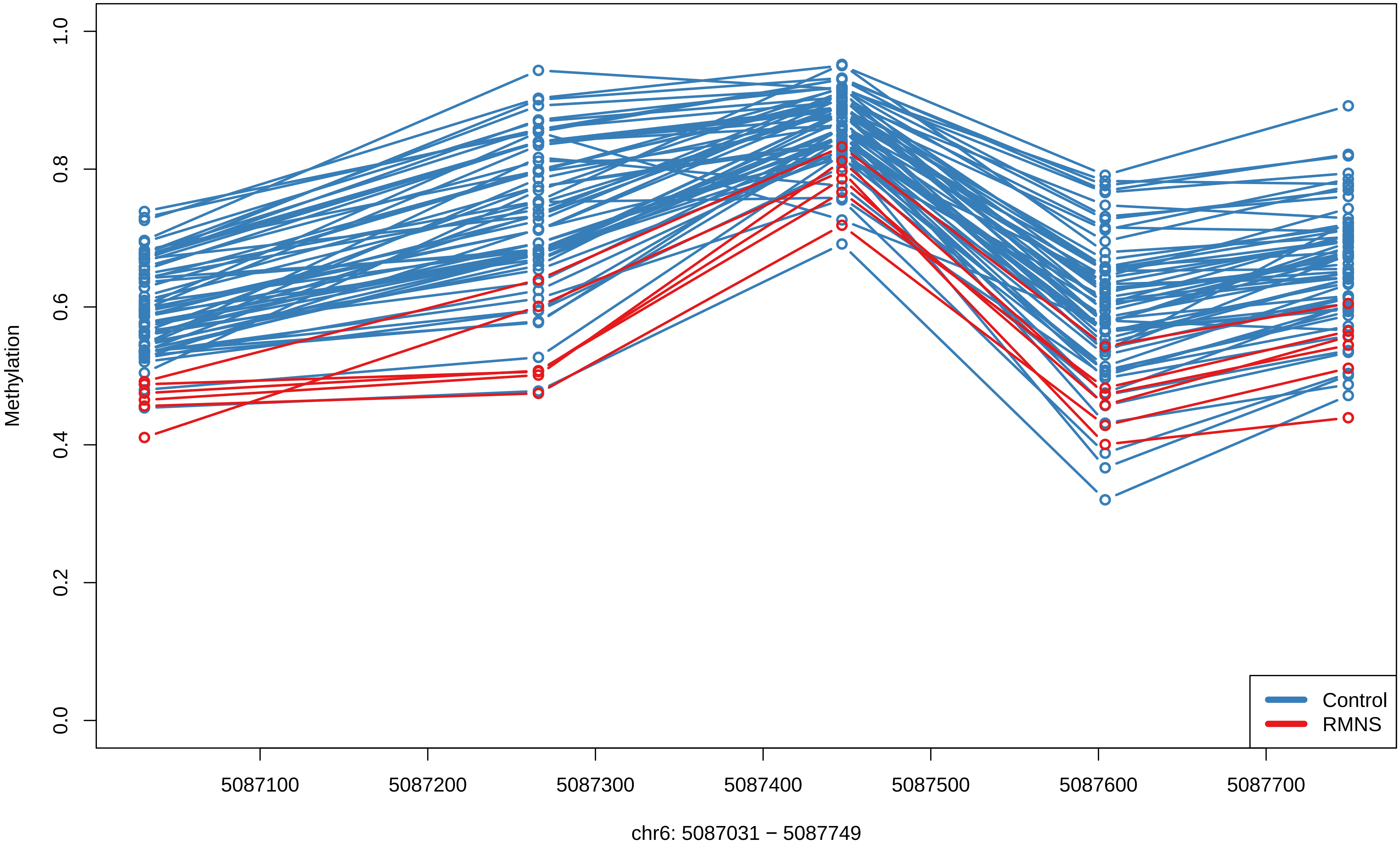

Methylation
1.0
0.8
0.6
0.4
0.2
0.0
5087100
5087200
5087300
5087400
5087500
5087600
5087700
chr6: 5087031 − 5087749
Control
RMNS

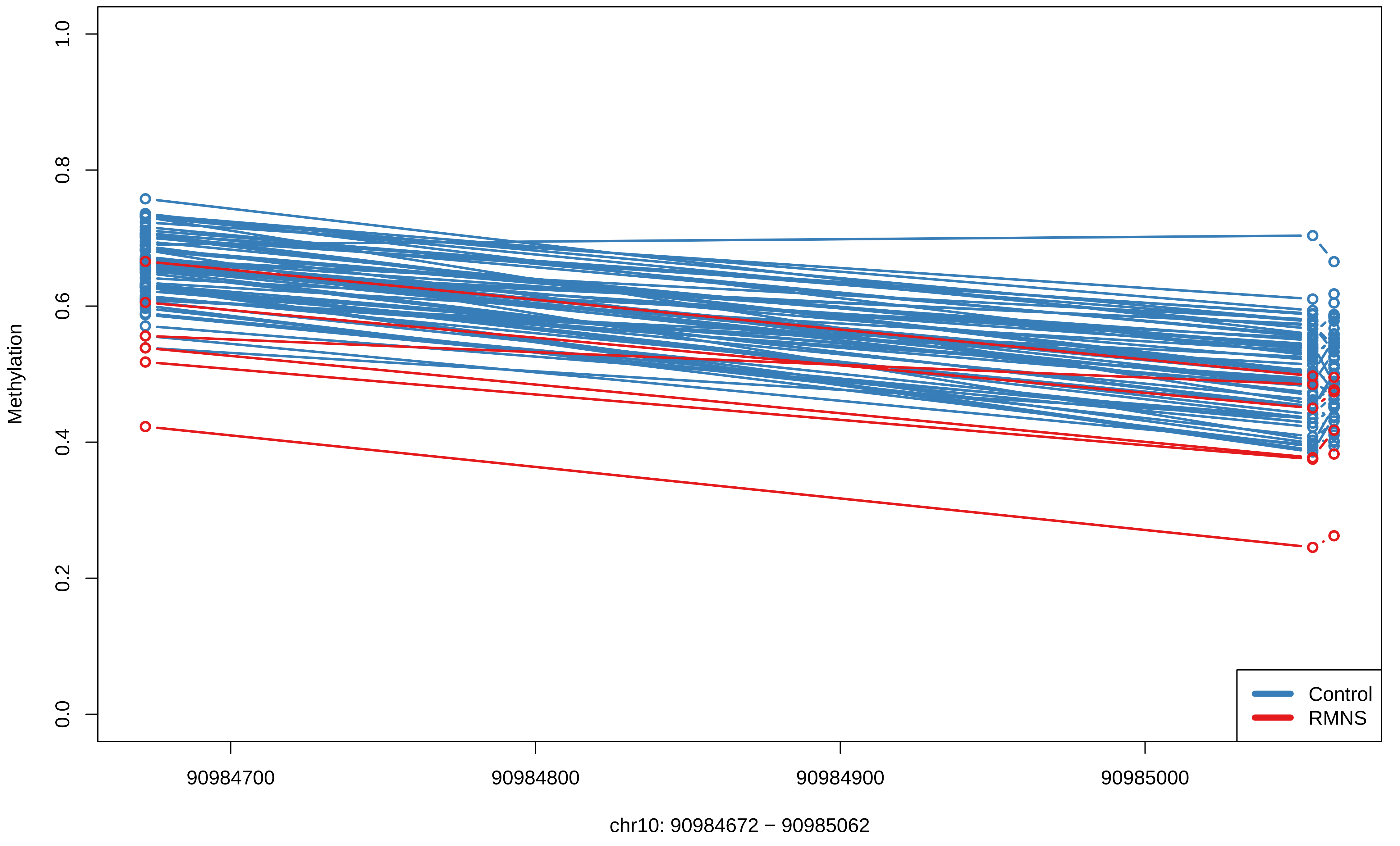

Methylation
1.0
0.8
0.6
0.4
0.2
0.0
90984700
90984800
90984900
90985000
chr10: 90984672 − 90985062
Control
RMNS

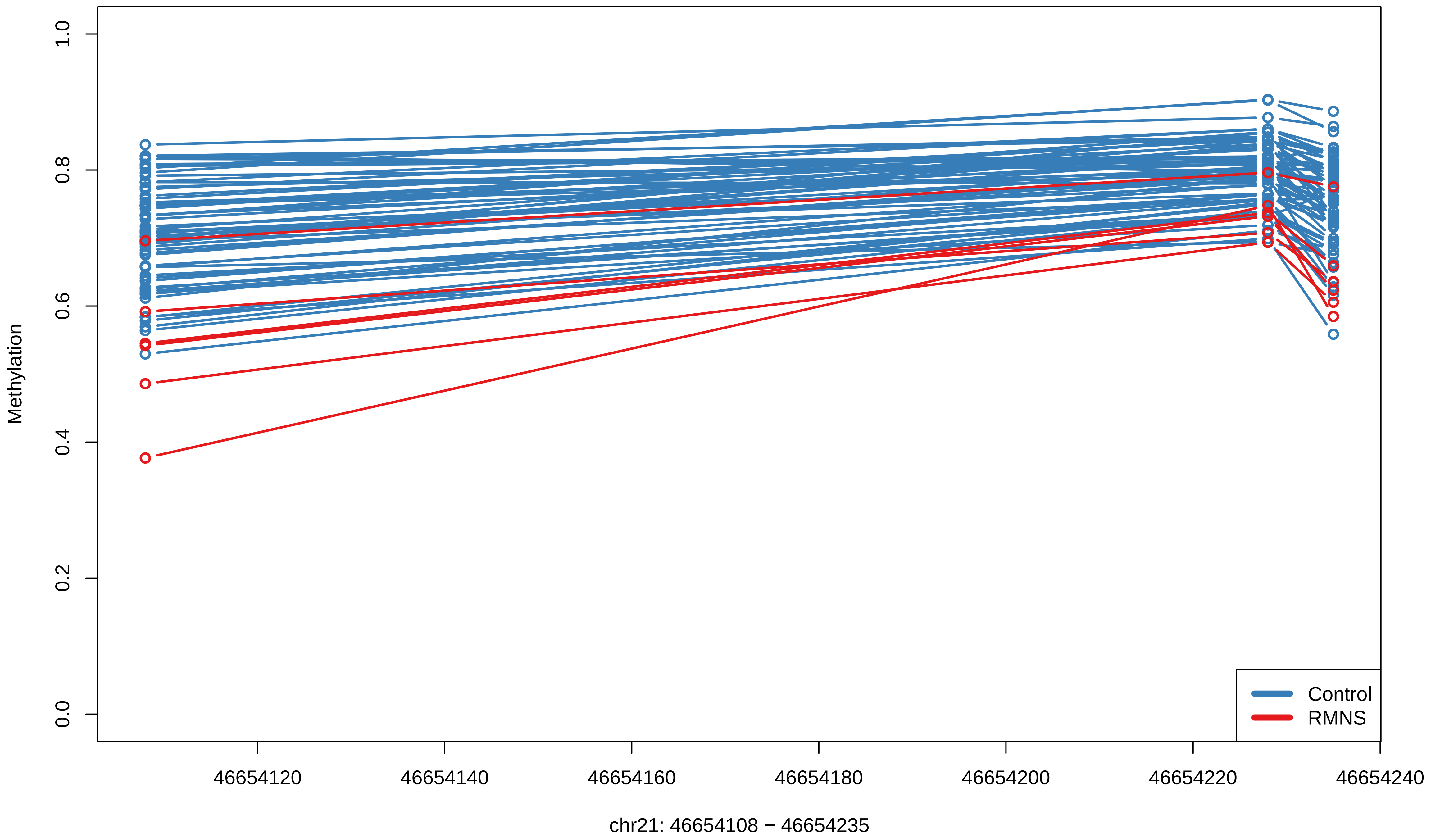

Methylation
0.0
0.2
0.4
0.6
0.8
1.0
46654120
46654140
46654160
46654180
46654200
46654220
46654240
chr21: 46654108 − 46654235
Control
RMNS

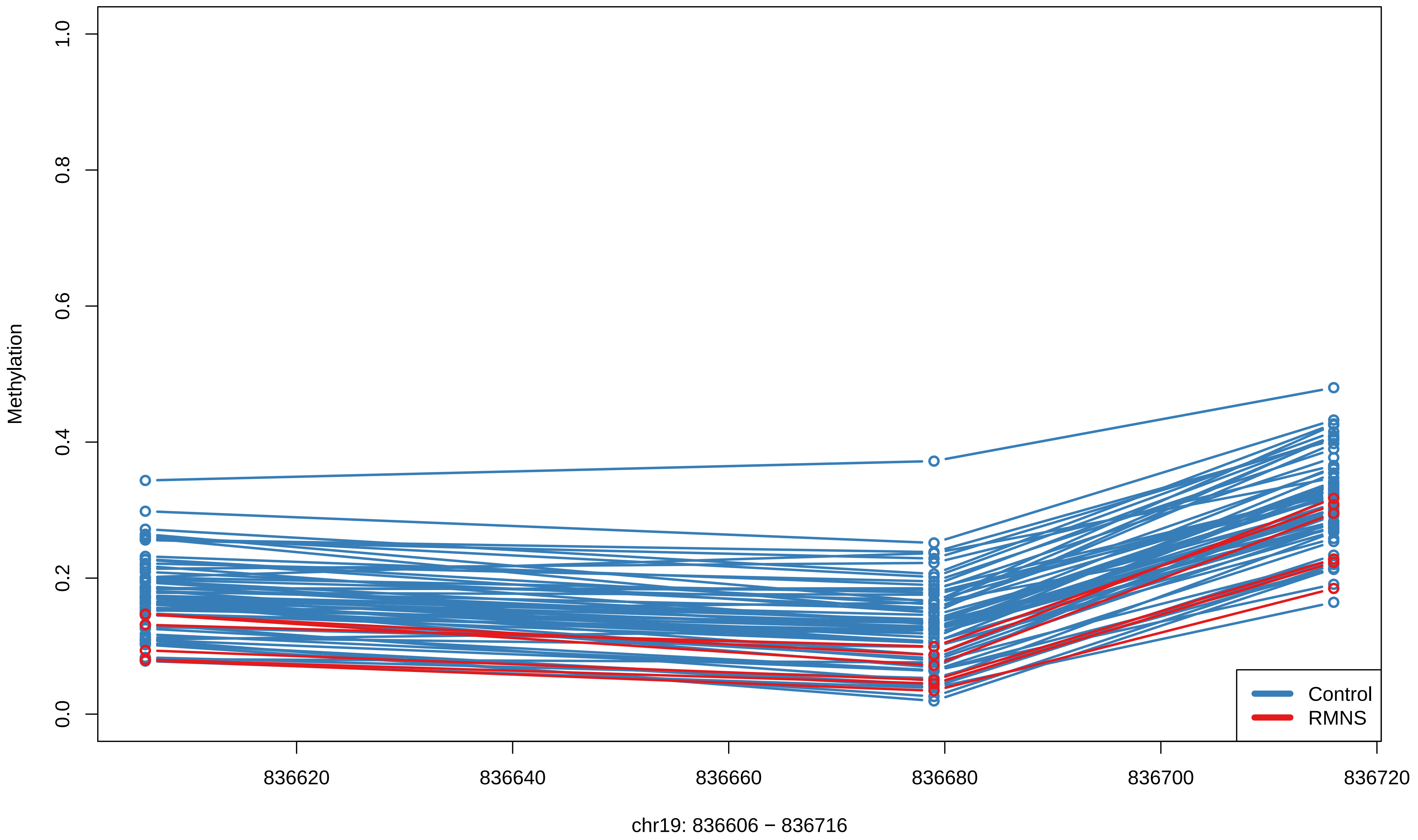

Methylation
0.0
0.2
0.4
0.6
0.8
1.0
836620
836640
836660
836680
836700
836720
chr19: 836606 − 836716
Control
RMNS

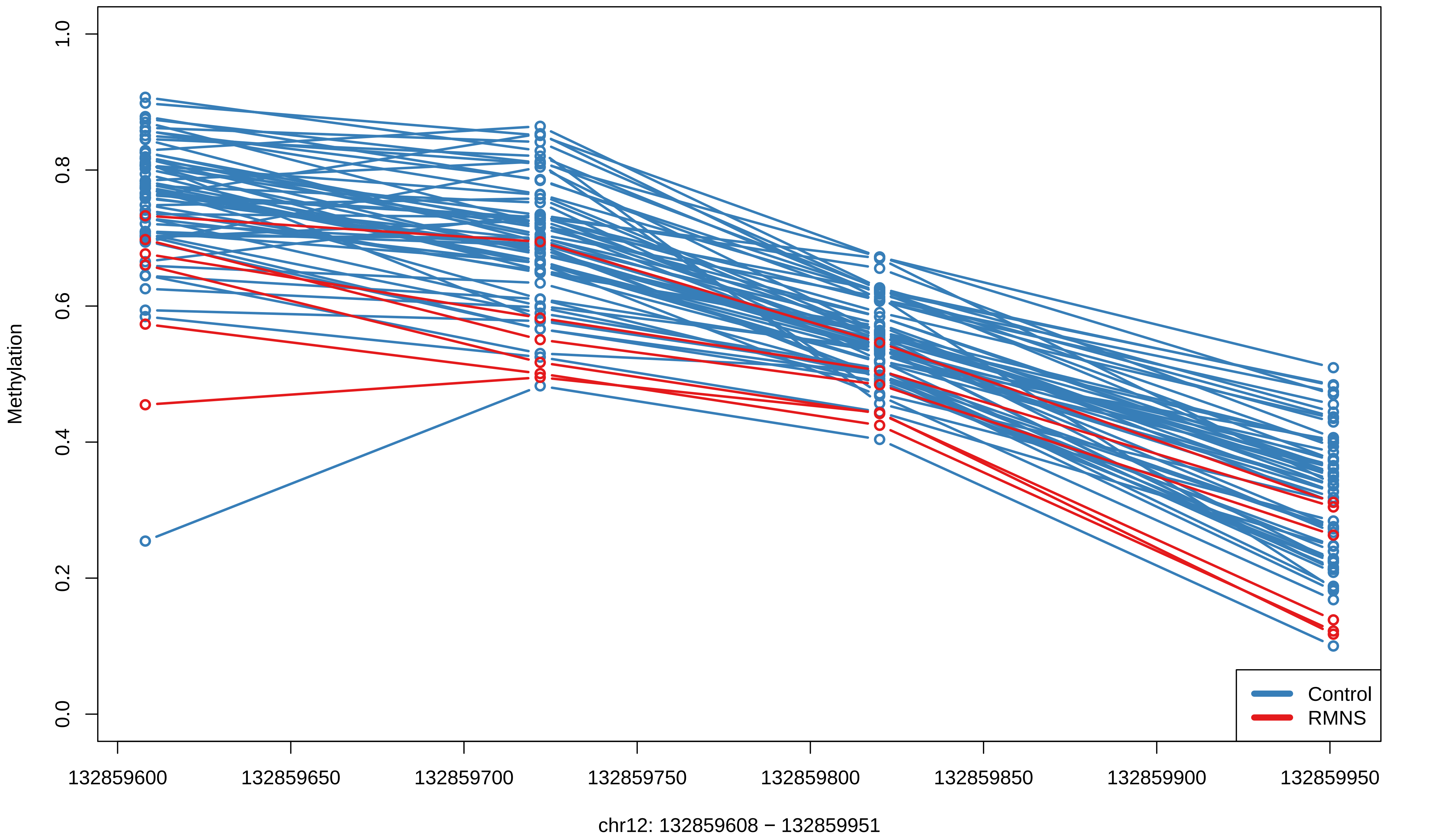
Methylation
0.0
0.2
0.4
0.6
0.8
1.0
132859600
132859650
132859700
132859750
132859800
132859850
132859900
132859950
chr12: 132859608 − 132859951
Control
RMNS

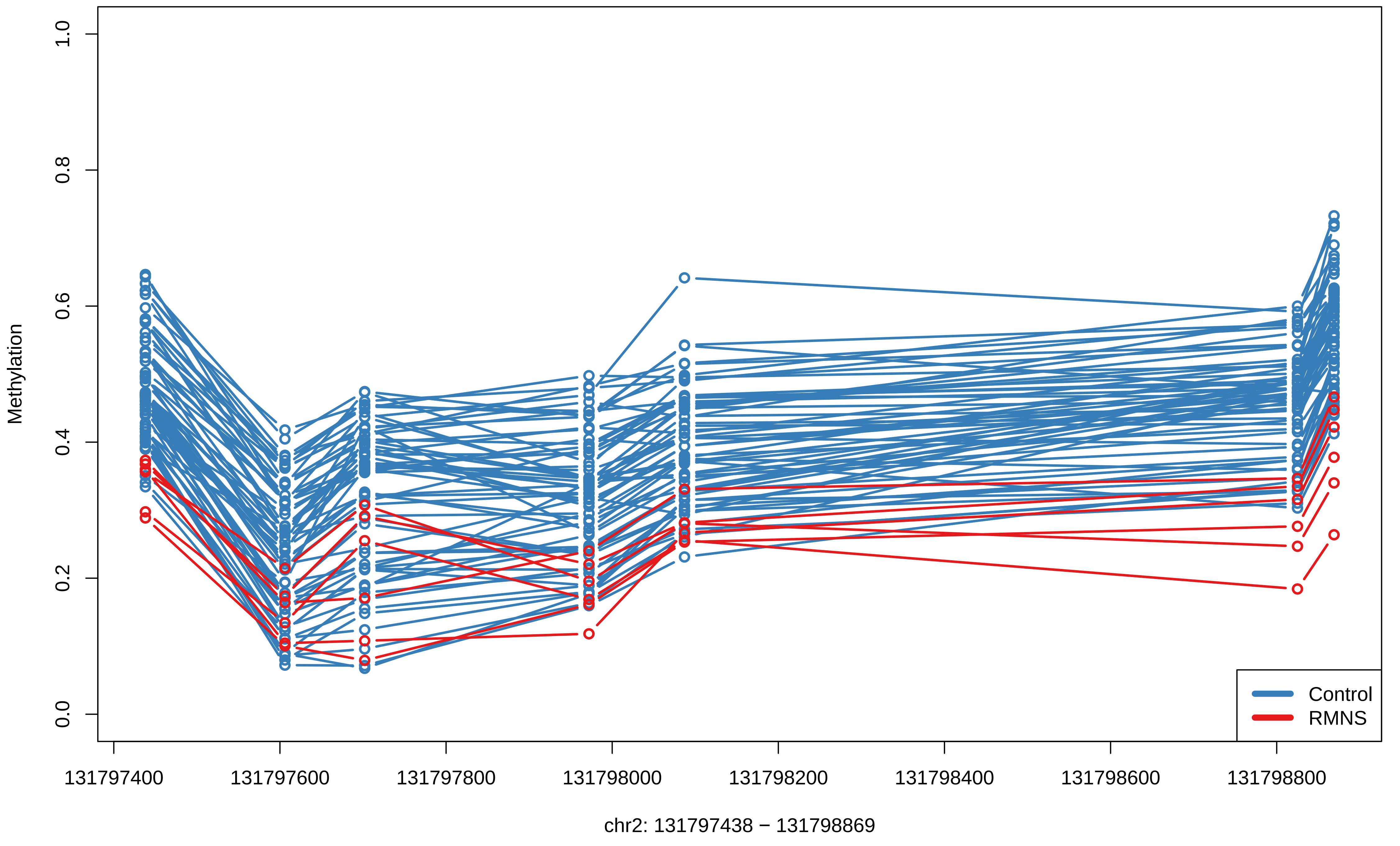

Methylation
0.0
0.2
0.4
0.6
0.8
1.0
131797400
131797600
131797800
131798000
131798200
131798400
131798600
131798800
chr2: 131797438 − 131798869
Control
RMNS

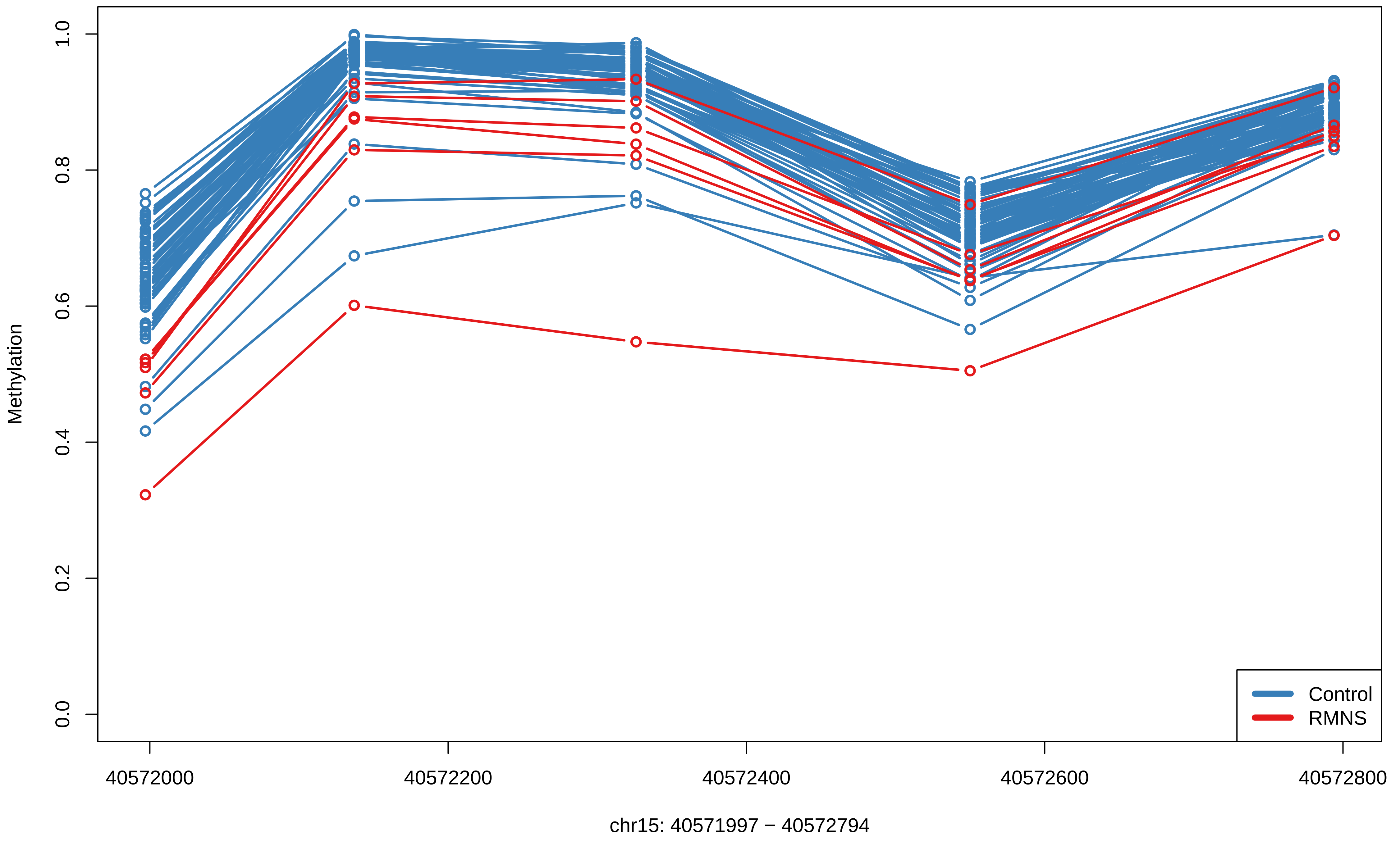

Methylation
1.0
0.8
0.6
0.4
0.2
0.0
40572000
40572200
40572400
40572600
40572800
chr15: 40571997 − 40572794
Control
RMNS

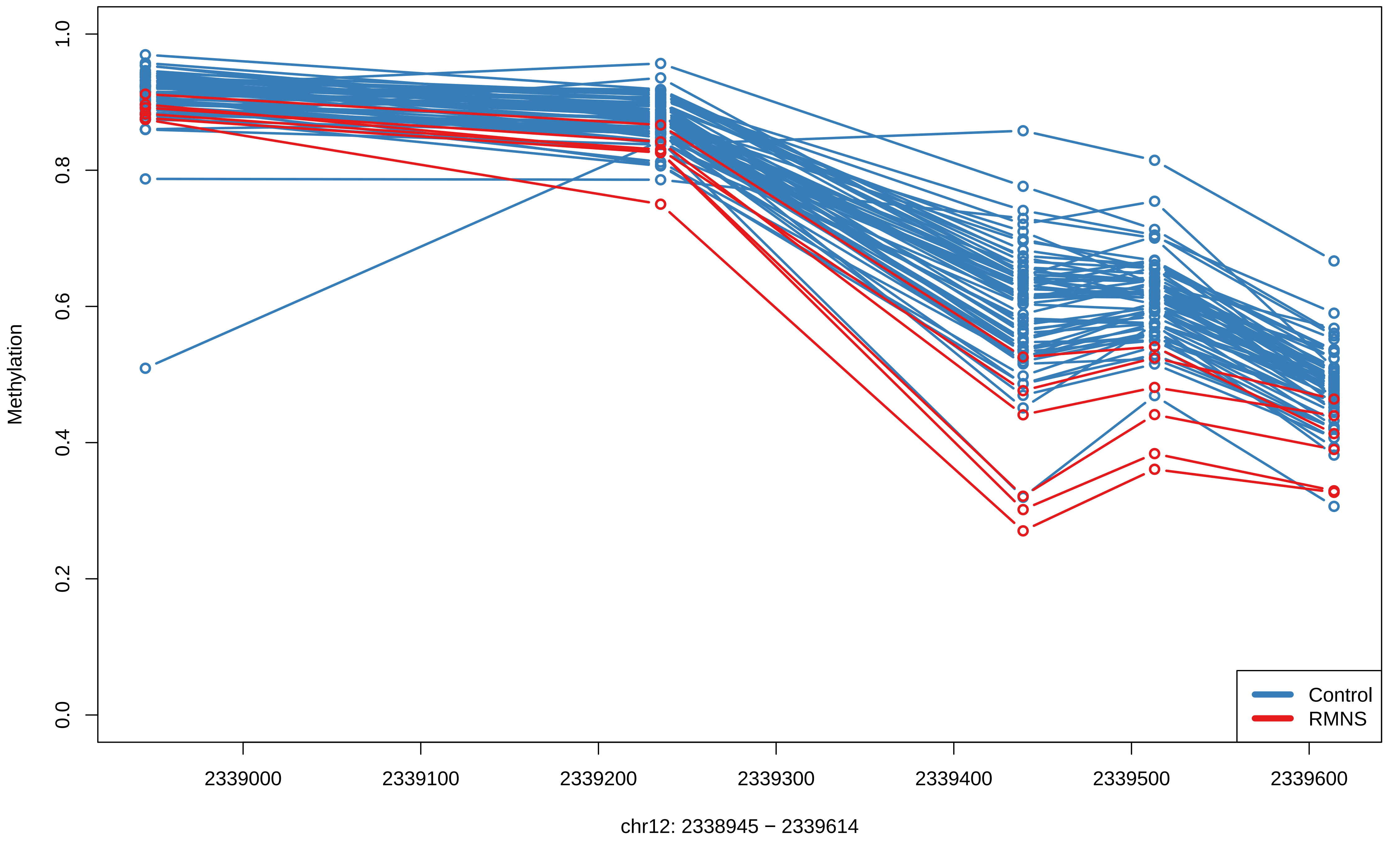

Methylation
0.0
0.2
0.4
0.6
0.8
1.0
2339000
2339100
2339200
2339300
2339400
2339500
2339600
chr12: 2338945 − 2339614
Control
RMNS

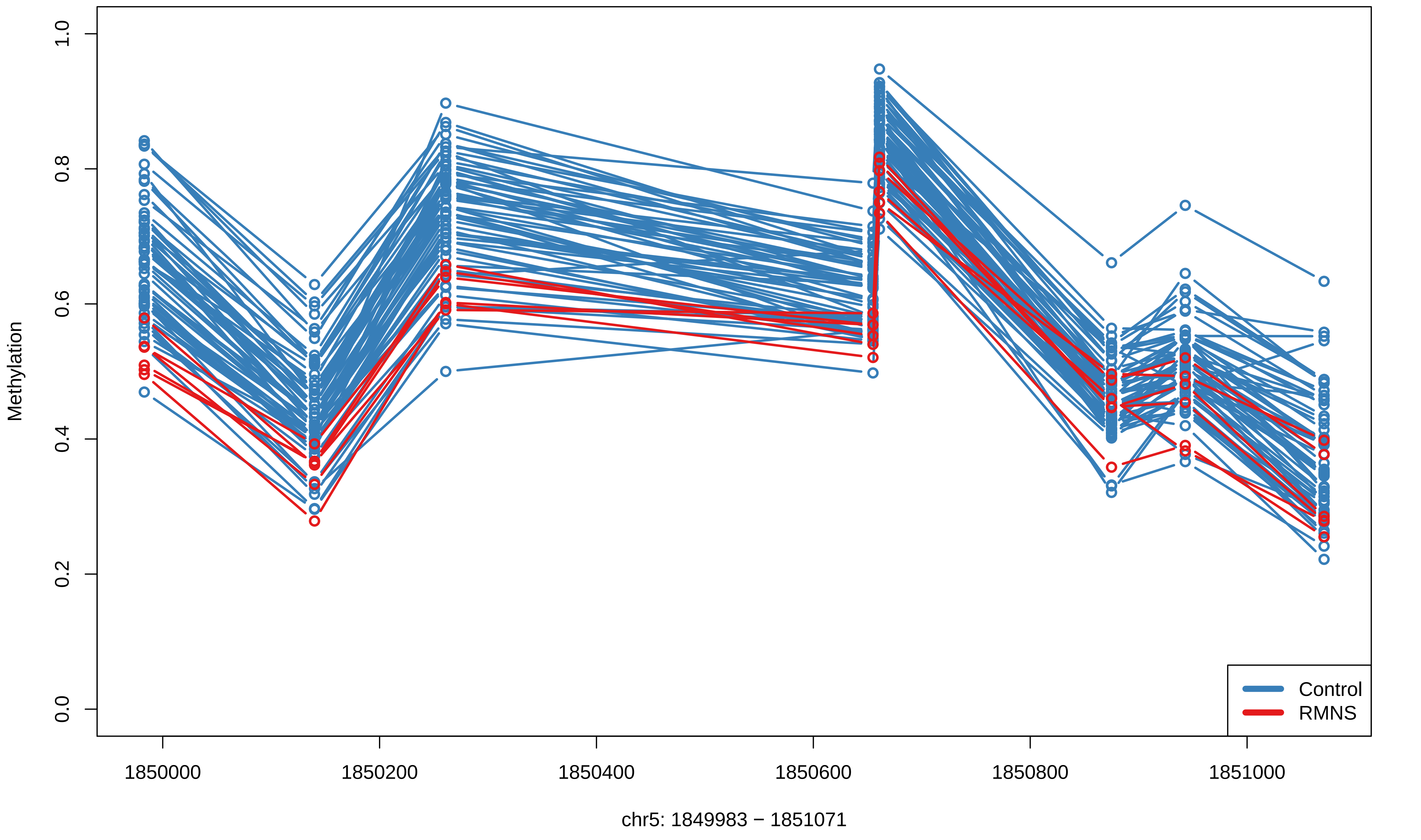

Methylation
0.0
0.2
0.4
0.6
0.8
1.0
1850000
1850200
1850400
1850600
1850800
1851000
chr5: 1849983 − 1851071
Control
RMNS

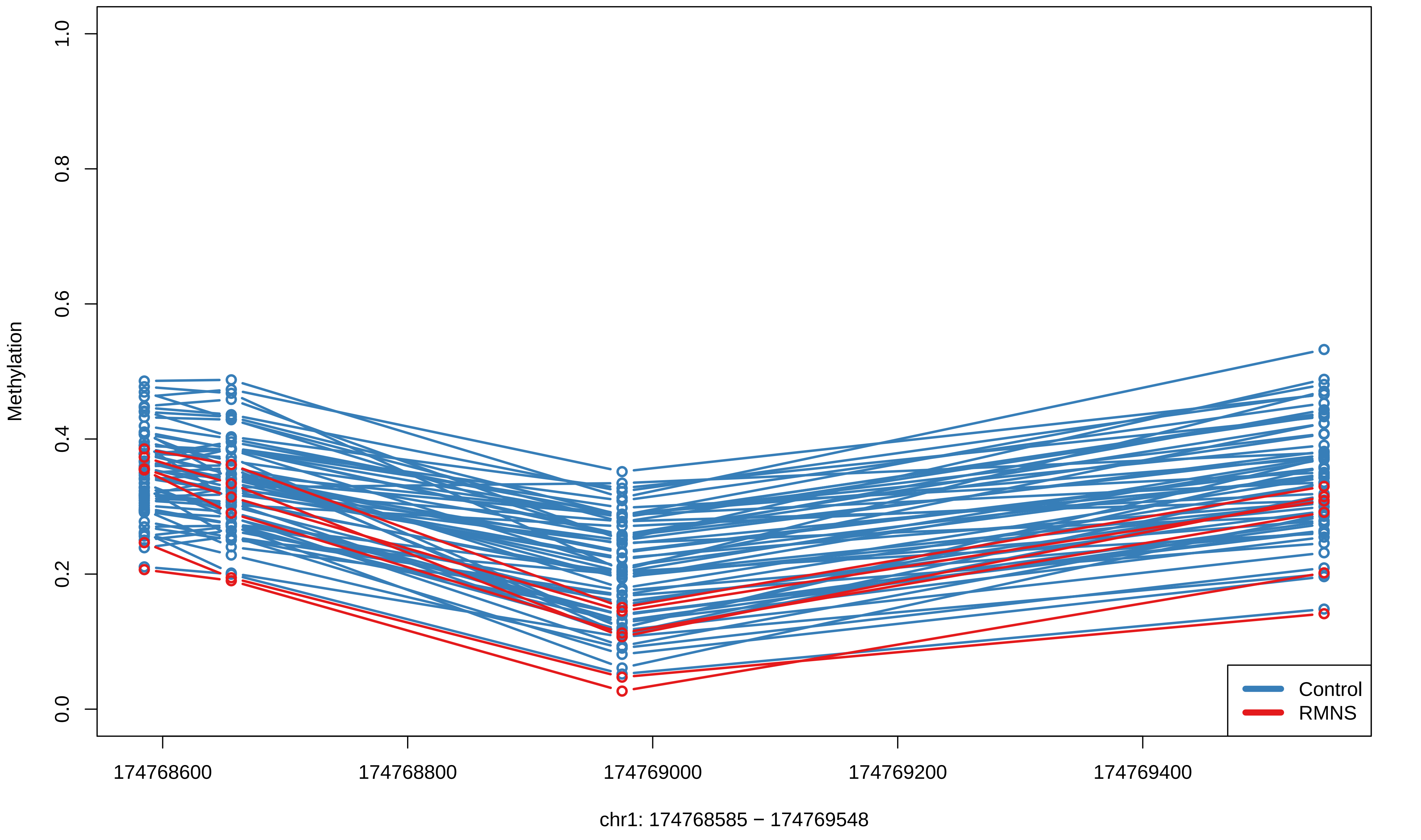

Methylation
0.0
0.2
0.4
0.6
0.8
1.0
174768600
174768800
174769000
174769200
174769400
chr1: 174768585 − 174769548
Control
RMNS

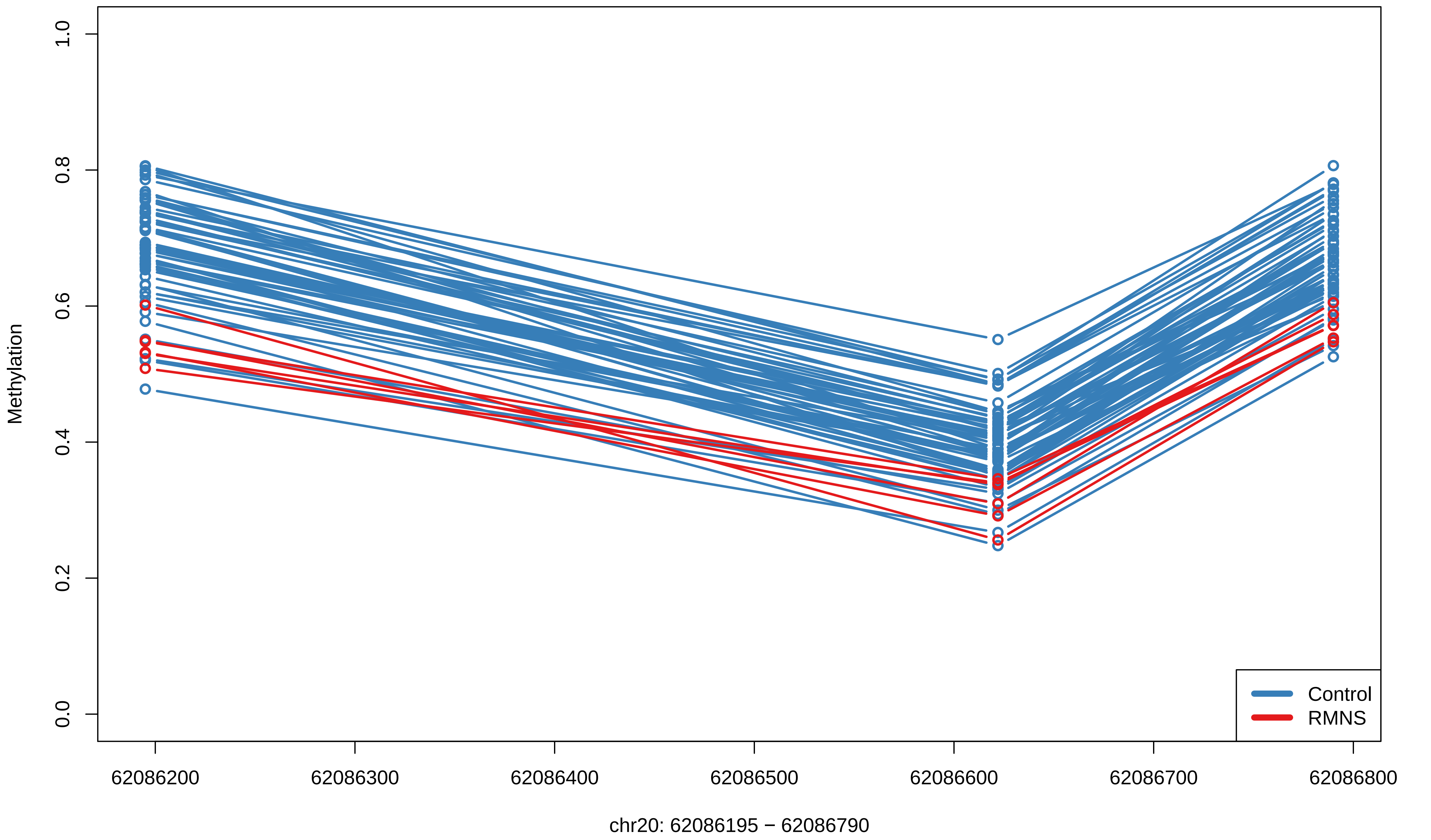

Methylation
1.0
0.8
0.6
0.4
0.2
0.0
62086200
62086300
62086400
62086500
62086600
62086700
62086800
chr20: 62086195 − 62086790
Control
RMNS

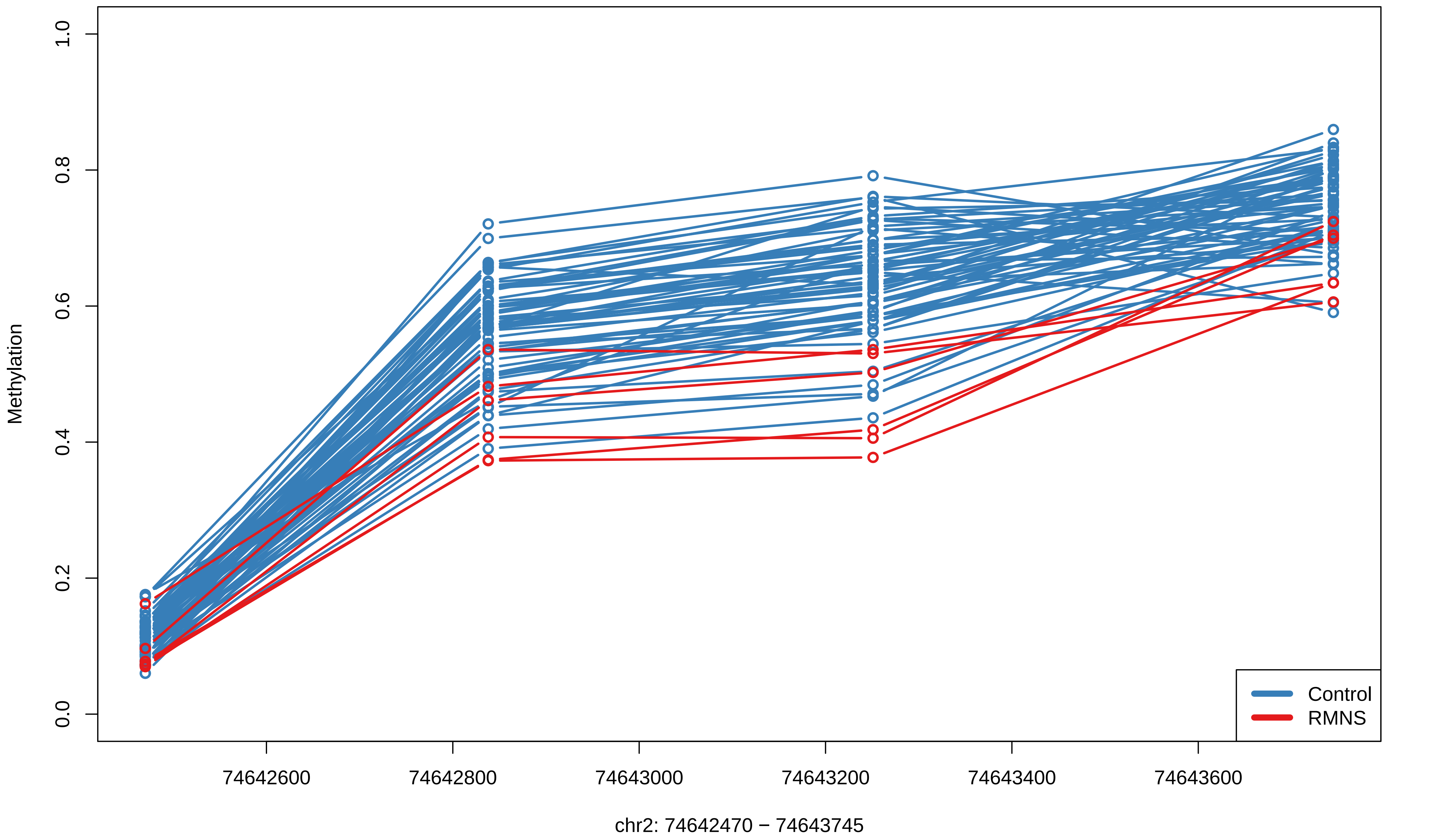

Methylation
1.0
0.8
0.6
0.4
0.2
0.0
74642600
74642800
74643000
74643200
74643400
74643600
chr2: 74642470 − 74643745
Control
RMNS

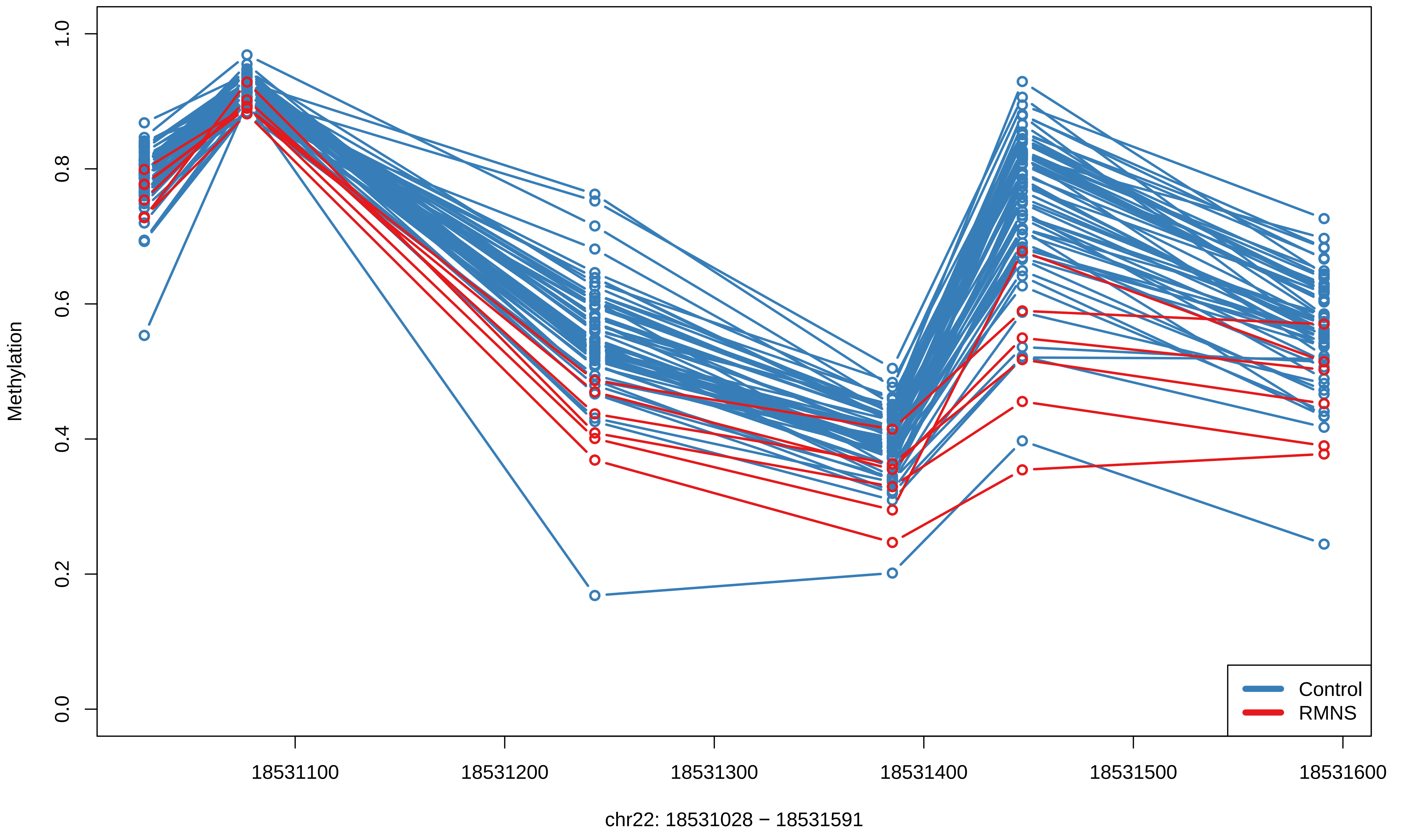

Methylation
0.0
0.2
0.4
0.6
0.8
1.0
18531100
18531200
18531300
18531400
18531500
18531600
chr22: 18531028 − 18531591
Control
RMNS

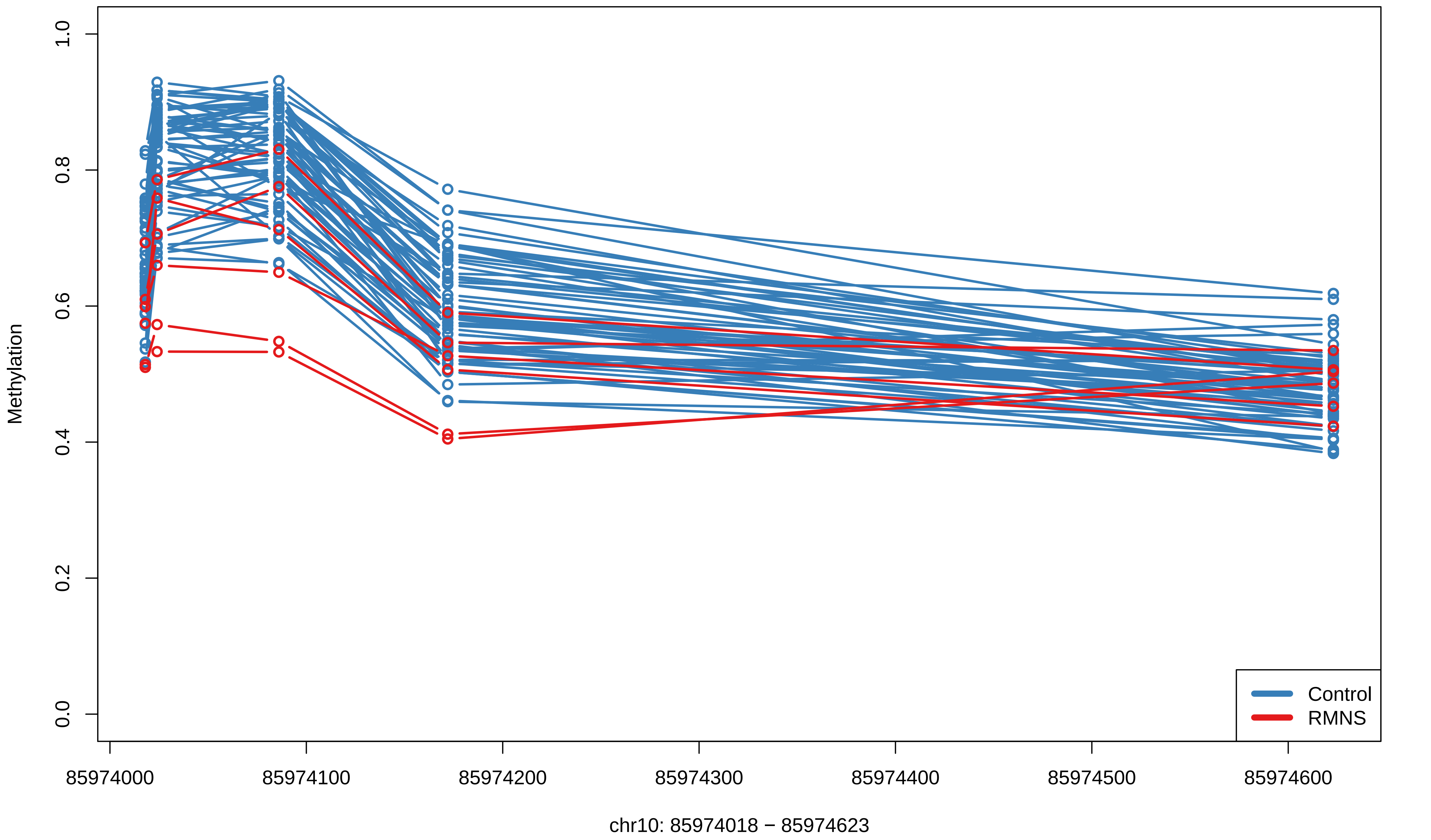

Methylation
1.0
0.8
0.6
0.4
0.2
0.0
85974000
85974100
85974200
85974300
85974400
85974500
85974600
chr10: 85974018 − 85974623
Control
RMNS

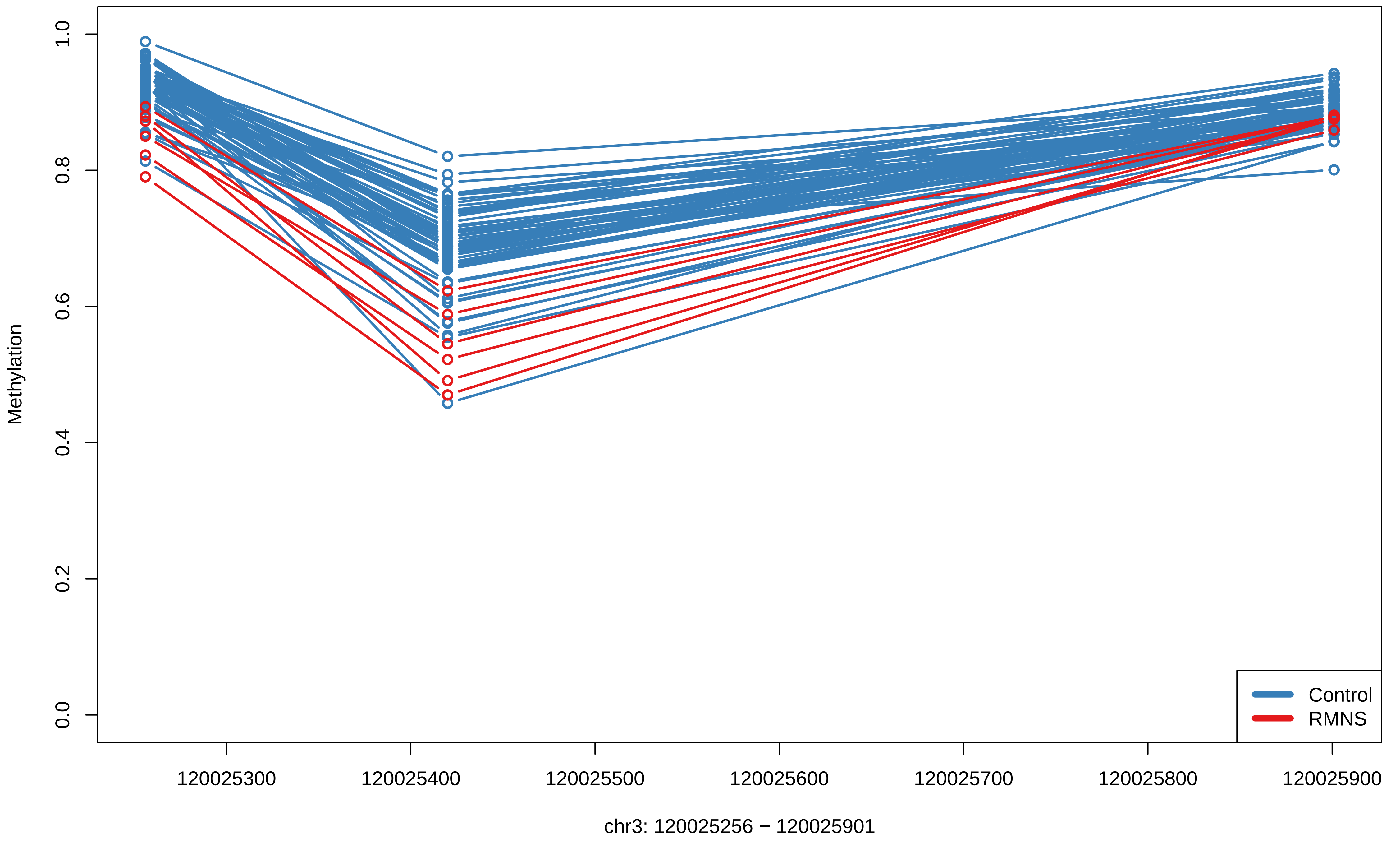
Methylation
1.0
0.8
0.6
0.4
0.2
0.0
120025300
120025400
120025500
120025600
120025700
120025800
120025900
chr3: 120025256 − 120025901
Control
RMNS

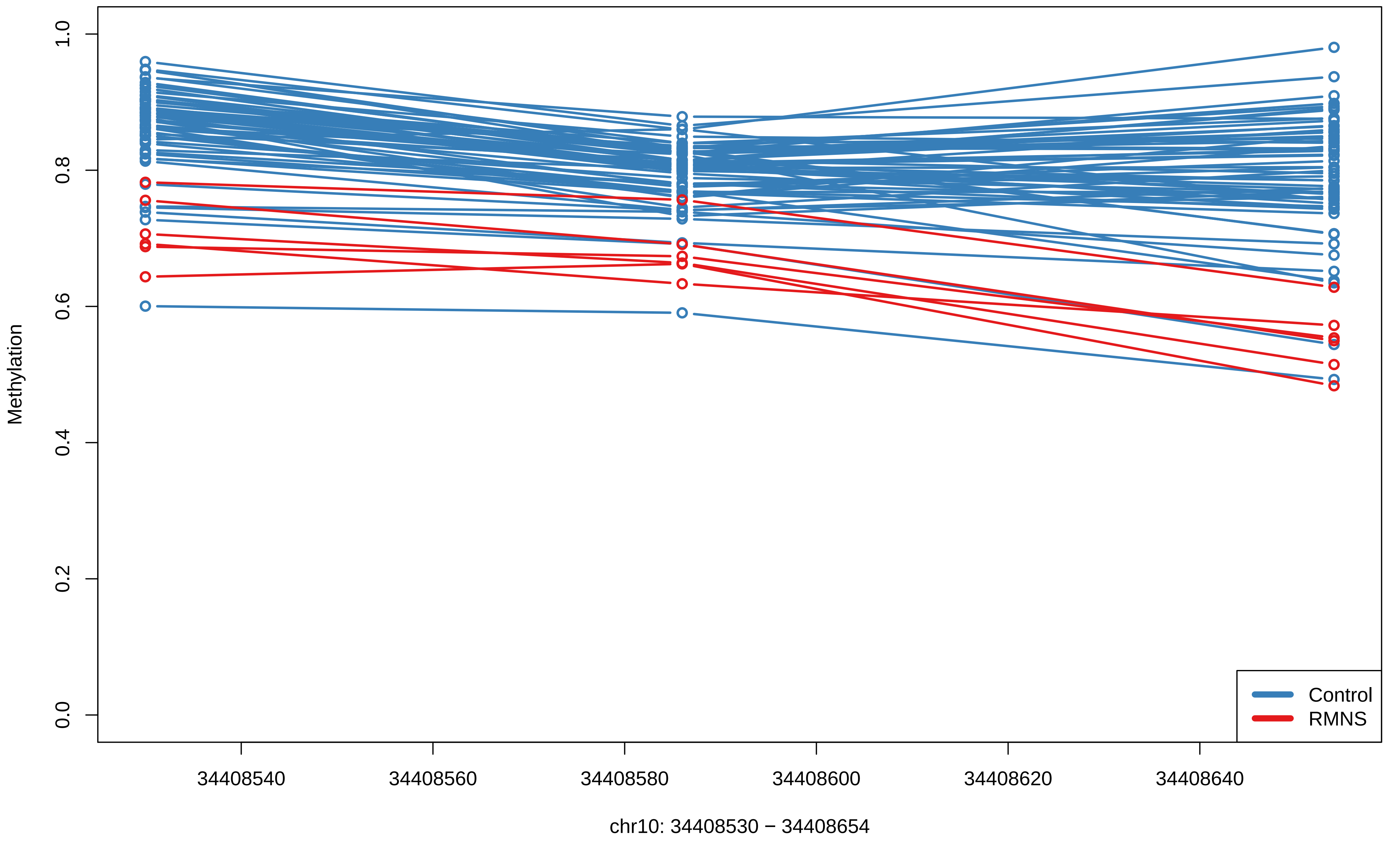
1.0
0.8
0.6
0.4
0.2
0.0
Methylation
34408540
34408560
34408580
34408600
34408620
34408640
chr10: 34408530 − 34408654
Control
RMNS

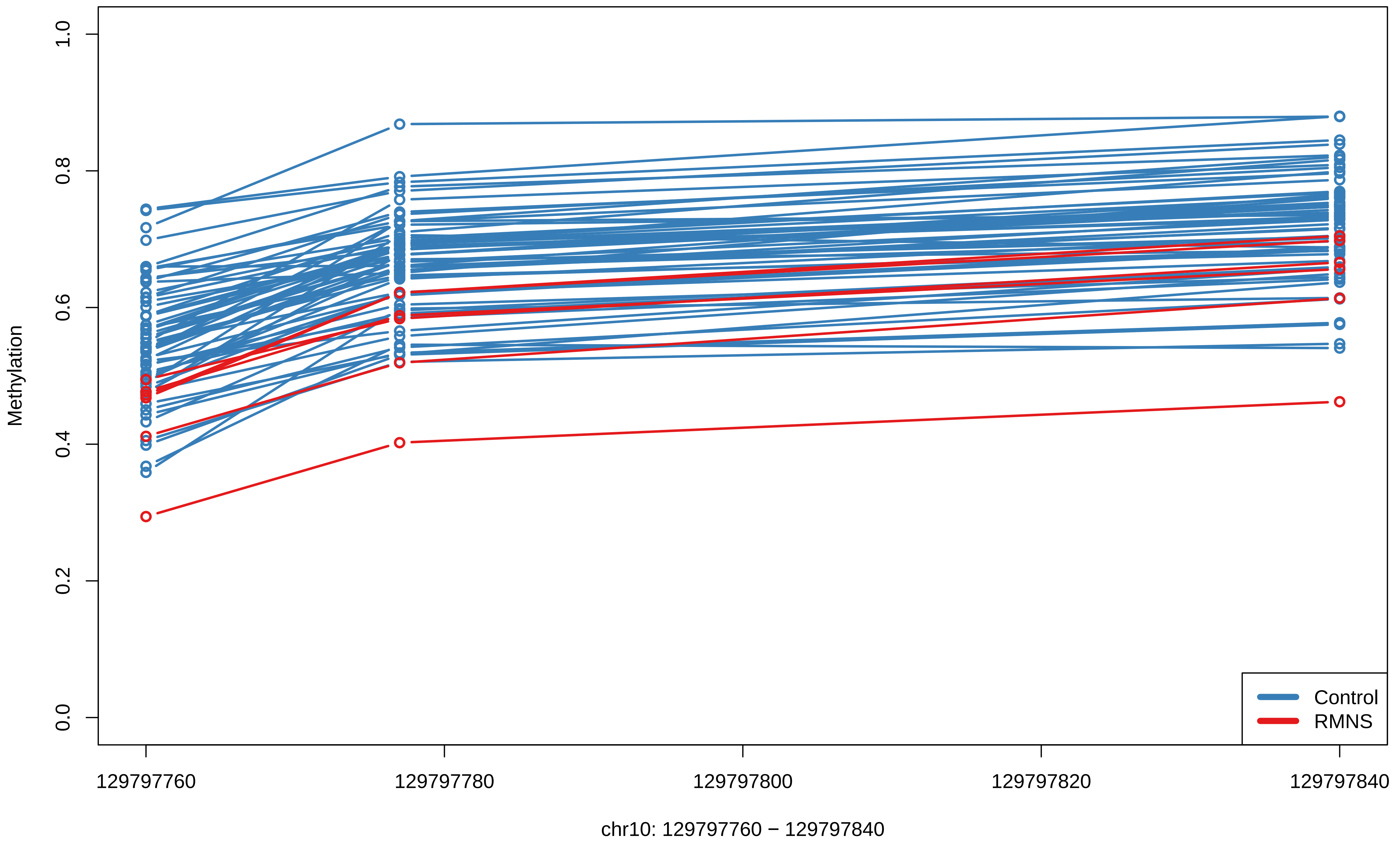

Methylation
0.0
0.2
0.4
0.6
0.8
1.0
129797760
129797780
129797800
129797820
129797840
chr10: 129797760 − 129797840
Control
RMNS

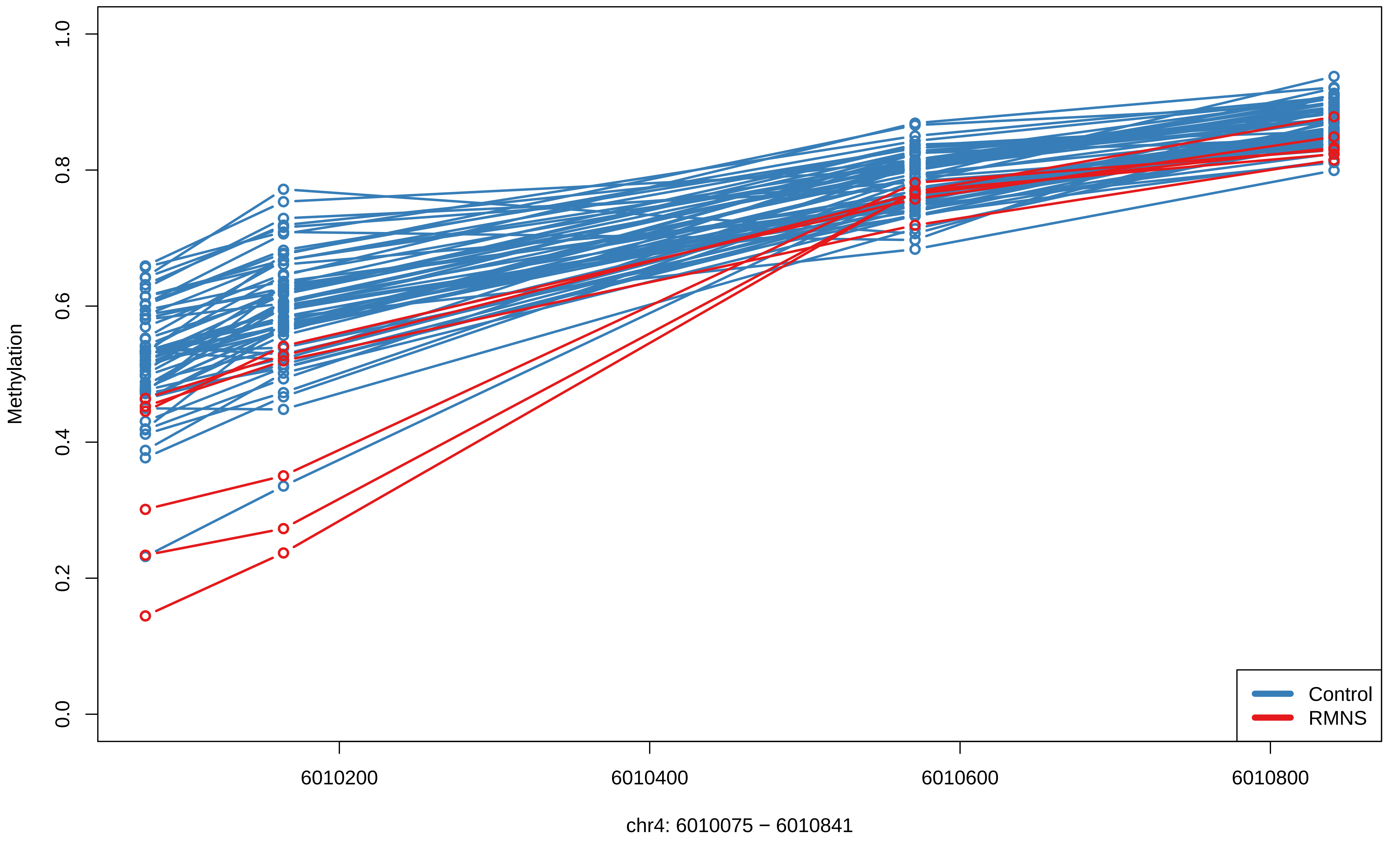

Methylation
0.0
0.2
0.4
0.6
0.8
1.0
6010200
6010400
6010600
6010800
chr4: 6010075 − 6010841
Control
RMNS

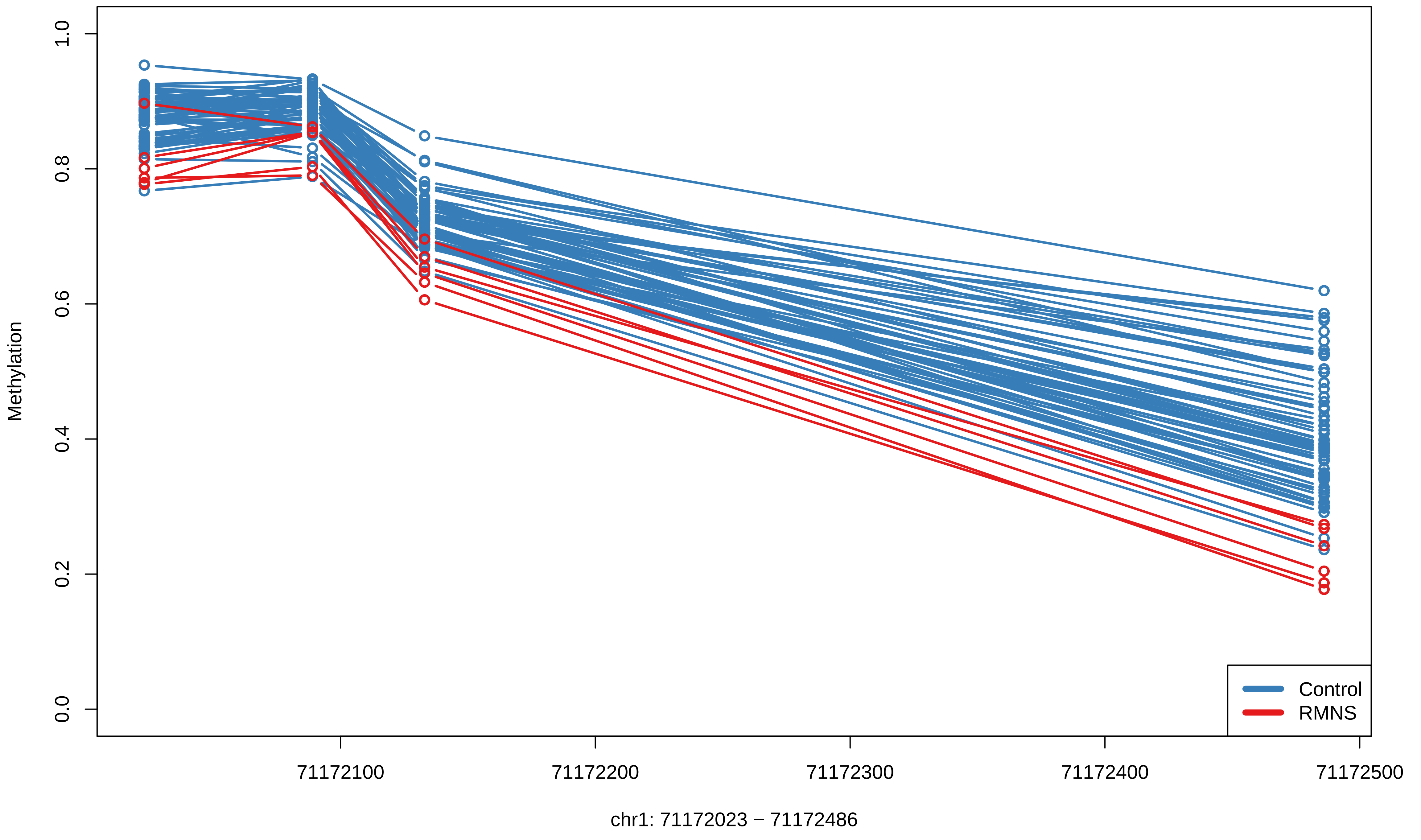

Methylation
0.0
0.2
0.4
0.6
0.8
1.0
71172100
71172200
71172300
71172400
71172500
chr1: 71172023 − 71172486
Control
RMNS

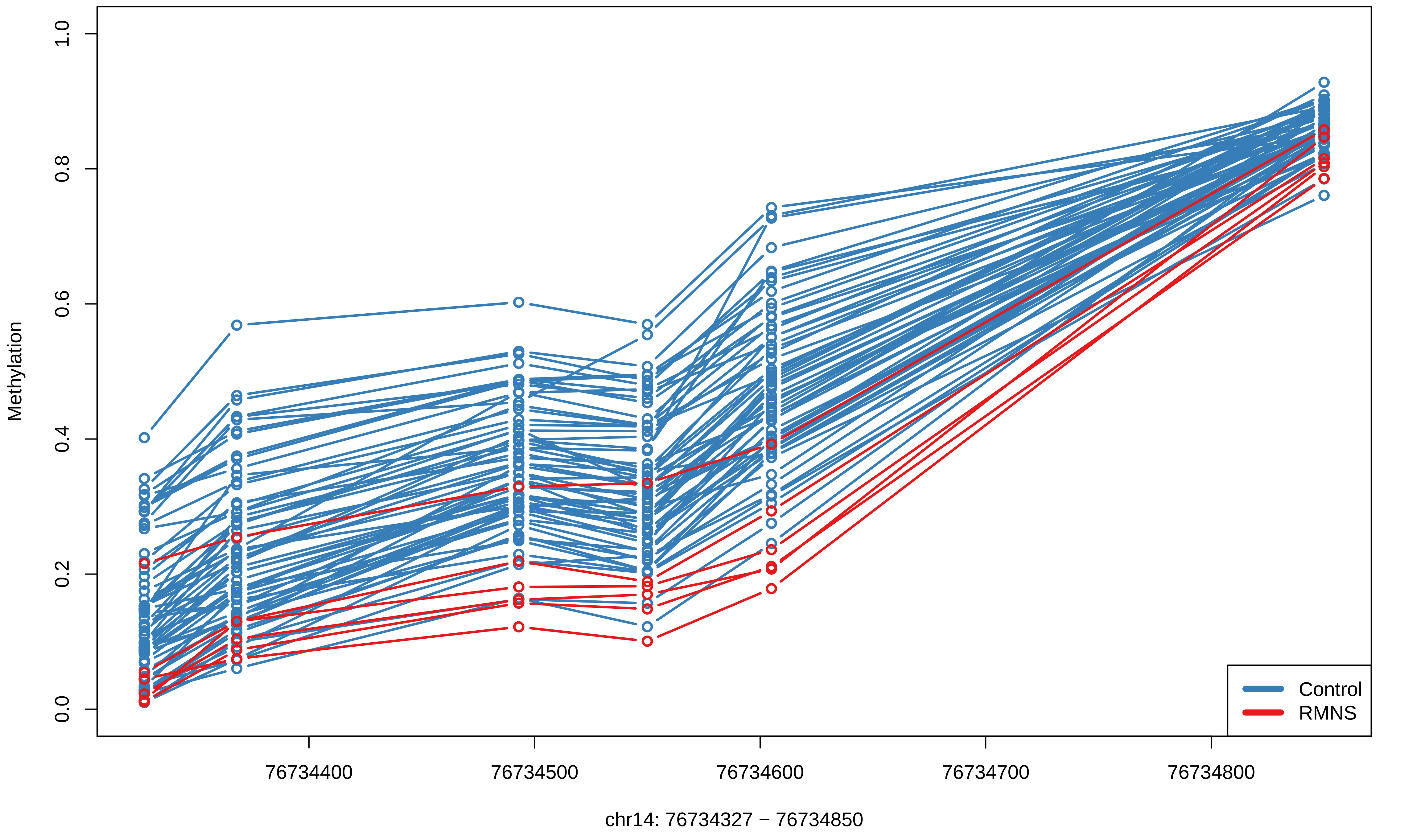

Methylation
0.0
0.2
0.4
0.6
0.8
1.0
76734400
76734500
76734600
76734700
76734800
chr14: 76734327 − 76734850
Control
RMNS

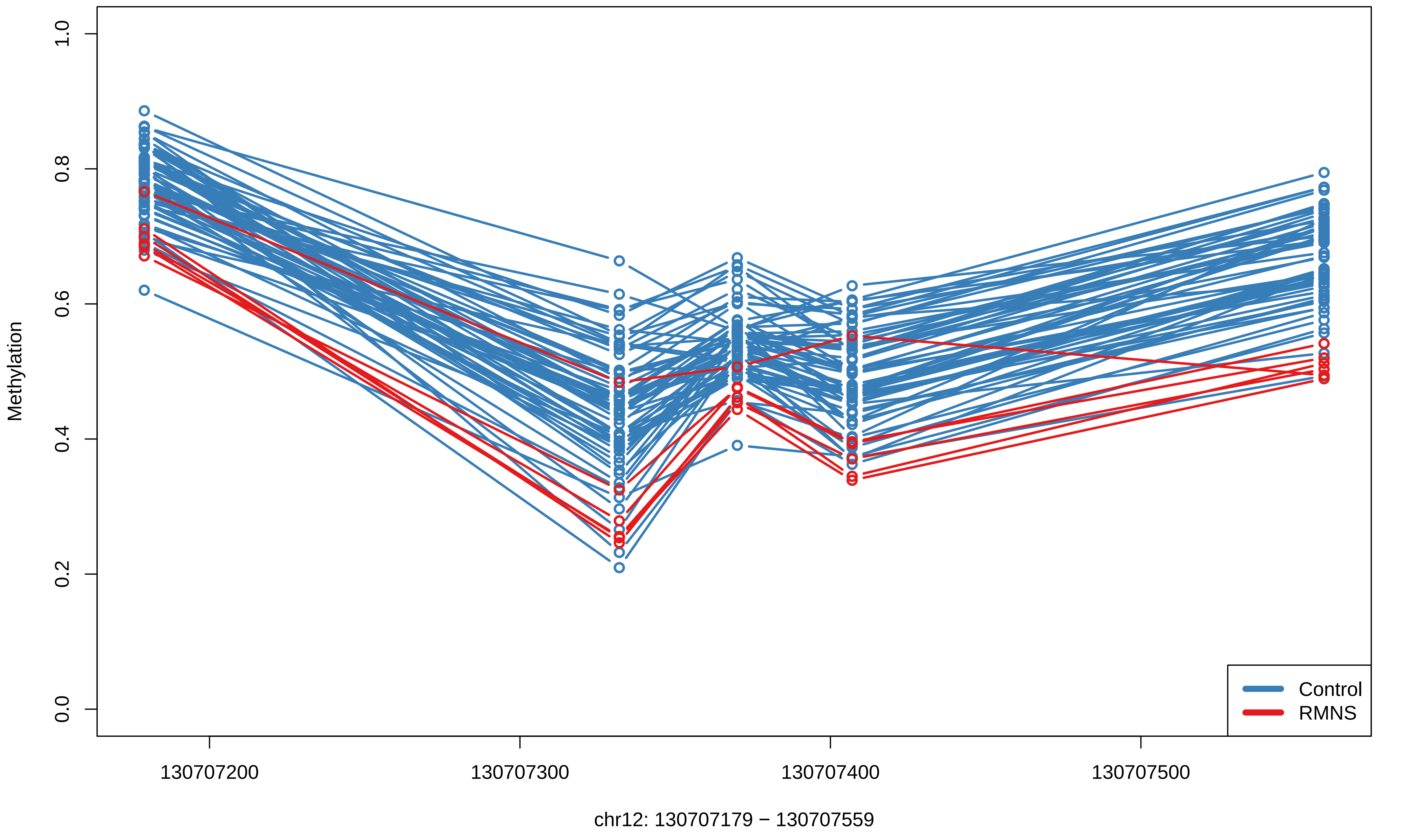

Methylation
1.0
0.8
0.6
0.4
0.2
0.0
130707200
130707300
130707400
130707500
chr12: 130707179 − 130707559
Control
RMNS

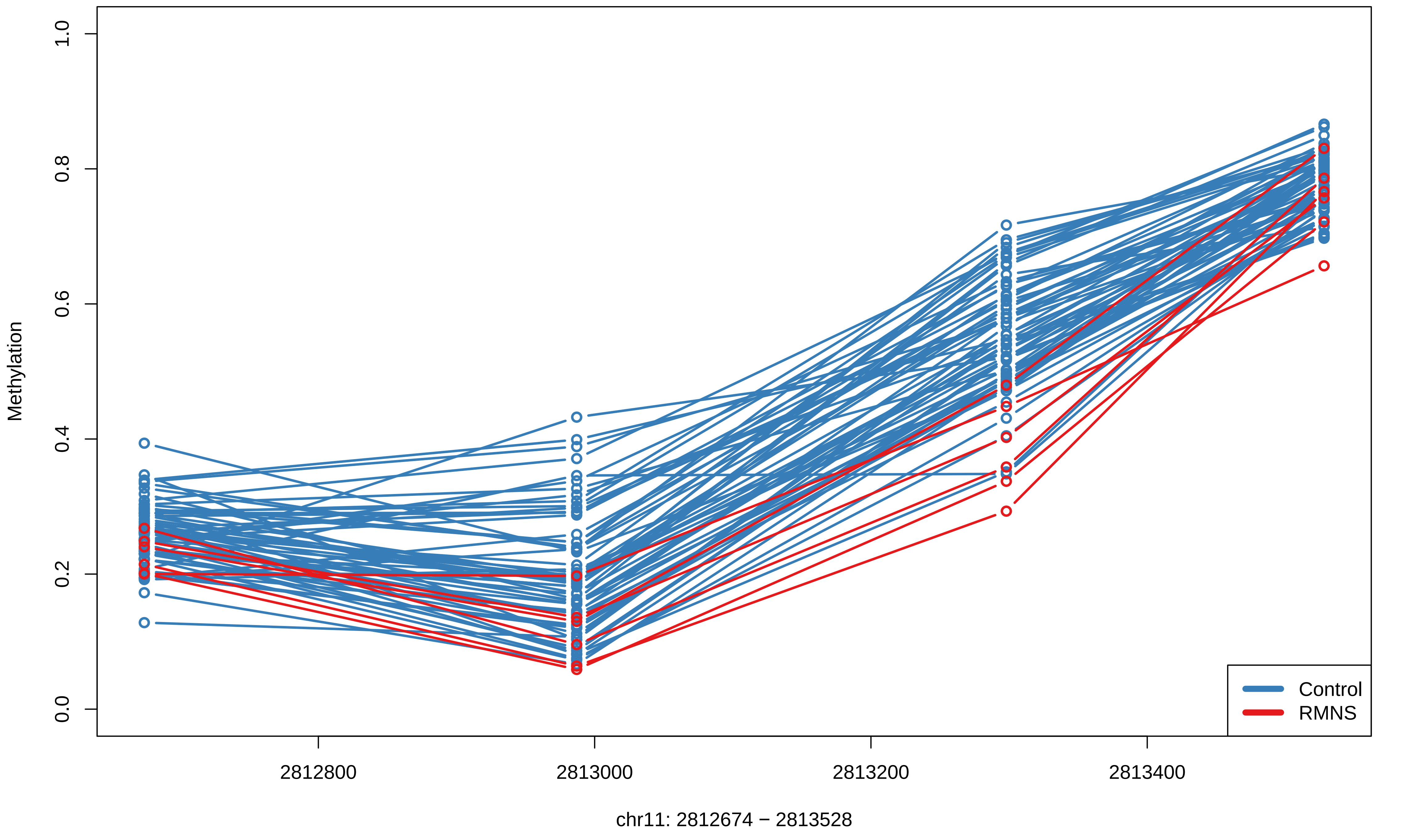

1.0
0.8
0.6
0.4
0.2
0.0
Methylation
2812800
2813000
2813200
2813400
chr11: 2812674 − 2813528
Control
RMNS

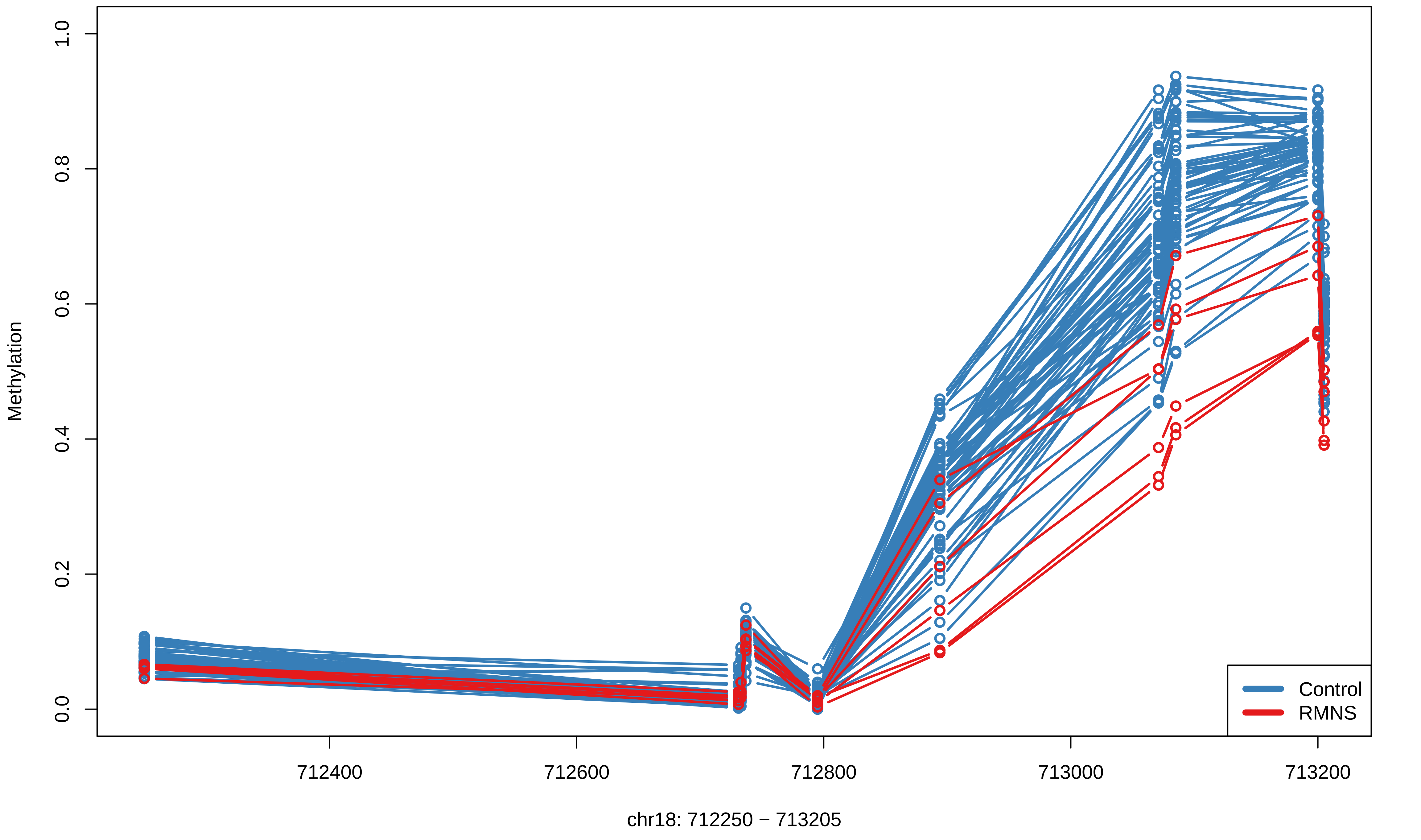

Methylation
0.0
0.2
0.4
0.6
0.8
1.0
712400
712600
712800
713000
713200
chr18: 712250 − 713205
Control
RMNS

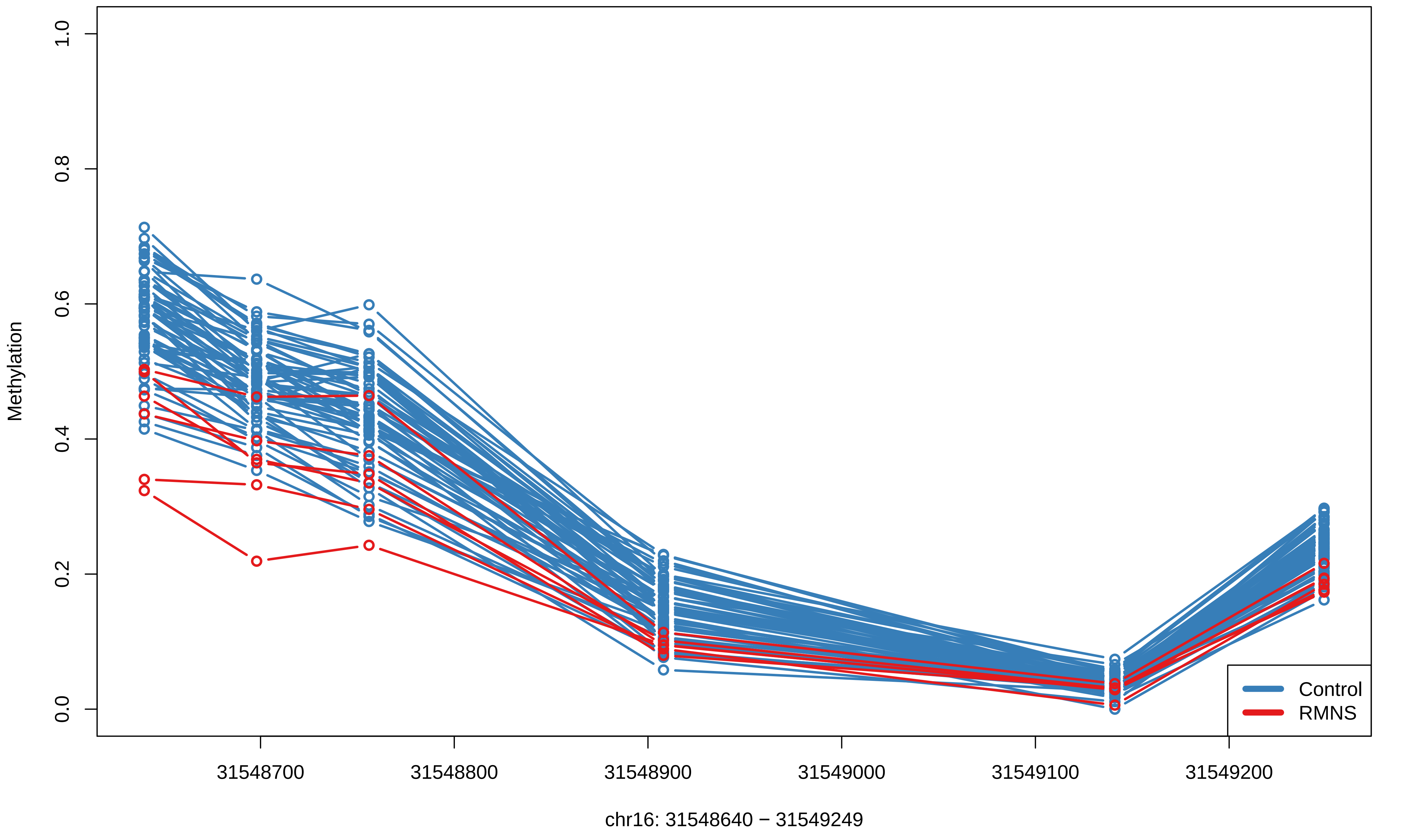

Methylation
0.0
0.2
0.4
0.6
0.8
1.0
31548700
31548800
31548900
31549000
31549100
31549200
chr16: 31548640 − 31549249
Control
RMNS

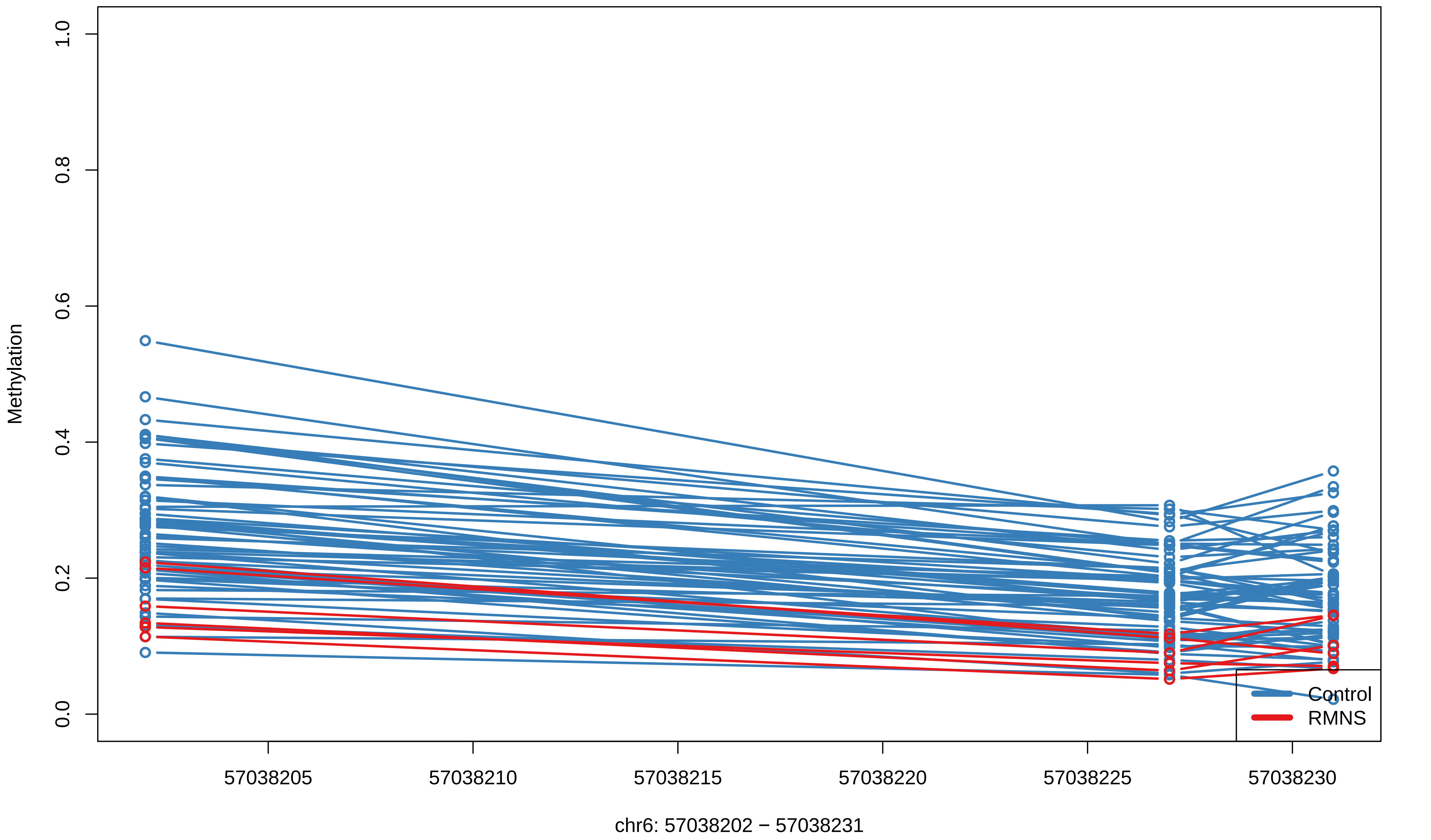

Methylation
0.0
0.2
0.4
0.6
0.8
1.0
57038205
57038210
57038215
57038220
57038225
57038230
chr6: 57038202 − 57038231
Control
RMNS

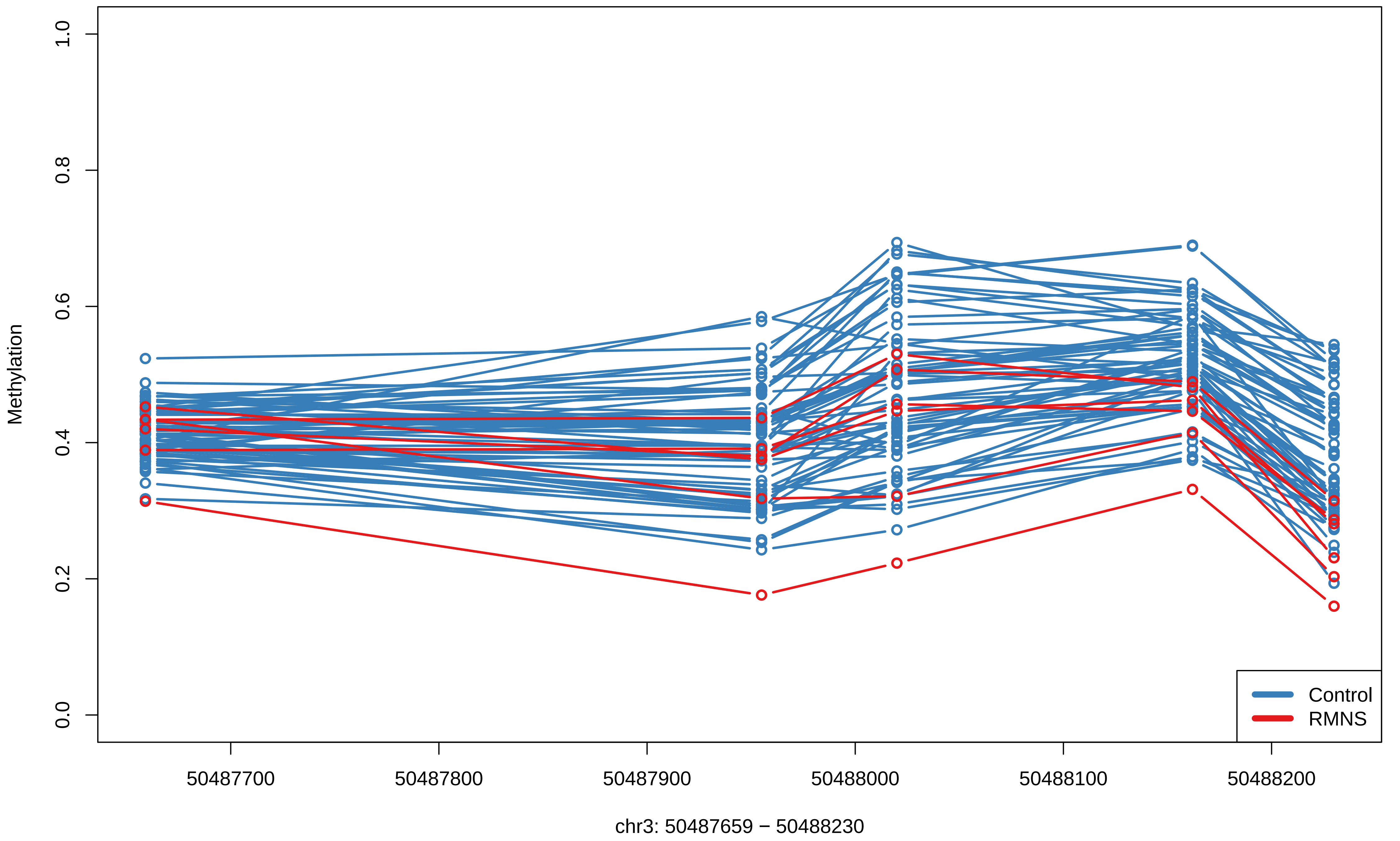

Methylation
1.0
0.8
0.6
0.4
0.2
0.0
50487700
50487800
50487900
50488000
50488100
50488200
chr3: 50487659 − 50488230
Control
RMNS

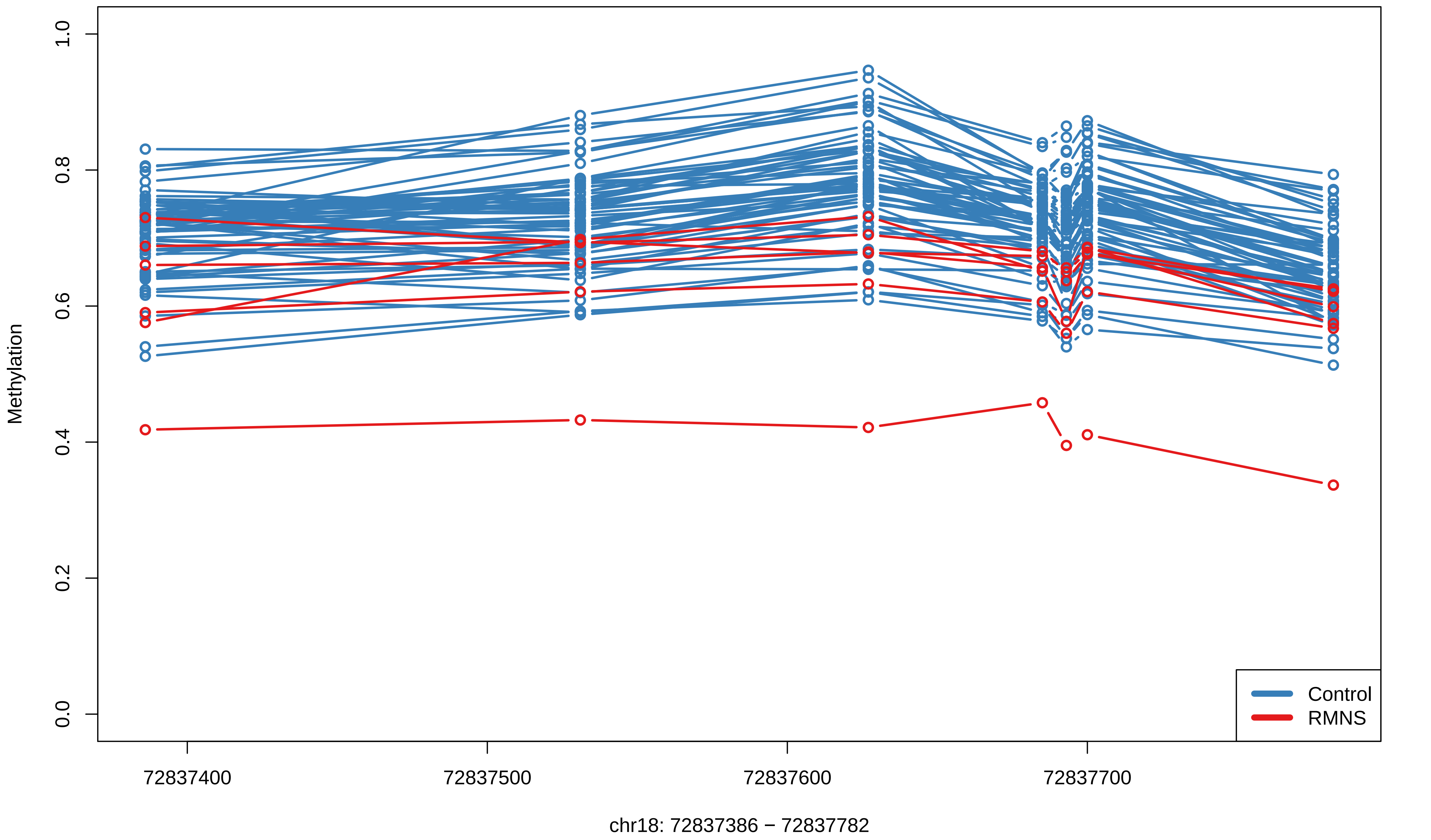

Methylation
0.0
0.2
0.4
0.6
0.8
1.0
72837400
72837500
72837600
72837700
chr18: 72837386 − 72837782
Control
RMNS

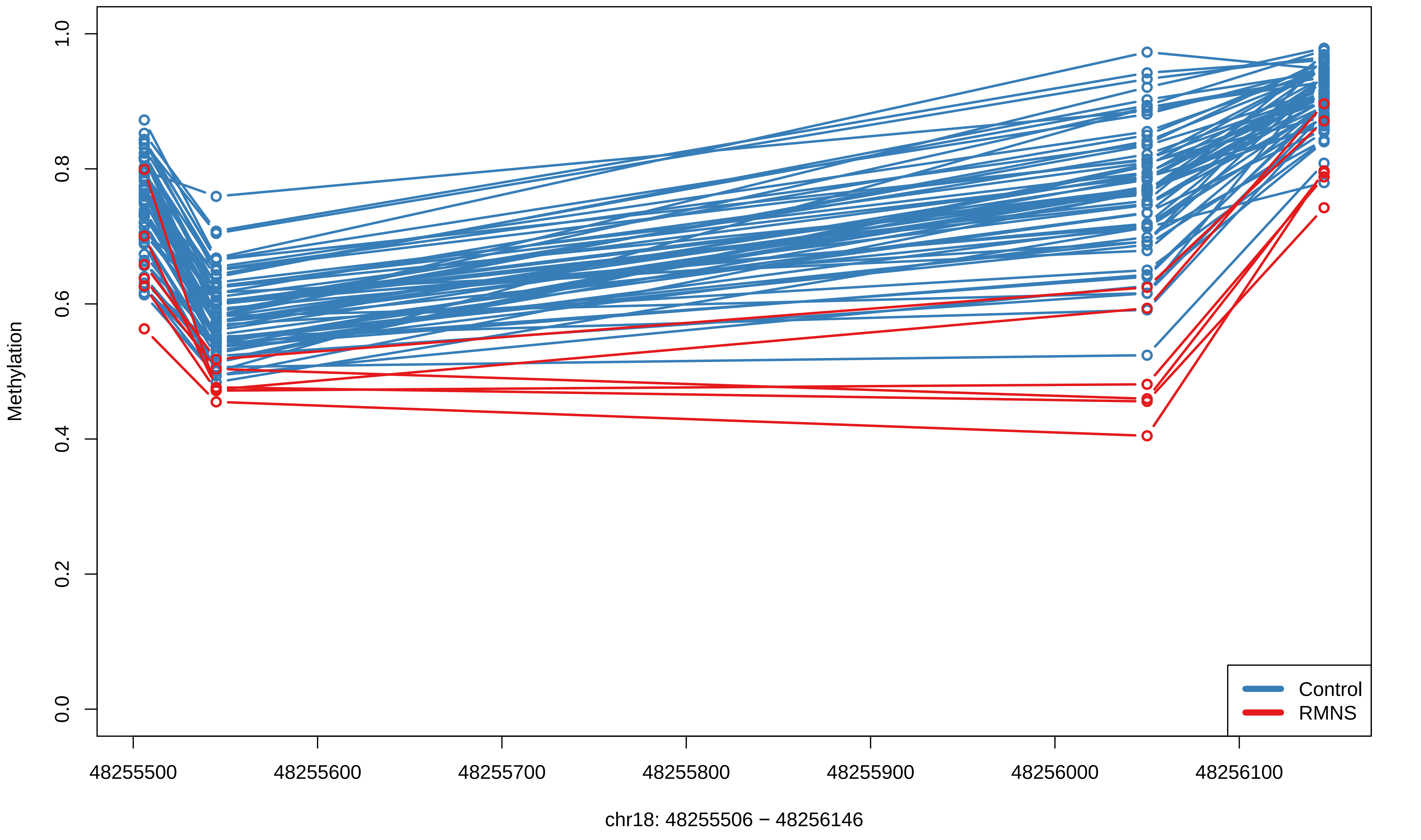
Methylation
0.0
0.2
0.4
0.6
0.8
1.0
48255500
48255600
48255700
48255800
48255900
48256000
48256100
chr18: 48255506 − 48256146
Control
RMNS

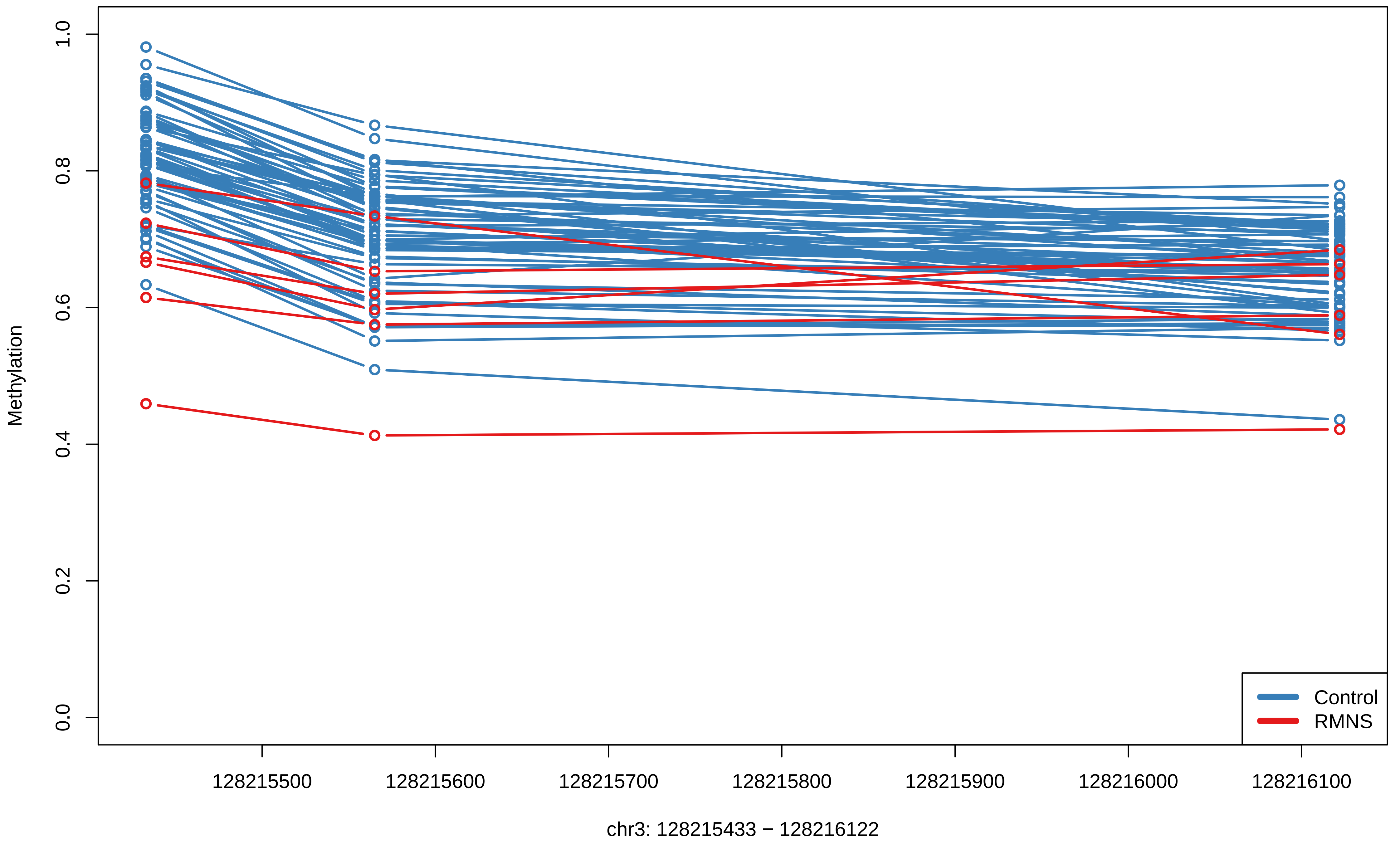

Methylation
1.0
0.8
0.6
0.4
0.2
0.0
128215500
128215600
128215700
128215800
128215900
128216000
128216100
chr3: 128215433 − 128216122
Control
RMNS

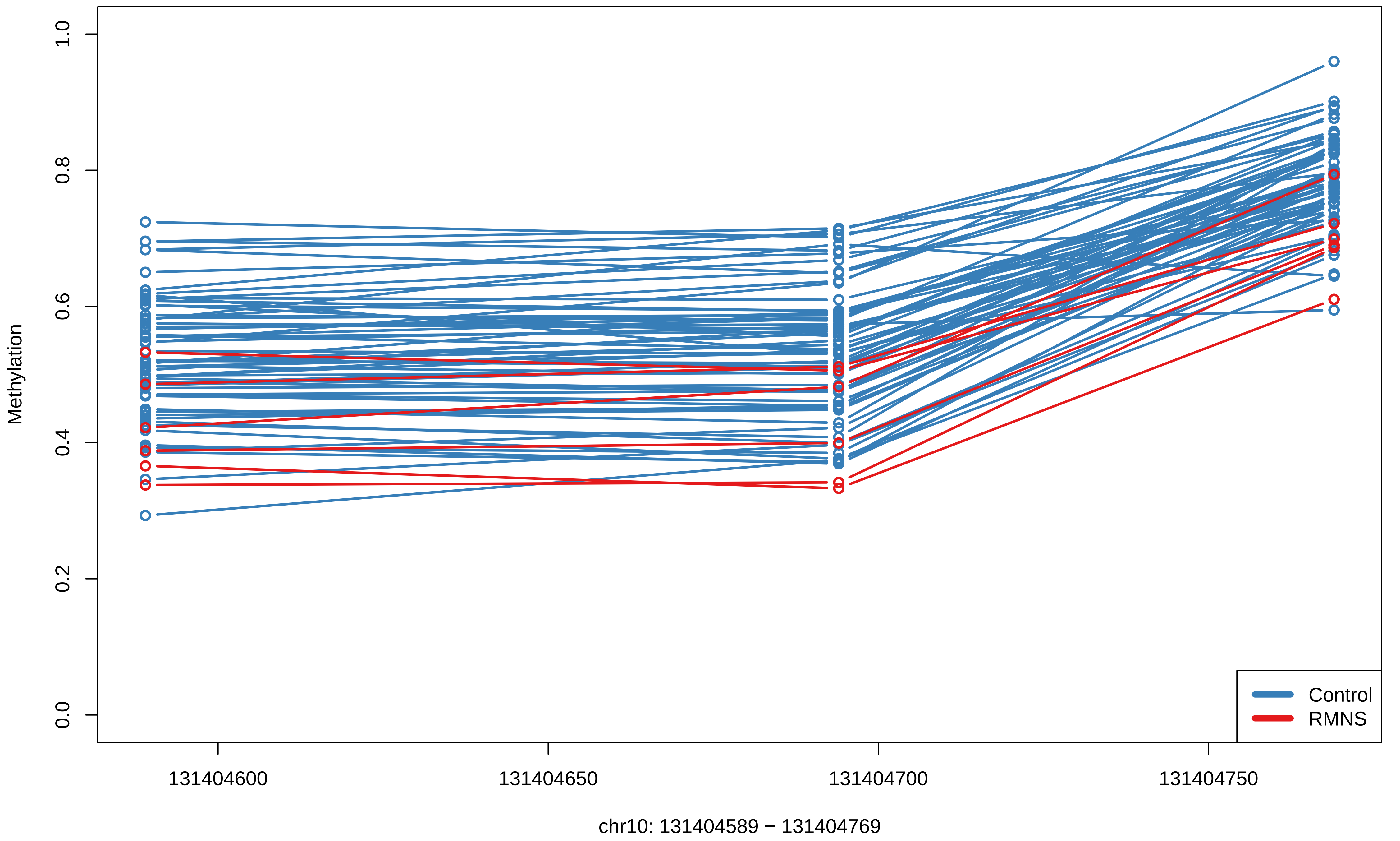

Methylation
1.0
0.8
0.6
0.4
0.2
0.0
131404600
131404650
131404700
131404750
chr10: 131404589 − 131404769
Control
RMNS

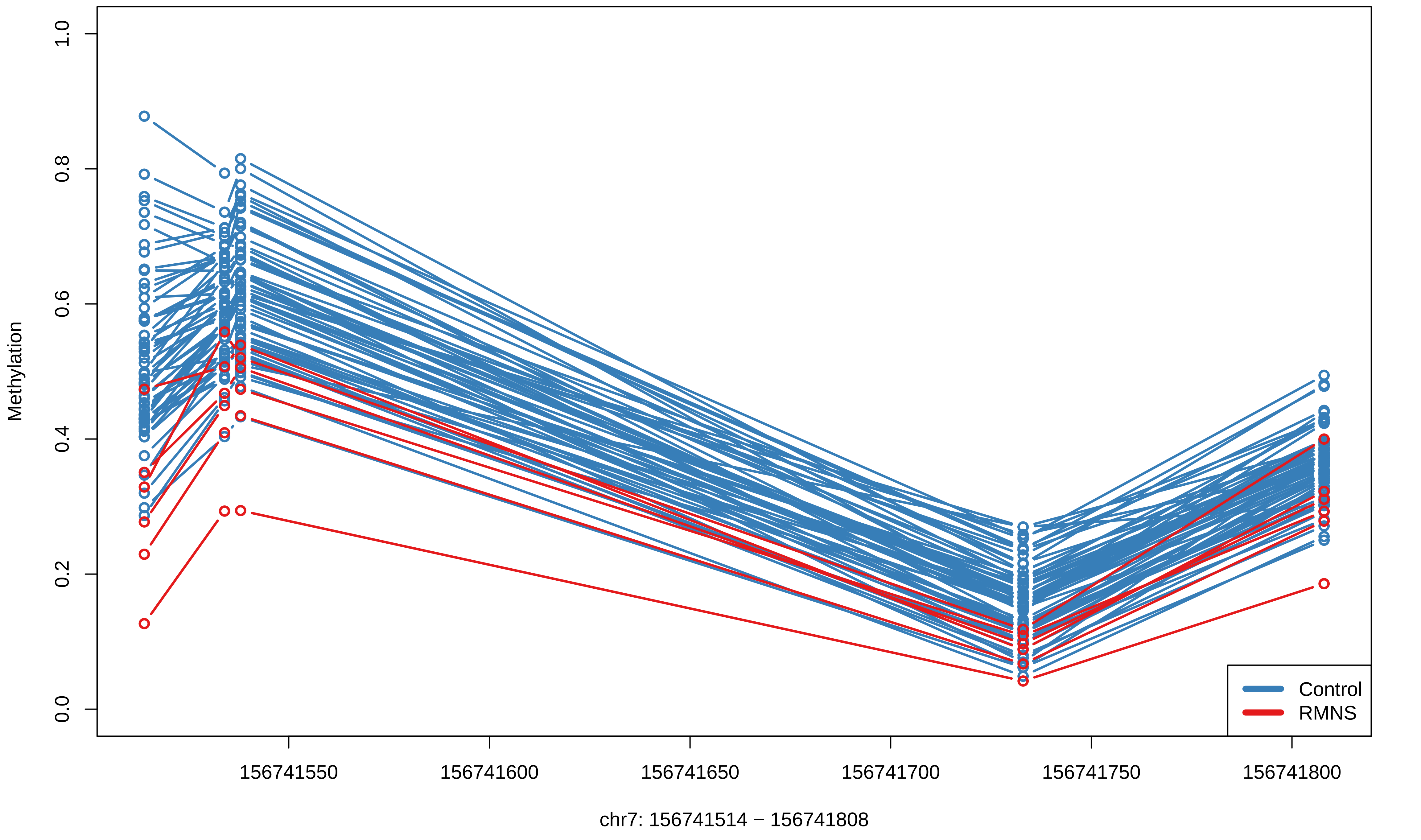

Methylation
0.0
0.2
0.4
0.6
0.8
1.0
156741550
156741600
156741650
156741700
156741750
156741800
chr7: 156741514 − 156741808
Control
RMNS

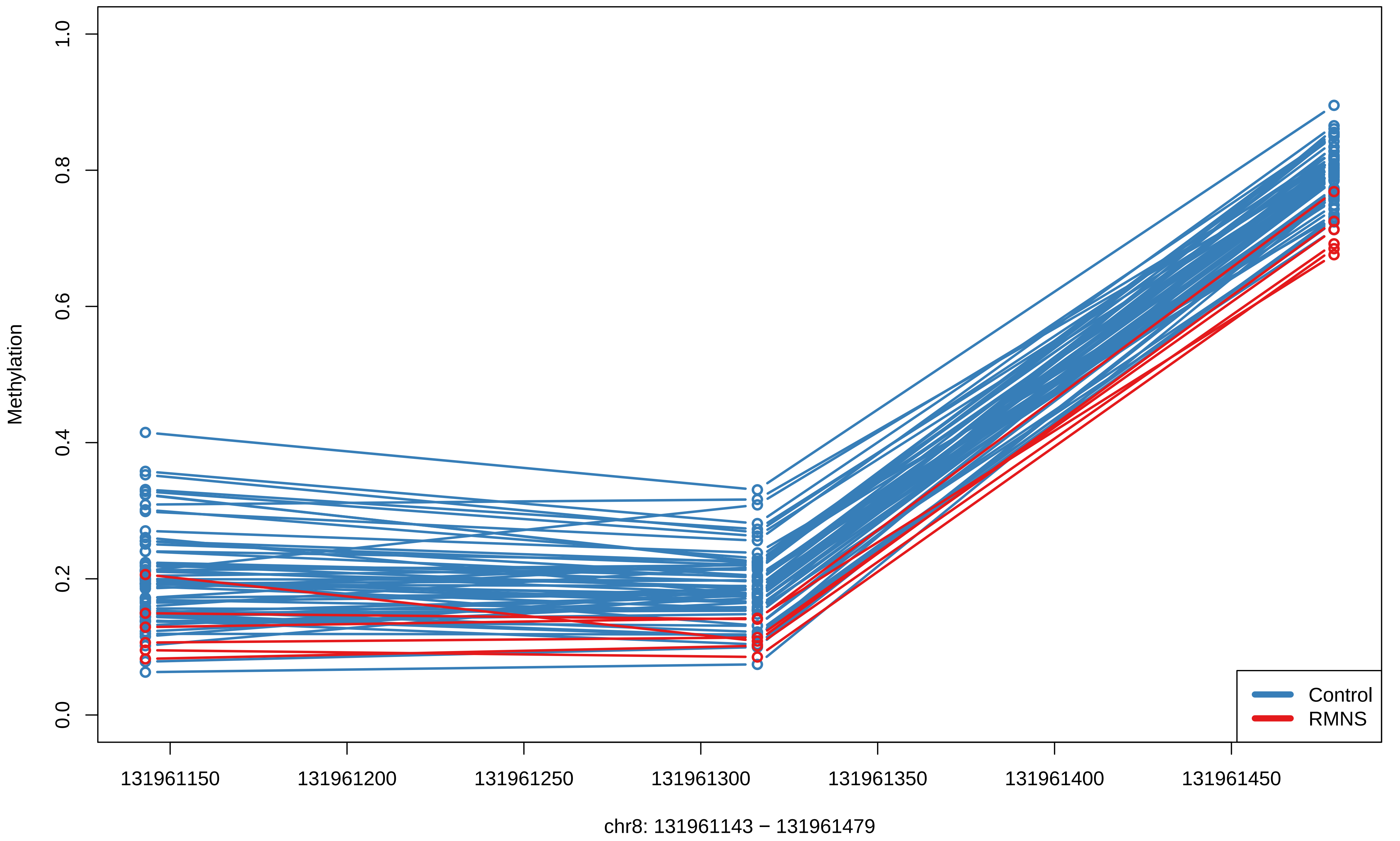

Methylation
0.0
0.2
0.4
0.6
0.8
1.0
131961150
131961200
131961250
131961300
131961350
131961400
131961450
chr8: 131961143 − 131961479
Control
RMNS

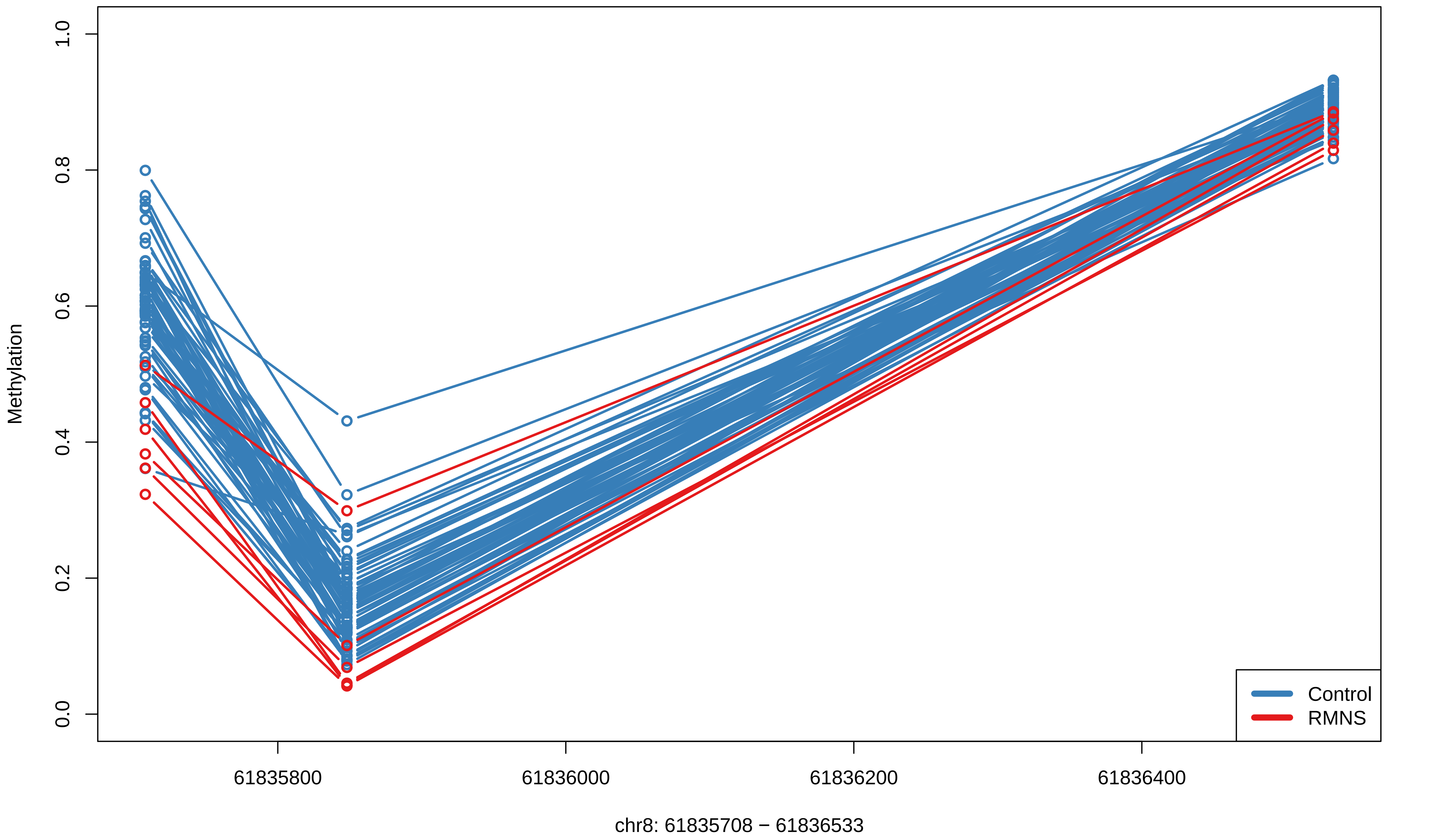

Methylation
0.0
0.2
0.4
0.6
0.8
1.0
61835800
61836000
61836200
61836400
chr8: 61835708 − 61836533
Control
RMNS

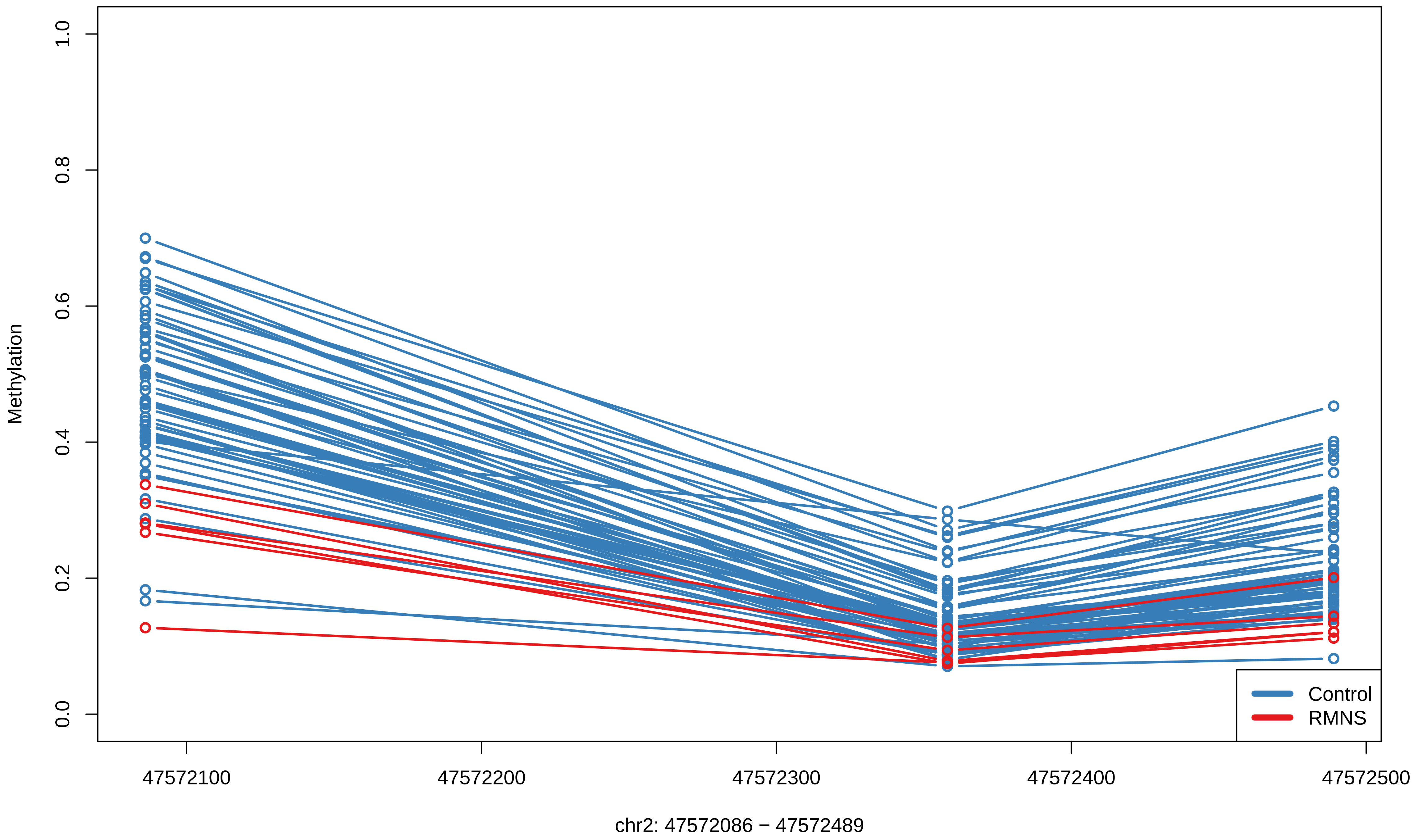

Methylation
1.0
0.8
0.6
0.4
0.2
0.0
47572100
47572200
47572300
47572400
47572500
chr2: 47572086 − 47572489
Control
RMNS

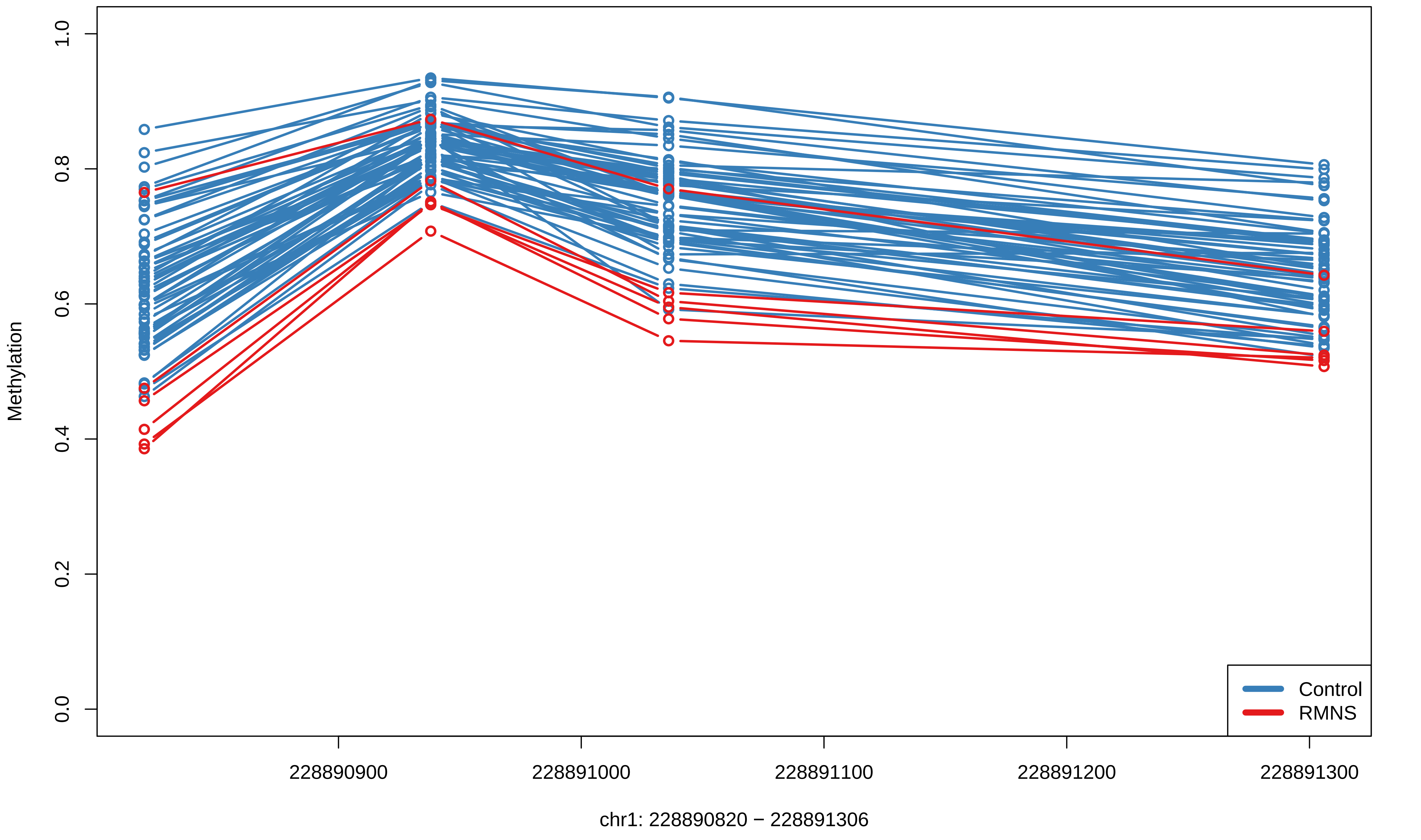

Methylation
0.0
0.2
0.4
0.6
0.8
1.0
228890900
228891000
228891100
228891200
228891300
chr1: 228890820 − 228891306
Control
RMNS

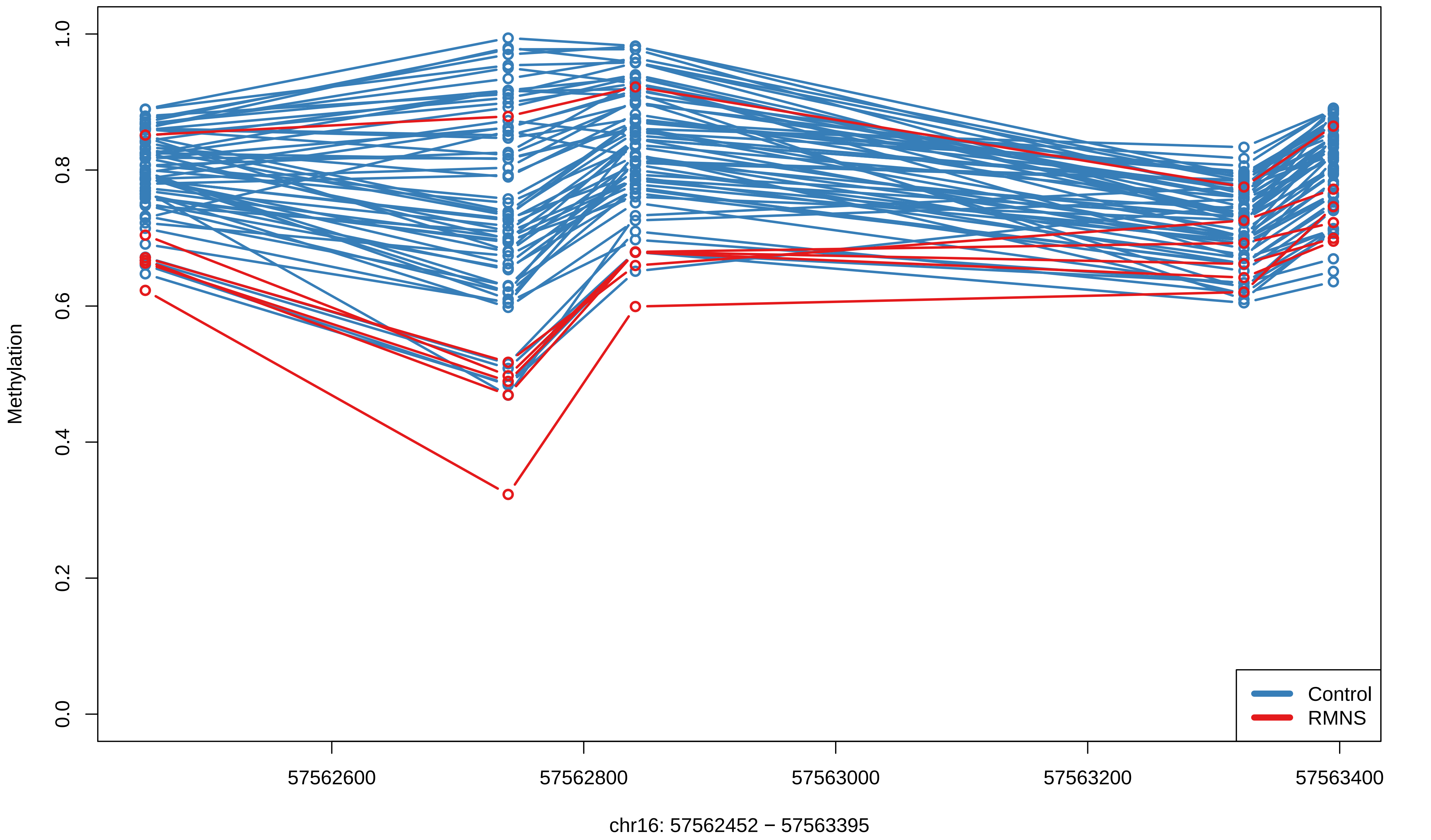

Methylation
1.0
0.8
0.6
0.4
0.2
0.0
57562600
57562800
57563000
57563200
57563400
chr16: 57562452 − 57563395
Control
RMNS

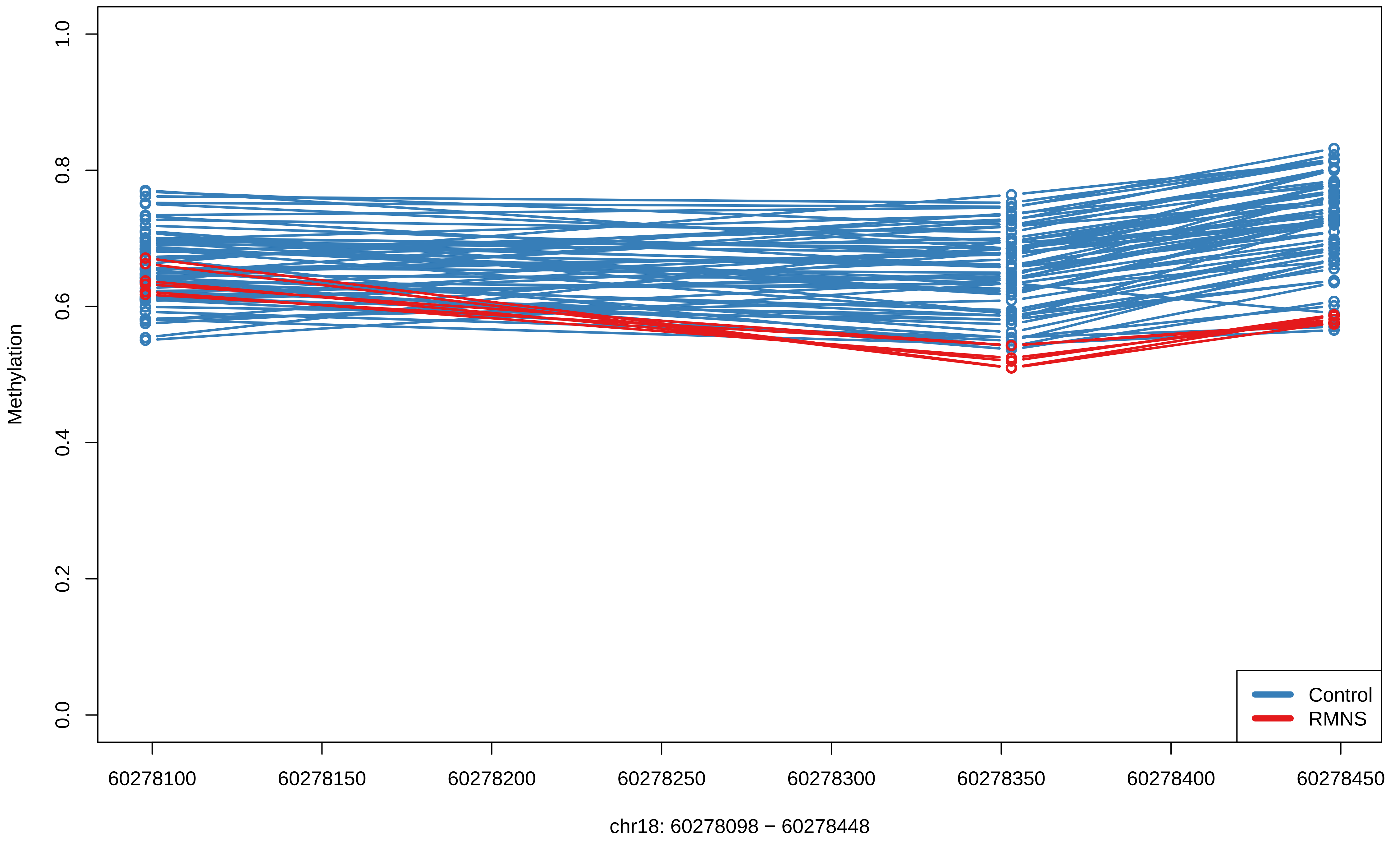

Methylation
0.0
0.2
0.4
0.6
0.8
1.0
60278100
60278150
60278200
60278250
60278300
60278350
60278400
60278450
chr18: 60278098 − 60278448
Control
RMNS

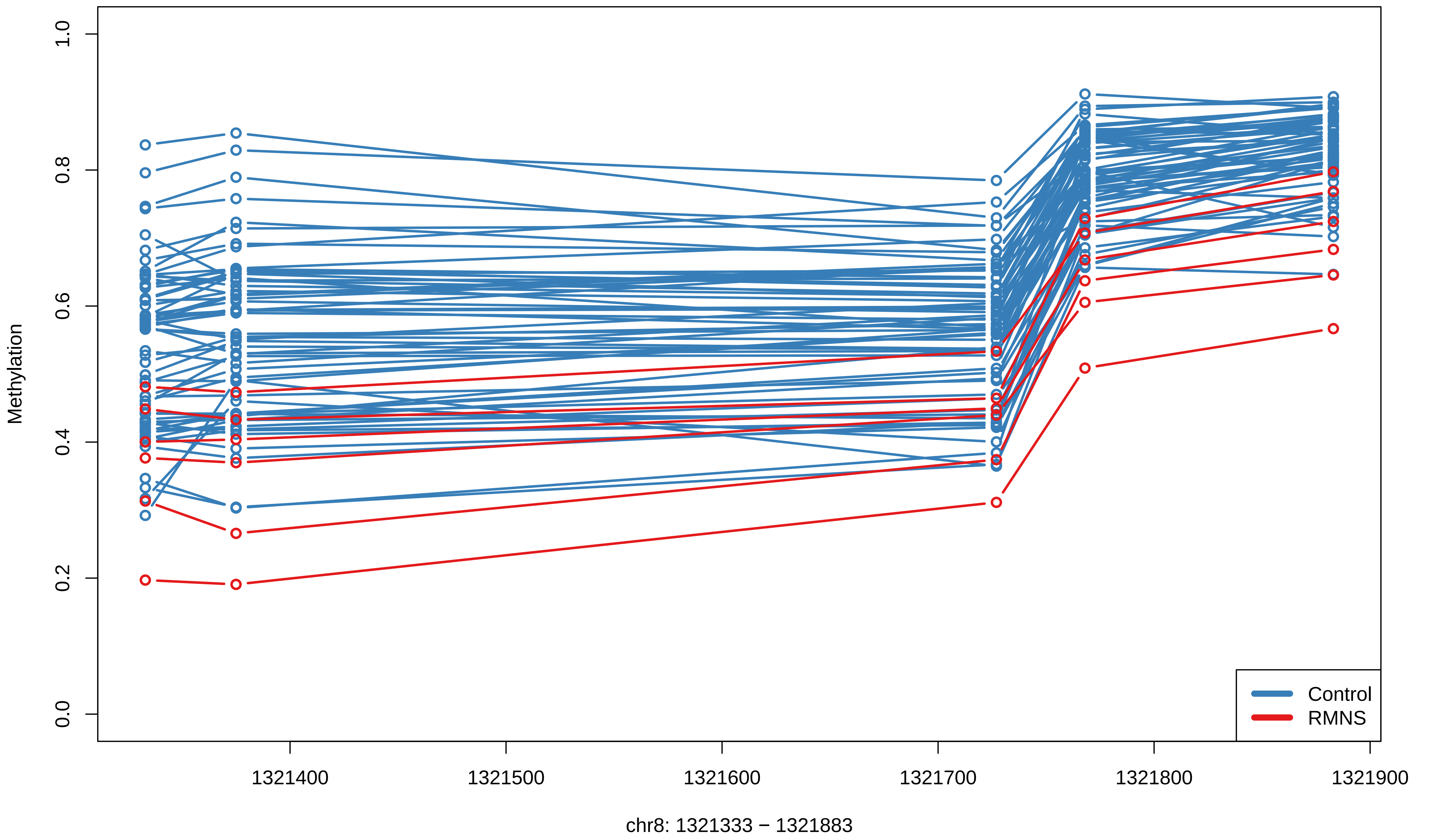
Methylation
0.0
0.2
0.4
0.6
0.8
1.0
1321400
1321500
1321600
1321700
1321800
1321900
chr8: 1321333 − 1321883
Control
RMNS

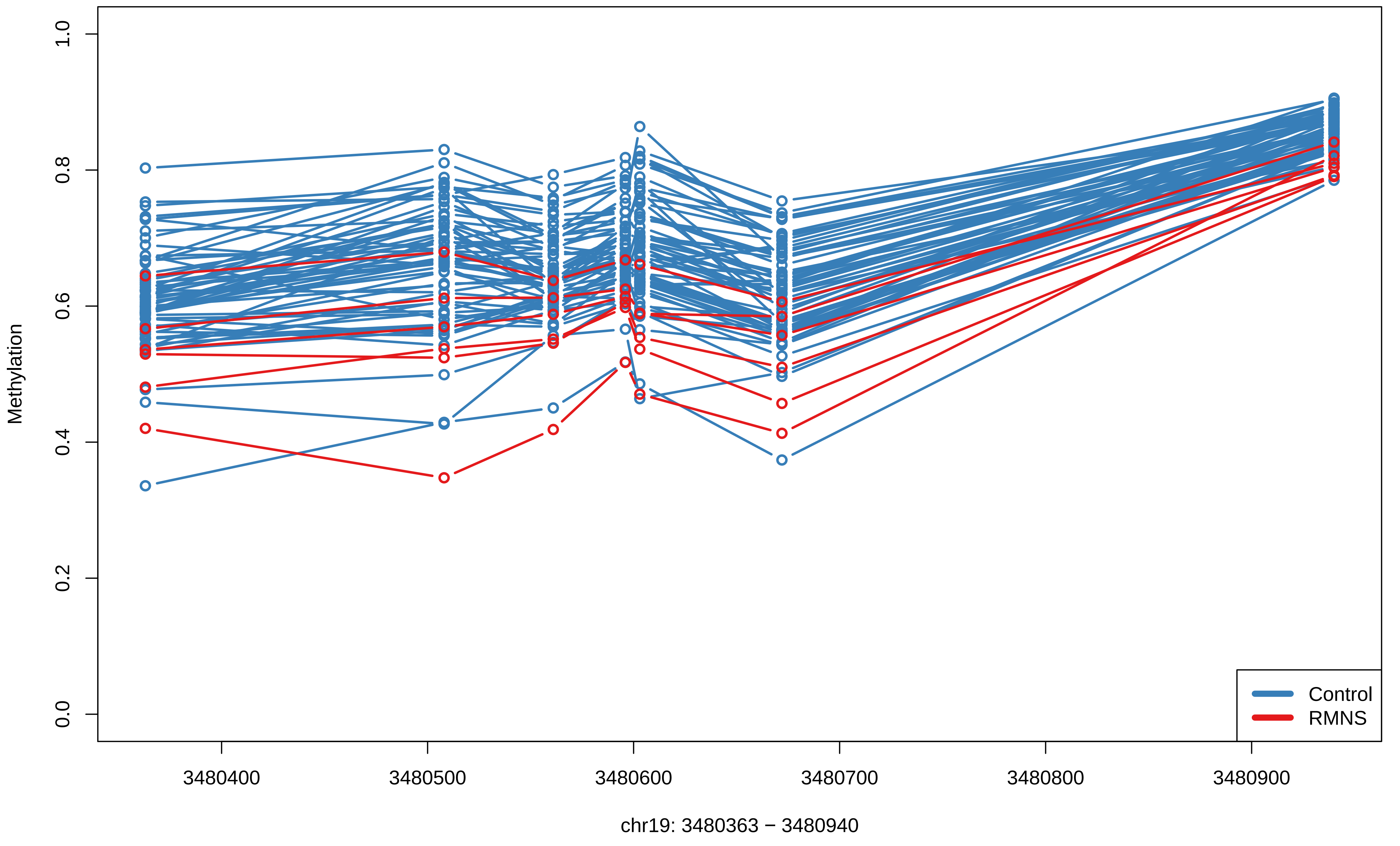

Methylation
1.0
0.8
0.6
0.4
0.2
0.0
3480400
3480500
3480600
3480700
3480800
3480900
chr19: 3480363 − 3480940
Control
RMNS

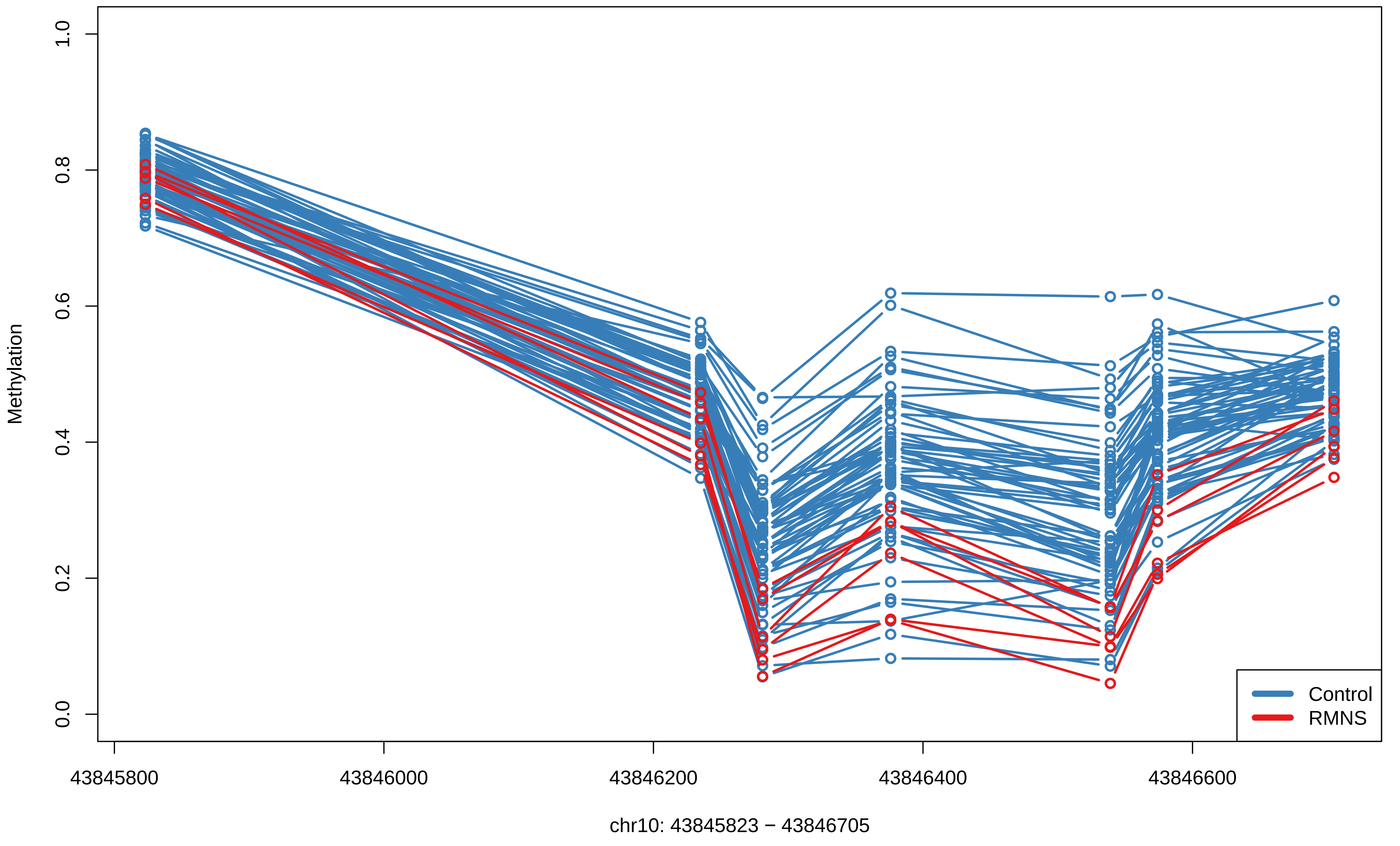

Methylation
0.0
0.2
0.4
0.6
0.8
1.0
43845800
43846000
43846200
43846400
43846600
chr10: 43845823 − 43846705
Control
RMNS

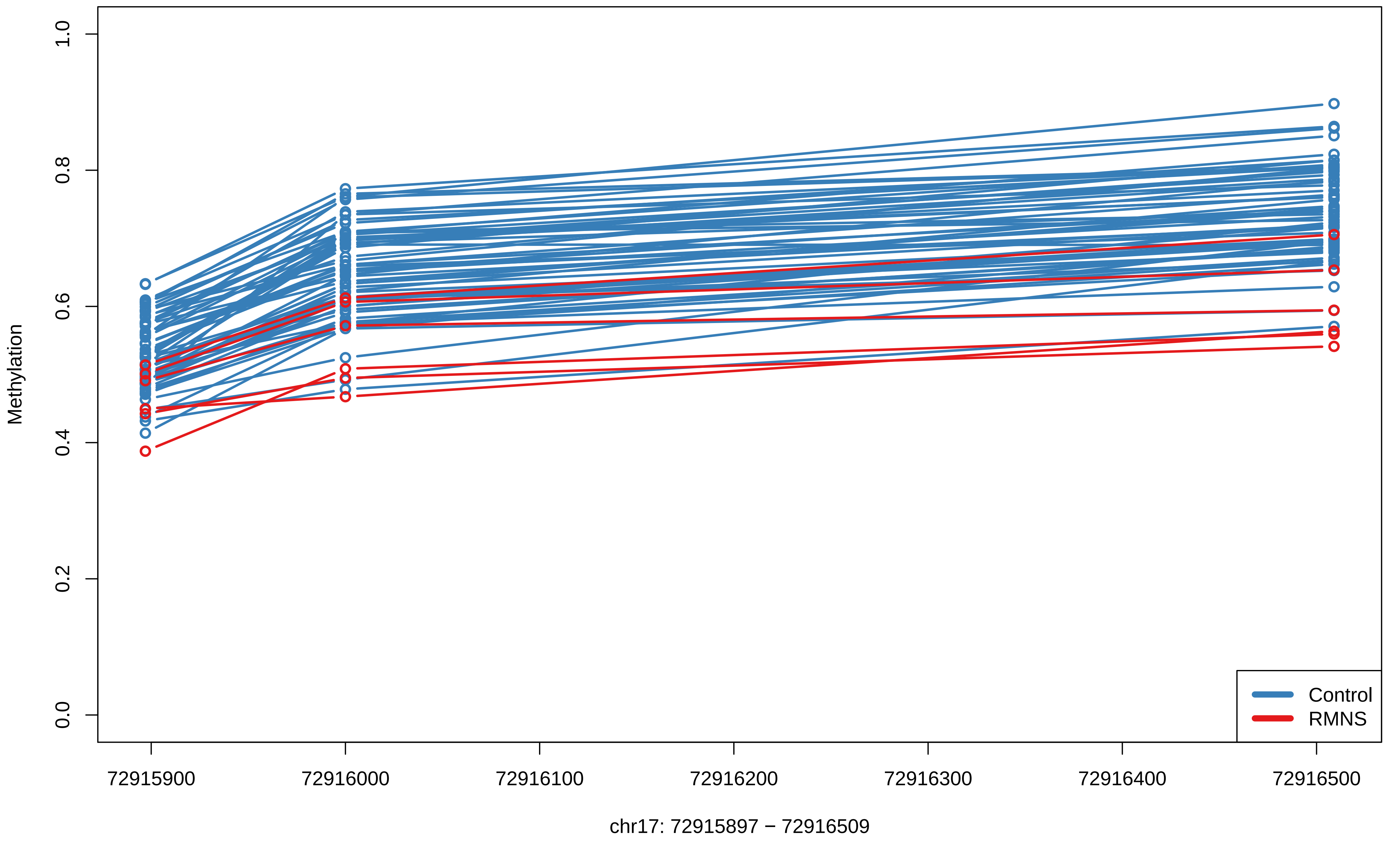

Methylation
1.0
0.8
0.6
0.4
0.2
0.0
72915900
72916000
72916100
72916200
72916300
72916400
72916500
chr17: 72915897 − 72916509
Control
RMNS

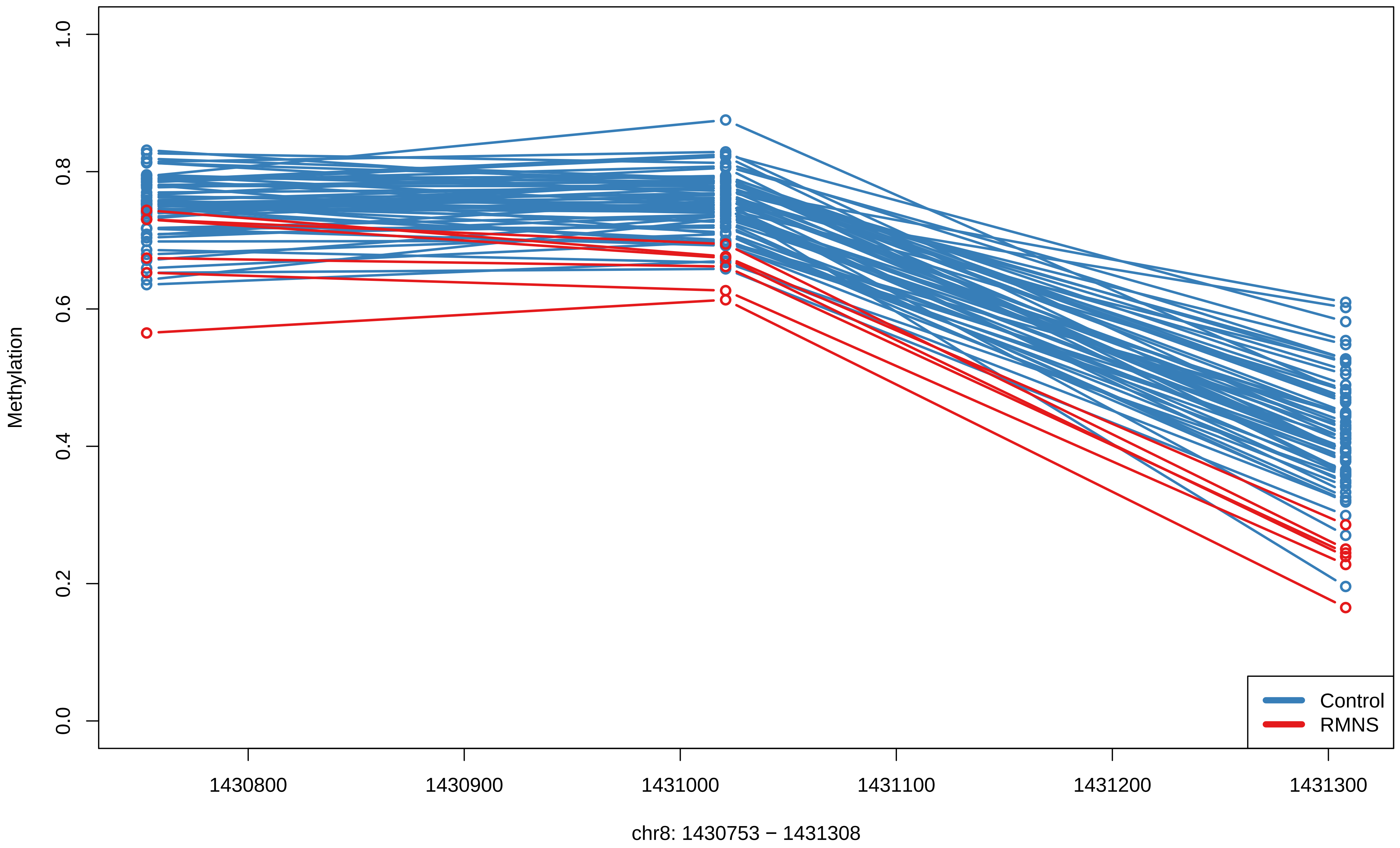

Methylation
0.0
0.2
0.4
0.6
0.8
1.0
1430800
1430900
1431000
1431100
1431200
1431300
chr8: 1430753 − 1431308
Control
RMNS

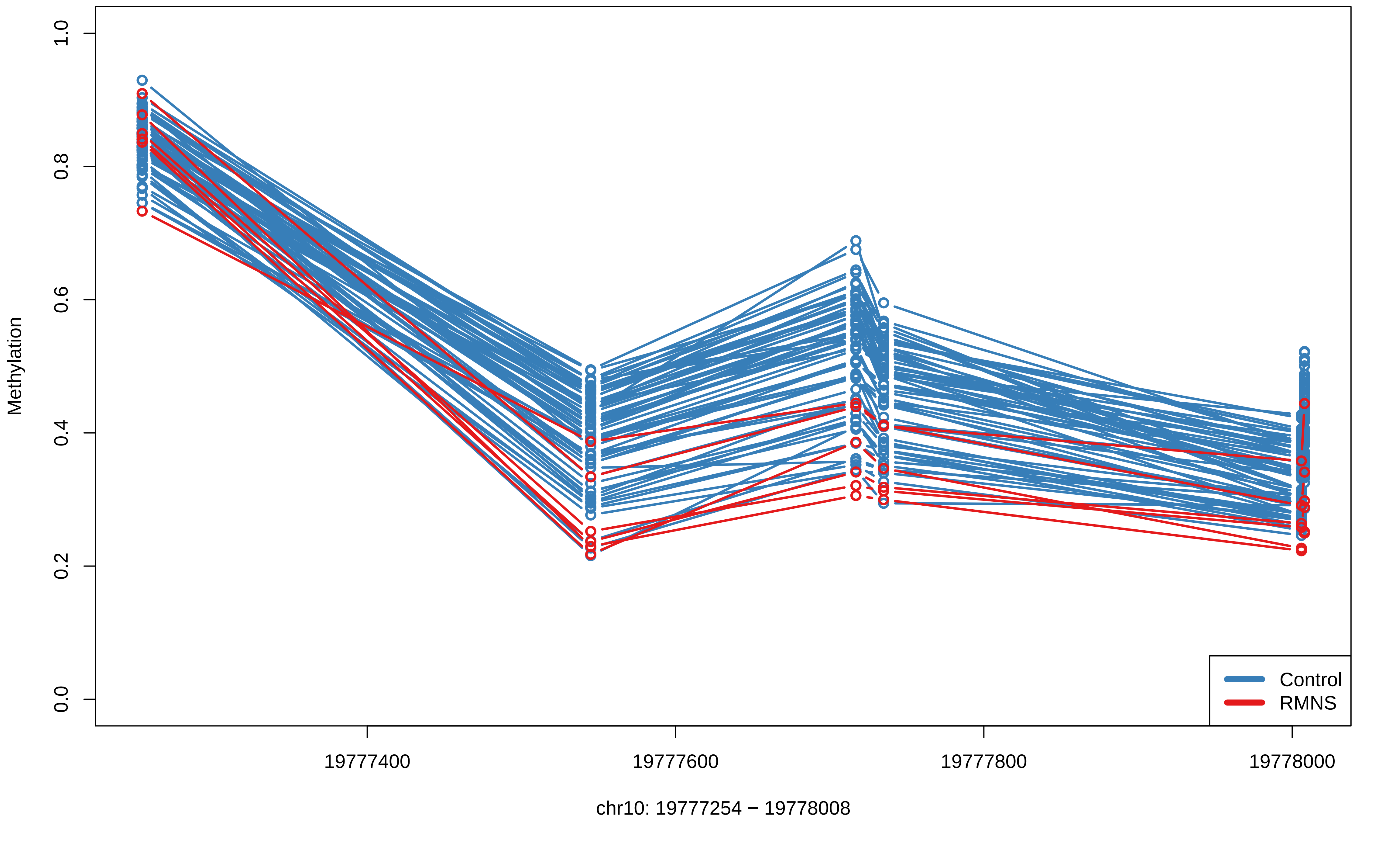

Methylation
0.0
0.2
0.4
0.6
0.8
1.0
19777400
19777600
19777800
19778000
chr10: 19777254 − 19778008
Control
RMNS

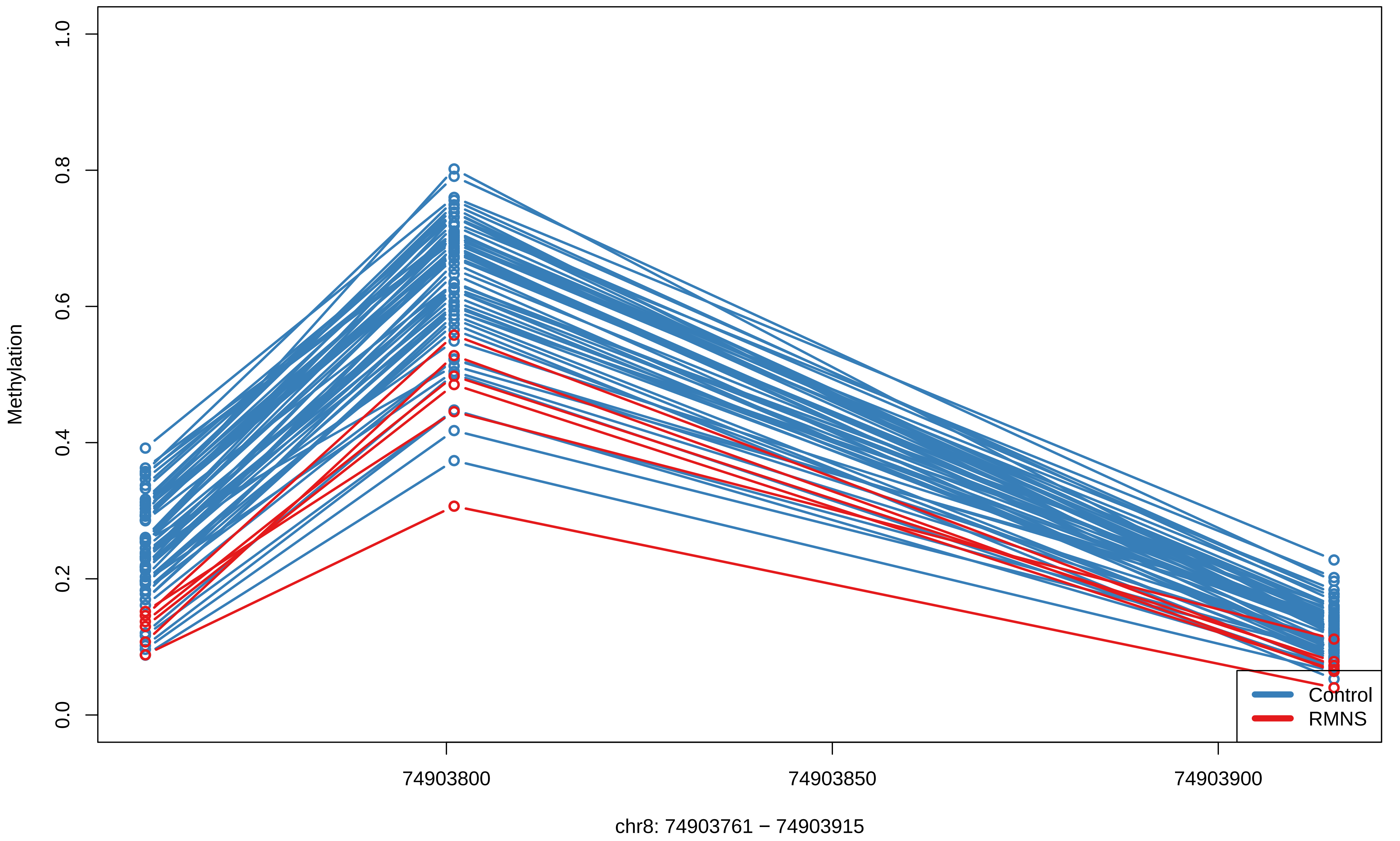

Methylation
0.0
0.2
0.4
0.6
0.8
1.0
74903800
74903850
74903900
chr8: 74903761 − 74903915
Control
RMNS

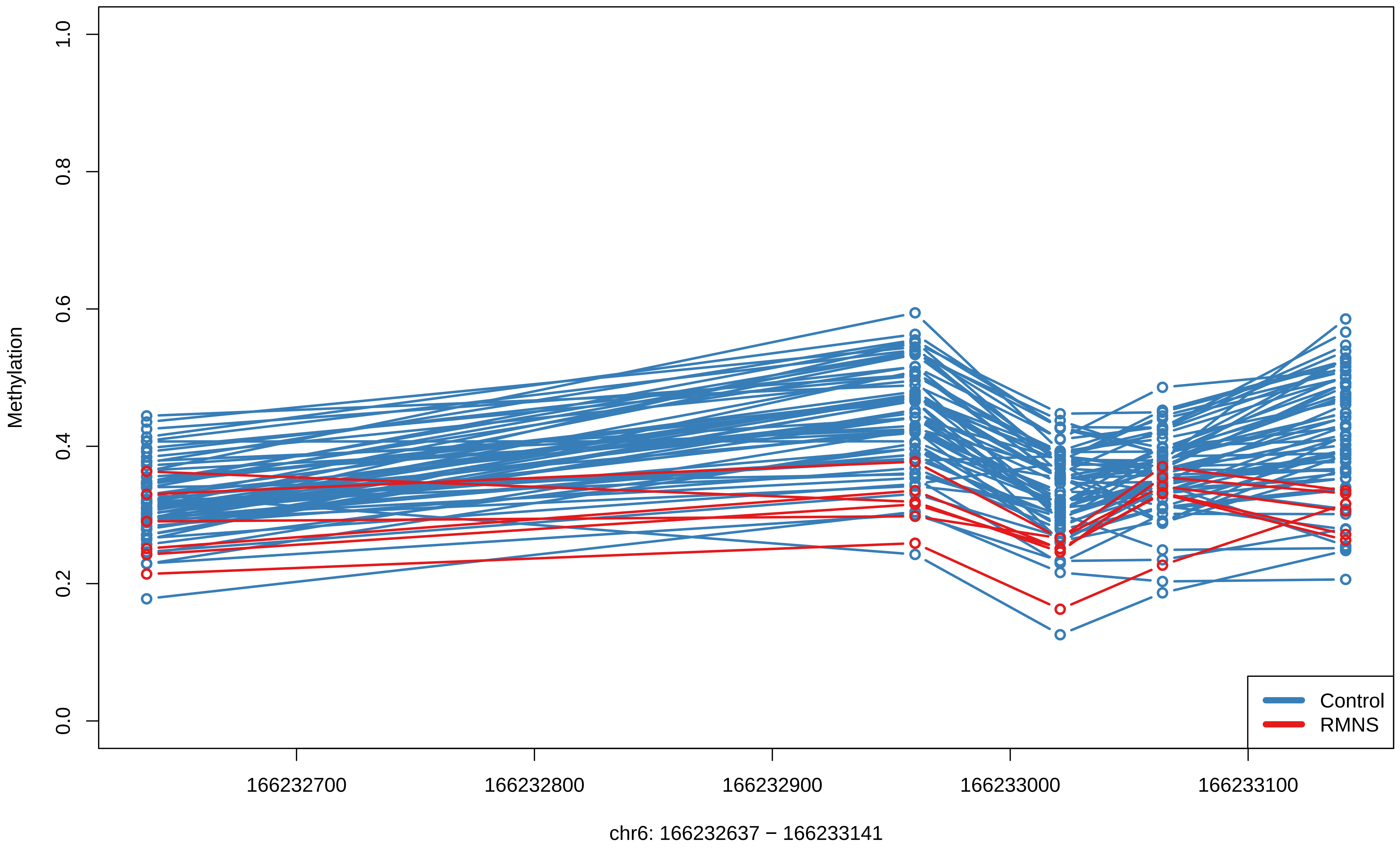
Methylation
0.0
0.2
0.4
0.6
0.8
1.0
166232700
166232800
166232900
166233000
166233100
chr6: 166232637 − 166233141
Control
RMNS

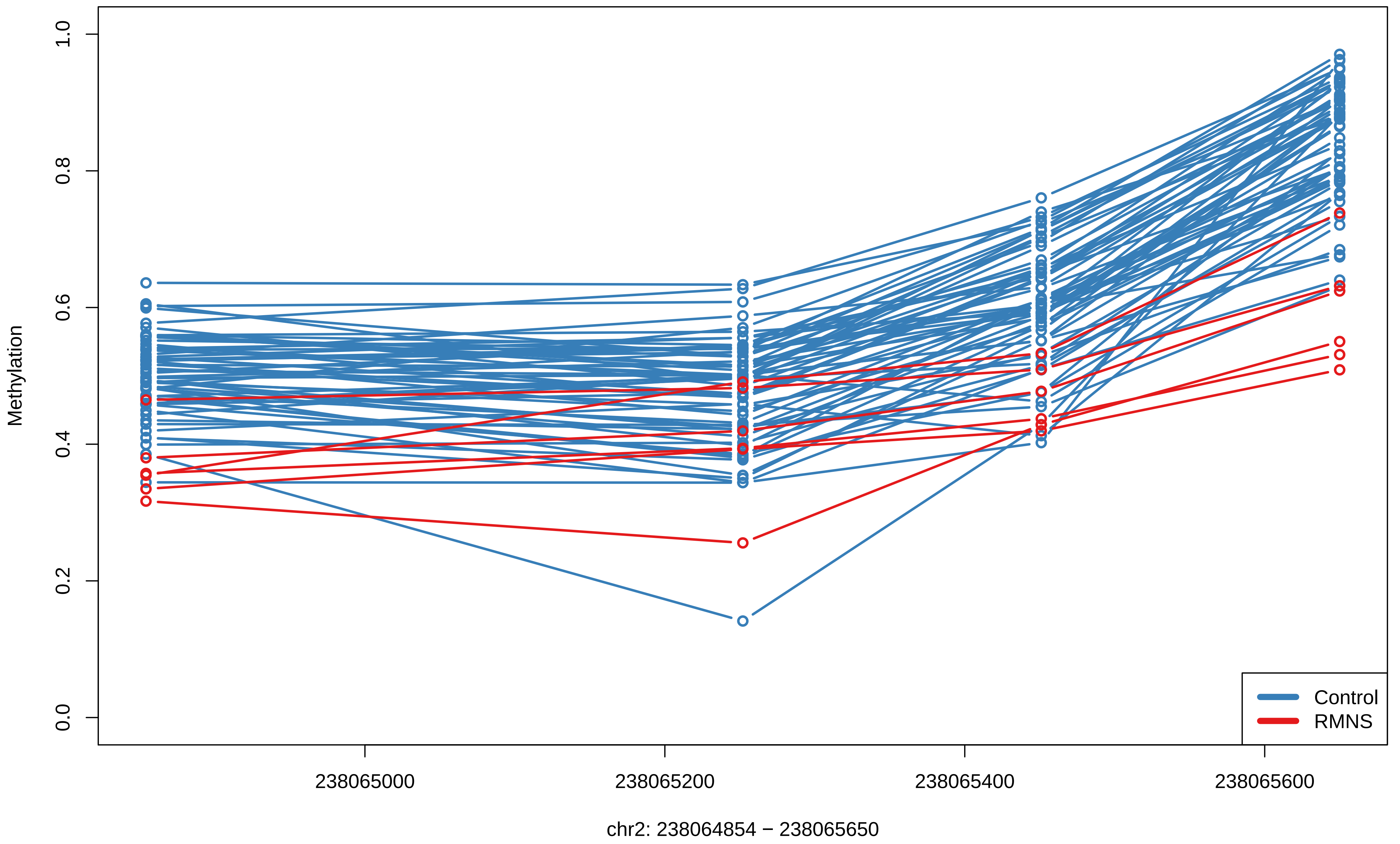

Methylation
1.0
0.8
0.6
0.4
0.2
0.0
238065000
238065200
238065400
238065600
chr2: 238064854 − 238065650
Control
RMNS

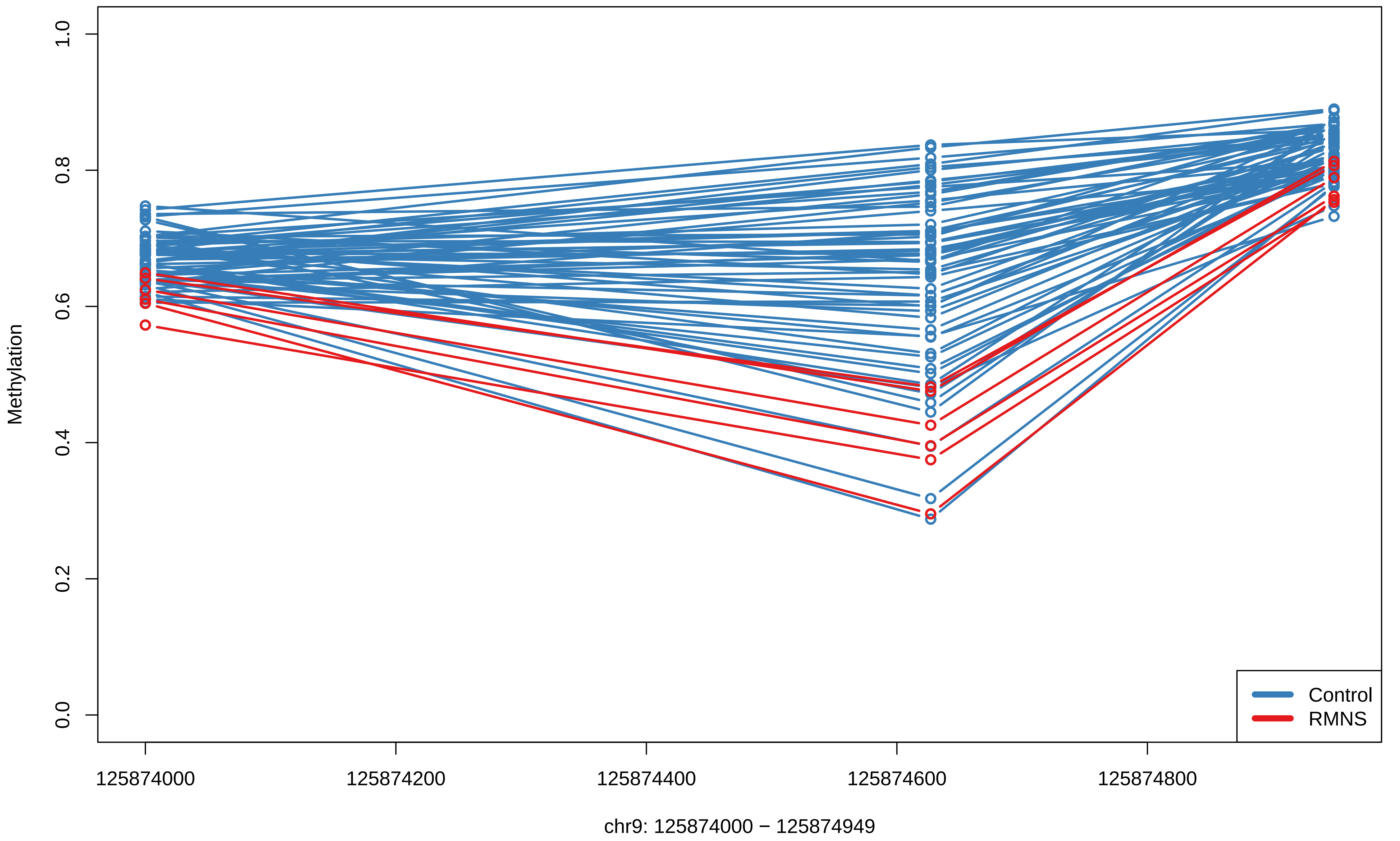

1.0
0.8
0.6
0.4
0.2
0.0
Methylation
125874000
125874200
125874400
125874600
125874800
chr9: 125874000 − 125874949
Control
RMNS

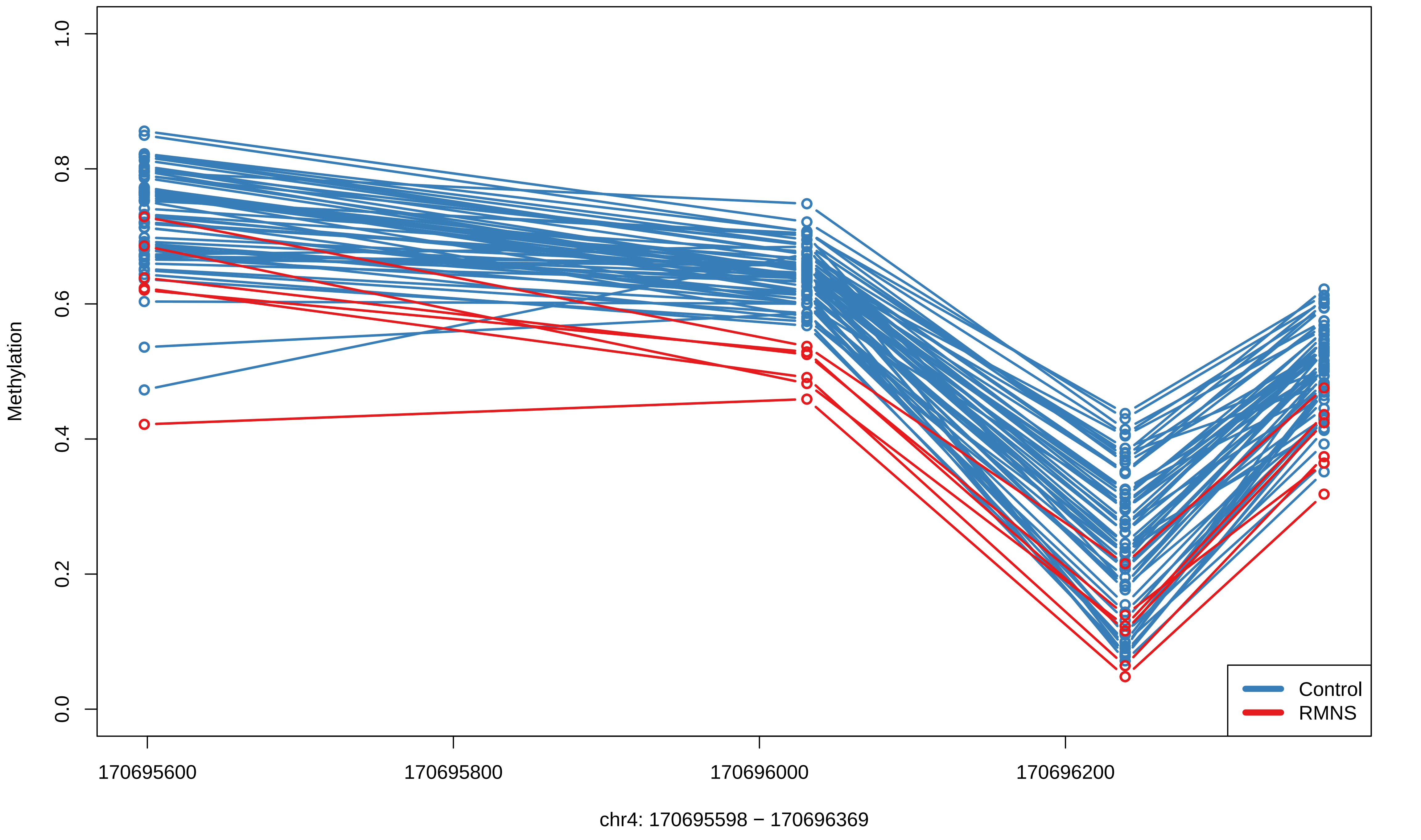

Methylation
1.0
0.8
0.6
0.4
0.2
0.0
170695600
170695800
170696000
170696200
chr4: 170695598 − 170696369
Control
RMNS

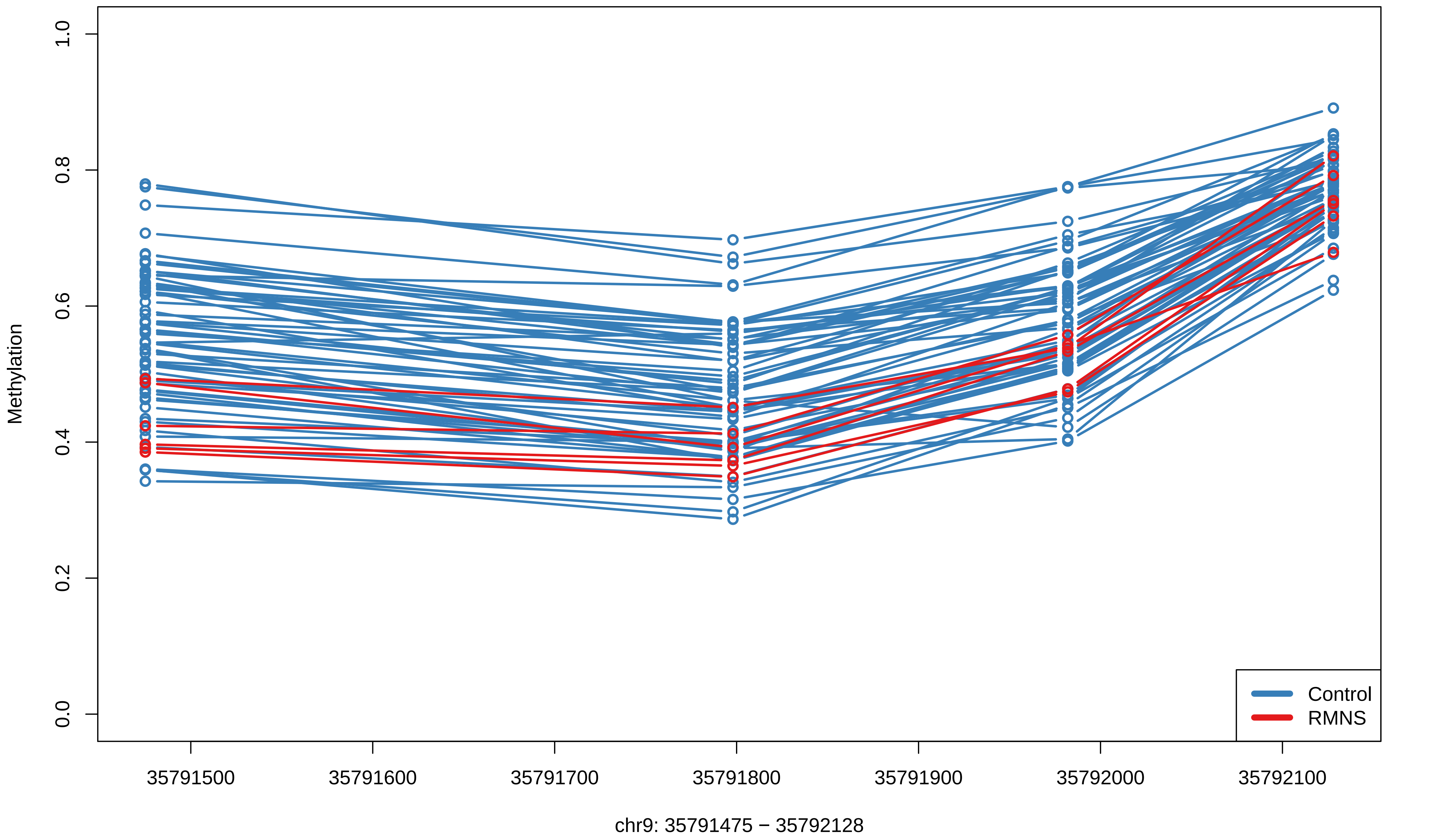

Methylation
0.0
0.2
0.4
0.6
0.8
1.0
35791500
35791600
35791700
35791800
35791900
35792000
35792100
chr9: 35791475 − 35792128
Control
RMNS

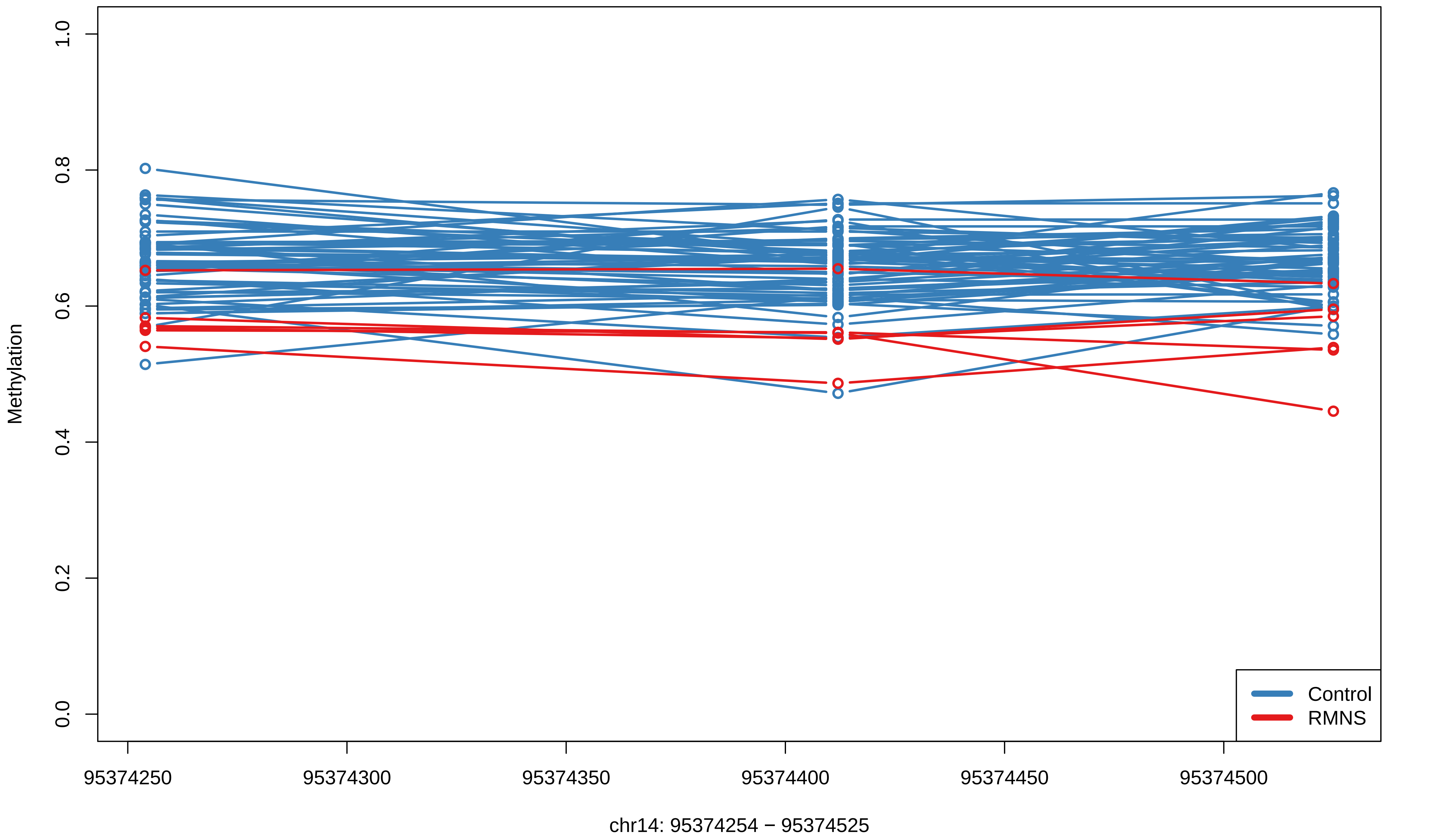

Methylation
0.0
0.2
0.4
0.6
0.8
1.0
95374250
95374300
95374350
95374400
95374450
95374500
chr14: 95374254 − 95374525
Control
RMNS

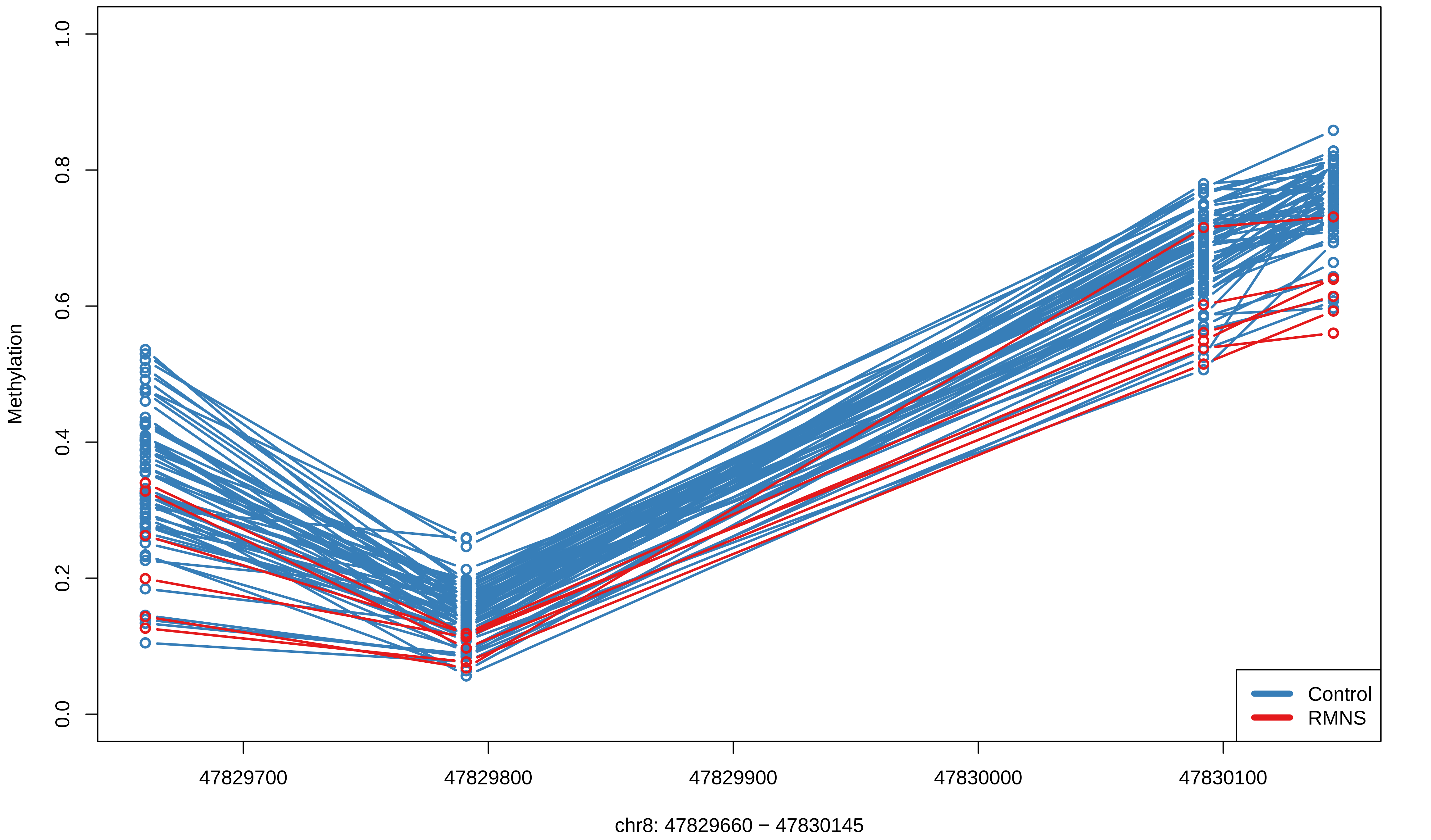

Methylation
0.0
0.2
0.4
0.6
0.8
1.0
47829700
47829800
47829900
47830000
47830100
chr8: 47829660 − 47830145
Control
RMNS

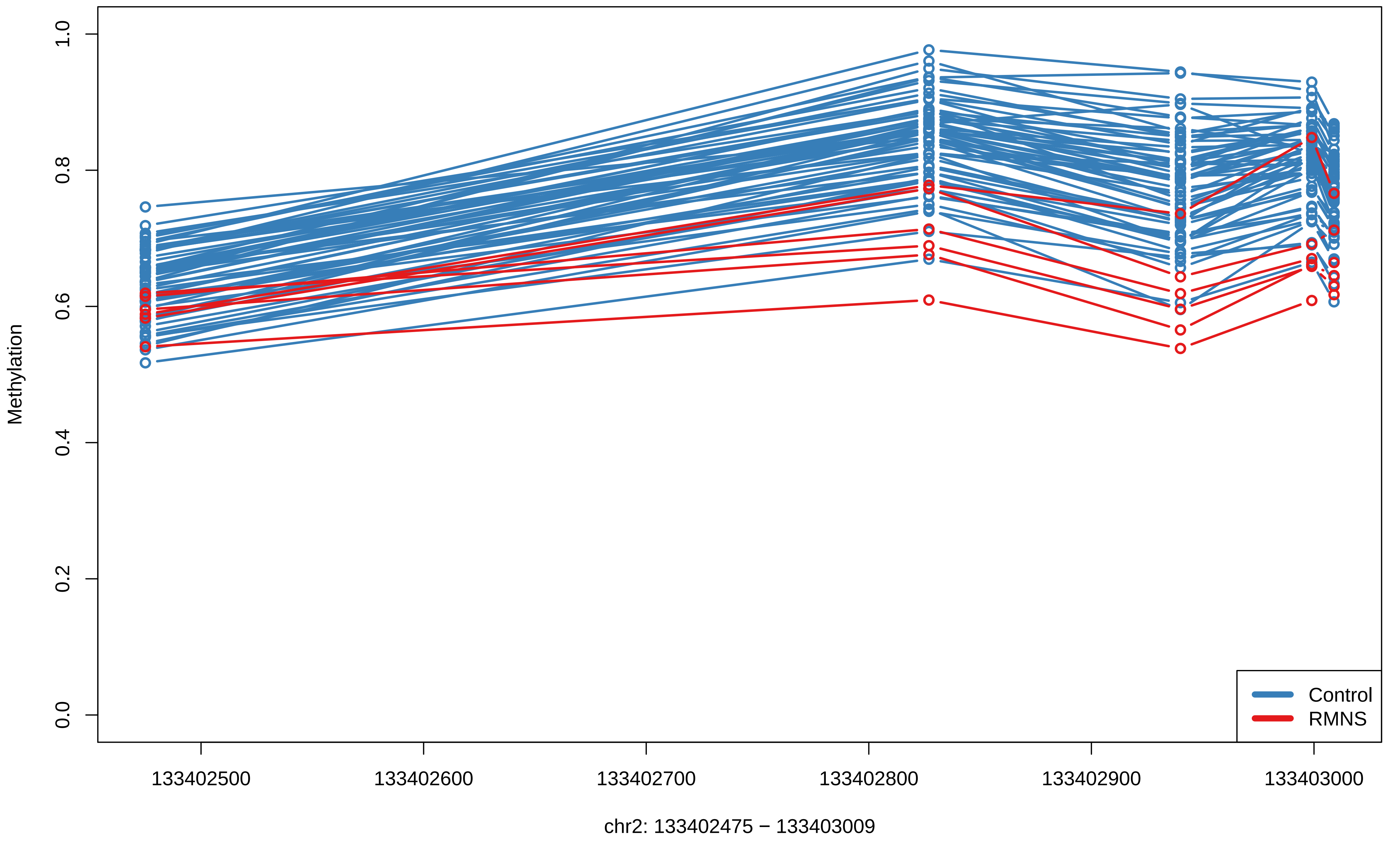

Methylation
0.0
0.2
0.4
0.6
0.8
1.0
133402500
133402600
133402700
133402800
133402900
133403000
chr2: 133402475 − 133403009
Control
RMNS

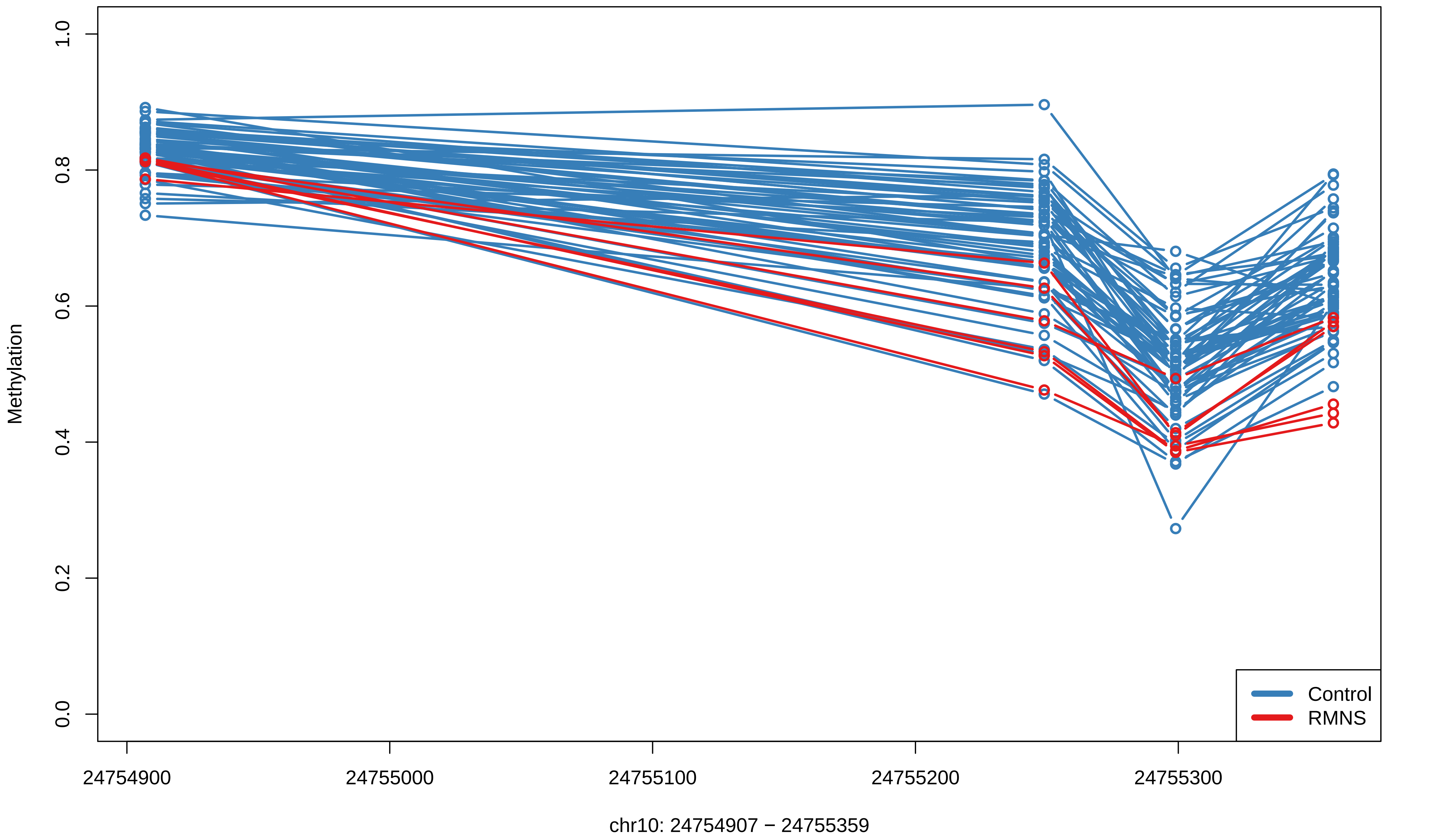

Methylation
1.0
0.8
0.6
0.4
0.2
0.0
24754900
24755000
24755100
24755200
24755300
chr10: 24754907 − 24755359
Control
RMNS

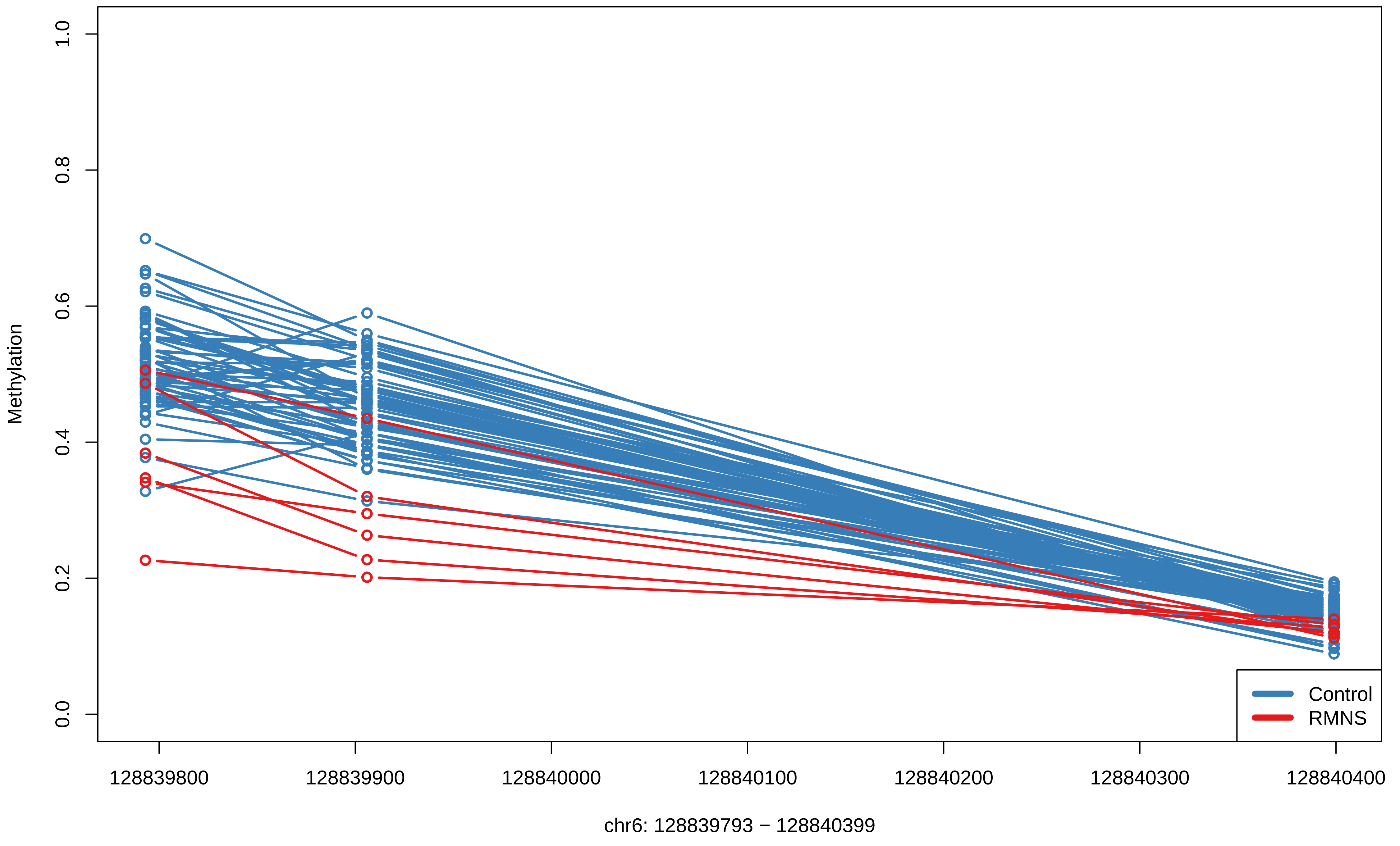

Methylation
0.0
0.2
0.4
0.6
0.8
1.0
128839800
128839900
128840000
128840100
128840200
128840300
128840400
chr6: 128839793 − 128840399
Control
RMNS

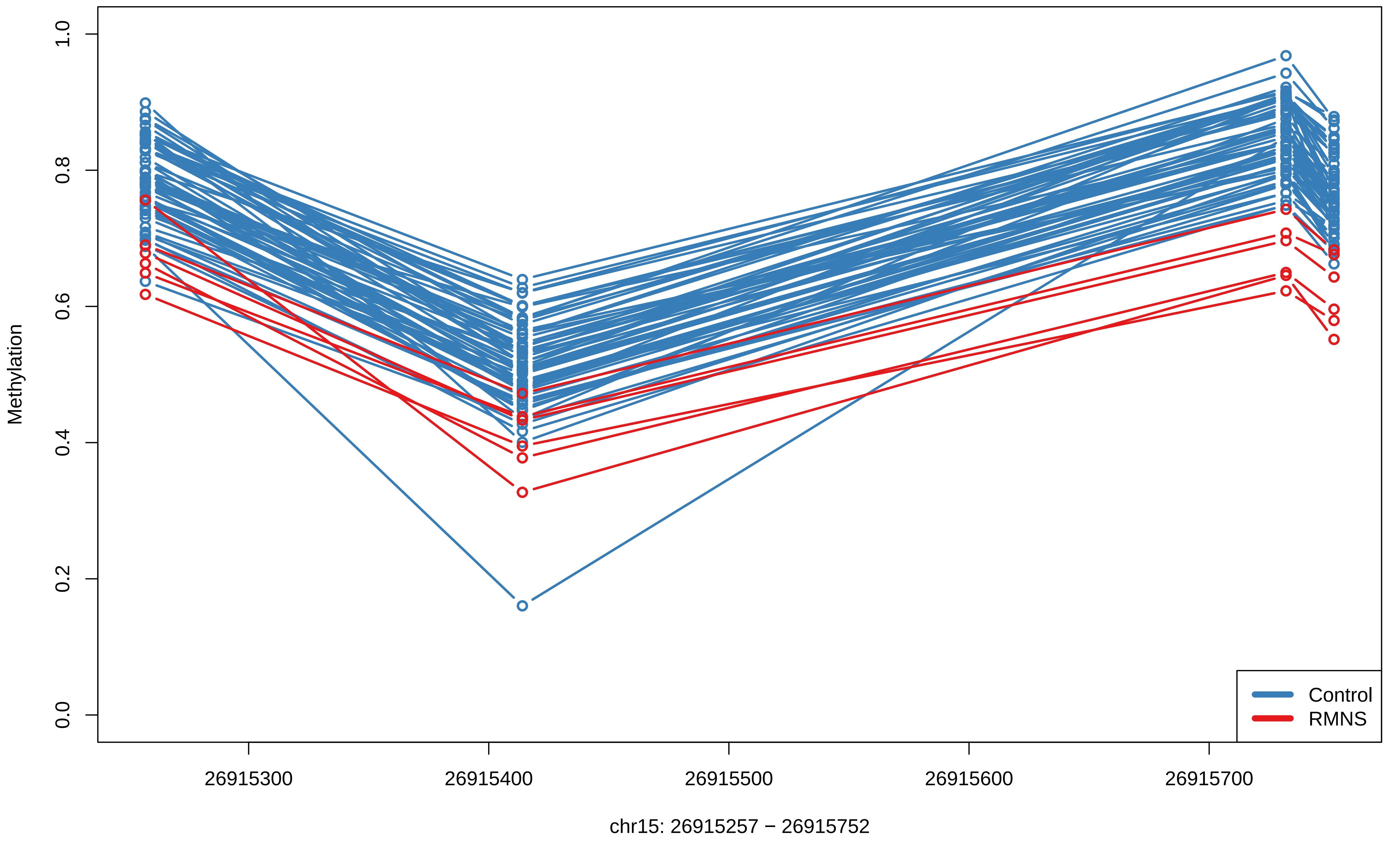

Methylation
1.0
0.8
0.6
0.4
0.2
0.0
26915300
26915400
26915500
26915600
26915700
chr15: 26915257 − 26915752
Control
RMNS

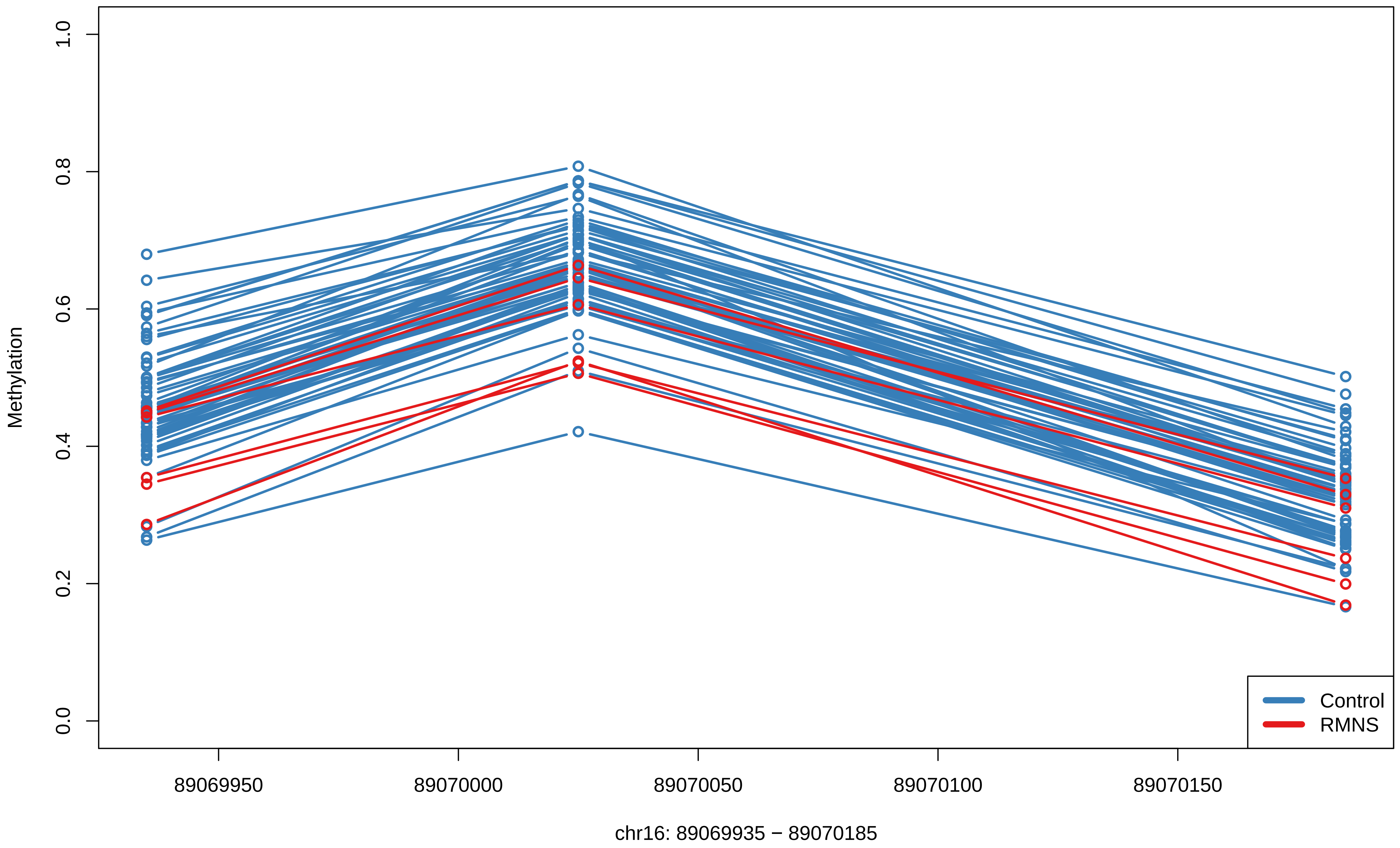

Methylation
1.0
0.8
0.6
0.4
0.2
0.0
89069950
89070000
89070050
89070100
89070150
chr16: 89069935 − 89070185
Control
RMNS

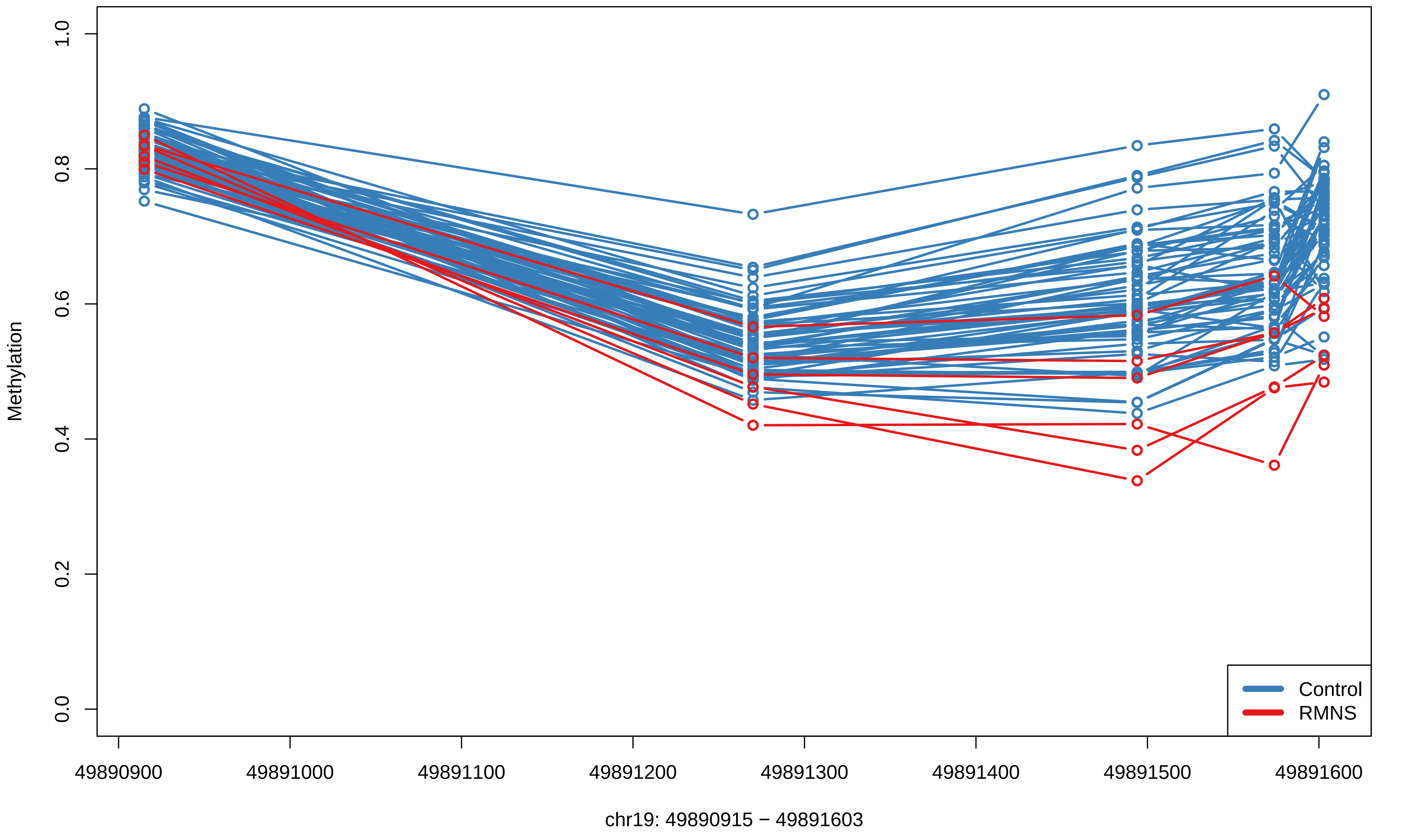

1.0
0.8
0.6
0.4
0.2
0.0
Methylation
49890900
49891000
49891100
49891200
49891300
49891400
49891500
49891600
chr19: 49890915 − 49891603
Control
RMNS

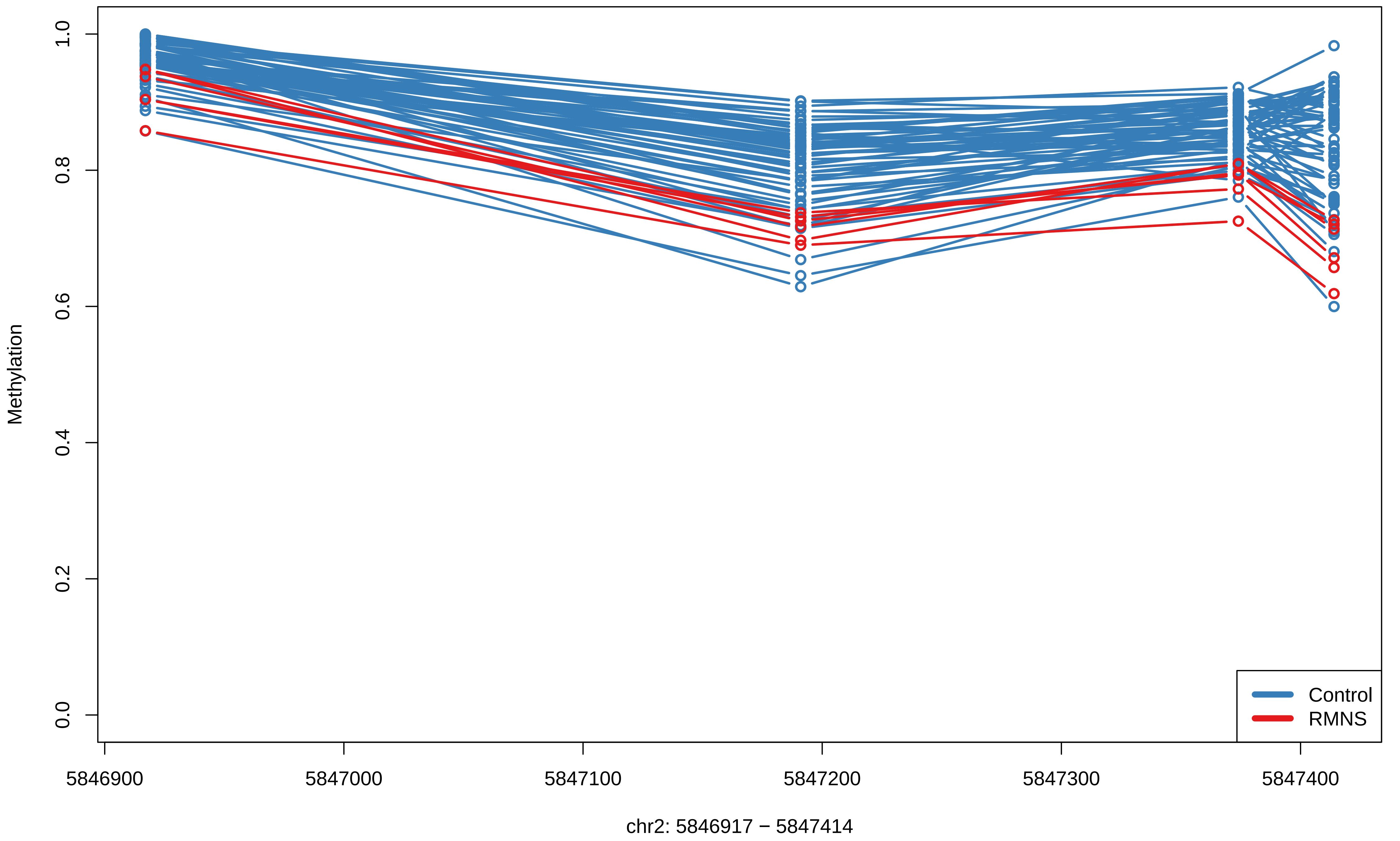

1.0
0.8
0.6
0.4
0.2
0.0
Methylation
5846900
5847000
5847100
5847200
5847300
5847400
chr2: 5846917 − 5847414
Control
RMNS

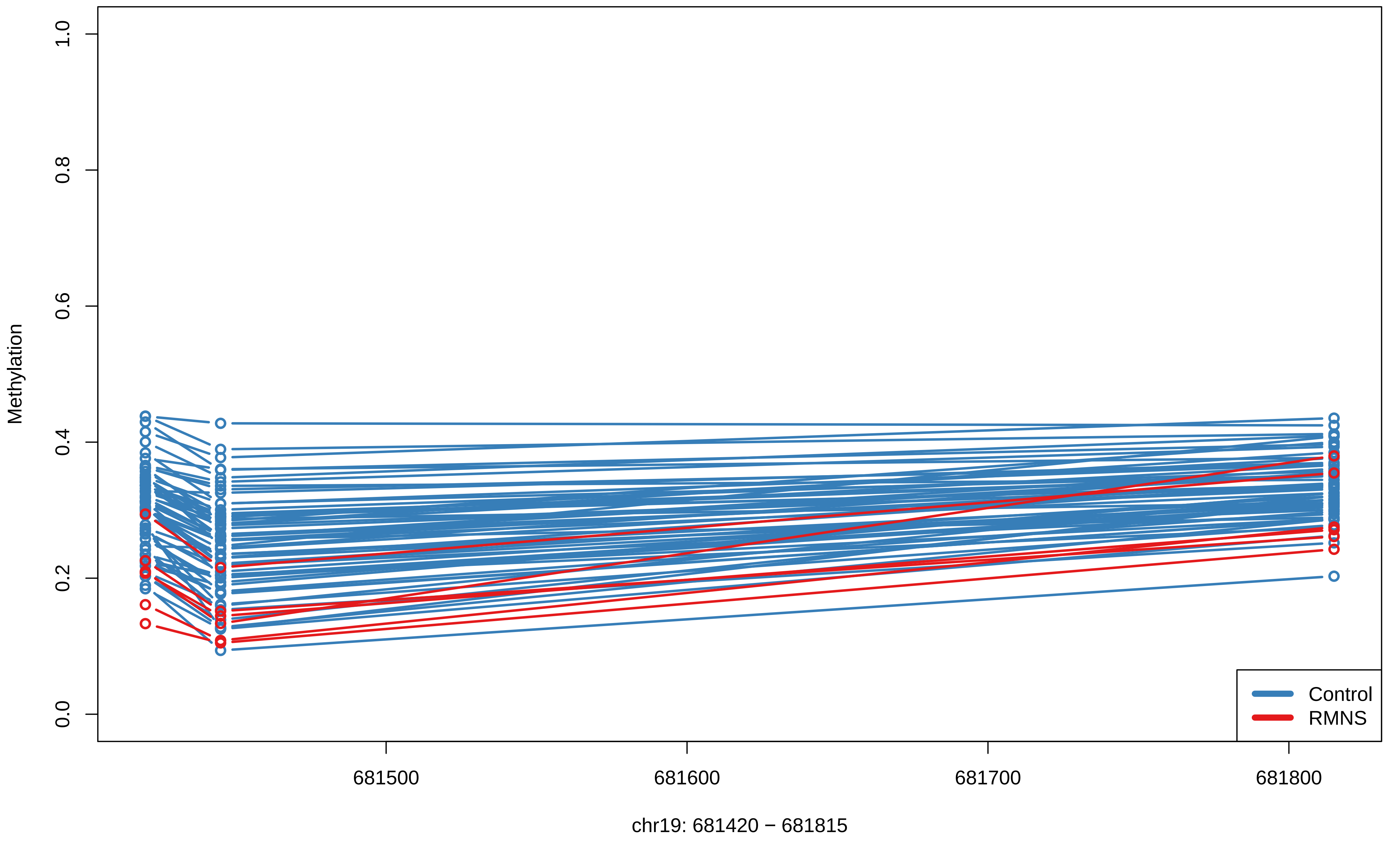

Methylation
0.0
0.2
0.4
0.6
0.8
1.0
681500
681600
681700
681800
chr19: 681420 − 681815
Control
RMNS

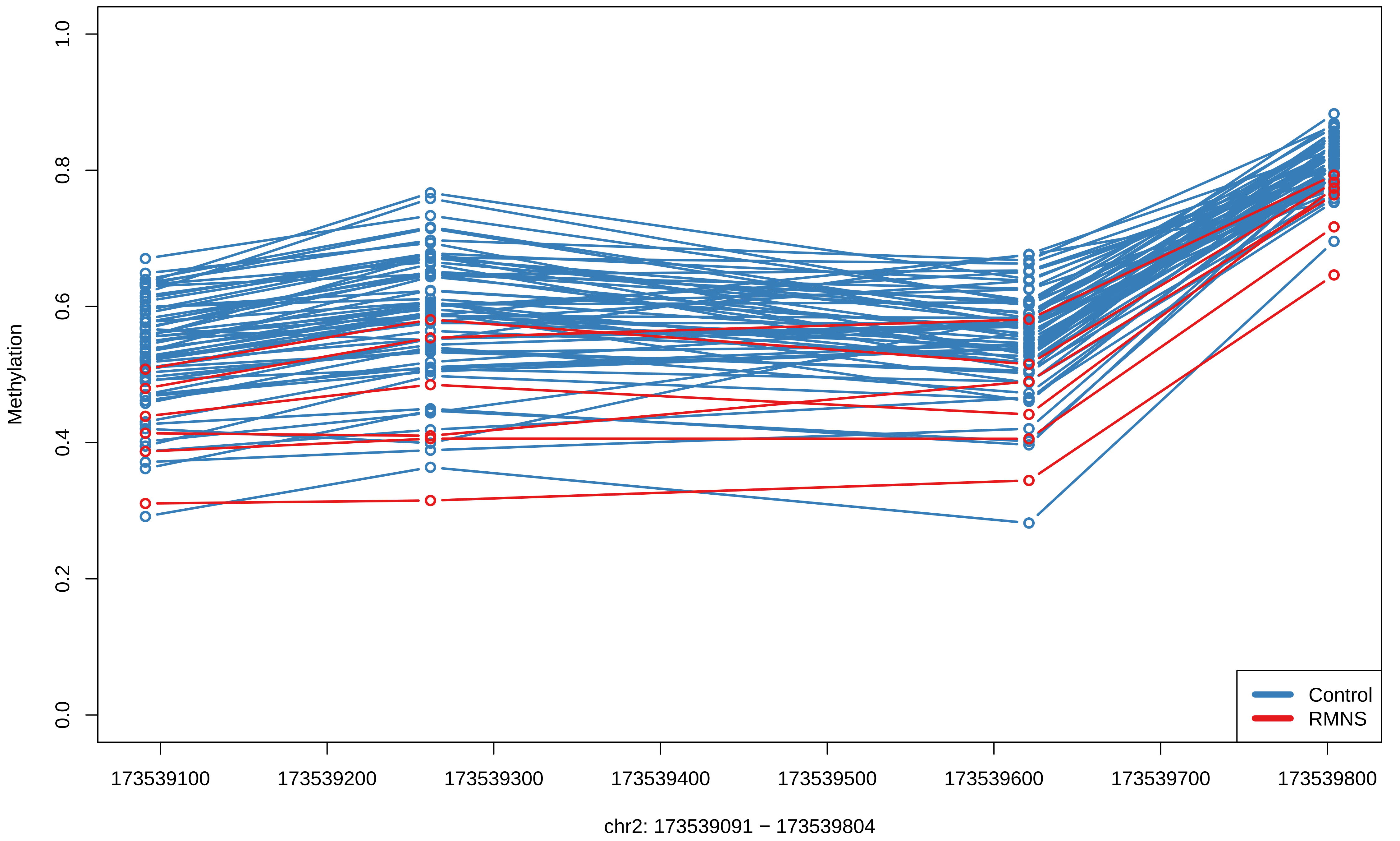

Methylation
0.0
0.2
0.4
0.6
0.8
1.0
173539100
173539200
173539300
173539400
173539500
173539600
173539700
173539800
chr2: 173539091 − 173539804
Control
RMNS

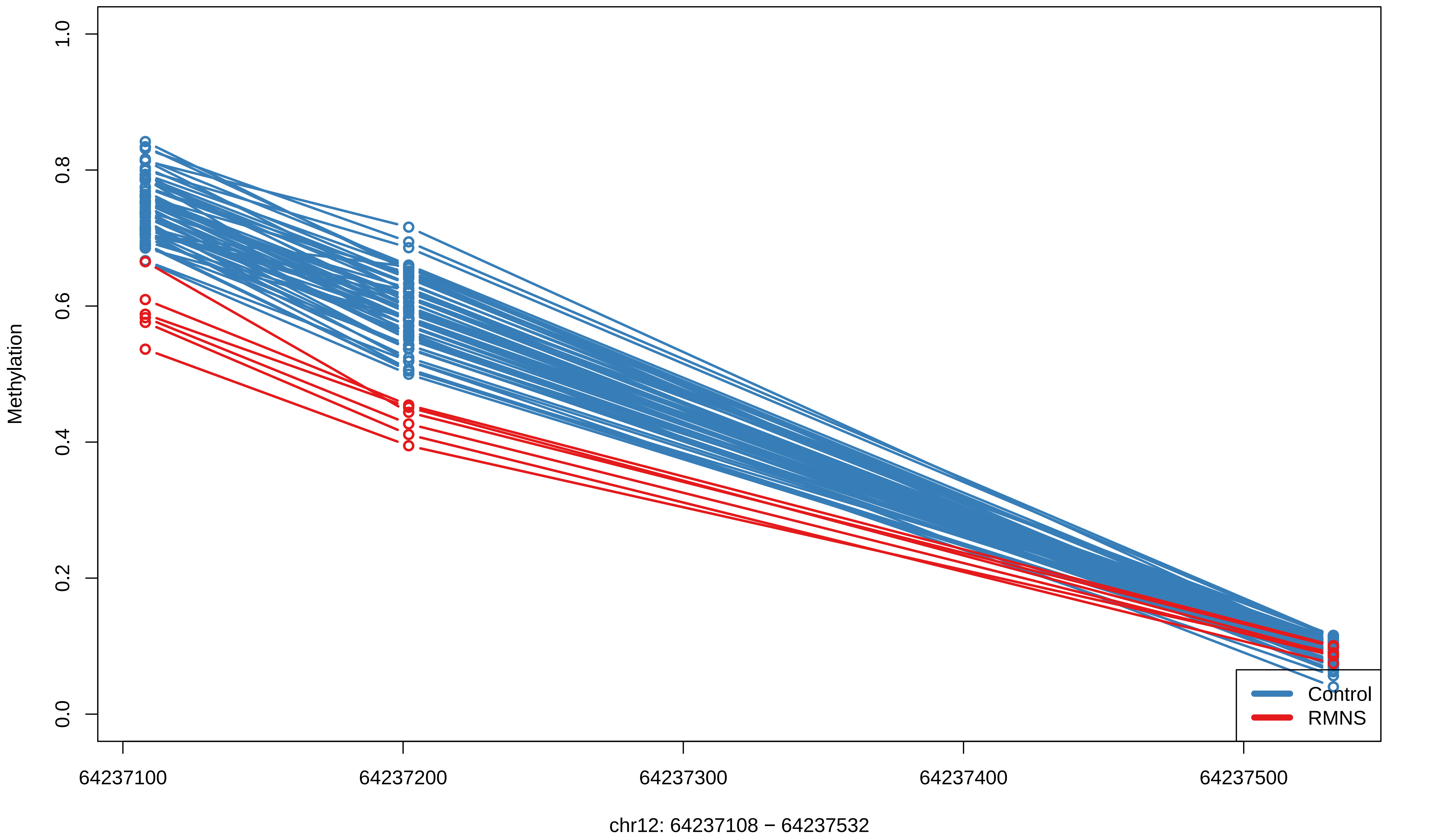

1.0
0.8
0.6
0.4
0.2
0.0
Methylation
64237100
64237200
64237300
64237400
64237500
chr12: 64237108 − 64237532
Control
RMNS

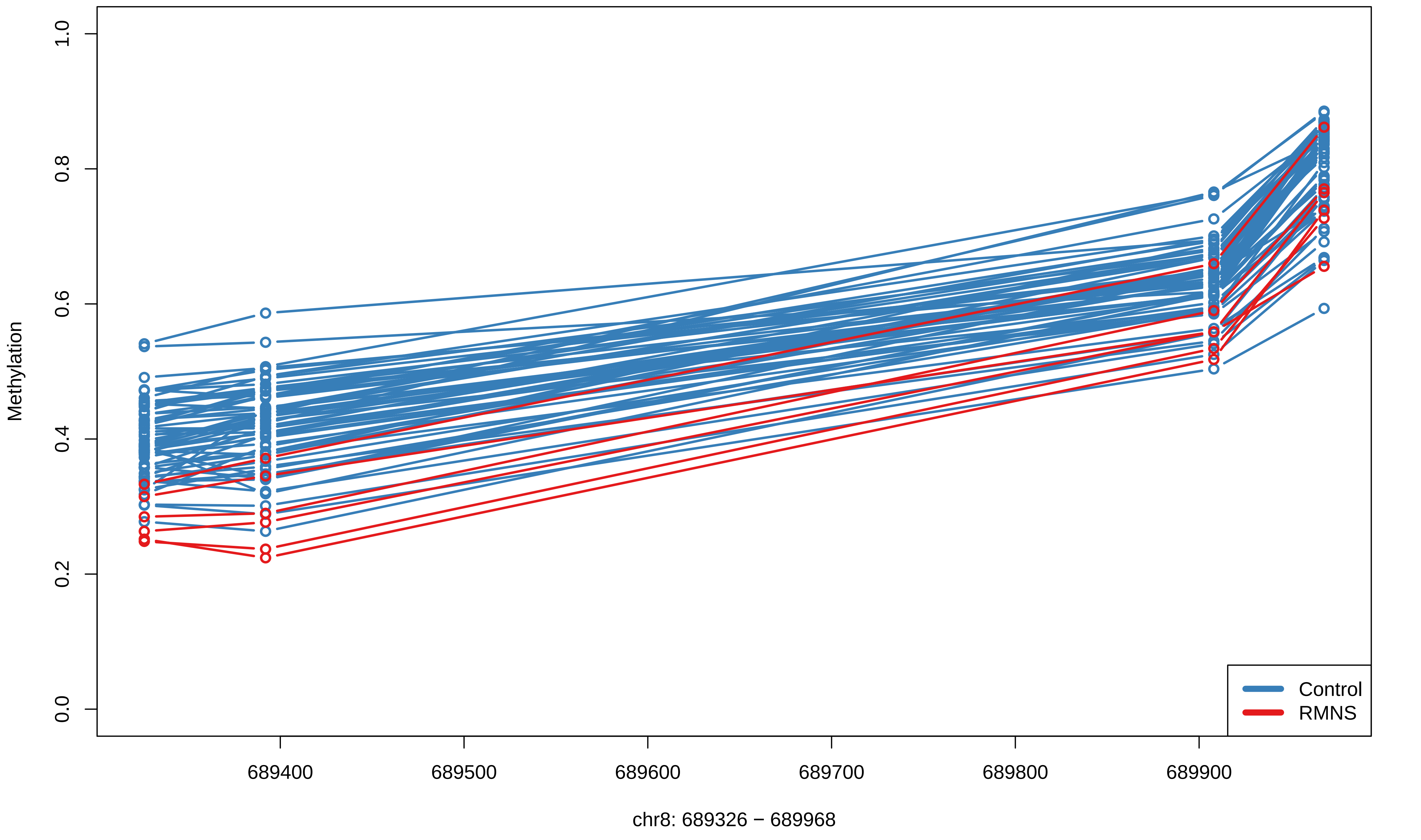
Methylation
0.0
0.2
0.4
0.6
0.8
1.0
689400
689500
689600
689700
689800
689900
chr8: 689326 − 689968
Control
RMNS

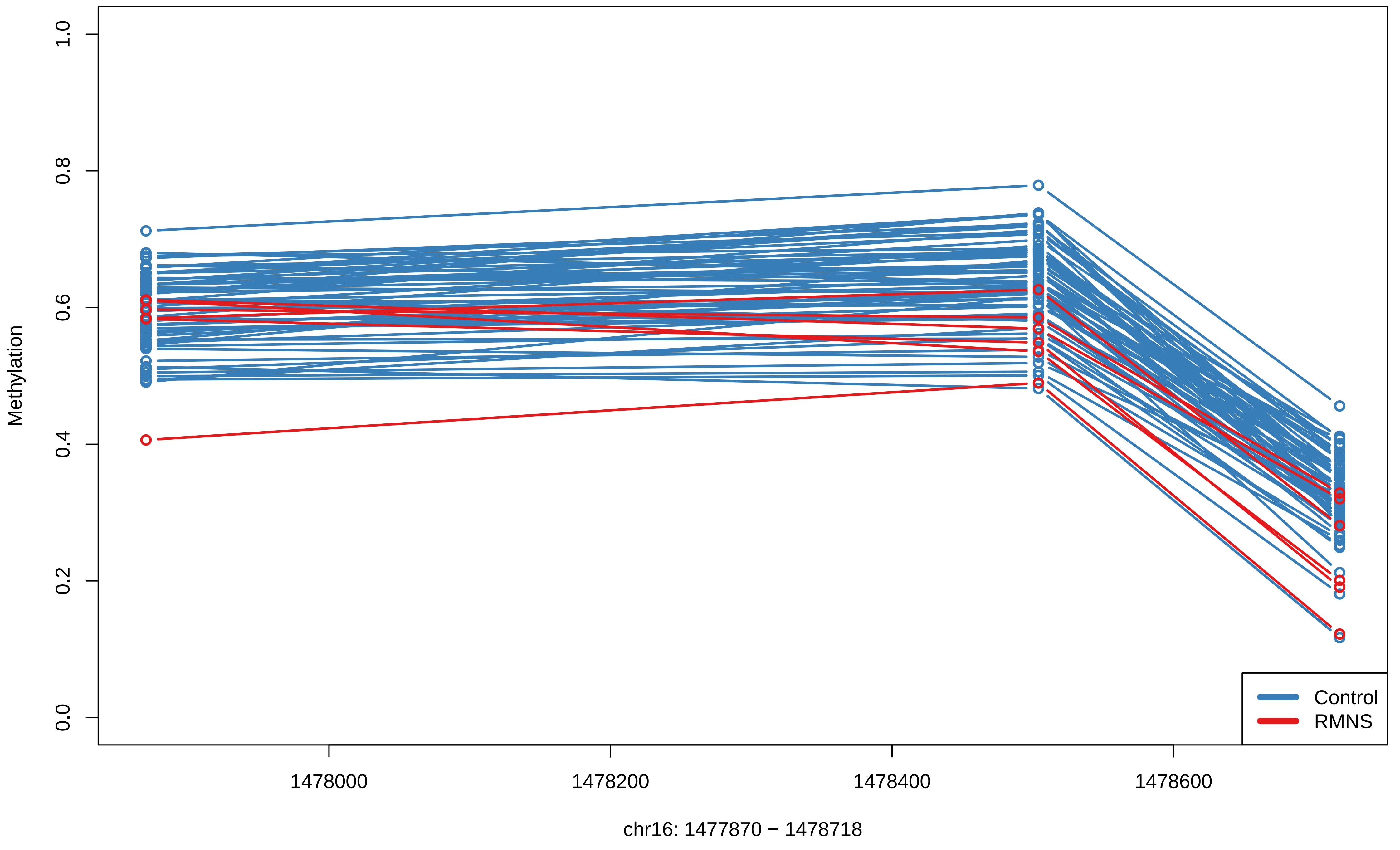

Methylation
0.0
0.2
0.4
0.6
0.8
1.0
1478000
1478200
1478400
1478600
chr16: 1477870 − 1478718
Control
RMNS

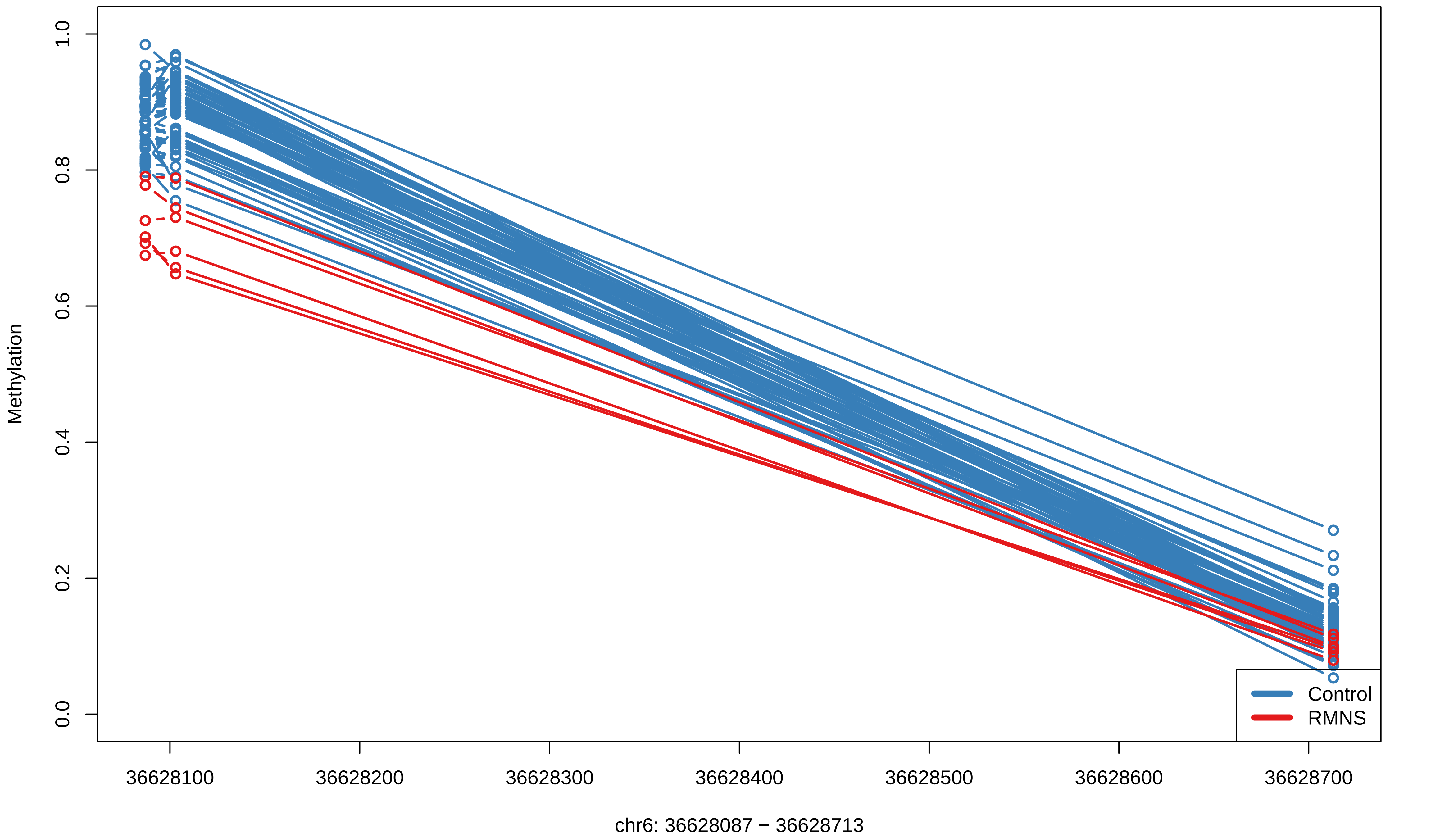

Methylation
0.0
0.2
0.4
0.6
0.8
1.0
36628100
36628200
36628300
36628400
36628500
36628600
36628700
chr6: 36628087 − 36628713
Control
RMNS

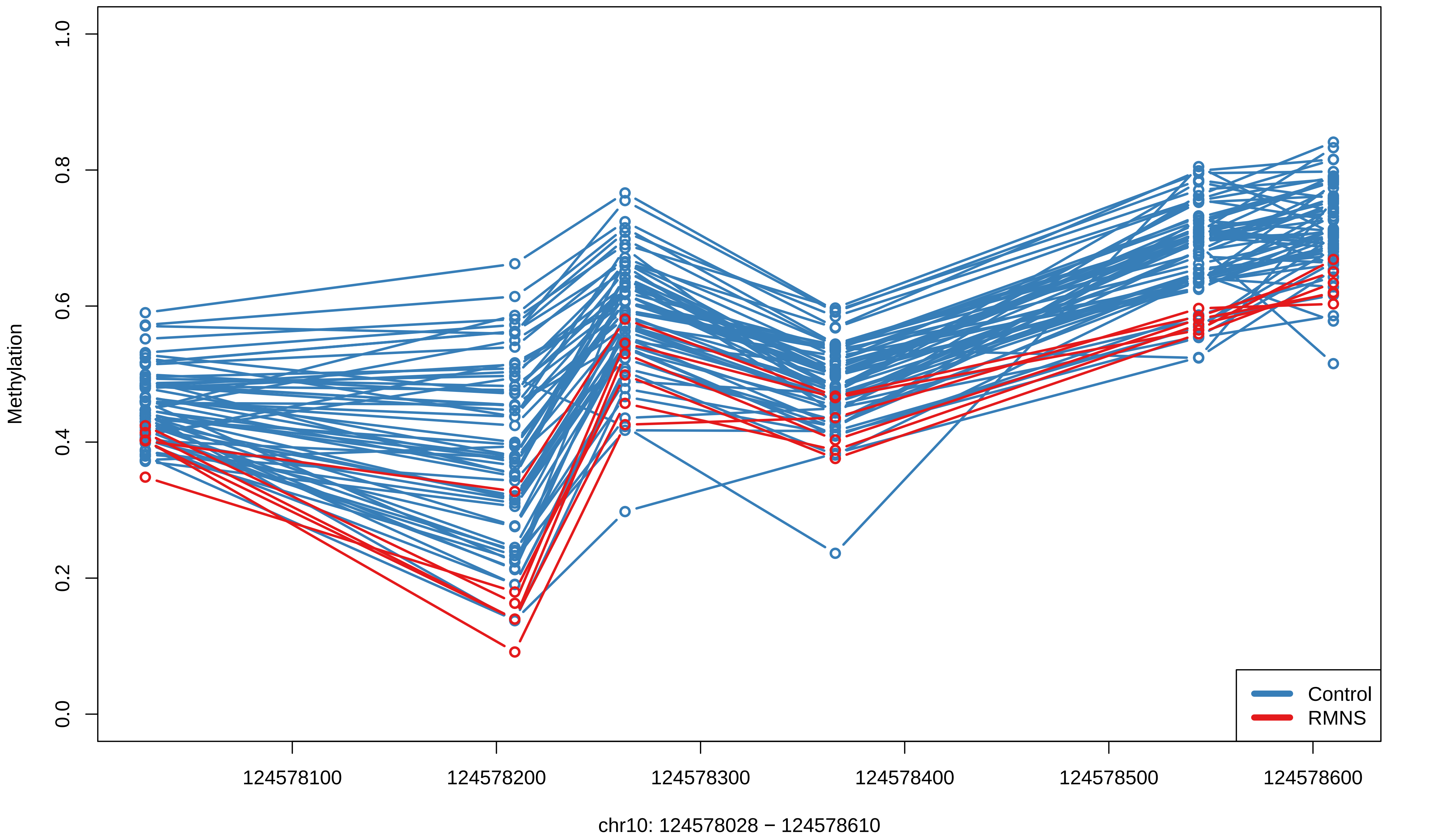

Methylation
0.0
0.2
0.4
0.6
0.8
1.0
124578100
124578200
124578300
124578400
124578500
124578600
chr10: 124578028 − 124578610
Control
RMNS

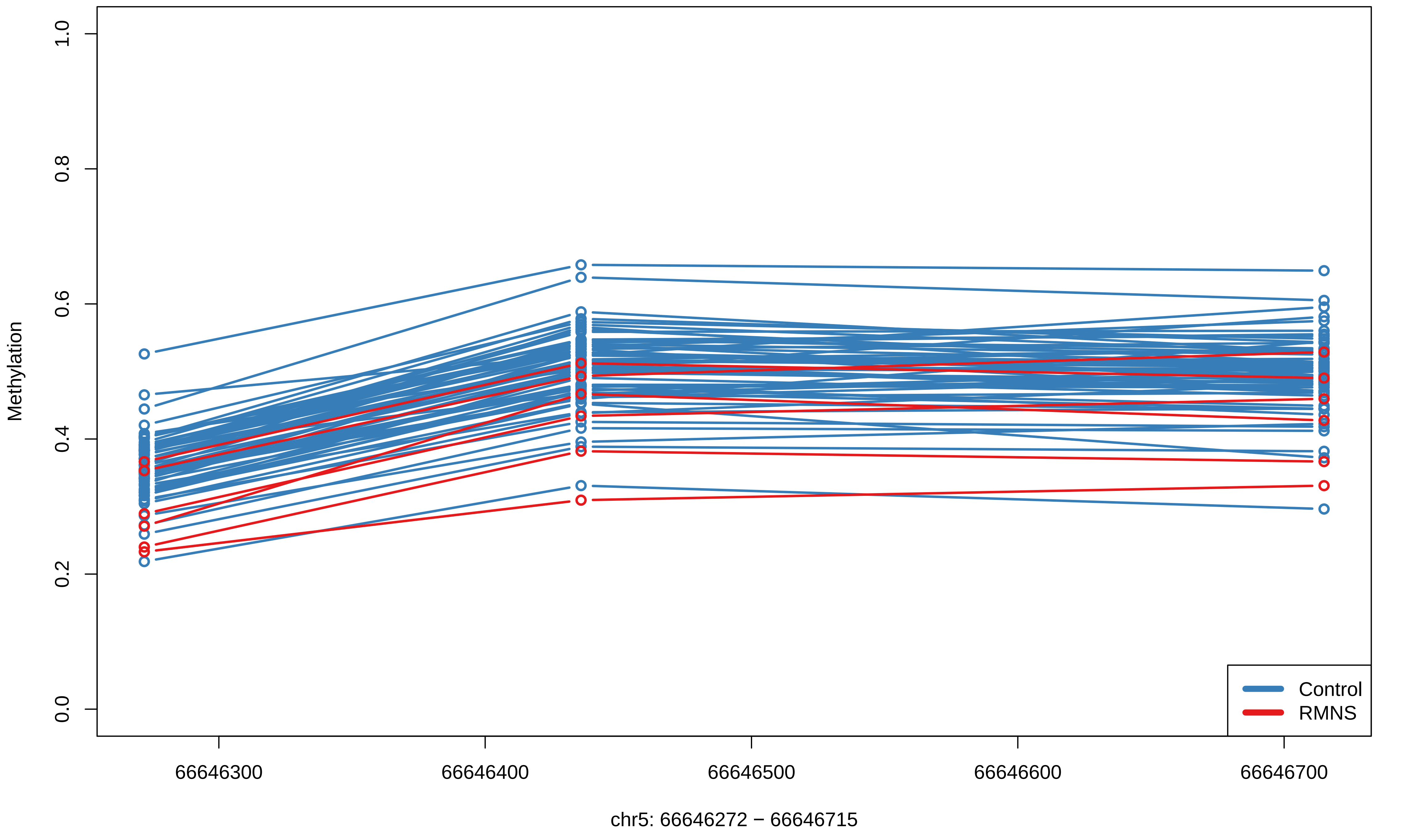

Methylation
1.0
0.8
0.6
0.4
0.2
0.0
66646300
66646400
66646500
66646600
66646700
chr5: 66646272 − 66646715
Control
RMNS

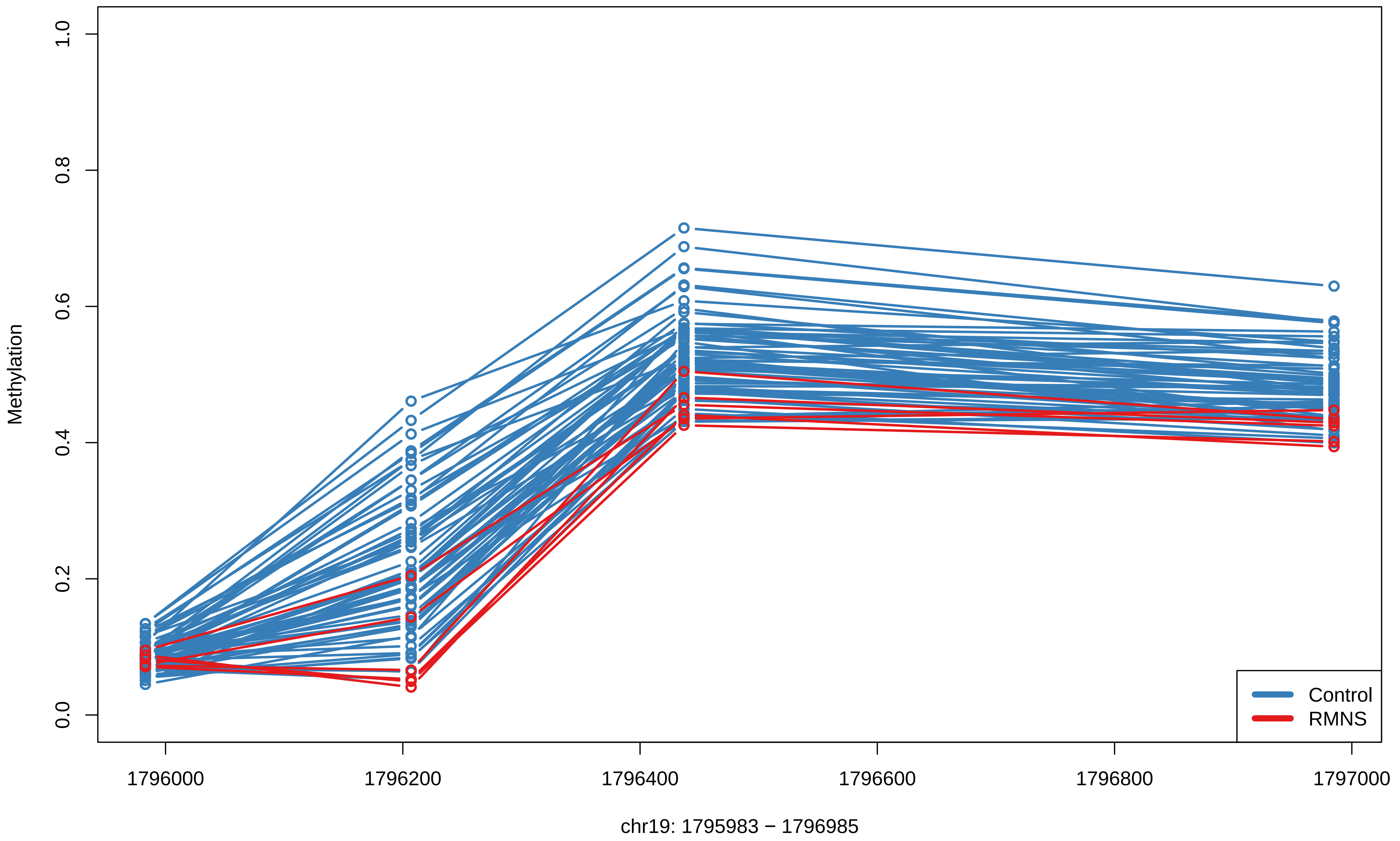

Methylation
0.0
0.2
0.4
0.6
0.8
1.0
1796000
1796200
1796400
1796600
1796800
1797000
chr19: 1795983 − 1796985
Control
RMNS

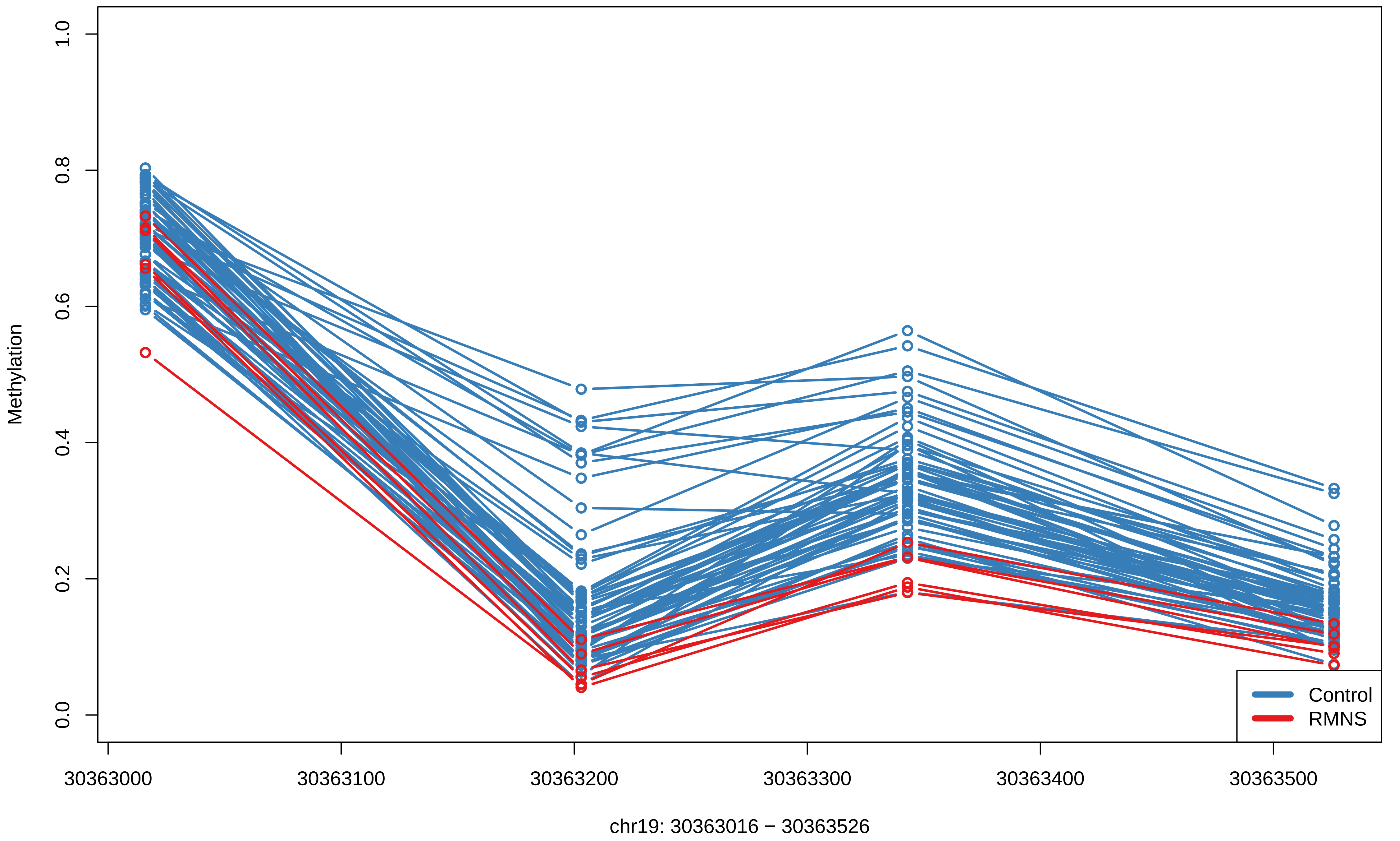

Methylation
0.0
0.2
0.4
0.6
0.8
1.0
30363000
30363100
30363200
30363300
30363400
30363500
chr19: 30363016 − 30363526
Control
RMNS

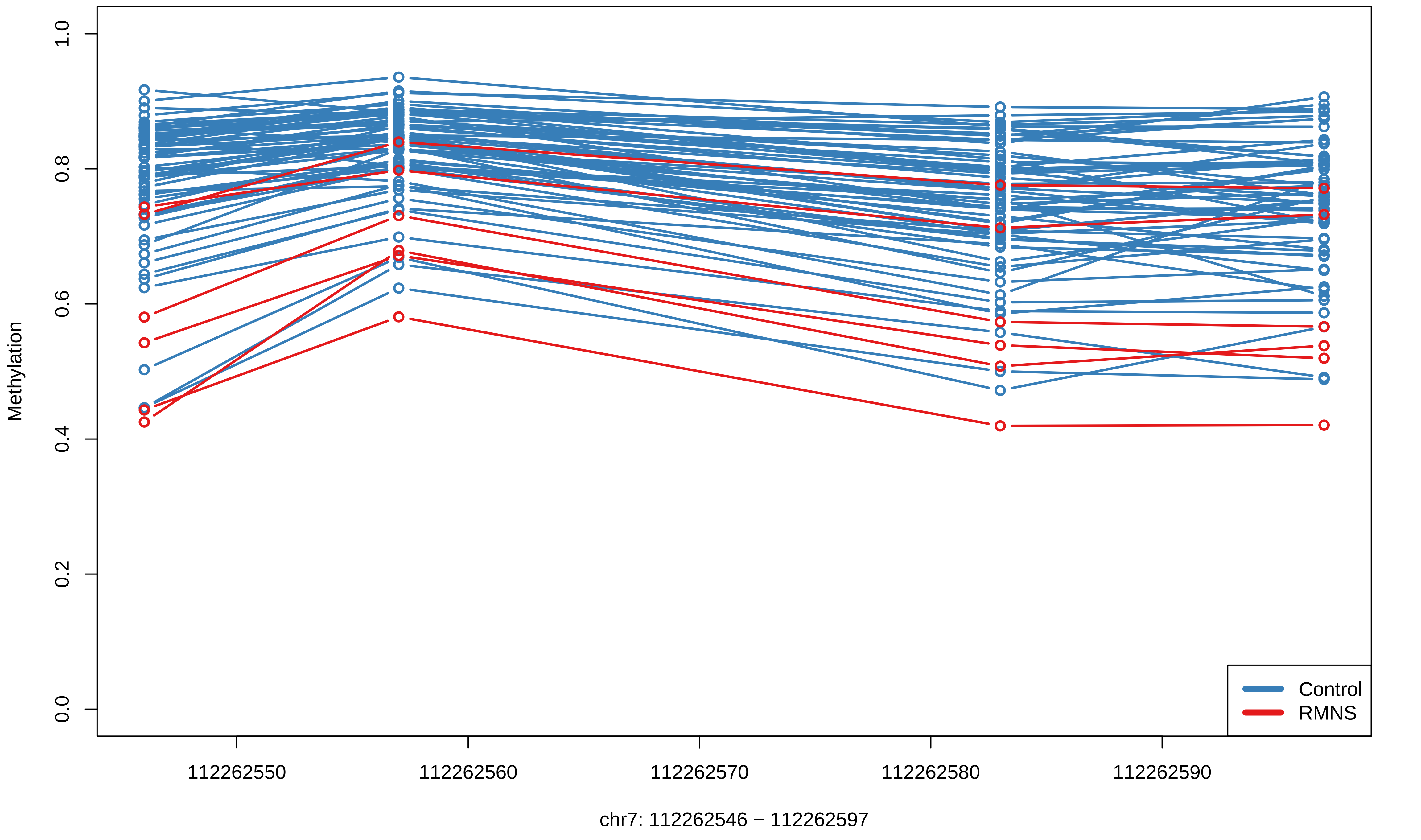

Methylation
1.0
0.8
0.6
0.4
0.2
0.0
112262550
112262560
112262570
112262580
112262590
chr7: 112262546 − 112262597
Control
RMNS

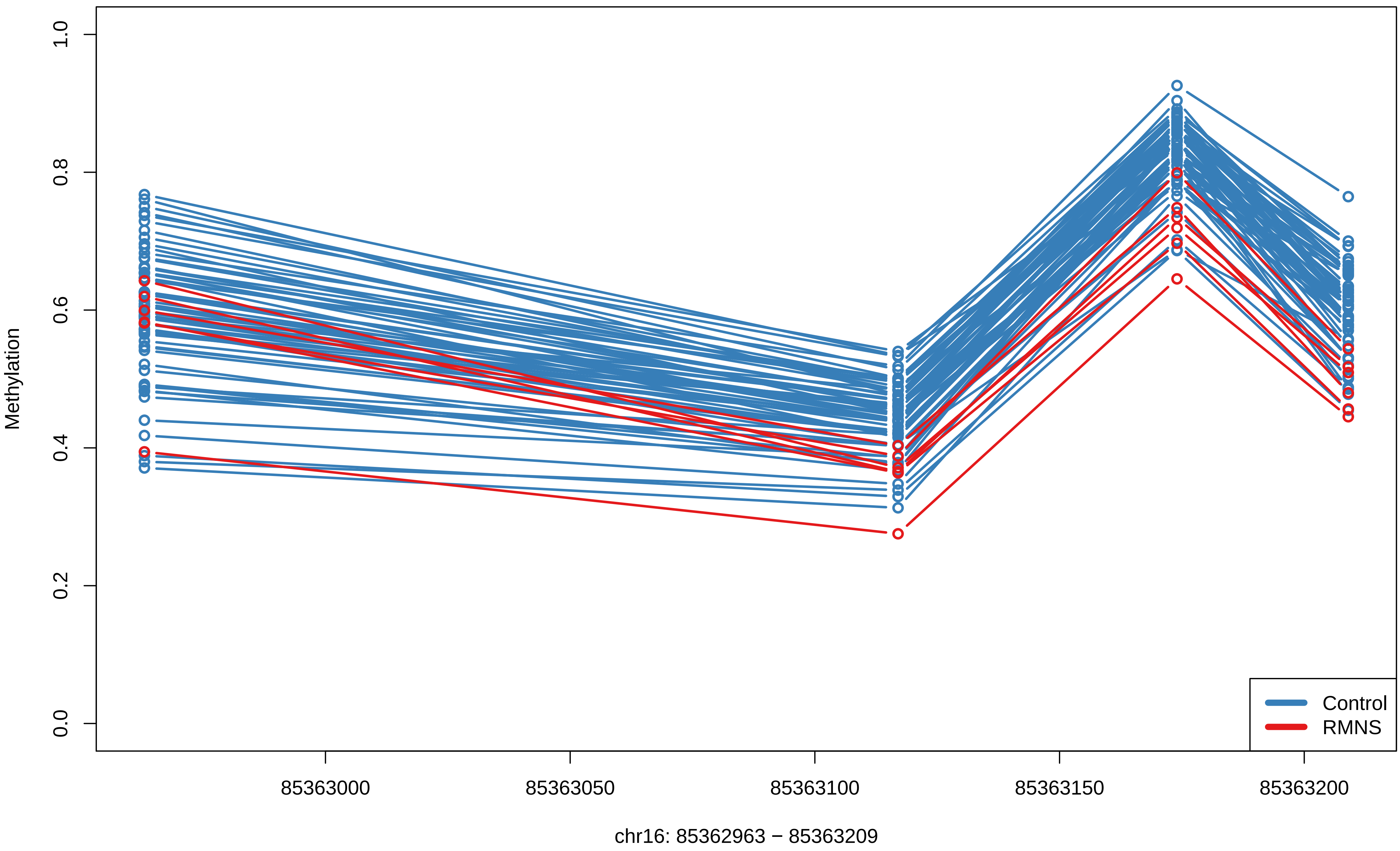

Methylation
0.0
0.2
0.4
0.6
0.8
1.0
85363000
85363050
85363100
85363150
85363200
chr16: 85362963 − 85363209
Control
RMNS

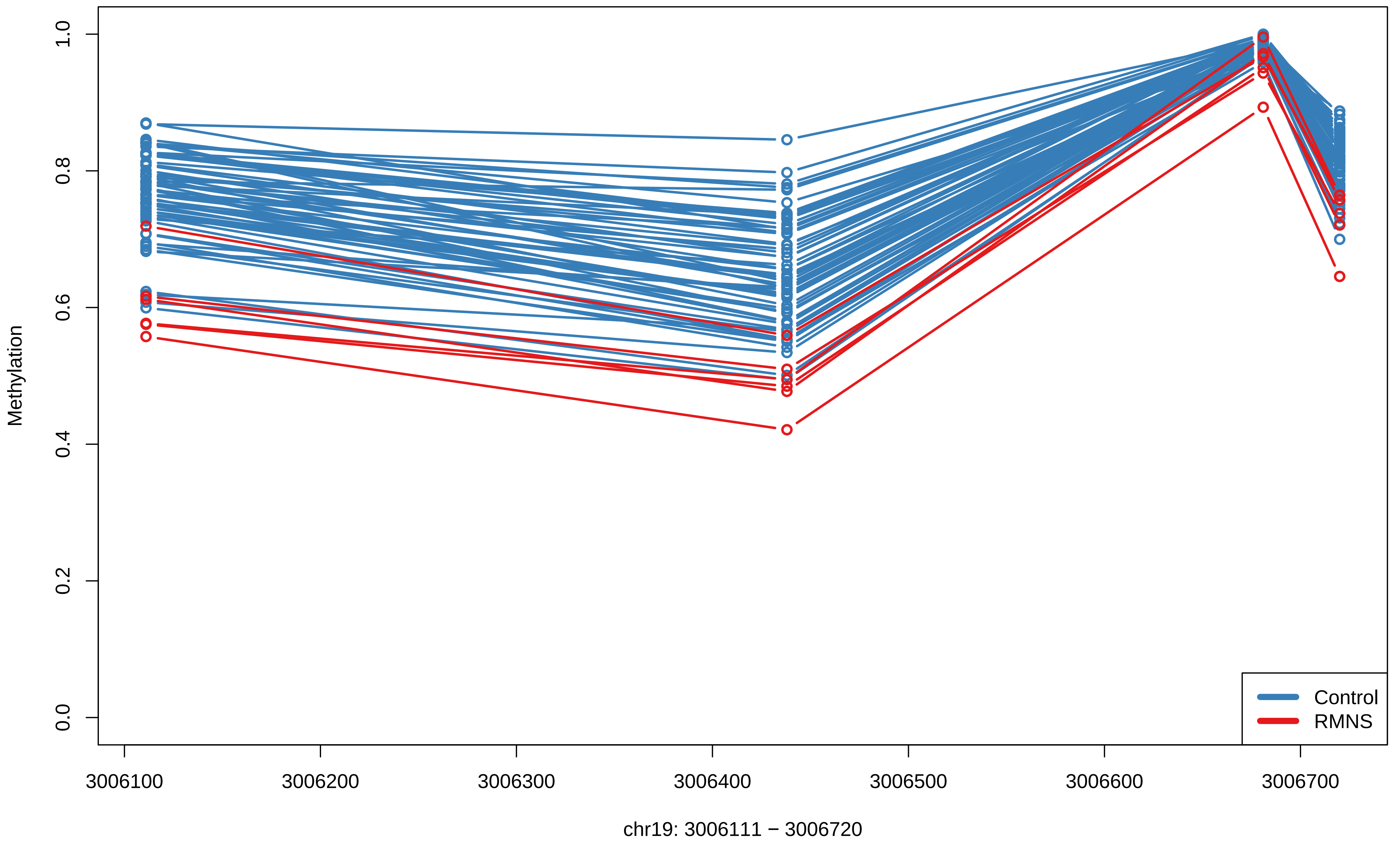

Methylation
1.0
0.8
0.6
0.4
0.2
0.0
3006100
3006200
3006300
3006400
3006500
3006600
3006700
chr19: 3006111 − 3006720
Control
RMNS

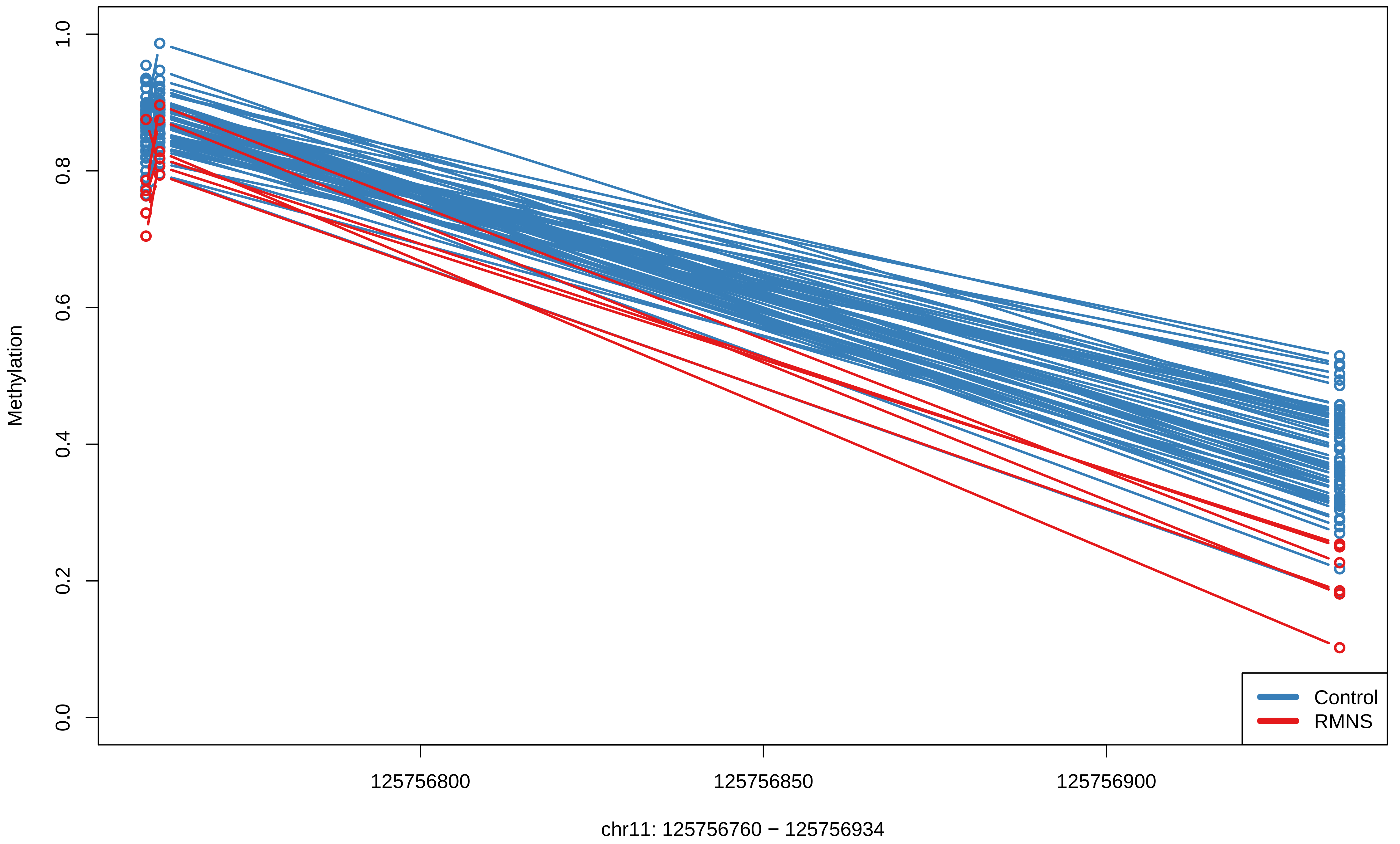

Methylation
0.0
0.2
0.4
0.6
0.8
1.0
125756800
125756850
125756900
chr11: 125756760 − 125756934
Control
RMNS

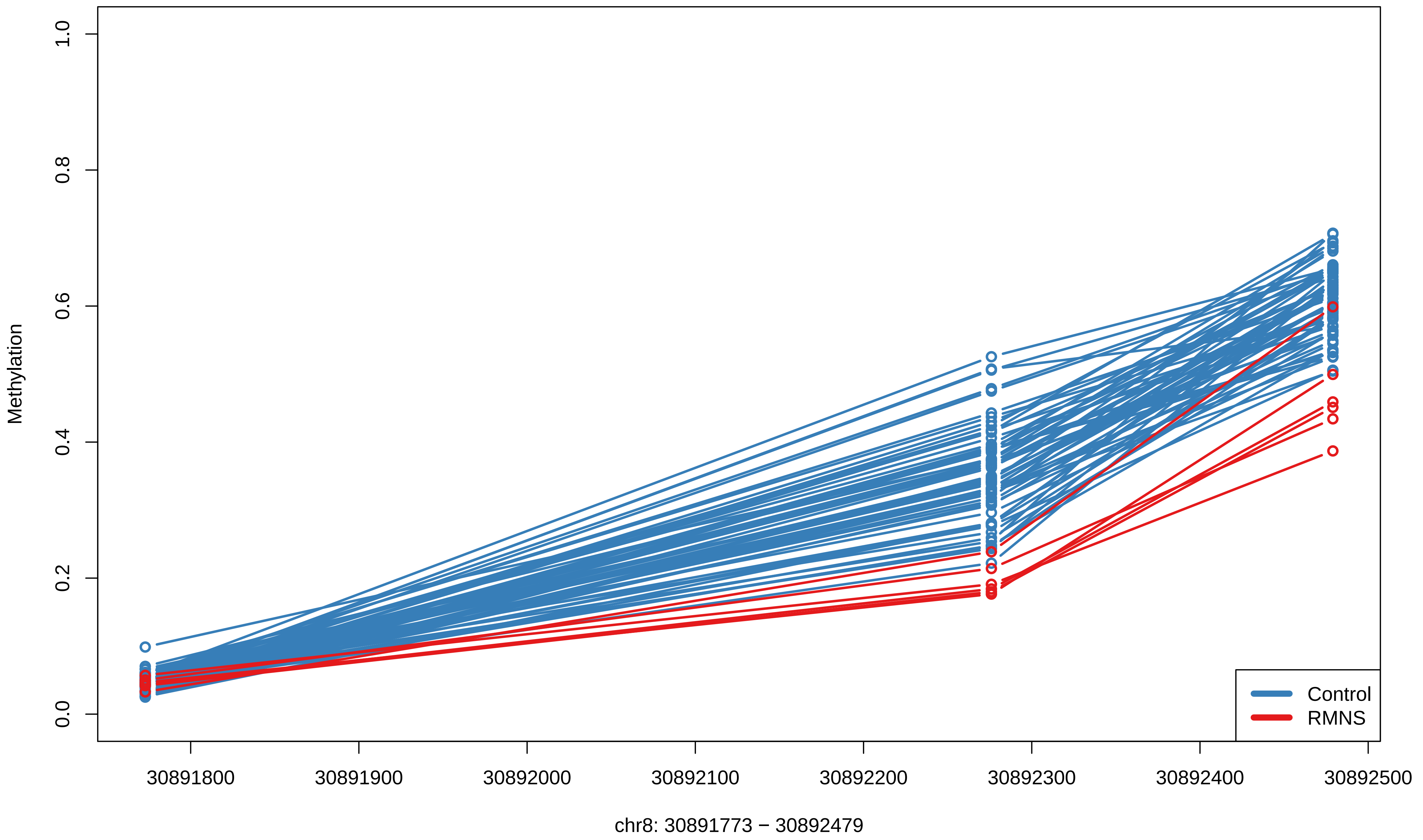

Methylation
0.0
0.2
0.4
0.6
0.8
1.0
30891800
30891900
30892000
30892100
30892200
30892300
30892400
30892500
chr8: 30891773 − 30892479
Control
RMNS

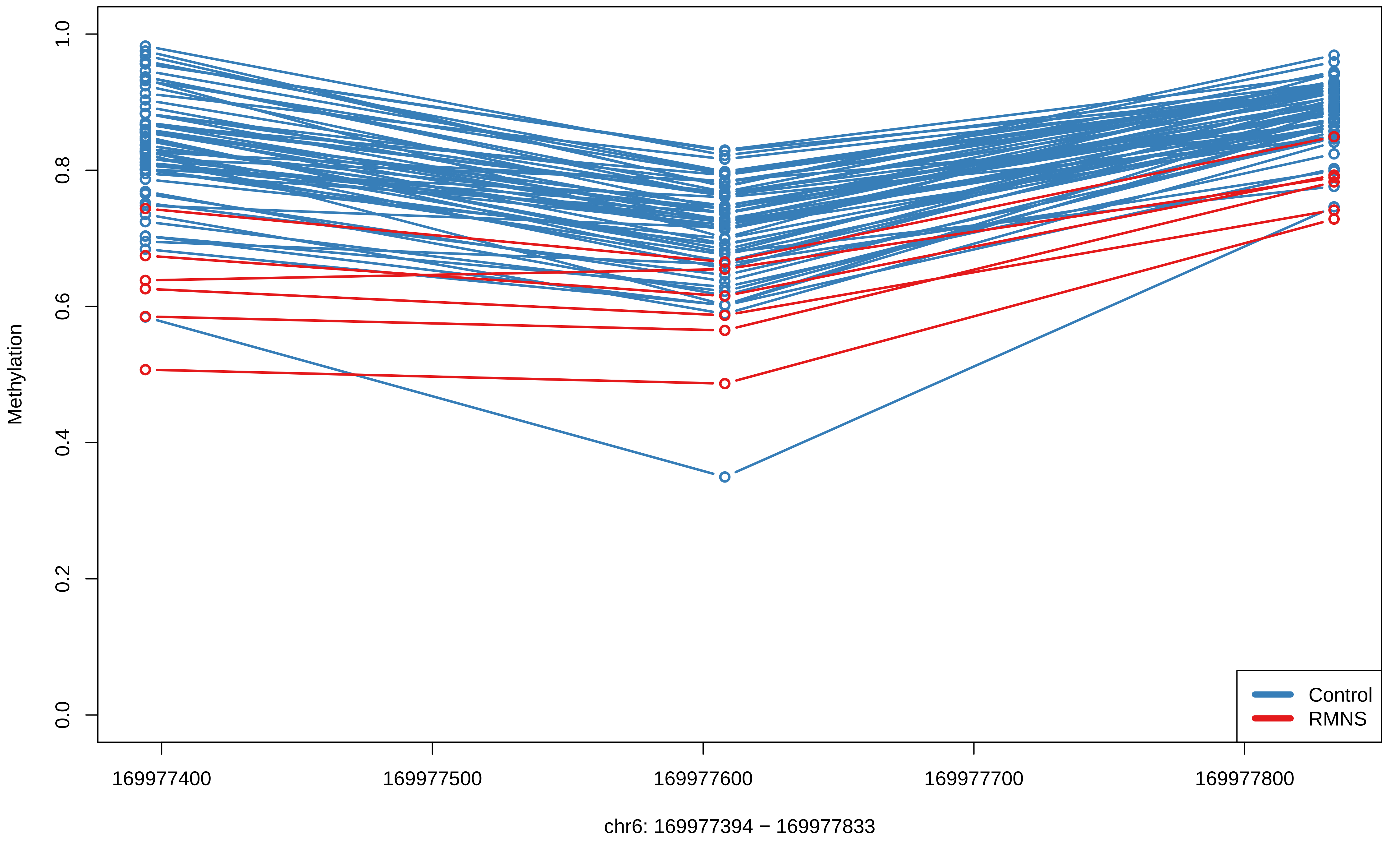

Methylation
0.0
0.2
0.4
0.6
0.8
1.0
169977400
169977500
169977600
169977700
169977800
chr6: 169977394 − 169977833
Control
RMNS

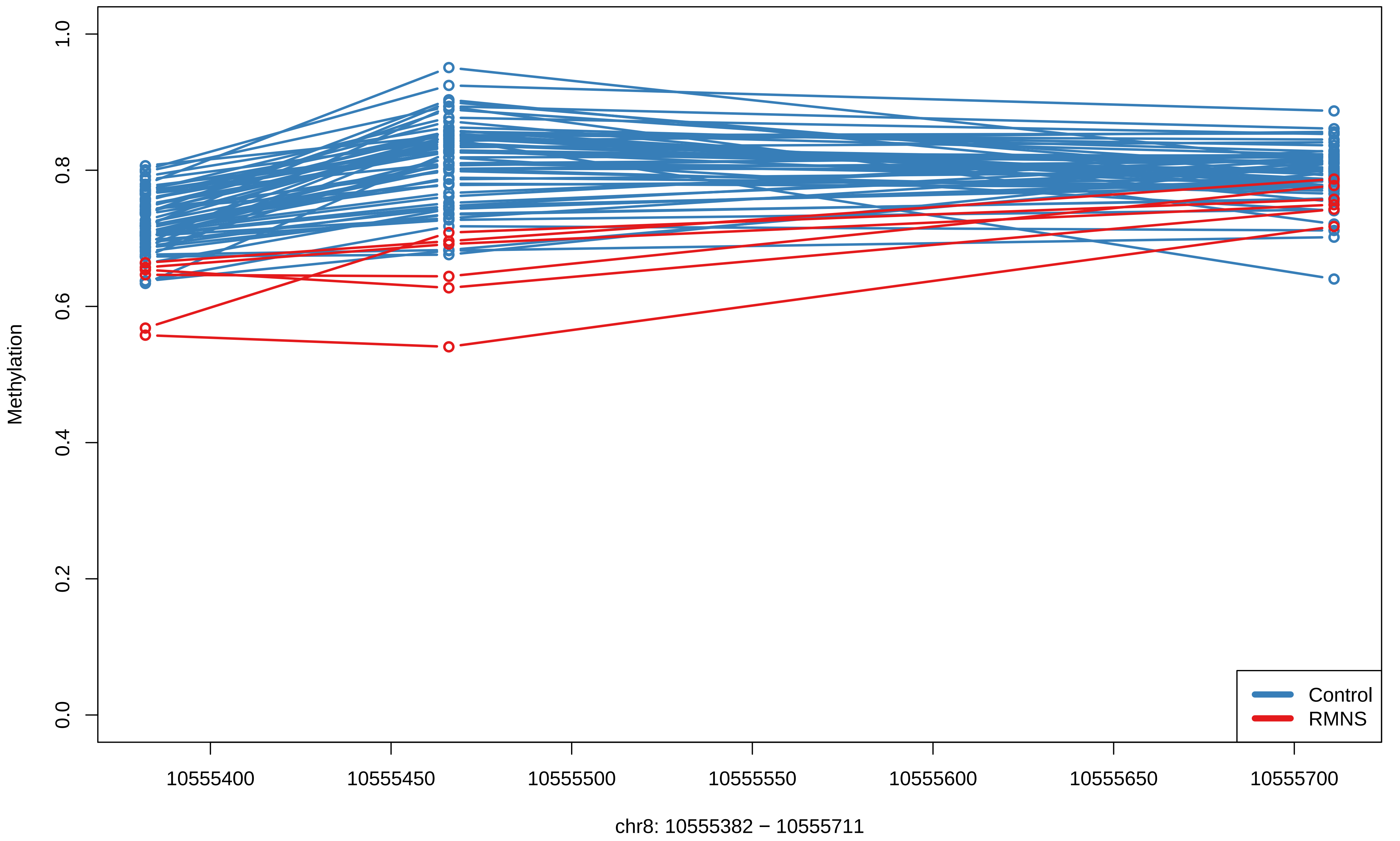

Methylation
0.0
0.2
0.4
0.6
0.8
1.0
10555400
10555450
10555500
10555550
10555600
10555650
10555700
chr8: 10555382 − 10555711
Control
RMNS

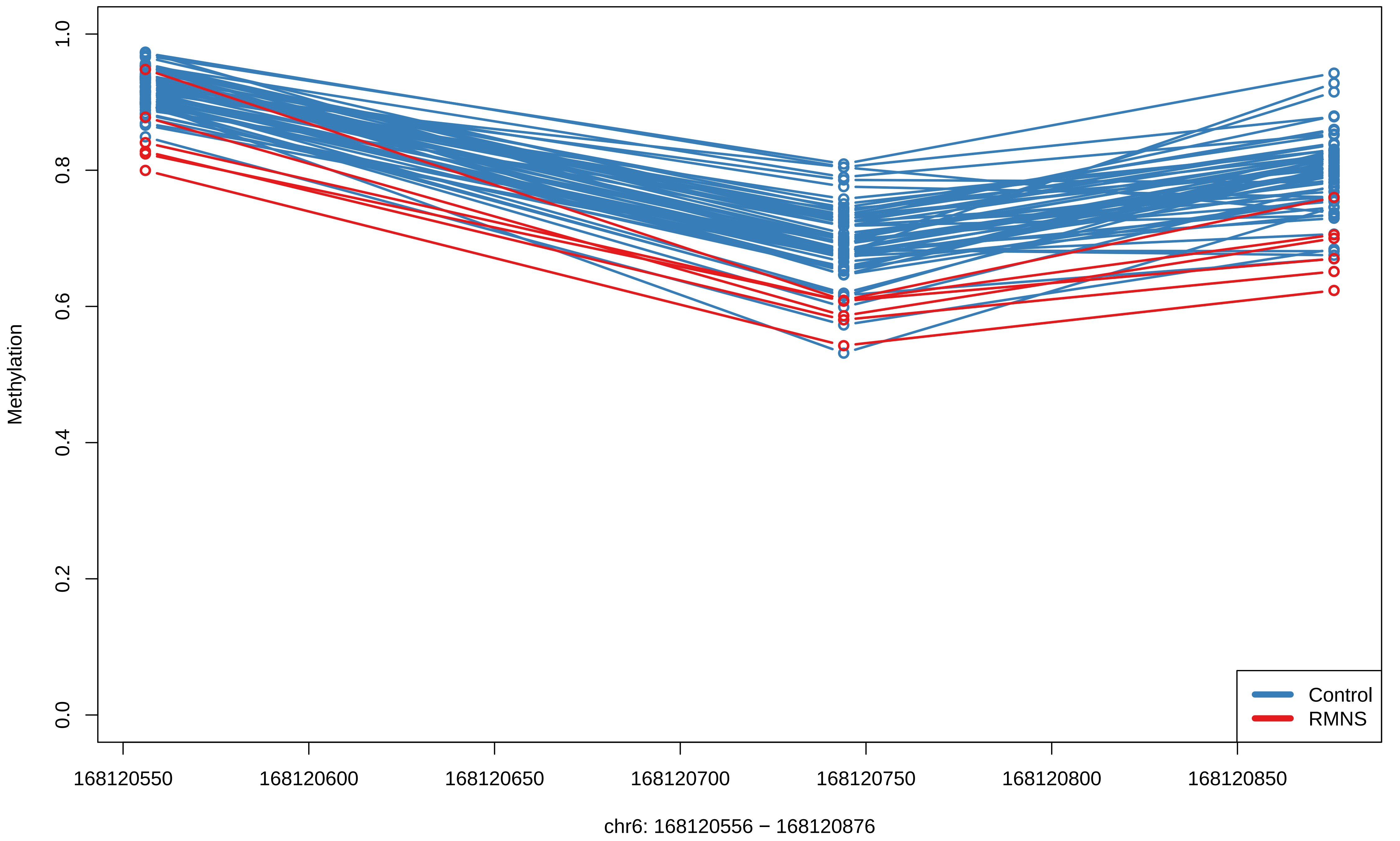

Methylation
1.0
0.8
0.6
0.4
0.2
0.0
168120550
168120600
168120650
168120700
168120750
168120800
168120850
chr6: 168120556 − 168120876
Control
RMNS

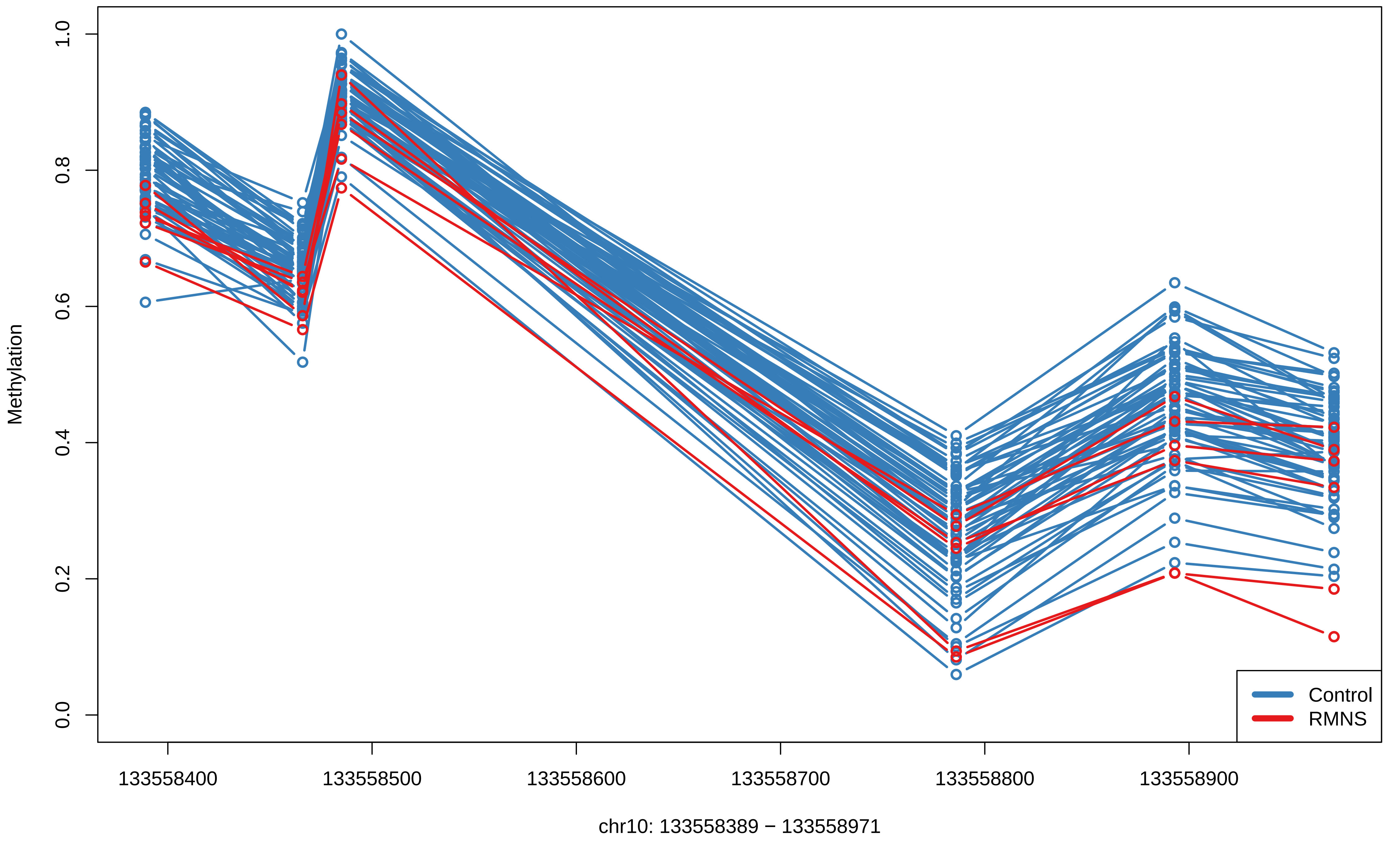

Methylation
0.0
0.2
0.4
0.6
0.8
1.0
133558400
133558500
133558600
133558700
133558800
133558900
chr10: 133558389 − 133558971
Control
RMNS

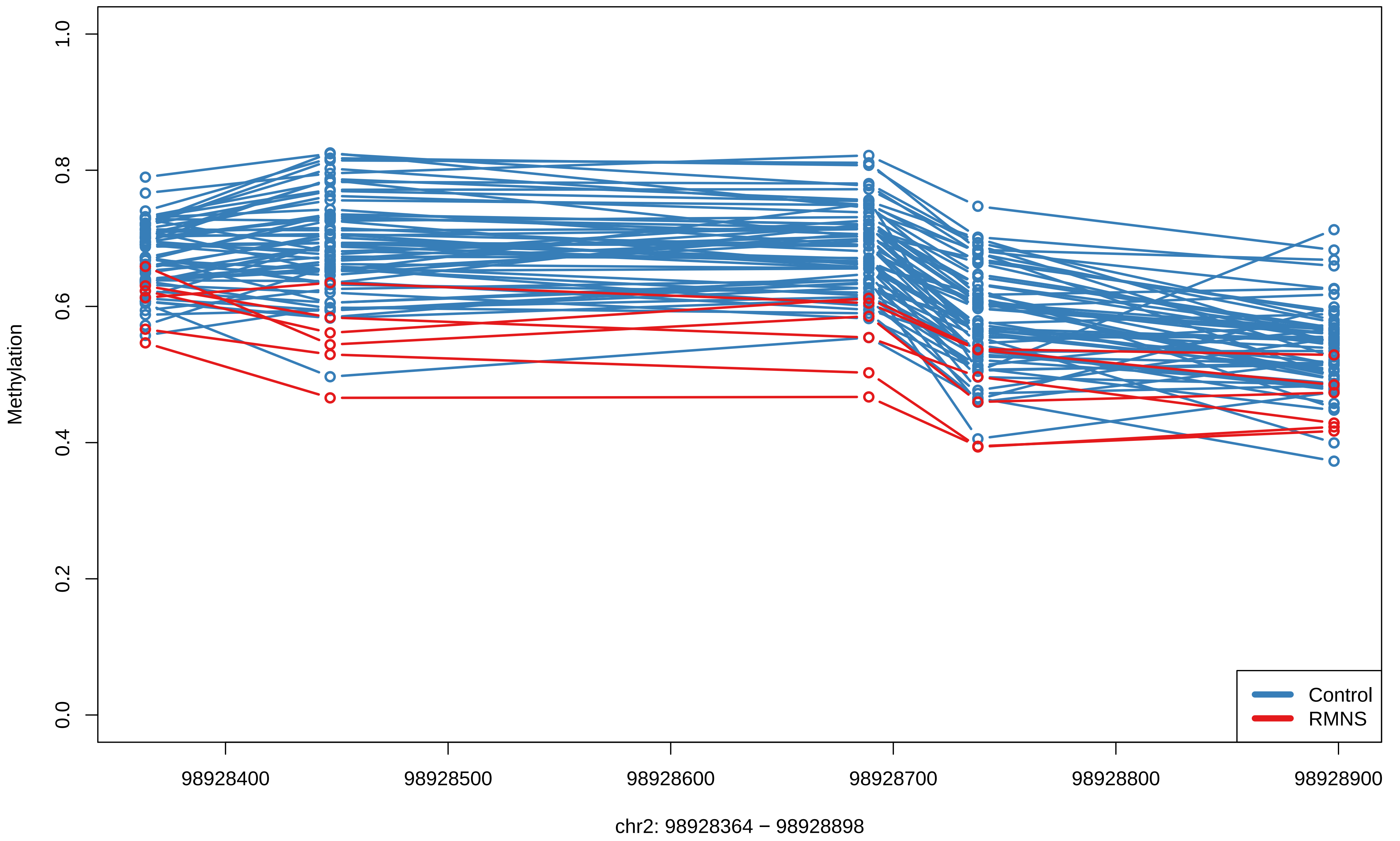

Methylation
0.0
0.2
0.4
0.6
0.8
1.0
98928400
98928500
98928600
98928700
98928800
98928900
chr2: 98928364 − 98928898
Control
RMNS

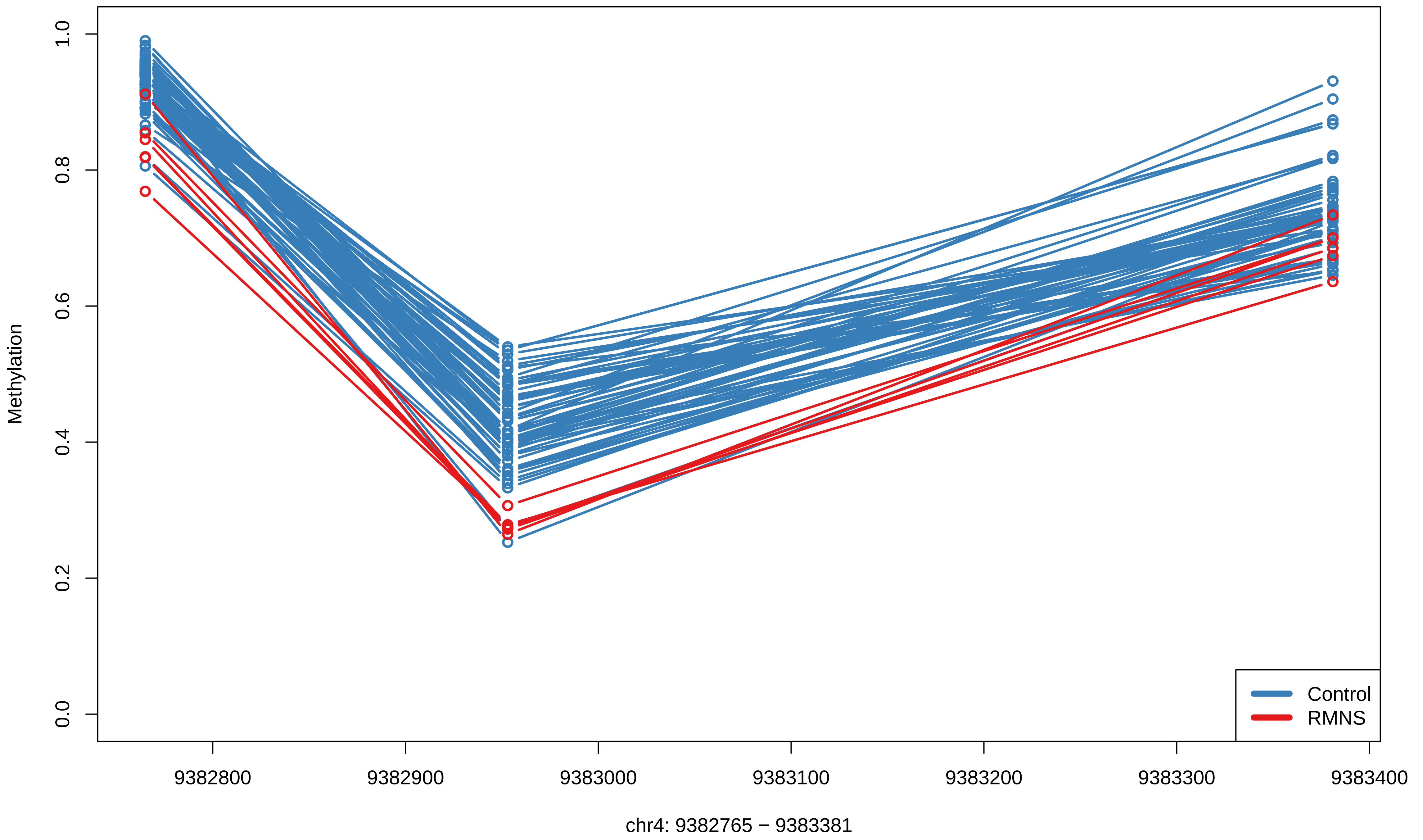

1.0
0.8
0.6
0.4
0.2
0.0
Methylation
9382800
9382900
9383000
9383100
9383200
9383300
9383400
chr4: 9382765 − 9383381
Control
RMNS

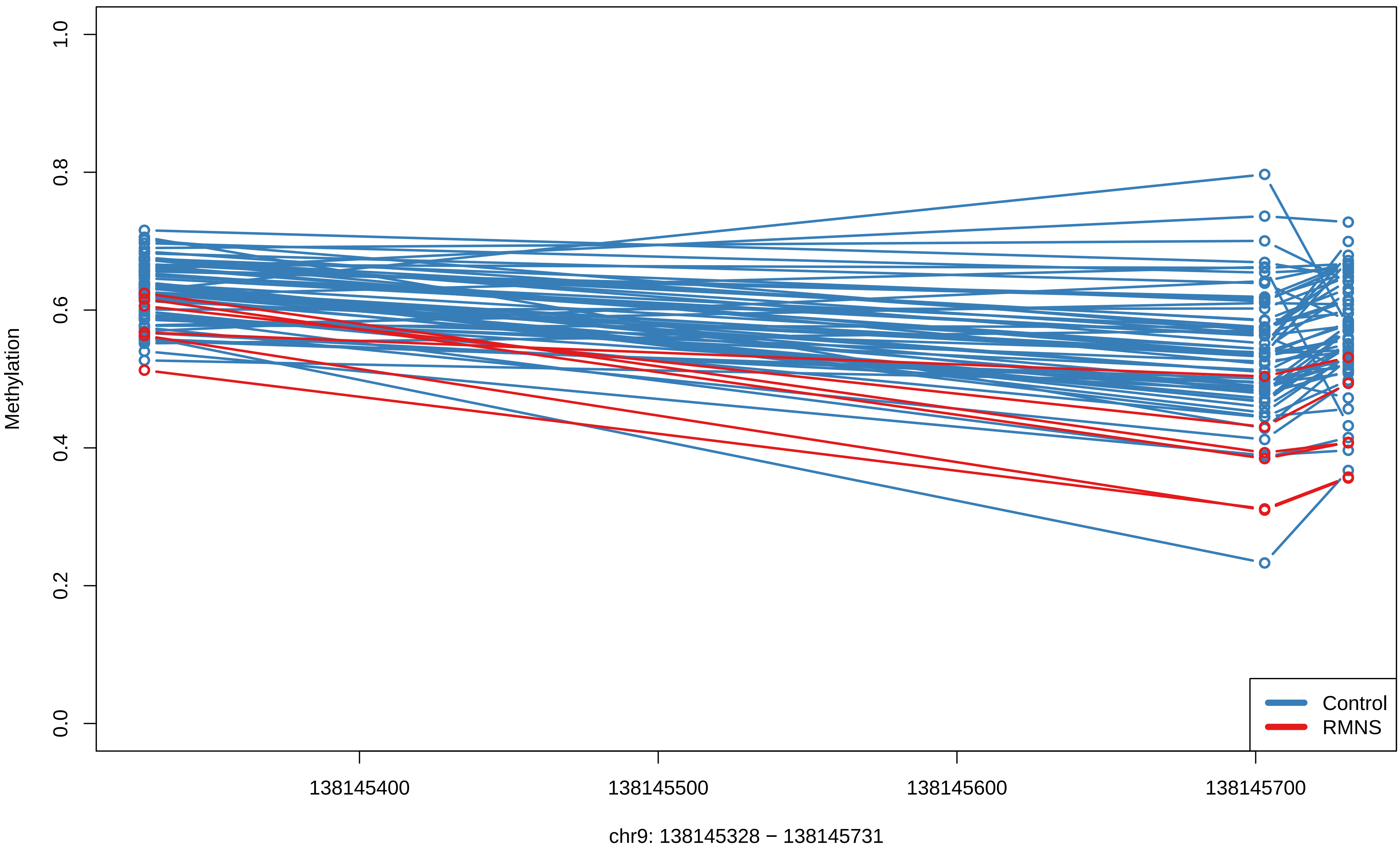

Methylation
0.0
0.2
0.4
0.6
0.8
1.0
138145400
138145500
138145600
138145700
chr9: 138145328 − 138145731
Control
RMNS

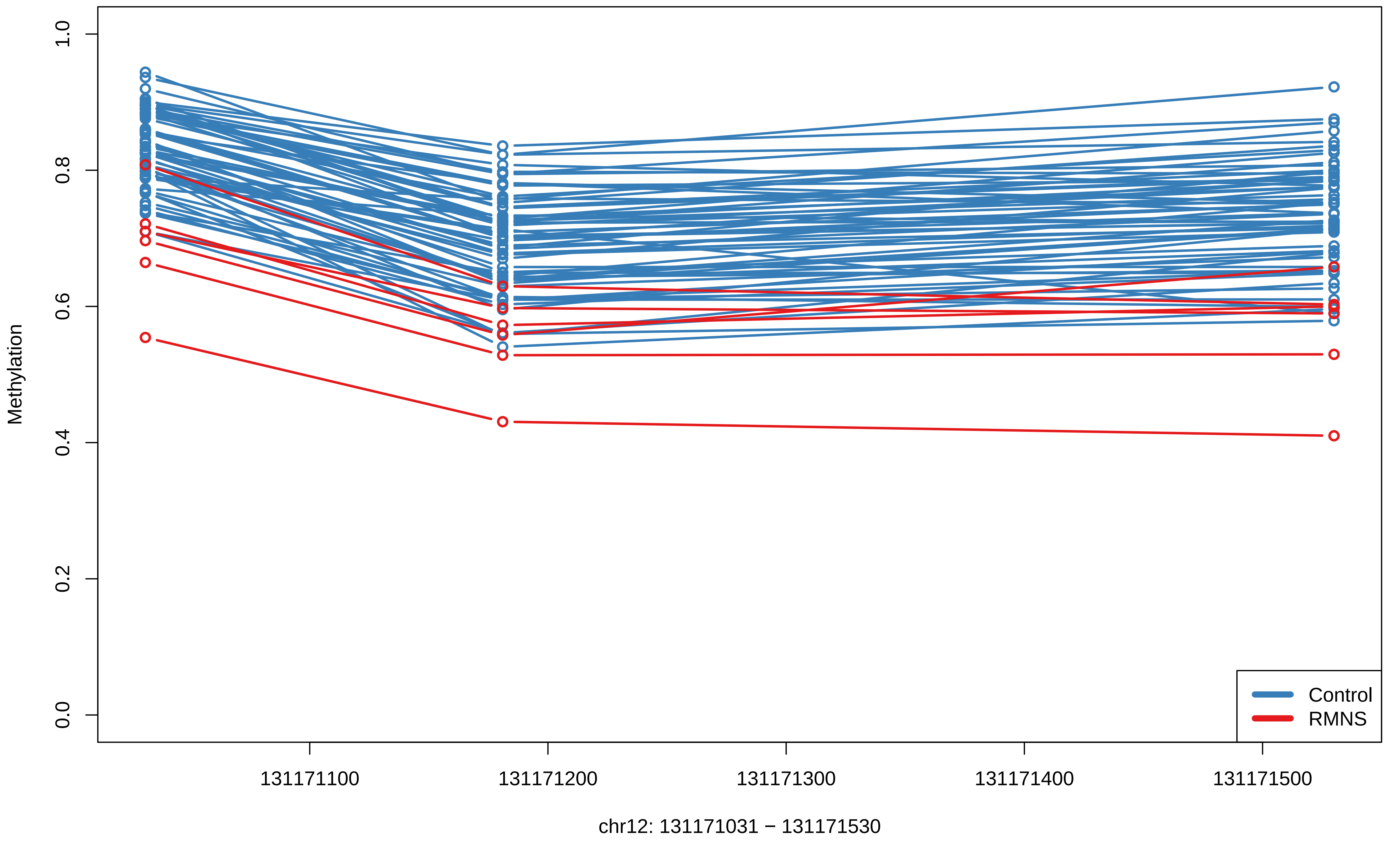

Methylation
0.0
0.2
0.4
0.6
0.8
1.0
131171100
131171200
131171300
131171400
131171500
chr12: 131171031 − 131171530
Control
RMNS

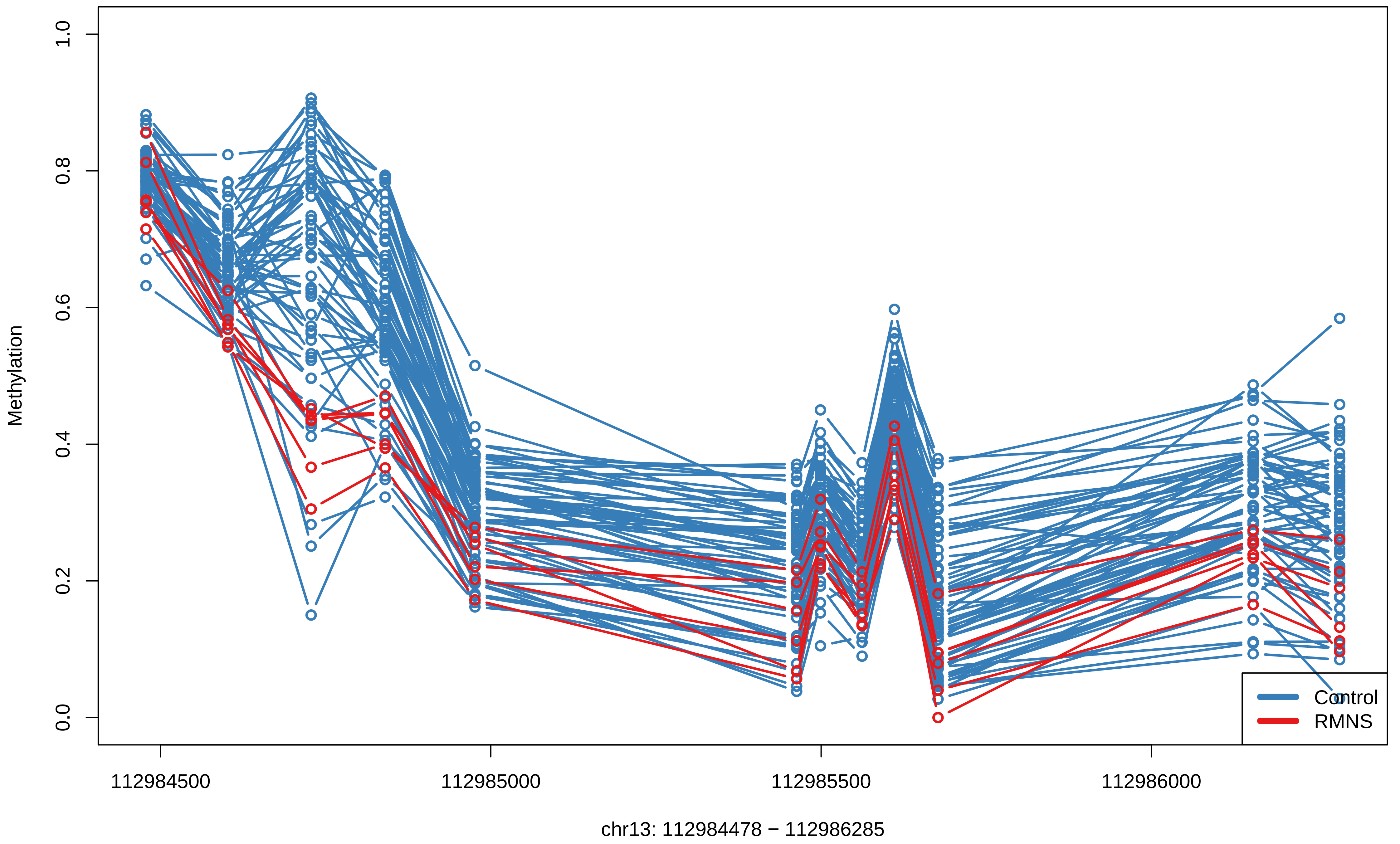

1.0
0.8
0.6
0.4
0.2
0.0
Methylation
112984500
112985000
112985500
112986000
chr13: 112984478 − 112986285
Control
RMNS

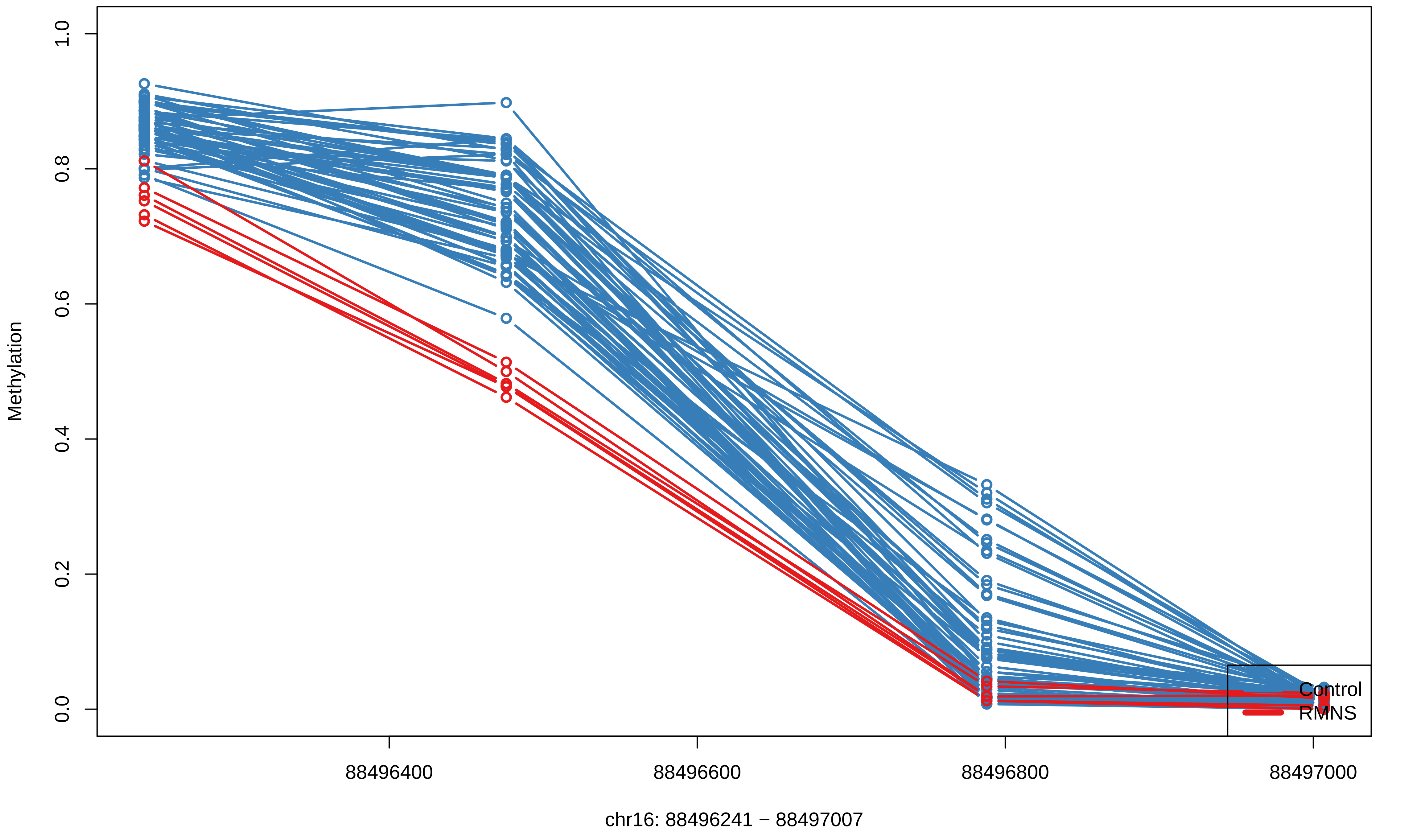

Methylation
1.0
0.8
0.6
0.4
0.2
0.0
88496400
88496600
88496800
88497000
chr16: 88496241 − 88497007
Control
RMNS

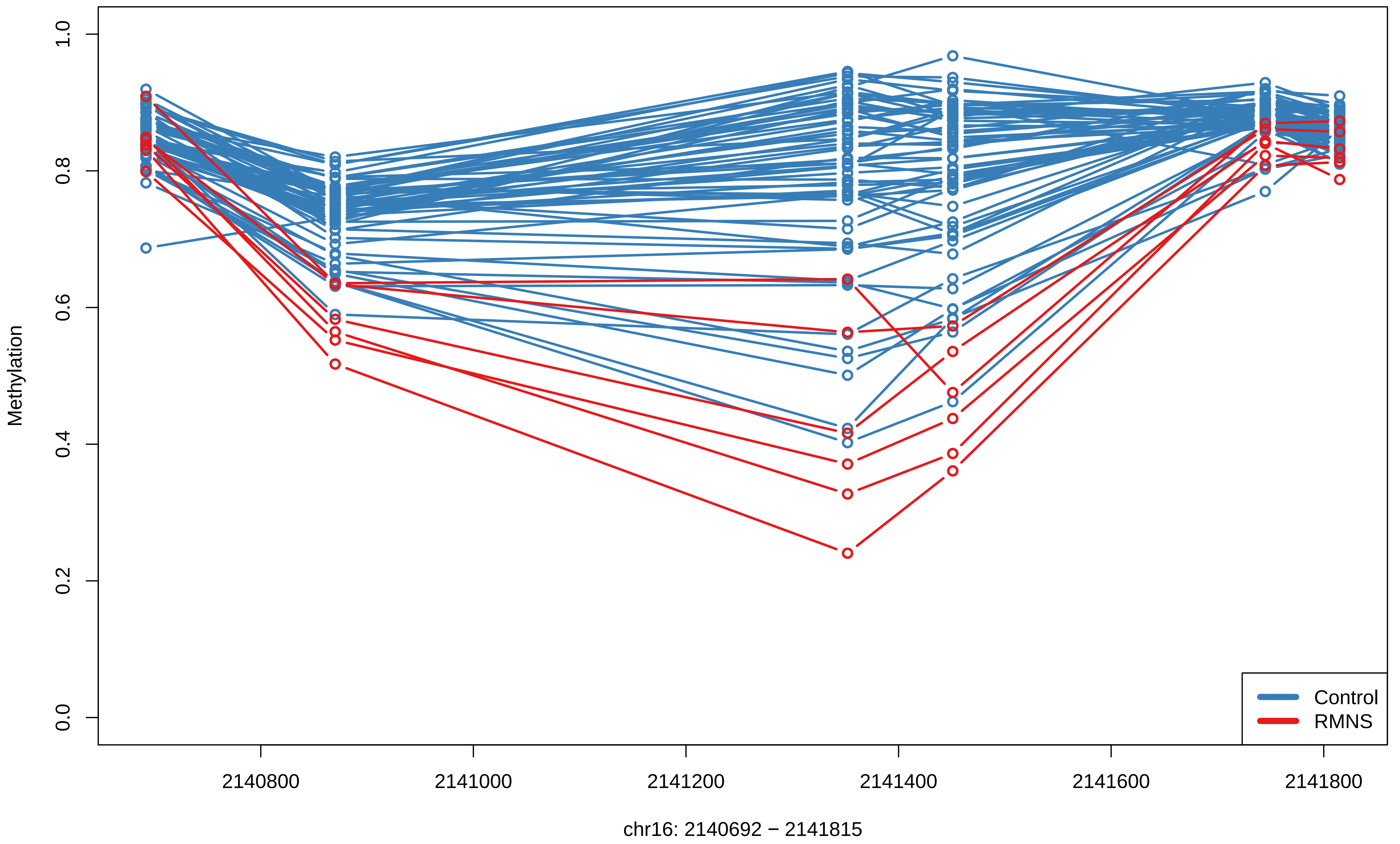

Methylation
0.0
0.2
0.4
0.6
0.8
1.0
2140800
2141000
2141200
2141400
2141600
2141800
chr16: 2140692 − 2141815
Control
RMNS

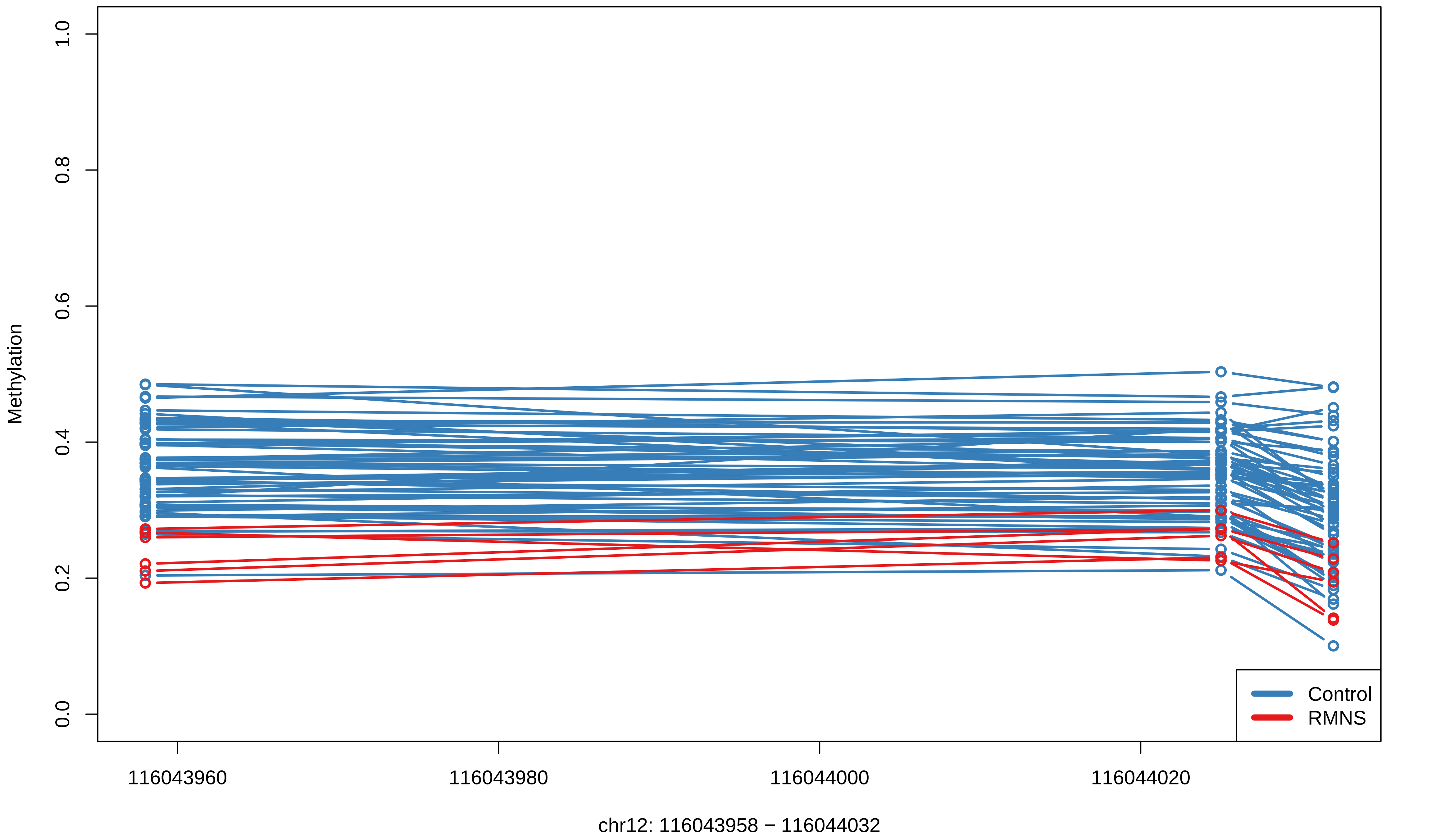

Methylation
1.0
0.8
0.6
0.4
0.2
0.0
116043960
116043980
116044000
116044020
chr12: 116043958 − 116044032
Control
RMNS

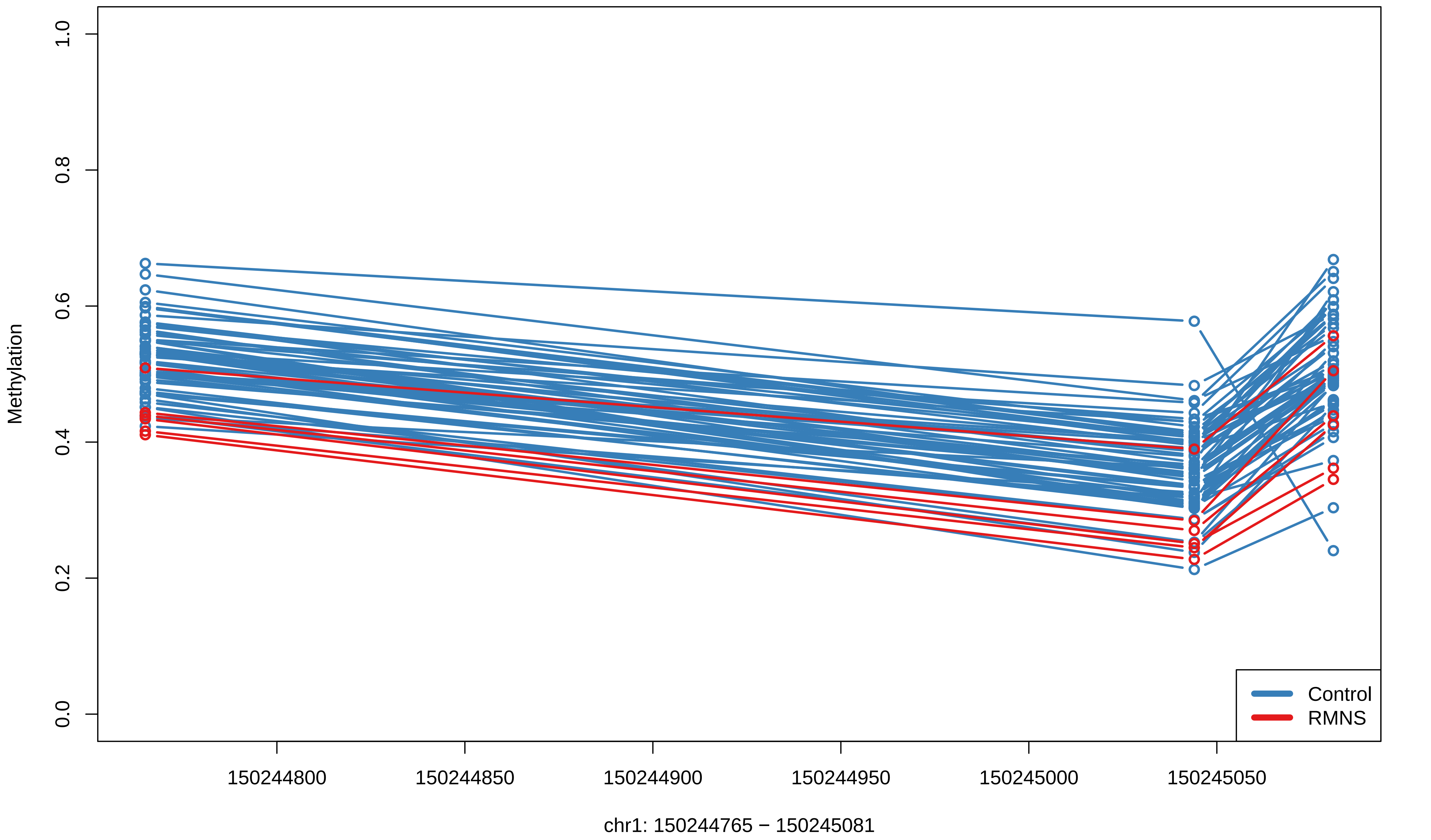

Methylation
0.0
0.2
0.4
0.6
0.8
1.0
150244800
150244850
150244900
150244950
150245000
150245050
chr1: 150244765 − 150245081
Control
RMNS

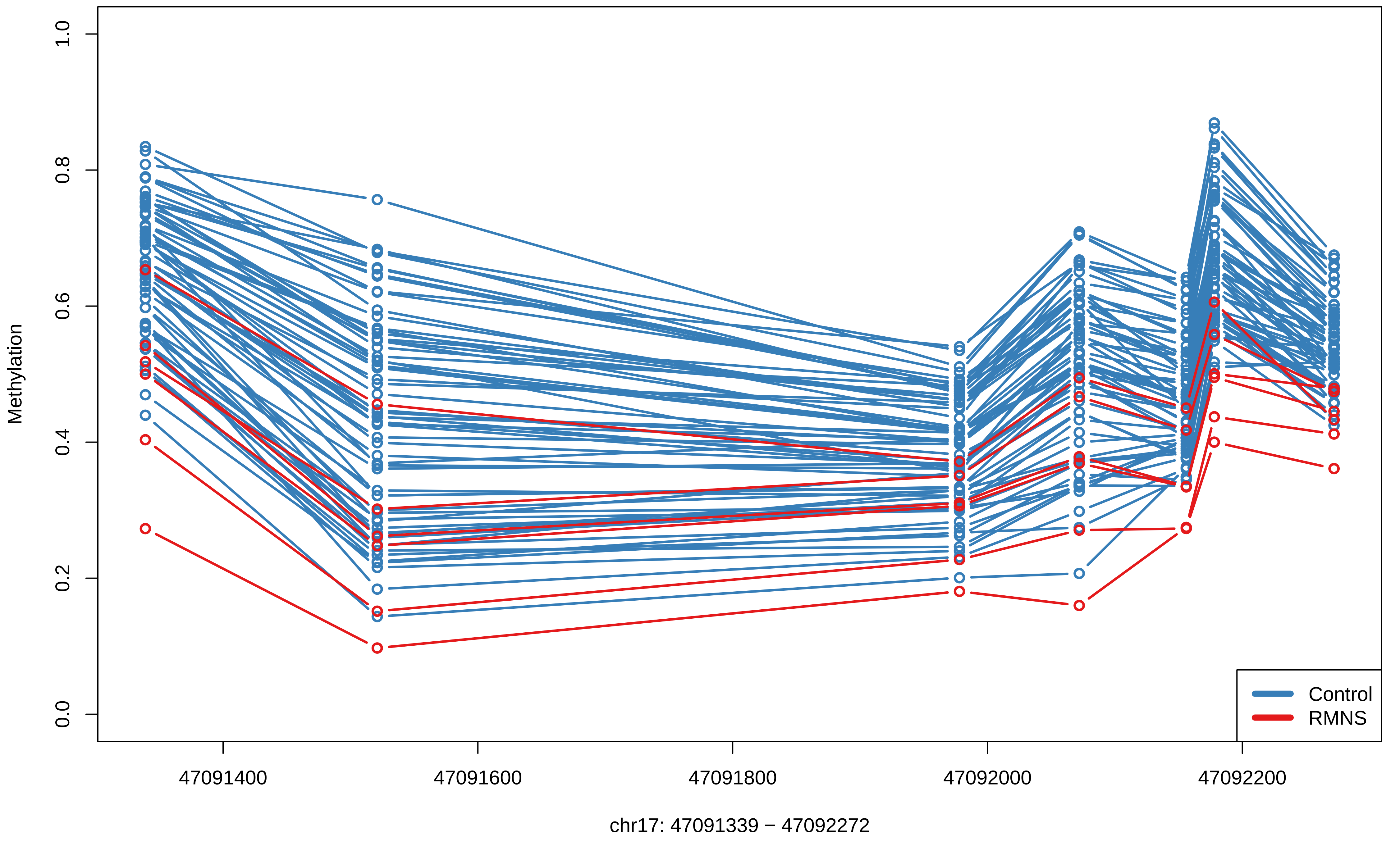
Methylation
0.0
0.2
0.4
0.6
0.8
1.0
47091400
47091600
47091800
47092000
47092200
chr17: 47091339 − 47092272
Control
RMNS

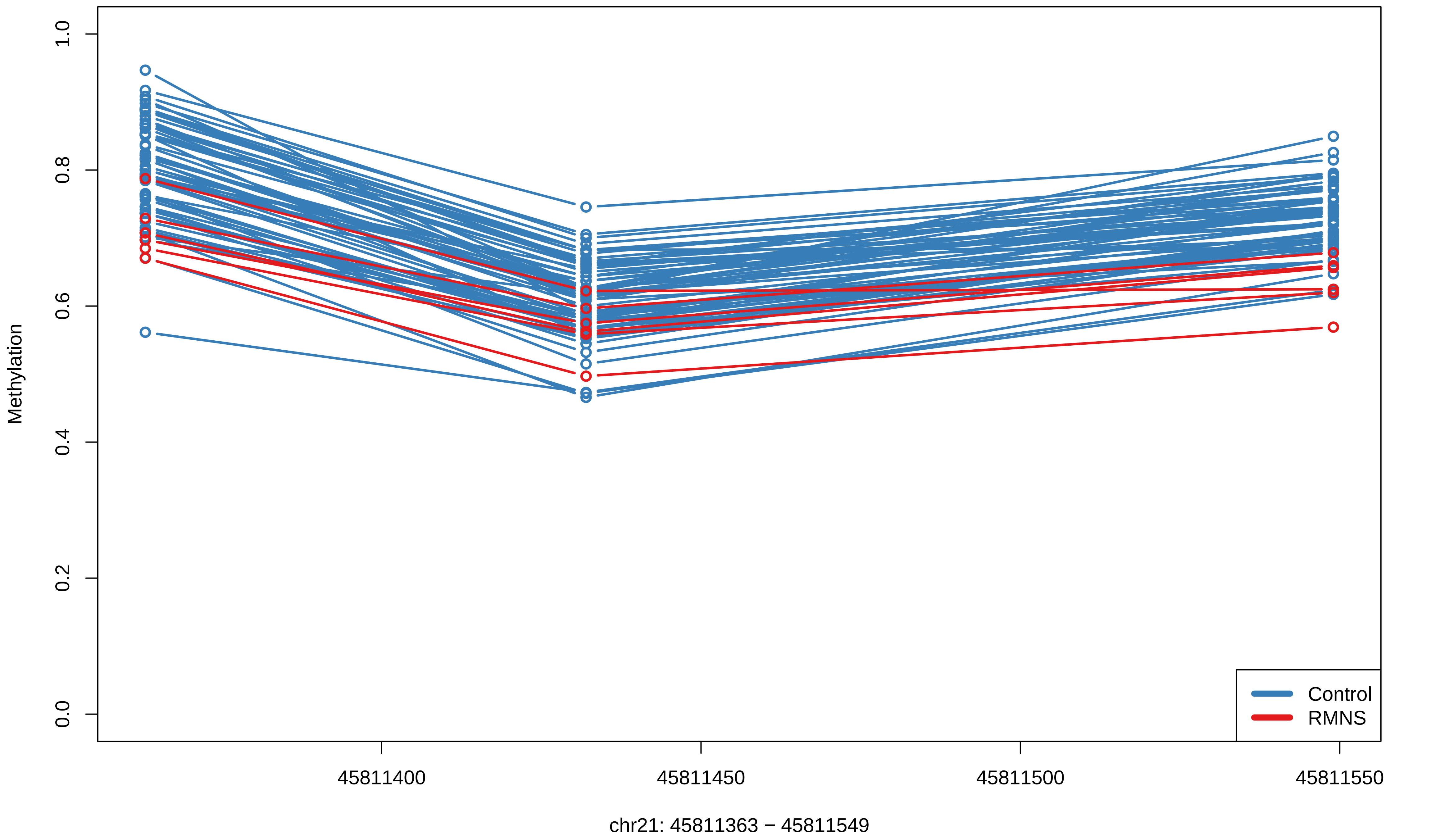

Methylation
0.0
0.2
0.4
0.6
0.8
1.0
45811400
45811450
45811500
45811550
chr21: 45811363 − 45811549
Control
RMNS

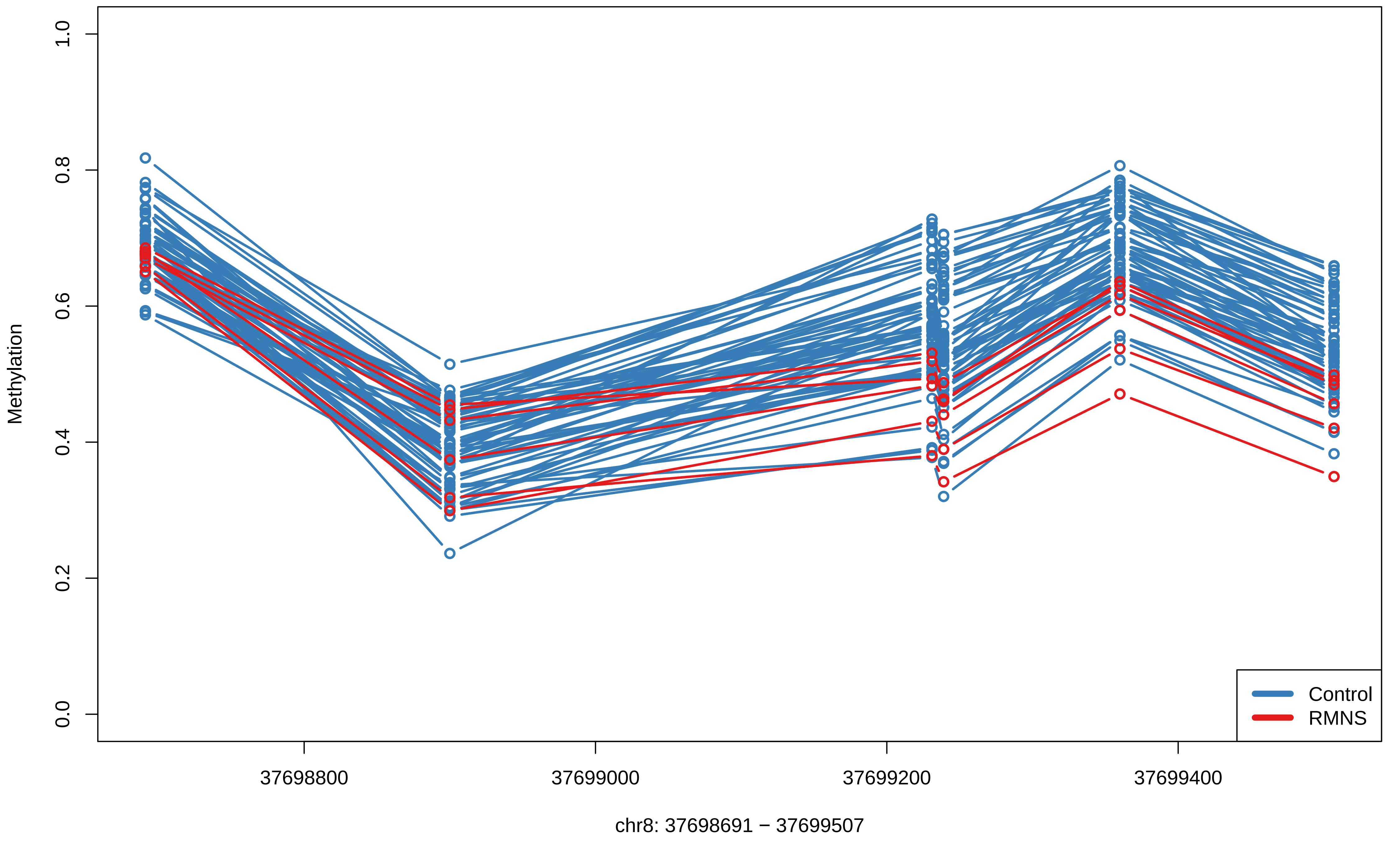
Methylation
0.0
0.2
0.4
0.6
0.8
1.0
37698800
37699000
37699200
37699400
chr8: 37698691 − 37699507
Control
RMNS

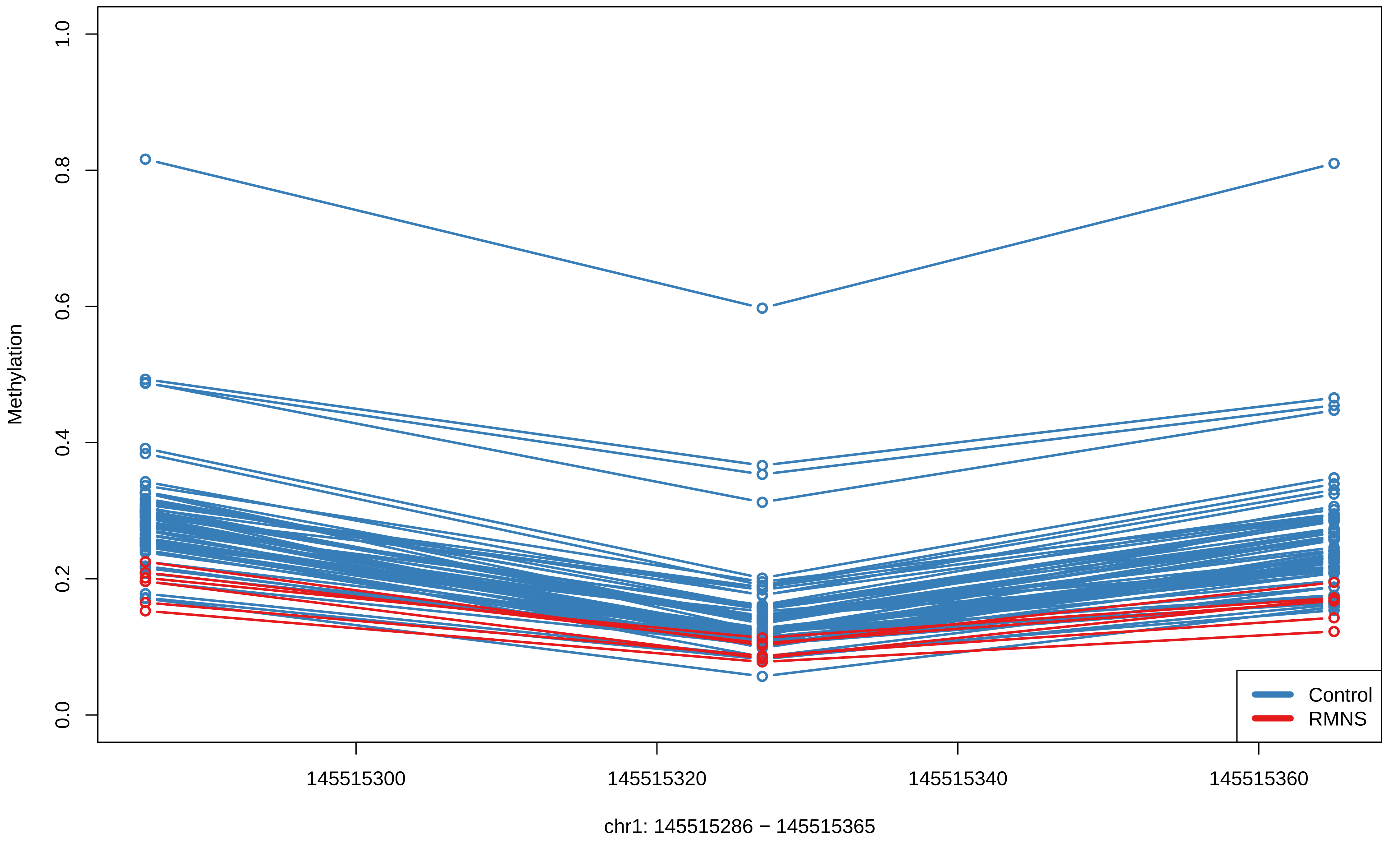

Methylation
1.0
0.8
0.6
0.4
0.2
0.0
145515300
145515320
145515340
145515360
chr1: 145515286 − 145515365
Control
RMNS

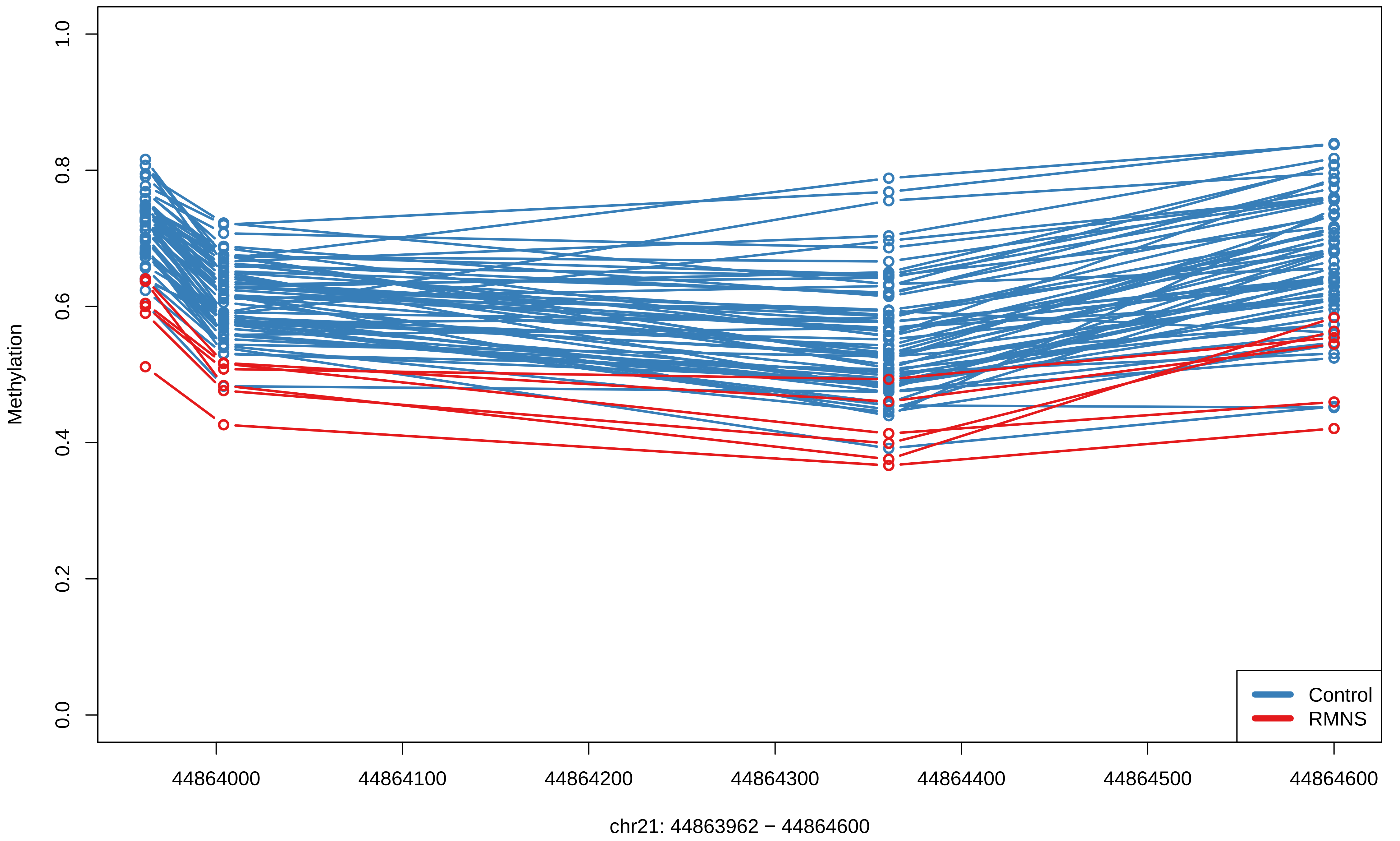

Methylation
0.0
0.2
0.4
0.6
0.8
1.0
44864000
44864100
44864200
44864300
44864400
44864500
44864600
chr21: 44863962 − 44864600
Control
RMNS

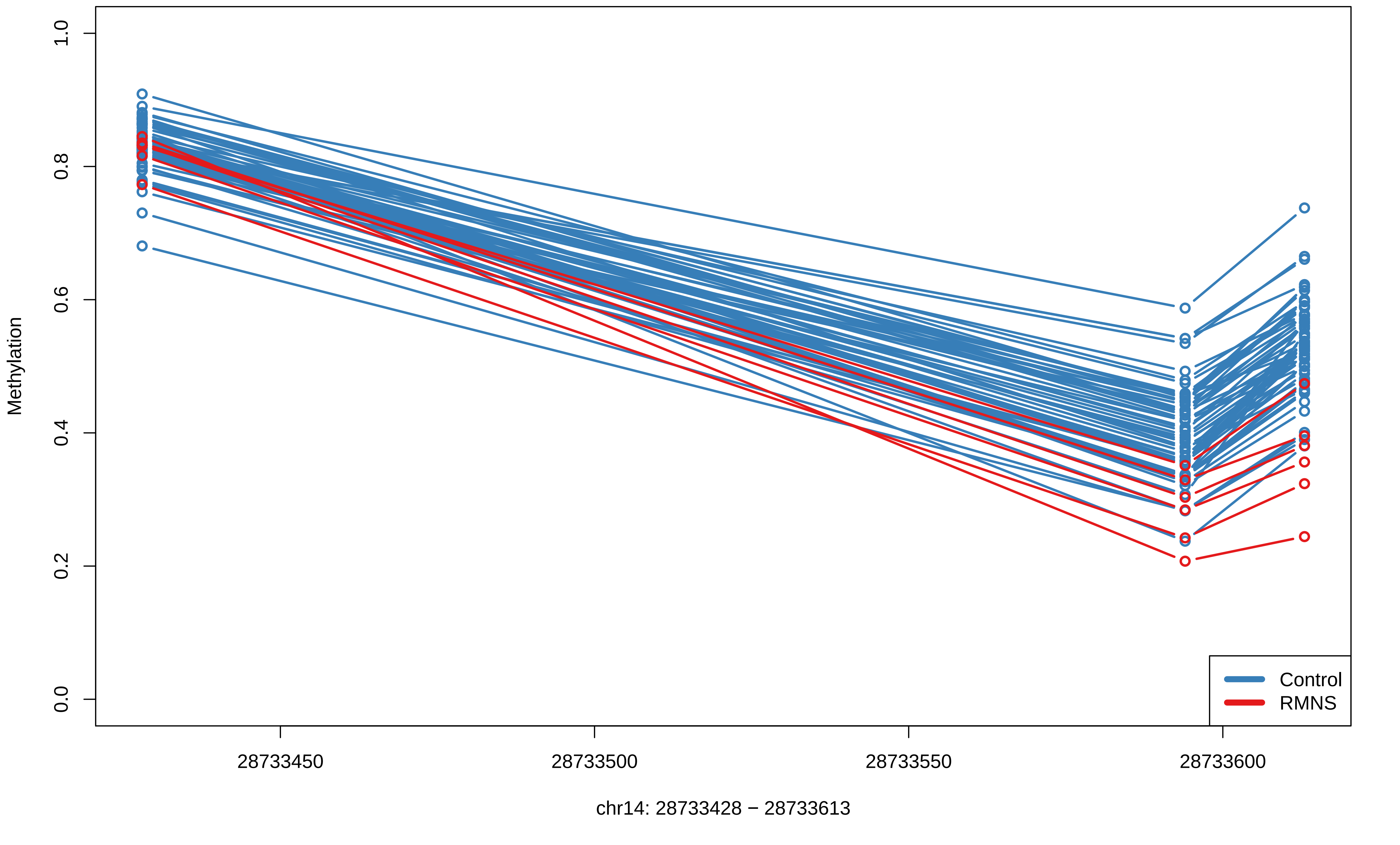

Methylation
0.0
0.2
0.4
0.6
0.8
1.0
28733450
28733500
28733550
28733600
chr14: 28733428 − 28733613
Control
RMNS

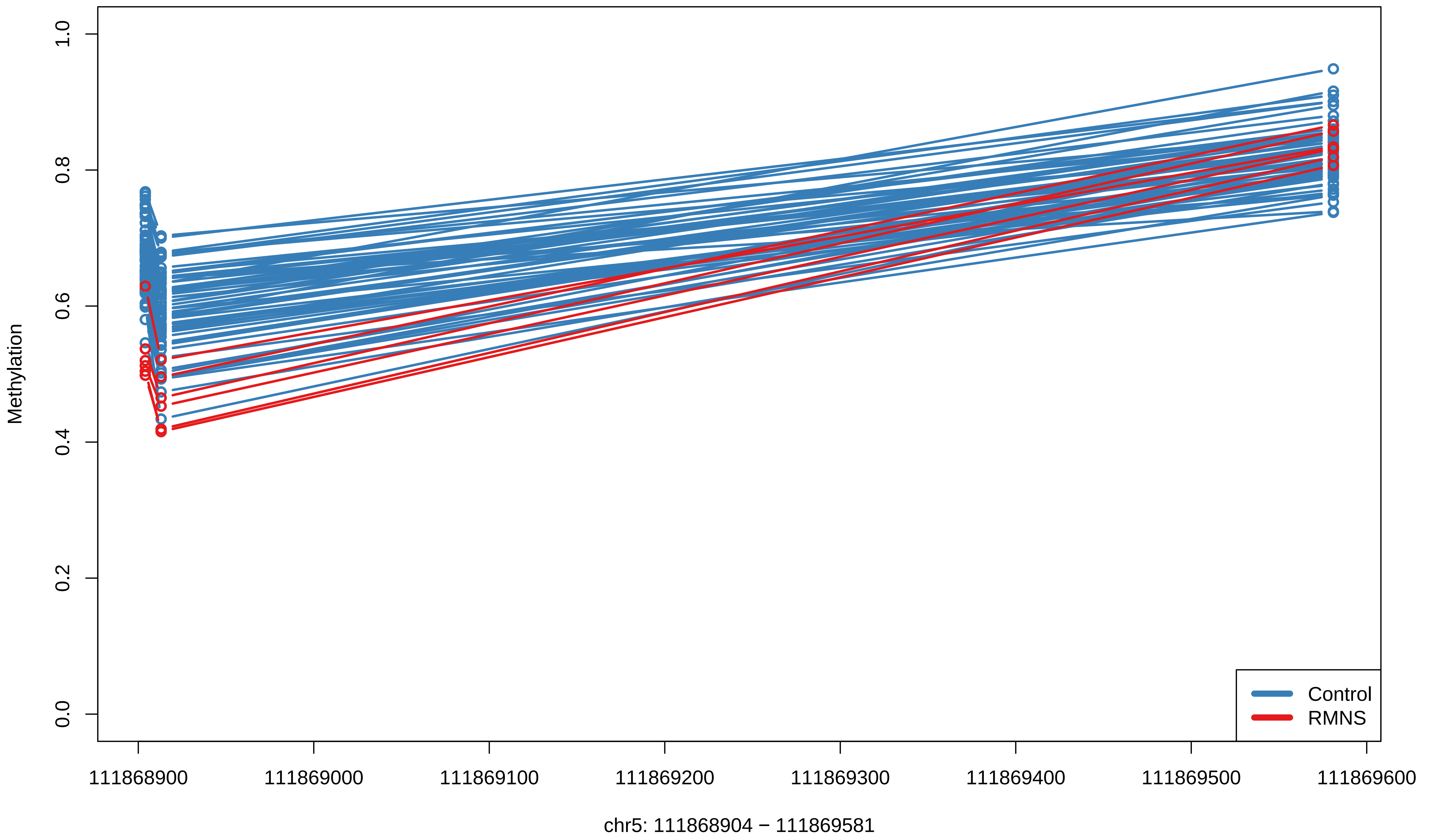

Methylation
0.0
0.2
0.4
0.6
0.8
1.0
111868900
111869000
111869100
111869200
111869300
111869400
111869500
111869600
chr5: 111868904 − 111869581
Control
RMNS

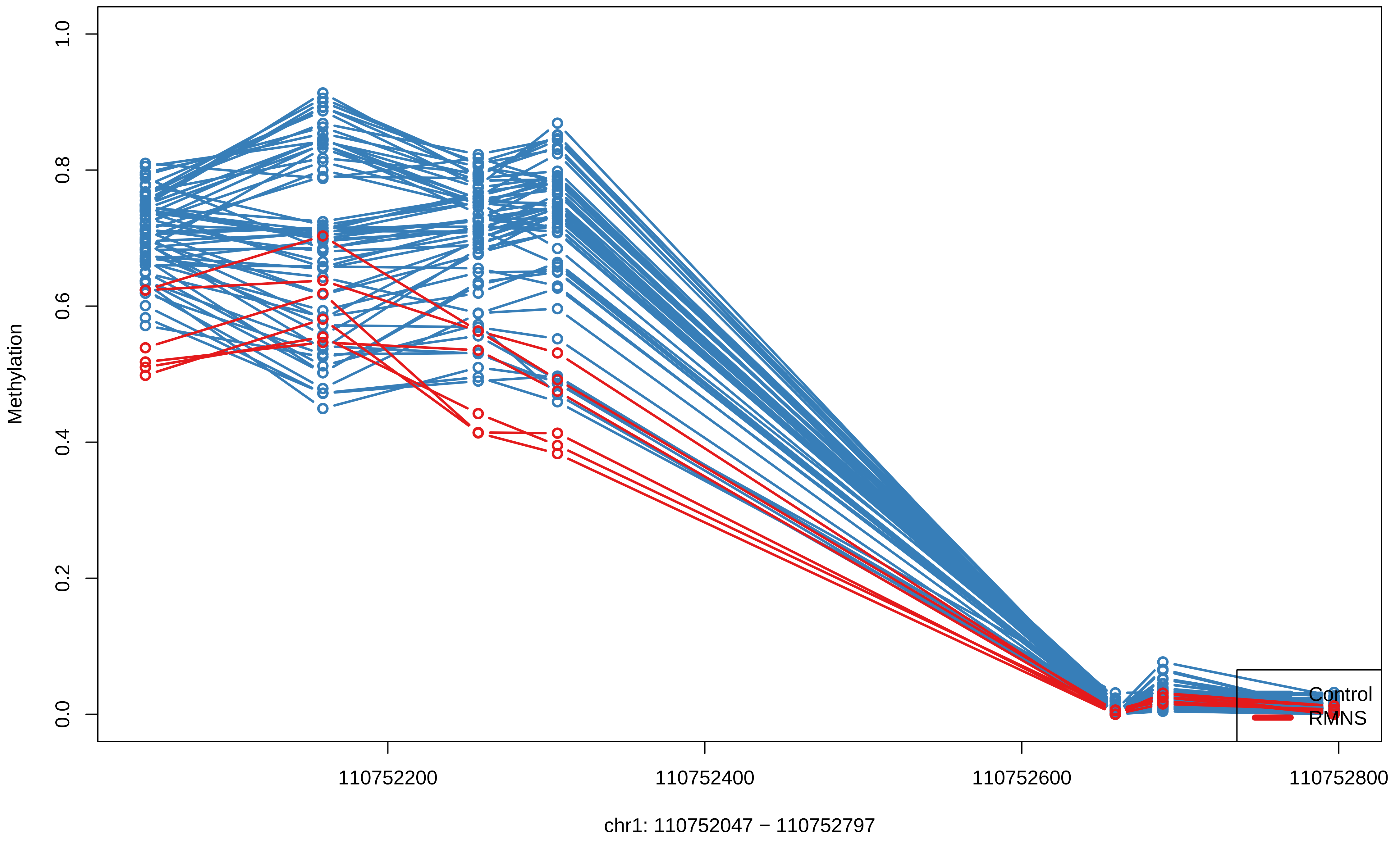

1.0
0.8
0.6
0.4
0.2
0.0
Methylation
110752200
110752400
110752600
110752800
chr1: 110752047 − 110752797
Control
RMNS

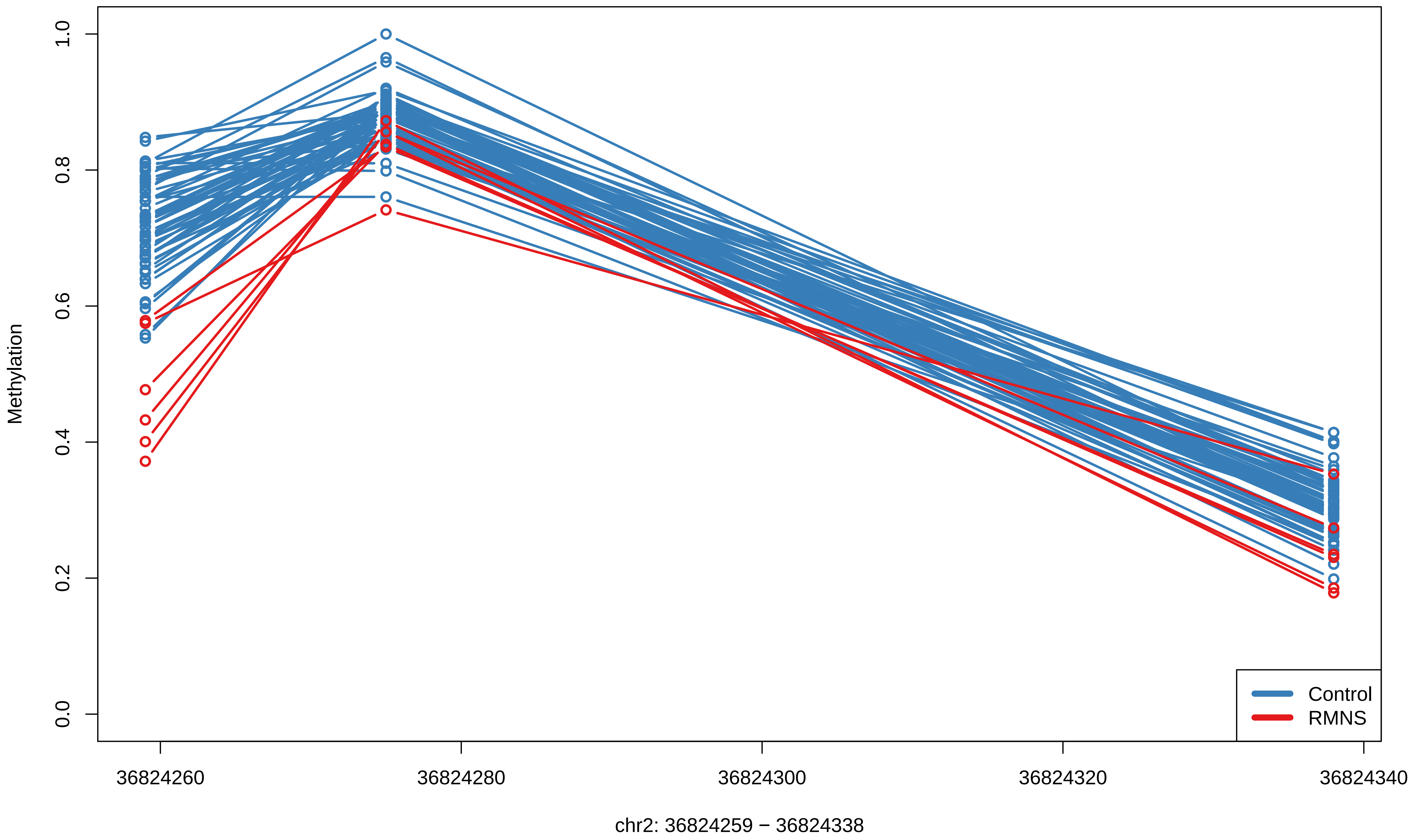
Methylation
0.0
0.2
0.4
0.6
0.8
1.0
36824260
36824280
36824300
36824320
36824340
chr2: 36824259 − 36824338
Control
RMNS

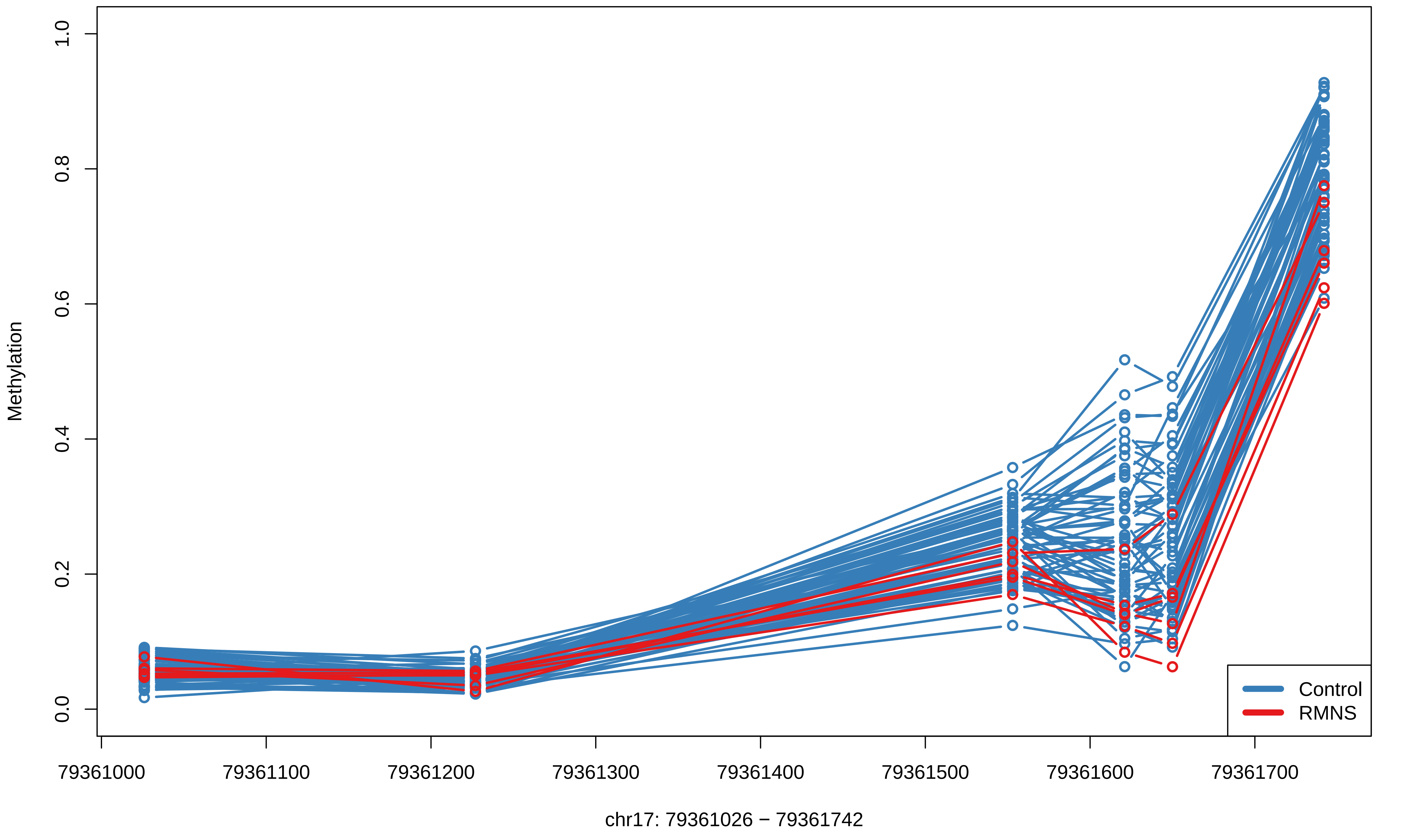

Methylation
0.0
0.2
0.4
0.6
0.8
1.0
79361000
79361100
79361200
79361300
79361400
79361500
79361600
79361700
chr17: 79361026 − 79361742
Control
RMNS

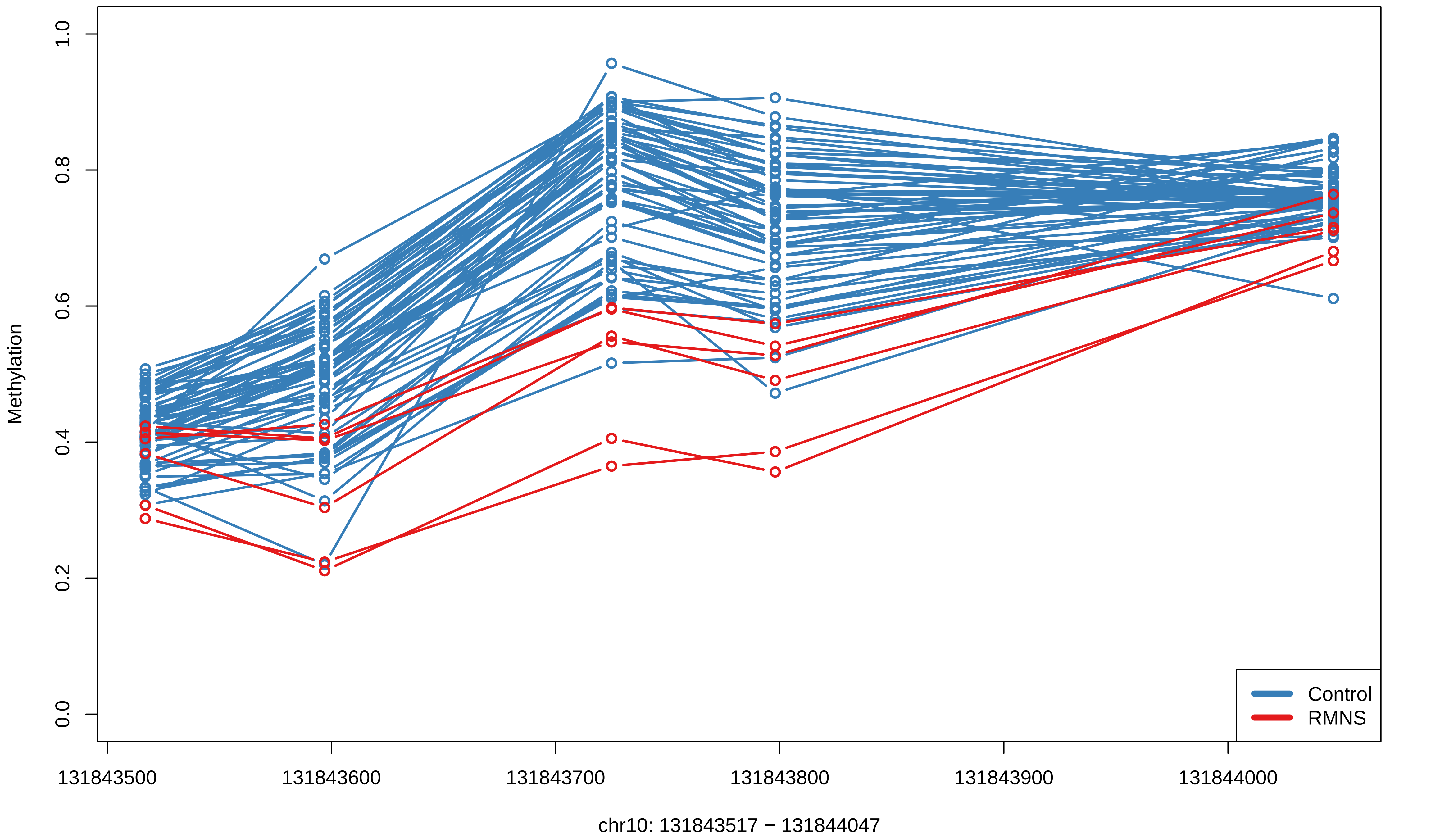

Methylation
1.0
0.8
0.6
0.4
0.2
0.0
131843500
131843600
131843700
131843800
131843900
131844000
chr10: 131843517 − 131844047
Control
RMNS

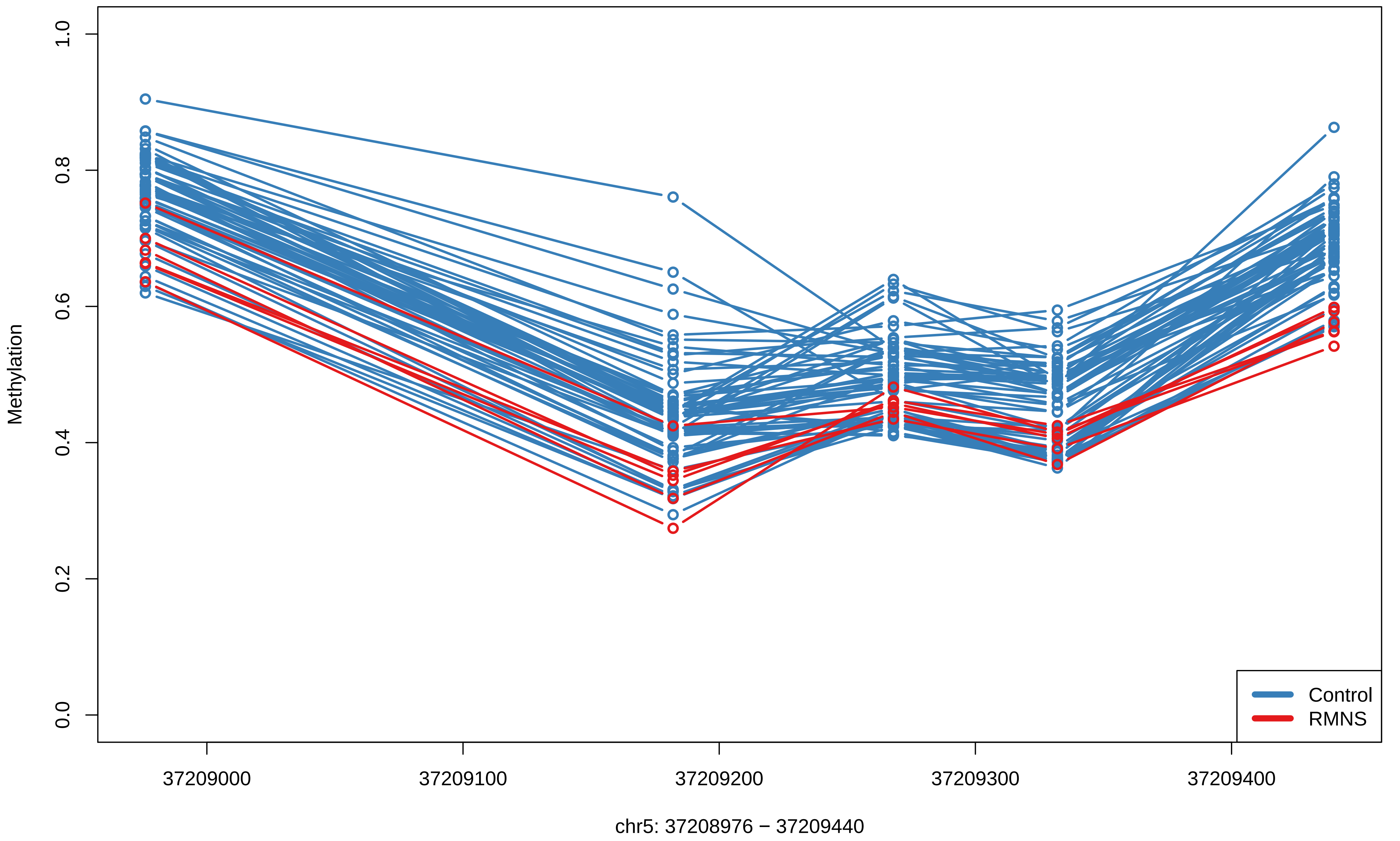

Methylation
0.0
0.2
0.4
0.6
0.8
1.0
37209000
37209100
37209200
37209300
37209400
chr5: 37208976 − 37209440
Control
RMNS

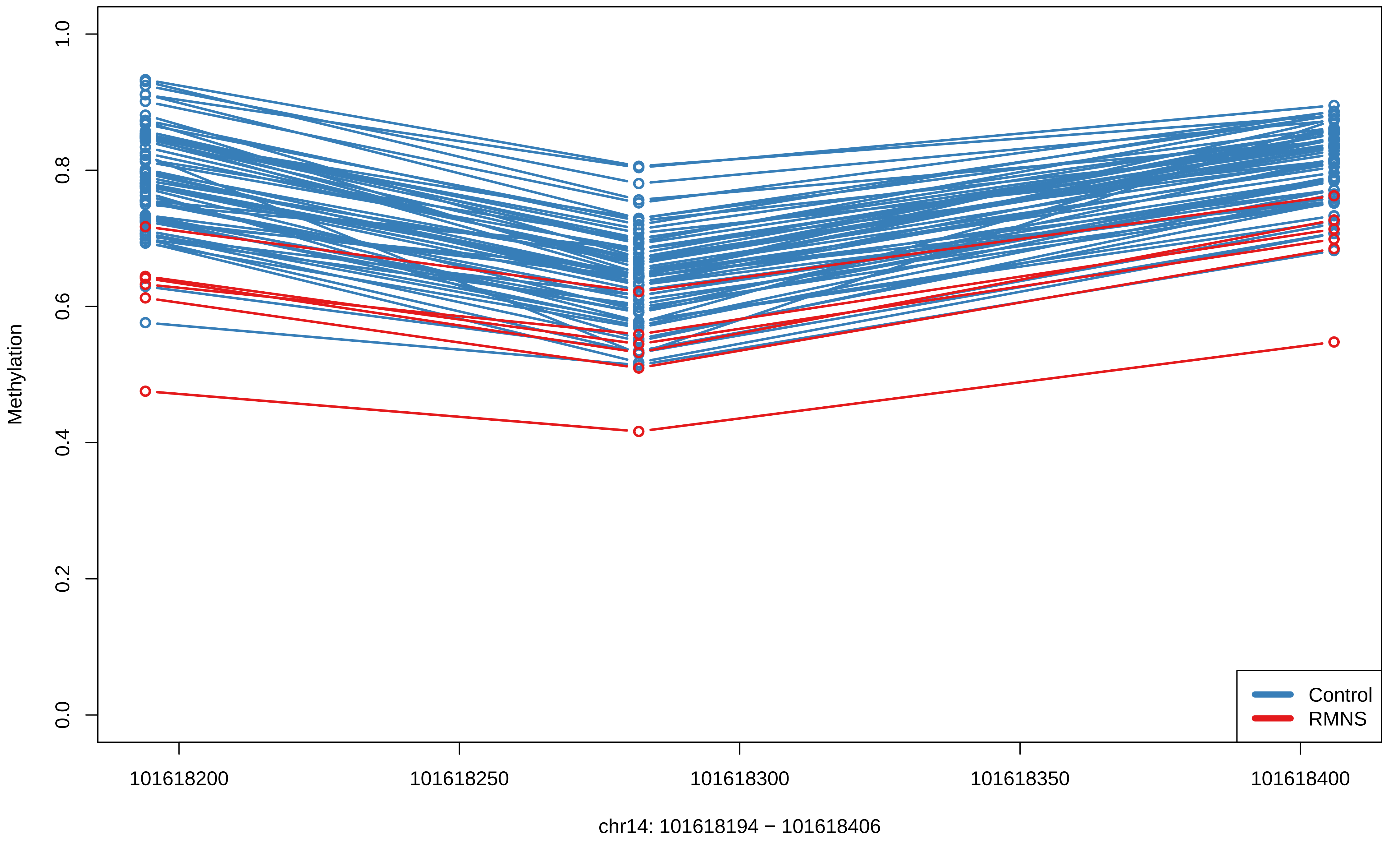

Methylation
0.0
0.2
0.4
0.6
0.8
1.0
101618200
101618250
101618300
101618350
101618400
chr14: 101618194 − 101618406
Control
RMNS

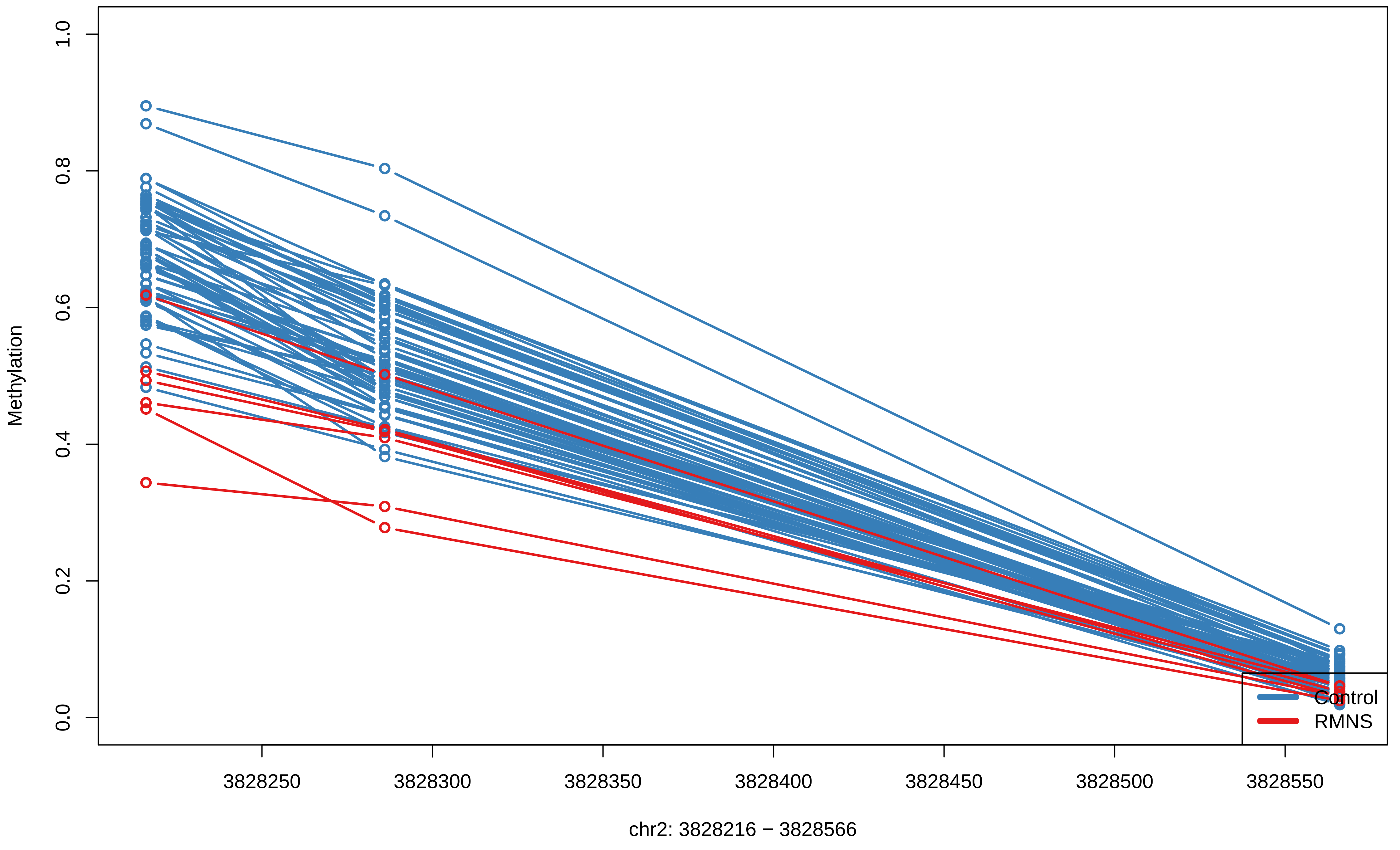

Methylation
0.0
0.2
0.4
0.6
0.8
1.0
3828250
3828300
3828350
3828400
3828450
3828500
3828550
chr2: 3828216 − 3828566
Control
RMNS

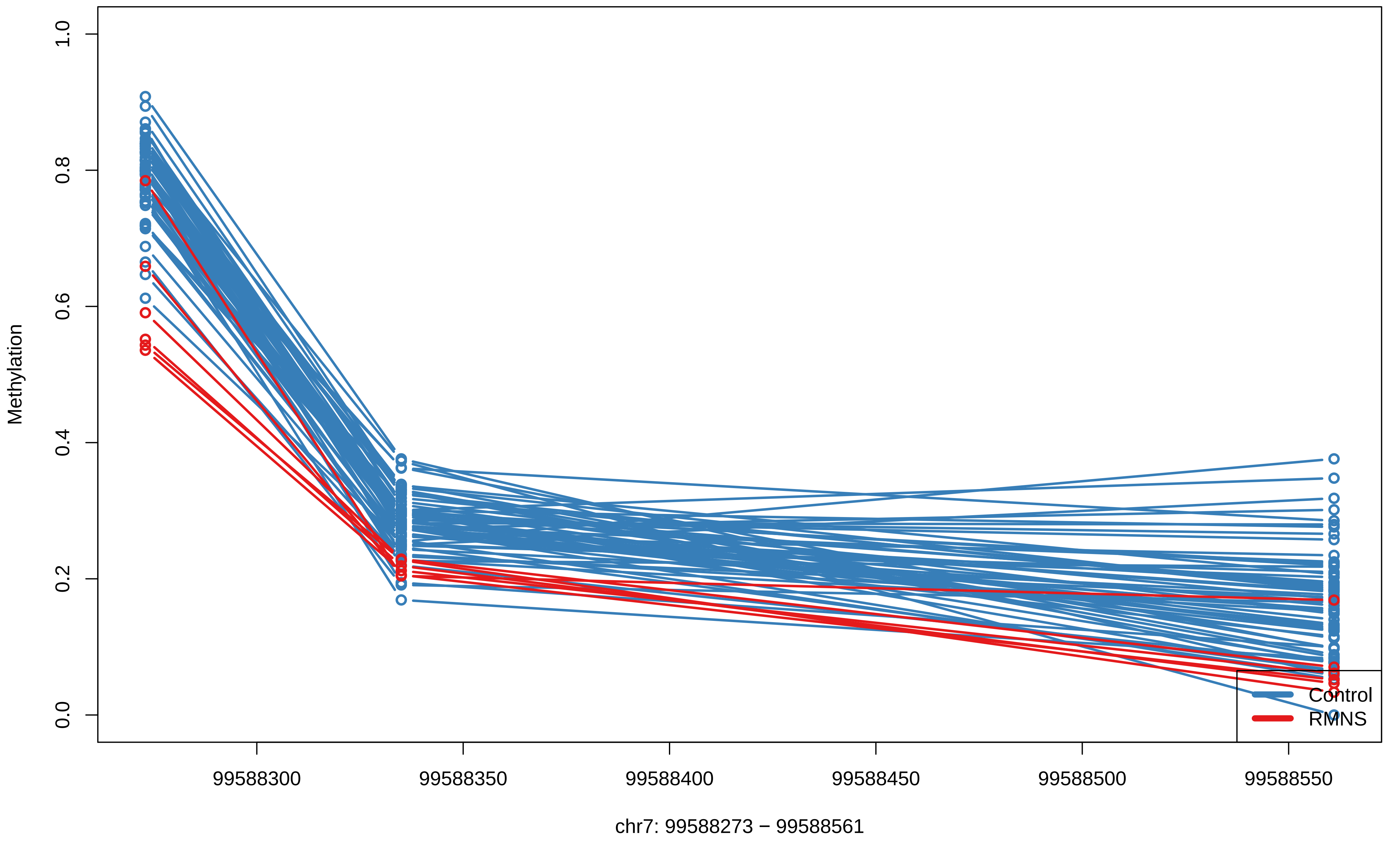

Methylation
0.0
0.2
0.4
0.6
0.8
1.0
99588300
99588350
99588400
99588450
99588500
99588550
chr7: 99588273 − 99588561
Control
RMNS

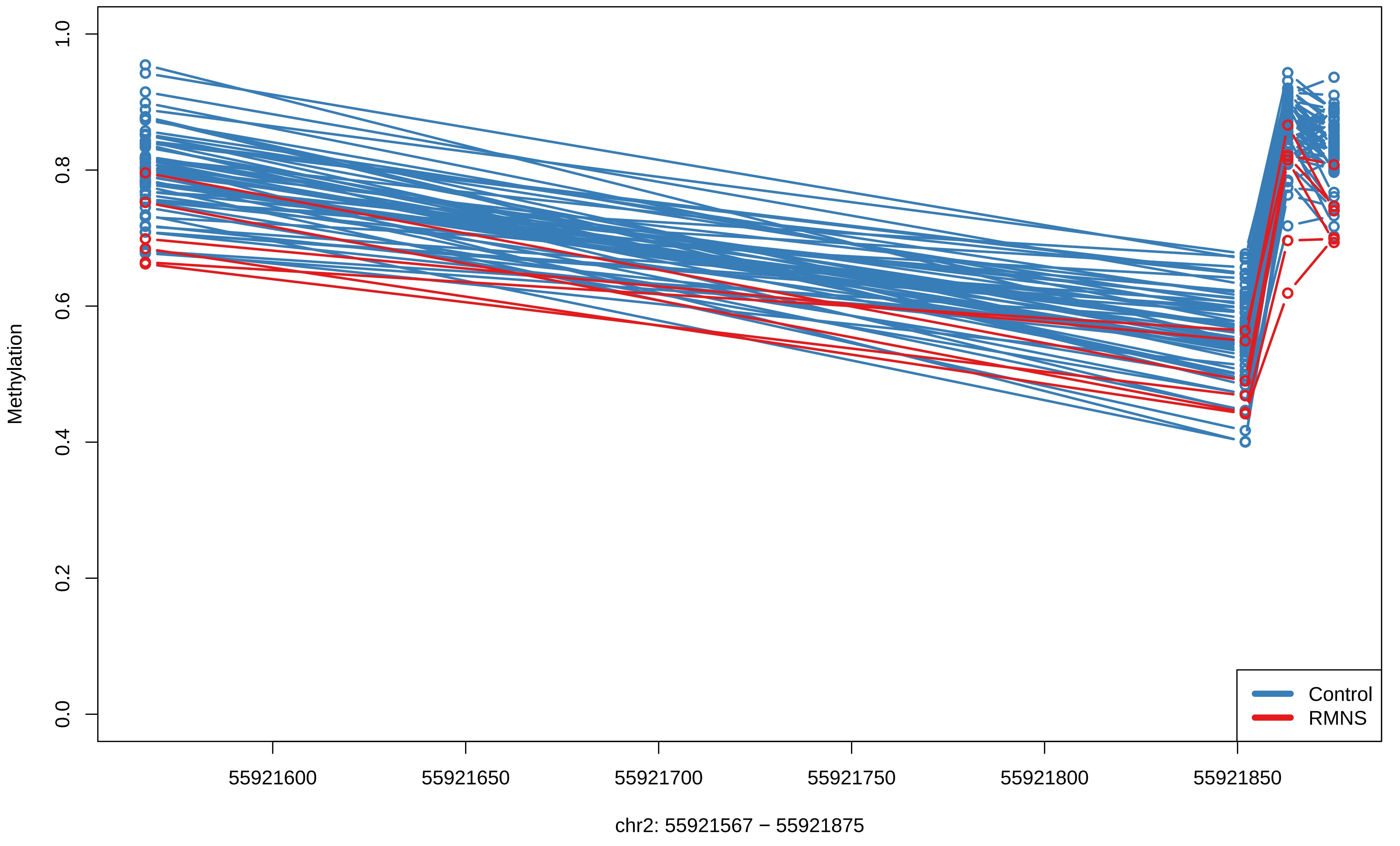

Methylation
0.0
0.2
0.4
0.6
0.8
1.0
55921600
55921650
55921700
55921750
55921800
55921850
chr2: 55921567 − 55921875
Control
RMNS

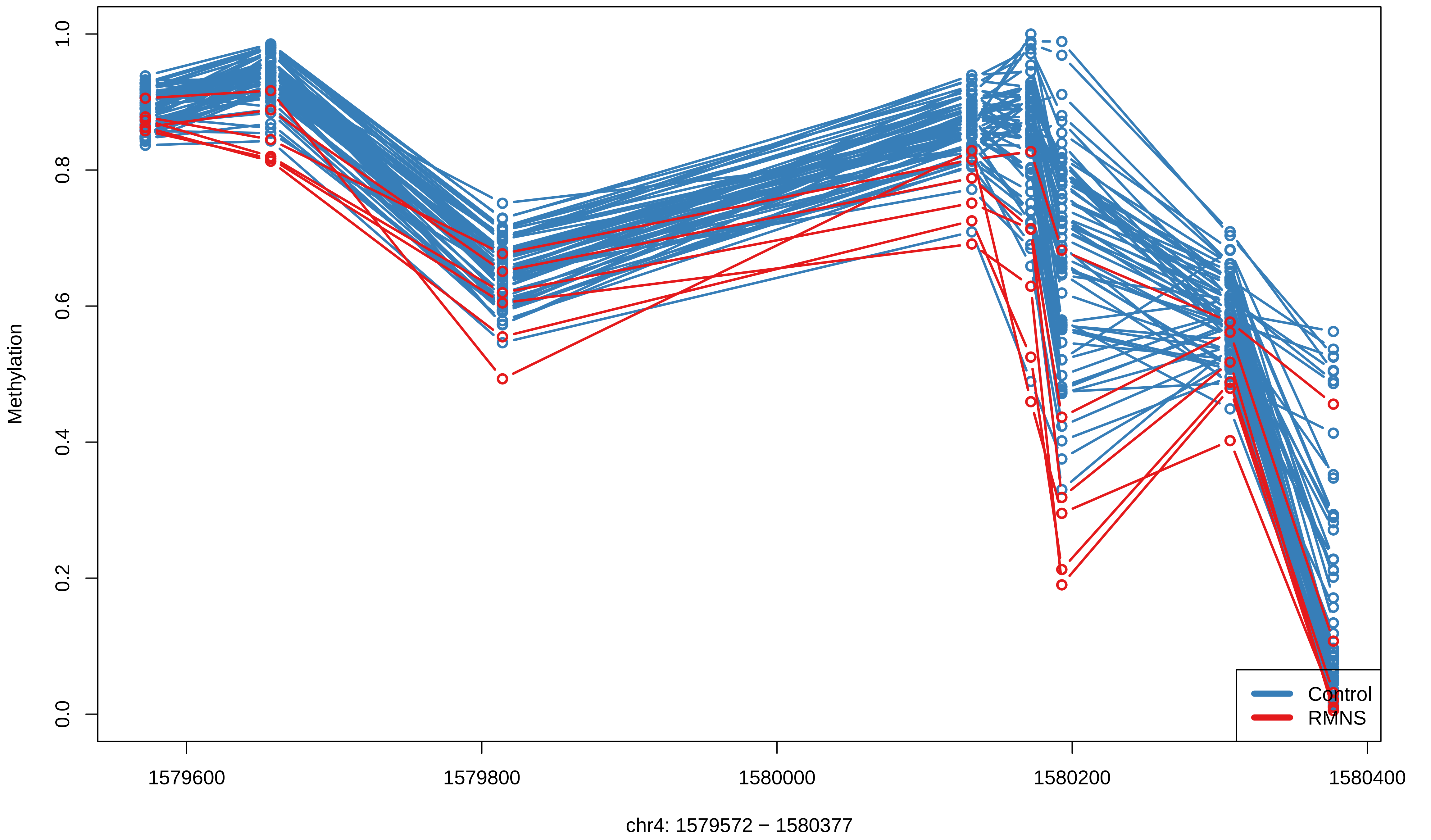

Methylation
0.0
0.2
0.4
0.6
0.8
1.0
1579600
1579800
1580000
1580200
1580400
chr4: 1579572 − 1580377
Control
RMNS

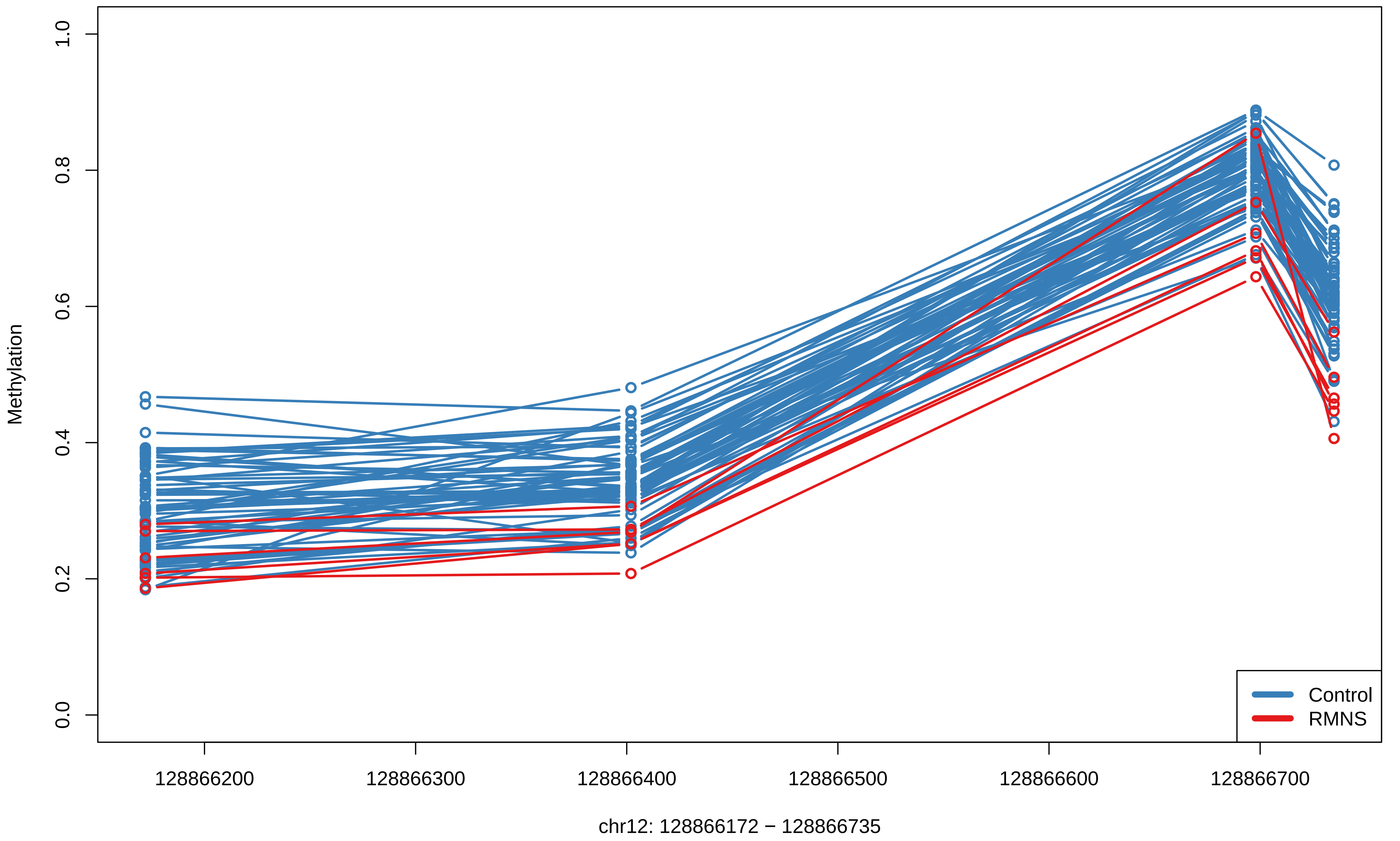

Methylation
0.0
0.2
0.4
0.6
0.8
1.0
128866200
128866300
128866400
128866500
128866600
128866700
chr12: 128866172 − 128866735
Control
RMNS

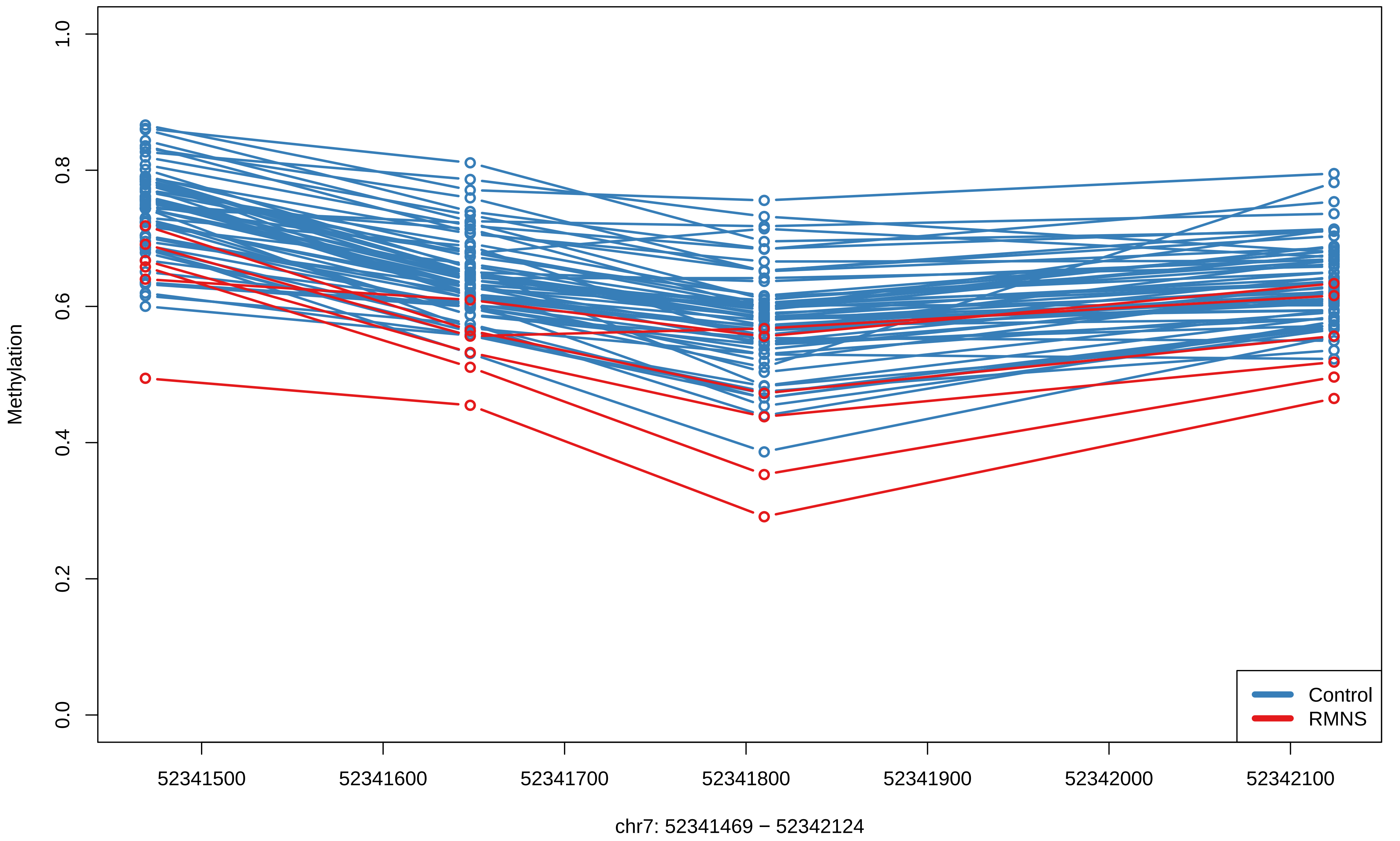

Methylation
0.0
0.2
0.4
0.6
0.8
1.0
52341500
52341600
52341700
52341800
52341900
52342000
52342100
chr7: 52341469 − 52342124
Control
RMNS

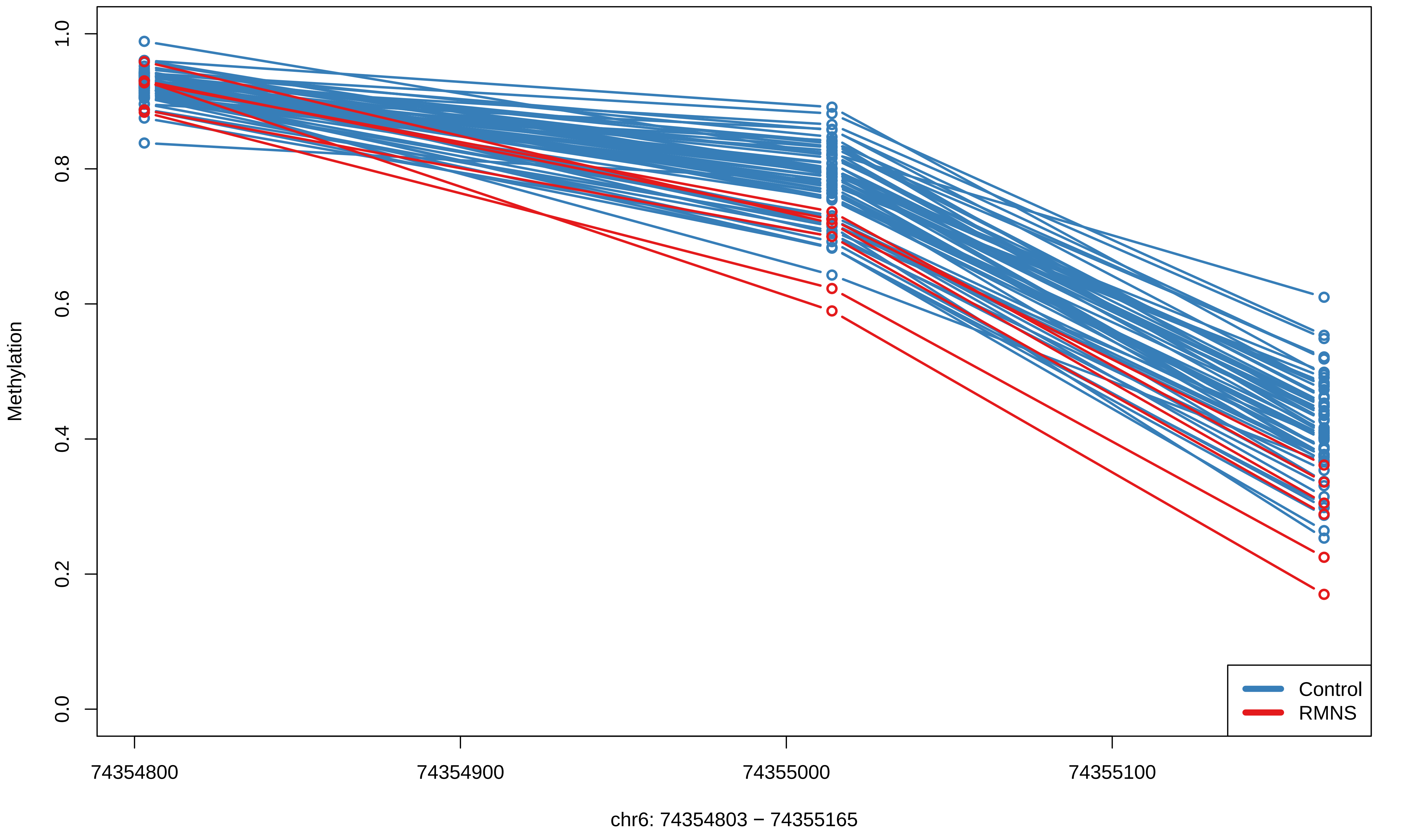

Methylation
0.0
0.2
0.4
0.6
0.8
1.0
74354800
74354900
74355000
74355100
chr6: 74354803 − 74355165
Control
RMNS

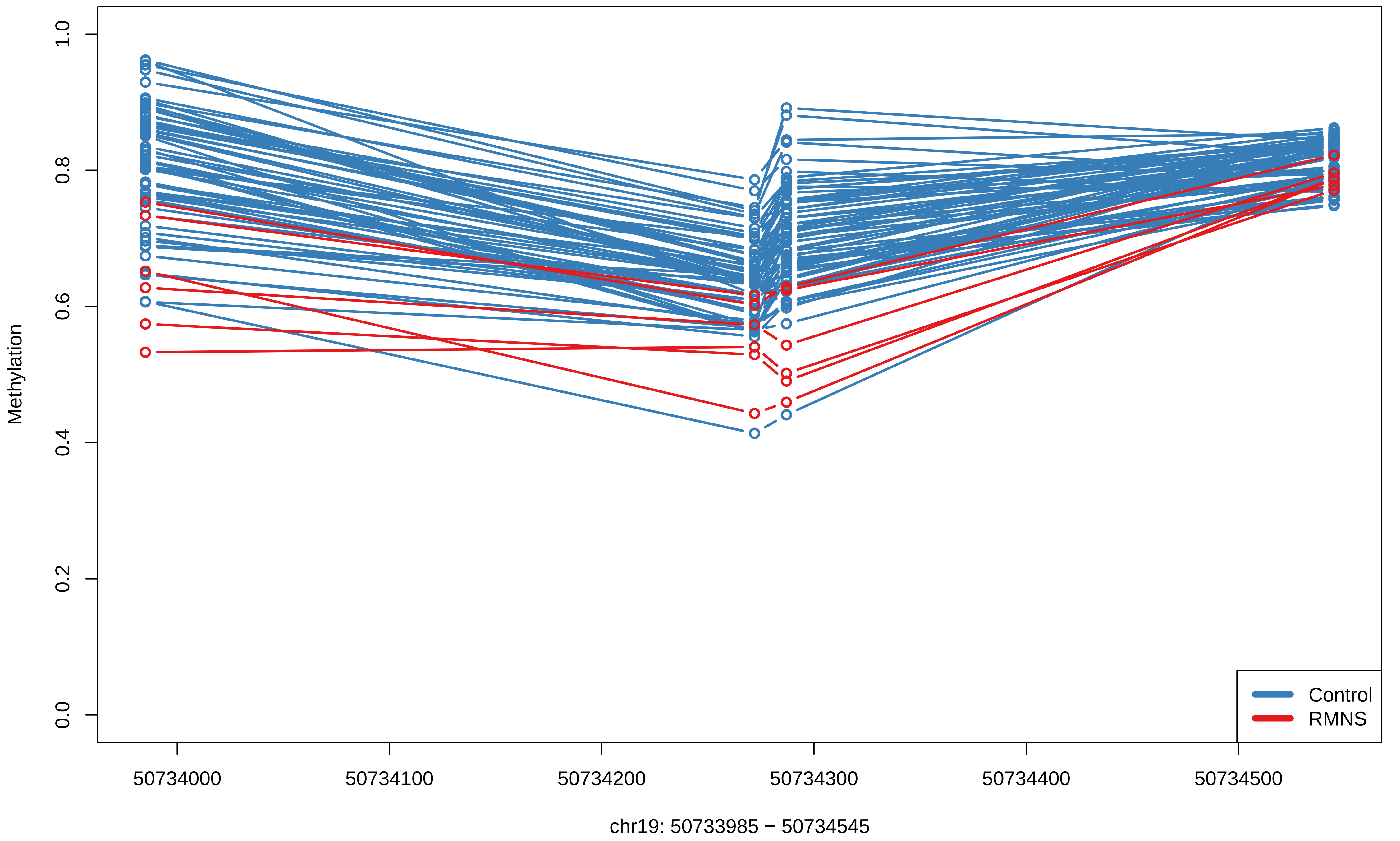

Methylation
0.0
0.2
0.4
0.6
0.8
1.0
50734000
50734100
50734200
50734300
50734400
50734500
chr19: 50733985 − 50734545
Control
RMNS

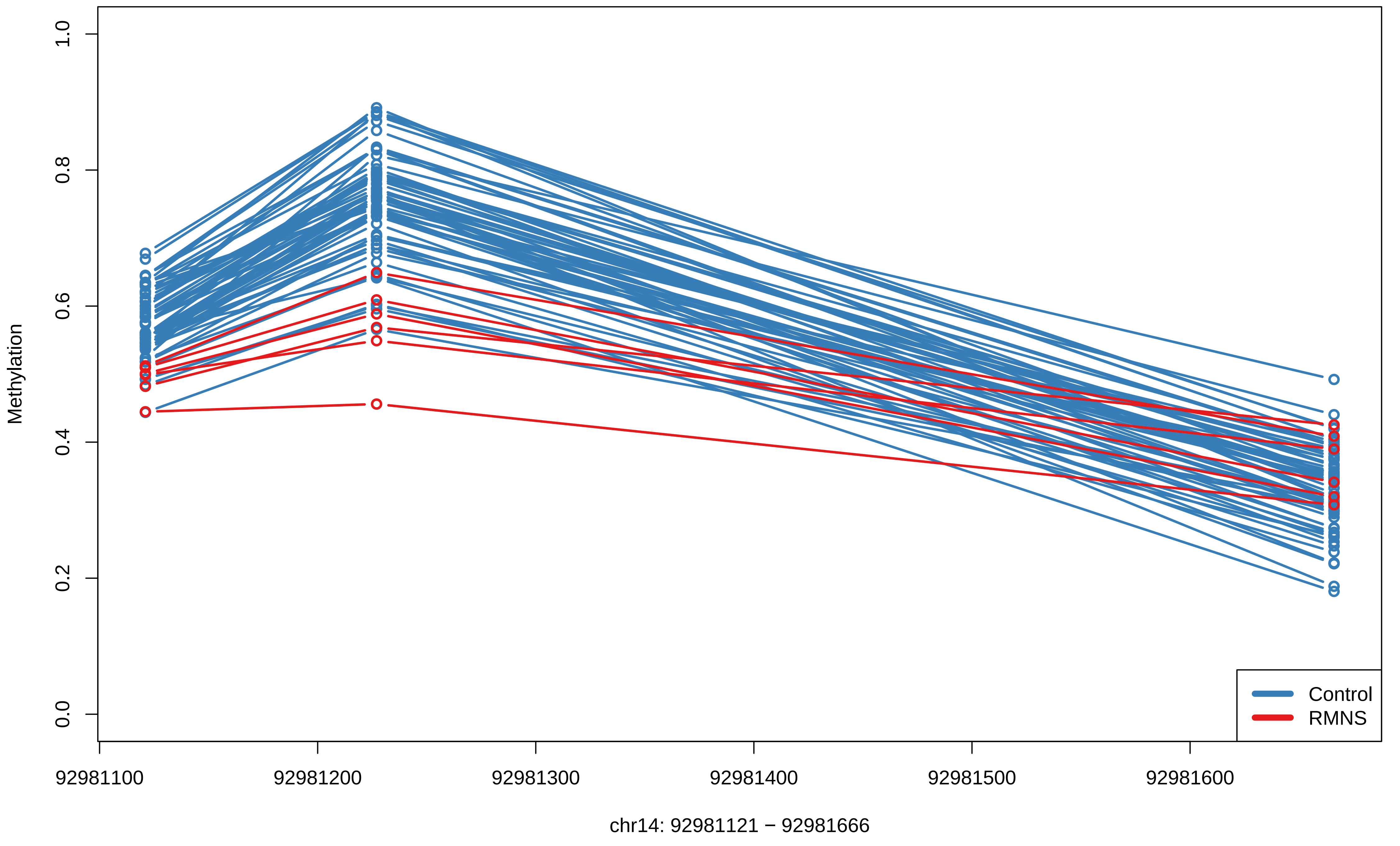

Methylation
0.0
0.2
0.4
0.6
0.8
1.0
92981100
92981200
92981300
92981400
92981500
92981600
chr14: 92981121 − 92981666
Control
RMNS

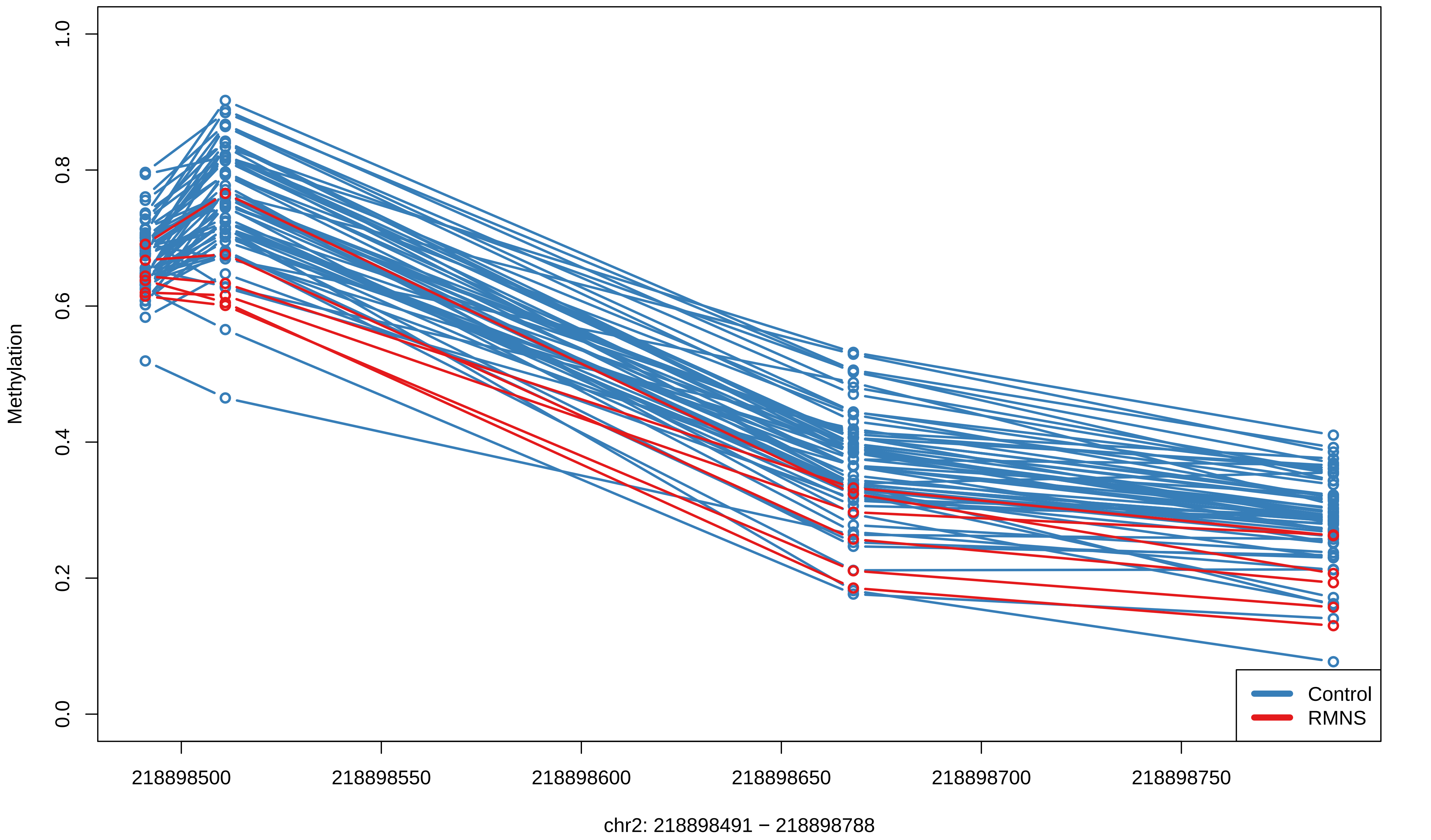

Methylation
0.0
0.2
0.4
0.6
0.8
1.0
218898500
218898550
218898600
218898650
218898700
218898750
chr2: 218898491 − 218898788
Control
RMNS

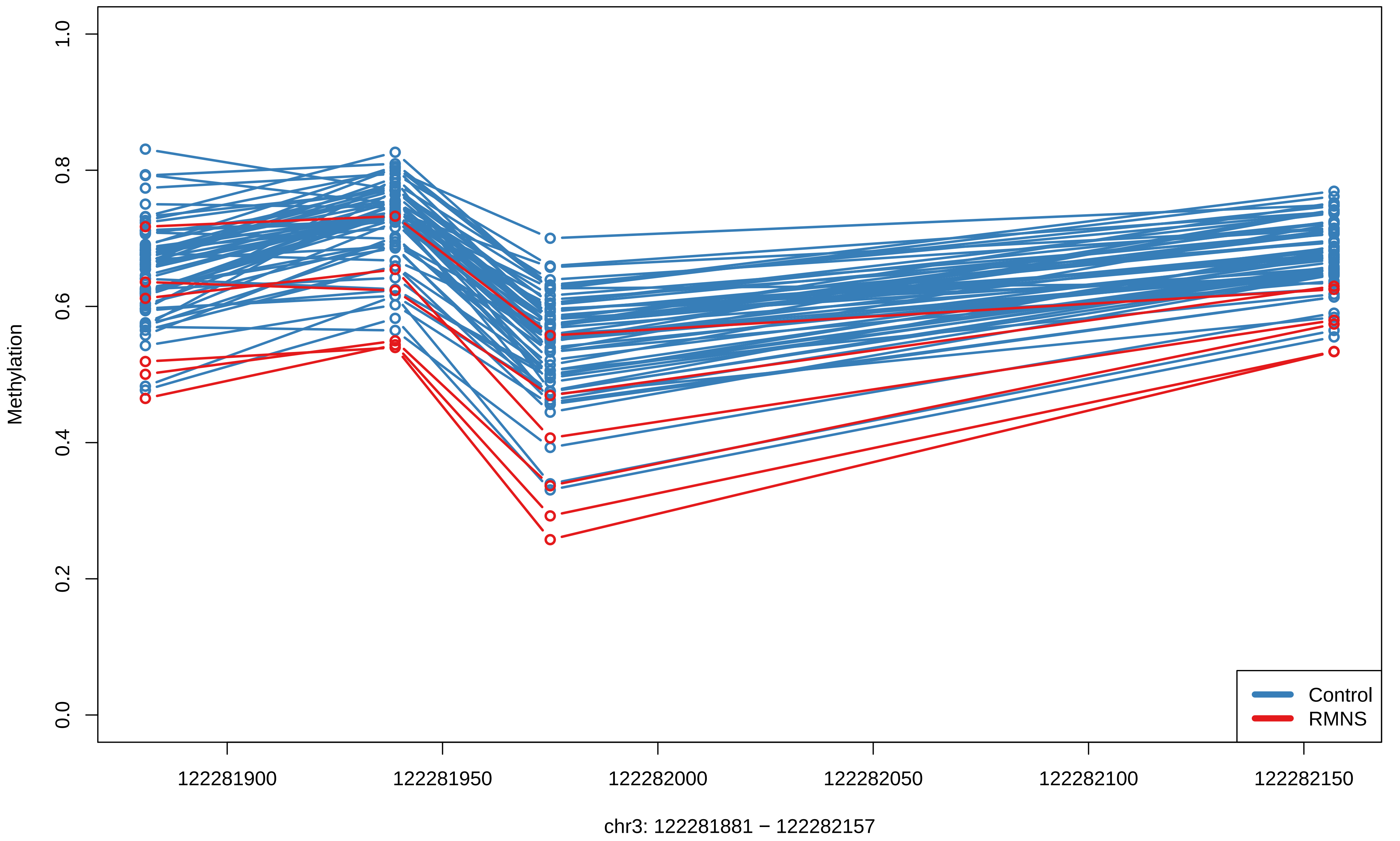

Methylation
0.0
0.2
0.4
0.6
0.8
1.0
122281900
122281950
122282000
122282050
122282100
122282150
chr3: 122281881 − 122282157
Control
RMNS

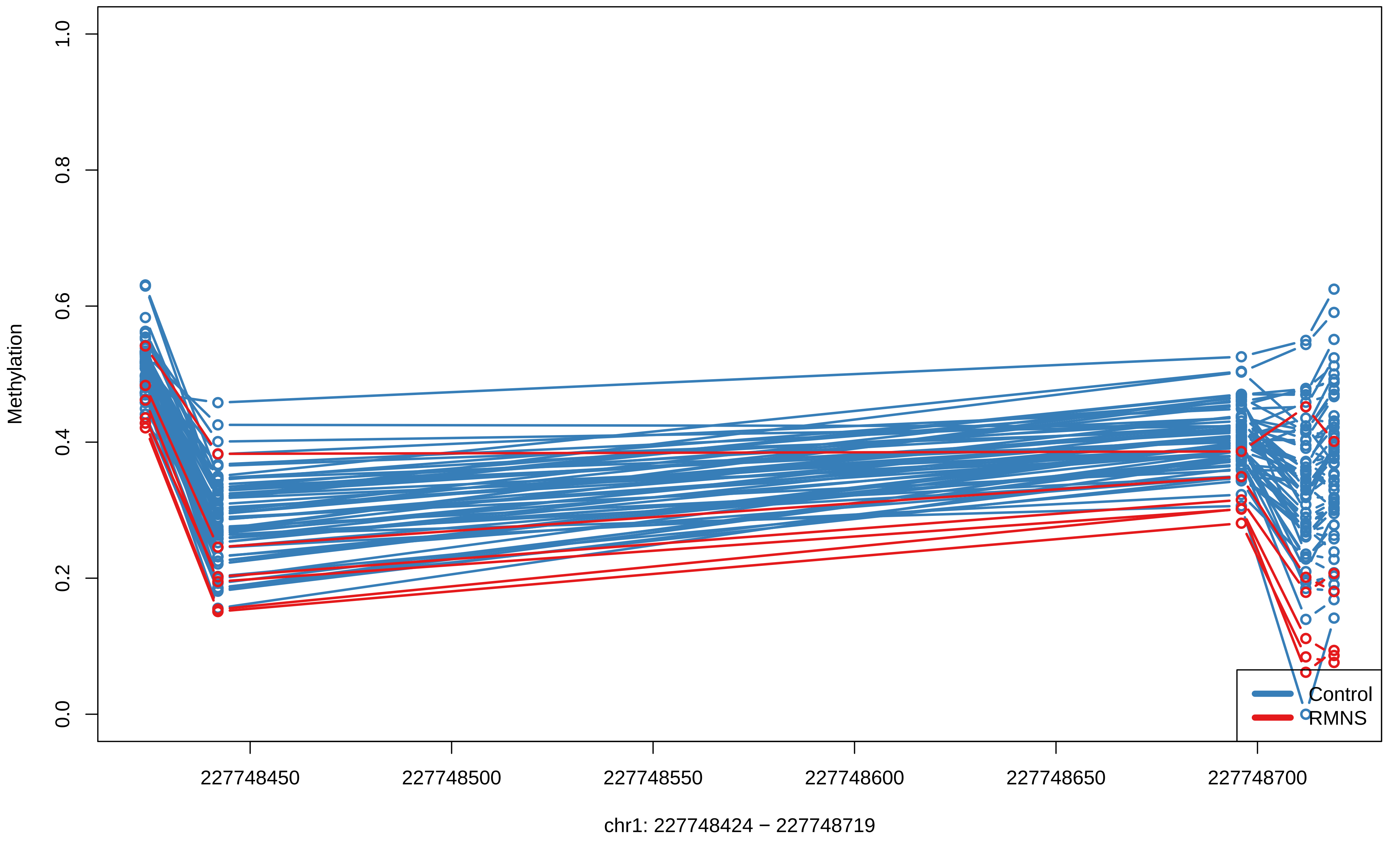

Methylation
1.0
0.8
0.6
0.4
0.2
0.0
227748450
227748500
227748550
227748600
227748650
227748700
chr1: 227748424 − 227748719
Control
RMNS

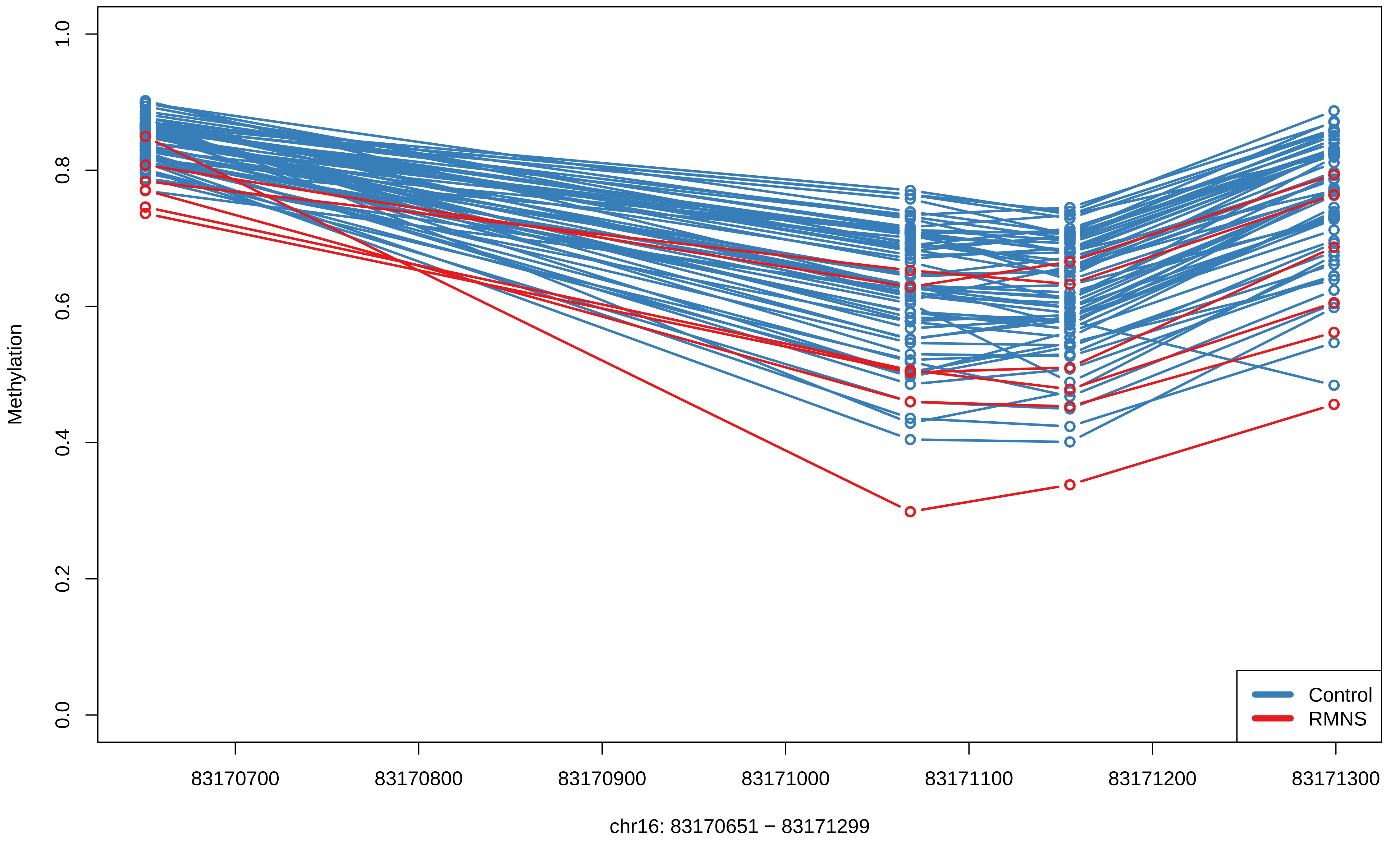
Methylation
1.0
0.8
0.6
0.4
0.2
0.0
83170700
83170800
83170900
83171000
83171100
83171200
83171300
chr16: 83170651 − 83171299
Control
RMNS

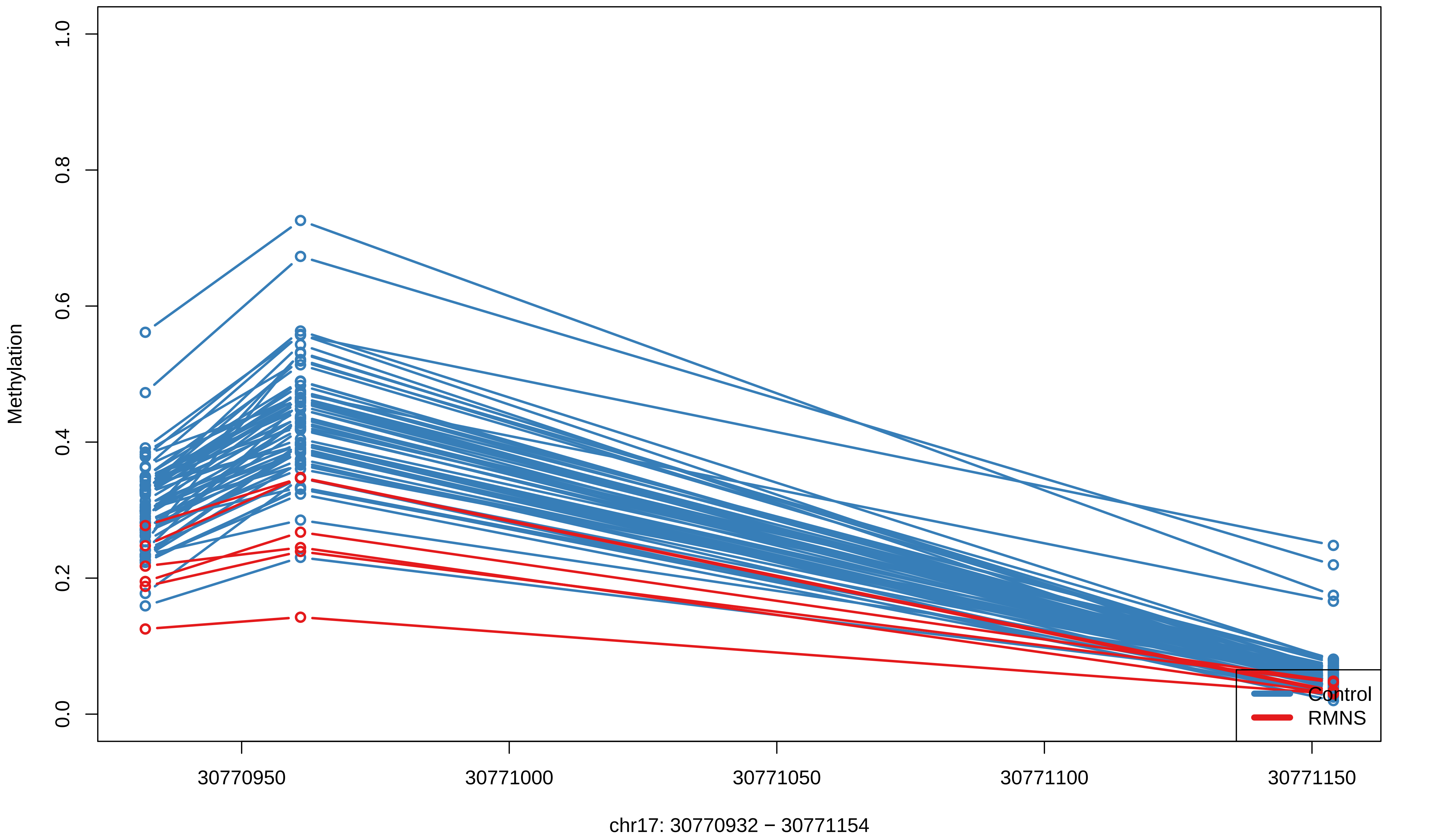

Methylation
0.0
0.2
0.4
0.6
0.8
1.0
30770950
30771000
30771050
30771100
30771150
chr17: 30770932 − 30771154
Control
RMNS

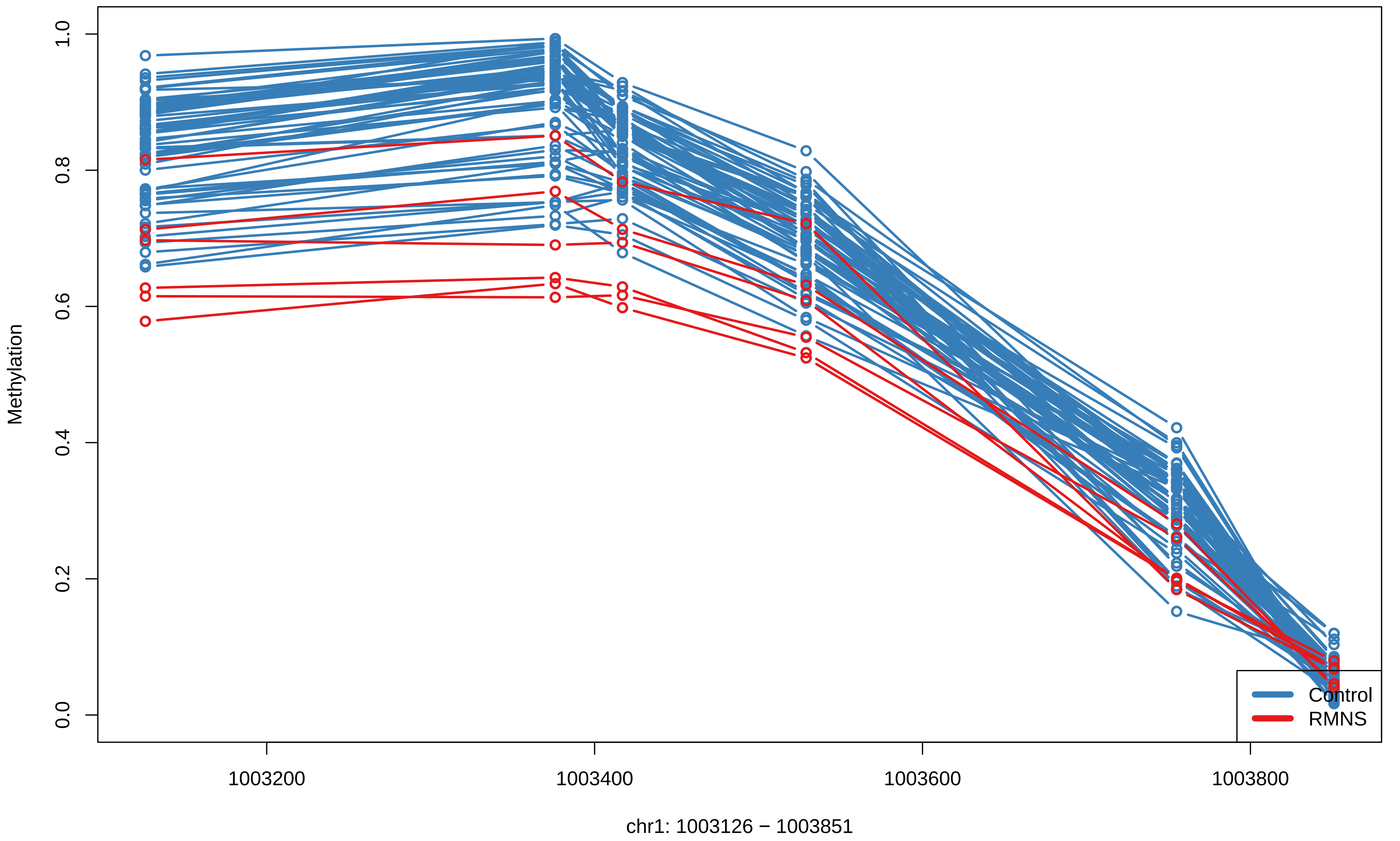

1.0
0.8
0.6
0.4
0.2
0.0
Methylation
1003200
1003400
1003600
1003800
chr1: 1003126 − 1003851
Control
RMNS

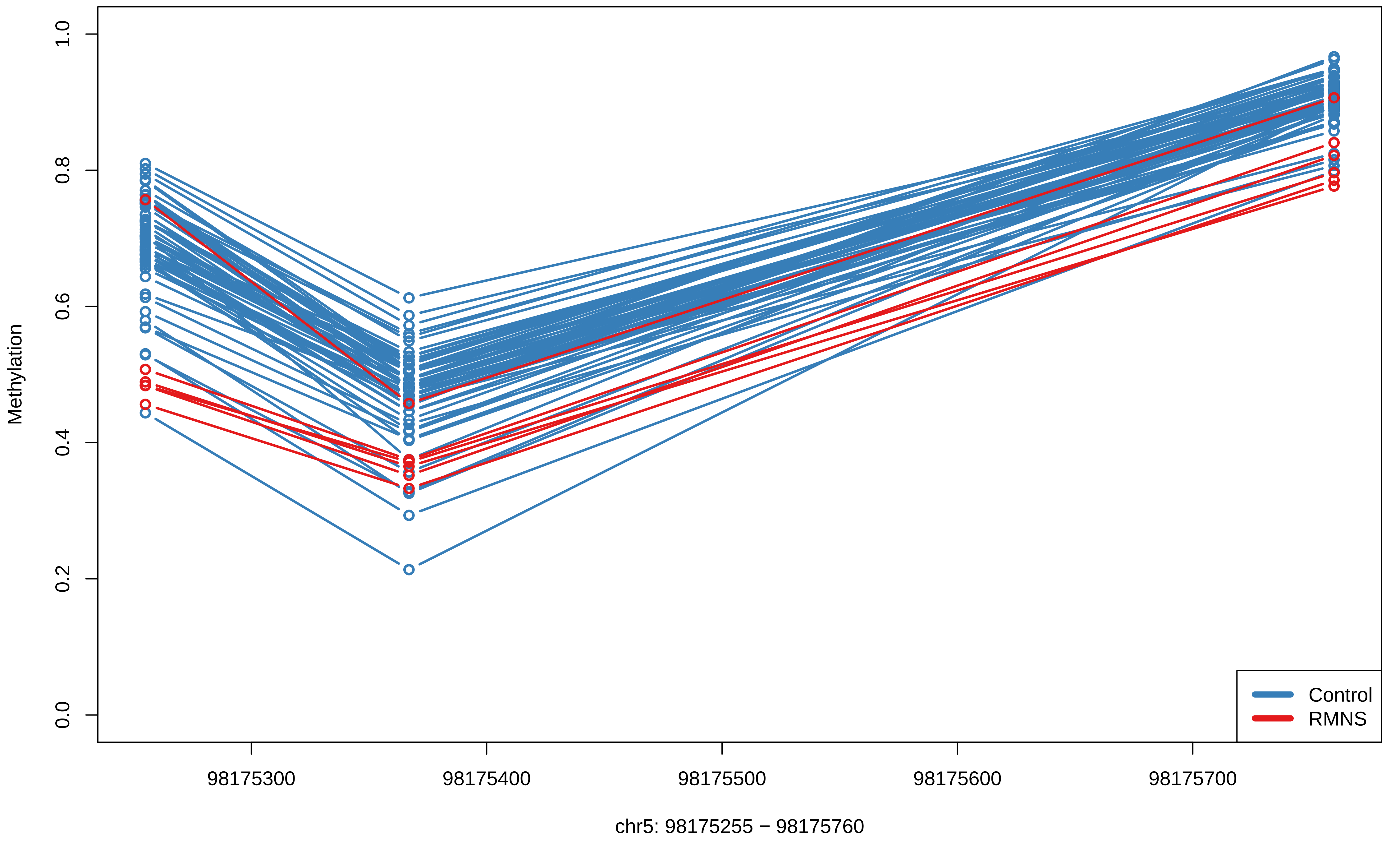

Methylation
0.0
0.2
0.4
0.6
0.8
1.0
98175300
98175400
98175500
98175600
98175700
chr5: 98175255 − 98175760
Control
RMNS

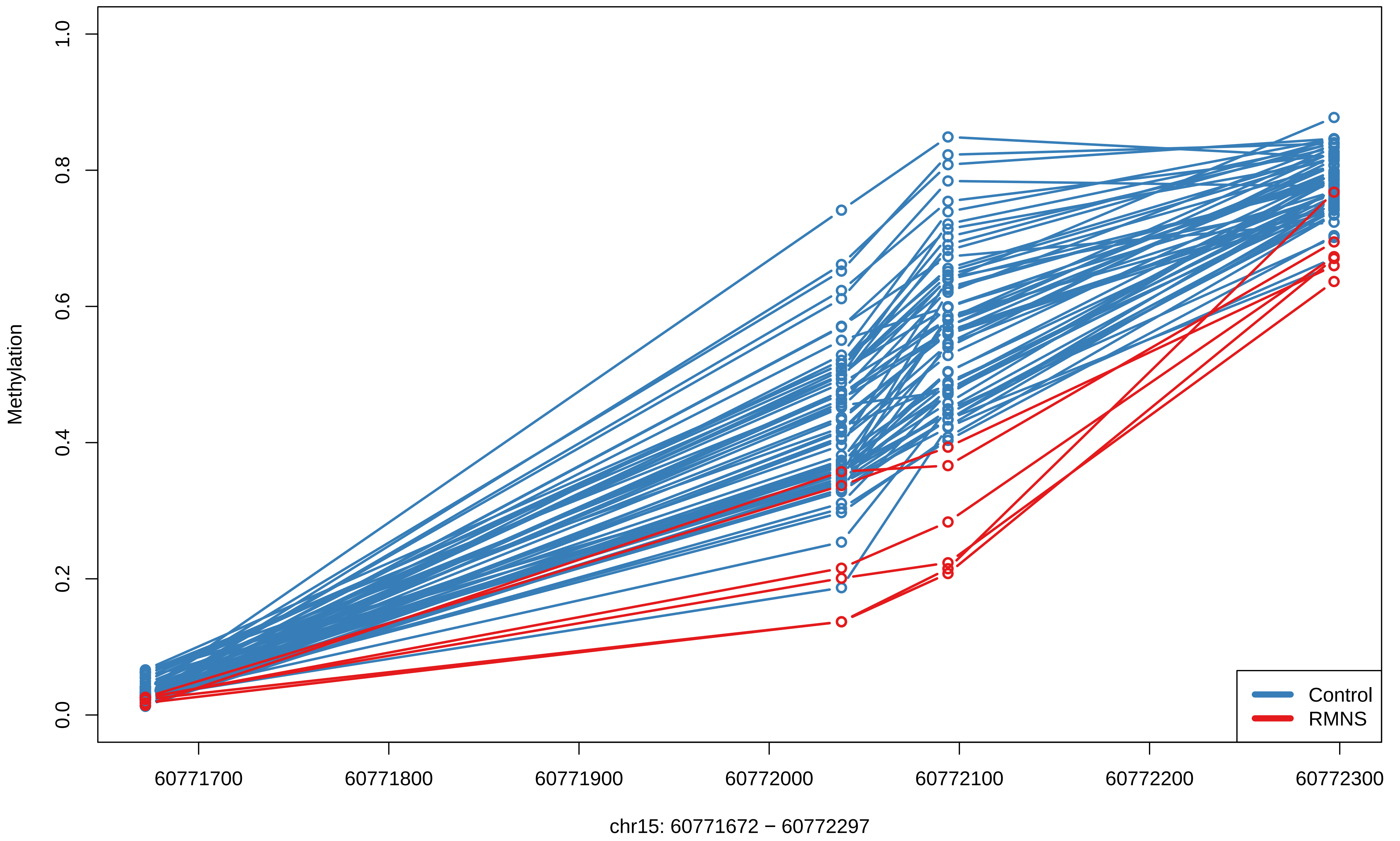

Methylation
1.0
0.8
0.6
0.4
0.2
0.0
60771700
60771800
60771900
60772000
60772100
60772200
60772300
chr15: 60771672 − 60772297
Control
RMNS

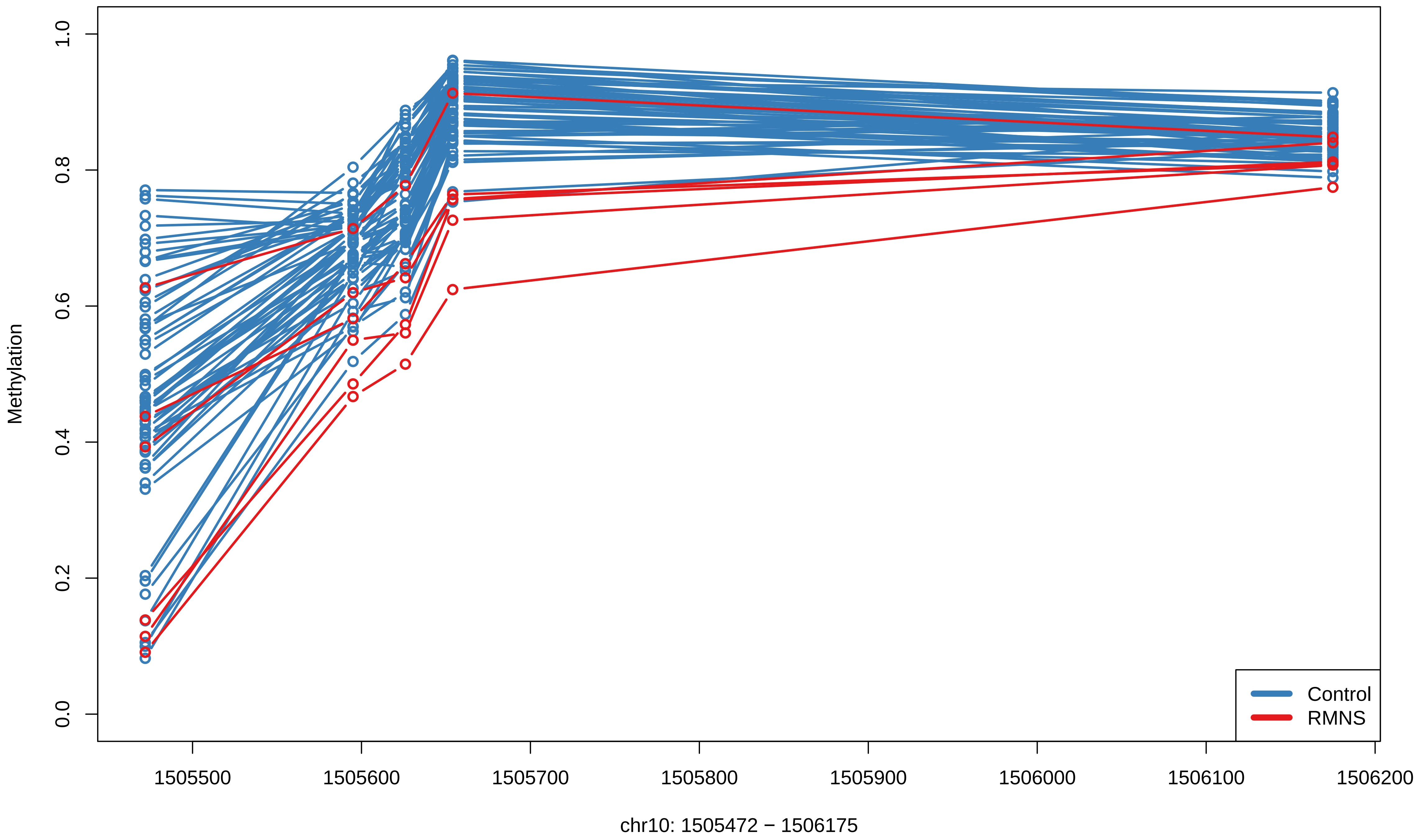

Methylation
0.0
0.2
0.4
0.6
0.8
1.0
1505500
1505600
1505700
1505800
1505900
1506000
1506100
1506200
chr10: 1505472 − 1506175
Control
RMNS

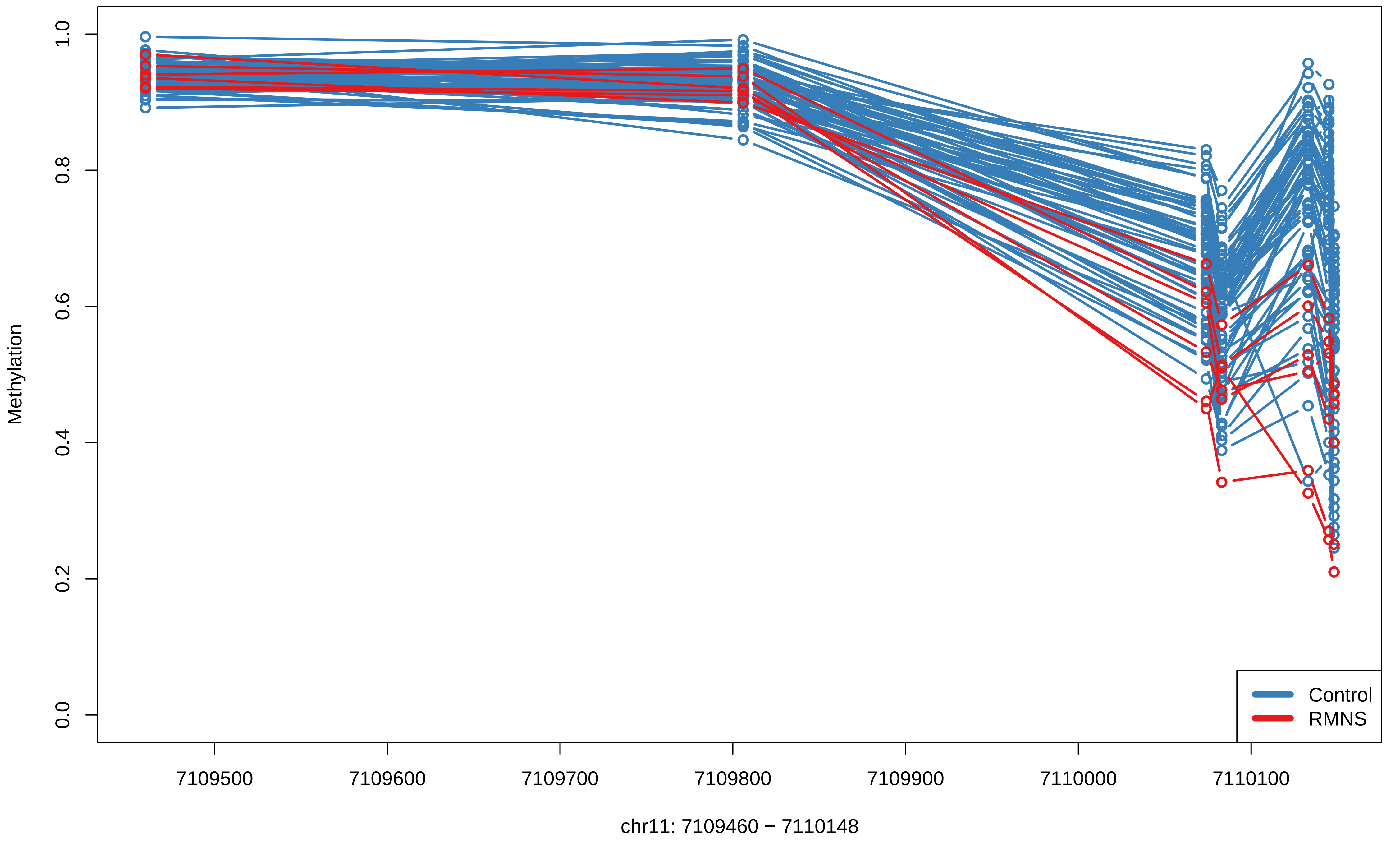

1.0
0.8
0.6
0.4
0.2
0.0
Methylation
7109500
7109600
7109700
7109800
7109900
7110000
7110100
chr11: 7109460 − 7110148
Control
RMNS

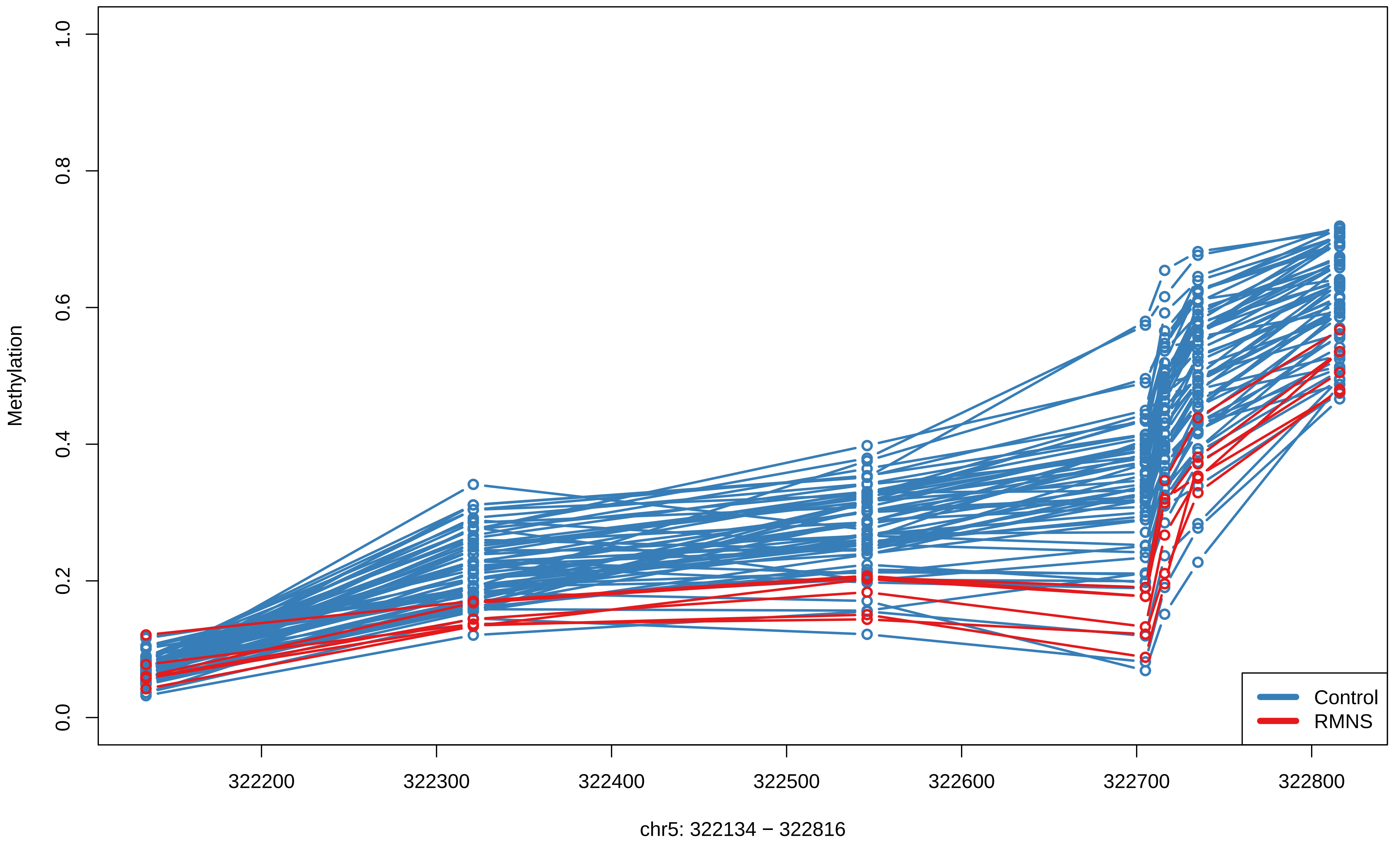

Methylation
0.0
0.2
0.4
0.6
0.8
1.0
322200
322300
322400
322500
322600
322700
322800
chr5: 322134 − 322816
Control
RMNS

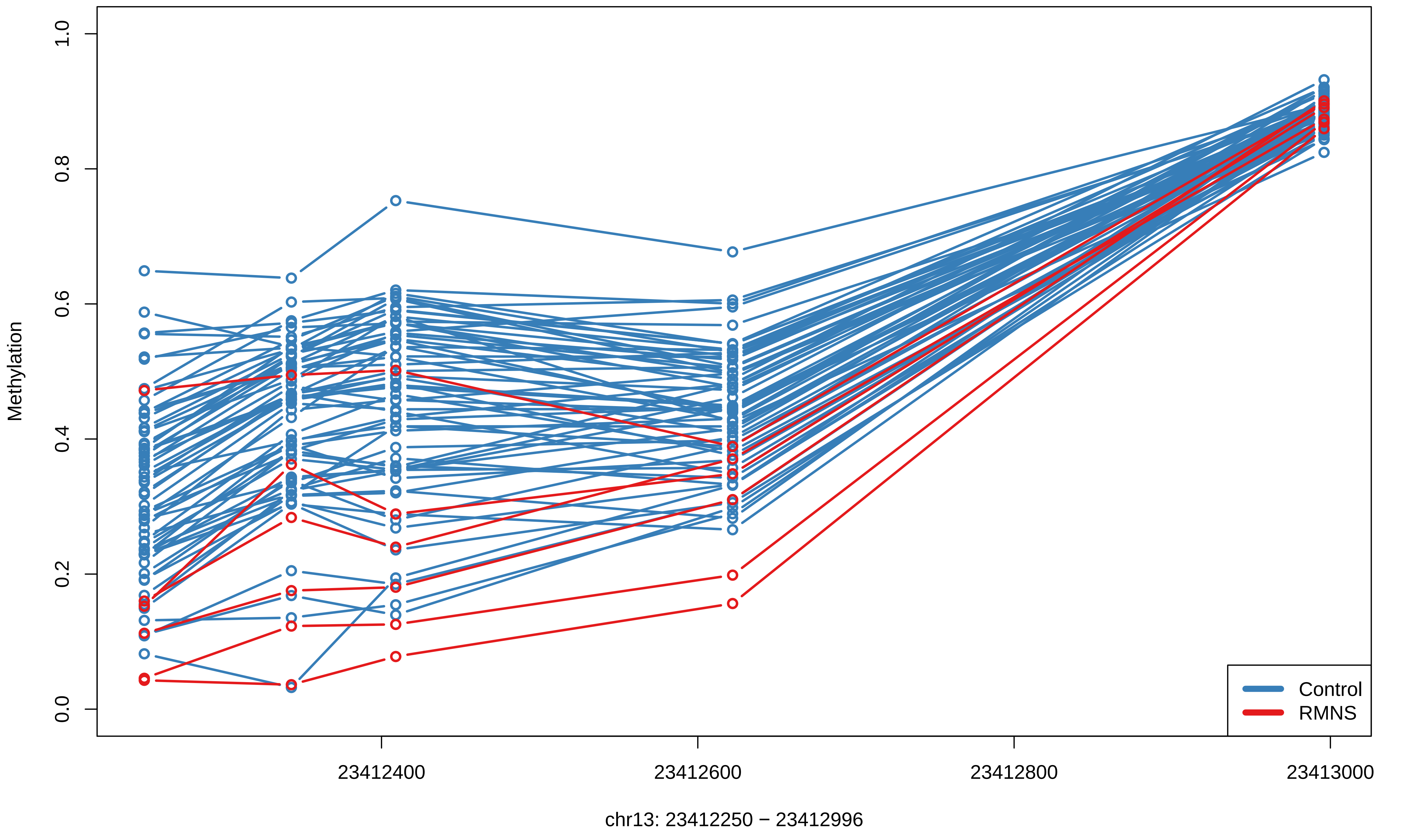

Methylation
0.0
0.2
0.4
0.6
0.8
1.0
23412400
23412600
23412800
23413000
chr13: 23412250 − 23412996
Control
RMNS

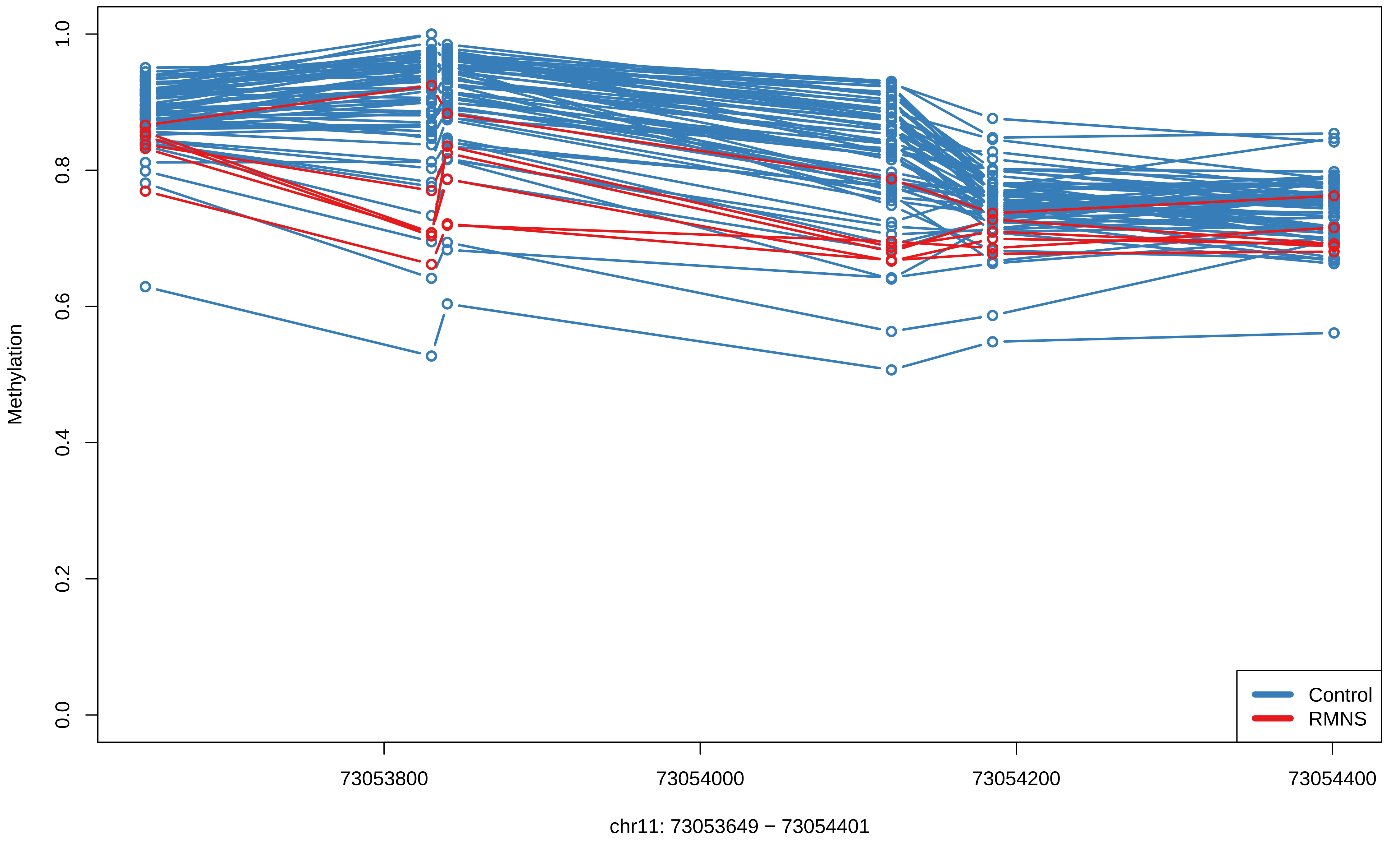

1.0
0.8
0.6
0.4
0.2
0.0
Methylation
73053800
73054000
73054200
73054400
chr11: 73053649 − 73054401
Control
RMNS

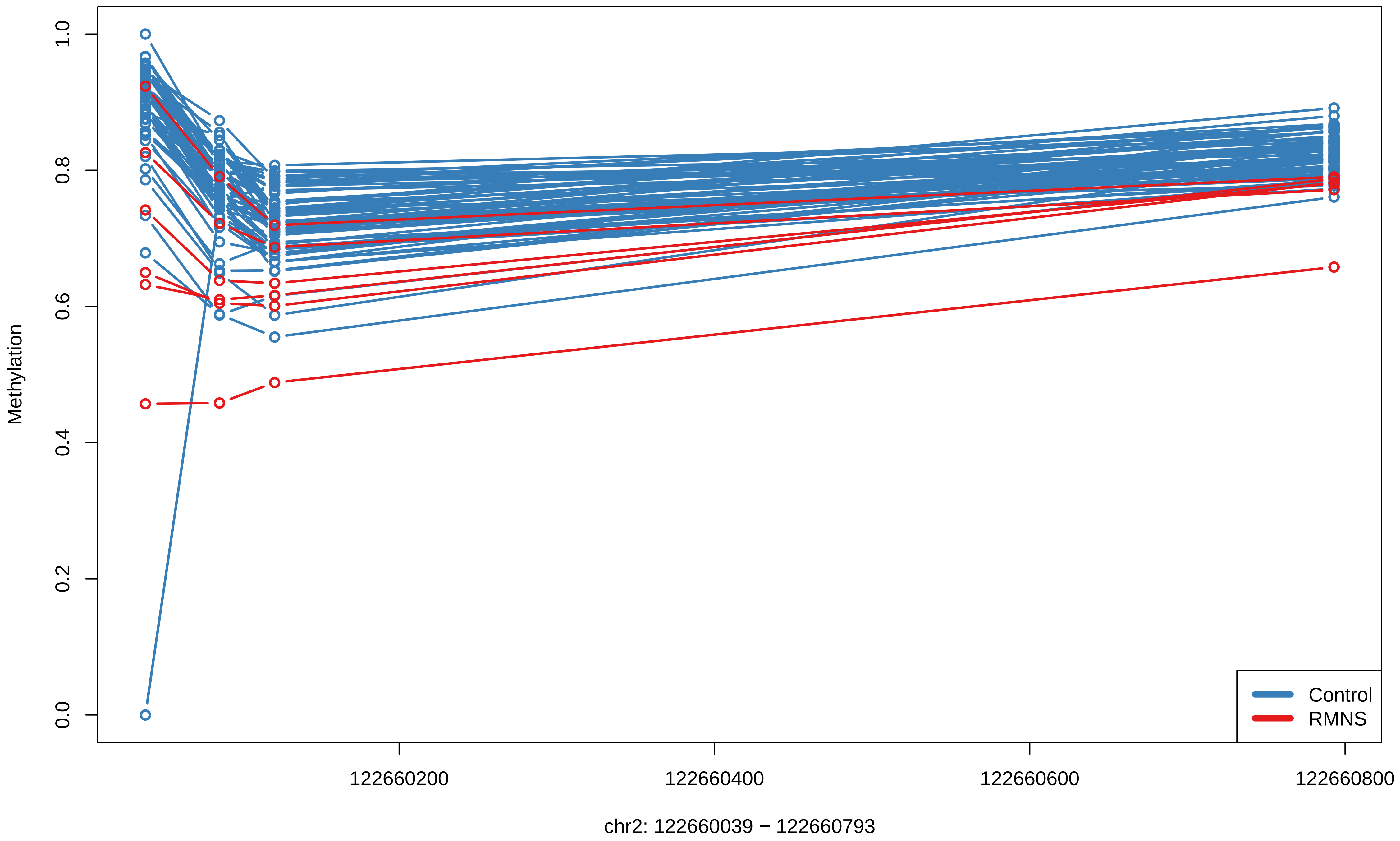

Methylation
1.0
0.8
0.6
0.4
0.2
0.0
122660200
122660400
122660600
122660800
chr2: 122660039 − 122660793
Control
RMNS

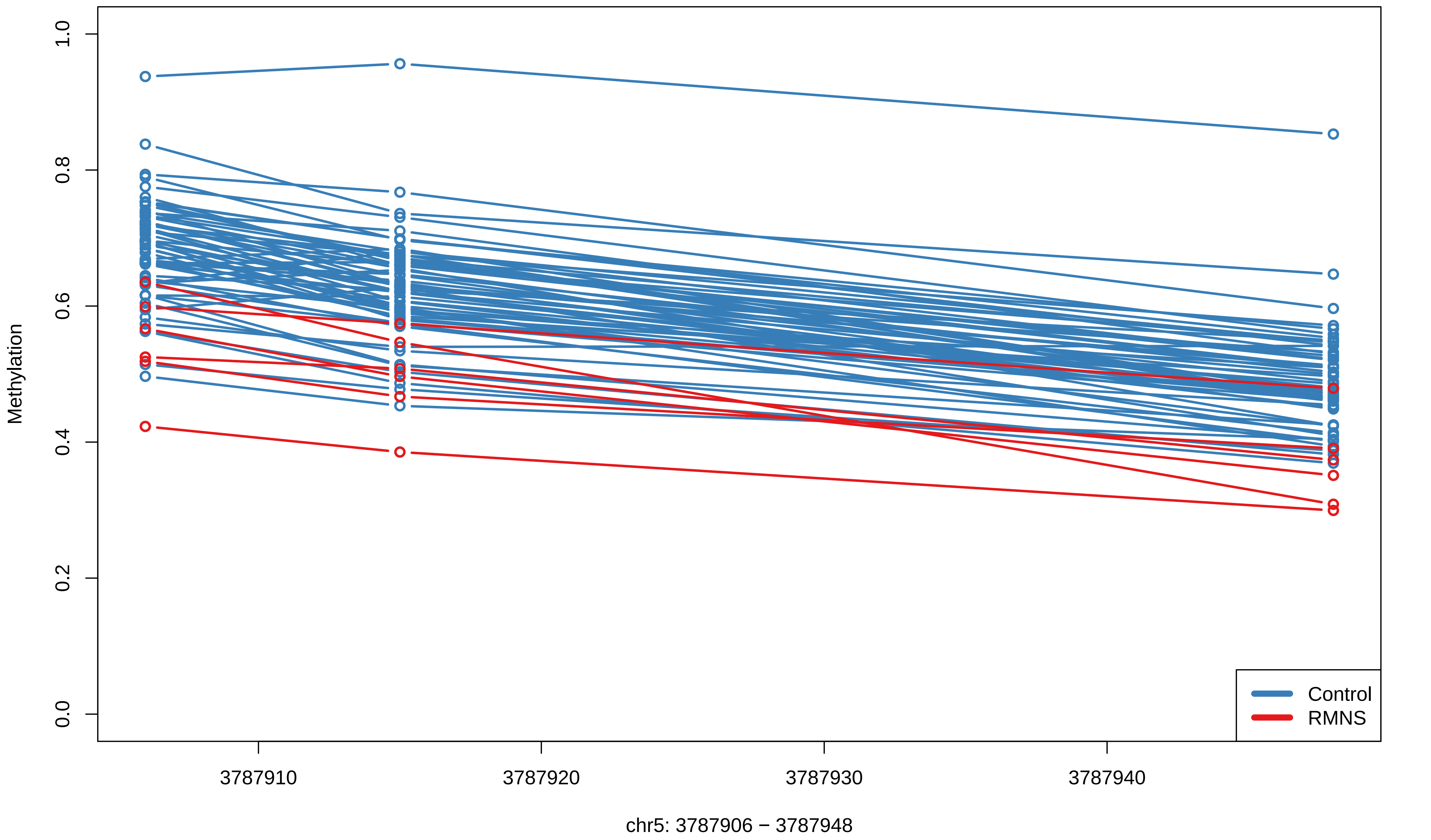

Methylation
1.0
0.8
0.6
0.4
0.2
0.0
3787910
3787920
3787930
3787940
chr5: 3787906 − 3787948
Control
RMNS

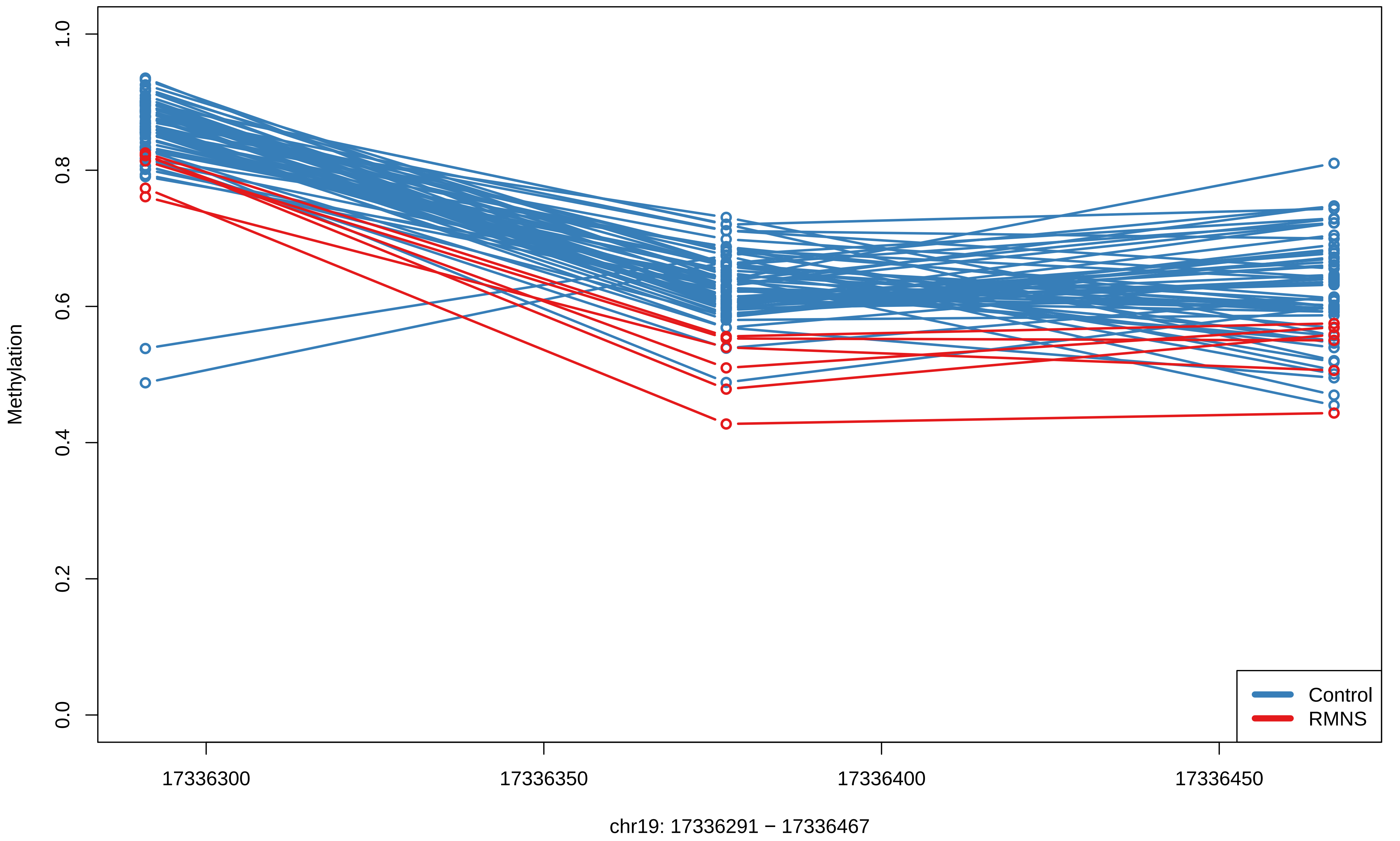

Methylation
0.0
0.2
0.4
0.6
0.8
1.0
17336300
17336350
17336400
17336450
chr19: 17336291 − 17336467
Control
RMNS

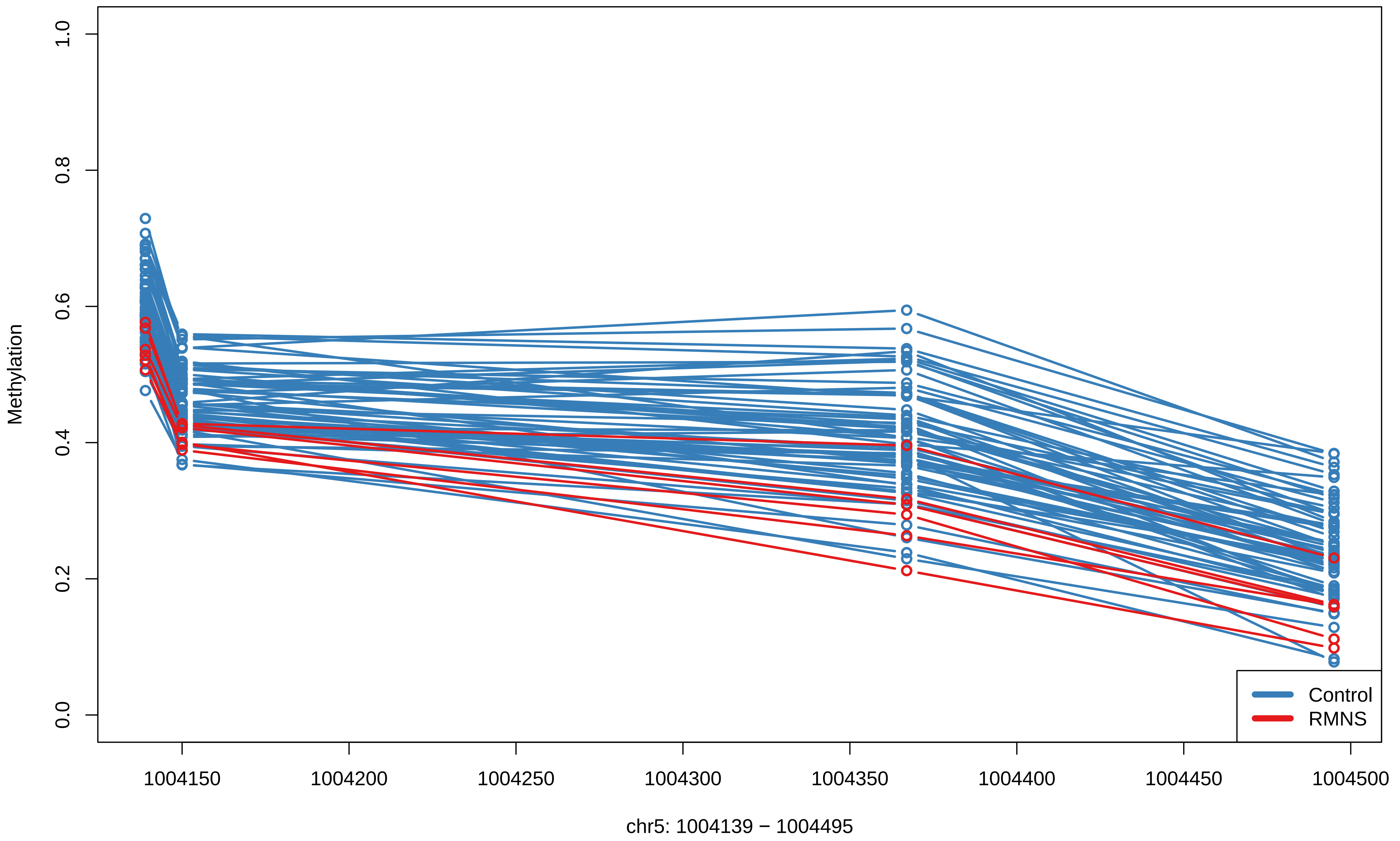

Methylation
0.0
0.2
0.4
0.6
0.8
1.0
1004150
1004200
1004250
1004300
1004350
1004400
1004450
1004500
chr5: 1004139 − 1004495
Control
RMNS

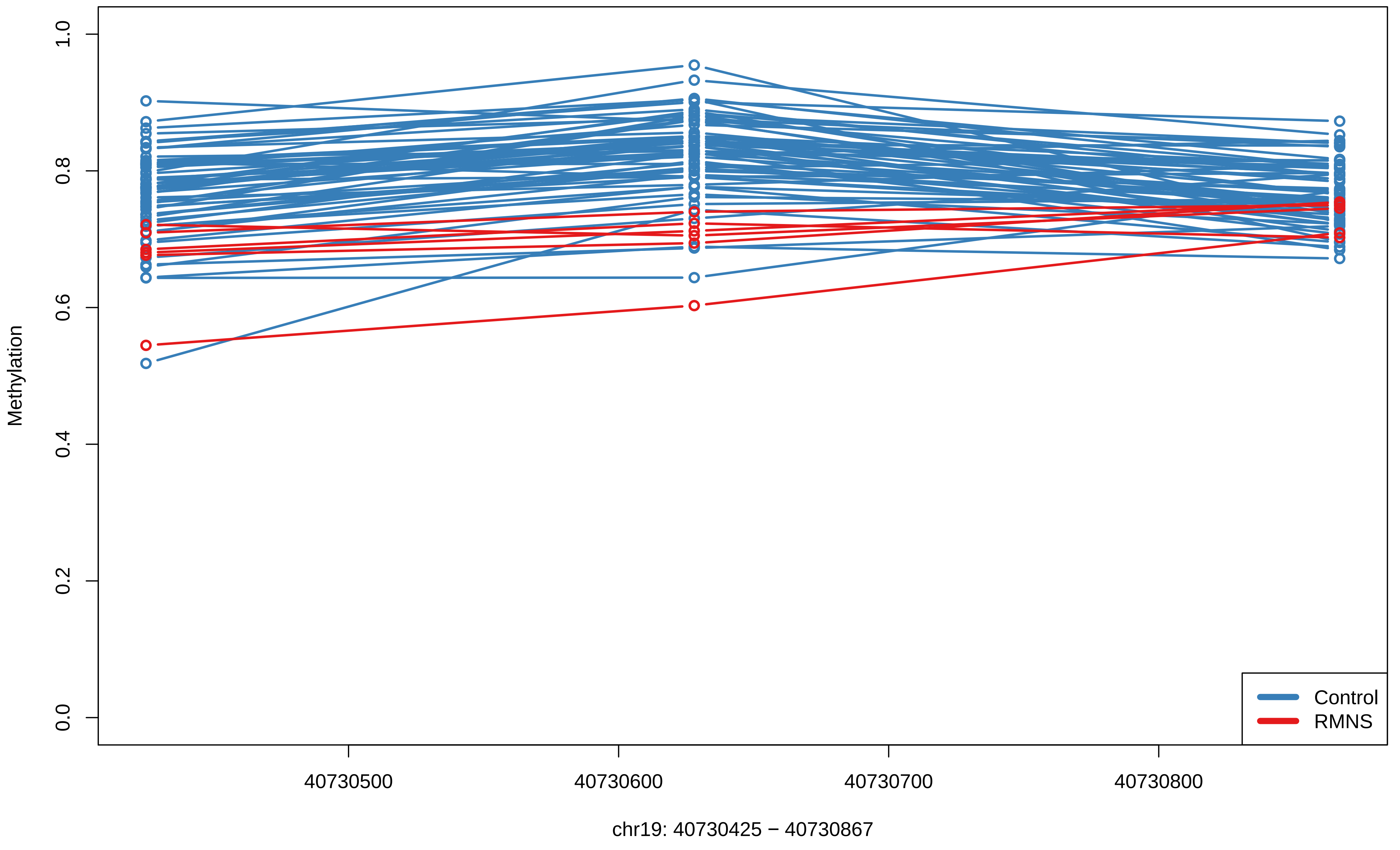

Methylation
0.0
0.2
0.4
0.6
0.8
1.0
40730500
40730600
40730700
40730800
chr19: 40730425 − 40730867
Control
RMNS

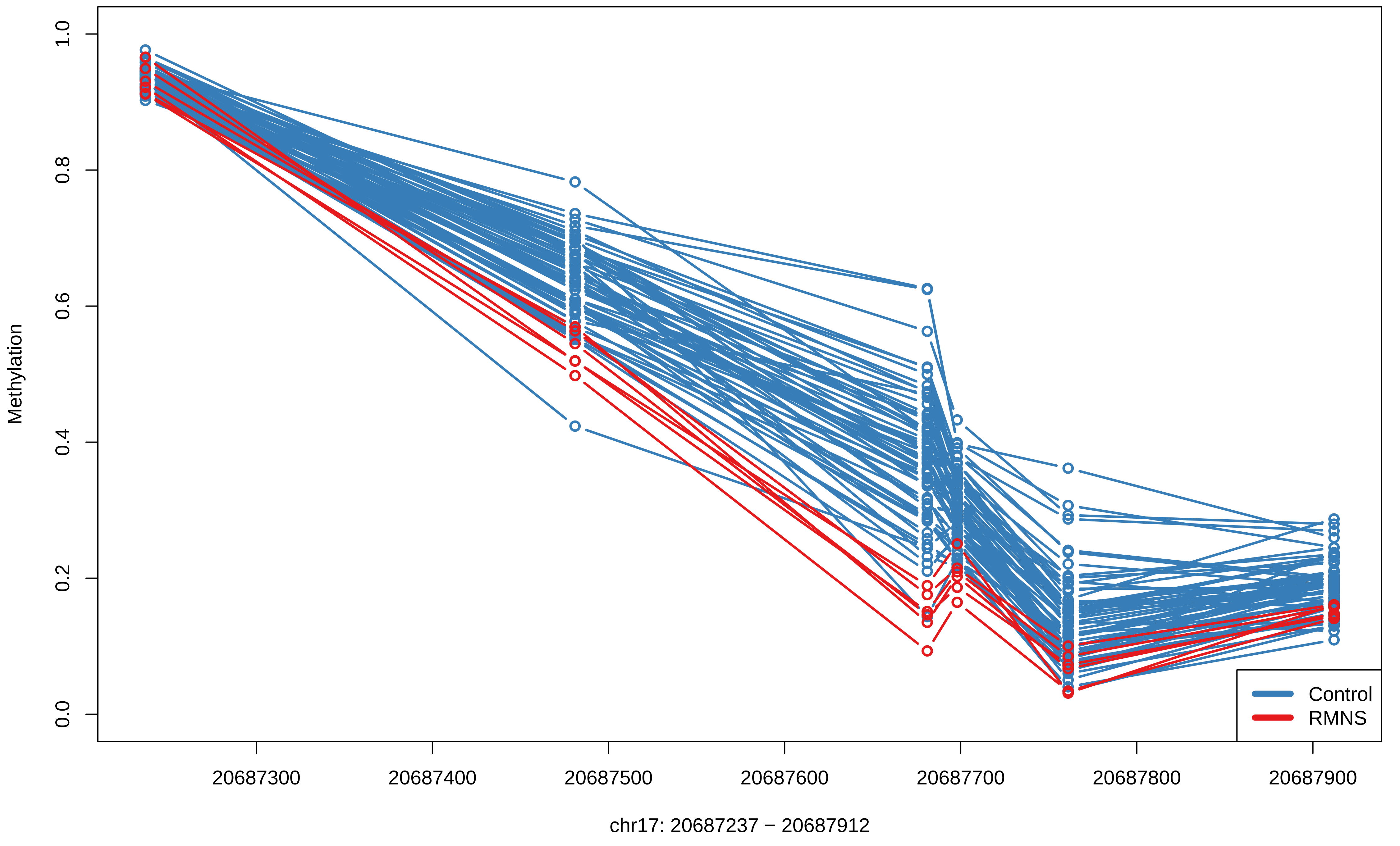

Methylation
1.0
0.8
0.6
0.4
0.2
0.0
20687300
20687400
20687500
20687600
20687700
20687800
20687900
chr17: 20687237 − 20687912
Control
RMNS

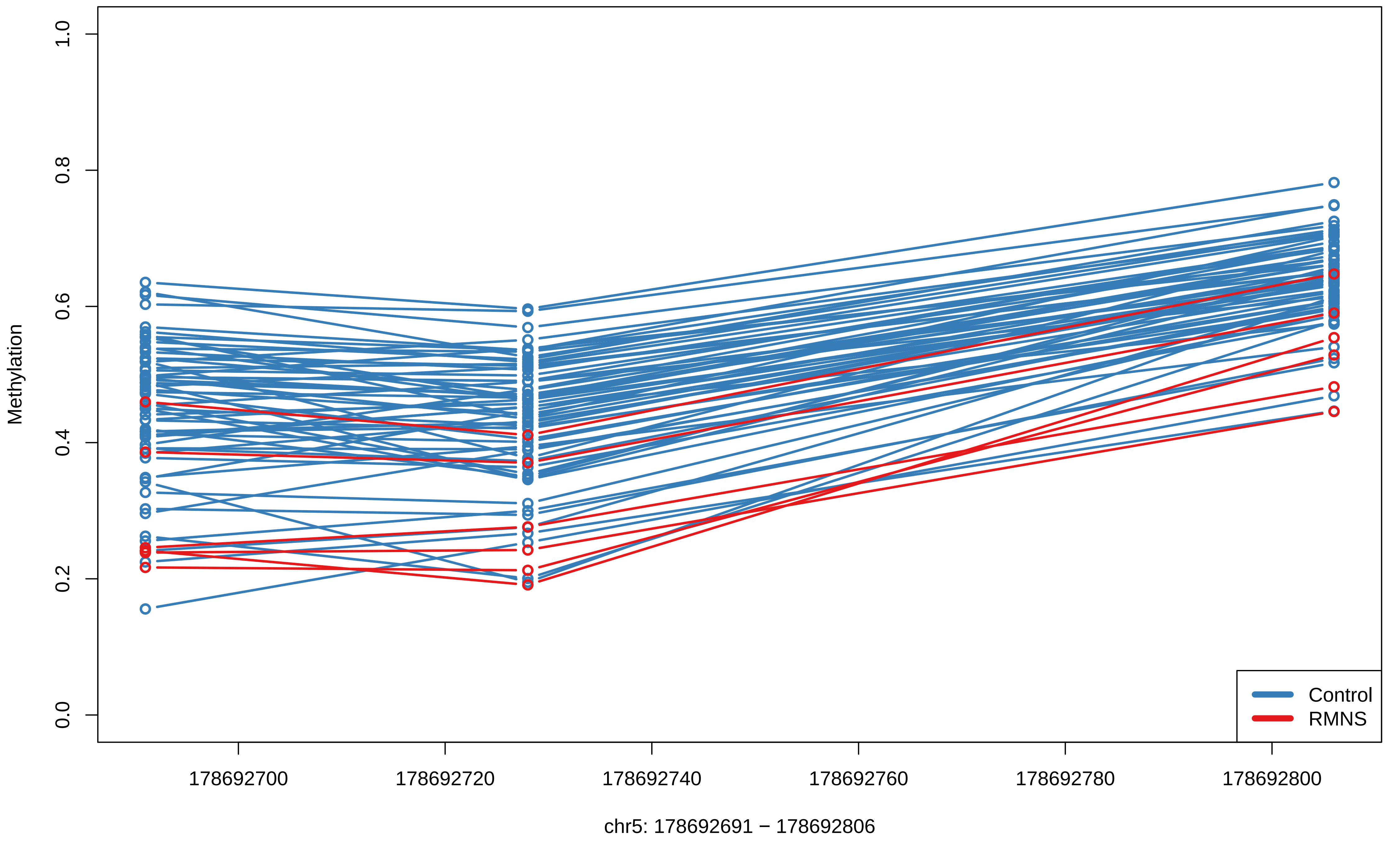

Methylation
0.0
0.2
0.4
0.6
0.8
1.0
178692700
178692720
178692740
178692760
178692780
178692800
chr5: 178692691 − 178692806
Control
RMNS

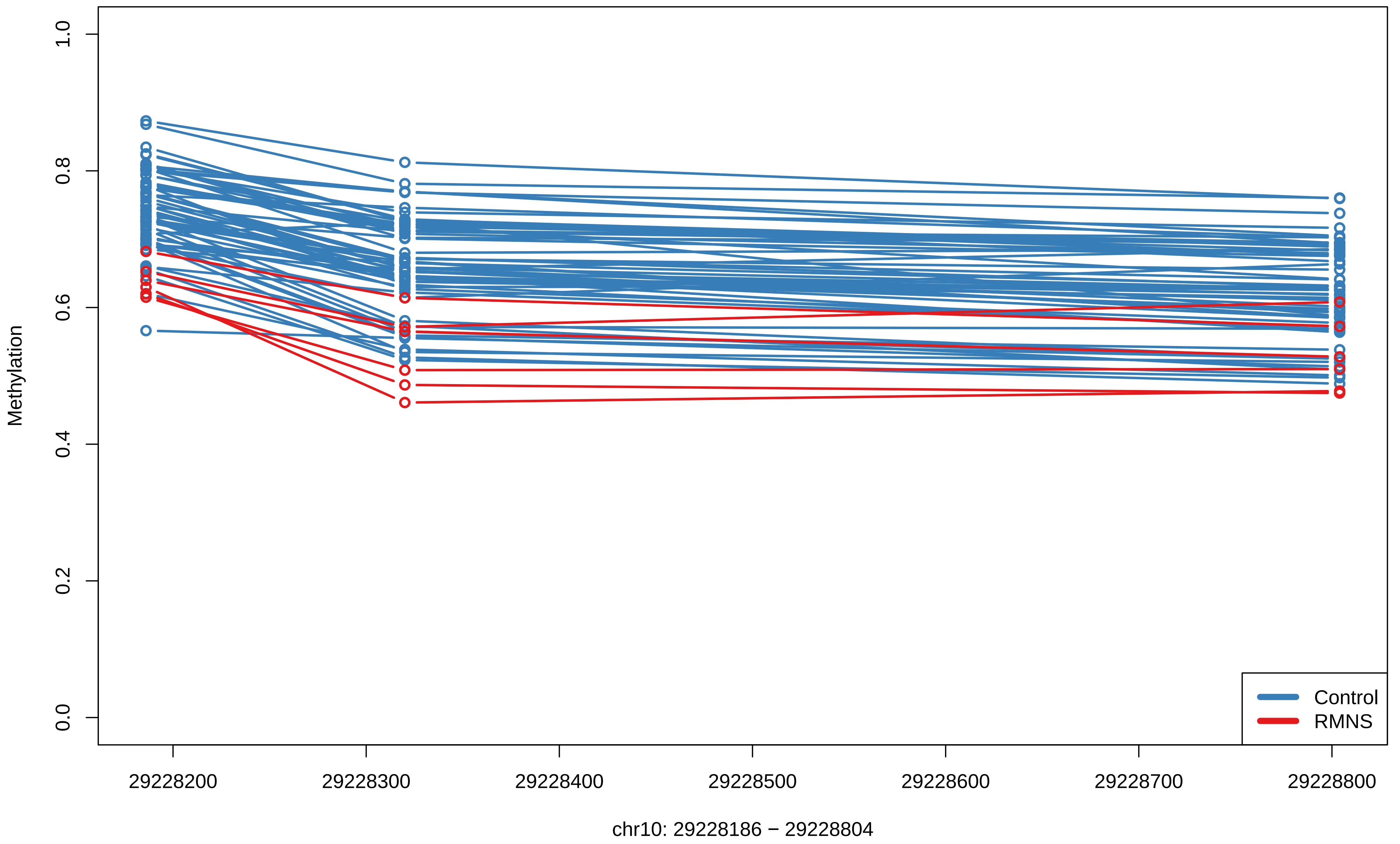

Methylation
0.0
0.2
0.4
0.6
0.8
1.0
29228200
29228300
29228400
29228500
29228600
29228700
29228800
chr10: 29228186 − 29228804
Control
RMNS

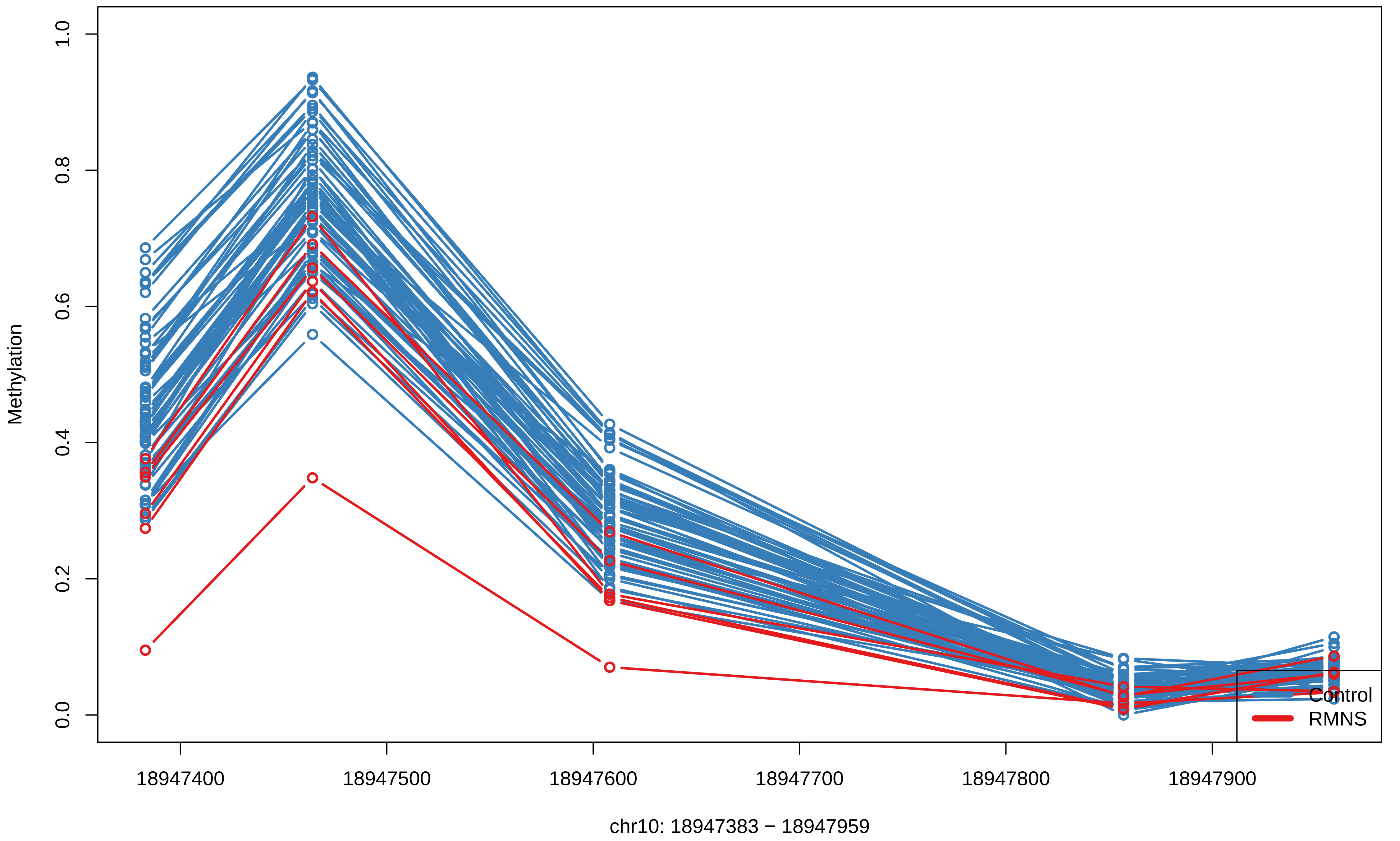

Methylation
1.0
0.8
0.6
0.4
0.2
0.0
18947400
18947500
18947600
18947700
18947800
18947900
chr10: 18947383 − 18947959
Control
RMNS

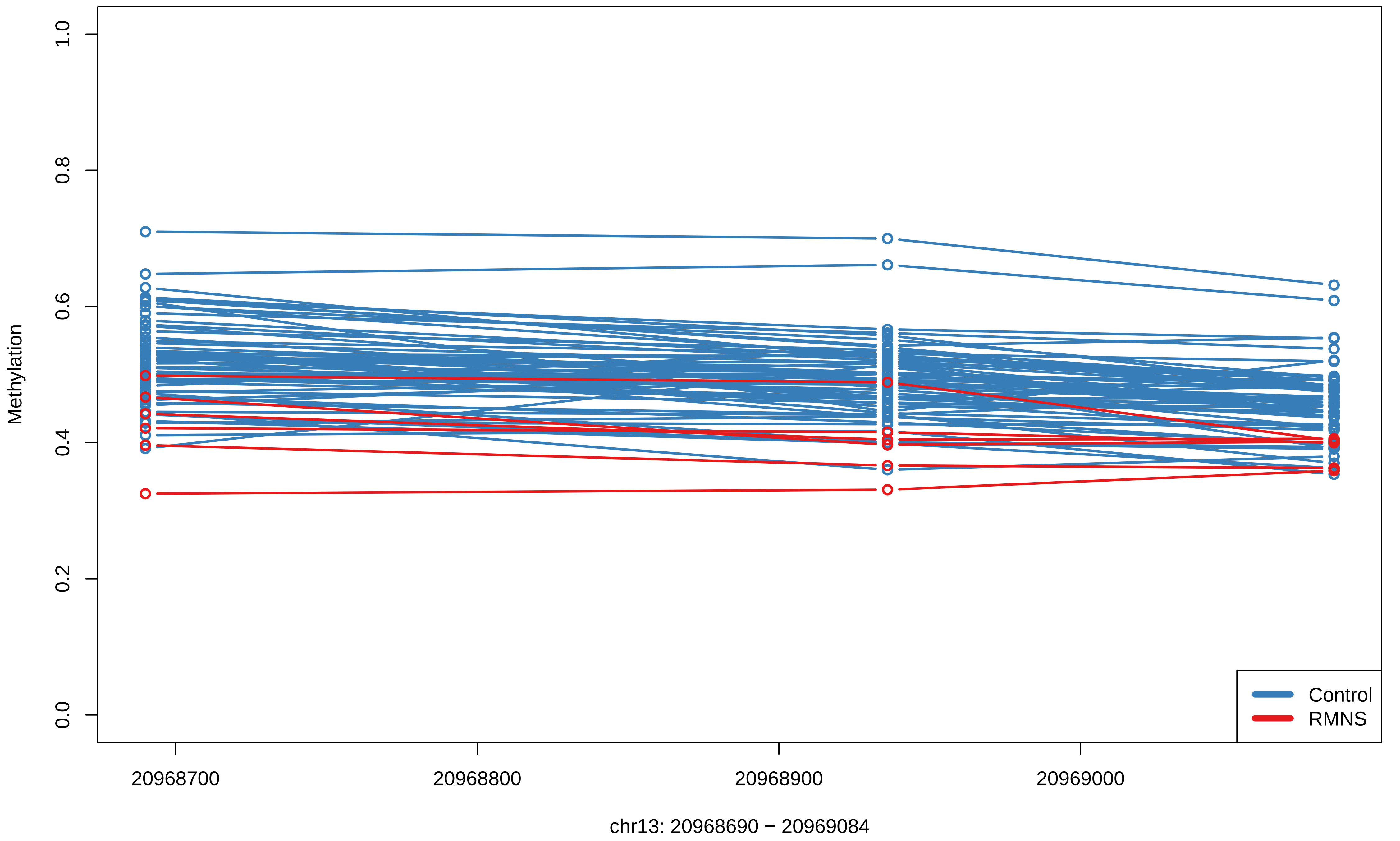

Methylation
0.0
0.2
0.4
0.6
0.8
1.0
20968700
20968800
20968900
20969000
chr13: 20968690 − 20969084
Control
RMNS

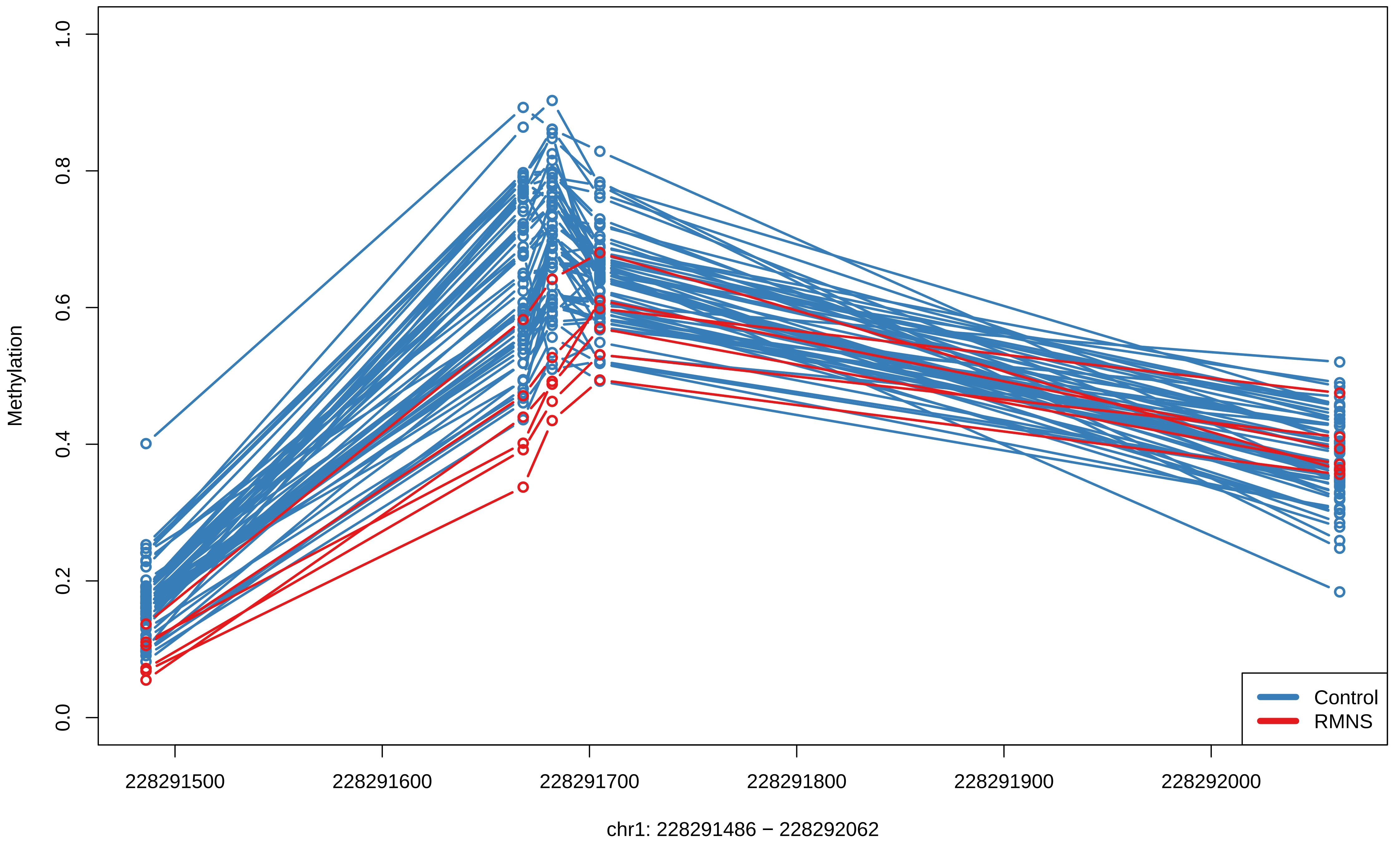

Methylation
0.0
0.2
0.4
0.6
0.8
1.0
228291500
228291600
228291700
228291800
228291900
228292000
chr1: 228291486 − 228292062
Control
RMNS

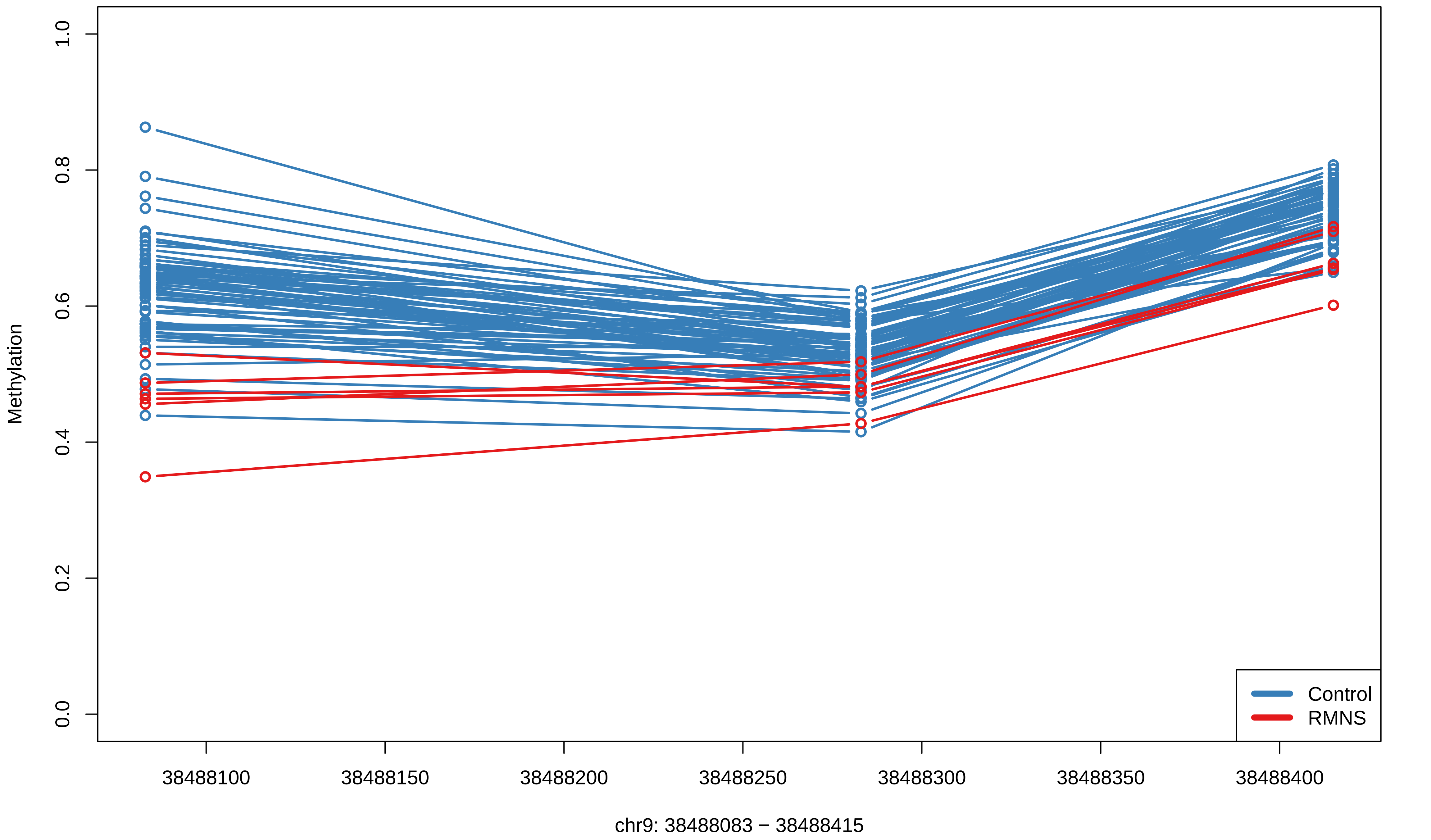

Methylation
1.0
0.8
0.6
0.4
0.2
0.0
38488100
38488150
38488200
38488250
38488300
38488350
38488400
chr9: 38488083 − 38488415
Control
RMNS

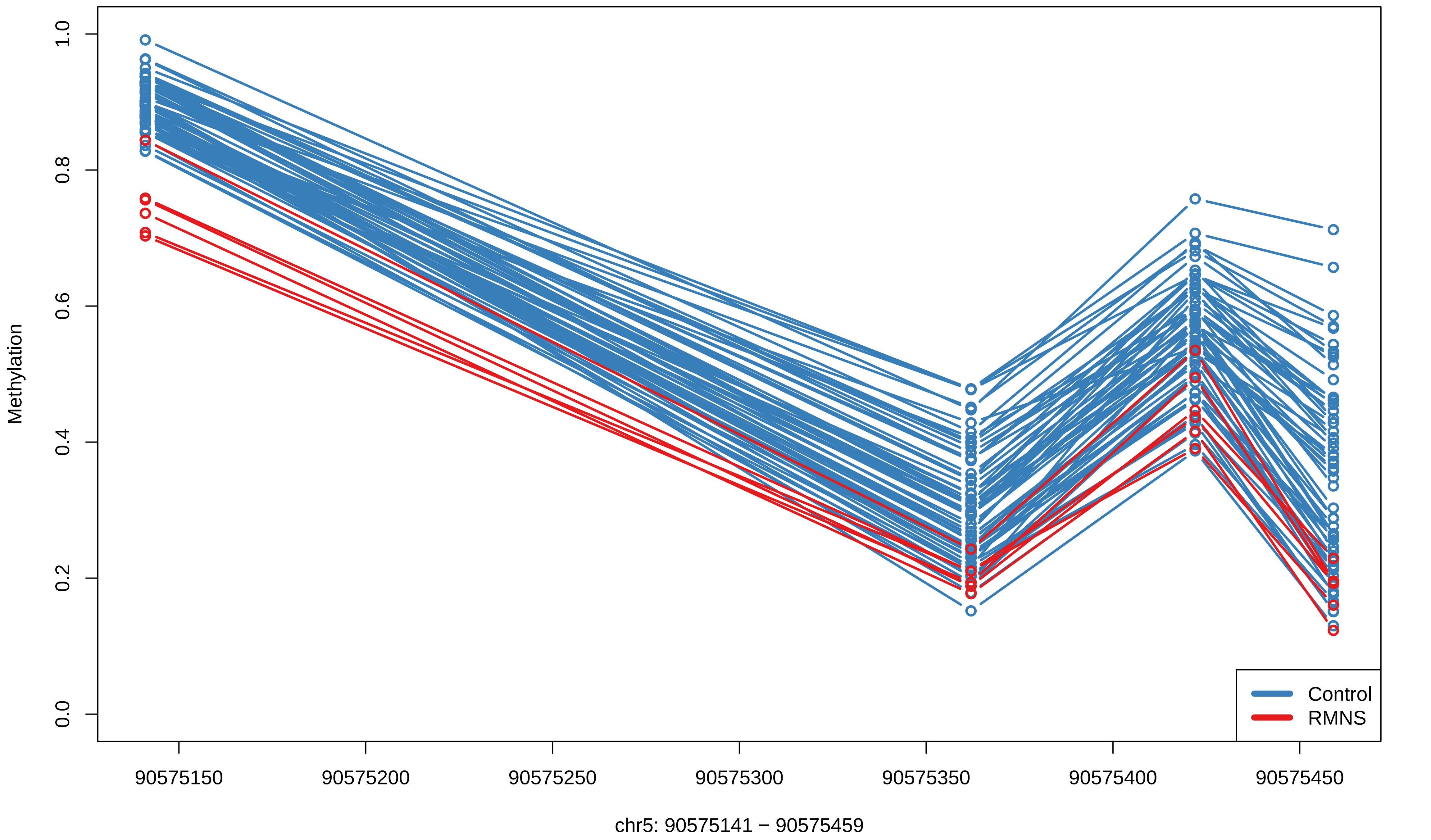

Methylation
1.0
0.8
0.6
0.4
0.2
0.0
90575150
90575200
90575250
90575300
90575350
90575400
90575450
chr5: 90575141 − 90575459
Control
RMNS

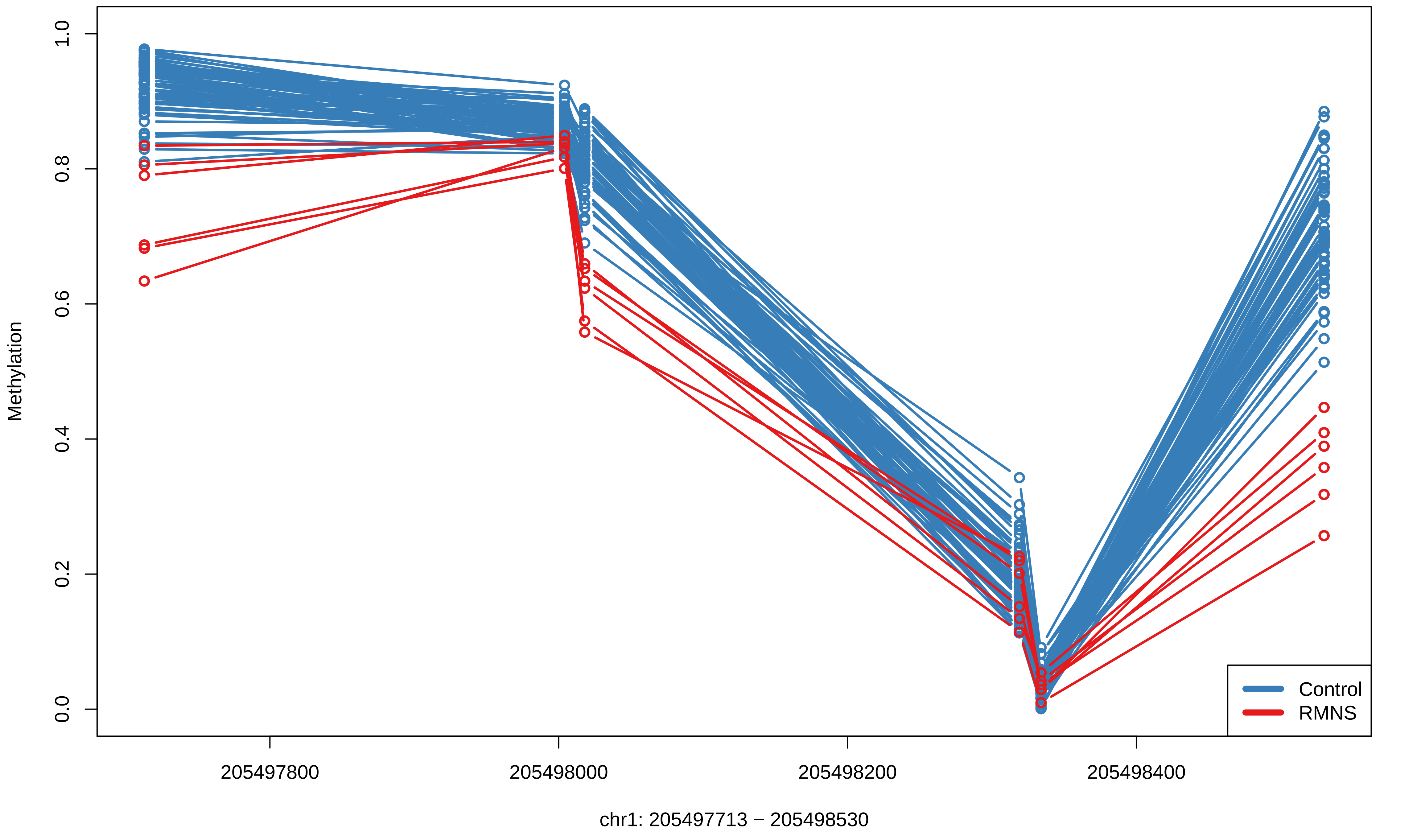
1.0
0.8
0.6
0.4
0.2
0.0
Methylation
205497800
205498000
205498200
205498400
chr1: 205497713 − 205498530
Control
RMNS

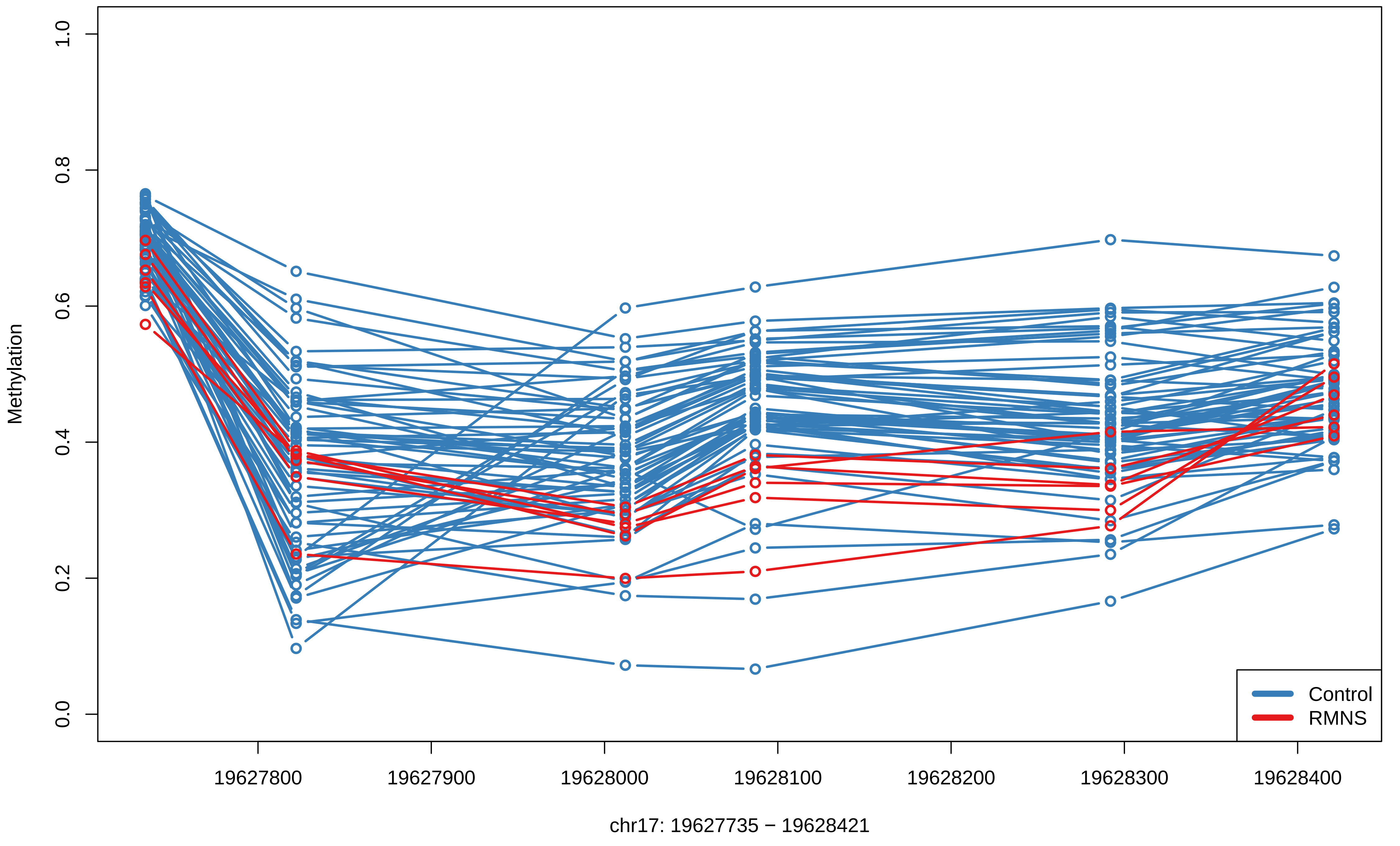

Methylation
1.0
0.8
0.6
0.4
0.2
0.0
19627800
19627900
19628000
19628100
19628200
19628300
19628400
chr17: 19627735 − 19628421
Control
RMNS

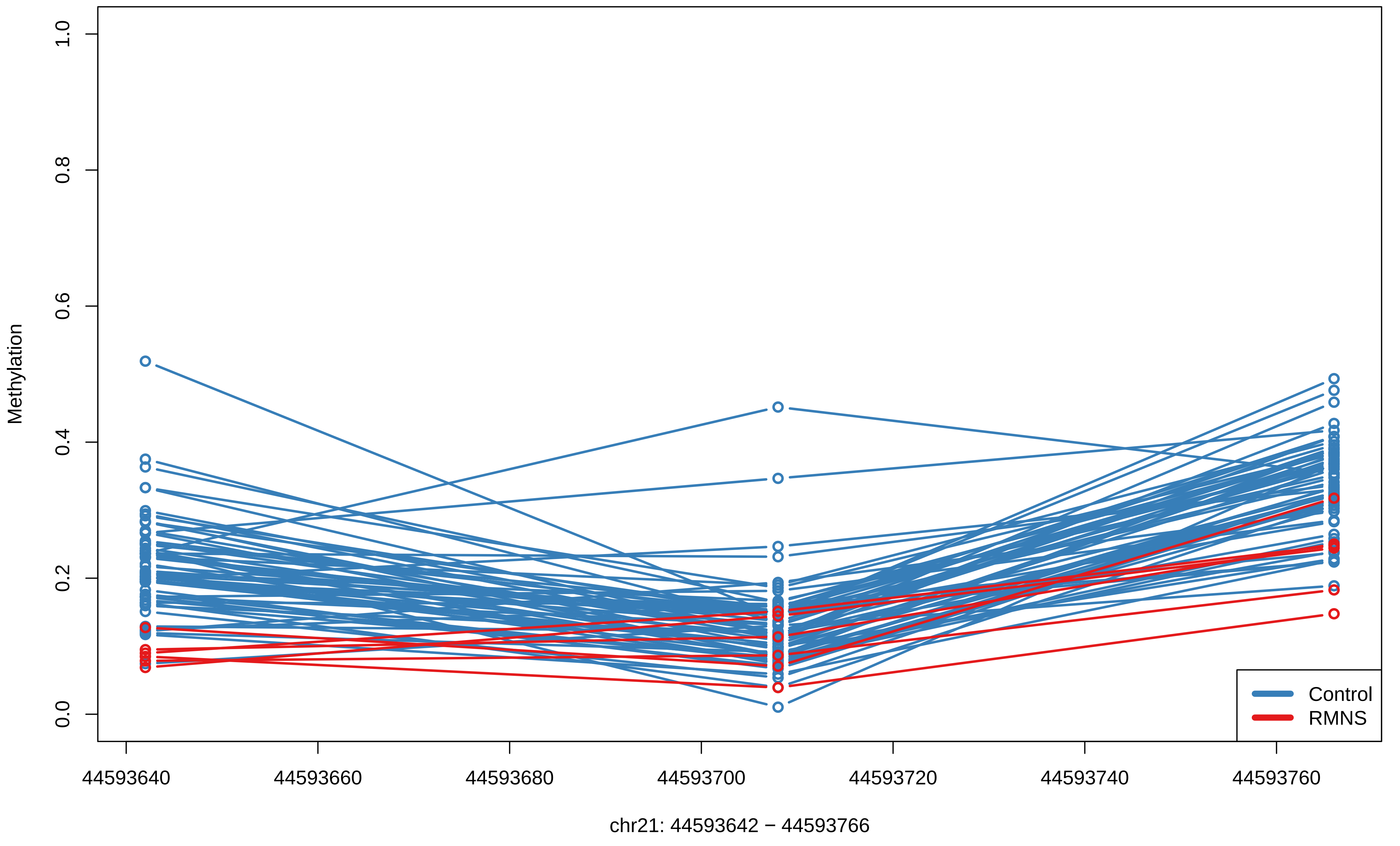

1.0
0.8
0.6
0.4
0.2
0.0
Methylation
44593640
44593660
44593680
44593700
44593720
44593740
44593760
chr21: 44593642 − 44593766
Control
RMNS

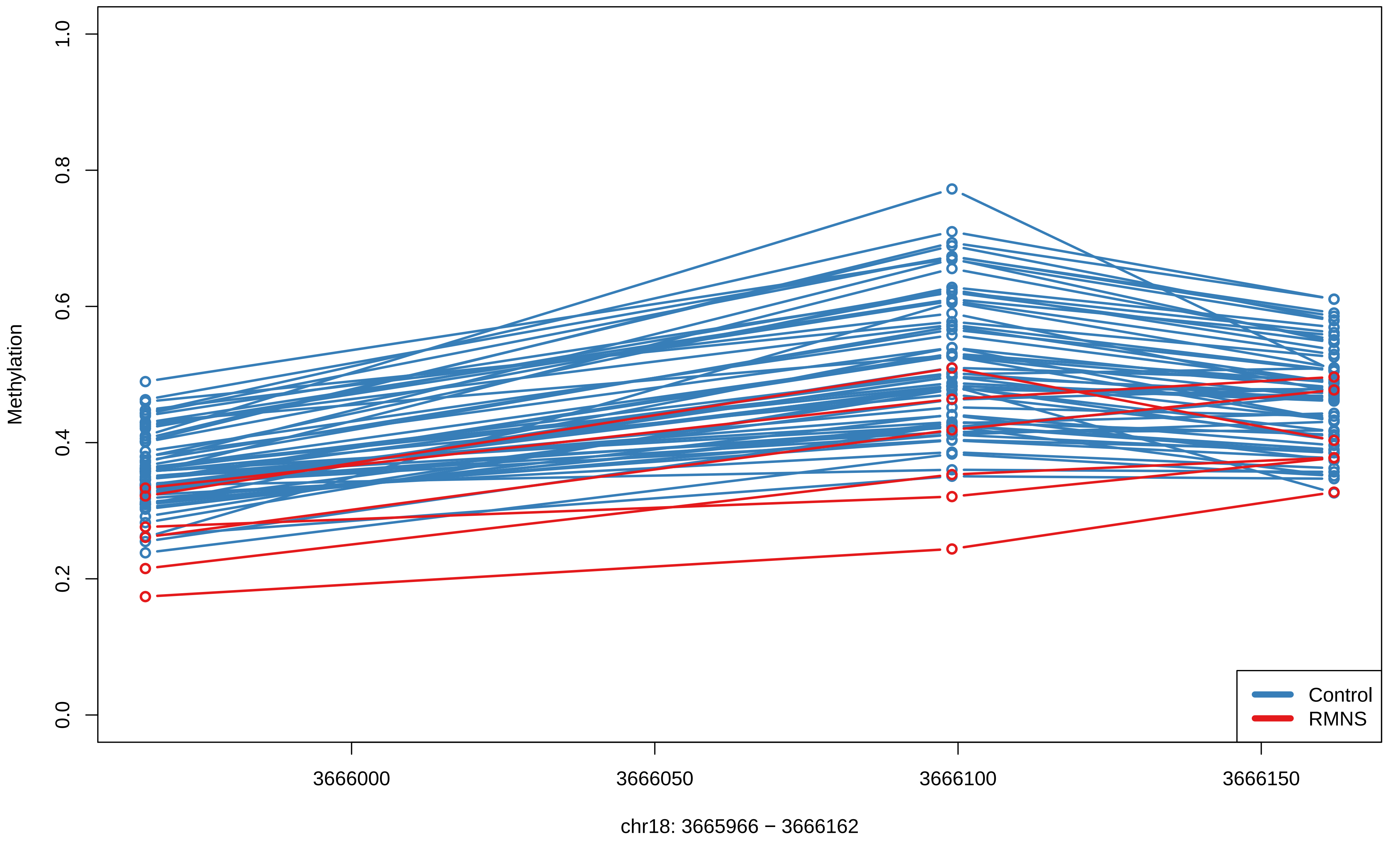

Methylation
0.0
0.2
0.4
0.6
0.8
1.0
3666000
3666050
3666100
3666150
chr18: 3665966 − 3666162
Control
RMNS

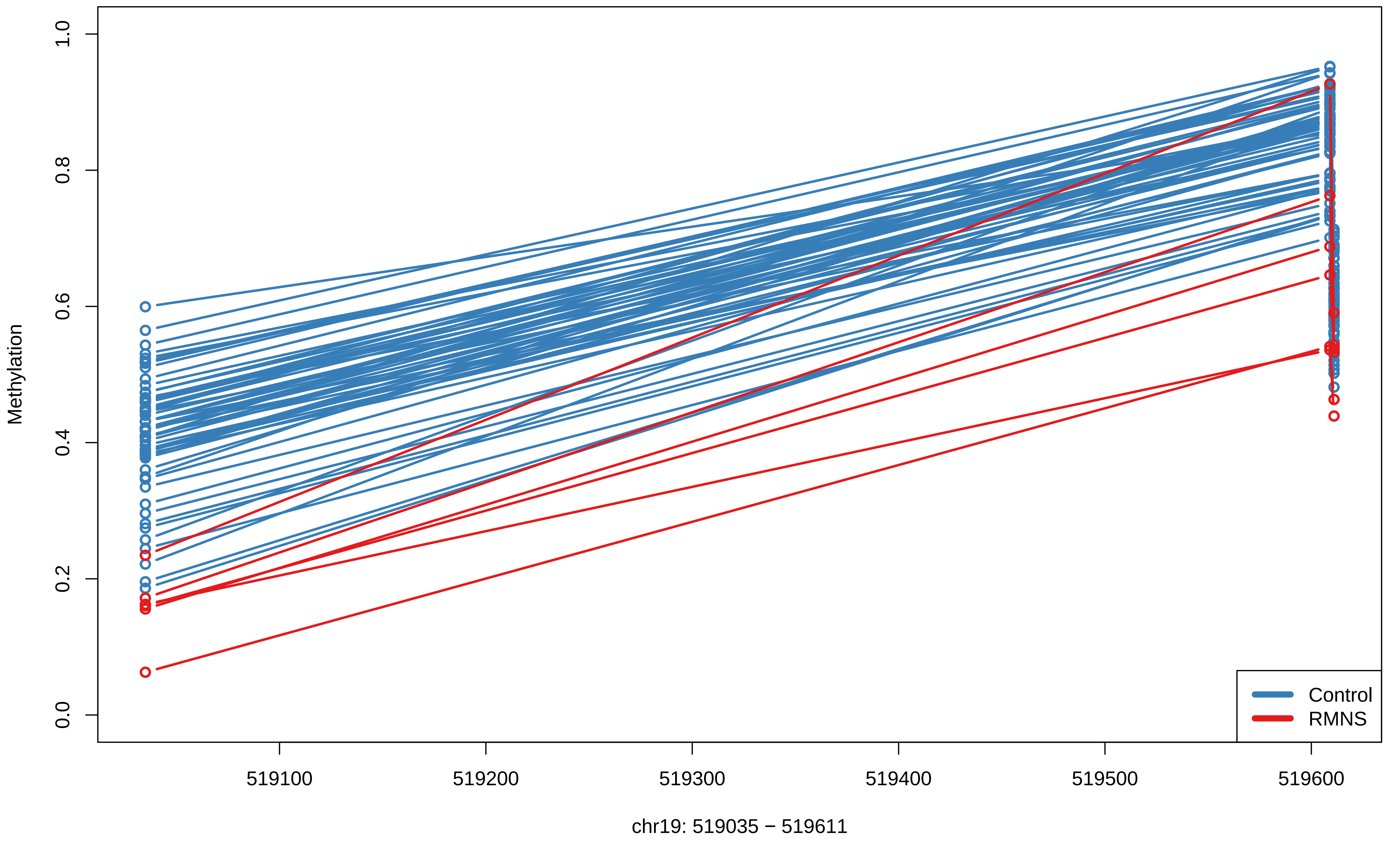

Methylation
1.0
0.8
0.6
0.4
0.2
0.0
519100
519200
519300
519400
519500
519600
chr19: 519035 − 519611
Control
RMNS

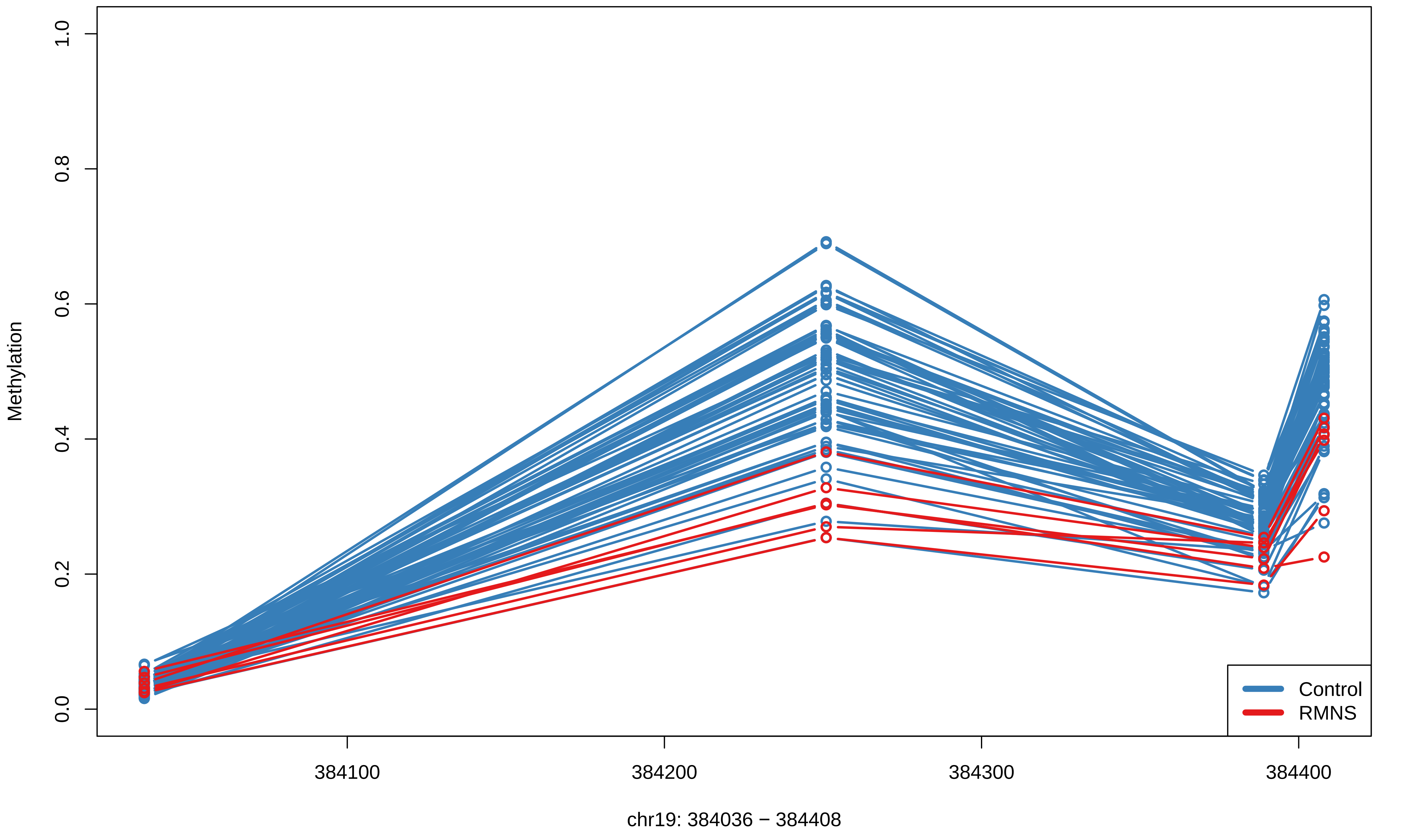

Methylation
0.0
0.2
0.4
0.6
0.8
1.0
384100
384200
384300
384400
chr19: 384036 − 384408
Control
RMNS

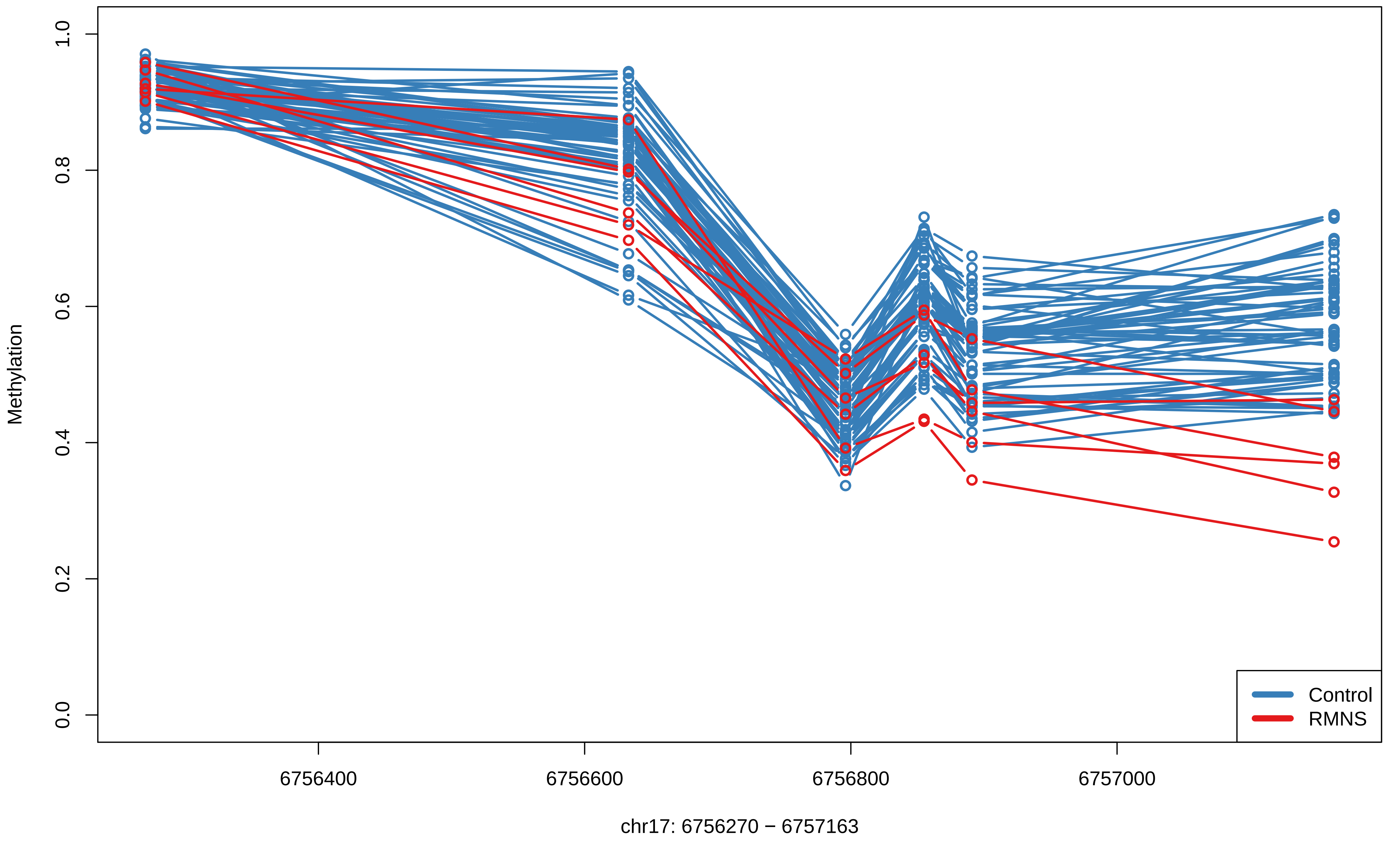

1.0
0.8
0.6
0.4
0.2
0.0
Methylation
6756400
6756600
6756800
6757000
chr17: 6756270 − 6757163
Control
RMNS

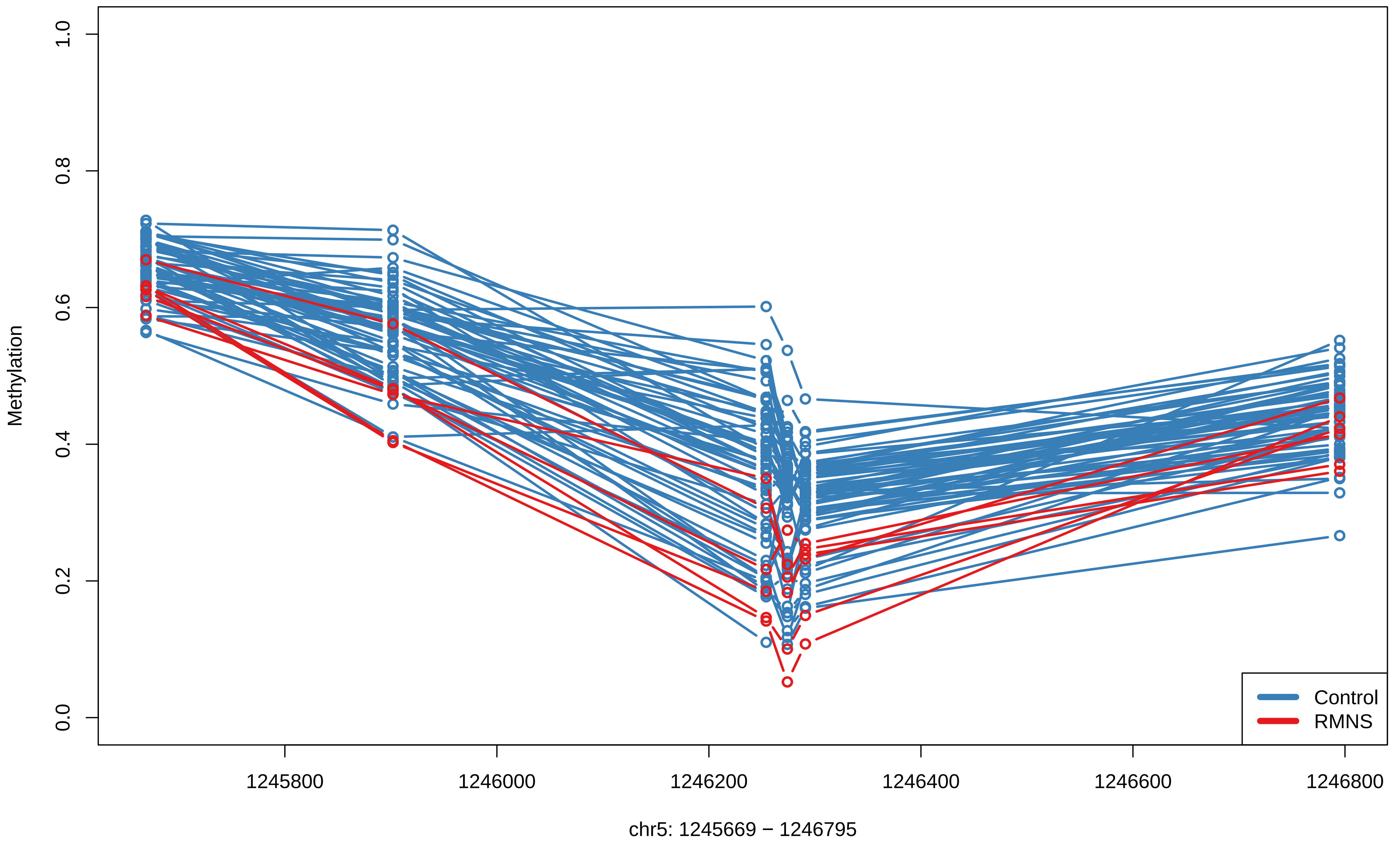

Methylation
1.0
0.8
0.6
0.4
0.2
0.0
1245800
1246000
1246200
1246400
1246600
1246800
chr5: 1245669 − 1246795
Control
RMNS

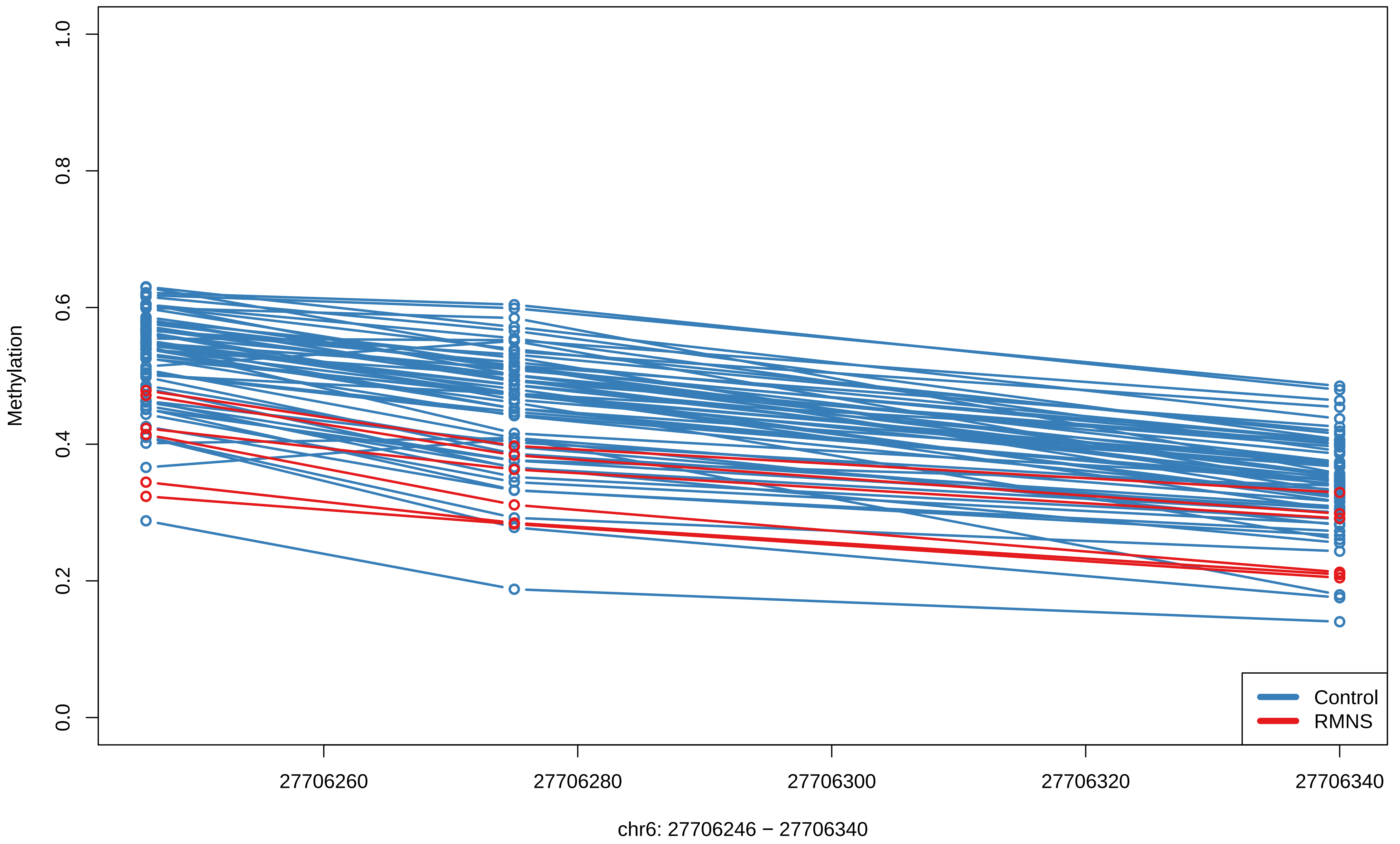

Methylation
1.0
0.8
0.6
0.4
0.2
0.0
27706260
27706280
27706300
27706320
27706340
chr6: 27706246 − 27706340
Control
RMNS

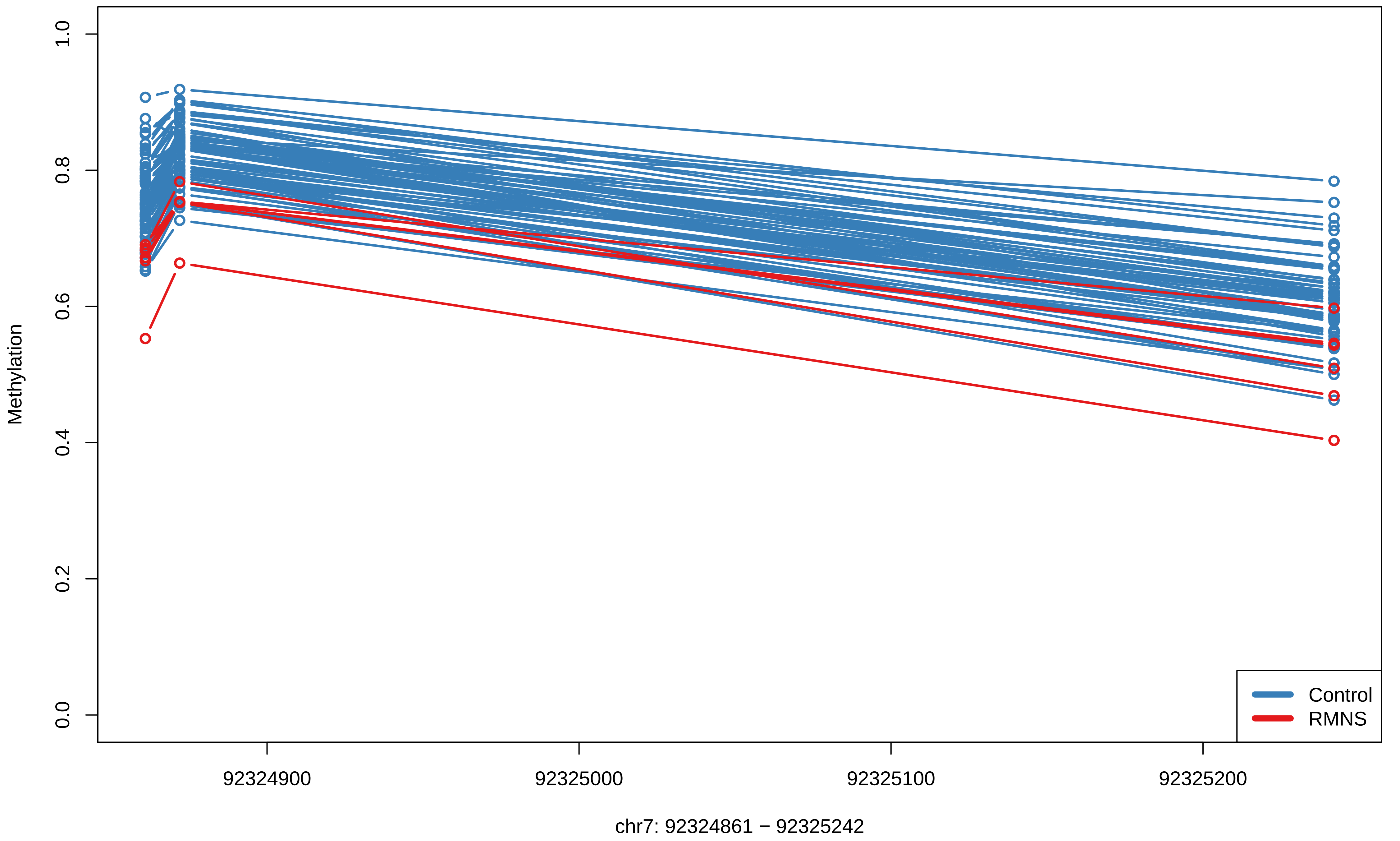

Methylation
1.0
0.8
0.6
0.4
0.2
0.0
92324900
92325000
92325100
92325200
chr7: 92324861 − 92325242
Control
RMNS

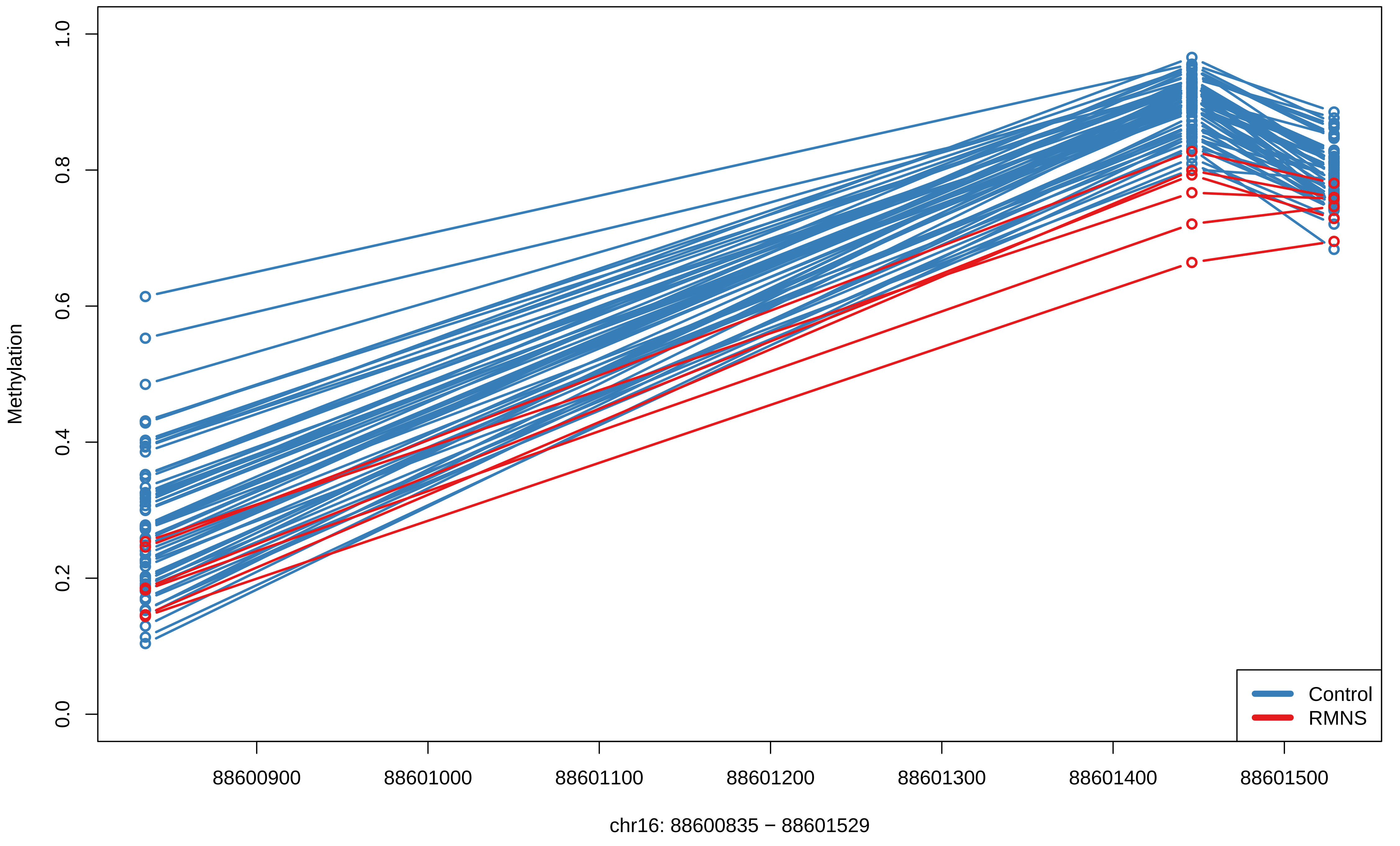

1.0
0.8
0.6
0.4
0.2
0.0
Methylation
88600900
88601000
88601100
88601200
88601300
88601400
88601500
chr16: 88600835 − 88601529
Control
RMNS

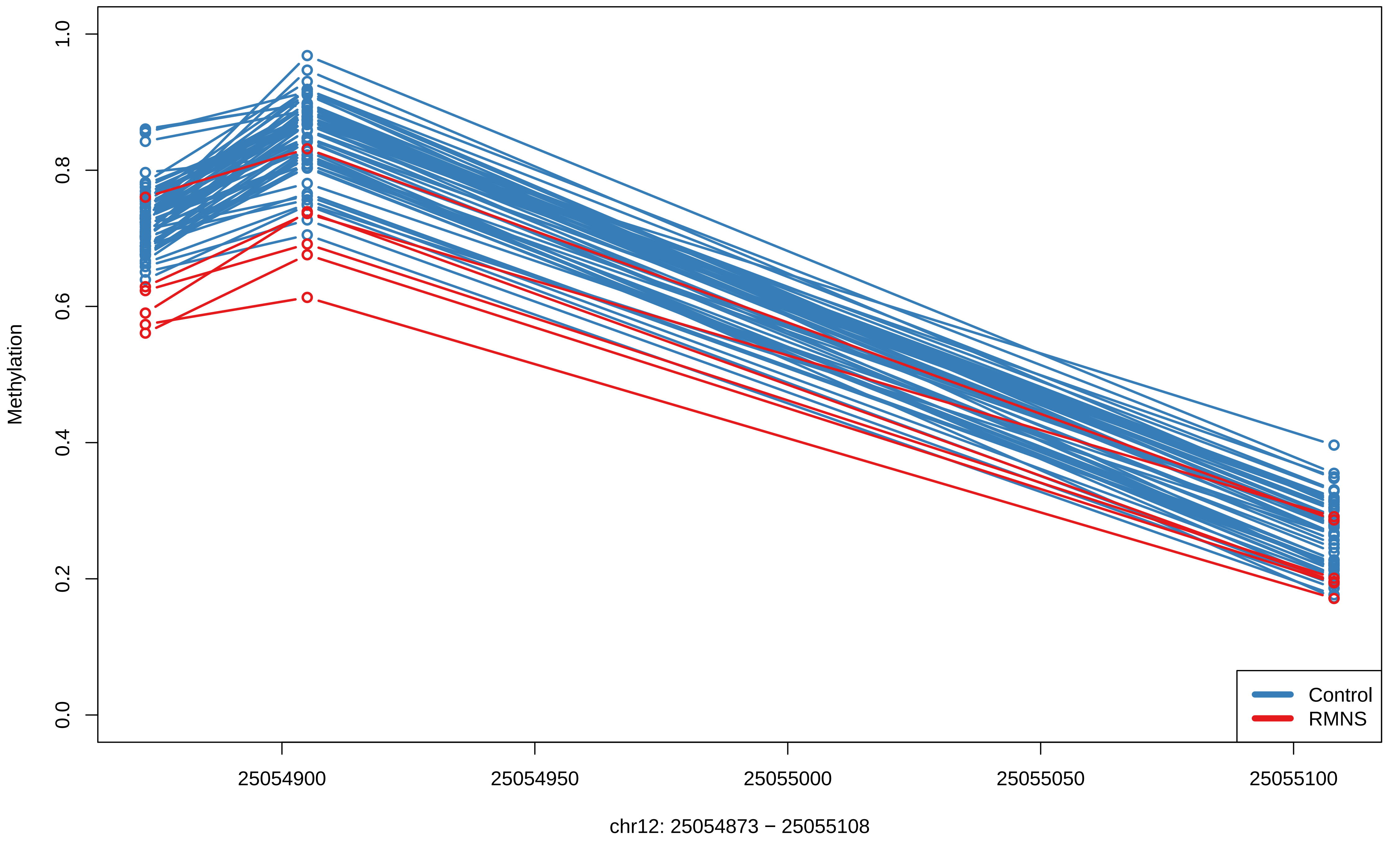

Methylation
0.0
0.2
0.4
0.6
0.8
1.0
25054900
25054950
25055000
25055050
25055100
chr12: 25054873 − 25055108
Control
RMNS

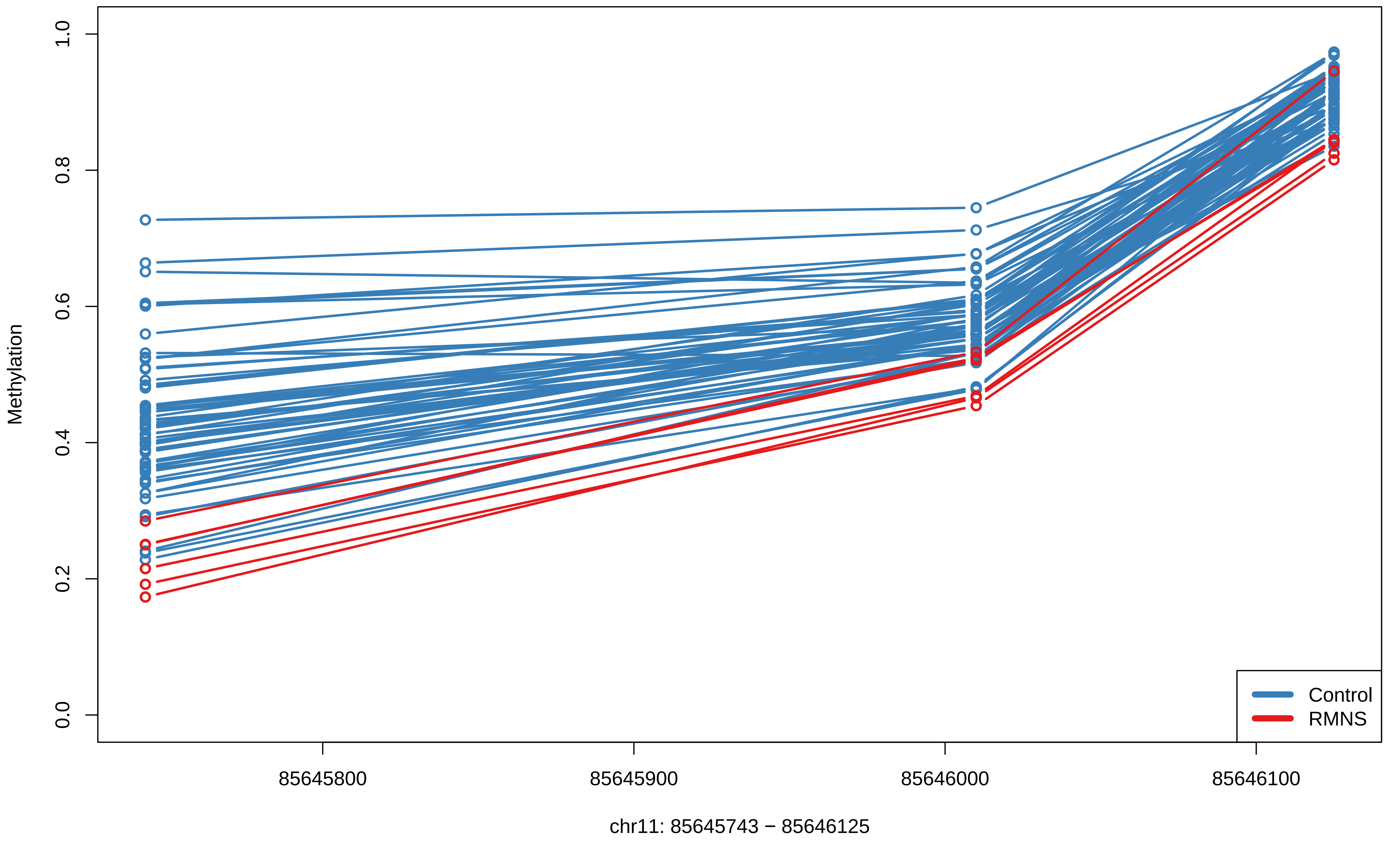

Methylation
0.0
0.2
0.4
0.6
0.8
1.0
85645800
85645900
85646000
85646100
chr11: 85645743 − 85646125
Control
RMNS

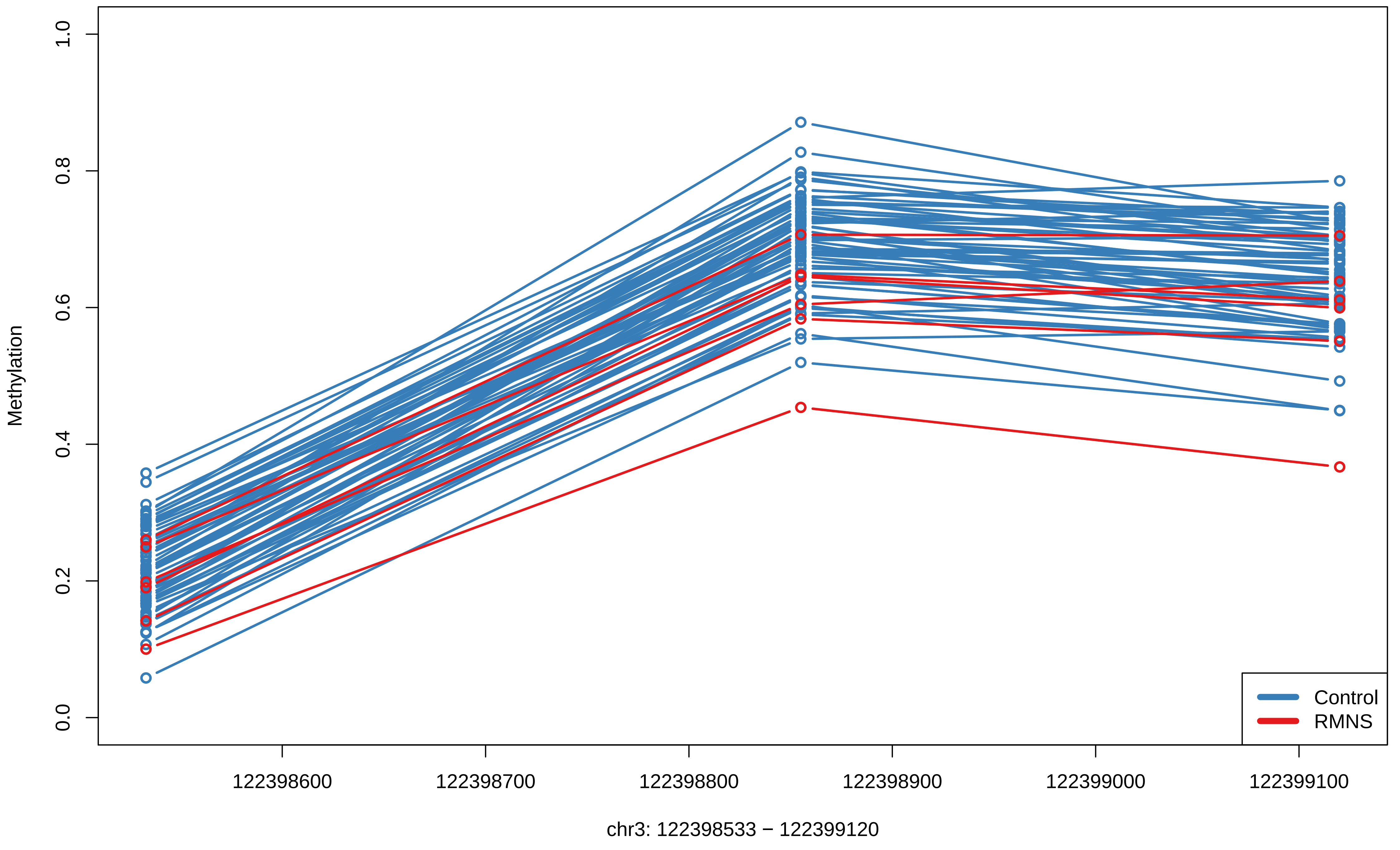

Methylation
0.0
0.2
0.4
0.6
0.8
1.0
122398600
122398700
122398800
122398900
122399000
122399100
chr3: 122398533 − 122399120
Control
RMNS

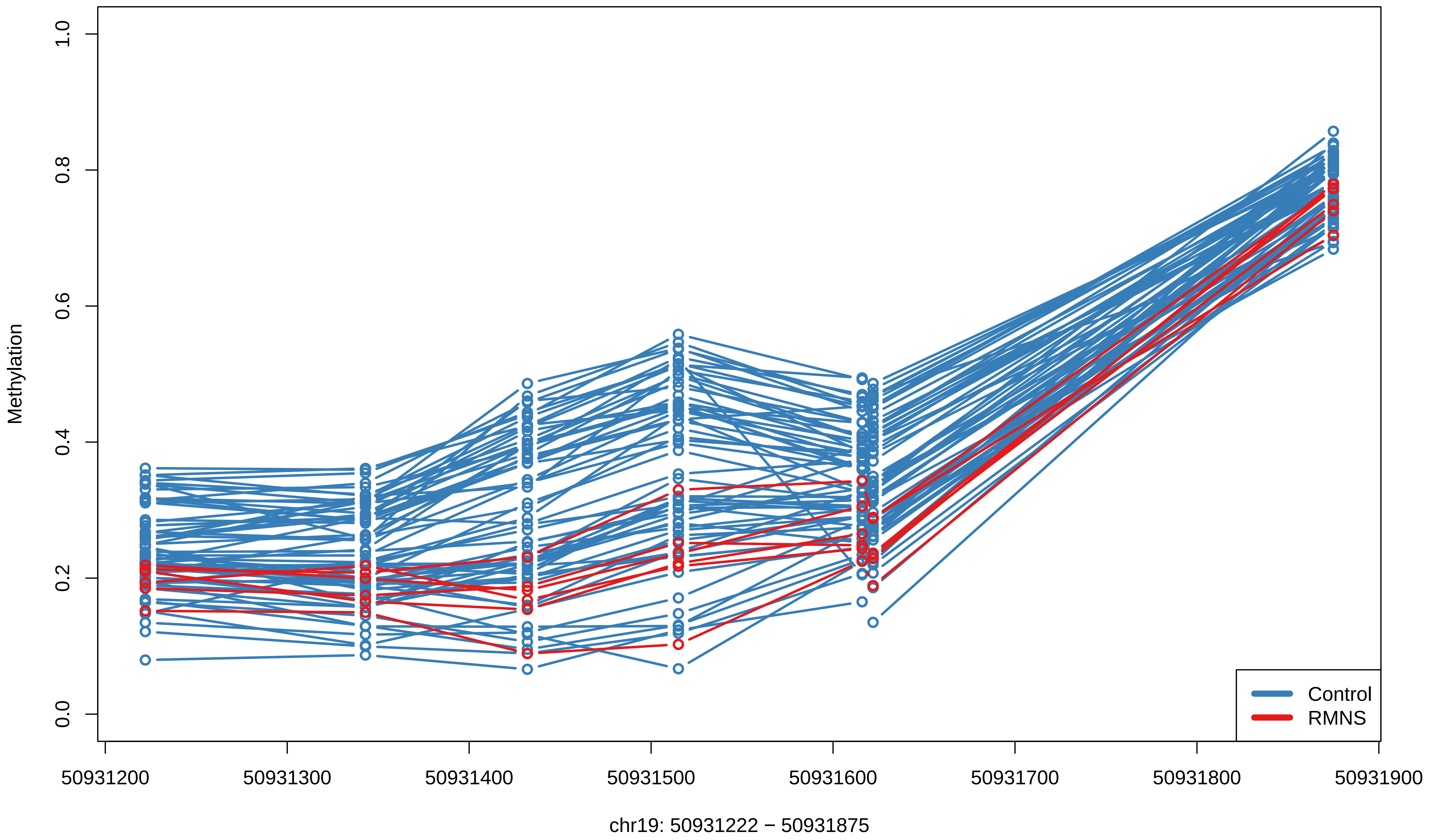

Methylation
0.0
0.2
0.4
0.6
0.8
1.0
50931200
50931300
50931400
50931500
50931600
50931700
50931800
50931900
chr19: 50931222 − 50931875
Control
RMNS

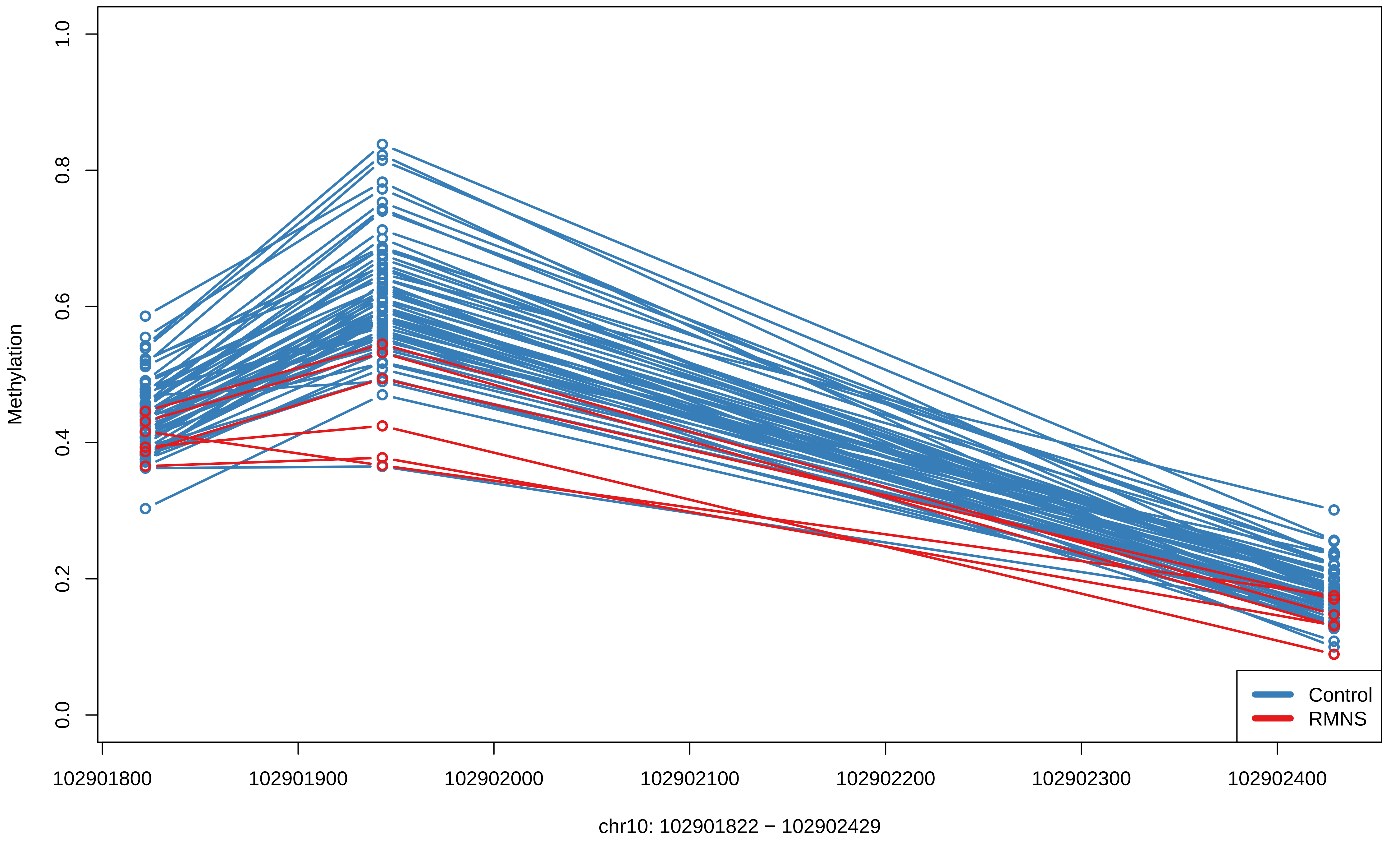
Methylation
0.0
0.2
0.4
0.6
0.8
1.0
102901800
102901900
102902000
102902100
102902200
102902300
102902400
chr10: 102901822 − 102902429
Control
RMNS

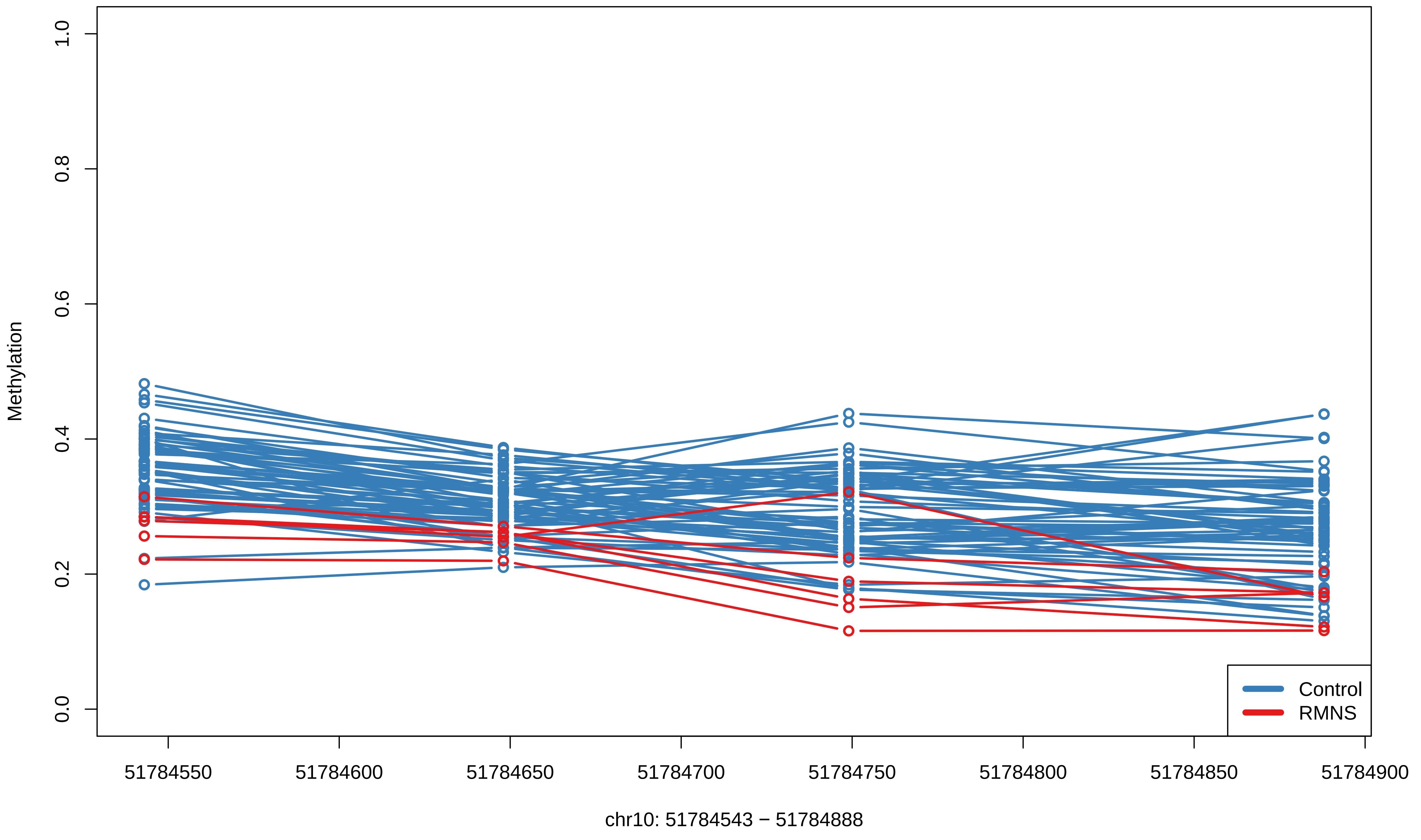
Methylation
0.0
0.2
0.4
0.6
0.8
1.0
51784550
51784600
51784650
51784700
51784750
51784800
51784850
51784900
chr10: 51784543 − 51784888
Control
RMNS

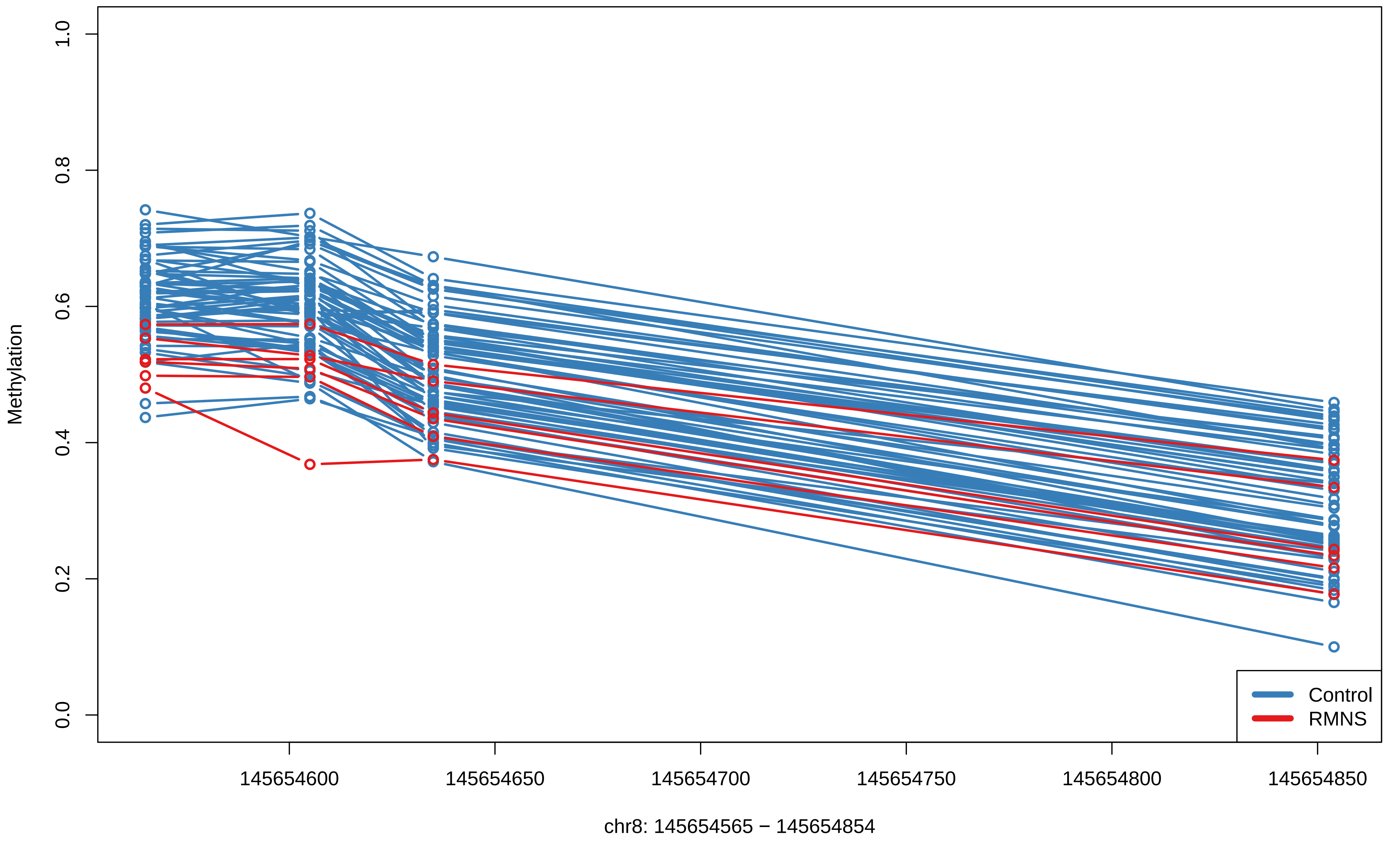

1.0
0.8
0.6
0.4
0.2
0.0
Methylation
145654600
145654650
145654700
145654750
145654800
145654850
chr8: 145654565 − 145654854
Control
RMNS

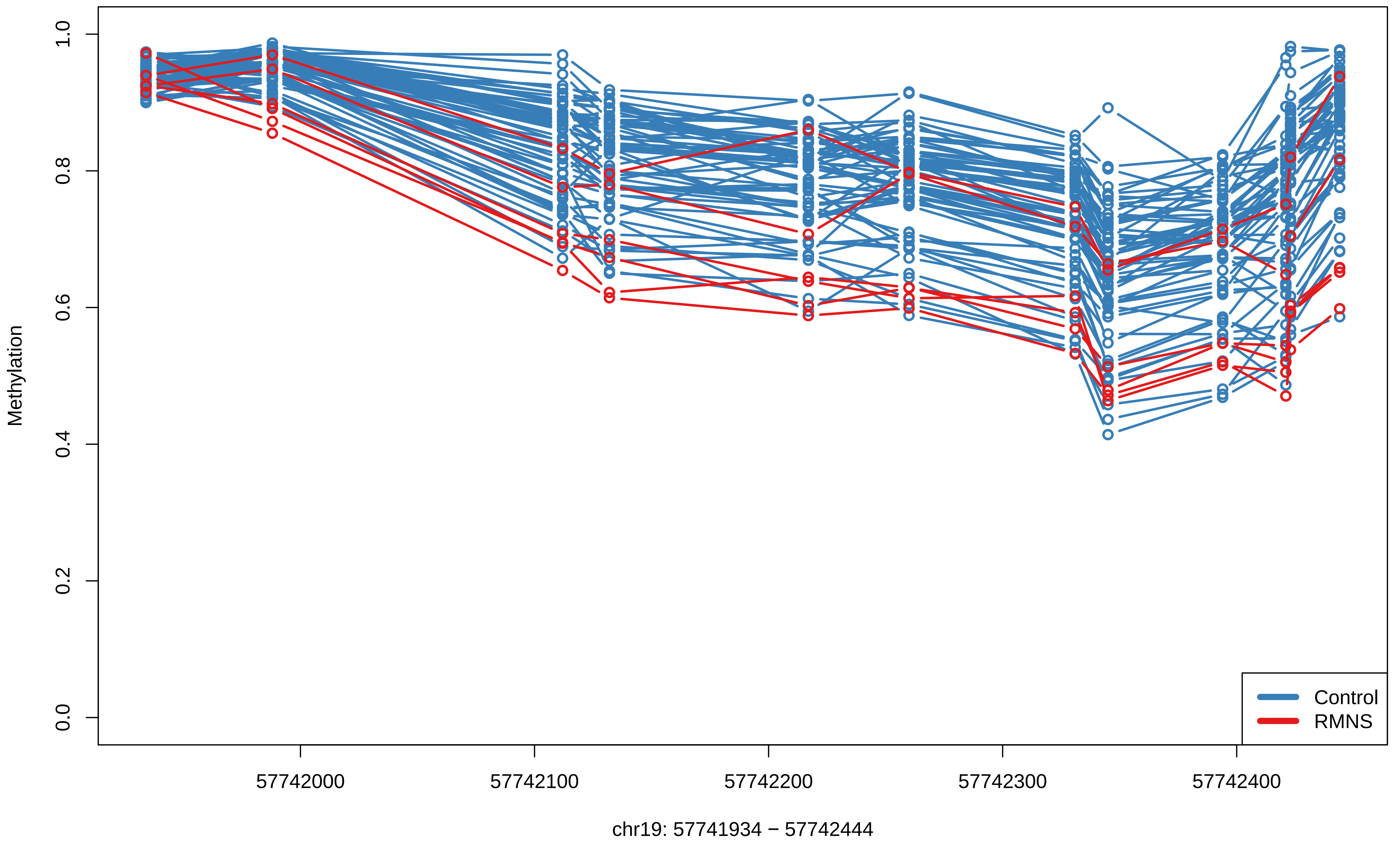
Methylation
1.0
0.8
0.6
0.4
0.2
0.0
57742000
57742100
57742200
57742300
57742400
chr19: 57741934 − 57742444
Control
RMNS

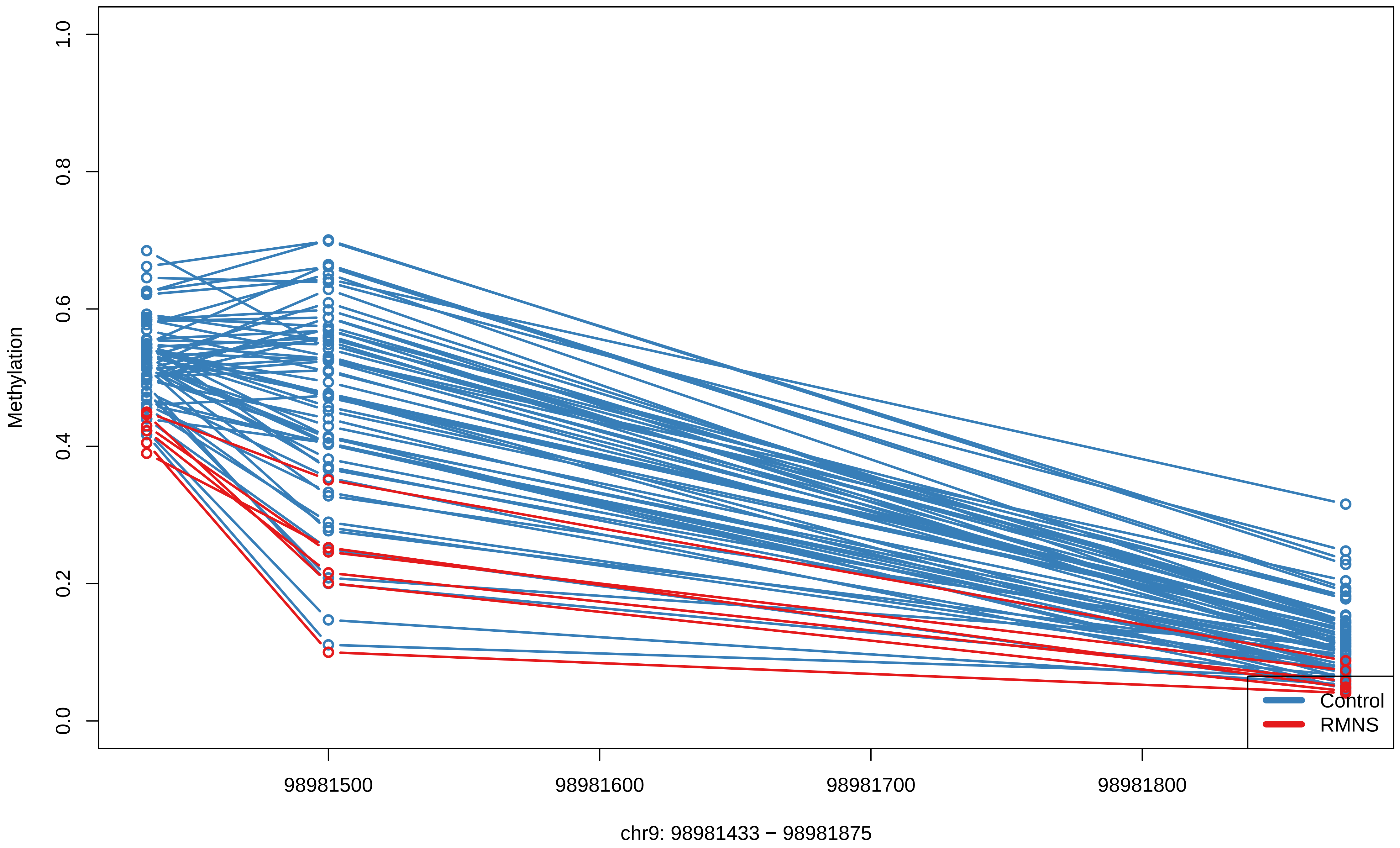

Methylation
0.0
0.2
0.4
0.6
0.8
1.0
98981500
98981600
98981700
98981800
chr9: 98981433 − 98981875
Control
RMNS

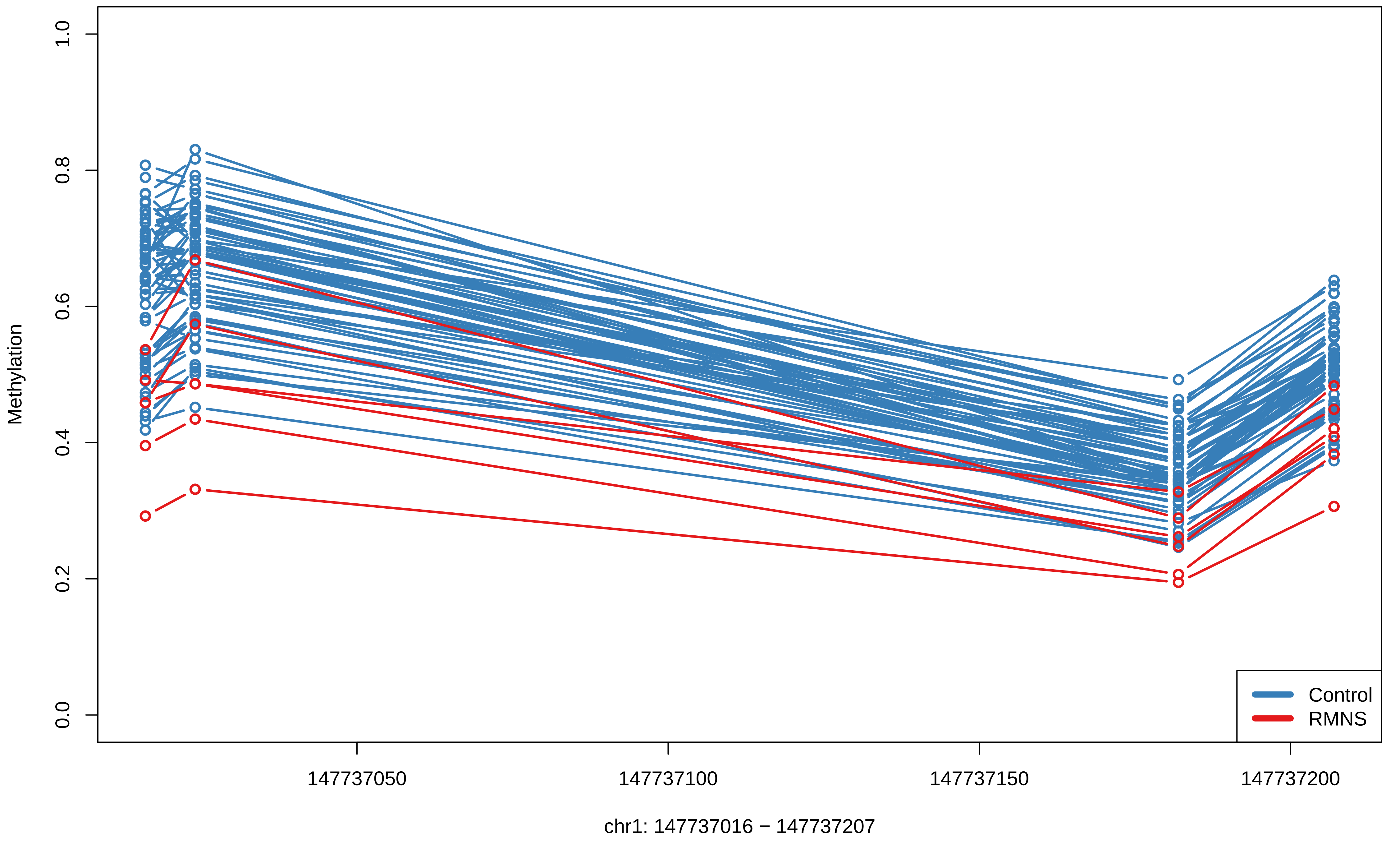

Methylation
0.0
0.2
0.4
0.6
0.8
1.0
147737050
147737100
147737150
147737200
chr1: 147737016 − 147737207
Control
RMNS

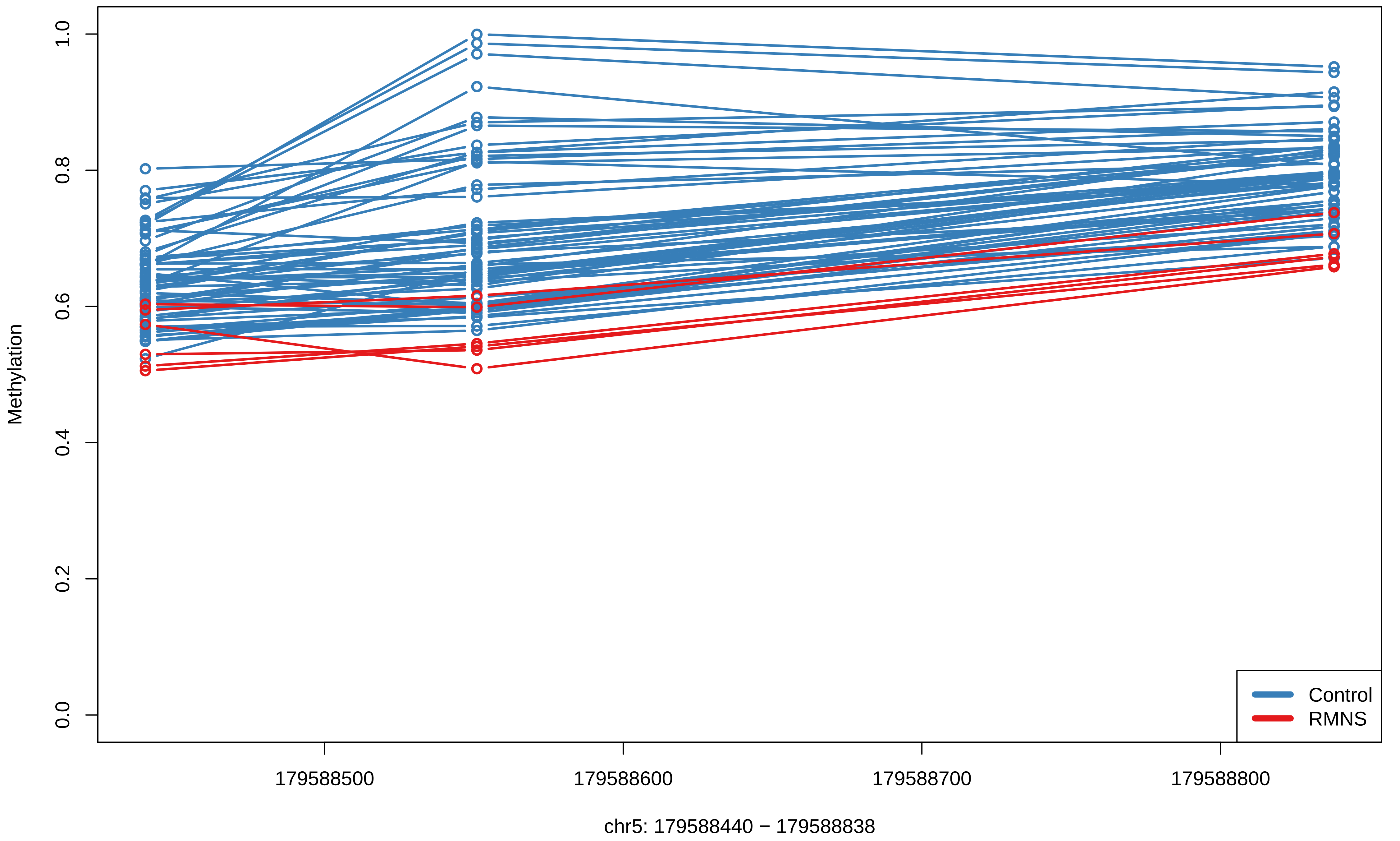

Methylation
0.0
0.2
0.4
0.6
0.8
1.0
179588500
179588600
179588700
179588800
chr5: 179588440 − 179588838
Control
RMNS

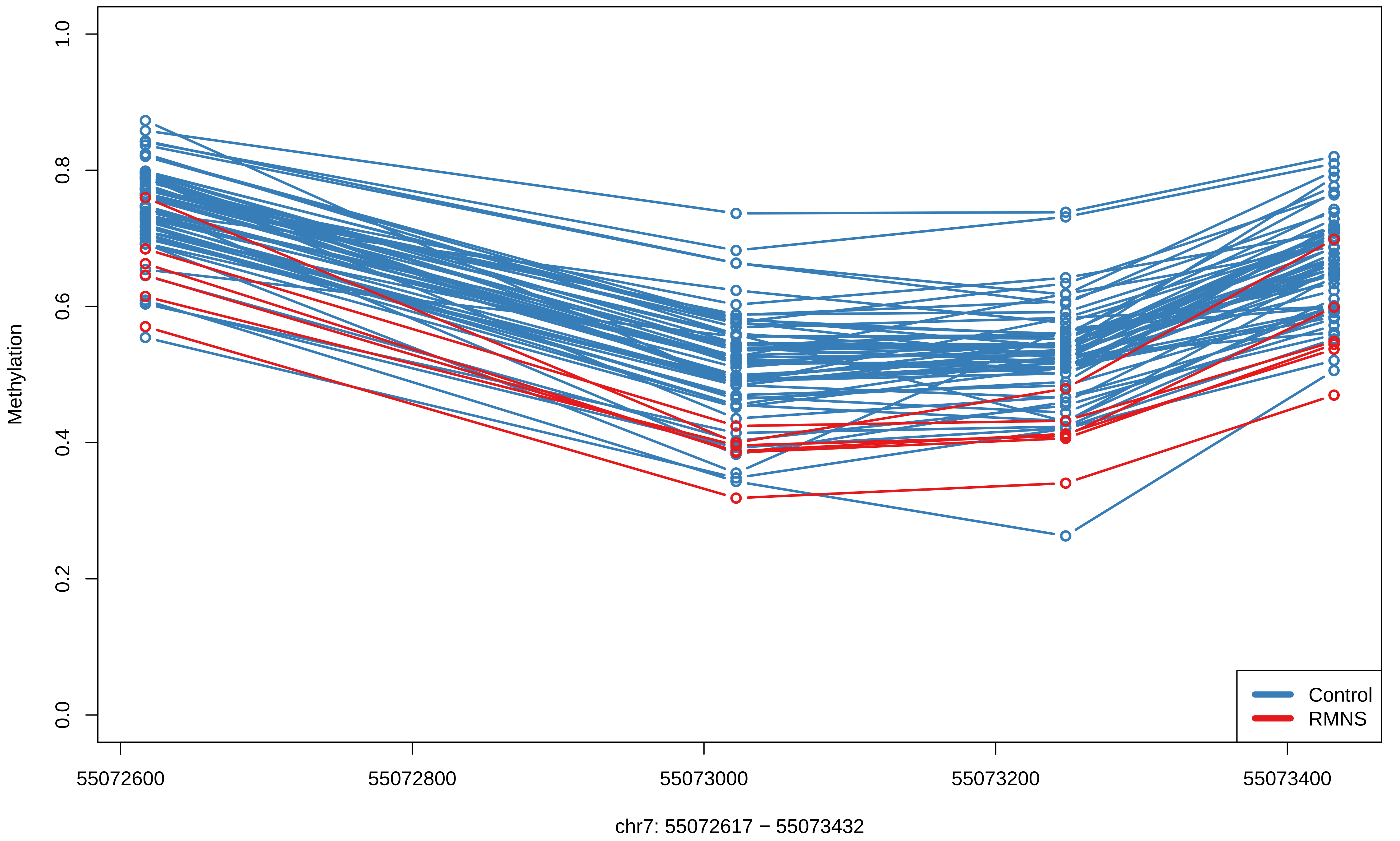

Methylation
0.0
0.2
0.4
0.6
0.8
1.0
55072600
55072800
55073000
55073200
55073400
chr7: 55072617 − 55073432
Control
RMNS

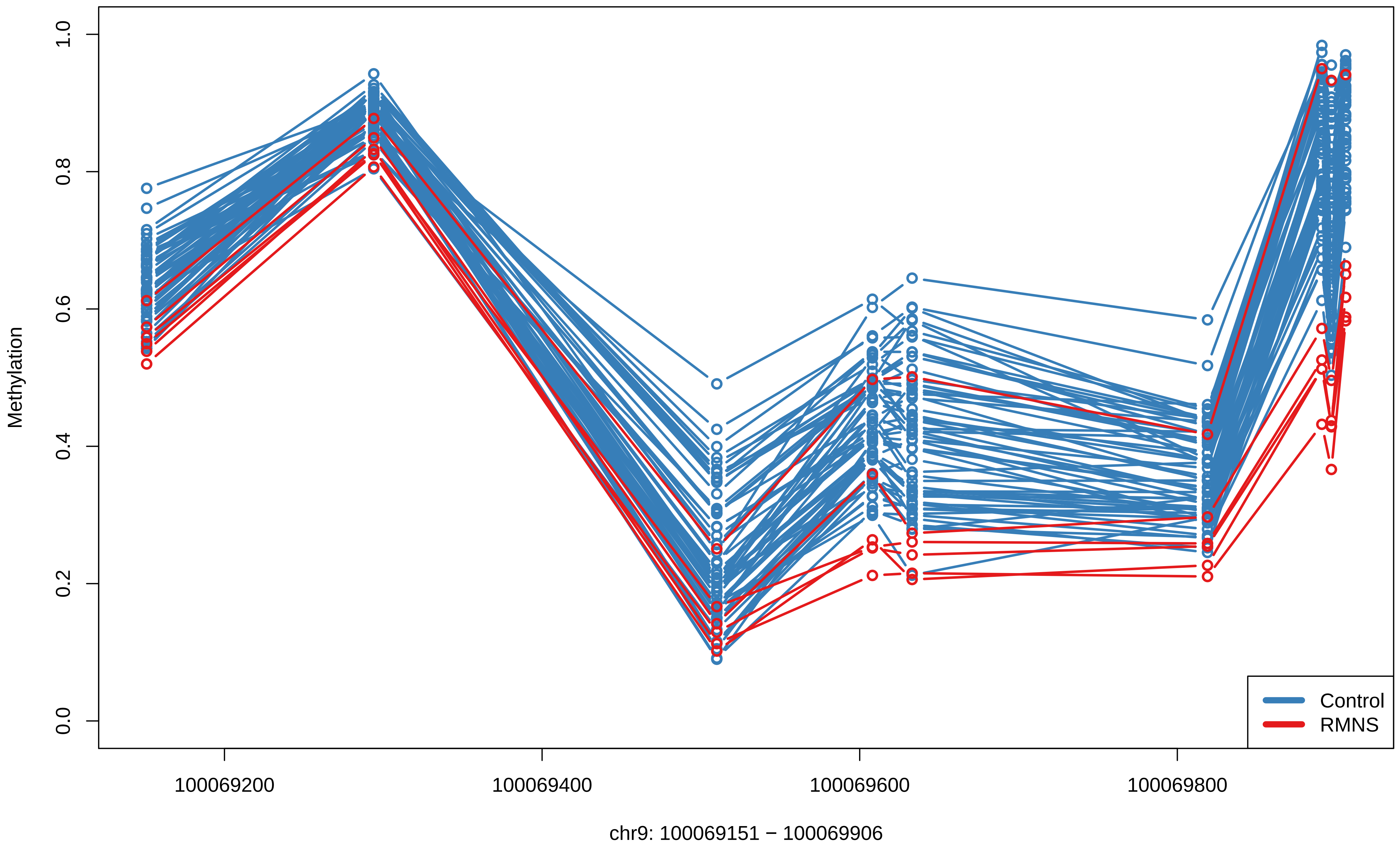

Methylation
0.0
0.2
0.4
0.6
0.8
1.0
100069200
100069400
100069600
100069800
chr9: 100069151 − 100069906
Control
RMNS

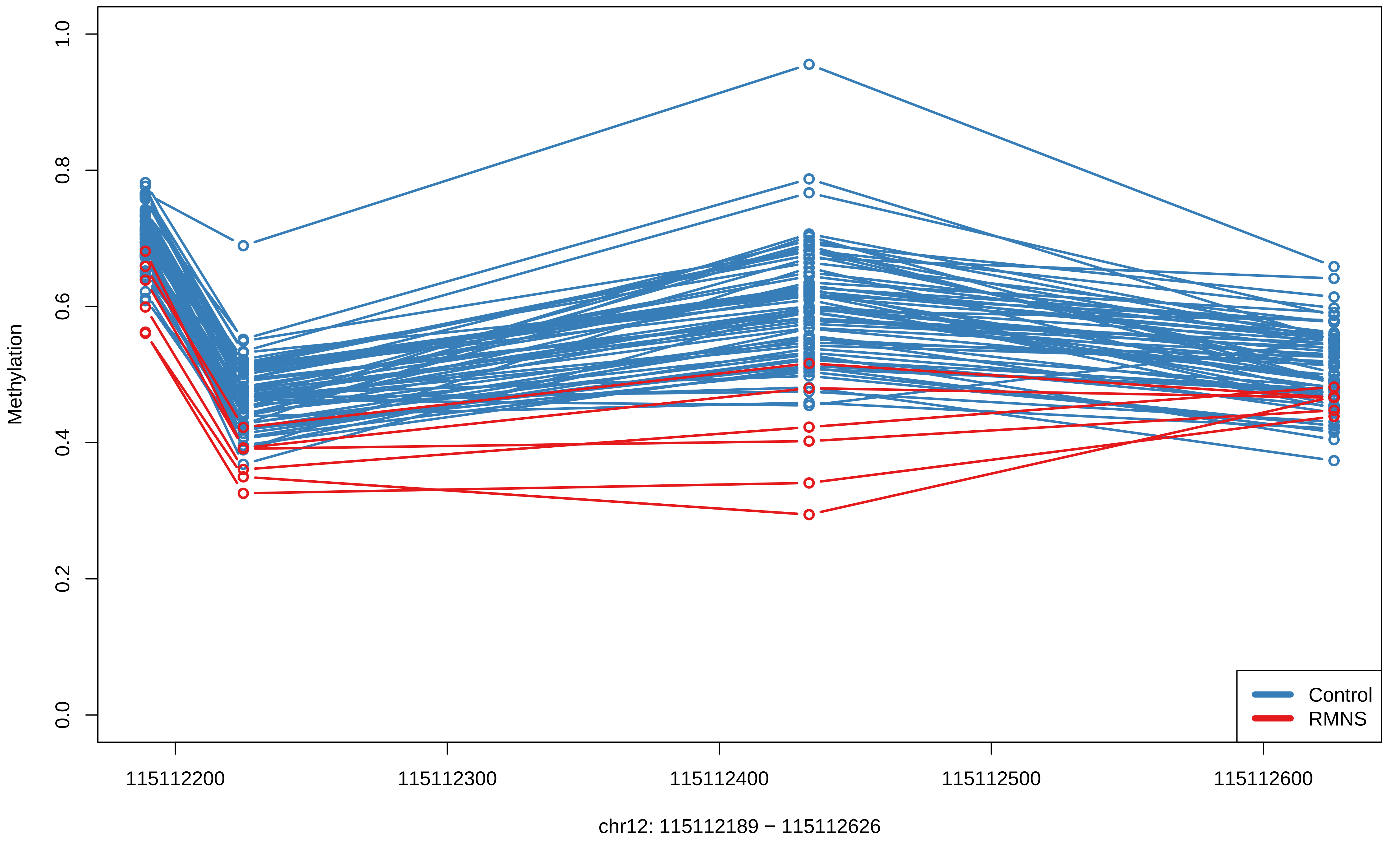

Methylation
0.0
0.2
0.4
0.6
0.8
1.0
115112200
115112300
115112400
115112500
115112600
chr12: 115112189 − 115112626
Control
RMNS

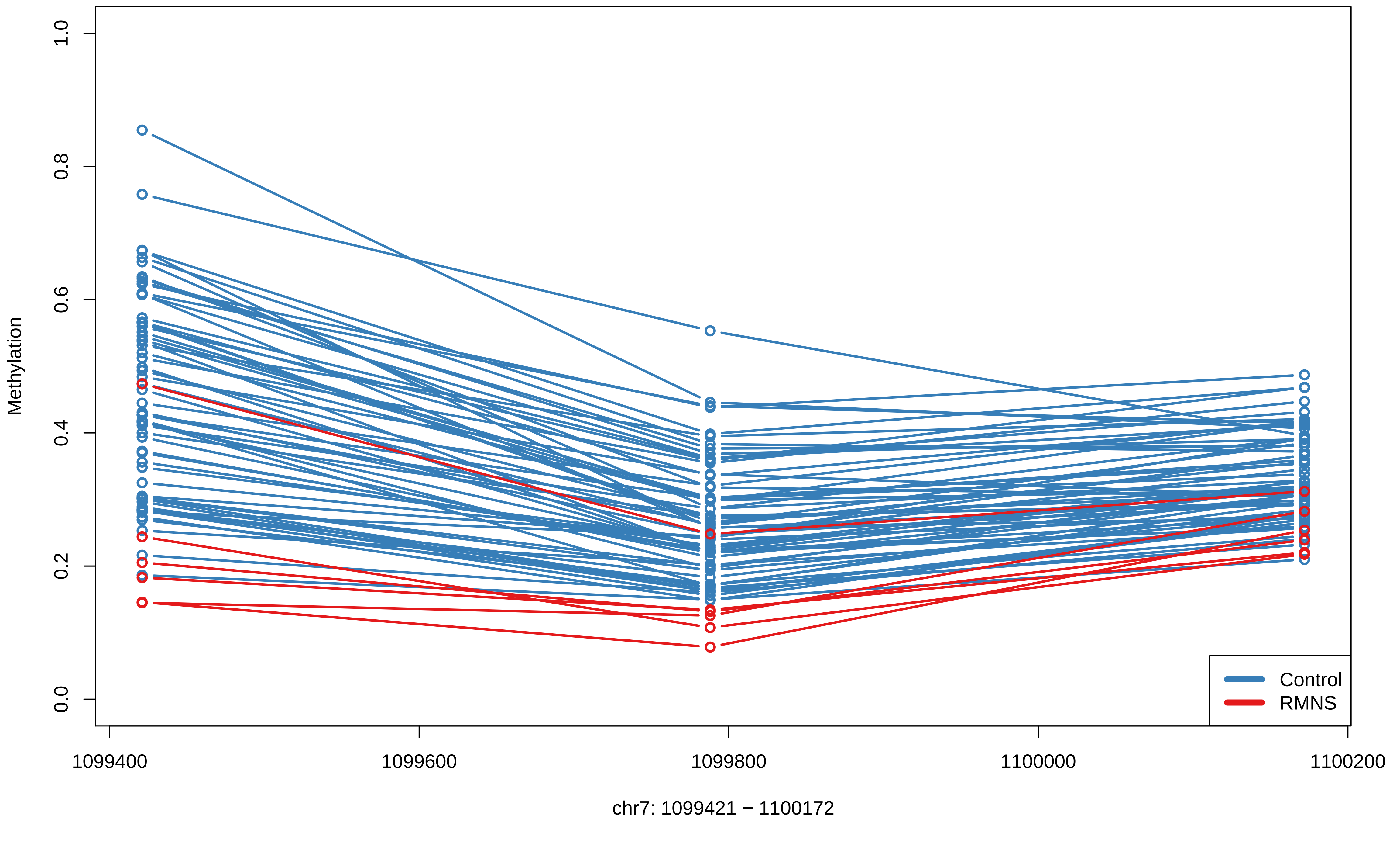
Methylation
0.0
0.2
0.4
0.6
0.8
1.0
1099400
1099600
1099800
1100000
1100200
chr7: 1099421 − 1100172
Control
RMNS

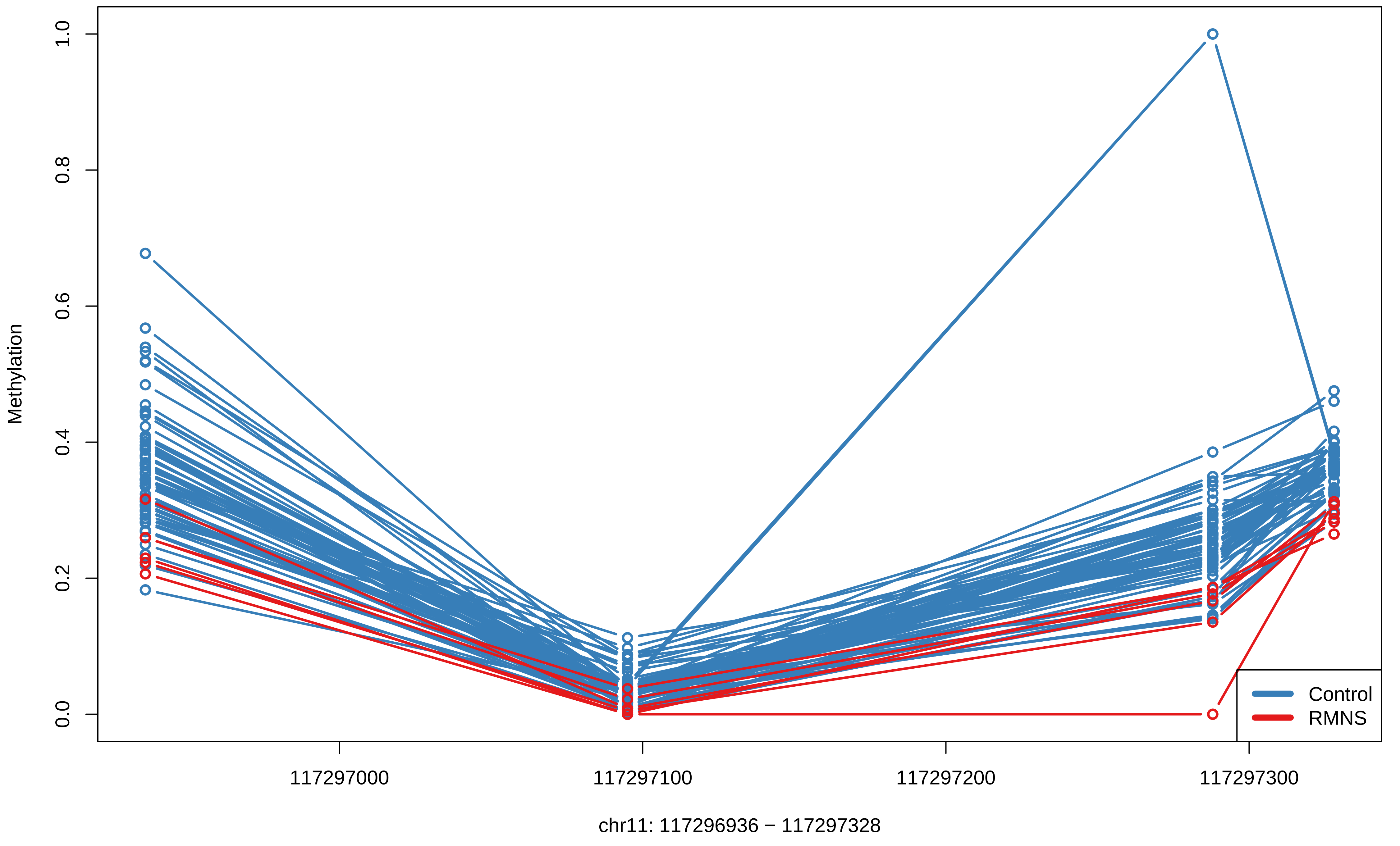

Methylation
0.0
0.2
0.4
0.6
0.8
1.0
117297000
117297100
117297200
117297300
chr11: 117296936 − 117297328
Control
RMNS

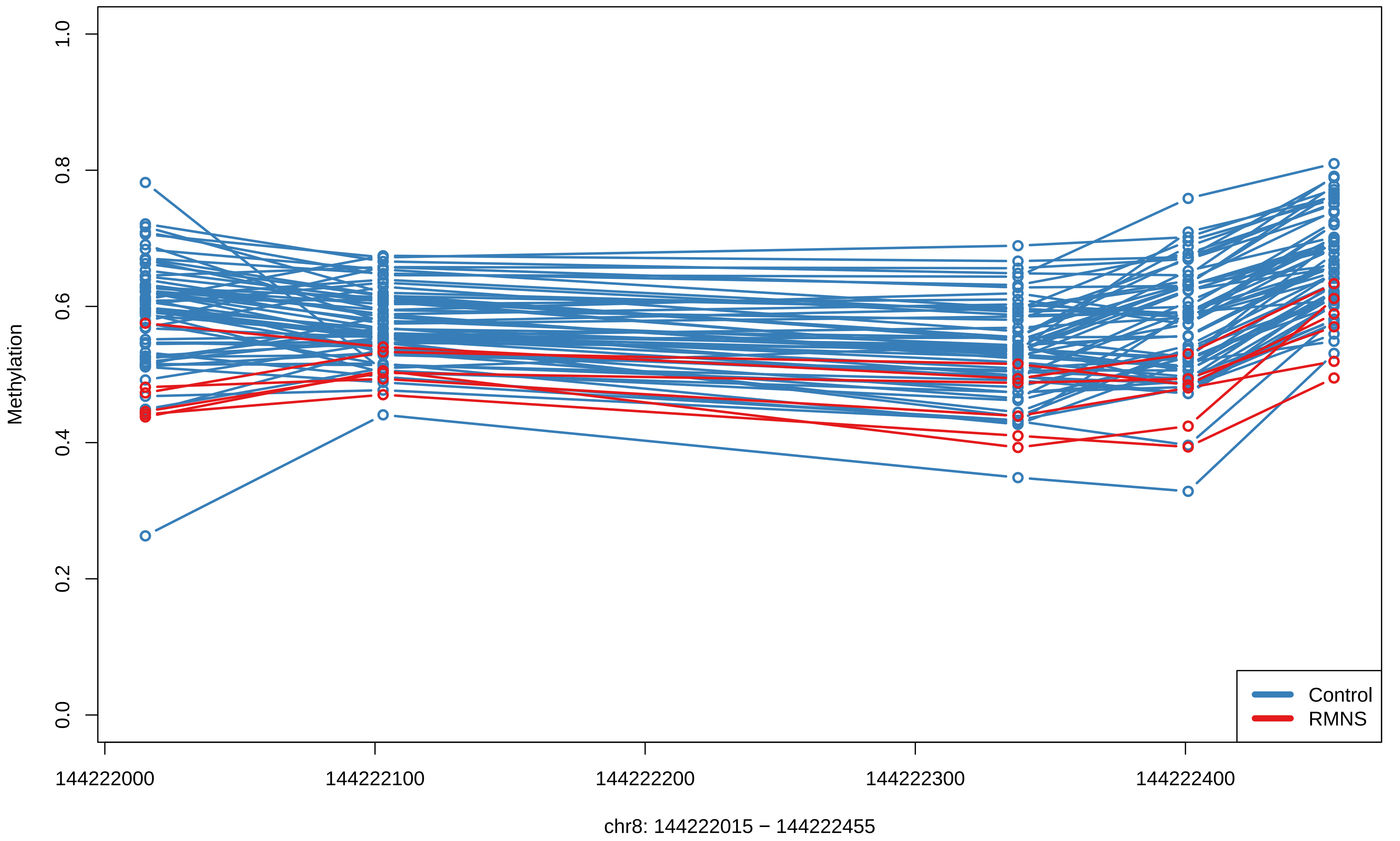

1.0
0.8
0.6
0.4
0.2
0.0
Methylation
144222000
144222100
144222200
144222300
144222400
chr8: 144222015 − 144222455
Control
RMNS

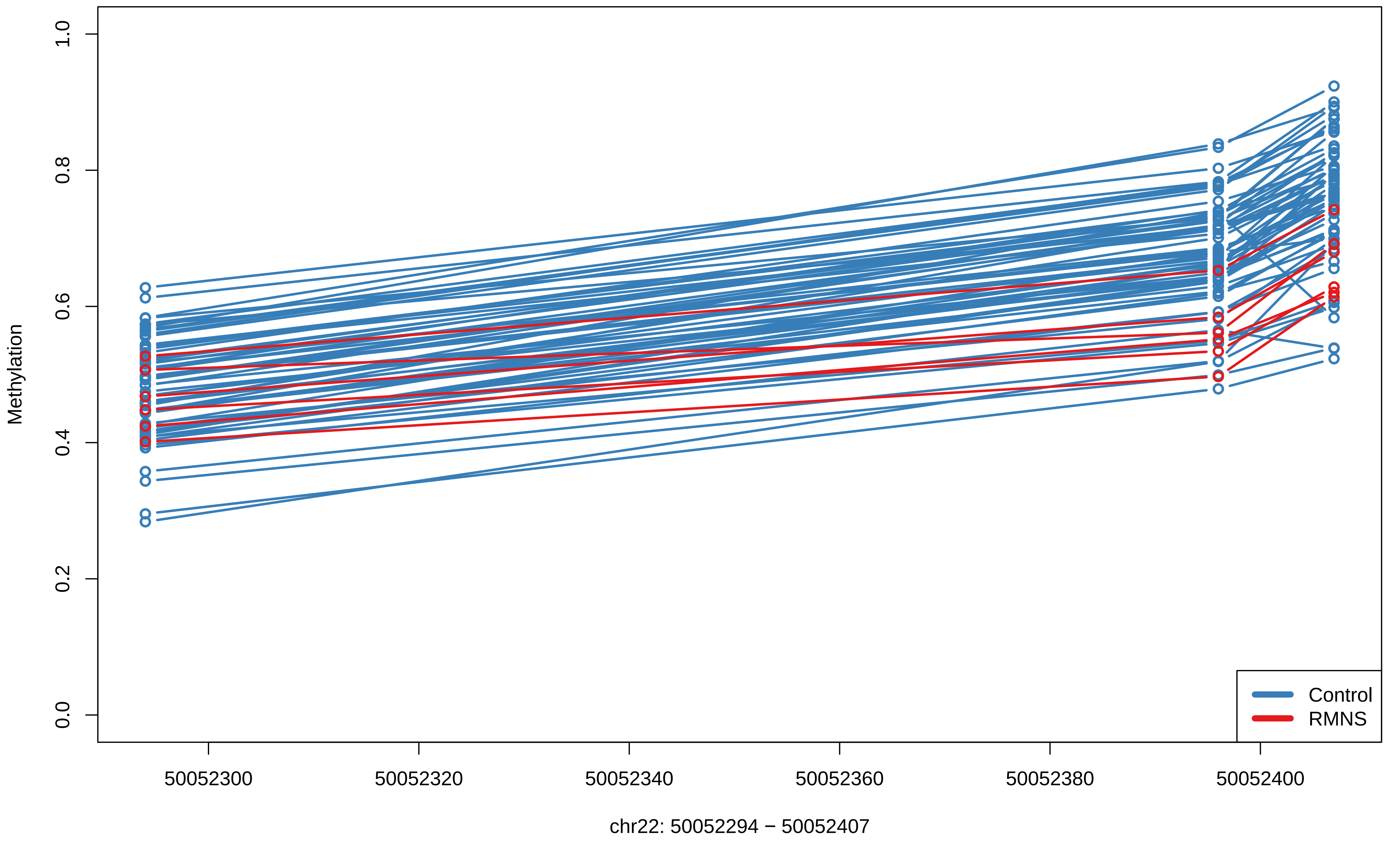

Methylation
0.0
0.2
0.4
0.6
0.8
1.0
50052300
50052320
50052340
50052360
50052380
50052400
chr22: 50052294 − 50052407
Control
RMNS

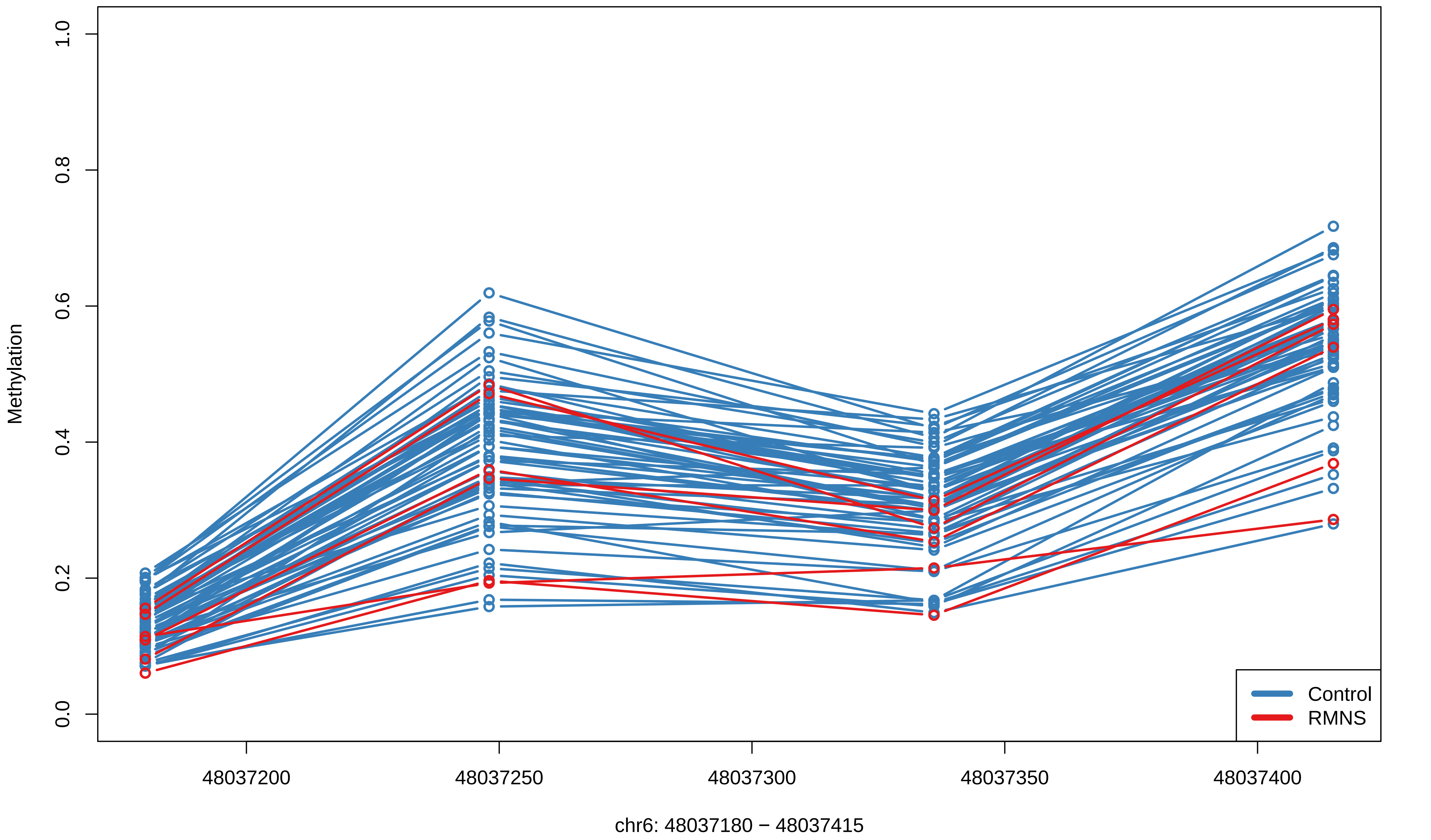

Methylation
1.0
0.8
0.6
0.4
0.2
0.0
48037200
48037250
48037300
48037350
48037400
chr6: 48037180 − 48037415
Control
RMNS

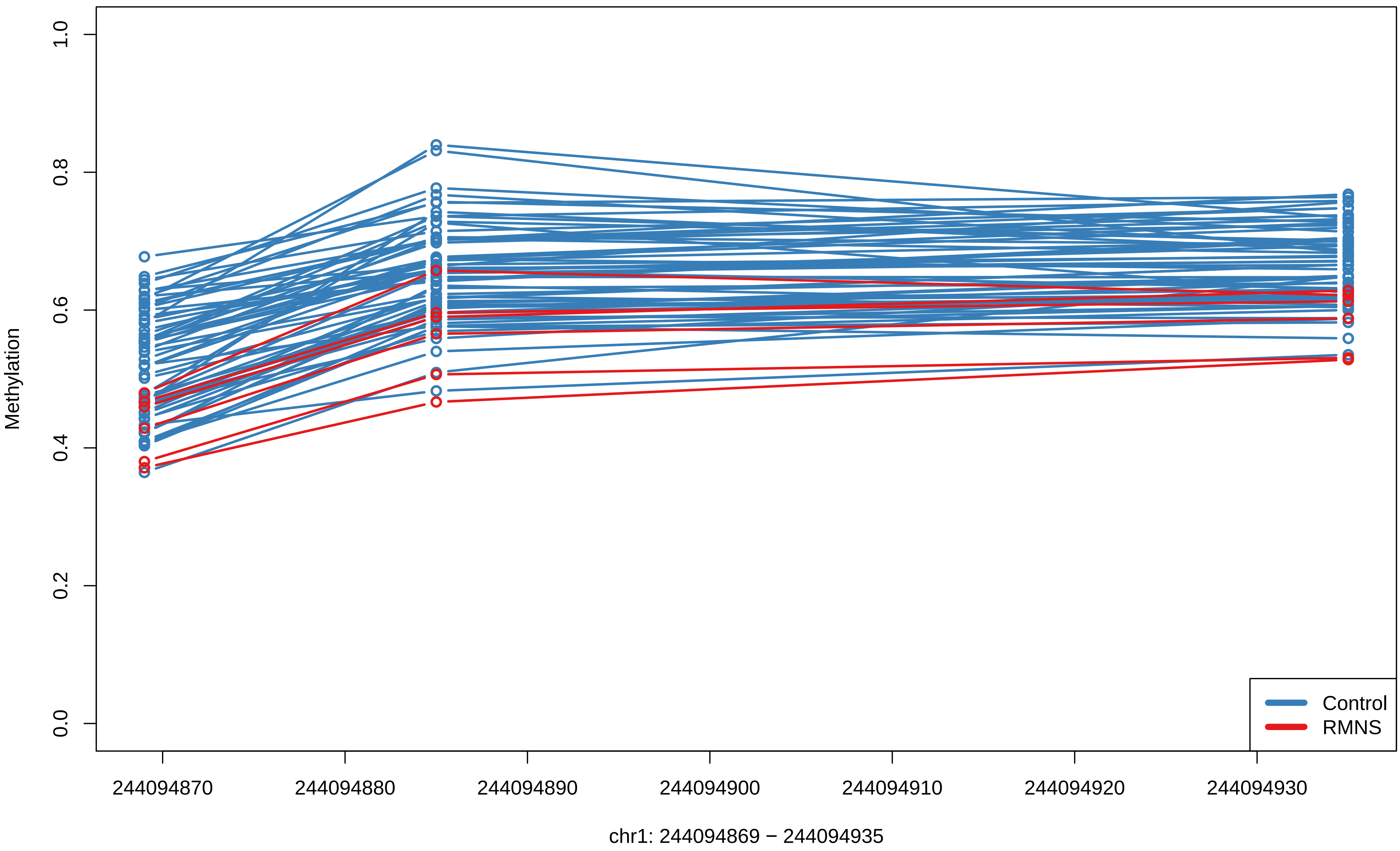

Methylation
0.0
0.2
0.4
0.6
0.8
1.0
244094870
244094880
244094890
244094900
244094910
244094920
244094930
chr1: 244094869 − 244094935
Control
RMNS

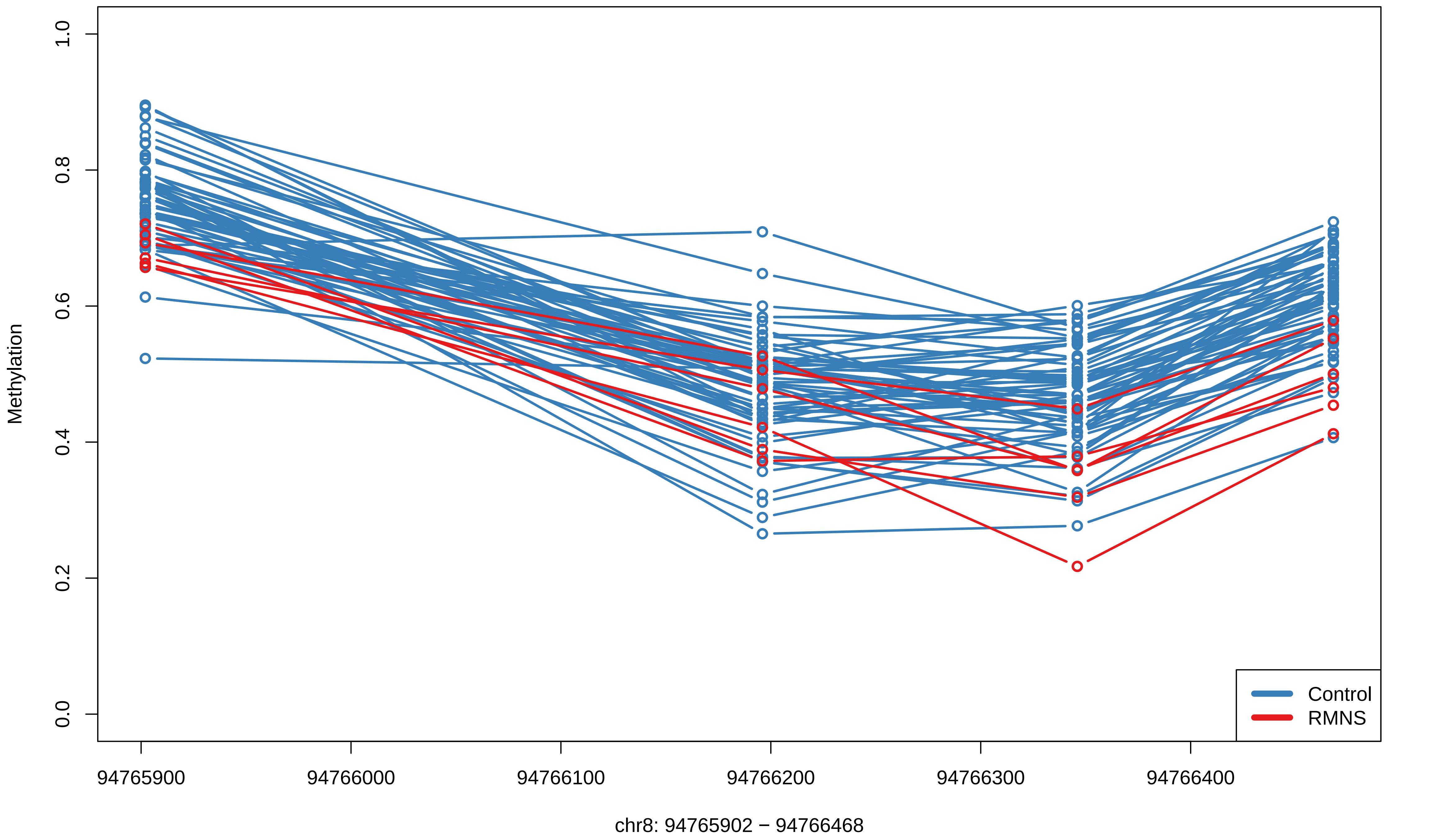

Methylation
0.0
0.2
0.4
0.6
0.8
1.0
94765900
94766000
94766100
94766200
94766300
94766400
chr8: 94765902 − 94766468
Control
RMNS

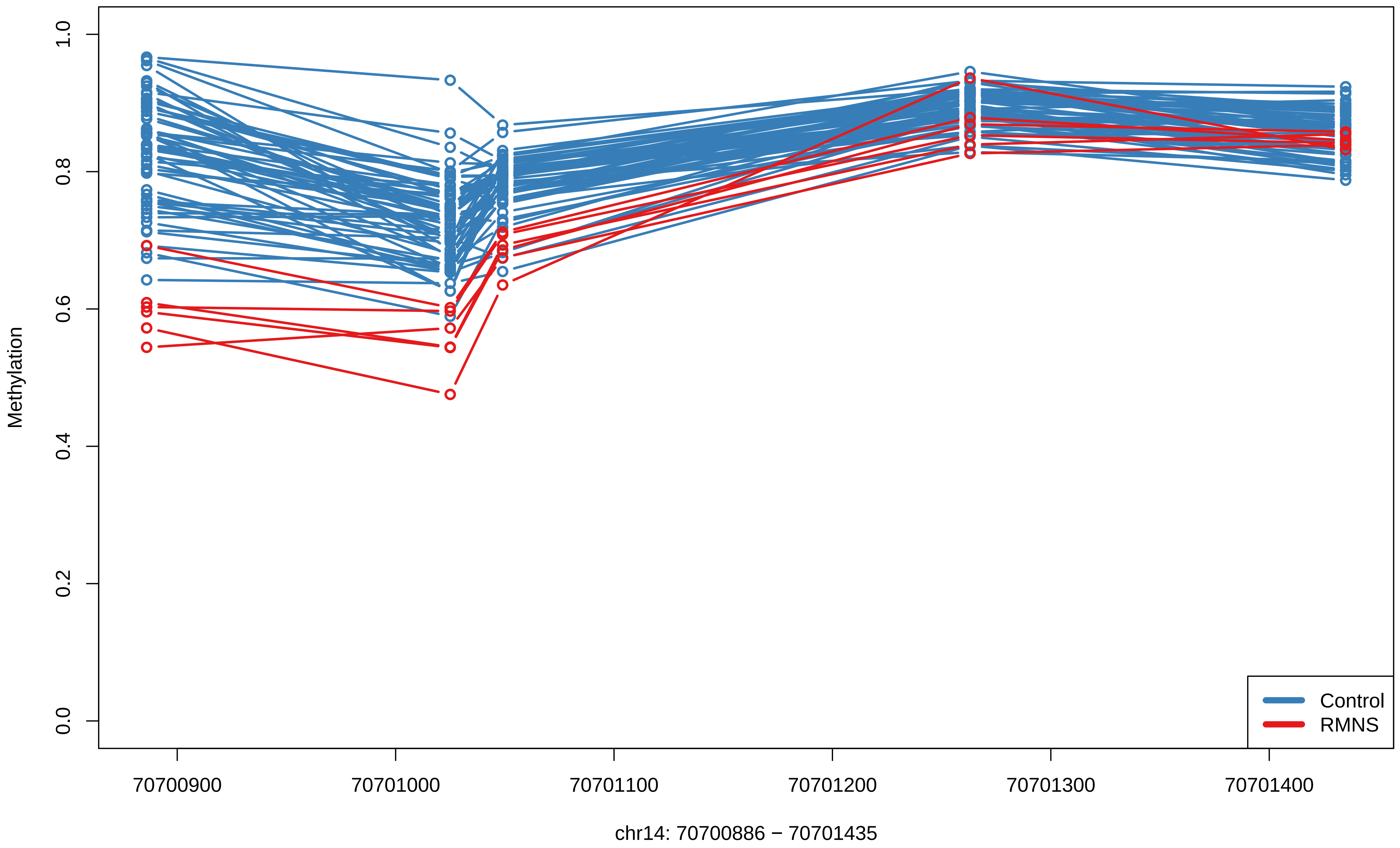

1.0
0.8
0.6
0.4
0.2
0.0
Methylation
70700900
70701000
70701100
70701200
70701300
70701400
chr14: 70700886 − 70701435
Control
RMNS

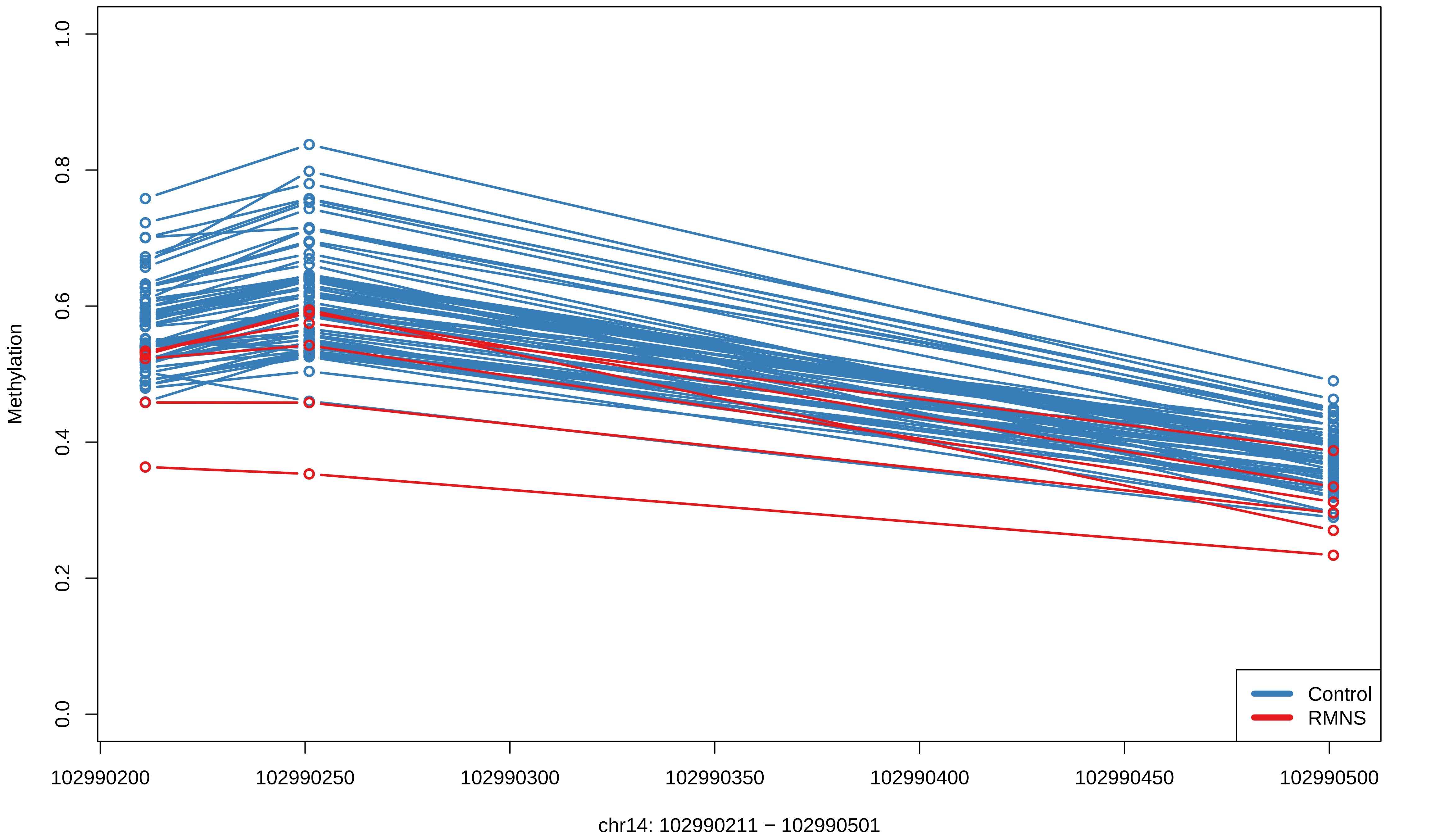

Methylation
1.0
0.8
0.6
0.4
0.2
0.0
102990200
102990250
102990300
102990350
102990400
102990450
102990500
chr14: 102990211 − 102990501
Control
RMNS

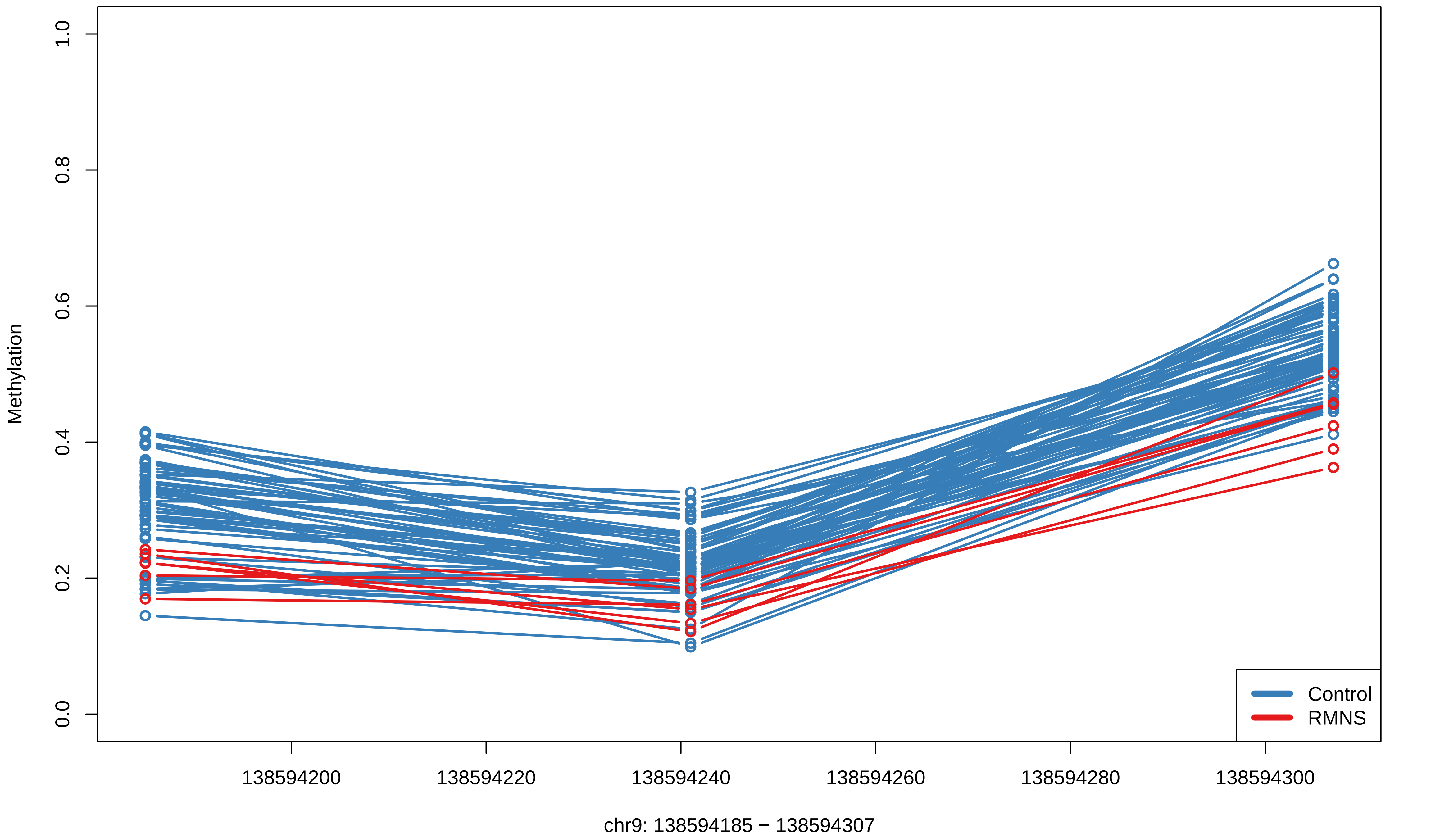

Methylation
0.0
0.2
0.4
0.6
0.8
1.0
138594200
138594220
138594240
138594260
138594280
138594300
chr9: 138594185 − 138594307
Control
RMNS

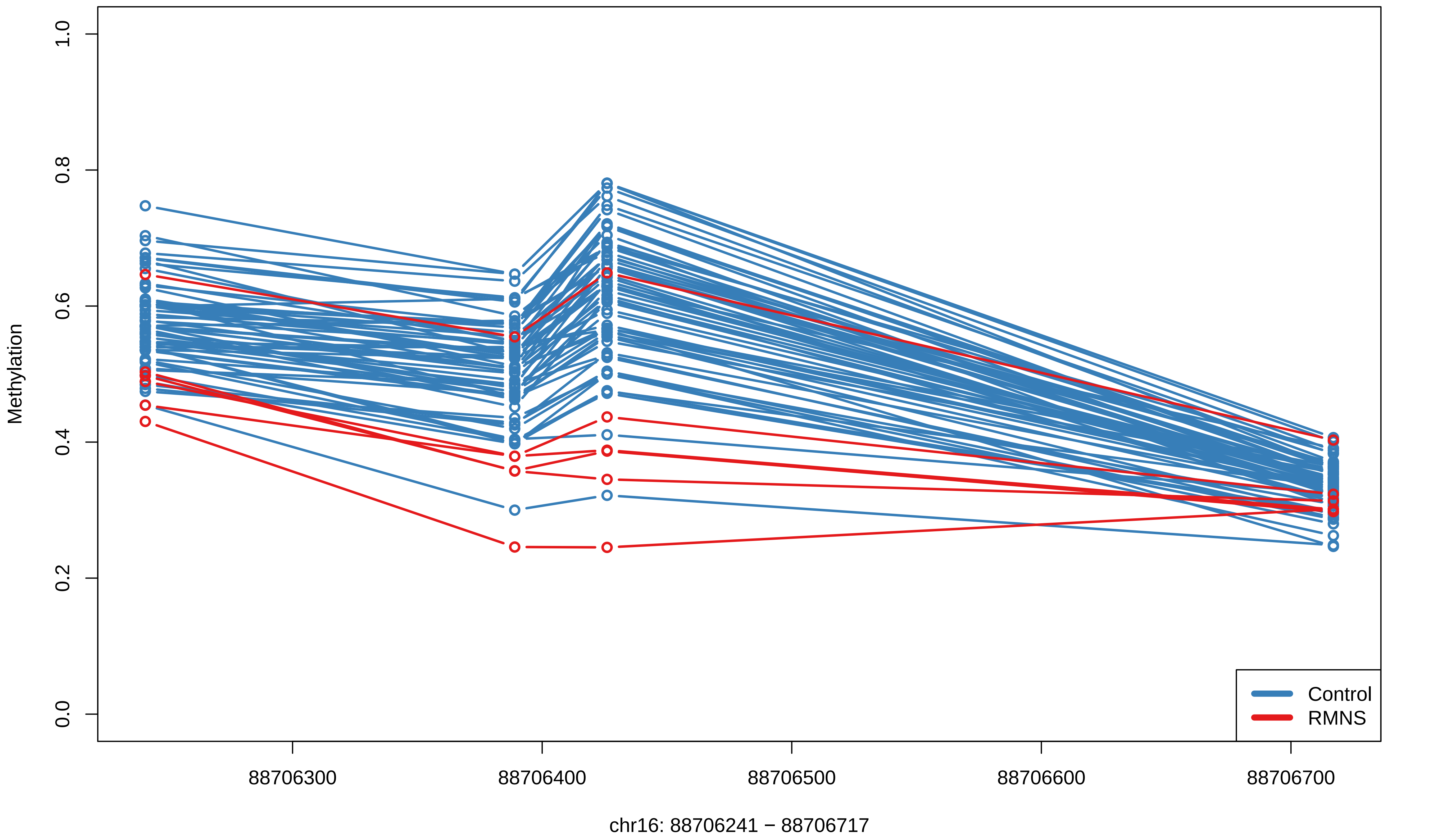

Methylation
0.0
0.2
0.4
0.6
0.8
1.0
88706300
88706400
88706500
88706600
88706700
chr16: 88706241 − 88706717
Control
RMNS

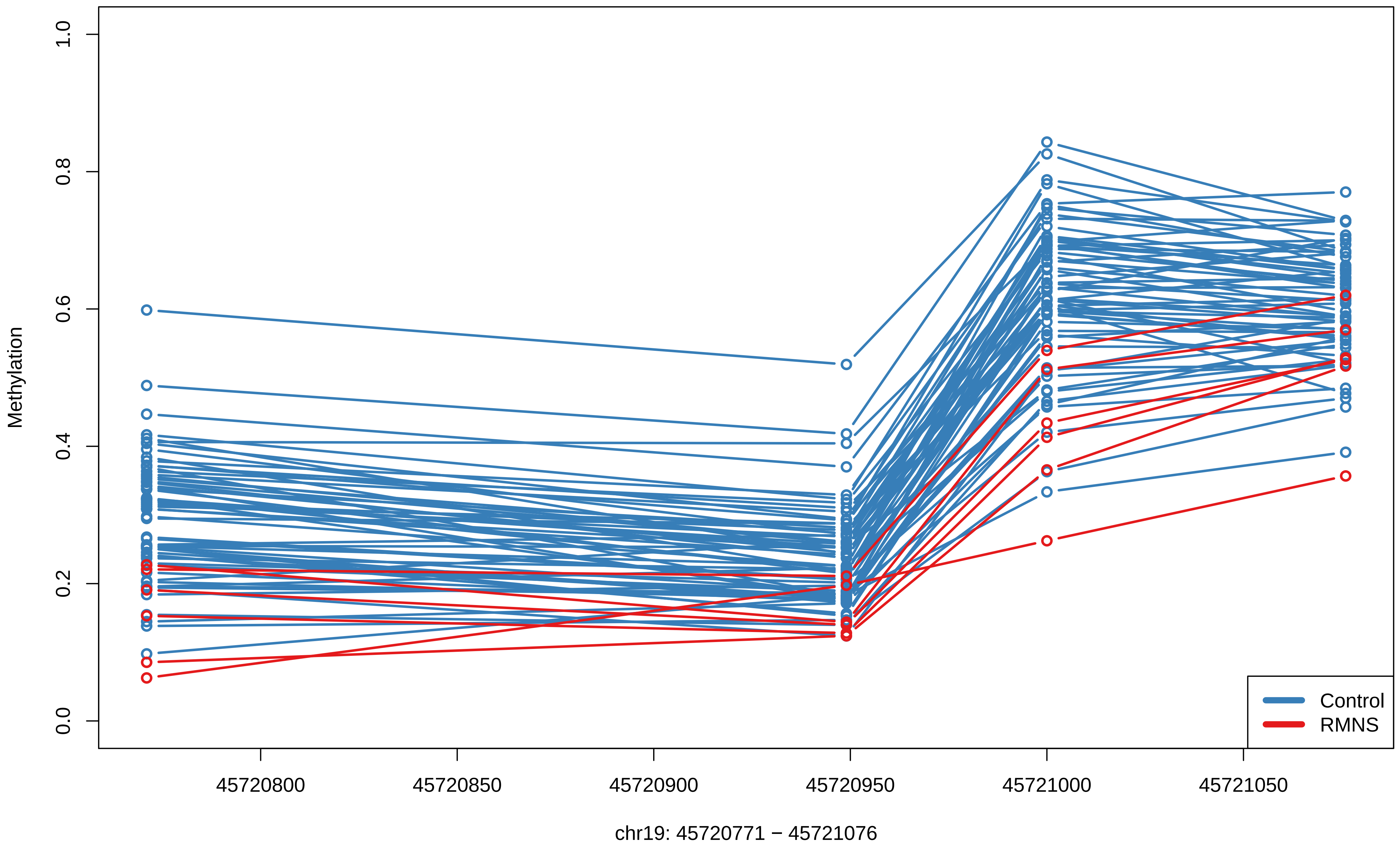

Methylation
0.0
0.2
0.4
0.6
0.8
1.0
45720800
45720850
45720900
45720950
45721000
45721050
chr19: 45720771 − 45721076
Control
RMNS

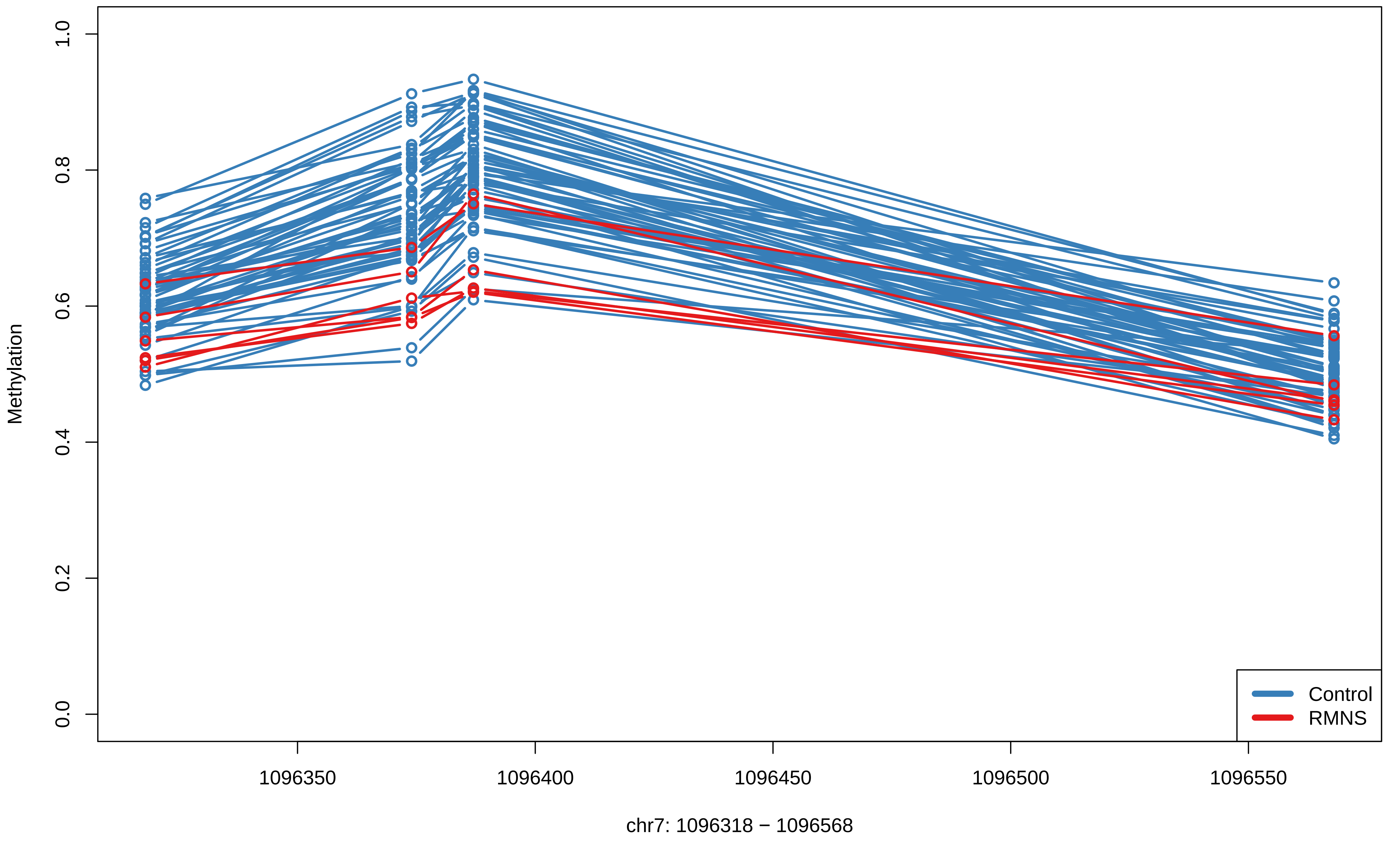

Methylation
0.0
0.2
0.4
0.6
0.8
1.0
1096350
1096400
1096450
1096500
1096550
chr7: 1096318 − 1096568
Control
RMNS

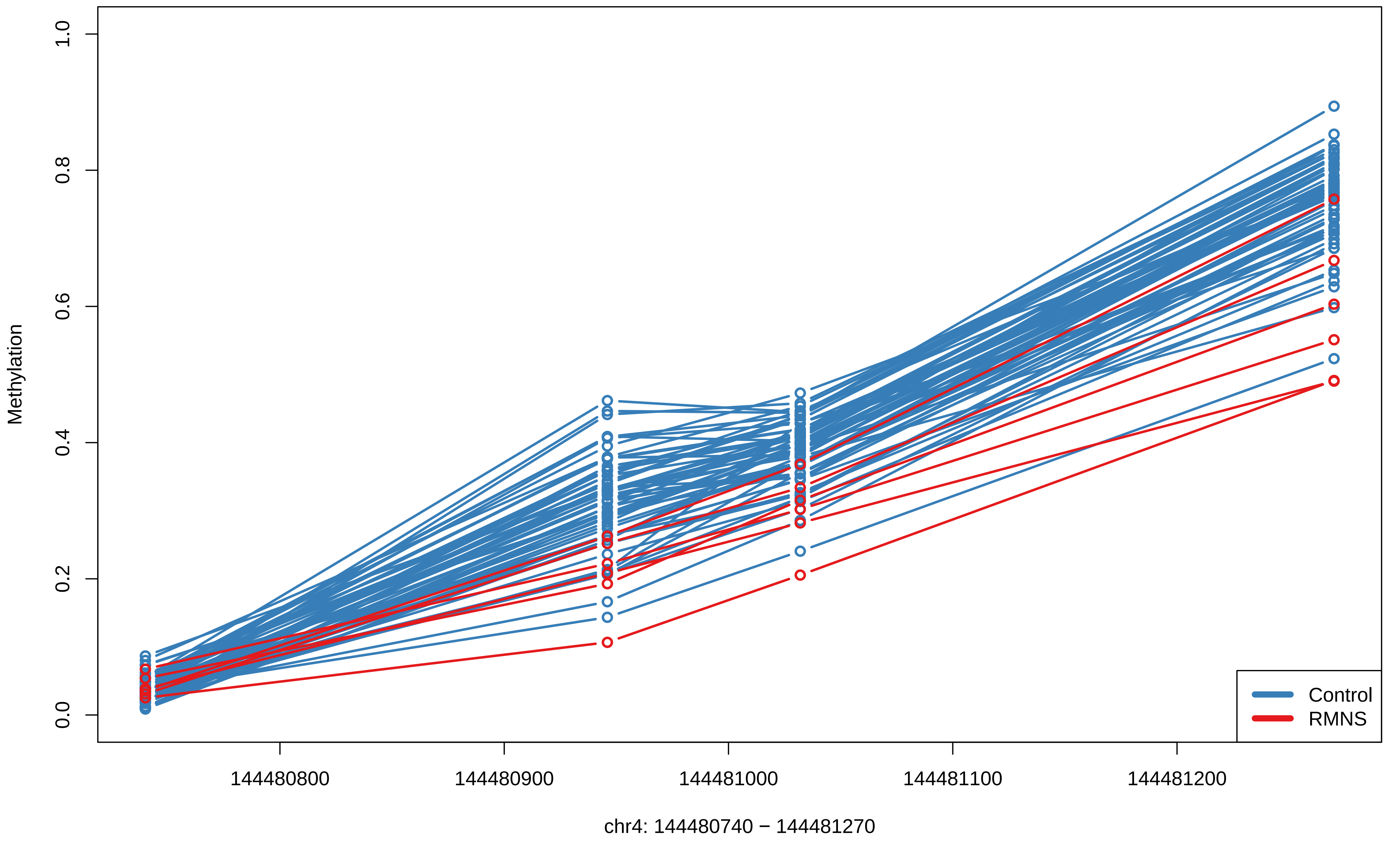

Methylation
0.0
0.2
0.4
0.6
0.8
1.0
144480800
144480900
144481000
144481100
144481200
chr4: 144480740 − 144481270
Control
RMNS

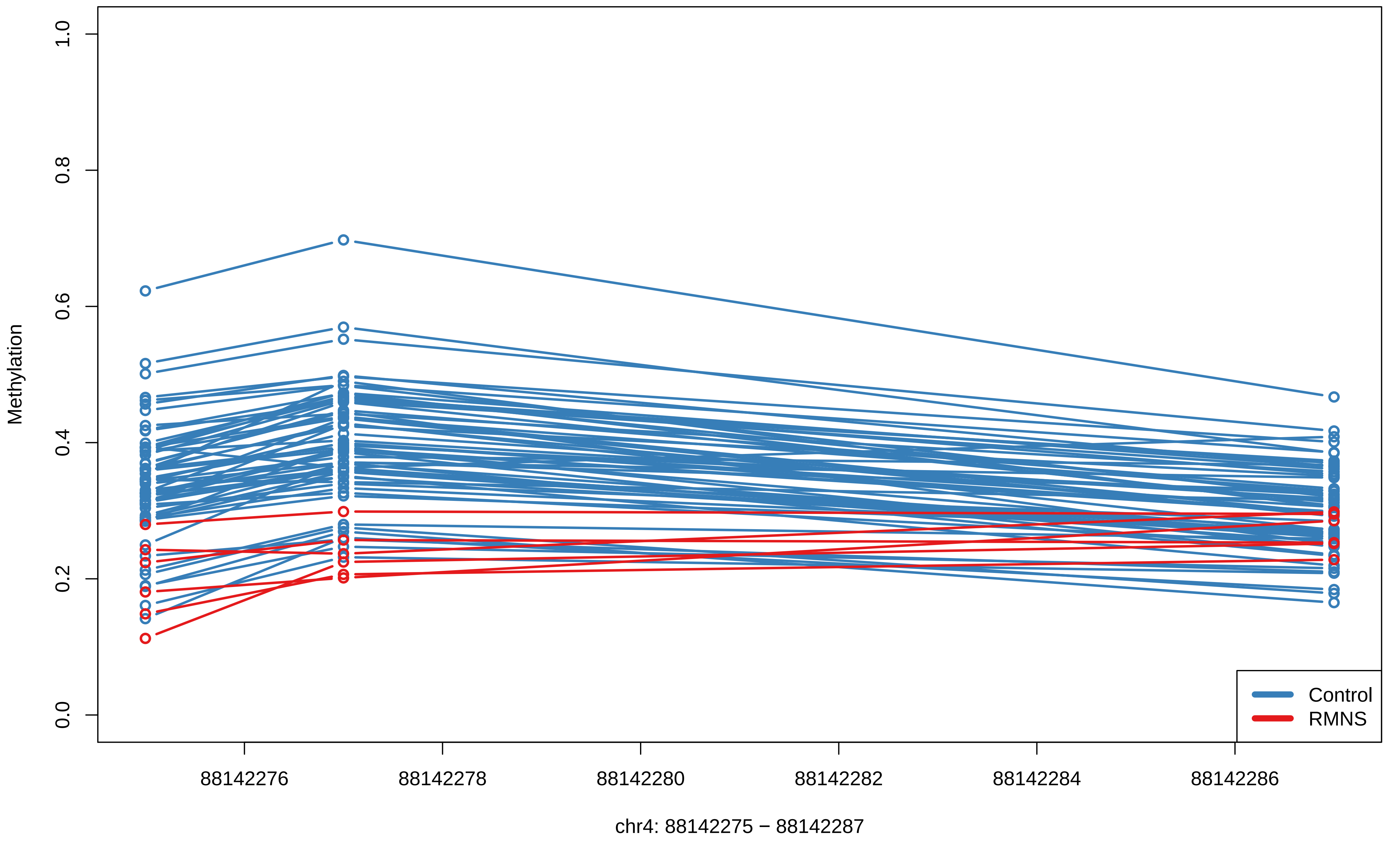

Methylation
1.0
0.8
0.6
0.4
0.2
0.0
88142276
88142278
88142280
88142282
88142284
88142286
chr4: 88142275 − 88142287
Control
RMNS

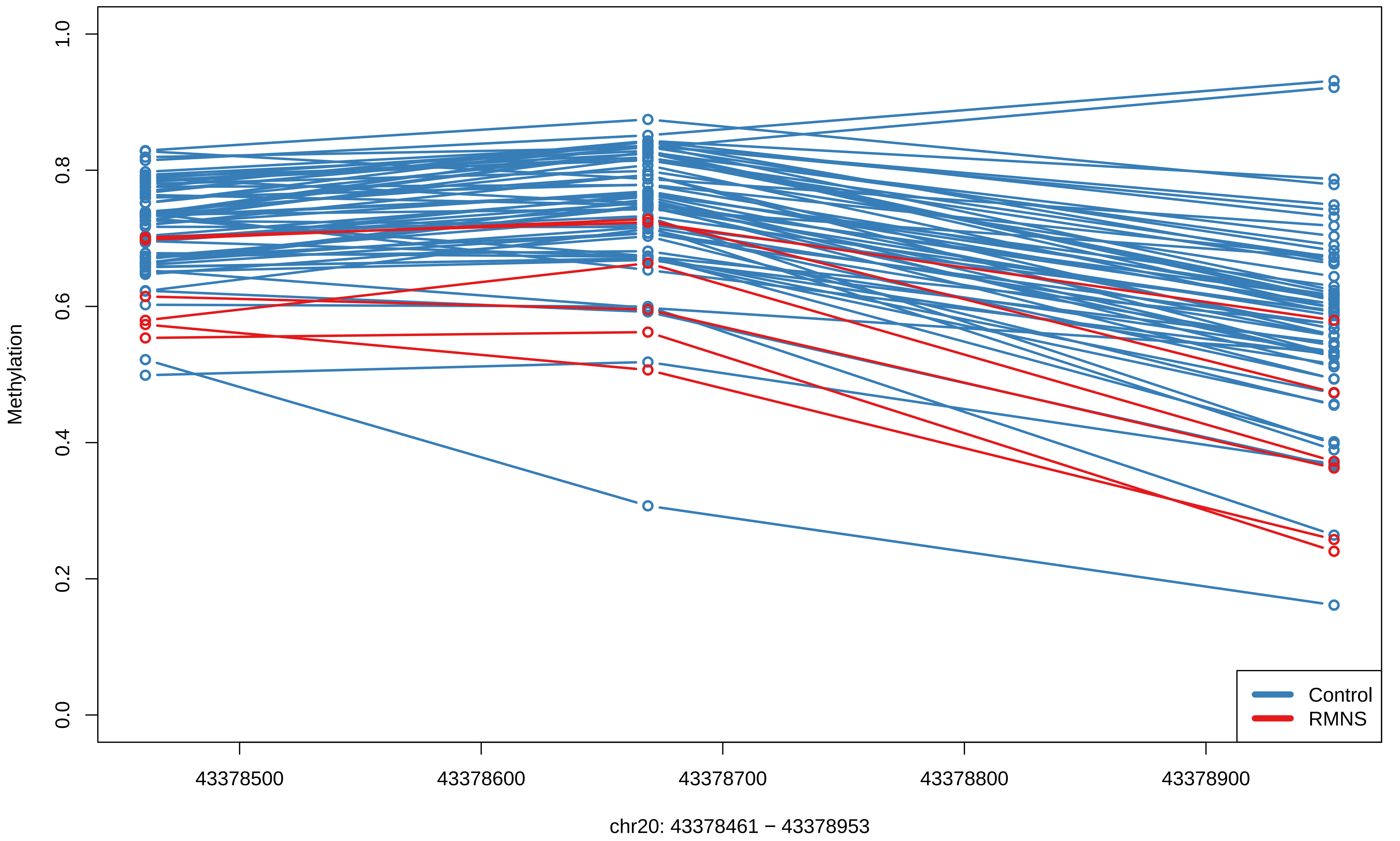

Methylation
0.0
0.2
0.4
0.6
0.8
1.0
43378500
43378600
43378700
43378800
43378900
chr20: 43378461 − 43378953
Control
RMNS

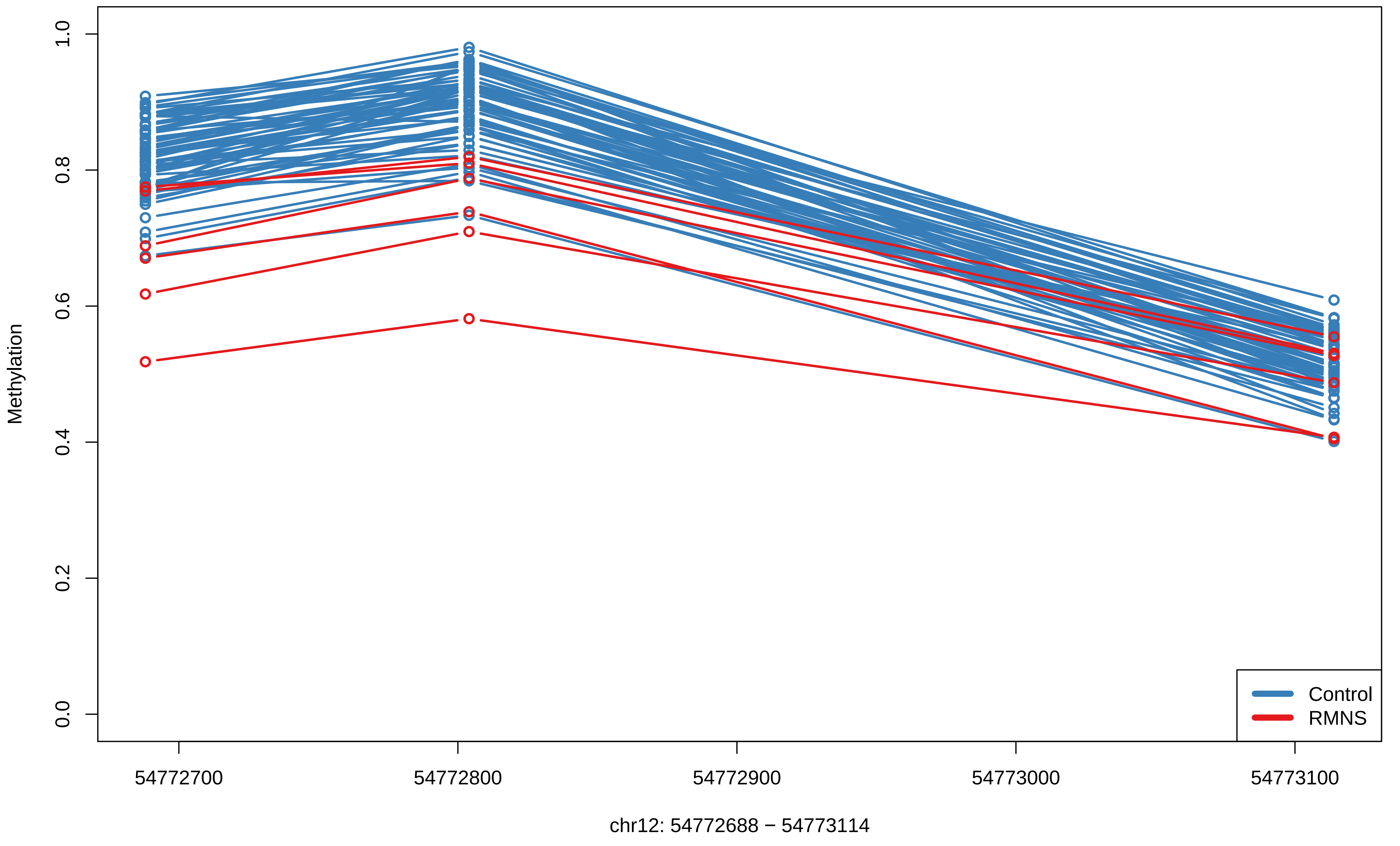

Methylation
1.0
0.8
0.6
0.4
0.2
0.0
54772700
54772800
54772900
54773000
54773100
chr12: 54772688 − 54773114
Control
RMNS

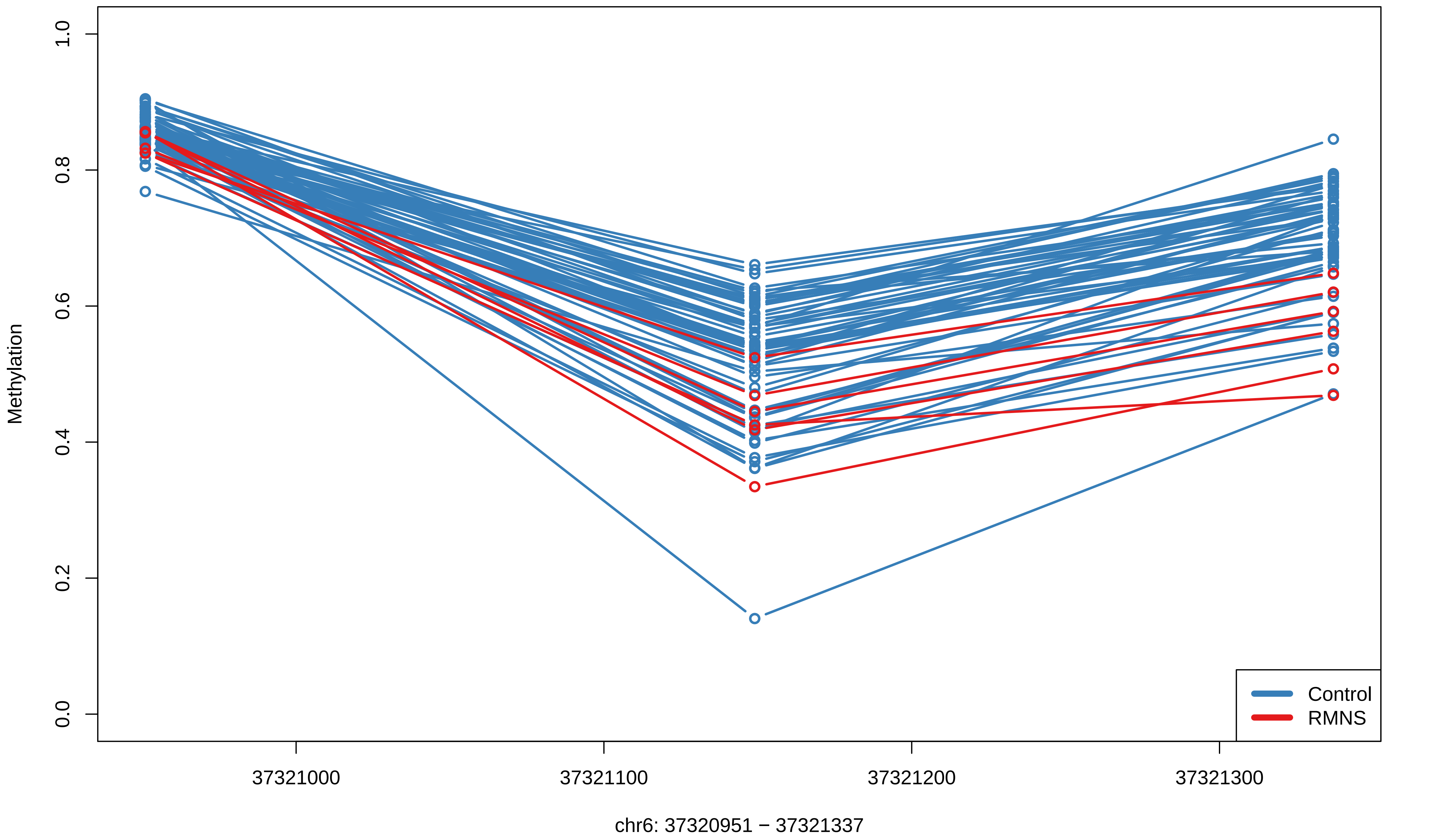

1.0
0.8
0.6
0.4
0.2
0.0
Methylation
37321000
37321100
37321200
37321300
chr6: 37320951 − 37321337
Control
RMNS

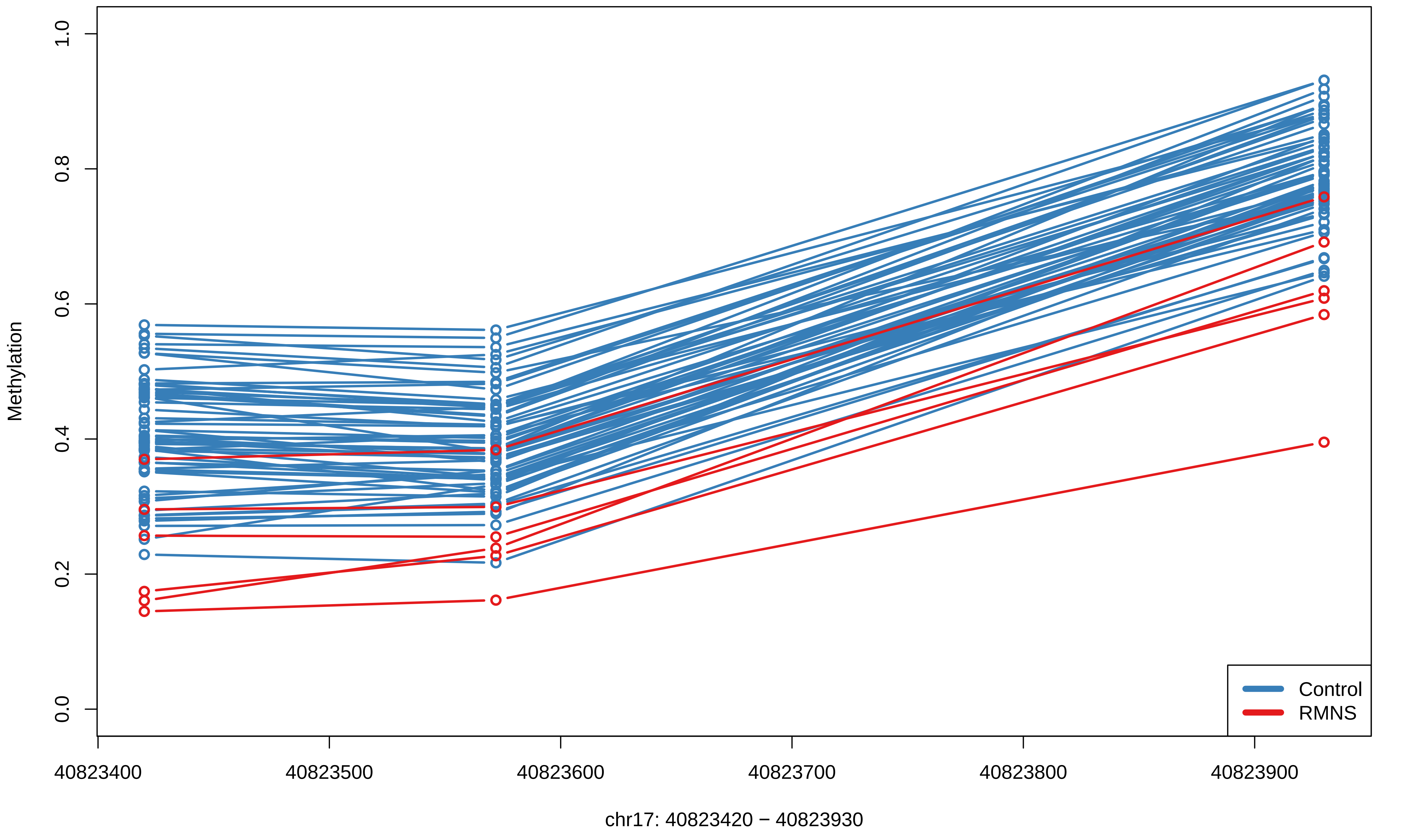

1.0
0.8
0.6
0.4
0.2
0.0
Methylation
40823400
40823500
40823600
40823700
40823800
40823900
chr17: 40823420 − 40823930
Control
RMNS

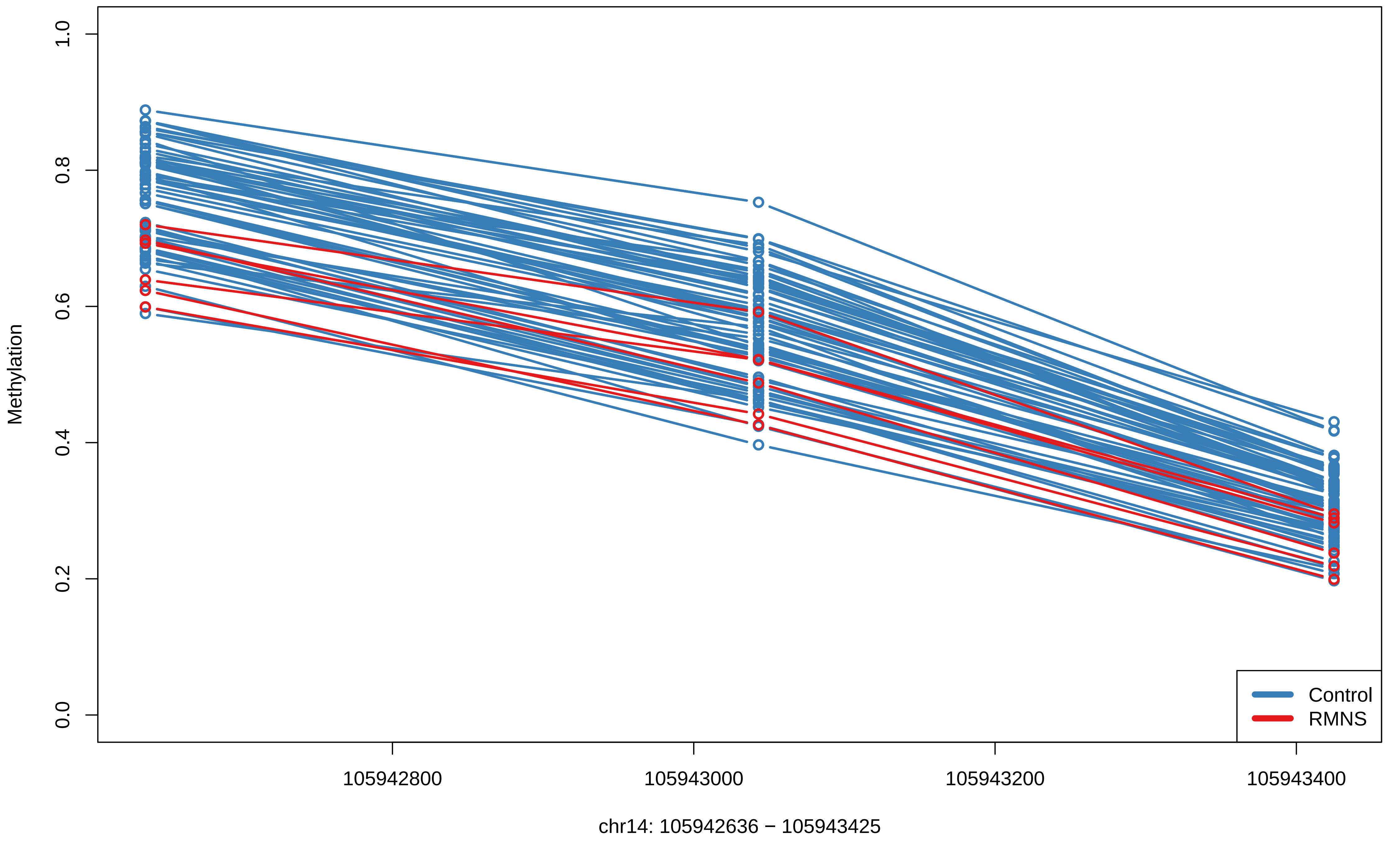
Methylation
0.0
0.2
0.4
0.6
0.8
1.0
105942800
105943000
105943200
105943400
chr14: 105942636 − 105943425
Control
RMNS

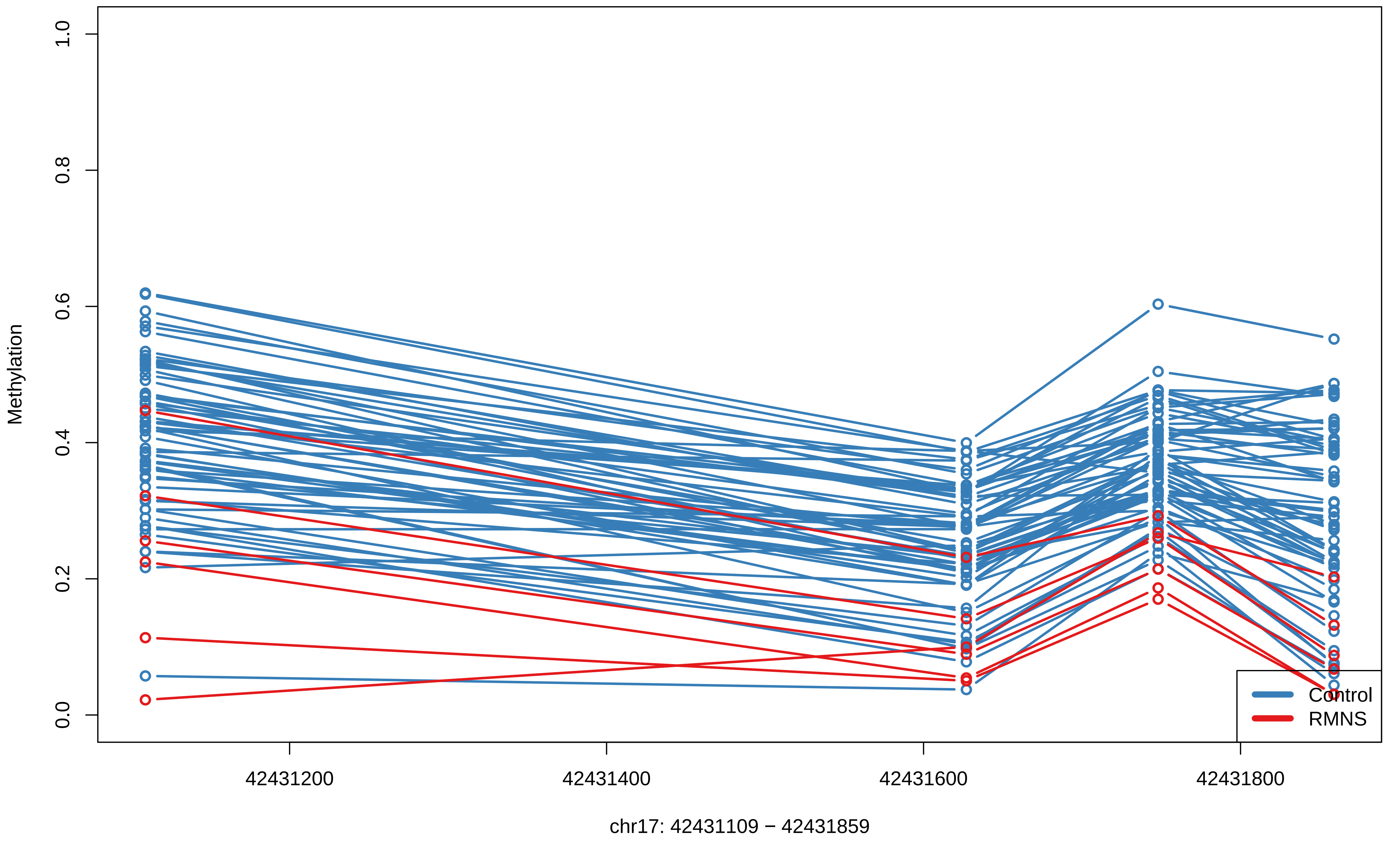

1.0
0.8
0.6
0.4
0.2
0.0
Methylation
42431200
42431400
42431600
42431800
chr17: 42431109 − 42431859
Control
RMNS

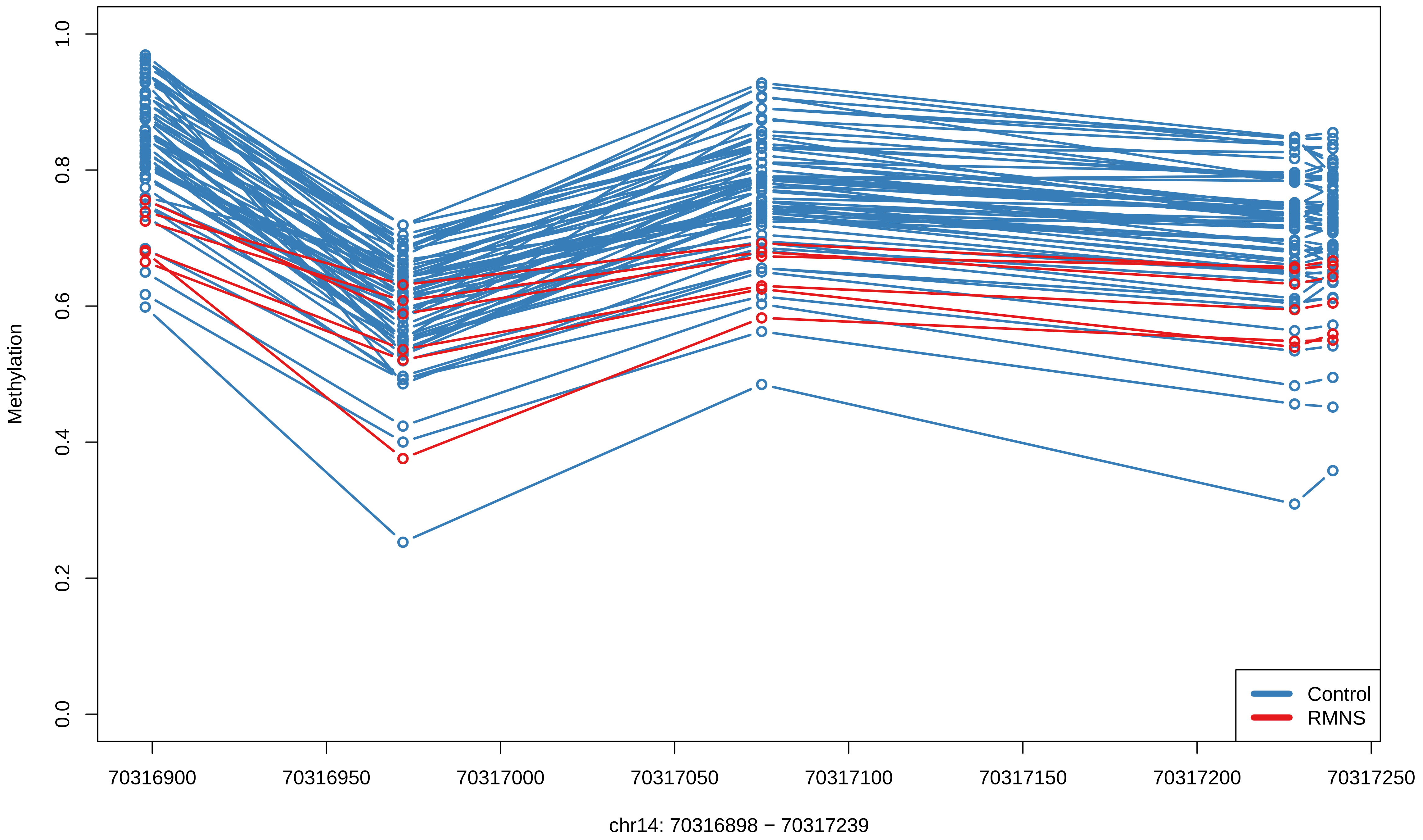
1.0
0.8
0.6
0.4
0.2
0.0
Methylation
70316900
70316950
70317000
70317050
70317100
70317150
70317200
70317250
chr14: 70316898 − 70317239
Control
RMNS

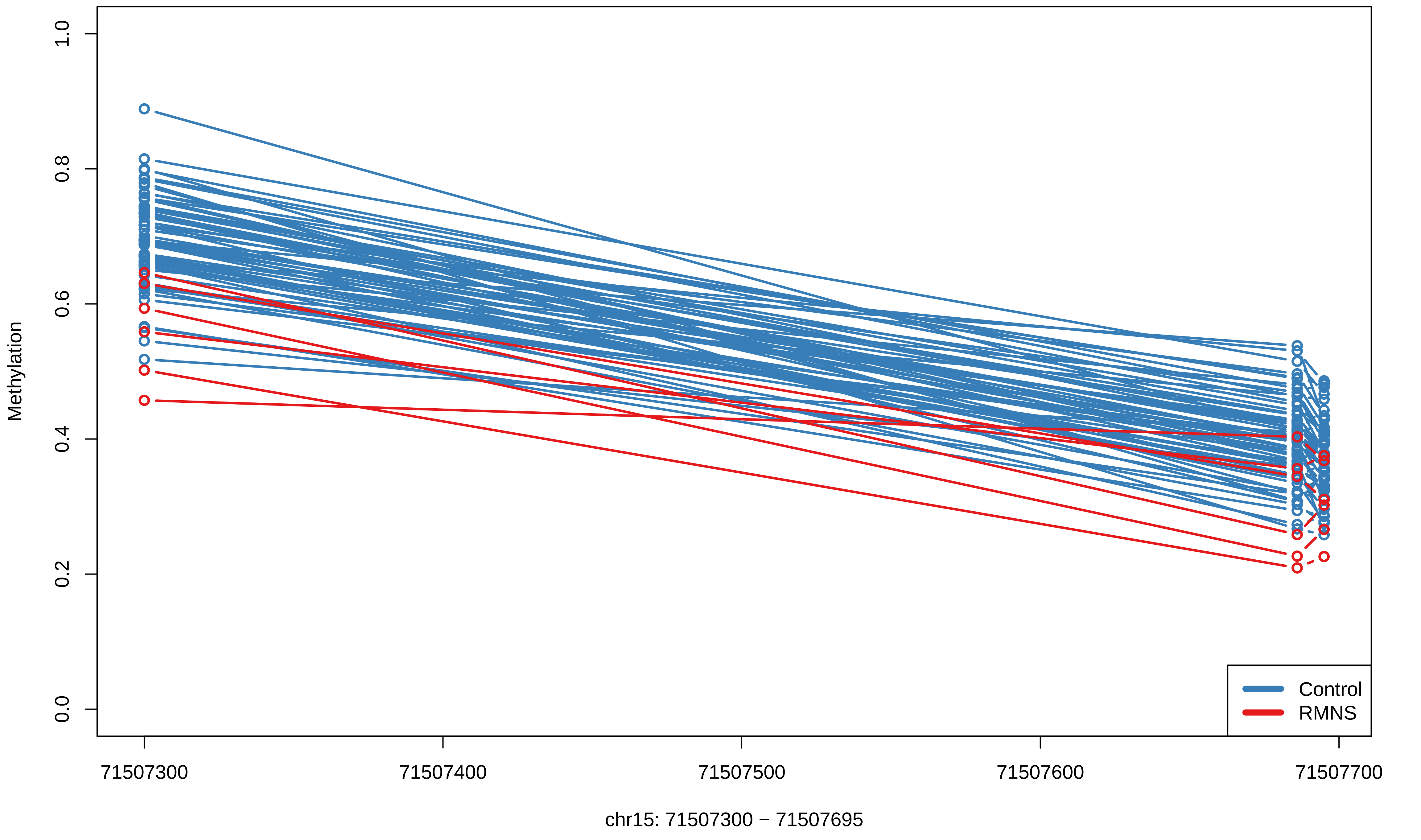

Methylation
0.0
0.2
0.4
0.6
0.8
1.0
71507300
71507400
71507500
71507600
71507700
chr15: 71507300 − 71507695
Control
RMNS

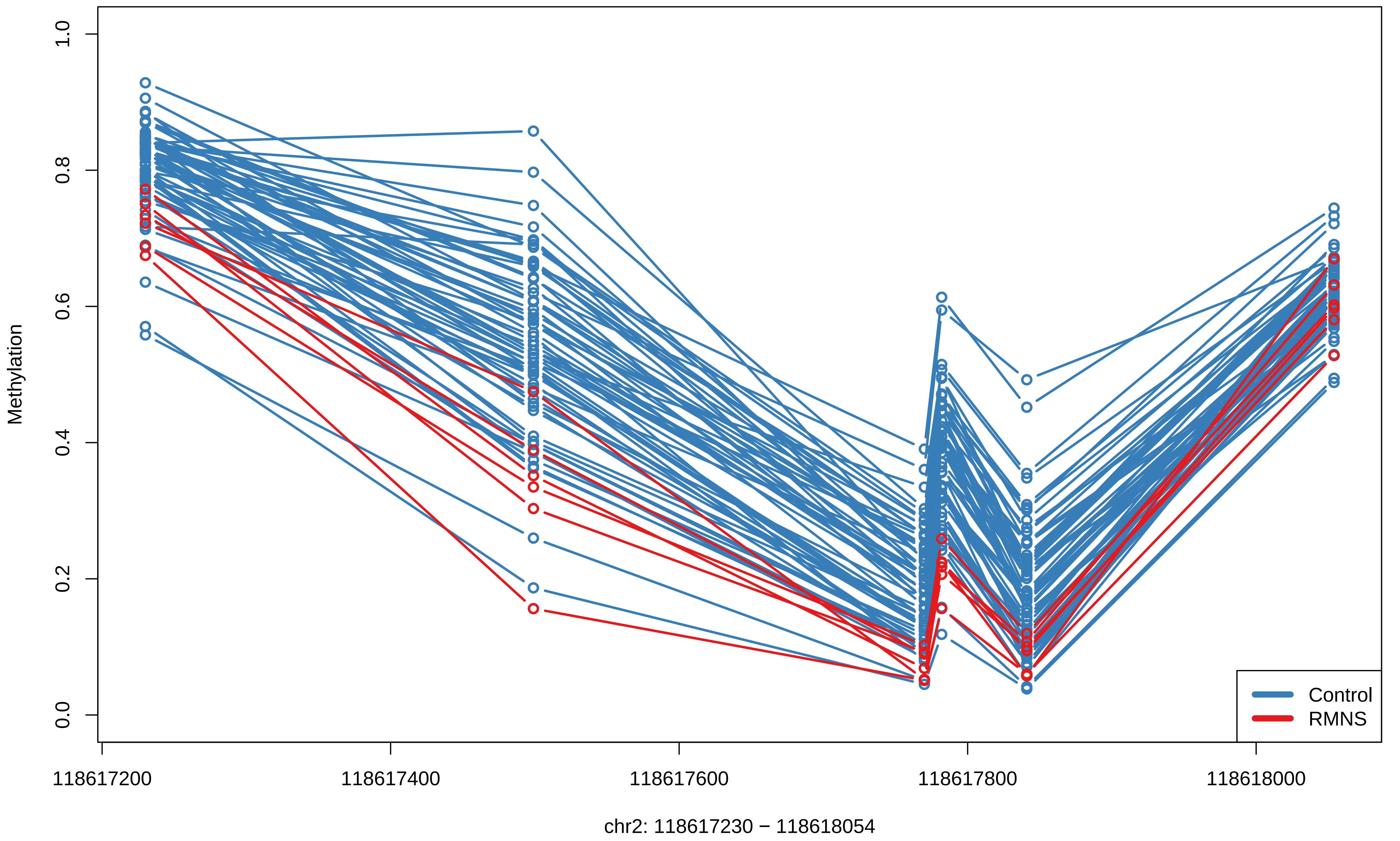

Methylation
1.0
0.8
0.6
0.4
0.2
0.0
118617200
118617400
118617600
118617800
118618000
chr2: 118617230 − 118618054
Control
RMNS

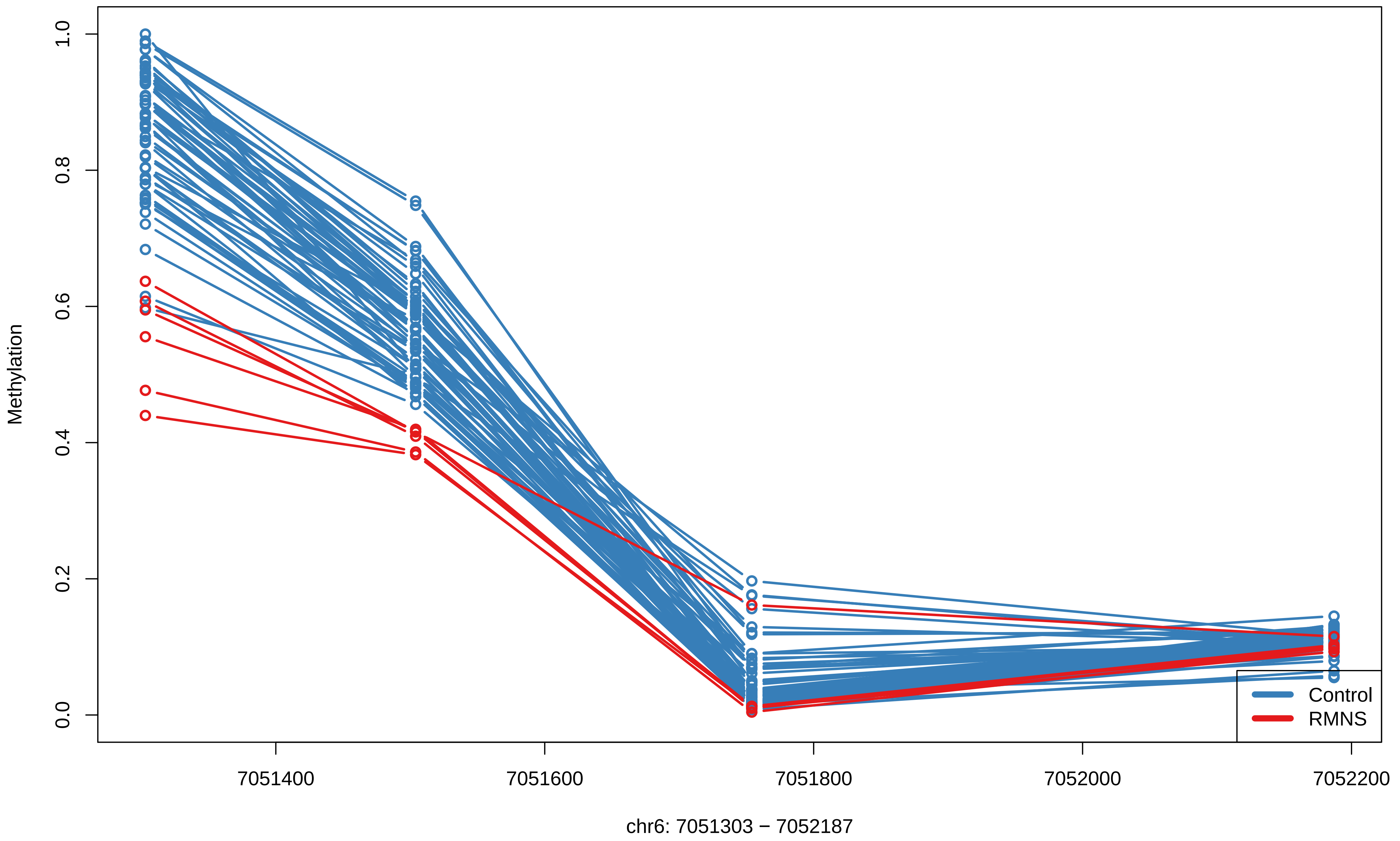

Methylation
1.0
0.8
0.6
0.4
0.2
0.0
7051400
7051600
7051800
7052000
7052200
chr6: 7051303 − 7052187
Control
RMNS

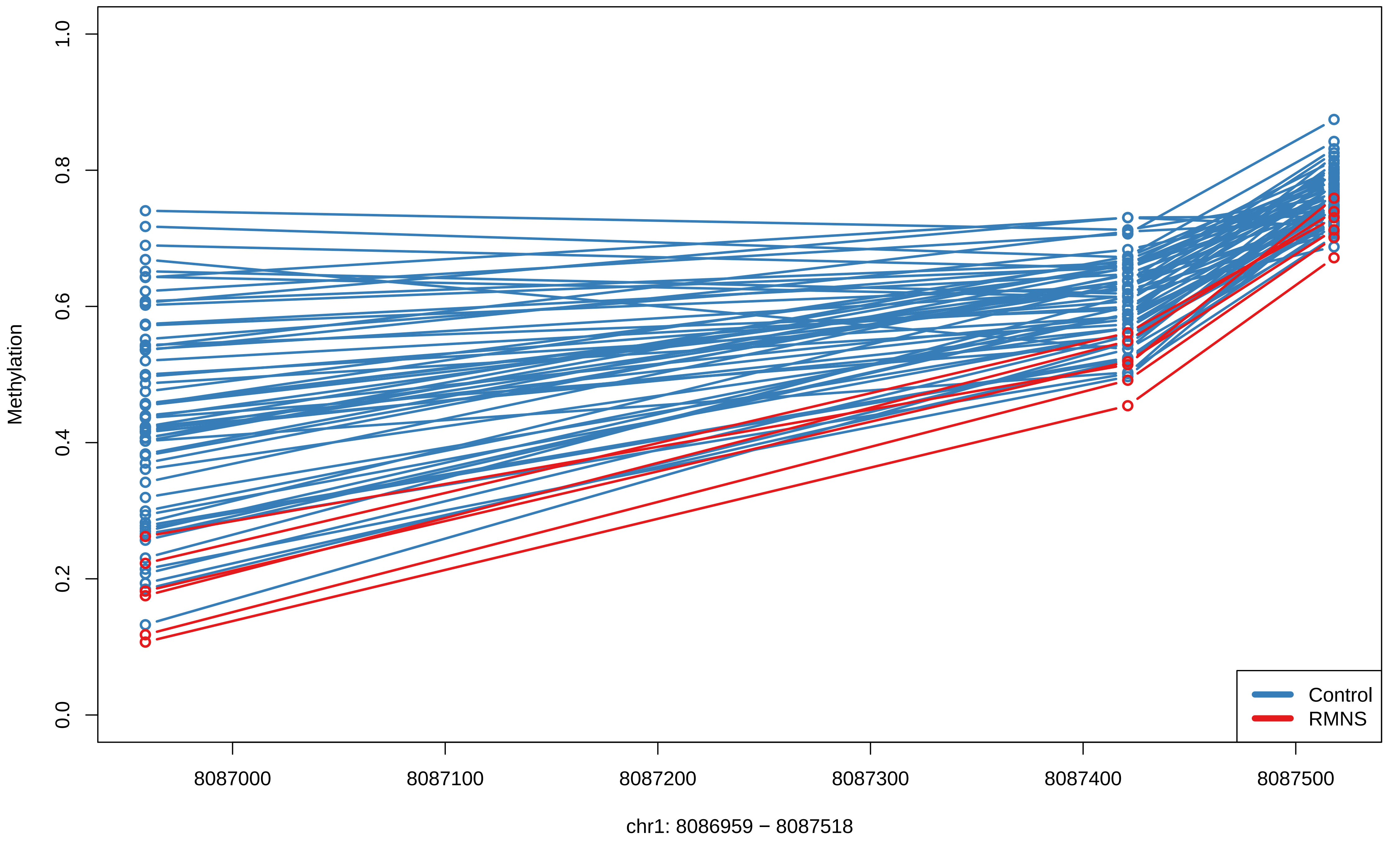

Methylation
0.0
0.2
0.4
0.6
0.8
1.0
8087000
8087100
8087200
8087300
8087400
8087500
chr1: 8086959 − 8087518
Control
RMNS

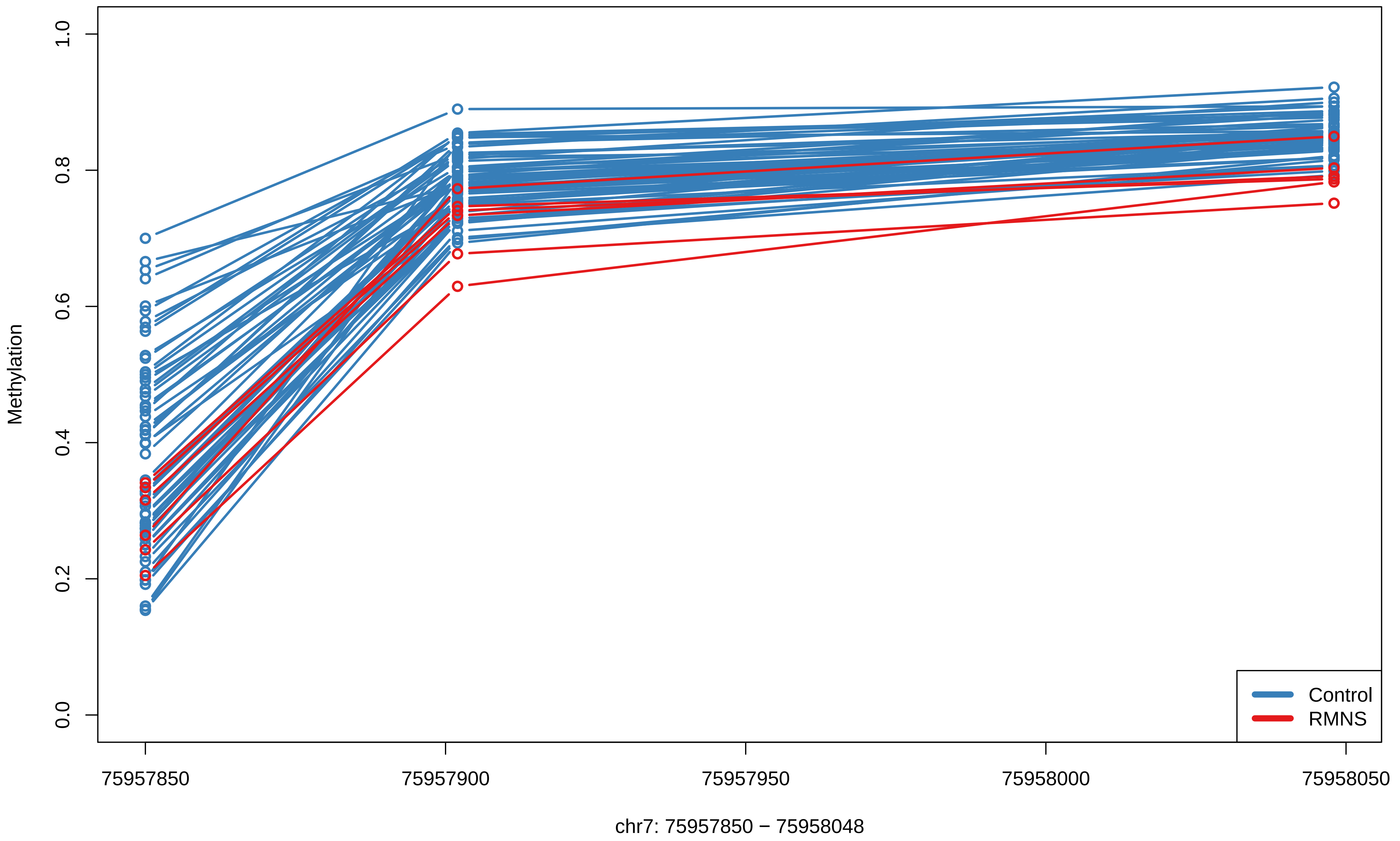

Methylation
0.0
0.2
0.4
0.6
0.8
1.0
75957850
75957900
75957950
75958000
75958050
chr7: 75957850 − 75958048
Control
RMNS

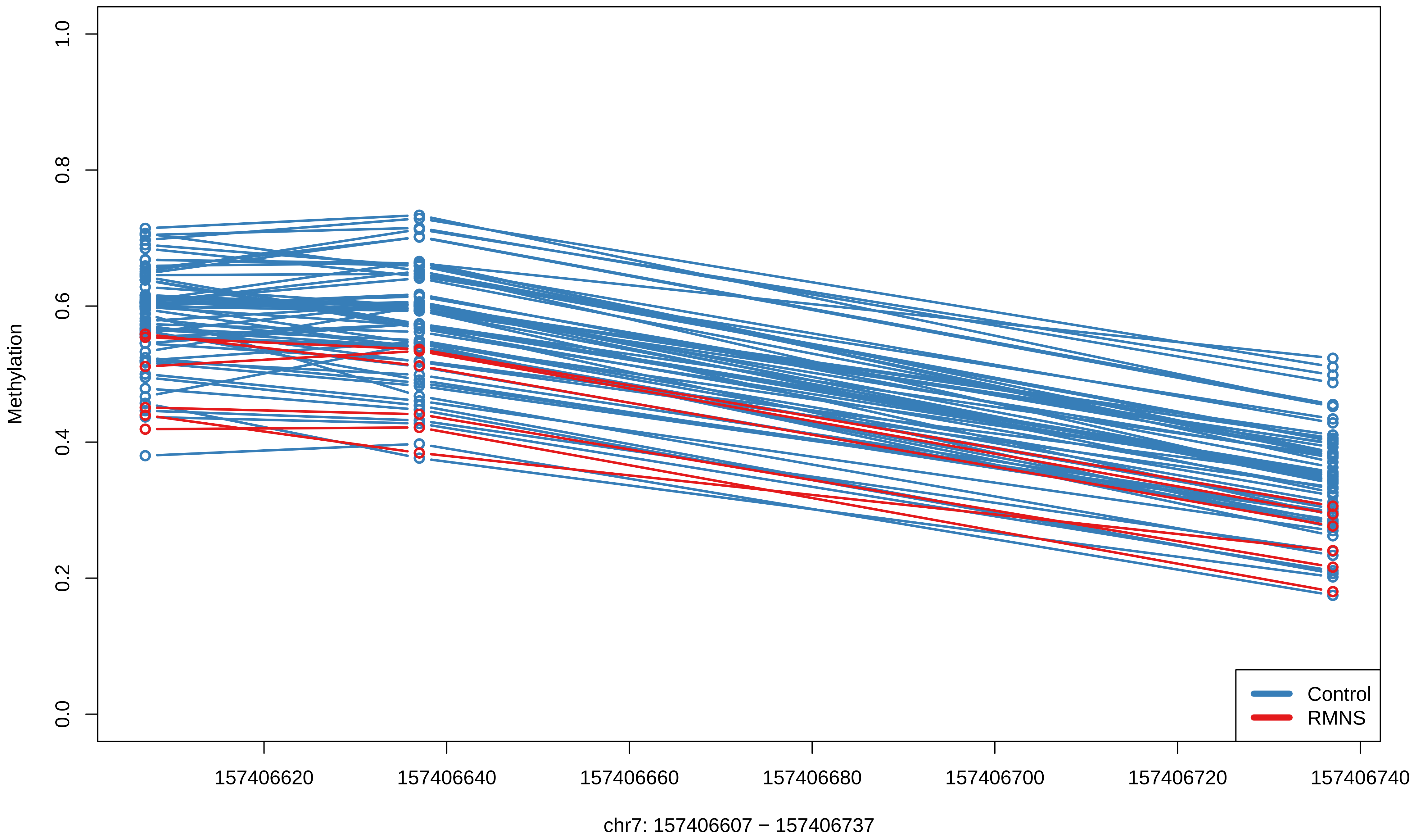

Methylation
0.0
0.2
0.4
0.6
0.8
1.0
157406620
157406640
157406660
157406680
157406700
157406720
157406740
chr7: 157406607 − 157406737
Control
RMNS

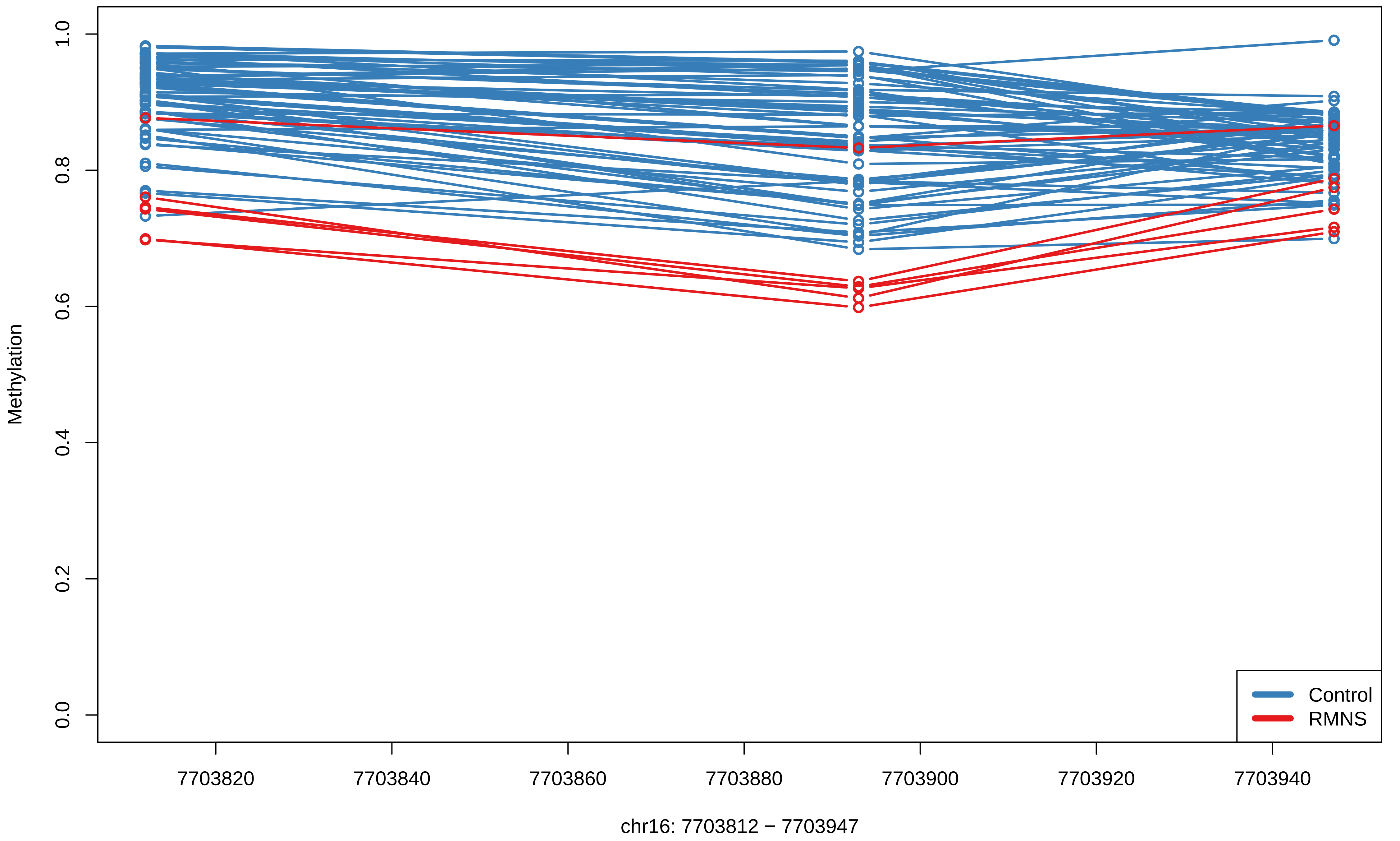

Methylation
1.0
0.8
0.6
0.4
0.2
0.0
7703820
7703840
7703860
7703880
7703900
7703920
7703940
chr16: 7703812 − 7703947
Control
RMNS

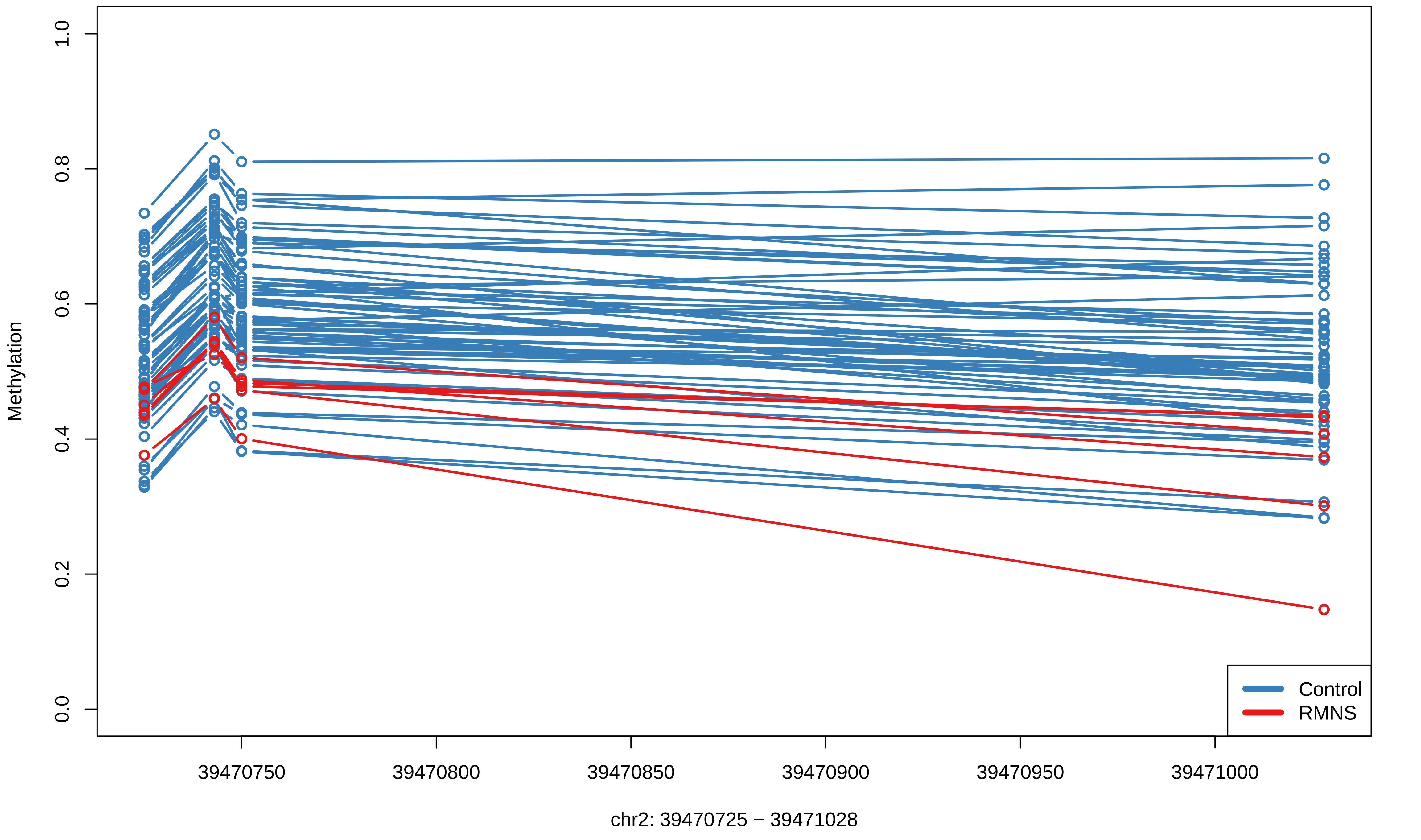

1.0
0.8
0.6
0.4
0.2
0.0
Methylation
39470750
39470800
39470850
39470900
39470950
39471000
chr2: 39470725 − 39471028
Control
RMNS

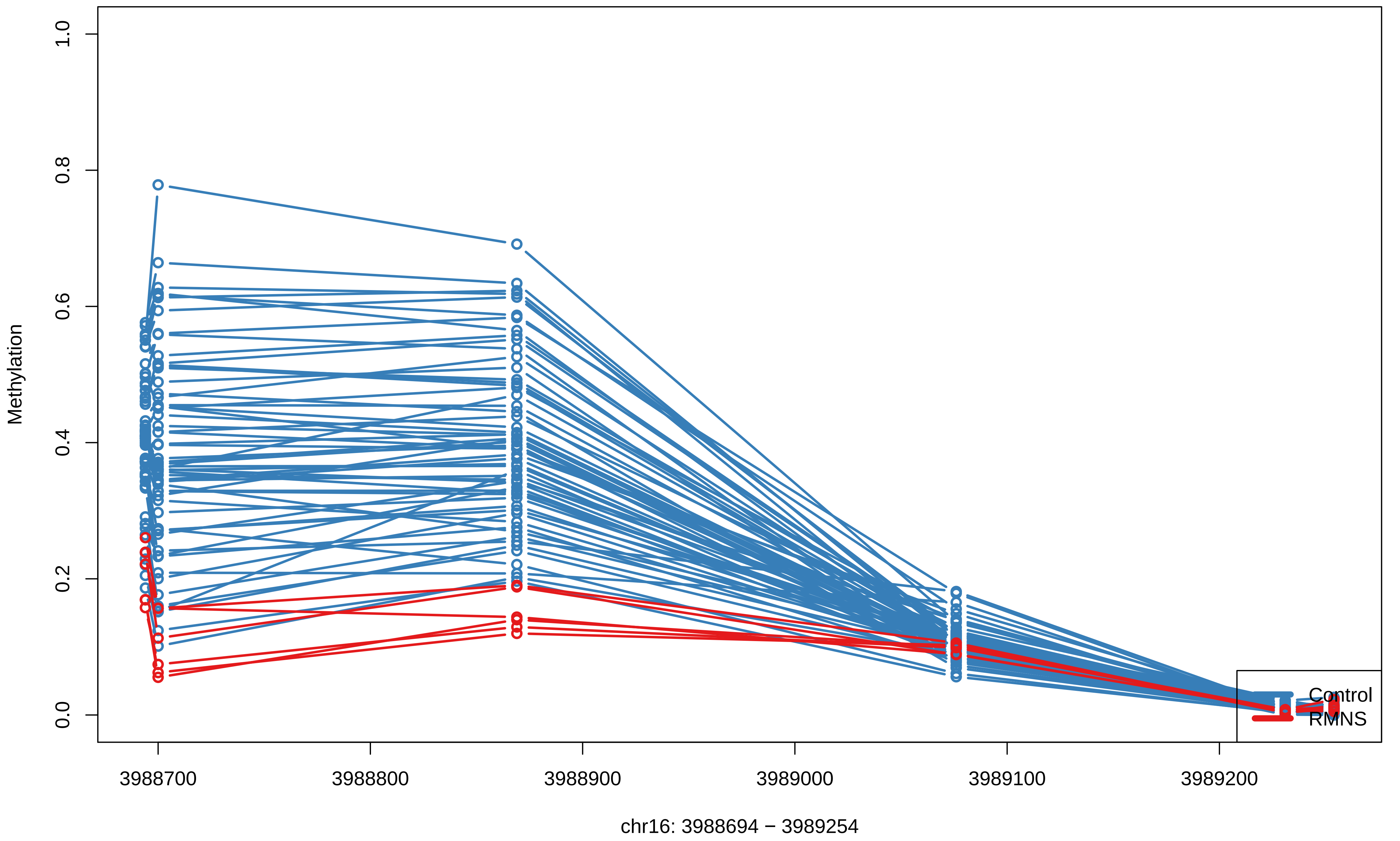

Methylation
0.0
0.2
0.4
0.6
0.8
1.0
3988700
3988800
3988900
3989000
3989100
3989200
chr16: 3988694 − 3989254
Control
RMNS

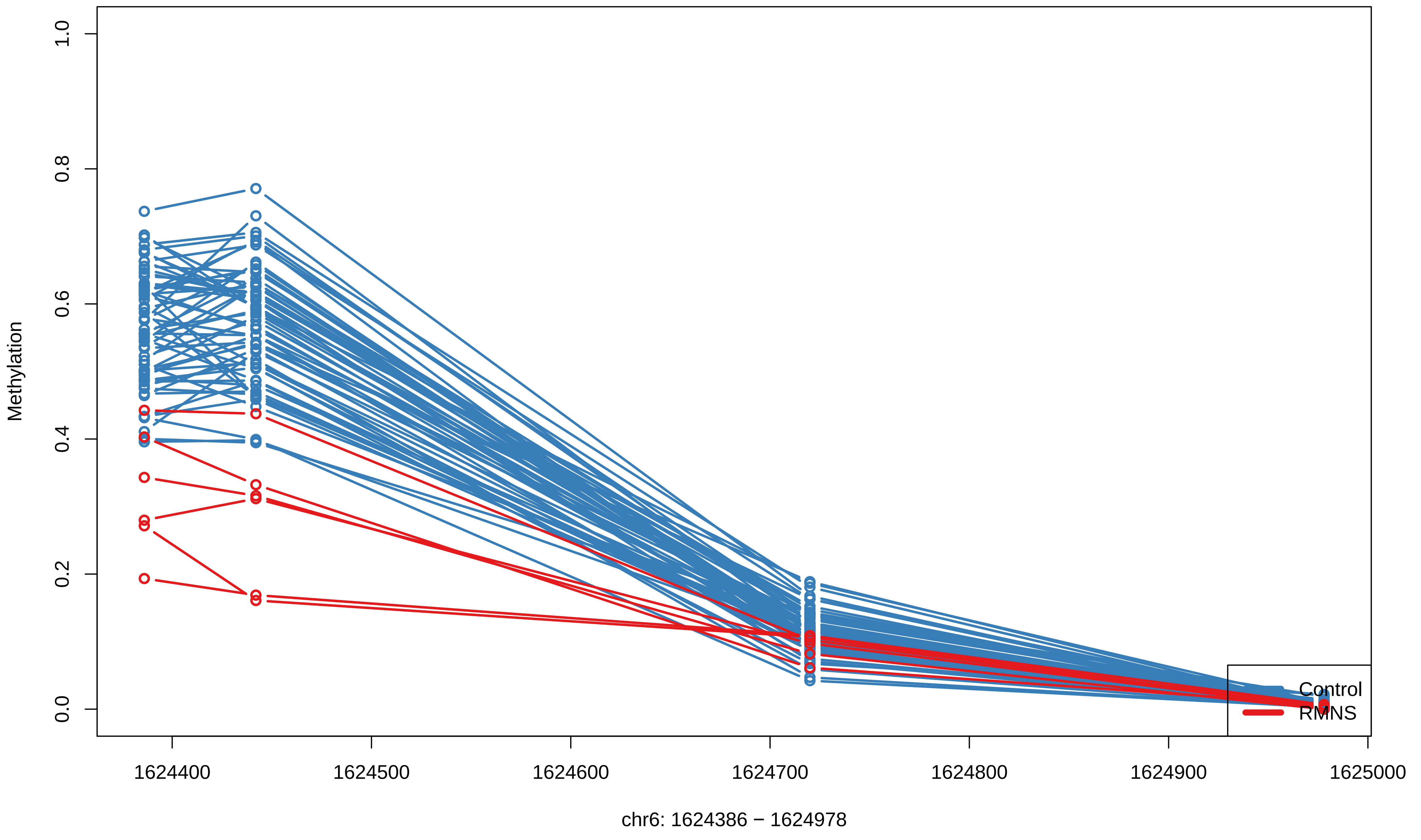

Methylation
0.0
0.2
0.4
0.6
0.8
1.0
1624400
1624500
1624600
1624700
1624800
1624900
1625000
chr6: 1624386 − 1624978
Control
RMNS

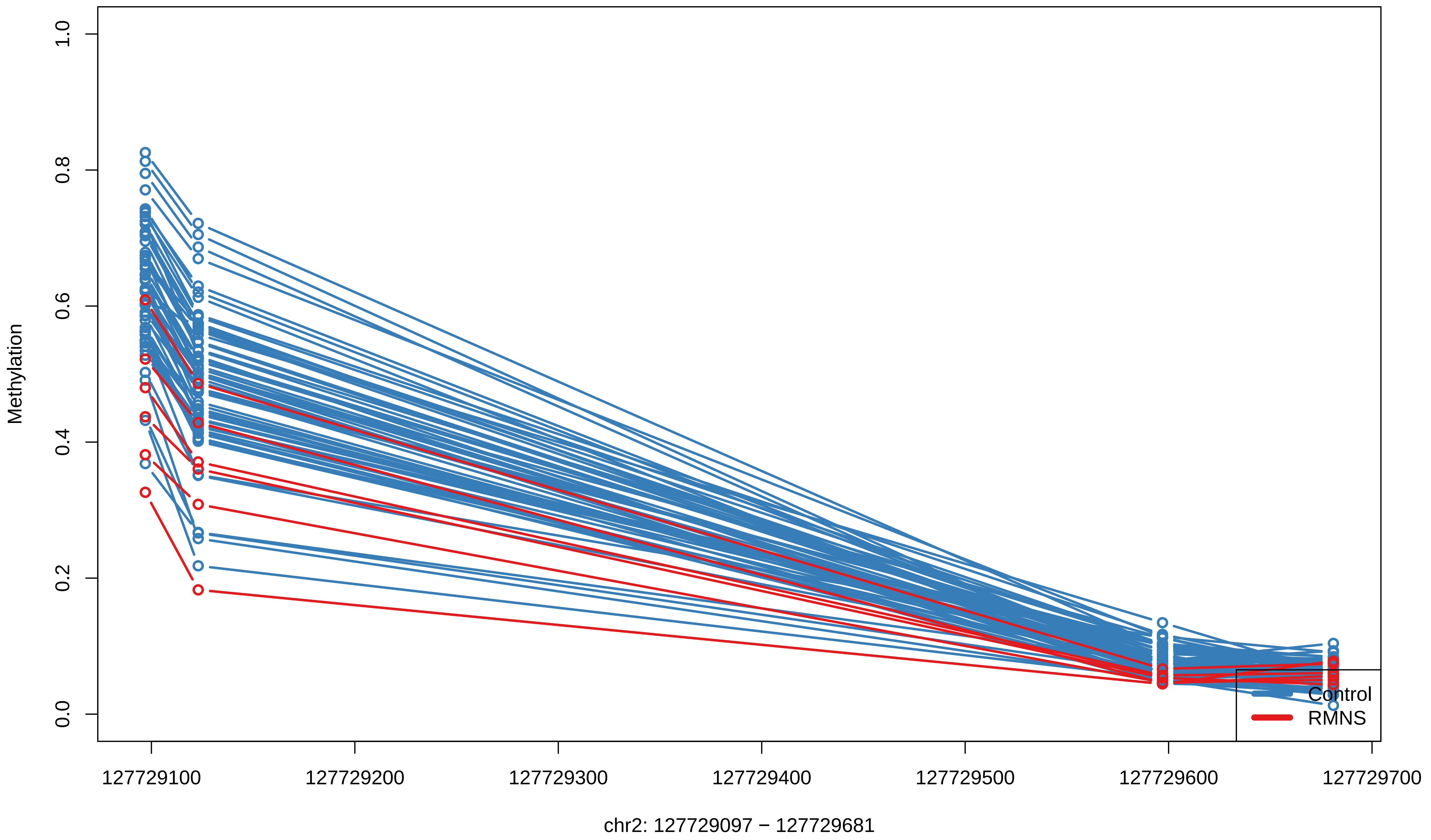

Methylation
0.0
0.2
0.4
0.6
0.8
1.0
127729100
127729200
127729300
127729400
127729500
127729600
127729700
chr2: 127729097 − 127729681
Control
RMNS

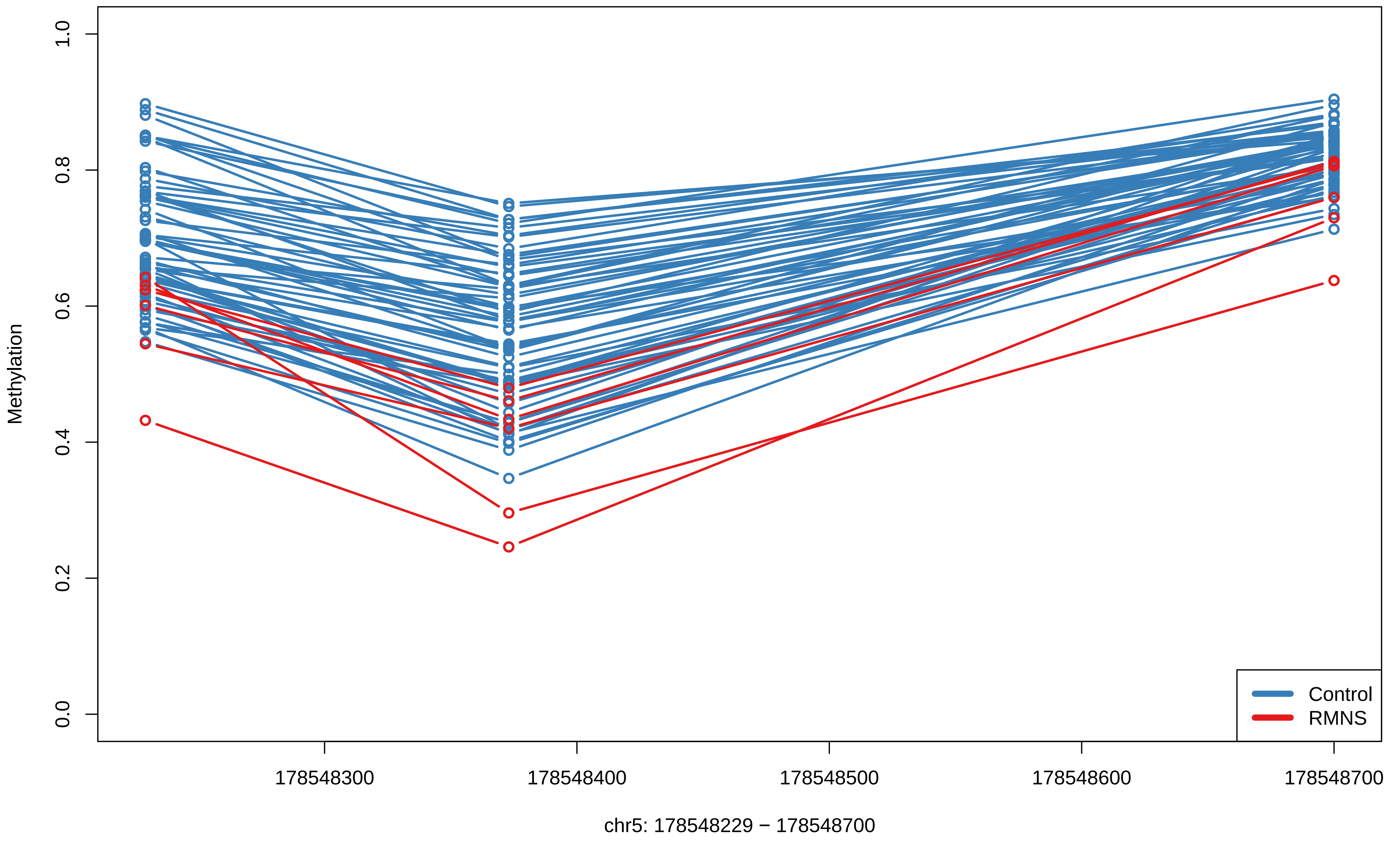

Methylation
0.0
0.2
0.4
0.6
0.8
1.0
178548300
178548400
178548500
178548600
178548700
chr5: 178548229 − 178548700
Control
RMNS

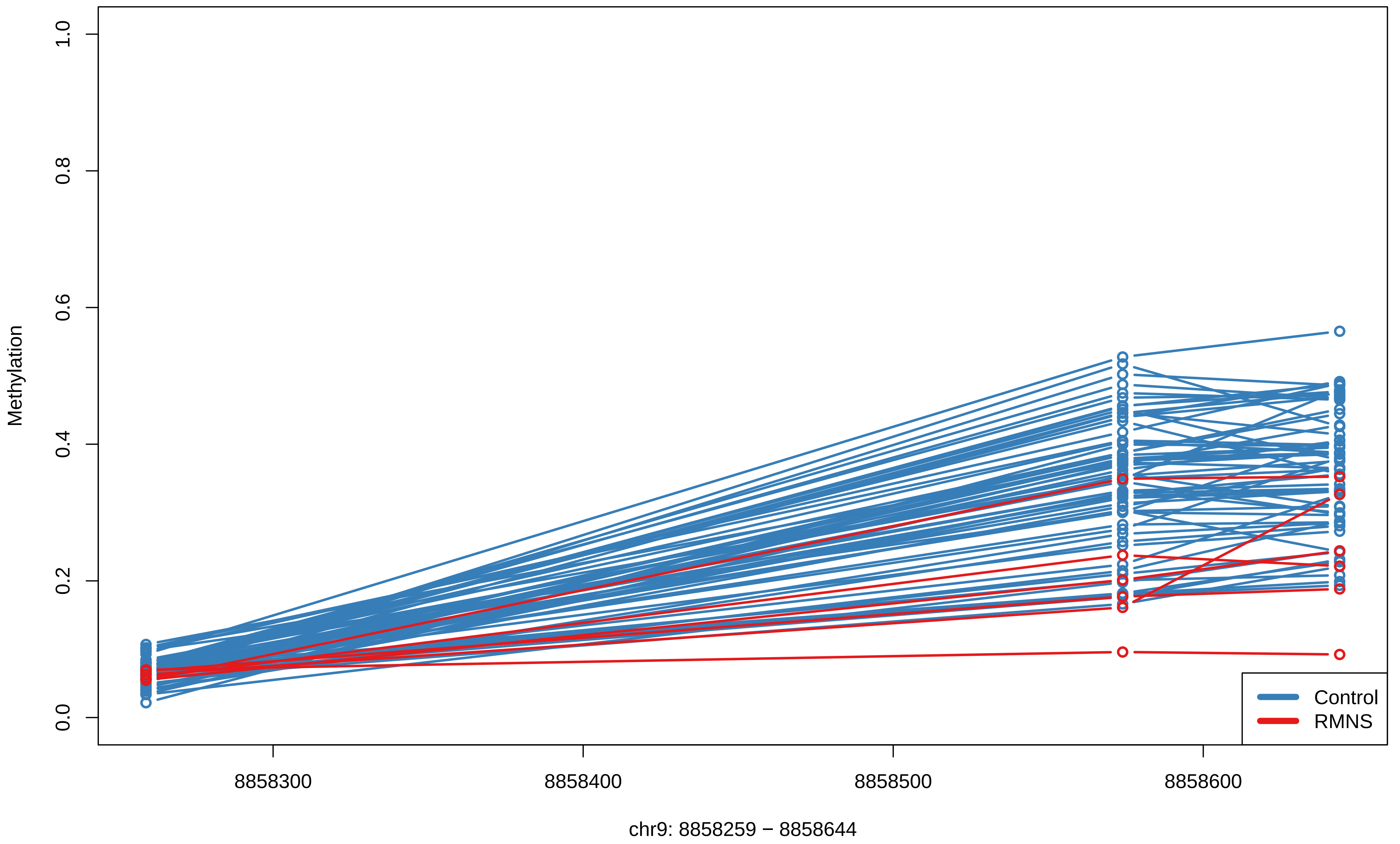

Methylation
0.0
0.2
0.4
0.6
0.8
1.0
8858300
8858400
8858500
8858600
chr9: 8858259 − 8858644
Control
RMNS

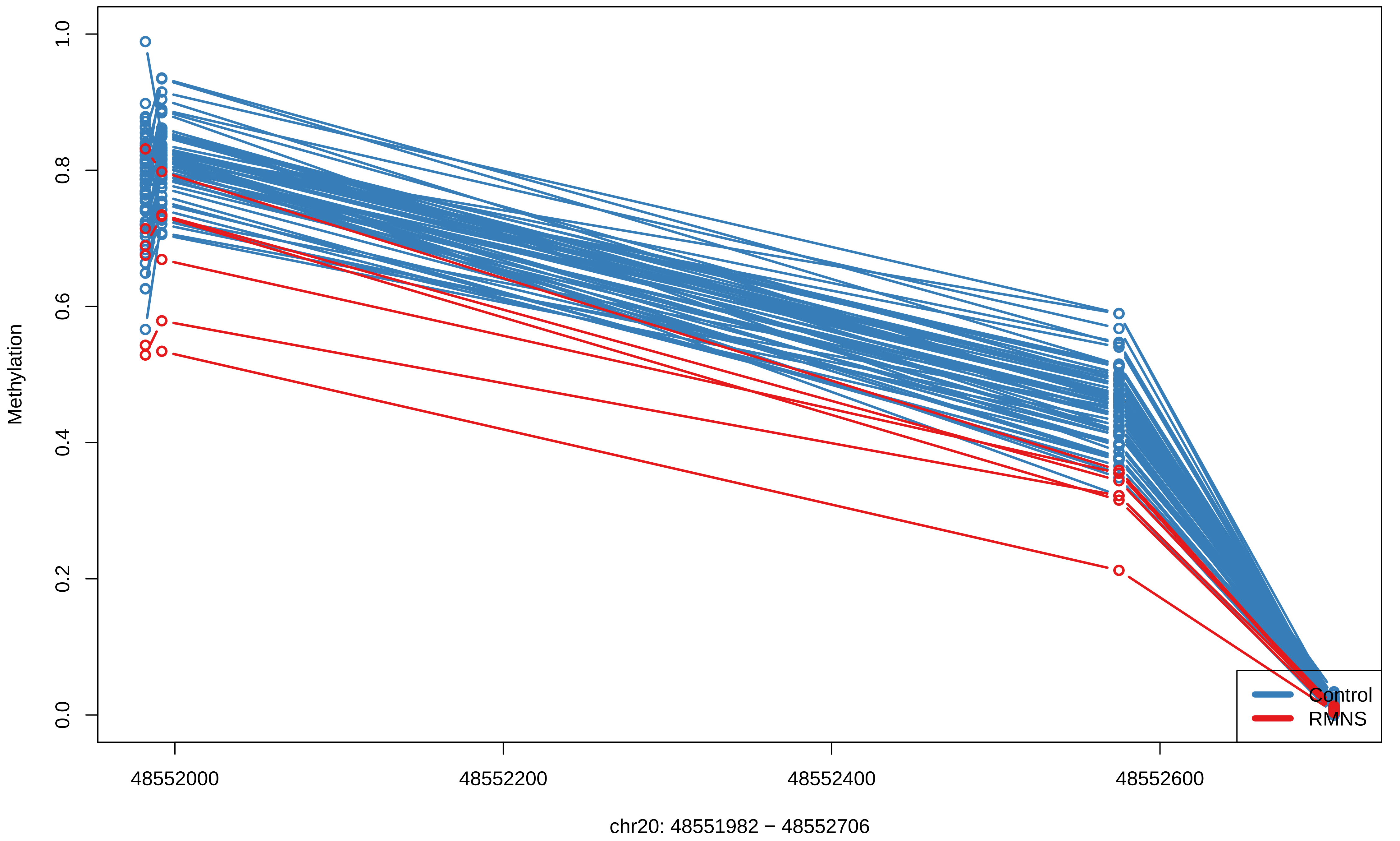

Methylation
0.0
0.2
0.4
0.6
0.8
1.0
48552000
48552200
48552400
48552600
chr20: 48551982 − 48552706
Control
RMNS

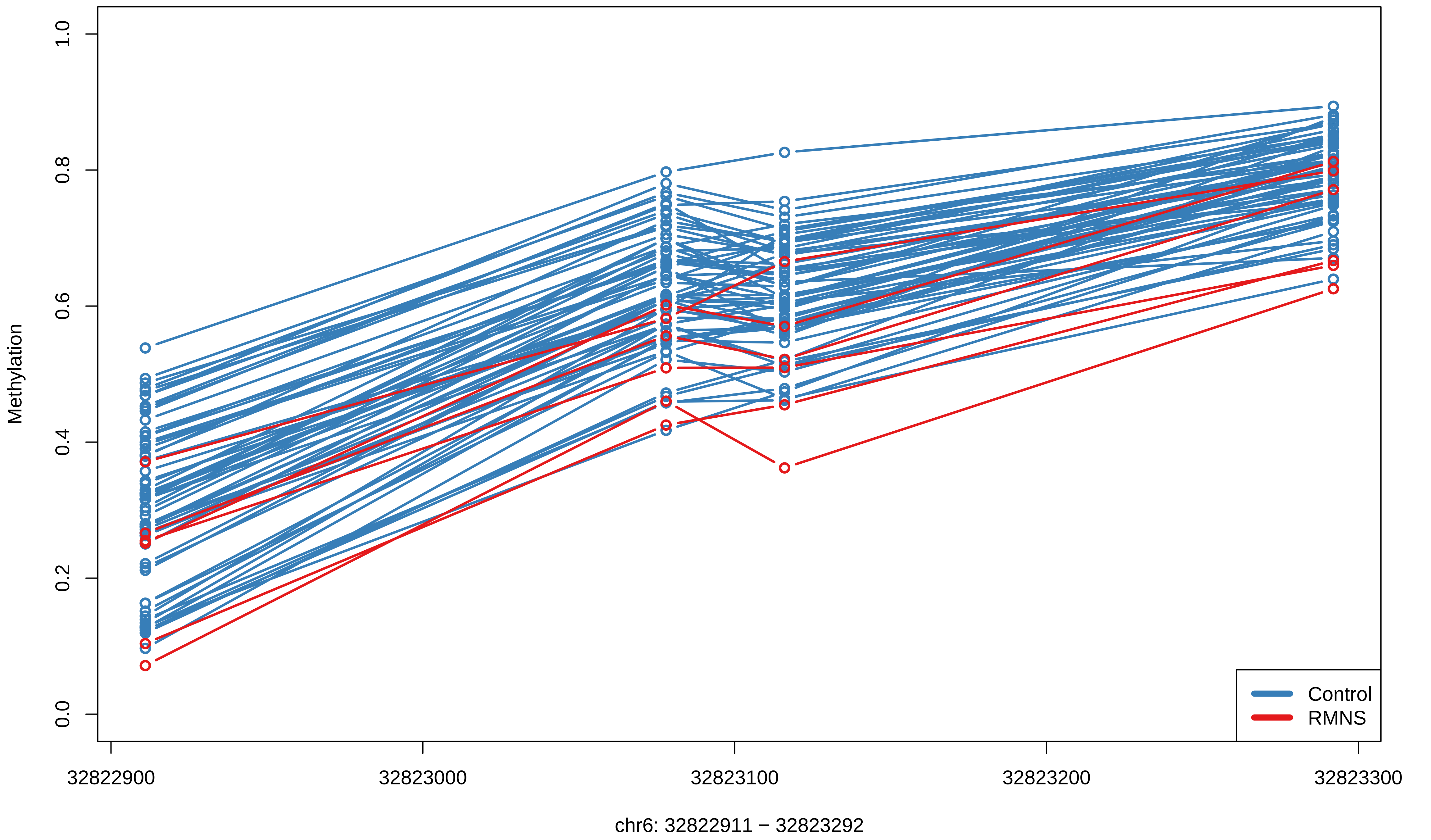

Methylation
0.0
0.2
0.4
0.6
0.8
1.0
32822900
32823000
32823100
32823200
32823300
chr6: 32822911 − 32823292
Control
RMNS

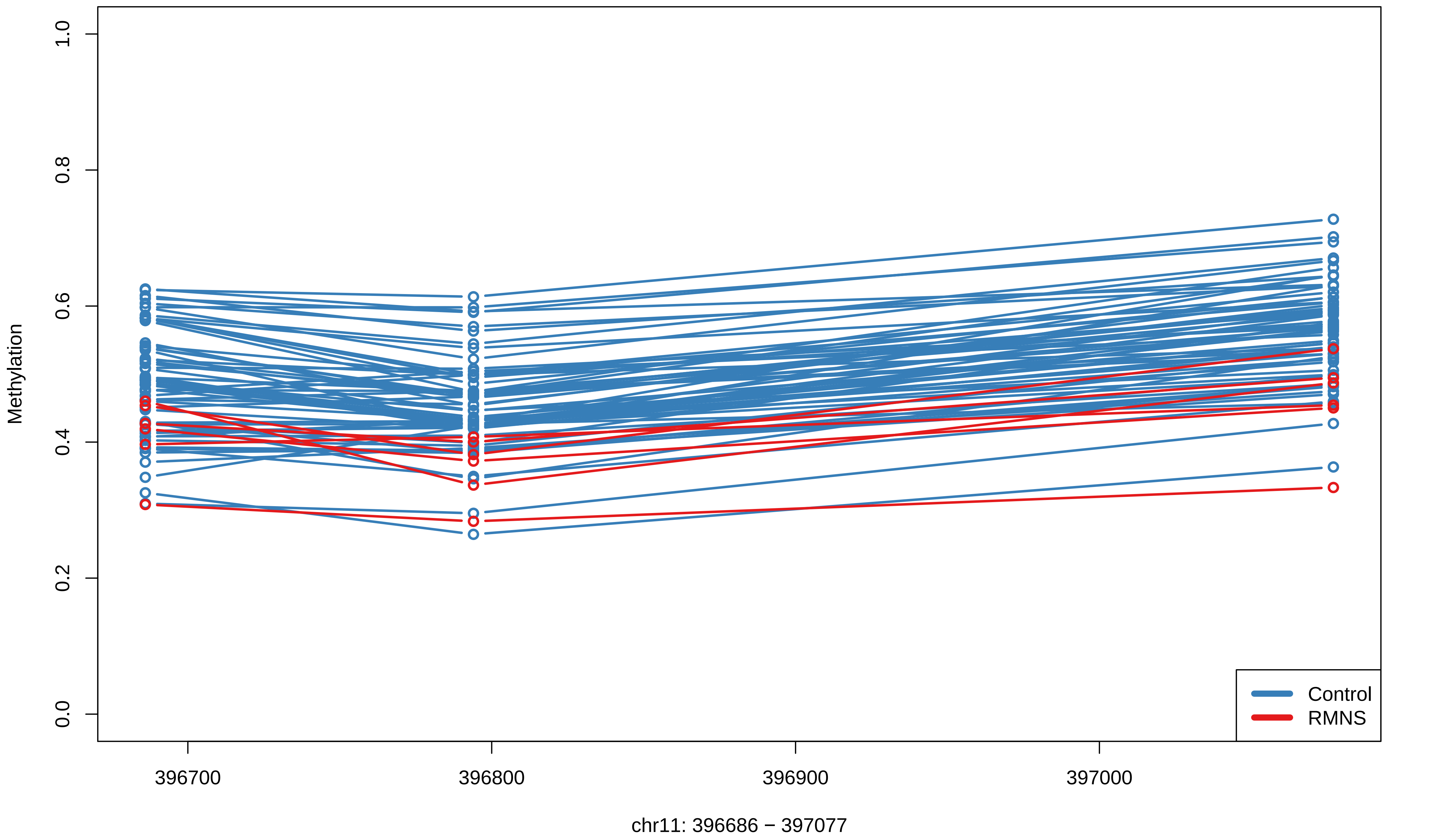
Methylation
1.0
0.8
0.6
0.4
0.2
0.0
396700
396800
396900
397000
chr11: 396686 − 397077
Control
RMNS

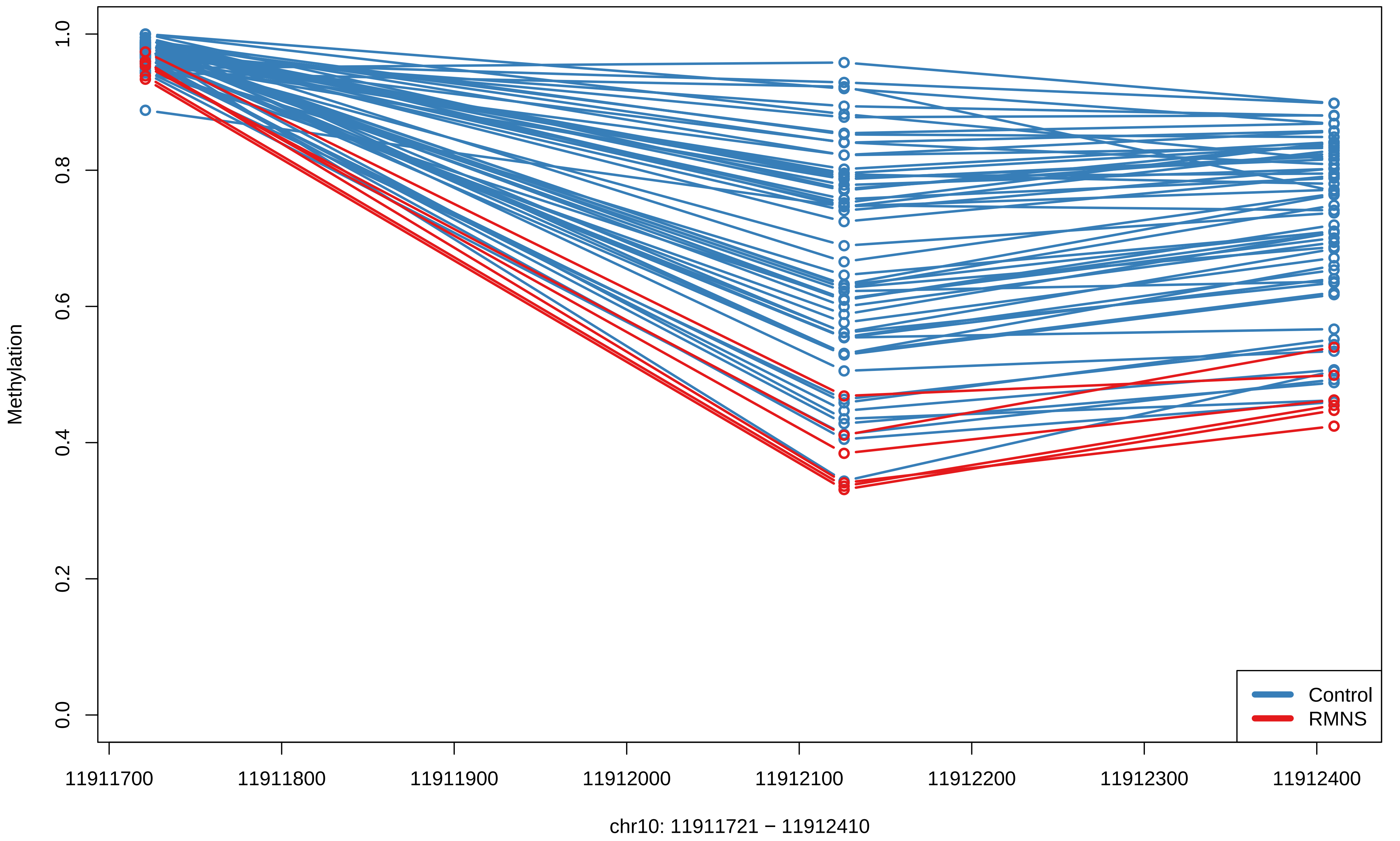

Methylation
1.0
0.8
0.6
0.4
0.2
0.0
11911700
11911800
11911900
11912000
11912100
11912200
11912300
11912400
chr10: 11911721 − 11912410
Control
RMNS

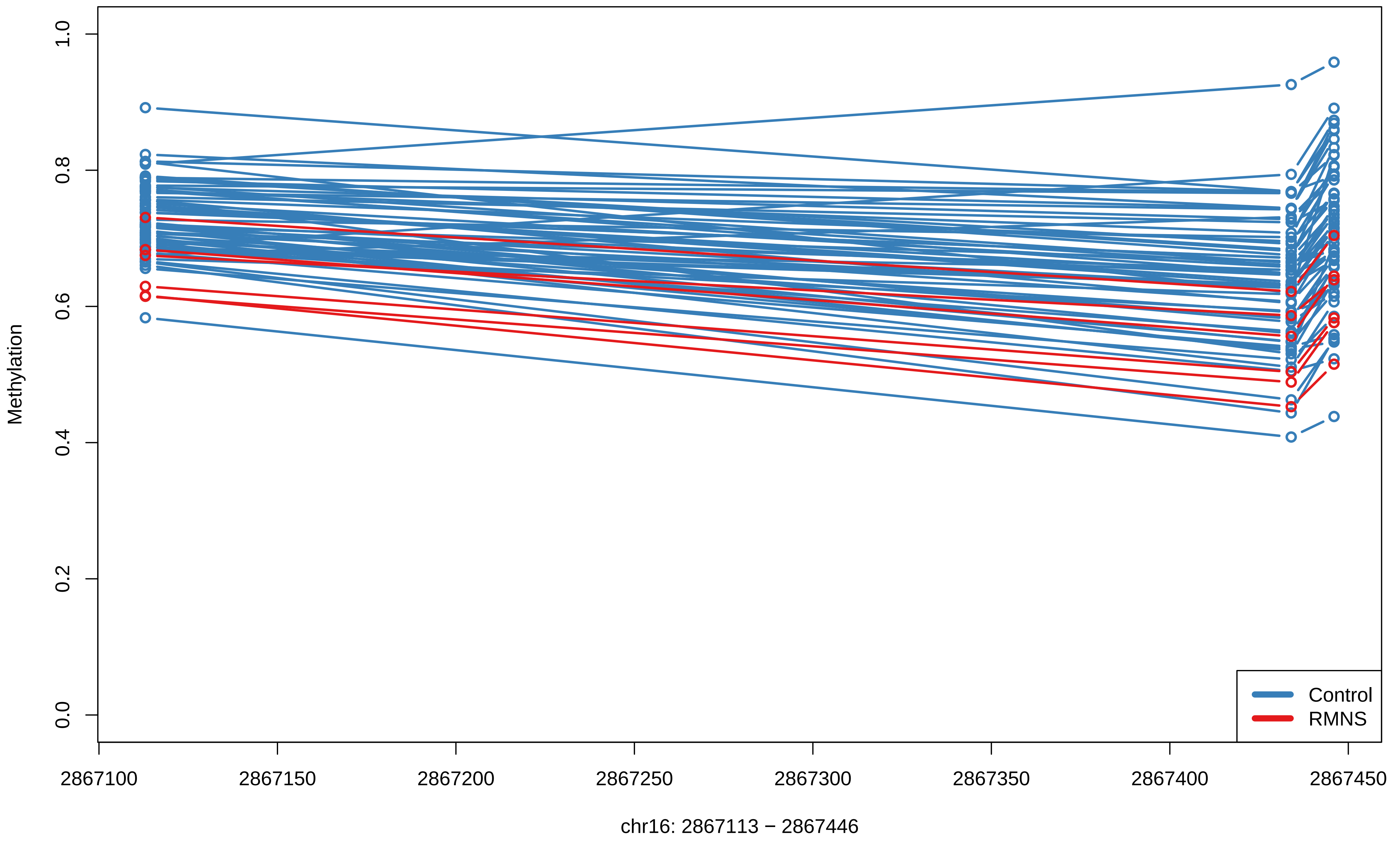
1.0
0.8
0.6
0.4
0.2
0.0
Methylation
2867100
2867150
2867200
2867250
2867300
2867350
2867400
2867450
chr16: 2867113 − 2867446
Control
RMNS

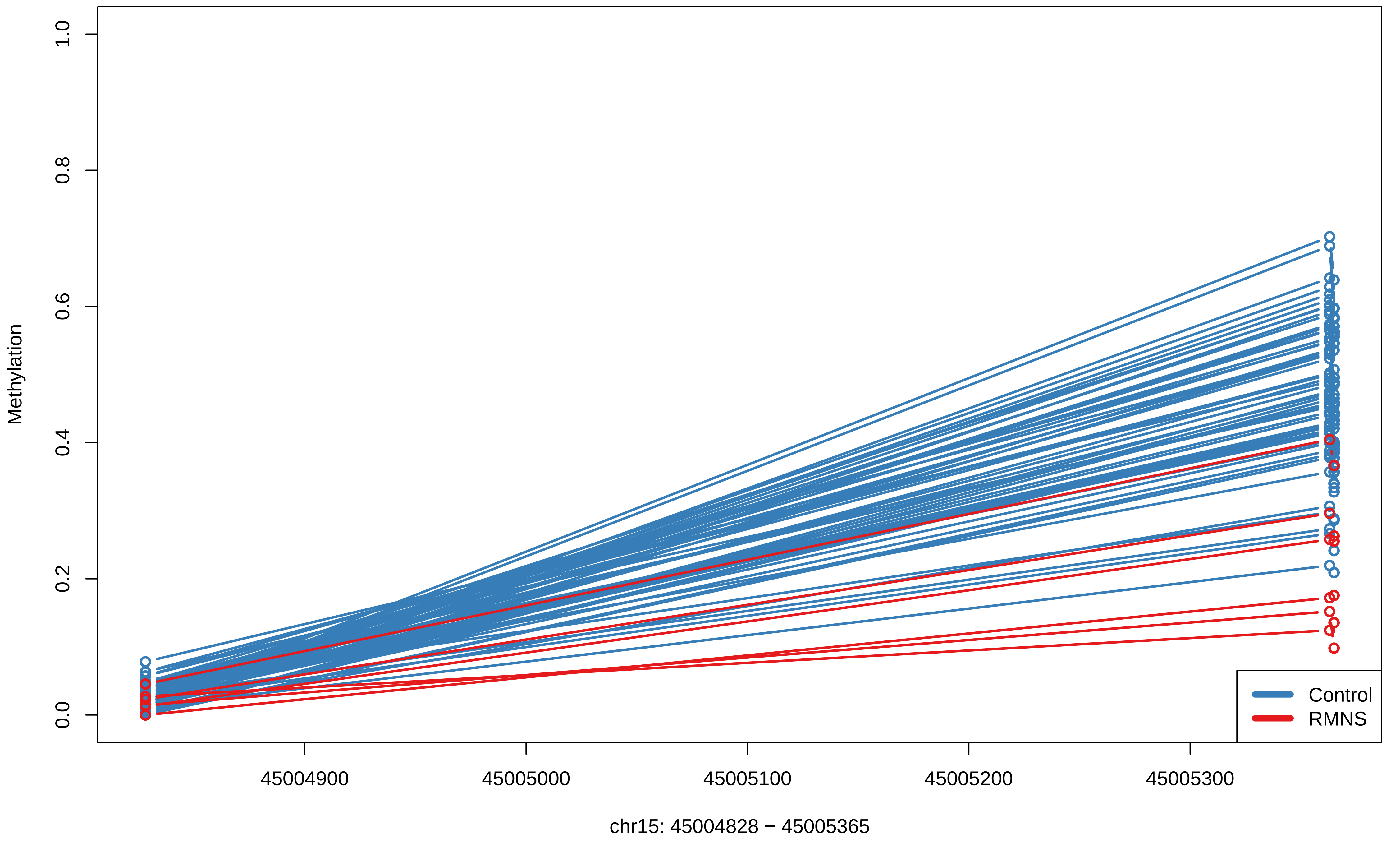

Methylation
0.0
0.2
0.4
0.6
0.8
1.0
45004900
45005000
45005100
45005200
45005300
chr15: 45004828 − 45005365
Control
RMNS
