# Supplementary material for: Frameshift mutations at the C-terminus of HIST1H1E result in a specific DNA hypomethylation signature
Source: Clin Epigenetics. 2020 Jan 7;12:7. doi: 10.1186/s13148-019-0804-0 (PMC6947958; doi:10.1186/s13148-019-0804-0)
Supplement: Supplementary file 2 — Additional file 2: Figure S2. Tissue-specific expression profiles for hypomethylated genes in Rahman syndrome (RMNS). The expression profiles from 416 tissues/anatomical parts are calculated using 65761 Affymetrix Human Genome U133 Plus 2.0 arrays in Genevestigator; hierarchical clustering is performed using Pearson correlation as similarity measure and optimal-leaf ordering. Black square highlights gene cluster with maximum expression in brain tissues (displayed in Fig. 3). [file 13148_2019_804_MOESM2_ESM.pdf]

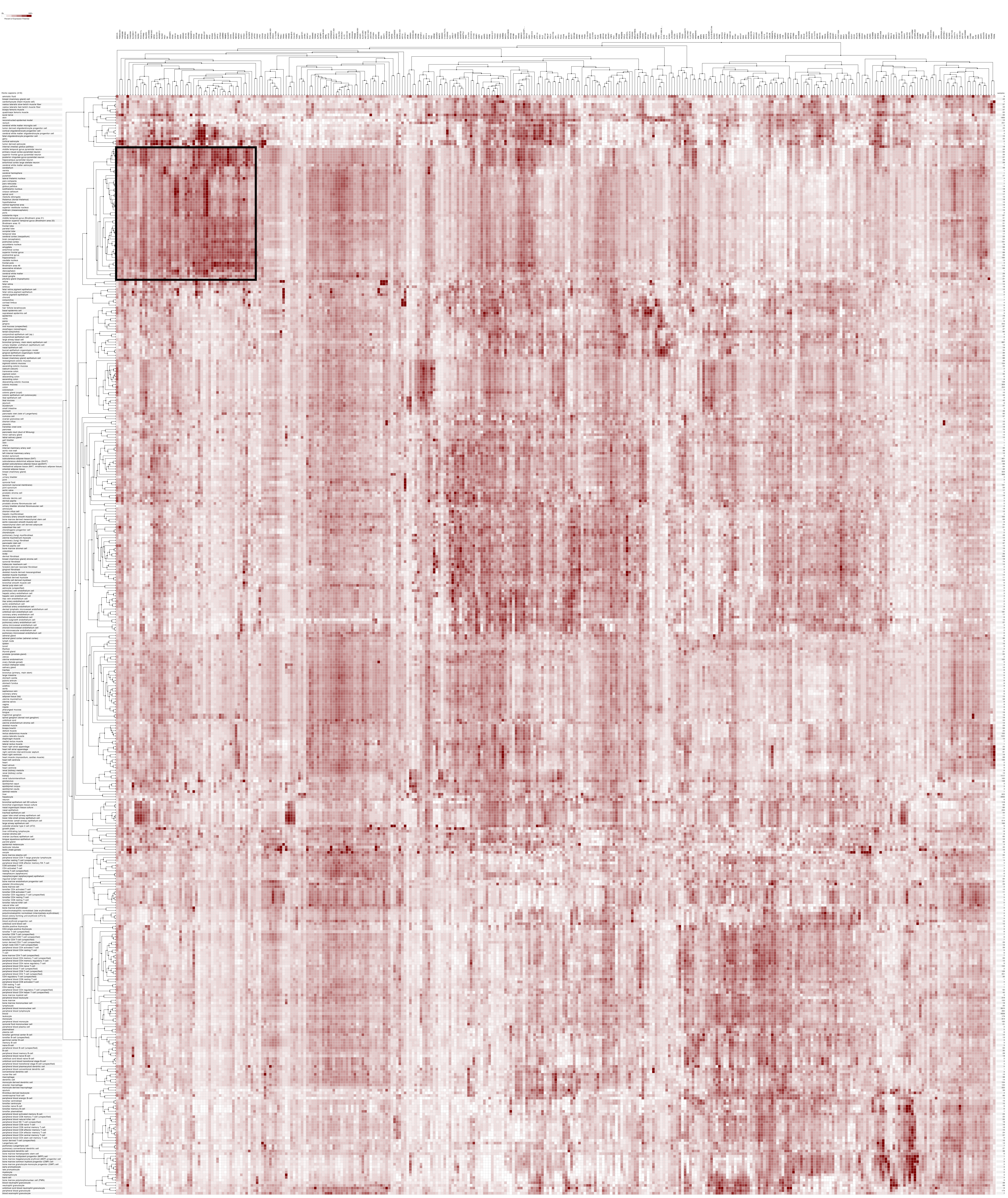

0%
100%
Percent of Expression Potential
Homo sapiens (416)
